# Supplementary material for: Response mechanism of carbon metabolism of Pinus massoniana to gradient high temperature and drought stress
Source: BMC Genomics. 2024 Feb 12;25:166. doi: 10.1186/s12864-024-10054-2 (PMC10860282; doi:10.1186/s12864-024-10054-2)
Supplement: Supplementary file 1 — Additional file 1. [file 12864_2024_10054_MOESM1_ESM.docx]

Table S1 The statistics of raw data. Reads No.: The overall count of Reads; Bases(bp): Number of bases; Q30 (bp): The total count of bases exhibiting an accuracy exceeding 99.9% was determined.; N(%): The proportion of bases with ambiguous assignments.; Q20(%): Percentage of bases that were identified with more than 99 % accuracy; Q30(%)：Percentage of bases that were identified with more than 99.9 % accuracy。

| **Sample** | **Reads No.** | **Bases (bp)** | **Q30 (bp)** | **N (%)** | **Q20 (%)** | **Q30 (%)** |
| --- | --- | --- | --- | --- | --- | --- |
| T25CK1 | 40651082 | 6138313382 | 5793681302 | 0.001139 | 98.1 | 94.38 |
| T25CK2 | 40160896 | 6064295296 | 5727766924 | 0.001149 | 98.16 | 94.45 |
| T25CK3 | 38639068 | 5834499268 | 5509854516 | 0.001125 | 98.12 | 94.43 |
| T25Z1 | 36949042 | 5579305342 | 5270158693 | 0.001174 | 98.12 | 94.45 |
| T25Z2 | 46602818 | 7037025518 | 6663301084 | 0.001176 | 98.22 | 94.68 |
| T25Z3 | 42352046 | 6395158946 | 6069354972 | 0.00116 | 98.3 | 94.9 |
| T30CK1 | 39152846 | 5912079746 | 5597165114 | 0.001148 | 98.23 | 94.67 |
| T30CK2 | 39230734 | 5923840834 | 5575811147 | 0.001149 | 98 | 94.12 |
| T30CK3 | 40692646 | 6144589546 | 5795045400 | 0.001156 | 98.08 | 94.31 |
| T30Z1 | 45105096 | 6810869496 | 6452382064 | 0.001137 | 98.26 | 94.73 |
| T30Z2 | 45225782 | 6829093082 | 6403921128 | 0.001123 | 97.85 | 93.77 |
| T30Z3 | 45419054 | 6858277154 | 6482869185 | 0.00114 | 98.16 | 94.52 |
| T35CK1 | 39756730 | 6003266230 | 5662831692 | 0.001162 | 98.07 | 94.32 |
| T35CK2 | 44912260 | 6781751260 | 6380091324 | 0.001149 | 97.98 | 94.07 |
| T35CK3 | 44009176 | 6645385576 | 6251922606 | 0.001123 | 97.97 | 94.07 |
| T35Z1 | 42802754 | 6463215854 | 6104240724 | 0.001151 | 98.13 | 94.44 |
| T35Z2 | 41423928 | 6255013128 | 5919212040 | 0.001146 | 98.2 | 94.63 |
| T35Z3 | 43440540 | 6559521540 | 6207028849 | 0.001125 | 98.2 | 94.62 |

Table S2 The statistics of data filtering. Clean Reads No: The quantity of sequence reads with high quality.; Clean Data (bp)：The count of bases in high-quality sequences.; Clean Reads %: The proportion of high-quality sequence reads among the total sequenced reads.; Clean Data %: The proportion of high-quality bases in the total sequenced bases.

| **Sample** | **Clean Reads No.** | **Clean Data (bp)** | **Clean Reads %** | **Clean Data %** |
| --- | --- | --- | --- | --- |
| T25CK1 | 38348600 | 5790638600 | 94.33 | 94.33 |
| T25CK2 | 37924222 | 5726557522 | 94.43 | 94.43 |
| T25CK3 | 36433556 | 5501466956 | 94.29 | 94.29 |
| T25Z1 | 34832706 | 5259738606 | 94.27 | 94.27 |
| T25Z2 | 43923726 | 6632482626 | 94.25 | 94.25 |
| T25Z3 | 39865744 | 6019727344 | 94.12 | 94.12 |
| T30CK1 | 36920674 | 5575021774 | 94.29 | 94.29 |
| T30CK2 | 37041144 | 5593212744 | 94.41 | 94.41 |
| T30CK3 | 38429136 | 5802799536 | 94.43 | 94.43 |
| T30Z1 | 42492918 | 6416430618 | 94.2 | 94.2 |
| T30Z2 | 42609752 | 6434072552 | 94.21 | 94.21 |
| T30Z3 | 42799492 | 6462723292 | 94.23 | 94.23 |
| T35CK1 | 37510860 | 5664139860 | 94.35 | 94.35 |
| T35CK2 | 42351116 | 6395018516 | 94.29 | 94.29 |
| T35CK3 | 41485354 | 6264288454 | 94.26 | 94.26 |
| T35Z1 | 40300800 | 6085420800 | 94.15 | 94.15 |
| T35Z2 | 38982238 | 5886317938 | 94.1 | 94.1 |
| T35Z3 | 40852800 | 6168772800 | 94.04 | 94.04 |


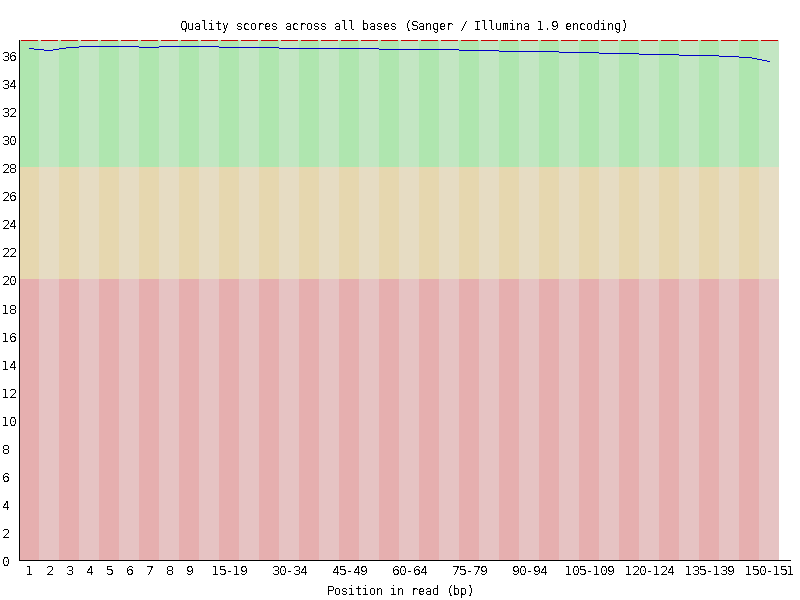

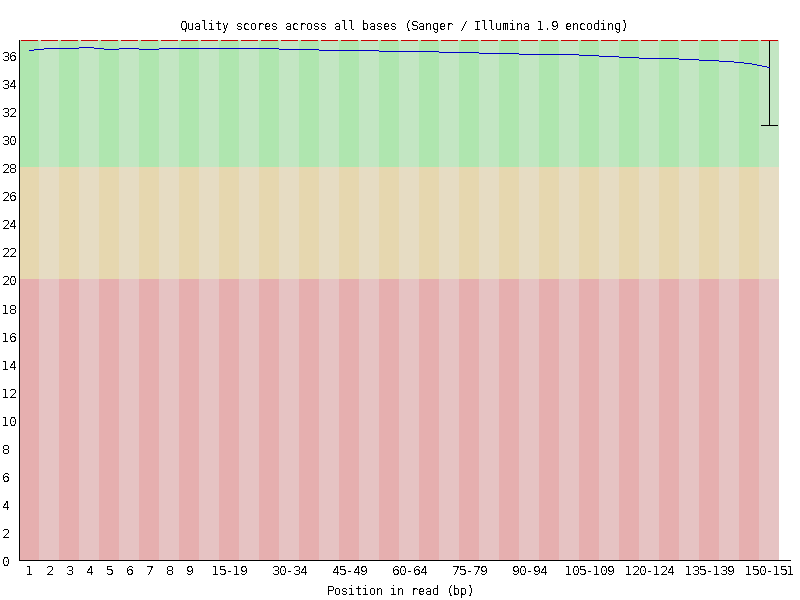

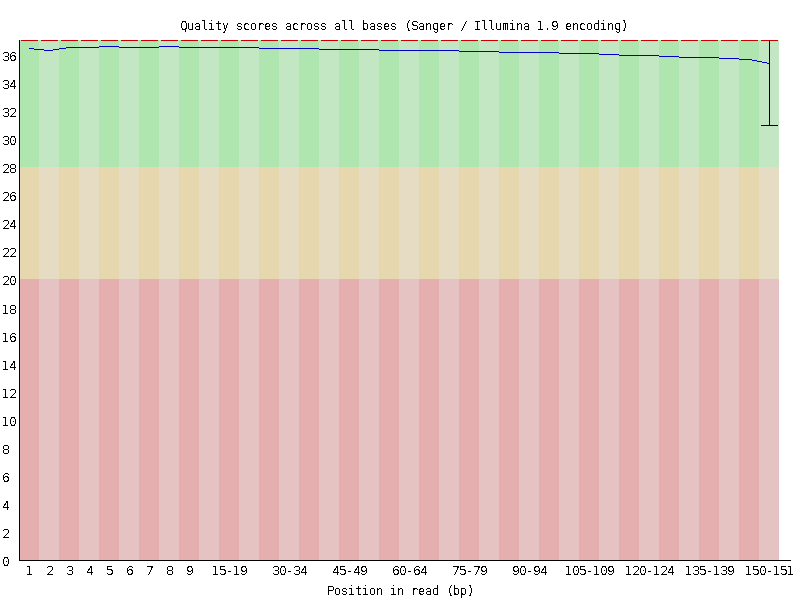


T30CK2 T30Z1 T35Z3

Fig. S1 per_base_quality


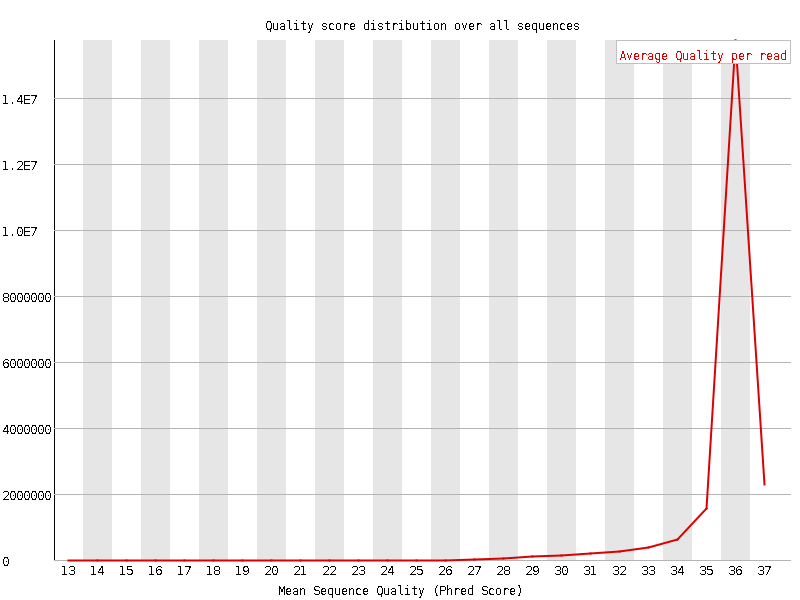

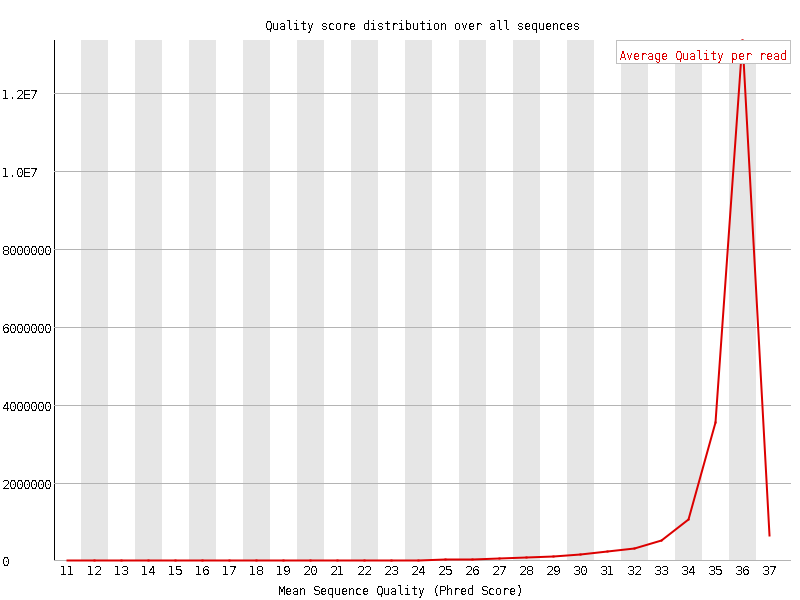

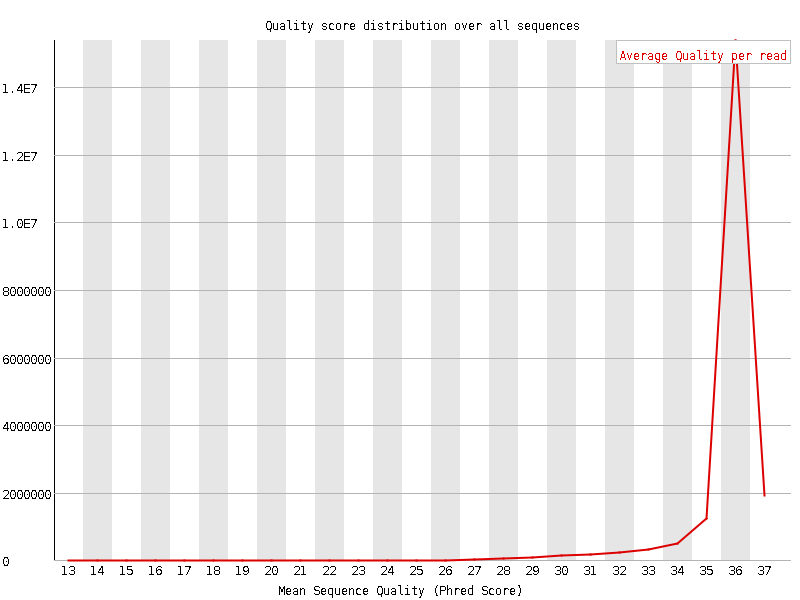


T35Z3 T30CK3 T25CK1

Fig. S2 per_sequence_quality
