# Supplementary material for: Response mechanism of carbon metabolism of Pinus massoniana to gradient high temperature and drought stress
Source: BMC Genomics. 2024 Feb 12;25:166. doi: 10.1186/s12864-024-10054-2 (PMC10860282; doi:10.1186/s12864-024-10054-2)
Supplement: Supplementary file 2 — Additional file 2. [file 12864_2024_10054_MOESM2_ESM.docx]

Table S3 A total of 3014 differentially expressed genes were identified in the T25CK vs T25Z group

| **gene names** | **baseMean_T25CK** | **baseMean_T25Z** | **foldChange(T25Z/T25CK)** | **log2FoldChange** | **pval** | **padj** | **KEGG** |
| --- | --- | --- | --- | --- | --- | --- | --- |
| *SWEET3B* | 1.053205759 | 4470.719031 | 4244.867628 | 12.05150385 | 0 | 0 | K15382 |
| *YPQ1* | 1.676835131 | 510.204889 | 304.2665791 | 8.249192065 | 2.3763E-139 | 7.7014E-136 | - |
| *CYP709B2* | 31.46763279 | 1027.429453 | 32.65035728 | 5.029026874 | 9.7503E-119 | 1.9447E-115 | - |
| *LEA14-A* | 127.2182066 | 2224.326795 | 17.48434327 | 4.127991703 | 2.1833E-116 | 3.7739E-113 | - |
| *SRK2A* | 272.0017223 | 1888.342283 | 6.942390903 | 2.795432601 | 2.23762E-97 | 2.07203E-94 | K14498 |
| *RFS2* | 896.1090526 | 4704.652268 | 5.250088987 | 2.392341876 | 6.06621E-90 | 5.0737E-87 | K06617 |
| *P5CS* | 94.91687909 | 872.4232582 | 9.191444836 | 3.200291662 | 6.64007E-89 | 5.38012E-86 | K12657 |
| *BAM1* | 1105.809986 | 5535.647652 | 5.005966414 | 2.323648612 | 4.18445E-87 | 3.19102E-84 | K01177 |
| *ATL78* | 0 | 413.720475 | Inf | Inf | 5.23111E-84 | 3.76757E-81 | K19040 |
| *uncharacterized protein_00087* | 115.394254 | 909.9363533 | 7.885456353 | 2.97919425 | 2.20995E-82 | 1.50788E-79 | - |
| *GAD* | 1219.026623 | 5324.269596 | 4.367640127 | 2.12685399 | 6.48853E-74 | 3.57946E-71 | K01580 |
| *SUS3* | 1934.368904 | 7875.804408 | 4.071511071 | 2.025564325 | 2.16334E-70 | 1.09983E-67 | K00695 |
| *PARP1* | 159.8845469 | 939.1244022 | 5.873765918 | 2.554285772 | 5.52266E-66 | 2.42697E-63 | K10798 |
| *PCMP-E76* | 75.05863508 | 587.1893711 | 7.823075526 | 2.967735893 | 1.17611E-65 | 4.84034E-63 | - |
| *ADH1* | 8.628330134 | 223.6340273 | 25.91857565 | 4.695914532 | 7.14922E-57 | 2.47153E-54 | K18857 |
| *uncharacterized protein_00744* | 1.69387969 | 167.6943809 | 99.00017219 | 6.629359129 | 3.692E-56 | 1.25955E-53 | - |
| *ADH1* | 11.12248175 | 233.5260323 | 20.99585665 | 4.392032747 | 2.07963E-55 | 6.91291E-53 | K18857 |
| *At2g39510* | 0 | 181.6250693 | Inf | Inf | 2.17127E-46 | 5.74455E-44 | - |
| *MRF1* | 602.9827186 | 2022.105321 | 3.353504601 | 1.745669582 | 2.63851E-45 | 6.77339E-43 | - |
| *NCED3* | 433.4723509 | 1523.442865 | 3.514509892 | 1.813323516 | 6.14721E-45 | 1.54743E-42 | K09840 |
| *Os01g0656200* | 449.0501116 | 2347.533201 | 5.227775566 | 2.386197206 | 3.52899E-44 | 8.71426E-42 | K14497 |
| *uncharacterized protein_18539* | 14.45136028 | 417.4189424 | 28.88440496 | 4.852218869 | 1.72793E-43 | 4.1483E-41 | - |
| *MSL2* | 943.3815657 | 2939.423397 | 3.115837222 | 1.639619866 | 1.80247E-43 | 4.28757E-41 | - |
| *At5g04720* | 16.00681913 | 207.3564699 | 12.95425832 | 3.695354513 | 2.94991E-42 | 6.70923E-40 | - |
| *At1g75220* | 162.3821648 | 674.7961689 | 4.155605204 | 2.0550586 | 1.05027E-40 | 2.28834E-38 | K08145 |
| *COR413PM2* | 118.9739981 | 540.7182423 | 4.544843838 | 2.184230724 | 4.6403E-40 | 9.86179E-38 | - |
| *Cyp1* | 4973.926898 | 13556.52603 | 2.725517747 | 1.446530315 | 1.26275E-39 | 2.64037E-37 | K01802 |
| *Os01g0656200* | 155.3395504 | 1398.592595 | 9.003454634 | 3.170478671 | 1.46487E-38 | 2.89933E-36 | K14497 |
| *OLE9* | 204.7150646 | 753.8174753 | 3.682276518 | 1.880597969 | 6.33727E-37 | 1.17366E-34 | - |
| *LBD40* | 0.682510222 | 123.5613305 | 181.0395309 | 7.500160941 | 1.65123E-35 | 2.91245E-33 | - |
| *EMB1187* | 387.2552561 | 1191.584389 | 3.076999911 | 1.621524402 | 2.0652E-34 | 3.47705E-32 | K00894 |
| *uncharacterized protein_20905* | 736.4330248 | 2053.283021 | 2.78814631 | 1.47930627 | 7.86196E-34 | 1.31513E-31 | - |
| *ARAC11* | 148.8011014 | 562.2450119 | 3.778500338 | 1.917813752 | 1.47946E-33 | 2.39746E-31 | K04392 |
| *TSJT1* | 4.762077208 | 198.1082008 | 41.60121563 | 5.378553781 | 3.51003E-32 | 5.38509E-30 | - |
| *At5g15710* | 19.95741095 | 179.3077188 | 8.984518045 | 3.167441116 | 4.30923E-32 | 6.57234E-30 | - |
| *W09H1.5* | 344.8291033 | 1212.111397 | 3.515107586 | 1.813568846 | 8.29857E-32 | 1.25096E-29 | - |
| *EPSPS* | 2653.288633 | 6593.631197 | 2.485078749 | 1.313291569 | 8.90494E-32 | 1.33461E-29 | K00800 |
| *NIT4A* | 1250.031711 | 3204.216483 | 2.563308158 | 1.358006928 | 2.31013E-31 | 3.38401E-29 | K13035 |
| *At1g56220* | 608.3315361 | 4374.945652 | 7.19171273 | 2.846335394 | 3.71272E-31 | 5.37784E-29 | - |
| *uncharacterized protein_26773* | 24.50255952 | 344.9118976 | 14.07656605 | 3.815223529 | 3.47845E-30 | 4.90158E-28 | - |
| *PGMP* | 874.5398882 | 2857.90213 | 3.267892258 | 1.708360419 | 8.85627E-30 | 1.20223E-27 | K01835 |
| *NFYC2* | 20.22738934 | 1235.825085 | 61.09661828 | 5.933020624 | 2.82118E-29 | 3.75116E-27 | K08066 |
| *ADCS* | 978.629701 | 2393.725568 | 2.445997261 | 1.290422789 | 9.72722E-28 | 1.18407E-25 | K13950 |
| *At2g34160* | 377.4966933 | 1035.717389 | 2.743646254 | 1.456094483 | 3.01074E-27 | 3.59734E-25 | - |
| *TSJT1* | 4.700613966 | 97.38717595 | 20.71796932 | 4.372810699 | 5.70954E-27 | 6.75968E-25 | - |
| *ANR* | 184.5682474 | 579.4339706 | 3.139402247 | 1.650489891 | 9.35324E-27 | 1.09239E-24 | K08695 |
| *uncharacterized protein_19099* | 541.42532 | 1391.119665 | 2.569365735 | 1.361412264 | 1.38379E-26 | 1.59462E-24 | - |
| *Stard7* | 1652.244931 | 3953.342415 | 2.392709665 | 1.258645349 | 2.07402E-26 | 2.37943E-24 | - |
| *SYT5* | 125.4473693 | 437.2549426 | 3.485564862 | 1.801392475 | 3.34064E-26 | 3.79896E-24 | - |
| *uncharacterized protein_09249* | 1292.538215 | 3789.022728 | 2.931458958 | 1.551618858 | 7.08003E-26 | 7.84492E-24 | - |
| *uncharacterized protein_07382* | 27.34106473 | 175.3436917 | 6.4131991 | 2.681044197 | 8.93819E-26 | 9.86168E-24 | - |
| *CYP75B137* | 17.48805107 | 139.8106096 | 7.9946364 | 2.999032421 | 7.33038E-25 | 7.91925E-23 | - |
| *CCR1* | 933.4822731 | 20501.78448 | 21.96269289 | 4.456983052 | 1.28399E-24 | 1.3644E-22 | - |
| *At1g80440* | 5.012428652 | 91.17909109 | 18.19060129 | 4.185121327 | 1.44043E-24 | 1.5182E-22 | - |
| *At2g37240* | 401.2568257 | 1039.902606 | 2.591613499 | 1.373850577 | 1.48219E-24 | 1.55588E-22 | - |
| *COR2* | 235.389437 | 663.9260505 | 2.820543092 | 1.495972978 | 2.3758E-24 | 2.48386E-22 | - |
| *FAMA* | 234.5755868 | 664.6589173 | 2.833453073 | 1.502561309 | 2.39917E-24 | 2.49822E-22 | - |
| *fmdA* | 947.7316195 | 2205.223139 | 2.326843479 | 1.218374167 | 4.55447E-24 | 4.68605E-22 | K01455 |
| *RR23* | 251.2561368 | 697.8011093 | 2.777250013 | 1.473657057 | 5.14876E-24 | 5.26744E-22 | K14491 |
| *JA2L* | 93.80479248 | 3054.585111 | 32.56320951 | 5.025170997 | 5.16018E-24 | 5.26744E-22 | - |
| *BLH1* | 1926.421312 | 4254.161165 | 2.208323349 | 1.142951431 | 6.22368E-24 | 6.30343E-22 | - |
| *PGMP* | 606.151564 | 1752.145683 | 2.890606553 | 1.531372254 | 7.52712E-24 | 7.56446E-22 | K01835 |
| *uncharacterized protein_15771* | 69.2541997 | 277.42786 | 4.005935541 | 2.002139207 | 9.55027E-24 | 9.52382E-22 | - |
| *SODCP* | 3016.599907 | 6437.304093 | 2.133960184 | 1.093533258 | 6.48838E-23 | 6.02977E-21 | K04565 |
| *uncharacterized protein_47185* | 90.86298774 | 441.2087655 | 4.855758945 | 2.279696803 | 8.15619E-23 | 7.55263E-21 | - |
| *uncharacterized protein_45954* | 440.5914086 | 1079.165141 | 2.449355842 | 1.292402383 | 1.26476E-22 | 1.13079E-20 | - |
| *CRYJA* | 75.68071811 | 643.593003 | 8.504055182 | 3.088150958 | 1.47385E-22 | 1.29979E-20 | - |
| *HCT* | 1.030996417 | 62.32048329 | 60.44684759 | 5.917595197 | 1.80276E-22 | 1.5738E-20 | K13065 |
| *YPQ1* | 0.351068586 | 296.2813225 | 843.9414237 | 9.720999058 | 3.30299E-22 | 2.81711E-20 | - |
| *uncharacterized protein_36957* | 69.37919055 | 284.3743972 | 4.098842823 | 2.035216668 | 3.81502E-22 | 3.23254E-20 | - |
| *At4g33300* | 13.75852049 | 116.5874584 | 8.47383689 | 3.083015359 | 5.44327E-22 | 4.53804E-20 | - |
| *MSL3* | 73.84443326 | 275.0799226 | 3.725127412 | 1.897289772 | 6.40221E-22 | 5.3204E-20 | - |
| *Os04g0338000* | 1355.50978 | 2933.100804 | 2.163835958 | 1.113591131 | 1.01496E-21 | 8.32778E-20 | - |
| *STA* | 0.702137172 | 56.80695538 | 80.90577968 | 6.338170863 | 1.86764E-21 | 1.50854E-19 | - |
| *At4g33300* | 15.71721379 | 196.9605623 | 12.53151894 | 3.647489389 | 2.1015E-21 | 1.68172E-19 | - |
| *Patl_0974* | 25.88978931 | 150.8048428 | 5.824877173 | 2.542227629 | 3.04098E-21 | 2.38929E-19 | - |
| *uncharacterized protein_04262* | 60.23967968 | 238.2016962 | 3.954232451 | 1.983397683 | 3.57382E-21 | 2.79946E-19 | - |
| *uncharacterized protein_43448* | 104.6826056 | 355.9151189 | 3.399945166 | 1.765511479 | 4.38596E-21 | 3.3946E-19 | - |
| *GSTU20* | 72.82634881 | 280.8533224 | 3.856479517 | 1.947284448 | 6.6151E-21 | 5.0298E-19 | K00799 |
| *At1g51860* | 0 | 73.42885014 | Inf | Inf | 1.42043E-20 | 1.04332E-18 | - |
| *ARGF* | 405.1242669 | 956.7594925 | 2.36164449 | 1.239791805 | 1.95699E-20 | 1.41843E-18 | K00611 |
| *CBSX1* | 1280.247273 | 2704.706654 | 2.112643948 | 1.079049645 | 2.76348E-20 | 1.96845E-18 | - |
| *At2g19810* | 1047.006018 | 2220.827634 | 2.121122128 | 1.084827689 | 3.64842E-20 | 2.5846E-18 | - |
| *uncharacterized protein_22198* | 231.5547563 | 604.033402 | 2.608598553 | 1.383274939 | 4.42256E-20 | 3.10754E-18 | - |
| *At2g16250* | 94.08406826 | 308.0493141 | 3.274192111 | 1.711138974 | 5.70465E-20 | 3.94427E-18 | - |
| *At5g48480* | 1168.469007 | 2926.841129 | 2.504851314 | 1.324724969 | 6.63105E-20 | 4.54841E-18 | - |
| *At1g56220* | 15.15866223 | 111.9774384 | 7.387026421 | 2.884993738 | 8.16851E-20 | 5.54432E-18 | - |
| *BSPA* | 271.5900744 | 946.6270283 | 3.485499352 | 1.801365359 | 9.27786E-20 | 6.24822E-18 | - |
| *AMT1-1* | 232.4798053 | 594.6690108 | 2.557938355 | 1.354981497 | 9.67247E-20 | 6.4803E-18 | K03320 |
| *At4g33300* | 399.6970899 | 1166.989756 | 2.919685397 | 1.545812924 | 1.11479E-19 | 7.41138E-18 | - |
| *ICI1* | 279.1290453 | 685.7014444 | 2.456575036 | 1.296648307 | 1.27935E-19 | 8.3977E-18 | - |
| *LKHA4* | 363.4871551 | 859.0739607 | 2.36342316 | 1.24087796 | 1.51403E-19 | 9.86323E-18 | K01254 |
| *AATP2* | 814.1752138 | 1731.435944 | 2.12661343 | 1.088557808 | 5.547E-19 | 3.49228E-17 | K03301 |
| *D6PKL1* | 69.46529725 | 1460.21733 | 21.02081742 | 4.393746866 | 6.92013E-19 | 4.3235E-17 | - |
| *Os01g0794400* | 451.7994097 | 1210.811559 | 2.679975964 | 1.422220062 | 7.0803E-19 | 4.41293E-17 | K17609 |
| *LDOX* | 1472.677537 | 3408.645763 | 2.314590722 | 1.210757111 | 7.74712E-19 | 4.81696E-17 | K05277 |
| *TSJT1* | 555.4686508 | 3786.45937 | 6.816693192 | 2.769072051 | 8.20257E-19 | 5.08795E-17 | - |
| *CSA* | 126.1216144 | 2786.056221 | 22.09023596 | 4.465336924 | 8.47246E-19 | 5.24281E-17 | K00432 |
| *RBCS* | 1857.738299 | 4022.388225 | 2.165207138 | 1.114505049 | 1.09091E-18 | 6.62415E-17 | K01602 |
| *ATHB-13* | 17.79470097 | 114.7770923 | 6.450071425 | 2.689315136 | 1.92977E-18 | 1.15555E-16 | K09338 |
| *apaG* | 918.0730418 | 2007.12032 | 2.186231627 | 1.12844626 | 2.31518E-18 | 1.37679E-16 | K06195 |
| *uncharacterized protein_38317* | 109.4235056 | 328.5877653 | 3.002899271 | 1.586356082 | 2.57697E-18 | 1.52897E-16 | - |
| *uncharacterized protein_25948* | 367.4508149 | 837.1273531 | 2.278202467 | 1.187895967 | 2.74332E-18 | 1.62394E-16 | K03122 |
| *uncharacterized protein_46371* | 368.3255275 | 846.406908 | 2.297986007 | 1.200370013 | 2.98505E-18 | 1.75901E-16 | - |
| *BEH3* | 190.1374598 | 488.079943 | 2.566984662 | 1.360074676 | 3.09943E-18 | 1.81814E-16 | - |
| *ACX2* | 497.4568379 | 1354.073699 | 2.721992333 | 1.444663003 | 3.40558E-18 | 1.97982E-16 | K00232 |
| *OAT* | 40.97510852 | 169.920463 | 4.14691917 | 2.052039927 | 1.75909E-17 | 9.91516E-16 | K00819 |
| *NSN1* | 306.5629278 | 712.0726832 | 2.322761882 | 1.215841264 | 1.79777E-17 | 1.01112E-15 | K14538 |
| *sll0005* | 680.2881715 | 1428.260794 | 2.099493793 | 1.070041523 | 2.00473E-17 | 1.12265E-15 | K08869 |
| *GLYI4* | 608.8771757 | 1648.596373 | 2.707600874 | 1.437015087 | 3.14227E-17 | 1.73346E-15 | - |
| *eIF2gamma* | 173.0282173 | 443.7850664 | 2.564813261 | 1.35885379 | 3.8311E-17 | 2.10006E-15 | K03242 |
| *BLOS2* | 149.3567702 | 394.5099868 | 2.641393399 | 1.401299187 | 4.61386E-17 | 2.51849E-15 | K16750 |
| *CRJ34* | 14.39247943 | 122.0095461 | 8.477312524 | 3.083606974 | 6.91396E-17 | 3.69619E-15 | - |
| *AHRI* | 402.9858345 | 875.3326731 | 2.172117723 | 1.119102295 | 7.52592E-17 | 3.99861E-15 | K00053 |
| *ATL8* | 14.96977403 | 134.6877714 | 8.997314933 | 3.169494522 | 1.24353E-16 | 6.47437E-15 | K19040 |
| *IRL1* | 137.0402316 | 369.1487692 | 2.693725521 | 1.429602854 | 1.30013E-16 | 6.72848E-15 | - |
| *tfa2* | 452.7512359 | 967.7669101 | 2.137524613 | 1.095941032 | 2.13406E-16 | 1.08921E-14 | K03137 |
| *RAB28* | 14.14212798 | 100.3934222 | 7.098890799 | 2.827593621 | 2.28656E-16 | 1.16247E-14 | - |
| *ACR4* | 0.660300881 | 275.5913031 | 417.3723087 | 8.705191075 | 2.54462E-16 | 1.2861E-14 | - |
| *ERD7* | 133.6070175 | 355.2206376 | 2.658697458 | 1.410719617 | 2.58875E-16 | 1.30586E-14 | K19366 |
| *Os12g0192500* | 19.00337212 | 109.0432432 | 5.738099666 | 2.520573027 | 2.90353E-16 | 1.45615E-14 | - |
| *HPPD* | 1405.77632 | 4714.273596 | 3.353501926 | 1.74566843 | 4.03039E-16 | 1.98292E-14 | K00457 |
| *PCMP-H42* | 138.3899059 | 362.0148055 | 2.6159047 | 1.387309983 | 4.36149E-16 | 2.12966E-14 | - |
| *At1g56220* | 91.26637433 | 269.0400211 | 2.947854816 | 1.559665472 | 4.90534E-16 | 2.36845E-14 | - |
| *IAA30* | 495.2770044 | 1032.196215 | 2.084078619 | 1.059409702 | 6.18191E-16 | 2.95728E-14 | K14484 |
| *MSRB2* | 2300.813582 | 4679.474508 | 2.033834703 | 1.024202431 | 8.17805E-16 | 3.87642E-14 | K07305 |
| *ABCG14* | 6.667054442 | 171.7987824 | 25.76831852 | 4.687526494 | 1.50791E-15 | 7.00664E-14 | - |
| *PMLN* | 49.2845431 | 177.0692231 | 3.592794251 | 1.845106319 | 2.31637E-15 | 1.05737E-13 | - |
| *hxnY* | 60.75912562 | 266.4856155 | 4.385935656 | 2.132884647 | 2.69212E-15 | 1.22458E-13 | - |
| *GGCT2;2* | 657.1192913 | 1317.46503 | 2.004909987 | 1.003537467 | 3.23797E-15 | 1.44748E-13 | - |
| *SBE1* | 332.3704426 | 832.4364727 | 2.504544225 | 1.324548087 | 3.46602E-15 | 1.54146E-13 | K00700 |
| *PIN2* | 210.490358 | 543.8804794 | 2.583873601 | 1.369535497 | 3.82742E-15 | 1.69636E-13 | K13947 |
| *At5g01750* | 186.8786756 | 456.5987796 | 2.443289894 | 1.288825048 | 4.53615E-15 | 2.00023E-13 | - |
| *uncharacterized protein_03044* | 0.351068586 | 43.21054848 | 123.082925 | 6.943486824 | 7.61986E-15 | 3.28732E-13 | - |
| *uncharacterized protein_42569* | 14.40332702 | 89.71206805 | 6.22856566 | 2.638899972 | 8.29015E-15 | 3.56463E-13 | - |
| *PAO* | 1645.689515 | 3547.487816 | 2.155624001 | 1.108105555 | 1.83667E-14 | 7.66847E-13 | K13071 |
| *NRPB1* | 8.473531048 | 112.4211109 | 13.26732743 | 3.729805878 | 2.24338E-14 | 9.24042E-13 | K03006 |
| *uncharacterized protein_24130* | 0 | 33.11967413 | Inf | Inf | 2.30252E-14 | 9.44617E-13 | - |
| *CRPK1* | 19.83809904 | 101.4359644 | 5.113189737 | 2.354223562 | 2.92985E-14 | 1.19068E-12 | - |
| *CHS* | 682.5251257 | 2122.98087 | 3.110480172 | 1.637137309 | 3.8654E-14 | 1.54664E-12 | K00660 |
| *HMT-2* | 404.7412428 | 824.9741105 | 2.03827538 | 1.027348979 | 6.13301E-14 | 2.40934E-12 | K00547 |
| *PAA2* | 248.5215974 | 575.9078321 | 2.317335145 | 1.212466709 | 7.09966E-14 | 2.77229E-12 | K01533 |
| *TRZ1* | 14.10287408 | 85.06128048 | 6.031485497 | 2.592513368 | 8.69218E-14 | 3.36877E-12 | K00784 |
| *NBR1* | 367.39038 | 751.1657706 | 2.044598366 | 1.031817473 | 9.18171E-14 | 3.55318E-12 | K17987 |
| *ncd-2* | 43.4828384 | 153.3424402 | 3.526504844 | 1.818239021 | 1.18909E-13 | 4.5675E-12 | - |
| *ERD4* | 682.8829054 | 1414.751253 | 2.071733297 | 1.050838291 | 1.37543E-13 | 5.25217E-12 | - |
| *pgk* | 225.4309112 | 495.0863653 | 2.196177812 | 1.134994866 | 1.50204E-13 | 5.71877E-12 | K00927 |
| *GEK1* | 257.6466462 | 550.0310893 | 2.134827282 | 1.094119353 | 1.5824E-13 | 6.0076E-12 | K09716 |
| *Ttc39a* | 341.6080882 | 701.7199992 | 2.054166817 | 1.038553346 | 1.76842E-13 | 6.65479E-12 | - |
| *CA2* | 2.086784567 | 372.0320207 | 178.2800326 | 7.47800132 | 1.81327E-13 | 6.81367E-12 | K15746 |
| *CSE* | 35.6766891 | 134.4314005 | 3.768045856 | 1.913816522 | 1.9364E-13 | 7.25535E-12 | - |
| *FG2* | 7.340267281 | 210.1379021 | 28.6280995 | 4.839359994 | 2.24063E-13 | 8.2993E-12 | - |
| *Dctpp1* | 11.89899412 | 89.91982723 | 7.556926773 | 2.917799643 | 2.93274E-13 | 1.06648E-11 | K16904 |
| *At1g48650* | 277.5799317 | 575.3492808 | 2.072733707 | 1.051534779 | 3.91743E-13 | 1.40292E-11 | K13026 |
| *uncharacterized protein_37718* | 171.8009514 | 391.5341306 | 2.278998617 | 1.188400049 | 4.03469E-13 | 1.44093E-11 | - |
| *AKR4C9* | 108.2814625 | 276.0511354 | 2.549384992 | 1.350149256 | 4.2418E-13 | 1.51073E-11 | - |
| *DCR* | 18.79832326 | 93.89405774 | 4.994810251 | 2.320429872 | 4.36137E-13 | 1.54694E-11 | K19747 |
| *uncharacterized protein_24093* | 30.23520209 | 120.1201275 | 3.972856775 | 1.990176784 | 4.96586E-13 | 1.74939E-11 | - |
| *CYP750A1* | 0 | 1400.118617 | Inf | Inf | 5.0155E-13 | 1.76448E-11 | - |
| *COR2* | 138.793358 | 365.2099167 | 2.631321282 | 1.395787411 | 5.59191E-13 | 1.95928E-11 | - |
| *GDH1* | 47.22188396 | 157.0626327 | 3.326056046 | 1.733812479 | 6.16959E-13 | 2.15877E-11 | K00261 |
| *MC410* | 272.5387308 | 566.5230106 | 2.078688078 | 1.055673288 | 7.18935E-13 | 2.50545E-11 | - |
| *RZPF34* | 281.7395567 | 578.3894915 | 2.052922559 | 1.037679206 | 7.42134E-13 | 2.57936E-11 | K10144 |
| *NUP133* | 261.7215664 | 1177.142182 | 4.497688891 | 2.169183872 | 1.03501E-12 | 3.56385E-11 | - |
| *CLINT1* | 103.7253181 | 264.2459031 | 2.547554522 | 1.349113023 | 1.06196E-12 | 3.65178E-11 | K12471 |
| *RECA* | 328.4125523 | 661.0765425 | 2.012945418 | 1.009308053 | 1.20514E-12 | 4.12772E-11 | K03553 |
| *rpsF* | 203.244089 | 502.2022292 | 2.470931537 | 1.305055038 | 1.2231E-12 | 4.18371E-11 | - |
| *PIRL4* | 30.31437619 | 117.7845545 | 3.885435535 | 1.958076326 | 1.24697E-12 | 4.25973E-11 | - |
| *CYP750A1* | 0 | 44.9238139 | Inf | Inf | 1.37678E-12 | 4.68465E-11 | - |
| *Hdhd3* | 227.9775252 | 544.1230974 | 2.386740083 | 1.255041465 | 1.60624E-12 | 5.42979E-11 | - |
| *CYP707A1* | 0.331441636 | 31.36124736 | 94.62072332 | 6.564084284 | 1.84628E-12 | 6.20888E-11 | K09843 |
| *Dctpp1* | 2.064575226 | 40.27305131 | 19.50670085 | 4.285897892 | 2.62814E-12 | 8.69162E-11 | K16904 |
| *ATPD* | 316.6099945 | 1067.662507 | 3.372169312 | 1.753676974 | 2.96722E-12 | 9.7385E-11 | K02113 |
| *SAT5* | 185.3154696 | 404.6651665 | 2.183655619 | 1.126745349 | 3.03954E-12 | 9.96325E-11 | K00640 |
| *At3g22104* | 62.02756152 | 193.2972481 | 3.116312222 | 1.639839784 | 3.1835E-12 | 1.03957E-10 | - |
| *CPL4* | 7.383653781 | 59.30055753 | 8.031329649 | 3.005638857 | 3.38246E-12 | 1.099E-10 | K18999 |
| *CYP750A1* | 0 | 85.09757851 | Inf | Inf | 5.52073E-12 | 1.76282E-10 | - |
| *USF* | 1291.292732 | 2837.145542 | 2.197135841 | 1.135624069 | 6.16758E-12 | 1.95732E-10 | K01061 |
| *Galm* | 243.4470392 | 1837.891948 | 7.549452864 | 2.916372091 | 6.62945E-12 | 2.09876E-10 | K01785 |
| *NRAMP2* | 241.7870311 | 604.3286997 | 2.499425618 | 1.321596594 | 6.68297E-12 | 2.11312E-10 | K12347 |
| *Os03g0326500* | 1091.565674 | 2191.489948 | 2.007657442 | 1.005513129 | 6.85206E-12 | 2.16395E-10 | - |
| *ADT2* | 22.87014307 | 98.69490466 | 4.315447627 | 2.109510213 | 7.04041E-12 | 2.21803E-10 | K05359 |
| *Pnpla2* | 1277.998015 | 3242.298498 | 2.537013719 | 1.343131321 | 9.22555E-12 | 2.86467E-10 | - |
| *uncharacterized protein_45269* | 5.756402116 | 105.4529993 | 18.31925518 | 4.195288943 | 9.30071E-12 | 2.88293E-10 | - |
| *uncharacterized protein_38539* | 182.2713975 | 393.5770339 | 2.159291251 | 1.110557851 | 1.00569E-11 | 3.10055E-10 | K14315 |
| *uncharacterized protein_00735* | 364.5425774 | 910.6623898 | 2.498096097 | 1.320828976 | 1.01056E-11 | 3.11187E-10 | - |
| *ARF19* | 204.4405877 | 431.2074187 | 2.109206511 | 1.076700355 | 1.02461E-11 | 3.15139E-10 | - |
| *MSL6* | 232.8288056 | 790.4147216 | 3.394832179 | 1.763340258 | 1.15158E-11 | 3.52934E-10 | - |
| *BGAL* | 24.22689832 | 300.0523163 | 12.38509001 | 3.630532448 | 1.30466E-11 | 3.97032E-10 | - |
| *CHI4* | 6.171516336 | 1745.878918 | 282.8930238 | 8.14411279 | 1.60716E-11 | 4.83724E-10 | K01183 |
| *RAMDAZC7* | 181.113342 | 429.3159375 | 2.370426899 | 1.245146903 | 1.6129E-11 | 4.84124E-10 | - |
| *nsa2* | 257.8230711 | 516.5012356 | 2.00331659 | 1.002390432 | 2.35215E-11 | 6.96193E-10 | K14842 |
| *PCK* | 123.7675821 | 286.1634116 | 2.312103111 | 1.209205738 | 2.45194E-11 | 7.23253E-10 | K01610 |
| *GRDP2* | 18.53092726 | 201.8330111 | 10.89168438 | 3.445155177 | 2.52588E-11 | 7.41688E-10 | - |
| *AHRI* | 2133.418543 | 4744.93592 | 2.224099877 | 1.153221576 | 2.59364E-11 | 7.59006E-10 | K00053 |
| *Def1* | 6.377449097 | 54.3866952 | 8.527970098 | 3.09220238 | 3.23123E-11 | 9.35038E-10 | - |
| *CIPK32* | 62.05685173 | 174.5959544 | 2.813483919 | 1.492357717 | 3.33452E-11 | 9.63851E-10 | - |
| *PAR* | 65.12158296 | 207.2671406 | 3.182771843 | 1.670283741 | 3.73588E-11 | 1.07507E-09 | - |
| *FBX6* | 38.35766455 | 125.7387509 | 3.278060654 | 1.712842549 | 3.96728E-11 | 1.13787E-09 | - |
| *RPL13B* | 131.6696535 | 299.3756843 | 2.273687797 | 1.18503417 | 4.26232E-11 | 1.21711E-09 | K02873 |
| *uncharacterized protein_23012* | 158.5696279 | 342.9139588 | 2.162545018 | 1.112730166 | 4.35482E-11 | 1.24079E-09 | - |
| *HSP22* | 77.23219264 | 201.4397146 | 2.608235086 | 1.383073908 | 4.83496E-11 | 1.37156E-09 | K13993 |
| *At5g08460* | 22.98480436 | 93.88497112 | 4.084653916 | 2.030213847 | 5.72862E-11 | 1.61447E-09 | - |
| *SMC1* | 757.4608305 | 1760.722037 | 2.324505725 | 1.216923979 | 6.34363E-11 | 1.77048E-09 | K06636 |
| *CYP97C1* | 211.3627886 | 429.1904586 | 2.030586658 | 1.021896597 | 6.522E-11 | 1.81831E-09 | K09837 |
| *ATJ8* | 161.3584014 | 346.3799405 | 2.146649555 | 1.102086688 | 6.61418E-11 | 1.84202E-09 | - |
| *At3g19810* | 108.4572212 | 254.3165332 | 2.3448557 | 1.229499143 | 6.79259E-11 | 1.88766E-09 | - |
| *BAG6* | 1779.474794 | 3854.053632 | 2.16583772 | 1.11492515 | 8.60593E-11 | 2.36121E-09 | - |
| *uncharacterized protein_30277* | 1263.499288 | 2785.447319 | 2.204549971 | 1.140484179 | 9.39146E-11 | 2.56048E-09 | - |
| *uncharacterized protein_32300* | 78.35460882 | 199.5334386 | 2.54654374 | 1.348540498 | 1.00539E-10 | 2.73532E-09 | - |
| *ROPGEF8* | 42.46150524 | 131.3505471 | 3.093402987 | 1.62919479 | 1.00941E-10 | 2.7434E-09 | - |
| *MYB2* | 3.017063843 | 44.84920676 | 14.8651832 | 3.893865338 | 1.03224E-10 | 2.80133E-09 | K09422 |
| *PME51* | 11.39762492 | 64.91232876 | 5.695250474 | 2.509759292 | 1.28538E-10 | 3.43936E-09 | - |
| *XYLA* | 156.04707 | 432.2519735 | 2.770010187 | 1.469891282 | 1.33182E-10 | 3.54897E-09 | K01805 |
| *TOP6B* | 342.8231124 | 760.6576276 | 2.218804976 | 1.149782866 | 1.41752E-10 | 3.75802E-09 | - |
| *uncharacterized protein_34176* | 7.836471692 | 54.15179955 | 6.910227163 | 2.788733138 | 1.5967E-10 | 4.20298E-09 | - |
| *uncharacterized protein_24004* | 0 | 42.17830963 | Inf | Inf | 1.72136E-10 | 4.51278E-09 | - |
| *PYD2* | 695.8613644 | 1619.279554 | 2.327014599 | 1.218480262 | 1.77846E-10 | 4.64837E-09 | K01464 |
| *Hgsnat* | 130.7460853 | 287.2702583 | 2.197161449 | 1.135640884 | 1.85598E-10 | 4.84123E-09 | K10532 |
| *BAM1* | 2199.058688 | 4514.448974 | 2.052900633 | 1.037663798 | 2.17307E-10 | 5.64562E-09 | K01177 |
| *uncharacterized protein_31661* | 46.6355924 | 137.6105464 | 2.950762269 | 1.561087693 | 2.17591E-10 | 5.64736E-09 | - |
| *PME51* | 20.38027234 | 84.01439292 | 4.122339069 | 2.043463174 | 2.52186E-10 | 6.50614E-09 | - |
| *At5g48740* | 0 | 28.97946963 | Inf | Inf | 2.66609E-10 | 6.86459E-09 | - |
| *UNC* | 3.075944693 | 37.90368716 | 12.32261661 | 3.623236728 | 2.90261E-10 | 7.43664E-09 | - |
| *DG1* | 110.3687651 | 252.8889368 | 2.291308928 | 1.196171984 | 3.25219E-10 | 8.26694E-09 | - |
| *RR23* | 0.331441636 | 25.59020603 | 77.20878499 | 6.270693105 | 3.54632E-10 | 8.96189E-09 | K14491 |
| *Dnajc28* | 197.9537865 | 441.3229461 | 2.229424119 | 1.156671097 | 3.67968E-10 | 9.28081E-09 | K19373 |
| *uncharacterized protein_51568* | 200.4290468 | 456.1180367 | 2.275708256 | 1.186315617 | 3.77906E-10 | 9.52219E-09 | - |
| *XYL1* | 792.5796382 | 2439.706639 | 3.078184856 | 1.622079873 | 4.01783E-10 | 1.00749E-08 | K15925 |
| *DLO1* | 57.48573096 | 158.0406485 | 2.749215255 | 1.45901987 | 4.30833E-10 | 1.07513E-08 | - |
| *NAC025* | 27.35655908 | 98.49163921 | 3.600293404 | 1.848114483 | 4.38749E-10 | 1.09384E-08 | - |
| *CYP707A1* | 0.351068586 | 24.94311974 | 71.04913603 | 6.150745201 | 4.57257E-10 | 1.13888E-08 | K09843 |
| *CYP75B137* | 18.17985867 | 78.31336584 | 4.307699374 | 2.106917571 | 4.635E-10 | 1.15332E-08 | - |
| *NRPB4* | 147.9495475 | 314.0450469 | 2.122649593 | 1.085866231 | 4.69022E-10 | 1.16594E-08 | K03012 |
| *PCBER* | 137.1466277 | 338.3649898 | 2.467176886 | 1.302861154 | 5.00447E-10 | 1.23695E-08 | - |
| *BZIP53* | 3.736245574 | 39.75581432 | 10.6405785 | 3.411504683 | 5.7042E-10 | 1.39923E-08 | - |
| *BETA-OHASE 1* | 0 | 22.20327596 | Inf | Inf | 5.7826E-10 | 1.41445E-08 | K15746 |
| *OAT* | 10.34183677 | 59.80533099 | 5.782853887 | 2.531781651 | 6.15916E-10 | 1.50372E-08 | K00819 |
| *EXPA8* | 88.06920165 | 423.0794283 | 4.80394304 | 2.264219045 | 6.40212E-10 | 1.55571E-08 | - |
| *CA2* | 3.343340696 | 38.25941968 | 11.44347022 | 3.516452709 | 6.61427E-10 | 1.60425E-08 | K15746 |
| *At3g21360* | 89.7727446 | 214.357355 | 2.387777671 | 1.255668512 | 6.66314E-10 | 1.6146E-08 | - |
| *SSL4* | 47.94519829 | 171.1198177 | 3.569071018 | 1.835548609 | 7.18369E-10 | 1.73103E-08 | - |
| *GFA2* | 184.1982182 | 368.8350437 | 2.00238117 | 1.00171663 | 8.88665E-10 | 2.11972E-08 | K03686 |
| *At1g54570* | 529.8324578 | 1074.265555 | 2.027557088 | 1.019742536 | 8.93054E-10 | 2.12823E-08 | - |
| *MMS21* | 66.77259417 | 171.3388736 | 2.566005945 | 1.359524513 | 9.34054E-10 | 2.22185E-08 | - |
| *CHI4* | 0 | 91.95481147 | Inf | Inf | 9.49792E-10 | 2.2542E-08 | K01183 |
| *GSTL3* | 134.1340623 | 287.5727355 | 2.143920274 | 1.100251257 | 1.24608E-09 | 2.91855E-08 | K00799 |
| *MYBS3* | 168.9300594 | 434.5173589 | 2.572173125 | 1.362987749 | 1.60896E-09 | 3.72142E-08 | - |
| *At5g01750* | 313.9613442 | 859.7221933 | 2.738305875 | 1.453283608 | 1.647E-09 | 3.806E-08 | - |
| *CIPK5* | 90.72538149 | 934.8439906 | 10.30410647 | 3.365147501 | 1.85636E-09 | 4.26699E-08 | K07198 |
| *uncharacterized protein_43266* | 146.4390254 | 305.0112151 | 2.082854719 | 1.058562214 | 1.87283E-09 | 4.30104E-08 | - |
| *ANT1* | 15.38577215 | 168.0547298 | 10.92273616 | 3.449262393 | 1.90113E-09 | 4.36217E-08 | K14209 |
| *uncharacterized protein_40515* | 97.96596768 | 222.0179134 | 2.266275918 | 1.180323519 | 2.11685E-09 | 4.83149E-08 | - |
| *ATJ8* | 0 | 21.13823938 | Inf | Inf | 2.49965E-09 | 5.63084E-08 | - |
| *GBF* | 135.2291043 | 283.2813049 | 2.094824973 | 1.066829709 | 2.6743E-09 | 6.0034E-08 | K06630 |
| *ORRM2* | 351.7845788 | 718.8084552 | 2.043319971 | 1.030915139 | 2.74704E-09 | 6.16135E-08 | - |
| *At4g33300* | 12.49783176 | 61.81561068 | 4.946106803 | 2.306293393 | 2.88546E-09 | 6.45507E-08 | - |
| *dnaJ* | 118.0063849 | 254.0624709 | 2.152955292 | 1.106318361 | 2.98422E-09 | 6.65303E-08 | - |
| *TH* | 7.400698339 | 55.95427087 | 7.560674453 | 2.918514936 | 4.85815E-09 | 1.06029E-07 | K00500 |
| *PHOS34* | 6.083711153 | 53.60181479 | 8.810710016 | 3.139258284 | 5.03655E-09 | 1.09737E-07 | - |
| *uncharacterized protein_41869* | 1.053205759 | 26.06230589 | 24.74569255 | 4.629105514 | 5.21701E-09 | 1.13384E-07 | - |
| *XYLA* | 90.92165099 | 233.3591391 | 2.566595926 | 1.359856182 | 5.70141E-09 | 1.23505E-07 | K01805 |
| *CAISE5* | 25.37462425 | 87.98519731 | 3.467448283 | 1.793874365 | 5.92934E-09 | 1.2822E-07 | - |
| *CBSPPR1* | 134.4971589 | 277.5283134 | 2.063451122 | 1.045059265 | 6.27374E-09 | 1.35555E-07 | - |
| *ZDS* | 387.4246002 | 778.5413642 | 2.009530019 | 1.006858129 | 7.46546E-09 | 1.59312E-07 | K00514 |
| *uncharacterized protein_42611* | 18.2072328 | 111.4399164 | 6.120639949 | 2.613682503 | 7.76521E-09 | 1.65437E-07 | - |
| *OBGL* | 2432.849802 | 5003.753006 | 2.056745551 | 1.040363323 | 7.8905E-09 | 1.67968E-07 | - |
| *uncharacterized protein_15056* | 54.94627676 | 143.3996134 | 2.609814929 | 1.383947504 | 8.22541E-09 | 1.74524E-07 | - |
| *PCMP-E61* | 73.41330667 | 175.6563981 | 2.392705166 | 1.258642636 | 8.2971E-09 | 1.75645E-07 | - |
| *BGLU1* | 544.6145972 | 1150.50883 | 2.112519268 | 1.0789645 | 9.48884E-09 | 1.99697E-07 | K05350 |
| *ELI_A* | 1798.440318 | 3781.997108 | 2.102931674 | 1.072401976 | 9.72027E-09 | 2.03412E-07 | - |
| *UGT85A1* | 8.083208562 | 48.10859609 | 5.951670765 | 2.573294722 | 1.00846E-08 | 2.10527E-07 | - |
| *uncharacterized protein_34236* | 137.9033648 | 281.275519 | 2.039656678 | 1.028326333 | 1.01191E-08 | 2.10906E-07 | - |
| *Acx* | 30.06010975 | 96.01161817 | 3.193987613 | 1.675358718 | 1.10313E-08 | 2.28998E-07 | K00232 |
| *At3g20650* | 81.79453406 | 188.4272077 | 2.30366503 | 1.203930953 | 1.19762E-08 | 2.47821E-07 | K00565 |
| *uncharacterized protein_44007* | 1683.583361 | 4551.725911 | 2.703594022 | 1.434878529 | 1.20096E-08 | 2.48314E-07 | K17710 |
| *SLAH3* | 84.3529489 | 223.2196031 | 2.646257256 | 1.40395332 | 1.25955E-08 | 2.59393E-07 | - |
| *uncharacterized protein_08775* | 9.074951079 | 67.79669477 | 7.47075044 | 2.90125317 | 1.36931E-08 | 2.81327E-07 | - |
| *ILL1* | 120.335404 | 250.5015478 | 2.081694493 | 1.057758356 | 1.38322E-08 | 2.83961E-07 | K14664 |
| *Os03g0255100* | 49.25081986 | 294.881039 | 5.987332592 | 2.581913413 | 1.53486E-08 | 3.13601E-07 | - |
| *SLC25A24* | 64.91136931 | 273.5800019 | 4.214670016 | 2.075419681 | 1.69099E-08 | 3.428E-07 | - |
| *AKR2A* | 6841.042407 | 16545.95186 | 2.418630213 | 1.274190211 | 1.74516E-08 | 3.52952E-07 | - |
| *SWEET1A* | 115.6120665 | 499.0474091 | 4.316568538 | 2.109884896 | 1.75122E-08 | 3.53902E-07 | K15382 |
| *At4g16230* | 402.0354102 | 962.8181867 | 2.394859165 | 1.259940818 | 1.75298E-08 | 3.53983E-07 | - |
| *Pole3* | 145.8640956 | 334.0280246 | 2.289994829 | 1.195344341 | 1.89107E-08 | 3.80386E-07 | K02326 |
| *At5g13200* | 79.81658355 | 1501.778263 | 18.81536639 | 4.233839478 | 1.92928E-08 | 3.8747E-07 | - |
| *BAM4* | 958.2156361 | 2516.518261 | 2.626254641 | 1.393006806 | 2.04417E-08 | 4.09909E-07 | K01177 |
| *Os01g0810000* | 0 | 18.60737075 | Inf | Inf | 2.04702E-08 | 4.10162E-07 | K14769 |
| *SAMDC* | 128.3643823 | 347.3920685 | 2.706296421 | 1.436319866 | 2.19789E-08 | 4.37687E-07 | K01611 |
| *HSD_PINMS* | 0.328859245 | 20.80398285 | 63.26105527 | 5.983245717 | 2.40811E-08 | 4.7735E-07 | - |
| *PGMP* | 40.55327931 | 114.9181642 | 2.833757617 | 1.502716364 | 2.42778E-08 | 4.80882E-07 | K01835 |
| *SDHAF4* | 94.65309766 | 205.6556937 | 2.172730728 | 1.119509388 | 2.93466E-08 | 5.75131E-07 | - |
| *TOGT1* | 27.09174547 | 345.8909099 | 12.7673911 | 3.674391848 | 2.99084E-08 | 5.84815E-07 | - |
| *ATJ8* | 16.95067667 | 67.27831598 | 3.969063731 | 1.988798728 | 2.99561E-08 | 5.85306E-07 | - |
| *CYP720B1* | 476.6638854 | 1343.532175 | 2.818615414 | 1.494986643 | 3.18281E-08 | 6.1862E-07 | - |
| *CSLE6* | 216.5570246 | 553.333879 | 2.555141677 | 1.353403287 | 3.45615E-08 | 6.67742E-07 | - |
| *CRF2* | 22.43710039 | 82.77883865 | 3.689373279 | 1.883375764 | 4.66762E-08 | 8.81443E-07 | - |
| *TMEM205* | 96.70461265 | 285.8853414 | 2.956274097 | 1.563780038 | 4.93532E-08 | 9.24588E-07 | - |
| *DODA* | 0 | 47.93807917 | Inf | Inf | 5.03247E-08 | 9.41427E-07 | K15777 |
| *uncharacterized protein_47075* | 112.0946657 | 233.3273113 | 2.081520203 | 1.057637561 | 5.63079E-08 | 1.04656E-06 | - |
| *PRCP* | 125.282389 | 250.7669569 | 2.001613786 | 1.001163631 | 5.87702E-08 | 1.08998E-06 | K01285 |
| *PME51* | 30.4247566 | 91.77873169 | 3.016580639 | 1.592914149 | 6.55467E-08 | 1.20703E-06 | - |
| *At1g31830* | 54.37909413 | 134.2992427 | 2.469685177 | 1.304327146 | 6.60669E-08 | 1.21574E-06 | - |
| *SUR1* | 94.04584654 | 209.5757997 | 2.228442907 | 1.156035999 | 6.68509E-08 | 1.22843E-06 | K00815 |
| *uncharacterized protein_18332* | 3.004151885 | 85.10994214 | 28.33077201 | 4.824298011 | 7.05428E-08 | 1.2926E-06 | - |
| *DLO1* | 234.7506792 | 489.8979838 | 2.086886332 | 1.061352022 | 7.06008E-08 | 1.29275E-06 | - |
| *Os04g0338000* | 55.48689986 | 166.8823633 | 3.007599338 | 1.588612389 | 7.45167E-08 | 1.35774E-06 | - |
| *PHOS34* | 302.7252295 | 744.3038183 | 2.458677856 | 1.297882721 | 7.49307E-08 | 1.36337E-06 | - |
| *KAO2* | 161.5002192 | 455.889695 | 2.822842578 | 1.497148676 | 7.69939E-08 | 1.39504E-06 | K04123 |
| *At2g02240* | 15.91038287 | 62.1601206 | 3.906890306 | 1.966020749 | 7.86988E-08 | 1.42195E-06 | - |
| *HHT1* | 0 | 47.34732762 | Inf | Inf | 9.07228E-08 | 1.62113E-06 | K15400 |
| *SELENOF* | 114.2978793 | 231.0495108 | 2.021468046 | 1.015403399 | 9.22387E-08 | 1.64708E-06 | - |
| *CYP750A1* | 0 | 209.6088457 | Inf | Inf | 9.55597E-08 | 1.70404E-06 | - |
| *bsn* | 62.46680117 | 146.1880939 | 2.3402526 | 1.226664259 | 9.84032E-08 | 1.75233E-06 | - |
| *CPK17* | 230.0899893 | 592.5064601 | 2.575107513 | 1.364632667 | 1.00149E-07 | 1.78098E-06 | K13412 |
| *At5g48740* | 0 | 17.79426127 | Inf | Inf | 1.00435E-07 | 1.78483E-06 | - |
| *SB09* | 28.1954186 | 223.3494751 | 7.921481084 | 2.985770197 | 1.01222E-07 | 1.79636E-06 | K03671 |
| *At5g01750* | 29.28891044 | 89.46123354 | 3.054440476 | 1.610908126 | 1.02227E-07 | 1.81296E-06 | - |
| *CBSX6* | 28.4634809 | 87.30302971 | 3.067194417 | 1.616919616 | 1.03699E-07 | 1.83529E-06 | - |
| *At3g01520* | 95.48059543 | 202.3511173 | 2.119290484 | 1.083581347 | 1.07823E-07 | 1.90235E-06 | - |
| *Prcp* | 115.6054998 | 231.2515603 | 2.000350854 | 1.000253065 | 1.40922E-07 | 2.44895E-06 | K01285 |
| *At4g19900* | 6.691846175 | 49.43896754 | 7.387941421 | 2.885172427 | 1.79947E-07 | 3.07153E-06 | K01988 |
| *EMB2001* | 89.32265736 | 188.0591351 | 2.105391181 | 1.074088311 | 1.88042E-07 | 3.20338E-06 | K03978 |
| *RR41* | 46.62887741 | 149.1516107 | 3.198696151 | 1.677483955 | 1.9933E-07 | 3.37793E-06 | - |
| *uncharacterized protein_25685* | 89.89773545 | 540.3850012 | 6.011108049 | 2.587630953 | 2.09968E-07 | 3.54431E-06 | - |
| *KCS1* | 0.331441636 | 18.47861404 | 55.75224118 | 5.800957896 | 2.27034E-07 | 3.81252E-06 | K15397 |
| *GH5FP* | 10.39813523 | 48.77261467 | 4.690515518 | 2.229746493 | 2.33503E-07 | 3.91609E-06 | K01179 |
| *CRK19* | 726.4985512 | 1544.929371 | 2.126541572 | 1.088509058 | 2.5317E-07 | 4.21592E-06 | - |
| *CRY2* | 35.09024926 | 95.80949527 | 2.730373744 | 1.449098446 | 2.55153E-07 | 4.24622E-06 | K12119 |
| *uncharacterized protein_00488* | 56.33520504 | 170.5414063 | 3.027261659 | 1.598013379 | 2.56315E-07 | 4.26009E-06 | - |
| *At5g08460* | 19.19226032 | 82.98543107 | 4.323900867 | 2.112333447 | 2.85729E-07 | 4.73379E-06 | - |
| *uncharacterized protein_09728* | 63.93822156 | 146.3573706 | 2.289043502 | 1.194744881 | 3.01859E-07 | 4.98509E-06 | - |
| *NTAQ1* | 56.587773 | 133.2996767 | 2.355626836 | 1.236111014 | 3.2419E-07 | 5.33688E-06 | - |
| *UGT89B2* | 163.0686593 | 395.4834113 | 2.425257024 | 1.278137649 | 3.32139E-07 | 5.46081E-06 | K13496 |
| *At1g56140* | 15.03676793 | 79.75213559 | 5.303808368 | 2.40702865 | 3.64073E-07 | 5.9369E-06 | K04733 |
| *At1g18250* | 0.702137172 | 45.45465622 | 64.73757266 | 6.016531368 | 3.81754E-07 | 6.20182E-06 | - |
| *TSJT1* | 9.102325204 | 45.9379045 | 5.046831823 | 2.335378012 | 3.87075E-07 | 6.27716E-06 | - |
| *Pdcd2l* | 763.2227671 | 1663.177122 | 2.1791503 | 1.123765705 | 3.95375E-07 | 6.39507E-06 | K14801 |
| *EXL2* | 153.7624003 | 736.8395975 | 4.792066176 | 2.260647831 | 4.34463E-07 | 6.9794E-06 | - |
| *CHI4* | 0 | 32.67918025 | Inf | Inf | 4.59651E-07 | 7.34025E-06 | K01183 |
| *At2g42990* | 0.989160125 | 58.68284285 | 59.32592847 | 5.890590869 | 4.92747E-07 | 7.84281E-06 | - |
| *At3g17800* | 2006.973891 | 5417.806487 | 2.699490268 | 1.432687016 | 5.2421E-07 | 8.31297E-06 | - |
| *At5g16420* | 87.43664077 | 187.7644786 | 2.147434724 | 1.102614278 | 5.25373E-07 | 8.32124E-06 | - |
| *ISU1* | 50.37375406 | 121.6277447 | 2.414506264 | 1.271728206 | 5.4941E-07 | 8.6649E-06 | - |
| *At4g00755* | 68.27138869 | 148.6139411 | 2.176811457 | 1.122216455 | 6.0556E-07 | 9.47553E-06 | - |
| *BZIP53* | 0 | 15.98727125 | Inf | Inf | 6.16991E-07 | 9.63696E-06 | - |
| *IAA11* | 76.53315589 | 298.6256884 | 3.901912641 | 1.964181478 | 6.20312E-07 | 9.68299E-06 | K14484 |
| *uncharacterized protein_24738* | 71.35389623 | 154.6966205 | 2.168019248 | 1.116377565 | 6.35621E-07 | 9.88625E-06 | - |
| *TIC32B* | 22.61049425 | 70.86637552 | 3.134224965 | 1.648108736 | 6.48978E-07 | 1.00819E-05 | - |
| *PCK* | 478.7586348 | 1282.412092 | 2.678619242 | 1.421489521 | 7.43509E-07 | 1.14002E-05 | K01610 |
| *PCMP-H40* | 67.653808 | 163.9076488 | 2.422740917 | 1.276640134 | 7.49883E-07 | 1.14911E-05 | - |
| *GDPDL4* | 47.38922913 | 115.2423399 | 2.431825586 | 1.28203976 | 7.54636E-07 | 1.15503E-05 | - |
| *BAM3* | 1.011369467 | 20.28334471 | 20.05532634 | 4.325913537 | 8.126E-07 | 1.23717E-05 | K01177 |
| *At2g42960* | 2.047530667 | 38.66028932 | 18.88142138 | 4.238895469 | 8.30047E-07 | 1.26226E-05 | - |
| *uncharacterized protein_24919* | 56.56437932 | 129.3718235 | 2.287160666 | 1.193557714 | 8.44021E-07 | 1.2805E-05 | - |
| *ATL48* | 54.92163331 | 125.9487445 | 2.2932447 | 1.197390305 | 9.64994E-07 | 1.45383E-05 | - |
| *At4g02290* | 2.039783493 | 132.2065109 | 64.81399199 | 6.018233389 | 1.12487E-06 | 1.67523E-05 | - |
| *HAT14* | 14.45394267 | 53.65377918 | 3.712051474 | 1.892216716 | 1.13576E-06 | 1.68949E-05 | K09338 |
| *MANA* | 35.98488921 | 97.78250848 | 2.717321371 | 1.4421852 | 1.2057E-06 | 1.78027E-05 | - |
| *uncharacterized protein_17843* | 87.58863986 | 177.2262581 | 2.023393198 | 1.0167767 | 1.22195E-06 | 1.80323E-05 | - |
| *ROQ1* | 1.030996417 | 42.25629293 | 40.98587758 | 5.357054984 | 1.24169E-06 | 1.83028E-05 | - |
| *ATJ8* | 12.06567298 | 54.69272192 | 4.5329193 | 2.180440477 | 1.29515E-06 | 1.89936E-05 | - |
| *uncharacterized protein_34118* | 57.51568748 | 130.1375122 | 2.262643775 | 1.178009468 | 1.41304E-06 | 2.05827E-05 | - |
| *MFSD14A* | 14.69396455 | 54.46912304 | 3.706904482 | 1.890214941 | 1.4426E-06 | 2.09897E-05 | - |
| *MYB306* | 16.98734818 | 82.43549626 | 4.852758382 | 2.27880503 | 1.722E-06 | 2.4613E-05 | K09422 |
| *PAP15* | 5.062012119 | 31.88416983 | 6.298714638 | 2.655057452 | 1.74057E-06 | 2.48237E-05 | - |
| *uncharacterized protein_05393* | 5.764149291 | 112.8763042 | 19.5824741 | 4.291491145 | 1.74279E-06 | 2.48417E-05 | - |
| *ERD4* | 1182.318564 | 2801.979554 | 2.369902359 | 1.244827621 | 1.83208E-06 | 2.6E-05 | - |
| *STA* | 22.78130571 | 108.4742152 | 4.761545127 | 2.251429806 | 1.96351E-06 | 2.76684E-05 | - |
| *CHI4* | 1.053205759 | 110.3341146 | 104.7602652 | 6.710947807 | 2.01349E-06 | 2.82652E-05 | K01183 |
| *OsI_29059* | 397.0166324 | 902.7449898 | 2.273821588 | 1.18511906 | 2.09315E-06 | 2.93199E-05 | - |
| *At5g03610* | 1.983485034 | 43.30988514 | 21.83524675 | 4.448586929 | 2.11915E-06 | 2.96201E-05 | - |
| *MYB2* | 0.331441636 | 61.56375851 | 185.7453976 | 7.537182654 | 2.1454E-06 | 2.99548E-05 | K09422 |
| *pcrA* | 59.37086365 | 154.4187728 | 2.60091842 | 1.379021148 | 2.17025E-06 | 3.02691E-05 | K03657 |
| *BHLH25* | 0.351068586 | 24.16491823 | 68.83247085 | 6.105017394 | 2.21334E-06 | 3.08369E-05 | - |
| *ALP1* | 87.82054869 | 193.2214984 | 2.200185506 | 1.137625168 | 2.31159E-06 | 3.21194E-05 | - |
| *NSFBx* | 12.78485471 | 48.78507821 | 3.815849246 | 1.932004175 | 2.4669E-06 | 3.40404E-05 | - |
| *CBWD1* | 80.6637044 | 164.6044651 | 2.040626157 | 1.029011905 | 2.53945E-06 | 3.49416E-05 | - |
| *ATJ8* | 8.736794451 | 46.05985817 | 5.271940232 | 2.398334015 | 2.75459E-06 | 3.76693E-05 | - |
| *E134* | 52.11721722 | 118.4531168 | 2.272821211 | 1.184484201 | 2.862E-06 | 3.89942E-05 | - |
| *CHI4* | 1.013951859 | 55.13316735 | 54.37454143 | 5.764859424 | 2.89122E-06 | 3.93097E-05 | K01183 |
| *MSL2* | 26.93628008 | 81.30734611 | 3.018506857 | 1.593835078 | 2.91912E-06 | 3.96474E-05 | - |
| *uncharacterized protein_06362* | 213.9113919 | 656.5961219 | 3.069477114 | 1.617992913 | 2.99385E-06 | 4.0535E-05 | - |
| *PCMP-H40* | 26.68592863 | 89.2872905 | 3.345856602 | 1.742375615 | 3.06848E-06 | 4.14804E-05 | - |
| *At5g63930* | 10.38367307 | 43.53127387 | 4.192280862 | 2.067735373 | 3.12753E-06 | 4.21688E-05 | - |
| *ATG13B* | 124.6203896 | 278.6939034 | 2.236342739 | 1.161141311 | 3.17032E-06 | 4.27014E-05 | K08331 |
| *CRK2* | 0 | 13.22823583 | Inf | Inf | 3.19882E-06 | 4.30404E-05 | - |
| *EFL3* | 48.88838953 | 116.3083949 | 2.37905965 | 1.250391445 | 3.26302E-06 | 4.38133E-05 | - |
| *CAT3* | 0.328859245 | 70.76249669 | 215.1756347 | 7.749370914 | 3.2876E-06 | 4.41205E-05 | K03781 |
| *At1g60710* | 34.28392865 | 183.5382233 | 5.353476994 | 2.420476202 | 3.29867E-06 | 4.42233E-05 | - |
| *HIPP23* | 0 | 75.71255789 | Inf | Inf | 3.59333E-06 | 4.79499E-05 | - |
| *GSTU20* | 295.5542273 | 777.8274654 | 2.631758891 | 1.396027322 | 3.61283E-06 | 4.81612E-05 | K00799 |
| *uncharacterized protein_07176* | 23.26924492 | 68.5127277 | 2.944346838 | 1.557947628 | 3.62174E-06 | 4.82551E-05 | - |
| *NPC6* | 10.07444077 | 43.19806072 | 4.287886713 | 2.10026679 | 3.66332E-06 | 4.87337E-05 | K01114 |
| *ANN1* | 261.9779626 | 807.0522646 | 3.080611271 | 1.623216647 | 3.72651E-06 | 4.94478E-05 | K17095 |
| *BPC3* | 546.8794224 | 1342.162573 | 2.454220287 | 1.295264749 | 3.81711E-06 | 5.05207E-05 | - |
| *ATL77* | 0 | 44.29239473 | Inf | Inf | 3.83386E-06 | 5.06907E-05 | K19040 |
| *IFRD2* | 5.10384841 | 30.57184823 | 5.98996008 | 2.582546388 | 3.88604E-06 | 5.13544E-05 | - |
| *At1g64390* | 24.75136076 | 102.0357042 | 4.122428062 | 2.043494318 | 4.59034E-06 | 5.98383E-05 | - |
| *PLAT1* | 19.50820761 | 60.93924003 | 3.123774426 | 1.643290277 | 4.67986E-06 | 6.08522E-05 | - |
| *Os01g0270100* | 14.73063605 | 82.35979653 | 5.591055012 | 2.48312054 | 4.69123E-06 | 6.0939E-05 | - |
| *At4g34215* | 61.79115422 | 131.3877145 | 2.126319149 | 1.088358154 | 4.7335E-06 | 6.14573E-05 | - |
| *uncharacterized protein_06673* | 52.4398795 | 116.7839458 | 2.227006373 | 1.155105687 | 4.85463E-06 | 6.28099E-05 | - |
| *RR41* | 14.20359122 | 50.11093095 | 3.528046546 | 1.818869595 | 5.38461E-06 | 6.90466E-05 | - |
| *ATJ72* | 38.10834529 | 93.10232508 | 2.443095453 | 1.288710231 | 5.49374E-06 | 7.04111E-05 | - |
| *MAVI* | 13.86026981 | 49.70206728 | 3.585937933 | 1.842350518 | 5.82174E-06 | 7.40658E-05 | - |
| *NFD4* | 246.448757 | 589.5407376 | 2.392143279 | 1.258303803 | 6.11262E-06 | 7.76141E-05 | - |
| *AAE5* | 527.7202152 | 1106.478148 | 2.096713593 | 1.068129805 | 6.20382E-06 | 7.85414E-05 | - |
| *RPOT2-TOM* | 72.47941282 | 147.8831111 | 2.040346429 | 1.028814127 | 6.38941E-06 | 8.06547E-05 | K10908 |
| *OsI_03083* | 3.71145384 | 28.21037972 | 7.600897365 | 2.926169754 | 6.81865E-06 | 8.54077E-05 | - |
| *Os12g0628600* | 2.003111984 | 21.31670113 | 10.64179202 | 3.411669208 | 7.2784E-06 | 9.06845E-05 | - |
| *DAD2* | 29.57076861 | 196.3397659 | 6.639657172 | 2.731108752 | 7.35892E-06 | 9.14928E-05 | - |
| *PA1024* | 18.64197397 | 72.62591986 | 3.895827769 | 1.961929899 | 7.79564E-06 | 9.64338E-05 | - |
| *CIPK23* | 18.55830139 | 56.86117913 | 3.063921528 | 1.615379348 | 7.90607E-06 | 9.77067E-05 | - |
| *XYL1* | 30.90376817 | 118.9838856 | 3.850141672 | 1.944911533 | 7.97826E-06 | 9.83644E-05 | K15925 |
| *NPF8.3* | 35.78567144 | 87.96712323 | 2.458166067 | 1.297582384 | 8.1155E-06 | 9.98665E-05 | K14638 |
| *At5g03610* | 0.351068586 | 39.21362603 | 111.6979062 | 6.803458333 | 8.46896E-06 | 0.000103773 | - |
| *SYT2* | 76.5548472 | 167.2405784 | 2.184585099 | 1.127359306 | 8.49799E-06 | 0.00010403 | - |
| *GALM* | 77.57898035 | 225.043598 | 2.90083212 | 1.536466805 | 8.66686E-06 | 0.000105947 | K01785 |
| *ROQ1* | 13.13747351 | 47.29315307 | 3.599866675 | 1.847943476 | 9.13509E-06 | 0.000111304 | - |
| *At5g03610* | 0.351068586 | 14.40273832 | 41.02542603 | 5.35844641 | 9.64479E-06 | 0.000117129 | - |
| *CYP750A1* | 0.657718489 | 24.48460023 | 37.22656521 | 5.218260604 | 1.03655E-05 | 0.000125412 | - |
| *OBAP1A* | 1.030996417 | 17.36844033 | 16.84626643 | 4.074356983 | 1.05271E-05 | 0.000127189 | - |
| *ANR* | 118.1510027 | 287.3529337 | 2.432082057 | 1.282191905 | 1.06198E-05 | 0.000128129 | K08695 |
| *BHLH25* | 0.328859245 | 14.14187295 | 43.00281408 | 5.426359167 | 1.07637E-05 | 0.000129734 | - |
| *45569* | 35.79341862 | 99.91245751 | 2.791363926 | 1.480970229 | 1.13821E-05 | 0.000136312 | K08202 |
| *At3g09310* | 58.21074378 | 121.7452788 | 2.091457194 | 1.06450847 | 1.19193E-05 | 0.00014222 | K08998 |
| *Os01g0656200* | 5.34645268 | 152.75874 | 28.57198018 | 4.836529121 | 1.20819E-05 | 0.000144028 | K14497 |
| *EXL2* | 0 | 22.43705405 | Inf | Inf | 1.21091E-05 | 0.000144286 | - |
| *FLS* | 0.660300881 | 15.86412583 | 24.0256015 | 4.586500645 | 1.21911E-05 | 0.000145195 | K05278 |
| *CAD* | 0 | 12.06383836 | Inf | Inf | 1.24513E-05 | 0.00014809 | K00083 |
| *CHI4* | 0 | 12.02205317 | Inf | Inf | 1.26803E-05 | 0.000150193 | K01183 |
| *GRXC5* | 45.65609554 | 102.4387832 | 2.243704417 | 1.165882629 | 1.38178E-05 | 0.000162332 | K03676 |
| *GSTU19* | 33.64517081 | 92.61555153 | 2.752714559 | 1.460855018 | 1.39823E-05 | 0.000163894 | K00799 |
| *AAP2* | 0.662883273 | 15.43386036 | 23.28292326 | 4.5412003 | 1.41619E-05 | 0.000165774 | - |
| *HSP17.7* | 30.48038875 | 78.47365435 | 2.574562122 | 1.364327082 | 1.4488E-05 | 0.000168961 | K13993 |
| *PYRD* | 66.63159094 | 134.8096766 | 2.023209633 | 1.016645811 | 1.497E-05 | 0.000174289 | K11752 |
| *ABCB26* | 39.74076174 | 91.05827921 | 2.29130684 | 1.19617067 | 1.57364E-05 | 0.000182393 | K05656 |
| *RhGT1* | 13.40228712 | 72.16275826 | 5.384361462 | 2.428775264 | 1.58998E-05 | 0.000184205 | K08237 |
| *ATL76* | 64.28412537 | 219.5232179 | 3.414890016 | 1.771839114 | 1.73537E-05 | 0.000199444 | K19040 |
| *DRE21* | 87.02890784 | 174.4877294 | 2.004939896 | 1.003558988 | 1.79439E-05 | 0.000205318 | - |
| *At3g21360* | 15.22942286 | 49.08775374 | 3.223218253 | 1.688501881 | 1.80545E-05 | 0.00020631 | - |
| *ADH2* | 561.5063199 | 3360.249256 | 5.98434806 | 2.581194087 | 1.81566E-05 | 0.000207112 | K18857 |
| *Os04g0656100* | 3.058900135 | 23.37889602 | 7.642909213 | 2.934121893 | 1.81805E-05 | 0.000207293 | K01535 |
| *TIC32* | 10.7151147 | 124.9209487 | 11.65838651 | 3.543296232 | 1.9731E-05 | 0.000223595 | - |
| *At3g01520* | 17.56655887 | 79.22814551 | 4.510168787 | 2.173181426 | 2.05089E-05 | 0.000231903 | - |
| *uncharacterized protein_00302* | 554.5339346 | 1135.339507 | 2.047376069 | 1.033776125 | 2.06916E-05 | 0.000233562 | - |
| *uncharacterized protein_46853* | 24.6957286 | 99.52290962 | 4.029964502 | 2.010767131 | 2.22679E-05 | 0.000249185 | - |
| *uncharacterized protein_19224* | 42.52023781 | 99.12191659 | 2.331170325 | 1.221054418 | 2.27794E-05 | 0.000254209 | - |
| *FSD3* | 57.00723741 | 125.5794566 | 2.202868659 | 1.13938348 | 2.35467E-05 | 0.000262114 | K04564 |
| *At3g51250* | 63.99230351 | 129.4813144 | 2.023388865 | 1.016773611 | 2.53293E-05 | 0.000279701 | - |
| *SRG1* | 7.381071389 | 38.66031354 | 5.237764479 | 2.388951189 | 2.5977E-05 | 0.000286366 | - |
| *MES1* | 99.29070204 | 624.2400574 | 6.286994094 | 2.652370408 | 2.66874E-05 | 0.000293572 | - |
| *SNAT2* | 23.88254472 | 70.92837077 | 2.969883301 | 1.570406243 | 2.67655E-05 | 0.000294307 | - |
| *uncharacterized protein_40904* | 36.40000343 | 84.54858718 | 2.322763165 | 1.215842061 | 2.74751E-05 | 0.000300961 | - |
| *ABCI17* | 6.7977281 | 36.91211593 | 5.430066544 | 2.440969878 | 2.85142E-05 | 0.000311291 | - |
| *RCA1* | 29.90376045 | 74.48591691 | 2.490854521 | 1.316640763 | 3.06549E-05 | 0.000331867 | - |
| *PCMP-H40* | 24.8520779 | 64.95868185 | 2.613812902 | 1.386155876 | 3.16046E-05 | 0.000341117 | - |
| *GH5FP* | 104.8503166 | 245.1416552 | 2.338015402 | 1.225284434 | 3.16146E-05 | 0.000341117 | - |
| *OPT7* | 1.975737859 | 19.23191346 | 9.734041065 | 3.283038861 | 3.18994E-05 | 0.000343903 | - |
| *ISA2* | 16.50405573 | 50.0725469 | 3.033954062 | 1.601199241 | 3.51101E-05 | 0.000375706 | - |
| *uncharacterized protein_07436* | 24.5428456 | 99.92159408 | 4.071312499 | 2.025493962 | 3.52601E-05 | 0.000377155 | - |
| *ALDR* | 0 | 10.78203016 | Inf | Inf | 3.64804E-05 | 0.000389565 | - |
| *CYC2* | 56.87154725 | 115.5247312 | 2.031327382 | 1.022422772 | 3.87343E-05 | 0.000411937 | - |
| *uncharacterized protein_32472* | 0 | 89.68029025 | Inf | Inf | 4.00567E-05 | 0.000423051 | - |
| *At3g19950* | 91.89428459 | 198.9765759 | 2.165276946 | 1.114551562 | 4.02369E-05 | 0.000424608 | K11982 |
| *ufd1* | 54.52356365 | 112.3251761 | 2.060121691 | 1.04272956 | 4.3557E-05 | 0.00045667 | - |
| *GLDH* | 126.7524729 | 258.3108754 | 2.037915865 | 1.027094491 | 4.4199E-05 | 0.00046284 | K00225 |
| *NAGS1* | 233.2298274 | 652.3728163 | 2.797124294 | 1.483944363 | 4.46467E-05 | 0.000466962 | K14682 |
| *WSD5* | 55.98657056 | 114.1660807 | 2.039169028 | 1.027981367 | 4.48516E-05 | 0.000468917 | - |
| *CYL3* | 78.13287741 | 217.9669889 | 2.789696171 | 1.480108005 | 4.69271E-05 | 0.000488645 | - |
| *CNMT* | 3.365550038 | 22.39638643 | 6.654599153 | 2.734351766 | 4.87075E-05 | 0.000505155 | K13384 |
| *uncharacterized protein_18735* | 36.86883372 | 83.62246153 | 2.268107046 | 1.181488732 | 5.12776E-05 | 0.00052906 | - |
| *FAH* | 883.8173019 | 1865.012904 | 2.110179219 | 1.077365533 | 5.19335E-05 | 0.000535188 | K01555 |
| *HXK1* | 2126.086555 | 5680.601873 | 2.671858237 | 1.417843463 | 5.23849E-05 | 0.000538768 | K00844 |
| *PCMP-H40* | 38.37574129 | 86.36342287 | 2.250469176 | 1.170225805 | 5.28617E-05 | 0.000543026 | - |
| *uncharacterized protein_31257* | 33.15221509 | 77.33758511 | 2.332802948 | 1.222064448 | 5.46767E-05 | 0.000559233 | - |
| *DLO1* | 36.12486025 | 82.13736366 | 2.273707444 | 1.185046636 | 5.54566E-05 | 0.000566355 | - |
| *At3g21360* | 10.45443369 | 37.80427708 | 3.616099944 | 1.854434552 | 5.55113E-05 | 0.000566652 | - |
| *RBOHC* | 115.3772094 | 276.7781692 | 2.398898106 | 1.26237188 | 5.6385E-05 | 0.000574215 | K13447 |
| *NIT4A* | 273.7176631 | 595.5455525 | 2.175765882 | 1.121523327 | 5.99282E-05 | 0.00060625 | K13035 |
| *RhGT1* | 12.73268885 | 41.81579905 | 3.284129498 | 1.715511016 | 6.02812E-05 | 0.000608871 | - |
| *uncharacterized protein_05124* | 3.03152601 | 55.63473723 | 18.3520567 | 4.197869849 | 6.21591E-05 | 0.000626862 | - |
| *CRK2* | 40.95289918 | 120.1155588 | 2.933017227 | 1.552385545 | 6.25505E-05 | 0.000630563 | - |
| *UGT89B1* | 73.70704461 | 250.3149694 | 3.396079313 | 1.763870152 | 6.68811E-05 | 0.000669534 | K13496 |
| *DES5* | 9.47302074 | 39.94212174 | 4.216408138 | 2.076014523 | 6.7008E-05 | 0.000670287 | - |
| *AVT1C* | 0 | 64.23698767 | Inf | Inf | 6.70456E-05 | 0.000670404 | K15015 |
| *S-RBP11* | 48.75424957 | 101.3783145 | 2.079373909 | 1.056149204 | 6.71075E-05 | 0.000670585 | K13195 |
| *RNR1* | 46.1719269 | 98.15616671 | 2.125884131 | 1.088062967 | 7.05043E-05 | 0.00070255 | - |
| *CIPK2* | 47.17554919 | 98.5176339 | 2.088319809 | 1.062342666 | 7.27498E-05 | 0.000721597 | K07198 |
| *uncharacterized protein_09829* | 8.787928126 | 50.21257541 | 5.713812708 | 2.514453747 | 8.04631E-05 | 0.000791982 | - |
| *B2* | 1208.075546 | 2562.496696 | 2.121139447 | 1.084839469 | 8.07844E-05 | 0.000793701 | - |
| *CRPK1* | 13.13075851 | 41.65429457 | 3.172268725 | 1.665514988 | 9.0202E-05 | 0.000877912 | - |
| *HSP70* | 5583.245425 | 13753.27398 | 2.463311736 | 1.300599215 | 9.32603E-05 | 0.000903945 | K03283 |
| *GSTU17* | 2.663412865 | 42.26188001 | 15.86756622 | 3.988008958 | 9.49561E-05 | 0.00091798 | K00799 |
| *PGMP* | 13.74664071 | 49.24352354 | 3.582222346 | 1.840854887 | 9.78532E-05 | 0.000942473 | K01835 |
| *uncharacterized protein_20390* | 29.86089198 | 72.19215409 | 2.41761546 | 1.273584791 | 9.94316E-05 | 0.000954485 | K15032 |
| *ZIF1* | 65.31976854 | 144.0005691 | 2.204548062 | 1.14048293 | 0.000103383 | 0.000990216 | - |
| *uncharacterized protein_04724* | 43.7945048 | 91.99783684 | 2.100670786 | 1.070850083 | 0.000103547 | 0.000991421 | - |
| *CPK3* | 32.98811862 | 76.76277242 | 2.32698243 | 1.218460317 | 0.000105133 | 0.001005123 | K13412 |
| *GATA9* | 51.08415644 | 102.5156247 | 2.006798817 | 1.004895993 | 0.000106193 | 0.001014504 | - |
| *CYP750A1* | 6.291194122 | 50.57506176 | 8.039024195 | 3.007020393 | 0.000107414 | 0.001024661 | - |
| *At3g26720* | 36.72266571 | 79.71948622 | 2.170852379 | 1.118261624 | 0.000109889 | 0.001045961 | K01191 |
| *SWEET16* | 65.89639684 | 252.3042651 | 3.828802138 | 1.936893108 | 0.000112971 | 0.001071754 | K15382 |
| *Os01g0656200* | 26.22551182 | 151.8968917 | 5.791951467 | 2.534049513 | 0.000113732 | 0.00107751 | K14497 |
| *UBQ4* | 40.96286287 | 106.3011151 | 2.595060688 | 1.375768278 | 0.000115361 | 0.00109124 | K08770 |
| *CRJ34* | 0.328859245 | 11.38623868 | 34.62344107 | 5.11367721 | 0.000117478 | 0.001110051 | - |
| *MTM1* | 34.75209263 | 77.02252022 | 2.216341935 | 1.148180476 | 0.000118091 | 0.001114617 | K18081 |
| *PCMP-H22* | 23.15111734 | 63.92634384 | 2.761263869 | 1.46532876 | 0.000119488 | 0.001126983 | - |
| *MAOM* | 17.27437113 | 48.69582235 | 2.818963537 | 1.495164817 | 0.00012042 | 0.001133907 | K00028 |
| *ERD4* | 32.6942324 | 73.64574514 | 2.252560765 | 1.171566024 | 0.00012044 | 0.001133907 | - |
| *uncharacterized protein_17552* | 281.1798246 | 1020.335086 | 3.628763506 | 1.859478036 | 0.0001313 | 0.001224149 | - |
| *DREB2A* | 34.46337119 | 76.60240973 | 2.222719574 | 1.152325944 | 0.000132588 | 0.001235268 | - |
| *L6* | 3.340758304 | 20.9925746 | 6.283775326 | 2.651631599 | 0.00013385 | 0.00124568 | - |
| *PGMP* | 15.51386342 | 45.41954994 | 2.927674991 | 1.549755405 | 0.000134718 | 0.001252854 | K01835 |
| *At2g37240* | 75.90302912 | 185.6919311 | 2.446436371 | 1.29068176 | 0.000140119 | 0.001296112 | - |
| *ATJ11* | 34.50985424 | 107.4305547 | 3.113039942 | 1.638324088 | 0.000144381 | 0.001332211 | - |
| *SPBC21C3.03* | 37.53946417 | 81.8922396 | 2.181497297 | 1.125318686 | 0.000146605 | 0.001349846 | K08869 |
| *PCMP-H81* | 8.800840084 | 33.39407064 | 3.794418524 | 1.923878817 | 0.000157252 | 0.00143564 | - |
| *PCBER* | 283.706363 | 574.0915046 | 2.023541166 | 1.016882199 | 0.000168604 | 0.001529051 | - |
| *CBSX3* | 2429.691446 | 4971.905268 | 2.046311385 | 1.033025695 | 0.000169451 | 0.001535661 | - |
| *PDS5A* | 2.37380752 | 17.61920066 | 7.422337537 | 2.89187361 | 0.000172224 | 0.001556986 | - |
| *ILL7* | 39.12642975 | 239.4830609 | 6.120749131 | 2.613708238 | 0.000184594 | 0.001650401 | K14664 |
| *uncharacterized protein_45410* | 85.10208715 | 172.0908829 | 2.022169945 | 1.015904248 | 0.00018581 | 0.001660124 | K11674 |
| *mybI* | 0.331441636 | 11.0078884 | 33.21214716 | 5.053639091 | 0.000188731 | 0.001682162 | - |
| *At1g27930* | 38.2601961 | 91.41632027 | 2.389332246 | 1.25660748 | 0.000191383 | 0.001703795 | - |
| *PCMP-H81* | 53.37185726 | 132.1330933 | 2.475707237 | 1.30784072 | 0.000193627 | 0.00172048 | - |
| *uncharacterized protein_11466* | 3.362967646 | 20.50920295 | 6.098543045 | 2.608464621 | 0.000195227 | 0.001732982 | - |
| *uncharacterized protein_48210* | 17.88767094 | 48.05325477 | 2.686389689 | 1.425668598 | 0.000201396 | 0.001782184 | - |
| *VPY* | 36.09903634 | 77.00221101 | 2.133082177 | 1.092939546 | 0.000203743 | 0.001801107 | K15502 |
| *uncharacterized protein_06115* | 0 | 25.27407353 | Inf | Inf | 0.000206716 | 0.001824331 | - |
| *CYP750A1* | 3.471431962 | 33.74533365 | 9.72086851 | 3.281085217 | 0.000206865 | 0.001824969 | - |
| *PCMP-H73* | 28.8087184 | 66.60300063 | 2.311904324 | 1.209081694 | 0.000211492 | 0.001862625 | - |
| *Os07g0638300* | 0 | 43.96054591 | Inf | Inf | 0.000220707 | 0.001932618 | K11188 |
| *htxA* | 189.2373547 | 433.389404 | 2.290189507 | 1.195466983 | 0.000220824 | 0.001932989 | - |
| *MES1* | 52.02904616 | 299.9097817 | 5.764275993 | 2.527139413 | 0.000231499 | 0.002016902 | - |
| *PHYPADRAFT_128349* | 28.33125704 | 76.43181863 | 2.697791296 | 1.431778744 | 0.00023166 | 0.002017626 | K08967 |
| *LIR1* | 4792.219804 | 13455.70282 | 2.807822547 | 1.489451761 | 0.000235975 | 0.002050095 | - |
| *D14* | 26.25376985 | 67.41834449 | 2.567949093 | 1.360616603 | 0.000244598 | 0.002117509 | - |
| *ROQ1* | 6.006753561 | 51.12854607 | 8.511843469 | 3.08947162 | 0.000250422 | 0.002161435 | - |
| *uncharacterized protein_34960* | 0 | 11.95318071 | Inf | Inf | 0.000251497 | 0.002168544 | - |
| *APUM12* | 44.69947432 | 96.74346583 | 2.164308804 | 1.113906358 | 0.000252354 | 0.002175211 | - |
| *At1g18440* | 14.47253743 | 42.35443785 | 2.92653849 | 1.549195253 | 0.000268566 | 0.002293605 | K01056 |
| *FKBP12* | 17.53763454 | 46.57267561 | 2.655584795 | 1.409029596 | 0.000269683 | 0.002301622 | K01802 |
| *ELI* | 5.30719878 | 39.53442483 | 7.449207477 | 2.897086945 | 0.000273763 | 0.002331846 | - |
| *At3g60360* | 0 | 11.32297751 | Inf | Inf | 0.000278045 | 0.002364037 | K14769 |
| *uncharacterized protein_46359* | 0.331441636 | 10.41159974 | 31.41307125 | 4.973293097 | 0.000279405 | 0.002371333 | - |
| *EPHX2* | 20.39886711 | 56.35739911 | 2.762771031 | 1.466116001 | 0.000288258 | 0.002435663 | - |
| *uncharacterized protein_42434* | 7.475073539 | 32.77188645 | 4.384155725 | 2.132299044 | 0.000293233 | 0.002470894 | - |
| *BGLU43* | 35.37985461 | 77.97456637 | 2.203925574 | 1.140075505 | 0.000298005 | 0.002506814 | K05350 |
| *CCR1* | 70.92033939 | 179.1938326 | 2.526691696 | 1.337249639 | 0.000309195 | 0.002587733 | K09753 |
| *Os04g0338000* | 16.42709813 | 45.13946795 | 2.747866214 | 1.458311765 | 0.000314197 | 0.002626211 | - |
| *SUP35* | 18.60530246 | 49.07412343 | 2.637641798 | 1.399248654 | 0.000319377 | 0.002664356 | K03267 |
| *CAD* | 0.679927831 | 11.48676632 | 16.89409641 | 4.078447284 | 0.000326132 | 0.002711979 | K00083 |
| *uncharacterized protein_26646* | 95.90345682 | 215.7963958 | 2.250141996 | 1.170016046 | 0.000330928 | 0.002746573 | - |
| *S-RBP11* | 18.61976463 | 48.53431787 | 2.606602116 | 1.38217038 | 0.00033119 | 0.002747874 | K13195 |
| *At5g03810* | 16.3229147 | 45.28513273 | 2.774328824 | 1.472138791 | 0.000343655 | 0.002834441 | - |
| *thiO* | 11.84166348 | 36.97301859 | 3.122282495 | 1.642601074 | 0.000350557 | 0.002882729 | - |
| *uncharacterized protein_18630* | 0.657718489 | 11.69340794 | 17.77874293 | 4.152081415 | 0.000359617 | 0.002946015 | K15032 |
| *DTX54* | 5.062012119 | 23.34954939 | 4.612701203 | 2.205611842 | 0.000364908 | 0.002985589 | K03327 |
| *Os01g0810000* | 2.665995257 | 20.87854079 | 7.831424581 | 2.969274766 | 0.000368663 | 0.003007367 | K14769 |
| *VTE5* | 45.03246617 | 90.51263981 | 2.009941882 | 1.007153786 | 0.000370664 | 0.003020291 | K18678 |
| *At1g56220* | 63.19166571 | 255.6300692 | 4.045313039 | 2.016251347 | 0.000378105 | 0.003074167 | - |
| *PAO6* | 318.2849135 | 1472.823797 | 4.627375458 | 2.210194162 | 0.000386214 | 0.003134194 | K13366 |
| *PCMP-H44* | 8.073911178 | 29.46053273 | 3.648855193 | 1.867443898 | 0.000390412 | 0.003163229 | - |
| *uncharacterized protein_35308* | 9.843716276 | 43.50868032 | 4.419944572 | 2.144028278 | 0.000390524 | 0.003163229 | - |
| *CYP704C1* | 14.4043592 | 41.34479178 | 2.870297193 | 1.521200122 | 0.000403894 | 0.003253239 | - |
| *PXG* | 9.75487891 | 32.24556284 | 3.305583097 | 1.724904782 | 0.000409925 | 0.003295667 | K17991 |
| *TOP3A* | 18.51130031 | 49.36212527 | 2.666594158 | 1.414998271 | 0.000410666 | 0.003298557 | K03165 |
| *ABCA2* | 22.51855646 | 54.36184231 | 2.414090903 | 1.271480002 | 0.000411196 | 0.003300771 | - |
| *CYP704C1* | 9.762626085 | 32.04341496 | 3.282253635 | 1.714686727 | 0.000418596 | 0.003349803 | - |
| *LOG3* | 8.971651546 | 38.81278136 | 4.326157917 | 2.113086329 | 0.000423753 | 0.003384805 | K06966 |
| *CYP707A1* | 0 | 22.70584002 | Inf | Inf | 0.000438225 | 0.003485366 | K09843 |
| *At1g60710* | 0.662883273 | 11.55787316 | 17.43575927 | 4.123977285 | 0.000442012 | 0.00351333 | - |
| *uncharacterized protein_50841* | 40.71184512 | 91.3677328 | 2.244254284 | 1.166236149 | 0.00044746 | 0.003549019 | - |
| *PCMP-H85* | 35.71904342 | 75.11947205 | 2.103065056 | 1.072493479 | 0.000458015 | 0.003627184 | - |
| *rsmH* | 35.84646838 | 86.87403016 | 2.423503181 | 1.277093976 | 0.000463427 | 0.003665568 | - |
| *At1g56220* | 3.632946041 | 158.4586503 | 43.6171219 | 5.446822671 | 0.000468601 | 0.003703104 | - |
| *uncharacterized protein_42929* | 10.42292697 | 33.13889076 | 3.179422716 | 1.668764841 | 0.000470645 | 0.003718122 | - |
| *At3g21360* | 5.062012119 | 24.22033372 | 4.784724563 | 2.258435877 | 0.000473167 | 0.003735774 | - |
| *AIL1* | 34.89051347 | 72.44745736 | 2.076422791 | 1.054100228 | 0.000486039 | 0.00382227 | K09285 |
| *DCR* | 1.688714906 | 14.5890957 | 8.63917032 | 3.110892767 | 0.000487557 | 0.003831884 | K19747 |
| *HHT1* | 2.351598179 | 16.45371061 | 6.996820614 | 2.806699504 | 0.000488057 | 0.003834649 | K15400 |
| *Zswim2* | 0.331441636 | 9.805131867 | 29.58328343 | 4.88671028 | 0.000488862 | 0.003836324 | - |
| *D6PKL1* | 0.351068586 | 9.721561489 | 27.69134542 | 4.791363246 | 0.000500939 | 0.003920418 | - |
| *PCMP-H73* | 32.05975544 | 67.8238812 | 2.115545807 | 1.081029924 | 0.00051193 | 0.003996781 | - |
| *WSD11* | 1.345393495 | 17.21036197 | 12.79206569 | 3.677177347 | 0.00052028 | 0.004052427 | - |
| *uncharacterized protein_21477* | 0.682510222 | 20.0823144 | 29.42419576 | 4.878931078 | 0.000524433 | 0.00408088 | - |
| *uncharacterized protein_24925* | 13.55258773 | 38.02000531 | 2.805368693 | 1.488190388 | 0.000534762 | 0.004147564 | - |
| *PXG* | 0 | 8.1652826 | Inf | Inf | 0.000538463 | 0.004173775 | K17991 |
| *At5g15710* | 36.23007587 | 74.36853115 | 2.0526739 | 1.037504451 | 0.000541319 | 0.00419215 | - |
| *GSTU16* | 368.6235463 | 971.7308585 | 2.636106316 | 1.398408556 | 0.000544543 | 0.004213016 | K00799 |
| *DDB_G0268948* | 0 | 22.6301403 | Inf | Inf | 0.00055938 | 0.004307573 | - |
| *uncharacterized protein_08738* | 3.338175913 | 18.60737075 | 5.5741133 | 2.478742327 | 0.000574935 | 0.004415552 | - |
| *ALL8* | 0.351068586 | 30.87233859 | 87.93819726 | 6.458418052 | 0.000578396 | 0.004436876 | K13448 |
| *CHS* | 25.33692056 | 57.57261918 | 2.272281631 | 1.184141656 | 0.000584802 | 0.00448204 | K00660 |
| *HSP22* | 13.22889326 | 37.6427726 | 2.845496736 | 1.508680525 | 0.000595012 | 0.004549534 | K13993 |
| *FAO1* | 31.36846585 | 130.5158883 | 4.160735462 | 2.056838565 | 0.000621294 | 0.004736522 | K17756 |
| *CFAT* | 8.517949731 | 47.95379549 | 5.629734502 | 2.493066887 | 0.000622773 | 0.004742218 | - |
| *sti1* | 25.37757252 | 58.10567165 | 2.289646561 | 1.195124915 | 0.000628035 | 0.004778077 | - |
| *PCMP-H81* | 26.60483844 | 60.38466238 | 2.269687242 | 1.182493511 | 0.000648403 | 0.004907345 | - |
| *BACOVA_02659* | 158.9430541 | 2902.529344 | 18.26144187 | 4.190728776 | 0.00066117 | 0.004990628 | K05349 |
| *GAMT2* | 28.90272055 | 63.50737514 | 2.197280184 | 1.135718846 | 0.00068428 | 0.005138476 | K18886 |
| *GRF6* | 7.351114873 | 63.05896141 | 8.57814937 | 3.100666438 | 0.000695535 | 0.005201566 | - |
| *SDH* | 33.40256654 | 101.1547656 | 3.028353091 | 1.598533426 | 0.000697724 | 0.005212385 | K00008 |
| *PI4KG7* | 293.2417309 | 674.6548223 | 2.30067808 | 1.20205913 | 0.000710595 | 0.005294343 | - |
| *uncharacterized protein_06768* | 2.702666765 | 20.53745699 | 7.598960129 | 2.925802008 | 0.000721749 | 0.005366649 | - |
| *NIT4B* | 278.6447168 | 713.1427547 | 2.55932631 | 1.3557641 | 0.000728112 | 0.00541086 | K13035 |
| *PCMP-H32* | 33.94149115 | 70.66415346 | 2.081940159 | 1.057928602 | 0.000745612 | 0.005528229 | - |
| *At4g33300* | 1.31801937 | 110.7485404 | 84.02648919 | 6.392772301 | 0.000748042 | 0.005541498 | - |
| *PAO6* | 225.7535696 | 1141.421781 | 5.056051973 | 2.338011294 | 0.000766752 | 0.00566069 | K13366 |
| *uncharacterized protein_23270* | 71.71146221 | 155.9817072 | 2.175129364 | 1.121101207 | 0.00078048 | 0.005747312 | - |
| *uncharacterized protein_38958* | 1.691297298 | 13.77596124 | 8.145203835 | 3.025950803 | 0.00080968 | 0.005938723 | - |
| *CYP704C1* | 34.7273009 | 71.40049561 | 2.056033546 | 1.039863803 | 0.00082012 | 0.006000018 | - |
| *uncharacterized protein_05222* | 21.71983475 | 51.83767756 | 2.386651563 | 1.254987957 | 0.000832098 | 0.006070519 | - |
| *uncharacterized protein_06781* | 29.26308652 | 62.59490478 | 2.139039733 | 1.096963281 | 0.000833672 | 0.006080295 | - |
| *uncharacterized protein_25839* | 4.29066453 | 25.89970882 | 6.03629313 | 2.593662867 | 0.00083751 | 0.00610485 | - |
| *HSCB* | 21.99461205 | 51.10250218 | 2.323410027 | 1.216243778 | 0.00085391 | 0.00620695 | K04082 |
| *RhGT1* | 20.81331502 | 51.23461083 | 2.461626645 | 1.299611965 | 0.00089076 | 0.006446949 | - |
| *uncharacterized protein_43071* | 57.48293097 | 126.3981025 | 2.198880612 | 1.136769276 | 0.000890906 | 0.006446949 | - |
| *MYB106* | 54.81316899 | 168.26696 | 3.069827253 | 1.618157474 | 0.000921595 | 0.006644917 | K09422 |
| *COR413IM2* | 20.85876589 | 49.83194155 | 2.389016772 | 1.256416982 | 0.000927359 | 0.006679046 | - |
| *CXXS1* | 0 | 20.25402306 | Inf | Inf | 0.000937411 | 0.006742076 | K03671 |
| *SWEET1* | 42.11198687 | 89.33250181 | 2.12130817 | 1.084954222 | 0.000938753 | 0.006749858 | K15382 |
| *At1g09680* | 5.70268605 | 23.02542286 | 4.037645184 | 2.013514137 | 0.000960561 | 0.006866671 | K17964 |
| *UBP12* | 35.18425141 | 70.81664551 | 2.012737025 | 1.009158688 | 0.000961023 | 0.006868084 | K11838 |
| *ROQ1* | 31.24657155 | 64.97673171 | 2.079483555 | 1.056225276 | 0.000970419 | 0.006929501 | - |
| *At2g04570* | 1.315436978 | 101.8569465 | 77.43202307 | 6.274858431 | 0.000995427 | 0.007084666 | - |
| *KNL2* | 19.21860227 | 46.69909878 | 2.429890485 | 1.280891293 | 0.001008385 | 0.007165375 | - |
| *uncharacterized protein_17741* | 30.84695169 | 64.25620505 | 2.083064988 | 1.05870785 | 0.001016586 | 0.007221381 | - |
| *BBD1* | 10.19478486 | 30.43973958 | 2.985814806 | 1.578124685 | 0.00102 | 0.007239681 | - |
| *At4g21250* | 9.35939164 | 42.83895129 | 4.577108528 | 2.1944365 | 0.001026797 | 0.007279955 | - |
| *At4g33300* | 2.33455362 | 262.3153763 | 112.3621124 | 6.812011842 | 0.001073148 | 0.007563086 | - |
| *BAM1* | 0.331441636 | 9.016825327 | 27.20486608 | 4.765792821 | 0.001077654 | 0.007592774 | - |
| *UGT91C1* | 0 | 8.719786073 | Inf | Inf | 0.001079124 | 0.007599 | - |
| *At1g75220* | 247.3934985 | 587.1815292 | 2.37347195 | 1.246999 | 0.001082642 | 0.007621706 | K08145 |
| *CSPL5* | 161.5142326 | 391.5713994 | 2.424377053 | 1.277614093 | 0.001100477 | 0.007736758 | - |
| *arcB* | 21.37614746 | 49.81386672 | 2.330348198 | 1.220545537 | 0.00110424 | 0.007754801 | - |
| *PCMP-E53* | 27.7333033 | 58.97533841 | 2.126516909 | 1.088492327 | 0.001125653 | 0.007881702 | - |
| *VCR* | 22.88977002 | 52.60800842 | 2.298319658 | 1.200579467 | 0.001141226 | 0.007973511 | K12616 |
| *LECRK81* | 129.5026587 | 275.5360851 | 2.127648095 | 1.089259554 | 0.001175373 | 0.008179033 | - |
| *GSTF9* | 24.50462389 | 54.40136814 | 2.220045016 | 1.150588931 | 0.001188852 | 0.008259873 | K00799 |
| *AHP1* | 0 | 12.52014772 | Inf | Inf | 0.001189195 | 0.008259873 | K14490 |
| *HAK26* | 5.666014541 | 22.35460124 | 3.945383669 | 1.980165602 | 0.001201963 | 0.008326356 | K03549 |
| *cys-12* | 9.399677723 | 29.09352843 | 3.095162332 | 1.630015077 | 0.001203695 | 0.008335843 | K01738 |
| *uncharacterized protein_15644* | 1.696462081 | 19.85978312 | 11.70658828 | 3.549248779 | 0.001223752 | 0.00845442 | - |
| *At1g56220* | 1.652043398 | 131.7434697 | 79.74576809 | 6.317336056 | 0.001225672 | 0.008460924 | - |
| *DBR* | 54.45243714 | 166.2637301 | 3.053375364 | 1.610404957 | 0.001246222 | 0.008582217 | K07119 |
| *uncharacterized protein_15372* | 22.28938218 | 50.76141838 | 2.277381131 | 1.187375754 | 0.001250179 | 0.008604895 | - |
| *SSL4* | 0.657718489 | 10.33590001 | 15.71477795 | 3.974049981 | 0.001263508 | 0.008685111 | - |
| *CYP90D1* | 75.50783841 | 159.2748869 | 2.109382155 | 1.076820491 | 0.001270425 | 0.008722053 | K12638 |
| *Kdsr* | 3.326296138 | 28.05448653 | 8.434151793 | 3.076242987 | 0.001297278 | 0.008879575 | K04708 |
| *MENG* | 4.986086711 | 22.71708684 | 4.556095422 | 2.187797963 | 0.001304861 | 0.008915002 | K03183 |
| *uncharacterized protein_28000* | 19.52525217 | 50.52761532 | 2.587808592 | 1.371730912 | 0.001324782 | 0.009026405 | - |
| *OMT2* | 0 | 7.190643659 | Inf | Inf | 0.001386035 | 0.009392866 | - |
| *CAR10* | 0 | 14.967421 | Inf | Inf | 0.001401359 | 0.009486798 | K12486 |
| *EXPA8* | 6.750727025 | 50.21476134 | 7.438422729 | 2.894996739 | 0.001404081 | 0.009500283 | - |
| *GMPM1* | 0 | 19.18563454 | Inf | Inf | 0.001416867 | 0.009571788 | - |
| *DBR4* | 8.355769348 | 27.07873002 | 3.240722535 | 1.696315505 | 0.001428255 | 0.00963617 | - |
| *RCOM_1506700* | 31.1558181 | 63.44642253 | 2.036422935 | 1.026037219 | 0.001431518 | 0.009653161 | K01267 |
| *CYP750A1* | 0 | 15.04421331 | Inf | Inf | 0.001451426 | 0.009779776 | - |
| *At1g56220* | 2.41306142 | 67.79113418 | 28.09341429 | 4.812160066 | 0.001455229 | 0.009800305 | - |
| *At4g27520* | 193.4410286 | 426.4141964 | 2.204362743 | 1.140361649 | 0.001457101 | 0.009810365 | - |
| *H1* | 2.997436893 | 16.22442626 | 5.412766585 | 2.436366176 | 0.001468343 | 0.009880924 | K11275 |
| *Os01g0290100* | 7.477655931 | 25.00635593 | 3.344143694 | 1.74163684 | 0.001469669 | 0.009882152 | - |
| *uncharacterized protein_22757* | 1.69387969 | 13.00121082 | 7.675403928 | 2.940242676 | 0.00153915 | 0.010295947 | - |
| *uncharacterized protein_47964* | 1.320601762 | 67.45121715 | 51.07612235 | 5.674577096 | 0.00154277 | 0.010312177 | - |
| *Sb03g046810* | 774.5907321 | 1811.006154 | 2.338016812 | 1.225285304 | 0.001563615 | 0.010432687 | K00430 |
| *AGLU* | 17.6340708 | 42.69668841 | 2.421261029 | 1.275758621 | 0.001570109 | 0.010473315 | K01187 |
| *TJ* | 69.93619577 | 152.4569822 | 2.179943884 | 1.124290998 | 0.001595866 | 0.010625994 | K08906 |
| *uncharacterized protein_38991* | 0.989160125 | 21.78316548 | 22.02187989 | 4.460865724 | 0.001603543 | 0.010660681 | - |
| *uncharacterized protein_34546* | 1.65462579 | 47.89962018 | 28.9489143 | 4.855437337 | 0.001628822 | 0.010806576 | - |
| *UBI11* | 1.691297298 | 12.76405582 | 7.546902506 | 2.915884637 | 0.001630266 | 0.01081339 | K08770 |
| *uncharacterized protein_39926* | 9.146743887 | 37.08938594 | 4.054927786 | 2.019676222 | 0.001646171 | 0.010902153 | - |
| *GAF1* | 41.3680134 | 88.31942886 | 2.134969064 | 1.094215165 | 0.001657389 | 0.010965242 | K03539 |
| *uncharacterized protein_02165* | 6.360404539 | 23.11691309 | 3.634503584 | 1.861758328 | 0.001679743 | 0.011077737 | - |
| *uncharacterized protein_24017* | 0 | 18.07086793 | Inf | Inf | 0.001687817 | 0.011121147 | - |
| *VIR* | 55.96192711 | 122.8566435 | 2.195361201 | 1.134458325 | 0.001764055 | 0.011552554 | - |
| *uncharacterized protein_11342* | 15.56344688 | 38.57676814 | 2.478677662 | 1.309570669 | 0.001770886 | 0.011583131 | - |
| *uncharacterized protein_02859* | 0 | 7.017842405 | Inf | Inf | 0.001779896 | 0.011636194 | - |
| *SSL4* | 426.4492806 | 1050.22405 | 2.462717367 | 1.300251067 | 0.001787638 | 0.011680915 | - |
| *nep1* | 7.373324214 | 25.39818815 | 3.444604824 | 1.784338482 | 0.001809394 | 0.011793355 | - |
| *GOLS4* | 4.131732843 | 31.30362247 | 7.576390744 | 2.921510738 | 0.00185771 | 0.012062788 | K18819 |
| *MFT1* | 0.331441636 | 19.91288931 | 60.07962527 | 5.908803909 | 0.001887492 | 0.012219449 | - |
| *CIPK5* | 29.47573427 | 61.54352273 | 2.087938579 | 1.062079272 | 0.001934755 | 0.012484898 | K07198 |
| *At3g01520* | 28.003948 | 126.8070395 | 4.528184367 | 2.178932699 | 0.001970747 | 0.012695039 | - |
| *yipf1* | 17.57172365 | 41.52101915 | 2.362945148 | 1.24058614 | 0.002005893 | 0.012886222 | - |
| *At3g03770* | 9.905179518 | 38.06851937 | 3.843294238 | 1.942343432 | 0.002061616 | 0.013191899 | - |
| *At4g22758* | 2.376389912 | 14.30223565 | 6.01847179 | 2.589397204 | 0.002068286 | 0.013222234 | - |
| *DRP1C* | 6.068216803 | 21.72330545 | 3.579849923 | 1.839899107 | 0.002069958 | 0.013225696 | K01528 |
| *VTC4* | 31.76328682 | 69.92324341 | 2.201385638 | 1.138411898 | 0.002129942 | 0.013548859 | K10047 |
| *SPX2* | 14.1762171 | 36.42529318 | 2.569464966 | 1.361467981 | 0.002201436 | 0.013952295 | - |
| *ASR1* | 0 | 22.0406297 | Inf | Inf | 0.002252128 | 0.01424571 | - |
| *CSLE6* | 10.82357902 | 31.04047278 | 2.867856623 | 1.519972899 | 0.002264402 | 0.014309385 | - |
| *RBOHC* | 132.0956114 | 291.9545661 | 2.210176121 | 1.144161338 | 0.002280365 | 0.014389217 | K13447 |
| *uncharacterized protein_10512* | 6.755891808 | 23.0751279 | 3.415556163 | 1.772120515 | 0.002309298 | 0.01454701 | - |
| *CHIT5* | 13.43895863 | 35.32641623 | 2.628657266 | 1.39432605 | 0.002340923 | 0.014721184 | K01183 |
| *DFR1* | 3.338175913 | 18.08561505 | 5.417813657 | 2.437710774 | 0.002351863 | 0.014782812 | K13082 |
| *RGP5* | 9.559275715 | 27.83200446 | 2.91151812 | 1.541771598 | 0.00235593 | 0.014799704 | K13379 |
| *RD19D* | 2.398599254 | 73.09014983 | 30.47201391 | 4.929412947 | 0.002460062 | 0.015388294 | K01373 |
| *At5g58480* | 18.76165176 | 42.5114978 | 2.265871809 | 1.180066243 | 0.002505473 | 0.015630875 | - |
| *MAN6* | 60.31250467 | 121.4155902 | 2.013108075 | 1.009424627 | 0.002519116 | 0.015697102 | K19355 |
| *PCMP-E53* | 4.705778749 | 19.04781543 | 4.047749893 | 2.01712015 | 0.002593974 | 0.016097785 | - |
| *GOLS4* | 4.713525924 | 27.42202398 | 5.817730594 | 2.54045649 | 0.002598166 | 0.016119946 | K18819 |
| *HIPP20* | 0.679927831 | 23.4817572 | 34.53566119 | 5.11001494 | 0.002638456 | 0.016346446 | - |
| *RUN1* | 12.72235929 | 33.23713407 | 2.612497676 | 1.385429754 | 0.0026699 | 0.016507327 | - |
| *CSLE6* | 43.95048435 | 88.12969532 | 2.005204189 | 1.003749153 | 0.002710923 | 0.016743402 | - |
| *PCMP-E90* | 25.233103 | 54.9513037 | 2.177746578 | 1.122836079 | 0.002802542 | 0.017247112 | - |
| *uncharacterized protein_28921* | 154.5167033 | 360.3249287 | 2.331948074 | 1.221535664 | 0.00286556 | 0.017564597 | - |
| *Eapp* | 21.30951943 | 46.78944723 | 2.195706354 | 1.134685126 | 0.00293774 | 0.01791386 | - |
| *CEL1* | 2.000529593 | 16.37009103 | 8.182878721 | 3.03260847 | 0.002944246 | 0.01794509 | K01179 |
| *RNR1* | 26.17334597 | 53.27314457 | 2.035396798 | 1.025310073 | 0.002992376 | 0.018191402 | - |
| *BGLU43* | 0.660300881 | 9.543099739 | 14.45265335 | 3.853262475 | 0.00300692 | 0.018271253 | K05350 |
| *DHNAT1* | 7.712513026 | 24.46650117 | 3.17231246 | 1.665534878 | 0.003018753 | 0.018330263 | - |
| *ZDS* | 287.2577047 | 584.3024218 | 2.034070496 | 1.02436968 | 0.003034476 | 0.01840849 | K00514 |
| *CRRSP38* | 0 | 6.153910317 | Inf | Inf | 0.003095993 | 0.018715994 | - |
| *CNMT* | 28.33642182 | 57.41446664 | 2.026172076 | 1.018756703 | 0.0031276 | 0.018858702 | K13384 |
| *PNC1* | 0.328859245 | 7.841156071 | 23.84350204 | 4.575524244 | 0.003221022 | 0.019363476 | K00430 |
| *PCMP-H40* | 3.340758304 | 15.76024624 | 4.717565537 | 2.23804256 | 0.003317093 | 0.019835237 | - |
| *CAD1* | 0.351068586 | 7.724837921 | 22.00378565 | 4.459679849 | 0.003330995 | 0.019900007 | K00083 |
| *PP2C27* | 9.816342151 | 28.67227539 | 2.920871639 | 1.546398959 | 0.003362732 | 0.020061875 | K14803 |
| *At3g03770* | 3.01189906 | 14.69980255 | 4.880576095 | 2.287051451 | 0.003373051 | 0.020114082 | - |
| *Ts* | 119.2083411 | 292.6680664 | 2.455097217 | 1.295780154 | 0.00337381 | 0.020114082 | K02357 |
| *GSTU19* | 12.42345656 | 32.59449231 | 2.623625088 | 1.391561576 | 0.003392307 | 0.020210415 | K00799 |
| *SbtS* | 154.3020528 | 360.5546082 | 2.336680567 | 1.224460526 | 0.003411986 | 0.020312882 | - |
| *CAD* | 0 | 6.288278324 | Inf | Inf | 0.003463141 | 0.020542738 | K00083 |
| *PAA2* | 4.686151799 | 18.54862829 | 3.958179139 | 1.984836907 | 0.003613902 | 0.021281229 | K01533 |
| *ATJ11* | 5.719730608 | 20.53969136 | 3.591024258 | 1.844395399 | 0.0036372 | 0.0214087 | - |
| *CYC2* | 44.35253834 | 90.78050578 | 2.046793919 | 1.033365853 | 0.003638335 | 0.021410522 | - |
| *prfC* | 20.70138441 | 44.46638698 | 2.147990979 | 1.102987935 | 0.003673468 | 0.021597661 | - |
| *SPP2* | 6.340777589 | 21.6848722 | 3.419907401 | 1.773957263 | 0.003680959 | 0.021626991 | - |
| *OEP16* | 0 | 6.132434339 | Inf | Inf | 0.003705271 | 0.021755044 | - |
| *APF2* | 0 | 6.568360309 | Inf | Inf | 0.003753333 | 0.021982477 | - |
| *PMRT15* | 0 | 6.09064915 | Inf | Inf | 0.00377403 | 0.022093713 | K02516 |
| *CYP750A1* | 0 | 18.22790291 | Inf | Inf | 0.003777546 | 0.022109302 | - |
| *HSR201* | 9.13641432 | 201.8812549 | 22.09633318 | 4.465735073 | 0.003793572 | 0.02219308 | K19861 |
| *PNC1* | 10.12402424 | 583.8700373 | 57.67173444 | 5.849792506 | 0.003839099 | 0.022408861 | K00430 |
| *CYP71AU50* | 442.013908 | 1145.33344 | 2.591170593 | 1.373604001 | 0.003906596 | 0.022766966 | - |
| *LOX1.1* | 0 | 21.04677413 | Inf | Inf | 0.003911977 | 0.022782961 | K15718 |
| *IREG2* | 0 | 8.020710409 | Inf | Inf | 0.00395033 | 0.022965056 | K14685 |
| *At5g48740* | 0 | 5.946101938 | Inf | Inf | 0.004025487 | 0.023339185 | - |
| *At1g13630* | 3.373297213 | 15.36158676 | 4.553878828 | 2.187095904 | 0.004038954 | 0.023406797 | - |
| *TSJT1* | 58.98828834 | 132.5160872 | 2.246481308 | 1.167667058 | 0.004058201 | 0.023502574 | - |
| *Os05g0277500* | 19.11530273 | 41.77175451 | 2.185252051 | 1.127799693 | 0.00407484 | 0.02358603 | - |
| *MYB2* | 0 | 5.906576104 | Inf | Inf | 0.00409813 | 0.023691486 | K09422 |
| *TET8* | 0.331441636 | 54.95271799 | 165.7990789 | 7.373292181 | 0.004150095 | 0.023938525 | - |
| *NO93* | 1.65462579 | 11.34671284 | 6.857570402 | 2.777697528 | 0.00416469 | 0.024012025 | - |
| *ABCG22* | 50.8229574 | 104.984349 | 2.065687523 | 1.046622034 | 0.004225908 | 0.024321718 | - |
| *PCMP-H44* | 6.834399608 | 21.85315475 | 3.197523704 | 1.676955054 | 0.004251485 | 0.024458064 | - |
| *CRK2* | 2.665995257 | 13.93180522 | 5.225742689 | 2.385636089 | 0.004276747 | 0.024576129 | - |
| *XTH7* | 4.379501896 | 29.08786793 | 6.641821061 | 2.731578855 | 0.004305672 | 0.024720429 | K08235 |
| *THA8L* | 334.9725405 | 737.328753 | 2.201161778 | 1.138265183 | 0.004418416 | 0.025244753 | - |
| *uncharacterized protein_42826* | 33.53567431 | 69.09995472 | 2.060490989 | 1.042988154 | 0.004499207 | 0.025661117 | - |
| *MKK4* | 31.09125444 | 67.58337425 | 2.173710114 | 1.120159556 | 0.004588713 | 0.026091259 | K20604 |
| *ASNS* | 0.657718489 | 9.863874325 | 14.99710665 | 3.906612287 | 0.004597963 | 0.026132397 | K01953 |
| *At4g16230* | 2.005694376 | 14.24575254 | 7.102653682 | 2.828358143 | 0.004626437 | 0.026276944 | - |
| *SB09* | 33.305982 | 83.51175544 | 2.507410094 | 1.326197972 | 0.004643834 | 0.026364209 | K03671 |
| *CYP78A4* | 55.29232885 | 237.5682879 | 4.296586758 | 2.103191027 | 0.004653529 | 0.026407683 | - |
| *At1g56140* | 5.415663097 | 18.8784403 | 3.485896364 | 1.801529679 | 0.00466042 | 0.026435217 | - |
| *BRL2* | 13.32031302 | 32.26701384 | 2.42239156 | 1.276432084 | 0.004715578 | 0.026701353 | - |
| *uncharacterized protein_08976* | 0.331441636 | 7.168075093 | 21.62696025 | 4.434758998 | 0.004838425 | 0.027248193 | - |
| *COMT1* | 0 | 19.93660042 | Inf | Inf | 0.004846936 | 0.027284272 | - |
| *UGT91B1* | 39.79536171 | 99.03370488 | 2.488574061 | 1.315319323 | 0.004904405 | 0.027559906 | - |
| *Noca_2408* | 15.37647477 | 36.23444207 | 2.356485645 | 1.236636893 | 0.005005336 | 0.028011734 | K07447 |
| *At1g30440* | 1.342811103 | 10.47821284 | 7.803191989 | 2.964064396 | 0.005019941 | 0.028081343 | - |
| *SWEET16* | 0.657718489 | 8.489409128 | 12.90735971 | 3.690122013 | 0.005052626 | 0.028221564 | K15382 |
| *uncharacterized protein_35311* | 12.40537982 | 31.7825496 | 2.5619973 | 1.357268955 | 0.005062202 | 0.028262874 | - |
| *EIX2* | 2.63603874 | 29.49546568 | 11.18931419 | 3.484049709 | 0.005171041 | 0.028759062 | - |
| *GIS2* | 0 | 9.124131038 | Inf | Inf | 0.005248493 | 0.029133577 | - |
| *uncharacterized protein_29636* | 7.048079545 | 104.2650898 | 14.79340423 | 3.886882176 | 0.005275265 | 0.029238362 | - |
| *OMT2* | 0.679927831 | 57.01915985 | 83.86060588 | 6.389921348 | 0.005364525 | 0.029638057 | - |
| *ANR* | 530.1798425 | 1065.154516 | 2.009043783 | 1.006509005 | 0.005414492 | 0.029856486 | K08695 |
| *DCR* | 0 | 8.722045428 | Inf | Inf | 0.005415576 | 0.029856486 | - |
| *RNR1* | 18.12252803 | 40.05394616 | 2.210174325 | 1.144160165 | 0.005503995 | 0.030196271 | K10807 |
| *DAO* | 0.331441636 | 43.19118426 | 130.3130915 | 7.025838216 | 0.005682579 | 0.031057736 | - |
| *DAD2* | 92.36709533 | 231.7867465 | 2.5094082 | 1.32734717 | 0.00573996 | 0.031292198 | - |
| *SUS3* | 0.351068586 | 7.109332635 | 20.25055192 | 4.339889324 | 0.005785666 | 0.031501628 | K00695 |
| *SCPL26* | 11.78691523 | 29.96873231 | 2.542542449 | 1.346271861 | 0.006074575 | 0.032888198 | K16297 |
| *uncharacterized protein_07413* | 10.58510735 | 27.46043224 | 2.594251654 | 1.375318434 | 0.006267103 | 0.03373333 | - |
| *MTERF4* | 0.328859245 | 9.049573099 | 27.51807421 | 4.782307605 | 0.00634747 | 0.034109262 | K15032 |
| *DOT4* | 0 | 5.633247203 | Inf | Inf | 0.006530643 | 0.034892103 | - |
| *TH* | 0.660300881 | 8.04896445 | 12.18984357 | 3.607607708 | 0.006774193 | 0.035999444 | K00500 |
| *BRXL4* | 11.46425295 | 28.32669787 | 2.470871673 | 1.305020085 | 0.006779734 | 0.036014125 | - |
| *uncharacterized protein_12594* | 1.404274345 | 11.76118781 | 8.375277847 | 3.066137052 | 0.006816757 | 0.036171151 | - |
| *MYB2* | 0 | 5.523707117 | Inf | Inf | 0.006846422 | 0.036294017 | K09422 |
| *PME51* | 62.94890929 | 135.9389686 | 2.159512692 | 1.110705796 | 0.006981323 | 0.036865937 | - |
| *ROQ1* | 1.362438053 | 16.28761324 | 11.95475508 | 3.579512669 | 0.006993378 | 0.036913963 | - |
| *uncharacterized protein_27858* | 6.330448022 | 20.16928592 | 3.18607559 | 1.671780495 | 0.007040915 | 0.037097507 | - |
| *DLO1* | 5.120892969 | 27.05953838 | 5.284144493 | 2.401669917 | 0.007069539 | 0.037210519 | - |
| *PTCH1* | 3.741410357 | 15.16055644 | 4.052096667 | 2.018668592 | 0.007076138 | 0.037227552 | K11101 |
| *ATL77* | 1.030996417 | 8.865500052 | 8.598963008 | 3.104162689 | 0.007111245 | 0.037376925 | K19040 |
| *UGT85A5* | 5.640190625 | 19.01506766 | 3.37135195 | 1.753327244 | 0.007191571 | 0.037734556 | - |
| *PCMP-H40* | 22.77459072 | 46.17508373 | 2.027482483 | 1.01968945 | 0.007275489 | 0.038116562 | - |
| *MYB83* | 5.817865358 | 18.71241711 | 3.216371634 | 1.685434112 | 0.007294331 | 0.038207559 | K09422 |
| *POR1* | 0 | 5.372381841 | Inf | Inf | 0.007318367 | 0.038313527 | K00218 |
| *PCBER* | 18.76939893 | 39.5548332 | 2.107410757 | 1.075471538 | 0.00732195 | 0.038313527 | - |
| *TKTC* | 10.1033651 | 26.76021479 | 2.648643745 | 1.405253808 | 0.007347633 | 0.03842465 | K00615 |
| *At5g57200* | 13.42707885 | 31.78364218 | 2.367130076 | 1.243138986 | 0.00735853 | 0.038458371 | K20043 |
| *RPS5* | 4.312873871 | 16.30573728 | 3.78071276 | 1.918658245 | 0.007479648 | 0.039008066 | K13459 |
| *MES17* | 2.663412865 | 13.15479545 | 4.939074834 | 2.304240828 | 0.007490873 | 0.039047721 | - |
| *CSLE6* | 0.331441636 | 6.855220358 | 20.68303921 | 4.370376289 | 0.00752575 | 0.039190128 | - |
| *OMT2* | 0.657718489 | 83.2337852 | 126.5492556 | 6.983555211 | 0.007706603 | 0.040027405 | - |
| *PCMP-H81* | 14.01558692 | 36.08535117 | 2.574658583 | 1.364381134 | 0.007721748 | 0.040092637 | - |
| *RAP* | 8.896392442 | 23.67032398 | 2.660665447 | 1.411787117 | 0.007839378 | 0.04065188 | K08245 |
| *PCMP-H85* | 18.014212 | 38.26843211 | 2.124346717 | 1.087019249 | 0.007911718 | 0.040977834 | - |
| *EMB2261* | 9.81375976 | 25.56537811 | 2.605054407 | 1.381313504 | 0.007929107 | 0.041051493 | - |
| *EXPA8* | 20.18452087 | 99.18420931 | 4.913874843 | 2.296861113 | 0.007947037 | 0.041127901 | - |
| *BACOVA_02659* | 42.61748866 | 106.9402823 | 2.509305116 | 1.327287904 | 0.007959739 | 0.041168983 | K05349 |
| *ANR* | 115.6931567 | 232.8104202 | 2.012309343 | 1.008852101 | 0.008011271 | 0.041402476 | K13082 |
| *CYP735A2* | 163.7437189 | 471.4678536 | 2.879303442 | 1.525719839 | 0.008022349 | 0.041451467 | K10717 |
| *PNC2* | 4.065104818 | 18.20195818 | 4.477611031 | 2.162729207 | 0.008069932 | 0.041664119 | K00430 |
| *PCMP-H81* | 11.09097502 | 28.403416 | 2.560948514 | 1.356678249 | 0.008266036 | 0.042524162 | - |
| *GAST1* | 31.45317062 | 111.3290378 | 3.539517179 | 1.823552578 | 0.008277664 | 0.042575538 | - |
| *PCMP-H24* | 37.03293019 | 75.95400831 | 2.050985648 | 1.036317396 | 0.008463544 | 0.043393864 | - |
| *uncharacterized protein_47665* | 1.011369467 | 8.804498239 | 8.705521104 | 3.121930659 | 0.008490312 | 0.043522502 | K06995 |
| *RBL1* | 44.41916636 | 140.795256 | 3.169696046 | 1.664344501 | 0.00849887 | 0.043557761 | - |
| *PSPTO_1126* | 2.747085448 | 16.13402861 | 5.873144071 | 2.554133028 | 0.008565863 | 0.043866424 | K06955 |
| *UGD5* | 6.110053094 | 21.97059047 | 3.595810073 | 1.846316821 | 0.008713465 | 0.044464225 | K00012 |
| *UGT86A1* | 4.327336038 | 16.08663213 | 3.717444633 | 1.894311255 | 0.008733447 | 0.044532303 | - |
| *ATJ11* | 8.988696104 | 24.14577578 | 2.68623786 | 1.425587057 | 0.008733676 | 0.044532303 | - |
| *ABCI17* | 0 | 6.906042965 | Inf | Inf | 0.00880643 | 0.044859156 | - |
| *KDM1B* | 0.986577734 | 8.657691673 | 8.775478481 | 3.13347779 | 0.008876482 | 0.045127339 | - |
| *At4g33300* | 4.997966486 | 20.23138032 | 4.047922365 | 2.017181621 | 0.008885041 | 0.045160013 | - |
| *HMT/HLT* | 3.71145384 | 81.26993279 | 21.89706144 | 4.45266537 | 0.008954339 | 0.045442964 | K19861 |
| *HVA22* | 0 | 11.13550332 | Inf | Inf | 0.009028355 | 0.045755898 | K17279 |
| *PAD4* | 54.54577299 | 251.9570546 | 4.6191857 | 2.207638546 | 0.009039661 | 0.045795299 | - |
| *KING1* | 1.735715981 | 10.43868701 | 6.014052485 | 2.588337458 | 0.00904745 | 0.045825803 | - |
| *R40C1* | 0 | 5.276372903 | Inf | Inf | 0.009083113 | 0.045948275 | - |
| *ARP* | 21.05850168 | 43.24776576 | 2.053696242 | 1.038222811 | 0.009084035 | 0.045948275 | K01142 |
| *FDH* | 139.7537421 | 502.1153357 | 3.592857896 | 1.845131875 | 0.009188089 | 0.046411217 | K15397 |
| *NAGS2* | 58.95161683 | 125.0792253 | 2.1217268 | 1.085238902 | 0.009249093 | 0.046692072 | K14682 |
| *SLAH2* | 0 | 5.197321233 | Inf | Inf | 0.009398236 | 0.047343592 | - |
| *ASPG2* | 0 | 6.516395914 | Inf | Inf | 0.009428615 | 0.047471947 | - |
| *EXPA8* | 0.328859245 | 6.689197168 | 20.34060857 | 4.346290938 | 0.009442217 | 0.047509761 | - |
| *CCL5* | 0 | 16.26164354 | Inf | Inf | 0.009465101 | 0.047597195 | K10526 |
| *MIF2* | 11.58459704 | 27.79925669 | 2.399674032 | 1.262838446 | 0.009476731 | 0.047646437 | - |
| *CRTISO* | 0.351068586 | 6.649671333 | 18.94123141 | 4.243458221 | 0.009497221 | 0.047730947 | K09835 |
| *PME51* | 9.006772846 | 55.23823718 | 6.132966615 | 2.616585098 | 0.009668284 | 0.048384341 | - |
| *uncharacterized protein_13234* | 15.51386342 | 327.9135407 | 21.13680724 | 4.401685566 | 0.009745751 | 0.048715605 | - |
| *XYL1* | 1.404274345 | 9.499055195 | 6.764387051 | 2.757959212 | 0.009870049 | 0.049229439 | K01187 |
| *HMGB15* | 19.22531726 | 39.75578934 | 2.067887297 | 1.048157559 | 0.009908212 | 0.049318509 | - |
| *NCED1* | 3.382594596 | 13.89002003 | 4.106321239 | 2.037846494 | 0.009977663 | 0.049626099 | K09840 |
| *CYP76B10* | 4.322171255 | 16.13745473 | 3.73364538 | 1.900584907 | 0.010198315 | 0.050597379 | - |
| *WSD9* | 0.351068586 | 6.423813097 | 18.29788636 | 4.193605104 | 0.010256825 | 0.050858475 | - |
| *GLIP6* | 1.65462579 | 38.06847016 | 23.00729893 | 4.524019715 | 0.01027971 | 0.050942722 | - |
| *H2AX* | 8.712002718 | 23.13612972 | 2.655661444 | 1.409071237 | 0.010354718 | 0.051236095 | K11251 |
| *At1g73050* | 0.351068586 | 9.277715668 | 26.42707446 | 4.723944819 | 0.010362624 | 0.051255648 | - |
| *uncharacterized protein_01834* | 1.983485034 | 10.93335544 | 5.512194571 | 2.462626814 | 0.010409926 | 0.051428549 | - |
| *At3g01520* | 20.53662164 | 591.5653322 | 28.80538692 | 4.848266731 | 0.010432294 | 0.051502931 | - |
| *HIR4* | 15.35426543 | 35.7837932 | 2.330544133 | 1.220666833 | 0.010511203 | 0.051792945 | - |
| *SIS8* | 6.792563317 | 19.95018077 | 2.937062172 | 1.554373805 | 0.010542076 | 0.05190697 | - |
| *uncharacterized protein_19721* | 11.37866428 | 33.88538713 | 2.977975824 | 1.574332042 | 0.010589477 | 0.052069783 | - |
| *SIS8* | 4.727988091 | 22.33650218 | 4.724314392 | 2.240104976 | 0.010729425 | 0.052597525 | - |
| *TSJT1* | 11.41983427 | 27.37462749 | 2.397112502 | 1.26129762 | 0.010913056 | 0.053397567 | - |
| *CRJ33* | 23.37512684 | 124.1101228 | 5.309495156 | 2.408574692 | 0.010965532 | 0.053613865 | - |
| *HSP12* | 14.88093666 | 32.28848982 | 2.169788808 | 1.117554628 | 0.011129234 | 0.054294102 | K13993 |
| *GME-1* | 5.380541797 | 23.13389534 | 4.299547558 | 2.104184853 | 0.01122242 | 0.054666877 | K10046 |
| *At3g13620* | 18.21394779 | 38.09900778 | 2.091749039 | 1.064709772 | 0.011280199 | 0.054914193 | - |
| *PBL7* | 4.629853341 | 33.60528092 | 7.258389942 | 2.859649565 | 0.011327871 | 0.055104886 | K04733 |
| *MYB305* | 0.994324909 | 8.291779955 | 8.339105137 | 3.059892578 | 0.011373914 | 0.05527701 | K09422 |
| *Os07g0631100* | 2.76929479 | 33.51495746 | 12.10234374 | 3.597214562 | 0.011430495 | 0.055510373 | - |
| *SMARCAL1* | 15.5582821 | 34.24899029 | 2.201334959 | 1.138378685 | 0.011775364 | 0.056918649 | K14440 |
| *mpaB&apos;* | 0 | 21.22066797 | Inf | Inf | 0.011799754 | 0.057025912 | - |
| *RBCX1* | 20.71584658 | 41.59887908 | 2.008070436 | 1.005809875 | 0.011840042 | 0.057177987 | - |
| *Ncoa7* | 5.694938875 | 17.61468195 | 3.093041443 | 1.629026164 | 0.01197509 | 0.05772265 | - |
| *BHLH25* | 13.82256612 | 31.53965991 | 2.281751423 | 1.190141631 | 0.012014398 | 0.057869088 | - |
| *IREG2* | 3.956640502 | 14.87369639 | 3.759173062 | 1.910415335 | 0.012226518 | 0.058727149 | K14685 |
| *OXR1* | 3.380012204 | 13.40779016 | 3.966787501 | 1.987971115 | 0.012245594 | 0.058775226 | - |
| *CRR21* | 14.33839748 | 31.94062796 | 2.227628855 | 1.155508885 | 0.012354924 | 0.059179468 | - |
| *CDT1A* | 44.85804013 | 95.52156761 | 2.129419104 | 1.090459923 | 0.012395699 | 0.059341893 | K10727 |
| *RXW8* | 4.961294977 | 16.43223463 | 3.312085798 | 1.727740046 | 0.012416306 | 0.059429572 | - |
| *CRK3* | 6.315985856 | 19.24318525 | 3.04674293 | 1.607267777 | 0.012428158 | 0.059475317 | - |
| *rib5* | 15.597536 | 33.09710557 | 2.12194449 | 1.085386916 | 0.012471925 | 0.05962974 | K00793 |
| *uncharacterized protein_47446* | 1.325766545 | 31.18390318 | 23.52141355 | 4.555902858 | 0.0125163 | 0.059808814 | - |
| *PP2C06* | 28.14908383 | 56.84645624 | 2.019478026 | 1.013982448 | 0.012626291 | 0.060223413 | K14497 |
| *GT4* | 0 | 4.910461185 | Inf | Inf | 0.012684017 | 0.060409843 | - |
| *uncharacterized protein_25213* | 3.738827965 | 16.90426032 | 4.521272568 | 2.176728894 | 0.012790816 | 0.060751103 | - |
| *At1g05080* | 0 | 4.877713413 | Inf | Inf | 0.012864671 | 0.061023635 | - |
| *At4g10955* | 0 | 4.835928224 | Inf | Inf | 0.013100579 | 0.061938697 | - |
| *Os12g0628600* | 0 | 9.660584655 | Inf | Inf | 0.01332986 | 0.06281654 | - |
| *uncharacterized protein_46559* | 0.328859245 | 8.146065976 | 24.77067654 | 4.630561367 | 0.01342981 | 0.06323009 | - |
| *PCMP-H42* | 14.34356226 | 34.14744424 | 2.380680866 | 1.251374238 | 0.013500534 | 0.063470868 | - |
| *At5g46170* | 6.065634411 | 17.93428977 | 2.956704701 | 1.563990162 | 0.013536531 | 0.063617036 | - |
| *uncharacterized protein_08850* | 0.351068586 | 6.099686568 | 17.37462937 | 4.118910298 | 0.013543027 | 0.063636026 | - |
| *PCMP-H40* | 20.31194583 | 40.71123664 | 2.004300178 | 1.003098593 | 0.013576755 | 0.063748299 | - |
| *uncharacterized protein_15757* | 70.75092599 | 144.172031 | 2.037740553 | 1.026970378 | 0.013675718 | 0.064189721 | - |
| *VIP5* | 7.062541711 | 19.9897066 | 2.830384218 | 1.500997909 | 0.013701167 | 0.064285894 | K15178 |
| *STY13* | 11.52055141 | 50.90710738 | 4.418808231 | 2.143657322 | 0.013902549 | 0.065077684 | - |
| *At4g18465* | 10.0666936 | 25.28864807 | 2.512110638 | 1.328900004 | 0.013916281 | 0.06511845 | K13117 |
| *uncharacterized protein_00552* | 5.471961555 | 23.00623122 | 4.204384659 | 2.071894668 | 0.013962305 | 0.065298459 | - |
| *yin* | 18.73944241 | 38.38360848 | 2.048279113 | 1.03441232 | 0.014001326 | 0.065449901 | K14638 |
| *LTPG14* | 20.08225352 | 72.87881617 | 3.629015843 | 1.859578355 | 0.014064069 | 0.065703274 | - |
| *CYP71AU50* | 35.34834788 | 87.96590726 | 2.488543667 | 1.315301703 | 0.014464662 | 0.067368377 | - |
| *SYT1* | 58.51584347 | 121.9385134 | 2.083854665 | 1.059254663 | 0.014497073 | 0.067470852 | - |
| *GLIP6* | 1.671670348 | 11.22699355 | 6.716033196 | 2.747609361 | 0.01457465 | 0.067765727 | - |
| *MSL2* | 3.959222893 | 14.50552532 | 3.663730411 | 1.873313349 | 0.014576113 | 0.067765727 | - |
| *CHX20* | 11.43687882 | 50.59646356 | 4.423974787 | 2.145343163 | 0.014725879 | 0.068327233 | - |
| *PUX7* | 0.328859245 | 5.867050269 | 17.84061225 | 4.157093221 | 0.014797456 | 0.068585708 | - |
| *At2g29640* | 11.87420239 | 27.12051521 | 2.28398627 | 1.191553978 | 0.01480303 | 0.068599279 | K15235 |
| *BEBT1* | 0.351068586 | 11.55563879 | 32.91561604 | 5.040700294 | 0.014975613 | 0.069275233 | K19861 |
| *uncharacterized protein_18636* | 12.39453222 | 28.3978047 | 2.291155825 | 1.196075582 | 0.01502451 | 0.069476636 | K15032 |
| *frr* | 15.48752148 | 32.72101464 | 2.112734093 | 1.079111203 | 0.015343928 | 0.070638735 | K02838 |
| *PCMP-H42* | 6.46112168 | 19.38438052 | 3.000157167 | 1.58503808 | 0.015767288 | 0.072224818 | - |
| *APC6* | 9.344929474 | 23.36876602 | 2.500689393 | 1.322325874 | 0.015916575 | 0.072707004 | K03353 |
| *AAE1* | 240.8554192 | 553.4077198 | 2.297676015 | 1.200175384 | 0.01598301 | 0.072946222 | - |
| *PCMP-E33* | 0 | 4.715091366 | Inf | Inf | 0.016110111 | 0.073435823 | - |
| *PNC1* | 0 | 10.63857554 | Inf | Inf | 0.016196029 | 0.073762626 | K00430 |
| *At2g15980* | 22.63890055 | 45.80462908 | 2.023270917 | 1.01668951 | 0.016354269 | 0.074403941 | - |
| *PCMP-E76* | 15.70533401 | 32.95253338 | 2.09817463 | 1.069134758 | 0.016418642 | 0.074645372 | - |
| *uncharacterized protein_03577* | 11.024347 | 25.59363215 | 2.321555387 | 1.2150917 | 0.016673848 | 0.07563323 | - |
| *COMT1* | 0 | 7.212119637 | Inf | Inf | 0.016684851 | 0.075669901 | - |
| *COMT1* | 0.328859245 | 21.35394339 | 64.93338333 | 6.020888477 | 0.016882169 | 0.076431093 | - |
| *PDF2* | 8.405352815 | 52.10080303 | 6.198526603 | 2.631925326 | 0.017064746 | 0.077082707 | K09338 |
| *CYC2* | 57.24998996 | 144.6011525 | 2.525784765 | 1.336731705 | 0.017120387 | 0.077280189 | - |
| *IPSP* | 15.15349745 | 32.65432736 | 2.154903676 | 1.107623382 | 0.017151609 | 0.077353786 | K15634 |
| *ORRM6* | 5.355750063 | 23.38338975 | 4.366034538 | 2.126323543 | 0.017162216 | 0.077388162 | - |
| *CLEB3J9* | 4.084731768 | 14.09782841 | 3.45134741 | 1.787159702 | 0.017165798 | 0.077390855 | K00434 |
| *uncharacterized protein_13643* | 6.765189192 | 18.40524785 | 2.720581395 | 1.443914992 | 0.017242706 | 0.07757999 | - |
| *FLZ8* | 259.3652521 | 549.4593254 | 2.118477016 | 1.083027477 | 0.017278891 | 0.077651781 | - |
| *SPCC777.06c* | 9.354226857 | 68.26877116 | 7.298173565 | 2.867535461 | 0.017311719 | 0.077764769 | - |
| *HSP21* | 12.16380773 | 27.69080919 | 2.276491852 | 1.186812296 | 0.01732219 | 0.077784855 | K13993 |
| *L6* | 1.053205759 | 9.888702245 | 9.389145628 | 3.230993885 | 0.017423633 | 0.078159162 | - |
| *Zmynd15* | 7.678423909 | 26.60444499 | 3.464831494 | 1.792785191 | 0.017493967 | 0.078406841 | - |
| *CRK2* | 8.043954662 | 40.03026079 | 4.976440379 | 2.315114159 | 0.017647298 | 0.078930333 | - |
| *uncharacterized protein_29403* | 18.87683106 | 38.10581007 | 2.018655035 | 1.013394392 | 0.017666602 | 0.078975804 | - |
| *uncharacterized protein_49948* | 1.011369467 | 7.755326338 | 7.668143631 | 2.93887736 | 0.017674584 | 0.078997865 | - |
| *MPS1* | 5.748654941 | 17.13580403 | 2.980837118 | 1.575717544 | 0.017873399 | 0.079749007 | K08866 |
| *DHAPS-1* | 12.83340599 | 28.2329233 | 2.199955594 | 1.137474403 | 0.018095245 | 0.080448135 | K01626 |
| *At3g02645* | 8.325812831 | 28.54118515 | 3.428035884 | 1.777382211 | 0.018147795 | 0.080643558 | - |
| *EXPA8* | 0 | 8.724304782 | Inf | Inf | 0.018241072 | 0.080999232 | - |
| *IDD14* | 10.3733435 | 66.73736939 | 6.433544729 | 2.685613847 | 0.018424131 | 0.081672229 | - |
| *v1g171563* | 8.723882493 | 22.58607077 | 2.588993008 | 1.372391069 | 0.018532086 | 0.082052399 | K03248 |
| *EO* | 8.449771498 | 41.61581213 | 4.925081363 | 2.300147558 | 0.018584072 | 0.082186384 | K18980 |
| *DLO1* | 10.0274397 | 24.06784168 | 2.400198097 | 1.263153482 | 0.018636679 | 0.082290838 | - |
| *ERF110* | 9.421887065 | 171.2550837 | 18.17630402 | 4.183986966 | 0.018768644 | 0.082788943 | - |
| *At3g01270* | 15.52419298 | 31.91013954 | 2.055510362 | 1.039496645 | 0.01908857 | 0.083902191 | K01728 |
| *CRRSP55* | 0 | 11.47993906 | Inf | Inf | 0.01914964 | 0.084126036 | - |
| *uncharacterized protein_17627* | 0.986577734 | 7.454935142 | 7.556358598 | 2.917691169 | 0.019161631 | 0.084150198 | - |
| *PIP2-3* | 6.419285389 | 17.92862928 | 2.792932265 | 1.481780585 | 0.019733732 | 0.086195453 | K09872 |
| *At1g73050* | 0 | 5.490959345 | Inf | Inf | 0.019768545 | 0.086303894 | - |
| *At4g34480* | 199.7933201 | 410.6190672 | 2.055219199 | 1.039292272 | 0.020238635 | 0.08800056 | - |
| *uncharacterized protein_21012* | 3.444057837 | 12.55398808 | 3.645115348 | 1.865964469 | 0.020355093 | 0.088403159 | K15032 |
| *MAOM* | 51.15543122 | 108.8511262 | 2.127850819 | 1.089397009 | 0.020722015 | 0.089735357 | K00028 |
| *7OMT* | 0.328859245 | 7.532820044 | 22.90590935 | 4.517647933 | 0.020927571 | 0.090359711 | - |
| *BHLH35* | 0.328859245 | 7.532820044 | 22.90590935 | 4.517647933 | 0.020927571 | 0.090359711 | - |
| *SWEET16* | 11.47355033 | 26.2124644 | 2.284599243 | 1.191941114 | 0.021288442 | 0.091704057 | K15382 |
| *EXPA8* | 20.41022886 | 90.78934639 | 4.44822775 | 2.153230656 | 0.021307349 | 0.091770257 | - |
| *uncharacterized protein_41885* | 7.084751053 | 18.74968359 | 2.646484463 | 1.404077184 | 0.021360289 | 0.091937182 | - |
| *chi1* | 116.8267864 | 277.3535268 | 2.374057658 | 1.247354974 | 0.021807534 | 0.093547009 | K20547 |
| *EXPA15* | 0 | 6.446381663 | Inf | Inf | 0.022292479 | 0.095237998 | - |
| *OPT7* | 0.989160125 | 9.946277937 | 10.05527587 | 3.329880757 | 0.022546579 | 0.095975652 | - |
| *XTH32* | 0.331441636 | 5.563232951 | 16.784955 | 4.069096764 | 0.022809037 | 0.09690197 | K08235 |
| *UGGT* | 0 | 4.334481733 | Inf | Inf | 0.023147003 | 0.098016577 | K11718 |
| *CCR1* | 0.657718489 | 8.703995571 | 13.23361851 | 3.726135691 | 0.023181025 | 0.098067756 | K09753 |
| *BABL* | 3.061482526 | 31.58256267 | 10.31610091 | 3.366825885 | 0.023264589 | 0.098337831 | - |
| *HSR201* | 11.55980531 | 175.7499282 | 15.20353704 | 3.926335095 | 0.023407489 | 0.098861277 | K19861 |
| *TSJT1* | 0.351068586 | 5.446914802 | 15.51524407 | 3.955614487 | 0.023548887 | 0.099264434 | - |
| *SDR2a* | 2471.534417 | 54.65426445 | 0.022113495 | -5.498929119 | 8.0588E-263 | 1.0447E-258 | - |
| *CYP71AU50* | 682.0890481 | 3.493118211 | 0.005121206 | -7.609300822 | 1.3366E-174 | 1.1551E-170 | - |
| *At5g48740* | 813.2098133 | 12.36313697 | 0.015202887 | -6.039510866 | 5.0527E-171 | 3.2752E-167 | - |
| *NRT3.2* | 3551.427806 | 287.5408851 | 0.080964868 | -3.626560148 | 3.4696E-170 | 1.7992E-166 | - |
| *NRT3.1* | 2419.689081 | 171.8395 | 0.071017182 | -3.815688074 | 8.9531E-163 | 3.8689E-159 | - |
| *CAS1* | 12184.08715 | 1543.581842 | 0.126688345 | -2.980644284 | 1.2434E-142 | 4.6056E-139 | K13034 |
| *NRT3.1* | 983.9799897 | 54.3831457 | 0.055268548 | -4.177397468 | 1.993E-136 | 5.7417E-133 | - |
| *NIA* | 4081.817362 | 509.715785 | 0.124874716 | -3.001446695 | 2.9114E-129 | 7.5486E-126 | K10534 |
| *At3g47110* | 1122.797256 | 83.01932138 | 0.073939726 | -3.757506493 | 2.4761E-125 | 5.8363E-122 | - |
| *BAM3* | 862.2769097 | 47.26380644 | 0.054812794 | -4.189343519 | 2.8717E-125 | 6.2047E-122 | K01177 |
| *ALDH2B4* | 483.9005566 | 9.198663999 | 0.01900941 | -5.717142447 | 1.1051E-118 | 2.0467E-115 | K12355 |
| *At5g42610* | 423.3526768 | 6.349280136 | 0.014997614 | -6.059123168 | 1.5709E-112 | 2.5456E-109 | - |
| *NRT2.4* | 2616.799741 | 354.0741659 | 0.135308087 | -2.885680031 | 1.2648E-110 | 1.9291E-107 | K02575 |
| *SPAC24B11.05* | 906.1584051 | 66.13224012 | 0.072980883 | -3.776337593 | 1.9699E-110 | 2.8375E-107 | K07025 |
| *45385* | 2623.469277 | 357.7818955 | 0.136377391 | -2.874323604 | 5.0491E-110 | 6.8902E-107 | K05907 |
| *At5g07050* | 1287.741311 | 132.9893288 | 0.103273326 | -3.275460413 | 4.277E-107 | 5.5447E-104 | - |
| *JAL3* | 1346.201378 | 147.2666624 | 0.109394229 | -3.192391458 | 1.5841E-105 | 1.9559E-102 | - |
| *ABCG36* | 1493.581962 | 177.9401558 | 0.119136519 | -3.069312383 | 2.3501E-103 | 2.76974E-100 | - |
| *CYP720B2* | 671.2292463 | 42.47529893 | 0.063279869 | -3.982109571 | 3.254E-103 | 3.66828E-100 | - |
| *DCOR* | 356.7346603 | 3.803713592 | 0.010662585 | -6.551298965 | 7.6765E-103 | 8.29322E-100 | K01581 |
| *SDR2a* | 410.5807302 | 9.171576724 | 0.022338059 | -5.484352334 | 6.0128E-102 | 6.23602E-99 | - |
| *PR1* | 429.9100133 | 12.73465999 | 0.029621687 | -5.077202368 | 5.7068E-101 | 5.69103E-98 | - |
| *uncharacterized protein_47823* | 1259.961669 | 148.0052631 | 0.117468068 | -3.08965946 | 5.55852E-98 | 5.33783E-95 | - |
| *RL6* | 345.6653843 | 0.860580146 | 0.002489634 | -8.649850864 | 9.61184E-95 | 8.59365E-92 | - |
| *TPS14* | 657.4710955 | 53.68084148 | 0.081647455 | -3.614448279 | 1.16177E-90 | 1.00408E-87 | K04120 |
| *CSLE1* | 3342.812922 | 612.808926 | 0.183321335 | -2.447553401 | 2.97864E-88 | 2.34031E-85 | - |
| *GSTU17* | 2012.150278 | 351.5160389 | 0.174696713 | -2.517075634 | 4.29389E-84 | 3.18091E-81 | K00799 |
| *DCOR* | 481.1622505 | 29.95301599 | 0.062251384 | -4.005750279 | 8.89013E-84 | 6.22982E-81 | K01581 |
| *CYP76T24* | 423.9031153 | 23.1056413 | 0.054506892 | -4.197417532 | 4.59072E-82 | 3.052E-79 | - |
| *CRRSP38* | 1709.551293 | 287.927623 | 0.168422921 | -2.569839601 | 9.78984E-82 | 6.34577E-79 | - |
| *SDR2a* | 265.5640598 | 3.938081598 | 0.014829121 | -6.075423097 | 6.35098E-80 | 4.0163E-77 | - |
| *UFO* | 579.8861319 | 54.4815124 | 0.093952087 | -3.411930978 | 3.17223E-79 | 1.95832E-76 | - |
| *GSTU17* | 1262.285514 | 209.9246065 | 0.16630517 | -2.588095079 | 2.06179E-76 | 1.24321E-73 | K00799 |
| *At4g32940* | 3956.51488 | 856.3443733 | 0.216439063 | -2.207967194 | 2.38502E-76 | 1.40543E-73 | K01369 |
| *AAP3* | 2637.514378 | 540.3544166 | 0.204872596 | -2.287201077 | 5.84492E-76 | 3.36771E-73 | - |
| *ag4* | 724.5493729 | 93.53812767 | 0.129098349 | -2.953457546 | 1.83617E-74 | 1.03496E-71 | K18108 |
| *dnaJ* | 2035.806361 | 417.7902452 | 0.205221014 | -2.284749631 | 2.34157E-72 | 1.26484E-69 | K09510 |
| *nep2* | 1107.197364 | 182.4451545 | 0.164781059 | -2.601377674 | 4.4537E-72 | 2.35664E-69 | - |
| *MAPKKK17* | 225.2349421 | 1.548359038 | 0.006874418 | -7.184546802 | 1.52392E-71 | 7.90246E-69 | - |
| *RFS* | 2746.108778 | 608.108606 | 0.221443743 | -2.174987862 | 1.86331E-69 | 9.29077E-67 | K06617 |
| *SWEET3B* | 736.6003699 | 102.0796988 | 0.138582199 | -2.851186145 | 2.46846E-69 | 1.20759E-66 | K15382 |
| *PAP22* | 349.2501411 | 22.38059593 | 0.064081852 | -3.963940342 | 1.69516E-67 | 8.13929E-65 | - |
| *LECRK41* | 404.4191775 | 33.62787371 | 0.083151036 | -3.588121946 | 3.31914E-67 | 1.5647E-64 | - |
| *FLZ5* | 914.8368329 | 149.9454793 | 0.163904069 | -2.609076425 | 3.74227E-67 | 1.73267E-64 | - |
| *GSTU18* | 773.2125859 | 119.6106135 | 0.15469305 | -2.69251971 | 1.00263E-66 | 4.56072E-64 | K00799 |
| *AIR3* | 1373.901555 | 268.2868458 | 0.195273704 | -2.356430408 | 1.60823E-66 | 7.18933E-64 | - |
| *HB1* | 345.7976043 | 22.49356213 | 0.065048346 | -3.942343821 | 6.8735E-66 | 2.97027E-63 | - |
| *At3g47110* | 320.7188517 | 19.13704631 | 0.059669228 | -4.066869077 | 9.28748E-66 | 3.94763E-63 | - |
| *PLT4* | 10579.83585 | 2748.874704 | 0.259822056 | -1.94440419 | 1.01772E-65 | 4.25604E-63 | - |
| *R53* | 910.5866894 | 154.5859643 | 0.169765236 | -2.558387035 | 1.93118E-65 | 7.82371E-63 | K00430 |
| *CB21* | 167717.9492 | 45792.37945 | 0.273032074 | -1.872857658 | 7.075E-65 | 2.82216E-62 | K08913 |
| *uncharacterized protein_41388* | 1655.876034 | 347.3944505 | 0.209794962 | -2.252948058 | 2.06611E-64 | 8.11669E-62 | - |
| *TUR2* | 3699.946763 | 938.9300296 | 0.253768524 | -1.978414957 | 1.26258E-62 | 4.886E-60 | - |
| *At5g48740* | 1247.705479 | 268.5315498 | 0.215220302 | -2.21611392 | 6.75594E-60 | 2.576E-57 | - |
| *NAC035* | 316.2544274 | 23.61610023 | 0.07467437 | -3.743243022 | 2.31996E-59 | 8.71766E-57 | - |
| *GSTU18* | 656.1869477 | 109.7637707 | 0.167275151 | -2.57970495 | 2.01614E-57 | 7.46778E-55 | K00799 |
| *PLD1* | 612.4893143 | 94.97721836 | 0.155067552 | -2.689031266 | 2.33052E-57 | 8.51067E-55 | K01115 |
| *CEPR1* | 996.0494949 | 185.9428398 | 0.186680321 | -2.421358239 | 4.10403E-57 | 1.47791E-54 | - |
| *nep2* | 441.6769396 | 27.90316126 | 0.063175499 | -3.984491025 | 4.27005E-57 | 1.51663E-54 | - |
| *TAT* | 685.0535109 | 117.7450037 | 0.17187709 | -2.540550842 | 6.91006E-57 | 2.42114E-54 | K15400 |
| *IP5P8* | 340.6417383 | 32.42963589 | 0.095201592 | -3.392870486 | 1.04194E-55 | 3.50851E-53 | - |
| *ag4* | 645.2717313 | 89.16868801 | 0.138187811 | -2.855297729 | 3.50673E-55 | 1.15092E-52 | K18108 |
| *nep2* | 4599.154252 | 1330.923077 | 0.289384309 | -1.788941395 | 2.03415E-53 | 6.59269E-51 | - |
| *MST3* | 478.8834735 | 69.19382769 | 0.144489905 | -2.790959393 | 5.77668E-53 | 1.84911E-50 | - |
| *R53* | 917.4184238 | 200.4107293 | 0.218450735 | -2.194620133 | 7.14777E-53 | 2.26009E-50 | K00430 |
| *DAD2* | 257.5555268 | 18.12514088 | 0.070373721 | -3.828819385 | 2.87789E-52 | 8.9901E-50 | - |
| *uncharacterized protein_14465* | 1043.003413 | 239.3175062 | 0.229450358 | -2.123746039 | 2.8879E-51 | 8.91399E-49 | - |
| *NPF5.7* | 285.9793051 | 25.35305102 | 0.088653446 | -3.495679471 | 3.1191E-51 | 9.51435E-49 | K14638 |
| *OPT4* | 289.6236129 | 25.69641915 | 0.088723495 | -3.494539993 | 4.74665E-51 | 1.43106E-48 | - |
| *At3g47570* | 298.383283 | 28.72193123 | 0.096258513 | -3.376942048 | 8.84624E-51 | 2.63638E-48 | - |
| *ACT* | 1494.339201 | 382.5433984 | 0.255995023 | -1.965812332 | 2.11235E-50 | 6.22374E-48 | K10355 |
| *uncharacterized protein_07790* | 3766.456452 | 1145.354615 | 0.304093418 | -1.717413506 | 3.75309E-49 | 1.09337E-46 | K20283 |
| *At3g47570* | 127.242332 | 0.286860049 | 0.002254439 | -8.793015947 | 5.71292E-49 | 1.64583E-46 | K04730 |
| *DIR* | 497.1974721 | 84.10245702 | 0.169153026 | -2.563599107 | 1.01614E-48 | 2.89523E-46 | - |
| *BHLH113* | 374.516301 | 50.27697912 | 0.134245102 | -2.897058641 | 3.28511E-48 | 9.25829E-46 | - |
| *dri1* | 1220.632563 | 307.0342301 | 0.251536981 | -1.991157573 | 3.4189E-48 | 9.53176E-46 | - |
| *NIA2* | 324.4593781 | 38.54963167 | 0.118811889 | -3.073248885 | 5.33883E-48 | 1.47261E-45 | K10534 |
| *GAD1* | 118.6556206 | 0 | 0 | -Inf | 1.95846E-47 | 5.34515E-45 | K01580 |
| *PR1* | 169.9295491 | 6.283759615 | 0.036978616 | -4.757164944 | 3.8666E-47 | 1.0443E-44 | - |
| *MPT3* | 172.1790283 | 6.938790736 | 0.04029986 | -4.633081362 | 1.44831E-46 | 3.87131E-44 | K15102 |
| *NIR1* | 2621.652791 | 795.0313986 | 0.303255794 | -1.721392885 | 4.5216E-46 | 1.1842E-43 | K00366 |
| *GSTU16* | 580.2473857 | 117.8759221 | 0.2031477 | -2.299399061 | 5.99516E-46 | 1.55443E-43 | K00799 |
| *SULTR3;1* | 1777.846499 | 515.744806 | 0.29009524 | -1.785401474 | 5.17745E-45 | 1.31609E-42 | K17471 |
| *RZFP34* | 1597.770951 | 455.9292958 | 0.285353352 | -1.809178587 | 1.20205E-44 | 2.99681E-42 | K10144 |
| *HSL1* | 1618.003133 | 471.7391744 | 0.291556403 | -1.778153086 | 8.45962E-44 | 2.06925E-41 | K00924 |
| *PT1* | 1153.830889 | 312.0891658 | 0.270480855 | -1.886401611 | 1.26422E-43 | 3.06342E-41 | K08176 |
| *At5g07050* | 178.3259011 | 7.418761249 | 0.041602264 | -4.587194141 | 5.70647E-43 | 1.34507E-40 | - |
| *CRK8* | 784.8818049 | 193.9531742 | 0.247111314 | -2.016767028 | 1.05815E-42 | 2.47169E-40 | - |
| *ASE1* | 1051.561196 | 283.9024964 | 0.269981906 | -1.889065371 | 1.77166E-42 | 4.10139E-40 | K00764 |
| *SLAC1* | 1089.44797 | 300.1515765 | 0.27550795 | -1.859834147 | 1.94742E-42 | 4.46839E-40 | - |
| *At3g47110* | 364.8631714 | 58.14067802 | 0.159349265 | -2.649735734 | 3.11903E-42 | 7.03219E-40 | - |
| *CYP720B2* | 222.6423553 | 20.07667812 | 0.090174568 | -3.47113559 | 3.58422E-42 | 8.01135E-40 | - |
| *MST3* | 958.4062998 | 258.7333209 | 0.269962041 | -1.889171528 | 6.58853E-42 | 1.46006E-39 | - |
| *NPF6.3* | 938.6720068 | 252.1244406 | 0.268596953 | -1.896485158 | 2.88491E-41 | 6.33897E-39 | K14638 |
| *NTF2B* | 710.3957349 | 172.133014 | 0.24230581 | -2.045099096 | 1.13964E-40 | 2.46239E-38 | - |
| *GOLS1* | 833.3744106 | 210.8948479 | 0.253061343 | -1.982440951 | 3.23652E-40 | 6.93524E-38 | K18819 |
| *ABCG39* | 1515.012201 | 467.5919935 | 0.308639094 | -1.69600728 | 6.31827E-40 | 1.33187E-37 | - |
| *At2g19130* | 297.3719135 | 43.30198951 | 0.1456156 | -2.779763177 | 1.37544E-39 | 2.85299E-37 | - |
| *PT30* | 248.6665811 | 30.79539792 | 0.123842125 | -3.013425959 | 2.08162E-39 | 4.2835E-37 | K12742 |
| *SBT5.6* | 436.8702877 | 88.60174598 | 0.20281019 | -2.301797955 | 2.83375E-39 | 5.78531E-37 | - |
| *ag4* | 213.2924093 | 21.9129898 | 0.102736848 | -3.28297438 | 9.99301E-39 | 2.02421E-36 | K04120 |
| *HSP90-1* | 794.5054267 | 211.3624048 | 0.266030159 | -1.910338287 | 1.12438E-38 | 2.25992E-36 | K04079 |
| *TIP4-1* | 284.7671677 | 38.97100808 | 0.136852181 | -2.869309664 | 1.24152E-38 | 2.47616E-36 | K09873 |
| *Gvin1* | 330.2606476 | 55.4380765 | 0.167861587 | -2.574655968 | 1.98146E-38 | 3.89206E-36 | - |
| *XTH9* | 906.4731065 | 250.4125887 | 0.27624933 | -1.855957127 | 2.83959E-38 | 5.5357E-36 | K08235 |
| *WAKL20* | 438.7671557 | 87.27611577 | 0.198912144 | -2.329796735 | 3.09527E-38 | 5.98911E-36 | - |
| *SBT5.1* | 1090.469817 | 322.2647281 | 0.295528334 | -1.75863164 | 4.81192E-38 | 9.24174E-36 | - |
| *RD21B* | 5208.536485 | 1888.237859 | 0.362527528 | -1.463837545 | 4.9851E-38 | 9.50394E-36 | - |
| *TPS14* | 354.2332102 | 22.44043097 | 0.063349314 | -3.980527186 | 6.94006E-38 | 1.31344E-35 | K04120 |
| *DEGP7* | 510.2107732 | 114.1706736 | 0.223771585 | -2.159901241 | 9.85341E-38 | 1.8513E-35 | - |
| *HMA2* | 327.1470686 | 55.48110264 | 0.169590707 | -2.559870978 | 5.72154E-37 | 1.06725E-34 | K01534 |
| *PT30* | 197.8938735 | 20.55434008 | 0.103865469 | -3.267211991 | 6.65726E-37 | 1.22418E-34 | K12742 |
| *XTH9* | 1251.324773 | 390.6464874 | 0.312186329 | -1.679520733 | 1.0232E-36 | 1.86828E-34 | K08235 |
| *CSE* | 362.8909037 | 67.8431228 | 0.186951842 | -2.419261411 | 1.89842E-36 | 3.44211E-34 | - |
| *CHX19* | 262.6220374 | 37.01378537 | 0.140939373 | -2.826853391 | 4.44306E-36 | 7.99998E-34 | - |
| *At1g17710* | 958.955995 | 283.4339711 | 0.295565148 | -1.758451931 | 5.20902E-36 | 9.31444E-34 | K13248 |
| *uncharacterized protein_31448* | 985.6577877 | 303.5125134 | 0.307928895 | -1.699330844 | 1.52215E-35 | 2.70317E-33 | - |
| *LRK10L-1.1* | 604.7005061 | 154.4709113 | 0.255450276 | -1.968885599 | 1.89353E-35 | 3.31727E-33 | - |
| *GLP1* | 457.8817844 | 72.77503498 | 0.15893848 | -2.653459639 | 3.03491E-35 | 5.28116E-33 | - |
| *BAM2* | 745.0819447 | 212.2127073 | 0.284817944 | -1.811888055 | 5.15899E-35 | 8.91748E-33 | - |
| *DAD2* | 278.5861325 | 22.80748448 | 0.0818687 | -3.610544205 | 8.80894E-35 | 1.51257E-32 | - |
| *AAP2* | 162.7282169 | 13.01816809 | 0.079999452 | -3.643866081 | 9.91356E-35 | 1.69105E-32 | - |
| *BGAL3* | 6581.924344 | 2528.40319 | 0.38414346 | -1.380282903 | 2.03555E-34 | 3.44953E-32 | - |
| *At1g80120* | 169.5557532 | 15.41806986 | 0.090932154 | -3.459065662 | 9.47509E-34 | 1.56819E-31 | - |
| *CYP80B2* | 257.8399674 | 39.75246238 | 0.154174943 | -2.697359778 | 9.49575E-34 | 1.56819E-31 | - |
| *NRT3.2* | 163.5655262 | 15.35475949 | 0.09387528 | -3.413110891 | 1.20052E-33 | 1.97006E-31 | - |
| *GSVIVT00023967001* | 81.00289321 | 0 | 0 | -Inf | 1.21394E-33 | 1.97957E-31 | K00430 |
| *ag4* | 372.6394374 | 40.9630396 | 0.109926743 | -3.185385689 | 2.0551E-33 | 3.3096E-31 | - |
| *CSLE6* | 1897.928162 | 679.2289455 | 0.357879165 | -1.48245554 | 2.54931E-33 | 4.08016E-31 | - |
| *SUS4* | 3321.935915 | 1253.540353 | 0.377352359 | -1.406015803 | 2.76949E-33 | 4.40536E-31 | K00695 |
| *At1g67720* | 104.0735867 | 2.921657468 | 0.028072997 | -5.154673108 | 8.18235E-33 | 1.29361E-30 | - |
| *DAD2* | 203.273231 | 17.10422302 | 0.084144001 | -3.570995764 | 1.45396E-32 | 2.27533E-30 | - |
| *XA21* | 361.1394719 | 76.94009238 | 0.213048139 | -2.230748646 | 1.45674E-32 | 2.27533E-30 | - |
| *Tmem45b* | 787.6843704 | 239.4496898 | 0.303991927 | -1.717895083 | 1.81646E-32 | 2.82019E-30 | - |
| *At5g18840* | 207.4686474 | 27.78229942 | 0.133910833 | -2.900655419 | 2.79213E-32 | 4.30919E-30 | K08145 |
| *At3g47570* | 114.4973975 | 5.045995958 | 0.044070835 | -4.504031946 | 6.76514E-32 | 1.02577E-29 | - |
| *CRK26* | 525.4157702 | 141.8456189 | 0.269968332 | -1.889137912 | 1.20166E-31 | 1.79062E-29 | - |
| *CYP735A2* | 133.9963078 | 9.880757415 | 0.073739027 | -3.761427801 | 1.26815E-31 | 1.87889E-29 | K10717 |
| *UGT86A1* | 293.2899163 | 57.6233676 | 0.196472379 | -2.347601589 | 1.33256E-31 | 1.96311E-29 | - |
| *Spast* | 1357.652781 | 483.1974649 | 0.355906511 | -1.490429767 | 2.72729E-31 | 3.97266E-29 | - |
| *ag4* | 186.7099285 | 24.49130411 | 0.131173014 | -2.930457145 | 1.02857E-30 | 1.4816E-28 | - |
| *PNC1* | 86.22037072 | 1.184706675 | 0.01374045 | -6.185426959 | 1.10265E-30 | 1.57953E-28 | K00430 |
| *OPR11* | 502.9569746 | 137.5766327 | 0.27353559 | -1.87019954 | 1.86606E-30 | 2.65841E-28 | K05894 |
| *UGT85A23* | 184.257099 | 23.79451278 | 0.129137563 | -2.953019385 | 2.08892E-30 | 2.95964E-28 | - |
| *CSLE1* | 423.8029085 | 105.8439365 | 0.249748018 | -2.001454865 | 4.48517E-30 | 6.26703E-28 | - |
| *HIPP23* | 89.43525427 | 1.912011402 | 0.021378722 | -5.547680605 | 4.49578E-30 | 6.26703E-28 | - |
| *CYP75B137* | 644.4524159 | 198.0380132 | 0.307296564 | -1.702296458 | 4.53862E-30 | 6.29291E-28 | K05280 |
| *At1g21890* | 1304.191572 | 464.4471338 | 0.356118797 | -1.489569507 | 4.96181E-30 | 6.84308E-28 | - |
| *PT1* | 645.0138503 | 170.4627755 | 0.264277698 | -1.919873412 | 6.87502E-30 | 9.43151E-28 | K08176 |
| *Os07g0190000* | 801.5893593 | 256.1689571 | 0.319576294 | -1.645767699 | 8.32618E-30 | 1.13622E-27 | K01662 |
| *FLA17* | 2562.073189 | 995.6148766 | 0.38859736 | -1.363651996 | 1.16327E-29 | 1.5709E-27 | - |
| *PT1* | 614.787493 | 184.6135375 | 0.300288375 | -1.735579468 | 2.02427E-29 | 2.71945E-27 | K12742 |
| *CCR3* | 171.1433851 | 20.55667361 | 0.120113749 | -3.057526793 | 2.08314E-29 | 2.78411E-27 | - |
| *At5g03795* | 113.8577558 | 6.736642854 | 0.059167185 | -4.079058942 | 3.63503E-29 | 4.80862E-27 | - |
| *TIP1-1* | 1404.804953 | 515.9223265 | 0.367255486 | -1.445144051 | 4.37522E-29 | 5.75841E-27 | K09873 |
| *CRK2* | 594.7527586 | 180.3861874 | 0.303296092 | -1.721201186 | 4.75878E-29 | 6.2316E-27 | - |
| *RK8* | 237.2367832 | 41.5131485 | 0.174986138 | -2.514687454 | 5.58922E-29 | 7.28228E-27 | - |
| *YSL3* | 410.1334468 | 106.4807936 | 0.25962475 | -1.945500173 | 8.05741E-29 | 1.04456E-26 | - |
| *INV1* | 151.2329752 | 15.22498437 | 0.100672385 | -3.31226009 | 9.31529E-29 | 1.20163E-26 | K01193 |
| *NRT3.1* | 276.2225756 | 21.13819018 | 0.076525933 | -3.707907469 | 9.92936E-29 | 1.2745E-26 | - |
| *ag4* | 568.3644868 | 163.8613707 | 0.288303324 | -1.794340625 | 1.12604E-28 | 1.43823E-26 | K18108 |
| *HMG1* | 107.0857033 | 6.117736425 | 0.057129348 | -4.129624122 | 1.19995E-28 | 1.52512E-26 | K00021 |
| *NDA2* | 2260.972103 | 768.1107023 | 0.339725865 | -1.557557035 | 1.22321E-28 | 1.54709E-26 | K17871 |
| *RANBP1C* | 616.8312608 | 189.9113614 | 0.307882193 | -1.699549666 | 1.51579E-28 | 1.89905E-26 | K15306 |
| *UGT74AC1* | 683.5762633 | 221.8890091 | 0.324600225 | -1.623264094 | 1.51614E-28 | 1.89905E-26 | K13691 |
| *DRP4C* | 957.6800334 | 337.8328316 | 0.352761695 | -1.503234183 | 2.88091E-28 | 3.59116E-26 | K14754 |
| *BAM3* | 70.90174462 | 0.286860049 | 0.004045881 | -7.949330261 | 3.32344E-28 | 4.12298E-26 | K01177 |
| *CYP76T24* | 70.83150202 | 0.324126529 | 0.004576022 | -7.77169022 | 3.36322E-28 | 4.15245E-26 | - |
| *Sfswap* | 1367.875693 | 518.276668 | 0.378891643 | -1.400142777 | 7.68549E-28 | 9.44404E-26 | - |
| *tmem214-a* | 1702.994915 | 670.8498988 | 0.393923606 | -1.34401222 | 7.97482E-28 | 9.75336E-26 | - |
| *FLZ2* | 536.2307181 | 163.6049498 | 0.305101786 | -1.712637469 | 1.05846E-27 | 1.28242E-25 | - |
| *ABCG39* | 403.4584276 | 106.1635193 | 0.263133726 | -1.926131924 | 1.22826E-27 | 1.48123E-25 | - |
| *DIT1* | 7210.446273 | 3138.277629 | 0.435240415 | -1.200115568 | 2.34026E-27 | 2.80917E-25 | - |
| *SPAC24B11.05* | 448.9891625 | 126.2017884 | 0.28107981 | -1.830948267 | 4.06069E-27 | 4.82961E-25 | K07025 |
| *PR1* | 147.2666714 | 16.9607684 | 0.115170447 | -3.118157528 | 6.8701E-27 | 8.09672E-25 | - |
| *ACR3* | 434.9889217 | 121.5511008 | 0.279434934 | -1.839415701 | 8.36565E-27 | 9.81468E-25 | - |
| *IRK* | 566.8724688 | 176.5668816 | 0.311475493 | -1.68280944 | 9.70464E-27 | 1.12835E-24 | - |
| *uncharacterized protein_09320* | 166.435542 | 17.48483266 | 0.10505468 | -3.250787657 | 1.22346E-26 | 1.41615E-24 | - |
| *NFP* | 414.9628183 | 119.8681269 | 0.288864741 | -1.791533978 | 3.26886E-26 | 3.7337E-24 | - |
| *uncharacterized protein_42501* | 440.3238643 | 125.9172377 | 0.285965054 | -1.80608924 | 3.77788E-26 | 4.27742E-24 | - |
| *CRK3* | 218.7342622 | 29.42666739 | 0.134531587 | -2.89398315 | 4.28902E-26 | 4.83504E-24 | - |
| *At1g32780* | 177.7520767 | 27.71228517 | 0.155904143 | -2.681268829 | 5.25421E-26 | 5.89745E-24 | K00001 |
| *ZIP11* | 1030.429721 | 388.3006376 | 0.376833693 | -1.40800013 | 6.0358E-26 | 6.74552E-24 | K14709 |
| *CYP720B2* | 638.3824178 | 213.9870487 | 0.335201977 | -1.576897435 | 6.16405E-26 | 6.85929E-24 | - |
| *DLO1* | 286.809316 | 66.88653372 | 0.23320907 | -2.100304198 | 1.55346E-25 | 1.7067E-23 | - |
| *APS2* | 2530.1813 | 1086.293544 | 0.429334271 | -1.219826756 | 2.81055E-25 | 3.07477E-23 | K13811 |
| *HSP7M* | 553.8295155 | 182.3230267 | 0.329204244 | -1.602945162 | 3.69931E-25 | 4.03008E-23 | - |
| *GGPS1* | 823.6218926 | 300.3438427 | 0.364662287 | -1.455367091 | 6.05902E-25 | 6.57315E-23 | K13789 |
| *uncharacterized protein_13825* | 58.40752744 | 0 | 0 | -Inf | 8.39133E-25 | 9.02782E-23 | - |
| *SUS4* | 1652.536678 | 680.0592597 | 0.411524457 | -1.280949921 | 1.03239E-24 | 1.10611E-22 | K00695 |
| *PBL34* | 638.562906 | 222.0788667 | 0.347779153 | -1.523756638 | 1.22633E-24 | 1.30849E-22 | - |
| *CRK8* | 114.2985456 | 10.79556131 | 0.094450557 | -3.404296884 | 1.38505E-24 | 1.46578E-22 | - |
| *SCRM* | 529.2181258 | 173.8315073 | 0.328468544 | -1.606172878 | 3.58249E-24 | 3.71547E-22 | - |
| *FLS2* | 136.6597206 | 16.97998502 | 0.124250108 | -3.008680991 | 4.31334E-24 | 4.45562E-22 | K13420 |
| *H1* | 914.0519976 | 350.2378536 | 0.383170601 | -1.38394122 | 5.3138E-24 | 5.40298E-22 | K11275 |
| *BCP* | 288.5397189 | 69.48528062 | 0.240817039 | -2.053990624 | 6.57541E-24 | 6.63375E-22 | - |
| *CAB6A* | 338.4053888 | 92.3570689 | 0.272918434 | -1.873458249 | 9.30284E-24 | 9.3129E-22 | K08907 |
| *MVD2* | 719.5491937 | 264.5835358 | 0.367707362 | -1.443370034 | 1.04184E-23 | 1.03497E-21 | K01597 |
| *GSTU18* | 119.4726367 | 12.68274479 | 0.106156063 | -3.235741318 | 1.11629E-23 | 1.1047E-21 | K00799 |
| *AGAL2* | 710.8465635 | 257.6562159 | 0.362463897 | -1.46409079 | 1.23097E-23 | 1.21356E-21 | K07407 |
| *CYCU4-1* | 774.4315 | 288.2697751 | 0.372234052 | -1.425718056 | 1.29834E-23 | 1.27513E-21 | - |
| *At4g17550* | 324.6547637 | 89.18797882 | 0.274716372 | -1.863985203 | 1.46544E-23 | 1.43381E-21 | K13783 |
| *At3g59480* | 683.8778138 | 248.537647 | 0.363424053 | -1.460274188 | 1.7015E-23 | 1.65851E-21 | K00847 |
| *UGT74D1* | 877.2032972 | 293.8304286 | 0.33496275 | -1.577927428 | 2.05195E-23 | 1.99262E-21 | K13691 |
| *SPAC24B11.05* | 517.9272667 | 173.1245118 | 0.334264139 | -1.580939509 | 2.22164E-23 | 2.14936E-21 | K07025 |
| *CRK2* | 63.58973537 | 0.935113106 | 0.01470541 | -6.087509219 | 2.51698E-23 | 2.42603E-21 | - |
| *NPF4.3* | 194.6793597 | 37.96126285 | 0.194993773 | -2.358500042 | 2.68123E-23 | 2.57478E-21 | - |
| *SRG1* | 516.1901488 | 178.8410327 | 0.346463475 | -1.529224827 | 3.49332E-23 | 3.34225E-21 | - |
| *PDLP8* | 113.0616165 | 10.85095183 | 0.09597379 | -3.381215725 | 3.63885E-23 | 3.46868E-21 | - |
| *H1* | 1201.825757 | 483.1658105 | 0.402026506 | -1.31463747 | 3.67786E-23 | 3.49302E-21 | K11275 |
| *NRT2.1* | 151.8324791 | 23.40829185 | 0.154171835 | -2.69738887 | 4.35089E-23 | 4.11715E-21 | K02575 |
| *ABCC3* | 933.395504 | 364.7344634 | 0.390760896 | -1.355641993 | 4.43508E-23 | 4.18155E-21 | - |
| *PFP-ALPHA* | 799.1937776 | 301.8669537 | 0.377714344 | -1.404632521 | 4.4738E-23 | 4.20277E-21 | K00895 |
| *MOT2* | 137.6986125 | 19.21495619 | 0.139543572 | -2.84121243 | 4.60373E-23 | 4.30923E-21 | - |
| *TAT* | 210.5619987 | 27.0279816 | 0.128361156 | -2.961719406 | 4.72738E-23 | 4.40905E-21 | K15400 |
| *CBG* | 1491.066042 | 641.0279473 | 0.429912512 | -1.217884997 | 8.91865E-23 | 8.22928E-21 | K01188 |
| *DIVARICATA* | 1168.301 | 479.2065723 | 0.410173896 | -1.285692416 | 9.88152E-23 | 9.08539E-21 | - |
| *At3g02645* | 148.9581863 | 22.6347082 | 0.151953436 | -2.718298799 | 1.00213E-22 | 9.18138E-21 | - |
| *UBC8* | 5308.509048 | 2457.775356 | 0.462987881 | -1.110953664 | 1.03755E-22 | 9.47241E-21 | K06689 |
| *ag1* | 88.19301203 | 5.999158921 | 0.068023064 | -3.877832194 | 1.05024E-22 | 9.55462E-21 | K16086 |
| *OPT4* | 220.318942 | 17.93654913 | 0.081411743 | -3.618619279 | 1.09842E-22 | 9.95795E-21 | - |
| *ERF053* | 420.1911434 | 131.4160919 | 0.312753122 | -1.67690381 | 1.17704E-22 | 1.06335E-20 | - |
| *CESA4* | 2402.256267 | 1066.364686 | 0.443901302 | -1.171689154 | 1.23615E-22 | 1.11288E-20 | K10999 |
| *ATHB-5* | 668.2644137 | 247.0446035 | 0.36968092 | -1.435647509 | 1.25404E-22 | 1.12507E-20 | K09338 |
| *LECRK41* | 155.8752225 | 26.48914525 | 0.169938139 | -2.556918423 | 1.31265E-22 | 1.16957E-20 | - |
| *SYNC1* | 1871.857022 | 814.3325796 | 0.435039947 | -1.200780214 | 1.32353E-22 | 1.17522E-20 | K01893 |
| *At1g11050* | 269.9492406 | 68.47327604 | 0.253652412 | -1.979075215 | 1.47005E-22 | 1.29979E-20 | - |
| *PHOS34* | 590.8580955 | 212.2805372 | 0.359274991 | -1.47683958 | 1.49672E-22 | 1.31549E-20 | - |
| *ABCC10* | 309.4783906 | 85.73555321 | 0.277032438 | -1.85187318 | 1.6458E-22 | 1.44163E-20 | - |
| *GT5* | 157.890584 | 26.89349021 | 0.170329918 | -2.553596233 | 1.82502E-22 | 1.58789E-20 | K13691 |
| *Tmem45b* | 55.81229281 | 0.324126529 | 0.00580744 | -7.427881998 | 2.27155E-22 | 1.96979E-20 | - |
| *NPF7.3* | 1637.576346 | 696.9808959 | 0.425617345 | -1.232371151 | 2.35218E-22 | 2.03291E-20 | K14638 |
| *TBR* | 198.836333 | 28.93768444 | 0.145535195 | -2.780560013 | 2.64029E-22 | 2.27434E-20 | - |
| *uncharacterized protein_49315* | 890.2904555 | 356.6174496 | 0.400563038 | -1.319898792 | 2.68299E-22 | 2.30347E-20 | - |
| *RPV1* | 462.4909825 | 157.6825083 | 0.340941801 | -1.552402606 | 3.08018E-22 | 2.63574E-20 | - |
| *KIN14F* | 693.5205445 | 263.2542577 | 0.379591145 | -1.397481757 | 3.74222E-22 | 3.18125E-20 | K10406 |
| *CRK8* | 678.7292637 | 259.4831442 | 0.382307288 | -1.387195391 | 3.8605E-22 | 3.26042E-20 | - |
| *TUBB6* | 1396.495654 | 604.6444885 | 0.432972696 | -1.207652045 | 4.86593E-22 | 4.09623E-20 | K07375 |
| *A6* | 676.8470793 | 252.309828 | 0.372772279 | -1.423633518 | 4.90978E-22 | 4.11976E-20 | - |
| *PCBER* | 990.847895 | 373.771847 | 0.377224243 | -1.406505699 | 5.05991E-22 | 4.23205E-20 | - |
| *PT1* | 411.9946678 | 132.6413928 | 0.321949295 | -1.635094605 | 7.24006E-22 | 5.99745E-20 | K12742 |
| *UGT85A1* | 878.1147641 | 300.8888122 | 0.342653175 | -1.545179039 | 7.78207E-22 | 6.42591E-20 | - |
| *HIPP23* | 73.23629824 | 3.324835666 | 0.04539874 | -4.461203931 | 8.64138E-22 | 7.11281E-20 | - |
| *EO* | 548.5423095 | 185.5178163 | 0.338201471 | -1.564045158 | 1.13845E-21 | 9.31161E-20 | K18980 |
| *BAM1* | 825.3796042 | 325.9679617 | 0.394930963 | -1.340327615 | 1.38557E-21 | 1.12972E-19 | - |
| *HSP23* | 854.5972399 | 344.5830313 | 0.403211028 | -1.310392998 | 1.50046E-21 | 1.21956E-19 | K13993 |
| *PXC3* | 75.64094618 | 3.749489843 | 0.049569579 | -4.33440119 | 1.53036E-21 | 1.23997E-19 | - |
| *uncharacterized protein_14429* | 379.8154522 | 111.9989636 | 0.294877323 | -1.761813218 | 1.95138E-21 | 1.57128E-19 | - |
| *IQD18* | 1069.764296 | 445.3856381 | 0.416339973 | -1.264166016 | 2.09228E-21 | 1.67953E-19 | - |
| *ABCC10* | 366.0662329 | 114.6629351 | 0.313230024 | -1.674705587 | 2.14357E-21 | 1.71011E-19 | - |
| *ITPK3* | 1900.7486 | 855.1733938 | 0.449913994 | -1.152278855 | 2.34042E-21 | 1.85695E-19 | K00913 |
| *MST4* | 2933.714448 | 1203.961488 | 0.410388096 | -1.284939209 | 2.34195E-21 | 1.85695E-19 | - |
| *PTEN1* | 358.1474213 | 113.3144638 | 0.316390562 | -1.660221532 | 2.52358E-21 | 1.99486E-19 | K01110 |
| *PM19L* | 205.860056 | 46.13223093 | 0.224095105 | -2.157816957 | 2.92382E-21 | 2.30422E-19 | - |
| *NPF5.1* | 80.52181727 | 5.008729478 | 0.062203383 | -4.006863134 | 3.81771E-21 | 2.98149E-19 | - |
| *At1g32780* | 152.7114765 | 26.93641719 | 0.176387642 | -2.503178611 | 3.92879E-21 | 3.05903E-19 | K00001 |
| *At1g27200* | 421.7068304 | 141.0155763 | 0.334392441 | -1.58038586 | 4.02641E-21 | 3.12565E-19 | - |
| *PAD4* | 284.922115 | 80.87134672 | 0.283836678 | -1.816867063 | 4.6357E-21 | 3.57722E-19 | - |
| *PT1* | 319.7559642 | 94.73206932 | 0.296263651 | -1.755046465 | 4.9449E-21 | 3.8045E-19 | K08176 |
| *BEBT1* | 79.93161071 | 4.817878367 | 0.060275007 | -4.052296285 | 5.45735E-21 | 4.18634E-19 | K19861 |
| *At2g26730* | 137.1692029 | 21.70860754 | 0.158261527 | -2.65961751 | 5.74303E-21 | 4.39248E-19 | - |
| *RNR1* | 307.804134 | 76.51322805 | 0.248577649 | -2.008231512 | 6.55227E-21 | 4.99669E-19 | K10807 |
| *MLP43* | 1128.382107 | 490.0287516 | 0.434275543 | -1.203317389 | 6.70622E-21 | 5.08418E-19 | - |
| *CPK17* | 408.6626927 | 133.3349306 | 0.326271355 | -1.615855763 | 7.42605E-21 | 5.61348E-19 | K13412 |
| *CYP76T24* | 61.2706761 | 1.778735983 | 0.029030788 | -5.106272478 | 7.55653E-21 | 5.69552E-19 | - |
| *R53* | 276.9899427 | 77.76223928 | 0.280740299 | -1.832691925 | 7.69352E-21 | 5.78196E-19 | K00430 |
| *APX2* | 547.7309763 | 198.1332505 | 0.361734609 | -1.466996461 | 7.87292E-21 | 5.89968E-19 | K00434 |
| *SDR2a* | 59.20610087 | 1.147440194 | 0.019380438 | -5.68925498 | 8.00153E-21 | 5.97878E-19 | - |
| *LRK10* | 213.9400987 | 51.89979694 | 0.24259032 | -2.043406112 | 8.72604E-21 | 6.5014E-19 | - |
| *LAC3* | 48.2442493 | 0 | 0 | -Inf | 9.20649E-21 | 6.839E-19 | K05909 |
| *NPF8.1* | 526.2539673 | 164.8948512 | 0.313337023 | -1.674212852 | 9.23192E-21 | 6.839E-19 | K14638 |
| *BAM3* | 4604.177103 | 2231.62122 | 0.484694913 | -1.044851154 | 1.11836E-20 | 8.26119E-19 | K01177 |
| *PAO2* | 1141.203354 | 492.541992 | 0.431598795 | -1.212237259 | 1.36427E-20 | 1.00491E-18 | K17839 |
| *CYP75A5* | 1006.880465 | 429.189145 | 0.426256303 | -1.230206926 | 1.49617E-20 | 1.09584E-18 | K13083 |
| *LOX1.5* | 125.7216286 | 18.004329 | 0.143207889 | -2.803817128 | 1.88657E-20 | 1.37789E-18 | K15718 |
| *SPDS2* | 3032.958359 | 1442.389705 | 0.475571879 | -1.072264685 | 1.94778E-20 | 1.41843E-18 | K00797 |
| *CYP90B2* | 113.5826126 | 14.37567602 | 0.126565816 | -2.982040296 | 1.95849E-20 | 1.41843E-18 | K09587 |
| *MYOB2* | 924.2761477 | 389.8376756 | 0.421776194 | -1.245450427 | 2.35187E-20 | 1.69859E-18 | - |
| *NAC022* | 100.2060011 | 10.27723173 | 0.10256104 | -3.285445293 | 2.51365E-20 | 1.81038E-18 | - |
| *LECRK42* | 214.7558649 | 45.499645 | 0.211866833 | -2.238770338 | 2.58838E-20 | 1.85904E-18 | - |
| *Os05g0235800* | 323.2064404 | 100.1439278 | 0.309845087 | -1.690381 | 2.6134E-20 | 1.87183E-18 | K02542 |
| *CYP707A1* | 715.2040775 | 290.7439139 | 0.406518815 | -1.298605969 | 2.70633E-20 | 1.93305E-18 | K09843 |
| *SCL6* | 1985.728423 | 912.8107112 | 0.459685575 | -1.121280701 | 3.21796E-20 | 2.28589E-18 | - |
| *ROQ1* | 220.5744621 | 55.50138763 | 0.251622002 | -1.99067002 | 3.95086E-20 | 2.79122E-18 | - |
| *FRI3* | 1522.423802 | 695.7512318 | 0.457002335 | -1.12972656 | 4.22327E-20 | 2.97557E-18 | K00522 |
| *DAD2* | 101.4983449 | 11.4675497 | 0.112982628 | -3.145827136 | 4.53776E-20 | 3.17987E-18 | - |
| *At1g06840* | 343.9782812 | 110.4030597 | 0.320959391 | -1.63953732 | 4.66298E-20 | 3.25881E-18 | - |
| *CYP750A1* | 129.157273 | 21.01740252 | 0.162727209 | -2.619472598 | 4.96799E-20 | 3.46263E-18 | - |
| *uncharacterized protein_03647* | 670.5934406 | 221.0616959 | 0.329650847 | -1.600989305 | 5.47115E-20 | 3.80311E-18 | - |
| *SDR2a* | 61.29871653 | 1.662417833 | 0.027119945 | -5.204501926 | 5.51577E-20 | 3.82388E-18 | - |
| *SPBC2A9.03* | 2386.909687 | 1128.876567 | 0.472944818 | -1.080256233 | 6.0628E-20 | 4.18075E-18 | - |
| *OsI_15603* | 60.84538059 | 2.026070197 | 0.033298669 | -4.908391672 | 6.33645E-20 | 4.35786E-18 | K03921 |
| *FDH1* | 1848.301561 | 867.761897 | 0.469491513 | -1.090829015 | 7.97179E-20 | 5.45363E-18 | K00122 |
| *CYP71AU50* | 1341.959409 | 595.5751974 | 0.443810143 | -1.171985455 | 8.00682E-20 | 5.46318E-18 | - |
| *YUC9* | 102.707534 | 12.61382313 | 0.122813027 | -3.025464499 | 8.11695E-20 | 5.52378E-18 | K11816 |
| *At1g67720* | 80.22446475 | 6.367329993 | 0.079368931 | -3.655281819 | 8.29989E-20 | 5.61879E-18 | - |
| *FRI3* | 76.35304704 | 4.776093178 | 0.062552752 | -3.998782844 | 9.25405E-20 | 6.24822E-18 | K00522 |
| *4CL* | 4250.126578 | 2063.869776 | 0.485601955 | -1.042153865 | 9.561E-20 | 6.42222E-18 | K01904 |
| *FAR4* | 146.4381415 | 24.22708681 | 0.165442463 | -2.595598526 | 1.07417E-19 | 7.17809E-18 | K13356 |
| *GSTU17* | 177.9116708 | 39.09400513 | 0.219738283 | -2.186141853 | 1.09451E-19 | 7.29522E-18 | K00799 |
| *At5g48740* | 61.74916965 | 2.231619221 | 0.036140069 | -4.790256949 | 1.12081E-19 | 7.43233E-18 | - |
| *HSL1* | 1890.506883 | 880.2668712 | 0.46562479 | -1.102760224 | 1.17562E-19 | 7.7759E-18 | - |
| *BRL2* | 256.4949398 | 72.11769531 | 0.281166152 | -1.830505166 | 1.18009E-19 | 7.78556E-18 | - |
| *ACT7* | 120.0110471 | 17.88910344 | 0.149062139 | -2.746014223 | 1.19493E-19 | 7.86348E-18 | K10355 |
| *AGPS1* | 3628.216559 | 1773.755521 | 0.488878073 | -1.032453394 | 1.36386E-19 | 8.92984E-18 | K00975 |
| *ALA1* | 545.0972234 | 210.427194 | 0.386036078 | -1.373192412 | 1.50501E-19 | 9.82922E-18 | K14802 |
| *uncharacterized protein_08602* | 499.5616163 | 170.4311695 | 0.341161458 | -1.551473424 | 1.70472E-19 | 1.10777E-17 | - |
| *XTH32* | 398.0914641 | 139.3372446 | 0.350013143 | -1.514518998 | 1.71642E-19 | 1.11258E-17 | K08235 |
| *UGT85K4* | 60.78753193 | 0 | 0 | -Inf | 1.99078E-19 | 1.2872E-17 | - |
| *CIPK26* | 198.971871 | 48.06907025 | 0.241587266 | -2.049383684 | 2.50386E-19 | 1.61493E-17 | K07198 |
| *PHT1-4* | 179.3467856 | 41.4261028 | 0.230983247 | -2.114139879 | 2.76633E-19 | 1.77978E-17 | K08176 |
| *GGP3* | 129.5238359 | 22.43926421 | 0.173244284 | -2.529120342 | 2.7828E-19 | 1.78595E-17 | - |
| *SERK2* | 773.0882537 | 297.3972339 | 0.384687301 | -1.378241889 | 3.24348E-19 | 2.07647E-17 | K13418 |
| *HAK17* | 161.1266408 | 33.54884702 | 0.208214153 | -2.263859959 | 3.47108E-19 | 2.21671E-17 | K03549 |
| *UGT85A23* | 833.9035988 | 353.4373837 | 0.423834822 | -1.23842597 | 3.53089E-19 | 2.24936E-17 | - |
| *H1* | 1945.386536 | 476.360417 | 0.244866718 | -2.029931395 | 3.81967E-19 | 2.42736E-17 | K11275 |
| *uncharacterized protein_48857* | 492.9741019 | 186.08503 | 0.377474251 | -1.405549858 | 5.38788E-19 | 3.41557E-17 | - |
| *ALMT9* | 125.6956564 | 14.48973482 | 0.115276337 | -3.1168317 | 5.50533E-19 | 3.48152E-17 | - |
| *MIOX1* | 61.58920578 | 2.595271585 | 0.042138416 | -4.5687201 | 5.55999E-19 | 3.49228E-17 | K00469 |
| *SCPL31* | 43.788822 | 0 | 0 | -Inf | 5.56276E-19 | 3.49228E-17 | K16297 |
| *AVT1B* | 440.3603837 | 140.267889 | 0.318529764 | -1.650499909 | 5.60869E-19 | 3.51261E-17 | K15015 |
| *PT1* | 132.0655066 | 22.95883473 | 0.173844294 | -2.524132381 | 9.12874E-19 | 5.63548E-17 | K08176 |
| *At2g39510* | 272.4471589 | 80.42424736 | 0.295192094 | -1.760274012 | 9.26436E-19 | 5.70561E-17 | - |
| *LOX1.5* | 110.8114017 | 15.98161075 | 0.144223523 | -2.793621606 | 9.80128E-19 | 6.02198E-17 | K15718 |
| *uncharacterized protein_33452* | 344.8722722 | 83.02167914 | 0.240731673 | -2.054502124 | 9.86365E-19 | 6.04288E-17 | - |
| *tmem53* | 197.2437539 | 49.61174381 | 0.251525044 | -1.991226042 | 9.88191E-19 | 6.04288E-17 | - |
| *CBP1* | 141.2317292 | 21.95594175 | 0.155460405 | -2.685380919 | 9.96678E-19 | 6.08044E-17 | K16296 |
| *UGT73C13* | 121.9310007 | 17.18444146 | 0.140935786 | -2.826890108 | 1.00991E-18 | 6.1467E-17 | K13496 |
| *NFD4* | 251.3203307 | 74.52201663 | 0.296522038 | -1.753788763 | 1.1102E-18 | 6.72553E-17 | - |
| *PHOS34* | 583.8973685 | 205.795944 | 0.352452255 | -1.50450026 | 1.28175E-18 | 7.74669E-17 | - |
| *BT4* | 272.2462427 | 82.95504181 | 0.30470592 | -1.714510566 | 1.31619E-18 | 7.93632E-17 | - |
| *SRG1* | 850.713642 | 374.1664869 | 0.439826598 | -1.184993243 | 1.58117E-18 | 9.51195E-17 | - |
| *CYP76T24* | 78.12100151 | 3.079760807 | 0.039422956 | -4.664820233 | 1.69377E-18 | 1.01657E-16 | - |
| *SULTR3;1* | 164.4938893 | 36.55633422 | 0.222235211 | -2.169840679 | 2.11442E-18 | 1.26319E-16 | K17471 |
| *Os07g0190000* | 535.0291279 | 210.930999 | 0.394242085 | -1.342846303 | 2.14788E-18 | 1.28023E-16 | K01662 |
| *NIP6-1* | 167.8554589 | 38.32829214 | 0.228341052 | -2.130737837 | 3.00579E-18 | 1.76721E-16 | K09874 |
| *At5g15710* | 134.7676554 | 24.74549057 | 0.183615946 | -2.445236739 | 3.21258E-18 | 1.88027E-16 | - |
| *UGT86A1* | 335.931748 | 116.3569581 | 0.346370829 | -1.529610661 | 3.39202E-18 | 1.97637E-16 | - |
| *CYP76B6* | 105.2663148 | 12.52124031 | 0.118948216 | -3.071594465 | 4.18583E-18 | 2.42797E-16 | - |
| *PCAP1* | 171.0717405 | 38.18496089 | 0.22321022 | -2.163525008 | 4.40383E-18 | 2.54872E-16 | - |
| *At1g32860* | 417.128259 | 154.789229 | 0.371083056 | -1.430185968 | 4.49283E-18 | 2.59443E-16 | - |
| *At3g47110* | 88.24621007 | 10.03550881 | 0.113721698 | -3.136420555 | 5.38384E-18 | 3.10205E-16 | - |
| *GSVIVT00037159001* | 190.783002 | 34.36426505 | 0.180122258 | -2.472951628 | 5.98533E-18 | 3.44097E-16 | K00430 |
| *ag4* | 95.37264914 | 10.10100435 | 0.105910913 | -3.239076842 | 6.08388E-18 | 3.48988E-16 | K18108 |
| *At1g06840* | 214.4654412 | 59.65174636 | 0.278141532 | -1.846108912 | 6.10168E-18 | 3.49237E-16 | - |
| *AAE3* | 532.1582359 | 204.202301 | 0.383724778 | -1.381856168 | 8.97638E-18 | 5.12642E-16 | - |
| *SMAX1* | 747.0466906 | 320.3132635 | 0.428772749 | -1.221714876 | 9.20348E-18 | 5.23306E-16 | - |
| *uncharacterized protein_50942* | 92.36178226 | 11.12537331 | 0.120454294 | -3.053442265 | 1.09245E-17 | 6.19802E-16 | - |
| *uncharacterized protein_08488* | 102.6507176 | 14.44343092 | 0.140704627 | -2.829258324 | 1.11034E-17 | 6.28576E-16 | - |
| *RAV2* | 210.6512058 | 58.53710389 | 0.277886394 | -1.847432899 | 1.38136E-17 | 7.80303E-16 | K09287 |
| *YUC9* | 920.5045498 | 376.2192186 | 0.408709787 | -1.290851304 | 1.89186E-17 | 1.06174E-15 | K11816 |
| *At1g11050* | 92.49518659 | 11.98252734 | 0.129547577 | -2.948446059 | 2.08964E-17 | 1.16768E-15 | - |
| *CYP76T24* | 39.63126524 | 0 | 0 | -Inf | 2.56574E-17 | 1.43064E-15 | - |
| *RBOHF* | 429.0560292 | 164.9659081 | 0.384485701 | -1.37899815 | 2.64419E-17 | 1.47121E-15 | K13447 |
| *CSLA9* | 592.2820661 | 244.9690016 | 0.413601923 | -1.273685201 | 2.70901E-17 | 1.50405E-15 | K13680 |
| *AGD11* | 155.1482936 | 35.07461251 | 0.226071533 | -2.145148758 | 2.75115E-17 | 1.52418E-15 | K12486 |
| *At4g27220* | 99.05599323 | 14.25141304 | 0.143872295 | -2.797139295 | 2.80219E-17 | 1.54915E-15 | K13459 |
| *uncharacterized protein_05335* | 39.41190249 | 0 | 0 | -Inf | 3.36127E-17 | 1.85034E-15 | - |
| *Kdsr* | 490.2127064 | 193.8709689 | 0.395483361 | -1.338311095 | 3.45724E-17 | 1.89914E-15 | K04708 |
| *CSPL3* | 333.9272379 | 77.96567731 | 0.233481036 | -2.098622723 | 4.15955E-17 | 2.27529E-15 | - |
| *At1g68400* | 263.630006 | 83.74791626 | 0.317672171 | -1.654389385 | 5.10762E-17 | 2.78215E-15 | - |
| *H1* | 1015.584946 | 471.3946887 | 0.464160768 | -1.107303506 | 5.13053E-17 | 2.78877E-15 | K11275 |
| *At1g01500* | 187.8387631 | 49.32041426 | 0.262567818 | -1.929237991 | 5.59627E-17 | 3.03557E-15 | - |
| *At5g41800* | 371.1906712 | 136.1446652 | 0.366778251 | -1.447020001 | 5.6094E-17 | 3.03634E-15 | - |
| *NAT2* | 346.7862464 | 122.252485 | 0.352529797 | -1.504182892 | 5.84733E-17 | 3.15853E-15 | K14611 |
| *PAD4* | 104.9820225 | 16.54855278 | 0.157632254 | -2.665365336 | 5.9467E-17 | 3.20553E-15 | - |
| *BPA1* | 736.1746354 | 327.3725944 | 0.444694205 | -1.16911449 | 6.31335E-17 | 3.38908E-15 | - |
| *uncharacterized protein_19330* | 230.441572 | 69.02437837 | 0.29953093 | -1.739223109 | 6.84533E-17 | 3.66706E-15 | - |
| *TCEA1* | 1123.35279 | 533.4473568 | 0.474870728 | -1.074393267 | 7.08112E-17 | 3.77776E-15 | - |
| *Os04g0338000* | 62.8442117 | 3.920031742 | 0.062376974 | -4.002842629 | 7.47473E-17 | 3.97957E-15 | - |
| *LAC6* | 745.0620867 | 237.5493211 | 0.318831578 | -1.649133568 | 8.51236E-17 | 4.51347E-15 | K05909 |
| *uncharacterized protein_37984* | 38.37160869 | 0 | 0 | -Inf | 8.80383E-17 | 4.65848E-15 | - |
| *AAP2* | 111.4095731 | 18.71807761 | 0.168011393 | -2.573369025 | 9.57824E-17 | 5.04765E-15 | - |
| *CBP1* | 1870.921801 | 929.7827037 | 0.496965027 | -1.008783766 | 1.11355E-16 | 5.85643E-15 | K16296 |
| *ABCB1* | 720.2037425 | 320.0566936 | 0.444397432 | -1.170077615 | 1.18141E-16 | 6.20072E-15 | K05658 |
| *NFD4* | 230.6934737 | 69.99901732 | 0.303428685 | -1.720570617 | 1.19209E-16 | 6.24413E-15 | - |
| *BAM1* | 271.1958369 | 89.01977045 | 0.32824903 | -1.607137346 | 1.20221E-16 | 6.28445E-15 | - |
| *TIP1-1* | 189.8676297 | 47.86242864 | 0.252083142 | -1.988028454 | 1.22722E-16 | 6.40231E-15 | K09873 |
| *TAT* | 460.8778887 | 64.5444544 | 0.140046759 | -2.836019503 | 1.27825E-16 | 6.64177E-15 | K15400 |
| *CRK2* | 66.1097109 | 5.199580588 | 0.078650784 | -3.668395044 | 1.28547E-16 | 6.66591E-15 | - |
| *CYP75A5* | 1500.457475 | 731.4088751 | 0.48745725 | -1.036652394 | 1.39768E-16 | 7.21893E-15 | K13083 |
| *ag4* | 75.49750885 | 7.533986811 | 0.099791197 | -3.324943629 | 1.48776E-16 | 7.66889E-15 | K18108 |
| *LAC17* | 191.274704 | 51.55883652 | 0.269553869 | -1.891354477 | 1.51652E-16 | 7.80163E-15 | K05909 |
| *GDH1* | 1207.204217 | 582.1094372 | 0.482196325 | -1.052307439 | 1.80089E-16 | 9.22796E-15 | K00261 |
| *PT1* | 236.81319 | 73.45715338 | 0.310190295 | -1.688774544 | 2.15249E-16 | 1.09646E-14 | K12742 |
| *At1g67720* | 239.0337448 | 75.93377328 | 0.31766968 | -1.654400695 | 2.50755E-16 | 1.27032E-14 | - |
| *At3g47110* | 154.6498765 | 26.0047302 | 0.168152286 | -2.572159706 | 2.50849E-16 | 1.27032E-14 | - |
| *BRH1* | 105.6454238 | 18.20079141 | 0.172281872 | -2.537157187 | 2.76138E-16 | 1.39023E-14 | K16281 |
| *NIP1-1* | 130.612681 | 28.29161656 | 0.216606966 | -2.206848454 | 3.00038E-16 | 1.50181E-14 | K09874 |
| *uncharacterized protein_23815* | 100.4518541 | 15.13011722 | 0.150620587 | -2.731009123 | 3.15237E-16 | 1.57485E-14 | - |
| *BOR2* | 269.2063032 | 90.13555546 | 0.334819632 | -1.578543971 | 3.26564E-16 | 1.6283E-14 | - |
| *uncharacterized protein_02058* | 516.7681751 | 217.0871188 | 0.420086083 | -1.251243102 | 3.33978E-16 | 1.66207E-14 | - |
| *At3g02645* | 136.4753309 | 30.46791944 | 0.223248548 | -2.163277304 | 3.62808E-16 | 1.79864E-14 | - |
| *At5g48900* | 141.2783605 | 32.48721158 | 0.229951788 | -2.12059668 | 3.96234E-16 | 1.9606E-14 | K01728 |
| *CYP76B6* | 138.7831767 | 31.66841662 | 0.228186279 | -2.131716054 | 4.00085E-16 | 1.97213E-14 | - |
| *TAT* | 82.68555943 | 9.705770987 | 0.117381693 | -3.090720668 | 4.09781E-16 | 2.01227E-14 | K15400 |
| *SBT1.5* | 72.75972078 | 7.363444912 | 0.101202215 | -3.304687227 | 4.13539E-16 | 2.02689E-14 | - |
| *AATL1* | 1601.962822 | 791.3819365 | 0.49400768 | -1.017394626 | 4.31866E-16 | 2.11272E-14 | - |
| *Prcp* | 146.5395904 | 33.61099062 | 0.229364573 | -2.124285519 | 4.41421E-16 | 2.15135E-14 | K01285 |
| *IAN9* | 299.5132981 | 103.9409617 | 0.347032878 | -1.526855746 | 4.4938E-16 | 2.18603E-14 | - |
| *PAP18* | 88.36655416 | 11.9949659 | 0.135741017 | -2.881071371 | 4.54391E-16 | 2.20626E-14 | - |
| *ARF* | 965.4826335 | 459.9382874 | 0.47638173 | -1.06981001 | 4.56982E-16 | 2.2147E-14 | K07937 |
| *UGT85A1* | 418.5871333 | 155.8440107 | 0.372309606 | -1.425425255 | 4.66452E-16 | 2.25638E-14 | - |
| *ACR4* | 98.95202739 | 16.97313278 | 0.171528904 | -2.543476395 | 5.19587E-16 | 2.50406E-14 | - |
| *RNP1* | 231.004104 | 71.56775974 | 0.309811638 | -1.690536757 | 5.62244E-16 | 2.70461E-14 | K14411 |
| *DTX48* | 327.1122438 | 117.390587 | 0.358869438 | -1.47846903 | 6.14093E-16 | 2.94856E-14 | K03327 |
| *At5g48380* | 1423.957794 | 709.2722357 | 0.498099198 | -1.005495006 | 6.16003E-16 | 2.95226E-14 | - |
| *RBOHC* | 276.7175302 | 93.29446634 | 0.337146932 | -1.568550626 | 6.66282E-16 | 3.18146E-14 | K13447 |
| *PHO1-3* | 281.0043626 | 97.17825001 | 0.345824702 | -1.531887171 | 7.79084E-16 | 3.71325E-14 | - |
| *At3g16150* | 989.3192883 | 481.3856844 | 0.486582734 | -1.039242966 | 7.98751E-16 | 3.8E-14 | K13051 |
| *At3g47570* | 49.0924062 | 2.063336677 | 0.042029651 | -4.572448729 | 8.6919E-16 | 4.11248E-14 | - |
| *uncharacterized protein_49306* | 224.5870351 | 72.79857276 | 0.324144146 | -1.625292576 | 8.94015E-16 | 4.22223E-14 | - |
| *ALDH3F1* | 621.8610306 | 275.6841077 | 0.443321086 | -1.17357611 | 9.25804E-16 | 4.36441E-14 | K00128 |
| *CDA1* | 298.4948477 | 109.5062323 | 0.366861382 | -1.44669305 | 1.07188E-15 | 5.03475E-14 | K01489 |
| *TDR* | 985.842992 | 467.9887388 | 0.474709201 | -1.074884083 | 1.07721E-15 | 5.0506E-14 | - |
| *P85* | 654.3542774 | 252.1744416 | 0.385379068 | -1.375649882 | 1.10142E-15 | 5.15481E-14 | - |
| *TPS-mISO1* | 95.97783202 | 15.47112684 | 0.161194794 | -2.633122946 | 1.14103E-15 | 5.33057E-14 | K04120 |
| *GPXMC1* | 156.2087323 | 40.7495957 | 0.260866311 | -1.938617454 | 1.32307E-15 | 6.16987E-14 | K00432 |
| *SULTR3;1* | 74.15469774 | 8.17772116 | 0.110279206 | -3.180767315 | 1.51716E-15 | 7.037E-14 | K17471 |
| *UGT83A1* | 63.71214383 | 5.640025266 | 0.088523552 | -3.497794852 | 1.65073E-15 | 7.6429E-14 | - |
| *MYR2* | 335.8087522 | 126.8510848 | 0.377748001 | -1.404503974 | 1.66228E-15 | 7.68262E-14 | - |
| *ALMT9* | 79.19708291 | 9.537488442 | 0.120427269 | -3.053765983 | 1.75024E-15 | 8.07479E-14 | - |
| *TUR2* | 574.5120875 | 250.098518 | 0.435323335 | -1.199840739 | 1.76041E-15 | 8.10725E-14 | - |
| *At1g54290* | 823.2465677 | 380.1059334 | 0.461715783 | -1.114923046 | 1.89016E-15 | 8.68937E-14 | K03113 |
| *GAT1* | 472.8911859 | 200.6401363 | 0.424283941 | -1.236898019 | 2.01478E-15 | 9.24587E-14 | - |
| *MWL2* | 908.7815319 | 429.9936614 | 0.473154049 | -1.079618124 | 2.06841E-15 | 9.4752E-14 | - |
| *HSL1* | 98.15847046 | 16.71343419 | 0.170269913 | -2.554104568 | 2.28841E-15 | 1.04645E-13 | - |
| *CPK17* | 283.4406694 | 100.3777551 | 0.35414027 | -1.497607191 | 2.71497E-15 | 1.23281E-13 | K13412 |
| *RACD* | 547.4713968 | 238.4242775 | 0.435500884 | -1.199252446 | 2.73737E-15 | 1.23978E-13 | - |
| *RUN1* | 118.2275944 | 24.70139682 | 0.208930893 | -2.25890227 | 2.80891E-15 | 1.2666E-13 | - |
| *GDI1* | 664.8225801 | 275.5149543 | 0.414418768 | -1.270838756 | 2.93796E-15 | 1.32249E-13 | K12462 |
| *RBOHF* | 476.1831715 | 180.4675491 | 0.378987667 | -1.399777195 | 3.02703E-15 | 1.36022E-13 | K13447 |
| *PGDH1* | 418.0538876 | 173.2700282 | 0.414468166 | -1.270666797 | 3.0628E-15 | 1.37314E-13 | K00058 |
| *At2g05160* | 549.4050114 | 240.1239376 | 0.43706179 | -1.19409084 | 3.36135E-15 | 1.49747E-13 | - |
| *At2g26730* | 944.5128345 | 453.7214626 | 0.480376175 | -1.057763495 | 3.7667E-15 | 1.67231E-13 | - |
| *IP5P2* | 119.0179027 | 24.90130957 | 0.209223226 | -2.256885078 | 3.98576E-15 | 1.76353E-13 | - |
| *SMXL3* | 184.3593625 | 53.33980688 | 0.289325186 | -1.789236178 | 4.8802E-15 | 2.14828E-13 | - |
| *RUN1* | 44.71098822 | 0.286860049 | 0.006415874 | -7.284138567 | 4.91131E-15 | 2.15466E-13 | - |
| *uncharacterized protein_27504* | 284.4167654 | 101.2564593 | 0.356014383 | -1.489992567 | 5.33352E-15 | 2.332E-13 | - |
| *TCEA1* | 33.94768811 | 0 | 0 | -Inf | 5.63253E-15 | 2.45859E-13 | - |
| *NFD4* | 413.0216838 | 168.0085758 | 0.406779068 | -1.29768265 | 5.7071E-15 | 2.48695E-13 | - |
| *TCP14* | 393.489499 | 157.9989867 | 0.401532918 | -1.316409829 | 5.79457E-15 | 2.52083E-13 | - |
| *At3g43860* | 179.3126965 | 46.74885303 | 0.260711338 | -1.939474769 | 6.236E-15 | 2.70833E-13 | K01179 |
| *At5g39450* | 972.6205133 | 475.8105065 | 0.489204679 | -1.031489891 | 6.78176E-15 | 2.94042E-13 | - |
| *NPF4.5* | 48.41675925 | 2.238397285 | 0.046231869 | -4.434968494 | 7.07103E-15 | 3.06073E-13 | K14638 |
| *BAM1* | 73.58256792 | 8.835011635 | 0.120069357 | -3.058060085 | 7.51222E-15 | 3.24628E-13 | - |
| *PR5K* | 77.55485492 | 10.20831007 | 0.131626964 | -2.925473042 | 8.55729E-15 | 3.6734E-13 | - |
| *uncharacterized protein_42636* | 92.02362563 | 15.71731927 | 0.170796566 | -2.549649124 | 8.82177E-15 | 3.78067E-13 | - |
| *CAT1* | 78.82985367 | 11.04967359 | 0.14017118 | -2.834738344 | 1.04433E-14 | 4.46087E-13 | K03294 |
| *Ankrd13b* | 556.7807659 | 247.7528157 | 0.444973733 | -1.16820792 | 1.08878E-14 | 4.63546E-13 | - |
| *CBP1* | 615.2922592 | 283.1976361 | 0.460265235 | -1.119462619 | 1.09708E-14 | 4.66311E-13 | K16296 |
| *IQD2* | 658.8297669 | 305.6540067 | 0.463934725 | -1.10800626 | 1.11867E-14 | 4.7471E-13 | - |
| *GDPD1* | 601.0302262 | 229.2705046 | 0.38146252 | -1.390386781 | 1.2375E-14 | 5.24279E-13 | K18696 |
| *BXL4* | 1146.334572 | 507.8847573 | 0.443051069 | -1.174455093 | 1.26134E-14 | 5.33506E-13 | K15920 |
| *ERF017* | 47.65987383 | 2.194352741 | 0.046041933 | -4.44090777 | 1.26601E-14 | 5.3461E-13 | - |
| *CRK2* | 36.02414311 | 0.363652364 | 0.010094685 | -6.630260351 | 1.2935E-14 | 5.45333E-13 | - |
| *KIN4C* | 337.7143957 | 133.0591697 | 0.393999105 | -1.343735742 | 1.39259E-14 | 5.86152E-13 | K10395 |
| *At3g18200* | 119.3989278 | 25.75739599 | 0.215725522 | -2.212731228 | 1.53495E-14 | 6.45026E-13 | - |
| *Tmem45b* | 154.5553563 | 28.17872453 | 0.182321242 | -2.455445437 | 1.55541E-14 | 6.52568E-13 | - |
| *GOLS1* | 105.0366224 | 20.91804164 | 0.199149984 | -2.328072734 | 1.78445E-14 | 7.46245E-13 | K18819 |
| *CRT3* | 3615.549844 | 1597.241802 | 0.441770096 | -1.178632331 | 1.84467E-14 | 7.68947E-13 | K08057 |
| *O10* | 466.2323928 | 202.8176059 | 0.435013974 | -1.20086635 | 1.85123E-14 | 7.70443E-13 | - |
| *HSL1* | 187.4000376 | 57.5139017 | 0.30690443 | -1.704138625 | 1.96849E-14 | 8.17932E-13 | - |
| *LTPG5* | 282.9325813 | 95.7553949 | 0.338438912 | -1.563032645 | 2.08215E-14 | 8.63775E-13 | - |
| *MLO6* | 71.12058934 | 8.469099917 | 0.119080846 | -3.069986724 | 2.09695E-14 | 8.68527E-13 | K08472 |
| *SAT1* | 312.9470919 | 120.0060694 | 0.383470793 | -1.382811396 | 2.20883E-14 | 9.11953E-13 | K00640 |
| *uncharacterized protein_22155* | 487.4791203 | 203.5743572 | 0.417606311 | -1.25978458 | 2.24524E-14 | 9.24042E-13 | - |
| *MAN1* | 423.9783706 | 179.9187054 | 0.424358217 | -1.236645482 | 2.28212E-14 | 9.3773E-13 | K19355 |
| *LAX4* | 1374.405002 | 610.2208012 | 0.443989072 | -1.171403928 | 2.3822E-14 | 9.75762E-13 | K13946 |
| *GLR3.3* | 453.4154479 | 196.6591769 | 0.433728444 | -1.205136035 | 2.5645E-14 | 1.04805E-12 | K05387 |
| *HST* | 544.5970347 | 247.0445793 | 0.453628212 | -1.140417727 | 2.71546E-14 | 1.10702E-12 | K13065 |
| *IAN9* | 274.292589 | 100.8995108 | 0.36785358 | -1.442796464 | 2.83407E-14 | 1.15356E-12 | - |
| *LRK10L-2.5* | 58.29958115 | 5.350905863 | 0.091782921 | -3.445630465 | 3.216E-14 | 1.30492E-12 | - |
| *CRK8* | 71.47062574 | 8.753700611 | 0.122479697 | -3.029385472 | 3.23376E-14 | 1.31008E-12 | - |
| *SUS3* | 304.4410181 | 116.5694336 | 0.382896609 | -1.38497321 | 3.29522E-14 | 1.33289E-12 | K00695 |
| *CRK2* | 64.92273106 | 7.113851344 | 0.109574123 | -3.190020958 | 3.50141E-14 | 1.4097E-12 | - |
| *CRK2* | 167.3219167 | 49.20064576 | 0.294047825 | -1.765877273 | 3.76785E-14 | 1.51447E-12 | - |
| *RITF1* | 660.9116081 | 314.252831 | 0.475483903 | -1.072531593 | 3.77334E-14 | 1.51447E-12 | - |
| *At5g48900* | 88.84416381 | 15.43271857 | 0.173705485 | -2.525284784 | 3.8542E-14 | 1.54454E-12 | K01728 |
| *XA21* | 161.0901176 | 46.20671469 | 0.286837674 | -1.801693569 | 4.07059E-14 | 1.62623E-12 | K04730 |
| *DTX16* | 263.2785716 | 96.90829803 | 0.36808274 | -1.441897994 | 4.36558E-14 | 1.73872E-12 | K03327 |
| *GT5* | 85.05833477 | 13.4823481 | 0.158507078 | -2.657380831 | 4.73894E-14 | 1.88453E-12 | K13691 |
| *LOX1.5* | 1642.071393 | 637.5643513 | 0.388268351 | -1.364873983 | 4.86584E-14 | 1.9279E-12 | K15718 |
| *At2g23540* | 106.7926317 | 21.03217462 | 0.196944061 | -2.34414218 | 4.87032E-14 | 1.9279E-12 | - |
| *CYP750A1* | 56.36929415 | 4.877713413 | 0.086531391 | -3.530632601 | 5.8879E-14 | 2.32716E-12 | - |
| *DUR3* | 274.4282792 | 101.201118 | 0.368770734 | -1.439203928 | 5.94546E-14 | 2.34633E-12 | - |
| *BXL4* | 523.9533468 | 235.3321523 | 0.44914715 | -1.154739916 | 6.02432E-14 | 2.37384E-12 | K15920 |
| *CSPL5* | 201.5378193 | 46.87991905 | 0.232611027 | -2.104008607 | 6.04698E-14 | 2.37915E-12 | - |
| *PUB52* | 955.9837985 | 417.0701644 | 0.436273256 | -1.196696058 | 6.64506E-14 | 2.60655E-12 | - |
| *At3g02645* | 42.29482638 | 1.741469503 | 0.041174528 | -4.602104087 | 6.98877E-14 | 2.73679E-12 | - |
| *At1g67720* | 171.5495678 | 52.14149488 | 0.303944193 | -1.718121641 | 6.99821E-14 | 2.73679E-12 | - |
| *OPT7* | 89.22142606 | 15.80203143 | 0.177110276 | -2.497280171 | 7.41083E-14 | 2.88848E-12 | - |
| *TUR2* | 288.6357026 | 87.43183638 | 0.302914143 | -1.723019158 | 7.4195E-14 | 2.88848E-12 | - |
| *NHL13* | 232.0303015 | 82.70202212 | 0.35642768 | -1.488318714 | 7.77854E-14 | 3.02372E-12 | - |
| *PT30* | 34.14034304 | 0.324126529 | 0.009493945 | -6.718776638 | 8.02811E-14 | 3.11606E-12 | K12742 |
| *C7A12* | 31.03030923 | 0 | 0 | -Inf | 9.63075E-14 | 3.71034E-12 | - |
| *FEI1* | 249.7387475 | 92.36484115 | 0.369845857 | -1.435003979 | 1.00681E-13 | 3.87307E-12 | - |
| *At5g48740* | 116.1115196 | 15.95904218 | 0.137445813 | -2.863065139 | 1.34016E-13 | 5.12502E-12 | - |
| *MIK1* | 316.9699946 | 127.7613458 | 0.403070789 | -1.310894861 | 1.46328E-13 | 5.57938E-12 | - |
| *CYP92C6* | 144.1702813 | 39.24875653 | 0.272238884 | -1.877054951 | 1.58253E-13 | 6.0076E-12 | - |
| *LRK10L-2.5* | 70.14057832 | 6.863165188 | 0.097848711 | -3.353303342 | 1.65785E-13 | 6.28434E-12 | K04733 |
| *UGT85A2* | 120.1305073 | 28.59434129 | 0.238027308 | -2.070800995 | 1.67867E-13 | 6.34468E-12 | - |
| *DTX40* | 210.3008035 | 71.31699941 | 0.339119006 | -1.560136453 | 1.72392E-13 | 6.49678E-12 | K03327 |
| *NPF5.6* | 150.6975169 | 43.11565711 | 0.286107283 | -1.805371872 | 1.81799E-13 | 6.82155E-12 | K14638 |
| *LCA1* | 570.4809274 | 269.1902303 | 0.471865434 | -1.083552603 | 2.04662E-13 | 7.63523E-12 | K01537 |
| *DSP1* | 338.1724477 | 140.7455994 | 0.416194756 | -1.264669308 | 2.10035E-13 | 7.81318E-12 | K18045 |
| *TPS1* | 219.9761424 | 78.29750115 | 0.355936331 | -1.490308894 | 2.1741E-13 | 8.07595E-12 | K16055 |
| *45566* | 88.61778951 | 16.73148404 | 0.188805026 | -2.405030926 | 2.19251E-13 | 8.13268E-12 | - |
| *uncharacterized protein_25487* | 74.98647639 | 11.36250334 | 0.151527367 | -2.722349714 | 2.30154E-13 | 8.51276E-12 | - |
| *SCPL35* | 444.7121456 | 199.0139423 | 0.447511821 | -1.160002302 | 2.32485E-13 | 8.58671E-12 | K16297 |
| *uncharacterized protein_06714* | 1839.8889 | 428.58319 | 0.232939712 | -2.101971483 | 2.37571E-13 | 8.76208E-12 | - |
| *CYP75B137* | 154.9059897 | 37.29600333 | 0.240765405 | -2.054299986 | 2.41351E-13 | 8.88883E-12 | K07408 |
| *CYP750A1* | 55.49309682 | 4.973722352 | 0.089627767 | -3.479910436 | 2.43104E-13 | 8.94073E-12 | - |
| *CRSP* | 257.209623 | 96.4328712 | 0.374919375 | -1.415347711 | 2.49933E-13 | 9.15291E-12 | - |
| *uncharacterized protein_28512* | 628.2507292 | 304.6825948 | 0.484969743 | -1.044033353 | 2.76432E-13 | 1.01091E-11 | - |
| *uncharacterized protein_02297* | 262.8537286 | 63.81685296 | 0.242784659 | -2.042250829 | 2.79402E-13 | 1.02033E-11 | - |
| *AMT3-1* | 70.71513838 | 4.299474607 | 0.060799918 | -4.039786814 | 3.09939E-13 | 1.12236E-11 | K03320 |
| *TUBB8* | 360.7267918 | 152.8603345 | 0.423756533 | -1.238692486 | 3.14972E-13 | 1.13899E-11 | K07375 |
| *At3g47110* | 196.0313989 | 64.54534943 | 0.32926026 | -1.602699697 | 3.31482E-13 | 1.19703E-11 | - |
| *NPF5.2* | 537.2946193 | 252.4700431 | 0.469891255 | -1.089601176 | 3.61059E-13 | 1.30021E-11 | K14638 |
| *PAPS4* | 761.6329148 | 379.4792797 | 0.498244328 | -1.005074714 | 3.63179E-13 | 1.30603E-11 | K14376 |
| *POLX* | 434.7513339 | 199.6912944 | 0.459323017 | -1.122419014 | 3.81518E-13 | 1.37008E-11 | - |
| *PLP7* | 418.9580464 | 185.9980835 | 0.443953959 | -1.171518027 | 3.99219E-13 | 1.42772E-11 | - |
| *FRI3* | 48.84102257 | 2.844865153 | 0.058247453 | -4.101661231 | 4.20013E-13 | 1.49795E-11 | K00522 |
| *UGT84A13* | 308.7833343 | 125.408914 | 0.406138868 | -1.299954992 | 4.33418E-13 | 1.53941E-11 | K13691 |
| *SCPL31* | 141.315032 | 38.66150529 | 0.273583813 | -1.869945222 | 4.40618E-13 | 1.5607E-11 | K16297 |
| *SPX1* | 116.7234868 | 29.11718958 | 0.249454419 | -2.003151869 | 4.42637E-13 | 1.56571E-11 | - |
| *At3g47110* | 74.67827628 | 11.93056294 | 0.159759485 | -2.646026509 | 4.59608E-13 | 1.62353E-11 | - |
| *CAT1* | 140.9405697 | 38.86131963 | 0.275728413 | -1.858680154 | 4.89435E-13 | 1.72654E-11 | K03294 |
| *MAO1B* | 17518.52465 | 6598.270965 | 0.376645357 | -1.408721351 | 6.52001E-13 | 2.27831E-11 | K05933 |
| *PATL4* | 890.9415245 | 433.6697093 | 0.486754402 | -1.038734068 | 6.82019E-13 | 2.38E-11 | K19996 |
| *CEPR1* | 407.0345533 | 145.4143876 | 0.357253178 | -1.484981251 | 8.43659E-13 | 2.92048E-11 | - |
| *PUB26* | 88.52445367 | 17.31990205 | 0.195651047 | -2.353645263 | 9.68644E-13 | 3.34867E-11 | - |
| *TAR4* | 52.3579054 | 5.029038689 | 0.096051182 | -3.380052826 | 1.01635E-12 | 3.50423E-11 | - |
| *ASPR* | 417.2854229 | 165.1615005 | 0.395799832 | -1.337157093 | 1.11169E-12 | 3.81772E-11 | K08245 |
| *QRT3* | 390.5496104 | 171.6608892 | 0.439536706 | -1.185944445 | 1.18188E-12 | 4.05341E-11 | - |
| *CYP704C1* | 372.0535078 | 162.2115148 | 0.435989747 | -1.197633887 | 1.34307E-12 | 4.57596E-11 | - |
| *ag4* | 57.33004797 | 6.572879018 | 0.114649808 | -3.12469415 | 1.42073E-12 | 4.82788E-11 | - |
| *PME34* | 88.30287441 | 17.76486544 | 0.201181055 | -2.31343364 | 1.483E-12 | 5.0263E-11 | - |
| *BPS1* | 93.24520874 | 19.5142548 | 0.209278901 | -2.256501226 | 1.4896E-12 | 5.04209E-11 | - |
| *RPK1* | 59.12567699 | 7.644619485 | 0.129294409 | -2.9512682 | 1.64847E-12 | 5.56529E-11 | - |
| *RHM1* | 1391.079555 | 563.9311681 | 0.405391026 | -1.302613942 | 1.65959E-12 | 5.59557E-11 | K12450 |
| *SDR2a* | 28.11499471 | 0 | 0 | -Inf | 1.66996E-12 | 5.6232E-11 | - |
| *SDR2a* | 27.78030438 | 0 | 0 | -Inf | 1.91282E-12 | 6.42431E-11 | - |
| *LAC1* | 135.2741893 | 38.32039651 | 0.283279439 | -1.819702201 | 2.00408E-12 | 6.72209E-11 | K05909 |
| *At5g48740* | 79.55988299 | 14.24014125 | 0.178986453 | -2.482077698 | 2.11523E-12 | 7.08574E-11 | - |
| *SB09* | 820.1746786 | 284.7585072 | 0.347192512 | -1.526192261 | 2.13534E-12 | 7.14389E-11 | K03671 |
| *AATP1* | 126.7407452 | 33.95771069 | 0.267930496 | -1.900069298 | 2.28534E-12 | 7.60645E-11 | K08900 |
| *FLZ10* | 448.0501 | 207.4785477 | 0.463069973 | -1.110697884 | 2.32529E-12 | 7.7196E-11 | - |
| *HAK5* | 27.72208983 | 0 | 0 | -Inf | 2.35982E-12 | 7.82423E-11 | K03549 |
| *MYB123* | 334.1573751 | 145.6210035 | 0.435785694 | -1.19830926 | 2.79249E-12 | 9.21167E-11 | K09422 |
| *HIPP23* | 34.3772645 | 1.014164776 | 0.029501032 | -5.083090773 | 2.82623E-12 | 9.31113E-11 | - |
| *comta* | 219.5208904 | 83.7635584 | 0.381574429 | -1.389963602 | 2.90514E-12 | 9.55894E-11 | K00545 |
| *SWEET3B* | 298.2894369 | 51.39172074 | 0.172288101 | -2.537105026 | 3.04428E-12 | 9.96617E-11 | K15382 |
| *BGLU13* | 68.54136708 | 10.68602122 | 0.155906158 | -2.68125018 | 3.50477E-12 | 1.1359E-10 | K01188 |
| *SDR2a* | 29.92441958 | 0.363652364 | 0.012152361 | -6.362619502 | 3.54104E-12 | 1.14622E-10 | - |
| *At4g16563* | 317.1076798 | 97.52618681 | 0.307549117 | -1.701111263 | 3.68097E-12 | 1.19003E-10 | - |
| *VSR6* | 1629.6734 | 705.2893644 | 0.432779577 | -1.208295676 | 4.07381E-12 | 1.31539E-10 | - |
| *At5g13200* | 486.0642988 | 230.0658844 | 0.473323972 | -1.079100105 | 4.10364E-12 | 1.32337E-10 | - |
| *At5g64970* | 359.884984 | 158.6754439 | 0.440905986 | -1.18145703 | 4.35133E-12 | 1.39803E-10 | - |
| *TCM_034089* | 31.88879569 | 0.573720097 | 0.017991275 | -5.796558748 | 4.4656E-12 | 1.43297E-10 | K08241 |
| *NORK* | 54.66766729 | 5.487607403 | 0.100381225 | -3.31643864 | 5.00617E-12 | 1.60445E-10 | - |
| *NRT3.2* | 40.18325008 | 2.16160497 | 0.053793682 | -4.216419454 | 5.06407E-12 | 1.621E-10 | - |
| *ZOX1* | 52.06505136 | 5.640025266 | 0.108326509 | -3.206541758 | 5.22291E-12 | 1.66979E-10 | K13495 |
| *BANGLUC* | 46.52041309 | 3.554120024 | 0.07639915 | -3.7102996 | 5.81673E-12 | 1.84824E-10 | - |
| *LAC11* | 173.8097462 | 58.55296857 | 0.336879662 | -1.569694761 | 6.20143E-12 | 1.96566E-10 | K05909 |
| *QS* | 556.7342 | 220.418684 | 0.395913677 | -1.336742189 | 6.93983E-12 | 2.189E-10 | K03517 |
| *OPT4* | 577.2068588 | 285.5503148 | 0.494710536 | -1.015343468 | 7.08508E-12 | 2.22939E-10 | - |
| *CKX7* | 83.64409674 | 17.34247062 | 0.207336456 | -2.269954284 | 7.15314E-12 | 2.24808E-10 | K00279 |
| *LOX1.1* | 66.25676281 | 10.24331719 | 0.154600327 | -2.693384725 | 7.26092E-12 | 2.27919E-10 | K15718 |
| *HHT1* | 512.494041 | 173.4805653 | 0.3385026 | -1.562761181 | 7.33812E-12 | 2.30064E-10 | K15400 |
| *RPK1* | 47.09187661 | 4.303993316 | 0.091395664 | -3.451730472 | 7.78334E-12 | 2.43433E-10 | K13420 |
| *BANGLUC* | 91.4573269 | 19.58543581 | 0.214148352 | -2.223317522 | 7.79743E-12 | 2.4358E-10 | - |
| *CYP720B2* | 126.9039578 | 36.76071648 | 0.289673522 | -1.787500273 | 8.06756E-12 | 2.51413E-10 | - |
| *uncharacterized protein_45338* | 198.2326964 | 71.5293265 | 0.360835159 | -1.470588177 | 8.14326E-12 | 2.53468E-10 | - |
| *HB1* | 40.9876546 | 2.485731499 | 0.060645858 | -4.043447064 | 8.5757E-12 | 2.66608E-10 | - |
| *PXC3* | 222.4334704 | 74.87226198 | 0.336605196 | -1.570870646 | 9.3066E-12 | 2.88293E-10 | - |
| *FTIP3* | 121.9955643 | 34.11578905 | 0.279647783 | -1.838317204 | 1.00229E-11 | 3.09373E-10 | - |
| *PUX10* | 292.9423101 | 123.6358634 | 0.422048503 | -1.244519288 | 1.04807E-11 | 3.2197E-10 | K18726 |
| *At3g02645* | 329.9184932 | 102.1721332 | 0.309689015 | -1.691107884 | 1.14289E-11 | 3.50683E-10 | - |
| *uncharacterized protein_31758* | 974.6656099 | 373.7144931 | 0.383428418 | -1.382970827 | 1.37677E-11 | 4.17997E-10 | - |
| *MAPKKK17* | 41.58907463 | 2.965702012 | 0.071309642 | -3.809759034 | 1.38711E-11 | 4.20644E-10 | - |
| *RK2* | 166.5443722 | 58.0672384 | 0.348659265 | -1.520110275 | 1.51866E-11 | 4.59461E-10 | - |
| *NRT2.1* | 40.14399618 | 2.406679829 | 0.059951177 | -4.060068107 | 1.55869E-11 | 4.71022E-10 | K02575 |
| *CRK2* | 63.26795699 | 6.552569807 | 0.103568538 | -3.271342287 | 1.56779E-11 | 4.7322E-10 | - |
| *uncharacterized protein_36021* | 148.3645135 | 47.54391341 | 0.320453404 | -1.641813499 | 1.5878E-11 | 4.78702E-10 | - |
| *At1g67720* | 32.13759693 | 0.897846626 | 0.027937578 | -5.161649228 | 1.85075E-11 | 5.53476E-10 | - |
| *At3g47110* | 73.90781259 | 13.8504942 | 0.187402302 | -2.415789422 | 1.85687E-11 | 5.54664E-10 | K13428 |
| *LOX1.5* | 70.28091524 | 6.579657081 | 0.0936194 | -3.417048678 | 2.07205E-11 | 6.16102E-10 | K15718 |
| *At4g08300* | 27.9213076 | 0.324126529 | 0.011608573 | -6.428665591 | 2.32192E-11 | 6.8882E-10 | - |
| *TIP1-1* | 113.9873973 | 32.12015807 | 0.281786924 | -1.827323427 | 2.32966E-11 | 6.90324E-10 | K09873 |
| *XA21* | 47.88130094 | 4.912720539 | 0.102602069 | -3.284868272 | 2.36637E-11 | 6.99603E-10 | - |
| *DTX40* | 2079.752952 | 770.556366 | 0.370503797 | -1.432439766 | 2.50121E-11 | 7.36949E-10 | K03327 |
| *CYP75A3* | 962.17893 | 475.2453802 | 0.493926197 | -1.017632605 | 2.50582E-11 | 7.37468E-10 | K13083 |
| *UBC23* | 86.44298216 | 17.75138349 | 0.205353668 | -2.283817375 | 2.52958E-11 | 7.41934E-10 | K10581 |
| *uncharacterized protein_21809* | 260.2752381 | 100.7629576 | 0.387140007 | -1.36907269 | 2.54112E-11 | 7.44477E-10 | - |
| *At4g02290* | 403.649228 | 191.9610428 | 0.475564003 | -1.072288577 | 2.67707E-11 | 7.82538E-10 | - |
| *UGT86A1* | 84.10776224 | 18.81853107 | 0.223743095 | -2.160084932 | 2.78586E-11 | 8.12507E-10 | - |
| *NAKR2* | 304.3950531 | 125.0329964 | 0.410758963 | -1.283636039 | 2.88934E-11 | 8.41741E-10 | - |
| *CYP71AU50* | 74.31842833 | 13.64385258 | 0.183586398 | -2.44546892 | 2.92758E-11 | 8.51922E-10 | - |
| *uncharacterized protein_29787* | 96.36800623 | 23.31570903 | 0.241944499 | -2.047251954 | 3.21776E-11 | 9.33223E-10 | - |
| *KCS11* | 556.4796505 | 230.9582196 | 0.415034439 | -1.26869704 | 3.22612E-11 | 9.34601E-10 | K15397 |
| *ureH* | 618.5958318 | 275.5270727 | 0.445407257 | -1.166803031 | 3.36497E-11 | 9.71571E-10 | - |
| *At4g33820* | 79.23685484 | 16.14311523 | 0.203732408 | -2.295252605 | 3.72272E-11 | 1.07247E-09 | - |
| *At1g12460* | 190.3796982 | 70.22604232 | 0.368873588 | -1.438801603 | 3.84356E-11 | 1.10483E-09 | - |
| *TMK1* | 295.1148355 | 127.8585957 | 0.433250316 | -1.206727294 | 4.22199E-11 | 1.20825E-09 | - |
| *TDR* | 297.1766108 | 108.8423605 | 0.366254801 | -1.449080423 | 4.23168E-11 | 1.20969E-09 | - |
| *BGLU13* | 50.29436236 | 5.501138551 | 0.109378831 | -3.192594549 | 4.44692E-11 | 1.26564E-09 | K01188 |
| *GASA2* | 52.931285 | 6.624843413 | 0.125159316 | -2.998162418 | 4.56553E-11 | 1.29797E-09 | - |
| *SLAC1* | 79.57191105 | 16.24816159 | 0.204194688 | -2.291982756 | 4.77484E-11 | 1.35599E-09 | - |
| *LRK10L-2.5* | 41.01436242 | 3.136243911 | 0.076466967 | -3.709019532 | 5.17463E-11 | 1.46471E-09 | - |
| *HPCA1* | 129.5920141 | 40.68298259 | 0.313931247 | -1.671479461 | 5.28802E-11 | 1.49518E-09 | - |
| *AAP6* | 151.3321421 | 50.94102268 | 0.336617337 | -1.570818613 | 5.2966E-11 | 1.49597E-09 | - |
| *WRKY72A* | 100.1603327 | 20.64590449 | 0.206128553 | -2.27838373 | 6.02844E-11 | 1.69529E-09 | - |
| *FRI3* | 74.24855161 | 14.1803062 | 0.19098428 | -2.388474197 | 6.12031E-11 | 1.71886E-09 | K00522 |
| *GT7* | 394.2704309 | 189.0010262 | 0.47936901 | -1.060791449 | 6.1783E-11 | 1.72992E-09 | - |
| *SBT5.6* | 349.8654398 | 161.6207875 | 0.461951279 | -1.114187393 | 6.27983E-11 | 1.75645E-09 | - |
| *BOR2* | 78.70759349 | 16.32269455 | 0.207383987 | -2.269623593 | 6.81985E-11 | 1.89157E-09 | - |
| *CAMBP25* | 222.3602795 | 90.04292344 | 0.404941582 | -1.304214298 | 6.9462E-11 | 1.92416E-09 | - |
| *XTH8* | 591.5002464 | 221.5075528 | 0.374484295 | -1.417022878 | 7.22268E-11 | 1.99683E-09 | K08235 |
| *LRK10L-1.2* | 55.99018514 | 8.042186387 | 0.143635646 | -2.799514273 | 7.22397E-11 | 1.99683E-09 | - |
| *PHN1* | 153.301683 | 52.32224096 | 0.341302456 | -1.550877298 | 7.65168E-11 | 2.11056E-09 | K11593 |
| *CER3* | 372.1399111 | 160.9094473 | 0.432389654 | -1.209596092 | 8.50245E-11 | 2.34025E-09 | - |
| *At5g07050* | 1858.693738 | 912.0703703 | 0.490705032 | -1.027072029 | 9.20766E-11 | 2.51832E-09 | - |
| *HSP21.7* | 238.6992066 | 97.50925376 | 0.40850263 | -1.291582727 | 9.35733E-11 | 2.55386E-09 | K13993 |
| *EXL2* | 42.21100552 | 4.166199189 | 0.098699359 | -3.340815474 | 1.03289E-10 | 2.80133E-09 | - |
| *HOMT3* | 75.54761034 | 15.78283979 | 0.208912495 | -2.259029311 | 1.09198E-10 | 2.9554E-09 | - |
| *At4g16820* | 38.84043898 | 3.035716263 | 0.07815865 | -3.677450647 | 1.09312E-10 | 2.95542E-09 | - |
| *CBP1* | 170.6533776 | 62.50229698 | 0.366252915 | -1.449087854 | 1.13796E-10 | 3.07344E-09 | K16296 |
| *uncharacterized protein_11623* | 347.831705 | 163.5858316 | 0.47030167 | -1.088341641 | 1.14539E-10 | 3.09029E-09 | - |
| *MDIS2* | 23.6988213 | 0 | 0 | -Inf | 1.14851E-10 | 3.09547E-09 | K13420 |
| *pgk* | 151.4915918 | 49.73595759 | 0.32830837 | -1.606876564 | 1.15038E-10 | 3.0973E-09 | K00927 |
| *LECA* | 23.55368548 | 0 | 0 | -Inf | 1.21862E-10 | 3.27084E-09 | - |
| *gmppA* | 226.2115561 | 92.84265147 | 0.410423999 | -1.284813002 | 1.25772E-10 | 3.36881E-09 | K00966 |
| *BOR2* | 70.39107804 | 14.16218217 | 0.201192858 | -2.313349002 | 1.28739E-10 | 3.44117E-09 | - |
| *PANC* | 315.7205193 | 146.6521263 | 0.464499826 | -1.106250039 | 1.29299E-10 | 3.4526E-09 | K01918 |
| *ACD11* | 690.2974651 | 249.6290484 | 0.36162533 | -1.467432362 | 1.33482E-10 | 3.55332E-09 | - |
| *At2g27500* | 206.6297879 | 82.94034389 | 0.401395872 | -1.316902315 | 1.44932E-10 | 3.83058E-09 | - |
| *LECRK41* | 44.81826821 | 4.85849679 | 0.108404385 | -3.205504983 | 1.48995E-10 | 3.93394E-09 | - |
| *NDL2* | 449.9780222 | 223.3947371 | 0.496456996 | -1.010259341 | 1.53094E-10 | 4.03806E-09 | K18266 |
| *Os04g0338000* | 31.49862149 | 0.860580146 | 0.0273212 | -5.193835325 | 1.66778E-10 | 4.38117E-09 | - |
| *ACA7* | 415.2136878 | 179.6727113 | 0.432723478 | -1.208482695 | 1.73573E-10 | 4.54586E-09 | K01674 |
| *WRKY4* | 195.7352961 | 76.93780805 | 0.393070691 | -1.347139301 | 1.75971E-10 | 4.60402E-09 | - |
| *MYB20* | 733.7773591 | 350.0145999 | 0.477003815 | -1.067927289 | 1.81301E-10 | 4.73391E-09 | K09422 |
| *CSLA9* | 327.743838 | 151.8451014 | 0.463304214 | -1.109968289 | 2.00986E-10 | 5.2321E-09 | K13680 |
| *PAT22* | 466.3528852 | 200.5817647 | 0.430107267 | -1.21723159 | 2.06974E-10 | 5.38258E-09 | K20027 |
| *uncharacterized protein_09482* | 39.07529607 | 3.057192241 | 0.078238492 | -3.675977625 | 2.36068E-10 | 6.10857E-09 | - |
| *At1g21890* | 53.27888729 | 7.608519771 | 0.142805531 | -2.807876241 | 2.60401E-10 | 6.71142E-09 | - |
| *PAD4* | 133.3675136 | 44.32747604 | 0.332370866 | -1.589134165 | 2.76449E-10 | 7.10384E-09 | - |
| *AGD12* | 82.30283584 | 19.70962461 | 0.239476859 | -2.062041842 | 2.83303E-10 | 7.27276E-09 | K12486 |
| *ALDH2B4* | 27.25842433 | 0.573720097 | 0.021047442 | -5.570211304 | 2.98004E-10 | 7.61996E-09 | K12355 |
| *uncharacterized protein_15657* | 137.1707531 | 46.76687866 | 0.340939141 | -1.552413862 | 3.05205E-10 | 7.79641E-09 | - |
| *PCR6* | 257.4099423 | 112.5590784 | 0.437275567 | -1.193385357 | 3.059E-10 | 7.80646E-09 | - |
| *ATL6* | 199.9884091 | 80.37672749 | 0.40190693 | -1.315066642 | 3.2496E-10 | 8.26694E-09 | K10664 |
| *CRK15* | 84.41352824 | 20.82655141 | 0.246720542 | -2.019050257 | 3.33887E-10 | 8.46404E-09 | - |
| *CRK8* | 49.94727809 | 1.988803716 | 0.03981806 | -4.650433257 | 3.33952E-10 | 8.46404E-09 | - |
| *AAP2* | 63.82304225 | 11.2191229 | 0.175784834 | -2.508117488 | 3.60718E-10 | 9.10682E-09 | - |
| *uncharacterized protein_21363* | 106.875638 | 31.5972848 | 0.295645344 | -1.758060536 | 3.84776E-10 | 9.68588E-09 | - |
| *SWEET3B* | 86.39944738 | 18.41093332 | 0.213090869 | -2.23045932 | 3.94365E-10 | 9.89844E-09 | K15382 |
| *CYP720B2* | 177.8544885 | 68.929487 | 0.387561133 | -1.3675042 | 4.11181E-10 | 1.03006E-08 | - |
| *nep2* | 42.62870213 | 4.400002255 | 0.103216895 | -3.276248964 | 4.218E-10 | 1.05462E-08 | - |
| *CYP86B1* | 109.4308869 | 27.71235935 | 0.253240745 | -1.981418549 | 4.82895E-10 | 1.19699E-08 | K15402 |
| *LOX1.1* | 41.34477188 | 4.259948773 | 0.103034763 | -3.278796923 | 4.85398E-10 | 1.20204E-08 | K15718 |
| *TCM_000168* | 32.92857147 | 1.951537236 | 0.059265773 | -4.07665703 | 4.94149E-10 | 1.22255E-08 | K08241 |
| *ag1* | 31.51308365 | 1.818261818 | 0.057698632 | -4.115319075 | 5.3954E-10 | 1.32977E-08 | K12742 |
| *At5g63930* | 39.11299976 | 3.420844605 | 0.087460553 | -3.515223713 | 5.47278E-10 | 1.34756E-08 | - |
| *DTX40* | 119.4226873 | 38.23568434 | 0.320171026 | -1.643085338 | 6.24759E-10 | 1.52387E-08 | K03327 |
| *CYP92C6* | 145.3407346 | 52.54015437 | 0.361496414 | -1.467946759 | 6.28807E-10 | 1.53227E-08 | - |
| *ASOL* | 239.0825137 | 103.1244261 | 0.431334038 | -1.213122525 | 6.9755E-10 | 1.68713E-08 | - |
| *PDR17* | 59.1074481 | 5.654723181 | 0.095668539 | -3.385811629 | 7.02209E-10 | 1.69682E-08 | - |
| *At3g47110* | 36.3752117 | 2.844865153 | 0.078208896 | -3.676523469 | 7.12159E-10 | 1.71766E-08 | - |
| *At2g14510* | 37.50434287 | 3.324835666 | 0.088652018 | -3.495702723 | 7.55119E-10 | 1.81621E-08 | - |
| *PAP4* | 107.5421358 | 33.67301084 | 0.313114581 | -1.6752374 | 7.81004E-10 | 1.87499E-08 | K14379 |
| *CRK2* | 74.42622635 | 17.68464701 | 0.237613109 | -2.073313666 | 7.8701E-10 | 1.88766E-08 | - |
| *At5g48740* | 107.2022152 | 27.12274959 | 0.253005496 | -1.982759373 | 8.19088E-10 | 1.95736E-08 | - |
| *CYP720B2* | 77.68589446 | 19.38207196 | 0.249492808 | -2.002929865 | 8.94315E-10 | 2.12927E-08 | - |
| *PNC1* | 38.23473807 | 3.385837479 | 0.08855396 | -3.49729936 | 9.50263E-10 | 2.2542E-08 | K00430 |
| *Prcp* | 23.98326187 | 0.286860049 | 0.011960844 | -6.385537021 | 9.63641E-10 | 2.28176E-08 | K01285 |
| *At3g02645* | 171.1959168 | 68.39636035 | 0.399520979 | -1.323656832 | 1.0046E-09 | 2.37658E-08 | - |
| *EFR* | 170.4377816 | 67.47142645 | 0.395871302 | -1.336896611 | 1.05212E-09 | 2.48672E-08 | K13420 |
| *ATS3A* | 230.1731438 | 100.6464911 | 0.437264267 | -1.193422637 | 1.058E-09 | 2.49834E-08 | - |
| *DAD2* | 38.1098955 | 3.690747384 | 0.096844857 | -3.368180752 | 1.07328E-09 | 2.53213E-08 | - |
| *uncharacterized protein_24519* | 454.8884224 | 127.429547 | 0.280133634 | -1.835812884 | 1.08341E-09 | 2.55369E-08 | - |
| *uncharacterized protein_20873* | 175.229149 | 69.21297013 | 0.394985483 | -1.340128463 | 1.13704E-09 | 2.67767E-08 | - |
| *At4g10440* | 117.7117631 | 38.13066297 | 0.323932477 | -1.626234978 | 1.17044E-09 | 2.75134E-08 | - |
| *UCNL* | 135.0033963 | 46.7386988 | 0.346203874 | -1.530306227 | 1.18404E-09 | 2.77825E-08 | - |
| *LRK10L-2.5* | 25.65079961 | 0.610986577 | 0.023819397 | -5.391719303 | 1.29463E-09 | 3.02679E-08 | - |
| *HMG1* | 23.57847721 | 0.363652364 | 0.015423064 | -6.018766783 | 1.3132E-09 | 3.06468E-08 | K00021 |
| *EPHX4* | 181.0086444 | 70.71852557 | 0.390691427 | -1.355898496 | 1.35189E-09 | 3.14931E-08 | - |
| *WRKY35* | 122.839371 | 41.01505395 | 0.333891761 | -1.582547599 | 1.39766E-09 | 3.25008E-08 | - |
| *HPCA1* | 108.0032189 | 31.38719285 | 0.290613494 | -1.7828264 | 1.43713E-09 | 3.3359E-08 | - |
| *At1g32860* | 83.69523041 | 21.7616895 | 0.260011107 | -1.943354843 | 1.51391E-09 | 3.51097E-08 | - |
| *uncharacterized protein_01239* | 110.4529518 | 35.16381917 | 0.318360158 | -1.651268299 | 1.58733E-09 | 3.67467E-08 | - |
| *ANT* | 55.41835575 | 9.560057008 | 0.172507049 | -2.535272781 | 1.75349E-09 | 4.04128E-08 | K05863 |
| *At3g15810* | 29.96883827 | 1.660158479 | 0.055396157 | -4.174070286 | 1.82155E-09 | 4.19443E-08 | - |
| *BLH2* | 219.6292065 | 94.52882885 | 0.430401905 | -1.216243632 | 1.94747E-09 | 4.46455E-08 | - |
| *uncharacterized protein_35949* | 292.9578084 | 140.7286671 | 0.480371791 | -1.057776661 | 2.0639E-09 | 4.72728E-08 | - |
| *ANN1* | 147.6249692 | 53.74415184 | 0.364058683 | -1.457757077 | 2.08836E-09 | 4.77469E-08 | K17095 |
| *DIR21* | 64.31289755 | 7.361185558 | 0.114458932 | -3.127098046 | 2.09012E-09 | 4.77469E-08 | - |
| *At1g07700* | 337.5668951 | 165.1998346 | 0.489383992 | -1.030961185 | 2.1761E-09 | 4.96236E-08 | - |
| *At3g47110* | 32.54821267 | 2.273404411 | 0.069847289 | -3.839652073 | 2.22271E-09 | 5.05975E-08 | - |
| *JOX2* | 28.22692532 | 1.301024824 | 0.046091624 | -4.439351594 | 2.26676E-09 | 5.15549E-08 | K05278 |
| *L6* | 33.7613823 | 2.332146869 | 0.069077351 | -3.855643422 | 2.28051E-09 | 5.17768E-08 | - |
| *At5g42610* | 28.28167357 | 1.221973155 | 0.043207243 | -4.532582998 | 2.28427E-09 | 5.18167E-08 | - |
| *At1g67720* | 117.7184781 | 38.81280558 | 0.329708693 | -1.600736168 | 2.32951E-09 | 5.27046E-08 | - |
| *CESA3* | 280.5288173 | 131.4759027 | 0.468671647 | -1.093350577 | 2.3473E-09 | 5.30208E-08 | K10999 |
| *LRK10L-1.2* | 178.9541811 | 73.83989823 | 0.412619017 | -1.277117782 | 2.41347E-09 | 5.44616E-08 | - |
| *ag1* | 24.77253792 | 0.687778892 | 0.027763764 | -5.170653027 | 2.47631E-09 | 5.58311E-08 | K18113 |
| *RUN1* | 57.71040677 | 11.00562904 | 0.190704409 | -2.390589893 | 2.50415E-09 | 5.63609E-08 | - |
| *HCT* | 143.9340262 | 53.31155284 | 0.370388811 | -1.432887577 | 2.82578E-09 | 6.32702E-08 | K13065 |
| *LRK10L-1.2* | 36.81770004 | 3.537162755 | 0.096072344 | -3.379734998 | 2.93618E-09 | 6.56287E-08 | - |
| *HIDM* | 137.3148567 | 48.56830659 | 0.353700304 | -1.499400635 | 2.96206E-09 | 6.60933E-08 | - |
| *BACOVA_02659* | 66.07303939 | 14.14979281 | 0.214153805 | -2.223280783 | 3.09917E-09 | 6.89745E-08 | K05349 |
| *LTPG15* | 75.30655628 | 18.34424604 | 0.243594276 | -2.037447863 | 3.47214E-09 | 7.70108E-08 | - |
| *uncharacterized protein_39065* | 82.4261282 | 16.9822194 | 0.206029566 | -2.279076712 | 3.58275E-09 | 7.93963E-08 | - |
| *PXC3* | 179.9633341 | 74.52775206 | 0.414127425 | -1.27185335 | 3.70643E-09 | 8.19969E-08 | - |
| *CYP71AU50* | 138.6545674 | 52.02177559 | 0.375189772 | -1.414307594 | 3.72338E-09 | 8.23017E-08 | - |
| *uncharacterized protein_22790* | 60.32025185 | 12.26486868 | 0.203329202 | -2.298110665 | 3.73185E-09 | 8.24186E-08 | - |
| *SMXL3* | 63.37708762 | 13.52862702 | 0.213462428 | -2.227945932 | 3.76198E-09 | 8.29427E-08 | - |
| *JAL3* | 219.6763558 | 97.40073208 | 0.443382865 | -1.173375077 | 3.86924E-09 | 8.52351E-08 | - |
| *AAPC* | 328.6640842 | 163.9946703 | 0.498973506 | -1.002964881 | 3.99712E-09 | 8.79028E-08 | K01792 |
| *LRL1* | 293.844549 | 142.161899 | 0.483799681 | -1.047518278 | 4.04856E-09 | 8.89585E-08 | - |
| *CBP3* | 120.0864545 | 41.26464752 | 0.343624497 | -1.541095205 | 4.07122E-09 | 8.93808E-08 | K16298 |
| *PIP1-5* | 143.952621 | 53.69104566 | 0.372977201 | -1.422840649 | 4.10107E-09 | 8.99599E-08 | K09872 |
| *LTPG5* | 261.363051 | 76.54945115 | 0.292885512 | -1.771591263 | 4.1071E-09 | 9.0016E-08 | - |
| *GAT1* | 83.46760633 | 23.21627397 | 0.278147116 | -1.846079948 | 4.13992E-09 | 9.06586E-08 | - |
| *WRKY6* | 325.0382191 | 134.6357836 | 0.414215239 | -1.271547464 | 4.86474E-09 | 1.06083E-07 | - |
| *At5g48740* | 84.44953344 | 23.20050845 | 0.274726307 | -1.86393303 | 5.04501E-09 | 1.0983E-07 | - |
| *uncharacterized protein_47569* | 155.2772687 | 61.4519833 | 0.395756467 | -1.337315169 | 5.21189E-09 | 1.13367E-07 | - |
| *uncharacterized protein_28826* | 31.27652807 | 2.122079135 | 0.067848935 | -3.881530009 | 5.49262E-09 | 1.19274E-07 | - |
| *GDI1* | 105.2845398 | 33.64033725 | 0.319518301 | -1.646029529 | 5.50838E-09 | 1.19516E-07 | K12462 |
| *At5g48740* | 27.90684543 | 1.415083619 | 0.050707402 | -4.301659841 | 5.70175E-09 | 1.23505E-07 | - |
| *IP5P4* | 97.50075197 | 29.31603472 | 0.30067496 | -1.733723368 | 6.29833E-09 | 1.35973E-07 | K20279 |
| *ABCG36* | 19.44674437 | 0 | 0 | -Inf | 6.3136E-09 | 1.36189E-07 | - |
| *GLR2.8* | 154.4934579 | 49.44239366 | 0.320029044 | -1.643725252 | 6.34071E-09 | 1.36546E-07 | K05387 |
| *PCS1* | 157.5823146 | 62.28880313 | 0.395277879 | -1.339060874 | 6.57507E-09 | 1.41358E-07 | - |
| *PBS1* | 142.572472 | 54.61143587 | 0.38304334 | -1.384420458 | 6.64029E-09 | 1.42643E-07 | K04733 |
| *uncharacterized protein_19914* | 21.78026581 | 0.286860049 | 0.01317064 | -6.246530695 | 6.74856E-09 | 1.44728E-07 | - |
| *UPM1* | 107.3719907 | 35.12317576 | 0.327116742 | -1.612122496 | 7.10838E-09 | 1.51942E-07 | - |
| *JOX4* | 55.10019195 | 10.56518437 | 0.191744965 | -2.382739402 | 7.28418E-09 | 1.55572E-07 | K05278 |
| *CYP71AU50* | 19.34152875 | 0 | 0 | -Inf | 7.96705E-09 | 1.69181E-07 | - |
| *At5g48740* | 23.52734354 | 0.573720097 | 0.024385248 | -5.35784757 | 8.29856E-09 | 1.75645E-07 | - |
| *AMO* | 40.58545234 | 4.780611887 | 0.117791268 | -3.085695502 | 8.36834E-09 | 1.76884E-07 | K00276 |
| *ANMT* | 19.22118466 | 0 | 0 | -Inf | 8.37075E-09 | 1.76884E-07 | K13066 |
| *CXE15* | 19.15972142 | 0 | 0 | -Inf | 8.58573E-09 | 1.81279E-07 | - |
| *CPK17* | 184.3376712 | 77.4302913 | 0.420045945 | -1.251380955 | 9.31983E-09 | 1.96299E-07 | K13412 |
| *uncharacterized protein_28087* | 184.2081819 | 79.89894215 | 0.433742635 | -1.205088835 | 9.50171E-09 | 1.99806E-07 | - |
| *uncharacterized protein_39470* | 27.87585673 | 1.548359038 | 0.055544805 | -4.170204194 | 1.00833E-08 | 2.10527E-07 | - |
| *uncharacterized protein_22163* | 86.38137064 | 25.24236915 | 0.292220058 | -1.774872886 | 1.15287E-08 | 2.38942E-07 | - |
| *R65* | 349.159091 | 170.7732475 | 0.489098671 | -1.03180255 | 1.21738E-08 | 2.51308E-07 | K00430 |
| *uncharacterized protein_47809* | 18.73944241 | 0 | 0 | -Inf | 1.23402E-08 | 2.54337E-07 | - |
| *PILS7* | 328.5678694 | 153.4522036 | 0.467033505 | -1.098402043 | 1.27726E-08 | 2.62833E-07 | K07088 |
| *CYP75A5* | 229.2976783 | 107.6676612 | 0.469554084 | -1.090636756 | 1.51231E-08 | 3.09481E-07 | K13083 |
| *ROQ1* | 77.24495632 | 20.68317097 | 0.267760796 | -1.900983352 | 1.51964E-08 | 3.10735E-07 | - |
| *ELIP1* | 38.38762107 | 5.101312295 | 0.132889514 | -2.911700829 | 1.55255E-08 | 3.16965E-07 | - |
| *At4g01130* | 350.0944658 | 154.7374872 | 0.441987813 | -1.177921506 | 1.57461E-08 | 3.21215E-07 | - |
| *At3g47110* | 33.2625955 | 3.173510391 | 0.09540778 | -3.389749276 | 1.63132E-08 | 3.32001E-07 | K13420 |
| *PT30* | 29.15617241 | 0.648253058 | 0.022233819 | -5.491100421 | 1.63967E-08 | 3.33439E-07 | K12742 |
| *TCM_034089* | 20.84068915 | 0.324126529 | 0.015552582 | -6.006702068 | 1.65586E-08 | 3.36466E-07 | K08241 |
| *SRD5A2* | 169.7489223 | 72.65973524 | 0.428042395 | -1.224174401 | 1.66587E-08 | 3.38235E-07 | K10258 |
| *FDH* | 77.99992566 | 21.00051943 | 0.269237685 | -1.893047737 | 1.69411E-08 | 3.43164E-07 | K15397 |
| *BOR2* | 124.3380134 | 45.43201348 | 0.36539118 | -1.452486284 | 1.71119E-08 | 3.46353E-07 | - |
| *cnot9* | 163.4362544 | 69.59124623 | 0.425800545 | -1.231750298 | 1.79613E-08 | 3.62132E-07 | K12606 |
| *At3g47110* | 47.04140924 | 8.128016119 | 0.172784282 | -2.532956112 | 2.09145E-08 | 4.18096E-07 | - |
| *AT9* | 161.7294628 | 68.60081679 | 0.424170189 | -1.237284864 | 2.12908E-08 | 4.25291E-07 | - |
| *GRIK1* | 43.40263211 | 6.861998421 | 0.158100974 | -2.661081837 | 2.13786E-08 | 4.26716E-07 | K07359 |
| *AAP2* | 53.95623273 | 11.27779118 | 0.209017394 | -2.258305087 | 2.17883E-08 | 4.34226E-07 | - |
| *At4g01130* | 97.32529375 | 31.83446479 | 0.327093436 | -1.612225285 | 2.2293E-08 | 4.43261E-07 | - |
| *At1g61300* | 53.27888729 | 10.11346789 | 0.189821305 | -2.397286173 | 2.31499E-08 | 4.59595E-07 | K13457 |
| *At4g02290* | 58.21022576 | 12.88941138 | 0.221428644 | -2.175086231 | 2.45731E-08 | 4.8636E-07 | K01179 |
| *ag4* | 52.59431271 | 7.532820044 | 0.143224993 | -2.803644832 | 2.46501E-08 | 4.87512E-07 | - |
| *uncharacterized protein_49166* | 98.56133903 | 31.7825496 | 0.322464669 | -1.632786994 | 2.48136E-08 | 4.90372E-07 | - |
| *CSPL7* | 135.3552795 | 52.32224096 | 0.386554859 | -1.371254923 | 2.49632E-08 | 4.92952E-07 | - |
| *DIR1* | 100.4148167 | 25.91440674 | 0.258073535 | -1.95414589 | 2.52151E-08 | 4.97169E-07 | - |
| *YSL3* | 87.87049803 | 26.57053045 | 0.302382837 | -1.725551841 | 2.71861E-08 | 5.35218E-07 | - |
| *CRK2* | 33.59558734 | 3.499896274 | 0.104177261 | -3.262887682 | 2.76349E-08 | 5.43151E-07 | - |
| *LTPG5* | 100.4747297 | 24.49932312 | 0.243835671 | -2.036018901 | 2.82638E-08 | 5.54329E-07 | - |
| *NAKR2* | 212.5191418 | 75.39064076 | 0.354747531 | -1.495135453 | 2.95171E-08 | 5.78035E-07 | - |
| *SDR2a* | 38.78119225 | 0.687778892 | 0.017734857 | -5.817268506 | 3.02172E-08 | 5.89076E-07 | - |
| *At1g48100* | 270.4321672 | 131.7481632 | 0.487176376 | -1.037483918 | 3.11535E-08 | 6.05963E-07 | - |
| *HSL1* | 44.24252381 | 7.666095463 | 0.173274371 | -2.528869818 | 3.29379E-08 | 6.38755E-07 | - |
| *MAPKKK17* | 33.31631156 | 3.574429235 | 0.107287664 | -3.220443894 | 3.41235E-08 | 6.60758E-07 | - |
| *HSL1* | 34.27138257 | 3.977607433 | 0.116062065 | -3.107031592 | 3.42891E-08 | 6.63468E-07 | - |
| *MYB5* | 93.52190213 | 30.77171178 | 0.329032142 | -1.603699572 | 3.58329E-08 | 6.90249E-07 | K09422 |
| *HPCA1* | 98.62383445 | 33.77353849 | 0.342448037 | -1.546043005 | 3.59933E-08 | 6.92825E-07 | - |
| *NEED* | 24.94866244 | 1.377817139 | 0.055226092 | -4.178506137 | 3.63507E-08 | 6.98666E-07 | - |
| *UNI* | 171.0152938 | 74.18895184 | 0.43381472 | -1.204849087 | 3.77729E-08 | 7.24926E-07 | K13459 |
| *AAPC* | 243.8523381 | 117.8862989 | 0.483433129 | -1.048611752 | 3.90695E-08 | 7.48149E-07 | K01792 |
| *CRK2* | 65.46165567 | 17.24646168 | 0.26345899 | -1.924349686 | 4.08035E-08 | 7.79626E-07 | - |
| *CEPR1* | 178.2203851 | 79.11068481 | 0.443892458 | -1.171717898 | 4.17609E-08 | 7.95574E-07 | - |
| *NFP* | 106.2835153 | 37.51967639 | 0.353015012 | -1.502198561 | 4.20193E-08 | 7.98736E-07 | - |
| *TPK1* | 80.32156732 | 23.68385513 | 0.29486296 | -1.761883487 | 4.20609E-08 | 7.98941E-07 | K00949 |
| *ROQ1* | 73.66912332 | 7.23582999 | 0.098220661 | -3.347829658 | 4.33926E-08 | 8.2303E-07 | - |
| *AMO* | 54.20813439 | 11.54434202 | 0.212963278 | -2.231323409 | 4.36408E-08 | 8.26529E-07 | K00276 |
| *LRK10* | 39.64056262 | 5.54292374 | 0.139829593 | -2.838258375 | 4.50322E-08 | 8.52258E-07 | - |
| *UGT84A23* | 263.5370399 | 112.4020684 | 0.42651336 | -1.229337162 | 4.50923E-08 | 8.52773E-07 | K13691 |
| *CRRSP41* | 38.80015289 | 5.332856006 | 0.137444201 | -2.863082058 | 4.5893E-08 | 8.67284E-07 | - |
| *XTH2* | 34.11761567 | 3.442320583 | 0.100895696 | -3.30906346 | 4.69437E-08 | 8.85205E-07 | K08235 |
| *LDHB* | 89.52689163 | 28.85746601 | 0.322332938 | -1.633376475 | 4.77703E-08 | 8.97529E-07 | K00016 |
| *LECRK44* | 257.0581381 | 126.8217882 | 0.493358386 | -1.019292062 | 4.80138E-08 | 9.00146E-07 | - |
| *CRK2* | 121.0339266 | 45.653353 | 0.377194678 | -1.406618773 | 5.05835E-08 | 9.45587E-07 | - |
| *CCL5* | 114.3987448 | 35.99391014 | 0.314635534 | -1.668246482 | 5.13641E-08 | 9.58796E-07 | K10526 |
| *UGT73C10* | 75.05037374 | 21.70294704 | 0.289178401 | -1.789968297 | 5.32267E-08 | 9.9285E-07 | K13496 |
| *XTH9* | 112.436071 | 40.60512267 | 0.361139644 | -1.469371294 | 5.33555E-08 | 9.94537E-07 | K08235 |
| *TBL7* | 174.0834913 | 77.4573044 | 0.444943422 | -1.168306196 | 5.91566E-08 | 1.09636E-06 | - |
| *ROQ1* | 41.80688717 | 6.824731941 | 0.163244202 | -2.614896342 | 6.02293E-08 | 1.11465E-06 | - |
| *CHIT1* | 22.75238137 | 1.014164776 | 0.044574006 | -4.487653575 | 6.25912E-08 | 1.15506E-06 | - |
| *XA21* | 55.0027235 | 12.31343193 | 0.223869495 | -2.159270139 | 6.4651E-08 | 1.19138E-06 | - |
| *At1g56140* | 230.7067515 | 114.8480999 | 0.497809879 | -1.006333235 | 7.06576E-08 | 1.29288E-06 | - |
| *CYP750A1* | 57.72140264 | 13.53314573 | 0.234456287 | -2.09260913 | 7.14724E-08 | 1.30687E-06 | - |
| *CRK2* | 61.92854287 | 15.78281481 | 0.254855259 | -1.972249973 | 7.34914E-08 | 1.34095E-06 | - |
| *ROQ1* | 122.6115948 | 46.70821038 | 0.380944481 | -1.39234734 | 7.49193E-08 | 1.36337E-06 | - |
| *T5AT* | 33.51346497 | 3.136243911 | 0.093581607 | -3.417631185 | 7.49992E-08 | 1.36366E-06 | K19861 |
| *PMEU1* | 226.5038922 | 111.6441511 | 0.492901689 | -1.020628169 | 7.50756E-08 | 1.36409E-06 | K01051 |
| *GATL3* | 43.88592456 | 7.950696157 | 0.181167339 | -2.464605209 | 7.76907E-08 | 1.40668E-06 | - |
| *GGPS1* | 140.7563322 | 57.16159531 | 0.406103189 | -1.30008174 | 7.83463E-08 | 1.41657E-06 | K13789 |
| *At5g63180* | 19.14164467 | 0.324126529 | 0.016933055 | -5.884013877 | 7.95579E-08 | 1.43348E-06 | K01728 |
| *HIP1* | 72.37367917 | 18.61531558 | 0.257211127 | -1.95897504 | 8.1611E-08 | 1.46843E-06 | K10635 |
| *At3g47570* | 16.85409213 | 0 | 0 | -Inf | 8.39177E-08 | 1.5068E-06 | - |
| *RAV1* | 51.52715894 | 10.70071914 | 0.207671437 | -2.267625293 | 8.53237E-08 | 1.52993E-06 | K09287 |
| *At1g67720* | 20.80815024 | 0.573720097 | 0.027571893 | -5.180657855 | 9.2591E-08 | 1.65224E-06 | - |
| *QKY* | 221.9610255 | 107.1223428 | 0.482617804 | -1.051046956 | 1.07516E-07 | 1.90026E-06 | - |
| *uncharacterized protein_38429* | 22.22636873 | 0.972379586 | 0.043748918 | -4.514608848 | 1.07854E-07 | 1.90235E-06 | - |
| *CCL9* | 898.0694483 | 382.560982 | 0.425981513 | -1.231137276 | 1.0952E-07 | 1.92909E-06 | - |
| *SRG1* | 220.7876278 | 109.3526718 | 0.495284418 | -1.01367086 | 1.09842E-07 | 1.93346E-06 | - |
| *Prcp* | 72.89194465 | 21.35396761 | 0.292953737 | -1.771255239 | 1.10537E-07 | 1.94437E-06 | K01285 |
| *HPT1* | 33.56304843 | 4.224941647 | 0.125880748 | -2.989870437 | 1.11059E-07 | 1.95223E-06 | K09833 |
| *SRG1* | 170.0009761 | 76.54590165 | 0.450267424 | -1.151145988 | 1.18271E-07 | 2.07198E-06 | - |
| *CRPK1* | 39.78776281 | 6.728723002 | 0.169115389 | -2.563920145 | 1.1961E-07 | 2.09403E-06 | - |
| *XA21* | 23.45968333 | 1.224232509 | 0.052184528 | -4.26023405 | 1.22269E-07 | 2.13913E-06 | - |
| *FAR1* | 77.55861778 | 15.49146103 | 0.199738746 | -2.323813878 | 1.22769E-07 | 2.14642E-06 | K13356 |
| *ROQ1* | 57.81318828 | 13.87874824 | 0.240061976 | -2.058521183 | 1.29405E-07 | 2.25941E-06 | K19613 |
| *uncharacterized protein_12090* | 29.43404626 | 2.888909697 | 0.098148575 | -3.348888868 | 1.35157E-07 | 2.35665E-06 | K06995 |
| *TPS-mISO1* | 109.8260738 | 36.9234127 | 0.336198968 | -1.572612797 | 1.3562E-07 | 2.36156E-06 | K04120 |
| *HPCA1* | 87.39738687 | 30.02178929 | 0.343509004 | -1.541580181 | 1.40729E-07 | 2.44724E-06 | - |
| *LAC17* | 234.1172382 | 116.9985323 | 0.499743347 | -1.000740734 | 1.41318E-07 | 2.45418E-06 | K05909 |
| *MO2* | 55.58245222 | 13.16949337 | 0.23693617 | -2.077429639 | 1.41445E-07 | 2.45474E-06 | - |
| *BHLH80* | 218.6179853 | 106.7134549 | 0.48812752 | -1.034670005 | 1.42515E-07 | 2.47166E-06 | - |
| *UGT89B2* | 120.3438175 | 40.36451656 | 0.335409973 | -1.576002509 | 1.48077E-07 | 2.56126E-06 | - |
| *R9* | 105.8632363 | 39.78290159 | 0.375795252 | -1.411981255 | 1.54981E-07 | 2.67533E-06 | K00430 |
| *uncharacterized protein_36165* | 26.85777228 | 2.238397285 | 0.083342627 | -3.584801622 | 1.59753E-07 | 2.75404E-06 | - |
| *COMT1* | 20.18297066 | 0.648253058 | 0.032118813 | -4.960437619 | 1.61498E-07 | 2.78043E-06 | - |
| *XA21* | 53.19263232 | 12.03449173 | 0.226243583 | -2.144051221 | 1.62755E-07 | 2.8002E-06 | - |
| *LTPG5* | 130.1423005 | 54.21612833 | 0.416591132 | -1.263295965 | 1.68301E-07 | 2.88987E-06 | - |
| *At5g48740* | 16.07086477 | 0 | 0 | -Inf | 1.698E-07 | 2.91369E-06 | - |
| *rnf126-b* | 128.9241144 | 52.7129806 | 0.408868278 | -1.29029196 | 1.70809E-07 | 2.92905E-06 | - |
| *HSL1* | 44.18689166 | 8.186758577 | 0.185275729 | -2.432254191 | 1.73917E-07 | 2.97841E-06 | - |
| *uncharacterized protein_15149* | 164.8757984 | 74.72649804 | 0.453229029 | -1.141687826 | 1.75954E-07 | 3.01132E-06 | - |
| *uncharacterized protein_15845* | 62.80717431 | 16.36222038 | 0.260515149 | -1.940560826 | 1.78953E-07 | 3.05658E-06 | - |
| *CYP720B2* | 88.18629704 | 29.55316475 | 0.33512196 | -1.577241866 | 1.82057E-07 | 3.10551E-06 | - |
| *CRK2* | 54.50357082 | 12.805841 | 0.234954166 | -2.089548749 | 1.91087E-07 | 3.25312E-06 | - |
| *RGI1* | 94.2708921 | 32.7854176 | 0.347778799 | -1.523758109 | 1.93804E-07 | 3.29505E-06 | - |
| *CML2* | 48.71824437 | 9.782563302 | 0.200798765 | -2.316177699 | 1.98105E-07 | 3.36156E-06 | K13448 |
| *ACO* | 26.52891303 | 2.350196726 | 0.088590012 | -3.496712136 | 2.00429E-07 | 3.39434E-06 | K05933 |
| *UBA2C* | 73.3311843 | 21.39242584 | 0.291723447 | -1.77732675 | 2.03944E-07 | 3.4516E-06 | K12741 |
| *FEI1* | 38.07425617 | 6.321026095 | 0.166018374 | -2.590585176 | 2.04989E-07 | 3.46703E-06 | - |
| *SCP26* | 32.75156304 | 4.227201001 | 0.12906868 | -2.953789141 | 2.12406E-07 | 3.57846E-06 | K16297 |
| *UGT85A23* | 38.13726962 | 5.987887127 | 0.157008805 | -2.671082628 | 2.14673E-07 | 3.61432E-06 | - |
| *BGLU41* | 220.845255 | 109.6237906 | 0.49638282 | -1.010474909 | 2.16434E-07 | 3.63924E-06 | K01188 |
| *DAO* | 31.87758222 | 2.545566545 | 0.079854442 | -3.646483526 | 2.17592E-07 | 3.65633E-06 | - |
| *At1g62810* | 219.0732373 | 109.065886 | 0.497851254 | -1.00621333 | 2.32943E-07 | 3.90922E-06 | K00276 |
| *At1g80120* | 50.26440584 | 11.71145779 | 0.23299704 | -2.101616466 | 2.35342E-07 | 3.94437E-06 | - |
| *WRI1* | 66.60628119 | 18.48429952 | 0.277515862 | -1.849357862 | 2.35909E-07 | 3.95133E-06 | K09285 |
| *At2g01630* | 199.0042616 | 96.22054411 | 0.483509968 | -1.048382463 | 2.36099E-07 | 3.95195E-06 | K19891 |
| *ag1* | 36.80670417 | 5.677291746 | 0.154246132 | -2.696693784 | 2.48288E-07 | 4.14261E-06 | K16086 |
| *PLR_Tp2* | 80.51576859 | 25.62637992 | 0.318277778 | -1.651641662 | 2.50786E-07 | 4.1816E-06 | - |
| *RUN1* | 210.3973881 | 98.45792299 | 0.467961717 | -1.095537584 | 2.52703E-07 | 4.21085E-06 | - |
| *At1g74360* | 112.4379871 | 43.92993336 | 0.390703662 | -1.355853315 | 2.55426E-07 | 4.24803E-06 | - |
| *ABCG36* | 187.475963 | 79.92146152 | 0.426302446 | -1.230050763 | 2.69802E-07 | 4.47851E-06 | - |
| *At1g67720* | 22.55781036 | 1.184706675 | 0.052518691 | -4.251025228 | 2.76681E-07 | 4.58975E-06 | - |
| *DBR* | 113.6665028 | 38.29663696 | 0.336921045 | -1.569517547 | 2.88539E-07 | 4.77424E-06 | - |
| *At5g54860* | 218.9802673 | 108.5633969 | 0.495767944 | -1.012263105 | 2.91661E-07 | 4.81975E-06 | - |
| *At2g01630* | 188.7781222 | 90.02487283 | 0.476881917 | -1.068296016 | 3.11758E-07 | 5.14529E-06 | K19891 |
| *GCH1* | 568.0649754 | 263.8820547 | 0.464527943 | -1.106162712 | 3.19967E-07 | 5.27407E-06 | K01495 |
| *WRKY71* | 23.51959636 | 1.662417833 | 0.070682243 | -3.82250836 | 3.36518E-07 | 5.5223E-06 | - |
| *WAV3* | 198.9131423 | 96.95800307 | 0.487438899 | -1.036706708 | 3.38108E-07 | 5.54488E-06 | - |
| *At5g48740* | 154.1622339 | 54.8756774 | 0.355960575 | -1.490210633 | 3.46975E-07 | 5.67953E-06 | - |
| *TPS-LAS2* | 112.9490195 | 44.61773723 | 0.39502545 | -1.339982493 | 3.55936E-07 | 5.81518E-06 | K16086 |
| *CSE* | 63.86141225 | 17.65420779 | 0.276445621 | -1.854932374 | 3.72168E-07 | 6.06129E-06 | - |
| *CCR1* | 165.3558421 | 76.20149089 | 0.460833376 | -1.117682888 | 3.7733E-07 | 6.13765E-06 | - |
| *TMK1* | 524.0504493 | 256.0809671 | 0.488657089 | -1.033105673 | 3.77963E-07 | 6.14408E-06 | - |
| *NIR1* | 63.54789908 | 17.50062316 | 0.275392632 | -1.860438136 | 3.87359E-07 | 6.27716E-06 | K00366 |
| *AATL1* | 113.0674476 | 44.4178487 | 0.392843826 | -1.347972208 | 3.91855E-07 | 6.34604E-06 | - |
| *PAD4* | 37.03293019 | 6.134693694 | 0.165655098 | -2.593745489 | 3.98583E-07 | 6.44294E-06 | - |
| *FRO2* | 259.8750174 | 127.8234644 | 0.49186515 | -1.023665255 | 4.03867E-07 | 6.52022E-06 | - |
| *XA21* | 161.186706 | 74.3978528 | 0.461563206 | -1.115399872 | 4.21587E-07 | 6.7936E-06 | - |
| *Os04g0590900* | 189.6765288 | 90.42924277 | 0.476755049 | -1.068679878 | 4.28518E-07 | 6.88817E-06 | - |
| *ROQ1* | 15.14420006 | 0 | 0 | -Inf | 4.4148E-07 | 7.08773E-06 | - |
| *OPT4* | 31.72573141 | 3.882765261 | 0.122385366 | -3.03049703 | 4.53306E-07 | 7.25066E-06 | - |
| *At1g67720* | 52.61755423 | 12.77535259 | 0.242796397 | -2.042181082 | 4.58371E-07 | 7.32715E-06 | - |
| *HSR201* | 23.19228733 | 1.434300243 | 0.061843846 | -4.015226151 | 4.69123E-07 | 7.48518E-06 | K19861 |
| *SDR2a* | 28.61466541 | 2.868600486 | 0.100249311 | -3.318335777 | 5.1939E-07 | 8.24158E-06 | - |
| *CKX3* | 62.06666714 | 16.54629343 | 0.266589044 | -1.907310606 | 5.25318E-07 | 8.32124E-06 | K00279 |
| *AAP3* | 107.4787565 | 42.41432209 | 0.394629818 | -1.341428126 | 5.26114E-07 | 8.3279E-06 | - |
| *CYP76T24* | 46.54004004 | 10.81812988 | 0.232447799 | -2.105021329 | 5.27546E-07 | 8.34546E-06 | - |
| *PBL27* | 53.55727917 | 10.83399456 | 0.202287994 | -2.305517399 | 5.30883E-07 | 8.39313E-06 | - |
| *PMLN* | 39.77381867 | 7.593821857 | 0.190925139 | -2.388921021 | 5.3604E-07 | 8.46434E-06 | - |
| *HMG1* | 135.6519657 | 45.05259559 | 0.332119003 | -1.590227823 | 5.53316E-07 | 8.71591E-06 | K00021 |
| *MYB1* | 158.4566651 | 72.650772 | 0.458489846 | -1.125038311 | 5.70167E-07 | 8.95956E-06 | K09422 |
| *TPS-Lon* | 25.16905737 | 2.273404411 | 0.09032537 | -3.468724936 | 5.72731E-07 | 8.99441E-06 | K18108 |
| *CRK2* | 43.43827144 | 9.023603391 | 0.207733943 | -2.26719113 | 5.95121E-07 | 9.32345E-06 | - |
| *At4g33300* | 34.71387092 | 5.490959345 | 0.158177674 | -2.660382113 | 6.02413E-07 | 9.43198E-06 | - |
| *PBL8* | 189.1668945 | 93.8443035 | 0.496092637 | -1.01131855 | 6.22081E-07 | 9.70476E-06 | - |
| *CRJ31* | 28.28580617 | 3.098977431 | 0.109559452 | -3.190214137 | 6.25464E-07 | 9.75168E-06 | - |
| *VEP1* | 45.41245909 | 10.38220391 | 0.228620165 | -2.128975433 | 6.32036E-07 | 9.84229E-06 | - |
| *GAPC* | 44.85390753 | 9.476486629 | 0.211274494 | -2.242809484 | 6.50225E-07 | 1.00919E-05 | K00134 |
| *At4g26790* | 56.96746549 | 14.14078037 | 0.248225548 | -2.010276483 | 6.504E-07 | 1.00919E-05 | - |
| *CYP716B2* | 37.65655956 | 6.820213232 | 0.181116207 | -2.465012446 | 7.16894E-07 | 1.10378E-05 | - |
| *LRK10L-1.2* | 60.29546011 | 16.79136829 | 0.278484786 | -1.844329583 | 7.18132E-07 | 1.10503E-05 | - |
| *FAR4* | 43.05001332 | 8.865500052 | 0.205934897 | -2.279739771 | 7.37592E-07 | 1.13228E-05 | K13356 |
| *uncharacterized protein_14829* | 186.0815002 | 91.21747513 | 0.49020174 | -1.028552491 | 7.43078E-07 | 1.14002E-05 | - |
| *GT4* | 75.49389427 | 25.77313729 | 0.341393666 | -1.550491805 | 7.53836E-07 | 1.15449E-05 | - |
| *SKOR* | 165.5505614 | 80.14059089 | 0.484085286 | -1.046666851 | 7.60456E-07 | 1.16325E-05 | - |
| *PAP16* | 164.2356425 | 76.87233673 | 0.468061229 | -1.095230826 | 7.61471E-07 | 1.16412E-05 | - |
| *LRK10L-1.2* | 50.78112111 | 12.48849254 | 0.245927862 | -2.023692903 | 7.64542E-07 | 1.16812E-05 | - |
| *T5AT* | 34.27293278 | 5.256063691 | 0.153359029 | -2.705014987 | 7.90901E-07 | 1.20626E-05 | K15400 |
| *RLK5* | 31.75052314 | 4.428231317 | 0.139469554 | -2.841977872 | 7.9207E-07 | 1.20734E-05 | - |
| *TUR2* | 14.60166088 | 0 | 0 | -Inf | 8.10011E-07 | 1.23396E-05 | - |
| *At5g22090* | 58.6175928 | 16.41301801 | 0.28000157 | -1.836493179 | 8.39902E-07 | 1.27575E-05 | - |
| *DAD2* | 38.39175367 | 6.49725347 | 0.169235652 | -2.562894568 | 8.42588E-07 | 1.27908E-05 | - |
| *TPS5* | 100.9059218 | 26.29489299 | 0.260588205 | -1.940156312 | 8.7875E-07 | 1.33085E-05 | K04120 |
| *EXLA2* | 56.160779 | 14.70432126 | 0.26182545 | -1.933322755 | 8.91053E-07 | 1.3487E-05 | - |
| *Os07g0190000* | 242.761729 | 115.3520291 | 0.475165627 | -1.073497619 | 9.22662E-07 | 1.3941E-05 | K01662 |
| *uncharacterized protein_28771* | 51.07744144 | 12.57320471 | 0.246159642 | -2.022333844 | 9.35063E-07 | 1.41202E-05 | - |
| *CYP75A5* | 121.6341662 | 52.70841269 | 0.433335586 | -1.206443377 | 9.51225E-07 | 1.43475E-05 | K13083 |
| *Os04g0338000* | 29.54081209 | 3.665919464 | 0.124096773 | -3.01046249 | 9.63339E-07 | 1.45218E-05 | - |
| *At2g25060* | 76.89558622 | 25.25145577 | 0.328386283 | -1.606534228 | 9.75354E-07 | 1.46858E-05 | - |
| *At3g47110* | 18.1679789 | 0.610986577 | 0.033629859 | -4.894113439 | 9.79593E-07 | 1.47411E-05 | K04730 |
| *At4g24290* | 180.9752216 | 89.62502235 | 0.495233666 | -1.013818704 | 9.83396E-07 | 1.47897E-05 | - |
| *At4g15970* | 84.31111261 | 30.0941121 | 0.356941228 | -1.486241546 | 1.00673E-06 | 1.51318E-05 | - |
| *At3g47570* | 21.01578149 | 1.29876547 | 0.061799532 | -4.016260267 | 1.02781E-06 | 1.5422E-05 | - |
| *ROQ1* | 62.7271163 | 18.19292076 | 0.290032793 | -1.785712063 | 1.04216E-06 | 1.56282E-05 | - |
| *MYB93* | 56.05268056 | 13.01481615 | 0.232189005 | -2.106628435 | 1.04428E-06 | 1.5651E-05 | K09422 |
| *ROQ1* | 36.20683435 | 6.258931695 | 0.17286603 | -2.532273707 | 1.05039E-06 | 1.57243E-05 | - |
| *LAX5* | 180.524468 | 90.17046343 | 0.499491645 | -1.00146755 | 1.05231E-06 | 1.57439E-05 | K13946 |
| *uncharacterized protein_50170* | 19.51079 | 0.897846626 | 0.046017953 | -4.441659366 | 1.111E-06 | 1.65646E-05 | - |
| *STP14* | 192.3607491 | 77.77693644 | 0.404328517 | -1.306400135 | 1.11954E-06 | 1.66825E-05 | - |
| *NPF5.7* | 23.05231628 | 2.063336677 | 0.089506696 | -3.481860567 | 1.13665E-06 | 1.68986E-05 | K14638 |
| *IMK3* | 33.62296147 | 5.565492306 | 0.165526535 | -2.594865586 | 1.1403E-06 | 1.69334E-05 | - |
| *PME31* | 1024.28395 | 471.6958225 | 0.460512754 | -1.118686983 | 1.14409E-06 | 1.698E-05 | K01051 |
| *BRADI_1g24377v3* | 21.74462648 | 1.33603195 | 0.061441936 | -4.024632513 | 1.15062E-06 | 1.70671E-05 | - |
| *PUB41* | 81.44641374 | 22.975792 | 0.282097037 | -1.825736581 | 1.15326E-06 | 1.70964E-05 | - |
| *At3g47110* | 23.22999102 | 1.797952606 | 0.077397904 | -3.691561699 | 1.16333E-06 | 1.72359E-05 | - |
| *PAP9* | 162.641078 | 78.3257552 | 0.481586547 | -1.054133003 | 1.18826E-06 | 1.75851E-05 | - |
| *CYP75B137* | 87.56937879 | 21.53131255 | 0.245877187 | -2.023990208 | 1.1923E-06 | 1.76349E-05 | K05280 |
| *At2g01630* | 14.00628954 | 0 | 0 | -Inf | 1.25577E-06 | 1.84997E-05 | K19891 |
| *uncharacterized protein_25517* | 201.0123901 | 93.723615 | 0.466257901 | -1.100799923 | 1.26533E-06 | 1.85984E-05 | K06995 |
| *TMKL1* | 125.4799082 | 53.97452879 | 0.43014479 | -1.217105731 | 1.27237E-06 | 1.86912E-05 | - |
| *XTH5* | 17.75286468 | 0.648253058 | 0.036515406 | -4.775350927 | 1.33784E-06 | 1.95753E-05 | K08235 |
| *uncharacterized protein_09970* | 123.4449237 | 52.91517768 | 0.428654141 | -1.222114015 | 1.34405E-06 | 1.96551E-05 | - |
| *MCM7* | 44.7547406 | 10.28510238 | 0.229810345 | -2.121484352 | 1.39424E-06 | 2.03432E-05 | K02210 |
| *uncharacterized protein_45688* | 181.713364 | 90.01245925 | 0.495354097 | -1.013467911 | 1.40271E-06 | 2.04438E-05 | - |
| *LRK10L-1.2* | 62.06666714 | 13.36602995 | 0.215349568 | -2.215247666 | 1.45904E-06 | 2.11933E-05 | - |
| *TIP1-1* | 104.5596791 | 42.65602079 | 0.407958605 | -1.293505325 | 1.48885E-06 | 2.159E-05 | K09873 |
| *MIK2* | 28.96676618 | 3.630912339 | 0.125347521 | -2.995994628 | 1.50216E-06 | 2.17587E-05 | - |
| *ag4* | 106.6283869 | 44.299222 | 0.415454301 | -1.267238301 | 1.53221E-06 | 2.21692E-05 | K16086 |
| *ORP1D* | 143.9438416 | 67.98188538 | 0.47228061 | -1.082283788 | 1.5712E-06 | 2.26575E-05 | K20456 |
| *CYP76T24* | 13.8669848 | 0 | 0 | -Inf | 1.61039E-06 | 2.31454E-05 | - |
| *COMT1* | 13.85252264 | 0 | 0 | -Inf | 1.62008E-06 | 2.32717E-05 | K13066 |
| *MFT* | 167.2282804 | 81.18988617 | 0.485503325 | -1.042446919 | 1.62876E-06 | 2.33704E-05 | - |
| *UGT85A24* | 36.59110815 | 6.401244531 | 0.174939893 | -2.515068781 | 1.63318E-06 | 2.34209E-05 | - |
| *At5g06940* | 26.95848942 | 2.945392801 | 0.109256597 | -3.194207708 | 1.66931E-06 | 2.39126E-05 | - |
| *CRK8* | 34.78049894 | 6.09064915 | 0.175116785 | -2.513610723 | 1.6759E-06 | 2.39937E-05 | - |
| *At1g17710* | 21.52578176 | 1.548359038 | 0.071930444 | -3.797253682 | 1.73053E-06 | 2.47213E-05 | K13248 |
| *HSL1* | 32.23573168 | 4.701560218 | 0.145849341 | -2.777449229 | 1.73933E-06 | 2.48197E-05 | - |
| *At1g17710* | 94.09233346 | 37.67209425 | 0.400373685 | -1.320580941 | 1.75758E-06 | 2.50388E-05 | K13248 |
| *HPCA1* | 66.58887075 | 20.45279402 | 0.307150336 | -1.702983133 | 1.7608E-06 | 2.50708E-05 | - |
| *CXE15* | 36.88004719 | 6.80442273 | 0.184501465 | -2.438295824 | 1.81639E-06 | 2.58198E-05 | - |
| *At1g67720* | 92.87414733 | 35.63703584 | 0.383713195 | -1.381899717 | 1.82134E-06 | 2.5876E-05 | - |
| *UGT85A2* | 22.72928813 | 1.835219087 | 0.08074248 | -3.630528294 | 1.85246E-06 | 2.62462E-05 | - |
| *CRK2* | 75.17381438 | 25.32598873 | 0.336899078 | -1.569611616 | 1.85843E-06 | 2.63164E-05 | - |
| *ROQ1* | 35.40567852 | 6.149391609 | 0.173683767 | -2.525465176 | 1.89645E-06 | 2.6811E-05 | - |
| *RAV1* | 62.8457619 | 19.67009878 | 0.31299006 | -1.675811255 | 1.95839E-06 | 2.76112E-05 | K09287 |
| *comta* | 131.5472411 | 57.89684486 | 0.440122076 | -1.184024357 | 1.97022E-06 | 2.7744E-05 | K00545 |
| *NPF5.2* | 86.26339134 | 30.07603802 | 0.348653555 | -1.520133905 | 2.01086E-06 | 2.82589E-05 | - |
| *uncharacterized protein_48451* | 105.4312258 | 42.37938915 | 0.401962406 | -1.314867519 | 2.07355E-06 | 2.90926E-05 | - |
| *CSLD3* | 98.96973826 | 40.33288636 | 0.407527463 | -1.29503081 | 2.07953E-06 | 2.91607E-05 | - |
| *FAO4A* | 54.02477685 | 14.4694256 | 0.267829438 | -1.900613556 | 2.08288E-06 | 2.91919E-05 | K17756 |
| *ATL5* | 115.797706 | 34.83293955 | 0.300808546 | -1.733082542 | 2.10954E-06 | 2.95018E-05 | - |
| *At1g56130* | 159.2833444 | 72.30283596 | 0.453925903 | -1.139471278 | 2.33773E-06 | 3.24479E-05 | - |
| *Gvin1* | 13.4033193 | 0 | 0 | -Inf | 2.36449E-06 | 3.27657E-05 | - |
| *AATL1* | 35.60799671 | 6.890227483 | 0.19350225 | -2.369577753 | 2.36568E-06 | 3.27657E-05 | - |
| *At3g02645* | 101.3068743 | 41.34595855 | 0.408125893 | -1.292913851 | 2.37888E-06 | 3.29309E-05 | - |
| *LPR1* | 104.850099 | 41.20026879 | 0.392944491 | -1.34760257 | 2.39142E-06 | 3.30869E-05 | - |
| *TMEM45B* | 50.37117167 | 12.80926713 | 0.254297581 | -1.975410354 | 2.59367E-06 | 3.56379E-05 | - |
| *FLZ2* | 150.3273355 | 72.39775231 | 0.481600715 | -1.05409056 | 2.60498E-06 | 3.57743E-05 | - |
| *ROQ1* | 163.5591116 | 71.55403104 | 0.437481167 | -1.192707182 | 2.67267E-06 | 3.66263E-05 | - |
| *At5g57670* | 148.8891242 | 69.94034904 | 0.469747871 | -1.090041473 | 2.78729E-06 | 3.80763E-05 | - |
| *RUN1* | 23.05748107 | 2.0825533 | 0.09032007 | -3.468809588 | 2.81838E-06 | 3.84808E-05 | - |
| *ROQ1* | 27.9646941 | 3.786756323 | 0.135412042 | -2.884572057 | 2.87466E-06 | 3.9105E-05 | - |
| *RPT3* | 58.93612248 | 18.22902047 | 0.30930132 | -1.692915105 | 2.90628E-06 | 3.94938E-05 | - |
| *aifm2* | 159.966521 | 62.54865084 | 0.391010884 | -1.354719327 | 2.92762E-06 | 3.97355E-05 | - |
| *R72* | 122.4649088 | 54.18571409 | 0.442459106 | -1.176383973 | 2.93659E-06 | 3.98221E-05 | K00430 |
| *NTR2* | 36.16153176 | 7.153377178 | 0.197817317 | -2.337759372 | 2.97554E-06 | 4.03292E-05 | K00384 |
| *At1g67750* | 15.1689918 | 0 | 0 | -Inf | 2.98298E-06 | 4.04089E-05 | K01728 |
| *RAD5A* | 83.83778384 | 32.20035153 | 0.384079231 | -1.380524143 | 3.02542E-06 | 4.09197E-05 | K15505 |
| *XTH2* | 16.89334603 | 0.648253058 | 0.038373278 | -4.703754192 | 3.09861E-06 | 4.1866E-05 | K08235 |
| *PAD4* | 238.8946615 | 105.6948705 | 0.442432953 | -1.176469252 | 3.11244E-06 | 4.2031E-05 | - |
| *AAP2* | 50.80886111 | 6.456560868 | 0.127075489 | -2.976242313 | 3.1176E-06 | 4.20787E-05 | - |
| *ag4* | 13.14780307 | 0 | 0 | -Inf | 3.18401E-06 | 4.28634E-05 | K04120 |
| *XTH2* | 13.09408701 | 0 | 0 | -Inf | 3.25609E-06 | 4.3743E-05 | K08235 |
| *7OMT* | 13.06413049 | 0 | 0 | -Inf | 3.29712E-06 | 4.42233E-05 | - |
| *LRK10L-2.4* | 65.50157587 | 21.06827433 | 0.321645305 | -1.636457466 | 3.3494E-06 | 4.4857E-05 | - |
| *At4g02290* | 91.41940561 | 33.92493794 | 0.371091211 | -1.430154262 | 3.38266E-06 | 4.52557E-05 | - |
| *CBP1* | 75.27608173 | 26.8268771 | 0.356379829 | -1.488512414 | 3.43577E-06 | 4.59426E-05 | K16296 |
| *HSL1* | 24.8913318 | 2.695799232 | 0.108302732 | -3.206858461 | 3.49785E-06 | 4.67485E-05 | - |
| *nep2* | 46.44603789 | 11.41449272 | 0.245758158 | -2.024688787 | 3.59513E-06 | 4.79499E-05 | - |
| *R12* | 84.36482867 | 32.13150404 | 0.380863738 | -1.392653158 | 3.66619E-06 | 4.87337E-05 | K00430 |
| *SIK1* | 88.28288158 | 35.28914975 | 0.399728114 | -1.32290905 | 3.66706E-06 | 4.87337E-05 | - |
| *HBII* | 20.63733878 | 1.546099684 | 0.07491759 | -3.738551701 | 3.72449E-06 | 4.94463E-05 | - |
| *UPS1* | 65.04204297 | 21.45901397 | 0.329925276 | -1.599788787 | 3.77096E-06 | 4.99864E-05 | - |
| *At5g10770* | 28.17165905 | 3.443413171 | 0.122229691 | -3.032333324 | 3.89187E-06 | 5.14052E-05 | - |
| *SDR1* | 110.2811082 | 46.68790117 | 0.423353573 | -1.24006503 | 3.93155E-06 | 5.18765E-05 | K00059 |
| *DLO1* | 41.31031688 | 3.690747384 | 0.089342026 | -3.484517227 | 3.95543E-06 | 5.21649E-05 | - |
| *uncharacterized protein_24642* | 108.2773299 | 46.32760075 | 0.427860576 | -1.224787343 | 4.02638E-06 | 5.30738E-05 | - |
| *PR1* | 21.43177961 | 1.909752047 | 0.089108421 | -3.488294408 | 4.25333E-06 | 5.58664E-05 | - |
| *CRRSP38* | 27.39064819 | 3.896296409 | 0.142249149 | -2.813508068 | 4.33546E-06 | 5.68391E-05 | - |
| *At3g07010* | 12.8659449 | 0 | 0 | -Inf | 4.33615E-06 | 5.68391E-05 | K01728 |
| *uncharacterized protein_07518* | 12.82152622 | 0 | 0 | -Inf | 4.41685E-06 | 5.7751E-05 | - |
| *UGT85A24* | 38.38607086 | 8.107706908 | 0.211214816 | -2.243217054 | 4.46361E-06 | 5.83329E-05 | - |
| *GLIP* | 100.8387103 | 39.92523865 | 0.395931667 | -1.336676635 | 4.55783E-06 | 5.95042E-05 | - |
| *CYP76T24* | 12.74456863 | 0 | 0 | -Inf | 4.56089E-06 | 5.95142E-05 | - |
| *PME68* | 107.3811398 | 46.29371119 | 0.431115849 | -1.213852495 | 4.58555E-06 | 5.98059E-05 | - |
| *At5g48740* | 17.80399835 | 0.935113106 | 0.052522646 | -4.250916586 | 4.59961E-06 | 5.9929E-05 | - |
| *At1g67720* | 55.71932284 | 15.82693353 | 0.284047485 | -1.815795964 | 4.65077E-06 | 6.05347E-05 | - |
| *XTH9* | 71.06156021 | 18.97668361 | 0.267045693 | -1.904841477 | 4.812E-06 | 6.23802E-05 | K08235 |
| *FLA4* | 114.7959305 | 50.77388192 | 0.44229688 | -1.176913029 | 4.82882E-06 | 6.25382E-05 | - |
| *CYP76T24* | 13.79002721 | 0 | 0 | -Inf | 5.15332E-06 | 6.64422E-05 | - |
| *UCC1* | 111.8286677 | 43.15632473 | 0.385914682 | -1.373646164 | 5.25398E-06 | 6.76056E-05 | - |
| *AAPT1* | 147.5907318 | 71.09680167 | 0.481715896 | -1.053745562 | 5.50322E-06 | 7.04977E-05 | K00993 |
| *uncharacterized protein_42143* | 90.45695331 | 37.04757653 | 0.409560295 | -1.287852234 | 5.53214E-06 | 7.0801E-05 | - |
| *MES17* | 31.88569528 | 5.694249015 | 0.178583185 | -2.48533185 | 5.53235E-06 | 7.0801E-05 | - |
| *IDD14* | 59.17975893 | 18.9868386 | 0.320833321 | -1.64010411 | 5.61036E-06 | 7.15873E-05 | - |
| *At5g10770* | 45.56622599 | 8.527842375 | 0.187152703 | -2.417712207 | 5.62048E-06 | 7.16812E-05 | - |
| *LAC3* | 71.25281321 | 21.16545004 | 0.297047219 | -1.751235811 | 5.72497E-06 | 7.29646E-05 | K05909 |
| *ALDH2B4* | 14.51024113 | 0.363652364 | 0.025061773 | -5.318367731 | 5.99662E-06 | 7.61785E-05 | K00128 |
| *HAK5* | 12.49938197 | 0 | 0 | -Inf | 6.11774E-06 | 7.76411E-05 | K03549 |
| *mpaB&apos;* | 12.49783176 | 0 | 0 | -Inf | 6.12168E-06 | 7.76531E-05 | - |
| *At1g56130* | 125.4454532 | 53.25288456 | 0.424510281 | -1.236128602 | 6.14298E-06 | 7.78624E-05 | - |
| *TAR4* | 42.54156325 | 11.3026191 | 0.265684151 | -1.912215928 | 6.17061E-06 | 7.8159E-05 | - |
| *At5g48740* | 12.45083068 | 0 | 0 | -Inf | 6.24263E-06 | 7.89941E-05 | - |
| *YSL12* | 38.61746166 | 8.578640003 | 0.222144067 | -2.170432482 | 6.27894E-06 | 7.94148E-05 | - |
| *ROQ1* | 50.45329405 | 13.74663959 | 0.272462678 | -1.875869475 | 6.3614E-06 | 8.03401E-05 | - |
| *DTX33* | 34.83163262 | 7.18612495 | 0.206310311 | -2.27711217 | 6.42165E-06 | 8.09434E-05 | K03327 |
| *LRK10L-1.2* | 79.55899909 | 30.55489097 | 0.384053235 | -1.380621793 | 6.51795E-06 | 8.21173E-05 | - |
| *UGT86A1* | 77.9438448 | 19.5142548 | 0.250363 | -1.997906728 | 6.63886E-06 | 8.34784E-05 | - |
| *LECRK51* | 19.23667901 | 1.301024824 | 0.067632507 | -3.886139361 | 6.69889E-06 | 8.40701E-05 | - |
| *BLH2* | 65.76137298 | 13.45409406 | 0.204589616 | -2.289195171 | 6.71638E-06 | 8.42488E-05 | - |
| *UGT89B2* | 70.38074848 | 25.75056872 | 0.365875176 | -1.450576561 | 6.77868E-06 | 8.49481E-05 | - |
| *At3g47570* | 27.91459261 | 1.741469503 | 0.062385632 | -4.002642388 | 6.91796E-06 | 8.65679E-05 | - |
| *At3g47570* | 26.68076385 | 3.707704653 | 0.138965461 | -2.847201738 | 7.09466E-06 | 8.86935E-05 | - |
| *CCR1* | 92.33160815 | 33.30608071 | 0.360722415 | -1.471039019 | 7.09837E-06 | 8.86971E-05 | K09753 |
| *ROQ1* | 48.49526705 | 12.54946937 | 0.258777199 | -1.950217588 | 7.20998E-06 | 8.99617E-05 | - |
| *At5g04720* | 135.7790248 | 56.43535895 | 0.415641216 | -1.266589373 | 7.22533E-06 | 9.01098E-05 | - |
| *At1g49730* | 134.2977929 | 63.47360896 | 0.472633299 | -1.081206817 | 7.33627E-06 | 9.13177E-05 | - |
| *At2g24580* | 91.34163344 | 38.06514244 | 0.416733761 | -1.262802112 | 7.35735E-06 | 9.14928E-05 | K00306 |
| *VIT_06s0061g00120* | 25.6952183 | 3.537162755 | 0.137658405 | -2.860835401 | 7.45645E-06 | 9.25914E-05 | - |
| *TOP2* | 51.11669534 | 15.15603773 | 0.296498778 | -1.753901934 | 7.75843E-06 | 9.60652E-05 | K03164 |
| *PT5* | 15.72496096 | 0.648253058 | 0.041224462 | -4.600355519 | 7.93892E-06 | 9.79259E-05 | K12742 |
| *SD25* | 91.56955794 | 35.71045199 | 0.389981701 | -1.358521664 | 8.1343E-06 | 0.00010005 | - |
| *R3* | 50.65989311 | 14.03124028 | 0.276969402 | -1.852201493 | 8.3431E-06 | 0.000102424 | K00430 |
| *WRKY53* | 22.76595964 | 2.639316129 | 0.115932566 | -3.108642206 | 8.42636E-06 | 0.000103397 | - |
| *TIP1-1* | 68.11540527 | 24.62234515 | 0.361479831 | -1.468012943 | 8.70896E-06 | 0.000106367 | K09873 |
| *SERINC3* | 74.80983384 | 27.50226663 | 0.36762903 | -1.443677401 | 9.15804E-06 | 0.000111531 | - |
| *LRL3* | 194.4935026 | 94.77047759 | 0.48726809 | -1.037212347 | 9.45071E-06 | 0.00011488 | - |
| *CSLE1* | 65.43856242 | 24.1197319 | 0.368585907 | -1.439927184 | 9.74257E-06 | 0.000118261 | - |
| *DOF5.3* | 122.1475634 | 57.61440437 | 0.471678704 | -1.08412363 | 1.02129E-05 | 0.000123622 | - |
| *DAD2* | 19.33378157 | 1.508833203 | 0.078041287 | -3.679618613 | 1.07061E-05 | 0.00012911 | - |
| *LTPG15* | 29.36741824 | 4.929677808 | 0.167862145 | -2.574651177 | 1.07975E-05 | 0.000130032 | - |
| *SAMDC* | 63.82407444 | 22.68548085 | 0.355437679 | -1.492331471 | 1.1213E-05 | 0.000134473 | K01611 |
| *SDI1* | 29.36896845 | 4.664293738 | 0.158817077 | -2.654562042 | 1.12775E-05 | 0.000135184 | - |
| *At3g47570* | 20.31364432 | 1.797952606 | 0.088509604 | -3.498022188 | 1.15111E-05 | 0.000137666 | - |
| *GGCT2;1* | 54.41664954 | 17.42492343 | 0.320213089 | -1.642895815 | 1.15989E-05 | 0.000138652 | - |
| *CRK2* | 11.82720131 | 0 | 0 | -Inf | 1.18805E-05 | 0.000141822 | - |
| *CYP76T24* | 11.73836394 | 0 | 0 | -Inf | 1.2329E-05 | 0.000146771 | - |
| *PLT4* | 45.20239372 | 12.40040345 | 0.274330681 | -1.866012114 | 1.24725E-05 | 0.000148275 | - |
| *GSTU17* | 30.51809244 | 5.58019022 | 0.182848592 | -2.451278581 | 1.26802E-05 | 0.000150193 | K00799 |
| *SWEET1* | 89.37792363 | 37.98043028 | 0.424941963 | -1.23466228 | 1.2819E-05 | 0.000151767 | K15382 |
| *NIP3-1* | 43.80483437 | 11.79393558 | 0.269238219 | -1.893044877 | 1.28665E-05 | 0.000152191 | K09874 |
| *ERD4* | 79.16919463 | 31.41773047 | 0.396842871 | -1.333360207 | 1.29549E-05 | 0.000153097 | - |
| *IAN9* | 49.08362684 | 9.740778112 | 0.198452697 | -2.333132928 | 1.32623E-05 | 0.000156516 | - |
| *CSE* | 71.10701108 | 27.32263812 | 0.384246753 | -1.379895026 | 1.33319E-05 | 0.000157265 | - |
| *CYP750A1* | 37.52485372 | 9.095876997 | 0.242396068 | -2.044561798 | 1.34055E-05 | 0.000158061 | - |
| *At3g47110* | 41.11662977 | 10.38111132 | 0.252479626 | -1.98576112 | 1.35533E-05 | 0.00015966 | - |
| *At3g61320* | 29.81558939 | 5.367863132 | 0.180035453 | -2.473647064 | 1.37061E-05 | 0.000161312 | - |
| *PM19L* | 19.72668645 | 0.972379586 | 0.049292596 | -4.342485226 | 1.37995E-05 | 0.000162191 | - |
| *uncharacterized protein_49231* | 184.0027633 | 87.01522543 | 0.472901732 | -1.080387669 | 1.38736E-05 | 0.00016284 | - |
| *YSL12* | 93.07889576 | 39.80551936 | 0.427653541 | -1.225485609 | 1.39767E-05 | 0.000163894 | - |
| *Bp10* | 362.4583752 | 181.1351179 | 0.499740468 | -1.000749046 | 1.41449E-05 | 0.00016565 | - |
| *Os12g0623900* | 131.8063719 | 63.39229794 | 0.480950177 | -1.056040646 | 1.4247E-05 | 0.000166521 | K00549 |
| *T5AT* | 15.06466008 | 0.648253058 | 0.043031376 | -4.538467204 | 1.42514E-05 | 0.000166521 | K19861 |
| *At3g47570* | 21.00648411 | 2.119819781 | 0.100912641 | -3.308821193 | 1.48851E-05 | 0.000173379 | - |
| *N* | 20.92436173 | 2.157086261 | 0.103089704 | -3.27802784 | 1.50428E-05 | 0.000175058 | - |
| *PLR_Tp2* | 67.24400685 | 25.55629149 | 0.380053074 | -1.395727192 | 1.51056E-05 | 0.00017571 | - |
| *PR5K* | 20.95690064 | 2.084812655 | 0.099480963 | -3.329435718 | 1.5184E-05 | 0.000176543 | - |
| *PLR_Tp1* | 118.9393949 | 56.69284739 | 0.476653235 | -1.068988006 | 1.52737E-05 | 0.000177471 | - |
| *PR4B* | 20.85360111 | 2.122079135 | 0.1017608 | -3.296746175 | 1.54222E-05 | 0.000178991 | - |
| *Tmem45b* | 35.99263639 | 8.408098105 | 0.233606064 | -2.097850373 | 1.55278E-05 | 0.000180136 | - |
| *NOV* | 143.3314218 | 68.36132824 | 0.47694586 | -1.068102585 | 1.60916E-05 | 0.000185845 | K17592 |
| *DGK1* | 90.24710554 | 39.58410565 | 0.438619116 | -1.188959406 | 1.63045E-05 | 0.000188052 | K00901 |
| *GLR2.8* | 13.30673476 | 0 | 0 | -Inf | 1.66215E-05 | 0.000191454 | K05387 |
| *uncharacterized protein_40250* | 91.7975479 | 40.12170106 | 0.437067242 | -1.194072843 | 1.75548E-05 | 0.00020131 | - |
| *EO* | 78.10617346 | 32.2274388 | 0.412610647 | -1.277147044 | 1.76423E-05 | 0.000202223 | K18980 |
| *PAP2* | 121.2114531 | 59.34003417 | 0.48955798 | -1.03044836 | 1.77235E-05 | 0.000202976 | - |
| *7OMT* | 161.8463367 | 64.76902249 | 0.400188375 | -1.321248836 | 1.77236E-05 | 0.000202976 | - |
| *uncharacterized protein_34696* | 27.72312201 | 4.87093535 | 0.17569938 | -2.508818994 | 1.77777E-05 | 0.000203506 | - |
| *At5g47260* | 63.38070219 | 22.83683111 | 0.360312056 | -1.47268117 | 1.80312E-05 | 0.000206134 | K13459 |
| *3BETAHSD/D2* | 114.7017801 | 53.49341649 | 0.466369541 | -1.100454528 | 1.8112E-05 | 0.000206876 | K07748 |
| *BCP* | 29.74896137 | 4.951153786 | 0.166431148 | -2.587002634 | 1.81567E-05 | 0.000207112 | - |
| *CYP76T24* | 69.25781428 | 26.64163729 | 0.384673377 | -1.378294111 | 1.84338E-05 | 0.000209996 | - |
| *UGT86A2* | 16.07344716 | 1.051431256 | 0.065414173 | -3.934252934 | 1.8492E-05 | 0.000210475 | - |
| *P85* | 34.92651867 | 8.000401197 | 0.229063803 | -2.126178594 | 1.86988E-05 | 0.000212641 | - |
| *CYP75B137* | 42.75937579 | 12.09884549 | 0.282951874 | -1.821371405 | 2.03898E-05 | 0.000230658 | K07408 |
| *CAT1* | 28.87534643 | 5.637765912 | 0.195244962 | -2.356642774 | 2.05565E-05 | 0.00023234 | K03294 |
| *APS1* | 80.06811546 | 32.68148881 | 0.408171076 | -1.292754143 | 2.09995E-05 | 0.000236625 | - |
| *3MMP* | 105.612001 | 41.03881426 | 0.388580975 | -1.363712828 | 2.12796E-05 | 0.00023947 | - |
| *CYP75B137* | 32.63638373 | 6.918481525 | 0.211986769 | -2.237953875 | 2.13998E-05 | 0.000240509 | - |
| *IGS1* | 17.26507375 | 1.224232509 | 0.070908038 | -3.817907007 | 2.16049E-05 | 0.000242604 | - |
| *YAB5* | 205.5652859 | 97.67190003 | 0.475138103 | -1.073581188 | 2.16606E-05 | 0.000243124 | - |
| *YSL12* | 94.19976559 | 41.93998785 | 0.445223909 | -1.167397026 | 2.19954E-05 | 0.000246455 | - |
| *L6* | 11.23352845 | 0 | 0 | -Inf | 2.23503E-05 | 0.00025 | - |
| *At1g56130* | 39.2564371 | 10.32236886 | 0.262947165 | -1.92715515 | 2.24297E-05 | 0.00025078 | - |
| *CRK8* | 57.01874744 | 19.93096414 | 0.349551069 | -1.516424846 | 2.3199E-05 | 0.0002586 | - |
| *WRKY42* | 11.12248175 | 0 | 0 | -Inf | 2.34044E-05 | 0.000260666 | - |
| *CRK2* | 39.23681015 | 9.784822656 | 0.249378648 | -2.003590151 | 2.37759E-05 | 0.000264122 | - |
| *KIN12F* | 200.7761311 | 97.35452734 | 0.484890942 | -1.044267791 | 2.39667E-05 | 0.000266014 | K10400 |
| *AZG1* | 103.4065708 | 48.36715289 | 0.467737713 | -1.09622834 | 2.39837E-05 | 0.000266088 | K06901 |
| *BANGLUC* | 27.90581325 | 5.201839942 | 0.186407036 | -2.423471777 | 2.4006E-05 | 0.000266222 | - |
| *CRK8* | 41.7097846 | 11.6324311 | 0.278889743 | -1.84223322 | 2.48067E-05 | 0.000274281 | - |
| *RGI1* | 14.42398615 | 0.650512412 | 0.045099351 | -4.470749522 | 2.53521E-05 | 0.000279834 | - |
| *RAM2* | 97.95408791 | 42.87857628 | 0.437741571 | -1.191848698 | 2.58176E-05 | 0.00028485 | K13508 |
| *Bp10* | 116.7863481 | 57.28235799 | 0.490488477 | -1.027708852 | 2.69725E-05 | 0.000296081 | - |
| *SILD* | 37.05772192 | 9.224633707 | 0.248926087 | -2.006210666 | 2.70121E-05 | 0.00029639 | - |
| *LBD12* | 134.554711 | 54.02634483 | 0.401519534 | -1.316457919 | 2.71576E-05 | 0.000297861 | - |
| *LECRK81* | 40.25850918 | 10.35854275 | 0.257300704 | -1.958472691 | 2.75292E-05 | 0.000301299 | - |
| *AMY1.3* | 49.70378992 | 13.65289 | 0.27468509 | -1.864149493 | 2.80257E-05 | 0.000306474 | K01176 |
| *uncharacterized protein_40403* | 82.23274152 | 34.0051314 | 0.413523017 | -1.273960461 | 2.81435E-05 | 0.000307633 | - |
| *LRK10L-2.5* | 18.34307124 | 1.701943668 | 0.092784008 | -3.429980023 | 2.9129E-05 | 0.000317334 | - |
| *CEQORH* | 12.74456863 | 0.324126529 | 0.025432523 | -5.297181626 | 2.95944E-05 | 0.000321459 | - |
| *CRK2* | 17.93208962 | 1.585625518 | 0.088423912 | -3.499419624 | 3.03122E-05 | 0.000328465 | - |
| *SMXL3* | 69.05756432 | 26.71055895 | 0.386786867 | -1.370389284 | 3.13625E-05 | 0.000338961 | - |
| *SRG1* | 20.45139884 | 1.471566723 | 0.071954331 | -3.796774662 | 3.17084E-05 | 0.000341986 | - |
| *DDB_G0293730* | 84.24411871 | 36.61390991 | 0.434616807 | -1.202184128 | 3.26233E-05 | 0.000351269 | - |
| *CYP76T24* | 22.80263115 | 1.147440194 | 0.050320517 | -4.312709437 | 3.27632E-05 | 0.000352629 | - |
| *UGT84A13* | 63.43493628 | 24.27448329 | 0.382667418 | -1.385837025 | 3.29126E-05 | 0.000354091 | K13691 |
| *NRT2.1* | 14.17879949 | 0.610986577 | 0.043091559 | -4.536450889 | 3.34109E-05 | 0.000359302 | K02575 |
| *At3g47570* | 15.48493908 | 0.860580146 | 0.055575301 | -4.169412341 | 3.34512E-05 | 0.000359587 | - |
| *At1g56140* | 40.54000148 | 11.22021549 | 0.276768996 | -1.853245758 | 3.46272E-05 | 0.000370691 | - |
| *MVD2* | 107.501998 | 40.60740625 | 0.377736293 | -1.404548689 | 3.53756E-05 | 0.000378234 | K01597 |
| *RPV1* | 20.66987769 | 2.755634278 | 0.133316429 | -2.907073517 | 3.54453E-05 | 0.000378823 | - |
| *TPS-mISO1* | 23.22999102 | 3.49763692 | 0.150565573 | -2.731536156 | 3.71144E-05 | 0.00039552 | K16086 |
| *STAD* | 16.36821728 | 1.224232509 | 0.074793271 | -3.740947712 | 3.93954E-05 | 0.000417775 | K03921 |
| *CRK2* | 18.50871792 | 2.028329551 | 0.109587793 | -3.189840987 | 3.97493E-05 | 0.000420661 | - |
| *CYP720B2* | 45.0221366 | 13.73869476 | 0.305154215 | -1.712389576 | 3.97988E-05 | 0.000421013 | - |
| *XI-F* | 49.75270708 | 16.57112135 | 0.333069743 | -1.586103794 | 3.98747E-05 | 0.000421644 | K10357 |
| *DTX27* | 61.39050216 | 22.22927065 | 0.362096251 | -1.465554857 | 3.99534E-05 | 0.000422304 | K03327 |
| *KIN14I* | 66.42499188 | 25.86127558 | 0.389330504 | -1.36093271 | 4.09632E-05 | 0.00043157 | K10406 |
| *At4g15970* | 10.57064518 | 0 | 0 | -Inf | 4.33046E-05 | 0.000454208 | - |
| *uncharacterized protein_35487* | 13.83031329 | 0.648253058 | 0.0468719 | -4.415132922 | 4.43966E-05 | 0.000464534 | - |
| *GSTZ* | 112.9450352 | 51.33968217 | 0.454554572 | -1.137474585 | 4.61931E-05 | 0.000481969 | K01800 |
| *CYP76T24* | 43.96893084 | 13.84031499 | 0.314774881 | -1.667607674 | 4.62592E-05 | 0.000482465 | - |
| *UGT86A2* | 10.40330002 | 0 | 0 | -Inf | 4.64243E-05 | 0.000483993 | - |
| *rumi* | 70.53776408 | 29.22221096 | 0.414277534 | -1.271330509 | 4.75656E-05 | 0.000494896 | - |
| *TPS1* | 81.47430589 | 34.91650917 | 0.428558535 | -1.222435825 | 4.79466E-05 | 0.000498061 | K16055 |
| *GGPS* | 57.98621625 | 20.78593299 | 0.358463344 | -1.480102496 | 4.96699E-05 | 0.000513903 | K13789 |
| *At5g63180* | 13.94807499 | 0.727304727 | 0.052143735 | -4.261362263 | 5.04793E-05 | 0.000521445 | K01728 |
| *RPV1* | 21.94089598 | 3.092199367 | 0.14093314 | -2.826917197 | 5.18316E-05 | 0.00053435 | - |
| *BACOVA_02659* | 47.50699083 | 15.32206092 | 0.322522236 | -1.632529464 | 5.19908E-05 | 0.000535406 | K05349 |
| *ABCC8* | 37.62040608 | 10.20379136 | 0.271230229 | -1.882410117 | 5.1996E-05 | 0.000535406 | - |
| *ATL8* | 19.12304991 | 2.159345615 | 0.112918474 | -3.146646557 | 5.26289E-05 | 0.000540849 | K19040 |
| *TCEA1* | 40.73405446 | 11.98478669 | 0.294220324 | -1.765031188 | 5.36528E-05 | 0.000550498 | - |
| *MAPKKK18* | 14.9449823 | 0.972379586 | 0.06506395 | -3.941997771 | 5.43537E-05 | 0.000557249 | K04373 |
| *uncharacterized protein_11316* | 73.72667156 | 30.99985435 | 0.42047001 | -1.24992519 | 5.45457E-05 | 0.000558411 | - |
| *DTX35* | 96.89999825 | 45.61040105 | 0.470695582 | -1.087133784 | 5.45974E-05 | 0.000558643 | K03327 |
| *KIN8A* | 34.10817001 | 8.690439444 | 0.254790551 | -1.972616317 | 5.53747E-05 | 0.000565926 | K10401 |
| *DTX35* | 111.8927172 | 52.91286913 | 0.472889304 | -1.080425586 | 5.55337E-05 | 0.000566658 | K03327 |
| *DMP4* | 46.22306057 | 14.56652713 | 0.315135496 | -1.665955829 | 5.60576E-05 | 0.000571104 | - |
| *PCNA* | 51.87616316 | 18.15222816 | 0.349914625 | -1.514925132 | 5.69124E-05 | 0.000579358 | K04802 |
| *uncharacterized protein_51382* | 23.39113922 | 3.133984556 | 0.133981698 | -2.899892151 | 5.72639E-05 | 0.00058225 | - |
| *TRN1* | 81.05727558 | 35.50035928 | 0.437966352 | -1.191108061 | 5.73879E-05 | 0.000583282 | - |
| *At3g47570* | 20.96206542 | 2.581740437 | 0.123162503 | -3.021365007 | 5.88763E-05 | 0.000596307 | - |
| *CRK2* | 31.8820807 | 8.225117645 | 0.257985598 | -1.954637567 | 6.17067E-05 | 0.000622541 | - |
| *Os06g0250600* | 40.4144926 | 11.97348992 | 0.296267234 | -1.755029018 | 6.27538E-05 | 0.00063228 | - |
| *At1g32780* | 39.01383283 | 11.83907271 | 0.303458334 | -1.720429652 | 6.33246E-05 | 0.000637376 | K00001 |
| *IGS1* | 13.36561561 | 0.573720097 | 0.042925078 | -4.542035422 | 6.35004E-05 | 0.000638897 | - |
| *MYB61* | 26.40923524 | 5.239106422 | 0.198381603 | -2.333649852 | 6.39493E-05 | 0.000643164 | K09422 |
| *CLINT1* | 51.92508032 | 17.94558655 | 0.345605369 | -1.532802465 | 6.58023E-05 | 0.00066052 | K12471 |
| *RBCS* | 5645.140355 | 2792.87555 | 0.494739789 | -1.015258163 | 6.58658E-05 | 0.000660901 | K01602 |
| *CDC20-1* | 23.82624627 | 4.371748213 | 0.183484556 | -2.44626946 | 6.71154E-05 | 0.000670585 | K03363 |
| *TAR3* | 45.18018438 | 14.04477143 | 0.310861313 | -1.685657013 | 6.84296E-05 | 0.00068319 | - |
| *EXLB1* | 29.6869801 | 6.267969113 | 0.211135289 | -2.243760367 | 6.9316E-05 | 0.000691507 | - |
| *CRK2* | 74.34513615 | 32.47251366 | 0.436780607 | -1.195019294 | 7.16071E-05 | 0.000711897 | - |
| *N* | 62.6995939 | 16.57452249 | 0.264348163 | -1.919488792 | 7.20427E-05 | 0.000715679 | - |
| *Prcp* | 115.5018999 | 33.48791939 | 0.289933927 | -1.786203934 | 7.30017E-05 | 0.000723543 | K01285 |
| *SPT* | 106.1448769 | 51.59267688 | 0.486059039 | -1.040796533 | 7.75103E-05 | 0.000765273 | - |
| *SD25* | 21.32243139 | 3.213036226 | 0.150688079 | -2.730362806 | 7.85941E-05 | 0.000774824 | - |
| *NEK5* | 111.6301163 | 55.22584857 | 0.494721769 | -1.015310712 | 8.01194E-05 | 0.000788962 | K08857 |
| *SDR2a* | 13.07859266 | 0.687778892 | 0.052588142 | -4.249118655 | 8.0566E-05 | 0.000792456 | - |
| *LRK10L-2.5* | 13.11113156 | 0.650512412 | 0.049615276 | -4.333071809 | 8.06888E-05 | 0.00079311 | - |
| *Os06g0508700* | 83.82332168 | 37.59986985 | 0.448560962 | -1.156624025 | 8.06936E-05 | 0.00079311 | K01874 |
| *ROQ1* | 13.04450354 | 0.648253058 | 0.049695495 | -4.330741122 | 8.25041E-05 | 0.00080937 | - |
| *uncharacterized protein_23721* | 18.67193049 | 2.04528682 | 0.109538048 | -3.190496017 | 8.33601E-05 | 0.000817459 | - |
| *At3g47110* | 18.54642161 | 2.159345615 | 0.116429232 | -3.102474778 | 8.35719E-05 | 0.000819226 | - |
| *At1g67720* | 73.82134002 | 30.83045424 | 0.417636069 | -1.25968178 | 8.57771E-05 | 0.000838984 | - |
| *RUN1* | 15.40643128 | 1.259239635 | 0.081734674 | -3.612907956 | 8.58052E-05 | 0.000838984 | - |
| *CYP76T24* | 9.846298668 | 0 | 0 | -Inf | 8.61298E-05 | 0.000841754 | - |
| *SUS2* | 48.10014566 | 13.12435624 | 0.272854813 | -1.873794604 | 8.70953E-05 | 0.000850549 | K00695 |
| *NAC068* | 9.804462376 | 0 | 0 | -Inf | 8.76391E-05 | 0.000855537 | - |
| *CYP720B2* | 150.0733734 | 64.15798671 | 0.427510792 | -1.225967255 | 8.78076E-05 | 0.000856537 | - |
| *At2g26850* | 128.3821587 | 55.83678595 | 0.434926368 | -1.201156919 | 8.94057E-05 | 0.000870816 | - |
| *ACR4* | 29.65237682 | 4.898022625 | 0.165181451 | -2.597876407 | 8.95019E-05 | 0.000871426 | - |
| *APM1* | 62.28824253 | 24.31631768 | 0.39038375 | -1.357035095 | 9.0244E-05 | 0.000877991 | K08776 |
| *D2* | 59.00171832 | 22.55563156 | 0.382287706 | -1.387269289 | 9.39059E-05 | 0.000909183 | K00705 |
| *UGT86A2* | 37.1873634 | 10.57648114 | 0.284410622 | -1.81395275 | 9.51174E-05 | 0.000919196 | - |
| *HSL1* | 11.45392338 | 0.363652364 | 0.031749153 | -4.977138095 | 9.83883E-05 | 0.000946924 | - |
| *IP5P11* | 36.32304584 | 10.32010951 | 0.284120158 | -1.815426905 | 9.84989E-05 | 0.000947636 | K20278 |
| *BOR1* | 35.802716 | 9.993723623 | 0.279133114 | -1.840974809 | 9.92328E-05 | 0.000952929 | - |
| *ROQ1* | 82.35241931 | 37.51741703 | 0.455571522 | -1.134250527 | 0.000103285 | 0.000989647 | - |
| *CRK8* | 82.7041542 | 37.03069344 | 0.447748892 | -1.159238232 | 0.000103723 | 0.000992738 | - |
| *ag1* | 15.09203421 | 1.338291304 | 0.088675343 | -3.49532319 | 0.000108893 | 0.001037624 | K16086 |
| *XTH33* | 73.01833743 | 32.3979807 | 0.443696499 | -1.172354924 | 0.000110429 | 0.001050718 | K08235 |
| *DTX40* | 12.73785364 | 0.610986577 | 0.047966211 | -4.381837704 | 0.000110578 | 0.001051752 | K03327 |
| *VIT_06s0061g00120* | 61.6165799 | 24.97470075 | 0.405324359 | -1.302851215 | 0.000110953 | 0.001054931 | - |
| *PME12* | 39.18789299 | 11.7566691 | 0.300007686 | -1.736928632 | 0.000117874 | 0.001113382 | K01051 |
| *ATL47* | 18.93054713 | 2.63479742 | 0.139182317 | -2.844952161 | 0.000119754 | 0.001128673 | - |
| *PRCP* | 9.458558573 | 0 | 0 | -Inf | 0.000122806 | 0.001151998 | K01285 |
| *GSVIVT00023967001* | 28.08762058 | 6.689197168 | 0.23815464 | -2.070029438 | 0.000124708 | 0.001168142 | K00430 |
| *SBT1.7* | 38.21769351 | 11.39753545 | 0.298226669 | -1.745518816 | 0.00012513 | 0.00117168 | - |
| *MCM4* | 46.9374434 | 16.26621144 | 0.346550861 | -1.52886099 | 0.000125779 | 0.001176478 | K02212 |
| *LRR1* | 77.00353252 | 31.22802114 | 0.405540111 | -1.302083479 | 0.000126849 | 0.001186058 | - |
| *SDR2a* | 12.52520588 | 0.324126529 | 0.02587794 | -5.272133402 | 0.000129801 | 0.001211909 | - |
| *RPV1* | 22.74979898 | 4.227201001 | 0.185812675 | -2.428079179 | 0.000130427 | 0.001216883 | - |
| *VQ4* | 109.5397171 | 54.74138433 | 0.499740056 | -1.000750236 | 0.000133509 | 0.001243395 | - |
| *At3g24670* | 11.1379761 | 0.324126529 | 0.029101026 | -5.102786185 | 0.000134791 | 0.00125309 | K01728 |
| *T5AT* | 24.38391392 | 4.9872535 | 0.204530475 | -2.289612273 | 0.000135447 | 0.001258284 | K19861 |
| *At4g34480* | 53.47205638 | 15.47455296 | 0.289395135 | -1.788887427 | 0.000137685 | 0.001277242 | - |
| *HKT6* | 34.91360671 | 10.07277529 | 0.288505721 | -1.793328165 | 0.000139372 | 0.001291046 | - |
| *GSTU18* | 44.86128883 | 7.832118653 | 0.174585235 | -2.517996542 | 0.000140049 | 0.001295932 | K00799 |
| *NRT2.1* | 34.23987585 | 9.592804779 | 0.280164707 | -1.835652868 | 0.000144839 | 0.00133596 | K02575 |
| *uncharacterized protein_24227* | 85.44091008 | 40.53617603 | 0.474435209 | -1.075717015 | 0.000146489 | 0.001349263 | - |
| *At1g61300* | 12.44308351 | 0.573720097 | 0.04610755 | -4.438853177 | 0.000147537 | 0.001357674 | K13459 |
| *CSLA9* | 71.78089022 | 31.27544261 | 0.435707087 | -1.198569514 | 0.00014756 | 0.001357674 | K13680 |
| *FEZ* | 45.60201359 | 16.10920069 | 0.353256346 | -1.501212617 | 0.000149146 | 0.001370572 | - |
| *CRK8* | 14.73580084 | 1.147440194 | 0.077867515 | -3.682834602 | 0.0001501 | 0.001378294 | - |
| *At3g47110* | 29.71487225 | 7.738369069 | 0.260420742 | -1.941083736 | 0.000150589 | 0.001381133 | - |
| *uncharacterized protein_19673* | 69.98267882 | 30.40130634 | 0.434411869 | -1.202864572 | 0.000155105 | 0.001418072 | K11666 |
| *SBT5.6* | 50.472921 | 18.33294926 | 0.363223465 | -1.461070687 | 0.000156109 | 0.001426715 | - |
| *ACR4* | 29.03855899 | 7.074325509 | 0.243618339 | -2.037305355 | 0.000157106 | 0.001434821 | - |
| *At4g19900* | 86.37568783 | 40.04724228 | 0.463640213 | -1.108922395 | 0.000160048 | 0.001458091 | K01988 |
| *NPF5.7* | 37.91687469 | 11.73745248 | 0.309557488 | -1.691720738 | 0.000160558 | 0.001462226 | K14638 |
| *FRI3* | 18.31503081 | 0.573720097 | 0.031325096 | -4.996537262 | 0.000165025 | 0.001501324 | K00522 |
| *uncharacterized protein_09875* | 94.24691495 | 45.87697686 | 0.486774309 | -1.038675069 | 0.000172527 | 0.001559175 | - |
| *7OMT* | 32.66375786 | 9.135402832 | 0.279680093 | -1.838150526 | 0.000173302 | 0.001565091 | - |
| *RTNLB5* | 69.70443522 | 29.98233764 | 0.430135293 | -1.217137586 | 0.000174965 | 0.001577363 | - |
| *XYN5* | 72.70807295 | 23.67600945 | 0.325631096 | -1.618689621 | 0.0001753 | 0.001579285 | - |
| *NPF2.13* | 32.21263844 | 8.622684547 | 0.26768017 | -1.901417826 | 0.000176056 | 0.00158554 | - |
| *At1g66480* | 74.8667986 | 24.36140561 | 0.325396652 | -1.619728688 | 0.000178091 | 0.001601921 | - |
| *At1g67750* | 10.84320597 | 0.363652364 | 0.033537347 | -4.898087613 | 0.000178122 | 0.001601921 | K01728 |
| *REL2* | 97.88244338 | 48.51966916 | 0.495693277 | -1.012480402 | 0.000179503 | 0.001613226 | - |
| *BANGLUC* | 14.39506182 | 1.454609454 | 0.101049198 | -3.306870221 | 0.000180311 | 0.001618241 | - |
| *LTPG15* | 34.01240005 | 5.367863132 | 0.157820769 | -2.66364102 | 0.000180817 | 0.001621661 | - |
| *CRRSP55* | 25.27390711 | 5.47288451 | 0.216542875 | -2.207275391 | 0.000182404 | 0.001633632 | - |
| *LBD12* | 16.41521836 | 1.949277882 | 0.118748215 | -3.074022266 | 0.000182843 | 0.001636433 | - |
| *At5g48740* | 30.81611127 | 7.371364763 | 0.239204898 | -2.063681162 | 0.000183806 | 0.001644099 | - |
| *CDC6B* | 10.7543686 | 0.363652364 | 0.033814385 | -4.886219062 | 0.000183826 | 0.001644099 | K02213 |
| *uncharacterized protein_17502* | 75.30433976 | 27.5282863 | 0.365560423 | -1.451818208 | 0.000187633 | 0.0016742 | - |
| *AAP3* | 12.13126882 | 0.687778892 | 0.05669472 | -4.1406418 | 0.000188524 | 0.001680902 | - |
| *GLR2.7* | 18.03125655 | 1.951537236 | 0.108230795 | -3.207817043 | 0.000191234 | 0.001703304 | K05387 |
| *BANGLUC* | 26.09483817 | 5.393857819 | 0.206702099 | -2.274375057 | 0.00019198 | 0.001707601 | - |
| *FLZ6* | 12.09459731 | 0.650512412 | 0.053785372 | -4.216642341 | 0.000192978 | 0.001715886 | - |
| *SD25* | 90.92459926 | 45.26253919 | 0.49780301 | -1.006353141 | 0.000199705 | 0.001768426 | - |
| *LTPG16* | 62.53306332 | 24.6506484 | 0.394201836 | -1.342993598 | 0.000204106 | 0.001803698 | - |
| *LTPG16* | 33.56666301 | 9.138828953 | 0.272259085 | -1.876947905 | 0.000209532 | 0.00184724 | - |
| *STP7* | 106.248394 | 48.60212197 | 0.45743865 | -1.128349826 | 0.000211073 | 0.001859567 | - |
| *RPK1* | 17.1318177 | 2.238397285 | 0.130657314 | -2.936140208 | 0.000217092 | 0.001908703 | - |
| *GSO1* | 30.98434034 | 8.263550892 | 0.266700882 | -1.906705499 | 0.000217886 | 0.001913735 | - |
| *CAD* | 60.21127337 | 24.72627394 | 0.410658545 | -1.283988775 | 0.000218396 | 0.001916919 | K00083 |
| *CRK8* | 92.55185093 | 45.60478975 | 0.492748544 | -1.021076488 | 0.000218396 | 0.001916919 | - |
| *BGLU12* | 10.76986295 | 0 | 0 | -Inf | 0.000218723 | 0.00191914 | K01188 |
| *At2g24130* | 23.51184919 | 4.605551279 | 0.195882138 | -2.351942249 | 0.000219908 | 0.001927575 | - |
| *At4g10440* | 51.17749228 | 16.24247611 | 0.317375381 | -1.655737873 | 0.000219988 | 0.001927626 | - |
| *LAC1* | 59.97811477 | 24.78610899 | 0.413252552 | -1.274904366 | 0.000222118 | 0.001943011 | K05909 |
| *CRRSP28* | 30.12673777 | 8.039927032 | 0.26687015 | -1.905790148 | 0.000223617 | 0.001954801 | - |
| *MYB4* | 29.6559914 | 7.645786252 | 0.257815905 | -1.955586828 | 0.000223698 | 0.001954852 | K09422 |
| *CRK2* | 13.67226551 | 0.860580146 | 0.062943493 | -3.989798952 | 0.000225189 | 0.001966551 | - |
| *LRK10* | 13.20668392 | 0.860580146 | 0.06516247 | -3.939814898 | 0.000227282 | 0.001982825 | - |
| *uncharacterized protein_47025* | 28.41647983 | 6.767156249 | 0.238141962 | -2.070106243 | 0.000231476 | 0.002016902 | - |
| *NAC035* | 8.859720934 | 0 | 0 | -Inf | 0.000232143 | 0.00202048 | - |
| *RAN1A* | 158.4751732 | 42.65942193 | 0.269186782 | -1.893320523 | 0.000234072 | 0.002034535 | K07936 |
| *ROQ1* | 14.25730729 | 1.221973155 | 0.085708551 | -3.544417036 | 0.000236635 | 0.002054748 | - |
| *PR13* | 8.765718784 | 0 | 0 | -Inf | 0.000241381 | 0.002091752 | K13449 |
| *XTH2* | 8.765718784 | 0 | 0 | -Inf | 0.000241381 | 0.002091752 | K08235 |
| *WNK4* | 45.08633051 | 12.89507188 | 0.286008458 | -1.805870282 | 0.000248129 | 0.002144494 | K08867 |
| *CAX1a* | 32.09384455 | 8.635123107 | 0.269058545 | -1.894007971 | 0.000251071 | 0.002166313 | K07300 |
| *WSD11* | 15.85460244 | 1.434300243 | 0.09046586 | -3.466482741 | 0.000251263 | 0.002167244 | - |
| *FPP7* | 67.99661139 | 29.55544908 | 0.434660617 | -1.20203871 | 0.000255036 | 0.002192011 | - |
| *GLR2.8* | 10.43738913 | 0.286860049 | 0.027483889 | -5.185270007 | 0.000255041 | 0.002192011 | K05387 |
| *CHIT1* | 14.87938645 | 1.224232509 | 0.082277083 | -3.603365549 | 0.000261088 | 0.002237111 | - |
| *JGB* | 63.13979641 | 15.28479444 | 0.242078615 | -2.046452459 | 0.000263755 | 0.002259213 | - |
| *BALDH* | 30.65961369 | 8.026395884 | 0.261790509 | -1.933515299 | 0.000264019 | 0.002259562 | K12355 |
| *GLR2.8* | 36.72886268 | 11.40205416 | 0.310438531 | -1.687620464 | 0.000265023 | 0.002267081 | K05387 |
| *CYP720B2* | 33.47007847 | 10.10552306 | 0.30192708 | -1.727727935 | 0.000266901 | 0.002281634 | - |
| *ZNFX1* | 28.59503846 | 7.396192683 | 0.258653007 | -1.950910131 | 0.00026896 | 0.002296214 | - |
| *TFIIIA* | 66.81443046 | 30.07149433 | 0.450074843 | -1.151763166 | 0.000270636 | 0.002308244 | K09191 |
| *SSL4* | 58.57059172 | 23.5495121 | 0.402070585 | -1.314479299 | 0.000278155 | 0.002364037 | - |
| *CPK10* | 59.89119349 | 26.04644121 | 0.434896012 | -1.201257615 | 0.000279729 | 0.0023733 | - |
| *SRG1* | 37.71182584 | 12.2739061 | 0.325465708 | -1.61942255 | 0.0002808 | 0.002380057 | - |
| *N* | 478.5304927 | 234.0031444 | 0.489003623 | -1.032082942 | 0.000281046 | 0.002381359 | - |
| *EXPA2* | 86.83522074 | 37.88228536 | 0.43625484 | -1.196756956 | 0.000284595 | 0.00240986 | - |
| *HLS1* | 73.07980067 | 33.76792719 | 0.46206923 | -1.113819075 | 0.000285577 | 0.002417378 | - |
| *N* | 9.910344301 | 0 | 0 | -Inf | 0.000286334 | 0.002422205 | - |
| *At1g67720* | 12.90261641 | 0.897846626 | 0.0695864 | -3.845050818 | 0.000292988 | 0.002469637 | - |
| *DCTPP1* | 34.04737307 | 10.54370839 | 0.309677589 | -1.691161116 | 0.000293706 | 0.002473274 | K16904 |
| *At3g47110* | 17.52472258 | 2.790641404 | 0.159240261 | -2.65072295 | 0.000296662 | 0.002496542 | K13420 |
| *LOX1.5* | 52.83846718 | 8.984077556 | 0.17002911 | -2.556146328 | 0.000298076 | 0.002506814 | K15718 |
| *RBOHA* | 40.16089246 | 13.01590874 | 0.324094111 | -1.625515289 | 0.000298476 | 0.002509368 | K13447 |
| *LRK10* | 19.09567578 | 3.329354375 | 0.17435122 | -2.519931633 | 0.000301155 | 0.002528611 | - |
| *HSL2* | 15.712049 | 1.872485567 | 0.119175135 | -3.068844831 | 0.000305195 | 0.002558388 | - |
| *At3g18200* | 34.05150567 | 10.05472544 | 0.295279907 | -1.759844905 | 0.000306296 | 0.002565961 | - |
| *LOX1.1* | 57.38804492 | 11.22138225 | 0.19553519 | -2.354499827 | 0.000306525 | 0.002567051 | K15718 |
| *UGT86A2* | 178.3641228 | 76.56723002 | 0.429274838 | -1.220026482 | 0.00031263 | 0.002613952 | - |
| *AMC9* | 67.44477483 | 29.3533012 | 0.435219797 | -1.200183913 | 0.000315836 | 0.00263906 | - |
| *NAKR2* | 72.19925314 | 31.16361819 | 0.431633526 | -1.212121169 | 0.000318982 | 0.002662771 | - |
| *PGDH1* | 27.63841725 | 7.072066155 | 0.255878117 | -1.966471325 | 0.000322448 | 0.002687379 | K00058 |
| *APS1* | 53.94073838 | 21.78088114 | 0.403792788 | -1.308312951 | 0.000322966 | 0.002690826 | - |
| *NPF2.9* | 35.07681928 | 10.97739998 | 0.312953119 | -1.675981542 | 0.000325547 | 0.002710593 | - |
| *OPT7* | 26.95590703 | 6.745680271 | 0.250248684 | -1.99856561 | 0.000325949 | 0.002711368 | - |
| *WRKY42* | 11.50092446 | 0.727304727 | 0.063238806 | -3.983046068 | 0.000327063 | 0.002718847 | - |
| *CRK8* | 23.62806068 | 4.929677808 | 0.208636582 | -2.260935958 | 0.000327285 | 0.002719551 | - |
| *At4g01130* | 84.93282589 | 40.71357017 | 0.47936201 | -1.060812515 | 0.000330106 | 0.002741509 | - |
| *DTX27* | 24.14219355 | 5.367863132 | 0.222343638 | -2.169136973 | 0.000333635 | 0.00276373 | K03327 |
| *uncharacterized protein_27506* | 33.65033559 | 10.89718155 | 0.323835746 | -1.626665849 | 0.000335243 | 0.002776165 | - |
| *At3g47110* | 11.43171404 | 0.687778892 | 0.060164109 | -4.054953083 | 0.00033827 | 0.002795935 | - |
| *At1g67720* | 8.375396298 | 0 | 0 | -Inf | 0.000344785 | 0.002840671 | - |
| *Prcp* | 51.48429046 | 20.79380364 | 0.403886379 | -1.307978603 | 0.000349869 | 0.002878897 | K01285 |
| *Fra a 1.06* | 58.00532518 | 19.86102407 | 0.342400013 | -1.54624534 | 0.000352075 | 0.002891544 | - |
| *LHT1* | 34.58902835 | 10.16987682 | 0.294020309 | -1.766012282 | 0.000352271 | 0.002892238 | - |
| *MUCI21* | 52.49979253 | 21.51207095 | 0.409755352 | -1.287165303 | 0.000354241 | 0.002906573 | K18134 |
| *uncharacterized protein_37868* | 69.73490976 | 33.41097871 | 0.479114103 | -1.061558815 | 0.000358541 | 0.002938129 | - |
| *PIP2-8* | 72.34977134 | 32.85093812 | 0.454057249 | -1.139053887 | 0.00035975 | 0.002946178 | K09872 |
| *PYL3* | 15.54123754 | 1.874744921 | 0.12063035 | -3.051335169 | 0.000374676 | 0.003051066 | K14496 |
| *RUK* | 27.91511063 | 7.393933329 | 0.264872077 | -1.916632334 | 0.000381256 | 0.003096868 | K17545 |
| *INV1* | 22.60894404 | 4.721869429 | 0.208849623 | -2.259463559 | 0.000384339 | 0.003119959 | K01193 |
| *CYP750A1* | 12.55154782 | 0.897846626 | 0.071532741 | -3.805252456 | 0.000386452 | 0.003135148 | - |
| *At1g32860* | 26.85363968 | 5.929144669 | 0.220794825 | -2.179221737 | 0.000387544 | 0.003143021 | - |
| *uncharacterized protein_37835* | 38.72836009 | 13.01364938 | 0.336023765 | -1.573364823 | 0.00038971 | 0.00315861 | - |
| *RKF3* | 14.43173333 | 1.701943668 | 0.117930648 | -3.083989394 | 0.000392269 | 0.003175386 | - |
| *LOX1.1* | 26.96195571 | 2.51847927 | 0.093408627 | -3.420300386 | 0.000397486 | 0.003211596 | K15718 |
| *ACR4* | 19.98323486 | 2.35245608 | 0.117721485 | -3.08655045 | 0.000397824 | 0.00321333 | - |
| *uncharacterized protein_32770* | 9.55152854 | 0 | 0 | -Inf | 0.000398873 | 0.003220798 | - |
| *CXE17* | 12.43378612 | 0.897846626 | 0.072210236 | -3.791652839 | 0.000399423 | 0.003224235 | - |
| *At3g47570* | 14.45652506 | 1.585625518 | 0.109682341 | -3.18859682 | 0.00040083 | 0.003232576 | - |
| *UGT86A1* | 50.56227639 | 20.96543813 | 0.414645851 | -1.270048438 | 0.000405255 | 0.00326318 | - |
| *uncharacterized protein_51264* | 34.19545716 | 8.68368636 | 0.253942689 | -1.977425153 | 0.000416396 | 0.003334258 | - |
| *CYP750A1* | 15.39196912 | 1.090957091 | 0.070878332 | -3.818511546 | 0.000420633 | 0.003363165 | K05280 |
| *ROQ1* | 58.41335853 | 15.85173647 | 0.27137177 | -1.881657448 | 0.000441295 | 0.003508705 | - |
| *GSVIVT00026920001* | 46.39815292 | 17.29281478 | 0.37270481 | -1.423894656 | 0.000442474 | 0.003515923 | - |
| *At3g47110* | 25.49016944 | 6.998625782 | 0.27456176 | -1.864797389 | 0.000446144 | 0.003540746 | - |
| *SCPL42* | 34.28067996 | 10.89608896 | 0.317849266 | -1.653585339 | 0.000449359 | 0.003561904 | K16297 |
| *RPV1* | 22.07761833 | 4.79640239 | 0.217251803 | -2.202559944 | 0.000474879 | 0.003748144 | - |
| *CRK2* | 11.01401743 | 0.573720097 | 0.052089994 | -4.262849931 | 0.000478869 | 0.003772745 | - |
| *XA21* | 8.049119445 | 0 | 0 | -Inf | 0.000479548 | 0.00377695 | - |
| *EXL2* | 16.86700409 | 2.602049648 | 0.154268632 | -2.696483351 | 0.000484149 | 0.003810875 | - |
| *GSVIVT00023967001* | 8.024327712 | 0 | 0 | -Inf | 0.000484564 | 0.003811825 | K00430 |
| *CRSP* | 15.0987492 | 1.870226212 | 0.123866301 | -3.013144353 | 0.000488676 | 0.003836324 | - |
| *TMN12* | 10.7977551 | 0 | 0 | -Inf | 0.000489296 | 0.003837938 | - |
| *At5g48740* | 19.243394 | 3.537162755 | 0.183811793 | -2.443698765 | 0.0004914 | 0.003851581 | - |
| *ROQ1* | 22.95743023 | 4.741086053 | 0.206516409 | -2.275671677 | 0.000492338 | 0.003857767 | - |
| *N* | 14.2547249 | 1.622891999 | 0.113849409 | -3.134801298 | 0.000494324 | 0.003872154 | K19613 |
| *HD6* | 29.26308652 | 8.217246994 | 0.280805888 | -1.832354908 | 0.000507119 | 0.003963999 | K03097 |
| *DAD2* | 16.76783715 | 2.446205664 | 0.145886774 | -2.777079003 | 0.000508435 | 0.003973088 | - |
| *NRT2.1* | 12.15089577 | 0.897846626 | 0.073891394 | -3.758449845 | 0.000516222 | 0.004025447 | K02575 |
| *R57* | 56.89287269 | 24.39196821 | 0.428735043 | -1.221841754 | 0.000524166 | 0.004080029 | K00430 |
| *RPV1* | 13.05638331 | 1.338291304 | 0.10250092 | -3.286291233 | 0.000527464 | 0.004103239 | - |
| *LRK10L-1.2* | 33.46506196 | 10.51547933 | 0.314222616 | -1.670141072 | 0.000530868 | 0.004123969 | - |
| *ROQ1* | 24.01735098 | 6.236313929 | 0.259658691 | -1.945311578 | 0.000534087 | 0.004144435 | - |
| *TDR* | 17.37958675 | 2.956664594 | 0.170122836 | -2.555351281 | 0.000543867 | 0.004209366 | - |
| *uncharacterized protein_01927* | 72.9115716 | 35.60423887 | 0.488320826 | -1.034098787 | 0.000548632 | 0.004237396 | - |
| *BHLH130* | 75.72100419 | 36.14076666 | 0.477288528 | -1.067066433 | 0.000551954 | 0.004259243 | - |
| *ROQ1* | 15.6367899 | 1.741469503 | 0.111370014 | -3.166567257 | 0.000554199 | 0.004275294 | K19613 |
| *NAT6* | 59.1597661 | 26.04986733 | 0.440330803 | -1.183340323 | 0.000555971 | 0.004287103 | K14611 |
| *HMG1* | 21.90422447 | 4.875454059 | 0.222580538 | -2.167600646 | 0.000557624 | 0.004296603 | K00021 |
| *AZG1* | 18.27386082 | 3.327095021 | 0.182068532 | -2.457446498 | 0.000558268 | 0.004300292 | K06901 |
| *MYB61* | 29.60227533 | 8.771750468 | 0.296320143 | -1.754771394 | 0.00056747 | 0.004367281 | K09422 |
| *GALS1* | 23.24703558 | 5.484181282 | 0.235908844 | -2.083698592 | 0.000569443 | 0.004381161 | - |
| *At5g48740* | 33.6560184 | 10.72780641 | 0.318748531 | -1.649509403 | 0.000570679 | 0.004389369 | - |
| *AMC9* | 29.55682447 | 8.701736216 | 0.294407007 | -1.764116088 | 0.000573569 | 0.004408358 | - |
| *HIPP37* | 40.97769091 | 15.34462949 | 0.37446301 | -1.41710488 | 0.000575367 | 0.004416253 | - |
| *HSL1* | 29.64566183 | 8.543632878 | 0.288191673 | -1.794899444 | 0.000577266 | 0.004429519 | K00924 |
| *GA3OX4* | 51.09190361 | 21.13714679 | 0.413708343 | -1.273314042 | 0.000583381 | 0.004472471 | - |
| *GULLO2* | 14.85872732 | 2.028329551 | 0.136507623 | -2.872946574 | 0.000588205 | 0.004505461 | - |
| *PRB1* | 21.00117104 | 0.573720097 | 0.027318481 | -5.19397891 | 0.000588854 | 0.004507764 | K13449 |
| *IAMT1* | 24.51133888 | 5.827524434 | 0.237748108 | -2.072494237 | 0.000592228 | 0.00453141 | K18848 |
| *ag1* | 78.35962532 | 38.8274051 | 0.49550269 | -1.013035207 | 0.000592292 | 0.00453141 | K16086 |
| *PMIR1* | 55.14099605 | 22.97805136 | 0.416714477 | -1.262868872 | 0.000613969 | 0.004686194 | - |
| *At3g47110* | 16.55777179 | 2.44394631 | 0.147601159 | -2.760224044 | 0.00062224 | 0.004739837 | - |
| *EFR* | 13.87214959 | 1.701943668 | 0.122687811 | -3.026936169 | 0.0006293 | 0.004783489 | - |
| *UGT86A2* | 19.54229673 | 3.859029929 | 0.197470644 | -2.340289894 | 0.000636531 | 0.004834209 | - |
| *ACA5* | 18.77094914 | 3.341792936 | 0.178030046 | -2.489807352 | 0.00063947 | 0.00485368 | K01537 |
| *DIR19* | 7.801350392 | 0 | 0 | -Inf | 0.000645643 | 0.004890518 | - |
| *DDB_G0268948* | 101.6495294 | 46.88106084 | 0.461202931 | -1.116526415 | 0.000651394 | 0.004926882 | - |
| *BANGLUC* | 11.76832046 | 1.051431256 | 0.089344207 | -3.484482005 | 0.000656929 | 0.004962953 | - |
| *ROQ1* | 11.83236609 | 0.972379586 | 0.08217964 | -3.605075178 | 0.000659627 | 0.004980433 | - |
| *ROQ1* | 12.76419558 | 1.415083619 | 0.110863517 | -3.173143408 | 0.000662703 | 0.005000749 | - |
| *LRK10L-1.2* | 20.09516548 | 4.224941647 | 0.210246671 | -2.249845137 | 0.000664373 | 0.005011891 | - |
| *TBL1* | 34.56253813 | 11.00679581 | 0.318460287 | -1.65081462 | 0.000676407 | 0.005093782 | - |
| *MT2788* | 53.7434367 | 22.70020375 | 0.422380948 | -1.243383333 | 0.000677464 | 0.005100259 | - |
| *uncharacterized protein_12414* | 17.21290789 | 2.768072838 | 0.160813783 | -2.636537031 | 0.000679585 | 0.005113254 | - |
| *At4g27220* | 17.20257832 | 2.770332193 | 0.161041685 | -2.634493926 | 0.000680562 | 0.005117635 | K13459 |
| *TBL36* | 50.34704624 | 20.85028675 | 0.414131281 | -1.271839916 | 0.000684591 | 0.005138547 | - |
| *MYB16* | 44.58289695 | 17.08048769 | 0.383117493 | -1.384141196 | 0.000684913 | 0.005138547 | K09422 |
| *At5g24080* | 62.18634493 | 19.22287604 | 0.309117316 | -1.693773622 | 0.000685055 | 0.005138547 | - |
| *LTPG5* | 22.11170744 | 5.239106422 | 0.236938121 | -2.07741776 | 0.000689244 | 0.005166441 | - |
| *AVT6A* | 60.16375427 | 27.45139483 | 0.456277956 | -1.13201514 | 0.000691072 | 0.005177243 | K14207 |
| *RAP2-4* | 22.02390226 | 3.917772387 | 0.177887294 | -2.490964624 | 0.000708931 | 0.005285227 | K09286 |
| *LRK10L-1.2* | 16.73699673 | 0.610986577 | 0.03650515 | -4.775756179 | 0.000713683 | 0.005314295 | - |
| *CXE15* | 14.46168984 | 2.102862512 | 0.145409183 | -2.781809708 | 0.000745186 | 0.005526656 | - |
| *GLP4* | 86.83418855 | 36.62076215 | 0.421732071 | -1.245601359 | 0.00074637 | 0.005532267 | - |
| *RNS1* | 51.59791956 | 21.44096411 | 0.415539314 | -1.266943118 | 0.000747662 | 0.005540264 | K01166 |
| *uncharacterized protein_41068* | 53.40114747 | 23.03786142 | 0.431411356 | -1.212863943 | 0.000748576 | 0.005542288 | - |
| *LDOX* | 32.59609765 | 10.14847502 | 0.311340183 | -1.683436303 | 0.000762222 | 0.005628851 | K05277 |
| *MANA* | 40.50332997 | 14.76529809 | 0.364545288 | -1.455830041 | 0.00076928 | 0.005677739 | K01191 |
| *CRRSP38* | 10.57064518 | 0.650512412 | 0.061539518 | -4.02234304 | 0.000771328 | 0.005689614 | - |
| *LECRK41* | 18.48134379 | 3.385837479 | 0.183202992 | -2.44848503 | 0.000775875 | 0.005719902 | - |
| *HAK17* | 19.14680946 | 3.940340953 | 0.205796217 | -2.280711633 | 0.000777812 | 0.005732548 | K03549 |
| *CDC73* | 51.69952061 | 22.33312526 | 0.431979349 | -1.210965749 | 0.000782269 | 0.005758849 | K15175 |
| *CYP720B2* | 27.64771463 | 8.432926025 | 0.305013493 | -1.713055028 | 0.000808349 | 0.005932317 | - |
| *At4g33300* | 28.86863143 | 8.473618626 | 0.293523392 | -1.768452612 | 0.000809932 | 0.005938893 | - |
| *CYP750A1* | 74.86871469 | 37.1887718 | 0.496719784 | -1.009495885 | 0.000814867 | 0.005971701 | - |
| *CYP76T24* | 51.71708319 | 15.93188073 | 0.308058377 | -1.698724326 | 0.000816443 | 0.005979865 | - |
| *ACA5* | 61.2314222 | 27.68522287 | 0.452140778 | -1.145156057 | 0.000819273 | 0.005995515 | K01537 |
| *uncharacterized protein_39659* | 46.13938799 | 18.00884771 | 0.39031397 | -1.357292994 | 0.000820947 | 0.006004375 | - |
| *TCM_000168* | 10.43480674 | 0.573720097 | 0.054981382 | -4.184913015 | 0.000822717 | 0.006012231 | K08241 |
| *TCM_000168* | 10.39142024 | 0.610986577 | 0.058797216 | -4.088108351 | 0.000824259 | 0.006021802 | K08241 |
| *HIPP22* | 102.0558604 | 46.2428636 | 0.45311326 | -1.142056384 | 0.000830774 | 0.006062573 | - |
| *CYP86B1* | 55.20865626 | 24.02267957 | 0.435125236 | -1.200497404 | 0.000841476 | 0.006126875 | K15402 |
| *TOGT1* | 46.58704112 | 19.02184572 | 0.408307659 | -1.292271463 | 0.000844319 | 0.006145847 | K13496 |
| *LAC3* | 10.34441917 | 0.573720097 | 0.055461799 | -4.172361775 | 0.000845866 | 0.006153648 | K05909 |
| *TUR2* | 45.42640323 | 18.1804822 | 0.400218395 | -1.321140616 | 0.000848356 | 0.006170037 | - |
| *PXG* | 21.08071103 | 4.7546172 | 0.225543493 | -2.14852243 | 0.000849242 | 0.006174744 | K17991 |
| *HSL2* | 16.93363211 | 2.619006917 | 0.154663034 | -2.692799676 | 0.000859006 | 0.006240493 | - |
| *PAP4* | 32.23831407 | 10.53243659 | 0.326705564 | -1.613937068 | 0.000860955 | 0.006252895 | K14379 |
| *LRK10L-2.5* | 11.39762492 | 1.051431256 | 0.092250031 | -3.438306789 | 0.000868263 | 0.006298917 | - |
| *AVT6A* | 42.77745253 | 16.46724176 | 0.384951436 | -1.377251644 | 0.000870692 | 0.006314773 | K14207 |
| *GGPS1* | 9.023817404 | 0.286860049 | 0.031789212 | -4.975318914 | 0.000886652 | 0.006425131 | K13789 |
| *PME53* | 8.991278496 | 0.286860049 | 0.031904256 | -4.97010731 | 0.000896856 | 0.006486386 | K01051 |
| *LRK10* | 9.997483179 | 0.286860049 | 0.028693226 | -5.123145988 | 0.000903166 | 0.006528375 | - |
| *DREB3* | 25.00392872 | 6.782946752 | 0.27127524 | -1.882170722 | 0.000925542 | 0.006669666 | K09286 |
| *ag1* | 210.5053344 | 102.8575793 | 0.488622199 | -1.033208686 | 0.000927089 | 0.006678953 | K16086 |
| *At4g15970* | 65.98073573 | 31.19639094 | 0.472810595 | -1.080665729 | 0.000930771 | 0.006701756 | - |
| *PUB26* | 7.388818564 | 0 | 0 | -Inf | 0.00093121 | 0.006703055 | - |
| *RPV1* | 14.0977093 | 2.102862512 | 0.149163418 | -2.745034329 | 0.000951095 | 0.006819691 | - |
| *XTH5* | 54.22001416 | 24.79849835 | 0.45736798 | -1.128572728 | 0.000953221 | 0.006829267 | K08235 |
| *ZAT9* | 24.30282373 | 6.382027908 | 0.262604378 | -1.929037127 | 0.000955381 | 0.006840958 | - |
| *uncharacterized protein_46533* | 75.54022904 | 29.27993501 | 0.387607178 | -1.367332807 | 0.000959947 | 0.00686418 | - |
| *BGLU40* | 62.80045932 | 28.95235738 | 0.461021427 | -1.11709429 | 0.000979219 | 0.006984647 | K01188 |
| *CCOAOMT* | 35.32613854 | 12.63755846 | 0.357739594 | -1.483018293 | 0.000988677 | 0.007044359 | K00588 |
| *ALIS5* | 11.41880208 | 0.860580146 | 0.075365186 | -3.729957942 | 0.00098982 | 0.007050563 | - |
| *HSP21* | 33.91168291 | 11.35123155 | 0.334729231 | -1.578933551 | 0.000994876 | 0.007082686 | K13993 |
| *NPF5.2* | 41.70373592 | 13.41682758 | 0.321717642 | -1.636133044 | 0.001002546 | 0.007127506 | - |
| *At1g56130* | 8.142089412 | 0 | 0 | -Inf | 0.001025722 | 0.007274325 | - |
| *PLDDELTA* | 51.57364586 | 22.74308152 | 0.440982621 | -1.181206294 | 0.001028438 | 0.007289598 | K01115 |
| *At5g07050* | 10.17515791 | 0.687778892 | 0.067593928 | -3.886962533 | 0.001031649 | 0.007310359 | - |
| *RUN1* | 33.04530099 | 11.36024399 | 0.343777894 | -1.540451316 | 0.001032685 | 0.007315698 | - |
| *CYP720B2* | 28.42164461 | 9.078919728 | 0.319436818 | -1.646397487 | 0.001036425 | 0.007340189 | - |
| *JOX1* | 63.32853633 | 30.64747378 | 0.483944136 | -1.047087576 | 0.001040878 | 0.007366887 | - |
| *LRK10L-1.4* | 10.12815684 | 0.687778892 | 0.067907607 | -3.880283 | 0.00104625 | 0.007398896 | - |
| *ag1* | 13.1751772 | 1.662417833 | 0.126178025 | -2.986467425 | 0.001046427 | 0.007398896 | K16086 |
| *PUB9* | 28.87534643 | 9.063129225 | 0.31387084 | -1.671757095 | 0.00104681 | 0.007399588 | - |
| *At3g47110* | 37.58026827 | 13.6686805 | 0.36371961 | -1.459101385 | 0.001064834 | 0.00751266 | - |
| *GOX* | 12.24489792 | 1.415083619 | 0.115565163 | -3.113221535 | 0.001067604 | 0.007530154 | K11517 |
| *At3g47570* | 20.85411913 | 1.29876547 | 0.062278606 | -4.005119536 | 0.001108852 | 0.007782159 | - |
| *MES13* | 24.06176967 | 6.423813097 | 0.266971764 | -1.905240929 | 0.001121167 | 0.007857688 | - |
| *CRK7* | 11.33771189 | 0.573720097 | 0.050602811 | -4.304638648 | 0.001121315 | 0.007857688 | - |
| *At2g24130* | 31.90753874 | 8.919723801 | 0.279549102 | -1.838826384 | 0.001137156 | 0.007955797 | - |
| *At1g56140* | 18.63422679 | 3.935822244 | 0.21121468 | -2.243217987 | 0.001139009 | 0.007962316 | - |
| *VIT_06s0061g00120* | 11.03106199 | 1.051431256 | 0.095315506 | -3.391145257 | 0.001144746 | 0.007995955 | - |
| *CCNB1* | 23.20674949 | 6.246493135 | 0.269167086 | -1.893426089 | 0.001147829 | 0.008015325 | K05868 |
| *nep2* | 8.776048351 | 0.324126529 | 0.036933084 | -4.758942463 | 0.001150075 | 0.008028847 | - |
| *PAD4* | 54.41318324 | 24.12884349 | 0.443437455 | -1.173197462 | 0.001154767 | 0.008055099 | - |
| *CPR5* | 52.24530849 | 23.01189172 | 0.440458529 | -1.182921906 | 0.001164954 | 0.008119604 | - |
| *SGS3* | 21.92281924 | 5.640025266 | 0.257267334 | -1.958659808 | 0.001166882 | 0.008129102 | - |
| *uncharacterized protein_36414* | 21.1277121 | 5.429957533 | 0.257006414 | -1.960123728 | 0.001176684 | 0.00818596 | - |
| *R53* | 19.26043856 | 4.189934521 | 0.217540972 | -2.20064095 | 0.001180437 | 0.008207661 | K00430 |
| *PDX1* | 61.95576871 | 29.3589125 | 0.473868909 | -1.077440089 | 0.00118922 | 0.008259873 | K06215 |
| *uncharacterized protein_40685* | 19.19122814 | 4.145889977 | 0.216030467 | -2.210693303 | 0.001199182 | 0.008313472 | - |
| *DRT100* | 36.13260743 | 12.86684282 | 0.35610059 | -1.48964327 | 0.001200986 | 0.008323756 | - |
| *At4g27220* | 20.6647129 | 4.647336469 | 0.22489238 | -2.152693315 | 0.00121714 | 0.008413228 | K13459 |
| *GLIP6* | 60.25347554 | 25.75173549 | 0.427390043 | -1.226374798 | 0.001218576 | 0.008420905 | - |
| *ROQ1* | 18.43021012 | 3.633171693 | 0.197131322 | -2.342771069 | 0.001226674 | 0.008465233 | - |
| *RUN1* | 9.006772846 | 0.324126529 | 0.035986977 | -4.796381266 | 0.00122695 | 0.008465233 | - |
| *GLR2.8* | 35.59161846 | 8.010605382 | 0.225069995 | -2.151554358 | 0.001230316 | 0.008485754 | K05387 |
| *At1g56130* | 195.8628732 | 96.12314587 | 0.490767568 | -1.026888183 | 0.001230578 | 0.008485754 | - |
| *PAD4* | 35.56372631 | 12.72678934 | 0.357858713 | -1.482537988 | 0.001242339 | 0.008557746 | - |
| *LRK10* | 31.87949831 | 11.23826534 | 0.352523281 | -1.504209557 | 0.001249071 | 0.008599555 | - |
| *RPP13L4* | 7.088883653 | 0 | 0 | -Inf | 0.001281741 | 0.008794122 | K13459 |
| *RNS1* | 24.87945202 | 7.037059029 | 0.282846223 | -1.82191019 | 0.00129416 | 0.00886525 | K01166 |
| *XTH16* | 8.365066731 | 0 | 0 | -Inf | 0.001298011 | 0.008882246 | K08235 |
| *SPAC24B11.05* | 7.049629753 | 0 | 0 | -Inf | 0.001302905 | 0.008903986 | K07025 |
| *LYK5* | 35.49178522 | 11.02936438 | 0.310758231 | -1.686135492 | 0.001315698 | 0.008979578 | - |
| *GSVIVT00023967001* | 7.02070542 | 0 | 0 | -Inf | 0.001318795 | 0.008995978 | K00430 |
| *CYP76B10* | 7.018123028 | 0 | 0 | -Inf | 0.001320226 | 0.009003371 | - |
| *At2g19130* | 7.012958245 | 0 | 0 | -Inf | 0.001323094 | 0.009018186 | - |
| *ANR* | 63.33266893 | 30.8078115 | 0.486444232 | -1.039653677 | 0.00133963 | 0.009116514 | K13082 |
| *ZOG1* | 28.583825 | 6.209226654 | 0.217228683 | -2.202713486 | 0.001340052 | 0.009116994 | K13495 |
| *DAZAP1* | 12.86078012 | 1.620632644 | 0.126013557 | -2.988349146 | 0.001342792 | 0.009132241 | - |
| *At5g63180* | 25.84950322 | 4.474535215 | 0.173099466 | -2.530326817 | 0.001355644 | 0.009210991 | K01728 |
| *LRK10* | 9.843716276 | 0.648253058 | 0.065854504 | -3.924574065 | 0.001383421 | 0.009384964 | - |
| *PAT18* | 33.20748137 | 7.636748834 | 0.229970733 | -2.120477825 | 0.001391724 | 0.009424034 | K20027 |
| *DCR* | 31.7717003 | 11.35232414 | 0.357309305 | -1.484754612 | 0.001411059 | 0.009540036 | - |
| *At3g47570* | 9.678953501 | 0.727304727 | 0.07514291 | -3.734219212 | 0.001419125 | 0.009584549 | - |
| *CRK2* | 24.46913671 | 5.006470123 | 0.204603464 | -2.289097527 | 0.001420922 | 0.009594184 | - |
| *GSO1* | 15.17828918 | 2.832426593 | 0.186610399 | -2.421898715 | 0.001424133 | 0.009613361 | - |
| *LTPG5* | 25.98120907 | 7.375883472 | 0.283893003 | -1.816580801 | 0.001460199 | 0.009828674 | - |
| *PLP6* | 26.66991626 | 7.880681905 | 0.295489563 | -1.75882092 | 0.001484202 | 0.009969531 | - |
| *At5g03810* | 36.05823223 | 13.36600498 | 0.370678321 | -1.431760353 | 0.001485667 | 0.009976784 | - |
| *ROQ1* | 15.0946166 | 2.639316129 | 0.174851485 | -2.515798046 | 0.001495475 | 0.010037455 | - |
| *At1g06840* | 15.13542071 | 2.560264459 | 0.169157139 | -2.563564033 | 0.001505686 | 0.010100756 | - |
| *uncharacterized protein_21996* | 38.15534636 | 15.13682111 | 0.3967156 | -1.333822967 | 0.001508166 | 0.010112163 | - |
| *uncharacterized protein_43345* | 23.80661932 | 7.017842405 | 0.294785342 | -1.762263308 | 0.001540229 | 0.010300508 | - |
| *ACA7* | 58.02738624 | 27.02567304 | 0.465739969 | -1.102403399 | 0.001544606 | 0.01032179 | K01674 |
| *AVT6A* | 13.37594518 | 1.951537236 | 0.145899016 | -2.776957944 | 0.001588531 | 0.010582588 | K14207 |
| *GATL7* | 8.402770423 | 0.286860049 | 0.034138746 | -4.872446107 | 0.001597795 | 0.010633377 | - |
| *At5g24080* | 15.84788745 | 2.844865153 | 0.179510686 | -2.477858364 | 0.001598221 | 0.010633482 | - |
| *WAV3* | 17.28728309 | 3.80480618 | 0.220092779 | -2.183816281 | 0.001618642 | 0.010747286 | - |
| *ROQ1* | 16.64144438 | 3.208517517 | 0.192802827 | -2.374801891 | 0.001627018 | 0.010797368 | K19613 |
| *uncharacterized protein_14209* | 54.2023033 | 23.79567955 | 0.439016021 | -1.187654505 | 0.001631914 | 0.010821553 | - |
| *At3g47570* | 13.35890062 | 1.835219087 | 0.137378003 | -2.863777079 | 0.001632458 | 0.01082239 | - |
| *CHI4* | 106.359589 | 47.22425639 | 0.444005631 | -1.171350123 | 0.001653552 | 0.01094512 | K01183 |
| *agn1* | 21.34722313 | 5.565492306 | 0.260712706 | -1.9394672 | 0.001673317 | 0.011048067 | K08254 |
| *EFR* | 12.51487632 | 1.664677188 | 0.133015872 | -2.910329689 | 0.001700348 | 0.011194836 | K13420 |
| *ERF2* | 9.663459151 | 0 | 0 | -Inf | 0.001705275 | 0.01122192 | K09286 |
| *CRRSP38* | 6.834399608 | 0 | 0 | -Inf | 0.001731921 | 0.011374175 | - |
| *At2g42960* | 64.36536384 | 31.37254413 | 0.487413451 | -1.036782029 | 0.001738293 | 0.011410245 | - |
| *AVT6A* | 17.03368295 | 3.5349034 | 0.207524316 | -2.268647707 | 0.001748927 | 0.011471332 | K14207 |
| *ag4* | 6.800310492 | 0 | 0 | -Inf | 0.001756424 | 0.011517589 | K18108 |
| *ADH1* | 6.7977281 | 0 | 0 | -Inf | 0.001758298 | 0.011524055 | - |
| *IGS1* | 6.7977281 | 0 | 0 | -Inf | 0.001758298 | 0.011524055 | - |
| *SULTR3;1* | 12.43636852 | 1.585625518 | 0.127499078 | -2.971441282 | 0.001759652 | 0.01153001 | K17471 |
| *uncharacterized protein_31688* | 12.35166375 | 1.664677188 | 0.134773519 | -2.89139104 | 0.001761828 | 0.011541354 | - |
| *LRK10L-1.4* | 49.37374635 | 21.7210461 | 0.439931091 | -1.184650531 | 0.001764429 | 0.011552554 | - |
| *CYCA2-2* | 19.9026627 | 4.759135909 | 0.239120563 | -2.064189896 | 0.001765836 | 0.01155593 | K06627 |
| *PLDALPHA1* | 6.733682467 | 0 | 0 | -Inf | 0.001805665 | 0.011777931 | K01115 |
| *EB1A* | 57.18682823 | 20.93273956 | 0.366041276 | -1.449921756 | 0.001814293 | 0.011816376 | K10436 |
| *TPS5* | 73.63266941 | 32.44326619 | 0.44060967 | -1.182426936 | 0.001827417 | 0.011898864 | K15086 |
| *TBL16* | 14.86130971 | 2.597530939 | 0.174784793 | -2.516348426 | 0.001842753 | 0.011983673 | - |
| *rumi* | 9.51227464 | 0.610986577 | 0.064231385 | -3.960577778 | 0.001851474 | 0.012031331 | - |
| *uncharacterized protein_44316* | 14.72547127 | 2.676582609 | 0.181765497 | -2.459849721 | 0.001861107 | 0.01208182 | - |
| *NPF5.2* | 11.47355033 | 1.301024824 | 0.113393395 | -3.140591488 | 0.001868126 | 0.012118071 | - |
| *GATA9* | 52.85550788 | 23.751635 | 0.449369157 | -1.154026989 | 0.001872613 | 0.012141311 | - |
| *KOR* | 10.49626998 | 1.051431256 | 0.100171895 | -3.319450301 | 0.001881382 | 0.012185977 | - |
| *IGS1* | 9.41413989 | 0.650512412 | 0.069099506 | -3.855180801 | 0.001883717 | 0.012198053 | - |
| *SRG1* | 10.47509282 | 1.014164776 | 0.096816782 | -3.368599051 | 0.001910124 | 0.01234439 | - |
| *DLO1* | 10.48439021 | 0.974638941 | 0.092960956 | -3.427231278 | 0.001925212 | 0.012429508 | - |
| *uncharacterized protein_35696* | 53.53285331 | 25.03570256 | 0.46766987 | -1.096437609 | 0.001948706 | 0.012565543 | - |
| *ROQ1* | 10.4084648 | 0.972379586 | 0.093421999 | -3.420093877 | 0.001964956 | 0.01266088 | - |
| *CCOAOMT* | 87.84312391 | 34.29884369 | 0.390455646 | -1.356769421 | 0.001977461 | 0.012728802 | K00588 |
| *LOC_Os07g01090* | 61.62277687 | 29.78244911 | 0.483302613 | -1.049001299 | 0.002024857 | 0.012999872 | - |
| *At3g47110* | 8.15500137 | 0.363652364 | 0.044592557 | -4.487053261 | 0.002043829 | 0.013097481 | - |
| *NFP* | 31.86503614 | 11.57708979 | 0.363316386 | -1.460701659 | 0.002049025 | 0.013124286 | - |
| *RBOHF* | 56.26599462 | 27.30118712 | 0.485216467 | -1.043299583 | 0.002049635 | 0.013124691 | K13447 |
| *At4g33300* | 26.97140138 | 8.545892232 | 0.316850137 | -1.658127454 | 0.0020501 | 0.013124691 | - |
| *CYP716B2* | 18.79160827 | 4.82123031 | 0.256562942 | -1.962615291 | 0.002067967 | 0.013222234 | - |
| *ZAR1* | 17.47875369 | 2.945392801 | 0.168512747 | -2.569070366 | 0.002073936 | 0.013247849 | K13420 |
| *NPC6* | 15.41314628 | 2.619006917 | 0.169920331 | -2.557069613 | 0.002086385 | 0.013314248 | K01114 |
| *ABCG39* | 17.00697513 | 3.616214424 | 0.212631253 | -2.233574435 | 0.002090772 | 0.013332402 | - |
| *ag4* | 8.110582687 | 0.324126529 | 0.039963408 | -4.645176554 | 0.002102622 | 0.0133915 | K18108 |
| *At4g01130* | 24.03439554 | 6.953488651 | 0.289314064 | -1.789291638 | 0.002117215 | 0.013477816 | - |
| *YSL7* | 41.39332316 | 16.9664289 | 0.409883228 | -1.286715139 | 0.002118153 | 0.013480479 | - |
| *CYP76T24* | 19.41265525 | 4.877713413 | 0.251264618 | -1.992720561 | 0.00213508 | 0.013576013 | - |
| *PAD4* | 29.74224637 | 10.36415405 | 0.348465745 | -1.520911251 | 0.00213751 | 0.013583665 | - |
| *GLYI4* | 12.04088125 | 1.818261818 | 0.151007371 | -2.727309126 | 0.002159059 | 0.013713884 | - |
| *AMY1.3* | 17.46325934 | 3.672697528 | 0.210309969 | -2.249410858 | 0.002196391 | 0.013930535 | K01176 |
| *At1g56140* | 76.74440171 | 20.63455776 | 0.268873785 | -1.894998995 | 0.002233101 | 0.014139157 | - |
| *LP2* | 66.91654566 | 32.01853784 | 0.478484619 | -1.063455546 | 0.002241592 | 0.014188067 | K07252 |
| *PLL5* | 27.25997454 | 9.128624768 | 0.334872828 | -1.578314776 | 0.002246148 | 0.014211351 | - |
| *At2g24130* | 14.59133132 | 2.599790294 | 0.178173618 | -2.48864436 | 0.002265466 | 0.014312626 | - |
| *PME2* | 42.50319325 | 17.98511238 | 0.423147322 | -1.24076806 | 0.002271175 | 0.014341702 | K01051 |
| *RPS5* | 58.93442399 | 26.0566454 | 0.442129466 | -1.177459209 | 0.002281054 | 0.014390066 | - |
| *ATPK2* | 63.80496551 | 31.81529737 | 0.498633564 | -1.003948098 | 0.002309918 | 0.014547381 | K04688 |
| *uncharacterized protein_19830* | 57.29544083 | 28.21144732 | 0.492385553 | -1.022139662 | 0.002328988 | 0.014660358 | - |
| *PYL4* | 6.656724875 | 0 | 0 | -Inf | 0.002338516 | 0.014709619 | K14496 |
| *WRKY51* | 9.21078952 | 0.727304727 | 0.078962257 | -3.662692967 | 0.00234665 | 0.014753624 | - |
| *At5g48740* | 59.87584742 | 24.39991304 | 0.407508438 | -1.295098163 | 0.002358134 | 0.01480787 | - |
| *KIN14R* | 14.42915093 | 2.446205664 | 0.169532197 | -2.5603688 | 0.002391918 | 0.015001849 | K10406 |
| *CSPL6* | 24.26770243 | 7.358926203 | 0.30323951 | -1.721470354 | 0.00240618 | 0.015084001 | - |
| *At5g48900* | 6.48074863 | 0 | 0 | -Inf | 0.002438574 | 0.01526862 | K01728 |
| *TUR2* | 44.38920984 | 19.89821637 | 0.448266965 | -1.157569911 | 0.002450533 | 0.015339795 | - |
| *N* | 6.466286464 | 0 | 0 | -Inf | 0.002453147 | 0.015352447 | - |
| *uncharacterized protein_38459* | 33.68442471 | 12.75953711 | 0.378796349 | -1.400505668 | 0.00247979 | 0.015493012 | - |
| *XTH5* | 25.24240039 | 8.507458985 | 0.337030506 | -1.569048912 | 0.00249776 | 0.015591253 | K08235 |
| *EDS1B* | 20.59653467 | 5.258323046 | 0.255301347 | -1.969726945 | 0.00249792 | 0.015591253 | K18875 |
| *CML17* | 10.09148533 | 1.011905421 | 0.10027319 | -3.317992177 | 0.002520579 | 0.015702444 | K13448 |
| *BGLU12* | 23.60843373 | 6.91622217 | 0.29295557 | -1.771246212 | 0.002525189 | 0.015727384 | K01188 |
| *At2g27500* | 17.31878982 | 3.861289283 | 0.222953759 | -2.165183574 | 0.002535636 | 0.01578487 | - |
| *uncharacterized protein_44222* | 6.377449097 | 0 | 0 | -Inf | 0.00254529 | 0.015837363 | - |
| *BEBT* | 11.22164868 | 0.860580146 | 0.076689279 | -3.704831285 | 0.002549214 | 0.015857969 | K19861 |
| *NAC018* | 12.81894383 | 1.795693252 | 0.140081217 | -2.835664572 | 0.002550014 | 0.015859142 | - |
| *Gvin1* | 13.51333383 | 2.545566545 | 0.188374429 | -2.408324955 | 0.002553837 | 0.015879112 | - |
| *CESA5* | 10.04809883 | 0 | 0 | -Inf | 0.002555966 | 0.015888537 | - |
| *MOCS2* | 34.62245116 | 13.11191768 | 0.378711421 | -1.400829165 | 0.002580597 | 0.016026282 | K03635 |
| *PR1* | 16.62181743 | 3.34405229 | 0.201184516 | -2.313408823 | 0.002588379 | 0.016070761 | - |
| *WRKY43* | 10.06152881 | 0.860580146 | 0.085531748 | -3.547396167 | 0.002641846 | 0.016363543 | - |
| *erkA* | 37.65914195 | 15.16847629 | 0.402783375 | -1.311923958 | 0.002653229 | 0.016428601 | K04371 |
| *At1g11050* | 49.14715445 | 22.44383212 | 0.456665953 | -1.130788863 | 0.00268321 | 0.016584094 | - |
| *PARC* | 56.22984114 | 27.71454452 | 0.492879652 | -1.020692673 | 0.002745099 | 0.016930287 | K00799 |
| *Daglb* | 62.73656196 | 27.76876827 | 0.442624961 | -1.175843285 | 0.002748877 | 0.016945527 | - |
| *PT10* | 14.22063578 | 2.639316129 | 0.185597618 | -2.4297499 | 0.002812009 | 0.017289421 | K12742 |
| *PUB23* | 20.84172133 | 5.891878189 | 0.282696333 | -1.822674927 | 0.002833709 | 0.017410522 | - |
| *uncharacterized protein_26016* | 32.344196 | 11.6223011 | 0.359331891 | -1.476611115 | 0.00285721 | 0.017538292 | - |
| *TPS-mISO1* | 22.10499245 | 5.886266892 | 0.266286763 | -1.908947379 | 0.002863085 | 0.017561878 | K04120 |
| *TAR4* | 35.25796031 | 14.41403509 | 0.408816476 | -1.290474754 | 0.0028823 | 0.017650516 | - |
| *SRC2* | 20.88614001 | 5.619716055 | 0.269064368 | -1.893976748 | 0.002901816 | 0.017748756 | - |
| *ROQ1* | 21.03385823 | 4.218163584 | 0.2005416 | -2.318026557 | 0.00290245 | 0.017748756 | - |
| *BHLH30* | 28.89239098 | 10.23879848 | 0.354376988 | -1.496643173 | 0.002917302 | 0.017831165 | - |
| *TUBB4* | 41.27401125 | 18.57571557 | 0.450058403 | -1.151815868 | 0.002918083 | 0.017831734 | K07375 |
| *MUCI21* | 35.73283928 | 12.42749073 | 0.347789064 | -1.523715526 | 0.002922384 | 0.017849607 | - |
| *UGT85A23* | 7.720260201 | 0.286860049 | 0.037156785 | -4.750230511 | 0.002932038 | 0.017895926 | - |
| *At4g33300* | 46.38302444 | 20.66735549 | 0.445580161 | -1.166243096 | 0.002940446 | 0.017926143 | - |
| *CRK2* | 7.703215642 | 0.286860049 | 0.037239 | -4.747041851 | 0.002949092 | 0.017966178 | - |
| *Os02g0190300* | 75.22686415 | 31.92481323 | 0.424380487 | -1.236569773 | 0.002983029 | 0.018153682 | K05658 |
| *STAD* | 40.26419199 | 17.42040472 | 0.432652535 | -1.20871924 | 0.002988541 | 0.01817235 | K03921 |
| *RUN1* | 59.47003058 | 29.36683235 | 0.493808933 | -1.01797516 | 0.002994564 | 0.018200436 | - |
| *At1g67820* | 16.81225584 | 4.131192063 | 0.24572503 | -2.024883277 | 0.003041358 | 0.018435315 | K17506 |
| *SCPL35* | 18.83757716 | 4.875454059 | 0.258815346 | -1.950004931 | 0.003048341 | 0.018470997 | K16297 |
| *At1g56130* | 91.29839522 | 43.43973368 | 0.475799532 | -1.071574243 | 0.003126975 | 0.018858702 | - |
| *EIX1* | 40.84200075 | 14.47161078 | 0.354331583 | -1.49682803 | 0.003158984 | 0.019021399 | - |
| *LRK10L-2.1* | 12.39453222 | 1.912011402 | 0.15426249 | -2.696540793 | 0.003192688 | 0.019210955 | - |
| *nep1* | 61.4710782 | 30.23637573 | 0.491879704 | -1.023622568 | 0.003208541 | 0.019301867 | - |
| *ACT7* | 32.93115386 | 12.98539534 | 0.394319476 | -1.342563125 | 0.003210328 | 0.019308137 | K10355 |
| *UGT86A2* | 32.98900253 | 12.87811461 | 0.390375993 | -1.357063762 | 0.003219247 | 0.019357291 | - |
| *CYP720B2* | 25.95383494 | 8.622684547 | 0.332231617 | -1.589738721 | 0.003228733 | 0.019400833 | - |
| *PLP7* | 8.798257693 | 0.648253058 | 0.073679708 | -3.762588846 | 0.003244965 | 0.019489336 | - |
| *At2g24130* | 10.38522327 | 0.650512412 | 0.062638269 | -3.996811839 | 0.003272332 | 0.019617785 | - |
| *VIT_06s0061g00120* | 8.697540551 | 0.687778892 | 0.0790774 | -3.660590754 | 0.003302605 | 0.019762278 | - |
| *TOGT1* | 9.801879985 | 0.935113106 | 0.095401403 | -3.389845701 | 0.003303858 | 0.01976521 | K13496 |
| *BHLH* | 16.36888359 | 2.639316129 | 0.161239837 | -2.632719861 | 0.003318085 | 0.019836592 | - |
| *PROT1* | 9.79413281 | 0.897846626 | 0.091671886 | -3.447376836 | 0.003341953 | 0.019956279 | - |
| *MIK1* | 9.73525196 | 0.937372461 | 0.09628641 | -3.376524003 | 0.003358511 | 0.020044922 | - |
| *EO* | 21.16541579 | 5.864790914 | 0.277093111 | -1.85155725 | 0.003381057 | 0.020152654 | K18980 |
| *GAPB* | 20.54591902 | 5.736034205 | 0.279181194 | -1.840726332 | 0.003408444 | 0.020301891 | K05298 |
| *PCMP-H32* | 28.55681675 | 10.01294025 | 0.350632227 | -1.511969494 | 0.003418305 | 0.02033727 | - |
| *FAB1A* | 44.36803269 | 20.31609248 | 0.457899331 | -1.126897638 | 0.003444996 | 0.020476058 | K00921 |
| *T5AT* | 43.58443944 | 19.67235813 | 0.451361963 | -1.14764325 | 0.003448597 | 0.020489283 | K19861 |
| *CRK2* | 6.107470703 | 0 | 0 | -Inf | 0.003462038 | 0.020542738 | - |
| *uncharacterized protein_10571* | 34.36177015 | 14.05264208 | 0.40896153 | -1.289962957 | 0.003462813 | 0.020542738 | K13464 |
| *LRK10L-2.6* | 32.81302628 | 11.42693128 | 0.348243749 | -1.52183064 | 0.003470295 | 0.020571058 | - |
| *R12* | 24.56350474 | 8.12349741 | 0.330714102 | -1.59634353 | 0.003525606 | 0.0208465 | K00430 |
| *ABCC9* | 53.511162 | 25.81047795 | 0.482338207 | -1.051883 | 0.003541959 | 0.020933648 | - |
| *CAT1* | 6.026380511 | 0 | 0 | -Inf | 0.003580741 | 0.021124336 | K03294 |
| *At3g09930* | 6.023798119 | 0 | 0 | -Inf | 0.003584612 | 0.021142363 | - |
| *GSO1* | 33.42824218 | 11.6482216 | 0.348454506 | -1.520957785 | 0.003626852 | 0.02135264 | - |
| *SDR1* | 17.29399808 | 4.378526277 | 0.253181841 | -1.98175416 | 0.003640415 | 0.021417901 | K00059 |
| *BAG6* | 43.78365721 | 19.79659614 | 0.452145787 | -1.145140075 | 0.003675855 | 0.021606792 | - |
| *NAKR2* | 60.41012526 | 22.81875627 | 0.377730656 | -1.404570218 | 0.003697585 | 0.021719753 | - |
| *At2g19130* | 35.69011908 | 15.17634694 | 0.42522545 | -1.23370015 | 0.003706632 | 0.021758104 | - |
| *JAL3* | 8.142089412 | 0.286860049 | 0.035231749 | -4.826980104 | 0.003723475 | 0.021832259 | - |
| *TCP14* | 49.58602822 | 23.76064744 | 0.479180291 | -1.061359524 | 0.003738964 | 0.021913166 | - |
| *CRK8* | 37.86574102 | 16.09341019 | 0.425012419 | -1.234423097 | 0.003761476 | 0.022025191 | - |
| *XTH* | 45.29433151 | 16.6355243 | 0.367276075 | -1.445063175 | 0.003789043 | 0.022171587 | K08235 |
| *PT5* | 12.31669073 | 0.363652364 | 0.029525168 | -5.081910919 | 0.003804148 | 0.022244915 | K12742 |
| *At5g07050* | 7.425490073 | 0.363652364 | 0.048973517 | -4.351854387 | 0.003805773 | 0.022249397 | - |
| *PME2* | 25.59346897 | 8.466840563 | 0.330820358 | -1.595880078 | 0.003806807 | 0.022250429 | K01051 |
| *At5g48740* | 7.420325289 | 0.363652364 | 0.049007604 | -4.350850574 | 0.003812314 | 0.02227759 | - |
| *At3g47570* | 9.275719057 | 0 | 0 | -Inf | 0.003820893 | 0.022307615 | - |
| *CHI4* | 25.11497542 | 8.261291538 | 0.328938866 | -1.604108614 | 0.003908328 | 0.02277194 | K01183 |
| *ABCG36* | 15.35493173 | 1.832959732 | 0.119372705 | -3.066455101 | 0.003934034 | 0.022901128 | - |
| *N* | 9.584067448 | 0.286860049 | 0.02993093 | -5.0622191 | 0.003947416 | 0.022955237 | - |
| *PR4B* | 30.5032644 | 11.26651938 | 0.369354546 | -1.436921759 | 0.003972585 | 0.023078915 | - |
| *uncharacterized protein_47086* | 7.332520106 | 0.324126529 | 0.044203974 | -4.499680111 | 0.003978466 | 0.023107901 | - |
| *CYP750A1* | 7.324772931 | 0.324126529 | 0.044250727 | -4.498155025 | 0.003988887 | 0.023152868 | - |
| *XTH32* | 52.19985762 | 25.07639516 | 0.480392022 | -1.057715902 | 0.003997604 | 0.023193082 | K08235 |
| *SCP1* | 10.45288348 | 1.259239635 | 0.12046816 | -3.053276211 | 0.004008041 | 0.023248431 | K16297 |
| *AIR3* | 10.4084648 | 1.29876547 | 0.124779734 | -3.002544455 | 0.00401248 | 0.023268972 | - |
| *HSP21* | 20.76373156 | 5.076484375 | 0.244488057 | -2.032164105 | 0.00406701 | 0.023548332 | - |
| *uncharacterized protein_26689* | 9.918091476 | 0.324126529 | 0.032680333 | -4.935433521 | 0.00407534 | 0.02358603 | - |
| *XTH32* | 19.62080453 | 5.560973597 | 0.283422303 | -1.818974806 | 0.004080776 | 0.023606951 | K08235 |
| *TIFY10B* | 44.73784432 | 14.79581149 | 0.330722495 | -1.596306918 | 0.00410216 | 0.023708373 | K13464 |
| *HPCA1* | 42.42380155 | 19.49277882 | 0.459477418 | -1.121934135 | 0.00410288 | 0.023708373 | - |
| *T5AT* | 12.78124014 | 2.38972256 | 0.186971103 | -2.419112784 | 0.004137003 | 0.023868317 | K15400 |
| *CRK2* | 8.700122943 | 0.610986577 | 0.07022735 | -3.831823197 | 0.004168599 | 0.024029223 | - |
| *BC10* | 39.40260511 | 17.73209269 | 0.450023359 | -1.151928208 | 0.004173951 | 0.024049376 | - |
| *TBL45* | 25.26977451 | 8.734483988 | 0.345649463 | -1.532618411 | 0.004331188 | 0.024844923 | - |
| *IPK1* | 42.674153 | 19.69380913 | 0.461492678 | -1.115620338 | 0.004347955 | 0.024926738 | K10572 |
| *CEPR1* | 68.4836667 | 30.79770647 | 0.449708784 | -1.152937031 | 0.004362968 | 0.024999564 | - |
| *ERF018* | 8.370231515 | 0.650512412 | 0.077717374 | -3.685619038 | 0.004395082 | 0.025138968 | - |
| *EFR* | 11.1379761 | 1.741469503 | 0.156354214 | -2.677109988 | 0.004399111 | 0.025151083 | K13420 |
| *GSTU17* | 11.18652738 | 1.583366164 | 0.141542242 | -2.82069542 | 0.004497693 | 0.025658125 | K00799 |
| *UGT86A2* | 15.84891963 | 3.166732328 | 0.199807457 | -2.323317668 | 0.00450804 | 0.02570019 | - |
| *DTX40* | 34.69321178 | 14.34968133 | 0.413616399 | -1.273634708 | 0.004568473 | 0.026010344 | K03327 |
| *ELI* | 14.12508342 | 2.849383862 | 0.2017251 | -2.309537492 | 0.004592832 | 0.026108955 | - |
| *RPA1B* | 17.51955779 | 4.24415827 | 0.242252591 | -2.045415998 | 0.00462479 | 0.026273347 | K07466 |
| *KAN4* | 29.89962785 | 11.65840081 | 0.389917924 | -1.358757621 | 0.004642307 | 0.026361311 | - |
| *CRK2* | 26.43646109 | 6.935438794 | 0.262343692 | -1.930469996 | 0.004680029 | 0.026523231 | - |
| *FZR2* | 20.04366592 | 5.806048456 | 0.289669987 | -1.787517879 | 0.004684384 | 0.026542112 | K03364 |
| *OLE9* | 31.08350727 | 12.22986155 | 0.393451789 | -1.345741226 | 0.004688106 | 0.026557397 | - |
| *uncharacterized protein_16866* | 32.03238131 | 12.99217341 | 0.405594991 | -1.30188826 | 0.004728214 | 0.026755373 | - |
| *DTX40* | 15.49165408 | 3.34405229 | 0.215861539 | -2.21182188 | 0.004760363 | 0.026896209 | K03327 |
| *At3g02645* | 16.17932908 | 3.593645858 | 0.222113404 | -2.170631636 | 0.004770104 | 0.026945371 | - |
| *mpaB&apos;* | 9.647446776 | 0.286860049 | 0.029734297 | -5.071728219 | 0.004810739 | 0.027127629 | - |
| *At3g47110* | 5.778611458 | 0 | 0 | -Inf | 0.004826561 | 0.027205018 | - |
| *R21* | 11.78794741 | 2.063336677 | 0.175037825 | -2.514261378 | 0.004837172 | 0.027247054 | K00430 |
| *CEL2* | 5.7615669 | 0 | 0 | -Inf | 0.004860619 | 0.027355357 | - |
| *CRJ35* | 5.788941025 | 0 | 0 | -Inf | 0.004872851 | 0.027412296 | - |
| *VIT_06s0061g00120* | 32.92598908 | 13.22145776 | 0.401550815 | -1.316345525 | 0.004896747 | 0.027528806 | - |
| *SDR2a* | 5.739357558 | 0 | 0 | -Inf | 0.0049055 | 0.02756009 | - |
| *OsI_03083* | 32.79531542 | 13.27002102 | 0.404631602 | -1.305319095 | 0.004917342 | 0.027620635 | - |
| *WNK4* | 60.59126243 | 19.62602926 | 0.323908572 | -1.626341449 | 0.00493157 | 0.027673765 | K08867 |
| *CRK8* | 21.07052974 | 5.922366606 | 0.281073456 | -1.830980882 | 0.004934352 | 0.027674213 | - |
| *At3g02645* | 5.709401041 | 0 | 0 | -Inf | 0.004966956 | 0.027814953 | - |
| *EFR* | 6.508122755 | 0 | 0 | -Inf | 0.004968356 | 0.027816787 | - |
| *At3g02645* | 15.34046956 | 3.248043352 | 0.211730374 | -2.23969985 | 0.005001896 | 0.027998524 | - |
| *At3g47570* | 5.6871917 | 0 | 0 | -Inf | 0.005013211 | 0.028049749 | - |
| *uncharacterized protein_08260* | 5.677894316 | 0 | 0 | -Inf | 0.005032753 | 0.028140869 | - |
| *RUN1* | 5.672729533 | 0 | 0 | -Inf | 0.005043654 | 0.028189665 | - |
| *ROQ1* | 48.56277898 | 24.03732828 | 0.494974316 | -1.01457443 | 0.005048369 | 0.02820994 | - |
| *AGL19* | 5.653102583 | 0 | 0 | -Inf | 0.005085381 | 0.028380058 | K09260 |
| *UGT86A2* | 5.6479378 | 0 | 0 | -Inf | 0.005096441 | 0.02843566 | - |
| *At1g80120* | 35.82064446 | 12.65786767 | 0.353367949 | -1.500756903 | 0.005110744 | 0.02850933 | - |
| *CRK2* | 24.29507655 | 8.833844868 | 0.363606381 | -1.459550576 | 0.005126137 | 0.028582897 | - |
| *CYP73A4* | 41.97009974 | 19.19012827 | 0.457233325 | -1.128997537 | 0.005143635 | 0.028650506 | K00487 |
| *LAZ5* | 7.124004953 | 0.324126529 | 0.045497797 | -4.458059508 | 0.005144002 | 0.028650506 | - |
| *IRL1* | 11.68206549 | 1.835219087 | 0.157097141 | -2.670271171 | 0.005147455 | 0.028658623 | - |
| *LRK10* | 7.07958627 | 0.363652364 | 0.05136633 | -4.283033194 | 0.005152296 | 0.028673265 | - |
| *ERF2* | 7.07958627 | 0.363652364 | 0.05136633 | -4.283033194 | 0.005152296 | 0.028673265 | K09286 |
| *YSL12* | 47.64696187 | 23.14514216 | 0.485763231 | -1.041674802 | 0.00517252 | 0.028761118 | - |
| *A6* | 50.75322896 | 24.82454224 | 0.489122421 | -1.031732496 | 0.005181784 | 0.028800279 | - |
| *EDS1* | 20.77251091 | 6.579657081 | 0.316748279 | -1.658591314 | 0.005187811 | 0.028827598 | - |
| *At2g05910* | 13.27331195 | 2.63479742 | 0.19850339 | -2.332764448 | 0.005293844 | 0.029316274 | - |
| *At1g67720* | 7.065124103 | 0.286860049 | 0.040602266 | -4.622295942 | 0.005310389 | 0.029401618 | - |
| *MAPKKK17* | 7.057376928 | 0.286860049 | 0.040646837 | -4.620713104 | 0.005324114 | 0.029452449 | - |
| *LSI2* | 37.91687469 | 16.671649 | 0.439689429 | -1.185443246 | 0.005356338 | 0.029605444 | - |
| *CRK2* | 17.27695352 | 4.437268735 | 0.256831665 | -1.961105012 | 0.005367376 | 0.029647494 | - |
| *CCOAOMT* | 45.36132541 | 21.86445152 | 0.482006452 | -1.052875637 | 0.005415073 | 0.029856486 | K00588 |
| *PRPX* | 7.00107847 | 0.286860049 | 0.040973694 | -4.609158216 | 0.005425317 | 0.029897477 | - |
| *BAM1* | 9.205624737 | 0.935113106 | 0.101580624 | -3.299302852 | 0.005459535 | 0.030028598 | - |
| *RDR1* | 17.71361078 | 4.607810634 | 0.260128253 | -1.942704991 | 0.005462969 | 0.030034746 | K11699 |
| *HB1* | 15.73012575 | 4.087147519 | 0.259829297 | -1.944363982 | 0.005496559 | 0.030168242 | - |
| *TRANK1* | 30.61209459 | 11.84815933 | 0.387041772 | -1.369438816 | 0.00550158 | 0.030189409 | - |
| *ROQ1* | 9.087863037 | 1.014164776 | 0.111595517 | -3.16364902 | 0.005511544 | 0.03023129 | - |
| *ETG1* | 49.22566225 | 24.18982033 | 0.491406702 | -1.025010562 | 0.005550556 | 0.030425964 | - |
| *ag4* | 9.13641432 | 0.935113106 | 0.10235012 | -3.288415295 | 0.005552993 | 0.030432892 | K18108 |
| *At3g02645* | 15.73012575 | 3.97082937 | 0.252434687 | -1.98601793 | 0.005576974 | 0.030538497 | - |
| *NAC022* | 53.71621086 | 23.45010202 | 0.436555402 | -1.195763341 | 0.005685777 | 0.031062066 | - |
| *At4g26790* | 30.92597751 | 8.437444733 | 0.272827099 | -1.873941144 | 0.00568912 | 0.031067291 | - |
| *At3g47570* | 10.84320597 | 1.587884873 | 0.146440534 | -2.771613155 | 0.00569539 | 0.031094261 | - |
| *LRK10* | 14.52470329 | 3.098977431 | 0.213359087 | -2.22864454 | 0.005696457 | 0.031094261 | - |
| *CYP720B2* | 8.085790953 | 0.650512412 | 0.080451302 | -3.635740416 | 0.005709062 | 0.031156509 | - |
| *DHBK* | 65.46955499 | 31.74751749 | 0.484920319 | -1.044180388 | 0.005759035 | 0.031382987 | K00863 |
| *uncharacterized protein_48737* | 8.012447937 | 0.687778892 | 0.085838797 | -3.542226332 | 0.005767133 | 0.031420511 | - |
| *IRL1* | 19.46895371 | 5.610678637 | 0.288185935 | -1.794928167 | 0.005783706 | 0.031497572 | - |
| *At4g28100* | 18.92899692 | 5.405129612 | 0.285547598 | -1.808196844 | 0.005863736 | 0.031884028 | - |
| *NPF3.1* | 10.76728056 | 1.508833203 | 0.140131317 | -2.835148687 | 0.005874816 | 0.031933382 | K14638 |
| *EDS1B* | 42.6715706 | 20.0924936 | 0.4708637 | -1.08661859 | 0.00599984 | 0.032537932 | - |
| *ABCG2* | 20.71584658 | 5.062953227 | 0.244400016 | -2.032683718 | 0.006040503 | 0.032741574 | - |
| *CDC20-2* | 11.47355033 | 2.023810842 | 0.176389242 | -2.503165524 | 0.006083963 | 0.032918402 | K03363 |
| *At3g47110* | 8.016580537 | 0.727304727 | 0.090725057 | -3.46235513 | 0.006206583 | 0.033455731 | - |
| *ILL8* | 29.83868264 | 9.239282421 | 0.309641097 | -1.69133113 | 0.006272125 | 0.033746348 | K14664 |
| *CRK41* | 24.47016889 | 5.965318562 | 0.243779215 | -2.036352971 | 0.006318657 | 0.033968511 | - |
| *PAD4* | 11.80344176 | 1.662417833 | 0.140841787 | -2.827852655 | 0.006348824 | 0.034109471 | - |
| *RTNLB21* | 16.77558433 | 4.665386325 | 0.278105742 | -1.846294563 | 0.006387416 | 0.034302595 | - |
| *At1g30440* | 106.2328958 | 49.95176 | 0.470209907 | -1.08862316 | 0.006404874 | 0.034360762 | - |
| *OsI_017523* | 16.74304542 | 4.66764568 | 0.278781163 | -1.84279501 | 0.006408947 | 0.034375502 | K11251 |
| *LTPG5* | 11.4095047 | 1.797952606 | 0.157583756 | -2.665809266 | 0.006414094 | 0.034395978 | - |
| *CYP720B1* | 11.42654926 | 1.758426772 | 0.153889572 | -2.700032624 | 0.006440689 | 0.034488678 | - |
| *SBT1.4* | 9.713042618 | 1.29876547 | 0.133713556 | -2.902782358 | 0.006597027 | 0.035216744 | - |
| *ROQ1* | 5.474543947 | 0 | 0 | -Inf | 0.006662006 | 0.035511458 | - |
| *XTH5* | 5.469379163 | 0 | 0 | -Inf | 0.006676094 | 0.035572909 | K08235 |
| *At2g19130* | 12.8700775 | 2.525257333 | 0.196211509 | -2.349518425 | 0.006716568 | 0.03575183 | - |
| *GAPB* | 12.14676317 | 2.04528682 | 0.168381222 | -2.570196842 | 0.006743251 | 0.035879134 | K05298 |
| *TCM_034089* | 5.415663097 | 0 | 0 | -Inf | 0.006825158 | 0.036203495 | K08241 |
| *ADC2* | 5.413080705 | 0 | 0 | -Inf | 0.006832442 | 0.036234722 | K01583 |
| *uncharacterized protein_35049* | 5.368662022 | 0 | 0 | -Inf | 0.006959483 | 0.036803073 | - |
| *uncharacterized protein_11699* | 18.55158639 | 5.409648321 | 0.291600309 | -1.777935847 | 0.006975888 | 0.036852244 | - |
| *NPF5.10* | 5.34645268 | 0 | 0 | -Inf | 0.007024259 | 0.03703986 | K14638 |
| *uncharacterized protein_45894* | 5.34645268 | 0 | 0 | -Inf | 0.007024259 | 0.03703986 | - |
| *NAT6* | 33.09230206 | 13.95779991 | 0.42178389 | -1.245424101 | 0.007034785 | 0.037072747 | K14611 |
| *EDS1B* | 41.90243953 | 17.40012049 | 0.415253162 | -1.267936943 | 0.007058475 | 0.03716737 | K18875 |
| *GAPB* | 6.691846175 | 0.363652364 | 0.054342607 | -4.201772425 | 0.007063008 | 0.03718369 | K05298 |
| *CSE* | 8.785345734 | 1.014164776 | 0.115438232 | -3.11480699 | 0.007077083 | 0.037227552 | - |
| *ROQ1* | 8.692375768 | 1.090957091 | 0.125507355 | -2.994156184 | 0.007104657 | 0.03734987 | K19613 |
| *CRK8* | 6.694428567 | 0.324126529 | 0.048417356 | -4.368331902 | 0.007149268 | 0.037553937 | - |
| *UGT86A2* | 17.24028202 | 5.182623319 | 0.300611284 | -1.734028929 | 0.007164022 | 0.037609432 | - |
| *ACR4* | 5.299451605 | 0 | 0 | -Inf | 0.007164185 | 0.037609432 | - |
| *GDI1* | 19.96154355 | 5.852352354 | 0.293181353 | -1.770134745 | 0.007177863 | 0.037673612 | K12462 |
| *At3g01520* | 31.89551069 | 9.861614971 | 0.309185047 | -1.693457546 | 0.007192386 | 0.037734556 | - |
| *PT5* | 6.661889658 | 0.286860049 | 0.043059862 | -4.537512498 | 0.007316284 | 0.038313527 | K12742 |
| *TBL16* | 39.83269952 | 16.07980486 | 0.403683533 | -1.308703358 | 0.007345799 | 0.038422812 | - |
| *GLR3.1* | 43.67327681 | 21.75148531 | 0.498050224 | -1.005636861 | 0.007355934 | 0.038452552 | K05387 |
| *SDR2a* | 7.747634325 | 0.727304727 | 0.093874426 | -3.413124005 | 0.007361419 | 0.038465715 | - |
| *uncharacterized protein_02415* | 38.89673744 | 17.91959186 | 0.460696527 | -1.118111372 | 0.007390156 | 0.038592541 | - |
| *ITIH4* | 10.65106907 | 1.434300243 | 0.134662561 | -2.892579283 | 0.007505087 | 0.039098232 | - |
| *nep1* | 11.23611085 | 1.872485567 | 0.166648905 | -2.585116259 | 0.007684728 | 0.039937788 | - |
| *UGT85K4* | 7.712513026 | 0.573720097 | 0.074388218 | -3.74878206 | 0.007755301 | 0.040250245 | - |
| *At2g27500* | 34.56925312 | 15.4779049 | 0.447736168 | -1.159279233 | 0.007817969 | 0.040565197 | - |
| *BGLU12* | 18.20465041 | 5.595980723 | 0.307392924 | -1.701844139 | 0.008121348 | 0.041854564 | K01188 |
| *PAD4* | 9.441514015 | 1.33603195 | 0.141506113 | -2.821063716 | 0.008233256 | 0.042372341 | - |
| *BPA1* | 38.70873314 | 17.8721212 | 0.461707727 | -1.114948217 | 0.008288172 | 0.042614755 | - |
| *KIN14R* | 13.18189219 | 2.926176177 | 0.221984533 | -2.171468938 | 0.008288576 | 0.042614755 | K10406 |
| *RUN1* | 9.463723357 | 1.261498989 | 0.13329838 | -2.907268851 | 0.008325408 | 0.042770198 | - |
| *GT11* | 28.12429209 | 11.13438575 | 0.395899236 | -1.336794811 | 0.008389687 | 0.043066284 | - |
| *GLR2.8* | 18.7845274 | 3.079760807 | 0.163951998 | -2.608654608 | 0.008504834 | 0.043579712 | K05387 |
| *CDKB1-1* | 27.81697588 | 11.10841605 | 0.399339457 | -1.324312469 | 0.008542419 | 0.043755004 | K07760 |
| *DTX35* | 30.87432581 | 12.89733123 | 0.417736449 | -1.259335065 | 0.008623406 | 0.044100131 | K03327 |
| *PAP2* | 13.18963936 | 2.619006917 | 0.198565468 | -2.332313343 | 0.008649371 | 0.04418934 | - |
| *At4g15970* | 17.5128428 | 5.004210769 | 0.2857452 | -1.807198831 | 0.008697873 | 0.044402136 | - |
| *GH5FP* | 20.94915346 | 7.304702454 | 0.348687238 | -1.519994534 | 0.008716123 | 0.044469034 | - |
| *SCR* | 12.1348834 | 1.548359038 | 0.127595708 | -2.970348294 | 0.008781064 | 0.044754539 | - |
| *FAO2* | 46.27094555 | 19.97056416 | 0.43160052 | -1.212231492 | 0.008823963 | 0.044921994 | K17756 |
| *ABCD1* | 20.75959896 | 7.304702454 | 0.351871078 | -1.50688116 | 0.008844202 | 0.045016187 | - |
| *At5g48740* | 11.27794714 | 1.221973155 | 0.108350672 | -3.206219989 | 0.008873071 | 0.045124473 | - |
| *CYP75A5* | 31.46866497 | 13.57493092 | 0.431379308 | -1.212971118 | 0.008896691 | 0.045194632 | K13083 |
| *OPT4* | 16.78488171 | 4.791883681 | 0.285488082 | -1.808497577 | 0.008991561 | 0.045596167 | - |
| *MAIL3* | 42.66862233 | 20.62668786 | 0.483415839 | -1.048663351 | 0.009033247 | 0.045771747 | - |
| *CDC20-1* | 10.1018149 | 1.662417833 | 0.164566254 | -2.603259571 | 0.009063951 | 0.045873533 | K03363 |
| *CDC20-1* | 8.481278223 | 1.011905421 | 0.119310485 | -3.067207258 | 0.009082154 | 0.045948275 | K03363 |
| *CIPK2* | 18.55830139 | 5.959633086 | 0.32113031 | -1.638769252 | 0.009131831 | 0.046163018 | K07198 |
| *At1g56130* | 8.400188031 | 1.051431256 | 0.125167586 | -2.998067094 | 0.009171429 | 0.046336091 | - |
| *ATJ11* | 8.353186956 | 1.051431256 | 0.125871869 | -2.9899722 | 0.009273852 | 0.046807947 | - |
| *RLK5* | 6.483331022 | 0.286860049 | 0.044245782 | -4.498316274 | 0.009352248 | 0.047139401 | - |
| *NFD4* | 44.12026363 | 21.84414231 | 0.495104528 | -1.014194951 | 0.009491804 | 0.04771297 | - |
| *VIT_06s0061g00120* | 6.394493655 | 0.324126529 | 0.050688381 | -4.302201114 | 0.009509241 | 0.0477723 | - |
| *XA21* | 8.355769348 | 0.937372461 | 0.112182663 | -3.156078354 | 0.009523184 | 0.047824349 | - |
| *GAPB* | 7.460611373 | 0.727304727 | 0.097485942 | -3.358662001 | 0.00954956 | 0.04794752 | K05298 |
| *At1g15890* | 21.89389491 | 7.301350512 | 0.333487967 | -1.584293386 | 0.009584681 | 0.048095916 | K13459 |
| *uncharacterized protein_23700* | 10.77141316 | 1.988803716 | 0.184637214 | -2.437234735 | 0.009617169 | 0.048212291 | K07025 |
| *R72* | 5.059429727 | 0 | 0 | -Inf | 0.009625932 | 0.048238311 | K00430 |
| *SUD1* | 6.38261388 | 0.286860049 | 0.044943977 | -4.475728413 | 0.009662329 | 0.048370364 | - |
| *DLO2* | 6.357822147 | 0.286860049 | 0.045119231 | -4.470113698 | 0.009740873 | 0.048700609 | - |
| *CRK10* | 6.350074972 | 0.286860049 | 0.045174277 | -4.468354664 | 0.009765602 | 0.048796014 | - |
| *NAKR2* | 14.72392106 | 3.975348079 | 0.269992488 | -1.889008828 | 0.009767972 | 0.048798455 | - |
| *Os02g0190300* | 6.347492581 | 0.286860049 | 0.045192656 | -4.467767843 | 0.009773864 | 0.048818483 | K05658 |
| *At2g24130* | 7.440984423 | 0.648253058 | 0.08711926 | -3.520864488 | 0.009806273 | 0.048952071 | - |
| *COMT1* | 5.000548877 | 0 | 0 | -Inf | 0.009864527 | 0.049223915 | - |
| *At4g31140* | 42.98596768 | 20.81644639 | 0.484261435 | -1.046141979 | 0.009873382 | 0.049229439 | - |
| *Dnajc7* | 36.41188321 | 17.44523264 | 0.479108223 | -1.061576521 | 0.009873471 | 0.049229439 | K09527 |
| *IAMT1* | 33.03342121 | 14.98549583 | 0.453646497 | -1.140359575 | 0.009875128 | 0.049229439 | K18848 |
| *At1g56130* | 12.17155491 | 2.674323254 | 0.219719114 | -2.186267718 | 0.009988382 | 0.049669884 | - |
| *CISZOG1* | 17.08481662 | 5.355424572 | 0.313461051 | -1.673641903 | 0.010053252 | 0.049954142 | K13495 |
| *At5g48740* | 14.1328306 | 3.495377565 | 0.247323248 | -2.015530238 | 0.010077255 | 0.050063188 | - |
| *CRRSP55* | 14.1328306 | 2.917138759 | 0.206408669 | -2.276424528 | 0.010092728 | 0.050119284 | - |
| *At1g18000* | 7.371774006 | 0.610986577 | 0.082881892 | -3.592799252 | 0.010094219 | 0.050119284 | - |
| *XTH31* | 35.51569305 | 16.33396634 | 0.459908422 | -1.120581478 | 0.010166956 | 0.050451449 | K08235 |
| *CRK8* | 7.349564664 | 0.573720097 | 0.07806178 | -3.679239837 | 0.010260216 | 0.05086556 | - |
| *CRK8* | 18.98116278 | 6.136953048 | 0.323318077 | -1.628973923 | 0.010291554 | 0.05099167 | - |
| *UGT85A24* | 17.02851817 | 5.081003084 | 0.298381987 | -1.744767651 | 0.010376621 | 0.051305307 | - |
| *XTH10* | 27.97502367 | 11.30944636 | 0.404269412 | -1.306611046 | 0.01038027 | 0.051313563 | K08235 |
| *At2g24130* | 21.51803459 | 8.174295039 | 0.379881118 | -1.39638009 | 0.010481849 | 0.051697431 | - |
| *ZNFX1* | 23.17782516 | 8.692698799 | 0.375043764 | -1.414869139 | 0.010489287 | 0.051714439 | - |
| *ATS3A* | 37.74694714 | 17.46787539 | 0.4627626 | -1.111655824 | 0.010493996 | 0.051727817 | K16911 |
| *At1g56130* | 72.48147719 | 35.76797772 | 0.493477494 | -1.018943806 | 0.010515434 | 0.051794975 | - |
| *GSVIVT00023967001* | 6.983001728 | 0 | 0 | -Inf | 0.010562127 | 0.051974727 | K00430 |
| *ZHD7* | 41.3328921 | 20.25399808 | 0.490021314 | -1.029083594 | 0.01063812 | 0.052239614 | - |
| *ABCG36* | 78.49008138 | 33.01797972 | 0.420664358 | -1.249258507 | 0.010652029 | 0.052288114 | - |
| *ATL11* | 31.31991457 | 13.89453874 | 0.443632715 | -1.172562336 | 0.010711876 | 0.052526918 | K10664 |
| *ag4* | 17.56655887 | 5.9121874 | 0.336559223 | -1.571067703 | 0.01098973 | 0.053711916 | K04120 |
| *R51* | 18.05538198 | 4.168458543 | 0.230870693 | -2.11484305 | 0.010997238 | 0.05373848 | K00430 |
| *EXL2* | 23.6538846 | 2.888909697 | 0.122132569 | -3.033480119 | 0.011145313 | 0.054339163 | - |
| *uncharacterized protein_45855* | 19.24597639 | 6.693715876 | 0.347798196 | -1.523677644 | 0.011308606 | 0.055021495 | - |
| *PRCP* | 15.15607984 | 4.227201001 | 0.278911239 | -1.842122026 | 0.011348619 | 0.055164416 | K01285 |
| *CYP76B10* | 5.970082053 | 0 | 0 | -Inf | 0.011407364 | 0.055418802 | - |
| *Trim21* | 22.41334084 | 6.202448591 | 0.276730213 | -1.85344793 | 0.011485228 | 0.055755286 | - |
| *uncharacterized protein_30903* | 9.742999135 | 1.622891999 | 0.166570065 | -2.585798944 | 0.01154091 | 0.055962732 | - |
| *VSR6* | 14.55207742 | 3.940340953 | 0.27077515 | -1.884832749 | 0.011568299 | 0.056085051 | - |
| *PUB23* | 9.192712779 | 0.286860049 | 0.031205157 | -5.002071704 | 0.011617452 | 0.056302297 | - |
| *uncharacterized protein_12016* | 9.771923468 | 1.546099684 | 0.158218562 | -2.660009226 | 0.011644852 | 0.05642454 | - |
| *OsI_35105* | 32.66700656 | 10.46016299 | 0.320205739 | -1.642928928 | 0.011712508 | 0.056667644 | K00873 |
| *AAP2* | 15.12612332 | 3.882765261 | 0.256692688 | -1.961885892 | 0.011820595 | 0.057105343 | - |
| *nep1* | 34.29425822 | 11.68891421 | 0.340841727 | -1.552826128 | 0.012043312 | 0.057965284 | - |
| *SEOC* | 8.054284228 | 0.935113106 | 0.116101329 | -3.106543603 | 0.012181004 | 0.058551922 | K17609 |
| *At3g47570* | 10.40330002 | 1.909752047 | 0.183571756 | -2.445583993 | 0.012214079 | 0.058700028 | - |
| *SDR2a* | 12.55154782 | 2.963442657 | 0.23610177 | -2.082519238 | 0.01222459 | 0.058727149 | - |
| *GSVIVT00023967001* | 11.76573807 | 2.755634278 | 0.234208365 | -2.09413549 | 0.012242437 | 0.058770953 | K00430 |
| *NFD4* | 11.07548067 | 2.35245608 | 0.212402166 | -2.235129619 | 0.012292839 | 0.058947425 | - |
| *ZNFX1* | 6.137427219 | 0.363652364 | 0.059251597 | -4.077002153 | 0.01231086 | 0.059022923 | - |
| *At5g63180* | 13.87473198 | 3.304526455 | 0.23816867 | -2.069944452 | 0.012326304 | 0.059086045 | K01728 |
| *KIN10A* | 11.05430351 | 2.350196726 | 0.212604686 | -2.2337547 | 0.01233765 | 0.059129497 | K10398 |
| *RLK7* | 29.02461485 | 12.4704177 | 0.429649722 | -1.218767133 | 0.012339993 | 0.059129797 | - |
| *BLH2* | 25.85570019 | 10.64197668 | 0.411591123 | -1.280716227 | 0.012451016 | 0.059562722 | - |
| *HSL1* | 6.127097653 | 0.324126529 | 0.0529005 | -4.240574834 | 0.012506011 | 0.059770664 | K00924 |
| *DLO1* | 7.15809407 | 0.687778892 | 0.096084081 | -3.379558757 | 0.012552764 | 0.05994991 | - |
| *At3g47570* | 12.50454675 | 2.809858027 | 0.224706907 | -2.153883622 | 0.012558359 | 0.059965587 | - |
| *mhpC* | 6.073381586 | 0.363652364 | 0.059876423 | -4.061868157 | 0.012562369 | 0.059973689 | - |
| *AAP3* | 6.093008536 | 0.324126529 | 0.053196467 | -4.23252575 | 0.012642016 | 0.060265157 | - |
| *uncharacterized protein_47038* | 11.05172112 | 2.16160497 | 0.195589895 | -2.354096261 | 0.012711833 | 0.060508978 | - |
| *CYP76T24* | 12.39866482 | 2.807598673 | 0.22644363 | -2.142776137 | 0.012743593 | 0.060615647 | - |
| *WRKY28* | 35.59353454 | 16.57228812 | 0.46559827 | -1.102842396 | 0.012769259 | 0.060690474 | - |
| *GLR2.7* | 7.104378003 | 0.650512412 | 0.091565006 | -3.449059856 | 0.012857719 | 0.06100182 | K05387 |
| *CYCD2-2* | 40.76504316 | 20.31949363 | 0.498453872 | -1.004468093 | 0.012981773 | 0.061488916 | K18810 |
| *METK1* | 7.143631903 | 0.573720097 | 0.080312102 | -3.638238782 | 0.012986615 | 0.061488916 | K00789 |
| *NCL2* | 53.2453162 | 24.30385414 | 0.456450555 | -1.131469505 | 0.012988846 | 0.061488916 | - |
| *uncharacterized protein_12541* | 32.39599598 | 7.663836108 | 0.236567387 | -2.079676898 | 0.012994444 | 0.061504189 | - |
| *LRK10L-1.1* | 19.68743255 | 6.396725822 | 0.324914171 | -1.621869428 | 0.013008075 | 0.061557467 | - |
| *ROQ1* | 33.57079561 | 15.75572754 | 0.469328392 | -1.091330355 | 0.013057427 | 0.061768469 | - |
| *NAC068* | 17.24028202 | 5.79586925 | 0.336181812 | -1.57268642 | 0.013085091 | 0.061876753 | - |
| *DBAT* | 7.032585195 | 0.648253058 | 0.092178486 | -3.439426114 | 0.013113762 | 0.061989719 | K19861 |
| *CCOAOMT* | 17.78953619 | 5.615197346 | 0.315646079 | -1.663620265 | 0.013129773 | 0.062042783 | K00588 |
| *TCM_000168* | 4.784286549 | 0 | 0 | -Inf | 0.013132611 | 0.062044887 | K08241 |
| *GCH1* | 38.28432153 | 18.63788415 | 0.486828117 | -1.038515602 | 0.013214498 | 0.062409019 | K01495 |
| *AGO1A* | 31.73606097 | 14.73138355 | 0.464184373 | -1.107230141 | 0.013265447 | 0.062615422 | K11593 |
| *CRK2* | 27.42576949 | 11.31170572 | 0.412448071 | -1.277715607 | 0.013313234 | 0.062761626 | - |
| *XTH32* | 7.012958245 | 0.610986577 | 0.087122517 | -3.520810547 | 0.013315423 | 0.062761626 | K08235 |
| *PSE1* | 4.739867866 | 0 | 0 | -Inf | 0.013373631 | 0.062988466 | - |
| *uncharacterized protein_30412* | 4.725405699 | 0 | 0 | -Inf | 0.013453515 | 0.063307214 | - |
| *N* | 4.725405699 | 0 | 0 | -Inf | 0.013453515 | 0.063307214 | - |
| *TS2* | 4.720240916 | 0 | 0 | -Inf | 0.013482215 | 0.06340321 | - |
| *N* | 17.32137221 | 5.335115361 | 0.308007662 | -1.698961854 | 0.013523439 | 0.063567029 | - |
| *uncharacterized protein_27474* | 14.7487128 | 4.299474607 | 0.291515244 | -1.778356768 | 0.013561259 | 0.06368707 | - |
| *NHL2* | 41.71045091 | 20.6413116 | 0.494871457 | -1.014874262 | 0.013639701 | 0.064032258 | - |
| *FLZ10* | 4.666524849 | 0 | 0 | -Inf | 0.013786136 | 0.064626097 | - |
| *MYB46* | 28.63945715 | 12.63981781 | 0.441342786 | -1.180028478 | 0.013881096 | 0.064988996 | K09422 |
| *N* | 4.644315508 | 0 | 0 | -Inf | 0.013914755 | 0.06511845 | - |
| *TPX2* | 14.75801018 | 3.994564702 | 0.270670955 | -1.885388013 | 0.013991813 | 0.065424659 | - |
| *SDR2a* | 9.495230082 | 1.660158479 | 0.174841311 | -2.515881991 | 0.014164873 | 0.066150367 | - |
| *RPS11B* | 20.39628472 | 4.79640239 | 0.235160592 | -2.08828178 | 0.01417763 | 0.066186098 | K02949 |
| *PME53* | 42.104906 | 19.12008904 | 0.454105967 | -1.138899102 | 0.014484156 | 0.067434942 | K01051 |
| *uncharacterized protein_16959* | 41.41295011 | 19.00940716 | 0.45902084 | -1.123368439 | 0.014624419 | 0.067933413 | - |
| *XI-E* | 16.36047011 | 4.971462997 | 0.303870425 | -1.71847183 | 0.014634206 | 0.067962687 | K10357 |
| *DDB_G0268948* | 23.00443131 | 9.172669312 | 0.398734887 | -1.326498257 | 0.014723723 | 0.068327233 | - |
| *TT2* | 9.444096407 | 1.471566723 | 0.155818689 | -2.682059811 | 0.01479741 | 0.068585708 | K09422 |
| *OBL1* | 12.90003402 | 3.168991682 | 0.245657622 | -2.025279092 | 0.014826713 | 0.068696751 | - |
| *APF2* | 30.04373149 | 12.99334017 | 0.432480905 | -1.20929166 | 0.01483872 | 0.0687401 | - |
| *STAD* | 10.00523035 | 2.102862512 | 0.210176322 | -2.250327951 | 0.014859164 | 0.068803429 | K03921 |
| *CRK2* | 8.959771771 | 0.897846626 | 0.100208649 | -3.31892106 | 0.014932136 | 0.069086441 | - |
| *XI-K* | 24.94504787 | 10.39464247 | 0.416701644 | -1.262913301 | 0.015009389 | 0.069419093 | K10357 |
| *SCRM2* | 22.78233789 | 9.019084682 | 0.39588056 | -1.33686287 | 0.015049708 | 0.069568342 | - |
| *UPS2* | 24.95021265 | 10.25009525 | 0.41082196 | -1.283414795 | 0.015118758 | 0.069825284 | - |
| *BHLH36* | 17.50406344 | 5.69650837 | 0.325439198 | -1.619540066 | 0.015234797 | 0.07024859 | - |
| *uncharacterized protein_32048* | 10.80653446 | 2.233878576 | 0.206715537 | -2.274281266 | 0.015283098 | 0.070408701 | - |
| *APF1* | 54.34227434 | 25.72350643 | 0.473360873 | -1.078987634 | 0.015333707 | 0.070604217 | - |
| *OPT1* | 10.11627706 | 1.795693252 | 0.177505345 | -2.494065626 | 0.01534936 | 0.070651199 | - |
| *CYP720B1* | 7.698050859 | 0.974638941 | 0.126608535 | -2.981553432 | 0.015600123 | 0.07160205 | - |
| *AZF2* | 36.57096317 | 14.81269458 | 0.405039772 | -1.303864518 | 0.015669172 | 0.071855376 | - |
| *OFUT20* | 29.3426265 | 12.97412355 | 0.442159585 | -1.177360932 | 0.015675718 | 0.071872682 | - |
| *PME8* | 7.717677809 | 0.897846626 | 0.116336371 | -3.103625893 | 0.01580532 | 0.072328889 | K01051 |
| *BACOVA_02659* | 16.18257778 | 0.324126529 | 0.020029351 | -5.641740522 | 0.015822348 | 0.072378588 | K05349 |
| *AGL42* | 24.12514899 | 10.41947039 | 0.431892478 | -1.211255904 | 0.015854166 | 0.072498559 | - |
| *CEL1* | 27.72880482 | 12.18467522 | 0.439423022 | -1.186317637 | 0.015905807 | 0.072689582 | K01179 |
| *APS1* | 11.39504253 | 2.371672703 | 0.208131975 | -2.264429476 | 0.015912197 | 0.072699813 | - |
| *NPF5.3* | 26.46398349 | 10.09199192 | 0.381348179 | -1.390819284 | 0.016007705 | 0.073046071 | K14638 |
| *Gvin1* | 12.44411569 | 2.196612096 | 0.176518135 | -2.502111684 | 0.016033613 | 0.073138551 | - |
| *R72* | 26.27856159 | 10.87577975 | 0.413865109 | -1.272767469 | 0.016329788 | 0.074306552 | K00430 |
| *HSFB4* | 27.59916335 | 12.34166099 | 0.447175186 | -1.161087962 | 0.016769097 | 0.0759988 | K09419 |
| *LECRK59* | 16.18965865 | 5.087781147 | 0.314261175 | -1.669964048 | 0.016824035 | 0.076207825 | - |
| *At1g06840* | 13.83702829 | 3.859029929 | 0.278891526 | -1.842223997 | 0.016854537 | 0.076332652 | - |
| *VIII-A* | 6.684099 | 0.727304727 | 0.108811184 | -3.200101244 | 0.016866864 | 0.07637514 | - |
| *R12* | 20.44106928 | 7.531727457 | 0.368460542 | -1.440417965 | 0.017046276 | 0.077026115 | K00430 |
| *Fra a 1.06* | 5.719730608 | 0.324126529 | 0.056668146 | -4.141318188 | 0.01717057 | 0.077398912 | - |
| *CRK2* | 17.85358182 | 5.755250828 | 0.32235833 | -1.633262829 | 0.017201449 | 0.077452731 | - |
| *CSPL5* | 22.42455431 | 9.051832453 | 0.403657184 | -1.308797526 | 0.017243655 | 0.07757999 | - |
| *RFS6* | 6.674801617 | 0.650512412 | 0.097457939 | -3.359076469 | 0.017250587 | 0.077584254 | K06617 |
| *MOF1* | 17.72718904 | 3.707704653 | 0.209153558 | -2.257365558 | 0.017283571 | 0.077651781 | - |
| *CRK42* | 6.736264858 | 0.573720097 | 0.085168875 | -3.553529903 | 0.017320237 | 0.077784855 | - |
| *uncharacterized protein_47785* | 17.34099916 | 5.766522622 | 0.33253693 | -1.588413527 | 0.017505843 | 0.078446507 | - |
| *EPHX2* | 32.39894425 | 15.04763943 | 0.46444845 | -1.106409617 | 0.017583227 | 0.078738846 | - |
| *LBD4* | 19.83964925 | 7.549777313 | 0.380539858 | -1.393880523 | 0.017659993 | 0.078959872 | - |
| *uncharacterized protein_49302* | 6.642262708 | 0.573720097 | 0.086374195 | -3.533255823 | 0.017756379 | 0.079322432 | - |
| *DTX35* | 33.57854278 | 16.45706255 | 0.490106514 | -1.028832773 | 0.017779353 | 0.079411381 | K03327 |
| *uncharacterized protein_38119* | 12.47717262 | 3.250302706 | 0.260499939 | -1.940645061 | 0.017929253 | 0.079916934 | - |
| *uncharacterized protein_49712* | 9.099742812 | 1.664677188 | 0.182936729 | -2.450583332 | 0.017931409 | 0.079916934 | - |
| *uncharacterized protein_32367* | 48.80759976 | 22.16375013 | 0.454104489 | -1.138903798 | 0.017947014 | 0.079926174 | - |
| *ROQ1* | 29.97916783 | 13.36034448 | 0.445654281 | -1.166003131 | 0.017951287 | 0.079927768 | - |
| *At4g27270* | 9.13228172 | 1.625151353 | 0.177956769 | -2.490401281 | 0.017953538 | 0.079927768 | K03809 |
| *uncharacterized protein_22443* | 9.163788445 | 1.587884873 | 0.173278212 | -2.528837837 | 0.017971809 | 0.079995377 | - |
| *TCM_000168* | 14.76059257 | 4.85849679 | 0.329153235 | -1.603168719 | 0.017986641 | 0.080045715 | K08241 |
| *EDS1B* | 13.04708593 | 3.385837479 | 0.259509096 | -1.946142986 | 0.017989293 | 0.080045715 | - |
| *OFUT20* | 19.7063932 | 7.396192683 | 0.375319451 | -1.413809033 | 0.017998378 | 0.080072402 | - |
| *CRK42* | 10.48955499 | 2.50604071 | 0.238908201 | -2.065471718 | 0.018028387 | 0.080192146 | - |
| *OFP17* | 38.71389792 | 19.26356864 | 0.497587938 | -1.006976581 | 0.018277568 | 0.081133504 | - |
| *PAP1* | 31.6146847 | 15.45416957 | 0.488828838 | -1.032598698 | 0.018388477 | 0.081569962 | - |
| *uncharacterized protein_34412* | 10.50143477 | 2.347937371 | 0.223582532 | -2.161120619 | 0.018414996 | 0.081645695 | - |
| *NFD4* | 9.091995637 | 1.511092558 | 0.166200317 | -2.58900496 | 0.018452565 | 0.081756341 | - |
| *SEN1* | 29.44592604 | 13.30502814 | 0.451846144 | -1.146096483 | 0.018478787 | 0.081844547 | K10706 |
| *CCR1* | 23.98739447 | 8.742403839 | 0.364458251 | -1.456174532 | 0.018535209 | 0.082052399 | K09753 |
| *CRK8* | 18.2072328 | 6.307494947 | 0.346427984 | -1.529372622 | 0.018559479 | 0.082117775 | - |
| *PLP5* | 4.418755796 | 0 | 0 | -Inf | 0.01859533 | 0.082212836 | - |
| *uncharacterized protein_15710* | 30.2300373 | 13.52753444 | 0.447486528 | -1.160083845 | 0.018596817 | 0.082212836 | - |
| *uncharacterized protein_45416* | 4.413591013 | 0 | 0 | -Inf | 0.018634631 | 0.082290838 | - |
| *ERF1A* | 5.02534061 | 0 | 0 | -Inf | 0.018676869 | 0.082454258 | K14517 |
| *LTPG15* | 4.40429363 | 0 | 0 | -Inf | 0.018705698 | 0.082553418 | - |
| *CRK8* | 30.69731738 | 14.65459124 | 0.477389964 | -1.066759857 | 0.018818654 | 0.082981301 | - |
| *hsaD* | 4.382084288 | 0 | 0 | -Inf | 0.018877131 | 0.083210856 | - |
| *At4g27220* | 4.374337113 | 0 | 0 | -Inf | 0.018937492 | 0.083401575 | K13459 |
| *R12* | 9.728536968 | 1.874744921 | 0.192705741 | -2.375528546 | 0.018951666 | 0.08342594 | K00430 |
| *GAT1* | 28.47277829 | 12.5844765 | 0.441982738 | -1.177938071 | 0.019021808 | 0.083706285 | - |
| *At1g67720* | 4.362457338 | 0 | 0 | -Inf | 0.01903062 | 0.083730851 | - |
| *CRJ35* | 4.359874946 | 0 | 0 | -Inf | 0.019050955 | 0.083806102 | - |
| *uncharacterized protein_39797* | 5.003131269 | 0 | 0 | -Inf | 0.019070889 | 0.083865332 | - |
| *CYP76C2* | 5.003131269 | 0 | 0 | -Inf | 0.019070889 | 0.083865332 | - |
| *RAX3* | 6.964406961 | 0 | 0 | -Inf | 0.019092214 | 0.083902191 | K09422 |
| *CRK2* | 4.34954538 | 0 | 0 | -Inf | 0.019132627 | 0.084065542 | - |
| *ADH2* | 14.22838296 | 4.185415812 | 0.294159626 | -1.765328846 | 0.01919569 | 0.084284099 | K18857 |
| *SBT1.7* | 4.337665605 | 0 | 0 | -Inf | 0.019227213 | 0.084381041 | - |
| *At1g67720* | 4.307709088 | 0 | 0 | -Inf | 0.01946889 | 0.08513904 | K10737 |
| *UGT85A1* | 7.408445514 | 1.051431256 | 0.14192333 | -2.816816332 | 0.019487696 | 0.085206909 | - |
| *ATL47* | 22.16490548 | 9.130884123 | 0.411952315 | -1.279450746 | 0.019575856 | 0.085577946 | - |
| *BOR1* | 30.93822316 | 9.060869871 | 0.292869756 | -1.771668878 | 0.019667963 | 0.085922652 | - |
| *CML27* | 62.89431319 | 30.35845354 | 0.48268996 | -1.050831275 | 0.019754529 | 0.086271758 | K13448 |
| *SLC15A2* | 23.34258793 | 9.355649771 | 0.400797452 | -1.319054757 | 0.019852029 | 0.086595459 | K14638 |
| *CCR1* | 7.472491148 | 0.897846626 | 0.120153588 | -3.057048359 | 0.019876566 | 0.086687907 | - |
| *uncharacterized protein_29764* | 7.393983348 | 0.974638941 | 0.131815139 | -2.923412024 | 0.019884127 | 0.086706296 | - |
| *PR5K* | 4.716108316 | 0 | 0 | -Inf | 0.019907219 | 0.086777801 | - |
| *RD21B* | 7.374356398 | 0.972379586 | 0.131859587 | -2.922925631 | 0.019980953 | 0.087055311 | - |
| *CAT1* | 13.51333383 | 3.784496968 | 0.2800565 | -1.836210184 | 0.020277383 | 0.08813948 | K03294 |
| *SRD5A2* | 12.8308236 | 3.80480618 | 0.296536395 | -1.753718911 | 0.020338409 | 0.088345497 | K10258 |
| *At1g74460* | 32.28686536 | 15.64618745 | 0.484599148 | -1.045136226 | 0.020384367 | 0.088514738 | - |
| *CRK2* | 7.366609223 | 0.860580146 | 0.116821745 | -3.097619252 | 0.020522156 | 0.089069043 | - |
| *CRK8* | 19.53299934 | 7.391673975 | 0.378419814 | -1.401940466 | 0.020584309 | 0.089293956 | - |
| *uncharacterized protein_38188* | 20.66987769 | 8.04896445 | 0.389405519 | -1.360654763 | 0.020679382 | 0.089601438 | - |
| *CRK43* | 30.51617635 | 14.89174625 | 0.487995156 | -1.035061267 | 0.02078287 | 0.089929614 | - |
| *CS* | 33.58112518 | 16.47401982 | 0.490573789 | -1.02745794 | 0.020827729 | 0.090078624 | K01647 |
| *LECRK59* | 23.55368548 | 10.06373787 | 0.427268076 | -1.226786567 | 0.020874366 | 0.090190062 | - |
| *FLZ5* | 12.05534341 | 3.557471966 | 0.295095033 | -1.760748456 | 0.021080496 | 0.090973608 | - |
| *N* | 21.52164916 | 8.622684547 | 0.400651664 | -1.319579627 | 0.021095543 | 0.090992883 | - |
| *uncharacterized protein_35375* | 27.98432105 | 12.73130804 | 0.454944325 | -1.136238093 | 0.021185739 | 0.091307153 | - |
| *LTPG15* | 21.5087372 | 8.420536665 | 0.391493772 | -1.352938737 | 0.021353061 | 0.091921331 | - |
| *BANGLUC* | 12.71977689 | 3.420844605 | 0.268939041 | -1.894648891 | 0.021469063 | 0.092328723 | - |
| *RPV1* | 16.73633043 | 5.428864945 | 0.324376061 | -1.624260741 | 0.021480672 | 0.092363328 | - |
| *PHT1-4* | 12.04501385 | 3.327095021 | 0.276221768 | -1.856101077 | 0.021672198 | 0.093109654 | K08176 |
| *LRK10L-1.4* | 7.987656203 | 1.375557785 | 0.172210439 | -2.537755493 | 0.021681729 | 0.093135166 | - |
| *ROQ1* | 31.64360903 | 15.43611972 | 0.487811605 | -1.035604014 | 0.021692743 | 0.093167043 | - |
| *RS2* | 16.27074884 | 5.444655447 | 0.334628449 | -1.579367991 | 0.02180282 | 0.093546833 | K09422 |
| *R12* | 8.090955737 | 1.224232509 | 0.151308764 | -2.724432546 | 0.021884582 | 0.093789 | K00430 |
| *uncharacterized protein_20280* | 7.215424711 | 0 | 0 | -Inf | 0.022014461 | 0.094283273 | - |
| *FLZ5* | 8.088373345 | 1.184706675 | 0.146470325 | -2.771319689 | 0.022064733 | 0.094467369 | - |
| *PR1* | 23.73549281 | 9.180589163 | 0.386787384 | -1.370387358 | 0.022146801 | 0.094771785 | - |
| *TCEA1* | 8.051701837 | 1.184706675 | 0.147137425 | -2.764763847 | 0.022223395 | 0.095023229 | - |
| *CYP720B2* | 11.50247467 | 2.884390988 | 0.250762646 | -1.995605634 | 0.022391717 | 0.095488887 | K09587 |
| *At5g48740* | 5.466796772 | 0.324126529 | 0.059290027 | -4.076066733 | 0.022427917 | 0.095600118 | - |
| *CAT1* | 18.89232541 | 6.685845225 | 0.353892127 | -1.498618428 | 0.022451408 | 0.09568049 | K03294 |
| *LRK10L-1.4* | 13.74405832 | 4.492585072 | 0.32687471 | -1.613190335 | 0.022477338 | 0.095759516 | - |
| *CYP78A4* | 11.42499905 | 2.886650342 | 0.252660883 | -1.984725774 | 0.022481428 | 0.095761208 | - |
| *At1g80120* | 6.340777589 | 0.650512412 | 0.102591899 | -3.285011284 | 0.022515594 | 0.095875238 | - |
| *UGT85A24* | 21.89905969 | 8.942292367 | 0.408341385 | -1.292152304 | 0.022533976 | 0.095937754 | - |
| *ATL60* | 9.514857032 | 1.988803716 | 0.209020872 | -2.258281082 | 0.022606788 | 0.096184577 | - |
| *Gvin1* | 5.474543947 | 0.286860049 | 0.052398894 | -4.254319826 | 0.022631708 | 0.096227367 | - |
| *LOX1.1* | 5.430125263 | 0.324126529 | 0.059690433 | -4.066356468 | 0.022681504 | 0.096407545 | K15718 |
| *At2g24130* | 6.360404539 | 0.573720097 | 0.090201825 | -3.470699567 | 0.022849746 | 0.097043115 | - |
| *DNAJB12* | 6.360404539 | 0.573720097 | 0.090201825 | -3.470699567 | 0.022849746 | 0.097043115 | - |
| *PSKR1* | 17.17881877 | 6.048863961 | 0.352111751 | -1.505894719 | 0.022876528 | 0.09712504 | - |
| *uncharacterized protein_07625* | 8.798257693 | 1.508833203 | 0.171492272 | -2.543784528 | 0.022898259 | 0.097201386 | - |
| *UGT74D1* | 10.07185838 | 2.350196726 | 0.233342908 | -2.099476473 | 0.022925489 | 0.097301044 | K11820 |
| *BHLH* | 5.38570658 | 0.324126529 | 0.060182731 | -4.054506621 | 0.022994473 | 0.097545926 | - |
| *At2g24130* | 9.401227932 | 1.988803716 | 0.211547229 | -2.240948308 | 0.023008217 | 0.097588262 | - |
| *PHT1-4* | 8.765718784 | 1.511092558 | 0.172386611 | -2.536280365 | 0.02301871 | 0.097616801 | K08176 |
| *PLY* | 10.76883077 | 2.525257333 | 0.234496891 | -2.0923593 | 0.023044626 | 0.097705363 | K01728 |
| *UGT85A23* | 5.329408122 | 0.363652364 | 0.068235038 | -3.87334346 | 0.02311434 | 0.097926242 | - |
| *VIP5* | 7.379521181 | 0.860580146 | 0.116617342 | -3.100145749 | 0.023118493 | 0.097927836 | K15178 |
| *Os12g0623900* | 10.08115576 | 2.275663765 | 0.225734412 | -2.147301731 | 0.023139604 | 0.098001251 | K00549 |
| *At3g27390* | 5.400168747 | 0.286860049 | 0.053120571 | -4.234585529 | 0.023157675 | 0.098031014 | - |
| *CYP716B2* | 9.475603132 | 1.872485567 | 0.197611228 | -2.339263177 | 0.023159038 | 0.098031014 | - |
| *YSL12* | 10.1452014 | 2.19887145 | 0.216740049 | -2.205962336 | 0.023181783 | 0.098067756 | - |
| *FATB* | 28.97503138 | 11.25864873 | 0.388563815 | -1.363776539 | 0.023238964 | 0.098245535 | K10781 |
| *uncharacterized protein_16807* | 5.360914847 | 0.286860049 | 0.053509533 | -4.224060258 | 0.023442699 | 0.09892941 | - |
| *PLP6* | 10.04706665 | 2.196612096 | 0.218632181 | -2.193422323 | 0.023517963 | 0.099167995 | - |
| *GGPS* | 17.68003969 | 2.593012231 | 0.146663258 | -2.769420597 | 0.023601088 | 0.099371386 | K13789 |
| *PIP2-8* | 21.12579601 | 5.845574291 | 0.276703149 | -1.85358903 | 0.02363045 | 0.099452042 | K09872 |
