# Supplementary material for: Response mechanism of carbon metabolism of Pinus massoniana to gradient high temperature and drought stress
Source: BMC Genomics. 2024 Feb 12;25:166. doi: 10.1186/s12864-024-10054-2 (PMC10860282; doi:10.1186/s12864-024-10054-2)
Supplement: Supplementary file 3 — Additional file 3. [file 12864_2024_10054_MOESM3_ESM.docx]

Table S4 A total of 4639 differentially expressed genes were identified in the T30CK vs T30Z group

| **gene names** | **baseMean_T30CK** | **baseMean_T30Z** | **foldChange(T30Z/T30CK)** | **log2FoldChange** | **pval** | **padj** | **KEGG** |
| --- | --- | --- | --- | --- | --- | --- | --- |
| *At5g13200* | 88.21312537 | 4220.725972 | 47.84691568 | 5.580354023 | 1.1984E-125 | 3.0788E-121 | - |
| *CSA* | 72.82390232 | 3824.989459 | 52.52381894 | 5.714899911 | 3.3887E-109 | 2.1765E-105 | K00432 |
| *PCBER* | 207.254665 | 5797.131515 | 27.97105442 | 4.805862735 | 2.1488E-106 | 1.1041E-102 | - |
| *MYB2* | 2.203131414 | 616.0766531 | 279.6368157 | 8.127410502 | 6.42201E-97 | 2.35697E-93 | K09422 |
| *NFYC2* | 29.04837027 | 2570.717077 | 88.4978074 | 6.467569807 | 1.26608E-94 | 4.06587E-91 | K08066 |
| *GRDP2* | 9.901098589 | 753.6412459 | 76.11693178 | 6.250145504 | 3.76218E-91 | 9.66542E-88 | - |
| *SWEET3B* | 12.10250722 | 18631.09397 | 1539.440847 | 10.58819072 | 2.26597E-83 | 5.29228E-80 | K15382 |
| *chi1* | 28.3849028 | 983.1423838 | 34.63610183 | 5.114204664 | 3.33889E-83 | 7.14829E-80 | K20547 |
| *JA2L* | 44.14370678 | 9393.163176 | 212.7860087 | 7.733259482 | 5.44134E-77 | 1.07533E-73 | - |
| *NAC068* | 36.03965664 | 983.1532275 | 27.27976122 | 4.769759111 | 8.40625E-77 | 1.54261E-73 | - |
| *Galm* | 290.0434159 | 4048.419254 | 13.95797675 | 3.803017928 | 6.4E-73 | 1.02764E-69 | K01785 |
| *ACR4* | 2.215226832 | 1277.633763 | 576.7507618 | 9.171804194 | 1.18684E-68 | 1.60479E-65 | - |
| *CCR1* | 2518.85682 | 27925.45984 | 11.08656102 | 3.470740015 | 3.97977E-68 | 5.11222E-65 | - |
| *RFS2* | 714.1466357 | 21959.74062 | 30.74962413 | 4.94249687 | 4.07224E-65 | 4.54869E-62 | K06617 |
| *RR23* | 0.362869633 | 273.6168468 | 754.0362209 | 9.558490016 | 6.71048E-63 | 7.18329E-60 | K14491 |
| *GAD* | 1485.190934 | 14758.91252 | 9.937383936 | 3.312866105 | 1.76214E-62 | 1.81084E-59 | K01580 |
| *At2g39510* | 0.364592412 | 354.5963711 | 972.58297 | 9.92567752 | 7.35307E-62 | 7.26568E-59 | - |
| *uncharacterized protein_07382* | 41.8869894 | 755.6097664 | 18.03924744 | 4.173067249 | 1.064E-59 | 9.42595E-57 | - |
| *TOGT1* | 14.02399137 | 918.5421576 | 65.49791235 | 6.033377018 | 2.39404E-59 | 2.05017E-56 | - |
| *PCK* | 548.0928234 | 5258.139766 | 9.593520551 | 3.262060341 | 2.15958E-58 | 1.7338E-55 | K01610 |
| *CHI4* | 12.46189533 | 2379.942015 | 190.977532 | 7.577259109 | 2.93265E-58 | 2.28311E-55 | K01183 |
| *Os01g0656200* | 262.7555702 | 2714.42159 | 10.3305958 | 3.368851557 | 7.12186E-58 | 5.22765E-55 | K14497 |
| *CRK19* | 506.0894434 | 4489.05628 | 8.870084801 | 3.148947897 | 2.37928E-54 | 1.49088E-51 | - |
| *uncharacterized protein_32472* | 0 | 506.6523972 | Inf | Inf | 1.6459E-53 | 9.83365E-51 | - |
| *LBD40* | 2.569446605 | 258.8040908 | 100.7236696 | 6.654258939 | 8.87628E-53 | 4.9574E-50 | - |
| *HPPD* | 1790.446658 | 13803.45072 | 7.709501233 | 2.946637528 | 2.81861E-51 | 1.41986E-48 | K00457 |
| *At5g53970* | 241.9824886 | 2097.134268 | 8.666471198 | 3.115444678 | 1.69794E-49 | 8.07812E-47 | K00815 |
| *HAK5* | 22.43212087 | 432.2450085 | 19.26902101 | 4.26821137 | 7.53402E-49 | 3.51921E-46 | K03549 |
| *D6PKL1* | 259.6848993 | 2272.385464 | 8.750549107 | 3.129373551 | 8.01893E-49 | 3.67883E-46 | - |
| *ATL78* | 2.593637441 | 1860.096739 | 717.1768535 | 9.486185117 | 6.72536E-48 | 2.9285E-45 | K19040 |
| *CAD* | 32.07224555 | 500.0943014 | 15.5927436 | 3.962802892 | 8.7322E-48 | 3.67769E-45 | K00083 |
| *EXL2* | 215.282064 | 2255.347807 | 10.47624575 | 3.389049902 | 2.29323E-47 | 9.35166E-45 | - |
| *NCED3* | 168.8560858 | 3564.916021 | 21.11215597 | 4.400002009 | 6.78337E-45 | 2.60107E-42 | K09840 |
| *At1g80440* | 11.10725207 | 292.8950042 | 26.36970893 | 4.720809742 | 1.7008E-44 | 6.42578E-42 | - |
| *EMB1187* | 413.8576916 | 2939.86307 | 7.103560304 | 2.828542285 | 3.27326E-44 | 1.21874E-41 | K00894 |
| *CHI4* | 0.739557463 | 199.5954366 | 269.8849603 | 8.076200773 | 3.60184E-44 | 1.30331E-41 | K01183 |
| *NFD4* | 153.7419284 | 1935.922043 | 12.59202394 | 3.654438284 | 9.81106E-44 | 3.40616E-41 | - |
| *CHI4* | 0.362869633 | 167.295284 | 461.0341256 | 8.848729732 | 9.98554E-44 | 3.42051E-41 | K01183 |
| *ACX2* | 518.2319631 | 3534.853462 | 6.820986959 | 2.769980504 | 2.89499E-43 | 9.53527E-41 | K00232 |
| *BAM1* | 1945.040577 | 12156.0911 | 6.249787916 | 2.643807233 | 1.54442E-42 | 4.9597E-40 | K01177 |
| *uncharacterized protein_05393* | 9.531301876 | 609.9330422 | 63.99262662 | 5.999833779 | 3.72233E-42 | 1.18062E-39 | - |
| *Os01g0656200* | 642.6126699 | 4051.387894 | 6.304556514 | 2.65639489 | 6.92273E-41 | 2.09237E-38 | K14497 |
| *SRK2A* | 383.8846353 | 3770.358067 | 9.821591491 | 3.295956818 | 4.37024E-40 | 1.29053E-37 | K14498 |
| *TSJT1* | 9.590092151 | 595.9466434 | 62.14191001 | 5.95749468 | 6.96243E-40 | 2.03263E-37 | - |
| *RAB28* | 12.83517357 | 479.3468034 | 37.34634369 | 5.222895101 | 1.57736E-39 | 4.50267E-37 | - |
| *STP7* | 66.44112721 | 707.5893747 | 10.64987011 | 3.41276393 | 2.65129E-39 | 7.48509E-37 | - |
| *uncharacterized protein_47185* | 111.0446037 | 880.513613 | 7.929368771 | 2.987206023 | 3.24974E-39 | 9.07491E-37 | - |
| *DTX54* | 56.41048941 | 553.9888704 | 9.820671229 | 3.295821634 | 5.19409E-39 | 1.43485E-36 | K03327 |
| *TMEM205* | 98.75536014 | 804.9068799 | 8.150513337 | 3.026890926 | 6.98907E-39 | 1.85187E-36 | - |
| *ATG13B* | 137.4420244 | 1021.920296 | 7.435282625 | 2.894387582 | 1.04487E-38 | 2.73916E-36 | K08331 |
| *TSJT1* | 2.935761796 | 322.3896615 | 109.8146525 | 6.778926755 | 2.01769E-38 | 5.13231E-36 | - |
| *HCT* | 7.782671066 | 218.6631729 | 28.09616018 | 4.81230107 | 5.84713E-38 | 1.47273E-35 | K13065 |
| *uncharacterized protein_34546* | 0.739557463 | 143.4350776 | 193.9471707 | 7.59951992 | 2.03373E-37 | 4.97604E-35 | - |
| *OLE9* | 204.9270331 | 1342.943943 | 6.55327861 | 2.712216869 | 4.56205E-37 | 1.05589E-34 | - |
| *NCPR* | 6726.630974 | 35508.79907 | 5.278838575 | 2.40022055 | 1.17011E-36 | 2.66029E-34 | K00327 |
| *XYL1* | 35.49363296 | 683.598214 | 19.25974202 | 4.267516473 | 1.73981E-36 | 3.85323E-34 | K15925 |
| *uncharacterized protein_51568* | 163.513502 | 1082.843363 | 6.622348305 | 2.727342892 | 9.65859E-36 | 2.0852E-33 | - |
| *HIPP33* | 372.2190625 | 2589.439202 | 6.956761388 | 2.798415838 | 1.11542E-35 | 2.38803E-33 | - |
| *MRF1* | 860.7230693 | 4509.605026 | 5.239321666 | 2.389380038 | 1.39349E-34 | 2.91058E-32 | - |
| *SYT5* | 176.2362993 | 1109.751796 | 6.296953584 | 2.654654033 | 2.16846E-34 | 4.49274E-32 | - |
| *MSL6* | 285.644224 | 1648.962901 | 5.772785733 | 2.529267678 | 5.54788E-34 | 1.10489E-31 | - |
| *iolG* | 0.374965051 | 121.9299765 | 325.1769096 | 8.345081007 | 7.01097E-34 | 1.38553E-31 | - |
| *COR413PM2* | 127.8851326 | 1690.004449 | 13.21501894 | 3.724106587 | 7.83248E-34 | 1.53606E-31 | - |
| *CIPK5* | 4.781227878 | 167.9782858 | 35.13287593 | 5.134749773 | 1.45341E-33 | 2.74556E-31 | K07198 |
| *ANN1* | 232.3581065 | 1357.063226 | 5.840395442 | 2.546066055 | 1.59546E-33 | 2.97021E-31 | K17095 |
| *MO2* | 81.12348964 | 640.9719609 | 7.901188223 | 2.98206963 | 3.02092E-33 | 5.52475E-31 | - |
| *MYB306* | 7.711785373 | 188.3978775 | 24.42986525 | 4.610574001 | 7.76733E-33 | 1.37621E-30 | K09422 |
| *LFS* | 496.2404744 | 2556.953725 | 5.152650492 | 2.365314737 | 2.49459E-32 | 4.38963E-30 | - |
| *SUS3* | 2783.911408 | 13049.48539 | 4.687464319 | 2.228807709 | 7.53381E-32 | 1.299E-29 | K00695 |
| *uncharacterized protein_25685* | 153.1355644 | 2293.615629 | 14.97768097 | 3.90474236 | 7.87261E-32 | 1.34837E-29 | - |
| *uncharacterized protein_09249* | 1336.42499 | 8698.606692 | 6.508862645 | 2.70240547 | 2.15719E-31 | 3.62224E-29 | - |
| *RD19D* | 3.269272256 | 230.3036879 | 70.44494001 | 6.138424178 | 5.35317E-31 | 8.9304E-29 | K01373 |
| *IRL1* | 2.579819244 | 134.1317721 | 51.9927016 | 5.700237216 | 7.42307E-31 | 1.22248E-28 | - |
| *GOLS4* | 2.572892163 | 208.1124805 | 80.88659271 | 6.337828685 | 8.99765E-31 | 1.47235E-28 | K18819 |
| *MYBS3* | 156.1073389 | 907.0325376 | 5.810313237 | 2.538615942 | 1.31605E-30 | 2.12646E-28 | - |
| *STA* | 4.048561532 | 204.0741782 | 50.40658925 | 5.655540433 | 2.62332E-30 | 4.13471E-28 | - |
| *BLH1* | 1502.533888 | 6842.311988 | 4.553848698 | 2.187086359 | 3.32692E-30 | 5.18011E-28 | - |
| *GA2OX2* | 16.91994943 | 492.5631777 | 29.11138593 | 4.86351162 | 6.71987E-30 | 1.02762E-27 | K04125 |
| *NPF6.4* | 465.6908089 | 3492.094915 | 7.498741329 | 2.906648458 | 8.01579E-30 | 1.21854E-27 | K14638 |
| *RBL1* | 24.20670126 | 660.5813259 | 27.28919231 | 4.77025779 | 1.1722E-29 | 1.77147E-27 | - |
| *OAT* | 53.61456753 | 414.249278 | 7.726431399 | 2.949802232 | 1.21567E-29 | 1.8158E-27 | K00819 |
| *uncharacterized protein_38539* | 175.9408338 | 969.6678144 | 5.51132897 | 2.462400244 | 1.26934E-29 | 1.87417E-27 | K14315 |
| *At4g33300* | 39.56996765 | 344.3166594 | 8.701464265 | 3.121258195 | 1.30485E-29 | 1.91559E-27 | - |
| *ATL77* | 0.374965051 | 103.587261 | 276.2584424 | 8.109874743 | 1.41237E-29 | 2.06165E-27 | K19040 |
| *LEA14-A* | 192.8815285 | 2158.724614 | 11.19197173 | 3.484392318 | 3.46972E-29 | 4.97992E-27 | - |
| *NAC068* | 6.217093506 | 264.7030617 | 42.57665763 | 5.411990794 | 3.74057E-29 | 5.33883E-27 | - |
| *mgl* | 335.8842499 | 1645.342243 | 4.898539434 | 2.292351654 | 4.29147E-29 | 6.09128E-27 | K01739 |
| *MO2* | 19.09720319 | 232.5600029 | 12.17769956 | 3.60616972 | 7.4503E-29 | 1.02906E-26 | - |
| *HMT-2* | 349.3655983 | 2033.786603 | 5.821370544 | 2.541358851 | 1.0897E-28 | 1.48124E-26 | K00547 |
| *BEH3* | 119.494087 | 695.6988479 | 5.822035761 | 2.541523701 | 1.44517E-28 | 1.94387E-26 | - |
| *HIPP23* | 45.45348586 | 517.3753367 | 11.38252275 | 3.508748437 | 5.34136E-28 | 6.93055E-26 | - |
| *At4g33300* | 9.249690575 | 197.9752368 | 21.40344428 | 4.419771071 | 8.98829E-28 | 1.13753E-25 | - |
| *FAMA* | 284.8615611 | 1673.546754 | 5.874947634 | 2.554575992 | 2.13264E-27 | 2.59667E-25 | - |
| *Pdcd2l* | 903.4701956 | 3858.302357 | 4.2705364 | 2.094417291 | 2.99318E-27 | 3.57664E-25 | K14801 |
| *GIF3* | 507.2963238 | 2252.368069 | 4.439945578 | 2.150541993 | 3.45422E-27 | 4.10844E-25 | - |
| *uncharacterized protein_25844* | 0 | 85.87641526 | Inf | Inf | 1.46631E-26 | 1.68928E-24 | - |
| *uncharacterized protein_42611* | 10.32448128 | 166.7472351 | 16.15066468 | 4.013521635 | 1.70754E-26 | 1.95842E-24 | - |
| *XRN4* | 158.0392965 | 801.8022817 | 5.073436161 | 2.342963194 | 3.1949E-26 | 3.60001E-24 | K20553 |
| *MC410* | 206.2436673 | 1423.197686 | 6.900564289 | 2.786714342 | 3.62772E-26 | 4.06985E-24 | - |
| *uncharacterized protein_45269* | 12.86105122 | 366.0698874 | 28.46344992 | 4.83103863 | 7.88697E-26 | 8.69632E-24 | - |
| *RBOHC* | 144.3590084 | 733.9069597 | 5.083901364 | 2.34593604 | 1.17971E-25 | 1.27882E-23 | K13447 |
| *NAAT1* | 31.22393721 | 265.4133075 | 8.500315181 | 3.087516336 | 1.6466E-25 | 1.7553E-23 | K00815 |
| *LRL1* | 8.461715475 | 148.7865358 | 17.58349548 | 4.136149992 | 2.59483E-25 | 2.73212E-23 | - |
| *uncharacterized protein_26773* | 45.59525724 | 625.0019251 | 13.70760827 | 3.776904963 | 4.34765E-25 | 4.52208E-23 | - |
| *uncharacterized protein_36957* | 149.6604684 | 734.93281 | 4.910667578 | 2.295919164 | 1.06841E-24 | 1.07642E-22 | - |
| *CPK17* | 215.6867767 | 1278.808749 | 5.929008576 | 2.567790884 | 1.48847E-24 | 1.47646E-22 | K13412 |
| *CAF1-7* | 327.8372455 | 1406.647301 | 4.290687897 | 2.101208964 | 1.54389E-24 | 1.52554E-22 | K12581 |
| *XRN3* | 165.1861795 | 787.9811935 | 4.770261021 | 2.25406821 | 1.57756E-24 | 1.55284E-22 | K20553 |
| *ADCS* | 1271.587578 | 4886.061288 | 3.842489006 | 1.942041132 | 3.2104E-24 | 3.06611E-22 | K13950 |
| *GH3.6* | 52.23569747 | 355.3603447 | 6.80301713 | 2.766174722 | 3.68465E-24 | 3.49307E-22 | K14487 |
| *CIPK26* | 630.2354567 | 2507.661871 | 3.978928581 | 1.992380004 | 4.15432E-24 | 3.92384E-22 | K07198 |
| *uncharacterized protein_34118* | 55.46864751 | 354.1188729 | 6.384126688 | 2.674489281 | 4.4672E-24 | 4.20392E-22 | - |
| *ARF19* | 180.6305244 | 1010.61004 | 5.594901767 | 2.484112801 | 4.64966E-24 | 4.34058E-22 | - |
| *PM19L* | 590.935789 | 4694.952243 | 7.944944832 | 2.990037202 | 4.66312E-24 | 4.34058E-22 | - |
| *At5g58730* | 159.2989422 | 746.6224068 | 4.686926331 | 2.228642119 | 1.099E-23 | 1.00122E-21 | K19517 |
| *AVT1C* | 0.725739266 | 84.13437651 | 115.9292055 | 6.857100253 | 1.34873E-23 | 1.21154E-21 | K15015 |
| *uncharacterized protein_08496* | 85.99279504 | 463.2211063 | 5.386743228 | 2.429413297 | 2.6818E-23 | 2.35953E-21 | - |
| *uncharacterized protein_02495* | 0 | 73.29310127 | Inf | Inf | 3.31126E-23 | 2.9034E-21 | - |
| *PNC1* | 99.87497906 | 517.5088458 | 5.181566501 | 2.373388322 | 5.08188E-23 | 4.38116E-21 | - |
| *10HGO* | 7.341988659 | 128.7792502 | 17.54010476 | 4.132585459 | 7.71513E-23 | 6.52004E-21 | K00083 |
| *uncharacterized protein_18332* | 6.300074617 | 120.938924 | 19.19642723 | 4.262765922 | 1.32827E-22 | 1.09725E-20 | - |
| *OPR2* | 266.7733664 | 2422.95123 | 9.082433012 | 3.18307882 | 1.37396E-22 | 1.13136E-20 | K05894 |
| *UGT85A1* | 3.694341758 | 104.1165452 | 28.18270534 | 4.816738202 | 1.56146E-22 | 1.27756E-20 | - |
| *LAR* | 470.8968115 | 6232.462158 | 13.23530337 | 3.726319359 | 2.89074E-22 | 2.30982E-20 | K13081 |
| *Os01g0656200* | 32.22604752 | 594.0755738 | 18.43463967 | 4.204347312 | 2.92858E-22 | 2.32936E-20 | K14497 |
| *AMC4* | 345.3293698 | 1363.134971 | 3.947347345 | 1.980883474 | 3.36035E-22 | 2.64818E-20 | - |
| *At5g63930* | 15.86253037 | 167.6996947 | 10.57206453 | 3.402185231 | 4.5937E-22 | 3.58713E-20 | - |
| *At2g42990* | 4.031297776 | 292.17825 | 72.47746661 | 6.179460623 | 4.66387E-22 | 3.63089E-20 | - |
| *CHX20* | 9.147722929 | 179.6059697 | 19.63395384 | 4.295278823 | 5.20784E-22 | 4.04213E-20 | - |
| *CYP750A1* | 0 | 68.82166903 | Inf | Inf | 5.43327E-22 | 4.2044E-20 | - |
| *At1g60710* | 11.84160524 | 211.1723392 | 17.83308386 | 4.156484304 | 6.42703E-22 | 4.94362E-20 | - |
| *ERF016* | 48.07131413 | 299.5723615 | 6.231832162 | 2.639656379 | 7.50071E-22 | 5.73514E-20 | - |
| *BGLU24* | 33.8089248 | 242.8518534 | 7.183069406 | 2.844600456 | 8.12667E-22 | 6.14066E-20 | K01188 |
| *RHY1A* | 44.36315418 | 283.277081 | 6.385413442 | 2.674780034 | 1.23265E-21 | 9.17913E-20 | - |
| *RLT1* | 65.53046806 | 361.3818561 | 5.514715015 | 2.463286333 | 1.43363E-21 | 1.05534E-19 | - |
| *ATHB-13* | 12.42733186 | 232.7367305 | 18.72781166 | 4.227110426 | 1.60384E-21 | 1.16396E-19 | K09338 |
| *uncharacterized protein_15771* | 63.36545357 | 351.6156307 | 5.549011502 | 2.472230793 | 1.91176E-21 | 1.38352E-19 | - |
| *uncharacterized protein_12566* | 170.6413914 | 728.0999587 | 4.266842604 | 2.093168893 | 2.58527E-21 | 1.84458E-19 | - |
| *At1g56220* | 8.159358896 | 156.384766 | 19.16630558 | 4.260500371 | 2.59194E-21 | 1.84458E-19 | - |
| *PARP1* | 174.6396757 | 2024.252035 | 11.59102035 | 3.534935666 | 3.16804E-21 | 2.24834E-19 | K10798 |
| *RR23* | 416.7988162 | 1552.062844 | 3.723769798 | 1.896763889 | 4.05559E-21 | 2.85458E-19 | K14491 |
| *CKL12* | 1315.101956 | 4566.212691 | 3.472135882 | 1.795823409 | 4.57603E-21 | 3.19464E-19 | K02218 |
| *CYP750A1* | 24.66981576 | 198.606414 | 8.050583593 | 3.009093369 | 5.10121E-21 | 3.54203E-19 | - |
| *FAH* | 946.2693795 | 3309.47045 | 3.497387236 | 1.806277542 | 6.95874E-21 | 4.72955E-19 | K01555 |
| *PHOS34* | 6.230947667 | 144.099335 | 23.12639148 | 4.531468268 | 9.96015E-21 | 6.71617E-19 | - |
| *BAM4* | 1072.276321 | 3667.696153 | 3.420476683 | 1.774197395 | 1.99068E-20 | 1.31811E-18 | K01177 |
| *CCL5* | 262.4431865 | 1011.61248 | 3.85459609 | 1.946579693 | 2.00728E-20 | 1.32568E-18 | K10526 |
| *RING1* | 320.5368122 | 1204.022373 | 3.756268632 | 1.909300242 | 2.08498E-20 | 1.37347E-18 | K11982 |
| *DI19-4* | 785.3620207 | 2725.442558 | 3.47030094 | 1.795060777 | 2.33273E-20 | 1.52883E-18 | - |
| *At2g19810* | 1199.625364 | 4157.329588 | 3.465523249 | 1.793073197 | 2.45481E-20 | 1.60474E-18 | - |
| *exgA* | 12.51379449 | 435.8663332 | 34.83086872 | 5.122294551 | 2.472E-20 | 1.61188E-18 | - |
| *ERD4* | 1233.127824 | 4162.374965 | 3.37546107 | 1.75508458 | 3.53729E-20 | 2.26061E-18 | - |
| *MO2* | 23.48272074 | 187.5958859 | 7.988677631 | 2.997956713 | 3.67665E-20 | 2.34384E-18 | - |
| *exgA* | 3.277886152 | 236.5971258 | 72.17978749 | 6.173522991 | 4.65043E-20 | 2.92829E-18 | - |
| *RZPF34* | 298.9629119 | 1291.774626 | 4.320852437 | 2.111315962 | 6.23809E-20 | 3.88046E-18 | K10144 |
| *SPS3* | 89.05791622 | 420.0500041 | 4.716593672 | 2.23774532 | 9.61209E-20 | 5.92192E-18 | K00696 |
| *MSSP2* | 637.4164716 | 2189.737879 | 3.435333062 | 1.780449978 | 1.15907E-19 | 7.07305E-18 | - |
| *AERO1* | 212.8597799 | 912.2809326 | 4.285830479 | 2.099574787 | 1.22886E-19 | 7.4459E-18 | K10950 |
| *PLA2-ALPHA* | 3.28133171 | 88.46170213 | 26.9590855 | 4.752699654 | 1.44029E-19 | 8.68606E-18 | K01047 |
| *CHI4* | 0.364592412 | 64.70710675 | 177.4779304 | 7.471495824 | 1.51319E-19 | 9.10428E-18 | K01183 |
| *CHI4* | 1.816070944 | 80.85981272 | 44.52458918 | 5.476530395 | 1.61166E-19 | 9.67413E-18 | K01183 |
| *ERD4* | 698.6603327 | 2367.614248 | 3.388791573 | 1.760770907 | 1.8401E-19 | 1.10196E-17 | - |
| *Acx* | 42.28790403 | 251.9699813 | 5.958441003 | 2.574934906 | 2.18989E-19 | 1.30838E-17 | K00232 |
| *INT1* | 1677.075836 | 7818.455493 | 4.661957035 | 2.220935709 | 2.36083E-19 | 1.40398E-17 | K08150 |
| *MYB2* | 1.84026178 | 175.2160659 | 95.21257668 | 6.573080247 | 2.68657E-19 | 1.59401E-17 | K09422 |
| *At1g32860* | 90.60450724 | 672.1562866 | 7.418574495 | 2.891141995 | 3.40912E-19 | 2.0088E-17 | - |
| *ARAC11* | 212.2188813 | 807.4042271 | 3.804582429 | 1.92773812 | 3.56836E-19 | 2.09782E-17 | K04392 |
| *PI4KG7* | 270.986412 | 1216.477437 | 4.489071713 | 2.166417144 | 4.08951E-19 | 2.37164E-17 | - |
| *GEK1* | 335.2968507 | 1196.525009 | 3.568554273 | 1.835339714 | 4.89974E-19 | 2.82241E-17 | K09716 |
| *Os10g0513300* | 84.12310216 | 390.0022242 | 4.636089424 | 2.212908394 | 6.46519E-19 | 3.71582E-17 | - |
| *HXK1* | 2343.062911 | 7423.871621 | 3.168447413 | 1.663776071 | 7.56363E-19 | 4.32778E-17 | K00844 |
| *SRK2A* | 4080.463597 | 12812.63762 | 3.13999557 | 1.650762524 | 8.45992E-19 | 4.82986E-17 | K14498 |
| *WRKY11* | 435.3223518 | 1489.010628 | 3.420478231 | 1.774198048 | 1.07617E-18 | 6.07647E-17 | - |
| *At1g18250* | 94.76544502 | 420.4591486 | 4.436840333 | 2.149532636 | 1.14592E-18 | 6.42792E-17 | - |
| *ABCF4* | 247.223666 | 903.2786788 | 3.653690172 | 1.869354301 | 1.18517E-18 | 6.61917E-17 | K06184 |
| *uncharacterized protein_35980* | 316.2218567 | 1116.4712 | 3.530657911 | 1.819937044 | 1.34749E-18 | 7.50941E-17 | - |
| *GT-3B* | 317.5576213 | 1334.773631 | 4.203248611 | 2.071504791 | 1.63889E-18 | 9.03535E-17 | - |
| *At4g16230* | 419.1652298 | 1432.529797 | 3.417577831 | 1.772974194 | 1.67555E-18 | 9.21765E-17 | - |
| *ERF4* | 486.752897 | 1635.229314 | 3.359464985 | 1.748231493 | 1.7321E-18 | 9.50843E-17 | K09286 |
| *IRL1* | 84.62081609 | 382.9751997 | 4.525780032 | 2.178166466 | 2.09242E-18 | 1.14375E-16 | - |
| *CDF3* | 351.0244795 | 1213.040167 | 3.455713884 | 1.788983774 | 2.13985E-18 | 1.16719E-16 | - |
| *AAEL011121* | 276.949286 | 983.4739238 | 3.551097524 | 1.828264982 | 2.6482E-18 | 1.43837E-16 | - |
| *exgA* | 3.291704349 | 136.1509581 | 41.3618429 | 5.370228562 | 3.14361E-18 | 1.70021E-16 | - |
| *MFT1* | 1.45664687 | 457.8046027 | 314.2866073 | 8.295936986 | 3.1438E-18 | 1.70021E-16 | - |
| *uncharacterized protein_43448* | 180.8465334 | 683.4759069 | 3.779314395 | 1.918124539 | 3.34214E-18 | 1.80006E-16 | - |
| *uncharacterized protein_25948* | 356.3519899 | 1221.273664 | 3.427155449 | 1.777011631 | 3.40975E-18 | 1.83264E-16 | K03122 |
| *GOLS4* | 14.35915268 | 136.0627744 | 9.475682683 | 3.244229887 | 3.56545E-18 | 1.90833E-16 | K18819 |
| *KAB1* | 682.1416216 | 2211.387115 | 3.241829914 | 1.6968084 | 3.79467E-18 | 2.0268E-16 | - |
| *WRKY46* | 88.98551644 | 589.2314446 | 6.621655615 | 2.72719198 | 4.2347E-18 | 2.25713E-16 | K18835 |
| *UGD3* | 111.1239017 | 556.134881 | 5.004637818 | 2.323265666 | 4.3926E-18 | 2.33162E-16 | K00012 |
| *CRY2* | 48.61216948 | 259.9610492 | 5.347653725 | 2.41890605 | 4.51007E-18 | 2.38904E-16 | K12119 |
| *At2g02240* | 6.239597527 | 121.7250387 | 19.50847601 | 4.286029175 | 4.64825E-18 | 2.45717E-16 | - |
| *At2g34160* | 363.6472619 | 1976.539543 | 5.435320845 | 2.4423652 | 4.96726E-18 | 2.60969E-16 | - |
| *Os01g0656200* | 7.338579065 | 555.2942891 | 75.66782127 | 6.241608001 | 1.06833E-17 | 5.52241E-16 | K14497 |
| *RBOHC* | 138.4703289 | 772.9928821 | 5.582371963 | 2.480878257 | 1.40012E-17 | 7.19412E-16 | K13447 |
| *FG2* | 22.31974463 | 941.2737246 | 42.17224437 | 5.398221898 | 1.53469E-17 | 7.85412E-16 | - |
| *11S2* | 254.3913232 | 887.0500656 | 3.486950948 | 1.801966069 | 1.60321E-17 | 8.17224E-16 | - |
| *At5g15710* | 13.61794437 | 773.7563687 | 56.8188816 | 5.828298529 | 1.90754E-17 | 9.7043E-16 | - |
| *TUR2* | 9.527856318 | 110.6145793 | 11.60959776 | 3.537246082 | 2.13409E-17 | 1.07504E-15 | - |
| *HSL1* | 94.8208257 | 755.7250692 | 7.970032571 | 2.99458562 | 2.2442E-17 | 1.12829E-15 | - |
| *uncharacterized protein_16959* | 6.249970166 | 94.39332319 | 15.1030038 | 3.916763608 | 2.72835E-17 | 1.3637E-15 | - |
| *uncharacterized protein_09037* | 393.6580324 | 1289.406458 | 3.27544811 | 1.711692293 | 2.95057E-17 | 1.46905E-15 | - |
| *At4g33300* | 463.5692311 | 1546.378355 | 3.335808875 | 1.738036632 | 3.12075E-17 | 1.54778E-15 | - |
| *MSL2* | 1404.102104 | 4252.821408 | 3.028854808 | 1.598772422 | 3.23339E-17 | 1.60056E-15 | - |
| *uncharacterized protein_27854* | 1.45664687 | 65.55939088 | 45.0070585 | 5.492079373 | 3.91376E-17 | 1.92991E-15 | - |
| *AGD7* | 1191.061796 | 3617.986356 | 3.037614311 | 1.602938701 | 3.9957E-17 | 1.96654E-15 | K12492 |
| *uncharacterized protein_50841* | 38.48814906 | 217.1435287 | 5.641828303 | 2.496162761 | 4.04602E-17 | 1.9875E-15 | - |
| *EPHX2* | 33.73287077 | 199.8887824 | 5.925638045 | 2.566970505 | 5.13747E-17 | 2.48563E-15 | - |
| *BACOVA_02659* | 146.6799721 | 4579.790648 | 31.22301281 | 4.964537849 | 5.68144E-17 | 2.7385E-15 | K05349 |
| *AHRI* | 546.6864249 | 1720.91698 | 3.147905091 | 1.654392044 | 5.87135E-17 | 2.81945E-15 | K00053 |
| *TIFY10B* | 4.399335747 | 107.881556 | 24.5222375 | 4.616018717 | 6.31691E-17 | 3.02776E-15 | K13464 |
| *MED26A* | 111.9827554 | 440.9445864 | 3.937611507 | 1.977320779 | 9.29173E-17 | 4.37074E-15 | - |
| *LOG3* | 2.942688877 | 128.4749176 | 43.65902172 | 5.448207899 | 1.31277E-16 | 6.09878E-15 | K06966 |
| *MYB123* | 15.95412538 | 164.6162621 | 10.31810006 | 3.367105438 | 1.55127E-16 | 7.16794E-15 | K09422 |
| *STY13* | 452.2281953 | 1422.569328 | 3.145689153 | 1.653376115 | 1.76558E-16 | 8.08547E-15 | - |
| *At1g56220* | 842.4832483 | 7173.185905 | 8.51433654 | 3.089894116 | 1.92921E-16 | 8.80346E-15 | - |
| *EIL1* | 2746.90484 | 7969.763064 | 2.901361179 | 1.536729902 | 1.94528E-16 | 8.86101E-15 | K14514 |
| *CINV2* | 631.4521844 | 1931.129668 | 3.058235787 | 1.612699641 | 1.95928E-16 | 8.90899E-15 | - |
| *SBE1* | 432.7300415 | 1359.80956 | 3.142396944 | 1.651865432 | 2.26643E-16 | 1.01973E-14 | K00700 |
| *uncharacterized protein_18539* | 26.07459944 | 1041.563171 | 39.94550993 | 5.319961441 | 2.47922E-16 | 1.11353E-14 | - |
| *NHX2* | 417.6970198 | 1313.732151 | 3.145179613 | 1.653142408 | 2.83213E-16 | 1.26981E-14 | - |
| *ATJ49* | 724.4311765 | 2181.491364 | 3.011316237 | 1.590394222 | 3.0955E-16 | 1.38307E-14 | K09518 |
| *uncharacterized protein_26414* | 509.5907625 | 1567.656622 | 3.076305023 | 1.621198557 | 3.56689E-16 | 1.58541E-14 | - |
| *At5g04720* | 35.88596256 | 252.9212212 | 7.047915205 | 2.817196567 | 3.62512E-16 | 1.60851E-14 | - |
| *B&apos;BETA* | 442.2736905 | 1374.247725 | 3.10723372 | 1.635630762 | 3.9261E-16 | 1.73606E-14 | K11584 |
| *uncharacterized protein_13325* | 171.0212442 | 722.4760372 | 4.224481238 | 2.078774191 | 3.96751E-16 | 1.75136E-14 | - |
| *At2g16250* | 111.3761468 | 428.6703369 | 3.848852285 | 1.944428303 | 4.0506E-16 | 1.78497E-14 | - |
| *OBAP1A* | 0 | 90.11585108 | Inf | Inf | 4.71458E-16 | 2.06693E-14 | - |
| *CFAT* | 6.576553545 | 178.0679319 | 27.07617762 | 4.758952181 | 4.76615E-16 | 2.08244E-14 | - |
| *CRK2* | 16.07501473 | 129.5436243 | 8.058693975 | 3.010546049 | 5.06858E-16 | 2.21081E-14 | - |
| *ERD4* | 411.5079011 | 1277.256196 | 3.103843674 | 1.634055898 | 5.81667E-16 | 2.52426E-14 | - |
| *SAP4* | 1903.269215 | 7940.90656 | 4.172245576 | 2.060824076 | 6.12819E-16 | 2.65496E-14 | - |
| *TKL-1* | 76.98504879 | 529.1886371 | 6.873914422 | 2.781131891 | 6.90002E-16 | 2.9743E-14 | K00615 |
| *COR2* | 267.6928122 | 869.8153716 | 3.249304173 | 1.700130803 | 7.67743E-16 | 3.29284E-14 | - |
| *TH* | 6.982636513 | 90.60861086 | 12.97627489 | 3.697804382 | 8.2991E-16 | 3.54762E-14 | K00500 |
| *LHW* | 609.1733762 | 1819.293386 | 2.986495236 | 1.578453421 | 1.01111E-15 | 4.27948E-14 | - |
| *PCO1* | 138.2457563 | 497.106946 | 3.595820656 | 1.846321067 | 1.21239E-15 | 5.09781E-14 | K10712 |
| *SAC9* | 732.6958265 | 2150.948141 | 2.935663153 | 1.553686439 | 1.25579E-15 | 5.27167E-14 | - |
| *AEL1* | 239.6656107 | 784.4479237 | 3.27309338 | 1.710654763 | 1.30902E-15 | 5.48614E-14 | - |
| *HSP70* | 7077.894392 | 19723.0455 | 2.786569622 | 1.478490199 | 1.45644E-15 | 6.06443E-14 | K03283 |
| *PTEN1* | 125.0512017 | 485.297022 | 3.880786554 | 1.956349086 | 1.69811E-15 | 7.01386E-14 | K01110 |
| *exgA* | 1.104149876 | 56.32883127 | 51.01556638 | 5.672865619 | 1.87314E-15 | 7.67508E-14 | - |
| *DLO1* | 388.6763148 | 1188.968098 | 3.059018655 | 1.613068905 | 2.07138E-15 | 8.47384E-14 | - |
| *uncharacterized protein_08975* | 166.0846713 | 570.1999003 | 3.433187998 | 1.77954886 | 2.37529E-15 | 9.68627E-14 | - |
| *At2g45590* | 249.4873682 | 799.7119296 | 3.20542052 | 1.680513637 | 2.5549E-15 | 1.04022E-13 | - |
| *CPK3* | 35.67682297 | 190.1605344 | 5.330085993 | 2.414158809 | 3.12101E-15 | 1.26271E-13 | K13412 |
| *NET1D* | 859.4004893 | 2460.944809 | 2.863559935 | 1.5178098 | 3.17971E-15 | 1.28443E-13 | - |
| *HIPP23* | 0 | 309.5098604 | Inf | Inf | 3.69261E-15 | 1.48461E-13 | - |
| *chi1* | 0 | 44.95552001 | Inf | Inf | 3.78064E-15 | 1.51763E-13 | K20547 |
| *VPS2.2* | 692.037487 | 1993.768477 | 2.88101225 | 1.526575795 | 5.28208E-15 | 2.0974E-13 | K12191 |
| *NCPR* | 42.69746852 | 209.2246114 | 4.900164311 | 2.292830126 | 5.87013E-15 | 2.32015E-13 | K00327 |
| *FGT1* | 98.50989123 | 372.220916 | 3.778513115 | 1.91781863 | 5.95141E-15 | 2.34866E-13 | - |
| *ALP1* | 132.8580205 | 465.6014554 | 3.504503932 | 1.809210243 | 7.92296E-15 | 3.09815E-13 | - |
| *PAD4* | 39.95348176 | 776.6500869 | 19.43885871 | 4.280871613 | 7.99804E-15 | 3.12276E-13 | - |
| *MAMYB* | 176.6042005 | 850.6102635 | 4.816478097 | 2.267978605 | 9.31502E-15 | 3.63144E-13 | - |
| *P5CS* | 108.0188689 | 2907.184128 | 26.91366941 | 4.750267197 | 9.77886E-15 | 3.8065E-13 | K12657 |
| *ERD4* | 22.14706401 | 141.4509721 | 6.386894987 | 2.67511473 | 1.03576E-14 | 4.0075E-13 | - |
| *uncharacterized protein_11911* | 219.3604952 | 769.5674095 | 3.508231547 | 1.81074397 | 1.06451E-14 | 4.11252E-13 | - |
| *CDT1A* | 24.62487965 | 241.2578047 | 9.797319138 | 3.292387035 | 1.07774E-14 | 4.15737E-13 | K10727 |
| *UGT86A1* | 447.0639857 | 1309.933259 | 2.930080036 | 1.550940073 | 1.09056E-14 | 4.20054E-13 | - |
| *SPCC1223.01* | 652.8324 | 1857.745201 | 2.845669427 | 1.508768078 | 1.19285E-14 | 4.57394E-13 | - |
| *uncharacterized protein_08349* | 659.8921295 | 1880.858318 | 2.850251176 | 1.511089061 | 1.20511E-14 | 4.61409E-13 | - |
| *At4g27520* | 210.6340078 | 715.6712587 | 3.39770043 | 1.764558658 | 1.25422E-14 | 4.78785E-13 | - |
| *OAT* | 21.68908189 | 139.2110796 | 6.418486516 | 2.682233149 | 1.39151E-14 | 5.2962E-13 | K00819 |
| *gtf2a1* | 184.5720361 | 601.7755207 | 3.260382956 | 1.70504143 | 1.47462E-14 | 5.57945E-13 | K03122 |
| *PUB17* | 159.6478858 | 533.9974415 | 3.344845056 | 1.741939382 | 1.48294E-14 | 5.60266E-13 | - |
| *CYP735A2* | 194.0877468 | 624.3908 | 3.217054194 | 1.68574024 | 1.70844E-14 | 6.41688E-13 | K10717 |
| *shv* | 1032.162542 | 2848.093953 | 2.759346361 | 1.464326559 | 1.84376E-14 | 6.89492E-13 | - |
| *uncharacterized protein_47260* | 135.0109826 | 465.6300075 | 3.448830594 | 1.786107266 | 1.85345E-14 | 6.92108E-13 | K19476 |
| *PUMP4* | 77.36002093 | 341.8966925 | 4.419552741 | 2.143900376 | 1.89342E-14 | 7.04984E-13 | K15104 |
| *Os01g0939600* | 416.8107673 | 1217.87538 | 2.921890402 | 1.546902065 | 2.06039E-14 | 7.66041E-13 | K00006 |
| *LARP6B* | 1272.043384 | 3466.23633 | 2.724935622 | 1.446222146 | 2.59702E-14 | 9.57246E-13 | K15191 |
| *FBL3* | 70.38609911 | 497.6730963 | 7.070616252 | 2.821835961 | 2.66123E-14 | 9.78106E-13 | K10268 |
| *uncharacterized protein_32500* | 205.6163853 | 651.0381872 | 3.166275811 | 1.662786933 | 2.71005E-14 | 9.94627E-13 | - |
| *HSP12* | 9.866571079 | 120.5857721 | 12.22164936 | 3.61136709 | 2.72493E-14 | 9.98662E-13 | K13993 |
| *At4g19900* | 1.499860204 | 82.4384732 | 54.96410463 | 5.780417841 | 2.75459E-14 | 1.00809E-12 | K01988 |
| *CRPK1* | 21.41271085 | 135.8267477 | 6.343276601 | 2.665228253 | 3.00329E-14 | 1.09443E-12 | - |
| *PHOS34* | 1219.568709 | 3319.14724 | 2.721574615 | 1.44444159 | 3.1981E-14 | 1.16213E-12 | - |
| *IMA* | 0.362869633 | 50.80620787 | 140.0122888 | 7.129409647 | 3.33367E-14 | 1.20797E-12 | - |
| *PILS6* | 91.10222118 | 339.0916361 | 3.722100643 | 1.896117066 | 3.4886E-14 | 1.26233E-12 | - |
| *Dnajc2* | 254.0246843 | 774.795664 | 3.050080216 | 1.608847185 | 3.84422E-14 | 1.38516E-12 | K09522 |
| *SWI3B* | 237.5748195 | 731.5298694 | 3.079155741 | 1.622534839 | 3.97186E-14 | 1.42715E-12 | K11649 |
| *SB09* | 50.25555981 | 351.8939682 | 7.002090306 | 2.807785668 | 4.05367E-14 | 1.45386E-12 | K03671 |
| *PIN2* | 189.9042402 | 766.557279 | 4.03654641 | 2.013121479 | 4.05751E-14 | 1.45386E-12 | K13947 |
| *MYOB5* | 111.2309658 | 474.3951892 | 4.264956129 | 2.092530901 | 4.70255E-14 | 1.68029E-12 | K11844 |
| *uncharacterized protein_04262* | 80.63610528 | 582.6955689 | 7.226236521 | 2.853244476 | 4.7314E-14 | 1.68826E-12 | - |
| *HXK1* | 205.3436326 | 641.6504364 | 3.124764223 | 1.643747336 | 5.02067E-14 | 1.78404E-12 | K00844 |
| *At3g01520* | 8.052186949 | 262.7360032 | 32.62914843 | 5.028089431 | 5.4819E-14 | 1.93988E-12 | - |
| *STK38L* | 329.2508376 | 965.1455513 | 2.931338181 | 1.551559418 | 6.02983E-14 | 2.12209E-12 | K08790 |
| *MSL3* | 124.8351927 | 425.8363281 | 3.411188133 | 1.770274324 | 6.16768E-14 | 2.16467E-12 | - |
| *At1g54290* | 2442.083107 | 6611.687723 | 2.70739669 | 1.436906288 | 6.70186E-14 | 2.34255E-12 | K03113 |
| *CSE* | 82.09802838 | 309.2745153 | 3.767136938 | 1.913468477 | 7.2271E-14 | 2.51929E-12 | - |
| *NRPB1* | 16.92002136 | 120.6195833 | 7.128807982 | 2.833660862 | 7.31246E-14 | 2.54559E-12 | K03006 |
| *COR2* | 149.4602233 | 499.1924962 | 3.33996889 | 1.739834665 | 8.02913E-14 | 2.78752E-12 | - |
| *uncharacterized protein_24093* | 65.24882079 | 285.8291608 | 4.380602705 | 2.131129377 | 8.16546E-14 | 2.83102E-12 | - |
| *PUB45* | 175.3393575 | 558.8082634 | 3.187009872 | 1.672203488 | 8.73733E-14 | 3.02521E-12 | K08332 |
| *ADH1* | 12.49821755 | 102.1156554 | 8.170417499 | 3.0304098 | 9.12812E-14 | 3.15202E-12 | K18857 |
| *At1g56220* | 2.61265994 | 104.1599583 | 39.86739977 | 5.317137608 | 9.34822E-14 | 3.21937E-12 | - |
| *CIPK5* | 139.9806266 | 1330.615512 | 9.505711924 | 3.248794681 | 9.71507E-14 | 3.33676E-12 | K07198 |
| *GSTF9* | 57.25011902 | 395.7200185 | 6.912125691 | 2.789129452 | 9.81293E-14 | 3.36587E-12 | K00799 |
| *Os04g0338000* | 1828.289445 | 4805.170063 | 2.628232677 | 1.394093003 | 1.05306E-13 | 3.58809E-12 | - |
| *NTF2* | 600.8090212 | 1652.692288 | 2.750778083 | 1.459839756 | 1.16364E-13 | 3.95439E-12 | - |
| *GAT1* | 24.32428181 | 140.4714337 | 5.774946812 | 2.529807659 | 1.29104E-13 | 4.38151E-12 | - |
| *CYP750A1* | 1.104149876 | 1237.476493 | 1120.750471 | 10.13024939 | 1.32709E-13 | 4.492E-12 | - |
| *FUM1* | 165.6767577 | 527.8153424 | 3.18581405 | 1.671662062 | 1.37343E-13 | 4.64274E-12 | K01679 |
| *uncharacterized protein_30216* | 173.5561992 | 548.4090456 | 3.159835535 | 1.65984947 | 1.41179E-13 | 4.76614E-12 | K10268 |
| *SDL5A* | 1310.052634 | 3886.552795 | 2.966714997 | 1.568866338 | 1.45496E-13 | 4.89901E-12 | - |
| *clz9* | 241.3381804 | 723.6246025 | 2.998384265 | 1.584185287 | 1.48109E-13 | 4.98045E-12 | - |
| *SMG7* | 1403.823075 | 3695.995775 | 2.632807396 | 1.396601985 | 1.48725E-13 | 4.99463E-12 | K14409 |
| *Cyp1* | 6940.809935 | 18030.03317 | 2.597684324 | 1.377226122 | 1.76137E-13 | 5.8768E-12 | K01802 |
| *uncharacterized protein_32300* | 55.43591471 | 230.9506632 | 4.166083745 | 2.05869184 | 1.8165E-13 | 6.04504E-12 | - |
| *STA* | 9.151168487 | 305.4279191 | 33.37583824 | 5.060732166 | 1.91662E-13 | 6.36998E-12 | - |
| *IRE1A* | 283.2979221 | 827.3411386 | 2.920392541 | 1.5461623 | 1.97306E-13 | 6.54064E-12 | K08852 |
| *SDH* | 3737.388809 | 9597.982108 | 2.56809837 | 1.360700465 | 2.06374E-13 | 6.81485E-12 | K00008 |
| *EPHX2* | 149.9888323 | 481.1030787 | 3.207592667 | 1.681490945 | 2.09491E-13 | 6.90892E-12 | - |
| *RAMDAZC7* | 233.1995308 | 698.0339597 | 2.993290584 | 1.58173234 | 2.16346E-13 | 7.11671E-12 | - |
| *PAD4* | 92.08017661 | 330.4894524 | 3.589148768 | 1.843641723 | 2.16904E-13 | 7.12594E-12 | - |
| *XERICO* | 298.968296 | 866.1073968 | 2.896987434 | 1.534553427 | 2.251E-13 | 7.37632E-12 | K16285 |
| *HEL* | 0.362869633 | 42.82727854 | 118.0238704 | 6.882934865 | 2.29519E-13 | 7.51157E-12 | - |
| *CA2* | 2.192758775 | 55.4202826 | 25.2742268 | 4.659595052 | 2.57421E-13 | 8.3714E-12 | K15746 |
| *CBSPPR1* | 149.3064143 | 477.0096415 | 3.194836898 | 1.675742281 | 2.78009E-13 | 8.99537E-12 | - |
| *APY7* | 229.8166128 | 683.7872366 | 2.975360346 | 1.573064404 | 2.9168E-13 | 9.414E-12 | - |
| *UGT85A24* | 96.91639666 | 427.2421941 | 4.408358222 | 2.140241462 | 2.97875E-13 | 9.57785E-12 | - |
| *BLOS2* | 226.0184079 | 673.6133415 | 2.980347254 | 1.575480436 | 3.09954E-13 | 9.92895E-12 | K16750 |
| *uncharacterized protein_24004* | 0 | 70.99872975 | Inf | Inf | 3.19169E-13 | 1.01987E-11 | - |
| *At1g54290* | 6529.978984 | 16550.12691 | 2.534483947 | 1.341692026 | 3.46962E-13 | 1.10319E-11 | K03113 |
| *SFR2* | 118.3018166 | 478.4153016 | 4.044023291 | 2.015791306 | 3.54628E-13 | 1.12617E-11 | - |
| *At4g33300* | 3.687414678 | 77.26680657 | 20.9541951 | 4.3891672 | 3.58069E-13 | 1.1357E-11 | - |
| *XRN3* | 518.3358986 | 1408.138003 | 2.716651512 | 1.441829511 | 3.8338E-13 | 1.21149E-11 | K12619 |
| *TRAF1A* | 775.2601087 | 2054.269306 | 2.649780741 | 1.405872987 | 3.95003E-13 | 1.24211E-11 | - |
| *uncharacterized protein_07478* | 1219.951706 | 3167.398923 | 2.596331402 | 1.376474544 | 4.14903E-13 | 1.30309E-11 | - |
| *At1g75220* | 171.3046431 | 1595.721711 | 9.315110682 | 3.219572911 | 4.15747E-13 | 1.30414E-11 | K08145 |
| *AKR2A* | 9709.616576 | 24413.7018 | 2.514383715 | 1.330204833 | 4.5425E-13 | 1.41973E-11 | - |
| *uncharacterized protein_44540* | 35.4573467 | 169.94271 | 4.79287724 | 2.260891989 | 5.40178E-13 | 1.68011E-11 | - |
| *PBL27* | 737.0828647 | 1953.508932 | 2.650324713 | 1.406169127 | 5.49966E-13 | 1.70849E-11 | - |
| *CYP750A1* | 27.4967188 | 185.0624825 | 6.730347859 | 2.750681073 | 5.75522E-13 | 1.78572E-11 | - |
| *HSP17.8* | 111.4991185 | 405.9615853 | 3.640939863 | 1.864310912 | 5.97504E-13 | 1.85168E-11 | K13993 |
| *uncharacterized protein_19224* | 61.39896138 | 241.0137172 | 3.92537124 | 1.972829103 | 6.03667E-13 | 1.86853E-11 | - |
| *PIRL1* | 388.1475189 | 1071.336981 | 2.760128377 | 1.46473537 | 6.96474E-13 | 2.14289E-11 | - |
| *ASK5* | 1323.469034 | 3393.278445 | 2.563927344 | 1.35835538 | 7.80473E-13 | 2.38704E-11 | K03083 |
| *UBP5* | 689.6093083 | 2342.147673 | 3.396339992 | 1.763980888 | 9.48304E-13 | 2.8866E-11 | K11835 |
| *Dctpp1* | 23.06281958 | 169.127136 | 7.333324332 | 2.874467347 | 9.95136E-13 | 3.02199E-11 | K16904 |
| *BBX32* | 240.099366 | 694.1134071 | 2.890942274 | 1.531539801 | 1.01244E-12 | 3.07092E-11 | - |
| *Os08g0500300* | 66.41862319 | 278.3564197 | 4.190939323 | 2.067273634 | 1.04599E-12 | 3.16149E-11 | - |
| *smek1* | 309.2829299 | 865.2773805 | 2.797688773 | 1.48423548 | 1.05104E-12 | 3.16929E-11 | K17491 |
| *PYL8* | 1298.770687 | 3306.270735 | 2.545692453 | 1.348058136 | 1.07683E-12 | 3.24323E-11 | K14496 |
| *LOX3.1* | 333.1268983 | 923.4902778 | 2.772187663 | 1.471024924 | 1.09098E-12 | 3.28202E-11 | K00454 |
| *CPK10* | 14.66850827 | 103.1586206 | 7.032659267 | 2.81407032 | 1.12727E-12 | 3.37146E-11 | - |
| *ADH2* | 729.260549 | 7668.557761 | 10.51552531 | 3.394449018 | 1.19217E-12 | 3.55727E-11 | K18857 |
| *PLIP2* | 109.7673199 | 379.6735663 | 3.458894383 | 1.790310962 | 1.31215E-12 | 3.90619E-11 | - |
| *tfa2* | 623.1550751 | 1635.605769 | 2.62471708 | 1.392161922 | 1.32019E-12 | 3.92559E-11 | K03137 |
| *Sb03g046810* | 663.1402898 | 2250.885023 | 3.39428181 | 1.763106349 | 1.4785E-12 | 4.37607E-11 | K00430 |
| *ATL8* | 8.411646988 | 176.4800975 | 20.98044506 | 4.390973377 | 1.67844E-12 | 4.94505E-11 | K19040 |
| *UGT85A5* | 1.475669368 | 47.45192139 | 32.15620139 | 5.007025086 | 2.1526E-12 | 6.25593E-11 | - |
| *CNGC5* | 204.6643435 | 595.7336833 | 2.910783936 | 1.541407754 | 2.2147E-12 | 6.42914E-11 | K05391 |
| *THE1* | 376.188362 | 1931.166732 | 5.133510038 | 2.359945606 | 2.32057E-12 | 6.72887E-11 | - |
| *CHIT5B* | 35.20329987 | 163.1221738 | 4.633718272 | 2.212170332 | 2.3269E-12 | 6.7396E-11 | K01183 |
| *NAAT1* | 14.99850124 | 102.3315416 | 6.822784484 | 2.770360646 | 2.44119E-12 | 7.04682E-11 | K00815 |
| *RITF1* | 30.07653804 | 147.9216152 | 4.91817293 | 2.298122463 | 2.7545E-12 | 7.92451E-11 | - |
| *HSP17.4B* | 166.8447365 | 654.1921594 | 3.920963724 | 1.971208294 | 2.80603E-12 | 8.06372E-11 | K13993 |
| *CYN* | 488.9299056 | 1290.23941 | 2.638904668 | 1.399939234 | 2.90119E-12 | 8.32787E-11 | K01725 |
| *RHC1A* | 725.685697 | 1852.814676 | 2.553191669 | 1.352301845 | 3.98691E-12 | 1.1318E-10 | K11982 |
| *uncharacterized protein_06062* | 366.9753751 | 981.483679 | 2.674521904 | 1.419281019 | 4.11539E-12 | 1.16698E-10 | - |
| *uncharacterized protein_32482* | 73.75368477 | 373.183389 | 5.059860943 | 2.339097737 | 4.23518E-12 | 1.19567E-10 | - |
| *UGT85A19* | 106.5881357 | 346.1314826 | 3.247373457 | 1.699273308 | 4.46544E-12 | 1.25771E-10 | - |
| *ASNSD1* | 54.15714566 | 211.1693046 | 3.899195611 | 1.963176532 | 4.46963E-12 | 1.25771E-10 | - |
| *luxQ* | 124.6832285 | 390.6284931 | 3.132967422 | 1.647529768 | 4.64528E-12 | 1.30571E-10 | - |
| *ATG13A* | 246.2179592 | 685.8862445 | 2.78568731 | 1.478033326 | 4.96574E-12 | 1.39426E-10 | K08331 |
| *TUR2* | 28.56802798 | 358.3815721 | 12.54484812 | 3.649023099 | 5.29728E-12 | 1.48572E-10 | - |
| *FATB1* | 1240.846234 | 3076.625015 | 2.479457108 | 1.310024268 | 5.37459E-12 | 1.50577E-10 | K10781 |
| *URA6* | 28.96553301 | 150.1539944 | 5.183885081 | 2.374033736 | 6.44853E-12 | 1.79296E-10 | K13800 |
| *PNS1* | 9.928770948 | 82.27246995 | 8.286269306 | 3.050722709 | 6.53812E-12 | 1.81394E-10 | - |
| *TCTP* | 26598.42593 | 63503.44711 | 2.387488917 | 1.255494036 | 6.68184E-12 | 1.85181E-10 | - |
| *MLS* | 25.33159642 | 454.4765262 | 17.94109296 | 4.165195876 | 7.48597E-12 | 2.07021E-10 | K01638 |
| *ZAT9* | 147.541969 | 602.1661247 | 4.081320921 | 2.029036157 | 7.70587E-12 | 2.12873E-10 | - |
| *NAC025* | 101.06211 | 467.5405588 | 4.626269514 | 2.209849316 | 7.79591E-12 | 2.15129E-10 | - |
| *At4g33300* | 25.0084586 | 129.0171213 | 5.158939356 | 2.367074487 | 8.20487E-12 | 2.25687E-10 | - |
| *DDB_G0268948* | 24.13936193 | 410.4609655 | 17.00380344 | 4.087785582 | 8.4306E-12 | 2.31648E-10 | - |
| *NAC045* | 1263.011605 | 3103.424575 | 2.457162359 | 1.296993188 | 8.50759E-12 | 2.33264E-10 | - |
| *HIPP20* | 0 | 97.09556988 | Inf | Inf | 8.81828E-12 | 2.41525E-10 | - |
| *NAGS1* | 381.878735 | 1001.418289 | 2.622346303 | 1.390858218 | 9.6333E-12 | 2.63007E-10 | K14682 |
| *uncharacterized protein_49086* | 27.92875134 | 137.0098481 | 4.905691859 | 2.294456617 | 9.98391E-12 | 2.72289E-10 | - |
| *PBF* | 1113.188426 | 3442.52497 | 3.092490804 | 1.628769305 | 1.00707E-11 | 2.74365E-10 | - |
| *CYP707A1* | 0.374965051 | 37.25661655 | 99.36023755 | 6.634596718 | 1.01254E-11 | 2.75563E-10 | K09843 |
| *SUD1* | 135.3462448 | 729.3381532 | 5.388684071 | 2.429933007 | 1.01823E-11 | 2.76819E-10 | - |
| *uncharacterized protein_00744* | 11.38362311 | 575.9981112 | 50.59883884 | 5.661032373 | 1.04892E-11 | 2.84861E-10 | - |
| *MMS21* | 112.90551 | 353.8709022 | 3.134221724 | 1.648107244 | 1.14261E-11 | 3.08674E-10 | - |
| *CYP750A1* | 0 | 33.49110789 | Inf | Inf | 1.19847E-11 | 3.23423E-10 | - |
| *CBG06644* | 166.162477 | 604.0340713 | 3.635201413 | 1.862035301 | 1.20778E-11 | 3.25593E-10 | - |
| *B2* | 1390.852514 | 3374.381751 | 2.42612478 | 1.278653753 | 1.25323E-11 | 3.36786E-10 | - |
| *BAT1* | 222.8544769 | 1092.091468 | 4.900469053 | 2.292919845 | 1.30988E-11 | 3.5091E-10 | - |
| *At5g07050* | 88.44807069 | 293.1946728 | 3.314879234 | 1.728956312 | 1.32038E-11 | 3.53352E-10 | - |
| *ADH1* | 21.33662086 | 116.5669453 | 5.463233663 | 2.449755129 | 1.44746E-11 | 3.85354E-10 | K18857 |
| *CBSX5* | 443.9497058 | 1138.456913 | 2.564382627 | 1.35861154 | 1.51168E-11 | 4.01204E-10 | - |
| *SPS3* | 108.7997442 | 387.4697962 | 3.561311647 | 1.83240869 | 1.53393E-11 | 4.06269E-10 | K00696 |
| *sympk* | 223.9223406 | 617.048015 | 2.755634 | 1.462384284 | 1.67737E-11 | 4.41982E-10 | K06100 |
| *SAT5* | 248.9741923 | 676.9340298 | 2.718892362 | 1.443019038 | 1.71222E-11 | 4.50241E-10 | K00640 |
| *PARP3* | 3.309004068 | 120.4935489 | 36.41384127 | 5.186415033 | 1.75033E-11 | 4.59323E-10 | K10798 |
| *pabpn1-b* | 570.0082509 | 1432.929073 | 2.513874266 | 1.329912494 | 1.7898E-11 | 4.69202E-10 | - |
| *uncharacterized protein_47446* | 0.727462045 | 58.30557966 | 80.14930818 | 6.324618163 | 1.82005E-11 | 4.76645E-10 | - |
| *DDI1* | 296.7136899 | 788.1085157 | 2.656124549 | 1.409322798 | 1.93178E-11 | 5.04876E-10 | K11885 |
| *SUVH1* | 878.9615475 | 2151.801448 | 2.448117843 | 1.291673006 | 1.95312E-11 | 5.09936E-10 | K11420 |
| *GLPK* | 554.1093482 | 1387.112262 | 2.503318644 | 1.323841941 | 2.19883E-11 | 5.72922E-10 | K00864 |
| *At4g32940* | 893.9263591 | 4162.529127 | 4.656456412 | 2.219232474 | 2.55974E-11 | 6.63594E-10 | K01369 |
| *TUR2* | 1007.623902 | 2502.041227 | 2.483110237 | 1.312148311 | 2.71121E-11 | 7.00038E-10 | - |
| *CYP707A1* | 0.364592412 | 35.75282903 | 98.06246054 | 6.615629056 | 2.8244E-11 | 7.2707E-10 | K09843 |
| *AVP* | 4545.623229 | 12175.01952 | 2.678404897 | 1.421374071 | 2.87948E-11 | 7.40509E-10 | K01507 |
| *LHY* | 156.4910546 | 450.5824059 | 2.879285381 | 1.525710789 | 2.91391E-11 | 7.48612E-10 | K12133 |
| *GOLS4* | 1.465296729 | 43.19831703 | 29.48093459 | 4.881710356 | 3.15022E-11 | 8.08515E-10 | K18819 |
| *uncharacterized protein_00147* | 388.5986677 | 992.3360588 | 2.553627023 | 1.352547823 | 3.27045E-11 | 8.3603E-10 | - |
| *SEC23* | 492.2399349 | 1233.856686 | 2.506616385 | 1.325741222 | 3.60014E-11 | 9.1485E-10 | - |
| *CYSZ* | 612.2754456 | 1750.092621 | 2.858341998 | 1.515178544 | 3.81532E-11 | 9.67921E-10 | K01647 |
| *SWEET1A* | 127.6744429 | 615.0601419 | 4.817410029 | 2.268257723 | 3.81653E-11 | 9.67921E-10 | K15382 |
| *ORP1A* | 1265.013988 | 3020.599185 | 2.387799039 | 1.255681422 | 3.8332E-11 | 9.7119E-10 | K20456 |
| *PBF* | 35.09974621 | 150.8429389 | 4.2975507 | 2.10351466 | 3.9656E-11 | 1.00276E-09 | - |
| *GRMZM2G124911* | 227.7981926 | 906.1990586 | 3.978078352 | 1.992071692 | 4.10777E-11 | 1.03667E-09 | K15108 |
| *uncharacterized protein_31982* | 2433.270903 | 5693.825797 | 2.339988445 | 1.226501406 | 4.27194E-11 | 1.07704E-09 | - |
| *DDB_G0268948* | 0.725739266 | 52.14203015 | 71.84678105 | 6.166851616 | 4.46376E-11 | 1.121E-09 | - |
| *GLCAT14B* | 573.932161 | 1412.459319 | 2.461021381 | 1.299257192 | 4.69878E-11 | 1.17887E-09 | - |
| *ADH1* | 322.5762943 | 832.5130386 | 2.580825229 | 1.367832447 | 4.705E-11 | 1.17928E-09 | K18857 |
| *TOM1* | 246.2110321 | 893.0996626 | 3.627374675 | 1.85892577 | 4.75205E-11 | 1.18991E-09 | - |
| *WIN2* | 96.04231147 | 303.354458 | 3.158550157 | 1.659262482 | 5.02191E-11 | 1.25382E-09 | K17506 |
| *DMR6* | 31.09426125 | 415.7051627 | 13.36919245 | 3.740840419 | 5.1673E-11 | 1.28761E-09 | - |
| *uncharacterized protein_07223* | 268.9276917 | 797.5956117 | 2.965836678 | 1.568439154 | 5.54329E-11 | 1.3773E-09 | - |
| *GAPCP1* | 1022.440331 | 9142.756608 | 8.942093081 | 3.160612563 | 6.0505E-11 | 1.49753E-09 | K00134 |
| *uncharacterized protein_21477* | 0 | 31.10353777 | Inf | Inf | 6.62757E-11 | 1.6372E-09 | - |
| *NAKR2* | 256.5026079 | 722.121041 | 2.815258087 | 1.493267186 | 7.25868E-11 | 1.78623E-09 | - |
| *At1g17710* | 271.4234473 | 708.2053968 | 2.609227036 | 1.383622482 | 8.0466E-11 | 1.96881E-09 | K13248 |
| *uncharacterized protein_34968* | 450.3392641 | 1115.815501 | 2.477721997 | 1.309014325 | 8.1161E-11 | 1.98052E-09 | - |
| *UGT86A1* | 60.63004474 | 258.8973868 | 4.270117033 | 2.094275611 | 8.11759E-11 | 1.98052E-09 | - |
| *UPL5* | 746.6316107 | 1790.743266 | 2.39842948 | 1.262090021 | 8.35589E-11 | 2.03287E-09 | K10591 |
| *uncharacterized protein_21879* | 220.7809496 | 588.6941584 | 2.666417368 | 1.41490262 | 8.44178E-11 | 2.04795E-09 | K19525 |
| *SEI1* | 84.30299047 | 270.0998067 | 3.203917266 | 1.679836894 | 8.69455E-11 | 2.10728E-09 | K19365 |
| *FLZ8* | 2.624755358 | 47.28360538 | 18.01448095 | 4.171085179 | 9.0449E-11 | 2.18601E-09 | - |
| *UCC1* | 1.468742288 | 41.49377817 | 28.25123135 | 4.820241845 | 9.2477E-11 | 2.23082E-09 | - |
| *PSI1* | 466.7484442 | 1147.454148 | 2.458399513 | 1.297719386 | 9.312E-11 | 2.24423E-09 | - |
| *GABA-TP1* | 1565.381385 | 4832.18121 | 3.086903457 | 1.626160363 | 9.62801E-11 | 2.31821E-09 | K16871 |
| *SGPP* | 422.2137203 | 1100.150345 | 2.605671707 | 1.381655328 | 1.00428E-10 | 2.40906E-09 | - |
| *ERD7* | 175.8854172 | 484.1329508 | 2.752547417 | 1.460767417 | 1.01231E-10 | 2.42606E-09 | K19366 |
| *MAPKKK20* | 0.739557463 | 36.62528313 | 49.52324186 | 5.630033853 | 1.02742E-10 | 2.45768E-09 | - |
| *PPC* | 1533.471023 | 4621.726838 | 3.013899035 | 1.591631088 | 1.07773E-10 | 2.57563E-09 | K01595 |
| *uncharacterized protein_08775* | 11.35605864 | 114.9285759 | 10.1204634 | 3.339203446 | 1.1133E-10 | 2.64831E-09 | - |
| *CML11* | 65.45452192 | 223.1675383 | 3.409505283 | 1.76956242 | 1.15749E-10 | 2.7458E-09 | K13448 |
| *ASR3* | 590.9060201 | 1425.837977 | 2.412969116 | 1.27080945 | 1.17594E-10 | 2.787E-09 | - |
| *uncharacterized protein_39161* | 1088.680882 | 2544.139782 | 2.336901312 | 1.22459681 | 1.3571E-10 | 3.18986E-09 | - |
| *YPQ1* | 1.112799735 | 117.0907168 | 105.2217332 | 6.717288909 | 1.38519E-10 | 3.25292E-09 | - |
| *APRR2* | 307.9297146 | 786.9691905 | 2.555677978 | 1.353706065 | 1.41229E-10 | 3.31353E-09 | - |
| *RBL1* | 142.1767732 | 403.0939509 | 2.835160356 | 1.503430336 | 1.41453E-10 | 3.31576E-09 | - |
| *P85* | 47.88819605 | 214.5407648 | 4.480034383 | 2.163509805 | 1.43373E-10 | 3.35463E-09 | K19366 |
| *WAPL2* | 592.845315 | 1418.226857 | 2.392237605 | 1.25836069 | 1.54452E-10 | 3.60729E-09 | - |
| *LOX1.1* | 17033.0947 | 45108.85381 | 2.648306407 | 1.40507005 | 1.55745E-10 | 3.63418E-09 | K15718 |
| *PNC1* | 4.814068574 | 410.7489158 | 85.32261422 | 6.414856264 | 1.57284E-10 | 3.66677E-09 | K00430 |
| *AGT2* | 931.8726275 | 4564.457897 | 4.898156424 | 2.292238847 | 1.8639E-10 | 4.3179E-09 | K00827 |
| *PHY* | 22.39928018 | 289.5076874 | 12.92486567 | 3.692077381 | 1.87363E-10 | 4.33652E-09 | K12121 |
| *ATL77* | 2.559073966 | 45.74919744 | 17.87724702 | 4.160052683 | 2.18745E-10 | 5.02662E-09 | K19040 |
| *Os12g0192500* | 24.560957 | 233.9295887 | 9.524449259 | 3.251635673 | 2.8561E-10 | 6.47626E-09 | - |
| *SSL4* | 586.2397614 | 1390.308206 | 2.371569275 | 1.245842011 | 2.90111E-10 | 6.57252E-09 | - |
| *HVA22* | 0 | 261.7365689 | Inf | Inf | 2.98007E-10 | 6.73953E-09 | K17279 |
| *SPL15* | 540.5951013 | 1285.412728 | 2.377773541 | 1.249611319 | 3.02569E-10 | 6.83066E-09 | - |
| *ERD4* | 76.94355823 | 245.0914042 | 3.185340136 | 1.671447434 | 3.0544E-10 | 6.87736E-09 | - |
| *uncharacterized protein_26866* | 60.30859374 | 205.3872938 | 3.405605753 | 1.767911432 | 3.23237E-10 | 7.25265E-09 | - |
| *ADH2* | 5.835237339 | 58.89307014 | 10.09266063 | 3.335234643 | 3.27572E-10 | 7.3371E-09 | K18857 |
| *ABCI17* | 4.008793755 | 71.39637863 | 17.80994059 | 4.154610799 | 3.63903E-10 | 8.10844E-09 | - |
| *GGCT2;2* | 238.3094244 | 610.4161799 | 2.561443725 | 1.356957196 | 3.69284E-10 | 8.21575E-09 | - |
| *uncharacterized protein_48557* | 314.7375805 | 1752.733703 | 5.568873281 | 2.477385465 | 3.73791E-10 | 8.30714E-09 | - |
| *VAC14* | 194.9257545 | 586.9094872 | 3.010938646 | 1.59021331 | 3.89238E-10 | 8.62805E-09 | K15305 |
| *Cwc22* | 703.8758843 | 2172.223161 | 3.086088343 | 1.625779361 | 4.02077E-10 | 8.88964E-09 | K13100 |
| *11S2* | 46.66304894 | 170.7926134 | 3.660125459 | 1.871893101 | 4.30351E-10 | 9.46588E-09 | - |
| *OsI_12825* | 82.61814554 | 312.8459867 | 3.786649829 | 1.920922014 | 4.36311E-10 | 9.58058E-09 | K16275 |
| *Ttc39a* | 278.2955311 | 696.6464597 | 2.503261396 | 1.323808948 | 4.37495E-10 | 9.59836E-09 | - |
| *EPSPS* | 3312.947259 | 7365.604988 | 2.223278673 | 1.152688792 | 4.47203E-10 | 9.80297E-09 | K00800 |
| *PXG* | 69.40976566 | 610.7756953 | 8.799564289 | 3.13743209 | 4.57559E-10 | 1.00214E-08 | K17991 |
| *At1g56220* | 3.728905233 | 49.36193452 | 13.2376479 | 3.726574898 | 4.6568E-10 | 1.01906E-08 | - |
| *SDH* | 12.47923102 | 79.83001119 | 6.397029679 | 2.677402177 | 5.31008E-10 | 1.15416E-08 | K00008 |
| *At4g33300* | 1408.903929 | 3172.563738 | 2.251795649 | 1.171075908 | 5.60884E-10 | 1.2109E-08 | - |
| *At1g64760* | 498.3021728 | 1175.582496 | 2.359175939 | 1.238283014 | 5.93962E-10 | 1.27801E-08 | - |
| *ARF22* | 132.9080961 | 485.9355378 | 3.656177103 | 1.870335955 | 6.08926E-10 | 1.30911E-08 | - |
| *ROQ1* | 23.32723196 | 137.7406937 | 5.904716599 | 2.561867818 | 6.12694E-10 | 1.31578E-08 | - |
| *Os03g0326500* | 1303.745183 | 2927.691459 | 2.245600979 | 1.167101598 | 6.14262E-10 | 1.31728E-08 | - |
| *PAD4* | 112.5959888 | 345.2710019 | 3.066459167 | 1.61657374 | 6.32642E-10 | 1.35556E-08 | - |
| *CDKG-2* | 233.0493972 | 591.3873396 | 2.537605103 | 1.343467577 | 6.46552E-10 | 1.38306E-08 | K08818 |
| *FLZ10* | 396.9531965 | 952.2649898 | 2.398935185 | 1.262394179 | 6.53805E-10 | 1.39623E-08 | - |
| *ALE2* | 237.9738604 | 600.952549 | 2.525288063 | 1.336447967 | 6.54338E-10 | 1.39623E-08 | - |
| *NHX3* | 128.4378387 | 358.3991354 | 2.790448197 | 1.480496864 | 6.63944E-10 | 1.41438E-08 | - |
| *Os04g0338000* | 93.37279273 | 278.0735244 | 2.978100111 | 1.574392252 | 6.74652E-10 | 1.436E-08 | - |
| *GOLS4* | 0.364592412 | 80.9296414 | 221.9729174 | 7.794239856 | 6.85172E-10 | 1.45477E-08 | K18819 |
| *BZW2* | 1337.412213 | 4570.697103 | 3.41756794 | 1.772970018 | 6.89852E-10 | 1.4635E-08 | - |
| *Rpp25l* | 1037.669206 | 2347.636649 | 2.262413334 | 1.177862528 | 7.04877E-10 | 1.49414E-08 | - |
| *SWEET16* | 0.737834684 | 33.70836995 | 45.68553183 | 5.513665445 | 7.24218E-10 | 1.53387E-08 | K15382 |
| *TRIP4* | 245.4955358 | 617.7346378 | 2.51627646 | 1.331290438 | 7.34125E-10 | 1.55357E-08 | - |
| *HAT14* | 22.05374623 | 146.5576504 | 6.645476415 | 2.73237263 | 7.73814E-10 | 1.6296E-08 | K09338 |
| *PSS1* | 365.5133801 | 877.4588005 | 2.400620192 | 1.263407169 | 7.95817E-10 | 1.67311E-08 | K08730 |
| *CYP707A1* | 0 | 57.68478494 | Inf | Inf | 7.99286E-10 | 1.67902E-08 | K09843 |
| *BRPF3* | 221.7399114 | 561.2664429 | 2.53119269 | 1.339817339 | 8.28078E-10 | 1.73525E-08 | K11723 |
| *TKTC* | 19.4860224 | 98.60993466 | 5.060547126 | 2.339293372 | 8.4966E-10 | 1.77613E-08 | K00615 |
| *uncharacterized protein_00302* | 566.1485583 | 1313.197959 | 2.319528928 | 1.213831839 | 8.60263E-10 | 1.79537E-08 | - |
| *CBSX1* | 1565.18938 | 3477.537718 | 2.22179997 | 1.151728936 | 8.66075E-10 | 1.80603E-08 | - |
| *VSR3* | 312.1732881 | 759.102228 | 2.431669387 | 1.281947091 | 9.00692E-10 | 1.87214E-08 | - |
| *AFP3* | 769.3983889 | 1899.867848 | 2.46929013 | 1.304096356 | 9.25536E-10 | 1.92067E-08 | - |
| *ATL60* | 61.95360605 | 203.7988458 | 3.289539687 | 1.717885718 | 9.8636E-10 | 2.04524E-08 | - |
| *CYP750A1* | 0 | 39.78094539 | Inf | Inf | 9.94377E-10 | 2.05689E-08 | - |
| *CLINT1* | 66.64840726 | 645.8628339 | 9.690596677 | 3.276585499 | 1.03557E-09 | 2.13865E-08 | K12471 |
| *NFYA7* | 124.2529188 | 345.1323989 | 2.777660294 | 1.473870169 | 1.04312E-09 | 2.15252E-08 | K08064 |
| *Os01g0794400* | 679.1725156 | 1552.789845 | 2.286296646 | 1.193012605 | 1.05585E-09 | 2.17703E-08 | K17609 |
| *ASK5* | 490.4229182 | 1141.621122 | 2.327829879 | 1.218985628 | 1.11259E-09 | 2.28852E-08 | K03083 |
| *SR34* | 692.9807138 | 1580.437427 | 2.280636958 | 1.18943681 | 1.12782E-09 | 2.31798E-08 | K12890 |
| *uncharacterized protein_40296* | 597.0679127 | 1373.27443 | 2.300030534 | 1.201653014 | 1.12896E-09 | 2.31847E-08 | - |
| *MAVI* | 23.4775524 | 300.3247532 | 12.79199586 | 3.677169472 | 1.15355E-09 | 2.36551E-08 | - |
| *LHY* | 124.4534663 | 857.3954069 | 6.889285069 | 2.784354276 | 1.1537E-09 | 2.36551E-08 | K12133 |
| *HGO* | 1335.970762 | 2963.983433 | 2.218599027 | 1.149648949 | 1.17147E-09 | 2.39621E-08 | K00451 |
| *MAOM* | 49.72666312 | 174.2626492 | 3.504410677 | 1.809171852 | 1.19341E-09 | 2.43719E-08 | K00028 |
| *PXG* | 0 | 84.17021769 | Inf | Inf | 1.22734E-09 | 2.49853E-08 | K17991 |
| *uncharacterized protein_37013* | 96.19100994 | 280.9014052 | 2.92024593 | 1.546089871 | 1.23945E-09 | 2.51921E-08 | - |
| *At1g75220* | 360.4057056 | 1154.012782 | 3.201982555 | 1.678965448 | 1.24502E-09 | 2.52853E-08 | K08145 |
| *GGCT2;2* | 859.0794121 | 1932.90183 | 2.249968749 | 1.169904963 | 1.25859E-09 | 2.55205E-08 | - |
| *RBL1* | 419.8136167 | 988.1695472 | 2.353829195 | 1.235009635 | 1.26091E-09 | 2.55473E-08 | - |
| *CID7* | 558.5780338 | 1329.288097 | 2.379771521 | 1.250823069 | 1.30569E-09 | 2.63923E-08 | - |
| *CYP750A1* | 0 | 26.92673894 | Inf | Inf | 1.37024E-09 | 2.76534E-08 | - |
| *APS1* | 130.203949 | 356.1067259 | 2.734991745 | 1.451536479 | 1.37193E-09 | 2.76658E-08 | - |
| *MYC2* | 129.2847479 | 553.6308597 | 4.282259652 | 2.098372275 | 1.40504E-09 | 2.82669E-08 | K13422 |
| *BPC3* | 509.4767283 | 2061.062737 | 4.045450209 | 2.016300266 | 1.41021E-09 | 2.83487E-08 | - |
| *GPXMC1* | 430.7811438 | 1009.048886 | 2.342370136 | 1.227969065 | 1.43884E-09 | 2.88657E-08 | K00432 |
| *At3g20650* | 82.69592235 | 248.0067559 | 2.999020373 | 1.584491323 | 1.6375E-09 | 3.26877E-08 | K00565 |
| *DDB_G0268948* | 3.649405644 | 654.9753079 | 179.474515 | 7.487635188 | 1.67038E-09 | 3.33182E-08 | - |
| *BIOF* | 35.41767973 | 245.1175756 | 6.920768878 | 2.790932326 | 1.72914E-09 | 3.44368E-08 | K00652 |
| *ULP2B* | 261.5619007 | 639.4284985 | 2.44465458 | 1.289630633 | 1.81632E-09 | 3.60054E-08 | - |
| *XXT2* | 1740.183042 | 3796.328721 | 2.181568622 | 1.125365855 | 1.87449E-09 | 3.70442E-08 | K08238 |
| *KAPP* | 56.41559292 | 187.6055466 | 3.325420099 | 1.733536607 | 1.8808E-09 | 3.71404E-08 | K01090 |
| *uncharacterized protein_01913* | 236.7817698 | 582.9559114 | 2.461996596 | 1.299828767 | 1.94758E-09 | 3.82533E-08 | - |
| *LPA1* | 46.05299471 | 208.1800167 | 4.520444718 | 2.176464711 | 1.95561E-09 | 3.83817E-08 | - |
| *EXPA8* | 26.64488588 | 114.2392696 | 4.28747453 | 2.100128101 | 2.13473E-09 | 4.18013E-08 | - |
| *uncharacterized protein_22138* | 237.9616571 | 585.1790691 | 2.459131762 | 1.298149038 | 2.15398E-09 | 4.21462E-08 | - |
| *HSP18.2* | 0.737834684 | 31.95807584 | 43.31332822 | 5.436739129 | 2.34903E-09 | 4.56542E-08 | K13993 |
| *MYB2* | 7.691040095 | 164.6703315 | 21.41067131 | 4.420258126 | 2.3557E-09 | 4.57447E-08 | K09422 |
| *MYC2* | 281.4660584 | 676.4730735 | 2.403391291 | 1.26507155 | 2.4217E-09 | 4.69414E-08 | K13422 |
| *MYB2* | 0.374965051 | 90.54563223 | 241.4775243 | 7.915745105 | 2.42686E-09 | 4.69846E-08 | K09422 |
| *AP2-3* | 1787.12299 | 3875.727553 | 2.1686966 | 1.116828235 | 2.4563E-09 | 4.7483E-08 | K09284 |
| *uncharacterized protein_26646* | 112.3075512 | 311.6207023 | 2.77470837 | 1.472336148 | 2.52954E-09 | 4.8862E-08 | - |
| *RBX1A* | 132.1116869 | 354.3951513 | 2.682542019 | 1.423600769 | 2.59708E-09 | 5.00537E-08 | K03868 |
| *BC10* | 312.3149657 | 742.2586156 | 2.376634798 | 1.248920231 | 2.65061E-09 | 5.10471E-08 | - |
| *GGCT2;2* | 1036.94242 | 2596.98634 | 2.50446533 | 1.32450264 | 2.75289E-09 | 5.29375E-08 | - |
| *MYB5* | 201.8047147 | 505.142149 | 2.503123624 | 1.323729545 | 2.77199E-09 | 5.3265E-08 | K09422 |
| *CIPK2* | 56.91847519 | 186.285151 | 3.272841558 | 1.710543762 | 2.79958E-09 | 5.37548E-08 | K07198 |
| *ASHH2* | 1099.124401 | 2404.378976 | 2.187540349 | 1.129309628 | 2.87873E-09 | 5.51921E-08 | K11423 |
| *uncharacterized protein_31073* | 4.026093475 | 222.1743789 | 55.18361167 | 5.786167977 | 2.89658E-09 | 5.5493E-08 | - |
| *GALM* | 110.9460457 | 465.4981974 | 4.195716887 | 2.068917333 | 2.91985E-09 | 5.58554E-08 | K01785 |
| *BSK2* | 358.9937284 | 838.1575504 | 2.334741485 | 1.223262816 | 3.08857E-09 | 5.89075E-08 | K14500 |
| *AMY1.6* | 97.80335429 | 280.1724331 | 2.864650555 | 1.518359162 | 3.15145E-09 | 6.00177E-08 | K01176 |
| *ANR* | 286.377135 | 953.4191283 | 3.329243196 | 1.735194261 | 3.23498E-09 | 6.15629E-08 | K08695 |
| *VAMP714* | 109.2076578 | 302.9659922 | 2.774219301 | 1.472081837 | 3.24775E-09 | 6.176E-08 | K08515 |
| *PUB62* | 515.0424857 | 1167.651001 | 2.26709647 | 1.180845782 | 3.28865E-09 | 6.24917E-08 | - |
| *SDIR1* | 150.4727422 | 391.9500311 | 2.604790909 | 1.38116757 | 3.36163E-09 | 6.369E-08 | K16283 |
| *NAD-ME1* | 19.73996135 | 95.45061316 | 4.835400206 | 2.273635301 | 3.40124E-09 | 6.43456E-08 | K00028 |
| *CPSF30* | 72.1104312 | 219.5356361 | 3.044436602 | 1.60617527 | 3.43747E-09 | 6.49354E-08 | K14404 |
| *ERF110* | 3.664946621 | 732.1942027 | 199.7830469 | 7.642290355 | 3.44795E-09 | 6.50855E-08 | - |
| *DDI1* | 166.0310854 | 425.4883767 | 2.562703097 | 1.357666344 | 3.46333E-09 | 6.53278E-08 | K11885 |
| *ASNSD1* | 63.62631959 | 200.4945691 | 3.151126301 | 1.65586758 | 3.47469E-09 | 6.5494E-08 | - |
| *chi1* | 0.725739266 | 31.32261474 | 43.15959768 | 5.431609511 | 3.55979E-09 | 6.6947E-08 | K20547 |
| *DCR* | 0 | 166.6445523 | Inf | Inf | 3.5622E-09 | 6.6947E-08 | - |
| *SAP8* | 848.1628019 | 2515.305617 | 2.96559294 | 1.568320586 | 3.72972E-09 | 6.98398E-08 | - |
| *IBR5* | 546.2267701 | 1229.917398 | 2.251660785 | 1.170989501 | 3.74643E-09 | 7.01017E-08 | K04459 |
| *DLO1* | 5.0853432 | 99.65251968 | 19.59602642 | 4.292489236 | 3.76214E-09 | 7.03444E-08 | - |
| *BACOVA_02659* | 32.84127008 | 263.448426 | 8.021870816 | 3.003938733 | 4.01212E-09 | 7.47465E-08 | K05349 |
| *MAPKKK17* | 9.531301876 | 65.58275164 | 6.880775837 | 2.782571244 | 4.25963E-09 | 7.93001E-08 | - |
| *uncharacterized protein_47075* | 87.67746724 | 695.576045 | 7.933350118 | 2.98793022 | 4.27089E-09 | 7.94522E-08 | - |
| *HXK5* | 22.0640829 | 100.3052813 | 4.546088851 | 2.184625881 | 4.36584E-09 | 8.10424E-08 | K00844 |
| *TUBB1* | 58.16773412 | 186.7597414 | 3.210710272 | 1.682892485 | 4.4224E-09 | 8.1974E-08 | K07375 |
| *uncharacterized protein_06900* | 751.1089243 | 2063.498142 | 2.747268839 | 1.457998095 | 4.58822E-09 | 8.48639E-08 | - |
| *PLAT1* | 20.10458972 | 95.47256862 | 4.748794675 | 2.247561379 | 4.64428E-09 | 8.57772E-08 | - |
| *NAC025* | 40.90042007 | 155.099295 | 3.792119855 | 1.923004563 | 4.75107E-09 | 8.76236E-08 | - |
| *CBP60B* | 78.3897965 | 231.4062739 | 2.951994828 | 1.561690194 | 4.87869E-09 | 8.97839E-08 | - |
| *At3g01520* | 40.88487909 | 146.0737798 | 3.572806941 | 1.837057959 | 4.94938E-09 | 9.09546E-08 | - |
| *FBL8* | 1131.344615 | 2440.450263 | 2.157123684 | 1.109108899 | 5.16178E-09 | 9.45871E-08 | - |
| *uncharacterized protein_26986* | 487.6613152 | 1099.480591 | 2.25459875 | 1.1728707 | 5.33708E-09 | 9.76602E-08 | - |
| *ADH2* | 1726.308371 | 3678.355471 | 2.13076385 | 1.09137071 | 5.67289E-09 | 1.0351E-07 | K18857 |
| *HSL1* | 8.777926215 | 62.51712658 | 7.122083855 | 2.832299422 | 5.77265E-09 | 1.05256E-07 | - |
| *GAPC* | 60.3569825 | 378.4859306 | 6.270789474 | 2.648647086 | 5.85131E-09 | 1.06614E-07 | K00134 |
| *Sec24a* | 435.1166218 | 986.4433429 | 2.267078051 | 1.180834061 | 5.91536E-09 | 1.07705E-07 | - |
| *At1g76660* | 191.6114164 | 475.2426217 | 2.480241681 | 1.310480708 | 5.97196E-09 | 1.08658E-07 | - |
| *AATP2* | 1331.943594 | 2850.228216 | 2.139901592 | 1.097544453 | 6.05384E-09 | 1.09992E-07 | K03301 |
| *DDB_G0289029* | 402.7347907 | 914.5720931 | 2.270904114 | 1.183266792 | 6.43464E-09 | 1.16335E-07 | K19476 |
| *APS1* | 19.44621866 | 92.44221891 | 4.753737501 | 2.249062241 | 6.45712E-09 | 1.16659E-07 | - |
| *TEB* | 151.6925341 | 388.4766577 | 2.560947775 | 1.356677833 | 6.47454E-09 | 1.16892E-07 | K14574 |
| *ORRM2* | 569.2326301 | 1262.171522 | 2.2173211 | 1.148817709 | 6.58014E-09 | 1.18715E-07 | - |
| *At3g53970* | 1630.582687 | 3466.440961 | 2.12589094 | 1.088067587 | 6.654E-09 | 1.19964E-07 | K06700 |
| *CIPK5* | 687.6101046 | 4394.441429 | 6.390891291 | 2.676017147 | 6.7174E-09 | 1.21022E-07 | K07198 |
| *uncharacterized protein_42366* | 95.05925254 | 265.2180597 | 2.790028878 | 1.480280055 | 6.94235E-09 | 1.24987E-07 | K06100 |
| *RHM1* | 385.8253076 | 875.3362003 | 2.268737128 | 1.181889458 | 7.12192E-09 | 1.2804E-07 | K12450 |
| *DREB2A* | 46.22932957 | 157.2317322 | 3.401125078 | 1.766012063 | 7.26013E-09 | 1.30434E-07 | - |
| *V* | 1345.809423 | 2871.029369 | 2.133310496 | 1.093093961 | 7.38636E-09 | 1.32239E-07 | K01369 |
| *At3g05675* | 677.3928317 | 1495.997518 | 2.208463757 | 1.143043157 | 7.56348E-09 | 1.35316E-07 | - |
| *NAC068* | 181.0815506 | 2371.627795 | 13.09701506 | 3.711166139 | 7.67091E-09 | 1.37142E-07 | - |
| *uncharacterized protein_02690* | 195.0034016 | 478.2977669 | 2.452766274 | 1.294409765 | 7.7753E-09 | 1.38912E-07 | - |
| *IP5P4* | 76.70161391 | 224.8607284 | 2.931629687 | 1.551702879 | 8.35099E-09 | 1.48474E-07 | - |
| *MFP* | 2721.069286 | 6290.770292 | 2.311874352 | 1.209062991 | 8.56888E-09 | 1.52033E-07 | K10527 |
| *CA2* | 5.940686506 | 880.8133254 | 148.2679358 | 7.212062826 | 8.58573E-09 | 1.52226E-07 | K15746 |
| *ATL76* | 80.57925357 | 453.2326332 | 5.624681454 | 2.491771394 | 9.0247E-09 | 1.59569E-07 | K19040 |
| *GRMZM2G118515* | 156.5602606 | 573.0790582 | 3.66043756 | 1.872016115 | 9.70622E-09 | 1.71148E-07 | K15108 |
| *ZHD1* | 278.6429606 | 947.9065366 | 3.401867876 | 1.76632711 | 9.75876E-09 | 1.71956E-07 | - |
| *HMGS* | 1145.341232 | 2817.484848 | 2.459952344 | 1.298630367 | 1.01929E-08 | 1.79114E-07 | K01641 |
| *ATHB-13* | 35.98958815 | 183.6568313 | 5.103054543 | 2.351361062 | 1.03749E-08 | 1.82187E-07 | K09338 |
| *VATA* | 990.8330239 | 2114.68202 | 2.134246608 | 1.093726886 | 1.05711E-08 | 1.85127E-07 | K02145 |
| *STY13* | 10.57156507 | 298.2093246 | 28.20862595 | 4.818064488 | 1.07551E-08 | 1.88094E-07 | - |
| *uncharacterized protein_00087* | 197.1240919 | 765.959834 | 3.885673368 | 1.958164632 | 1.09169E-08 | 1.90276E-07 | - |
| *PAB8* | 536.0335868 | 1179.828327 | 2.201034331 | 1.138181648 | 1.10248E-08 | 1.91896E-07 | K13126 |
| *yoxD* | 130.9176577 | 729.9939788 | 5.575977996 | 2.479224868 | 1.11446E-08 | 1.9385E-07 | - |
| *RZFP34* | 1119.040733 | 2379.171364 | 2.126081111 | 1.088196637 | 1.16282E-08 | 2.01852E-07 | K10144 |
| *SWEET16* | 38.90288189 | 137.904072 | 3.544829208 | 1.825716119 | 1.19896E-08 | 2.07985E-07 | K15382 |
| *SDH2-1* | 851.0999268 | 1823.871639 | 2.142958284 | 1.099603766 | 1.24897E-08 | 2.16221E-07 | K00235 |
| *EXD1* | 841.4390781 | 1804.319859 | 2.144326198 | 1.100524387 | 1.26841E-08 | 2.19291E-07 | K18740 |
| *DTX40* | 591.7152328 | 4132.225709 | 6.983470223 | 2.803944118 | 1.2915E-08 | 2.23133E-07 | K03327 |
| *SCL1* | 558.0822802 | 1214.892006 | 2.17690482 | 1.12227833 | 1.37662E-08 | 2.36884E-07 | - |
| *FIPS5* | 1242.893514 | 2616.464504 | 2.105139719 | 1.073915989 | 1.40701E-08 | 2.41951E-07 | - |
| *CYP750A1* | 162.6992967 | 1141.764559 | 7.017636723 | 2.810985267 | 1.41057E-08 | 2.42401E-07 | - |
| *DRP5A* | 76.32137263 | 220.9250594 | 2.894668319 | 1.533398049 | 1.4248E-08 | 2.4452E-07 | - |
| *MPK* | 138.7675602 | 353.5803762 | 2.54800456 | 1.349367859 | 1.42785E-08 | 2.44879E-07 | K14512 |
| *MYB73* | 29.78627688 | 115.5205743 | 3.878315331 | 1.955430108 | 1.43416E-08 | 2.45633E-07 | K09422 |
| *At1g62600* | 131.8315467 | 338.6790086 | 2.569028561 | 1.361222929 | 1.44698E-08 | 2.47664E-07 | - |
| *uncharacterized protein_34483* | 4.010552498 | 44.45185048 | 11.08372238 | 3.470370575 | 1.46603E-08 | 2.50591E-07 | - |
| *AKR4C9* | 105.0623618 | 281.9210979 | 2.683369125 | 1.424045526 | 1.49617E-08 | 2.55554E-07 | - |
| *PMLN* | 35.70801281 | 328.750362 | 9.206627198 | 3.202672729 | 1.49705E-08 | 2.55554E-07 | - |
| *BZIP17* | 320.7353634 | 729.6291551 | 2.274863449 | 1.185779949 | 1.51198E-08 | 2.5793E-07 | - |
| *BZIP1-A* | 77.64514263 | 222.5205722 | 2.865865973 | 1.518971141 | 1.51577E-08 | 2.58404E-07 | K09060 |
| *POT1* | 459.9037304 | 1014.242049 | 2.205335556 | 1.140998188 | 1.5203E-08 | 2.59006E-07 | K03549 |
| *Tbc1d5* | 15.81411274 | 121.5749123 | 7.687747921 | 2.942561031 | 1.55675E-08 | 2.64864E-07 | K18469 |
| *UBP5* | 13.27402531 | 72.99893629 | 5.499382034 | 2.459269512 | 1.59953E-08 | 2.71782E-07 | K11835 |
| *DCR* | 1.829889142 | 146.6856562 | 80.16095228 | 6.324827742 | 1.60443E-08 | 2.72434E-07 | - |
| *CIPK32* | 13.25159321 | 72.94401824 | 5.504547043 | 2.460623852 | 1.61709E-08 | 2.74403E-07 | K07198 |
| *PAP27* | 15.41840241 | 98.80124039 | 6.408007638 | 2.679875867 | 1.68814E-08 | 2.86271E-07 | - |
| *ALDH7B4* | 97.14308063 | 263.6311439 | 2.713843767 | 1.440337669 | 1.73519E-08 | 2.94055E-07 | K14085 |
| *uncharacterized protein_42826* | 17.96882652 | 108.5142359 | 6.039027414 | 2.594316222 | 1.7568E-08 | 2.96953E-07 | - |
| *TSJT1* | 1448.775635 | 5317.542075 | 3.670369618 | 1.875925354 | 1.76752E-08 | 2.98354E-07 | - |
| *TPC1* | 257.7986047 | 597.8612121 | 2.319101815 | 1.21356616 | 1.78844E-08 | 3.01687E-07 | K16900 |
| *At1g47710* | 86.46628218 | 240.5614535 | 2.782141749 | 1.476195926 | 1.81891E-08 | 3.06624E-07 | K13963 |
| *PP2A15* | 908.2446615 | 1921.867356 | 2.11602384 | 1.081355882 | 1.83499E-08 | 3.08953E-07 | - |
| *GOLS4* | 3.30555851 | 45.9111704 | 13.88908115 | 3.795879254 | 1.83513E-08 | 3.08953E-07 | K18819 |
| *RUN1* | 0 | 23.42521387 | Inf | Inf | 1.84817E-08 | 3.10945E-07 | - |
| *At3g55350* | 178.7866155 | 434.3611203 | 2.429494619 | 1.280656237 | 1.87857E-08 | 3.15647E-07 | - |
| *DRB7* | 910.2450987 | 1923.266907 | 2.112911028 | 1.079232018 | 1.97189E-08 | 3.30893E-07 | - |
| *ERDJ7* | 554.3515296 | 1198.507595 | 2.161999257 | 1.112366028 | 2.08589E-08 | 3.49567E-07 | K19371 |
| *CHIT1* | 348.5051444 | 778.9316249 | 2.235064926 | 1.160316741 | 2.11053E-08 | 3.53005E-07 | - |
| *Os07g0679700* | 441.0139298 | 1157.112255 | 2.623754436 | 1.391632701 | 2.15602E-08 | 3.60145E-07 | - |
| *MED26B* | 116.874464 | 303.8095617 | 2.59945202 | 1.378207527 | 2.17306E-08 | 3.62756E-07 | - |
| *BZIP53* | 13.32764724 | 72.08713799 | 5.408841986 | 2.435319751 | 2.21995E-08 | 3.70343E-07 | - |
| *BZR2* | 570.9830126 | 1230.898946 | 2.155754058 | 1.108192596 | 2.24052E-08 | 3.73532E-07 | - |
| *At3g61320* | 189.1907691 | 452.5924676 | 2.392254494 | 1.258370875 | 2.25786E-08 | 3.76178E-07 | - |
| *uncharacterized protein_40185* | 8.420260884 | 58.32350483 | 6.926567434 | 2.79214058 | 2.28605E-08 | 3.80629E-07 | - |
| *DVL11* | 157.8423313 | 456.4707699 | 2.891941382 | 1.53203831 | 2.33639E-08 | 3.88506E-07 | - |
| *DET2* | 190.9860299 | 456.148345 | 2.388385921 | 1.256035969 | 2.42559E-08 | 4.02297E-07 | K09591 |
| *uncharacterized protein_07876* | 188.7104769 | 471.6804327 | 2.499492558 | 1.321635231 | 2.43456E-08 | 4.03524E-07 | - |
| *uncharacterized protein_23012* | 212.5330958 | 607.6271362 | 2.858976547 | 1.515498785 | 2.61452E-08 | 4.32516E-07 | - |
| *PP2A* | 1248.410379 | 2590.981506 | 2.075424515 | 1.053406461 | 2.64046E-08 | 4.36328E-07 | K04382 |
| *At1g60710* | 33.29060234 | 3170.003649 | 95.22217762 | 6.573225717 | 2.71593E-08 | 4.48136E-07 | - |
| *HSL1* | 22.77431716 | 96.23234658 | 4.225476703 | 2.07911411 | 2.72473E-08 | 4.49301E-07 | - |
| *serinc* | 484.6202192 | 1052.189513 | 2.17116305 | 1.118468074 | 2.75857E-08 | 4.54298E-07 | - |
| *ALY2* | 940.2062966 | 1967.112436 | 2.092213638 | 1.065030174 | 2.81954E-08 | 4.63745E-07 | K12881 |
| *RSL1* | 141.4181932 | 352.3740236 | 2.491716345 | 1.317139842 | 2.82389E-08 | 4.64163E-07 | K11975 |
| *PXG* | 8.4513788 | 62.77745092 | 7.428072082 | 2.892987815 | 2.94978E-08 | 4.83926E-07 | K17991 |
| *ERF2* | 2.54353299 | 37.22778163 | 14.63624878 | 3.871473939 | 2.97616E-08 | 4.87942E-07 | K09286 |
| *Os07g0301200* | 326.316604 | 729.5492376 | 2.235709825 | 1.160732951 | 3.00327E-08 | 4.92072E-07 | K14811 |
| *MAOM* | 19.38922309 | 87.66382905 | 4.521265685 | 2.176726698 | 3.02066E-08 | 4.94607E-07 | K00028 |
| *PDP5* | 256.7482206 | 587.5232556 | 2.288324547 | 1.19429168 | 3.1026E-08 | 5.07377E-07 | - |
| *HSL1* | 1114.320508 | 2943.04152 | 2.64110864 | 1.401143647 | 3.16412E-08 | 5.1678E-07 | - |
| *DJ1D* | 1057.376147 | 2200.5816 | 2.081171971 | 1.057396182 | 3.17341E-08 | 5.17968E-07 | K18881 |
| *INPS1* | 1967.986525 | 4622.495208 | 2.348844949 | 1.231951482 | 3.36288E-08 | 5.47155E-07 | K01858 |
| *RR41* | 78.41244438 | 219.300019 | 2.796750194 | 1.483751398 | 3.39274E-08 | 5.51664E-07 | - |
| *MUB3* | 125.1583377 | 317.4263567 | 2.536198248 | 1.342667522 | 3.4255E-08 | 5.56639E-07 | - |
| *DRE22* | 96.69371949 | 379.1496113 | 3.921140001 | 1.971273153 | 3.47035E-08 | 5.62859E-07 | - |
| *ATHB-15* | 325.0119862 | 930.9919843 | 2.864485077 | 1.518275822 | 3.53862E-08 | 5.73207E-07 | K09338 |
| *Os02g0194200* | 771.1409203 | 1619.074861 | 2.09958364 | 1.070103262 | 3.60077E-08 | 5.8254E-07 | - |
| *CRK25* | 10.68042383 | 101.0762612 | 9.463693839 | 3.242403402 | 3.62549E-08 | 5.85802E-07 | - |
| *SRP* | 963.8182471 | 2423.295317 | 2.514265863 | 1.330137211 | 3.84087E-08 | 6.18657E-07 | - |
| *NFD4* | 93.9412556 | 251.5164775 | 2.677380411 | 1.420822136 | 3.89112E-08 | 6.26359E-07 | - |
| *PYL8* | 188.1210459 | 443.9877287 | 2.360117267 | 1.238858544 | 4.11213E-08 | 6.58632E-07 | K14496 |
| *At2g30600/At2g30610* | 212.6182601 | 494.5400141 | 2.325952691 | 1.217821753 | 4.24677E-08 | 6.79774E-07 | - |
| *BSK2* | 200.56908 | 467.8123182 | 2.332424909 | 1.221830635 | 4.41441E-08 | 7.0529E-07 | K14500 |
| *RMR2* | 110.7472428 | 289.6148009 | 2.615097168 | 1.386864553 | 4.47653E-08 | 7.13884E-07 | K15692 |
| *URT1* | 574.9930616 | 1219.101444 | 2.120202008 | 1.084201728 | 4.48895E-08 | 7.15089E-07 | - |
| *UNC* | 8.038368752 | 118.0498997 | 14.68580297 | 3.876350244 | 4.58845E-08 | 7.30371E-07 | - |
| *uncharacterized protein_06213* | 0.364592412 | 25.14301381 | 68.96197772 | 6.107729244 | 4.6066E-08 | 7.32352E-07 | - |
| *NAC018* | 41.01965856 | 288.6938306 | 7.037938411 | 2.815152889 | 4.62535E-08 | 7.34425E-07 | - |
| *LAR* | 0 | 48.63552876 | Inf | Inf | 4.65828E-08 | 7.39197E-07 | K13081 |
| *uncharacterized protein_39902* | 401.8709125 | 871.9506396 | 2.16972817 | 1.117514309 | 4.68145E-08 | 7.42415E-07 | - |
| *HSP12* | 1.477392147 | 31.40546026 | 21.25736238 | 4.409890693 | 4.92241E-08 | 7.79665E-07 | K13993 |
| *uncharacterized protein_29727* | 256.868959 | 580.486091 | 2.259853013 | 1.176228939 | 4.93488E-08 | 7.81159E-07 | - |
| *IAA11* | 56.17543621 | 450.0375293 | 8.011286777 | 3.002033988 | 4.99318E-08 | 7.89842E-07 | K14484 |
| *SKIP31* | 79.37306412 | 218.9573084 | 2.758584551 | 1.4639282 | 4.99589E-08 | 7.89842E-07 | - |
| *PCAP1* | 685.4294706 | 1433.253129 | 2.091029334 | 1.064213301 | 5.10651E-08 | 8.06339E-07 | - |
| *uncharacterized protein_37327* | 42.88231647 | 140.7011762 | 3.281100178 | 1.714179643 | 5.34263E-08 | 8.41038E-07 | - |
| *NHL13* | 49.30165846 | 390.9347014 | 7.929443221 | 2.987219568 | 5.42824E-08 | 8.53993E-07 | - |
| *TGA21* | 1327.4896 | 2707.845314 | 2.039824128 | 1.028444769 | 5.44445E-08 | 8.56018E-07 | K14431 |
| *CYP750A1* | 0.364592412 | 194.7757425 | 534.2287332 | 9.061313762 | 5.45533E-08 | 8.5668E-07 | - |
| *RRN3* | 433.0502013 | 929.3239499 | 2.145995885 | 1.10164731 | 5.72822E-08 | 8.96247E-07 | K15216 |
| *ERI2* | 423.8558554 | 909.8686147 | 2.146646326 | 1.102084517 | 6.11739E-08 | 9.55391E-07 | K18417 |
| *UBC16* | 319.9700433 | 703.5583412 | 2.198825659 | 1.13673322 | 6.21312E-08 | 9.69164E-07 | K10688 |
| *Os12g0481100* | 261.7882681 | 587.0900575 | 2.242614086 | 1.16518138 | 6.28043E-08 | 9.78602E-07 | K13181 |
| *PTPA* | 503.9231093 | 1067.702737 | 2.118781054 | 1.083234514 | 6.46106E-08 | 1.00418E-06 | K17605 |
| *FBL8* | 250.0870209 | 831.8856308 | 3.326384663 | 1.733955012 | 6.50386E-08 | 1.009E-06 | K10268 |
| *Os01g0192000* | 505.072555 | 1070.139928 | 2.118784553 | 1.083236896 | 6.59567E-08 | 1.02263E-06 | - |
| *BPS1* | 0 | 26.80270489 | Inf | Inf | 6.86106E-08 | 1.06313E-06 | - |
| *Os08g0174900* | 202.3353053 | 467.0524814 | 2.308309371 | 1.206836594 | 6.87332E-08 | 1.06375E-06 | - |
| *uncharacterized protein_36665* | 169.7600554 | 400.3618056 | 2.358398179 | 1.237807316 | 7.06536E-08 | 1.0915E-06 | - |
| *LCB2a* | 1206.301514 | 2448.276891 | 2.029572924 | 1.021176178 | 7.26992E-08 | 1.12175E-06 | K00654 |
| *AHP1* | 1.453201311 | 30.75858241 | 21.16608495 | 4.403682537 | 7.30346E-08 | 1.12625E-06 | K14490 |
| *At2g46620* | 205.8844231 | 472.3825702 | 2.294406557 | 1.198121052 | 7.45979E-08 | 1.14967E-06 | K08900 |
| *uncharacterized protein_26639* | 292.798027 | 644.840494 | 2.202338932 | 1.139036511 | 7.69553E-08 | 1.18316E-06 | - |
| *IAA16* | 28.41602071 | 105.6628383 | 3.718424877 | 1.894691626 | 7.86577E-08 | 1.20861E-06 | K14484 |
| *HMA5* | 33.59636852 | 207.2723258 | 6.169486017 | 2.625150303 | 7.93708E-08 | 1.21714E-06 | K17686 |
| *DTX41* | 285.9224687 | 631.7066824 | 2.209363556 | 1.143630837 | 7.94022E-08 | 1.21714E-06 | K03327 |
| *DGK1* | 102.6068056 | 264.6265684 | 2.579035249 | 1.366831491 | 7.9867E-08 | 1.2228E-06 | K00901 |
| *XRN3* | 122.1171556 | 303.1921048 | 2.482796978 | 1.311966295 | 8.36576E-08 | 1.27552E-06 | K12619 |
| *CATHB3* | 890.6091469 | 1925.089635 | 2.161542626 | 1.112061287 | 8.46592E-08 | 1.29002E-06 | K01363 |
| *NLP3* | 487.1967443 | 1025.14803 | 2.104176684 | 1.07325585 | 8.50938E-08 | 1.29588E-06 | - |
| *POD1* | 178.4271555 | 416.6544905 | 2.335151785 | 1.223516328 | 8.68759E-08 | 1.31989E-06 | - |
| *AAE1* | 394.2802322 | 864.9396868 | 2.193718112 | 1.133378155 | 8.77012E-08 | 1.33164E-06 | - |
| *AO* | 369.5840353 | 906.0736175 | 2.451603779 | 1.293725833 | 8.87543E-08 | 1.34683E-06 | K00278 |
| *MPT1* | 59.40835028 | 223.7326931 | 3.76601424 | 1.913038455 | 8.88649E-08 | 1.34771E-06 | K15102 |
| *At1g01540* | 555.1860845 | 1598.938206 | 2.880004112 | 1.526070871 | 8.97627E-08 | 1.36053E-06 | - |
| *pip* | 174.6897802 | 409.1175398 | 2.34196608 | 1.227720181 | 9.14217E-08 | 1.38404E-06 | - |
| *AGAL2* | 556.0021352 | 1357.856589 | 2.442178731 | 1.288168788 | 9.16306E-08 | 1.38638E-06 | K07407 |
| *PAP18* | 1527.342273 | 3061.552685 | 2.004496791 | 1.003240108 | 9.20353E-08 | 1.39169E-06 | - |
| *SbtS* | 173.027533 | 521.8672803 | 3.016093863 | 1.592681327 | 9.97196E-08 | 1.50434E-06 | - |
| *CRK8* | 1.104149876 | 28.40804374 | 25.72843087 | 4.685291567 | 9.99117E-08 | 1.50636E-06 | - |
| *MYB2* | 16.52082951 | 245.7686012 | 14.876287 | 3.894942581 | 9.99765E-08 | 1.50645E-06 | K09422 |
| *uncharacterized protein_01942* | 308.3167173 | 670.5497849 | 2.174873262 | 1.120931332 | 1.05595E-07 | 1.58739E-06 | - |
| *IDH1* | 378.6371351 | 904.6530668 | 2.389234924 | 1.256548716 | 1.08387E-07 | 1.62556E-06 | K00030 |
| *At3g21360* | 299.5666793 | 652.7360505 | 2.178934093 | 1.123622559 | 1.09578E-07 | 1.64054E-06 | - |
| *SWEET16* | 5.897473172 | 46.75203779 | 7.927469347 | 2.986860394 | 1.10097E-07 | 1.64639E-06 | K15382 |
| *ctdspl2b* | 399.8684506 | 1072.688808 | 2.682604256 | 1.423634241 | 1.10391E-07 | 1.64982E-06 | K17616 |
| *PLIP2* | 12.5188909 | 114.2725058 | 9.128005566 | 3.190299671 | 1.10894E-07 | 1.65638E-06 | - |
| *uncharacterized protein_51532* | 132.8406848 | 327.3184808 | 2.463992723 | 1.300997996 | 1.13096E-07 | 1.68731E-06 | - |
| *GATA21* | 73.812511 | 202.3554799 | 2.741479421 | 1.454954644 | 1.16742E-07 | 1.73768E-06 | - |
| *B3GALT7* | 36.64271895 | 138.898286 | 3.790610794 | 1.922430333 | 1.19687E-07 | 1.77841E-06 | - |
| *uncharacterized protein_12346* | 80.09873146 | 215.0294264 | 2.684554704 | 1.424682804 | 1.20186E-07 | 1.78376E-06 | - |
| *ANT1* | 29.41479335 | 240.5977538 | 8.179481355 | 3.032009368 | 1.20289E-07 | 1.78427E-06 | K14209 |
| *CYTB5-B* | 67.07181882 | 187.5482863 | 2.796230811 | 1.483483451 | 1.21177E-07 | 1.7964E-06 | - |
| *MYB2* | 4.012275277 | 72.42667801 | 18.05127341 | 4.174028709 | 1.22901E-07 | 1.8209E-06 | K09422 |
| *TIFY6A* | 77.37373124 | 209.1114577 | 2.70261566 | 1.43435636 | 1.28578E-07 | 1.89844E-06 | K13464 |
| *uncharacterized protein_33679* | 5.161361267 | 43.93757426 | 8.512788 | 3.089631702 | 1.28835E-07 | 1.90115E-06 | - |
| *uncharacterized protein_02007* | 2602.023072 | 5778.617634 | 2.220817215 | 1.151090657 | 1.29131E-07 | 1.90442E-06 | - |
| *AAP2* | 0.364592412 | 23.55096545 | 64.59532524 | 6.013357856 | 1.36669E-07 | 2.00637E-06 | - |
| *MED33A* | 385.4623154 | 906.2586113 | 2.351095231 | 1.233332976 | 1.38154E-07 | 2.02587E-06 | - |
| *AAP4* | 269.7822331 | 713.7939949 | 2.64581543 | 1.403712424 | 1.38997E-07 | 2.03706E-06 | - |
| *znf593* | 265.4673277 | 613.5866442 | 2.31134524 | 1.208732768 | 1.39522E-07 | 2.04282E-06 | K14821 |
| *X13* | 1076.0102 | 2158.203142 | 2.005745989 | 1.004138912 | 1.39636E-07 | 2.04293E-06 | K13344 |
| *At5g47540* | 126.2486622 | 306.8853158 | 2.430800536 | 1.281431515 | 1.4206E-07 | 2.07721E-06 | K08272 |
| *YPQ1* | 29.88830936 | 810.3396642 | 27.11226167 | 4.760873561 | 1.47653E-07 | 2.15653E-06 | - |
| *BicC* | 570.869065 | 1172.273917 | 2.053490002 | 1.038077923 | 1.49399E-07 | 2.17943E-06 | - |
| *MFPA* | 57.34174999 | 166.2860914 | 2.899913091 | 1.536009664 | 1.54304E-07 | 2.24602E-06 | K10527 |
| *OXI1* | 2.203131414 | 60.69174447 | 27.54794566 | 4.783872831 | 1.55368E-07 | 2.26023E-06 | - |
| *OPR1* | 63.98746645 | 180.2607704 | 2.817126235 | 1.494224212 | 1.57664E-07 | 2.29232E-06 | K05894 |
| *Os12g0580900* | 67.63687209 | 187.5386845 | 2.772728524 | 1.47130637 | 1.58859E-07 | 2.3084E-06 | - |
| *sll0005* | 1264.107775 | 2563.099661 | 2.027595836 | 1.019770106 | 1.61402E-07 | 2.34137E-06 | K08869 |
| *CIPK26* | 25.3333192 | 96.23006323 | 3.798557247 | 1.925451564 | 1.65661E-07 | 2.39774E-06 | K07198 |
| *DLO1* | 0.739557463 | 27.99081099 | 37.84805424 | 5.242147228 | 1.68399E-07 | 2.43326E-06 | - |
| *PMK* | 702.7768095 | 1422.838229 | 2.024594736 | 1.017633152 | 1.72879E-07 | 2.49659E-06 | K00938 |
| *HIDM* | 33.96444245 | 180.2268121 | 5.306338015 | 2.407716578 | 1.75952E-07 | 2.53811E-06 | - |
| *MKK9* | 24.64045659 | 94.17875409 | 3.82211887 | 1.934372648 | 1.82885E-07 | 2.62486E-06 | K20604 |
| *MYB44* | 925.9640123 | 1855.82181 | 2.004205115 | 1.003030165 | 1.88181E-07 | 2.69484E-06 | K09422 |
| *SRD5A2* | 28.63202255 | 103.2647001 | 3.606615631 | 1.850645679 | 1.90296E-07 | 2.72059E-06 | K10258 |
| *SPAC24B11.05* | 22.65497786 | 134.1824063 | 5.92286636 | 2.566295534 | 1.91665E-07 | 2.73863E-06 | K07025 |
| *ADT2* | 79.61831724 | 255.8894812 | 3.213952393 | 1.684348559 | 1.92392E-07 | 2.74749E-06 | K05359 |
| *At1g56220* | 56.01987549 | 221.7803101 | 3.95895757 | 1.985120605 | 1.99415E-07 | 2.83952E-06 | - |
| *uncharacterized protein_40163* | 41.50692794 | 131.4078822 | 3.165926479 | 1.662627753 | 2.02371E-07 | 2.8788E-06 | K11666 |
| *CXXS1* | 3.37468546 | 36.63716831 | 10.85646907 | 3.440483056 | 2.05112E-07 | 2.91457E-06 | K03671 |
| *At3g01520* | 45.06473857 | 3157.49097 | 70.06566708 | 6.130635776 | 2.07163E-07 | 2.94045E-06 | - |
| *EPHX2* | 36.6685966 | 228.7238078 | 6.237593718 | 2.640989587 | 2.10703E-07 | 2.98741E-06 | - |
| *MPK* | 111.6200008 | 274.7386861 | 2.46137506 | 1.29946451 | 2.14513E-07 | 3.03974E-06 | K14512 |
| *CIPK8* | 191.5320465 | 430.6897765 | 2.248656475 | 1.169063279 | 2.15019E-07 | 3.04523E-06 | - |
| *MTR4* | 268.2474138 | 580.4770255 | 2.163961312 | 1.113674707 | 2.22839E-07 | 3.15078E-06 | K12598 |
| *VIR* | 83.3541065 | 243.5547809 | 2.921928998 | 1.546921122 | 2.26646E-07 | 3.19932E-06 | - |
| *SUS4* | 1683.022022 | 4427.845945 | 2.630890081 | 1.395550974 | 2.3785E-07 | 3.34827E-06 | K00695 |
| *KAO2* | 277.1945031 | 945.9690056 | 3.412654273 | 1.770894266 | 2.46619E-07 | 3.46792E-06 | K04123 |
| *uncharacterized protein_27700* | 15.14199541 | 70.20961902 | 4.636748138 | 2.213113364 | 2.47545E-07 | 3.47904E-06 | - |
| *RH14* | 446.2136309 | 919.8269854 | 2.061404945 | 1.043627938 | 2.47766E-07 | 3.48023E-06 | K12823 |
| *uncharacterized protein_18354* | 747.6493268 | 1497.70762 | 2.003222054 | 1.00232235 | 2.55668E-07 | 3.58144E-06 | - |
| *HSL1* | 101.2661173 | 405.3883133 | 4.003197952 | 2.001152957 | 2.56332E-07 | 3.58879E-06 | K00924 |
| *At1g60710* | 2.98934777 | 34.81134718 | 11.64513127 | 3.541654997 | 2.58744E-07 | 3.62058E-06 | - |
| *ANT* | 4.83825941 | 119.5142253 | 24.70190521 | 4.626550413 | 2.63237E-07 | 3.68145E-06 | K05863 |
| *At5g26710* | 420.2322773 | 868.4413276 | 2.066574546 | 1.047241406 | 2.65002E-07 | 3.7021E-06 | K01885 |
| *RLA2* | 643.7778865 | 1299.835095 | 2.019073849 | 1.013693679 | 2.68265E-07 | 3.74362E-06 | K02943 |
| *uncharacterized protein_31661* | 62.05219297 | 287.0587586 | 4.626085635 | 2.209791973 | 2.74547E-07 | 3.8292E-06 | - |
| *ATG101* | 62.9922113 | 174.3600343 | 2.767961795 | 1.46882403 | 2.76562E-07 | 3.85521E-06 | - |
| *NSP1* | 123.2125907 | 295.750129 | 2.400323922 | 1.263229109 | 2.83148E-07 | 3.94488E-06 | - |
| *CTNNBL1* | 193.2479516 | 431.4537796 | 2.232643482 | 1.158752894 | 2.94929E-07 | 4.10012E-06 | K12864 |
| *DODA* | 0.364592412 | 210.4592536 | 577.2452924 | 9.173040692 | 2.97534E-07 | 4.13187E-06 | K15777 |
| *STK11IP* | 387.9764173 | 805.6040612 | 2.076425332 | 1.054101994 | 2.99281E-07 | 4.15388E-06 | - |
| *HAT14* | 256.5131532 | 595.90756 | 2.323107227 | 1.216055746 | 3.02456E-07 | 4.19568E-06 | K09338 |
| *GLIP4* | 24.32945015 | 96.09554929 | 3.949762477 | 1.981765898 | 3.03494E-07 | 4.2078E-06 | - |
| *UBI11* | 1262.191128 | 3769.284697 | 2.98630264 | 1.57836038 | 3.04423E-07 | 4.21777E-06 | K08770 |
| *POX1* | 451.7443786 | 1748.532204 | 3.870623048 | 1.952565814 | 3.05327E-07 | 4.22637E-06 | K00318 |
| *NDL2* | 188.8278346 | 678.5025357 | 3.593233683 | 1.845282763 | 3.10087E-07 | 4.28304E-06 | - |
| *DEGP7* | 223.5523711 | 489.2076714 | 2.188335865 | 1.129834179 | 3.14632E-07 | 4.34348E-06 | - |
| *BGAL* | 77.0888471 | 482.1904727 | 6.25499655 | 2.645009089 | 3.154E-07 | 4.35174E-06 | - |
| *DEGP7* | 434.2422777 | 891.5538946 | 2.053125502 | 1.037821818 | 3.18301E-07 | 4.38941E-06 | - |
| *DDR4* | 660.3815102 | 1323.091203 | 2.003525512 | 1.00254088 | 3.30775E-07 | 4.55165E-06 | - |
| *ATL40* | 0 | 19.49544889 | Inf | Inf | 3.41034E-07 | 4.6753E-06 | K19038 |
| *uncharacterized protein_25742* | 177.2920315 | 398.9655029 | 2.25032958 | 1.170136312 | 3.42032E-07 | 4.68648E-06 | - |
| *At1g52360* | 287.021544 | 608.5976275 | 2.120390055 | 1.084329679 | 3.45877E-07 | 4.73663E-06 | K17302 |
| *At3g02290* | 181.0883409 | 405.9769646 | 2.241872461 | 1.164704207 | 3.49377E-07 | 4.77947E-06 | - |
| *uncharacterized protein_45656* | 432.2066297 | 887.2447367 | 2.052825375 | 1.037610909 | 3.57501E-07 | 4.88021E-06 | - |
| *Tmem209* | 523.6738247 | 1058.933058 | 2.02212333 | 1.01587099 | 3.60088E-07 | 4.91031E-06 | - |
| *SPL16* | 293.2074188 | 620.3238281 | 2.115648474 | 1.081099936 | 3.65794E-07 | 4.98282E-06 | - |
| *VSR4* | 157.1717929 | 358.9169349 | 2.283596364 | 1.191307671 | 3.68204E-07 | 5.00769E-06 | - |
| *INO1* | 8443.350677 | 19323.67742 | 2.288626656 | 1.194482135 | 3.71314E-07 | 5.04465E-06 | K01858 |
| *HSP21* | 105.5963332 | 258.8601993 | 2.451412768 | 1.293613425 | 3.82232E-07 | 5.18202E-06 | K13993 |
| *JOX1* | 0.364592412 | 23.29110954 | 63.88259536 | 5.997351021 | 3.9522E-07 | 5.33881E-06 | - |
| *CBP1* | 467.9217494 | 1083.251561 | 2.315027165 | 1.211029122 | 3.95252E-07 | 5.33881E-06 | K16296 |
| *CRJ34* | 60.31731552 | 481.8708477 | 7.9889306 | 2.998002397 | 3.99709E-07 | 5.39335E-06 | - |
| *uncharacterized protein_21041* | 0 | 19.26808715 | Inf | Inf | 4.14311E-07 | 5.57281E-06 | - |
| *uncharacterized protein_46853* | 27.90628329 | 98.70144515 | 3.536889672 | 1.822481217 | 4.29829E-07 | 5.76344E-06 | - |
| *uncharacterized protein_44979* | 872.5359415 | 2141.24212 | 2.454044605 | 1.295161472 | 4.52494E-07 | 6.0484E-06 | - |
| *cys-12* | 10.29680892 | 56.15867093 | 5.453987869 | 2.447311491 | 4.8495E-07 | 6.45662E-06 | K01738 |
| *ABCG14* | 20.31372931 | 588.7007272 | 28.98043575 | 4.857007382 | 4.89913E-07 | 6.5113E-06 | - |
| *D6PKL1* | 1.8333347 | 29.23149296 | 15.94443882 | 3.994981416 | 5.13307E-07 | 6.80111E-06 | - |
| *RAP2-9* | 138.0556032 | 319.0450834 | 2.310989746 | 1.208510859 | 5.21671E-07 | 6.90126E-06 | K09286 |
| *At1g67340* | 53.27416596 | 167.5049448 | 3.14420586 | 1.652695678 | 5.26602E-07 | 6.95932E-06 | - |
| *Os01g0693900* | 63.15103775 | 171.5140814 | 2.715934489 | 1.441448681 | 5.35541E-07 | 7.07019E-06 | K01056 |
| *HMA5* | 4.380313248 | 38.70861799 | 8.836952017 | 3.143548851 | 5.37355E-07 | 7.09049E-06 | K17686 |
| *PHM8* | 79.29866094 | 245.8702793 | 3.100560292 | 1.632528944 | 5.50212E-07 | 7.24526E-06 | K07025 |
| *OsI_07012* | 201.6803439 | 440.0827775 | 2.182080658 | 1.12570443 | 5.52561E-07 | 7.27246E-06 | - |
| *HSP17.3-B* | 10.61994674 | 56.86825375 | 5.354853009 | 2.420846973 | 5.58978E-07 | 7.35314E-06 | K13993 |
| *MYB78* | 0.364592412 | 21.62547587 | 59.31411391 | 5.890303533 | 5.60855E-07 | 7.37407E-06 | K09422 |
| *SCP1* | 101.788137 | 247.6042281 | 2.432545042 | 1.282466519 | 5.66531E-07 | 7.43941E-06 | K16297 |
| *CYP709B2* | 290.7545355 | 1393.297334 | 4.7920055 | 2.260629564 | 5.66693E-07 | 7.43941E-06 | - |
| *CAT3* | 1.843707339 | 261.521825 | 141.8456278 | 7.148177872 | 5.76987E-07 | 7.5668E-06 | K03781 |
| *uncharacterized protein_41851* | 148.6307146 | 337.4164391 | 2.270166297 | 1.182797984 | 5.88898E-07 | 7.7112E-06 | - |
| *MED13* | 383.7359657 | 781.2361255 | 2.035868919 | 1.025644675 | 5.94989E-07 | 7.78699E-06 | K15164 |
| *OsI_12825* | 11.36639532 | 58.49823187 | 5.146594873 | 2.363618222 | 6.04877E-07 | 7.9003E-06 | K16275 |
| *TPPD* | 2.215226832 | 62.41048128 | 28.17340436 | 4.816261999 | 6.07754E-07 | 7.93385E-06 | K01087 |
| *PILS7* | 126.7532025 | 341.2681493 | 2.692382856 | 1.428883575 | 6.16841E-07 | 8.04353E-06 | K07088 |
| *GOLS4* | 0.737834684 | 23.63747021 | 32.03626871 | 5.001634221 | 6.25358E-07 | 8.1471E-06 | K18819 |
| *DDB_G0289029* | 90.8517278 | 392.2221047 | 4.317167259 | 2.110084988 | 6.32628E-07 | 8.23346E-06 | K19476 |
| *ATL78* | 27.90624732 | 97.27781019 | 3.485879311 | 1.801522621 | 6.37155E-07 | 8.28399E-06 | K19040 |
| *SFC1* | 212.7685375 | 477.2841839 | 2.243208463 | 1.165563697 | 6.39047E-07 | 8.30018E-06 | K15100 |
| *DDB_G0268948* | 33.38381933 | 208.5787972 | 6.247900971 | 2.643371587 | 6.48125E-07 | 8.40959E-06 | - |
| *uncharacterized protein_40470* | 56.34487995 | 156.5249103 | 2.777979302 | 1.47403585 | 6.56073E-07 | 8.50841E-06 | K13456 |
| *NAC018* | 31.89436773 | 163.5608165 | 5.128203759 | 2.358453586 | 6.64361E-07 | 8.59421E-06 | - |
| *uncharacterized protein_22493* | 170.3025399 | 378.3488485 | 2.22162775 | 1.151617103 | 6.73145E-07 | 8.6947E-06 | - |
| *VAMP711* | 223.1982952 | 478.1531349 | 2.142279512 | 1.099146727 | 6.74519E-07 | 8.7037E-06 | K08515 |
| *Hgsnat* | 126.0811211 | 293.1348464 | 2.324970177 | 1.21721221 | 6.75851E-07 | 8.71651E-06 | K10532 |
| *MYB2* | 0.725739266 | 371.1558284 | 511.4175927 | 8.99835798 | 7.26193E-07 | 9.32365E-06 | K09422 |
| *EDS1B* | 91.93857795 | 226.3076947 | 2.461509627 | 1.299543382 | 7.34037E-07 | 9.41495E-06 | K18875 |
| *WRKY42* | 0.727462045 | 23.42246208 | 32.19750396 | 5.008876946 | 7.44891E-07 | 9.5494E-06 | - |
| *RPOT2-TOM* | 147.5593695 | 333.178506 | 2.257928501 | 1.174999803 | 7.59547E-07 | 9.71306E-06 | K10908 |
| *At4g33300* | 231.2834236 | 491.2023449 | 2.123811284 | 1.086655578 | 7.65408E-07 | 9.78313E-06 | - |
| *At4g33300* | 37.36662754 | 117.1241276 | 3.13445808 | 1.648216035 | 7.69097E-07 | 9.82052E-06 | - |
| *LHT1* | 41.82299482 | 146.4246483 | 3.501056033 | 1.807790152 | 7.75923E-07 | 9.89783E-06 | - |
| *AATP1* | 1256.674223 | 2733.797204 | 2.175422361 | 1.121295529 | 7.88322E-07 | 1.0051E-05 | K03301 |
| *BIOF* | 32.34190529 | 176.7830654 | 5.466068366 | 2.450503504 | 8.02487E-07 | 1.02063E-05 | K00652 |
| *PFK2* | 354.953723 | 993.4215117 | 2.798735292 | 1.484775042 | 8.09598E-07 | 1.02836E-05 | K00850 |
| *TPS5* | 80.4997398 | 376.683821 | 4.679317249 | 2.226298044 | 8.29146E-07 | 1.05089E-05 | K16055 |
| *uncharacterized protein_48480* | 242.6876122 | 605.8899086 | 2.496583584 | 1.319955208 | 8.45014E-07 | 1.06942E-05 | K19525 |
| *PTI1* | 327.5782679 | 669.1882211 | 2.042834604 | 1.030572402 | 8.73624E-07 | 1.10291E-05 | K13436 |
| *crcB* | 12.49477199 | 61.04414512 | 4.885574954 | 2.288528354 | 8.78825E-07 | 1.10893E-05 | - |
| *CBSCBS2* | 57.15163289 | 156.890436 | 2.745161041 | 1.456890785 | 9.1184E-07 | 1.1489E-05 | - |
| *PDIL1-5* | 113.150835 | 265.5616686 | 2.346970471 | 1.23079969 | 9.40925E-07 | 1.18265E-05 | K09580 |
| *PAP2* | 302.042765 | 620.1908463 | 2.053321311 | 1.037959403 | 9.4933E-07 | 1.19204E-05 | - |
| *DIR21* | 3.697787316 | 72.36175766 | 19.56893447 | 4.290493298 | 9.6278E-07 | 1.20657E-05 | - |
| *HCT* | 0.374965051 | 32.38168106 | 86.35919793 | 6.432277939 | 9.77027E-07 | 1.22324E-05 | K13065 |
| *SEC22* | 241.5525962 | 506.4688196 | 2.096722733 | 1.068136094 | 9.96196E-07 | 1.24541E-05 | K08517 |
| *AM9* | 311.7484703 | 638.2503837 | 2.047324829 | 1.033740019 | 1.0047E-06 | 1.25482E-05 | - |
| *TH* | 0 | 18.06950117 | Inf | Inf | 1.02127E-06 | 1.27305E-05 | K00500 |
| *URGT6* | 190.8875798 | 412.5724904 | 2.161337531 | 1.111924392 | 1.02886E-06 | 1.28126E-05 | - |
| *GID1C* | 365.0294843 | 867.4020194 | 2.376251938 | 1.248687803 | 1.04543E-06 | 1.29997E-05 | K14493 |
| *FKBP20-1* | 282.0073742 | 626.3018585 | 2.220870501 | 1.151125272 | 1.04881E-06 | 1.30295E-05 | K01802 |
| *smpd4* | 40.6601266 | 122.1961166 | 3.005305858 | 1.587511825 | 1.11834E-06 | 1.38464E-05 | - |
| *uncharacterized protein_01173* | 285.1329796 | 586.682807 | 2.05757611 | 1.040945797 | 1.14589E-06 | 1.41466E-05 | - |
| *At5g19680* | 170.4979332 | 372.3443842 | 2.183864504 | 1.126883348 | 1.14956E-06 | 1.41783E-05 | K17550 |
| *KPHMT1* | 211.2583766 | 458.8417615 | 2.171945884 | 1.118988158 | 1.15249E-06 | 1.42076E-05 | K00606 |
| *GFAT1* | 119.3403929 | 605.8511782 | 5.076664851 | 2.34388102 | 1.17718E-06 | 1.44772E-05 | K00820 |
| *uncharacterized protein_29057* | 280.2375736 | 575.1459135 | 2.052351175 | 1.03727761 | 1.23288E-06 | 1.5126E-05 | - |
| *uncharacterized protein_41869* | 0.749930102 | 27.06850339 | 36.09470177 | 5.173715179 | 1.31849E-06 | 1.61301E-05 | - |
| *GULLO5* | 0 | 17.68107392 | Inf | Inf | 1.32942E-06 | 1.62561E-05 | - |
| *PP2C06* | 363.5457481 | 1253.527982 | 3.448061182 | 1.785785374 | 1.33373E-06 | 1.62934E-05 | K14497 |
| *ABCG36* | 2.545255769 | 30.65059063 | 12.04224385 | 3.590032332 | 1.36821E-06 | 1.66907E-05 | - |
| *At5g51380* | 93.61122666 | 225.1799422 | 2.405480093 | 1.26632486 | 1.37329E-06 | 1.67368E-05 | K10268 |
| *PAPS1* | 303.9884041 | 617.0736005 | 2.029924801 | 1.021426283 | 1.41124E-06 | 1.71667E-05 | K14376 |
| *SIS8* | 141.2937505 | 404.9579351 | 2.866071102 | 1.519074401 | 1.48535E-06 | 1.80255E-05 | - |
| *CPL4* | 19.49291352 | 103.7171696 | 5.320762827 | 2.411633097 | 1.50245E-06 | 1.82244E-05 | K18999 |
| *UGT85A24* | 34.5156056 | 109.69637 | 3.178167328 | 1.668195083 | 1.51039E-06 | 1.83122E-05 | - |
| *THO2* | 288.1808517 | 584.9192518 | 2.029695063 | 1.021262996 | 1.61667E-06 | 1.95822E-05 | K12879 |
| *GH5FP* | 93.6682511 | 271.8778721 | 2.902561635 | 1.537326703 | 1.61971E-06 | 1.96098E-05 | - |
| *uncharacterized protein_06380* | 46.75647461 | 132.6331171 | 2.83667916 | 1.504202985 | 1.63419E-06 | 1.97572E-05 | - |
| *APSR1* | 34.43782879 | 161.4417084 | 4.687917737 | 2.228947254 | 1.67765E-06 | 2.02445E-05 | - |
| *At4g33300* | 2.550424106 | 882.2721635 | 345.9315497 | 8.434342786 | 1.67895E-06 | 2.02506E-05 | - |
| *SPCC777.06c* | 13.9565872 | 103.3584645 | 7.405711942 | 2.888638434 | 1.71924E-06 | 2.06784E-05 | - |
| *MED32* | 188.8846575 | 402.8936183 | 2.133013998 | 1.092893433 | 1.74481E-06 | 2.09662E-05 | - |
| *Vps13* | 27.21507153 | 91.80722625 | 3.373396471 | 1.754201886 | 1.74808E-06 | 2.09958E-05 | K19525 |
| *At5g63930* | 228.7225838 | 474.3831753 | 2.07405481 | 1.05245402 | 1.78911E-06 | 2.14384E-05 | - |
| *Rpp25l* | 12.38932283 | 90.85008232 | 7.332933655 | 2.874390487 | 1.84032E-06 | 2.2011E-05 | - |
| *CYSK* | 93.20707516 | 221.8126189 | 2.379783064 | 1.250830066 | 1.87282E-06 | 2.23893E-05 | K01738 |
| *ZAT5* | 39.22942931 | 381.481716 | 9.724375878 | 3.281605659 | 1.88865E-06 | 2.25575E-05 | - |
| *PR4B* | 0.737834684 | 22.46034184 | 30.44088645 | 4.927938466 | 1.93786E-06 | 2.30916E-05 | - |
| *CYP76T24* | 76.9644114 | 440.8144516 | 5.727510203 | 2.517908124 | 1.95926E-06 | 2.33141E-05 | - |
| *uncharacterized protein_09943* | 158.8202437 | 344.4661226 | 2.168905641 | 1.11696729 | 1.97234E-06 | 2.34373E-05 | - |
| *PCMP-E76* | 238.4593564 | 529.3292385 | 2.219788087 | 1.150421956 | 2.01268E-06 | 2.38725E-05 | - |
| *KCS11* | 252.8443077 | 518.3437798 | 2.050051213 | 1.035659951 | 2.03347E-06 | 2.41079E-05 | K15397 |
| *TSJT1* | 49.21178623 | 136.5632237 | 2.775010505 | 1.472493233 | 2.04088E-06 | 2.41661E-05 | - |
| *BAG6* | 15.39080198 | 65.17515929 | 4.234682467 | 2.082253795 | 2.07546E-06 | 2.45378E-05 | - |
| *MOT2* | 152.4685217 | 332.1704357 | 2.178616491 | 1.123412257 | 2.0851E-06 | 2.4625E-05 | - |
| *uncharacterized protein_29269* | 1.124895153 | 23.69601809 | 21.0650904 | 4.396782202 | 2.10519E-06 | 2.48322E-05 | - |
| *TCEA1* | 42.73203199 | 122.9944582 | 2.878273099 | 1.525203486 | 2.17422E-06 | 2.56229E-05 | - |
| *uncharacterized protein_13083* | 222.3446387 | 622.9862439 | 2.801894606 | 1.486402689 | 2.18037E-06 | 2.56836E-05 | K19525 |
| *uncharacterized protein_33506* | 84.79887373 | 204.4128199 | 2.410560552 | 1.26936867 | 2.18977E-06 | 2.57707E-05 | K13917 |
| *MES1* | 0.739557463 | 50.48740362 | 68.26704634 | 6.093117428 | 2.19592E-06 | 2.5822E-05 | - |
| *PLIP2* | 22.43039809 | 80.73033392 | 3.599148512 | 1.847655633 | 2.19614E-06 | 2.5822E-05 | - |
| *XYLA* | 122.9689166 | 276.7250069 | 2.250365496 | 1.170159338 | 2.28544E-06 | 2.68107E-05 | K01805 |
| *DIVARICATA* | 232.9698617 | 480.128322 | 2.060903151 | 1.043276709 | 2.29525E-06 | 2.68888E-05 | - |
| *HSP7M* | 315.9352857 | 637.9219574 | 2.019153878 | 1.013750861 | 2.30043E-06 | 2.69373E-05 | - |
| *uncharacterized protein_12040* | 194.8668634 | 408.7057426 | 2.097358861 | 1.068573729 | 2.41383E-06 | 2.81753E-05 | - |
| *NAC068* | 4.421803803 | 35.63570386 | 8.059087522 | 3.010616501 | 2.42517E-06 | 2.82819E-05 | - |
| *CBSDUF1* | 282.1194409 | 567.932861 | 2.013093671 | 1.009414304 | 2.4823E-06 | 2.89218E-05 | K16302 |
| *PDC4* | 487.0546564 | 3469.05889 | 7.122524844 | 2.832388749 | 2.50101E-06 | 2.91266E-05 | K01568 |
| *DMT1* | 259.4296408 | 569.2616293 | 2.194281377 | 1.133748537 | 2.543E-06 | 2.95621E-05 | K00558 |
| *Tm-1* | 157.0561798 | 337.8536856 | 2.151164545 | 1.105117882 | 2.65081E-06 | 3.06766E-05 | - |
| *PAT07* | 158.6666646 | 340.7622461 | 2.147661242 | 1.10276645 | 2.71222E-06 | 3.13415E-05 | K16675 |
| *ZIP4* | 75.16553938 | 185.8804373 | 2.472947561 | 1.306231648 | 2.71315E-06 | 3.13415E-05 | K14709 |
| *uncharacterized protein_42197* | 179.2204716 | 378.0217118 | 2.1092552 | 1.076733658 | 2.72768E-06 | 3.14952E-05 | - |
| *ESD4* | 276.3187023 | 556.7550805 | 2.014901909 | 1.010709606 | 2.75836E-06 | 3.18352E-05 | K08592 |
| *BAM1* | 2704.826118 | 8789.632573 | 3.249610951 | 1.700267007 | 2.76204E-06 | 3.18633E-05 | K01177 |
| *SWEET1A* | 20.18416124 | 118.7069543 | 5.881193325 | 2.556108915 | 2.77437E-06 | 3.19768E-05 | K15382 |
| *uncharacterized protein_32502* | 41.56223669 | 119.2865808 | 2.870071254 | 1.521086555 | 2.93756E-06 | 3.37669E-05 | - |
| *uncharacterized protein_49326* | 253.149078 | 534.979019 | 2.113296336 | 1.079495083 | 3.01611E-06 | 3.45616E-05 | - |
| *VQ22* | 22.72589952 | 80.39588785 | 3.537632813 | 1.822784312 | 3.05133E-06 | 3.49028E-05 | - |
| *IRL1* | 0.737834684 | 21.27642224 | 28.83629992 | 4.849814154 | 3.08466E-06 | 3.5237E-05 | - |
| *EMB2271* | 146.2529711 | 316.9649688 | 2.167237809 | 1.115857468 | 3.1039E-06 | 3.54362E-05 | K14793 |
| *NSP2* | 81.1701845 | 195.7961461 | 2.412168302 | 1.270330571 | 3.10486E-06 | 3.54362E-05 | - |
| *BABL* | 2.942688877 | 70.69229938 | 24.02302871 | 4.586346146 | 3.21268E-06 | 3.6618E-05 | - |
| *TH2* | 134.3493388 | 293.9318414 | 2.187817552 | 1.129492433 | 3.33371E-06 | 3.79301E-05 | - |
| *DHNAT1* | 1.479114927 | 55.10463974 | 37.25514411 | 5.219367738 | 3.48281E-06 | 3.94519E-05 | - |
| *uncharacterized protein_50989* | 232.9248106 | 473.0344244 | 2.030846019 | 1.022080857 | 3.53295E-06 | 3.99846E-05 | - |
| *OST3B* | 180.7358728 | 482.2585052 | 2.668305399 | 1.415923799 | 3.54548E-06 | 4.00911E-05 | K12669 |
| *SFC1* | 9.184009183 | 48.27681448 | 5.256616529 | 2.394134494 | 3.69507E-06 | 4.16177E-05 | K15100 |
| *At2g01680* | 90.82416333 | 212.202668 | 2.336412032 | 1.22429472 | 3.79834E-06 | 4.26687E-05 | - |
| *MYB305* | 0 | 32.41915154 | Inf | Inf | 3.806E-06 | 4.2736E-05 | K09422 |
| *HSD* | 1.114522514 | 122.7691758 | 110.1540563 | 6.783378812 | 3.84234E-06 | 4.30688E-05 | - |
| *CRK6* | 15.13162277 | 121.4083616 | 8.023485874 | 3.004229164 | 4.02382E-06 | 4.49461E-05 | - |
| *GT7* | 46.71326128 | 252.2908155 | 5.40083926 | 2.433183611 | 4.04972E-06 | 4.51961E-05 | K13496 |
| *PLIP2* | 11.37845477 | 53.53873334 | 4.705272764 | 2.234278358 | 4.05337E-06 | 4.52171E-05 | - |
| *ABCD1* | 12.85588288 | 57.06496566 | 4.438821213 | 2.150176601 | 4.06751E-06 | 4.53535E-05 | K05677 |
| *SEC23* | 148.0275444 | 316.5041079 | 2.138143338 | 1.096358572 | 4.06913E-06 | 4.53535E-05 | K14006 |
| *CRRSP55* | 0 | 16.21622101 | Inf | Inf | 4.07821E-06 | 4.5435E-05 | - |
| *NCER2* | 93.49895121 | 216.5039399 | 2.315576133 | 1.211371192 | 4.12301E-06 | 4.59143E-05 | K12349 |
| *CLT1* | 24.22751847 | 167.7112199 | 6.922344117 | 2.791260661 | 4.30486E-06 | 4.77737E-05 | - |
| *At1g33420* | 51.32332263 | 158.6453557 | 3.091096747 | 1.628118809 | 4.32258E-06 | 4.7929E-05 | - |
| *TAF1* | 212.1100226 | 432.8977113 | 2.040911156 | 1.029213381 | 4.32557E-06 | 4.79414E-05 | K03125 |
| *RAD4* | 233.3240095 | 471.6241773 | 2.021327245 | 1.015302907 | 4.38868E-06 | 4.85781E-05 | K10838 |
| *SEC23* | 27.87168385 | 89.94672499 | 3.227172262 | 1.690270589 | 4.5288E-06 | 4.99998E-05 | - |
| *PBL27* | 82.91016545 | 196.4421754 | 2.369337635 | 1.2444838 | 4.73194E-06 | 5.19523E-05 | - |
| *CYP78A4* | 57.62704441 | 773.9932863 | 13.43107727 | 3.747503119 | 4.82841E-06 | 5.29662E-05 | - |
| *NCER1* | 49.96526269 | 148.2165314 | 2.966391518 | 1.568709024 | 4.84489E-06 | 5.31243E-05 | K12349 |
| *PAB1* | 147.0375366 | 313.8586328 | 2.13454768 | 1.093930389 | 4.88426E-06 | 5.35331E-05 | K13126 |
| *MKK4* | 36.54253892 | 149.9393371 | 4.103145035 | 2.03673015 | 5.28351E-06 | 5.76385E-05 | K20604 |
| *RT* | 53.5247961 | 139.415617 | 2.604692165 | 1.381112878 | 5.29384E-06 | 5.77266E-05 | - |
| *CPK11* | 139.6868262 | 299.3652083 | 2.143116974 | 1.099710596 | 5.34887E-06 | 5.82132E-05 | K13412 |
| *APS1* | 2.196204333 | 42.08657745 | 19.16332502 | 4.260275999 | 5.34978E-06 | 5.82132E-05 | - |
| *HSF24* | 119.3523156 | 262.4783132 | 2.199189113 | 1.13697167 | 5.45487E-06 | 5.92524E-05 | K09419 |
| *At1g60420* | 177.6965214 | 398.2932931 | 2.241424255 | 1.164415747 | 5.46849E-06 | 5.9354E-05 | K17609 |
| *WRKY71* | 11.48566268 | 171.965974 | 14.97222919 | 3.904217132 | 5.53582E-06 | 6.00341E-05 | - |
| *Ifrd1* | 152.2732651 | 444.0604748 | 2.916207743 | 1.544093497 | 5.60122E-06 | 6.07177E-05 | - |
| *uncharacterized protein_24738* | 91.38731401 | 210.8371419 | 2.307072313 | 1.206063225 | 5.6185E-06 | 6.08794E-05 | - |
| *At1g18250* | 10.63376494 | 50.70456829 | 4.768261156 | 2.253463254 | 5.76363E-06 | 6.22419E-05 | - |
| *uncharacterized protein_27624* | 46.70278075 | 239.4324134 | 5.126727136 | 2.358038114 | 5.80885E-06 | 6.26775E-05 | - |
| *TOP3A* | 23.95444914 | 80.43432461 | 3.357803143 | 1.747517652 | 5.88725E-06 | 6.33904E-05 | K03165 |
| *CTU2* | 57.5576799 | 147.3978051 | 2.560871206 | 1.356634697 | 6.09986E-06 | 6.54601E-05 | K14169 |
| *DDB_G0268948* | 56.95465355 | 145.669867 | 2.557646441 | 1.354816845 | 6.21961E-06 | 6.66339E-05 | - |
| *mpaB&apos;* | 0 | 32.10446581 | Inf | Inf | 6.28774E-06 | 6.73076E-05 | - |
| *ABCI1* | 151.1498841 | 351.4209485 | 2.324983249 | 1.217220322 | 6.38469E-06 | 6.82748E-05 | - |
| *RRP5* | 316.6869168 | 640.8416518 | 2.023581076 | 1.016910652 | 6.40316E-06 | 6.84007E-05 | K14792 |
| *CRJ33* | 50.60967879 | 133.3177979 | 2.634235212 | 1.39738417 | 6.52869E-06 | 6.96141E-05 | - |
| *APY1* | 371.4884284 | 903.8375313 | 2.433016649 | 1.282746193 | 6.53031E-06 | 6.96141E-05 | K14641 |
| *PUMP5* | 42.81846575 | 215.2593839 | 5.027255884 | 2.329771123 | 6.57499E-06 | 7.00397E-05 | K15104 |
| *ABF2* | 316.5347227 | 659.2539128 | 2.082722259 | 1.058470462 | 6.6325E-06 | 7.05571E-05 | K14432 |
| *LECRKS5* | 66.68820391 | 163.0736374 | 2.445314581 | 1.290020075 | 6.865E-06 | 7.29097E-05 | - |
| *At2g48060/At2g48040/At2g48050* | 295.3518248 | 748.7399721 | 2.5350782 | 1.342030251 | 6.92938E-06 | 7.35327E-05 | - |
| *At4g33300* | 12.08693028 | 1819.519108 | 150.5360803 | 7.233965502 | 6.96602E-06 | 7.3891E-05 | - |
| *CCC1* | 152.9401782 | 320.0234902 | 2.092474941 | 1.065210345 | 7.16226E-06 | 7.58161E-05 | K13627 |
| *COI1B* | 72.0447498 | 173.1445665 | 2.40329194 | 1.265011911 | 7.18077E-06 | 7.59807E-05 | K13463 |
| *SELENOF* | 163.1091058 | 338.7394688 | 2.076766144 | 1.05433877 | 7.21181E-06 | 7.62777E-05 | - |
| *IAA27* | 164.8284134 | 341.0918224 | 2.069375148 | 1.049195209 | 7.85861E-06 | 8.28459E-05 | K14484 |
| *PME51* | 11.71196523 | 52.65439407 | 4.495777866 | 2.168570755 | 7.88844E-06 | 8.30922E-05 | - |
| *PNC1* | 0.729184824 | 58.46610875 | 80.18009535 | 6.325172228 | 7.89792E-06 | 8.3158E-05 | K00430 |
| *PLT4* | 3714.166033 | 9169.378796 | 2.468758455 | 1.303785689 | 7.94494E-06 | 8.36188E-05 | - |
| *YUC9* | 6.635307856 | 39.58789272 | 5.966248075 | 2.576823966 | 7.99733E-06 | 8.41357E-05 | K11816 |
| *LTA3* | 157.2599713 | 326.1076092 | 2.073684782 | 1.052196609 | 8.05798E-06 | 8.47391E-05 | K00627 |
| *CNGC8* | 56.27033292 | 171.5757816 | 3.049133934 | 1.608399522 | 8.06739E-06 | 8.47594E-05 | K05391 |
| *ERF2* | 0 | 82.24458105 | Inf | Inf | 8.11713E-06 | 8.52216E-05 | K09286 |
| *PLSP1* | 1306.918942 | 2871.999512 | 2.197534538 | 1.13588584 | 8.35779E-06 | 8.7434E-05 | K03100 |
| *uncharacterized protein_15056* | 54.79649422 | 281.2135357 | 5.131962176 | 2.359510537 | 8.35849E-06 | 8.7434E-05 | - |
| *MANA* | 45.56762085 | 122.3155546 | 2.684264667 | 1.424526927 | 8.37717E-06 | 8.75938E-05 | - |
| *THO2* | 201.4659858 | 404.0353059 | 2.005476529 | 1.003945082 | 8.39914E-06 | 8.7752E-05 | K12879 |
| *chi1* | 4.808864273 | 144.932814 | 30.13867844 | 4.913544252 | 8.40402E-06 | 8.77674E-05 | K20547 |
| *2-Aug* | 45.57451197 | 129.4294457 | 2.839952423 | 1.505866761 | 8.51954E-06 | 8.88654E-05 | K16585 |
| *CDCA7L* | 91.02957675 | 206.7336162 | 2.271059842 | 1.183365721 | 8.98746E-06 | 9.33671E-05 | - |
| *SULTR4;1* | 227.1552187 | 460.6645167 | 2.027972412 | 1.020038026 | 9.09154E-06 | 9.44101E-05 | K18059 |
| *HIPP23* | 94.94357459 | 213.4004734 | 2.247655771 | 1.168421103 | 9.11301E-06 | 9.45949E-05 | - |
| *ISU1* | 57.15687316 | 161.5776571 | 2.826915612 | 1.499228817 | 9.35381E-06 | 9.68596E-05 | - |
| *ENO1* | 190.4209766 | 1060.039455 | 5.566820806 | 2.476853644 | 9.48333E-06 | 9.80033E-05 | K01689 |
| *RPOT1-TOM* | 144.2451682 | 302.2605756 | 2.095464128 | 1.067269824 | 9.49811E-06 | 9.81166E-05 | K10908 |
| *PIRL4* | 27.2858853 | 225.7241665 | 8.272561585 | 3.048334127 | 9.84633E-06 | 0.000101469 | - |
| *OFUT20* | 2.192758775 | 25.68236832 | 11.7123546 | 3.549959233 | 9.86187E-06 | 0.000101588 | - |
| *uncharacterized protein_08103* | 19.99748261 | 69.79063933 | 3.489971247 | 1.803215151 | 1.06338E-05 | 0.000108842 | K06100 |
| *UGT86A1* | 1.454924091 | 73.72368334 | 50.6718418 | 5.663112363 | 1.06909E-05 | 0.000109382 | - |
| *UBI11* | 5.135447652 | 34.85757085 | 6.787640186 | 2.762910089 | 1.07865E-05 | 0.000110142 | K08770 |
| *uncharacterized protein_25719* | 2.925389157 | 28.11115639 | 9.609373274 | 3.264442341 | 1.08321E-05 | 0.00011055 | - |
| *FLS2* | 32.07755774 | 94.63673637 | 2.950247557 | 1.560836017 | 1.08351E-05 | 0.00011055 | K13420 |
| *AMY2* | 197.7768985 | 395.6626608 | 2.000550438 | 1.000397002 | 1.10131E-05 | 0.000112154 | K01176 |
| *BZIP44* | 15.75019009 | 60.97230674 | 3.871210849 | 1.952784887 | 1.10798E-05 | 0.000112778 | - |
| *NPF8.3* | 29.04496067 | 316.66654 | 10.90263277 | 3.446604654 | 1.11238E-05 | 0.00011318 | K14638 |
| *R12* | 7.281583497 | 61.36714502 | 8.427719746 | 3.07514234 | 1.12331E-05 | 0.000114202 | K00430 |
| *uncharacterized protein_24130* | 0.364592412 | 39.66973339 | 108.8057021 | 6.765610354 | 1.14122E-05 | 0.000115931 | - |
| *VAC14* | 133.0947676 | 279.9808446 | 2.103620222 | 1.07287427 | 1.14591E-05 | 0.000116316 | K15305 |
| *uncharacterized protein_09829* | 36.78621311 | 125.0440498 | 3.399209628 | 1.765199335 | 1.14876E-05 | 0.000116513 | - |
| *ncd-2* | 162.4143117 | 354.3195363 | 2.181578289 | 1.125372248 | 1.19036E-05 | 0.000120494 | - |
| *XAC3343* | 168.6177957 | 342.6122857 | 2.031886873 | 1.022820081 | 1.20357E-05 | 0.000121735 | - |
| *ufd1* | 65.07255786 | 157.0450905 | 2.413384316 | 1.271057674 | 1.21904E-05 | 0.000123252 | - |
| *MYB2* | 3.99845708 | 31.36017345 | 7.84306867 | 2.971418232 | 1.24581E-05 | 0.000125613 | K09422 |
| *DDB_G0268948* | 2.921943599 | 29.53798215 | 10.10901859 | 3.337571038 | 1.24731E-05 | 0.000125667 | - |
| *uncharacterized protein_23691* | 121.1994326 | 281.7887203 | 2.325000326 | 1.217230919 | 1.26555E-05 | 0.000127403 | - |
| *uncharacterized protein_44618* | 88.94054436 | 502.6019637 | 5.650988166 | 2.498503168 | 1.27383E-05 | 0.000128086 | - |
| *BETA-OHASE 1* | 2.946134435 | 27.73642924 | 9.414515817 | 3.2348869 | 1.29036E-05 | 0.000129697 | K15746 |
| *uncharacterized protein_28640* | 81.67801933 | 186.4873665 | 2.283201381 | 1.191058113 | 1.3132E-05 | 0.000131787 | - |
| *uncharacterized protein_35463* | 105.0916491 | 229.2960752 | 2.181867705 | 1.125563628 | 1.31554E-05 | 0.000131971 | - |
| *uncharacterized protein_15354* | 14.68232647 | 57.87008925 | 3.941479532 | 1.978737283 | 1.32633E-05 | 0.000132949 | - |
| *At1g17710* | 25.30923625 | 140.4730338 | 5.550267594 | 2.47255733 | 1.32775E-05 | 0.000133039 | K13248 |
| *uncharacterized protein_28782* | 45.53998445 | 120.125174 | 2.637795674 | 1.399332816 | 1.34396E-05 | 0.000134559 | - |
| *EAP3* | 12.12669806 | 51.95491092 | 4.284341102 | 2.099073346 | 1.35358E-05 | 0.000135416 | - |
| *TAF8* | 93.55606176 | 208.1558164 | 2.224931368 | 1.153760834 | 1.3798E-05 | 0.000137771 | K14649 |
| *rbm24* | 349.7113769 | 707.5004099 | 2.023098065 | 1.016566253 | 1.41434E-05 | 0.000141055 | - |
| *CRPK1* | 2.548701327 | 26.33202736 | 10.33154693 | 3.368984378 | 1.45537E-05 | 0.000144754 | - |
| *INV1* | 1.093777237 | 20.68349617 | 18.91015416 | 4.24108922 | 1.45604E-05 | 0.000144764 | K01193 |
| *CRJ34* | 0 | 64.27057293 | Inf | Inf | 1.49143E-05 | 0.000148054 | - |
| *PDAT1* | 4.75355552 | 33.17361155 | 6.978694456 | 2.802957169 | 1.49893E-05 | 0.000148741 | K00679 |
| *uncharacterized protein_34176* | 10.00478901 | 47.4340642 | 4.741135883 | 2.245232742 | 1.50211E-05 | 0.000148999 | - |
| *LECRK81* | 132.2290086 | 422.9269685 | 3.198443162 | 1.677369846 | 1.53846E-05 | 0.000152194 | - |
| *LYK5* | 9.246209052 | 44.60002595 | 4.823601294 | 2.270110663 | 1.5531E-05 | 0.000153524 | - |
| *SKIP5* | 31.84943161 | 93.13757438 | 2.924308839 | 1.548095684 | 1.57541E-05 | 0.000155429 | - |
| *uncharacterized protein_10251* | 0.362869633 | 62.02860102 | 170.9390794 | 7.417338448 | 1.58115E-05 | 0.000155816 | - |
| *uncharacterized protein_10512* | 2.204854193 | 24.91383715 | 11.29953955 | 3.49819208 | 1.58647E-05 | 0.000156281 | - |
| *uncharacterized protein_43301* | 268.1728019 | 855.4089409 | 3.189767698 | 1.67345136 | 1.64181E-05 | 0.000161423 | - |
| *CRPK1* | 15.48236102 | 58.9090536 | 3.80491409 | 1.92786388 | 1.65889E-05 | 0.000162972 | - |
| *chi1* | 0 | 108.4729888 | Inf | Inf | 1.65947E-05 | 0.000162972 | K20547 |
| *CCL9* | 280.7764109 | 1881.904652 | 6.702502698 | 2.744699895 | 1.67372E-05 | 0.000164245 | - |
| *FUM1* | 35.74429907 | 99.9764362 | 2.796989696 | 1.483874939 | 1.69041E-05 | 0.000165757 | K01679 |
| *CYP76T24* | 6.633585077 | 47.12341792 | 7.103763255 | 2.828583502 | 1.69265E-05 | 0.000165913 | - |
| *At1g73050* | 1.114522514 | 84.70276071 | 75.99914728 | 6.247911326 | 1.71794E-05 | 0.0001682 | - |
| *UBP23* | 153.9007979 | 313.0516557 | 2.034113273 | 1.02440002 | 1.72627E-05 | 0.000168951 | K11855 |
| *DHS* | 124.5516859 | 261.0599667 | 2.095997053 | 1.067636689 | 1.74959E-05 | 0.000170907 | K00809 |
| *At1g56140* | 3.307281289 | 28.39750504 | 8.586359174 | 3.102046523 | 1.75226E-05 | 0.000171038 | - |
| *uncharacterized protein_00488* | 95.38242633 | 273.495965 | 2.867362212 | 1.519724161 | 1.75406E-05 | 0.000171149 | - |
| *paxip1* | 35.9637105 | 100.5446256 | 2.795724474 | 1.483222186 | 1.75506E-05 | 0.000171182 | - |
| *At2g34160* | 97.81193222 | 213.1058014 | 2.178730105 | 1.123487491 | 1.83153E-05 | 0.000178099 | - |
| *MTR4* | 103.7941452 | 223.6071949 | 2.154333411 | 1.107241543 | 1.84284E-05 | 0.000178995 | K12598 |
| *GSTU19* | 11.81396884 | 50.96586807 | 4.314034407 | 2.109037683 | 1.88449E-05 | 0.00018275 | K00799 |
| *CRJ33* | 7.011995686 | 482.3021923 | 68.78244282 | 6.103968449 | 1.91166E-05 | 0.000185051 | - |
| *uncharacterized protein_04301* | 150.3500871 | 304.3982333 | 2.024596322 | 1.017634282 | 1.93104E-05 | 0.000186715 | - |
| *At4g33300* | 39.2708839 | 106.4428836 | 2.710478427 | 1.438547525 | 1.93426E-05 | 0.000186957 | - |
| *GPX4* | 10.23457309 | 432.5883825 | 42.26735975 | 5.401472091 | 1.94547E-05 | 0.000187969 | K00432 |
| *GSTU19* | 49.49178264 | 141.2428526 | 2.853864724 | 1.512916951 | 2.10873E-05 | 0.000202449 | K00799 |
| *DDB_G0268948* | 0.727462045 | 28.46023942 | 39.1226451 | 5.28993201 | 2.16484E-05 | 0.000207386 | - |
| *CDC25* | 101.4491776 | 219.0630169 | 2.159337533 | 1.110588773 | 2.16581E-05 | 0.000207386 | K18065 |
| *At3g03770* | 8.859148583 | 52.4636156 | 5.921970391 | 2.566077277 | 2.19556E-05 | 0.000210078 | - |
| *LEA34* | 0 | 15.578945 | Inf | Inf | 2.20913E-05 | 0.00021122 | - |
| *DLO1* | 3.685691898 | 43.62651837 | 11.83672417 | 3.565197963 | 2.3235E-05 | 0.000221289 | - |
| *RHY1A* | 158.4091363 | 317.3600311 | 2.003419995 | 1.002464898 | 2.33266E-05 | 0.000221875 | - |
| *At1g12340* | 76.07439673 | 172.6441081 | 2.269411464 | 1.182318206 | 2.34997E-05 | 0.000223356 | K20368 |
| *RUS4* | 93.62508082 | 204.1154347 | 2.180136272 | 1.124418315 | 2.36775E-05 | 0.00022456 | - |
| *OTU9* | 55.89023549 | 136.0874431 | 2.434905523 | 1.283865796 | 2.36789E-05 | 0.00022456 | - |
| *uncharacterized protein_25301* | 1.819516503 | 55.55669968 | 30.5337707 | 4.932333858 | 2.43697E-05 | 0.000230686 | - |
| *IFRD2* | 12.86108719 | 116.641868 | 9.069362978 | 3.181001221 | 2.45973E-05 | 0.000232609 | - |
| *APF1* | 105.4269542 | 224.4914166 | 2.129355042 | 1.09041652 | 2.4703E-05 | 0.000233325 | - |
| *R40C1* | 0 | 13.85060635 | Inf | Inf | 2.50724E-05 | 0.000236573 | - |
| *MYB2* | 0.364592412 | 16.24596339 | 44.55924711 | 5.477652951 | 2.54189E-05 | 0.000239383 | K09422 |
| *At4g35930* | 9.159818347 | 56.08642294 | 6.123093365 | 2.614260681 | 2.61792E-05 | 0.000245733 | - |
| *PME3* | 4.810587052 | 31.8880818 | 6.628729811 | 2.728732449 | 2.67297E-05 | 0.000250352 | K01051 |
| *GLB* | 122.029114 | 252.5463084 | 2.06955783 | 1.049322563 | 2.72051E-05 | 0.000254618 | - |
| *APM1* | 134.2749067 | 274.380128 | 2.043420731 | 1.030986279 | 2.76025E-05 | 0.000258055 | K08776 |
| *CYP71AU50* | 8.131722502 | 70.6675039 | 8.690348679 | 3.119414063 | 2.77695E-05 | 0.000259239 | - |
| *FDX3* | 161.5435642 | 595.3197795 | 3.685196513 | 1.881741553 | 2.81426E-05 | 0.000262287 | K02639 |
| *uncharacterized protein_46616* | 123.0259481 | 253.6519989 | 2.061776421 | 1.043887895 | 2.88315E-05 | 0.000268276 | - |
| *SULTR1;3* | 126.8084823 | 290.6545553 | 2.292075024 | 1.196654267 | 2.95697E-05 | 0.00027435 | K17470 |
| *Os06g0701100* | 301.4428748 | 672.535775 | 2.231055471 | 1.157726384 | 2.96563E-05 | 0.000274856 | K03257 |
| *UGT89B1* | 234.8688134 | 737.4614517 | 3.13988665 | 1.650712479 | 3.04857E-05 | 0.000281831 | K13496 |
| *GRF6* | 12.57079006 | 50.42782147 | 4.011507728 | 2.004144577 | 3.09156E-05 | 0.000285292 | - |
| *SUR1* | 110.8577954 | 232.2993663 | 2.095471639 | 1.067274996 | 3.11379E-05 | 0.000287137 | K00815 |
| *pgk* | 186.2134879 | 4031.131498 | 21.64790287 | 4.436155366 | 3.19174E-05 | 0.000293757 | K00927 |
| *MRD1* | 49.89258938 | 123.7186394 | 2.47969971 | 1.310165422 | 3.21398E-05 | 0.000295422 | K14787 |
| *NAAT1* | 20.29122529 | 66.90487383 | 3.297231826 | 1.721255325 | 3.21515E-05 | 0.000295423 | K00815 |
| *OPR11* | 295.4263789 | 921.639621 | 3.11969305 | 1.641404088 | 3.33246E-05 | 0.000305111 | K05894 |
| *RCOM_0464280* | 116.0434554 | 240.1125164 | 2.069160346 | 1.049045449 | 3.48353E-05 | 0.000318263 | - |
| *uncharacterized protein_42751* | 48.02304035 | 119.8598053 | 2.495881237 | 1.319549287 | 3.52736E-05 | 0.000321809 | - |
| *PME51* | 58.67220241 | 214.1861397 | 3.650555644 | 1.868116071 | 3.62327E-05 | 0.000330208 | - |
| *GBP1* | 96.93236211 | 239.0345 | 2.465992727 | 1.302168545 | 3.66586E-05 | 0.000333851 | - |
| *FOLT1* | 69.34408427 | 157.7659247 | 2.275117285 | 1.18594092 | 3.72917E-05 | 0.000339137 | K15115 |
| *TYRAAT2* | 132.5574587 | 267.2693983 | 2.016253186 | 1.011676813 | 3.73804E-05 | 0.00033973 | K15227 |
| *FBL3* | 15.0590143 | 55.5497614 | 3.688804612 | 1.883153375 | 3.79083E-05 | 0.000344256 | K10268 |
| *ChiC* | 1.092054457 | 41.28518107 | 37.80505706 | 5.240507327 | 3.79702E-05 | 0.000344697 | K01183 |
| *LAMP1* | 98.86600651 | 208.8998351 | 2.112959171 | 1.07926489 | 3.84687E-05 | 0.000348607 | K01301 |
| *D1* | 408.1762274 | 1265.247537 | 3.099758027 | 1.632155601 | 3.9784E-05 | 0.000359055 | K07513 |
| *AGL104* | 16.50877005 | 58.50645781 | 3.543962247 | 1.825363235 | 4.00455E-05 | 0.000361239 | K09260 |
| *SUR1* | 137.5459306 | 275.4510004 | 2.002611049 | 1.001882245 | 4.1708E-05 | 0.000375314 | K00815 |
| *CPRF2* | 56.24104567 | 133.3644901 | 2.371301751 | 1.245679259 | 4.28948E-05 | 0.000385588 | - |
| *AMT1-1* | 353.4838627 | 956.7879909 | 2.706737398 | 1.436554927 | 4.37393E-05 | 0.000392629 | K03320 |
| *At3g21360* | 19.2078207 | 63.41835677 | 3.301694543 | 1.723206655 | 4.39625E-05 | 0.000394495 | - |
| *Eef1akmt4* | 53.06509829 | 128.0565037 | 2.413196392 | 1.27094533 | 4.53269E-05 | 0.000405464 | - |
| *SGF11* | 93.61115473 | 199.0996716 | 2.126879774 | 1.088738485 | 4.55232E-05 | 0.000406937 | K11363 |
| *uncharacterized protein_23496* | 2.191035996 | 22.99788143 | 10.49635035 | 3.391815875 | 4.56296E-05 | 0.000407552 | - |
| *SSL3* | 638.7213559 | 1292.801871 | 2.024046729 | 1.017242598 | 4.62765E-05 | 0.000412666 | - |
| *AMY1.6* | 67.85638432 | 223.1183774 | 3.288097055 | 1.717252884 | 4.6394E-05 | 0.00041357 | K01176 |
| *uncharacterized protein_34114* | 2.546978548 | 24.20563022 | 9.503664739 | 3.248483943 | 4.65781E-05 | 0.00041478 | - |
| *TIFY10B* | 1.124895153 | 20.00734437 | 17.78596371 | 4.152667242 | 4.79023E-05 | 0.000425245 | K13464 |
| *NAC002* | 7.369661018 | 152.2225363 | 20.65529689 | 4.368439892 | 4.84891E-05 | 0.000430008 | - |
| *RSH1* | 60.53841377 | 140.9062114 | 2.327550437 | 1.218812431 | 4.85306E-05 | 0.000430226 | - |
| *ARGAH1* | 81.94078795 | 178.2776018 | 2.175688156 | 1.121471788 | 4.85472E-05 | 0.000430226 | K01476 |
| *uncharacterized protein_27363* | 18.32304629 | 2787.231706 | 152.1161745 | 7.249029753 | 4.93311E-05 | 0.000436872 | - |
| *SD25* | 62.35972498 | 401.1317717 | 6.432545554 | 2.685389769 | 4.96828E-05 | 0.000439381 | - |
| *CAT7* | 0 | 12.96172979 | Inf | Inf | 4.97921E-05 | 0.000440195 | - |
| *uncharacterized protein_22163* | 7.385237958 | 37.24833178 | 5.043619717 | 2.334459502 | 5.02438E-05 | 0.000443579 | - |
| *CHI4* | 0 | 21.00189995 | Inf | Inf | 5.19963E-05 | 0.000458263 | K01183 |
| *AAE6* | 17.2121061 | 139.5225837 | 8.106072722 | 3.019003118 | 5.22755E-05 | 0.00046025 | - |
| *MAPKKK17* | 2.908125402 | 25.13522689 | 8.643102829 | 3.111549325 | 5.39048E-05 | 0.000473459 | - |
| *SYT1* | 64.36928665 | 252.2667915 | 3.919055261 | 1.970505916 | 5.42983E-05 | 0.000476752 | - |
| *uncharacterized protein_30746* | 4.770855239 | 30.37106262 | 6.365957694 | 2.670377569 | 5.49697E-05 | 0.000481989 | - |
| *RCOM_0530710* | 48.49466086 | 118.1296224 | 2.435930477 | 1.284472959 | 5.59166E-05 | 0.000488952 | - |
| *HVA22A* | 0.364592412 | 15.58992273 | 42.7598661 | 5.41818543 | 5.59351E-05 | 0.000488952 | K17279 |
| *At1g64890* | 254.6777574 | 893.8621834 | 3.509777189 | 1.811379447 | 5.6354E-05 | 0.000492446 | - |
| *CAD* | 30.06268388 | 190.6379737 | 6.341349112 | 2.664789804 | 5.74936E-05 | 0.000501551 | K00083 |
| *CBSX6* | 63.60726113 | 144.5164704 | 2.272012154 | 1.183970553 | 5.87937E-05 | 0.00051185 | - |
| *TBL11* | 163.0883317 | 366.6947508 | 2.24844259 | 1.168926048 | 5.88929E-05 | 0.000512366 | - |
| *PAD4* | 37.85224606 | 118.4643807 | 3.129652609 | 1.646002527 | 5.97246E-05 | 0.000518586 | - |
| *RXW8* | 10.31407267 | 43.75005457 | 4.241782655 | 2.084670701 | 5.97289E-05 | 0.000518586 | - |
| *MSL2* | 47.90900616 | 116.4593632 | 2.430844897 | 1.281457844 | 6.02095E-05 | 0.000522581 | - |
| *At1g51550* | 87.84683905 | 186.8845855 | 2.127391123 | 1.089085298 | 6.05978E-05 | 0.000525774 | - |
| *At1g77540* | 91.28903657 | 192.4812718 | 2.108481797 | 1.076204566 | 6.10986E-05 | 0.000529583 | - |
| *At3g51250* | 78.57478831 | 170.7812941 | 2.173487167 | 1.120011578 | 6.15205E-05 | 0.000532701 | - |
| *CRJ34* | 13.59371757 | 88.04687953 | 6.47702728 | 2.69533182 | 6.17266E-05 | 0.000534263 | - |
| *DEGP7* | 109.3561694 | 223.311722 | 2.042058745 | 1.03002437 | 6.18079E-05 | 0.000534648 | - |
| *PHT1-4* | 72.54243487 | 297.4925979 | 4.100945859 | 2.035956697 | 6.19701E-05 | 0.000535871 | K08176 |
| *TIFY6A* | 0 | 12.68220177 | Inf | Inf | 6.21681E-05 | 0.000537312 | K13464 |
| *At1g30090* | 13.94101026 | 60.31995474 | 4.326799393 | 2.113300233 | 6.21785E-05 | 0.000537312 | - |
| *uncharacterized protein_35310* | 101.805149 | 210.116552 | 2.063908888 | 1.045379284 | 6.3954E-05 | 0.000551912 | - |
| *SSL3* | 100.1064859 | 208.040701 | 2.078194026 | 1.055330354 | 6.45485E-05 | 0.000556295 | - |
| *At3g22104* | 21.65972271 | 504.7071782 | 23.3016454 | 4.542359927 | 6.48329E-05 | 0.000558559 | - |
| *CYP78A4* | 302.5528692 | 874.0366652 | 2.888872505 | 1.530506535 | 6.51431E-05 | 0.000561043 | - |
| *At1g74360* | 49.78882703 | 130.0354381 | 2.611739337 | 1.385010917 | 6.53964E-05 | 0.000563036 | - |
| *AAE3* | 161.3063997 | 848.9305515 | 5.262844828 | 2.395842858 | 6.5759E-05 | 0.000565968 | - |
| *uncharacterized protein_11719* | 123.3975755 | 246.8261153 | 2.000250932 | 1.000180998 | 6.58409E-05 | 0.000566483 | K12501 |
| *uncharacterized protein_20926* | 93.18608523 | 195.679528 | 2.099879263 | 1.070306379 | 6.61318E-05 | 0.000568795 | - |
| *NAC018* | 1.45664687 | 28.45154504 | 19.53221857 | 4.287783922 | 6.62298E-05 | 0.000569448 | - |
| *uncharacterized protein_00552* | 7.309183928 | 43.44532147 | 5.943935999 | 2.571418582 | 6.63394E-05 | 0.000570009 | - |
| *MYB123* | 8.454824359 | 145.0077477 | 17.15088825 | 4.100211391 | 6.65538E-05 | 0.000571468 | K09422 |
| *uncharacterized protein_32689* | 11.8260283 | 46.62209056 | 3.942328683 | 1.979048063 | 6.76491E-05 | 0.000580098 | - |
| *uncharacterized protein_34960* | 0 | 15.86945074 | Inf | Inf | 6.86008E-05 | 0.000587475 | - |
| *Os03g0255100* | 125.6060479 | 449.5160026 | 3.578776738 | 1.839466544 | 6.93329E-05 | 0.000592953 | - |
| *HMGR1* | 23.03687 | 70.01387339 | 3.039209467 | 1.603696111 | 6.94387E-05 | 0.00059366 | K00021 |
| *At1g73050* | 0.362869633 | 24.34427183 | 67.08820362 | 6.067987209 | 6.98238E-05 | 0.000596754 | K00108 |
| *uncharacterized protein_15858* | 21.65968675 | 67.11897449 | 3.0987971 | 1.631708295 | 6.99447E-05 | 0.000597589 | - |
| *Os01g0970400* | 61.02227355 | 148.4056218 | 2.431991028 | 1.282137907 | 7.07148E-05 | 0.000603366 | K03259 |
| *uncharacterized protein_06470* | 269.4331457 | 574.5897359 | 2.132587416 | 1.09260488 | 7.22424E-05 | 0.000614766 | - |
| *ALDH7B4* | 148.2815984 | 318.0412585 | 2.144846441 | 1.100874362 | 7.34227E-05 | 0.000623983 | K14085 |
| *CBSDUF1* | 60.9393284 | 138.7600448 | 2.277019594 | 1.187146706 | 7.37108E-05 | 0.000626225 | K16302 |
| *PP2C06* | 22.31292544 | 88.94653904 | 3.986323501 | 1.995058793 | 7.39125E-05 | 0.000627523 | K14497 |
| *TPPD* | 0 | 23.33777224 | Inf | Inf | 7.45811E-05 | 0.000632573 | K01087 |
| *GIP2* | 0.374965051 | 28.51834828 | 76.05601696 | 6.248990482 | 7.47112E-05 | 0.000633258 | - |
| *PDC4* | 1745.040719 | 6163.129307 | 3.531796846 | 1.820402359 | 7.78906E-05 | 0.000656524 | K01568 |
| *DGK1* | 12.52757673 | 47.74790129 | 3.811423576 | 1.930329949 | 7.92199E-05 | 0.000667073 | K00901 |
| *CYP71AU50* | 122.9741857 | 265.2692689 | 2.157113441 | 1.109102049 | 8.10087E-05 | 0.000681242 | - |
| *FACE1* | 70.94587616 | 155.4109462 | 2.190556443 | 1.131297389 | 8.23278E-05 | 0.000690978 | K06013 |
| *NIT4A* | 1933.987839 | 4571.467366 | 2.363751868 | 1.241078598 | 8.3087E-05 | 0.000697122 | K13035 |
| *uncharacterized protein_48461* | 66.54471683 | 148.2454638 | 2.227757075 | 1.155591923 | 8.77807E-05 | 0.000732437 | - |
| *PAZX* | 90.37834855 | 188.1403344 | 2.08169697 | 1.057760073 | 8.98424E-05 | 0.000747939 | K13963 |
| *ICL 8* | 1.819516503 | 23.31265539 | 12.81255507 | 3.6794863 | 9.03226E-05 | 0.000751207 | K01637 |
| *GDPDL1* | 253.9933795 | 602.082675 | 2.370466019 | 1.245170712 | 9.18923E-05 | 0.00076352 | - |
| *R3HDM1* | 105.5271991 | 213.4695305 | 2.022886349 | 1.016415268 | 9.3742E-05 | 0.000777023 | - |
| *uncharacterized protein_27648* | 66.15775716 | 146.2320255 | 2.210353431 | 1.144277072 | 9.46134E-05 | 0.000781579 | - |
| *SBT2.5* | 32.50773075 | 86.89348297 | 2.673009803 | 1.418465129 | 9.5369E-05 | 0.000787062 | - |
| *At4g32285* | 97.19843244 | 199.3238426 | 2.050689889 | 1.03610934 | 9.7605E-05 | 0.000803194 | - |
| *SIS8* | 29.84847675 | 81.18915567 | 2.720043517 | 1.443629733 | 9.78658E-05 | 0.000804567 | - |
| *PHOS34* | 60.46067293 | 139.7348603 | 2.311169452 | 1.20862304 | 9.82354E-05 | 0.000807137 | - |
| *JAL3* | 94.55996677 | 214.5910868 | 2.269365082 | 1.18228872 | 9.8845E-05 | 0.000811577 | - |
| *UGT72B1* | 3.328026567 | 28.29229445 | 8.501222537 | 3.087670326 | 0.000100261 | 0.000822661 | K08237 |
| *NPC6* | 15.46509727 | 72.14608633 | 4.665091017 | 2.221905229 | 0.000100601 | 0.000824945 | K01114 |
| *PPC4-2* | 97.07061601 | 198.3201039 | 2.043049813 | 1.03072438 | 0.000103575 | 0.000847163 | K17499 |
| *ATL23* | 8.459992696 | 38.0370329 | 4.496107061 | 2.168676389 | 0.00010433 | 0.000852561 | - |
| *TP53I3* | 1360.862949 | 2775.842493 | 2.039766382 | 1.028403928 | 0.000106378 | 0.000867059 | - |
| *Os04g0338000* | 235.7984515 | 719.4012219 | 3.050915802 | 1.609242365 | 0.000107947 | 0.000879003 | - |
| *CYP71AU50* | 25.69105645 | 73.2395297 | 2.850779213 | 1.51135631 | 0.000108242 | 0.000881128 | - |
| *TFCA* | 41.41185141 | 102.4303705 | 2.473455474 | 1.306527929 | 0.000108412 | 0.000881957 | K17292 |
| *C7A12* | 56.96158063 | 129.6375449 | 2.275876889 | 1.186422519 | 0.000108658 | 0.000883673 | - |
| *SCPL50* | 35.99299775 | 523.4667947 | 14.54357312 | 3.862309855 | 0.000110814 | 0.000898935 | K09645 |
| *Herc6* | 105.3492494 | 212.1762818 | 2.014027467 | 1.010083359 | 0.000111478 | 0.000903748 | - |
| *FBL4* | 93.53523747 | 191.3069744 | 2.045293085 | 1.032307592 | 0.000111826 | 0.000906282 | K10268 |
| *CYP71AU50* | 71.07555213 | 183.5269226 | 2.582138543 | 1.36856641 | 0.000112159 | 0.000908697 | - |
| *ADH2* | 10.94128985 | 59.31443058 | 5.421155218 | 2.438600315 | 0.000112296 | 0.000909231 | K18857 |
| *UAM1* | 1.46357395 | 18.87371731 | 12.89563626 | 3.688811052 | 0.000112364 | 0.000909493 | K13379 |
| *BTS* | 70.32210454 | 218.0667915 | 3.100970782 | 1.632719933 | 0.000113218 | 0.000915835 | K16276 |
| *OTU9* | 54.34547514 | 126.8702034 | 2.334512728 | 1.223121454 | 0.000117719 | 0.000947598 | - |
| *NPF7.3* | 1.104149876 | 17.38234224 | 15.74273803 | 3.976614576 | 0.000117927 | 0.000948841 | K14638 |
| *ALMT9* | 0 | 35.02188599 | Inf | Inf | 0.000119775 | 0.000961994 | - |
| *SAT1* | 81.09416643 | 234.3700648 | 2.890097711 | 1.531118269 | 0.000119799 | 0.000961994 | K00640 |
| *smek1* | 1.8333347 | 20.08331043 | 10.95452479 | 3.453454997 | 0.000119823 | 0.000961994 | K19525 |
| *LBD11* | 2.954784295 | 85.23220095 | 28.84549004 | 4.850273868 | 0.000119921 | 0.000962479 | - |
| *DBR* | 81.50179236 | 251.5969625 | 3.087011405 | 1.626210813 | 0.00012114 | 0.000970742 | K07119 |
| *CEPR1* | 146.6279362 | 350.8673281 | 2.392909136 | 1.258765616 | 0.000122646 | 0.00098159 | - |
| *SUN3* | 10.98278041 | 69.68864896 | 6.345264712 | 2.665680352 | 0.000123732 | 0.000989578 | - |
| *alaS* | 66.57066641 | 251.2048945 | 3.773507283 | 1.915906059 | 0.000125193 | 0.001000107 | - |
| *uncharacterized protein_23825* | 16.88201232 | 55.41296411 | 3.282367235 | 1.714736658 | 0.000128829 | 0.001025323 | - |
| *GMPM1* | 1.8333347 | 32.89589846 | 17.94320397 | 4.165365618 | 0.000130523 | 0.001038481 | - |
| *CSA* | 69.74977873 | 581.9884716 | 8.34394721 | 3.06073003 | 0.000131771 | 0.001046794 | K00432 |
| *AAP2* | 4.015720836 | 26.57033742 | 6.616579815 | 2.726085664 | 0.000132393 | 0.001051081 | - |
| *PAP2* | 32.04292234 | 144.7745408 | 4.518144109 | 2.175730287 | 0.00013278 | 0.00105383 | - |
| *GALS1* | 29.8225991 | 210.6232764 | 7.06253924 | 2.820186978 | 0.000135442 | 0.001073201 | - |
| *ATL60* | 35.98955219 | 91.20516677 | 2.534212326 | 1.341537404 | 0.000135997 | 0.001076369 | - |
| *PCMP-H81* | 14.63390883 | 82.11818652 | 5.611500486 | 2.488386591 | 0.000136966 | 0.001083376 | - |
| *Os01g0810000* | 0 | 23.19647623 | Inf | Inf | 0.000141581 | 0.00111644 | K14769 |
| *RPP25L* | 2.581542023 | 22.19000605 | 8.595640069 | 3.103605073 | 0.000141784 | 0.001117008 | - |
| *SDR2a* | 3.265826698 | 495.0605611 | 151.5881297 | 7.244012976 | 0.000142274 | 0.001120165 | - |
| *EO* | 0 | 50.04672186 | Inf | Inf | 0.000143413 | 0.001128118 | K18980 |
| *DDR4* | 88.56913276 | 181.3773798 | 2.047862208 | 1.034118646 | 0.00014511 | 0.001140766 | - |
| *BZIP53* | 2.191035996 | 20.93190591 | 9.553428584 | 3.256018588 | 0.000147077 | 0.001154111 | - |
| *W09H1.5* | 403.4662306 | 1104.564535 | 2.737687695 | 1.452957879 | 0.000148969 | 0.001167531 | - |
| *WRKY6* | 224.9883446 | 1074.087589 | 4.773969916 | 2.255189475 | 0.000150597 | 0.001179208 | - |
| *uncharacterized protein_13939* | 13.94452775 | 48.93094284 | 3.508970954 | 1.811048006 | 0.000152262 | 0.001189712 | - |
| *CYP750A1* | 20.51925062 | 75.0946836 | 3.659718622 | 1.871732731 | 0.000153352 | 0.001197496 | - |
| *CAR10* | 0 | 11.53440617 | Inf | Inf | 0.000153412 | 0.001197599 | K12486 |
| *RSL1* | 6.614562578 | 32.82133771 | 4.961981586 | 2.310916381 | 0.000154277 | 0.001202892 | K11975 |
| *SCPL50* | 39.6009488 | 97.0677104 | 2.45114608 | 1.293456466 | 0.000157459 | 0.00122473 | K09645 |
| *TUR2* | 7.668608002 | 45.57130901 | 5.942579017 | 2.571089181 | 0.000158751 | 0.001234028 | - |
| *OsI_03083* | 4.071029588 | 64.05110465 | 15.73339207 | 3.97575784 | 0.000158943 | 0.001235147 | - |
| *uncharacterized protein_14219* | 68.44038817 | 146.693228 | 2.143372239 | 1.099882425 | 0.00016106 | 0.001249709 | - |
| *FSD3* | 65.71886946 | 142.0804318 | 2.161942726 | 1.112328304 | 0.000164413 | 0.001274188 | K04564 |
| *HEL* | 1.114522514 | 16.91254276 | 15.17469817 | 3.923595916 | 0.000164736 | 0.001276307 | - |
| *R35* | 8.999161226 | 51.49834311 | 5.72257145 | 2.516663571 | 0.000166978 | 0.00129212 | K00430 |
| *uncharacterized protein_03821* | 68.96225698 | 500.1863611 | 7.253045116 | 2.858586823 | 0.000167156 | 0.001293073 | - |
| *DES5* | 17.31745446 | 59.27142712 | 3.422640853 | 1.775109914 | 0.000167498 | 0.001294975 | - |
| *IRL7* | 42.15309569 | 101.4934848 | 2.407735023 | 1.267676629 | 0.000168924 | 0.001305212 | - |
| *At2g29640* | 22.37167975 | 65.23917218 | 2.916149923 | 1.544064892 | 0.00017015 | 0.001312864 | K15235 |
| *HIPP20* | 2.180663357 | 166.5664297 | 76.38337629 | 6.255186786 | 0.00017017 | 0.001312864 | - |
| *GLO1* | 84.78158111 | 174.1634198 | 2.054260106 | 1.038618864 | 0.000170481 | 0.001314477 | K11517 |
| *RUS6* | 18.31963669 | 57.96622526 | 3.164158014 | 1.661821648 | 0.000170553 | 0.001314637 | - |
| *LECRK42* | 0.362869633 | 13.54821543 | 37.33631638 | 5.222507692 | 0.000172423 | 0.001327854 | - |
| *RAD50* | 31.19106055 | 96.69442712 | 3.10006859 | 1.632300136 | 0.000174723 | 0.00134436 | K10866 |
| *DDB_G0268948* | 3.31420837 | 23.94120761 | 7.223808808 | 2.85275971 | 0.000176894 | 0.001358216 | - |
| *SRC2* | 84.76761905 | 173.8271882 | 2.050631953 | 1.03606858 | 0.000177585 | 0.001363109 | - |
| *TCHQD* | 37.19226012 | 92.08085023 | 2.475806793 | 1.307898734 | 0.000179473 | 0.001376367 | - |
| *uncharacterized protein_41885* | 11.70683286 | 48.01413881 | 4.101377323 | 2.036108476 | 0.000180617 | 0.001384316 | - |
| *nep1* | 1.852357199 | 48.64826258 | 26.26289499 | 4.71495405 | 0.00018169 | 0.001390467 | - |
| *TPR2* | 1304.90564 | 2640.308429 | 2.023371152 | 1.016760982 | 0.000185507 | 0.00141378 | - |
| *PAP23* | 53.46259624 | 120.0076098 | 2.244702245 | 1.166524087 | 0.00018582 | 0.001415745 | - |
| *uncharacterized protein_47964* | 4.103870284 | 38.540234 | 9.391192053 | 3.231308295 | 0.0001861 | 0.001416621 | - |
| *JGB* | 0.364592412 | 20.70275866 | 56.78329545 | 5.827394674 | 0.000193416 | 0.001466662 | - |
| *At1g18900* | 170.9037355 | 380.8876492 | 2.228667782 | 1.156181576 | 0.000195511 | 0.001479927 | - |
| *Znf330* | 37.52745739 | 95.02550525 | 2.532159434 | 1.340368245 | 0.000197135 | 0.001489588 | - |
| *BACOVA_02659* | 53.20679775 | 119.4589951 | 2.245182949 | 1.166833008 | 0.000199619 | 0.001507025 | K05349 |
| *Elac2* | 60.31896637 | 131.5552182 | 2.180992582 | 1.124984863 | 0.000199829 | 0.001508168 | K00784 |
| *AMY2* | 63.09572899 | 136.4754313 | 2.162990008 | 1.113027 | 0.000200447 | 0.001512387 | K01176 |
| *uncharacterized protein_19242* | 92.39839075 | 184.9571837 | 2.001735984 | 1.001251705 | 0.000200901 | 0.001514922 | - |
| *uncharacterized protein_00911* | 26.76584716 | 92.46376476 | 3.454542807 | 1.788494789 | 0.000206377 | 0.001549396 | - |
| *LAMP1* | 142.9458985 | 288.3629741 | 2.017287498 | 1.012416708 | 0.000207194 | 0.001554168 | K01301 |
| *UGT85A8* | 26.97829554 | 99.66797588 | 3.694376307 | 1.885330826 | 0.000207545 | 0.001555894 | - |
| *uncharacterized protein_11342* | 21.26573517 | 62.44722992 | 2.936518744 | 1.554106847 | 0.000212645 | 0.001590874 | - |
| *CCR1* | 32.65122491 | 95.17192463 | 2.914804112 | 1.543398931 | 0.000215389 | 0.00160953 | - |
| *uncharacterized protein_35345* | 55.51710111 | 122.9107346 | 2.213925658 | 1.146606778 | 0.000215816 | 0.00161225 | - |
| *CRK8* | 22.76907689 | 64.95736082 | 2.852876343 | 1.512417215 | 0.000221124 | 0.001649024 | - |
| *TUR2* | 89.75615585 | 331.877217 | 3.697542679 | 1.8865668 | 0.000224655 | 0.001674769 | - |
| *ALDR* | 4.124615562 | 30.64189625 | 7.42903085 | 2.893174017 | 0.000226769 | 0.001688676 | - |
| *ATJ11* | 1.465296729 | 17.73599198 | 12.10402755 | 3.597415271 | 0.00022698 | 0.001689754 | - |
| *uncharacterized protein_45647* | 44.84704992 | 104.287681 | 2.325407829 | 1.217483757 | 0.000228522 | 0.001700744 | - |
| *uncharacterized protein_08456* | 0 | 37.44241871 | Inf | Inf | 0.000230721 | 0.001715614 | - |
| *DMP3* | 0 | 17.34662786 | Inf | Inf | 0.000231845 | 0.001722476 | - |
| *PARP3* | 1.458369649 | 31.19961495 | 21.39348894 | 4.419099877 | 0.000234575 | 0.001740244 | K10798 |
| *At3g51280* | 2.204854193 | 20.13913594 | 9.133998975 | 3.191246628 | 0.0002353 | 0.001745122 | - |
| *CT1* | 87.69304418 | 176.3533614 | 2.011030214 | 1.007934757 | 0.000238512 | 0.001766901 | K00967 |
| *uncharacterized protein_31766* | 53.32258359 | 148.2532709 | 2.780309223 | 1.475245347 | 0.000242218 | 0.001793324 | - |
| *At3g02290* | 104.4299115 | 334.5669353 | 3.203746231 | 1.679759876 | 0.000244665 | 0.001809873 | - |
| *uncharacterized protein_35311* | 13.96347832 | 47.50452668 | 3.40205539 | 1.76640663 | 0.000245105 | 0.001812082 | - |
| *uncharacterized protein_36356* | 52.46200002 | 284.7449981 | 5.427642828 | 2.440325786 | 0.000246195 | 0.001818793 | - |
| *HCT* | 0.749930102 | 14.74314216 | 19.65935507 | 4.29714409 | 0.000246225 | 0.001818793 | K13065 |
| *Def1* | 4.001902639 | 54.70660151 | 13.67014804 | 3.772956961 | 0.000250344 | 0.001845505 | - |
| *FPG1* | 42.74585019 | 99.77283567 | 2.334094075 | 1.22286271 | 0.000257078 | 0.001888643 | K10563 |
| *At2g30020* | 0.739557463 | 17.65911847 | 23.87795316 | 4.577607268 | 0.000258953 | 0.00190079 | K17506 |
| *PCK2* | 31.37769613 | 79.70184946 | 2.540079716 | 1.344873774 | 0.000259591 | 0.001904925 | K01610 |
| *RCD1* | 97.60961181 | 385.4562223 | 3.948957639 | 1.981471892 | 0.000260522 | 0.00191012 | - |
| *CIPK26* | 55.41689221 | 127.1313175 | 2.294089626 | 1.197921756 | 0.000263199 | 0.001927553 | K07198 |
| *At4g21250* | 20.62997602 | 60.387197 | 2.927157886 | 1.549500565 | 0.000264167 | 0.001933536 | - |
| *OPR11* | 471.4772619 | 1256.305949 | 2.664616199 | 1.413927748 | 0.000264246 | 0.001933565 | K05894 |
| *CIPK32* | 495.8846109 | 1201.950582 | 2.423851347 | 1.277301222 | 0.000279586 | 0.0020325 | - |
| *uncharacterized protein_42143* | 14.25725697 | 47.80691761 | 3.353163776 | 1.745522949 | 0.000284651 | 0.002066975 | - |
| *SWEET2A* | 76.23684146 | 178.6122333 | 2.342859829 | 1.228270642 | 0.00028604 | 0.002075893 | K15382 |
| *At1g48650* | 397.5598417 | 956.2143463 | 2.40520859 | 1.266162016 | 0.000286838 | 0.002080507 | K13026 |
| *MKK9* | 16.92518969 | 57.809326 | 3.415579207 | 1.772130249 | 0.000292105 | 0.002115128 | K20604 |
| *Vps13* | 57.16545109 | 146.8738885 | 2.569277172 | 1.361362536 | 0.000297425 | 0.002149409 | K19525 |
| *HMG1* | 26.48068241 | 70.49737297 | 2.662218892 | 1.412629197 | 0.000298855 | 0.002157923 | K00021 |
| *CYP94B1* | 1.093777237 | 16.17825269 | 14.79117699 | 3.886664952 | 0.000304539 | 0.002195263 | - |
| *uncharacterized protein_11339* | 35.10488567 | 86.20538716 | 2.455652127 | 1.2961062 | 0.00030583 | 0.002202712 | - |
| *Os08g0536000* | 4.036466113 | 24.75689932 | 6.133310333 | 2.61666595 | 0.000314745 | 0.002260585 | K00162 |
| *HSP17.7* | 30.83859952 | 98.43343127 | 3.191890449 | 1.674411137 | 0.000328342 | 0.002349701 | K13993 |
| *NPF2.9* | 1.862729837 | 18.35403491 | 9.853299464 | 3.300606905 | 0.000329135 | 0.00235342 | - |
| *HIPP39* | 20.63679521 | 83.31727034 | 4.037316332 | 2.01339663 | 0.000329136 | 0.00235342 | - |
| *LYK3* | 36.78097285 | 88.40040246 | 2.403427523 | 1.265093299 | 0.000334405 | 0.002388433 | - |
| *UTR2* | 49.97729327 | 110.9957267 | 2.220923131 | 1.15115946 | 0.000346929 | 0.002468473 | K15277 |
| *PDC4* | 16.14934598 | 50.71097932 | 3.140125884 | 1.650822396 | 0.000349596 | 0.00248381 | K01568 |
| *HAK5* | 306.0898125 | 1121.776486 | 3.664860573 | 1.873758313 | 0.000355821 | 0.002521067 | K03549 |
| *YY1* | 66.08338995 | 137.7572724 | 2.084597545 | 1.059768882 | 0.000356473 | 0.002524995 | K09201 |
| *LTPG7* | 0.374965051 | 14.76875686 | 39.38702238 | 5.29964845 | 0.000362295 | 0.002561991 | - |
| *HSP18.2* | 0.749930102 | 14.15528063 | 18.87546664 | 4.238440406 | 0.000364704 | 0.002578319 | K13993 |
| *NAC018* | 0 | 26.90709625 | Inf | Inf | 0.000365776 | 0.002585184 | - |
| *CHR12* | 60.36045693 | 127.913773 | 2.119165088 | 1.083495981 | 0.000369001 | 0.002606543 | K11647 |
| *SF3B4* | 187.8426426 | 602.9635592 | 3.209939718 | 1.682546204 | 0.000370102 | 0.002612886 | K12831 |
| *INT2* | 1.102427096 | 195.5981762 | 177.4250441 | 7.471065855 | 0.000373036 | 0.002632154 | K08150 |
| *GRXC1* | 69.94883339 | 357.3396688 | 5.108586541 | 2.352924177 | 0.000374607 | 0.002640906 | K03676 |
| *PA1024* | 75.36247566 | 152.4570309 | 2.022983316 | 1.016484422 | 0.000374688 | 0.002640906 | - |
| *PI4KG6* | 2.947857214 | 37.07265872 | 12.57613786 | 3.652617032 | 0.000377747 | 0.002660281 | - |
| *ayr1* | 70.88884463 | 145.2536096 | 2.04903339 | 1.034943494 | 0.000381677 | 0.002686484 | - |
| *SNRNP59* | 16.60897895 | 50.99369814 | 3.070248826 | 1.618355583 | 0.000393405 | 0.002759208 | - |
| *HGO* | 68.5249482 | 140.2024444 | 2.046005843 | 1.032810265 | 0.000412355 | 0.002877189 | K00451 |
| *CRK8* | 0 | 14.09626431 | Inf | Inf | 0.000423598 | 0.002946834 | - |
| *DLO1* | 65.98331781 | 135.6766305 | 2.056226256 | 1.03999902 | 0.000441542 | 0.003062539 | - |
| *ALMT9* | 65.7188335 | 135.6990544 | 2.064842712 | 1.04603189 | 0.000444876 | 0.003082579 | - |
| *CYSKP* | 141.4596767 | 307.5259389 | 2.1739477 | 1.120317233 | 0.000452083 | 0.003128896 | K01738 |
| *ATJ8* | 28.02379191 | 71.44664175 | 2.549499439 | 1.350214021 | 0.000453263 | 0.003136216 | - |
| *ACT2* | 157.7490206 | 469.5040936 | 2.976272637 | 1.573506688 | 0.000453703 | 0.003138416 | K10355 |
| *At5g15710* | 69.3802986 | 141.3602809 | 2.037470056 | 1.026778857 | 0.000455781 | 0.00315025 | - |
| *uncharacterized protein_11987* | 19.4808181 | 56.05890509 | 2.877646349 | 1.524889302 | 0.00046055 | 0.003178936 | - |
| *ACO1* | 10.28815906 | 38.13267106 | 3.706462044 | 1.890042738 | 0.000470618 | 0.003240592 | K14677 |
| *ROQ1* | 0 | 10.09976407 | Inf | Inf | 0.0004772 | 0.003278882 | - |
| *NFXL2* | 57.38492736 | 127.5963355 | 2.223516546 | 1.152843141 | 0.000482781 | 0.003316343 | K15683 |
| *uncharacterized protein_37019* | 0 | 10.03571262 | Inf | Inf | 0.000490641 | 0.003363142 | - |
| *RUS6* | 13.57473103 | 44.69613254 | 3.292598021 | 1.71922639 | 0.00049731 | 0.003404312 | - |
| *UBA2C* | 39.21905667 | 90.65984026 | 2.311627254 | 1.208908784 | 0.000516609 | 0.003526089 | K12741 |
| *At4g01130* | 164.088741 | 365.2899395 | 2.22617309 | 1.15456577 | 0.00052156 | 0.003557046 | - |
| *S-RBP11* | 52.28066955 | 125.9081897 | 2.408312494 | 1.268022603 | 0.000523147 | 0.003565979 | K13195 |
| *ATL11* | 9.247931831 | 35.46416762 | 3.834821479 | 1.939159418 | 0.000531221 | 0.003614298 | K10664 |
| *GDPDL4* | 62.24895653 | 148.7597014 | 2.389754137 | 1.256862199 | 0.000531421 | 0.003614704 | - |
| *4-Oct* | 74.79577154 | 224.8220472 | 3.005812261 | 1.587754903 | 0.000536865 | 0.003647872 | K08202 |
| *AGLU* | 16.16320014 | 49.27955744 | 3.048873801 | 1.608276436 | 0.000543521 | 0.003688218 | K01187 |
| *SRC2* | 30.42379477 | 147.2183075 | 4.83891995 | 2.274685073 | 0.000549005 | 0.003722485 | - |
| *At5g46170* | 1.84198456 | 17.399731 | 9.446187214 | 3.239732129 | 0.000560976 | 0.003794637 | - |
| *CDC20-1* | 8.51185589 | 35.35254601 | 4.153329951 | 2.054268488 | 0.000571255 | 0.003854023 | K03363 |
| *At1g07160* | 4.746664403 | 25.34338496 | 5.339198816 | 2.416623272 | 0.000573264 | 0.003865543 | K17506 |
| *MNAT1* | 66.15262479 | 134.475381 | 2.032804918 | 1.023471771 | 0.000581331 | 0.003917882 | K10842 |
| *LARP6B* | 22.48918837 | 60.64702349 | 2.696719085 | 1.431205245 | 0.00058685 | 0.00394731 | K15191 |
| *BGLU12* | 3.352217403 | 21.53900052 | 6.425299417 | 2.683763685 | 0.000587261 | 0.003948528 | K01188 |
| *EDR2L* | 8.439247418 | 33.57613936 | 3.978570327 | 1.992250101 | 0.000589138 | 0.00395804 | - |
| *NRAMP6* | 65.10547048 | 132.2638256 | 2.031531676 | 1.02256786 | 0.000607702 | 0.004072113 | K03322 |
| *CRK2* | 0.364592412 | 11.78097159 | 32.31271742 | 5.014030178 | 0.000608386 | 0.00407563 | - |
| *EXPA8* | 0 | 9.769914121 | Inf | Inf | 0.000610493 | 0.004087615 | - |
| *Dctpp1* | 21.68563633 | 59.04212366 | 2.722637361 | 1.445004837 | 0.0006186 | 0.00413758 | K16904 |
| *LTPG14* | 20.37072488 | 60.48139129 | 2.96903481 | 1.569994008 | 0.000625752 | 0.004177631 | - |
| *pgk* | 29.71714993 | 73.35030268 | 2.468281879 | 1.303507161 | 0.00063676 | 0.004244679 | K00927 |
| *JOX2* | 77.24271391 | 309.8923453 | 4.011929794 | 2.00429636 | 0.00064019 | 0.004264226 | K05278 |
| *GLYI4* | 56.60736086 | 118.4330181 | 2.092184061 | 1.065009779 | 0.000641285 | 0.004269304 | - |
| *D14* | 2.216949611 | 20.58100796 | 9.283480264 | 3.214665756 | 0.000705215 | 0.004659899 | - |
| *uncharacterized protein_14386* | 15.04175054 | 46.31141486 | 3.078858058 | 1.622395358 | 0.000707067 | 0.004669735 | - |
| *DLO2* | 2.552146885 | 19.18480261 | 7.517123219 | 2.910180652 | 0.000709012 | 0.004680667 | - |
| *NPF5.10* | 56.70240143 | 117.436023 | 2.071094346 | 1.050393275 | 0.000713668 | 0.004708489 | K14638 |
| *MHF1* | 59.56393986 | 122.2450921 | 2.052333885 | 1.037265456 | 0.0007198 | 0.004741635 | K11511 |
| *SDR2a* | 0.725739266 | 44.26964874 | 60.99938477 | 5.930722787 | 0.000734542 | 0.00483008 | - |
| *GTE4* | 23.91812692 | 62.39459522 | 2.608673975 | 1.383316651 | 0.000734966 | 0.004831632 | - |
| *GIS2* | 0.362869633 | 17.92307264 | 49.39259449 | 5.626222847 | 0.000735897 | 0.004834481 | - |
| *SD25* | 8.745121484 | 59.88624068 | 6.847959836 | 2.77567424 | 0.000756888 | 0.004955459 | - |
| *RXW8* | 6.581721882 | 29.00506809 | 4.406911839 | 2.139768036 | 0.00076952 | 0.005026632 | - |
| *At5g37930* | 35.19131234 | 81.90743287 | 2.327490151 | 1.218775063 | 0.000773783 | 0.005049341 | K04506 |
| *RAMDAZC7* | 0 | 9.457921359 | Inf | Inf | 0.000775222 | 0.005057447 | - |
| *gluA* | 20.25834863 | 68.06767745 | 3.35998154 | 1.748453307 | 0.000776081 | 0.005061764 | K05349 |
| *PYL4* | 0 | 9.434121575 | Inf | Inf | 0.000783299 | 0.005102369 | K14496 |
| *uncharacterized protein_26439* | 42.10309913 | 112.5726899 | 2.673738804 | 1.418858536 | 0.000793653 | 0.005159346 | - |
| *ACO* | 0.725739266 | 12.99925908 | 17.91174832 | 4.162834257 | 0.000798736 | 0.005189763 | K05933 |
| *pgk* | 25.69446605 | 181.4462718 | 7.061686802 | 2.820012837 | 0.000802051 | 0.005209988 | K00927 |
| *PHOS34* | 1.090331678 | 103.3779329 | 94.81328934 | 6.567017381 | 0.00080682 | 0.005239639 | - |
| *ACO* | 0 | 9.693040611 | Inf | Inf | 0.000807189 | 0.005240713 | K05933 |
| *3MMP* | 39.28484595 | 118.4408345 | 3.014924244 | 1.592121752 | 0.000815328 | 0.005284207 | - |
| *LHT1* | 41.33055711 | 127.5550293 | 3.086216064 | 1.625839068 | 0.000824783 | 0.005335796 | - |
| *uncharacterized protein_10096* | 21.31932114 | 57.3435568 | 2.689745908 | 1.427469892 | 0.000825157 | 0.005335796 | - |
| *APCB1* | 5.5242309 | 26.24508358 | 4.750902715 | 2.248201665 | 0.000830556 | 0.005362605 | - |
| *ZIP5* | 3.298631429 | 23.74956025 | 7.199822338 | 2.847961307 | 0.000842192 | 0.005426825 | K14709 |
| *uncharacterized protein_03044* | 8.036645973 | 86.06146617 | 10.70862975 | 3.420701983 | 0.000842693 | 0.005428693 | - |
| *Ncoa7* | 13.2394978 | 42.29527193 | 3.194628119 | 1.675647999 | 0.000845608 | 0.005442013 | - |
| *uncharacterized protein_00451* | 20.12361222 | 55.23823707 | 2.744946407 | 1.456777982 | 0.000852219 | 0.005482451 | - |
| *LOC_Os07g01090* | 7.393887818 | 37.38975459 | 5.056846346 | 2.338237942 | 0.000853225 | 0.005486908 | - |
| *HSL1* | 12.50338589 | 40.80386745 | 3.263425428 | 1.706387073 | 0.000861499 | 0.005533192 | - |
| *At4g33300* | 5.470644926 | 341.7460191 | 62.46905506 | 5.965069802 | 0.000869904 | 0.005576027 | - |
| *TOP3A* | 28.67695867 | 90.69365149 | 3.162596583 | 1.661109539 | 0.000888041 | 0.005671054 | K03165 |
| *ABCG36* | 0.374965051 | 11.25809838 | 30.02439387 | 4.908063216 | 0.000899568 | 0.005730427 | - |
| *Os04g0671100* | 8.39955157 | 32.58437413 | 3.879299253 | 1.955796071 | 0.000904257 | 0.00575601 | K00939 |
| *WRKY4* | 59.00747161 | 120.1447781 | 2.036094325 | 1.025804398 | 0.000914556 | 0.005811492 | - |
| *TIFY10B* | 0.364592412 | 11.19814519 | 30.71414768 | 4.940831444 | 0.00092333 | 0.005857103 | K13464 |
| *CYP71AU50* | 1.489487565 | 15.37081634 | 10.31953317 | 3.367305803 | 0.000929333 | 0.005890817 | - |
| *CXE15* | 18.06738457 | 58.08381887 | 3.214843779 | 1.684748633 | 0.000936037 | 0.005926007 | - |
| *Parp6* | 107.3466797 | 275.5567474 | 2.566979699 | 1.360071886 | 0.000943023 | 0.005965819 | K10582 |
| *MYB106* | 60.03215077 | 121.0858503 | 2.017016695 | 1.012223025 | 0.00094726 | 0.005991151 | K09422 |
| *Patl_0974* | 61.51295961 | 124.0063345 | 2.015938354 | 1.011451523 | 0.000957781 | 0.006051736 | - |
| *DIM1A* | 3.303835731 | 41.72701453 | 12.62986962 | 3.658767841 | 0.000967914 | 0.006109544 | K14191 |
| *PM19L* | 35.8340705 | 82.60778491 | 2.305286108 | 1.204945814 | 0.000968119 | 0.006109544 | - |
| *HSP21.7* | 0 | 9.178861782 | Inf | Inf | 0.000970655 | 0.00612404 | K13993 |
| *BANGLUC* | 0 | 9.165571291 | Inf | Inf | 0.000976284 | 0.006149471 | - |
| *RUN1* | 118.3429688 | 279.1060686 | 2.358450793 | 1.237839501 | 0.000976361 | 0.006149471 | - |
| *uncharacterized protein_06871* | 2.923666378 | 19.52656716 | 6.678794581 | 2.739587742 | 0.00101814 | 0.006389115 | - |
| *Dctpp1* | 5.923386787 | 26.56483385 | 4.484737332 | 2.16502349 | 0.001037982 | 0.006490268 | K16904 |
| *DLO1* | 12.50686741 | 40.27045554 | 3.219867471 | 1.687001308 | 0.00103878 | 0.00649168 | - |
| *FLZ8* | 0.725739266 | 41.65745951 | 57.40003532 | 5.842979719 | 0.001046372 | 0.00653222 | - |
| *CAD* | 96.57290207 | 343.8712142 | 3.560742267 | 1.832178015 | 0.001046538 | 0.00653222 | K00083 |
| *CYP59* | 37.43065808 | 83.93491281 | 2.242410823 | 1.165050613 | 0.001079533 | 0.006716951 | K12735 |
| *At4g01130* | 1763.101107 | 3614.203601 | 2.049912842 | 1.035562571 | 0.001084939 | 0.00674242 | - |
| *Rfwd3* | 54.04835882 | 111.0191168 | 2.054070082 | 1.038485405 | 0.001086318 | 0.006749359 | K15691 |
| *GLOX1* | 0.749930102 | 13.00567011 | 17.34250975 | 4.116240791 | 0.00112744 | 0.006977854 | - |
| *CYP734A1* | 14.34540641 | 43.43255824 | 3.027628287 | 1.598188091 | 0.00113236 | 0.007003241 | K15639 |
| *uncharacterized protein_15757* | 39.99490039 | 126.9839521 | 3.175003586 | 1.666758221 | 0.001135248 | 0.007017726 | - |
| *uncharacterized protein_04846* | 35.19648068 | 92.16507164 | 2.618587707 | 1.388788927 | 0.001154233 | 0.007123087 | - |
| *Os01g0270100* | 40.61350367 | 89.12965739 | 2.194581835 | 1.133946068 | 0.001172961 | 0.007223042 | - |
| *PIP2-8* | 34.49145073 | 78.46116748 | 2.274800446 | 1.185739992 | 0.001186951 | 0.007303941 | K09872 |
| *DDB_G0290631* | 11.00180291 | 36.94872205 | 3.358424284 | 1.747784504 | 0.001196541 | 0.007348874 | K13348 |
| *GATA26* | 51.49441723 | 105.9995677 | 2.058467178 | 1.041570446 | 0.001209821 | 0.007423337 | - |
| *LIP1* | 10.27606364 | 35.41018644 | 3.445890146 | 1.78487671 | 0.001215139 | 0.007445297 | K01052 |
| *ERF113* | 16.55715172 | 47.21908549 | 2.851884568 | 1.511915589 | 0.001222262 | 0.007481803 | - |
| *FDM1* | 12.17856125 | 41.90412231 | 3.440810572 | 1.782748469 | 0.001230818 | 0.007527006 | - |
| *uncharacterized protein_15372* | 20.29815237 | 53.64343692 | 2.642774374 | 1.40205326 | 0.001261096 | 0.007690203 | - |
| *CRJ31* | 0.729184824 | 70.54920676 | 96.75078855 | 6.596201515 | 0.001261859 | 0.007693031 | - |
| *ELI* | 20.32234321 | 279.4279078 | 13.74978785 | 3.781337454 | 0.001268037 | 0.007727024 | - |
| *At5g22090* | 45.48115822 | 96.30790302 | 2.117534091 | 1.082385196 | 0.001269697 | 0.007735306 | - |
| *2 SV* | 28.3762889 | 67.55124468 | 2.380552472 | 1.251296429 | 0.001293907 | 0.007858278 | K05658 |
| *DJ1B* | 47.3023975 | 134.1574751 | 2.836166498 | 1.503942229 | 0.001319508 | 0.007993273 | K03152 |
| *RBOHC* | 43.23484943 | 92.54289221 | 2.140469862 | 1.097927523 | 0.001330476 | 0.00805101 | K13447 |
| *SDR2a* | 1.100704317 | 161.4035139 | 146.6365775 | 7.196101209 | 0.001336742 | 0.00808433 | - |
| *SYT2* | 80.48582789 | 317.0172342 | 3.938795717 | 1.977754595 | 0.001339842 | 0.008097359 | - |
| *RABB1C* | 13.29136099 | 40.872076 | 3.075085842 | 1.620626684 | 0.001340508 | 0.008099483 | K07877 |
| *At1g19450* | 37.07123401 | 82.08622877 | 2.214283688 | 1.146840068 | 0.001366786 | 0.008236947 | K08145 |
| *LTI6A* | 0.749930102 | 44.33694067 | 59.12143084 | 5.88560928 | 0.001368389 | 0.00824467 | - |
| *IQD17* | 20.65754048 | 54.09044147 | 2.618435699 | 1.388705177 | 0.001371114 | 0.00825528 | - |
| *SPX2* | 5.474126449 | 35.29634946 | 6.447850591 | 2.688818314 | 0.001375755 | 0.008281285 | - |
| *LNK1* | 339.1725806 | 712.4939939 | 2.100682763 | 1.070858309 | 0.001377085 | 0.008287351 | - |
| *uncharacterized protein_48210* | 20.60571325 | 54.07530665 | 2.62428706 | 1.391925539 | 0.001377534 | 0.008288108 | - |
| *OFUT20* | 2.909848181 | 18.92863536 | 6.505025068 | 2.701554617 | 0.001381634 | 0.008306943 | - |
| *COR413IM2* | 19.09716723 | 59.28959653 | 3.104627814 | 1.634420327 | 0.001383865 | 0.008318406 | - |
| *SERK4* | 32.29348765 | 74.12749881 | 2.29543181 | 1.198765575 | 0.00138495 | 0.008322982 | - |
| *FAO4A* | 20.56770422 | 54.0098793 | 2.62595566 | 1.392842556 | 0.001387519 | 0.008336472 | K17756 |
| *MYB5* | 7.748107591 | 29.79555471 | 3.845526712 | 1.943181214 | 0.001394124 | 0.008374195 | K09422 |
| *dnajb6-b* | 0.739557463 | 13.04410687 | 17.6377192 | 4.140592107 | 0.001400774 | 0.008409464 | - |
| *GDU2* | 5.144097511 | 24.2413446 | 4.71245822 | 2.236479828 | 0.001401705 | 0.008411868 | - |
| *LTI6B* | 1.477392147 | 82.53271716 | 55.86378491 | 5.803841418 | 0.001408458 | 0.008444502 | - |
| *uncharacterized protein_39668* | 0 | 9.140425027 | Inf | Inf | 0.001420687 | 0.008511866 | - |
| *COI1B* | 87.79831352 | 200.4288001 | 2.282832005 | 1.190824695 | 0.00144949 | 0.008660195 | K13463 |
| *PCO1* | 57.72881755 | 115.6052935 | 2.002557794 | 1.00184388 | 0.001455238 | 0.008686776 | K10712 |
| *uncharacterized protein_20061* | 16.65391507 | 48.0621774 | 2.885938664 | 1.529040638 | 0.001457566 | 0.008696313 | - |
| *BACOVA_02659* | 42.85119856 | 91.45351769 | 2.134211429 | 1.093703106 | 0.00147416 | 0.008774943 | K05349 |
| *At1g15890* | 13.27750683 | 40.45115459 | 3.04659264 | 1.60719661 | 0.001490897 | 0.008860199 | K13459 |
| *CNGC20* | 256.8342446 | 573.721847 | 2.233821459 | 1.159513881 | 0.001497835 | 0.008897316 | K05391 |
| *CHI4* | 0 | 19.35915861 | Inf | Inf | 0.001512123 | 0.00897388 | K01183 |
| *ctf8* | 21.40054351 | 54.90610377 | 2.565640623 | 1.359319102 | 0.001529362 | 0.009060582 | K11270 |
| *TERT* | 19.07821666 | 51.03444765 | 2.675011432 | 1.419545057 | 0.001531868 | 0.009070111 | K11126 |
| *uncharacterized protein_15644* | 5.100884177 | 34.10955128 | 6.686987999 | 2.741356528 | 0.001532657 | 0.009072693 | - |
| *MYB2* | 0 | 11.23611351 | Inf | Inf | 0.00156695 | 0.009252334 | K09422 |
| *CDC48B* | 25.78613298 | 62.33464203 | 2.417370688 | 1.273438718 | 0.001606152 | 0.009459798 | - |
| *uncharacterized protein_25754* | 1.454924091 | 23.02033473 | 15.82236137 | 3.983893023 | 0.001609225 | 0.009471385 | - |
| *L6* | 0 | 30.07924896 | Inf | Inf | 0.001616296 | 0.009508647 | - |
| *NPF7.3* | 2.92022082 | 18.58277254 | 6.363481973 | 2.669816396 | 0.001618124 | 0.009517224 | K14638 |
| *HMG1* | 38.95646787 | 84.4185392 | 2.166996749 | 1.115696989 | 0.001620416 | 0.009527202 | K00021 |
| *IDD14* | 4.784673436 | 91.63953489 | 19.15272507 | 4.25947777 | 0.00164974 | 0.009685434 | - |
| *CAX1a* | 4.385517549 | 32.70324625 | 7.457100759 | 2.898614835 | 0.001685453 | 0.009847842 | K07300 |
| *GOLS4* | 0.362869633 | 10.37744776 | 28.59828107 | 4.83785653 | 0.00169149 | 0.009876382 | K18819 |
| *XTH5* | 0 | 13.59490754 | Inf | Inf | 0.001705268 | 0.009948937 | K08235 |
| *UGT92A1* | 32.50783154 | 78.9772292 | 2.429483157 | 1.280649431 | 0.001709161 | 0.009968228 | - |
| *At3g01520* | 1.477392147 | 14.70698875 | 9.95469536 | 3.315377166 | 0.001748339 | 0.010175934 | - |
| *HEL* | 0 | 17.75425878 | Inf | Inf | 0.001760464 | 0.0102332 | - |
| *VCR* | 27.97533831 | 101.3316092 | 3.622176365 | 1.856856793 | 0.001769182 | 0.010276294 | K12616 |
| *NRP* | 248.0061491 | 551.6426453 | 2.224310354 | 1.153358099 | 0.001774458 | 0.01030461 | - |
| *OFUT23* | 22.02090553 | 58.89753946 | 2.674619323 | 1.419333568 | 0.001776128 | 0.010307821 | - |
| *Os08g0191100* | 22.10036916 | 55.52643005 | 2.512466179 | 1.329104176 | 0.001821861 | 0.010544137 | K01681 |
| *SIS8* | 112.4855729 | 412.6203841 | 3.668207163 | 1.875075118 | 0.001825837 | 0.01056477 | - |
| *SD25* | 1.468742288 | 14.23537436 | 9.692220668 | 3.276827252 | 0.001853894 | 0.010705415 | - |
| *ABCI17* | 0 | 8.821552802 | Inf | Inf | 0.001878002 | 0.010832454 | - |
| *CYP750A1* | 4.421803803 | 21.82215837 | 4.935125875 | 2.303086883 | 0.001902273 | 0.010946739 | - |
| *FLS2* | 0.362869633 | 11.26860767 | 31.05414906 | 4.956714131 | 0.001912245 | 0.010997872 | K13420 |
| *TCEA1* | 21.69263534 | 61.68267022 | 2.843484402 | 1.50765989 | 0.001995167 | 0.01142363 | - |
| *RFS6* | 69.38352837 | 326.7891467 | 4.709895192 | 2.235694956 | 0.00203199 | 0.011616347 | K06617 |
| *ABCC2* | 12.88696484 | 38.76125269 | 3.007787573 | 1.588702679 | 0.002087861 | 0.011890765 | - |
| *BSP* | 0 | 16.92761875 | Inf | Inf | 0.002091445 | 0.011908534 | - |
| *CRK41* | 0.374965051 | 10.04027932 | 26.77657369 | 4.742899461 | 0.002107917 | 0.01198904 | - |
| *CNX3* | 49.04417315 | 99.24855716 | 2.023656446 | 1.016964386 | 0.002118992 | 0.012036751 | K03639 |
| *SETH3* | 4.784673436 | 22.43291222 | 4.688493901 | 2.229124556 | 0.002122677 | 0.01205431 | K06041 |
| *PAS1* | 33.98515176 | 74.55026455 | 2.193612818 | 1.133308907 | 0.002126214 | 0.012069061 | - |
| *BHLH148* | 1.826443583 | 15.07621233 | 8.254408989 | 3.045164923 | 0.002127076 | 0.012071287 | - |
| *EOBII* | 0.362869633 | 10.02517391 | 27.62748106 | 4.788032125 | 0.00212905 | 0.01207982 | K09422 |
| *At3g21360* | 4.741460102 | 22.43838638 | 4.732379035 | 2.242565629 | 0.002135837 | 0.012112978 | - |
| *uncharacterized protein_12319* | 5.875005115 | 24.8378711 | 4.227719059 | 2.07987951 | 0.002176857 | 0.012321136 | - |
| *GOLS2* | 0 | 8.851734201 | Inf | Inf | 0.002180163 | 0.012337128 | K18819 |
| *R17* | 4.045115973 | 20.67345531 | 5.11072005 | 2.353526567 | 0.00219524 | 0.012416976 | K00430 |
| *CYP76T24* | 13.64209924 | 39.91408343 | 2.92580216 | 1.548832219 | 0.002205072 | 0.012458589 | - |
| *GT4* | 23.46208335 | 60.31120153 | 2.570581675 | 1.362094852 | 0.002244875 | 0.012658708 | - |
| *uncharacterized protein_19196* | 28.26222584 | 108.5851577 | 3.842059656 | 1.94187992 | 0.002252463 | 0.012695925 | - |
| *uncharacterized protein_38961* | 42.69915533 | 88.41916711 | 2.070747452 | 1.050151614 | 0.002265553 | 0.012766906 | - |
| *BANGLUC* | 54.58759219 | 1908.278337 | 34.9580969 | 5.127554742 | 0.002278265 | 0.012821664 | - |
| *HISN4* | 37.58965726 | 80.03901789 | 2.129282992 | 1.090367704 | 0.002292543 | 0.012893549 | K01663 |
| *SINAT3* | 38.16332443 | 81.16766865 | 2.126850055 | 1.088718325 | 0.002312873 | 0.012990821 | K04506 |
| *uncharacterized protein_04789* | 19.85061481 | 50.79107305 | 2.558664984 | 1.355391262 | 0.002319605 | 0.013022941 | - |
| *At3g21360* | 2.194481554 | 56.83720346 | 25.90005979 | 4.694883523 | 0.002379787 | 0.013322971 | - |
| *SIS8* | 33.09700373 | 121.5201211 | 3.671635115 | 1.876422691 | 0.002388719 | 0.013370061 | - |
| *UVR8* | 44.83667728 | 91.44394527 | 2.039489784 | 1.028208281 | 0.002393609 | 0.013391908 | - |
| *ERF9* | 16.90788997 | 56.66562866 | 3.351431122 | 1.744777284 | 0.002393665 | 0.013391908 | K09286 |
| *DCR* | 34.2444029 | 74.58410519 | 2.177994033 | 1.123000002 | 0.002410258 | 0.013470077 | K13065 |
| *BEBT* | 0 | 8.01777569 | Inf | Inf | 0.002438892 | 0.013609377 | K19861 |
| *CKX7* | 0 | 7.97980737 | Inf | Inf | 0.002479598 | 0.013793844 | K00279 |
| *WAKL20* | 0 | 7.979338935 | Inf | Inf | 0.002480106 | 0.013793844 | - |
| *ROQ1* | 0.364592412 | 12.63003551 | 34.64152046 | 5.114430348 | 0.002485463 | 0.013815238 | - |
| *WRKY6* | 1.102427096 | 15.37175321 | 13.94355532 | 3.801526561 | 0.002501305 | 0.013885269 | - |
| *At3g15810* | 0 | 7.949128123 | Inf | Inf | 0.0025131 | 0.013935693 | - |
| *CAT1* | 16.13376904 | 373.6393045 | 23.15883559 | 4.533490812 | 0.002517953 | 0.01395959 | K03294 |
| *uncharacterized protein_24770* | 14.38341545 | 49.76127152 | 3.459628327 | 1.790617055 | 0.002558782 | 0.014146257 | K16056 |
| *CIPK2* | 0.729184824 | 11.22788757 | 15.39786237 | 3.944658176 | 0.002559951 | 0.014149676 | K07198 |
| *VCL* | 165.0859418 | 558.2022327 | 3.381282662 | 1.757570625 | 0.002587225 | 0.014281994 | - |
| *FRL3* | 14.31773406 | 43.88916462 | 3.065370851 | 1.616061623 | 0.002606619 | 0.014385963 | - |
| *At3g27390* | 1.45664687 | 13.63012407 | 9.357191749 | 3.226075618 | 0.00263762 | 0.014541435 | - |
| *PNC1* | 15.09871015 | 41.95169247 | 2.77849512 | 1.474303707 | 0.002642185 | 0.014560357 | K00430 |
| *CIPK5* | 0.364592412 | 43.12479055 | 118.2821943 | 6.886089103 | 0.002660545 | 0.014658387 | K07198 |
| *Os07g0638300* | 0 | 19.0626423 | Inf | Inf | 0.002662419 | 0.014662426 | K11188 |
| *SbtS* | 67.16682342 | 148.8807301 | 2.216581379 | 1.14833633 | 0.002666284 | 0.014674272 | - |
| *MAP65-1* | 2.546978548 | 17.09505672 | 6.711896625 | 2.746720496 | 0.002678311 | 0.014734152 | K16732 |
| *NAC047* | 1.104149876 | 12.42512707 | 11.25311639 | 3.492252686 | 0.002685321 | 0.014769553 | - |
| *SKI2* | 25.319501 | 59.34044573 | 2.343665688 | 1.228766792 | 0.002804188 | 0.015364129 | K12599 |
| *CNR9* | 43.98815316 | 89.40680466 | 2.032520082 | 1.023269607 | 0.0028197 | 0.015442529 | - |
| *HIPP23* | 0.374965051 | 11.75579591 | 31.35171099 | 4.970472274 | 0.002828389 | 0.015483514 | - |
| *YUC8* | 16.56059728 | 44.22166897 | 2.670294327 | 1.416998768 | 0.002851112 | 0.015591297 | K11816 |
| *uncharacterized protein_06768* | 3.716809815 | 19.24109656 | 5.176777267 | 2.372054247 | 0.002872729 | 0.015676142 | - |
| *KING1* | 7.342024623 | 27.18246717 | 3.702312178 | 1.888426548 | 0.002882884 | 0.01571487 | - |
| *NFD4* | 25.35061891 | 75.00277266 | 2.958617023 | 1.564922959 | 0.002916657 | 0.015872026 | - |
| *At2g27310* | 40.53231017 | 114.0760475 | 2.814447216 | 1.492851591 | 0.002958898 | 0.016078058 | - |
| *PAD4* | 64.07737464 | 236.3183473 | 3.688015445 | 1.882844698 | 0.003001625 | 0.016286116 | - |
| *AAE13* | 50.60454641 | 110.2056993 | 2.177782574 | 1.122859925 | 0.003003158 | 0.01628755 | K18660 |
| *uncharacterized protein_02415* | 23.09225068 | 69.09350751 | 2.992064675 | 1.58114136 | 0.003037733 | 0.016447289 | - |
| *NAGS2* | 80.93516725 | 188.9499567 | 2.334584126 | 1.223165576 | 0.003048986 | 0.016497786 | K14682 |
| *AOX1A* | 43.92767607 | 89.10770194 | 2.028509357 | 1.020419957 | 0.00310273 | 0.016746266 | K17893 |
| *ROQ1* | 19.46003686 | 49.06408088 | 2.52127379 | 1.334152792 | 0.00312269 | 0.016836311 | - |
| *uncharacterized protein_37760* | 4.807141493 | 21.52936928 | 4.478621922 | 2.163054881 | 0.003124995 | 0.016841669 | - |
| *ASNS* | 0 | 7.674196223 | Inf | Inf | 0.003144844 | 0.016927339 | K01953 |
| *CEPR1* | 8.930034276 | 35.33056114 | 3.95637464 | 1.984179045 | 0.00314771 | 0.016935669 | - |
| *TOR* | 51.69135351 | 108.0582928 | 2.090451989 | 1.06381491 | 0.003159717 | 0.016989595 | K07203 |
| *SAT5* | 40.51684112 | 83.05519906 | 2.049893248 | 1.035548781 | 0.003168956 | 0.017032141 | K00640 |
| *PNC1* | 0 | 7.654524117 | Inf | Inf | 0.003172056 | 0.017045237 | K00430 |
| *At5g48740* | 1.453201311 | 17.62249663 | 12.12667267 | 3.600111851 | 0.003174204 | 0.017049648 | - |
| *HTR4* | 0 | 7.651772332 | Inf | Inf | 0.003175885 | 0.017055114 | K11253 |
| *ZIP5* | 0.362869633 | 9.451041896 | 26.04528193 | 4.702950149 | 0.003212684 | 0.017234719 | K14709 |
| *NCL2* | 96.71987067 | 220.4576701 | 2.279342068 | 1.188617451 | 0.003237432 | 0.017352987 | - |
| *ELIP1* | 1.092054457 | 120.4013846 | 110.25218 | 6.784663372 | 0.003239592 | 0.017360942 | - |
| *uncharacterized protein_21626* | 1.104149876 | 12.07376068 | 10.93489294 | 3.45086719 | 0.003270299 | 0.017492659 | - |
| *NIP2-1* | 111.4385264 | 294.8053481 | 2.645452677 | 1.40351461 | 0.003290355 | 0.017592615 | K09874 |
| *DDB_G0268948* | 11.37849074 | 34.61056642 | 3.04175371 | 1.604903343 | 0.003296818 | 0.017619834 | - |
| *Mge2* | 3.642478564 | 18.96478877 | 5.20656153 | 2.380330916 | 0.003332514 | 0.017784716 | K10866 |
| *ABCC1* | 15.06938694 | 41.14012843 | 2.730046591 | 1.448925572 | 0.003408273 | 0.018136277 | - |
| *uncharacterized protein_00637* | 17.40212239 | 44.86535601 | 2.578154262 | 1.366338589 | 0.003410251 | 0.018139287 | K06100 |
| *OsI_35105* | 6.619730916 | 33.37948628 | 5.042423431 | 2.334117272 | 0.00343115 | 0.018239121 | K00873 |
| *ZW10* | 20.62642257 | 50.51845391 | 2.449210654 | 1.292316864 | 0.003445324 | 0.018306893 | K11578 |
| *BAM3* | 11.3438913 | 73.81009071 | 6.506593617 | 2.70190245 | 0.003454983 | 0.018346832 | K01177 |
| *uncharacterized protein_42058* | 15.41840241 | 41.67310132 | 2.702815779 | 1.434463183 | 0.003483912 | 0.01846545 | - |
| *NCER1* | 28.65449061 | 63.79080057 | 2.226206057 | 1.154587135 | 0.003494652 | 0.018511568 | K12349 |
| *PAD4* | 2.975529573 | 19.11158835 | 6.422919981 | 2.683229323 | 0.003505223 | 0.018562676 | - |
| *NFYA7* | 40.68790685 | 83.13435592 | 2.043220268 | 1.030844741 | 0.003538328 | 0.018719764 | K08064 |
| *LPL1* | 18.75852439 | 47.28632775 | 2.520791442 | 1.333876761 | 0.00357221 | 0.018875701 | - |
| *HSP12* | 4.798491634 | 21.18901003 | 4.415764712 | 2.142663302 | 0.003582327 | 0.018921376 | K13993 |
| *BANGLUC* | 0.374965051 | 47.27688213 | 126.0834363 | 6.97823495 | 0.003587998 | 0.018935754 | - |
| *AMC1* | 5.869800813 | 24.13332341 | 4.11143822 | 2.039643151 | 0.003593298 | 0.018954058 | - |
| *CDCA7L* | 14.25032988 | 39.69544548 | 2.785580811 | 1.47797817 | 0.003606328 | 0.019016865 | - |
| *uncharacterized protein_27926* | 20.86489246 | 53.87546276 | 2.582110732 | 1.368550871 | 0.003638384 | 0.019166801 | - |
| *aifm2* | 60.66811861 | 212.7592167 | 3.506936124 | 1.810211153 | 0.003680924 | 0.0193665 | - |
| *GIS2* | 1.465296729 | 13.03494405 | 8.895770933 | 3.153119639 | 0.003713149 | 0.019504093 | - |
| *GOLS1* | 147.4506617 | 712.5403538 | 4.832398483 | 2.272739425 | 0.003715916 | 0.019514636 | K18819 |
| *PTI1* | 0.364592412 | 13.03034794 | 35.73949294 | 5.159447261 | 0.003868247 | 0.020227793 | K13436 |
| *UVR3* | 52.26519341 | 131.1256998 | 2.508853239 | 1.32702808 | 0.003938603 | 0.020508035 | K02295 |
| *FEY* | 0.364592412 | 12.32673712 | 33.80963703 | 5.079362623 | 0.003948887 | 0.020544927 | K11153 |
| *EXLA1* | 19.88341955 | 48.64456477 | 2.446488878 | 1.290712724 | 0.003977403 | 0.020676539 | - |
| *CEPR1* | 0.374965051 | 9.118908593 | 24.31935608 | 4.604033125 | 0.003992265 | 0.020745402 | - |
| *At5g07050* | 37.83681298 | 286.7521907 | 7.578656026 | 2.921942028 | 0.003993154 | 0.02074583 | - |
| *SWEET16* | 0.374965051 | 10.08694202 | 26.90101915 | 4.749588925 | 0.00399501 | 0.020751273 | K15382 |
| *Os12g0628600* | 4.465017138 | 21.36061424 | 4.783993785 | 2.258215515 | 0.004062883 | 0.021061243 | - |
| *RAV2* | 31.52457101 | 67.77451731 | 2.149894991 | 1.104266195 | 0.004191818 | 0.021646632 | K09287 |
| *GDPD1* | 291.7793581 | 761.7975074 | 2.610868406 | 1.384529744 | 0.004198027 | 0.021674339 | K18696 |
| *NCED1* | 2.572892163 | 28.35447217 | 11.02046661 | 3.462113404 | 0.004288962 | 0.022103859 | K09840 |
| *CRJ34* | 0 | 14.35884259 | Inf | Inf | 0.004314307 | 0.022221101 | - |
| *SAC3A* | 16.81808967 | 43.54327239 | 2.589073625 | 1.372435991 | 0.004317284 | 0.022227524 | - |
| *uncharacterized protein_46359* | 2.60401008 | 15.72309018 | 6.038029693 | 2.594077852 | 0.004361715 | 0.022429308 | - |
| *ACO3* | 57.55086071 | 119.6307261 | 2.078695689 | 1.05567857 | 0.004364857 | 0.022440972 | K01681 |
| *At4g37250* | 3.310726847 | 17.4779804 | 5.27919735 | 2.400318599 | 0.004431899 | 0.022731068 | - |
| *uncharacterized protein_04905* | 3.671873701 | 18.28494832 | 4.979732367 | 2.316068208 | 0.004434105 | 0.022736637 | K19613 |
| *ROQ1* | 4.796768855 | 24.05607885 | 5.015059008 | 2.326266676 | 0.0044785 | 0.022919751 | - |
| *PME51* | 25.7844102 | 57.92866655 | 2.246654707 | 1.167778411 | 0.004482391 | 0.022930525 | - |
| *UGT85A1* | 15.40978851 | 40.70916616 | 2.64177319 | 1.401506609 | 0.004515133 | 0.023079644 | - |
| *RUN1* | 6.616285357 | 24.53182093 | 3.707793664 | 1.890560961 | 0.00452016 | 0.023096148 | - |
| *UCC1* | 2.546978548 | 16.52455453 | 6.487904873 | 2.697752667 | 0.004544808 | 0.023199021 | - |
| *SULTR3;4* | 423.7524603 | 937.9368169 | 2.213407366 | 1.146268996 | 0.004566023 | 0.023284179 | K17471 |
| *GTE9* | 35.0979515 | 72.44538383 | 2.064091514 | 1.045506936 | 0.004585755 | 0.02336625 | - |
| *PAP2* | 69.40979454 | 180.716958 | 2.603623296 | 1.380520728 | 0.004592699 | 0.023396992 | - |
| *HSP18.1* | 360.3676099 | 740.4058002 | 2.054584762 | 1.03884685 | 0.004597368 | 0.023416136 | K13993 |
| *B3GALT2* | 253.3231141 | 515.6239975 | 2.035439992 | 1.025340689 | 0.004617429 | 0.023496953 | - |
| *LAMP1* | 135.8302773 | 274.8431251 | 2.023430495 | 1.016803293 | 0.004640481 | 0.023594068 | K01301 |
| *PCMP-H73* | 8.795225935 | 28.70905878 | 3.264163876 | 1.706713489 | 0.004779832 | 0.024211095 | - |
| *HHP4* | 222.9688495 | 710.9027864 | 3.188350247 | 1.672810121 | 0.004841045 | 0.024482535 | K07297 |
| *uncharacterized protein_08487* | 18.79829217 | 46.12070437 | 2.453451832 | 1.294812948 | 0.004851367 | 0.024520257 | - |
| *SEC23* | 15.43573809 | 40.38754215 | 2.616495688 | 1.387635881 | 0.004908597 | 0.024785137 | K14006 |
| *NAC056* | 7.350674483 | 26.69161969 | 3.631179663 | 1.860438313 | 0.004943876 | 0.02494366 | - |
| *FRL4A* | 0 | 7.111510368 | Inf | Inf | 0.004956112 | 0.024999838 | - |
| *DCR* | 19.48950392 | 65.17603733 | 3.344160918 | 1.74164427 | 0.004984938 | 0.025116306 | - |
| *uncharacterized protein_38654* | 2.182386136 | 14.51077469 | 6.649040907 | 2.733146254 | 0.005041151 | 0.025374652 | - |
| *RPS4D* | 0 | 7.044238694 | Inf | Inf | 0.005104406 | 0.025642804 | K02987 |
| *CRJ37* | 0.374965051 | 23.47831701 | 62.61468086 | 5.968429051 | 0.005138475 | 0.02579378 | - |
| *uncharacterized protein_11372* | 2.579819244 | 15.37904228 | 5.961286752 | 2.575623772 | 0.005144354 | 0.025818247 | - |
| *ZAT3* | 2.966879713 | 16.2386449 | 5.473307473 | 2.452412905 | 0.005176891 | 0.025961256 | - |
| *ADH* | 1.092054457 | 13.00201087 | 11.90600961 | 3.573618059 | 0.005201036 | 0.02606199 | K18857 |
| *uncharacterized protein_23461* | 3.276163372 | 17.1083178 | 5.222058809 | 2.384618705 | 0.005203746 | 0.026070485 | - |
| *CYP707A1* | 330.4983448 | 722.3994484 | 2.1857884 | 1.128153745 | 0.005298187 | 0.026481656 | K09843 |
| *NPF2.13* | 2.61265994 | 27.83891745 | 10.65539262 | 3.413511848 | 0.005365561 | 0.026776737 | - |
| *EPHX2* | 22.00188303 | 60.17221829 | 2.734866747 | 1.451470541 | 0.005480549 | 0.027255282 | - |
| *PUB23* | 1.102427096 | 21.74206464 | 19.72199768 | 4.301733787 | 0.005495477 | 0.027318941 | - |
| *At1g67340* | 333.2825309 | 669.2889016 | 2.008172765 | 1.005883391 | 0.005599911 | 0.027789708 | - |
| *vps13A* | 42.78723285 | 101.7103372 | 2.377118838 | 1.249214029 | 0.005643967 | 0.027970518 | K19525 |
| *LOX1.1* | 0.729184824 | 62.82856267 | 86.16274032 | 6.428992229 | 0.005652489 | 0.028001948 | K15718 |
| *RCOM_1506700* | 42.25506333 | 94.55123646 | 2.237630925 | 1.161972098 | 0.005679431 | 0.028129993 | K01267 |
| *APCB1* | 5.876727894 | 22.45486768 | 3.82098135 | 1.933943216 | 0.005697397 | 0.02819723 | - |
| *HSP18.1* | 1.092054457 | 33.50076855 | 30.67682964 | 4.939077487 | 0.005703856 | 0.02822376 | K13993 |
| *NHX2* | 9.489883248 | 29.48171762 | 3.106647031 | 1.635358336 | 0.005741894 | 0.028384645 | - |
| *SbtS* | 33.75537479 | 81.85516921 | 2.424952166 | 1.27795629 | 0.005765433 | 0.02847909 | - |
| *ASR1* | 1.088608899 | 28.18471229 | 25.89057678 | 4.6943552 | 0.005820623 | 0.028718575 | - |
| *MES1* | 329.9817523 | 692.0950106 | 2.097373585 | 1.068583858 | 0.005831896 | 0.028763146 | - |
| *HMGS* | 23.53282519 | 53.24181657 | 2.26244899 | 1.177885265 | 0.005856804 | 0.028852761 | K01641 |
| *uncharacterized protein_17502* | 12.49473603 | 76.93558988 | 6.157440196 | 2.622330711 | 0.005941555 | 0.02917517 | - |
| *CNR9* | 11.71889231 | 33.35656454 | 2.846392274 | 1.5091345 | 0.00598407 | 0.029338917 | - |
| *F3H-2* | 0 | 7.633007683 | Inf | Inf | 0.006052945 | 0.029625874 | - |
| *CCT2* | 35.3035807 | 71.57571094 | 2.027434881 | 1.019655578 | 0.006114733 | 0.029848679 | K00968 |
| *TCHQD* | 12.50686741 | 45.31961107 | 3.623578117 | 1.857414996 | 0.006128185 | 0.029908661 | - |
| *R40G2* | 91.22666398 | 404.4561023 | 4.43352946 | 2.148455662 | 0.00617847 | 0.030112967 | - |
| *uncharacterized protein_04285* | 19.84889204 | 47.0255938 | 2.369179787 | 1.244387683 | 0.006187536 | 0.030135354 | - |
| *XRN3* | 20.29470682 | 47.58049273 | 2.344477955 | 1.229266714 | 0.006190272 | 0.030142183 | K12619 |
| *At3g27220* | 28.64928631 | 68.9246845 | 2.40580808 | 1.266521558 | 0.006208395 | 0.030214032 | - |
| *TIFY10B* | 0 | 6.822380521 | Inf | Inf | 0.00624524 | 0.030376079 | K13464 |
| *CRK14* | 0 | 6.799956629 | Inf | Inf | 0.006306739 | 0.030628814 | - |
| *7-Oct* | 22.37857086 | 51.06875673 | 2.282038341 | 1.190323031 | 0.00632157 | 0.030683442 | - |
| *ATJ8* | 0 | 12.51663755 | Inf | Inf | 0.006337455 | 0.030737317 | - |
| *SBT6.1* | 18.4198456 | 44.22898746 | 2.401159512 | 1.263731247 | 0.006612334 | 0.031919857 | K08653 |
| *MAPKKK17* | 1.114522514 | 10.91680226 | 9.795048663 | 3.29205266 | 0.006636969 | 0.032026742 | - |
| *mshA* | 0.725739266 | 9.740171744 | 13.42103452 | 3.746423977 | 0.006707892 | 0.032314357 | - |
| *PT3* | 42.83210413 | 102.1403921 | 2.384669026 | 1.253789045 | 0.006726269 | 0.032390738 | K08176 |
| *LOX1.5* | 0.729184824 | 9.717747853 | 13.32686519 | 3.736265557 | 0.006747593 | 0.032444771 | K15718 |
| *LAMP1* | 23.83177218 | 53.21479657 | 2.232934931 | 1.158941211 | 0.006749268 | 0.032446752 | K01301 |
| *At5g25050* | 40.11416775 | 88.36139988 | 2.202747928 | 1.139304409 | 0.006812329 | 0.032719303 | - |
| *CYP76T24* | 2.191035996 | 13.91512624 | 6.350934566 | 2.666968906 | 0.006862753 | 0.032943009 | - |
| *ANR* | 0 | 31.85662189 | Inf | Inf | 0.006916419 | 0.033169635 | K08695 |
| *TCEA1* | 7.36449268 | 24.85206905 | 3.37457991 | 1.754707917 | 0.006931508 | 0.033223391 | - |
| *At3g03770* | 4.475389777 | 18.87556164 | 4.21763524 | 2.07643433 | 0.006959999 | 0.033353729 | - |
| *DGAT2D* | 35.93944774 | 71.99068976 | 2.003110629 | 1.002242101 | 0.007155977 | 0.034184491 | K14457 |
| *WNK2* | 11.74308315 | 32.76229198 | 2.789922507 | 1.48022505 | 0.007174828 | 0.034255437 | K08867 |
| *MSH5* | 1.46357395 | 47.38159486 | 32.37389874 | 5.016759212 | 0.007222241 | 0.034468992 | K08741 |
| *OFUT20* | 5.880173452 | 21.79469934 | 3.706472184 | 1.890046684 | 0.007266122 | 0.034659104 | - |
| *At3g21360* | 0.364592412 | 10.71142539 | 29.37917804 | 4.876722128 | 0.00729111 | 0.034752486 | - |
| *VIT_06s0061g00120* | 0 | 14.79071231 | Inf | Inf | 0.007327625 | 0.034907104 | - |
| *ROQ1* | 0 | 13.66899985 | Inf | Inf | 0.007397628 | 0.035168916 | - |
| *At2g26970* | 15.84695343 | 39.59155196 | 2.498369931 | 1.320987111 | 0.007398827 | 0.035168916 | K13288 |
| *PNC1* | 1.46357395 | 17.07034948 | 11.66346905 | 3.543925047 | 0.007411446 | 0.035200739 | K00430 |
| *SULTR3;3* | 24.80814159 | 91.66390029 | 3.694912009 | 1.885540008 | 0.007450322 | 0.035353937 | K17471 |
| *uncharacterized protein_23707* | 0.362869633 | 10.36324981 | 28.55915421 | 4.835881349 | 0.007461901 | 0.035402345 | - |
| *CDC20-1* | 0.362869633 | 8.22590435 | 22.66903483 | 4.502651062 | 0.007491272 | 0.035513331 | K03363 |
| *uncharacterized protein_27586* | 0 | 51.7855515 | Inf | Inf | 0.007491487 | 0.035513331 | - |
| *uncharacterized protein_02859* | 0.362869633 | 8.224528458 | 22.66524313 | 4.502409732 | 0.00749497 | 0.035513331 | - |
| *GOLS4* | 1.838539001 | 19.61257407 | 10.66747785 | 3.41514721 | 0.007630849 | 0.03607068 | K18819 |
| *MGST3* | 18.78099245 | 44.29807404 | 2.358665239 | 1.237970674 | 0.007689286 | 0.036293488 | K00799 |
| *Ccdc90b* | 25.67027521 | 55.4157159 | 2.158750362 | 1.110196419 | 0.00770554 | 0.036343499 | - |
| *MAPKKK17* | 6.243043085 | 36.44864378 | 5.838281633 | 2.545543807 | 0.007826291 | 0.036838631 | - |
| *ANR* | 164.1994446 | 344.0015234 | 2.095022454 | 1.066965707 | 0.007918088 | 0.03720256 | K08695 |
| *uncharacterized protein_25383* | 0 | 6.516330352 | Inf | Inf | 0.007929933 | 0.03724459 | - |
| *uncharacterized protein_29508* | 0 | 6.498941595 | Inf | Inf | 0.007990494 | 0.037487905 | - |
| *CRRSP55* | 1.100704317 | 10.59061155 | 9.621668044 | 3.266287026 | 0.008025816 | 0.037639875 | - |
| *XTH2* | 0 | 6.481552839 | Inf | Inf | 0.008051646 | 0.037735601 | K08235 |
| *uncharacterized protein_09498* | 31.12365638 | 166.2589448 | 5.341883447 | 2.417348499 | 0.008063357 | 0.037761878 | - |
| *uncharacterized protein_08416* | 0 | 25.29040863 | Inf | Inf | 0.00818188 | 0.038153448 | K19037 |
| *SBT6.1* | 25.4353228 | 54.51905241 | 2.143438589 | 1.099927083 | 0.008196841 | 0.038204833 | K08653 |
| *CAT1* | 86.5390345 | 381.8351488 | 4.412288061 | 2.141526982 | 0.008205475 | 0.038238139 | K03294 |
| *DAO* | 0.362869633 | 12.11263646 | 33.38013257 | 5.060917779 | 0.008323999 | 0.038699213 | - |
| *At1g13630* | 13.60753577 | 35.41934925 | 2.602921636 | 1.380131878 | 0.008354068 | 0.038824957 | - |
| *WRKY51* | 1.817793724 | 18.40710863 | 10.12607118 | 3.340002625 | 0.008516702 | 0.039452323 | - |
| *MIMI_L728* | 11.73787885 | 32.14149726 | 2.738271342 | 1.453265414 | 0.008630232 | 0.039927839 | - |
| *uncharacterized protein_35207* | 31.59369796 | 64.02272901 | 2.026439864 | 1.018947363 | 0.008633599 | 0.039934112 | - |
| *AATP1* | 8.919661637 | 63.33708192 | 7.100839079 | 2.827989512 | 0.008634697 | 0.039934112 | K08900 |
| *CHI4* | 0.364592412 | 18.17198938 | 49.84192971 | 5.639288021 | 0.008690819 | 0.040164746 | K01183 |
| *pgk* | 74.19605399 | 165.279377 | 2.227603331 | 1.155492355 | 0.008730735 | 0.040327458 | K00927 |
| *Os02g0677700* | 10.35384045 | 29.50232659 | 2.849409042 | 1.51066274 | 0.008768007 | 0.040455974 | - |
| *SAP8* | 25.67027521 | 93.38248109 | 3.637767041 | 1.863053157 | 0.008776232 | 0.040486653 | - |
| *WIN1* | 0.374965051 | 15.08716065 | 40.23617829 | 5.330421377 | 0.00882846 | 0.04069106 | - |
| *uncharacterized protein_43367* | 30.05238317 | 61.6611832 | 2.051790131 | 1.036883171 | 0.008845117 | 0.04075321 | - |
| *PUB45* | 17.68036006 | 41.94800381 | 2.372576331 | 1.246454503 | 0.0088711 | 0.04085827 | - |
| *MTERF15* | 5.080174863 | 19.8111009 | 3.899688777 | 1.963358991 | 0.009198224 | 0.042160853 | K15032 |
| *At1g56140* | 27.3343389 | 115.0518587 | 4.209059497 | 2.073497903 | 0.009220219 | 0.042239059 | K04733 |
| *GGCT2;1* | 8.083304866 | 25.38410507 | 3.140312717 | 1.650908232 | 0.009277663 | 0.042464014 | - |
| *GOLS1* | 26.48247711 | 239.3682353 | 9.038740384 | 3.176121736 | 0.00955 | 0.043564869 | K18819 |
| *Mgst3* | 74.93044311 | 180.8277402 | 2.413274667 | 1.270992125 | 0.009550323 | 0.043564869 | K00799 |
| *uncharacterized protein_34335* | 7.345470182 | 23.93207421 | 3.258072475 | 1.704018696 | 0.009633283 | 0.043912115 | K14861 |
| *GFAT1* | 0.749930102 | 9.173358211 | 12.2322843 | 3.612621937 | 0.009658515 | 0.044003709 | K00820 |
| *uncharacterized protein_43566* | 18.32480503 | 42.80441563 | 2.335872909 | 1.223961781 | 0.009675328 | 0.044072491 | - |
| *PCO3* | 0.729184824 | 9.183896917 | 12.59474499 | 3.654750007 | 0.0096886 | 0.044117304 | K10712 |
| *uncharacterized protein_07518* | 1.487764786 | 20.70407573 | 13.91622918 | 3.798696438 | 0.009719959 | 0.04422874 | - |
| *DTX16* | 27.48638212 | 57.19847475 | 2.080975026 | 1.057259651 | 0.009751449 | 0.044340617 | K03327 |
| *CIPK18* | 1.114522514 | 16.3589903 | 14.67802588 | 3.87558604 | 0.009842414 | 0.044667279 | K07198 |
| *NPC2* | 0 | 6.24230591 | Inf | Inf | 0.009931598 | 0.045000472 | K01114 |
| *syn7A* | 29.70164492 | 60.61821798 | 2.040904406 | 1.02920861 | 0.009955385 | 0.045078017 | K08488 |
| *LRK10L-1.2* | 7.350674483 | 27.34390372 | 3.719917646 | 1.895270683 | 0.010033774 | 0.04535146 | - |
| *MAPKKK18* | 0.374965051 | 9.805160068 | 26.14953058 | 4.708713143 | 0.010099751 | 0.045617565 | K04373 |
| *CHIT5* | 14.42659282 | 48.24150055 | 3.343928893 | 1.74154417 | 0.010115982 | 0.045682845 | K01183 |
| *GATL3* | 10.303736 | 31.18763237 | 3.026827587 | 1.597806499 | 0.010133943 | 0.045739834 | - |
| *uncharacterized protein_29023* | 0 | 6.195174777 | Inf | Inf | 0.010138378 | 0.045743776 | - |
| *uncharacterized protein_08850* | 0 | 6.193798884 | Inf | Inf | 0.010144497 | 0.045763349 | - |
| *AGD3* | 31.28093278 | 62.71525295 | 2.004903543 | 1.003532829 | 0.01025 | 0.046166332 | K12489 |
| *ALKBH8* | 20.95821025 | 46.75341368 | 2.230792283 | 1.157556185 | 0.010261821 | 0.046203373 | K10770 |
| *CRK8* | 0 | 6.166339857 | Inf | Inf | 0.010267628 | 0.04622142 | - |
| *MYB5* | 48.28214763 | 127.7301182 | 2.645493718 | 1.403536992 | 0.010306559 | 0.046368887 | K09422 |
| *ACA5* | 17.63883354 | 41.39310488 | 2.346703073 | 1.23063531 | 0.010335426 | 0.046469623 | K01537 |
| *At5g03610* | 12.04203013 | 53.14165028 | 4.413014225 | 2.141764398 | 0.010356386 | 0.046549824 | - |
| *HSP18.1* | 1.112799735 | 43.02001899 | 38.65926422 | 5.272742275 | 0.010423068 | 0.046773633 | K13993 |
| *BGAL8* | 4.82960955 | 19.488101 | 4.035129713 | 2.012615051 | 0.010428758 | 0.046790994 | - |
| *CYP704C1* | 9.602187569 | 34.41795277 | 3.584386633 | 1.841726263 | 0.010508151 | 0.047065013 | - |
| *BRL2* | 26.46514143 | 64.50259876 | 2.437266354 | 1.285263923 | 0.010537638 | 0.047155976 | - |
| *uncharacterized protein_43773* | 7.718712454 | 43.81003717 | 5.675821899 | 2.50482932 | 0.010555974 | 0.047213357 | - |
| *H2AX* | 14.56998618 | 37.04113999 | 2.542290674 | 1.346128991 | 0.010660529 | 0.047639528 | K11251 |
| *MFP* | 4.069306809 | 17.17146179 | 4.219751078 | 2.077157897 | 0.010670906 | 0.047677607 | K10527 |
| *RABA1F* | 19.05050833 | 58.89300217 | 3.091413685 | 1.628266725 | 0.010768094 | 0.048086757 | K07904 |
| *mpaB* | 0.364592412 | 22.25352109 | 61.03670934 | 5.931605279 | 0.010884009 | 0.048511639 | - |
| *PHYPADRAFT_128349* | 27.09932165 | 79.07845917 | 2.918097367 | 1.545028022 | 0.010887451 | 0.048518562 | K08967 |
| *SWEET3B* | 0 | 22.91197191 | Inf | Inf | 0.011077823 | 0.049213274 | K15382 |
| *CRK8* | 11.86407329 | 31.30387951 | 2.638544009 | 1.399742047 | 0.011134833 | 0.049389676 | - |
| *VPS13D* | 0.374965051 | 7.66597028 | 20.44449278 | 4.353640366 | 0.011148751 | 0.049434337 | K19525 |
| *hrq1* | 10.70461467 | 32.94755773 | 3.077883582 | 1.621938664 | 0.011245911 | 0.049796399 | K06877 |
| *BCE2* | 29.30413988 | 59.51614821 | 2.030980894 | 1.022176668 | 0.011262582 | 0.04985303 | K09699 |
| *ACO* | 1.088608899 | 15.90241334 | 14.60801335 | 3.868688084 | 0.011338197 | 0.050161807 | K05933 |
| *uncharacterized protein_41526* | 24.27062391 | 51.54231285 | 2.123650098 | 1.086546081 | 0.011372132 | 0.050260009 | - |
| *At4g22758* | 1.090331678 | 10.02517391 | 9.194609414 | 3.20078829 | 0.011376662 | 0.050271383 | - |
| *DCL3A* | 9.51403812 | 27.39241075 | 2.879157136 | 1.525646529 | 0.011401303 | 0.05034563 | K11592 |
| *ETR1* | 0 | 6.17228245 | Inf | Inf | 0.011414608 | 0.05039572 | K14509 |
| *uncharacterized protein_25609* | 177.979546 | 1221.191455 | 6.861414601 | 2.778506044 | 0.011524282 | 0.050749112 | - |
| *CRK15* | 1.124895153 | 10.87057859 | 9.663637147 | 3.272566285 | 0.011708271 | 0.051462309 | - |
| *CMLN* | 3.30555851 | 44.78038336 | 13.54699462 | 3.759900923 | 0.011775507 | 0.051695948 | - |
| *At5g01750* | 5.095715839 | 19.14864921 | 3.757793765 | 1.909885891 | 0.011776592 | 0.051695948 | - |
| *SDR1* | 2.546978548 | 24.92897197 | 9.787664677 | 3.290964676 | 0.011777524 | 0.051695948 | K00059 |
| *uncharacterized protein_10660* | 0.729184824 | 8.859521122 | 12.14989784 | 3.602872278 | 0.011803325 | 0.051791498 | - |
| *RBL14* | 7.354120041 | 59.2914023 | 8.062338113 | 3.011198287 | 0.011831381 | 0.051896877 | K09651 |
| *XTH2* | 3.680523561 | 28.88009716 | 7.846736119 | 2.972092684 | 0.011894017 | 0.052118231 | K08235 |
| *uncharacterized protein_17210* | 16.90272164 | 289.6240027 | 17.13475551 | 4.098853702 | 0.012310517 | 0.053641367 | - |
| *BSP* | 1.829889142 | 16.57030977 | 9.055362642 | 3.178772418 | 0.012466248 | 0.054200438 | - |
| *uncharacterized protein_39322* | 22.34748891 | 48.17660962 | 2.155795213 | 1.108220138 | 0.012553248 | 0.054501145 | - |
| *CRK8* | 2.22559947 | 12.72567366 | 5.717863359 | 2.515476144 | 0.012554504 | 0.054501145 | - |
| *uncharacterized protein_38381* | 0 | 5.939446548 | Inf | Inf | 0.012598901 | 0.054666165 | - |
| *MKK9* | 55.84196171 | 113.872739 | 2.039196609 | 1.028000879 | 0.01261864 | 0.054733322 | K20604 |
| *SDR2a* | 5.443044496 | 151.7818047 | 27.88546094 | 4.801441214 | 0.012659727 | 0.054883741 | - |
| *At1g54200* | 21.37466586 | 46.31967022 | 2.167035992 | 1.115723115 | 0.012674792 | 0.054921246 | - |
| *At4g33300* | 0 | 5.924809577 | Inf | Inf | 0.012679688 | 0.054933198 | - |
| *PGMP* | 26.01412235 | 53.78573778 | 2.067559192 | 1.047928633 | 0.012718224 | 0.055081573 | K01835 |
| *MOR1* | 8.047018612 | 24.46135845 | 3.039803886 | 1.603978251 | 0.012721248 | 0.055085385 | K16803 |
| *GSTU17* | 19.94565538 | 43.9201855 | 2.201992598 | 1.138809619 | 0.012837212 | 0.055475327 | K00799 |
| *uncharacterized protein_02990* | 16.09924153 | 41.2068637 | 2.559553109 | 1.355891942 | 0.012906962 | 0.055720511 | - |
| *Os02g0831100* | 18.76021121 | 42.15065831 | 2.246811501 | 1.167879093 | 0.012988968 | 0.056008658 | - |
| *PLT4* | 8.848775944 | 25.75329923 | 2.910379853 | 1.541207461 | 0.013069139 | 0.056307104 | - |
| *At2g24130* | 10.54733827 | 68.1756104 | 6.463773957 | 2.692376747 | 0.013167122 | 0.056684551 | - |
| *ROQ1* | 26.93686982 | 54.68606051 | 2.030156468 | 1.021590923 | 0.013242753 | 0.056978656 | - |
| *PR4B* | 22.42350698 | 47.88425955 | 2.135449178 | 1.094539564 | 0.013371972 | 0.057438445 | - |
| *PAP8* | 17.3278271 | 45.09187828 | 2.602281175 | 1.379776853 | 0.013441023 | 0.0576483 | K14379 |
| *MYB106* | 78.86338444 | 211.3630112 | 2.680115908 | 1.422295395 | 0.013529421 | 0.05797904 | K09422 |
| *ISA1* | 12.92156428 | 32.53083197 | 2.51756144 | 1.332026987 | 0.013600428 | 0.058247065 | K01214 |
| *SYP81* | 5.930313867 | 25.57346908 | 4.312329777 | 2.10846751 | 0.013814538 | 0.059102299 | K08492 |
| *LAX4* | 131.3580236 | 426.9383114 | 3.250188301 | 1.700523304 | 0.01382118 | 0.05912087 | K13946 |
| *2-Aug* | 51.54628042 | 141.857861 | 2.752048446 | 1.460505867 | 0.013927969 | 0.059488521 | K16585 |
| *MSL2* | 6.590371742 | 21.51244895 | 3.264223901 | 1.706740018 | 0.013958405 | 0.059588799 | - |
| *DB3S* | 0.749930102 | 10.06317165 | 13.41881279 | 3.746185132 | 0.014408551 | 0.061195254 | K01674 |
| *MFSD14A* | 18.06910735 | 40.45709718 | 2.239020246 | 1.162867574 | 0.014994481 | 0.06334606 | - |
| *At1g67720* | 13.54705868 | 33.38996616 | 2.46473917 | 1.301434982 | 0.015080301 | 0.063658893 | - |
| *UGT86A1* | 6.215406691 | 20.59611336 | 3.313719341 | 1.728451417 | 0.015109815 | 0.063734751 | - |
| *LAR* | 470.3166564 | 5461.222677 | 11.61179942 | 3.537519651 | 0.015110676 | 0.063734751 | K13081 |
| *PME53* | 0 | 11.46894941 | Inf | Inf | 0.015235102 | 0.06417985 | K01051 |
| *guaA* | 25.42498613 | 51.72845664 | 2.03455201 | 1.024711161 | 0.015443879 | 0.064895109 | K01951 |
| *MPT3* | 8.907566219 | 355.0406336 | 39.85832099 | 5.316809034 | 0.015819969 | 0.066193944 | K15102 |
| *BST1* | 5.897473172 | 19.88981874 | 3.372600122 | 1.753861273 | 0.015834088 | 0.066242232 | - |
| *LTPG5* | 4.793323296 | 27.79597282 | 5.798893815 | 2.535777721 | 0.015907941 | 0.066491672 | - |
| *uncharacterized protein_06759* | 4.420081024 | 17.70534214 | 4.005660088 | 2.002040002 | 0.015935942 | 0.066559954 | - |
| *RPK1* | 15.15574168 | 41.41693407 | 2.732755345 | 1.450356306 | 0.015951854 | 0.06660476 | K13420 |
| *CHS* | 22.38201642 | 66.43238139 | 2.968114228 | 1.569546615 | 0.015967241 | 0.066636516 | K00660 |
| *uncharacterized protein_38918* | 0 | 5.633396379 | Inf | Inf | 0.016008398 | 0.066764894 | - |
| *APF2* | 0 | 5.630644594 | Inf | Inf | 0.016027653 | 0.066815254 | - |
| *At1g64760* | 112.1242173 | 262.8914293 | 2.344644499 | 1.229369194 | 0.01610298 | 0.067083129 | - |
| *GOLS4* | 0 | 5.613255838 | Inf | Inf | 0.01615004 | 0.067235566 | K18819 |
| *uncharacterized protein_49948* | 2.951338737 | 13.85335814 | 4.693923461 | 2.230794317 | 0.016182274 | 0.067347937 | - |
| *NAC035* | 0 | 5.60456146 | Inf | Inf | 0.016211696 | 0.06743761 | - |
| *MAPR4* | 9.235872377 | 25.67733318 | 2.780174101 | 1.47517523 | 0.01624409 | 0.067561424 | - |
| *MTH_273* | 0.725739266 | 13.1931898 | 18.17896649 | 4.184198276 | 0.016301261 | 0.067722461 | K06910 |
| *uncharacterized protein_32547* | 11.75001023 | 32.85614463 | 2.796265194 | 1.48350119 | 0.016329235 | 0.06782771 | - |
| *PDC4* | 2.569446605 | 12.99147216 | 5.056136266 | 2.338035346 | 0.016728632 | 0.069251576 | K01568 |
| *TIC32B* | 28.78229994 | 68.057666 | 2.364566631 | 1.241575796 | 0.016770744 | 0.069403542 | - |
| *TBL11* | 74.50024124 | 151.9050784 | 2.038987738 | 1.027853099 | 0.016848837 | 0.069670604 | - |
| *P4H10* | 1.487764786 | 10.32209068 | 6.937985613 | 2.794516849 | 0.016913665 | 0.069890608 | K00472 |
| *PNC2* | 4.409708385 | 16.78853812 | 3.807176495 | 1.928721453 | 0.016942471 | 0.069956448 | K00430 |
| *NRAMP5* | 0.737834684 | 8.284013211 | 11.22746516 | 3.48896034 | 0.017049392 | 0.070328045 | K12347 |
| *uncharacterized protein_02987* | 13.95830998 | 68.55956834 | 4.911738487 | 2.29623375 | 0.017085 | 0.070431761 | - |
| *DLO2* | 1.850634419 | 11.24161708 | 6.074466661 | 2.602757745 | 0.017102825 | 0.07049393 | - |
| *EFR* | 3.317653928 | 26.97893463 | 8.131931543 | 3.02359807 | 0.017127133 | 0.070582799 | - |
| *Kdsr* | 2.184108915 | 12.10441051 | 5.542036127 | 2.470416116 | 0.017189032 | 0.070826533 | K04708 |
| *LRK10L-2.4* | 6.998141525 | 21.58566322 | 3.084485094 | 1.625029674 | 0.017589685 | 0.072141857 | - |
| *INV1* | 1.465296729 | 10.64596863 | 7.26540121 | 2.861042468 | 0.0176215 | 0.072230582 | K01193 |
| *ACS1* | 0 | 6.506728516 | Inf | Inf | 0.017726245 | 0.07256293 | K01762 |
| *DLO1* | 2.913293739 | 18.43412864 | 6.327590105 | 2.661656146 | 0.017971491 | 0.073461508 | - |
| *UGT86A1* | 1.088608899 | 37.45936844 | 34.41030885 | 5.104768936 | 0.018179938 | 0.074171953 | - |
| *Os12g0628600* | 1.104149876 | 567.9150397 | 514.3459709 | 9.006595293 | 0.018247844 | 0.074391908 | - |
| *POX1* | 7.331651985 | 22.09389946 | 3.01349539 | 1.591437858 | 0.018250697 | 0.074391908 | K00318 |
| *CIPK5* | 0 | 7.38231459 | Inf | Inf | 0.018251224 | 0.074391908 | K07198 |
| *At4g33300* | 5.178660986 | 18.06355858 | 3.488075128 | 1.802431114 | 0.018417538 | 0.07490343 | - |
| *uncharacterized protein_29748* | 2.92887068 | 13.62508893 | 4.651994036 | 2.217849247 | 0.018432628 | 0.074952936 | - |
| *PDC4* | 31.75090953 | 69.63051069 | 2.193024128 | 1.132921685 | 0.018442163 | 0.074979842 | K01568 |
| *CBK1* | 10.23633183 | 27.25521301 | 2.66259569 | 1.412833375 | 0.018481441 | 0.075068252 | - |
| *RFS2* | 25.64088008 | 51.47225997 | 2.007429535 | 1.005349347 | 0.0185663 | 0.075365273 | K06617 |
| *VAB* | 57.69611362 | 165.8960641 | 2.875342094 | 1.523733611 | 0.018757902 | 0.07598736 | - |
| *BOR1* | 1.100704317 | 9.194874645 | 8.353628219 | 3.062402937 | 0.018868986 | 0.076376733 | - |
| *slr0889* | 1.104149876 | 9.157345348 | 8.293570964 | 3.051993417 | 0.019036538 | 0.07694583 | K08869 |
| *LRK10L-1.1* | 8.437524639 | 23.95997226 | 2.839692124 | 1.505734523 | 0.019137637 | 0.077257231 | - |
| *RAMDAZC7* | 1.114522514 | 37.44335558 | 33.59587186 | 5.070212066 | 0.019143163 | 0.0772674 | - |
| *ERF1-3* | 2755.843986 | 6298.297243 | 2.285433165 | 1.192467629 | 0.019228734 | 0.077472242 | K03265 |
| *PHOS34* | 98.15571452 | 368.1500317 | 3.750673443 | 1.907149658 | 0.019230099 | 0.077472242 | - |
| *At1g75140* | 3.670150922 | 15.00252963 | 4.087714633 | 2.031294484 | 0.019253166 | 0.077553009 | - |
| *VIT_06s0061g00120* | 0 | 13.43206568 | Inf | Inf | 0.01934607 | 0.077853992 | - |
| *PHT1-4* | 47.20745773 | 262.6888228 | 5.564561944 | 2.47626812 | 0.019485704 | 0.078268798 | K08176 |
| *LTI6B* | 4.026093475 | 42.82082895 | 10.63582582 | 3.410860151 | 0.019832326 | 0.079338567 | - |
| *GGCT2;2* | 4.781227878 | 17.17236925 | 3.591623259 | 1.844636027 | 0.019884816 | 0.079499036 | - |
| *Os04g0656100* | 4.363049493 | 33.18912657 | 7.606864563 | 2.927301919 | 0.019893845 | 0.07951039 | K01535 |
| *EXLA2* | 0 | 9.827583959 | Inf | Inf | 0.019975893 | 0.079788661 | - |
| *DREB3* | 1.45664687 | 10.03020905 | 6.885820619 | 2.783628598 | 0.020042774 | 0.080006045 | K09286 |
| *SNAA* | 18.35247739 | 39.85000256 | 2.171369114 | 1.118604992 | 0.020077251 | 0.080084448 | K15296 |
| *At4g33300* | 5.878450673 | 26.12981188 | 4.44501678 | 2.152188865 | 0.020080178 | 0.080084448 | - |
| *GIP2* | 3.28825879 | 14.20334863 | 4.319413263 | 2.110835354 | 0.020151174 | 0.080326426 | - |
| *uncharacterized protein_45084* | 0 | 5.347925774 | Inf | Inf | 0.020162951 | 0.080348438 | - |
| *AGAL1* | 0 | 5.345642424 | Inf | Inf | 0.020183034 | 0.080403524 | K07407 |
| *REV1* | 24.64734771 | 49.32668857 | 2.001298037 | 1.000936032 | 0.02023086 | 0.080544093 | K03515 |
| *SAP* | 0 | 5.333288803 | Inf | Inf | 0.020292153 | 0.080750575 | - |
| *RAB28* | 0 | 5.331005453 | Inf | Inf | 0.020312409 | 0.080806146 | - |
| *uncharacterized protein_21925* | 0 | 5.32184264 | Inf | Inf | 0.020393962 | 0.081067815 | - |
| *uncharacterized protein_22845* | 3.326303788 | 53.60537121 | 16.11559696 | 4.010385725 | 0.020449764 | 0.081264482 | - |
| *Os02g0190300* | 14.97944278 | 73.27331152 | 4.891591269 | 2.29030386 | 0.0204808 | 0.081362648 | K05658 |
| *BGLU22* | 29.78283132 | 130.6839655 | 4.387895968 | 2.133529322 | 0.020521569 | 0.081474214 | K01188 |
| *At4g33300* | 0 | 5.307205669 | Inf | Inf | 0.02052515 | 0.081475838 | - |
| *TIR* | 57.94133077 | 151.0869859 | 2.607585705 | 1.382714671 | 0.020568235 | 0.08159966 | - |
| *uncharacterized protein_50920* | 0.364592412 | 6.788510466 | 18.61945076 | 4.218738611 | 0.020639779 | 0.081829717 | - |
| *Os07g0673200* | 8.133409317 | 23.05783462 | 2.834953181 | 1.503324909 | 0.020689166 | 0.081987561 | K16275 |
| *ccdc90b* | 0 | 5.28844102 | Inf | Inf | 0.020694988 | 0.081997984 | - |
| *HAT14* | 6.256861283 | 19.74886436 | 3.156353237 | 1.658258671 | 0.020779929 | 0.082283778 | K09338 |
| *DI19-1* | 0.737834684 | 7.939526287 | 10.76057613 | 3.427683417 | 0.020837201 | 0.082459723 | - |
| *GH3.6* | 0 | 8.871406308 | Inf | Inf | 0.020902439 | 0.082641516 | K14487 |
| *CYP76B10* | 12.13362514 | 31.22609854 | 2.573517657 | 1.363741681 | 0.020919909 | 0.082697858 | - |
| *At4g16563* | 40.80875314 | 83.03636643 | 2.034768525 | 1.024864683 | 0.021114613 | 0.083317029 | - |
| *uncharacterized protein_33602* | 5.106052514 | 17.67832214 | 3.462228814 | 1.791701074 | 0.021115456 | 0.083317029 | - |
| *At3g57050* | 6.612839799 | 20.40721779 | 3.085999118 | 1.625737649 | 0.021214912 | 0.083632393 | K01760 |
| *TMEM45B* | 2.937484575 | 13.29433152 | 4.525753645 | 2.178158055 | 0.021239088 | 0.083689175 | - |
| *At1g52310* | 151.5490688 | 377.7087636 | 2.492319924 | 1.31748927 | 0.021263557 | 0.083761774 | - |
| *At2g04570* | 7.768852869 | 57.73524284 | 7.431630359 | 2.893678746 | 0.021492741 | 0.084520131 | - |
| *At2g16250* | 4.416599502 | 16.23132642 | 3.67507319 | 1.877772982 | 0.021499085 | 0.084526495 | - |
| *SDR2a* | 0 | 5.861197146 | Inf | Inf | 0.02186668 | 0.08568897 | - |
| *CRK8* | 9.939143587 | 26.04242908 | 2.620188435 | 1.389670569 | 0.02193052 | 0.085873645 | - |
| *ADH2* | 22.13669138 | 121.5599925 | 5.491335199 | 2.457156978 | 0.022057957 | 0.0862806 | K18857 |
| *uncharacterized protein_02137* | 3.99845708 | 15.6119076 | 3.904482975 | 1.965131522 | 0.022091861 | 0.086347481 | - |
| *DCR* | 1.124895153 | 8.887887606 | 7.901080897 | 2.982050033 | 0.022354688 | 0.087202291 | K19747 |
| *CSTF77* | 14.07757735 | 32.52535781 | 2.310437159 | 1.208165851 | 0.022369957 | 0.087248605 | K14408 |
| *PAP4* | 39.70474712 | 198.1544811 | 4.990699991 | 2.319242181 | 0.022551739 | 0.087826992 | K14379 |
| *uncharacterized protein_03024* | 22.75698147 | 46.0776715 | 2.024770796 | 1.017758604 | 0.022552437 | 0.087826992 | K10581 |
| *uncharacterized protein_19790* | 0 | 9.823924717 | Inf | Inf | 0.022570481 | 0.087870621 | - |
| *RZPF34* | 1.453201311 | 9.989488945 | 6.874126018 | 2.7811763 | 0.022579495 | 0.087892396 | K10144 |
| *OMT2* | 0 | 26.86644412 | Inf | Inf | 0.022595067 | 0.087939684 | - |
| *CHX19* | 32.00494927 | 77.66098163 | 2.426530378 | 1.278894921 | 0.022741356 | 0.088415282 | - |
| *FEI1* | 22.39935211 | 45.46281938 | 2.029648856 | 1.021230152 | 0.022761858 | 0.088481601 | - |
| *NO93* | 1.114522514 | 15.9921089 | 14.34884329 | 3.842862536 | 0.022811598 | 0.088661537 | - |
| *OPT1* | 1.092054457 | 14.71887394 | 13.47815014 | 3.752550597 | 0.02287178 | 0.088855118 | - |
| *PAP1* | 18.07603443 | 38.76216015 | 2.144395128 | 1.100570762 | 0.023055871 | 0.089475585 | - |
| *Os01g0270100* | 1.479114927 | 9.788678769 | 6.617929813 | 2.726379991 | 0.023163551 | 0.089798518 | - |
| *At2g29640* | 1.84198456 | 10.65009631 | 5.781859708 | 2.531533603 | 0.023351947 | 0.090419725 | K15235 |
| *PLT4* | 20.59882214 | 42.62055519 | 2.069077295 | 1.048987541 | 0.023547635 | 0.091053927 | - |
| *RSH1* | 6.623212438 | 20.03708675 | 3.025282208 | 1.597069728 | 0.023747681 | 0.091786018 | - |
| *LKR/SDH* | 32.29689725 | 88.81565494 | 2.749974843 | 1.459418421 | 0.023886063 | 0.092263697 | K14157 |
| *RAB7* | 23.12333263 | 46.31097584 | 2.002781199 | 1.002004817 | 0.023899777 | 0.092284738 | K07897 |
| *uncharacterized protein_07671* | 92.65232969 | 249.4712578 | 2.692552455 | 1.428974451 | 0.02420031 | 0.093198946 | - |
| *PRP40B* | 14.32290239 | 34.13013084 | 2.382906055 | 1.252722075 | 0.02433009 | 0.093614547 | K12821 |
| *AHK4* | 15.02272804 | 33.90189105 | 2.256706701 | 1.174218927 | 0.024389227 | 0.093828037 | K14489 |
| *D6PKL1* | 2.92887068 | 12.97774265 | 4.430971548 | 2.147623063 | 0.024459743 | 0.094071147 | - |
| *TSO2* | 0.739557463 | 7.675572116 | 10.37860139 | 3.375540135 | 0.024834828 | 0.095228592 | K10808 |
| *OXR1* | 8.13516806 | 23.29892587 | 2.863975975 | 1.51801939 | 0.024913015 | 0.095428697 | - |
| *UBA2C* | 7.74290329 | 21.82309524 | 2.818464137 | 1.49490921 | 0.025029297 | 0.09584553 | K12741 |
| *uncharacterized protein_38987* | 31.21525139 | 90.89004203 | 2.911719047 | 1.541871156 | 0.025143521 | 0.096125326 | - |
| *MYB4* | 0.374965051 | 8.82931031 | 23.54702201 | 4.557472709 | 0.025239715 | 0.096407004 | K09422 |
| *ATG13B* | 8.085063609 | 22.46950465 | 2.779137647 | 1.474637291 | 0.025302702 | 0.096604505 | K08331 |
| *INV1* | 2.197927112 | 22.20186182 | 10.10127301 | 3.336465215 | 0.025405037 | 0.096817196 | K01193 |
| *PAP4* | 10.64934188 | 27.28999052 | 2.562598782 | 1.357607618 | 0.025408754 | 0.096817196 | K14379 |
| *At5g51140* | 0 | 5.052853333 | Inf | Inf | 0.02550748 | 0.097140922 | - |
| *At5g47540* | 35.23104415 | 93.79871815 | 2.662388255 | 1.412720974 | 0.025570995 | 0.097298625 | K08272 |
| *CNR10* | 0 | 5.04462739 | Inf | Inf | 0.02559909 | 0.09738875 | - |
| *At1g17710* | 17.36411336 | 235.9900408 | 13.59067612 | 3.764545325 | 0.025717657 | 0.097767434 | K13248 |
| *NFD4* | 24.84432704 | 79.25416164 | 3.190030525 | 1.673570229 | 0.025806073 | 0.098031026 | - |
| *GOLS2* | 0 | 5.024486849 | Inf | Inf | 0.025825284 | 0.098058726 | K18819 |
| *ABR1* | 4.416599502 | 56.1809386 | 12.72040595 | 3.669072808 | 0.025828633 | 0.098058726 | - |
| *uncharacterized protein_33727* | 0 | 5.351585017 | Inf | Inf | 0.025891349 | 0.09825327 | - |
| *VIT_06s0061g00120* | 0 | 5.351585017 | Inf | Inf | 0.025891349 | 0.09825327 | - |
| *MAPKKK17* | 0 | 5.012133228 | Inf | Inf | 0.025965368 | 0.09844691 | - |
| *uncharacterized protein_48766* | 0 | 5.012133228 | Inf | Inf | 0.025965368 | 0.09844691 | - |
| *At3g02910* | 17.12395665 | 50.05368956 | 2.923021273 | 1.547460329 | 0.025969866 | 0.098449437 | K19761 |
| *EXPA1* | 0 | 5.008473986 | Inf | Inf | 0.026007059 | 0.098557509 | - |
| *ZAT9* | 0.362869633 | 9.983546352 | 27.51276338 | 4.782029146 | 0.026009883 | 0.098557509 | - |
| *ABCC9* | 16.06636487 | 61.69817609 | 3.840207577 | 1.941184296 | 0.026049766 | 0.098694076 | - |
| *RLP39* | 0 | 5.002062958 | Inf | Inf | 0.026080323 | 0.098780713 | - |
| *uncharacterized protein_05772* | 3.350494624 | 19.08459775 | 5.696053836 | 2.509962782 | 0.026200957 | 0.099179135 | - |
| *HIPP20* | 0 | 4.99108523 | Inf | Inf | 0.026206425 | 0.099185217 | - |
| *TTA* | 0 | 5.566124705 | Inf | Inf | 0.0262341 | 0.099260715 | - |
| *RAP2-2* | 21.86190636 | 44.33241253 | 2.027838369 | 1.019942666 | 0.026284935 | 0.099394504 | K09286 |
| *NHX1* | 14.69962619 | 33.04913848 | 2.248297886 | 1.168833196 | 0.026321136 | 0.099487465 | - |
| *SPAC24B11.05* | 1438.018276 | 2.068258871 | 0.00143827 | -9.441449526 | 2.3925E-121 | 3.0733E-117 | K07025 |
| *TAT* | 847.999336 | 3.26593739 | 0.003851344 | -8.020422191 | 1.6723E-118 | 1.4321E-114 | K15400 |
| *nep2* | 3861.111809 | 54.49904782 | 0.014114859 | -6.146641498 | 4.5746E-102 | 1.95877E-98 | - |
| *nep2* | 581.5647775 | 4.171295254 | 0.007172538 | -7.123300652 | 8.374E-94 | 2.3904E-90 | - |
| *CYP750A1* | 2431.171365 | 5.945857576 | 0.002445676 | -8.675550966 | 1.68998E-76 | 2.89449E-73 | - |
| *AIR3* | 669.2668099 | 21.93884452 | 0.032780416 | -4.931022022 | 4.21766E-72 | 6.37388E-69 | - |
| *At5g63180* | 337.7970123 | 2.095717897 | 0.006204075 | -7.332568228 | 3.54309E-71 | 5.05698E-68 | K01728 |
| *ag4* | 308.1369081 | 1.780504915 | 0.005778292 | -7.435141267 | 8.72649E-68 | 1.06758E-64 | K18108 |
| *BXL4* | 441.0397928 | 10.61897804 | 0.024077143 | -5.376191994 | 1.10924E-65 | 1.29534E-62 | K15920 |
| *LAC6* | 514.8442293 | 1.785540051 | 0.003468117 | -8.171631693 | 4.11594E-60 | 3.91639E-57 | K05909 |
| *CYP75A3* | 366.1195496 | 3.525324861 | 0.00962889 | -6.698414792 | 6.96848E-60 | 6.39383E-57 | K13083 |
| *OPT4* | 616.4639909 | 32.12320105 | 0.052108804 | -4.262329058 | 1.3447E-58 | 1.11441E-55 | - |
| *DHAPS-1* | 13803.77972 | 156.0534024 | 0.011305121 | -6.466879734 | 3.07858E-58 | 2.32623E-55 | K01626 |
| *TAT* | 482.2596751 | 0 | 0 | -Inf | 4.7986E-57 | 3.42447E-54 | K15400 |
| *GAPB* | 62137.2696 | 7355.299456 | 0.118371784 | -3.078602865 | 2.15421E-56 | 1.49578E-53 | K05298 |
| *A6* | 869.1620569 | 62.80951521 | 0.072264447 | -3.790570156 | 2.89707E-56 | 1.95865E-53 | - |
| *MYB20* | 494.9914313 | 23.14524683 | 0.046758884 | -4.418615698 | 3.06787E-56 | 2.02094E-53 | K09422 |
| *uncharacterized protein_25495* | 2545.076708 | 268.8676023 | 0.105642239 | -3.242741309 | 1.09661E-54 | 7.04326E-52 | - |
| *At3g59480* | 375.1842772 | 2.075577356 | 0.005532154 | -7.497942858 | 8.3887E-54 | 5.13128E-51 | K00847 |
| *CTL2* | 1196.317276 | 37.52100974 | 0.031363762 | -4.994757595 | 2.28488E-53 | 1.33411E-50 | - |
| *MWL2* | 554.6690746 | 32.88998528 | 0.059296591 | -4.075907023 | 2.64291E-53 | 1.50887E-50 | - |
| *GAUT13* | 263.3226558 | 5.041875605 | 0.019147139 | -5.706727319 | 4.46671E-52 | 2.44158E-49 | K13648 |
| *4CL* | 2714.9389 | 310.5200469 | 0.114374599 | -3.128161414 | 6.42943E-52 | 3.44122E-49 | K01904 |
| *UGT74AC1* | 754.0521532 | 15.3717238 | 0.020385492 | -5.616313445 | 1.40339E-51 | 7.35806E-49 | K13691 |
| *R53* | 398.8159623 | 18.09877511 | 0.045381271 | -4.461759186 | 1.61293E-51 | 8.28755E-49 | K00430 |
| *FLA17* | 2137.888597 | 75.42128392 | 0.035278398 | -4.825071157 | 1.28166E-49 | 6.33212E-47 | - |
| *TAT* | 216.9067195 | 2.683579429 | 0.012372044 | -6.336772347 | 1.41561E-49 | 6.86199E-47 | K15400 |
| *EO* | 770.4599586 | 67.82892836 | 0.088036929 | -3.505747376 | 1.52017E-48 | 6.85171E-46 | K18980 |
| *agaA* | 169.22093 | 0.295072441 | 0.001743711 | -9.163623121 | 3.78922E-48 | 1.67843E-45 | - |
| *MAPKKK17* | 226.9927739 | 4.116845635 | 0.018136461 | -5.784963207 | 7.48046E-48 | 3.20301E-45 | - |
| *BGAL3* | 3572.512993 | 161.9473288 | 0.045331488 | -4.463342681 | 1.07856E-47 | 4.46926E-45 | - |
| *IP5P8* | 807.3060516 | 11.47995655 | 0.01422008 | -6.135926619 | 4.35267E-46 | 1.74726E-43 | - |
| *SAHH* | 1457.466333 | 122.1426425 | 0.083804778 | -3.57682369 | 5.04301E-46 | 1.99323E-43 | K01251 |
| *EXPA2* | 534.632544 | 6.804991764 | 0.012728353 | -6.295810434 | 1.09243E-45 | 4.25236E-43 | - |
| *BAM1* | 288.2551611 | 12.34184253 | 0.042815686 | -4.545716742 | 3.53141E-44 | 1.29608E-41 | - |
| *CYCU4-1* | 422.7583809 | 29.30608312 | 0.069321117 | -3.850561292 | 3.67816E-44 | 1.31244E-41 | - |
| *CBP1* | 1540.317503 | 197.8443416 | 0.12844387 | -2.960790053 | 4.91678E-44 | 1.73037E-41 | K16296 |
| *RBCS* | 1117.835507 | 134.5069474 | 0.120328033 | -3.05495531 | 1.35402E-43 | 4.57714E-41 | K01602 |
| *SBT5.1* | 1150.147874 | 139.2046686 | 0.121031975 | -3.046539858 | 1.83863E-43 | 6.13457E-41 | - |
| *DGAT3* | 2145.712128 | 201.5278736 | 0.093921207 | -3.412405236 | 8.45931E-43 | 2.75099E-40 | - |
| *CAHC* | 30754.05189 | 5074.586763 | 0.165005469 | -2.59941425 | 4.02828E-42 | 1.26208E-39 | K01673 |
| *GAT1* | 389.1621061 | 28.12119724 | 0.072260883 | -3.790641315 | 1.25691E-41 | 3.89051E-39 | - |
| *uncharacterized protein_17552* | 686.2033393 | 31.12959149 | 0.045364966 | -4.462277597 | 1.59915E-41 | 4.89092E-39 | - |
| *PTAC16* | 4374.322359 | 710.6403094 | 0.162457234 | -2.621868112 | 1.98941E-40 | 5.94302E-38 | - |
| *XTH32* | 308.9248183 | 1.781880808 | 0.005768008 | -7.437711132 | 1.00216E-39 | 2.89287E-37 | K08235 |
| *CYP704C1* | 357.3261112 | 27.21996706 | 0.076176821 | -3.714504115 | 5.41592E-39 | 1.48022E-36 | - |
| *RFS2* | 783.260749 | 97.62975155 | 0.124645275 | -3.004099899 | 5.80922E-39 | 1.571E-36 | K06617 |
| *JAL3* | 1531.432102 | 42.53039118 | 0.027771647 | -5.170243469 | 6.99201E-39 | 1.85187E-36 | - |
| *PILS7* | 451.514983 | 42.22843928 | 0.093526108 | -3.41848703 | 1.08303E-38 | 2.81052E-36 | K07088 |
| *PCAP1* | 137.5701215 | 0.900322728 | 0.006544464 | -7.255509213 | 1.83877E-38 | 4.72399E-36 | - |
| *NAC035* | 147.2846204 | 1.795610321 | 0.012191431 | -6.357988687 | 6.40107E-38 | 1.5966E-35 | - |
| *PCBER* | 549.5868102 | 0.591520774 | 0.001076301 | -9.859702829 | 8.99262E-38 | 2.22144E-35 | - |
| *uncharacterized protein_17867* | 167.7088305 | 3.827715787 | 0.022823579 | -5.453331132 | 2.38188E-37 | 5.72983E-35 | - |
| *R3* | 168.334253 | 3.557819023 | 0.021135443 | -5.564191832 | 2.38641E-37 | 5.72983E-35 | K00430 |
| *At4g01130* | 247.8040014 | 11.19311006 | 0.045169206 | -4.468516627 | 3.18E-37 | 7.56456E-35 | - |
| *IQD2* | 387.6135046 | 34.56706511 | 0.089179207 | -3.487148812 | 3.43711E-37 | 8.10117E-35 | - |
| *DNAJB4* | 681.6525145 | 85.71045057 | 0.125739213 | -2.991493459 | 4.27932E-37 | 9.99454E-35 | - |
| *BMY1* | 1009.526032 | 146.1315176 | 0.1447526 | -2.788338836 | 6.39401E-37 | 1.46668E-34 | - |
| *TBL24* | 361.5006874 | 11.25353168 | 0.031130042 | -5.005548654 | 1.6053E-36 | 3.61769E-34 | - |
| *SRG1* | 682.4925685 | 87.73020242 | 0.128543821 | -2.959667835 | 1.71349E-36 | 3.82794E-34 | - |
| *uncharacterized protein_49315* | 611.4523458 | 11.49368607 | 0.018797354 | -5.73332661 | 4.92283E-36 | 1.08096E-33 | - |
| *Prcp* | 575.9508902 | 70.05728646 | 0.121637604 | -3.039338788 | 6.5924E-36 | 1.4353E-33 | K01285 |
| *CHS* | 156.8589558 | 0.900322728 | 0.005739696 | -7.444809946 | 2.95878E-35 | 6.28214E-33 | K00660 |
| *HSL1* | 1619.631561 | 274.2502873 | 0.169328812 | -2.562100624 | 3.64633E-35 | 7.67852E-33 | K00924 |
| *At1g74320* | 1133.539193 | 31.1341876 | 0.027466353 | -5.18619083 | 2.33707E-34 | 4.80334E-32 | K14156 |
| *CYP75A3* | 1566.579867 | 38.96941077 | 0.02487547 | -5.329132395 | 2.39253E-34 | 4.8783E-32 | K13083 |
| *IQD18* | 740.9809554 | 98.18035744 | 0.132500514 | -2.915930139 | 3.21748E-34 | 6.50869E-32 | - |
| *HMA2* | 278.3612054 | 21.67811057 | 0.077877629 | -3.682647236 | 5.34107E-34 | 1.07201E-31 | K01534 |
| *ag4* | 246.8190829 | 6.828323113 | 0.027665297 | -5.175778804 | 8.21726E-34 | 1.59931E-31 | K18108 |
| *OMT1* | 504.2341229 | 63.00401175 | 0.124949917 | -3.000578153 | 9.81363E-34 | 1.89565E-31 | K13066 |
| *MYB61* | 263.5371364 | 19.57185397 | 0.074266019 | -3.751153945 | 1.01703E-33 | 1.94988E-31 | K09422 |
| *TUBB2* | 3033.159197 | 576.9194985 | 0.19020416 | -2.394379292 | 1.10727E-33 | 2.10718E-31 | K07375 |
| *RBCS* | 1996.287929 | 165.1301176 | 0.082718587 | -3.59564464 | 1.58564E-33 | 2.97021E-31 | K01602 |
| *At1g80170* | 560.3368716 | 74.74694704 | 0.133396446 | -2.906207868 | 2.97248E-33 | 5.49396E-31 | - |
| *HIPP22* | 111.6249533 | 0.595180017 | 0.005331962 | -7.551117768 | 3.03215E-33 | 5.52475E-31 | - |
| *TUBB8* | 192.7139155 | 9.494513786 | 0.049267401 | -4.343222817 | 3.30043E-33 | 5.97122E-31 | K07375 |
| *AAP3* | 1611.297438 | 291.0478797 | 0.180629518 | -2.468894421 | 4.00215E-33 | 7.19016E-31 | - |
| *uncharacterized protein_33452* | 276.8943369 | 8.328860994 | 0.030079564 | -5.055072523 | 4.11331E-33 | 7.33855E-31 | - |
| *CHS* | 2937.579684 | 2.957135436 | 0.001006657 | -9.956211951 | 3.35288E-32 | 5.85979E-30 | K00660 |
| *At4g02290* | 474.2487194 | 9.205852373 | 0.019411444 | -5.68694876 | 4.02533E-32 | 6.98749E-30 | - |
| *Hgsnat* | 646.2308857 | 97.88162576 | 0.15146541 | -2.722939735 | 1.46903E-31 | 2.49939E-29 | K10532 |
| *CCOAOMT* | 176.1047926 | 8.569015381 | 0.048658615 | -4.36116092 | 1.97758E-31 | 3.34249E-29 | K00588 |
| *CESA4* | 1654.11229 | 41.25318476 | 0.024939773 | -5.325407866 | 6.50106E-31 | 1.07754E-28 | K10999 |
| *BRL2* | 213.1328062 | 6.556142999 | 0.030760835 | -5.022761543 | 1.04722E-30 | 1.7028E-28 | - |
| *At4g39970* | 3151.153794 | 662.1057479 | 0.210115339 | -2.250746608 | 1.73033E-30 | 2.77837E-28 | - |
| *AGPS1* | 3654.547528 | 628.240675 | 0.171906555 | -2.540303539 | 2.17682E-30 | 3.47358E-28 | K00975 |
| *CYP75B137* | 248.0254953 | 21.30982386 | 0.085917876 | -3.540897853 | 2.38528E-30 | 3.78273E-28 | K05280 |
| *DLO1* | 274.3054073 | 15.92571527 | 0.058058335 | -4.106352987 | 2.88071E-30 | 4.51271E-28 | - |
| *NPF5.6* | 89.7838353 | 0 | 0 | -Inf | 4.11248E-30 | 6.36468E-28 | K14638 |
| *PCBER* | 976.0030024 | 14.18915068 | 0.01453802 | -6.104025444 | 4.47247E-30 | 6.88038E-28 | - |
| *BXL4* | 817.6045696 | 40.94804205 | 0.050082942 | -4.319536884 | 1.19385E-29 | 1.79364E-27 | K15920 |
| *GGT3* | 1670.958995 | 338.1348523 | 0.202359755 | -2.305005699 | 1.24576E-29 | 1.84999E-27 | K18592 |
| *UFO* | 433.0222625 | 4.719344138 | 0.010898618 | -6.519711011 | 2.79959E-29 | 4.06352E-27 | - |
| *At2g27500* | 157.9875483 | 7.685173952 | 0.048644175 | -4.361589132 | 3.23254E-29 | 4.66557E-27 | - |
| *GAPA* | 51547.55713 | 12062.03547 | 0.233998198 | -2.095430676 | 5.28764E-29 | 7.46399E-27 | K05298 |
| *HHT1* | 340.7275409 | 41.74262694 | 0.122510281 | -3.029025265 | 5.90938E-29 | 8.29606E-27 | K15400 |
| *RCA1* | 314821.2911 | 74268.62377 | 0.235907246 | -2.083708363 | 6.50084E-29 | 9.0768E-27 | - |
| *At1g64710* | 362.6139977 | 44.99261029 | 0.124078526 | -3.010674644 | 7.01599E-29 | 9.74312E-27 | K00001 |
| *IQD30* | 1161.060412 | 199.3366444 | 0.171684989 | -2.542164187 | 8.09204E-29 | 1.11172E-26 | - |
| *CSLA9* | 441.2399158 | 7.050649722 | 0.015979175 | -5.967663288 | 8.94405E-29 | 1.22224E-26 | K13680 |
| *RACD* | 514.4087153 | 57.25758846 | 0.111307579 | -3.167376271 | 1.18691E-28 | 1.6049E-26 | - |
| *DAHP1* | 1367.231867 | 277.1772908 | 0.202728811 | -2.302376961 | 1.47817E-28 | 1.9779E-26 | K01626 |
| *OPT3* | 7259.599421 | 1679.067697 | 0.231289304 | -2.112229545 | 1.50563E-28 | 2.0042E-26 | - |
| *SUS4* | 2124.52621 | 24.92209251 | 0.011730659 | -6.413572127 | 1.60311E-28 | 2.12296E-26 | K00695 |
| *AHP1* | 333.1176872 | 41.10675623 | 0.123400101 | -3.01858452 | 1.7426E-28 | 2.29585E-26 | K14490 |
| *R53* | 199.221378 | 15.15124151 | 0.076052287 | -3.716864548 | 1.87476E-28 | 2.45737E-26 | K00430 |
| *AGAL2* | 649.0602242 | 5.563841355 | 0.00857215 | -6.866127253 | 1.8942E-28 | 2.47025E-26 | K07407 |
| *DIT1* | 7643.569255 | 1804.494402 | 0.236080075 | -2.082651811 | 6.14437E-28 | 7.93241E-26 | - |
| *PYRB3* | 672.1888685 | 119.0761099 | 0.177146804 | -2.496982655 | 7.12357E-28 | 9.15058E-26 | K00609 |
| *CCOAOMT* | 2349.24192 | 522.4864732 | 0.222406415 | -2.168729694 | 8.02308E-28 | 1.02548E-25 | K00588 |
| *PME68* | 101.3056764 | 1.486808367 | 0.014676457 | -6.090352491 | 8.06382E-28 | 1.02558E-25 | - |
| *FANCM* | 478.7336009 | 73.68569476 | 0.153917951 | -2.699766599 | 1.1127E-27 | 1.40129E-25 | K10896 |
| *CSLH1* | 2164.681144 | 287.8831429 | 0.132991015 | -2.910599314 | 1.33905E-27 | 1.67499E-25 | - |
| *LHCB4.1* | 42996.79095 | 10493.31832 | 0.244048872 | -2.034758013 | 1.34307E-27 | 1.67499E-25 | K08915 |
| *CCL7* | 813.0869157 | 155.0185952 | 0.190654396 | -2.390968295 | 1.59202E-27 | 1.97588E-25 | K01904 |
| *XTH9* | 991.3043344 | 197.1767188 | 0.198906342 | -2.32983882 | 1.66259E-27 | 2.05353E-25 | K08235 |
| *FPP4* | 494.0528121 | 79.38347508 | 0.160678116 | -2.637754644 | 1.78669E-27 | 2.19626E-25 | - |
| *uncharacterized protein_15657* | 85.46744471 | 0.295072441 | 0.003452454 | -8.178161997 | 1.83302E-27 | 2.24248E-25 | - |
| *GSTU20* | 1024.797729 | 1.464384476 | 0.00142895 | -9.450829083 | 2.40146E-27 | 2.91018E-25 | K00799 |
| *SUS4* | 1087.289162 | 10.12493975 | 0.009312095 | -6.746678539 | 2.70437E-27 | 3.25177E-25 | K00695 |
| *CCOAOMT* | 290.6448202 | 8.031973638 | 0.027635014 | -5.177358853 | 2.70865E-27 | 3.25177E-25 | K00588 |
| *DRP4C* | 927.4319527 | 184.1024691 | 0.198507792 | -2.332732457 | 3.73749E-27 | 4.42488E-25 | K14754 |
| *CSLA9* | 429.3588163 | 5.32962956 | 0.012412997 | -6.332004745 | 4.62874E-27 | 5.4549E-25 | K13680 |
| *At3g47570* | 206.4253144 | 18.11341209 | 0.087748017 | -3.51048966 | 4.92527E-27 | 5.77786E-25 | - |
| *DEGP7* | 653.4570019 | 40.71517673 | 0.062307354 | -4.004453739 | 8.30323E-27 | 9.69629E-25 | - |
| *ANR* | 1371.886641 | 135.3713521 | 0.098675319 | -3.341166911 | 8.72638E-27 | 1.01443E-24 | K08695 |
| *PATL6* | 767.0086584 | 102.5772583 | 0.133736767 | -2.902531947 | 1.34929E-26 | 1.56147E-24 | - |
| *WAV3* | 164.6417707 | 11.26357254 | 0.068412606 | -3.86959401 | 2.18473E-26 | 2.49293E-24 | - |
| *PSBQ2* | 35140.82323 | 7439.404924 | 0.211702636 | -2.239888859 | 2.193E-26 | 2.49293E-24 | K08901 |
| *GDCST* | 24466.94129 | 6185.340817 | 0.252804008 | -1.983908759 | 2.92016E-26 | 3.30492E-24 | K00605 |
| *ACA7* | 217.8487124 | 21.63141846 | 0.099295599 | -3.332126409 | 4.02818E-26 | 4.49948E-24 | K01674 |
| *CURT1B* | 20393.23785 | 5183.724712 | 0.25418841 | -1.976029842 | 5.4569E-26 | 6.06897E-24 | - |
| *At3g47110* | 490.7291879 | 29.82026195 | 0.060767247 | -4.040562248 | 7.62359E-26 | 8.44214E-24 | - |
| *At3g02645* | 289.3856922 | 17.22591141 | 0.059525788 | -4.070341372 | 1.00121E-25 | 1.09924E-23 | - |
| *PNC1* | 86.88438863 | 0.877898837 | 0.010104218 | -6.628898464 | 1.08958E-25 | 1.19117E-23 | K00430 |
| *MYB1* | 119.6980294 | 4.742675487 | 0.039622001 | -4.657554434 | 1.13114E-25 | 1.23136E-23 | K09422 |
| *TMK1* | 514.1456878 | 91.13204989 | 0.177249468 | -2.496146793 | 1.2512E-25 | 1.35061E-23 | - |
| *RBCS* | 14547.25678 | 1307.57714 | 0.089884791 | -3.47577916 | 1.34601E-25 | 1.44688E-23 | K01602 |
| *MSR1* | 1103.176214 | 77.03259478 | 0.069828006 | -3.840050423 | 1.37407E-25 | 1.47088E-23 | - |
| *AED1* | 234.3972142 | 26.01037394 | 0.110967078 | -3.171796378 | 1.77444E-25 | 1.88376E-23 | - |
| *LAC11* | 531.1790383 | 10.0535404 | 0.018926839 | -5.723422668 | 2.51879E-25 | 2.66297E-23 | K05909 |
| *CURT1A* | 3558.078968 | 887.7443465 | 0.249501024 | -2.002882357 | 3.8463E-25 | 4.03328E-23 | - |
| *CLH2* | 267.4782165 | 12.4196235 | 0.04643228 | -4.428728059 | 4.32433E-25 | 4.51611E-23 | K08099 |
| *Os12g0623900* | 1063.49893 | 34.07806196 | 0.032043344 | -4.96383148 | 4.58437E-25 | 4.74908E-23 | K00549 |
| *PSE1* | 105.7050121 | 3.277383553 | 0.031004997 | -5.011355453 | 5.23563E-25 | 5.40195E-23 | - |
| *LECRK41* | 190.74756 | 17.70806452 | 0.092835077 | -3.429186163 | 5.50367E-25 | 5.65579E-23 | - |
| *CAB6A* | 29627.85371 | 7805.217728 | 0.263441888 | -1.924443337 | 5.83849E-25 | 5.97597E-23 | K08907 |
| *PAE12* | 1947.355409 | 467.6701918 | 0.240156568 | -2.057952829 | 5.86486E-25 | 5.97914E-23 | K19882 |
| *Tmem45b* | 74.41546542 | 0 | 0 | -Inf | 9.55935E-25 | 9.70708E-23 | - |
| *APF2* | 409.6311514 | 68.88719377 | 0.168168836 | -2.572017719 | 9.62262E-25 | 9.73287E-23 | - |
| *PATL3* | 1240.068485 | 284.483599 | 0.229409587 | -2.124002412 | 1.07842E-24 | 1.08226E-22 | - |
| *CYP75A5* | 208.3290455 | 2.059564493 | 0.009886113 | -6.660380882 | 1.22723E-24 | 1.2268E-22 | K13083 |
| *DAD2* | 171.7245944 | 0 | 0 | -Inf | 1.26427E-24 | 1.25893E-22 | - |
| *NPF5.1* | 89.88583182 | 1.473078854 | 0.016388332 | -5.931187165 | 2.0535E-24 | 2.0136E-22 | - |
| *PT1* | 375.189287 | 2.361955419 | 0.00629537 | -7.311493089 | 2.08892E-24 | 2.04054E-22 | K12742 |
| *CBP1* | 656.7163331 | 100.0372968 | 0.15232954 | -2.714732357 | 2.50139E-24 | 2.43421E-22 | K16296 |
| *LHCB5* | 39033.56577 | 8579.915114 | 0.219808643 | -2.185679981 | 2.65208E-24 | 2.57112E-22 | K08916 |
| *NPF5.7* | 316.4677931 | 4.75412165 | 0.01502245 | -6.056736054 | 2.73109E-24 | 2.63776E-22 | K14638 |
| *AATL1* | 1115.480815 | 105.8087984 | 0.09485488 | -3.398134198 | 2.90331E-24 | 2.79359E-22 | - |
| *TIP4-1* | 226.0285217 | 6.228107961 | 0.027554522 | -5.181567069 | 2.92857E-24 | 2.80738E-22 | K09873 |
| *At5g24760* | 2020.030516 | 24.75139575 | 0.012252981 | -6.350723395 | 3.68358E-24 | 3.49307E-22 | K00001 |
| *ABCG36* | 152.9452746 | 11.5618652 | 0.075594785 | -3.725569471 | 4.58722E-24 | 4.30111E-22 | - |
| *Os07g0190000* | 73.27851081 | 0.300107576 | 0.004095438 | -7.931766635 | 5.24087E-24 | 4.86077E-22 | K01662 |
| *At5g48740* | 820.8195973 | 179.2986208 | 0.218438523 | -2.194700786 | 5.83951E-24 | 5.3965E-22 | - |
| *BHLH62* | 324.2070568 | 50.0815582 | 0.154473992 | -2.694564139 | 6.66813E-24 | 6.14017E-22 | - |
| *slr0537* | 2161.787626 | 494.6400114 | 0.228810641 | -2.127773951 | 7.48295E-24 | 6.86587E-22 | - |
| *At3g25290* | 752.3614419 | 162.4056913 | 0.215861263 | -2.211823723 | 9.49509E-24 | 8.68108E-22 | - |
| *ag4* | 279.245721 | 3.87256357 | 0.013867942 | -6.172102442 | 1.25024E-23 | 1.13357E-21 | K18108 |
| *uncharacterized protein_28594* | 464.5263187 | 86.85048866 | 0.186965701 | -2.419154466 | 1.2531E-23 | 1.13357E-21 | - |
| *ROPGAP1* | 1337.542979 | 321.8745256 | 0.240646118 | -2.055014944 | 1.26413E-23 | 1.13953E-21 | - |
| *DAD2* | 67.05449023 | 0 | 0 | -Inf | 1.63044E-23 | 1.4595E-21 | - |
| *At3g61750* | 154.4468935 | 12.44067149 | 0.080549833 | -3.63397459 | 1.64208E-23 | 1.46482E-21 | - |
| *CYP75A5* | 463.1880543 | 8.895674527 | 0.019205319 | -5.702350232 | 1.71006E-23 | 1.52018E-21 | K13083 |
| *LRK10* | 142.859407 | 10.39711986 | 0.072778686 | -3.780340194 | 1.98325E-23 | 1.75695E-21 | - |
| *MAN1* | 118.4087869 | 0 | 0 | -Inf | 2.13804E-23 | 1.88757E-21 | K19355 |
| *RAM1* | 290.8921988 | 43.64387771 | 0.150034542 | -2.736633412 | 3.41523E-23 | 2.98438E-21 | - |
| *AAP6* | 88.56389249 | 2.073294006 | 0.02341015 | -5.416722013 | 3.92165E-23 | 3.40782E-21 | - |
| *ag4* | 152.4752978 | 3.829999138 | 0.025118817 | -5.315087656 | 3.92634E-23 | 3.40782E-21 | - |
| *ALMT9* | 117.7610332 | 6.232235639 | 0.052922732 | -4.239968641 | 3.95906E-23 | 3.42465E-21 | - |
| *LAC11* | 90.49747206 | 2.375684932 | 0.026251396 | -5.251462072 | 5.4825E-23 | 4.71073E-21 | K05909 |
| *lhcA-P4* | 35934.33377 | 9097.322037 | 0.253165179 | -1.981849109 | 6.32724E-23 | 5.41844E-21 | K08910 |
| *tC1* | 22254.08135 | 6228.138492 | 0.279865001 | -1.837197017 | 6.39212E-23 | 5.45581E-21 | K02636 |
| *FLZ5* | 888.1899022 | 37.14456507 | 0.041820522 | -4.579645131 | 6.63524E-23 | 5.64457E-21 | - |
| *A6* | 297.7124053 | 10.06773835 | 0.033816993 | -4.886107805 | 7.56001E-23 | 6.41004E-21 | - |
| *LFNR1* | 48130.34426 | 13569.69409 | 0.28193636 | -1.826558545 | 8.1791E-23 | 6.88948E-21 | K02641 |
| *At1g07700* | 573.6058435 | 119.6236904 | 0.208546848 | -2.261556591 | 8.91175E-23 | 7.48208E-21 | - |
| *At3g02645* | 181.140276 | 18.96616466 | 0.104704294 | -3.25560749 | 9.76977E-23 | 8.17574E-21 | - |
| *At3g02645* | 189.2858887 | 14.85616907 | 0.078485349 | -3.671432817 | 9.99355E-23 | 8.33585E-21 | - |
| *FLA17* | 1293.130681 | 320.8970557 | 0.248155164 | -2.01068562 | 1.0945E-22 | 9.09994E-21 | - |
| *CYP75A5* | 2491.047856 | 65.7113524 | 0.026379 | -5.244466293 | 1.21168E-22 | 1.00417E-20 | K13083 |
| *PCR6* | 172.7804634 | 17.46653424 | 0.10109091 | -3.306274821 | 1.52257E-22 | 1.24972E-20 | - |
| *guaA* | 747.1963261 | 149.2938352 | 0.199805366 | -2.323332769 | 1.62257E-22 | 1.32334E-20 | K01246 |
| *UGT85A1* | 847.3274339 | 197.6664146 | 0.233282208 | -2.099851816 | 1.64975E-22 | 1.34125E-20 | - |
| *At4g27220* | 85.8147374 | 2.054529357 | 0.023941451 | -5.38434558 | 1.8641E-22 | 1.51074E-20 | K13459 |
| *PT1* | 460.0786231 | 90.26003482 | 0.196183935 | -2.349721185 | 1.93839E-22 | 1.56601E-20 | K08176 |
| *CSE* | 146.0647496 | 12.12455105 | 0.083008057 | -3.590604815 | 2.56269E-22 | 2.06389E-20 | - |
| *Xyl1* | 184.7168144 | 1.486808367 | 0.008049123 | -6.956952675 | 2.73473E-22 | 2.19556E-20 | K15920 |
| *PME34* | 620.7268822 | 134.9615441 | 0.217425003 | -2.201410242 | 2.89503E-22 | 2.30982E-20 | - |
| *GSTU20* | 392.9402957 | 0.595180017 | 0.001514683 | -9.366768318 | 3.2243E-22 | 2.55328E-20 | K00799 |
| *NDL2* | 342.8579848 | 51.47550961 | 0.150136534 | -2.735653009 | 3.22999E-22 | 2.55328E-20 | K18266 |
| *OPT7* | 83.90703548 | 0 | 0 | -Inf | 3.98948E-22 | 3.13437E-20 | - |
| *At1g32780* | 76.7084331 | 1.172971278 | 0.015291295 | -6.0311456 | 4.07556E-22 | 3.19223E-20 | K00001 |
| *PT1* | 329.8954264 | 57.22281095 | 0.173457424 | -2.527346503 | 5.85674E-22 | 4.51848E-20 | K08176 |
| *CYP75A1* | 313.8300863 | 2.966298249 | 0.009451924 | -6.725176195 | 7.38211E-22 | 5.66131E-20 | K13083 |
| *NRT2.4* | 815.0050404 | 193.2218756 | 0.23708059 | -2.076550544 | 7.67578E-22 | 5.85159E-20 | K02575 |
| *CYP75A5* | 794.6672966 | 18.63493881 | 0.023449988 | -5.414268986 | 7.85038E-22 | 5.96698E-20 | K13083 |
| *RK13* | 72.74629823 | 0.886593215 | 0.012187468 | -6.358457705 | 8.08098E-22 | 6.12414E-20 | - |
| *S17P* | 16732.49685 | 4849.102047 | 0.289801462 | -1.786863222 | 9.60444E-22 | 7.236E-20 | K01100 |
| *TPS5* | 75.34510401 | 1.195395169 | 0.015865598 | -5.977954245 | 1.00249E-21 | 7.53071E-20 | K15086 |
| *At1g31830* | 138.1782731 | 0 | 0 | -Inf | 1.01907E-21 | 7.63296E-20 | - |
| *NRT2.1* | 122.2103726 | 8.220869215 | 0.067268179 | -3.893931986 | 1.17912E-21 | 8.80606E-20 | K02575 |
| *PAP14* | 554.0665948 | 109.4403001 | 0.197521925 | -2.339915296 | 1.27346E-21 | 9.45565E-20 | - |
| *PSBW* | 17480.14417 | 5093.231176 | 0.291372378 | -1.779063978 | 1.32589E-21 | 9.81652E-20 | K02721 |
| *uncharacterized protein_33491* | 923.9478619 | 226.4806748 | 0.245122787 | -2.02842349 | 1.33917E-21 | 9.8864E-20 | - |
| *At2g39510* | 198.7341735 | 24.80631381 | 0.124821581 | -3.0020607 | 1.44553E-21 | 1.06106E-19 | - |
| *R53* | 705.5532692 | 62.68047544 | 0.088838757 | -3.492666982 | 1.4966E-21 | 1.09542E-19 | K00430 |
| *DIVARICATA* | 816.6998482 | 196.173546 | 0.240202746 | -2.057675452 | 1.57579E-21 | 1.15003E-19 | - |
| *BRG3* | 254.9183244 | 38.77998793 | 0.15212711 | -2.716650823 | 1.58017E-21 | 1.15003E-19 | K19042 |
| *GLYM1* | 21154.51183 | 6219.475186 | 0.294002302 | -1.766100641 | 2.15567E-21 | 1.55565E-19 | K00600 |
| *PGDH1* | 201.1651793 | 25.69791274 | 0.127745333 | -2.968657514 | 2.25398E-21 | 1.62204E-19 | K00058 |
| *uncharacterized protein_13035* | 524.6374225 | 98.76959486 | 0.18826258 | -2.409181822 | 2.44979E-21 | 1.75803E-19 | - |
| *TPS14* | 102.5809998 | 2.365614662 | 0.023060944 | -5.438404638 | 2.50497E-21 | 1.79263E-19 | K04120 |
| *CHLM* | 4660.457719 | 1340.958261 | 0.287731022 | -1.797207322 | 3.55929E-21 | 2.51906E-19 | K03428 |
| *EB1B* | 237.258357 | 35.10138448 | 0.14794583 | -2.756859065 | 3.74049E-21 | 2.64002E-19 | K10436 |
| *NFD4* | 63.379135 | 0.300107576 | 0.004735116 | -7.72238442 | 4.37532E-21 | 3.07121E-19 | - |
| *At1g48100* | 59.14565359 | 0 | 0 | -Inf | 4.57484E-21 | 3.19464E-19 | - |
| *GSVIVT00037159001* | 247.1477776 | 12.97361497 | 0.052493351 | -4.2517215 | 4.89791E-21 | 3.41008E-19 | K00430 |
| *HST* | 298.1648448 | 51.74218615 | 0.173535502 | -2.526697251 | 5.88686E-21 | 4.07653E-19 | K13065 |
| *uncharacterized protein_07790* | 2989.192684 | 851.2427466 | 0.284773461 | -1.812113391 | 5.92236E-21 | 4.09009E-19 | K20283 |
| *NHL1* | 1506.941089 | 405.7068528 | 0.269225424 | -1.893113442 | 5.94995E-21 | 4.09812E-19 | - |
| *CCR1* | 116.0124023 | 6.192422992 | 0.05337725 | -4.227631213 | 6.1724E-21 | 4.23998E-19 | K09753 |
| *At1g48100* | 153.9390298 | 0.591520774 | 0.003842565 | -8.023714508 | 6.53954E-21 | 4.47915E-19 | - |
| *SAUR32* | 259.1947096 | 38.15690986 | 0.147213305 | -2.764020027 | 6.55544E-21 | 4.47915E-19 | K14488 |
| *RD22* | 121.6282283 | 1.181665656 | 0.00971539 | -6.685512395 | 6.95727E-21 | 4.72955E-19 | - |
| *RBOHF* | 242.0122292 | 36.90844097 | 0.152506512 | -2.713057247 | 6.98738E-21 | 4.73649E-19 | K13447 |
| *RPI3* | 2332.566811 | 655.7049426 | 0.281108751 | -1.830799729 | 7.90004E-21 | 5.34105E-19 | K01807 |
| *DAD2* | 95.17174378 | 0 | 0 | -Inf | 1.48433E-20 | 9.9827E-19 | - |
| *At4g16563* | 131.644904 | 11.22332087 | 0.085254503 | -3.552080152 | 1.49103E-20 | 1.00016E-18 | - |
| *At1g12460* | 126.6476454 | 9.164195398 | 0.072359777 | -3.788668225 | 1.56849E-20 | 1.04938E-18 | - |
| *CYP720B1* | 2362.276153 | 83.02730101 | 0.035147161 | -4.830448016 | 1.64734E-20 | 1.09927E-18 | - |
| *MYB61* | 65.01032203 | 0.595180017 | 0.009155162 | -6.771198901 | 1.67422E-20 | 1.11431E-18 | K09422 |
| *PXG* | 194.8685573 | 25.86854152 | 0.132748669 | -2.913230704 | 1.93643E-20 | 1.2855E-18 | K17991 |
| *UCNL* | 181.6706509 | 22.78334174 | 0.12541014 | -2.995274095 | 2.09616E-20 | 1.3773E-18 | - |
| *TPS-mISO1* | 78.75604686 | 0 | 0 | -Inf | 2.50286E-20 | 1.62787E-18 | K04120 |
| *RANBP1C* | 357.5840712 | 48.59971699 | 0.135911303 | -2.879262654 | 2.51073E-20 | 1.62887E-18 | K15306 |
| *GAD* | 462.5562082 | 100.4553305 | 0.217174321 | -2.203074567 | 2.53595E-20 | 1.64109E-18 | K01580 |
| *LPA3* | 951.5966381 | 247.5626097 | 0.260154986 | -1.94255674 | 2.79345E-20 | 1.80318E-18 | - |
| *CB21* | 159390.6819 | 48979.32922 | 0.307291045 | -1.70232237 | 3.20749E-20 | 2.06525E-18 | K08913 |
| *VAB* | 460.8334698 | 100.7528223 | 0.218631738 | -2.19342525 | 3.30411E-20 | 2.12215E-18 | - |
| *RNR1* | 321.1289624 | 13.54593208 | 0.042182219 | -4.567221215 | 3.37628E-20 | 2.16309E-18 | K10807 |
| *FLA7* | 430.1602783 | 27.79916362 | 0.064625129 | -3.951760927 | 3.69541E-20 | 2.34997E-18 | - |
| *uncharacterized protein_18413* | 6487.843784 | 1954.00112 | 0.301178818 | -1.731307788 | 3.84951E-20 | 2.44192E-18 | - |
| *FLZ10* | 311.1710121 | 58.04488427 | 0.186536927 | -2.422466842 | 3.93267E-20 | 2.48853E-18 | - |
| *At5g48900* | 168.1250343 | 1.200430304 | 0.007140104 | -7.129839107 | 4.17837E-20 | 2.63751E-18 | K01728 |
| *uncharacterized protein_21809* | 148.8776905 | 15.69609959 | 0.105429494 | -3.245649575 | 5.10105E-20 | 3.20418E-18 | - |
| *CESA3* | 294.0264039 | 53.44994523 | 0.181786209 | -2.459685342 | 5.65929E-20 | 3.54617E-18 | K10999 |
| *PT1* | 229.6569876 | 1.17663052 | 0.005123426 | -7.608675506 | 6.15298E-20 | 3.84074E-18 | K12742 |
| *GDCSH* | 14544.81011 | 4473.408296 | 0.307560447 | -1.701058115 | 6.15929E-20 | 3.84074E-18 | K02437 |
| *XTHB* | 80.45811957 | 2.651085267 | 0.032949879 | -4.923583043 | 6.68148E-20 | 4.14623E-18 | K08235 |
| *EXPA7* | 441.1227668 | 96.02596486 | 0.217685352 | -2.199683761 | 7.10201E-20 | 4.39657E-18 | - |
| *PSB28* | 1632.125243 | 463.7277113 | 0.284125078 | -1.815401922 | 8.94963E-20 | 5.52704E-18 | K08903 |
| *PGK1* | 15820.41006 | 4908.718959 | 0.310277606 | -1.688368519 | 1.05885E-19 | 6.5079E-18 | K00927 |
| *GXM1* | 1090.652089 | 296.4185233 | 0.271781007 | -1.879483455 | 1.07894E-19 | 6.6155E-18 | K18801 |
| *BAM3* | 6397.948392 | 923.6250928 | 0.144362698 | -2.792230082 | 1.08811E-19 | 6.65586E-18 | K01177 |
| *PSAH* | 879.3389435 | 231.8500379 | 0.263664017 | -1.923227401 | 1.16573E-19 | 7.09686E-18 | K02695 |
| *PM19L* | 123.5702561 | 10.66976841 | 0.086345766 | -3.533730754 | 1.1929E-19 | 7.24509E-18 | - |
| *SRG1* | 184.0188984 | 24.8575432 | 0.135081469 | -2.888098316 | 1.29255E-19 | 7.81341E-18 | - |
| *ufaA1* | 380.6742972 | 80.81083726 | 0.212283408 | -2.235936478 | 2.30791E-19 | 1.37569E-17 | K00574 |
| *uncharacterized protein_04779* | 128.6555562 | 12.07832738 | 0.093881117 | -3.413021176 | 2.79122E-19 | 1.65229E-17 | - |
| *PMIR1* | 3188.212963 | 799.7374944 | 0.25084193 | -1.995149567 | 3.13272E-19 | 1.85018E-17 | - |
| *LAC11* | 97.23985106 | 5.954551954 | 0.061235717 | -4.029482821 | 3.67348E-19 | 2.15469E-17 | K05909 |
| *DCR* | 964.3083081 | 79.67764006 | 0.082626728 | -3.597247655 | 3.72186E-19 | 2.17809E-17 | K19747 |
| *TRXM2* | 3043.218043 | 924.7299529 | 0.303865822 | -1.71849368 | 3.92763E-19 | 2.29329E-17 | K03671 |
| *TUBA* | 3818.438308 | 383.9086687 | 0.100540755 | -3.314147673 | 4.0175E-19 | 2.34044E-17 | K07374 |
| *GSTU19* | 3303.813305 | 38.35681258 | 0.011609861 | -6.428505515 | 4.07138E-19 | 2.36647E-17 | K00799 |
| *SBT5.6* | 419.7901669 | 48.94967807 | 0.116605109 | -3.100297097 | 4.22635E-19 | 2.44547E-17 | - |
| *FRI3* | 188.5306175 | 24.3932767 | 0.129386288 | -2.950243366 | 4.4975E-19 | 2.59652E-17 | K00522 |
| *UGT86A1* | 146.7816303 | 16.89925227 | 0.115131929 | -3.118640107 | 6.51236E-19 | 3.73458E-17 | - |
| *UGT85A8* | 231.749696 | 23.37805332 | 0.100876306 | -3.309340739 | 8.59735E-19 | 4.89744E-17 | - |
| *XTH9* | 1524.14895 | 285.9906948 | 0.187639597 | -2.413963788 | 8.77817E-19 | 4.98938E-17 | K08235 |
| *IRKI* | 88.69009403 | 4.747710622 | 0.053531465 | -4.223469067 | 9.49365E-19 | 5.38414E-17 | - |
| *uncharacterized protein_00340* | 51.64990601 | 0 | 0 | -Inf | 9.67691E-19 | 5.47598E-17 | - |
| *ZOX1* | 781.2729574 | 209.7145017 | 0.268426674 | -1.897400051 | 1.08164E-18 | 6.09397E-17 | K13496 |
| *At3g51990* | 328.467966 | 68.03343635 | 0.207123505 | -2.271436812 | 1.118E-18 | 6.28503E-17 | K04733 |
| *ag4* | 58.80528797 | 0.600215152 | 0.010206823 | -6.614322343 | 1.15929E-18 | 6.48873E-17 | K16086 |
| *ABCB1* | 589.5439675 | 112.6087256 | 0.191009885 | -2.388280791 | 1.40382E-18 | 7.80638E-17 | K05658 |
| *BCP* | 124.7194429 | 12.10350306 | 0.09704584 | -3.365189824 | 1.43629E-18 | 7.95933E-17 | - |
| *FTIP3* | 451.9513638 | 45.40561798 | 0.100465717 | -3.315224811 | 1.43752E-18 | 7.95933E-17 | - |
| *CRK2* | 102.2475902 | 7.42121978 | 0.072580877 | -3.784266698 | 1.53621E-18 | 8.48749E-17 | - |
| *uncharacterized protein_34009* | 599.6685851 | 154.1814164 | 0.257111045 | -1.959536509 | 1.84008E-18 | 1.00796E-16 | - |
| *SGR1* | 161.2914488 | 10.47355435 | 0.064935584 | -3.944846924 | 2.28797E-18 | 1.24534E-16 | - |
| *KPPR* | 33876.29031 | 11114.10308 | 0.328079107 | -1.607884372 | 3.15012E-18 | 1.70021E-16 | K00855 |
| *At1g30440* | 75.69935975 | 2.951192843 | 0.038985704 | -4.680910998 | 3.4837E-18 | 1.86847E-16 | - |
| *RIC10* | 396.2080463 | 25.68283675 | 0.064821593 | -3.947381712 | 4.3667E-18 | 2.32267E-16 | - |
| *TLP1* | 164.4620263 | 22.69411461 | 0.137989998 | -2.8573644 | 4.79587E-18 | 2.52999E-16 | - |
| *ABA2* | 11146.78862 | 3646.3721 | 0.327123105 | -1.612094434 | 4.8174E-18 | 2.53615E-16 | K09838 |
| *uncharacterized protein_47823* | 810.0023901 | 37.28082594 | 0.046025575 | -4.441420437 | 5.66317E-18 | 2.96923E-16 | - |
| *PBL8* | 480.0848275 | 118.5587109 | 0.246953672 | -2.017687676 | 6.27489E-18 | 3.28326E-16 | - |
| *FEI1* | 185.6863717 | 28.77542299 | 0.154967878 | -2.689958893 | 6.36346E-18 | 3.32284E-16 | - |
| *PAE8* | 72.39555998 | 2.673509158 | 0.036929187 | -4.759094696 | 7.73172E-18 | 4.02912E-16 | K19882 |
| *ABCG11* | 121.7697909 | 12.47866923 | 0.102477545 | -3.286620276 | 8.0269E-18 | 4.17447E-16 | - |
| *TIC55* | 759.1059322 | 210.6843115 | 0.277542702 | -1.849218337 | 8.98207E-18 | 4.66179E-16 | - |
| *PSAF* | 31063.01744 | 10366.30181 | 0.333718443 | -1.583296676 | 1.00264E-17 | 5.19331E-16 | K02694 |
| *CYP75A5* | 383.7977912 | 10.64324626 | 0.02773139 | -5.172336255 | 1.08763E-17 | 5.61092E-16 | K13083 |
| *TBL6* | 267.7096803 | 10.36327922 | 0.038710887 | -4.691116819 | 1.28746E-17 | 6.62846E-16 | - |
| *NRT3.1* | 101.1122576 | 4.413293969 | 0.043647467 | -4.517958243 | 1.48235E-17 | 7.60143E-16 | - |
| *CYP92C6* | 158.5628663 | 22.26731858 | 0.140432114 | -2.832055202 | 1.5515E-17 | 7.92435E-16 | - |
| *uncharacterized protein_48451* | 88.04388323 | 5.629268702 | 0.063937079 | -3.967203358 | 1.99298E-17 | 1.01189E-15 | - |
| *uncharacterized protein_42926* | 582.5058282 | 48.75577677 | 0.083700067 | -3.578627414 | 2.01821E-17 | 1.02268E-15 | - |
| *CTL1* | 3893.288859 | 1180.456818 | 0.303202989 | -1.72164412 | 2.05651E-17 | 1.03877E-15 | - |
| *PLGG1* | 3503.975564 | 1141.118318 | 0.325663892 | -1.618544327 | 2.05806E-17 | 1.03877E-15 | - |
| *uncharacterized protein_38043* | 4840.00164 | 1449.610789 | 0.29950626 | -1.739341938 | 2.24938E-17 | 1.12869E-15 | - |
| *CB2A* | 56975.28703 | 18017.27969 | 0.316229731 | -1.660955083 | 2.63547E-17 | 1.31984E-15 | K08912 |
| *FLA11* | 433.7734554 | 33.73492152 | 0.07777083 | -3.68462706 | 2.93868E-17 | 1.46598E-15 | - |
| *GSTU20* | 599.718988 | 0.88155808 | 0.001469952 | -9.410015314 | 3.06777E-17 | 1.52445E-15 | K00799 |
| *CYP71AU50* | 806.9382512 | 233.4911206 | 0.289354384 | -1.789090593 | 3.69511E-17 | 1.8256E-15 | - |
| *ALDH3F1* | 326.8351139 | 73.49754128 | 0.224876515 | -2.152795096 | 4.1246E-17 | 2.02223E-15 | K00128 |
| *PPH* | 766.7331434 | 219.952878 | 0.286870184 | -1.801530066 | 4.35512E-17 | 2.12989E-15 | - |
| *At4g17486* | 74.98054756 | 3.541337725 | 0.047230086 | -4.404150021 | 4.36075E-17 | 2.12989E-15 | - |
| *At1g67720* | 122.3694077 | 13.63331488 | 0.111411137 | -3.166034635 | 4.44184E-17 | 2.16537E-15 | - |
| *mhpC* | 175.824969 | 27.75062719 | 0.15783098 | -2.66354768 | 4.59907E-17 | 2.23579E-15 | - |
| *At2g26730* | 639.889666 | 177.1100165 | 0.276782117 | -1.853177361 | 4.60369E-17 | 2.23579E-15 | - |
| *CESA9* | 61.80841798 | 0.586485639 | 0.009488766 | -6.719563747 | 4.73919E-17 | 2.29726E-15 | K10999 |
| *uncharacterized protein_50037* | 4322.581341 | 1379.579632 | 0.319156431 | -1.647664378 | 5.20275E-17 | 2.51248E-15 | - |
| *APS1* | 712.3483062 | 15.0967919 | 0.021192992 | -5.560268915 | 5.82827E-17 | 2.80401E-15 | - |
| *At4g01130* | 66.9974516 | 2.356012826 | 0.035165708 | -4.829686922 | 6.38757E-17 | 3.05592E-15 | - |
| *GGPS1* | 396.968464 | 17.47613607 | 0.044023991 | -4.505566252 | 6.60073E-17 | 3.15203E-15 | K13789 |
| *GDI1* | 459.4510245 | 117.914019 | 0.256641106 | -1.962175831 | 6.81571E-17 | 3.24865E-15 | K12462 |
| *RGI1* | 52.84041063 | 0.591520774 | 0.011194477 | -6.481069031 | 6.97589E-17 | 3.31885E-15 | - |
| *At1g32780* | 70.38251679 | 2.970864949 | 0.042210269 | -4.566262175 | 7.24955E-17 | 3.44267E-15 | K00001 |
| *At1g12760* | 75.70800961 | 3.832750923 | 0.050625435 | -4.303993789 | 7.62071E-17 | 3.61224E-15 | - |
| *FPP4* | 261.9315393 | 10.37882365 | 0.039624185 | -4.657474945 | 7.83607E-17 | 3.70748E-15 | - |
| *H1* | 518.0920943 | 54.93859793 | 0.106040217 | -3.237316572 | 8.59887E-17 | 4.06091E-15 | K11275 |
| *XTH8* | 902.5933735 | 64.31458123 | 0.071255322 | -3.810858423 | 8.74648E-17 | 4.12304E-15 | K08235 |
| *ATS3A* | 197.3723944 | 34.71389411 | 0.175880189 | -2.507335104 | 9.30597E-17 | 4.37074E-15 | - |
| *BGAL3* | 276.7330825 | 22.45761947 | 0.081152637 | -3.62321821 | 9.75416E-17 | 4.57289E-15 | - |
| *SAMDC* | 741.2876313 | 14.15850085 | 0.019099875 | -5.710293019 | 1.01653E-16 | 4.75693E-15 | K01611 |
| *uncharacterized protein_07436* | 52.24775693 | 0.591520774 | 0.011321458 | -6.464796454 | 1.12254E-16 | 5.2435E-15 | - |
| *CHX19* | 290.371952 | 63.73403818 | 0.219491028 | -2.187766127 | 1.1542E-16 | 5.38161E-15 | - |
| *tyrP-A* | 510.1334485 | 136.6963912 | 0.267962024 | -1.899899542 | 1.16022E-16 | 5.39985E-15 | K03834 |
| *GGPS1* | 104.7287435 | 10.14095261 | 0.096830653 | -3.368392367 | 1.37743E-16 | 6.38764E-15 | K13789 |
| *SRG1* | 127.4132603 | 15.71345893 | 0.123326716 | -3.019442737 | 1.48337E-16 | 6.86655E-15 | - |
| *MYB4* | 56.86478133 | 1.200430304 | 0.02111026 | -5.565911859 | 1.55709E-16 | 7.17105E-15 | K09422 |
| *GT11* | 454.2912852 | 118.3216408 | 0.260453248 | -1.940903666 | 1.55753E-16 | 7.17105E-15 | - |
| *uncharacterized protein_15377* | 274.4902122 | 41.38075126 | 0.150754925 | -2.729722964 | 1.5614E-16 | 7.17601E-15 | - |
| *DAD2* | 115.4888898 | 0 | 0 | -Inf | 1.69525E-16 | 7.77727E-15 | - |
| *ZIF1* | 217.5062572 | 12.68082588 | 0.05830097 | -4.100336291 | 1.76901E-16 | 8.08678E-15 | - |
| *At5g64970* | 729.5012735 | 210.8921959 | 0.289090922 | -1.790404787 | 2.00257E-16 | 9.08976E-15 | - |
| *CATHB1* | 1723.292095 | 561.6699938 | 0.325928492 | -1.61737262 | 2.14912E-16 | 9.73775E-15 | K01363 |
| *WTF1* | 1558.000111 | 502.9957858 | 0.32284708 | -1.631077118 | 2.16756E-16 | 9.80401E-15 | - |
| *uncharacterized protein_33396* | 1737.823332 | 566.1493012 | 0.3257807 | -1.618026958 | 2.24332E-16 | 1.01289E-14 | - |
| *pds* | 1031.527735 | 319.4860663 | 0.309721257 | -1.690957695 | 2.26108E-16 | 1.01911E-14 | - |
| *At5g18840* | 243.9236284 | 5.867608174 | 0.024055104 | -5.377513171 | 2.93915E-16 | 1.3155E-14 | K08145 |
| *MYB123* | 238.972705 | 49.19224261 | 0.205848792 | -2.280343113 | 3.12028E-16 | 1.39172E-14 | K09422 |
| *LAC17* | 71.3328647 | 3.54637286 | 0.049715834 | -4.330150781 | 3.50201E-16 | 1.55927E-14 | K05909 |
| *uncharacterized protein_13825* | 43.74630964 | 0 | 0 | -Inf | 3.72935E-16 | 1.65191E-14 | - |
| *SLAC1* | 145.3995304 | 21.3450404 | 0.146802678 | -2.768049812 | 4.15974E-16 | 1.82993E-14 | - |
| *EO* | 1461.066856 | 474.6698472 | 0.324878937 | -1.622025883 | 4.31973E-16 | 1.89706E-14 | K18980 |
| *uncharacterized protein_36021* | 98.20064354 | 9.136297349 | 0.093037042 | -3.426050969 | 4.73885E-16 | 2.07403E-14 | - |
| *ANR* | 1637.949695 | 248.2004922 | 0.151531206 | -2.722313167 | 5.08843E-16 | 2.21571E-14 | K08695 |
| *PME34* | 43.25207722 | 0 | 0 | -Inf | 5.11644E-16 | 2.22414E-14 | - |
| *CA1P* | 1089.040968 | 346.8735687 | 0.318512874 | -1.650576408 | 6.37974E-16 | 2.75929E-14 | K15634 |
| *RGI1* | 100.0910457 | 9.735136609 | 0.097262812 | -3.361967881 | 6.45035E-16 | 2.78514E-14 | - |
| *AMT3-1* | 2447.031568 | 830.1827468 | 0.339261151 | -1.559531858 | 6.97982E-16 | 3.00366E-14 | K03320 |
| *ATHB-5* | 526.799653 | 148.0967519 | 0.281125379 | -1.830714396 | 7.11493E-16 | 3.05668E-14 | K09338 |
| *LAC17* | 141.9035671 | 4.169011903 | 0.02937919 | -5.089061554 | 7.91765E-16 | 3.39021E-14 | K05909 |
| *CSP41B* | 3207.842875 | 1018.638496 | 0.317546256 | -1.654961333 | 8.3511E-16 | 3.56392E-14 | - |
| *SCPL31* | 109.7728838 | 0.590144882 | 0.005376053 | -7.539236826 | 8.44158E-16 | 3.59656E-14 | K16297 |
| *SRG1* | 434.3528876 | 40.23355089 | 0.092628717 | -3.432396652 | 8.87663E-16 | 3.77566E-14 | - |
| *3BETAHSD/D2* | 77.38579069 | 5.014416578 | 0.06479764 | -3.947914928 | 9.24155E-16 | 3.92437E-14 | K07748 |
| *AGD12* | 67.46929498 | 0.885217322 | 0.013120299 | -6.252055593 | 1.00534E-15 | 4.26207E-14 | K12486 |
| *uncharacterized protein_26134* | 259.3780005 | 57.5732019 | 0.221966403 | -2.171586766 | 1.11556E-15 | 4.71377E-14 | - |
| *At2g05160* | 52.63305865 | 0.300107576 | 0.005701884 | -7.454345686 | 1.16214E-15 | 4.90254E-14 | - |
| *CRK2* | 626.7574186 | 90.35576122 | 0.144163848 | -2.79421867 | 1.1658E-15 | 4.90995E-14 | - |
| *GUX1* | 79.83262517 | 5.608689138 | 0.070255602 | -3.831242924 | 1.31164E-15 | 5.48817E-14 | - |
| *CDS4* | 1187.750608 | 386.6716198 | 0.325549503 | -1.61905116 | 1.322E-15 | 5.52252E-14 | K00981 |
| *SLC4A1* | 55.86608062 | 1.465760368 | 0.026237036 | -5.252251442 | 1.40685E-15 | 5.86742E-14 | - |
| *ALF1* | 1670.942371 | 536.9581556 | 0.321350494 | -1.637780407 | 1.47455E-15 | 6.12989E-14 | K01623 |
| *PXN* | 633.0387661 | 188.2199394 | 0.297327667 | -1.74987438 | 1.49503E-15 | 6.20498E-14 | K13354 |
| *Os07g0190000* | 328.4900166 | 25.77343977 | 0.078460344 | -3.671892525 | 1.56673E-15 | 6.49206E-14 | K01662 |
| *CYP75A5* | 1723.717546 | 35.7624897 | 0.020747303 | -5.590932402 | 1.64175E-15 | 6.79199E-14 | K13083 |
| *algC* | 956.2548796 | 206.8028865 | 0.216263353 | -2.209138882 | 1.73281E-15 | 7.14567E-14 | - |
| *Prcp* | 136.7683352 | 20.11715107 | 0.147089244 | -2.76523634 | 1.76409E-15 | 7.263E-14 | K01285 |
| *SBE1* | 991.7371587 | 317.6254677 | 0.320271823 | -1.642631218 | 1.80226E-15 | 7.40828E-14 | K00700 |
| *CCOAOMT* | 361.1018263 | 93.19843502 | 0.25809461 | -1.954028082 | 1.81637E-15 | 7.45439E-14 | K00588 |
| *CSLE1* | 708.4706209 | 66.8944031 | 0.094420857 | -3.404750614 | 2.16854E-15 | 8.85722E-14 | - |
| *COB* | 884.9951882 | 34.99255322 | 0.039539823 | -4.660549772 | 2.57997E-15 | 1.04876E-13 | - |
| *O10* | 384.0059491 | 101.8677141 | 0.265276396 | -1.914431785 | 2.66389E-15 | 1.08117E-13 | - |
| *PLD1* | 783.621101 | 64.85847302 | 0.082767645 | -3.594789283 | 3.03822E-15 | 1.23115E-13 | K01115 |
| *EXPA8* | 44.31132695 | 0.295072441 | 0.006659075 | -7.230462538 | 3.46386E-15 | 1.39702E-13 | - |
| *At1g67720* | 198.8326596 | 39.36868894 | 0.197999106 | -2.336434175 | 3.58303E-15 | 1.44282E-13 | - |
| *CSLA9* | 202.8273763 | 7.992600013 | 0.039405923 | -4.665443689 | 3.90424E-15 | 1.5648E-13 | K13680 |
| *At5g18840* | 174.9404823 | 32.21655586 | 0.184157237 | -2.440990001 | 4.08399E-15 | 1.63429E-13 | K08145 |
| *COL16* | 498.5480951 | 63.66821038 | 0.127707258 | -2.969087571 | 4.14763E-15 | 1.65718E-13 | - |
| *uncharacterized protein_45787* | 2796.529341 | 987.174942 | 0.35300003 | -1.502259788 | 4.2702E-15 | 1.70351E-13 | - |
| *MAP65-1* | 146.8784225 | 9.208604158 | 0.062695418 | -3.99549617 | 4.82425E-15 | 1.92155E-13 | K16732 |
| *At1g67720* | 214.7383673 | 45.3086719 | 0.210994768 | -2.244720873 | 5.10714E-15 | 2.03108E-13 | - |
| *DTX40* | 212.3468416 | 44.5482987 | 0.209790258 | -2.252980409 | 5.65091E-15 | 2.24039E-13 | K03327 |
| *UGT85A8* | 260.9053172 | 60.69637 | 0.232637535 | -2.103844204 | 5.78586E-15 | 2.29036E-13 | - |
| *PCR6* | 60.04435408 | 0.600215152 | 0.009996196 | -6.644405047 | 6.08444E-15 | 2.39748E-13 | - |
| *uncharacterized protein_39336* | 6521.539465 | 2154.038636 | 0.330296036 | -1.598168438 | 6.423E-15 | 2.527E-13 | - |
| *CESA5* | 1698.648802 | 212.599516 | 0.125158017 | -2.998177387 | 6.77237E-15 | 2.66038E-13 | K10999 |
| *CYP720B2* | 89.73035722 | 0.89162835 | 0.009936752 | -6.653009855 | 7.10373E-15 | 2.78629E-13 | - |
| *Tmem45b* | 364.9667875 | 1.500537881 | 0.004111437 | -7.926141633 | 7.77216E-15 | 3.04382E-13 | - |
| *gmppA* | 195.8567775 | 14.19922095 | 0.072497981 | -3.78591537 | 9.85216E-15 | 3.82923E-13 | K00966 |
| *BHLH80* | 746.0025051 | 237.8037027 | 0.318770649 | -1.649409297 | 1.02104E-14 | 3.96246E-13 | - |
| *CYCD2-2* | 306.5803258 | 77.73335642 | 0.253549722 | -1.979659402 | 1.03267E-14 | 4.00154E-13 | K18810 |
| *UGT73B4* | 620.9779581 | 157.2340926 | 0.253203983 | -1.981627993 | 1.10142E-14 | 4.23601E-13 | - |
| *At1g28650* | 121.9441224 | 2.37202569 | 0.019451743 | -5.683956778 | 1.15236E-14 | 4.42531E-13 | - |
| *LOX1.5* | 48.07483162 | 0.900322728 | 0.018727527 | -5.738695754 | 1.20799E-14 | 4.61821E-13 | K15718 |
| *PXC3* | 192.2438379 | 39.24478169 | 0.204140648 | -2.292364616 | 1.36419E-14 | 5.1999E-13 | - |
| *uncharacterized protein_33341* | 131.7796835 | 20.34266848 | 0.154368776 | -2.695547122 | 1.43905E-14 | 5.46904E-13 | - |
| *PSBP* | 25454.88305 | 9549.052664 | 0.375136379 | -1.414512918 | 1.46817E-14 | 5.57144E-13 | K02717 |
| *At5g57670* | 92.29444858 | 3.249924527 | 0.035212568 | -4.827765754 | 1.47438E-14 | 5.57945E-13 | - |
| *ARC5* | 4893.440352 | 1803.218563 | 0.368497097 | -1.440274842 | 1.55596E-14 | 5.86991E-13 | - |
| *EO* | 9185.239713 | 3428.317504 | 0.373242029 | -1.421816646 | 1.59856E-14 | 6.02051E-13 | K18980 |
| *RPI3* | 3598.240103 | 1313.801985 | 0.36512349 | -1.453543607 | 1.60056E-14 | 6.02051E-13 | K01807 |
| *MMAR_1059* | 1175.51603 | 401.8491778 | 0.341849169 | -1.548568177 | 1.72158E-14 | 6.45682E-13 | - |
| *F16P2* | 4456.309366 | 831.5160802 | 0.186592988 | -2.422033323 | 1.82318E-14 | 6.8279E-13 | K03841 |
| *uncharacterized protein_20873* | 198.2259071 | 41.72433072 | 0.210488787 | -2.248184714 | 1.86497E-14 | 6.95397E-13 | - |
| *PPH1* | 682.2697186 | 217.5259931 | 0.318826979 | -1.649154381 | 2.11699E-14 | 7.85946E-13 | - |
| *AKT1* | 431.7678357 | 124.7406337 | 0.28890673 | -1.791324281 | 2.1574E-14 | 7.99794E-13 | - |
| *BGAL8* | 469.4224835 | 43.14295996 | 0.091906463 | -3.443689877 | 2.21367E-14 | 8.19471E-13 | - |
| *PNC1* | 424.9507446 | 1.200430304 | 0.002824869 | -8.467600175 | 2.28851E-14 | 8.4596E-13 | K00430 |
| *CRRSP38* | 680.4262636 | 68.34408263 | 0.10044304 | -3.315550491 | 2.35553E-14 | 8.69481E-13 | - |
| *CYP720B2* | 239.0366925 | 39.11121378 | 0.163620126 | -2.611577877 | 2.65977E-14 | 9.78106E-13 | - |
| *7-Oct* | 771.0417944 | 99.00336764 | 0.128402077 | -2.961259559 | 2.8894E-14 | 1.05592E-12 | - |
| *RPL11* | 2746.176177 | 1001.930015 | 0.364845498 | -1.454642443 | 2.91575E-14 | 1.06404E-12 | K02867 |
| *PAT22* | 415.4214314 | 64.77025074 | 0.155914563 | -2.681172412 | 3.11992E-14 | 1.13532E-12 | K20027 |
| *CIPK32* | 688.2023774 | 167.9539496 | 0.244047326 | -2.034767153 | 3.27048E-14 | 1.18675E-12 | K07198 |
| *CB21* | 113019.7394 | 18766.7622 | 0.166048535 | -2.590323097 | 3.49648E-14 | 1.2634E-12 | K08913 |
| *At5g56590* | 319.8471075 | 85.51645188 | 0.267366657 | -1.903108535 | 3.83179E-14 | 1.38262E-12 | - |
| *uncharacterized protein_08399* | 41.04901774 | 0.300107576 | 0.007310956 | -7.09572415 | 3.90922E-14 | 1.40661E-12 | - |
| *At5g03795* | 105.3819173 | 1.491843502 | 0.014156542 | -6.142387322 | 4.29346E-14 | 1.53626E-12 | - |
| *PT1* | 118.1825274 | 1.771810537 | 0.014992153 | -6.059648605 | 4.93625E-14 | 1.75891E-12 | K12742 |
| *UTR4* | 238.4871513 | 56.70715883 | 0.237778675 | -2.072308761 | 4.98874E-14 | 1.77515E-12 | K15277 |
| *MOT2* | 883.2878356 | 298.3734136 | 0.337798622 | -1.565764652 | 5.12715E-14 | 1.81936E-12 | - |
| *FTSZ2-1* | 3661.840395 | 1365.816137 | 0.372986255 | -1.422805627 | 5.39608E-14 | 1.91215E-12 | K03531 |
| *DIT2* | 4183.875656 | 1568.956285 | 0.375000697 | -1.415034819 | 5.59881E-14 | 1.97853E-12 | K03319 |
| *CSLA9* | 48.15084969 | 1.195395169 | 0.024826045 | -5.332001734 | 5.64241E-14 | 1.9912E-12 | K13680 |
| *BRN2* | 102.3200188 | 10.63408344 | 0.103929647 | -3.266320838 | 5.88936E-14 | 2.0755E-12 | - |
| *At3g46100* | 480.6755137 | 146.1250386 | 0.303999339 | -1.717859906 | 6.12399E-14 | 2.15227E-12 | K01892 |
| *CIPK26* | 80.00204004 | 7.41893643 | 0.092734341 | -3.430752505 | 6.23914E-14 | 2.18676E-12 | K07198 |
| *QWRF4* | 42.91163968 | 0.300107576 | 0.006993617 | -7.159745479 | 6.35909E-14 | 2.22577E-12 | - |
| *OPT4* | 82.39859025 | 7.954163258 | 0.096532759 | -3.372837576 | 6.95602E-14 | 2.42808E-12 | - |
| *uncharacterized protein_04893* | 1287.747981 | 329.6426902 | 0.255983853 | -1.965875283 | 7.40218E-14 | 2.57333E-12 | - |
| *SLAC1* | 781.3584383 | 262.579532 | 0.336055156 | -1.573230055 | 8.855E-14 | 3.06183E-12 | - |
| *uncharacterized protein_26016* | 80.95056437 | 5.659479513 | 0.069912786 | -3.838299855 | 9.26845E-14 | 3.19619E-12 | - |
| *CHS* | 204.7556867 | 13.38906223 | 0.065390429 | -3.934776698 | 9.49475E-14 | 3.26546E-12 | K00660 |
| *EFR* | 266.5003112 | 12.68908124 | 0.047613758 | -4.392477699 | 1.01001E-13 | 3.45976E-12 | K13420 |
| *LOX1.1* | 59.81612006 | 3.236195013 | 0.054102389 | -4.208163882 | 1.01167E-13 | 3.46081E-12 | K15718 |
| *RPL27* | 5004.742847 | 1905.584415 | 0.38075571 | -1.393062424 | 1.01877E-13 | 3.48048E-12 | K02899 |
| *IRK* | 501.5693551 | 112.7313452 | 0.224757243 | -2.153560489 | 1.04878E-13 | 3.57824E-12 | - |
| *At4g02290* | 74.86486252 | 1.795610321 | 0.023984687 | -5.381742556 | 1.0752E-13 | 3.65867E-12 | - |
| *MAN6* | 203.3097002 | 46.27717376 | 0.227619114 | -2.135306386 | 1.30397E-13 | 4.41958E-12 | K19355 |
| *LPS* | 212.4551751 | 0.295072441 | 0.001388869 | -9.491873589 | 1.4256E-13 | 4.80645E-12 | K04120 |
| *SMXL3* | 171.2165726 | 35.499882 | 0.207339053 | -2.269936217 | 1.51857E-13 | 5.09067E-12 | - |
| *MOT2* | 89.76477684 | 7.158641501 | 0.079748892 | -3.648391713 | 1.51981E-13 | 5.09067E-12 | - |
| *At5g10770* | 129.6458588 | 21.80752139 | 0.168208392 | -2.571678414 | 1.54529E-13 | 5.16928E-12 | - |
| *PGLP1A* | 2441.68471 | 916.1887493 | 0.375228114 | -1.414160168 | 1.76029E-13 | 5.8768E-12 | K19269 |
| *GAUT7* | 539.641394 | 172.7453178 | 0.320111318 | -1.643354409 | 1.79992E-13 | 5.99764E-12 | K13648 |
| *NAT2* | 404.3071945 | 120.7330787 | 0.298617191 | -1.743630874 | 1.93897E-13 | 6.43593E-12 | K14611 |
| *BGLU13* | 44.02447538 | 0.895287593 | 0.020336133 | -5.619810809 | 2.01751E-13 | 6.67936E-12 | K01188 |
| *QRT3* | 366.7086858 | 61.43898339 | 0.167541664 | -2.577408188 | 2.04381E-13 | 6.75771E-12 | - |
| *PIP2-8* | 5651.781087 | 1131.430974 | 0.200190162 | -2.320557014 | 2.15871E-13 | 7.11019E-12 | K09872 |
| *RPS20* | 2228.498434 | 837.8336779 | 0.375963324 | -1.411336165 | 2.22458E-13 | 7.29906E-12 | K02968 |
| *T5AT* | 80.83314946 | 0 | 0 | -Inf | 2.47513E-13 | 8.09016E-12 | K15400 |
| *LAC6* | 35.19299916 | 0 | 0 | -Inf | 2.50136E-13 | 8.16549E-12 | K05909 |
| *comta* | 123.3766863 | 20.39802556 | 0.165331281 | -2.596568386 | 2.53344E-13 | 8.25972E-12 | K00545 |
| *PLP7* | 194.3693652 | 44.22311284 | 0.227521003 | -2.135928368 | 2.56059E-13 | 8.33765E-12 | - |
| *CBP1* | 77.09391464 | 7.397419996 | 0.095953358 | -3.381522893 | 2.58114E-13 | 8.38333E-12 | K16296 |
| *D27* | 346.7220362 | 100.1694595 | 0.28890422 | -1.791336817 | 2.64372E-13 | 8.57575E-12 | - |
| *uncharacterized protein_07999* | 185.2525733 | 15.0652346 | 0.081322674 | -3.620198538 | 2.6782E-13 | 8.67662E-12 | - |
| *Os07g0190000* | 397.2636346 | 19.58239267 | 0.049293192 | -4.342467779 | 2.79707E-13 | 9.03892E-12 | K01662 |
| *SK1* | 725.4714468 | 246.9083748 | 0.340341961 | -1.554943062 | 2.93911E-13 | 9.46351E-12 | K00891 |
| *PLR_Tp1* | 71.70780088 | 0.600215152 | 0.008370291 | -6.900506524 | 2.9395E-13 | 9.46351E-12 | - |
| *HPR-A* | 16783.7533 | 6114.821175 | 0.364329782 | -1.456683164 | 2.9859E-13 | 9.58885E-12 | K15893 |
| *GSTF9* | 521.2506199 | 141.7534513 | 0.271948744 | -1.878593333 | 3.0879E-13 | 9.90404E-12 | K00799 |
| *PPH* | 2167.515439 | 818.3774778 | 0.377564774 | -1.405203923 | 3.13876E-13 | 1.00421E-11 | - |
| *OPT4* | 99.98046419 | 13.55140623 | 0.135540541 | -2.883203657 | 3.28996E-13 | 1.04997E-11 | - |
| *MIOX1* | 82.84107446 | 2.651992725 | 0.032013017 | -4.965197543 | 3.373E-13 | 1.07427E-11 | K00469 |
| *HSP90-1* | 758.761495 | 261.0984035 | 0.344111299 | -1.539052832 | 3.37449E-13 | 1.07427E-11 | K04079 |
| *FAD7* | 2687.376787 | 1026.27626 | 0.381887745 | -1.388779473 | 3.67881E-13 | 1.16538E-11 | K10257 |
| *Os04g0590900* | 144.3590874 | 27.44551389 | 0.190119752 | -2.395019671 | 3.7506E-13 | 1.18666E-11 | - |
| *NRT3.1* | 157.3843492 | 31.91551141 | 0.202787072 | -2.301962411 | 3.8478E-13 | 1.21442E-11 | - |
| *MAP65-6* | 621.7759102 | 207.3213895 | 0.333434258 | -1.584525755 | 3.91737E-13 | 1.23486E-11 | K16732 |
| *CSPL5* | 92.77834432 | 9.713620175 | 0.104697063 | -3.255707121 | 3.94791E-13 | 1.24211E-11 | - |
| *slr0575* | 5262.738064 | 2061.001245 | 0.391621475 | -1.352468215 | 4.45443E-13 | 1.39559E-11 | - |
| *SYNC1* | 2857.754229 | 894.1805075 | 0.312896224 | -1.676243846 | 4.48E-13 | 1.4019E-11 | K01893 |
| *TPS-LAS2* | 62.94882523 | 0 | 0 | -Inf | 4.80978E-13 | 1.50143E-11 | K16086 |
| *NRT3.2* | 68.85850172 | 3.819021409 | 0.055461872 | -4.172359877 | 5.20234E-13 | 1.62201E-11 | - |
| *EXPA10* | 44.85218229 | 1.186700791 | 0.026458039 | -5.24015005 | 5.33948E-13 | 1.66275E-11 | - |
| *NIP6-1* | 124.3375219 | 21.50741382 | 0.172976053 | -2.531355768 | 6.2678E-13 | 1.93774E-11 | K09874 |
| *At2g42960* | 71.64735266 | 6.498941595 | 0.090707351 | -3.462636711 | 6.32222E-13 | 1.95221E-11 | - |
| *MAN1* | 38.9131826 | 0 | 0 | -Inf | 6.53372E-13 | 2.0151E-11 | K19355 |
| *HOS3* | 109.6293537 | 13.00748503 | 0.118649655 | -3.075220186 | 6.70044E-13 | 2.06404E-11 | - |
| *BPA1* | 839.3987298 | 183.4888478 | 0.218595575 | -2.1936639 | 7.04377E-13 | 2.16461E-11 | - |
| *LHCA3* | 39775.81195 | 15997.24418 | 0.402185232 | -1.314067985 | 7.16684E-13 | 2.1998E-11 | K08909 |
| *At3g47110* | 70.00414215 | 6.24458926 | 0.08920314 | -3.486761703 | 7.21514E-13 | 2.21198E-11 | - |
| *ALMT9* | 71.22755939 | 6.501693381 | 0.091280586 | -3.453548132 | 7.65105E-13 | 2.34283E-11 | - |
| *TKTC* | 38235.41917 | 15384.99028 | 0.402375353 | -1.313386158 | 7.84587E-13 | 2.39677E-11 | K00615 |
| *PLR_Tp2* | 60.60937139 | 4.161224983 | 0.068656462 | -3.864460683 | 8.35229E-13 | 2.54844E-11 | - |
| *CYP75A5* | 49.45362975 | 1.191735926 | 0.024098048 | -5.374939921 | 9.48215E-13 | 2.8866E-11 | K13083 |
| *OsI_00941* | 646.2450853 | 221.5783189 | 0.342870412 | -1.544264681 | 9.56266E-13 | 2.90739E-11 | - |
| *UGT86A1* | 210.8348571 | 52.16532293 | 0.247422668 | -2.014950412 | 1.03806E-12 | 3.1449E-11 | - |
| *EFL2* | 203.9940568 | 49.76634522 | 0.24395978 | -2.035284775 | 1.04282E-12 | 3.1556E-11 | - |
| *uncharacterized protein_40390* | 728.3641897 | 255.0689583 | 0.35019426 | -1.513772659 | 1.04907E-12 | 3.16706E-11 | - |
| *uncharacterized protein_09062* | 1952.115434 | 750.8508429 | 0.384634448 | -1.378440117 | 1.09438E-12 | 3.28839E-11 | - |
| *ATPD* | 9289.195179 | 3726.106886 | 0.401122682 | -1.317884547 | 1.09675E-12 | 3.29165E-11 | K02113 |
| *TBR* | 74.53479762 | 0.595180017 | 0.007985264 | -6.968444226 | 1.11552E-12 | 3.34408E-11 | - |
| *DRT100* | 39.23459764 | 0.590144882 | 0.015041441 | -6.054913413 | 1.12026E-12 | 3.35437E-11 | - |
| *LRR1* | 65.35244638 | 5.305829777 | 0.081187929 | -3.622590946 | 1.17472E-12 | 3.50926E-11 | - |
| *RBCS* | 105.3180595 | 16.33290717 | 0.155081733 | -2.688899334 | 1.27063E-12 | 3.78698E-11 | K01602 |
| *DCOR* | 187.3832254 | 5.668173891 | 0.0302491 | -5.04696398 | 1.36569E-12 | 4.05618E-11 | K01581 |
| *Sb10g008780* | 1486.820415 | 564.0211277 | 0.379347177 | -1.398409292 | 1.43753E-12 | 4.26461E-11 | K01872 |
| *CSE* | 215.8736063 | 54.74554526 | 0.253599994 | -1.979373383 | 1.45753E-12 | 4.31895E-11 | K18368 |
| *At5g07050* | 63.64530613 | 5.047818198 | 0.079311712 | -3.656322262 | 1.52167E-12 | 4.49864E-11 | - |
| *PLR_Tp2* | 53.68545323 | 2.94981695 | 0.054946299 | -4.185833888 | 1.59047E-12 | 4.69664E-11 | - |
| *NRT3.2* | 1494.976521 | 70.82813038 | 0.04737742 | -4.399656556 | 1.67155E-12 | 4.9304E-11 | - |
| *SWEET3B* | 392.4039289 | 98.11486212 | 0.250035371 | -1.999795893 | 1.75064E-12 | 5.15186E-11 | K15382 |
| *uncharacterized protein_48389* | 3365.380112 | 1335.534272 | 0.396845 | -1.333352465 | 1.76121E-12 | 5.17703E-11 | - |
| *LAC11* | 277.4955042 | 5.884089472 | 0.02120427 | -5.559501403 | 1.76777E-12 | 5.19037E-11 | K05909 |
| *SPDS2* | 1984.975331 | 770.5173725 | 0.388174785 | -1.365221688 | 1.85852E-12 | 5.4506E-11 | K00797 |
| *CP33* | 6108.448824 | 2461.137356 | 0.402907093 | -1.311480892 | 1.86439E-12 | 5.46157E-11 | K11294 |
| *KLCR1* | 1481.514157 | 508.6525044 | 0.343332868 | -1.542320119 | 1.91279E-12 | 5.59697E-11 | - |
| *uncharacterized protein_09975* | 3115.185056 | 973.8260981 | 0.312606179 | -1.677581799 | 1.99116E-12 | 5.81967E-11 | - |
| *uncharacterized protein_25839* | 53.3001515 | 2.93746333 | 0.055111726 | -4.181496887 | 1.99345E-12 | 5.81974E-11 | - |
| *At4g02290* | 70.47238193 | 0.295072441 | 0.004187065 | -7.899844985 | 2.04457E-12 | 5.96221E-11 | - |
| *PSAK* | 17861.33634 | 6780.583633 | 0.379623535 | -1.397358661 | 2.07513E-12 | 6.04446E-11 | K02698 |
| *At3g24670* | 32.76001174 | 0 | 0 | -Inf | 2.07866E-12 | 6.04788E-11 | K01728 |
| *TPS-sab* | 35.62671852 | 0.300107576 | 0.008423666 | -6.891336057 | 2.34197E-12 | 6.77562E-11 | K18108 |
| *yugF* | 1154.915377 | 434.9939747 | 0.376645756 | -1.408719823 | 2.42119E-12 | 6.99694E-11 | - |
| *At3g13620* | 71.23617328 | 7.108758583 | 0.099791416 | -3.324940473 | 2.50939E-12 | 7.23555E-11 | - |
| *TBL26* | 210.7776746 | 53.87862415 | 0.255618268 | -1.967937151 | 2.59812E-12 | 7.483E-11 | - |
| *PHT2-1* | 971.6166106 | 361.2754442 | 0.371829218 | -1.427287956 | 2.98823E-12 | 8.56814E-11 | K14640 |
| *menG* | 1361.732438 | 521.5474198 | 0.383002861 | -1.384572926 | 3.0003E-12 | 8.59316E-11 | - |
| *uncharacterized protein_05993* | 108.7014308 | 17.95828917 | 0.165207477 | -2.597649109 | 3.05778E-12 | 8.74806E-11 | - |
| *IP5P11* | 35.21198569 | 0.300107576 | 0.008522881 | -6.874443037 | 3.13384E-12 | 8.95567E-11 | K20278 |
| *ASPG1* | 78.55917541 | 9.159628698 | 0.116595275 | -3.100418764 | 3.28721E-12 | 9.38353E-11 | - |
| *PGLP1A* | 2439.33815 | 968.0189227 | 0.396836709 | -1.333382609 | 3.34469E-12 | 9.537E-11 | K19269 |
| *RBCS* | 943.9375578 | 257.7119242 | 0.273017979 | -1.872932136 | 3.7113E-12 | 1.05706E-10 | K01602 |
| *uncharacterized protein_28512* | 516.889984 | 151.2588931 | 0.292632664 | -1.77283728 | 3.72624E-12 | 1.06014E-10 | - |
| *UNC* | 167.4774675 | 38.37786058 | 0.229152382 | -2.125620814 | 3.80601E-12 | 1.08164E-10 | - |
| *PIP2-8* | 95.44283858 | 14.18595987 | 0.148633047 | -2.750173179 | 4.1662E-12 | 1.18009E-10 | K09872 |
| *CPK17* | 345.8718972 | 107.6172308 | 0.311147658 | -1.684328708 | 4.17618E-12 | 1.18161E-10 | K13412 |
| *At4g32940* | 2235.259181 | 150.490801 | 0.067325884 | -3.892694916 | 4.19035E-12 | 1.18432E-10 | K01369 |
| *LOX1.1* | 43.66512324 | 1.495502745 | 0.034249365 | -4.867778948 | 4.30042E-12 | 1.21275E-10 | K15718 |
| *Os06g0254200* | 393.2072003 | 127.3139289 | 0.32378331 | -1.626899473 | 5.58188E-12 | 1.56214E-10 | - |
| *ag4* | 76.3127876 | 1.185324899 | 0.015532454 | -6.008570365 | 5.71301E-12 | 1.59709E-10 | - |
| *ATS1* | 2862.284379 | 1155.603215 | 0.403734592 | -1.308520893 | 5.93964E-12 | 1.65724E-10 | K00630 |
| *CSF7* | 353.1412202 | 111.2734986 | 0.315096319 | -1.666135194 | 5.94106E-12 | 1.65724E-10 | K00660 |
| *HIPP37* | 48.01252386 | 1.766775402 | 0.03679822 | -4.764220219 | 5.97134E-12 | 1.66388E-10 | - |
| *R9* | 49.61603852 | 0.300107576 | 0.0060486 | -7.369183001 | 6.22011E-12 | 1.73132E-10 | K00430 |
| *uncharacterized protein_13679* | 1130.034627 | 433.9152951 | 0.38398407 | -1.380881635 | 6.48297E-12 | 1.80058E-10 | - |
| *At4g01130* | 63.30142303 | 5.603654002 | 0.08852335 | -3.497798148 | 7.05655E-12 | 1.95355E-10 | - |
| *PSRP2* | 1523.986308 | 600.7099056 | 0.394170146 | -1.343109582 | 7.87229E-12 | 2.17003E-10 | K13126 |
| *HHT1* | 91.95928727 | 13.59625402 | 0.147850798 | -2.757786064 | 8.09264E-12 | 2.22838E-10 | K15400 |
| *SBT5.6* | 40.81227062 | 1.195395169 | 0.029290092 | -5.093443456 | 8.45629E-12 | 2.32105E-10 | - |
| *MAN1* | 268.1211905 | 78.50054111 | 0.292780071 | -1.772110741 | 9.01476E-12 | 2.46643E-10 | K19355 |
| *GLR3.3* | 308.183452 | 94.68705831 | 0.307242513 | -1.702550239 | 9.42196E-12 | 2.5751E-10 | K05387 |
| *uncharacterized protein_29718* | 515.0494411 | 125.7947826 | 0.244238266 | -2.033638841 | 1.12173E-11 | 3.04311E-10 | - |
| *At3g47110* | 48.88175729 | 2.656120402 | 0.054337662 | -4.2019037 | 1.13971E-11 | 3.08674E-10 | - |
| *JGB* | 51.80190618 | 3.296148202 | 0.063629863 | -3.974152171 | 1.14079E-11 | 3.08674E-10 | - |
| *WRKY35* | 101.1813845 | 16.88095605 | 0.166838556 | -2.583475367 | 1.14231E-11 | 3.08674E-10 | - |
| *UGT83A1* | 35.99651523 | 0.595180017 | 0.016534379 | -5.918387349 | 1.21833E-11 | 3.28094E-10 | - |
| *ZHD4* | 156.3270021 | 35.97975176 | 0.230156987 | -2.119309856 | 1.22067E-11 | 3.28378E-10 | - |
| *MYB46* | 84.92999186 | 11.8322304 | 0.139317456 | -2.843552062 | 1.25715E-11 | 3.37487E-10 | K09422 |
| *GGAT1* | 8193.762128 | 3440.654978 | 0.419911504 | -1.251842782 | 1.27914E-11 | 3.43032E-10 | K14272 |
| *TDR* | 805.2479502 | 227.0573436 | 0.281971961 | -1.826376386 | 1.352E-11 | 3.6144E-10 | - |
| *PPD* | 2826.975577 | 846.1815571 | 0.299323972 | -1.740220272 | 1.36781E-11 | 3.65286E-10 | K01006 |
| *GLP1* | 188.3786249 | 0.900322728 | 0.004779325 | -7.708977318 | 1.40192E-11 | 3.74004E-10 | - |
| *TIP2-1* | 2096.624225 | 157.0400057 | 0.07490136 | -3.738864277 | 1.40939E-11 | 3.75609E-10 | K09873 |
| *R42* | 498.7487465 | 8.569952251 | 0.017182905 | -5.862882239 | 1.4626E-11 | 3.88981E-10 | K00430 |
| *BAM3* | 42.73899504 | 0.590144882 | 0.013808113 | -6.178339993 | 1.48087E-11 | 3.93432E-10 | K01177 |
| *PCBER* | 3310.507987 | 1168.530708 | 0.352976254 | -1.502356964 | 1.53063E-11 | 4.05816E-10 | - |
| *NYC1* | 1797.004736 | 725.317958 | 0.403626069 | -1.308908737 | 1.59087E-11 | 4.20917E-10 | K13606 |
| *EPHX4* | 62.84175409 | 5.924809577 | 0.094281416 | -3.406882759 | 1.60873E-11 | 4.25204E-10 | - |
| *uncharacterized protein_32593* | 777.4354811 | 292.8329716 | 0.376665304 | -1.408644948 | 1.62183E-11 | 4.28226E-10 | - |
| *GALS1* | 316.6191459 | 77.99874531 | 0.246348796 | -2.021225676 | 1.63979E-11 | 4.32525E-10 | - |
| *FBP* | 2200.799319 | 520.8261172 | 0.236653162 | -2.079153898 | 1.70108E-11 | 4.4777E-10 | K03841 |
| *C4H2* | 4158.976642 | 703.3346204 | 0.169112424 | -2.563945444 | 1.74669E-11 | 4.58836E-10 | K00487 |
| *MAP1D* | 720.441391 | 268.635841 | 0.372876745 | -1.42322927 | 1.92097E-11 | 5.02564E-10 | K01265 |
| *TCP14* | 272.9231565 | 82.27650023 | 0.301463977 | -1.729942474 | 2.11362E-11 | 5.51279E-10 | - |
| *HAK9* | 129.0013419 | 26.91072608 | 0.20860811 | -2.261132851 | 2.2362E-11 | 5.8207E-10 | K03549 |
| *BOR2* | 159.3353364 | 38.10611949 | 0.239156739 | -2.063971649 | 2.39282E-11 | 6.22207E-10 | - |
| *ASOL* | 283.1284353 | 86.96476467 | 0.30715659 | -1.702953759 | 2.47795E-11 | 6.43306E-10 | - |
| *RK8* | 111.0497361 | 3.844197086 | 0.034616895 | -4.852379852 | 2.47897E-11 | 6.43306E-10 | - |
| *At2g05160* | 559.3746374 | 75.90166067 | 0.135690208 | -2.881611486 | 2.56977E-11 | 6.65523E-10 | - |
| *At1g68400* | 170.0623472 | 42.21058209 | 0.248206512 | -2.010387126 | 2.57608E-11 | 6.66487E-10 | - |
| *STK38L* | 824.6569657 | 315.3494714 | 0.382400785 | -1.386842608 | 2.58996E-11 | 6.69403E-10 | K08790 |
| *BGLU4* | 741.7742062 | 280.6854217 | 0.378397387 | -1.402025967 | 2.72308E-11 | 7.02397E-10 | K01188 |
| *At1g17710* | 595.3264101 | 166.703236 | 0.28001989 | -1.836398788 | 2.75252E-11 | 7.09277E-10 | K13248 |
| *tas* | 516.1241817 | 185.6837254 | 0.359765599 | -1.474870853 | 3.18123E-11 | 8.15659E-10 | - |
| *TUBB6* | 787.1827848 | 301.1177563 | 0.38252584 | -1.386370887 | 3.20122E-11 | 8.19965E-10 | K07375 |
| *PT5* | 36.97971093 | 0.295072441 | 0.007979306 | -6.969520955 | 3.23674E-11 | 8.28239E-10 | K12742 |
| *MDHG* | 12918.48435 | 5548.014058 | 0.429463233 | -1.219393468 | 3.28149E-11 | 8.3802E-10 | K00026 |
| *At4g01130* | 135.0316489 | 21.6259443 | 0.160154634 | -2.642462553 | 3.38179E-11 | 8.62776E-10 | - |
| *PHOS34* | 394.1802249 | 133.6751069 | 0.339121799 | -1.560124571 | 3.47736E-11 | 8.86279E-10 | - |
| *UGT73C13* | 102.2735109 | 9.455608596 | 0.092454131 | -3.435118403 | 3.56755E-11 | 9.08364E-10 | K13496 |
| *mcfF* | 1627.023694 | 314.2086324 | 0.193118658 | -2.372440538 | 3.57684E-11 | 9.09829E-10 | K15113 |
| *HSP22* | 373.7714692 | 54.02367679 | 0.144536652 | -2.790492713 | 3.88795E-11 | 9.84091E-10 | K13993 |
| *uncharacterized protein_22018* | 1858.89015 | 766.0681951 | 0.412110525 | -1.278896786 | 3.9695E-11 | 1.00276E-09 | - |
| *LTPG5* | 67.59530961 | 7.6993719 | 0.113903937 | -3.134110479 | 4.37849E-11 | 1.10282E-09 | - |
| *At1g60630* | 28.71320896 | 0 | 0 | -Inf | 4.40692E-11 | 1.1089E-09 | - |
| *PTC52* | 1164.022267 | 467.0530949 | 0.401240688 | -1.317460186 | 4.45612E-11 | 1.12018E-09 | - |
| *TRXM* | 3287.769559 | 1391.032188 | 0.42309297 | -1.240953379 | 4.86533E-11 | 1.21709E-09 | K03671 |
| *PRXQ* | 8101.519067 | 3495.657653 | 0.431481754 | -1.212628543 | 4.9824E-11 | 1.24516E-09 | K03564 |
| *THI1-2* | 25328.99635 | 11017.55898 | 0.434978111 | -1.200985292 | 5.13694E-11 | 1.28129E-09 | K03146 |
| *SWEET16* | 44.79515076 | 2.357388719 | 0.05262598 | -4.248080989 | 5.45208E-11 | 1.35726E-09 | K15382 |
| *MJ0531* | 47.7032402 | 2.943405923 | 0.061702432 | -4.018528841 | 5.50905E-11 | 1.37012E-09 | - |
| *LAC1* | 74.34475245 | 9.779515956 | 0.131542787 | -2.92639595 | 5.9342E-11 | 1.473E-09 | K05909 |
| *GLO1* | 39783.6704 | 17348.24024 | 0.436064347 | -1.197387057 | 5.98578E-11 | 1.48389E-09 | K11517 |
| *uncharacterized protein_06714* | 328.7393338 | 18.33573869 | 0.05577592 | -4.164213791 | 5.98962E-11 | 1.48389E-09 | - |
| *ISPG* | 9361.820332 | 4066.997719 | 0.434423817 | -1.202824896 | 6.61521E-11 | 1.63572E-09 | K03526 |
| *rumi* | 53.91871882 | 4.464084344 | 0.082792849 | -3.594350026 | 6.93249E-11 | 1.71088E-09 | - |
| *uncharacterized protein_14429* | 197.6782903 | 54.72956181 | 0.276861772 | -1.852762227 | 7.13169E-11 | 1.75835E-09 | - |
| *PMR5* | 1558.371793 | 390.3660708 | 0.250496109 | -1.9971399 | 7.24607E-11 | 1.78484E-09 | - |
| *uncharacterized protein_10281* | 4593.652403 | 1213.968214 | 0.264270804 | -1.919911047 | 7.38721E-11 | 1.81612E-09 | - |
| *uncharacterized protein_24642* | 78.52454001 | 11.25672249 | 0.143352925 | -2.802356754 | 7.65583E-11 | 1.88036E-09 | - |
| *MLP423* | 686.8323664 | 84.64311059 | 0.123236928 | -3.020493475 | 7.87823E-11 | 1.93314E-09 | - |
| *GAST1* | 27.99439677 | 0 | 0 | -Inf | 7.88906E-11 | 1.93395E-09 | - |
| *APS1* | 55.9973426 | 1.49046761 | 0.026616756 | -5.231521436 | 7.96723E-11 | 1.95125E-09 | - |
| *pitC* | 288.6475769 | 92.55752918 | 0.320659297 | -1.640886857 | 8.09103E-11 | 1.9778E-09 | - |
| *UGT86A2* | 91.62426981 | 9.43136979 | 0.102935279 | -3.280190571 | 8.18697E-11 | 1.99555E-09 | - |
| *NAKR2* | 87.98509295 | 14.22345976 | 0.161657609 | -2.62898668 | 8.23941E-11 | 2.00643E-09 | - |
| *uncharacterized protein_31365* | 155.5127897 | 9.675651855 | 0.062217724 | -4.006530565 | 8.41735E-11 | 2.04576E-09 | K15082 |
| *AHL10* | 2281.390729 | 963.3827068 | 0.422278698 | -1.243732623 | 8.42481E-11 | 2.04576E-09 | - |
| *rlmL* | 247.7214159 | 75.58090555 | 0.305104447 | -1.712624888 | 8.99058E-11 | 2.17698E-09 | - |
| *GLCAT14A* | 192.2472115 | 53.21523559 | 0.276806281 | -1.853051417 | 9.0072E-11 | 2.17894E-09 | - |
| *uncharacterized protein_42412* | 17893.5467 | 5255.255672 | 0.293695585 | -1.767606515 | 9.14726E-11 | 2.20867E-09 | - |
| *TET8* | 68.21394176 | 2.078329141 | 0.030467806 | -5.036570577 | 9.66537E-11 | 2.32313E-09 | - |
| *7OMT* | 64.73380713 | 3.873939463 | 0.059844147 | -4.06264604 | 9.66652E-11 | 2.32313E-09 | - |
| *GT7* | 420.6047638 | 48.95155181 | 0.116383731 | -3.10303869 | 9.85459E-11 | 2.36611E-09 | K13496 |
| *SPAC24B11.05* | 328.1622859 | 64.54235259 | 0.196678154 | -2.346091376 | 1.0245E-10 | 2.45299E-09 | K07025 |
| *CAR3* | 54.41467401 | 4.757780893 | 0.087435622 | -3.515635017 | 1.09088E-10 | 2.60463E-09 | K12486 |
| *CSP41A* | 10923.6191 | 4802.269675 | 0.439622586 | -1.185662587 | 1.10782E-10 | 2.64261E-09 | - |
| *GLR3.1* | 705.2992158 | 273.3630015 | 0.38758444 | -1.367417442 | 1.11137E-10 | 2.64831E-09 | K05387 |
| *Os12g0623900* | 87.09166868 | 14.1713229 | 0.16271732 | -2.619560274 | 1.11303E-10 | 2.64831E-09 | K00549 |
| *At1g67720* | 131.5085527 | 29.75527363 | 0.226261129 | -2.143939339 | 1.13077E-10 | 2.68737E-09 | - |
| *GAT1* | 692.2902072 | 205.7705094 | 0.297231576 | -1.750340708 | 1.15236E-10 | 2.73617E-09 | - |
| *RIR2* | 30.41173532 | 0.295072441 | 0.009702585 | -6.687415148 | 1.19186E-10 | 2.82213E-09 | K10808 |
| *OPS* | 196.9784646 | 50.95579779 | 0.258687151 | -1.950719699 | 1.2648E-10 | 2.992E-09 | - |
| *YSL12* | 58.87624559 | 5.911987522 | 0.1004138 | -3.315970547 | 1.26593E-10 | 2.992E-09 | - |
| *At4g34480* | 66.4962921 | 5.012133228 | 0.075374627 | -3.729777228 | 1.27341E-10 | 3.00691E-09 | - |
| *CKX5* | 34.94074703 | 0.882933972 | 0.025269465 | -5.306461089 | 1.28504E-10 | 3.02882E-09 | K00279 |
| *ISE2* | 547.0494455 | 205.3922498 | 0.375454635 | -1.413289493 | 1.28505E-10 | 3.02882E-09 | - |
| *BHLH62* | 43.3730314 | 2.350977691 | 0.054203675 | -4.205465525 | 1.3539E-10 | 3.18792E-09 | - |
| *AXS2* | 1386.604186 | 575.6908782 | 0.415180398 | -1.268189763 | 1.35503E-10 | 3.18792E-09 | K12449 |
| *SCPL42* | 186.0560965 | 51.57067933 | 0.277178122 | -1.851114709 | 1.42545E-10 | 3.3383E-09 | K16297 |
| *POPTRDRAFT_821063* | 559.2124298 | 211.52431 | 0.378253949 | -1.40257295 | 1.5246E-10 | 3.56402E-09 | K01872 |
| *FAD8* | 380.8487507 | 134.1944183 | 0.352356199 | -1.504893498 | 1.60211E-10 | 3.73162E-09 | K10257 |
| *FBP* | 164.5485968 | 43.28710514 | 0.263065781 | -1.926504496 | 1.63182E-10 | 3.79737E-09 | K03841 |
| *DTX44* | 314.777442 | 105.8797973 | 0.336363993 | -1.571904818 | 1.65854E-10 | 3.85607E-09 | - |
| *MCM4* | 32.37987836 | 0.600215152 | 0.018536671 | -5.753474016 | 1.77413E-10 | 4.12108E-09 | K02212 |
| *At3g02645* | 1045.703249 | 329.0121871 | 0.314632461 | -1.66826057 | 1.78621E-10 | 4.1425E-09 | - |
| *XCP1* | 5173.354208 | 2167.620125 | 0.418997045 | -1.254988027 | 1.78657E-10 | 4.1425E-09 | - |
| *At3g47110* | 116.7451117 | 10.69907177 | 0.091644709 | -3.447804602 | 1.87592E-10 | 4.33792E-09 | - |
| *GLB3* | 698.0884527 | 259.2023055 | 0.371302955 | -1.429331297 | 1.89627E-10 | 4.38103E-09 | - |
| *PSBY* | 4049.66824 | 1772.93989 | 0.437798799 | -1.191660099 | 1.94222E-10 | 4.48316E-09 | K02723 |
| *COL16* | 50.86544131 | 4.137425199 | 0.081340594 | -3.619880667 | 2.09164E-10 | 4.82372E-09 | - |
| *TUBA* | 13452.63829 | 5998.820911 | 0.44592152 | -1.16513827 | 2.10419E-10 | 4.84832E-09 | K07374 |
| *At3g07010* | 296.7491779 | 1.764492052 | 0.005946072 | -7.393847291 | 2.1588E-10 | 4.96968E-09 | K01728 |
| *mhpC* | 174.1922678 | 37.63585157 | 0.216059255 | -2.210501066 | 2.18365E-10 | 5.0224E-09 | - |
| *uncharacterized protein_30656* | 37.7590002 | 1.460725233 | 0.038685485 | -4.692063832 | 2.23736E-10 | 5.13672E-09 | - |
| *CRYD* | 347.2349604 | 120.8791361 | 0.348119141 | -1.522346953 | 2.23971E-10 | 5.13753E-09 | - |
| *EXPA2* | 96.13904595 | 0 | 0 | -Inf | 2.31851E-10 | 5.31354E-09 | - |
| *At3g43860* | 175.9994154 | 48.40575687 | 0.275033623 | -1.862320095 | 2.3342E-10 | 5.34473E-09 | K01179 |
| *NAC075* | 94.8000085 | 17.43863619 | 0.183951842 | -2.442599972 | 2.34587E-10 | 5.36667E-09 | - |
| *TPS5* | 2163.396506 | 932.7670114 | 0.431158601 | -1.213709434 | 2.35348E-10 | 5.37929E-09 | K16055 |
| *CXE20* | 227.5956205 | 52.54552424 | 0.230872299 | -2.114833009 | 2.42737E-10 | 5.54326E-09 | - |
| *THIM* | 526.2860886 | 142.7100183 | 0.271164337 | -1.882760644 | 2.53272E-10 | 5.77379E-09 | K00878 |
| *CYP720B2* | 129.7042965 | 7.427630808 | 0.057265881 | -4.126180353 | 2.53282E-10 | 5.77379E-09 | - |
| *PR1* | 42.39335319 | 2.390790338 | 0.056395405 | -4.148278561 | 2.60729E-10 | 5.93829E-09 | - |
| *CD4B* | 510.2299743 | 192.7067801 | 0.377686122 | -1.404740323 | 2.63826E-10 | 6.0035E-09 | K03696 |
| *MUCI21* | 26.42885518 | 0 | 0 | -Inf | 2.6512E-10 | 6.0276E-09 | - |
| *ISPF* | 3250.316313 | 1425.746073 | 0.438648407 | -1.188863065 | 2.73223E-10 | 6.20635E-09 | K01770 |
| *CHS* | 786.9213292 | 200.4471166 | 0.254723197 | -1.972997748 | 2.82258E-10 | 6.40592E-09 | K00660 |
| *CAB6A* | 271.1151742 | 2.630037268 | 0.009700812 | -6.687678809 | 2.90618E-10 | 6.57821E-09 | K08907 |
| *SHM1* | 1344.286452 | 492.9137025 | 0.366673116 | -1.4474336 | 3.00123E-10 | 6.78141E-09 | K00600 |
| *R53* | 51.62743086 | 1.200430304 | 0.023251792 | -5.426514257 | 3.03591E-10 | 6.84772E-09 | K00430 |
| *uncharacterized protein_19142* | 221.1317957 | 67.5549719 | 0.30549642 | -1.710772622 | 3.05243E-10 | 6.87736E-09 | - |
| *At4g27270* | 67.49685945 | 8.873250635 | 0.131461681 | -2.927285752 | 3.06102E-10 | 6.88415E-09 | K03809 |
| *UGT85K4* | 201.5196579 | 57.51147236 | 0.285388894 | -1.8089989 | 3.06475E-10 | 6.88415E-09 | - |
| *uncharacterized protein_04027* | 2431.535747 | 930.885539 | 0.382838517 | -1.38519211 | 3.06546E-10 | 6.88415E-09 | - |
| *LRK10L-2.6* | 43.4405075 | 2.692273807 | 0.061976113 | -4.012143912 | 3.24921E-10 | 7.28407E-09 | - |
| *At2g18940* | 142.8558895 | 35.8169099 | 0.250720569 | -1.995847736 | 3.36888E-10 | 7.53919E-09 | - |
| *RLK902* | 327.9751256 | 54.59135922 | 0.166449694 | -2.586841878 | 3.44408E-10 | 7.70078E-09 | - |
| *BPA1* | 186.1614378 | 42.69961466 | 0.229368741 | -2.124259303 | 3.50921E-10 | 7.83959E-09 | - |
| *At2g24580* | 81.09753297 | 8.896581984 | 0.109702252 | -3.188334955 | 3.54629E-10 | 7.91553E-09 | K00306 |
| *TPS14* | 297.9641175 | 12.51938933 | 0.042016433 | -4.572902506 | 3.62852E-10 | 8.09203E-09 | K04120 |
| *SCPL45* | 696.8029298 | 273.9610398 | 0.393168611 | -1.346779949 | 3.69359E-10 | 8.21575E-09 | K16297 |
| *uncharacterized protein_45779* | 66.05916315 | 8.551187603 | 0.12944741 | -2.949561991 | 3.792E-10 | 8.42008E-09 | - |
| *BASS3* | 4918.736455 | 2192.103756 | 0.445663999 | -1.165971672 | 3.81854E-10 | 8.47169E-09 | K03453 |
| *TMKL1* | 79.23642511 | 12.73346058 | 0.160702108 | -2.637539237 | 3.91665E-10 | 8.67436E-09 | - |
| *PPOX1* | 1810.038511 | 783.0847875 | 0.432634324 | -1.208779965 | 3.96573E-10 | 8.7755E-09 | K00231 |
| *CHUP1* | 118.5348735 | 5.662231299 | 0.047768485 | -4.387797075 | 4.07828E-10 | 9.00904E-09 | - |
| *CDA1* | 395.7923317 | 132.420666 | 0.334571075 | -1.57961537 | 4.11689E-10 | 9.08652E-09 | K01489 |
| *uncharacterized protein_33326* | 2899.664263 | 1278.434666 | 0.440890583 | -1.181507433 | 4.19999E-10 | 9.26021E-09 | - |
| *CEP1* | 211.5137146 | 64.01722544 | 0.302662291 | -1.724219155 | 4.2028E-10 | 9.26021E-09 | K16292 |
| *At5g09300* | 369.3304707 | 121.5277609 | 0.329048834 | -1.603626383 | 4.2837E-10 | 9.43039E-09 | K00166 |
| *3-Apr* | 520.7413211 | 163.9706131 | 0.314879205 | -1.667129612 | 4.33542E-10 | 9.52791E-09 | K05907 |
| *DHAPS-1* | 28.65097312 | 0.300107576 | 0.010474603 | -6.576960591 | 4.75813E-10 | 1.04035E-08 | K01626 |
| *nep2* | 25.70139313 | 0 | 0 | -Inf | 4.78406E-10 | 1.04513E-08 | - |
| *ADHX* | 725.6331935 | 193.0762867 | 0.266079733 | -1.910069466 | 4.8157E-10 | 1.05115E-08 | K00001 |
| *ABCB11* | 1144.635738 | 440.3359196 | 0.38469524 | -1.378212117 | 4.95332E-10 | 1.08027E-08 | K05658 |
| *uncharacterized protein_47155* | 101.8345442 | 20.64415195 | 0.202722486 | -2.302421973 | 5.03895E-10 | 1.09801E-08 | - |
| *MGP* | 1180.518845 | 475.2359076 | 0.402565287 | -1.31270532 | 5.06593E-10 | 1.10296E-08 | K12309 |
| *DHQS* | 7049.74464 | 3176.863204 | 0.450635217 | -1.149968031 | 5.13502E-10 | 1.11705E-08 | K01735 |
| *CYP90B2* | 37.82629648 | 0 | 0 | -Inf | 5.32453E-10 | 1.15632E-08 | K09587 |
| *LSMT-L* | 2063.332446 | 902.4221384 | 0.437361483 | -1.193101923 | 5.35325E-10 | 1.16157E-08 | K00592 |
| *uncharacterized protein_08788* | 298.605477 | 47.12992633 | 0.157833429 | -2.663525296 | 5.4176E-10 | 1.17454E-08 | K00574 |
| *PCMP-H40* | 99.72987001 | 20.09106793 | 0.201454869 | -2.311471418 | 5.45257E-10 | 1.18113E-08 | - |
| *KIN12F* | 159.3159543 | 7.963326071 | 0.049984486 | -4.322375806 | 5.52314E-10 | 1.19541E-08 | K10400 |
| *uncharacterized protein_13641* | 491.4822257 | 182.8546249 | 0.372047279 | -1.426442126 | 5.579E-10 | 1.20612E-08 | - |
| *UBI11* | 41154.68291 | 18760.2661 | 0.455847665 | -1.13337631 | 5.58202E-10 | 1.20612E-08 | K08770 |
| *R* | 14640.40615 | 6653.240202 | 0.45444369 | -1.137826552 | 5.6319E-10 | 1.21485E-08 | K01783 |
| *ATAT1* | 73.84700255 | 11.22698011 | 0.152030275 | -2.717569447 | 5.67888E-10 | 1.22396E-08 | K19573 |
| *ABCB15* | 119.8898262 | 6.197926562 | 0.051696852 | -4.273779767 | 5.87995E-10 | 1.26624E-08 | K05658 |
| *PAM68* | 1036.428849 | 436.7072962 | 0.421357719 | -1.246882539 | 6.1305E-10 | 1.31578E-08 | - |
| *LPD* | 5630.23262 | 2548.779238 | 0.452695192 | -1.14338811 | 6.38265E-10 | 1.36647E-08 | K00382 |
| *KIN4C* | 231.7030802 | 74.0104416 | 0.319419326 | -1.646476493 | 6.52543E-10 | 1.39472E-08 | K10395 |
| *DTX33* | 264.0384757 | 55.08637294 | 0.208630098 | -2.260980795 | 6.56096E-10 | 1.39882E-08 | K03327 |
| *CYCD2-2* | 52.1302483 | 5.049194091 | 0.096857281 | -3.367995691 | 6.80043E-10 | 1.44578E-08 | K18810 |
| *CYP750A1* | 57.61995169 | 6.530967322 | 0.113345588 | -3.141199856 | 6.80372E-10 | 1.44578E-08 | - |
| *uncharacterized protein_14829* | 105.535777 | 22.44983254 | 0.212722483 | -2.232955572 | 7.35051E-10 | 1.55426E-08 | - |
| *HEMA1* | 5183.815007 | 2341.440424 | 0.451682867 | -1.146617904 | 7.54176E-10 | 1.59338E-08 | K02492 |
| *LIGB* | 1063.668863 | 450.7586062 | 0.423777194 | -1.238622145 | 7.66998E-10 | 1.61914E-08 | K15777 |
| *OPT4* | 140.6422057 | 26.30316303 | 0.187021832 | -2.418721398 | 7.68081E-10 | 1.6201E-08 | - |
| *Prcp* | 49.59877477 | 4.43800121 | 0.089478041 | -3.482322516 | 7.73853E-10 | 1.6296E-08 | K01285 |
| *NEK5* | 102.6655958 | 21.62638333 | 0.210648788 | -2.24708848 | 7.85658E-10 | 1.6531E-08 | K08857 |
| *ADG2* | 713.9582774 | 291.6457436 | 0.408491298 | -1.291622749 | 8.04302E-10 | 1.68818E-08 | K00975 |
| *XTH8* | 796.7649642 | 85.75016583 | 0.107622912 | -3.215942843 | 8.13585E-10 | 1.70627E-08 | K08235 |
| *uncharacterized protein_09970* | 103.430959 | 5.309489019 | 0.051333654 | -4.283951245 | 8.29223E-10 | 1.73623E-08 | - |
| *CURT1A* | 513.997464 | 200.638558 | 0.390349315 | -1.357162356 | 8.48786E-10 | 1.77575E-08 | - |
| *AIR9* | 528.1918595 | 206.8736043 | 0.39166375 | -1.352312488 | 8.57527E-10 | 1.79112E-08 | - |
| *TPS-sab* | 24.96355845 | 0 | 0 | -Inf | 8.67754E-10 | 1.80807E-08 | K12742 |
| *At3g15890* | 236.8194263 | 59.434708 | 0.250970577 | -1.994409859 | 8.80152E-10 | 1.83241E-08 | - |
| *PARC* | 48.25622693 | 4.126447471 | 0.085511192 | -3.547742938 | 8.85443E-10 | 1.84194E-08 | K00799 |
| *SCP26* | 35.19823942 | 0 | 0 | -Inf | 9.22137E-10 | 1.91517E-08 | K16297 |
| *CYP71AU50* | 71.4625118 | 10.92502821 | 0.152877753 | -2.709549619 | 9.89474E-10 | 2.05005E-08 | - |
| *uncharacterized protein_23686* | 964.58696 | 55.83849079 | 0.057888498 | -4.110579453 | 9.93182E-10 | 2.05607E-08 | - |
| *H2A* | 2415.006242 | 1080.144402 | 0.447263607 | -1.160802722 | 1.00323E-09 | 2.07353E-08 | K11251 |
| *TDR* | 220.787668 | 70.22791523 | 0.318078976 | -1.65254308 | 1.06215E-09 | 2.18827E-08 | - |
| *RBOHF* | 265.7416452 | 80.96223295 | 0.304665206 | -1.714703349 | 1.11119E-09 | 2.28746E-08 | K13447 |
| *SPS2* | 593.8057762 | 238.7419643 | 0.402053961 | -1.314538952 | 1.16953E-09 | 2.39605E-08 | K05356 |
| *DEGP8* | 1027.916821 | 438.1791846 | 0.426278835 | -1.230130669 | 1.17136E-09 | 2.39621E-08 | - |
| *CYP71AU50* | 398.5253988 | 33.23947792 | 0.083406172 | -3.583702045 | 1.17282E-09 | 2.39706E-08 | - |
| *PDX1* | 2012.921099 | 772.6136342 | 0.383827083 | -1.381471582 | 1.20045E-09 | 2.44963E-08 | K06215 |
| *ROPGAP1* | 150.9286779 | 41.17171514 | 0.272789212 | -1.874141503 | 1.21695E-09 | 2.48133E-08 | - |
| *PAE8* | 369.0956692 | 123.2008399 | 0.333791074 | -1.582982719 | 1.22651E-09 | 2.49853E-08 | K19882 |
| *CPK17* | 252.097967 | 84.40518073 | 0.334811033 | -1.578581026 | 1.23641E-09 | 2.51501E-08 | K13412 |
| *PSBR* | 100058.7405 | 46552.42543 | 0.465250963 | -1.103918957 | 1.25808E-09 | 2.55205E-08 | K03541 |
| *LTPG5* | 134.9644894 | 13.00795346 | 0.096380563 | -3.375113962 | 1.27967E-09 | 2.5907E-08 | - |
| *PHOS34* | 166.1312654 | 47.72876559 | 0.287295504 | -1.79939268 | 1.29214E-09 | 2.61389E-08 | - |
| *ATL72* | 1848.502325 | 821.4407513 | 0.444381779 | -1.170128432 | 1.33424E-09 | 2.69481E-08 | K19040 |
| *LDOX* | 1242.660909 | 62.49526852 | 0.05029149 | -4.313541897 | 1.37371E-09 | 2.76801E-08 | K05277 |
| *LBD12* | 94.40451395 | 13.9370817 | 0.147631518 | -2.759927339 | 1.40313E-09 | 2.82507E-08 | - |
| *At5g48740* | 80.41146068 | 14.23399847 | 0.177014549 | -2.498060151 | 1.43284E-09 | 2.87811E-08 | - |
| *EXPB16* | 88.54821475 | 1.185324899 | 0.013386209 | -6.223108758 | 1.43929E-09 | 2.88657E-08 | - |
| *At1g73050* | 345.6072909 | 126.8207686 | 0.366950501 | -1.446342627 | 1.48343E-09 | 2.97276E-08 | K00108 |
| *PBL27* | 37.61219724 | 0 | 0 | -Inf | 1.53202E-09 | 3.06774E-08 | - |
| *At4g36390* | 1400.226101 | 615.6805069 | 0.439700779 | -1.185406007 | 1.55231E-09 | 3.10595E-08 | - |
| *PBL8* | 106.9370432 | 23.89316902 | 0.223432108 | -2.162091572 | 1.60317E-09 | 3.20522E-08 | - |
| *CSLE6* | 2046.511002 | 365.456604 | 0.178575441 | -2.485394413 | 1.61699E-09 | 3.23032E-08 | - |
| *At4g16563* | 43.25207722 | 3.267313283 | 0.075541188 | -3.726592724 | 1.68372E-09 | 3.35582E-08 | - |
| *KOR* | 3070.230482 | 1396.410241 | 0.45482261 | -1.136624121 | 1.75385E-09 | 3.49018E-08 | K08472 |
| *CYP90D2* | 49.2117143 | 4.762816028 | 0.09678216 | -3.369115046 | 1.77313E-09 | 3.52581E-08 | K12638 |
| *BGLU40* | 64.55420653 | 9.168323076 | 0.142025184 | -2.81578132 | 1.78457E-09 | 3.54582E-08 | K01188 |
| *CYP704B1* | 171.7849205 | 50.50375811 | 0.293994129 | -1.766140749 | 1.79224E-09 | 3.55831E-08 | - |
| *HPT1* | 76.30759039 | 12.9965073 | 0.170317359 | -2.553702607 | 1.81055E-09 | 3.59188E-08 | K09833 |
| *SCL6* | 1253.33559 | 548.4410308 | 0.437585141 | -1.192364346 | 1.84082E-09 | 3.6463E-08 | - |
| *PDRP1* | 246.2385748 | 83.22358305 | 0.33797947 | -1.564992479 | 1.84353E-09 | 3.64886E-08 | K20115 |
| *SLC1* | 76.90221153 | 3.814454709 | 0.049601366 | -4.333476349 | 1.84643E-09 | 3.65178E-08 | - |
| *KIN14D* | 609.6450185 | 198.760229 | 0.326026168 | -1.616940331 | 1.88372E-09 | 3.7147E-08 | K10405 |
| *TLP40* | 237.4745387 | 79.36796921 | 0.334216753 | -1.581144044 | 1.88403E-09 | 3.7147E-08 | - |
| *uncharacterized protein_39029* | 129.8273331 | 16.19426556 | 0.12473695 | -3.003039207 | 1.89427E-09 | 3.73203E-08 | - |
| *At4g10440* | 51.20925248 | 3.550032103 | 0.069324037 | -3.850500524 | 1.91803E-09 | 3.77594E-08 | - |
| *NPF4.3* | 140.3985745 | 22.63234651 | 0.161200686 | -2.633070213 | 1.92251E-09 | 3.78187E-08 | - |
| *PAE8* | 26.8626105 | 0.300107576 | 0.011171944 | -6.483975962 | 1.93177E-09 | 3.79718E-08 | K19882 |
| *FLA4* | 104.2432977 | 19.32119029 | 0.185347075 | -2.431698749 | 2.10988E-09 | 4.13779E-08 | - |
| *OEP37* | 547.6091075 | 221.0820266 | 0.403722333 | -1.308564698 | 2.12446E-09 | 4.16319E-08 | - |
| *DF1* | 874.8658094 | 373.7472928 | 0.42720528 | -1.226998618 | 2.16662E-09 | 4.23613E-08 | - |
| *PUB41* | 92.4674169 | 18.87280985 | 0.204102272 | -2.292635855 | 2.19063E-09 | 4.27981E-08 | - |
| *slr0537* | 159.9228147 | 45.8841504 | 0.28691435 | -1.801307969 | 2.19249E-09 | 4.28019E-08 | - |
| *UGT85K4* | 794.262055 | 291.2011786 | 0.36663111 | -1.447598887 | 2.22079E-09 | 4.33215E-08 | - |
| *CRD1* | 8585.943558 | 4003.066426 | 0.466234887 | -1.100871133 | 2.25032E-09 | 4.38641E-08 | K04035 |
| *uncharacterized protein_25584* | 265.3391374 | 59.94748153 | 0.225927777 | -2.146066438 | 2.285E-09 | 4.45064E-08 | - |
| *CAT1* | 41.44637892 | 2.942498465 | 0.070995309 | -3.816132494 | 2.33121E-09 | 4.53721E-08 | K03294 |
| *AMO* | 296.9991429 | 3.887668976 | 0.013089832 | -6.255409574 | 2.34926E-09 | 4.56542E-08 | K00276 |
| *SecA* | 3692.294305 | 1701.920902 | 0.460938582 | -1.117353563 | 2.38344E-09 | 4.62485E-08 | K03070 |
| *BGLU42* | 171.3168464 | 50.79426385 | 0.296493106 | -1.753929536 | 2.4228E-09 | 4.69414E-08 | K01188 |
| *PTAC5* | 1646.082668 | 738.6966855 | 0.448760381 | -1.155982782 | 2.44504E-09 | 4.73008E-08 | - |
| *LPA3* | 374.5555601 | 142.4276797 | 0.380257817 | -1.394950189 | 2.54786E-09 | 4.91789E-08 | - |
| *uncharacterized protein_08488* | 82.41427509 | 15.40284207 | 0.186895317 | -2.419697675 | 2.55013E-09 | 4.91858E-08 | - |
| *Os01g0723600* | 479.0338821 | 190.6555095 | 0.398000051 | -1.329159479 | 2.70504E-09 | 5.20563E-08 | - |
| *PT1* | 505.4961104 | 203.0920534 | 0.401767787 | -1.315566201 | 2.86024E-09 | 5.48785E-08 | K08176 |
| *MYB61* | 65.43022319 | 9.787302877 | 0.149583822 | -2.740973941 | 2.90105E-09 | 5.55371E-08 | K09422 |
| *TAT* | 199.4028093 | 50.38861322 | 0.252697609 | -1.984516079 | 2.95936E-09 | 5.65691E-08 | K15400 |
| *WSD5* | 69.90909449 | 11.30157027 | 0.161660945 | -2.62895691 | 3.03131E-09 | 5.79015E-08 | - |
| *At1g31830* | 44.7656837 | 0 | 0 | -Inf | 3.05506E-09 | 5.83117E-08 | - |
| *At4g15970* | 39.65970311 | 2.66709813 | 0.067249574 | -3.894331066 | 3.14917E-09 | 6.00177E-08 | - |
| *SBT1.7* | 1640.141924 | 739.3867633 | 0.450806575 | -1.149419538 | 3.29395E-09 | 6.25461E-08 | - |
| *MDAR5* | 410.3932778 | 159.4586464 | 0.388550824 | -1.363824772 | 3.32378E-09 | 6.30658E-08 | K08232 |
| *WAKL20* | 447.5133463 | 52.24948552 | 0.116755145 | -3.098441972 | 3.34155E-09 | 6.33564E-08 | - |
| *ag4* | 26.16623041 | 0.300107576 | 0.011469271 | -6.44608255 | 3.37012E-09 | 6.38037E-08 | K18108 |
| *MUCI21* | 38.15119305 | 2.364707204 | 0.061982523 | -4.011994706 | 3.40742E-09 | 6.4415E-08 | K18134 |
| *uncharacterized protein_08237* | 201.4036851 | 64.68468285 | 0.321169311 | -1.638594049 | 3.4984E-09 | 6.58924E-08 | - |
| *CPX1* | 1831.095438 | 830.6737982 | 0.453648554 | -1.140353034 | 3.51952E-09 | 6.62417E-08 | K00228 |
| *NEK5* | 67.6766039 | 10.70135512 | 0.158124884 | -2.660863677 | 3.61905E-09 | 6.79656E-08 | K08857 |
| *MYB46* | 28.24840764 | 0.590144882 | 0.020891262 | -5.580956553 | 3.62503E-09 | 6.80283E-08 | K09422 |
| *uncharacterized protein_06845* | 486.1256003 | 195.5518641 | 0.40226613 | -1.313777823 | 3.64411E-09 | 6.83363E-08 | - |
| *At1g74460* | 63.94762674 | 9.569572381 | 0.149647029 | -2.740364454 | 3.66346E-09 | 6.86491E-08 | - |
| *CP12-1* | 18997.50911 | 9002.773203 | 0.473892296 | -1.077368888 | 3.85115E-09 | 7.19398E-08 | - |
| *SLC1* | 45.21860538 | 1.764492052 | 0.039021373 | -4.679591661 | 3.85307E-09 | 7.19398E-08 | - |
| *GT7* | 358.0795011 | 107.0889128 | 0.299064628 | -1.741470809 | 3.87473E-09 | 7.22917E-08 | - |
| *PUMP1* | 437.2782125 | 172.8037389 | 0.395180308 | -1.339417035 | 3.95535E-09 | 7.37424E-08 | K15103 |
| *uncharacterized protein_16866* | 42.85292134 | 3.561009831 | 0.083098415 | -3.589035234 | 4.31337E-09 | 8.01844E-08 | - |
| *2-Sep* | 1217.790022 | 544.2342748 | 0.446903214 | -1.161965674 | 4.36198E-09 | 8.10294E-08 | - |
| *GLCAT14B* | 169.9325274 | 45.16133591 | 0.265760397 | -1.911801962 | 4.38631E-09 | 8.13636E-08 | - |
| *VTE4* | 672.9817962 | 285.2804379 | 0.423905133 | -1.238186659 | 4.51462E-09 | 8.36229E-08 | K05928 |
| *CYSEP* | 70.28051318 | 6.221257911 | 0.088520383 | -3.497846504 | 4.5207E-09 | 8.36752E-08 | K16292 |
| *APS1* | 107.8771335 | 25.7784455 | 0.238961165 | -2.065151919 | 4.60797E-09 | 8.51678E-08 | - |
| *uncharacterized protein_36408* | 46.3952918 | 4.398188563 | 0.094798166 | -3.398997045 | 4.68132E-09 | 8.63992E-08 | - |
| *PRXIIE-1* | 214.5898416 | 71.26509407 | 0.332099104 | -1.590314265 | 4.80553E-09 | 8.85644E-08 | - |
| *ENDO4* | 121.1717243 | 10.62538907 | 0.087688684 | -3.511465507 | 4.87732E-09 | 8.97839E-08 | - |
| *TPS4* | 474.8036447 | 192.4864044 | 0.40540212 | -1.30257446 | 4.92318E-09 | 9.05378E-08 | K04120 |
| *ISE2* | 556.2453773 | 230.4648306 | 0.41432224 | -1.271174832 | 4.96412E-09 | 9.11602E-08 | - |
| *uncharacterized protein_42544* | 56.70067865 | 7.431290051 | 0.131061748 | -2.931681417 | 5.02703E-09 | 9.22496E-08 | - |
| *IF2CP* | 6371.136528 | 3012.544381 | 0.47284254 | -1.080568258 | 5.1239E-09 | 9.39601E-08 | K02519 |
| *At5g63180* | 97.86207263 | 21.88211155 | 0.223601554 | -2.160997878 | 5.26971E-09 | 9.64961E-08 | K01728 |
| *SCPL24* | 794.2177667 | 343.7791787 | 0.432852541 | -1.208052465 | 5.46152E-09 | 9.98662E-08 | K16297 |
| *HCF173* | 717.6455912 | 308.1356088 | 0.429370169 | -1.219706133 | 5.55134E-09 | 1.01436E-07 | - |
| *CHI1* | 4356.368932 | 808.0594082 | 0.185489205 | -2.43059287 | 5.56223E-09 | 1.01563E-07 | K01859 |
| *ag4* | 95.10068536 | 5.055605118 | 0.053160554 | -4.233500051 | 6.00114E-09 | 1.09112E-07 | K18108 |
| *At3g47570* | 86.74786461 | 8.249704134 | 0.095099795 | -3.39441396 | 6.13643E-09 | 1.11414E-07 | - |
| *R9* | 33.80192579 | 0 | 0 | -Inf | 6.17904E-09 | 1.12109E-07 | K00430 |
| *ABCC3* | 728.2068267 | 313.9056371 | 0.431066595 | -1.214017329 | 6.22468E-09 | 1.12857E-07 | - |
| *CAS* | 9871.857561 | 3655.265001 | 0.370271246 | -1.433345575 | 6.31203E-09 | 1.1436E-07 | - |
| *XTH10* | 78.12538412 | 14.77791967 | 0.189156442 | -2.402348186 | 6.33172E-09 | 1.14636E-07 | K08235 |
| *UAF30* | 82.24996372 | 16.22263204 | 0.197235735 | -2.342007134 | 6.38164E-09 | 1.15458E-07 | K15223 |
| *LOX1.1* | 22.38718476 | 0 | 0 | -Inf | 7.05515E-09 | 1.26929E-07 | K15718 |
| *uncharacterized protein_19765* | 185.2925569 | 37.46021707 | 0.202167954 | -2.306373765 | 7.27758E-09 | 1.30581E-07 | - |
| *PAE8* | 249.5496182 | 88.58114007 | 0.354964038 | -1.494255226 | 7.27849E-09 | 1.30581E-07 | K19882 |
| *FTSH5* | 26678.9465 | 12845.31296 | 0.481477519 | -1.054459659 | 7.3321E-09 | 1.31372E-07 | K03798 |
| *At1g64390* | 40.90382966 | 3.246265284 | 0.079363358 | -3.655383117 | 7.33281E-09 | 1.31372E-07 | - |
| *uncharacterized protein_07225* | 190.5765662 | 61.54042819 | 0.32291708 | -1.630764346 | 7.89673E-09 | 1.40983E-07 | - |
| *uncharacterized protein_08769* | 100.059784 | 5.88271358 | 0.058791988 | -4.088236637 | 7.92281E-09 | 1.41351E-07 | - |
| *GYRBM* | 1059.050346 | 475.4213204 | 0.448912861 | -1.155492667 | 8.05427E-09 | 1.43596E-07 | K02470 |
| *HCT* | 46.50936195 | 0 | 0 | -Inf | 8.08358E-09 | 1.44019E-07 | K19747 |
| *GF14D* | 4123.528516 | 1956.478516 | 0.474467076 | -1.075620116 | 8.10896E-09 | 1.44371E-07 | K06630 |
| *dnaJ* | 623.7975096 | 266.0479971 | 0.42649737 | -1.22939125 | 8.17633E-09 | 1.4547E-07 | K03686 |
| *LTPG15* | 39.41261932 | 2.98093522 | 0.07563403 | -3.724820704 | 8.37189E-09 | 1.48743E-07 | - |
| *H2B* | 459.8073337 | 187.7518189 | 0.408327152 | -1.292202593 | 8.55485E-09 | 1.51889E-07 | K11252 |
| *At1g60630* | 28.93958343 | 0.874239594 | 0.030209128 | -5.048871626 | 8.85313E-09 | 1.56859E-07 | - |
| *KIN5A* | 516.8030179 | 123.7599345 | 0.239472159 | -2.062070157 | 8.94892E-09 | 1.58447E-07 | K10398 |
| *REC2* | 19455.66487 | 6439.022536 | 0.330958751 | -1.595276679 | 8.99118E-09 | 1.59086E-07 | K03255 |
| *CYP720B2* | 37.92837202 | 2.674885051 | 0.070524647 | -3.825728649 | 9.44644E-09 | 1.66911E-07 | - |
| *BHLH62* | 199.0984422 | 58.56594256 | 0.294155705 | -1.76534808 | 9.50943E-09 | 1.67908E-07 | - |
| *LAC3* | 38.24792043 | 0.295072441 | 0.007714732 | -7.018168317 | 9.60713E-09 | 1.69517E-07 | K05909 |
| *MAN2* | 61.60972295 | 9.406194113 | 0.152673858 | -2.711475038 | 9.9378E-09 | 1.74991E-07 | K19355 |
| *IMPL1* | 925.6728773 | 412.3924452 | 0.445505594 | -1.166484547 | 9.95507E-09 | 1.75175E-07 | K01092 |
| *POLX* | 370.0219124 | 107.371514 | 0.290176096 | -1.784999416 | 1.00332E-08 | 1.76429E-07 | - |
| *FRO2* | 157.8129651 | 47.76435317 | 0.302664316 | -1.724209501 | 1.03848E-08 | 1.82187E-07 | - |
| *NPF6.3* | 841.6060843 | 65.80226764 | 0.07818654 | -3.676935927 | 1.0389E-08 | 1.82187E-07 | K14638 |
| *At1g60630* | 66.7019213 | 8.242385649 | 0.123570438 | -3.016594446 | 1.04233E-08 | 1.82664E-07 | - |
| *CRK2* | 45.79388743 | 4.735357001 | 0.103405875 | -3.27360994 | 1.07013E-08 | 1.8728E-07 | - |
| *R72* | 73.11781063 | 0 | 0 | -Inf | 1.07675E-08 | 1.88182E-07 | K00430 |
| *uncharacterized protein_21490* | 423.936941 | 172.2777428 | 0.406375869 | -1.299113359 | 1.0778E-08 | 1.88237E-07 | - |
| *SIK1* | 26.77618383 | 0.582826396 | 0.021766597 | -5.521740332 | 1.08325E-08 | 1.89061E-07 | - |
| *PCMP-H40* | 386.1260275 | 153.8788601 | 0.398519782 | -1.327276757 | 1.08982E-08 | 1.90079E-07 | - |
| *SDR2a* | 990.0899196 | 445.0188473 | 0.449473163 | -1.153693118 | 1.10203E-08 | 1.91896E-07 | - |
| *THIC* | 1616.158202 | 750.1151972 | 0.464134759 | -1.107384351 | 1.14096E-08 | 1.98325E-07 | K03147 |
| *ISE2* | 733.8033642 | 321.7450945 | 0.438462278 | -1.189475366 | 1.15997E-08 | 2.01493E-07 | - |
| *PUB52* | 899.6312412 | 339.365532 | 0.377227375 | -1.406493719 | 1.20604E-08 | 2.09072E-07 | - |
| *FBP* | 1181.895143 | 539.9443608 | 0.456846247 | -1.130219391 | 1.20722E-08 | 2.09135E-07 | K03841 |
| *ZAR1* | 154.8961898 | 46.99585142 | 0.303402243 | -1.720696344 | 1.26791E-08 | 2.19291E-07 | - |
| *PSAL* | 4670.888201 | 953.1792824 | 0.2040681 | -2.292877415 | 1.30585E-08 | 2.25461E-07 | K02699 |
| *A6* | 246.2506631 | 88.79658722 | 0.360594307 | -1.471551475 | 1.32031E-08 | 2.27805E-07 | - |
| *SWEET3B* | 153.9080127 | 29.27999999 | 0.190243506 | -2.394080885 | 1.3224E-08 | 2.28012E-07 | K15382 |
| *IN37* | 5822.955733 | 2811.877431 | 0.48289521 | -1.050217942 | 1.32683E-08 | 2.28622E-07 | K12502 |
| *BAM2* | 539.4858186 | 155.7584291 | 0.288716448 | -1.792274797 | 1.37487E-08 | 2.36741E-07 | - |
| *uncharacterized protein_12783* | 1098.917775 | 501.3031027 | 0.456178901 | -1.132328374 | 1.42411E-08 | 2.4452E-07 | - |
| *Os07g0682400* | 738.8169008 | 166.3785774 | 0.225195955 | -2.150747183 | 1.4316E-08 | 2.45358E-07 | - |
| *ACA7* | 1941.634951 | 593.6611684 | 0.305753235 | -1.70956033 | 1.44934E-08 | 2.47903E-07 | K01674 |
| *DTX40* | 74.31532135 | 14.17817295 | 0.190783982 | -2.389988043 | 1.5486E-08 | 2.63652E-07 | K03327 |
| *UGT85A23* | 1527.500309 | 712.195622 | 0.466249085 | -1.100827199 | 1.57463E-08 | 2.67728E-07 | - |
| *At2g19130* | 128.5919213 | 17.96332431 | 0.139692479 | -2.839673743 | 1.74039E-08 | 2.94742E-07 | - |
| *uncharacterized protein_28014* | 584.503726 | 251.8426976 | 0.430865855 | -1.214689322 | 1.74989E-08 | 2.96156E-07 | - |
| *SRG1* | 29.69644062 | 1.181665656 | 0.039791491 | -4.651396224 | 1.75734E-08 | 2.96953E-07 | - |
| *TBL6* | 69.80719877 | 12.69502383 | 0.181858376 | -2.459112717 | 1.75807E-08 | 2.96953E-07 | - |
| *rglB* | 87.99708757 | 16.1019156 | 0.18298237 | -2.450223444 | 1.85204E-08 | 3.11393E-07 | K18195 |
| *PDR17* | 32.91557246 | 0.591520774 | 0.017970849 | -5.798197642 | 1.93971E-08 | 3.25706E-07 | - |
| *DIS1* | 134.5132976 | 38.94189292 | 0.28950218 | -1.788353882 | 2.00281E-08 | 3.35862E-07 | K04506 |
| *OPR11* | 144.2036417 | 43.44947856 | 0.301306389 | -1.73069683 | 2.10318E-08 | 3.52235E-07 | K05894 |
| *uncharacterized protein_35375* | 35.25344028 | 2.364707204 | 0.067077346 | -3.898030586 | 2.1087E-08 | 3.52929E-07 | - |
| *TLP40* | 767.1235992 | 343.0399103 | 0.447176844 | -1.16108261 | 2.12692E-08 | 3.55516E-07 | - |
| *MDHP* | 9449.457138 | 4638.823243 | 0.490908967 | -1.026472575 | 2.32105E-08 | 3.86206E-07 | K00051 |
| *DMP4* | 30.84721342 | 1.49046761 | 0.048317739 | -4.371303241 | 2.38662E-08 | 3.96602E-07 | - |
| *PME53* | 25.75846063 | 0.586485639 | 0.02276866 | -5.456806786 | 2.39944E-08 | 3.98475E-07 | K01051 |
| *uncharacterized protein_39659* | 30.79535023 | 1.49046761 | 0.048399112 | -4.368875607 | 2.41564E-08 | 4.00906E-07 | - |
| *T5AT* | 25.6391573 | 0.591520774 | 0.023070991 | -5.437776197 | 2.47872E-08 | 4.10579E-07 | K19861 |
| *RER6* | 656.1565996 | 289.8981333 | 0.441812417 | -1.178494129 | 2.5264E-08 | 4.18207E-07 | - |
| *nep1* | 34.92176049 | 2.345942556 | 0.067177099 | -3.895886699 | 2.64096E-08 | 4.36328E-07 | - |
| *ACLB-1* | 4401.393729 | 2144.876075 | 0.487317474 | -1.03706614 | 2.67377E-08 | 4.41465E-07 | K01648 |
| *At2g18940* | 473.8899862 | 200.9464911 | 0.424036162 | -1.23774079 | 2.75474E-08 | 4.53959E-07 | K17710 |
| *P67* | 459.7590747 | 194.1140697 | 0.42220824 | -1.24397336 | 2.76384E-08 | 4.54874E-07 | - |
| *KCS11* | 74.46732152 | 2.933804087 | 0.039397202 | -4.665763028 | 2.83587E-08 | 4.65833E-07 | K15397 |
| *At3g47110* | 110.1219282 | 13.08207519 | 0.118796278 | -3.07343846 | 2.87915E-08 | 4.72641E-07 | - |
| *At3g47110* | 31.94788177 | 1.776845673 | 0.05561701 | -4.168329993 | 3.0456E-08 | 4.98373E-07 | K13420 |
| *PMEI10* | 239.8955386 | 69.53350579 | 0.289849099 | -1.786626092 | 3.11829E-08 | 5.09619E-07 | - |
| *At3g02645* | 20.5261777 | 0 | 0 | -Inf | 3.21628E-08 | 5.24632E-07 | - |
| *CCR1* | 1652.72313 | 788.1486003 | 0.476878786 | -1.068305488 | 3.24964E-08 | 5.29737E-07 | K09753 |
| *DF1* | 251.5193762 | 94.11160922 | 0.374172402 | -1.41822494 | 3.27058E-08 | 5.32812E-07 | - |
| *SG1* | 547.6264574 | 238.6358757 | 0.435763964 | -1.1983812 | 3.32366E-08 | 5.41117E-07 | - |
| *SOQ1* | 1810.199362 | 867.1000475 | 0.479008039 | -1.061878225 | 3.43487E-08 | 5.57808E-07 | - |
| *TIP1-1* | 865.09676 | 314.2654739 | 0.363272051 | -1.460877723 | 3.45123E-08 | 5.60111E-07 | K09873 |
| *At1g17220* | 2653.170001 | 1289.162533 | 0.485895186 | -1.041282954 | 3.52609E-08 | 5.71538E-07 | K02519 |
| *CHLH* | 19796.93083 | 7716.189109 | 0.389766938 | -1.359316376 | 3.58979E-08 | 5.8113E-07 | K03403 |
| *NRT2.1* | 40.28006514 | 3.817645517 | 0.094777541 | -3.399310962 | 3.61963E-08 | 5.85223E-07 | K02575 |
| *ASP5* | 5348.790149 | 1990.973817 | 0.372228815 | -1.425738355 | 3.63516E-08 | 5.86995E-07 | K00811 |
| *PRS1* | 429.4661534 | 181.1262862 | 0.421747523 | -1.245548501 | 3.65354E-08 | 5.89592E-07 | K00948 |
| *CCB1* | 654.112791 | 292.0057069 | 0.446414916 | -1.163542861 | 3.76087E-08 | 6.06532E-07 | - |
| *TUBB5* | 824.9793843 | 376.781849 | 0.456716684 | -1.130628601 | 3.80823E-08 | 6.13784E-07 | K07375 |
| *LAX4* | 833.7263942 | 380.7087263 | 0.456635089 | -1.13088637 | 3.9693E-08 | 6.38543E-07 | K13946 |
| *CM1* | 1731.950311 | 422.3398677 | 0.243852185 | -2.035921195 | 3.97935E-08 | 6.39759E-07 | K01850 |
| *XTH31* | 21.96900637 | 0 | 0 | -Inf | 3.98632E-08 | 6.40479E-07 | K08235 |
| *uncharacterized protein_35696* | 55.90236687 | 8.536082197 | 0.152696257 | -2.711263394 | 4.02571E-08 | 6.46403E-07 | - |
| *HCT* | 108.8758342 | 29.3028629 | 0.269140192 | -1.893570244 | 4.04975E-08 | 6.4962E-07 | K13065 |
| *uncharacterized protein_14385* | 343.4890143 | 139.6288488 | 0.406501643 | -1.29866691 | 4.0508E-08 | 6.4962E-07 | - |
| *PXG* | 157.5725348 | 23.85563972 | 0.151394656 | -2.723613813 | 4.06377E-08 | 6.51293E-07 | K17991 |
| *IDD14* | 42.26888153 | 4.406882941 | 0.104258329 | -3.261765458 | 4.26575E-08 | 6.82388E-07 | - |
| *PHOS34* | 276.1616345 | 106.8087896 | 0.386761868 | -1.370482533 | 4.37943E-08 | 7.00137E-07 | - |
| *TOGT1* | 2142.116369 | 359.7516924 | 0.16794218 | -2.573963476 | 4.46573E-08 | 7.12789E-07 | - |
| *APD2* | 659.6605148 | 296.6006662 | 0.449626224 | -1.153201914 | 4.46689E-08 | 7.12789E-07 | - |
| *HSL1* | 1373.800123 | 653.1221173 | 0.475412767 | -1.072747449 | 4.48966E-08 | 7.15089E-07 | - |
| *RER4* | 3959.791389 | 1950.476969 | 0.492570637 | -1.021597465 | 4.60013E-08 | 7.31776E-07 | - |
| *LECRK72* | 35.54370144 | 2.081988384 | 0.058575452 | -4.093560004 | 4.61277E-08 | 7.3288E-07 | - |
| *At1g54780* | 2391.453393 | 1164.51304 | 0.48694783 | -1.03816088 | 4.86569E-08 | 7.71156E-07 | - |
| *FBN5* | 759.4377847 | 347.2985113 | 0.457310024 | -1.128755553 | 5.036E-08 | 7.95695E-07 | - |
| *uncharacterized protein_09320* | 132.2582741 | 29.82163784 | 0.225480319 | -2.148926581 | 5.11793E-08 | 8.07645E-07 | - |
| *TERC* | 385.1203779 | 161.0849744 | 0.41827175 | -1.257487533 | 5.23073E-08 | 8.24776E-07 | - |
| *PDH-E1 BETA* | 891.1116953 | 413.402165 | 0.463917337 | -1.108060332 | 5.23291E-08 | 8.24776E-07 | K00162 |
| *CYP71AU50* | 221.0296336 | 24.09770642 | 0.109024777 | -3.197272061 | 5.24101E-08 | 8.25548E-07 | - |
| *At3g61590* | 225.5744527 | 57.90859399 | 0.256716101 | -1.961754311 | 5.45047E-08 | 8.5644E-07 | - |
| *POSF21* | 348.6366151 | 143.0704795 | 0.410371353 | -1.284998071 | 5.52002E-08 | 8.6626E-07 | - |
| *At2g25737* | 192.3736076 | 43.67862581 | 0.22705103 | -2.138911511 | 5.52308E-08 | 8.6626E-07 | - |
| *uncharacterized protein_34540* | 107.2101485 | 29.03481046 | 0.270821474 | -1.884585957 | 5.53368E-08 | 8.67393E-07 | - |
| *tmem45b* | 115.5265032 | 6.494813918 | 0.056219255 | -4.15279186 | 5.61249E-08 | 8.7921E-07 | - |
| *UGT91C1* | 378.4401127 | 136.7105303 | 0.361247462 | -1.468940642 | 5.69515E-08 | 8.91616E-07 | - |
| *LPA3* | 261.0039543 | 101.0026079 | 0.386977309 | -1.36967912 | 5.91493E-08 | 9.24896E-07 | - |
| *LECRK41* | 87.80366877 | 5.039592255 | 0.057396147 | -4.122902305 | 6.04965E-08 | 9.45387E-07 | - |
| *uncharacterized protein_27126* | 61.76875809 | 10.91955405 | 0.176781182 | -2.499963386 | 6.15259E-08 | 9.60304E-07 | - |
| *LRL1* | 328.2016581 | 131.0418494 | 0.399272357 | -1.324554904 | 6.28125E-08 | 9.78602E-07 | - |
| *BOR1* | 71.3190465 | 14.51534139 | 0.203526857 | -2.296708911 | 6.31969E-08 | 9.83995E-07 | - |
| *PLP7* | 248.2273694 | 44.88546714 | 0.180824005 | -2.46734188 | 6.32586E-08 | 9.84359E-07 | - |
| *AAPC* | 201.8462342 | 72.53461097 | 0.35935578 | -1.476515203 | 6.3537E-08 | 9.88092E-07 | K01792 |
| *DEGP1* | 582.7711326 | 260.6515937 | 0.447262363 | -1.160806735 | 6.47944E-08 | 1.00643E-06 | - |
| *AATL1* | 231.443908 | 87.18083646 | 0.376682356 | -1.408579638 | 6.49076E-08 | 1.00758E-06 | - |
| *Os06g0194400* | 63.94425311 | 11.775029 | 0.184145227 | -2.441084093 | 6.86532E-08 | 1.06315E-06 | - |
| *RPL15* | 3771.624634 | 1875.00943 | 0.497135747 | -1.00828825 | 6.98629E-08 | 1.08058E-06 | K02876 |
| *uncharacterized protein_00298* | 579.9875868 | 259.3971437 | 0.447246027 | -1.16085943 | 7.00608E-08 | 1.08299E-06 | - |
| *PMIR1* | 70.11117024 | 14.15712496 | 0.201923957 | -2.308116004 | 7.20676E-08 | 1.11267E-06 | - |
| *CHS* | 646.0336901 | 155.0546329 | 0.240010135 | -2.058832769 | 7.46455E-08 | 1.14971E-06 | K00660 |
| *SPAC24B11.05* | 113.1560464 | 15.74460662 | 0.139140657 | -2.845384059 | 7.66795E-08 | 1.1802E-06 | K07025 |
| *CRK8* | 390.0455533 | 63.45823739 | 0.162694426 | -2.619763271 | 7.88269E-08 | 1.21049E-06 | - |
| *KCS9* | 28.05831942 | 0.300107576 | 0.01069585 | -6.54680505 | 7.90537E-08 | 1.21324E-06 | K15397 |
| *ag4* | 19.43588199 | 0 | 0 | -Inf | 7.96227E-08 | 1.21979E-06 | - |
| *ROQ1* | 71.28096554 | 10.97581858 | 0.153979656 | -2.699188338 | 8.02575E-08 | 1.22783E-06 | - |
| *uncharacterized protein_28771* | 38.95126356 | 3.840069408 | 0.098586517 | -3.342465837 | 8.02908E-08 | 1.22783E-06 | - |
| *LECRK41* | 21.99840151 | 0.300107576 | 0.013642245 | -6.195775146 | 8.12639E-08 | 1.24197E-06 | - |
| *At1g73050* | 57.71843782 | 9.778608499 | 0.169419147 | -2.561331164 | 8.21109E-08 | 1.25417E-06 | - |
| *NIA2* | 121.6505307 | 36.04289575 | 0.296282273 | -1.754955784 | 8.31225E-08 | 1.26887E-06 | K10534 |
| *DAD2* | 20.12529904 | 0 | 0 | -Inf | 8.35939E-08 | 1.2753E-06 | - |
| *NPF3.1* | 556.988704 | 249.5786637 | 0.448085683 | -1.158153465 | 8.59633E-08 | 1.30834E-06 | K14638 |
| *SWEET1* | 123.1433559 | 25.40696798 | 0.20632025 | -2.277042668 | 8.67922E-08 | 1.31939E-06 | K15382 |
| *A6* | 57.25014789 | 0.300107576 | 0.00524204 | -7.57565587 | 9.11332E-08 | 1.38048E-06 | - |
| *EXO70A1* | 23.90434468 | 0.600215152 | 0.02510904 | -5.315649306 | 9.44712E-08 | 1.42768E-06 | K07195 |
| *BOR2* | 40.74138493 | 4.424271697 | 0.108594043 | -3.202983133 | 9.49458E-08 | 1.43401E-06 | - |
| *AAF* | 2191.031381 | 1084.553539 | 0.494996808 | -1.014508874 | 9.95698E-08 | 1.50297E-06 | - |
| *CHS* | 8915.520395 | 2515.438825 | 0.282141559 | -1.825508906 | 1.0308E-07 | 1.55231E-06 | K00660 |
| *ACT7* | 30.84369593 | 0.600215152 | 0.019459897 | -5.683352101 | 1.04914E-07 | 1.579E-06 | K10355 |
| *SUN2* | 589.53794 | 267.2584297 | 0.45333542 | -1.141349208 | 1.05185E-07 | 1.58215E-06 | K19347 |
| *SUFE1* | 833.1114806 | 392.3550168 | 0.470951398 | -1.086349912 | 1.06521E-07 | 1.60037E-06 | - |
| *CYP71AU50* | 28.5663052 | 1.485432475 | 0.051999461 | -4.265359519 | 1.06871E-07 | 1.60469E-06 | - |
| *TUBB2* | 249.9343157 | 43.07706418 | 0.17235354 | -2.536557161 | 1.0858E-07 | 1.6275E-06 | K07375 |
| *murA* | 160.9387862 | 54.62060375 | 0.339387447 | -1.558994891 | 1.09033E-07 | 1.63334E-06 | - |
| *CRK8* | 360.4845426 | 18.76901373 | 0.052066071 | -4.263512652 | 1.09648E-07 | 1.64063E-06 | - |
| *uncharacterized protein_35297* | 219.8825368 | 82.70430111 | 0.376129466 | -1.410698764 | 1.11987E-07 | 1.67174E-06 | K15032 |
| *uncharacterized protein_50712* | 268.5150342 | 96.28736202 | 0.35859207 | -1.479584509 | 1.14528E-07 | 1.70769E-06 | - |
| *BXL4* | 1112.309706 | 39.67805672 | 0.035671771 | -4.809073361 | 1.15809E-07 | 1.72578E-06 | K15920 |
| *YAB5* | 285.0851014 | 20.03571086 | 0.070279754 | -3.830747044 | 1.16362E-07 | 1.73302E-06 | - |
| *IRK* | 302.357095 | 48.03938246 | 0.158882934 | -2.653963922 | 1.17067E-07 | 1.74149E-06 | - |
| *LRK10* | 36.08803831 | 3.252676312 | 0.090131702 | -3.471821557 | 1.19292E-07 | 1.77358E-06 | - |
| *RUN1* | 25.35065488 | 0.895287593 | 0.035316152 | -4.823528013 | 1.19853E-07 | 1.77985E-06 | - |
| *BRG3* | 423.4048075 | 178.237701 | 0.420962865 | -1.248235121 | 1.23165E-07 | 1.82377E-06 | K19042 |
| *HHT1* | 236.3665477 | 44.59818162 | 0.188682291 | -2.405969075 | 1.26089E-07 | 1.86491E-06 | K15400 |
| *CYP75A1* | 45.3877685 | 2.068258871 | 0.04556864 | -4.455814885 | 1.26366E-07 | 1.86793E-06 | K13083 |
| *At1g34300* | 164.0852665 | 56.62947526 | 0.345122243 | -1.534820636 | 1.27666E-07 | 1.88606E-06 | - |
| *A6* | 68.68229653 | 2.951192843 | 0.042968756 | -4.540568179 | 1.29897E-07 | 1.91352E-06 | - |
| *EIX2* | 41.2476768 | 4.709742302 | 0.114182002 | -3.130592836 | 1.32453E-07 | 1.95006E-06 | - |
| *IMPL1* | 1326.13678 | 646.397774 | 0.487429188 | -1.036735451 | 1.33566E-07 | 1.96531E-06 | K01092 |
| *HHT1* | 427.3371739 | 21.57055782 | 0.050476671 | -4.308239432 | 1.34471E-07 | 1.97751E-06 | K15400 |
| *CYP720B2* | 244.6884014 | 45.36174563 | 0.185385761 | -2.431397653 | 1.3494E-07 | 1.98326E-06 | - |
| *OPT7* | 371.8096708 | 159.2968002 | 0.428436409 | -1.222847007 | 1.35271E-07 | 1.98699E-06 | - |
| *CYP735A2* | 76.82261114 | 3.565137509 | 0.046407398 | -4.429501373 | 1.3787E-07 | 2.02286E-06 | K10717 |
| *MANA* | 46.12050678 | 5.045534848 | 0.109398946 | -3.192329251 | 1.39549E-07 | 2.04282E-06 | K01191 |
| *GLOX* | 688.008028 | 83.85403843 | 0.121879448 | -3.036473228 | 1.45307E-07 | 2.12348E-06 | - |
| *IGS1* | 18.65831549 | 0 | 0 | -Inf | 1.48624E-07 | 2.16949E-06 | - |
| *CXE17* | 141.0241768 | 36.87181913 | 0.261457432 | -1.935352015 | 1.49475E-07 | 2.17943E-06 | - |
| *uncharacterized protein_13235* | 375.8205398 | 161.8141999 | 0.430562417 | -1.215705701 | 1.4965E-07 | 2.18074E-06 | - |
| *AXY4L* | 197.664695 | 73.03186947 | 0.369473514 | -1.436457147 | 1.53977E-07 | 2.24253E-06 | - |
| *RAP2-13* | 24.89791302 | 0.885217322 | 0.035553876 | -4.813849325 | 1.59933E-07 | 2.32269E-06 | K09286 |
| *FPP7* | 40.86754341 | 4.708366409 | 0.115210409 | -3.117657022 | 1.61203E-07 | 2.33981E-06 | - |
| *uncharacterized protein_37386* | 1133.092807 | 551.6088613 | 0.486817018 | -1.038548493 | 1.63121E-07 | 2.36365E-06 | - |
| *At4g34480* | 225.976349 | 19.82714317 | 0.087739904 | -3.510623062 | 1.64649E-07 | 2.38444E-06 | - |
| *WAXY* | 2510.818313 | 888.4457071 | 0.353847072 | -1.498802116 | 1.67398E-07 | 2.42082E-06 | K13679 |
| *CLSC5* | 47.33696097 | 6.810934357 | 0.143881952 | -2.797042455 | 1.67444E-07 | 2.42082E-06 | - |
| *uncharacterized protein_06770* | 177.9400877 | 56.72413798 | 0.31878223 | -1.649356883 | 1.73964E-07 | 2.51085E-06 | - |
| *CESA1* | 3905.943945 | 1653.766592 | 0.423397421 | -1.239915614 | 1.76481E-07 | 2.54431E-06 | K10999 |
| *At1g67720* | 33.00716746 | 2.656120402 | 0.080471019 | -3.635386887 | 1.76794E-07 | 2.54598E-06 | - |
| *PME31* | 2272.66833 | 926.1937237 | 0.407535808 | -1.295001269 | 1.778E-07 | 2.55902E-06 | K01051 |
| *ANR* | 4607.157248 | 564.3388796 | 0.122491777 | -3.029243188 | 1.78142E-07 | 2.56252E-06 | K08695 |
| *DSE4* | 132.9773309 | 42.46218264 | 0.319318957 | -1.64692989 | 1.79663E-07 | 2.58295E-06 | K01180 |
| *LEC* | 3342.660645 | 2.954852086 | 0.000883982 | -10.14369527 | 1.81937E-07 | 2.61417E-06 | - |
| *PUB9* | 66.2613397 | 13.56926342 | 0.204784019 | -2.287824963 | 1.82357E-07 | 2.61874E-06 | - |
| *SPL12* | 103.5176015 | 29.37516971 | 0.283769806 | -1.817207004 | 1.83655E-07 | 2.63444E-06 | - |
| *At1g04430* | 467.5876706 | 209.828427 | 0.448746706 | -1.156026746 | 1.85243E-07 | 2.65574E-06 | - |
| *DCOR* | 22.66021813 | 0.295072441 | 0.013021606 | -6.262948756 | 1.87822E-07 | 2.69121E-06 | K01581 |
| *uncharacterized protein_49112* | 53.12378068 | 8.851295179 | 0.166616439 | -2.585397343 | 1.88996E-07 | 2.70351E-06 | - |
| *CAS1* | 2911.256384 | 606.854009 | 0.208450899 | -2.262220504 | 1.95389E-07 | 2.78854E-06 | K13034 |
| *D27* | 433.986069 | 140.9764791 | 0.324841024 | -1.622194253 | 1.95483E-07 | 2.78854E-06 | - |
| *YBEY* | 348.2805287 | 148.9627655 | 0.42770914 | -1.225298056 | 1.98864E-07 | 2.83519E-06 | - |
| *LOX1.1* | 743.7858011 | 48.58374269 | 0.065319535 | -3.936341668 | 1.99256E-07 | 2.8392E-06 | K15718 |
| *UBC8* | 4447.073579 | 2043.063061 | 0.459417418 | -1.12212254 | 1.99499E-07 | 2.83952E-06 | K06689 |
| *PT5* | 87.45623222 | 9.444630868 | 0.107992657 | -3.210994879 | 2.03033E-07 | 2.88663E-06 | K12742 |
| *At2g42960* | 164.576255 | 16.59091875 | 0.100809918 | -3.310290514 | 2.05995E-07 | 2.9255E-06 | - |
| *PAP10* | 369.0607173 | 160.1390232 | 0.433909695 | -1.204533274 | 2.10478E-07 | 2.98586E-06 | - |
| *LDOX* | 14040.91292 | 617.3157393 | 0.043965499 | -4.507484355 | 2.21159E-07 | 3.13046E-06 | K05277 |
| *CYCU2-1* | 163.4424435 | 43.88093868 | 0.268479458 | -1.897116386 | 2.26445E-07 | 3.19876E-06 | - |
| *dnaJ* | 1434.376889 | 198.4893954 | 0.138380224 | -2.853290313 | 2.26482E-07 | 3.19876E-06 | K09510 |
| *ROC1* | 16529.42832 | 7550.983892 | 0.456820632 | -1.130300284 | 2.28878E-07 | 3.22905E-06 | K11294 |
| *RBCS* | 2673.632235 | 1284.89591 | 0.480580647 | -1.057149542 | 2.33848E-07 | 3.29736E-06 | K01602 |
| *UNI* | 119.3058654 | 36.63760733 | 0.307089741 | -1.703267777 | 2.34235E-07 | 3.301E-06 | K13459 |
| *FAR1* | 40.59106448 | 1.183041548 | 0.029145369 | -5.100589528 | 2.36934E-07 | 3.33721E-06 | K13356 |
| *DTX44* | 86.35390593 | 22.22109491 | 0.257325881 | -1.95833153 | 2.42094E-07 | 3.40615E-06 | - |
| *MYB123* | 20.62994005 | 0.291413198 | 0.014125741 | -6.1455296 | 2.49392E-07 | 3.50117E-06 | K09422 |
| *PSRP6* | 3317.486345 | 1395.602949 | 0.420680842 | -1.249201976 | 2.49943E-07 | 3.50699E-06 | K19035 |
| *IGHMBP2* | 3355.658604 | 1435.707115 | 0.427846597 | -1.224834479 | 2.51527E-07 | 3.52728E-06 | - |
| *SUS3* | 270.2082843 | 53.7916124 | 0.199074623 | -2.328618768 | 2.64967E-07 | 3.7021E-06 | K00695 |
| *ROQ1* | 53.28626137 | 9.180676697 | 0.172289751 | -2.537091213 | 2.67598E-07 | 3.73634E-06 | - |
| *At1g18000* | 103.7629265 | 30.17300423 | 0.290787907 | -1.781960822 | 2.86452E-07 | 3.98875E-06 | - |
| *PMEU1* | 81.64708123 | 20.45616383 | 0.250543725 | -1.996865692 | 2.91409E-07 | 4.05558E-06 | K01051 |
| *PCMP-H32* | 59.37902707 | 11.55317082 | 0.194566523 | -2.361664595 | 2.93904E-07 | 4.08808E-06 | - |
| *uncharacterized protein_42501* | 263.198256 | 108.2385619 | 0.411243462 | -1.281935353 | 2.95453E-07 | 4.10518E-06 | - |
| *ALDH3H1* | 1072.139703 | 529.3115469 | 0.493696433 | -1.018303874 | 3.04541E-07 | 4.21777E-06 | K00128 |
| *At1g30440* | 94.4717383 | 25.96095946 | 0.274801331 | -1.863539101 | 3.06054E-07 | 4.23415E-06 | - |
| *CRK2* | 50.23998997 | 4.127823363 | 0.082162106 | -3.605383034 | 3.07096E-07 | 4.24629E-06 | - |
| *CHIT5* | 77.91645322 | 18.90299125 | 0.2426059 | -2.043313459 | 3.08222E-07 | 4.25957E-06 | K01183 |
| *RSH3* | 585.1556522 | 242.0839357 | 0.413708617 | -1.273313087 | 3.21565E-07 | 4.43204E-06 | - |
| *LOX1.1* | 50.86196688 | 5.612348381 | 0.110344698 | -3.179910783 | 3.22364E-07 | 4.44067E-06 | K15718 |
| *ANR* | 109.4806411 | 16.2596929 | 0.148516603 | -2.751303873 | 3.30743E-07 | 4.55165E-06 | K13082 |
| *FLS* | 103.4155548 | 21.75454506 | 0.210360473 | -2.249064447 | 3.30982E-07 | 4.55194E-06 | K05278 |
| *uncharacterized protein_36644* | 60.63855075 | 3.857458164 | 0.063613957 | -3.974512858 | 3.3115E-07 | 4.55194E-06 | - |
| *GSVIVT00023967001* | 71.27748402 | 7.939526287 | 0.111388981 | -3.166321576 | 3.33723E-07 | 4.58486E-06 | K00430 |
| *SCP26* | 267.0206229 | 110.2555234 | 0.412910142 | -1.276100239 | 3.35136E-07 | 4.60113E-06 | K16297 |
| *CYP75B2* | 154.4242168 | 33.70427168 | 0.218257683 | -2.195895658 | 3.35266E-07 | 4.60113E-06 | - |
| *MCM7* | 31.99454067 | 2.687238672 | 0.083990538 | -3.573629387 | 3.36083E-07 | 4.60988E-06 | K02210 |
| *KIN7A* | 872.9358699 | 319.3039822 | 0.365781718 | -1.450945125 | 3.46654E-07 | 4.74475E-06 | K11498 |
| *TIP1-1* | 816.9464787 | 396.7929592 | 0.485702515 | -1.041855139 | 3.49843E-07 | 4.78329E-06 | K09873 |
| *At3g28510* | 17.73042855 | 0 | 0 | -Inf | 3.50054E-07 | 4.78364E-06 | K08900 |
| *PCMP-H40* | 118.6266843 | 37.28220183 | 0.314281749 | -1.6698696 | 3.50994E-07 | 4.79393E-06 | - |
| *relA* | 429.1914403 | 194.5610743 | 0.453320025 | -1.141398204 | 3.62292E-07 | 4.93775E-06 | - |
| *TBL14* | 248.3759818 | 101.2716266 | 0.407735184 | -1.294295641 | 3.66246E-07 | 4.98635E-06 | - |
| *GSTU17* | 65.11928868 | 13.89317078 | 0.213349548 | -2.228709043 | 3.67153E-07 | 4.99604E-06 | K00799 |
| *XB3* | 145.687774 | 20.05309961 | 0.137644355 | -2.860982656 | 3.69042E-07 | 5.01643E-06 | - |
| *CSLH1* | 2491.658659 | 446.3608841 | 0.179142068 | -2.480823932 | 3.84667E-07 | 5.20953E-06 | - |
| *At1g54570* | 471.8059854 | 216.7799152 | 0.459468345 | -1.121962624 | 3.85875E-07 | 5.22314E-06 | - |
| *DMP6* | 76.95031259 | 10.06041986 | 0.130739168 | -2.935236671 | 3.87229E-07 | 5.2387E-06 | - |
| *ALKE* | 101.4959373 | 29.53845059 | 0.291030867 | -1.780755922 | 3.99691E-07 | 5.39335E-06 | - |
| *At2g24580* | 72.21257866 | 5.057888469 | 0.070041654 | -3.835643031 | 4.00128E-07 | 5.39616E-06 | K00306 |
| *At1g74320* | 174.9179711 | 64.34842187 | 0.367877705 | -1.442701848 | 4.0602E-07 | 5.46987E-06 | K14156 |
| *OPR7* | 41.83860772 | 5.664514649 | 0.135389655 | -2.884810591 | 4.09755E-07 | 5.51731E-06 | K05894 |
| *uncharacterized protein_38924* | 1088.256669 | 542.0292278 | 0.498071129 | -1.005576308 | 4.12379E-07 | 5.54972E-06 | - |
| *ABCB14* | 620.3260755 | 294.4949663 | 0.474742201 | -1.074783795 | 4.15564E-07 | 5.58674E-06 | K05658 |
| *Ta1476* | 322.2664783 | 139.3076932 | 0.432274849 | -1.209979196 | 4.16041E-07 | 5.5873E-06 | - |
| *DJC76* | 778.1181514 | 377.7171146 | 0.485423857 | -1.042683079 | 4.23879E-07 | 5.68959E-06 | - |
| *GGPS* | 31.56258005 | 2.685862779 | 0.085096427 | -3.554757637 | 4.2433E-07 | 5.69267E-06 | K13789 |
| *TTL1* | 272.0177235 | 45.33109579 | 0.166647582 | -2.585127713 | 4.31987E-07 | 5.78935E-06 | - |
| *CBP1* | 91.90918282 | 25.42986031 | 0.276684652 | -1.853685474 | 4.35986E-07 | 5.83888E-06 | K16296 |
| *ABCG3* | 87.97472031 | 23.68688469 | 0.26924649 | -1.893000555 | 4.36138E-07 | 5.83888E-06 | - |
| *TPS1* | 216.279826 | 85.50500572 | 0.395344343 | -1.338818314 | 4.4309E-07 | 5.92887E-06 | K16055 |
| *PCMP-H44* | 87.04163616 | 23.37989765 | 0.268605907 | -1.896437062 | 4.47081E-07 | 5.97915E-06 | - |
| *BGLU13* | 39.08428429 | 0.590144882 | 0.015099288 | -6.049375627 | 4.57944E-07 | 6.11806E-06 | K01188 |
| *At5g10770* | 19.77283801 | 0.295072441 | 0.01492312 | -6.066306964 | 4.60369E-07 | 6.14726E-06 | - |
| *ORP1D* | 102.0522328 | 30.16384142 | 0.295572577 | -1.758415673 | 4.67136E-07 | 6.23439E-06 | K20456 |
| *uncharacterized protein_15395* | 445.4703903 | 204.4897026 | 0.459042188 | -1.123301346 | 4.68124E-07 | 6.24433E-06 | - |
| *At1g71060* | 355.9490789 | 157.8378311 | 0.443428121 | -1.173227832 | 4.79636E-07 | 6.39457E-06 | - |
| *At2g25060* | 45.93379218 | 2.9475336 | 0.064169176 | -3.96197573 | 4.80098E-07 | 6.39741E-06 | - |
| *FL3H* | 2023.863563 | 621.6186972 | 0.307144567 | -1.703010232 | 4.85044E-07 | 6.45662E-06 | K00475 |
| *CXE6* | 83.37144927 | 21.93199447 | 0.263063611 | -1.926516399 | 4.86608E-07 | 6.47408E-06 | - |
| *SOT6* | 17.28633655 | 0 | 0 | -Inf | 4.88493E-07 | 6.49579E-06 | K01016 |
| *NRT2.1* | 30.14742373 | 2.37202569 | 0.078680875 | -3.667843181 | 5.04784E-07 | 6.70202E-06 | K02575 |
| *LTPG16* | 30.13012401 | 2.366083097 | 0.07852882 | -3.670633964 | 5.07032E-07 | 6.72839E-06 | - |
| *TPR2* | 175.6711381 | 65.51272819 | 0.372928239 | -1.423030049 | 5.10326E-07 | 6.76806E-06 | - |
| *LSI2* | 51.67409685 | 9.203100588 | 0.178098915 | -2.489249367 | 5.10548E-07 | 6.76806E-06 | - |
| *DRE21* | 119.5547439 | 37.53977439 | 0.313996527 | -1.671179492 | 5.19206E-07 | 6.87219E-06 | - |
| *GATB* | 620.1052001 | 296.7827503 | 0.478600648 | -1.063105746 | 5.25761E-07 | 6.95179E-06 | K02434 |
| *RBM39* | 672.4541264 | 209.3125306 | 0.311266631 | -1.683777171 | 5.4366E-07 | 7.17E-06 | - |
| *murA* | 242.6100152 | 99.61684387 | 0.410604829 | -1.2841775 | 5.46374E-07 | 7.20136E-06 | - |
| *NIA* | 1857.839977 | 517.0368105 | 0.27829997 | -1.84528734 | 5.46598E-07 | 7.20136E-06 | K10534 |
| *FMO1* | 794.7705878 | 390.8134852 | 0.491731188 | -1.024058235 | 5.64041E-07 | 7.41216E-06 | K00485 |
| *PRS1* | 209.1726819 | 82.78164305 | 0.39575743 | -1.337311658 | 5.73873E-07 | 7.52981E-06 | K00948 |
| *TOGT1* | 71.33122094 | 3.23025242 | 0.045285253 | -4.464814857 | 5.80544E-07 | 7.60957E-06 | K13496 |
| *At1g74460* | 28.72874993 | 2.090682762 | 0.072773189 | -3.780449148 | 5.84731E-07 | 7.66054E-06 | - |
| *uncharacterized protein_13448* | 185.9025174 | 71.27697926 | 0.383410511 | -1.383038205 | 6.01124E-07 | 7.85928E-06 | - |
| *SULTR3;1* | 28.56975076 | 2.095717897 | 0.073354434 | -3.768972015 | 6.03117E-07 | 7.88132E-06 | K17471 |
| *TRN1* | 76.3887768 | 19.18389515 | 0.251134996 | -1.993465011 | 6.30139E-07 | 8.20522E-06 | - |
| *PAP29* | 3298.732917 | 1528.422162 | 0.463336136 | -1.109868892 | 6.35915E-07 | 8.27204E-06 | - |
| *uncharacterized protein_22790* | 34.2201761 | 3.541337725 | 0.103486835 | -3.272480844 | 6.38861E-07 | 8.30018E-06 | - |
| *PAE8* | 20.64196354 | 0 | 0 | -Inf | 6.58077E-07 | 8.53009E-06 | K19882 |
| *Os05g0428700* | 412.407871 | 189.101029 | 0.458529146 | -1.124914653 | 6.60745E-07 | 8.55604E-06 | - |
| *BANGLUC* | 17.53514312 | 0 | 0 | -Inf | 6.62436E-07 | 8.57363E-06 | - |
| *uncharacterized protein_46533* | 44.51522628 | 6.830167441 | 0.153434409 | -2.704306038 | 6.67393E-07 | 8.62909E-06 | - |
| *uncharacterized protein_31610* | 46.90668717 | 7.70944217 | 0.164356995 | -2.605095232 | 6.78719E-07 | 8.74472E-06 | - |
| *AAP2* | 52.68330696 | 8.868683935 | 0.168339545 | -2.57055397 | 6.95859E-07 | 8.96106E-06 | - |
| *uncharacterized protein_41388* | 1047.33187 | 434.110936 | 0.414492243 | -1.270582993 | 7.09867E-07 | 9.13687E-06 | - |
| *KO* | 270.4745633 | 115.1422173 | 0.425704421 | -1.232076023 | 7.15316E-07 | 9.2024E-06 | K04122 |
| *Kdsr* | 136.8320421 | 23.66355334 | 0.172938684 | -2.531667476 | 7.20701E-07 | 9.26703E-06 | K04708 |
| *GATA9* | 44.56012643 | 5.885904388 | 0.132089041 | -2.920417314 | 7.22406E-07 | 9.2843E-06 | - |
| *BRH1* | 79.32971402 | 4.416953211 | 0.055678421 | -4.166737893 | 7.24474E-07 | 9.30624E-06 | K16281 |
| *RBCS* | 5490.753078 | 916.616146 | 0.166938147 | -2.58261443 | 7.27077E-07 | 9.33034E-06 | K01602 |
| *CRK2* | 43.35232209 | 6.497565703 | 0.149878147 | -2.738138049 | 7.52112E-07 | 9.63235E-06 | - |
| *GYRB* | 183.4831684 | 70.7399081 | 0.38553895 | -1.375051475 | 7.58901E-07 | 9.70962E-06 | K02470 |
| *ACA7* | 456.7317756 | 158.0026146 | 0.345941805 | -1.531398731 | 7.66245E-07 | 9.78896E-06 | K01674 |
| *DAD2* | 40.71041087 | 0.291413198 | 0.007158198 | -7.126187753 | 7.92926E-07 | 1.01047E-05 | - |
| *UGT86A1* | 375.0878877 | 146.6580501 | 0.390996497 | -1.354772413 | 7.95488E-07 | 1.01306E-05 | - |
| *At5g10820* | 140.3383421 | 49.46439331 | 0.352465282 | -1.504446937 | 7.96116E-07 | 1.01306E-05 | - |
| *uncharacterized protein_10454* | 19.13176667 | 0.295072441 | 0.015423167 | -6.018757109 | 7.96145E-07 | 1.01306E-05 | K10258 |
| *PHT2-1* | 376.3438007 | 138.3062968 | 0.367499867 | -1.444184366 | 8.05019E-07 | 1.02334E-05 | K14640 |
| *LRK10L-1.2* | 56.70250932 | 8.867747065 | 0.156390734 | -2.676773056 | 8.0977E-07 | 1.02836E-05 | - |
| *TAB2* | 601.2584832 | 273.3965096 | 0.454707114 | -1.13699052 | 8.11082E-07 | 1.02952E-05 | - |
| *GSTU18* | 70.3514708 | 13.38080687 | 0.190199391 | -2.394415472 | 8.11772E-07 | 1.02989E-05 | K00799 |
| *At1g80640* | 42.30175819 | 6.24458926 | 0.147620088 | -2.760039041 | 8.28045E-07 | 1.05002E-05 | - |
| *SERINC3* | 53.92553801 | 10.3495203 | 0.19192243 | -2.381404765 | 8.32947E-07 | 1.05467E-05 | - |
| *MO2* | 26.85392468 | 1.780504915 | 0.066303341 | -3.914774629 | 8.56378E-07 | 1.08289E-05 | - |
| *At3g47570* | 26.85051508 | 1.783256701 | 0.066414246 | -3.912363465 | 8.56497E-07 | 1.08289E-05 | - |
| *BRG3* | 90.88639916 | 26.06988811 | 0.286840367 | -1.801680025 | 8.70057E-07 | 1.09895E-05 | K19042 |
| *At5g60570* | 593.3218516 | 286.9010492 | 0.483550451 | -1.048261673 | 8.88951E-07 | 1.12116E-05 | - |
| *uncharacterized protein_21363* | 174.4546191 | 64.97472016 | 0.372444825 | -1.424901376 | 9.06345E-07 | 1.14254E-05 | - |
| *SWEET1* | 172.216946 | 55.67332702 | 0.323274383 | -1.629168904 | 9.29729E-07 | 1.16915E-05 | K15382 |
| *CSE* | 25.411096 | 1.49046761 | 0.058654204 | -4.091621673 | 9.42658E-07 | 1.18425E-05 | - |
| *At3g61320* | 27.87171981 | 2.073294006 | 0.074387014 | -3.748805407 | 9.55447E-07 | 1.19855E-05 | - |
| *FTIP7* | 318.535368 | 95.33296072 | 0.299285324 | -1.740406561 | 9.59914E-07 | 1.20357E-05 | - |
| *MYB1* | 116.6792505 | 38.44557128 | 0.329497928 | -1.601658702 | 9.88019E-07 | 1.2364E-05 | K09422 |
| *VEP1* | 20.82516065 | 0 | 0 | -Inf | 9.89894E-07 | 1.23814E-05 | - |
| *RNS1* | 36.36799876 | 4.436625318 | 0.121992561 | -3.035134915 | 9.99262E-07 | 1.24864E-05 | K01166 |
| *CYP76T24* | 388.6137265 | 153.4822364 | 0.394948057 | -1.340265169 | 1.01828E-06 | 1.27055E-05 | - |
| *DTX41* | 2138.673128 | 701.4994491 | 0.328006856 | -1.608202124 | 1.01948E-06 | 1.27143E-05 | K03327 |
| *ROQ1* | 55.30788959 | 9.47712503 | 0.171352136 | -2.544963917 | 1.03453E-06 | 1.2877E-05 | - |
| *CIPK26* | 66.67607252 | 15.68693678 | 0.235270858 | -2.087605465 | 1.04368E-06 | 1.29846E-05 | K07198 |
| *CCR1* | 244.726526 | 32.83691155 | 0.134177983 | -2.897780137 | 1.07885E-06 | 1.33962E-05 | K09753 |
| *At2g18940* | 487.6257918 | 136.4288458 | 0.279781849 | -1.837625724 | 1.09831E-06 | 1.36312E-05 | - |
| *XA21* | 45.94075523 | 7.697996007 | 0.167563549 | -2.577219746 | 1.10614E-06 | 1.37152E-05 | - |
| *CBP1* | 45.08896537 | 7.131182475 | 0.158158042 | -2.66056118 | 1.10851E-06 | 1.37379E-05 | K16296 |
| *ag4* | 98.69145927 | 2.380720068 | 0.024122858 | -5.37345534 | 1.12255E-06 | 1.38918E-05 | K04120 |
| *HIPP23* | 217.2902767 | 74.07577156 | 0.340906978 | -1.552549965 | 1.12542E-06 | 1.39206E-05 | - |
| *NAC075* | 82.55063348 | 22.69868131 | 0.274966773 | -1.8626708 | 1.13287E-06 | 1.4006E-05 | - |
| *MAN6* | 52.33063724 | 4.410542184 | 0.084282218 | -3.568627906 | 1.14121E-06 | 1.41023E-05 | K19355 |
| *At2g44130* | 43.24346333 | 6.834734141 | 0.158052423 | -2.661524946 | 1.14223E-06 | 1.41082E-05 | - |
| *At3g03770* | 319.4204737 | 106.928696 | 0.334758429 | -1.578807713 | 1.16304E-06 | 1.43307E-05 | - |
| *PCMP-H81* | 242.924568 | 102.4934466 | 0.421914701 | -1.244976737 | 1.16939E-06 | 1.44021E-05 | - |
| *ROQ1* | 101.7443194 | 0 | 0 | -Inf | 1.17017E-06 | 1.44048E-05 | - |
| *PUB45* | 71.47105376 | 16.34848101 | 0.228742689 | -2.128202463 | 1.17447E-06 | 1.44508E-05 | - |
| *At4g17560* | 1549.504378 | 653.1949697 | 0.421550903 | -1.246221246 | 1.17974E-06 | 1.45018E-05 | K02884 |
| *NRT3.1* | 227.3907607 | 2.370649797 | 0.010425445 | -6.583747269 | 1.20554E-06 | 1.48118E-05 | - |
| *WNK4* | 120.4187371 | 22.06550356 | 0.183239786 | -2.448195315 | 1.21639E-06 | 1.4938E-05 | K08867 |
| *Os04g0590900* | 668.2086493 | 215.2096661 | 0.322069561 | -1.634555779 | 1.21892E-06 | 1.4962E-05 | - |
| *SMAX1* | 558.2048416 | 271.4667473 | 0.486321019 | -1.040019149 | 1.26866E-06 | 1.55576E-05 | - |
| *At2g02240* | 257.3910725 | 82.97520272 | 0.322370166 | -1.633209861 | 1.27065E-06 | 1.55746E-05 | - |
| *HTR2* | 16.08366458 | 0 | 0 | -Inf | 1.27303E-06 | 1.55962E-05 | K11253 |
| *PCMP-H81* | 90.01885255 | 26.39607882 | 0.293228341 | -1.769903544 | 1.28997E-06 | 1.57888E-05 | - |
| *NIP3-1* | 35.1549182 | 3.239854256 | 0.092159346 | -3.439725717 | 1.33132E-06 | 1.62716E-05 | K09874 |
| *At2g23540* | 81.55888872 | 8.554846845 | 0.104891655 | -3.253028186 | 1.33594E-06 | 1.63126E-05 | - |
| *PAE9* | 187.5713249 | 65.31282547 | 0.348202613 | -1.522001066 | 1.33724E-06 | 1.63207E-05 | K19882 |
| *RR4* | 38.52619406 | 5.314524155 | 0.137945735 | -2.857827243 | 1.37251E-06 | 1.67352E-05 | K14492 |
| *PCMP-H24* | 140.0013501 | 50.53900406 | 0.360989405 | -1.469971601 | 1.3976E-06 | 1.70088E-05 | - |
| *Spast* | 1157.231449 | 573.9441075 | 0.495963109 | -1.011695281 | 1.44183E-06 | 1.7524E-05 | - |
| *HPCA1* | 199.4698538 | 37.97798717 | 0.190394621 | -2.392935376 | 1.44197E-06 | 1.7524E-05 | - |
| *uncharacterized protein_18663* | 80.41318346 | 22.11679178 | 0.275039376 | -1.862289919 | 1.47645E-06 | 1.79261E-05 | - |
| *At5g48900* | 22.92290773 | 0 | 0 | -Inf | 1.63051E-06 | 1.9722E-05 | K01728 |
| *BGLU12* | 15.85904885 | 0 | 0 | -Inf | 1.64822E-06 | 1.99174E-05 | K01188 |
| *At1g56130* | 169.4073498 | 65.78766008 | 0.388340058 | -1.364607562 | 1.67689E-06 | 2.02445E-05 | - |
| *FLN1* | 260.7412216 | 113.3383425 | 0.4346775 | -1.201982674 | 1.69626E-06 | 2.04307E-05 | - |
| *RPA1B* | 15.75191287 | 0 | 0 | -Inf | 1.7114E-06 | 2.06033E-05 | K07466 |
| *ag8* | 15.73981745 | 0 | 0 | -Inf | 1.7187E-06 | 2.06784E-05 | K12742 |
| *PNC1* | 46.18269955 | 0.900322728 | 0.019494805 | -5.680766456 | 1.75295E-06 | 2.10445E-05 | K00430 |
| *PCNA* | 48.03675066 | 8.913531718 | 0.185556508 | -2.430069492 | 1.75398E-06 | 2.10469E-05 | K04802 |
| *WDL5* | 549.3591298 | 270.2302609 | 0.491900919 | -1.023560344 | 1.77671E-06 | 2.13097E-05 | - |
| *uncharacterized protein_04764* | 575.2964322 | 220.441872 | 0.383179626 | -1.383907239 | 1.7808E-06 | 2.13488E-05 | - |
| *uncharacterized protein_13234* | 77.82118978 | 0.882933972 | 0.011345676 | -6.461713673 | 1.8024E-06 | 2.15876E-05 | - |
| *At5g15710* | 112.3180317 | 37.48301201 | 0.333722123 | -1.583280766 | 1.80965E-06 | 2.16644E-05 | - |
| *CYP720B2* | 823.3042282 | 132.37652 | 0.160786882 | -2.636778385 | 1.91416E-06 | 2.28516E-05 | - |
| *Bp10* | 73.74499894 | 15.87310998 | 0.215243206 | -2.215960396 | 1.93783E-06 | 2.30916E-05 | - |
| *CML21* | 46.30882916 | 8.274411376 | 0.178678916 | -2.484558691 | 1.94461E-06 | 2.31613E-05 | K13448 |
| *uncharacterized protein_32133* | 53.90665936 | 11.228356 | 0.208292559 | -2.263316796 | 1.94662E-06 | 2.31745E-05 | - |
| *At5g10770* | 30.08863346 | 2.957603871 | 0.098296384 | -3.346717843 | 1.9612E-06 | 2.33265E-05 | - |
| *OLE9* | 29.01904706 | 2.682203536 | 0.09242907 | -3.43550952 | 1.97095E-06 | 2.34316E-05 | - |
| *CRRSP41* | 27.85958843 | 2.363331312 | 0.084830087 | -3.559280147 | 1.98465E-06 | 2.35726E-05 | - |
| *CYP720B2* | 24.22920528 | 1.500537881 | 0.061930957 | -4.01319544 | 1.99547E-06 | 2.36793E-05 | - |
| *UGT75L6* | 17.21889641 | 0 | 0 | -Inf | 2.03746E-06 | 2.4144E-05 | K13692 |
| *MCM3* | 29.6220734 | 2.0581886 | 0.069481585 | -3.847225532 | 2.0412E-06 | 2.41661E-05 | K02541 |
| *KCS11* | 316.5636143 | 41.05687331 | 0.129695491 | -2.946799774 | 2.06319E-06 | 2.44039E-05 | K15397 |
| *At1g01500* | 72.42489029 | 8.554378411 | 0.118113792 | -3.081750661 | 2.08126E-06 | 2.45951E-05 | - |
| *PCMP-H40* | 300.7948763 | 136.2571736 | 0.452990341 | -1.142447807 | 2.14124E-06 | 2.52458E-05 | - |
| *FPP4* | 34.86465704 | 4.448071481 | 0.127581105 | -2.970513412 | 2.18202E-06 | 2.56912E-05 | - |
| *At3g18200* | 55.25099482 | 11.82810272 | 0.214079452 | -2.223781763 | 2.21775E-06 | 2.60557E-05 | - |
| *PCMP-H40* | 119.6288234 | 36.3923884 | 0.30421087 | -1.716856393 | 2.21805E-06 | 2.60557E-05 | - |
| *SBT5.6* | 101.8433308 | 9.812917576 | 0.09635307 | -3.375525556 | 2.23997E-06 | 2.63012E-05 | - |
| *R3* | 15.41495685 | 0 | 0 | -Inf | 2.30594E-06 | 2.69895E-05 | K00430 |
| *UTR4* | 63.39819346 | 15.40146617 | 0.242932256 | -2.041374034 | 2.3322E-06 | 2.72844E-05 | K15277 |
| *TSC10B* | 23.44119422 | 0 | 0 | -Inf | 2.33466E-06 | 2.73008E-05 | K04708 |
| *At2g39510* | 26.5377499 | 2.050870115 | 0.077281236 | -3.69373802 | 2.35115E-06 | 2.7481E-05 | - |
| *RPT3* | 99.49330271 | 26.92949073 | 0.270666366 | -1.885412468 | 2.35318E-06 | 2.74923E-05 | - |
| *dnaJ* | 85.01470284 | 25.15402095 | 0.295878479 | -1.756923332 | 2.43494E-06 | 2.83829E-05 | - |
| *ag4* | 17.65613326 | 0.300107576 | 0.016997356 | -5.878545874 | 2.52928E-06 | 2.94386E-05 | - |
| *NORK* | 19.52751296 | 0.586485639 | 0.030033811 | -5.057268626 | 2.53008E-06 | 2.94386E-05 | - |
| *PIP2-3* | 101.416229 | 15.92937452 | 0.157069284 | -2.670527012 | 2.54911E-06 | 2.95994E-05 | K09872 |
| *uncharacterized protein_32390* | 665.1492724 | 304.4982048 | 0.457789277 | -1.127244423 | 2.54967E-06 | 2.95994E-05 | - |
| *NOL* | 203.6656428 | 85.17790755 | 0.418224235 | -1.257651432 | 2.5854E-06 | 3.00006E-05 | K13606 |
| *Gvin1* | 297.5256471 | 135.410325 | 0.455121521 | -1.135676288 | 2.59998E-06 | 3.01563E-05 | - |
| *At1g06840* | 134.4305682 | 49.37563462 | 0.367294695 | -1.444990034 | 2.64647E-06 | 3.06401E-05 | - |
| *BALDH* | 24.61978324 | 0.300107576 | 0.012189692 | -6.35819451 | 2.66496E-06 | 3.08265E-05 | K12355 |
| *At3g02645* | 279.4739403 | 125.7204752 | 0.449846863 | -1.152494134 | 2.6887E-06 | 3.10871E-05 | - |
| *PIP2-8* | 48.43429166 | 6.520897052 | 0.134633889 | -2.892886494 | 2.76421E-06 | 3.1874E-05 | K09872 |
| *FT1* | 195.5872616 | 80.40961736 | 0.411118887 | -1.282372442 | 2.77981E-06 | 3.20252E-05 | K13681 |
| *uncharacterized protein_08602* | 512.9022734 | 139.2905102 | 0.271573197 | -1.880586995 | 2.79595E-06 | 3.21967E-05 | - |
| *ABCC8* | 9417.657073 | 3066.273678 | 0.32558774 | -1.618881721 | 2.82973E-06 | 3.25564E-05 | - |
| *HSP21* | 20.91844247 | 0.895287593 | 0.04279896 | -4.546280433 | 2.83494E-06 | 3.26018E-05 | K13993 |
| *BIC1* | 255.3676566 | 81.92354312 | 0.320806261 | -1.640225796 | 2.96934E-06 | 3.41017E-05 | - |
| *AGT1* | 22777.82052 | 7565.949173 | 0.332162999 | -1.590036721 | 2.99836E-06 | 3.44041E-05 | K00830 |
| *COL16* | 208.0583969 | 23.69098295 | 0.113866988 | -3.134578551 | 3.04641E-06 | 3.4862E-05 | - |
| *WRI1* | 27.20297611 | 2.353261041 | 0.086507485 | -3.531031228 | 3.06772E-06 | 3.50747E-05 | K09285 |
| *OVA2* | 540.1204957 | 268.7094557 | 0.497499091 | -1.007234205 | 3.13607E-06 | 3.57749E-05 | K01870 |
| *D6PKL1* | 266.2999152 | 119.6667527 | 0.449368347 | -1.154029589 | 3.13732E-06 | 3.57749E-05 | K08286 |
| *uncharacterized protein_48550* | 178.5101725 | 72.58211314 | 0.406599311 | -1.298320325 | 3.21637E-06 | 3.66438E-05 | - |
| *uncharacterized protein_49231* | 105.7620077 | 27.91904059 | 0.263979866 | -1.921500198 | 3.38239E-06 | 3.845E-05 | - |
| *TULP10* | 321.3747839 | 149.7559745 | 0.465985454 | -1.101643174 | 3.41064E-06 | 3.87368E-05 | - |
| *GID2* | 179.296281 | 73.31139749 | 0.408884094 | -1.290236152 | 3.42325E-06 | 3.88628E-05 | K14495 |
| *JOX2* | 17.23275057 | 0.291413198 | 0.016910429 | -5.885942964 | 3.44603E-06 | 3.91042E-05 | K05278 |
| *BLH2* | 336.8656798 | 158.3896072 | 0.470186239 | -1.088695777 | 3.45245E-06 | 3.91425E-05 | - |
| *PHL3* | 529.4330511 | 264.5459565 | 0.499677827 | -1.000929894 | 3.52895E-06 | 3.99569E-05 | - |
| *PBL34* | 88.87148934 | 27.38737561 | 0.308168298 | -1.698209639 | 3.54195E-06 | 4.00688E-05 | - |
| *LRK10L-2.4* | 60.3380608 | 14.54921145 | 0.241128257 | -2.052127368 | 3.55222E-06 | 4.01464E-05 | - |
| *DDB_G0293730* | 98.15229783 | 31.93152428 | 0.325326304 | -1.62004062 | 3.5535E-06 | 4.01464E-05 | - |
| *BLH2* | 179.1132997 | 73.19652624 | 0.408660476 | -1.291025378 | 3.6306E-06 | 4.09814E-05 | - |
| *TCP14* | 59.48777794 | 14.21751717 | 0.238998962 | -2.064923744 | 3.63394E-06 | 4.10011E-05 | - |
| *sA* | 113.6036129 | 39.69635293 | 0.349428614 | -1.516930344 | 3.68566E-06 | 4.15482E-05 | - |
| *IMK3* | 20.56074118 | 0.882933972 | 0.042942711 | -4.541442908 | 3.68807E-06 | 4.15571E-05 | - |
| *PIP1-5* | 167.5191019 | 28.09283076 | 0.167699268 | -2.576051704 | 3.73606E-06 | 4.20426E-05 | K09872 |
| *At4g37250* | 25.72210244 | 2.069634763 | 0.080461337 | -3.635560472 | 3.76864E-06 | 4.23906E-05 | - |
| *ORRM1* | 172.8234609 | 70.43889307 | 0.407577147 | -1.294854935 | 3.79527E-06 | 4.26687E-05 | - |
| *EO* | 129.8757867 | 17.03419607 | 0.131157597 | -2.930626722 | 3.809E-06 | 4.2751E-05 | K18980 |
| *MDIS1* | 62.18690051 | 15.35617937 | 0.246935918 | -2.017791396 | 3.86071E-06 | 4.32369E-05 | - |
| *PCMP-H42* | 134.8762032 | 50.54313174 | 0.374737208 | -1.416048865 | 3.91361E-06 | 4.38103E-05 | - |
| *FLZ2* | 579.8971239 | 99.47732421 | 0.171543055 | -2.543357376 | 3.99713E-06 | 4.47062E-05 | - |
| *VQ4* | 87.96255297 | 27.22043549 | 0.309454814 | -1.692199328 | 4.00846E-06 | 4.48134E-05 | - |
| *ABCG39* | 188.2006605 | 63.14383448 | 0.335513352 | -1.575557914 | 4.01913E-06 | 4.49132E-05 | - |
| *DJC76* | 397.4370781 | 193.0025543 | 0.485617888 | -1.042106531 | 4.04289E-06 | 4.51395E-05 | - |
| *BGLU12* | 14.75489898 | 0 | 0 | -Inf | 4.17153E-06 | 4.64344E-05 | K01188 |
| *At1g21780* | 164.3289407 | 66.14450063 | 0.402512791 | -1.312893464 | 4.18946E-06 | 4.66138E-05 | K10523 |
| *BDG1* | 56.13217981 | 2.347318448 | 0.041817696 | -4.579742603 | 4.25679E-06 | 4.73015E-05 | - |
| *CYP720B2* | 34.46553711 | 4.739016244 | 0.137500142 | -2.862494987 | 4.28821E-06 | 4.76095E-05 | - |
| *uncharacterized protein_51382* | 14.65982245 | 0 | 0 | -Inf | 4.31381E-06 | 4.78524E-05 | - |
| *PCMP-H32* | 100.5541602 | 33.42568054 | 0.332414695 | -1.588943933 | 4.34505E-06 | 4.81365E-05 | - |
| *CEL1* | 14.63566757 | 0 | 0 | -Inf | 4.35086E-06 | 4.81802E-05 | K01179 |
| *PPD2* | 434.8422111 | 180.2590235 | 0.414538927 | -1.270420512 | 4.39852E-06 | 4.86659E-05 | - |
| *PCMP-H32* | 364.6581016 | 151.4024725 | 0.415190206 | -1.268155681 | 4.42929E-06 | 4.89642E-05 | - |
| *PM19L* | 163.397788 | 59.95076972 | 0.366900742 | -1.446538272 | 4.44316E-06 | 4.90964E-05 | - |
| *GAT1* | 28.72706312 | 2.958511328 | 0.102986905 | -3.279467183 | 4.52149E-06 | 4.99405E-05 | - |
| *MT2788* | 41.21142651 | 7.084051342 | 0.17189532 | -2.540397829 | 4.61092E-06 | 5.08409E-05 | - |
| *LOX1.1* | 65.10199605 | 3.277383553 | 0.05034229 | -4.312085351 | 4.61864E-06 | 5.09041E-05 | K15718 |
| *TBL7* | 90.48014347 | 23.75597128 | 0.262554527 | -1.929311023 | 4.64911E-06 | 5.12016E-05 | - |
| *CNMT* | 111.3052611 | 39.04853821 | 0.350823832 | -1.511181339 | 4.65958E-06 | 5.12893E-05 | K13384 |
| *uncharacterized protein_46263* | 114.2152171 | 40.41040507 | 0.353809292 | -1.498956159 | 4.66565E-06 | 5.13142E-05 | - |
| *uncharacterized protein_00665* | 240.2878393 | 80.2646327 | 0.334035351 | -1.581927304 | 4.66584E-06 | 5.13142E-05 | - |
| *AMY1.3* | 153.4880325 | 60.51567095 | 0.394269638 | -1.342745481 | 4.72286E-06 | 5.18969E-05 | K01176 |
| *DEGP5* | 101.6946394 | 34.35021266 | 0.337778007 | -1.565852697 | 4.73155E-06 | 5.19523E-05 | - |
| *VLN2* | 236.0590947 | 105.2841103 | 0.446007431 | -1.164860349 | 4.95695E-06 | 5.43066E-05 | - |
| *EXL3* | 734.2742436 | 268.3170773 | 0.36541807 | -1.452380116 | 5.04487E-06 | 5.52251E-05 | - |
| *At4g30825* | 275.3494185 | 120.347598 | 0.437072279 | -1.194056214 | 5.04508E-06 | 5.52251E-05 | - |
| *OFUT19* | 126.0914578 | 46.73783984 | 0.370666187 | -1.43180758 | 5.12067E-06 | 5.60048E-05 | - |
| *HSP22* | 119.1088572 | 43.22255584 | 0.362882802 | -1.46242441 | 5.16359E-06 | 5.64261E-05 | K13993 |
| *COMT1* | 152.5701439 | 19.0270253 | 0.124710017 | -3.003350739 | 5.25611E-06 | 5.73883E-05 | - |
| *uncharacterized protein_24925* | 22.80195355 | 1.475362204 | 0.064703325 | -3.950016345 | 5.27303E-06 | 5.75486E-05 | - |
| *LRK10L-1.1* | 247.5175166 | 111.8964884 | 0.452075029 | -1.145365865 | 5.30523E-06 | 5.78263E-05 | - |
| *uncharacterized protein_37062* | 22.74492202 | 1.500537881 | 0.065972435 | -3.921992844 | 5.30998E-06 | 5.78535E-05 | K06678 |
| *VIT_19s0014g02480* | 628.2543732 | 311.903781 | 0.496460979 | -1.010247767 | 5.34217E-06 | 5.81796E-05 | K16054 |
| *PCMP-H81* | 243.1576609 | 109.477361 | 0.450232004 | -1.151259482 | 5.3555E-06 | 5.82391E-05 | - |
| *BSPA* | 220.6839416 | 97.26867679 | 0.440760103 | -1.181934456 | 5.45683E-06 | 5.92524E-05 | - |
| *AGPS1* | 2966.125001 | 1120.974445 | 0.377925557 | -1.40382601 | 5.47739E-06 | 5.94256E-05 | K00975 |
| *CRR2* | 292.9674059 | 136.8020022 | 0.466952977 | -1.098650821 | 5.65891E-06 | 6.12913E-05 | - |
| *CPIJ013394* | 144.0258935 | 41.35557558 | 0.287139865 | -1.800174456 | 5.70018E-06 | 6.16604E-05 | - |
| *IP5P4* | 62.9384455 | 4.44028456 | 0.070549638 | -3.825217505 | 5.70704E-06 | 6.17086E-05 | K20279 |
| *WAK3* | 143.7441455 | 56.2259426 | 0.391152923 | -1.354195351 | 5.76166E-06 | 6.22419E-05 | - |
| *BGLU42* | 77.12147911 | 22.73621061 | 0.294810355 | -1.762140898 | 5.81711E-06 | 6.27139E-05 | K01188 |
| *PAP27* | 35.72179505 | 5.36303118 | 0.150133306 | -2.735684033 | 5.8512E-06 | 6.3055E-05 | - |
| *LYM1* | 197.1408445 | 81.18631565 | 0.411818849 | -1.279918232 | 5.87688E-06 | 6.33052E-05 | - |
| *ATL8* | 27.13729472 | 2.673509158 | 0.098517895 | -3.343470384 | 5.92072E-06 | 6.37241E-05 | K19040 |
| *SCRM* | 363.4415608 | 176.4764291 | 0.485570304 | -1.042247902 | 5.94758E-06 | 6.3937E-05 | - |
| *ROQ1* | 21.30378016 | 1.169312035 | 0.054887538 | -4.187377572 | 5.94798E-06 | 6.3937E-05 | - |
| *PCR3* | 21.26408431 | 1.169312035 | 0.054990002 | -4.184686856 | 6.00382E-06 | 6.44834E-05 | - |
| *KLCR2* | 16.53640645 | 0.295072441 | 0.017843807 | -5.808432762 | 6.06741E-06 | 6.51391E-05 | - |
| *UGT85A19* | 687.2413731 | 280.4533756 | 0.408085698 | -1.293055944 | 6.25452E-06 | 6.698E-05 | - |
| *NAT12* | 401.8588819 | 197.9793351 | 0.492658851 | -1.021339118 | 6.38606E-06 | 6.82748E-05 | K14611 |
| *uncharacterized protein_10992* | 124.8333332 | 46.80967822 | 0.374977396 | -1.415124464 | 6.43232E-06 | 6.86551E-05 | - |
| *AGD11* | 48.72455282 | 8.30049451 | 0.170355478 | -2.553379751 | 6.5929E-06 | 7.01941E-05 | K12486 |
| *PIP2-8* | 22.49611545 | 1.500537881 | 0.066702088 | -3.906124259 | 6.59862E-06 | 7.02258E-05 | K09872 |
| *bshA* | 97.31084465 | 32.73211058 | 0.336366524 | -1.571893962 | 6.72786E-06 | 7.1542E-05 | - |
| *At5g10770* | 40.61181685 | 6.192422992 | 0.152478354 | -2.713323643 | 6.73873E-06 | 7.16279E-05 | - |
| *PCMP-H42* | 320.7943912 | 128.6154421 | 0.400927964 | -1.318585049 | 6.76642E-06 | 7.18925E-05 | - |
| *At4g10440* | 30.85245369 | 3.857926599 | 0.125044401 | -2.999487631 | 6.90769E-06 | 7.33329E-05 | - |
| *PLR_Lu2* | 23.89565886 | 1.183041548 | 0.049508639 | -4.3361759 | 7.06462E-06 | 7.48442E-05 | - |
| *ROC1* | 5649.097642 | 2587.279892 | 0.457998791 | -1.126584304 | 7.37372E-06 | 7.79383E-05 | K11294 |
| *Os05g0277500* | 38.08906511 | 4.422895804 | 0.116119831 | -3.106313717 | 7.37487E-06 | 7.79383E-05 | - |
| *APS1* | 65.77228271 | 3.560102373 | 0.0541277 | -4.207489109 | 7.41141E-06 | 7.82922E-05 | - |
| *BRH1* | 13.97905526 | 0 | 0 | -Inf | 7.87477E-06 | 8.29823E-05 | K16281 |
| *At5g47260* | 51.68619227 | 11.7992678 | 0.228286652 | -2.131081586 | 8.06981E-06 | 8.47594E-05 | K13459 |
| *SMXL3* | 34.07154956 | 5.028146092 | 0.147576091 | -2.760469089 | 8.13287E-06 | 8.53519E-05 | - |
| *At1g56130* | 79.56649001 | 24.54739477 | 0.308514235 | -1.696591039 | 8.14886E-06 | 8.54849E-05 | - |
| *At3g47570* | 16.14586445 | 0.291413198 | 0.018048783 | -5.791954657 | 8.17232E-06 | 8.56959E-05 | - |
| *PIP2-8* | 2337.603094 | 825.3923928 | 0.353093472 | -1.501877945 | 8.29634E-06 | 8.69255E-05 | K09872 |
| *ABCG3* | 89.77341961 | 27.5722409 | 0.307131454 | -1.703071825 | 8.49981E-06 | 8.86956E-05 | - |
| *SUN2* | 34.93554273 | 5.308581562 | 0.151953602 | -2.718297219 | 8.52849E-06 | 8.89226E-05 | K19347 |
| *PCMP-H32* | 214.1179764 | 95.22356364 | 0.44472475 | -1.169015397 | 8.53576E-06 | 8.89624E-05 | - |
| *NOL* | 274.7790601 | 128.9421012 | 0.469257378 | -1.091548667 | 8.65971E-06 | 9.0181E-05 | K13606 |
| *MIF2* | 21.95694692 | 1.483149124 | 0.067548058 | -3.887941897 | 8.76571E-06 | 9.1211E-05 | - |
| *AMC9* | 37.93346843 | 5.608689138 | 0.147855953 | -2.757735761 | 9.1772E-06 | 9.52227E-05 | - |
| *AMC9* | 29.37671239 | 3.536302589 | 0.120377752 | -3.054359318 | 9.25024E-06 | 9.59031E-05 | - |
| *ag1* | 53.93580275 | 0.291413198 | 0.005402964 | -7.532033228 | 9.375E-06 | 9.70399E-05 | K16086 |
| *SD31* | 26.9524108 | 0.300107576 | 0.011134721 | -6.488790773 | 9.3997E-06 | 9.72564E-05 | - |
| *PXC1* | 284.5487311 | 134.7671064 | 0.473616965 | -1.078207335 | 9.43008E-06 | 9.75314E-05 | - |
| *PME51* | 27.20638571 | 1.178006413 | 0.043298894 | -4.529526013 | 9.47504E-06 | 9.7957E-05 | K01051 |
| *NHL26* | 41.87492994 | 7.949596558 | 0.189841429 | -2.397133226 | 9.57952E-06 | 9.89178E-05 | - |
| *At1g80170* | 42.48836489 | 8.317883266 | 0.195768495 | -2.352779481 | 9.72163E-06 | 0.000100345 | K01213 |
| *TPS-LAS2* | 114.0410873 | 0 | 0 | -Inf | 9.76103E-06 | 0.000100711 | K04120 |
| *GLR2.7* | 46.9637187 | 10.0535404 | 0.214070365 | -2.223843007 | 9.78579E-06 | 0.000100886 | K05387 |
| *TIP1-1* | 103.8319815 | 36.90662605 | 0.355445649 | -1.49229912 | 9.871E-06 | 0.000101601 | K09873 |
| *NAGK* | 380.2303202 | 189.1513418 | 0.497465173 | -1.007332568 | 9.9762E-06 | 0.000102643 | K00930 |
| *ADT3* | 217.4561528 | 77.27184171 | 0.355344472 | -1.492709841 | 1.01346E-05 | 0.000104231 | K05359 |
| *LNG2* | 111.6699543 | 29.89256876 | 0.267686765 | -1.901382286 | 1.01499E-05 | 0.000104346 | - |
| *YSL3* | 217.7361993 | 98.51483292 | 0.452450411 | -1.144168414 | 1.01588E-05 | 0.000104387 | - |
| *CRK2* | 17.56105673 | 0.586485639 | 0.033396945 | -4.904140069 | 1.03325E-05 | 0.000106053 | - |
| *SWEET16* | 73.77780367 | 12.95303541 | 0.175568189 | -2.509896623 | 1.03479E-05 | 0.000106166 | K15382 |
| *DIS1* | 114.6282631 | 42.51985248 | 0.370936899 | -1.430754307 | 1.04295E-05 | 0.000106878 | K04506 |
| *TPS-mISO1* | 19.11105735 | 0.885217322 | 0.046319641 | -4.432232107 | 1.04556E-05 | 0.000107103 | K16086 |
| *PCBER* | 72.53008771 | 0 | 0 | -Inf | 1.06273E-05 | 0.000108819 | - |
| *PUB26* | 50.32289915 | 11.54034877 | 0.229325992 | -2.124528215 | 1.07352E-05 | 0.000109793 | - |
| *LRK10L-1.2* | 64.17245117 | 5.357996045 | 0.08349371 | -3.582188669 | 1.07652E-05 | 0.000110055 | - |
| *RITF1* | 13.60581299 | 0 | 0 | -Inf | 1.07713E-05 | 0.000110074 | - |
| *At5g03795* | 13.60409021 | 0 | 0 | -Inf | 1.07779E-05 | 0.000110097 | - |
| *HCF101* | 302.580074 | 145.8771855 | 0.482111011 | -1.052562713 | 1.08459E-05 | 0.000110616 | - |
| *SDR2a* | 174.1665411 | 25.41015879 | 0.145895754 | -2.776990201 | 1.08513E-05 | 0.000110628 | - |
| *uncharacterized protein_01834* | 41.72292977 | 3.253583769 | 0.077980712 | -3.680738863 | 1.08816E-05 | 0.000110892 | - |
| *UGT86A1* | 187.4646565 | 48.87430726 | 0.260712116 | -1.939470465 | 1.10141E-05 | 0.000112154 | - |
| *ET2* | 40.6376945 | 7.650864874 | 0.188270151 | -2.409123808 | 1.12453E-05 | 0.000114281 | - |
| *nep2* | 30.78677229 | 4.119128985 | 0.133795415 | -2.901899414 | 1.14457E-05 | 0.000116226 | - |
| *CDA1* | 209.5977225 | 94.35032889 | 0.45014959 | -1.151523588 | 1.15588E-05 | 0.000117189 | K01489 |
| *GLYI4* | 36.77060021 | 6.20111737 | 0.168643355 | -2.567952625 | 1.16417E-05 | 0.000117983 | - |
| *ATJ8* | 85.73878417 | 28.207702 | 0.328995825 | -1.60385882 | 1.18023E-05 | 0.000119563 | - |
| *CRK2* | 80.62563185 | 13.94442959 | 0.17295281 | -2.53154964 | 1.18674E-05 | 0.000120175 | - |
| *BHLH80* | 128.6073544 | 50.19817638 | 0.390321196 | -1.357266286 | 1.20016E-05 | 0.000121439 | - |
| *WRKY71* | 22.6653505 | 0 | 0 | -Inf | 1.22607E-05 | 0.000123865 | - |
| *At1g74320* | 78.34489635 | 24.42839585 | 0.311805835 | -1.681280167 | 1.22899E-05 | 0.000124063 | K14156 |
| *CHI4* | 88.32545856 | 28.18793251 | 0.31913712 | -1.647751669 | 1.23156E-05 | 0.000124244 | K01183 |
| *NAKR2* | 226.2568778 | 103.6215406 | 0.457981837 | -1.12663771 | 1.23175E-05 | 0.000124244 | - |
| *LOX1.4* | 105.8537106 | 38.5737036 | 0.364405777 | -1.456382266 | 1.24733E-05 | 0.000125667 | K15718 |
| *LHT1* | 562.1158473 | 218.6564294 | 0.388988196 | -1.362201718 | 1.25913E-05 | 0.000126807 | - |
| *CYP716B2* | 552.8100704 | 146.8258976 | 0.265599173 | -1.912677439 | 1.2673E-05 | 0.000127479 | - |
| *CYP720B1* | 20.81302927 | 0.295072441 | 0.014177294 | -6.140273968 | 1.29934E-05 | 0.000130498 | - |
| *TAR4* | 42.72338213 | 8.637662948 | 0.202176478 | -2.306312933 | 1.31136E-05 | 0.000131654 | - |
| *FZL* | 412.8570164 | 169.5368738 | 0.410643073 | -1.284043134 | 1.31864E-05 | 0.000132229 | - |
| *TBL11* | 17.25345989 | 0.595180017 | 0.03449627 | -4.8574158 | 1.33312E-05 | 0.000133525 | - |
| *At5g46170* | 150.1167207 | 62.04000861 | 0.41327847 | -1.274813888 | 1.37609E-05 | 0.000137454 | - |
| *WAV3* | 106.8144311 | 37.85770975 | 0.354425047 | -1.496447534 | 1.38971E-05 | 0.000138706 | - |
| *uncharacterized protein_23370* | 57.56292016 | 15.13707298 | 0.262965689 | -1.92705352 | 1.42228E-05 | 0.000141792 | - |
| *LRL1* | 286.8420153 | 138.1328391 | 0.481564177 | -1.054200019 | 1.42325E-05 | 0.000141834 | - |
| *GSTU17* | 127.1247291 | 25.66829717 | 0.201914272 | -2.308185203 | 1.44844E-05 | 0.000144232 | K00799 |
| *CB21* | 1685.426761 | 559.6526722 | 0.332053985 | -1.590510283 | 1.45353E-05 | 0.000144683 | K08913 |
| *WSD11* | 15.41495685 | 0.295072441 | 0.019141957 | -5.707117859 | 1.45509E-05 | 0.000144754 | - |
| *ag1* | 13.26020711 | 0 | 0 | -Inf | 1.45962E-05 | 0.000145064 | K16086 |
| *FEI1* | 31.27224696 | 4.441660453 | 0.142032022 | -2.815711869 | 1.47359E-05 | 0.000146396 | - |
| *uncharacterized protein_31688* | 13.21530696 | 0 | 0 | -Inf | 1.48308E-05 | 0.000147282 | - |
| *FKBP16-4* | 190.8065661 | 60.93521647 | 0.319355972 | -1.646762663 | 1.50525E-05 | 0.000149253 | - |
| *nep2* | 20.88395093 | 0.295072441 | 0.014129148 | -6.145181681 | 1.5137E-05 | 0.000149975 | - |
| *DIR1* | 43.8413502 | 9.140864049 | 0.208498689 | -2.261889781 | 1.53733E-05 | 0.00015214 | - |
| *BHLH94* | 30.98543136 | 2.358296176 | 0.076109838 | -3.715773231 | 1.55917E-05 | 0.000154064 | - |
| *NAC022* | 60.72336253 | 16.56436718 | 0.27278409 | -1.874168593 | 1.56648E-05 | 0.000154726 | - |
| *ACA7* | 71.32252802 | 21.66300517 | 0.303732997 | -1.719124448 | 1.57416E-05 | 0.000155366 | K01674 |
| *PCMP-H40* | 60.56264057 | 16.56711896 | 0.273553445 | -1.870105371 | 1.57669E-05 | 0.000155496 | - |
| *PCBER* | 496.7983631 | 161.2376486 | 0.324553502 | -1.623471771 | 1.62754E-05 | 0.000160142 | - |
| *LTPG5* | 62.86759577 | 7.959198394 | 0.126602557 | -2.981621549 | 1.63649E-05 | 0.000160962 | - |
| *At2g19130* | 173.9293696 | 75.83481887 | 0.436009278 | -1.197569259 | 1.6543E-05 | 0.000162589 | - |
| *XTH32* | 30.02122929 | 4.139269527 | 0.137878082 | -2.858534955 | 1.688E-05 | 0.000165583 | K08235 |
| *At3g47110* | 47.1504262 | 10.68396636 | 0.226593209 | -2.141823473 | 1.69719E-05 | 0.000166295 | K13428 |
| *Prcp* | 69.85206296 | 20.90854515 | 0.299326094 | -1.740210044 | 1.69881E-05 | 0.00016639 | K01285 |
| *uncharacterized protein_42035* | 25.32466934 | 2.690897914 | 0.106255994 | -3.234383867 | 1.73245E-05 | 0.000169492 | - |
| *uncharacterized protein_03647* | 204.5101601 | 31.63554438 | 0.154689353 | -2.692554192 | 1.7454E-05 | 0.000170574 | - |
| *CBP1* | 854.9557357 | 216.0059979 | 0.252651674 | -1.984778355 | 1.74551E-05 | 0.000170574 | K16296 |
| *At4g11680* | 381.9118852 | 151.2451636 | 0.396021097 | -1.336350808 | 1.75207E-05 | 0.000171038 | - |
| *GSTU6* | 491.4599015 | 214.2908029 | 0.436029068 | -1.197503778 | 1.75996E-05 | 0.000171594 | K00799 |
| *Mtarc1* | 342.1740886 | 171.0625487 | 0.499928412 | -1.000206574 | 1.79545E-05 | 0.000174723 | - |
| *PAP27* | 39.67524409 | 7.618370713 | 0.192018244 | -2.380684702 | 1.8077E-05 | 0.000175849 | - |
| *At4g26790* | 24.18078765 | 2.352353583 | 0.097281926 | -3.361684405 | 1.84273E-05 | 0.000178995 | - |
| *UGT85A1* | 386.4922632 | 145.3239159 | 0.376007309 | -1.411167388 | 1.84503E-05 | 0.000179141 | - |
| *7OMT* | 26.93694175 | 2.080612492 | 0.077240115 | -3.694505869 | 1.8738E-05 | 0.000181796 | - |
| *RBOHF* | 23.06457832 | 2.078329141 | 0.090109132 | -3.472182862 | 1.88841E-05 | 0.000183007 | K13447 |
| *CBG* | 184.2227329 | 82.19284465 | 0.446160164 | -1.16436639 | 1.89061E-05 | 0.000183151 | K05350 |
| *BAG1* | 1469.317792 | 658.1212727 | 0.447909415 | -1.158721103 | 1.90464E-05 | 0.000184441 | - |
| *murA* | 99.81288708 | 36.30178538 | 0.36369838 | -1.459185594 | 1.92524E-05 | 0.000186225 | - |
| *APSR1* | 185.4253978 | 82.80635029 | 0.446575018 | -1.163025549 | 1.9833E-05 | 0.000191483 | - |
| *UGT84A13* | 100.3468944 | 12.4603436 | 0.124172688 | -3.00958021 | 1.99911E-05 | 0.00019279 | K13691 |
| *MYB93* | 32.31423293 | 5.038216362 | 0.155913228 | -2.681184762 | 2.01704E-05 | 0.000194373 | K09422 |
| *MLP423* | 19.95947358 | 0.591520774 | 0.029636091 | -5.076501024 | 2.03024E-05 | 0.000195572 | - |
| *CYP720B2* | 45.22373775 | 10.03430731 | 0.221881424 | -2.172139208 | 2.03405E-05 | 0.000195864 | - |
| *uncharacterized protein_30808* | 204.6419545 | 94.12393343 | 0.459944461 | -1.120468431 | 2.03484E-05 | 0.000195864 | - |
| *At3g02060* | 144.4820521 | 60.33227894 | 0.417576288 | -1.259888305 | 2.03557E-05 | 0.000195864 | - |
| *R57* | 19.56031769 | 1.172971278 | 0.059966883 | -4.05969021 | 2.0408E-05 | 0.000196295 | K00430 |
| *PRP1* | 12.82652371 | 0 | 0 | -Inf | 2.04178E-05 | 0.000196315 | K00799 |
| *PAP27* | 67.07189074 | 17.19754493 | 0.256404654 | -1.963505647 | 2.07856E-05 | 0.000199777 | - |
| *TPK1* | 33.09876247 | 5.310864912 | 0.16045509 | -2.639758539 | 2.105E-05 | 0.000202242 | K00949 |
| *TRN2* | 260.4873546 | 125.4001977 | 0.481406085 | -1.054673716 | 2.10721E-05 | 0.000202379 | - |
| *At2g26730* | 68.88279335 | 15.65169083 | 0.227222069 | -2.13782513 | 2.1481E-05 | 0.000205998 | - |
| *BABL* | 199.4009138 | 91.41839854 | 0.458465294 | -1.12511557 | 2.15085E-05 | 0.000206185 | - |
| *NFD4* | 310.9711915 | 120.9688611 | 0.389003433 | -1.362145207 | 2.16559E-05 | 0.000207386 | - |
| *uncharacterized protein_36012* | 606.886556 | 129.0419847 | 0.2126295 | -2.233586329 | 2.17781E-05 | 0.000208458 | - |
| *PAM71* | 232.8713685 | 110.2678476 | 0.473513976 | -1.078521086 | 2.25284E-05 | 0.000215239 | - |
| *RD22* | 394.4709106 | 86.53117742 | 0.219360098 | -2.188626977 | 2.25407E-05 | 0.000215276 | - |
| *NHL2* | 2147.498548 | 1047.123294 | 0.487601398 | -1.036225832 | 2.26516E-05 | 0.000216255 | - |
| *FPP7* | 1170.145785 | 491.6510855 | 0.42016225 | -1.250981548 | 2.26841E-05 | 0.000216485 | - |
| *IAN9* | 256.807018 | 90.7010288 | 0.353187501 | -1.501493809 | 2.28428E-05 | 0.000217919 | - |
| *RIC7* | 254.9612931 | 123.1500495 | 0.483014688 | -1.049861033 | 2.31819E-05 | 0.000220908 | - |
| *At5g48740* | 22.13328178 | 0 | 0 | -Inf | 2.32392E-05 | 0.000221289 | - |
| *PM19L* | 132.0459336 | 54.029522 | 0.409172176 | -1.28922005 | 2.33101E-05 | 0.0002218 | - |
| *HIPP37* | 311.42348 | 155.3984657 | 0.498994057 | -1.002905462 | 2.34593E-05 | 0.000223055 | - |
| *STR4* | 1005.246504 | 401.9507586 | 0.399852929 | -1.32245864 | 2.36539E-05 | 0.000224489 | - |
| *RAM2* | 54.63411432 | 7.698903465 | 0.140917512 | -2.827077186 | 2.3984E-05 | 0.000227286 | K13508 |
| *MYB2* | 271.2989325 | 132.7387282 | 0.489271104 | -1.031294015 | 2.43886E-05 | 0.00023078 | K09422 |
| *GC6* | 278.2090974 | 136.5042368 | 0.49065339 | -1.027223866 | 2.46E-05 | 0.000232609 | - |
| *H1* | 1371.284843 | 355.7527619 | 0.259430244 | -1.946581417 | 2.46364E-05 | 0.000232813 | K11275 |
| *CSE* | 48.22514497 | 11.58932423 | 0.240317043 | -2.05698913 | 2.46397E-05 | 0.000232813 | - |
| *H1* | 1947.881297 | 778.9409328 | 0.399891376 | -1.322319925 | 2.50745E-05 | 0.000236573 | K11275 |
| *PME1* | 26.35621074 | 0 | 0 | -Inf | 2.51778E-05 | 0.000237461 | K01051 |
| *CYCA2-2* | 20.52790048 | 1.195395169 | 0.058232705 | -4.102026559 | 2.57551E-05 | 0.000242283 | K06627 |
| *RFS6* | 99.24431632 | 36.73646571 | 0.37016191 | -1.433771646 | 2.585E-05 | 0.000243087 | K06617 |
| *HPT1* | 14.67191786 | 0.291413198 | 0.01986197 | -5.653847439 | 2.60685E-05 | 0.000244962 | K09833 |
| *NAGK* | 207.9387552 | 96.88431839 | 0.465927183 | -1.101823594 | 2.61558E-05 | 0.000245602 | K00930 |
| *At4g09670* | 16.84220858 | 0.600215152 | 0.035637556 | -4.810457787 | 2.62775E-05 | 0.000246565 | - |
| *comta* | 234.8324548 | 90.22981486 | 0.384230599 | -1.379955678 | 2.65109E-05 | 0.000248664 | K00545 |
| *BCP* | 63.46714059 | 3.583902157 | 0.056468625 | -4.146406689 | 2.65668E-05 | 0.000249098 | - |
| *uncharacterized protein_17895* | 295.4985988 | 147.0014936 | 0.497469342 | -1.007320476 | 2.66411E-05 | 0.000249703 | - |
| *GALS1* | 12.4757495 | 0 | 0 | -Inf | 2.77441E-05 | 0.000259097 | - |
| *slr0537* | 70.61412444 | 22.22018745 | 0.314670579 | -1.668085797 | 2.78762E-05 | 0.000260141 | - |
| *GGPS1* | 12.456727 | 0 | 0 | -Inf | 2.79333E-05 | 0.000260579 | K13789 |
| *PCAP1* | 13.92205969 | 0 | 0 | -Inf | 2.80769E-05 | 0.000261824 | - |
| *DUR3* | 166.4975375 | 73.66914549 | 0.442463874 | -1.176368427 | 2.81471E-05 | 0.000262287 | - |
| *MYB5* | 350.526449 | 98.75288958 | 0.281727356 | -1.827628439 | 2.84363E-05 | 0.000264886 | K09422 |
| *PIP2-8* | 296.976034 | 148.4608135 | 0.499908398 | -1.000264331 | 2.84967E-05 | 0.000265353 | K09872 |
| *LRK10L-1.2* | 20.15300736 | 1.481773232 | 0.073526159 | -3.765598561 | 2.91372E-05 | 0.000270747 | - |
| *CESA9* | 17.6560973 | 0.900322728 | 0.050992171 | -4.293580434 | 2.94721E-05 | 0.000273742 | K10999 |
| *XTH32* | 17.66126563 | 0.89162835 | 0.050484963 | -4.308002433 | 2.9498E-05 | 0.000273882 | K08235 |
| *SWEET3B* | 36.84313675 | 4.092138394 | 0.111069218 | -3.170469053 | 2.95257E-05 | 0.000274041 | K15382 |
| *H1* | 485.4205126 | 212.0067368 | 0.436748615 | -1.195124965 | 2.96182E-05 | 0.000274602 | K11275 |
| *APX2* | 424.4332918 | 124.190195 | 0.292602389 | -1.772986548 | 2.96938E-05 | 0.000275035 | K00434 |
| *XTH32* | 22.33015323 | 2.075577356 | 0.092949535 | -3.427408544 | 2.9697E-05 | 0.000275035 | K08235 |
| *BPA1* | 50.98130617 | 4.147963905 | 0.081362449 | -3.619493091 | 3.05935E-05 | 0.000282726 | - |
| *GSVIVT00026920001* | 27.13725876 | 1.474454746 | 0.054333224 | -4.202021546 | 3.0721E-05 | 0.000283802 | - |
| *uncharacterized protein_30087* | 766.6308667 | 329.2684994 | 0.429500707 | -1.219267588 | 3.07856E-05 | 0.000284297 | - |
| *KRP7* | 362.770569 | 114.1902556 | 0.314772656 | -1.667617874 | 3.09435E-05 | 0.000285447 | - |
| *uncharacterized protein_04607* | 94.52887773 | 34.64027938 | 0.366451821 | -1.448304561 | 3.13467E-05 | 0.000288855 | - |
| *At1g18250* | 42.1462765 | 9.142708377 | 0.216928022 | -2.20471167 | 3.1503E-05 | 0.000290192 | - |
| *At2g01630* | 195.3542478 | 90.37299376 | 0.462610846 | -1.112129005 | 3.15792E-05 | 0.000290789 | K19891 |
| *IGS1* | 17.23626806 | 0 | 0 | -Inf | 3.21171E-05 | 0.000295319 | - |
| *PAP18* | 166.3300253 | 73.90610907 | 0.444334142 | -1.170283095 | 3.22934E-05 | 0.000296409 | - |
| *TBL36* | 33.69313895 | 5.92846882 | 0.175954779 | -2.506723396 | 3.2332E-05 | 0.000296658 | - |
| *ASPR* | 241.7489712 | 45.11970835 | 0.186638678 | -2.421680103 | 3.29878E-05 | 0.000302351 | K08245 |
| *At5g48740* | 34.76971727 | 0.600215152 | 0.017262584 | -5.856207786 | 3.31288E-05 | 0.000303426 | - |
| *NPF4.5* | 20.68176728 | 1.471702961 | 0.071159439 | -3.812801049 | 3.35754E-05 | 0.000307193 | K14638 |
| *At4g09670* | 171.8007062 | 77.29514364 | 0.449911676 | -1.152286287 | 3.35759E-05 | 0.000307193 | - |
| *ag1* | 108.8878866 | 37.84172629 | 0.347529257 | -1.524793658 | 3.4176E-05 | 0.000312477 | K16086 |
| *RBCX2* | 232.5137462 | 109.106791 | 0.469248777 | -1.091575111 | 3.41778E-05 | 0.000312477 | - |
| *SRD5A2* | 197.4988047 | 76.44285951 | 0.387054796 | -1.36939027 | 3.48557E-05 | 0.000318335 | K10258 |
| *uncharacterized protein_37868* | 143.7943725 | 0.882933972 | 0.006140254 | -7.347485947 | 3.51983E-05 | 0.000321351 | - |
| *YAB5* | 107.2847315 | 9.166039726 | 0.085436572 | -3.549002422 | 3.5272E-05 | 0.000321809 | - |
| *COMT1* | 178.8298288 | 81.42793416 | 0.455337539 | -1.134991692 | 3.59752E-05 | 0.000328093 | K13066 |
| *uncharacterized protein_08235* | 128.0611149 | 53.12603787 | 0.414849097 | -1.269341449 | 3.65205E-05 | 0.000332712 | - |
| *At5g04720* | 170.6392872 | 54.41935455 | 0.318914568 | -1.648758094 | 3.66891E-05 | 0.000334011 | - |
| *uncharacterized protein_41100* | 12.19927057 | 0 | 0 | -Inf | 3.67315E-05 | 0.000334279 | - |
| *DDB_G0268948* | 172.350456 | 19.7328515 | 0.114492598 | -3.126673763 | 3.72455E-05 | 0.000338837 | - |
| *GAD* | 167.6843808 | 28.26161521 | 0.168540535 | -2.568832487 | 3.74329E-05 | 0.00034006 | K01580 |
| *uncharacterized protein_11316* | 42.13931345 | 9.464771409 | 0.224606683 | -2.154527242 | 3.80207E-05 | 0.000345034 | - |
| *LECRK81* | 24.89098594 | 2.964922356 | 0.119116308 | -3.069557147 | 3.8037E-05 | 0.000345059 | - |
| *uncharacterized protein_10002* | 12.09041181 | 0 | 0 | -Inf | 3.8185E-05 | 0.00034628 | - |
| *LAC17* | 32.94137818 | 0.291413198 | 0.008846418 | -6.820690886 | 3.83882E-05 | 0.000348 | K05909 |
| *At1g56130* | 40.76385299 | 8.899333769 | 0.218314343 | -2.19552118 | 3.87467E-05 | 0.000350878 | - |
| *DTX42* | 278.4473656 | 125.9273548 | 0.452248325 | -1.144812934 | 3.91162E-05 | 0.000354099 | - |
| *PT30* | 115.8587154 | 27.62027949 | 0.238396217 | -2.06856675 | 3.91608E-05 | 0.000354378 | K12742 |
| *FLZ5* | 17.19301876 | 0.89162835 | 0.051859907 | -4.269236577 | 3.92322E-05 | 0.000354899 | - |
| *PAP27* | 39.27960569 | 8.328860994 | 0.212040341 | -2.237589327 | 3.94876E-05 | 0.000356959 | - |
| *EDR2* | 249.0017279 | 122.5433645 | 0.492138611 | -1.022863387 | 3.95117E-05 | 0.000357051 | - |
| *CYP76C4* | 67.31556494 | 15.12791017 | 0.224731237 | -2.153727427 | 3.97431E-05 | 0.000359015 | K05280 |
| *CSPL3* | 200.7661455 | 76.67106073 | 0.381892378 | -1.388761968 | 3.97813E-05 | 0.000359055 | - |
| *uncharacterized protein_49272* | 75.59921568 | 20.40994016 | 0.269975554 | -1.889099315 | 3.97894E-05 | 0.000359055 | - |
| *KIN14B* | 214.6363566 | 102.3842057 | 0.47701241 | -1.067901296 | 4.0443E-05 | 0.000364441 | K10406 |
| *PARC* | 34.61940392 | 3.253583769 | 0.093981508 | -3.41147928 | 4.09515E-05 | 0.000368894 | K00799 |
| *AAP2* | 48.08689107 | 12.05865527 | 0.250768037 | -1.995574622 | 4.11893E-05 | 0.000370775 | - |
| *DOF5.3* | 90.46477522 | 33.11822507 | 0.366089729 | -1.449730796 | 4.19624E-05 | 0.000377471 | - |
| *uncharacterized protein_23745* | 134.7794758 | 57.44554716 | 0.426218805 | -1.230333847 | 4.25914E-05 | 0.000382995 | - |
| *PCMP-H40* | 149.4842344 | 65.72915077 | 0.439706241 | -1.185388085 | 4.31384E-05 | 0.000387506 | - |
| *CYT1* | 201.9703462 | 82.06480972 | 0.406321083 | -1.299307872 | 4.45732E-05 | 0.000399557 | K00966 |
| *At1g49730* | 140.2050767 | 60.48001539 | 0.431368227 | -1.213008177 | 4.49399E-05 | 0.000402423 | - |
| *At5g48740* | 14.03608679 | 0.300107576 | 0.021381143 | -5.547517224 | 4.50212E-05 | 0.00040287 | - |
| *PCMP-H42* | 124.810973 | 52.04226431 | 0.416968661 | -1.261989139 | 4.5357E-05 | 0.000405592 | - |
| *TOGT1* | 45.19602943 | 10.90948378 | 0.241381465 | -2.050613197 | 4.556E-05 | 0.000407124 | - |
| *EXO70A1* | 26.42709643 | 3.553691345 | 0.134471502 | -2.894627636 | 4.59294E-05 | 0.000409855 | K07195 |
| *GT11* | 13.96695984 | 0.291413198 | 0.020864469 | -5.582807998 | 4.61076E-05 | 0.000411302 | - |
| *uncharacterized protein_02297* | 113.4601906 | 45.80546198 | 0.403713952 | -1.308594648 | 4.64634E-05 | 0.000414045 | - |
| *CAD* | 37.47911168 | 7.711725521 | 0.205760627 | -2.280961151 | 4.67009E-05 | 0.000415584 | K00083 |
| *SBT5.6* | 369.9913775 | 25.9798215 | 0.07021737 | -3.832028226 | 4.70369E-05 | 0.000417995 | - |
| *CBP1* | 46.35390913 | 2.060940385 | 0.044460983 | -4.491316331 | 4.71492E-05 | 0.000418849 | K16296 |
| *PCMP-H40* | 165.7822499 | 75.33934586 | 0.454447602 | -1.137814136 | 4.79197E-05 | 0.000425253 | - |
| *OMT1* | 191.5303957 | 88.78285771 | 0.46354448 | -1.109220313 | 4.89378E-05 | 0.000433538 | K13066 |
| *LTPG16* | 23.62790172 | 2.668474023 | 0.11293741 | -3.146404644 | 4.95148E-05 | 0.000438197 | - |
| *At5g63180* | 11.8035962 | 0 | 0 | -Inf | 5.07603E-05 | 0.000447984 | K01728 |
| *MST3* | 280.8479227 | 91.771589 | 0.326766131 | -1.613669639 | 5.09588E-05 | 0.000449582 | - |
| *UGT75L6* | 12.45496825 | 0 | 0 | -Inf | 5.11973E-05 | 0.000451531 | K13692 |
| *SAMDC* | 11.74139633 | 0 | 0 | -Inf | 5.19006E-05 | 0.000457577 | K01611 |
| *CSPL5* | 123.0621842 | 3.252676312 | 0.02643116 | -5.241616433 | 5.21779E-05 | 0.000459587 | - |
| *yabD* | 68.37991108 | 22.10121794 | 0.323212148 | -1.629446671 | 5.27755E-05 | 0.000464334 | K03424 |
| *At5g08100* | 109.3736849 | 43.72669381 | 0.399791722 | -1.322679495 | 5.30167E-05 | 0.000466137 | K13051 |
| *LOC_Os07g01090* | 24.37779586 | 1.178006413 | 0.048322925 | -4.371148391 | 5.34703E-05 | 0.000469964 | - |
| *CRK2* | 27.78697996 | 2.068258871 | 0.074432661 | -3.747920368 | 5.46521E-05 | 0.000479695 | - |
| *DTX16* | 115.0791814 | 47.3156311 | 0.411157175 | -1.282238091 | 5.50276E-05 | 0.000482332 | K03327 |
| *PCMP-H42* | 49.30510401 | 12.97223908 | 0.263101343 | -1.926309482 | 5.56058E-05 | 0.000486735 | - |
| *UGT72E1* | 89.20141748 | 10.89713016 | 0.122163195 | -3.033118395 | 5.58193E-05 | 0.000488271 | K13496 |
| *At4g26790* | 24.28279126 | 2.925109709 | 0.120460192 | -3.053371626 | 5.68399E-05 | 0.000496354 | - |
| *DUT* | 25.97439054 | 3.516630483 | 0.135388373 | -2.884824244 | 5.69451E-05 | 0.000497104 | K01520 |
| *GLNA1* | 154.8841015 | 69.71007716 | 0.450078972 | -1.151749933 | 5.74208E-05 | 0.000501086 | K01915 |
| *HCT* | 80.0763642 | 16.29631474 | 0.203509674 | -2.296830722 | 5.82845E-05 | 0.000508105 | K13065 |
| *BPS1* | 41.17000788 | 9.486726866 | 0.230428104 | -2.117611409 | 5.85684E-05 | 0.000510088 | - |
| *CYSEP* | 23.90775428 | 1.764492052 | 0.073804174 | -3.760153783 | 5.85714E-05 | 0.000510088 | K16292 |
| *CKX3* | 26.87987426 | 3.856550707 | 0.14347354 | -2.801143404 | 5.93966E-05 | 0.000516573 | K00279 |
| *Os01g0723500* | 163.2990142 | 69.82535801 | 0.427592036 | -1.225693111 | 6.07216E-05 | 0.00052667 | - |
| *CHS* | 2059.275627 | 858.6465434 | 0.416965331 | -1.26200066 | 6.08297E-05 | 0.00052743 | K00660 |
| *GGP3* | 98.56857362 | 21.18169154 | 0.21489295 | -2.218309943 | 6.17425E-05 | 0.000534263 | - |
| *CRK2* | 27.54861795 | 4.119128985 | 0.149522164 | -2.741568736 | 6.38034E-05 | 0.000550797 | - |
| *PT5* | 132.5795883 | 9.459736274 | 0.071351378 | -3.808914905 | 6.42934E-05 | 0.000554655 | K12742 |
| *TAR4* | 28.28121237 | 4.476437965 | 0.1582831 | -2.659420869 | 6.64731E-05 | 0.000570967 | - |
| *CRRSP38* | 15.13506833 | 0.595180017 | 0.039324568 | -4.668425289 | 6.70811E-05 | 0.00057542 | - |
| *UGT86A2* | 25.72737867 | 3.532643347 | 0.13731066 | -2.864484462 | 6.80957E-05 | 0.000583733 | - |
| *SWEET6B* | 15.02272804 | 0.586485639 | 0.039039889 | -4.67890723 | 6.93197E-05 | 0.000592953 | K15382 |
| *GAMT2* | 80.70871376 | 23.472345 | 0.290827891 | -1.781762462 | 7.10745E-05 | 0.000605969 | K18886 |
| *TBL1* | 21.00314636 | 2.074669899 | 0.098779005 | -3.339651754 | 7.10906E-05 | 0.000605969 | - |
| *At4g15970* | 56.76463726 | 16.90703919 | 0.297844574 | -1.747368419 | 7.12784E-05 | 0.000607166 | - |
| *WAXY* | 9128.243984 | 3352.515299 | 0.367268371 | -1.445093437 | 7.1833E-05 | 0.000611688 | K13679 |
| *RPT3* | 152.6049089 | 69.27417715 | 0.453944618 | -1.139411797 | 7.31021E-05 | 0.000621464 | - |
| *uncharacterized protein_36414* | 19.79699288 | 1.790575186 | 0.090446827 | -3.466786301 | 7.51227E-05 | 0.000636536 | - |
| *At1g56140* | 200.4980287 | 74.98572554 | 0.373997321 | -1.41890016 | 7.53496E-05 | 0.000638248 | - |
| *NAKR2* | 60.60761265 | 18.94830747 | 0.312639067 | -1.677430028 | 7.5543E-05 | 0.000639675 | - |
| *RL6* | 103.5956371 | 0.295072441 | 0.00284831 | -8.455678349 | 7.61771E-05 | 0.000644408 | - |
| *mhpC* | 108.1588167 | 44.36584357 | 0.410191651 | -1.285629968 | 7.63028E-05 | 0.000645047 | - |
| *DALL4* | 34.8785112 | 7.06162745 | 0.202463557 | -2.304265843 | 7.66657E-05 | 0.000647901 | - |
| *Prcp* | 72.94341434 | 25.02266841 | 0.343042187 | -1.543542085 | 7.68594E-05 | 0.000649324 | K01285 |
| *WNK11* | 24.60413437 | 3.23253577 | 0.131381813 | -2.92816252 | 7.70949E-05 | 0.0006511 | K08867 |
| *PME53* | 36.57354894 | 0 | 0 | -Inf | 7.76982E-05 | 0.000655333 | K01051 |
| *At3g47110* | 19.51197198 | 1.468043719 | 0.075238101 | -3.732392754 | 7.86671E-05 | 0.000662635 | K13420 |
| *surE* | 879.6389704 | 359.4862063 | 0.408674716 | -1.290975106 | 8.01403E-05 | 0.00067416 | K03787 |
| *GAST1* | 11.46836296 | 0 | 0 | -Inf | 8.14262E-05 | 0.000684081 | - |
| *At3g47570* | 21.65631312 | 2.392166231 | 0.110460456 | -3.178398102 | 8.39214E-05 | 0.000703331 | - |
| *NRT2.1* | 25.99337707 | 3.247641176 | 0.124941102 | -3.000679935 | 8.39692E-05 | 0.000703375 | K02575 |
| *CAD* | 71.28800051 | 24.51261726 | 0.343853343 | -1.540134725 | 8.42135E-05 | 0.000705192 | K00083 |
| *GLIP6* | 38.16849277 | 8.547059925 | 0.223929721 | -2.158882075 | 8.42597E-05 | 0.000705349 | - |
| *P85* | 682.832459 | 338.9115321 | 0.4963319 | -1.010622912 | 8.53385E-05 | 0.00071345 | - |
| *EMF1* | 131.7486015 | 58.00419358 | 0.440264207 | -1.183558535 | 8.56542E-05 | 0.000715856 | - |
| *At5g03810* | 88.40667384 | 2.359672069 | 0.026691108 | -5.227497 | 8.61682E-05 | 0.000719918 | - |
| *SOQ1* | 173.6685035 | 82.26971816 | 0.473716975 | -1.077902725 | 8.63941E-05 | 0.00072157 | - |
| *ATL13* | 150.2687279 | 68.49228751 | 0.455798678 | -1.133531355 | 8.78981E-05 | 0.000733178 | - |
| *CSF7* | 7335.821374 | 2184.25281 | 0.297751635 | -1.747818664 | 8.86352E-05 | 0.000738847 | K00660 |
| *SBT1.5* | 201.507059 | 55.74234563 | 0.27662726 | -1.853984761 | 8.91439E-05 | 0.000742606 | - |
| *HSP21* | 887.855636 | 167.6633559 | 0.188840786 | -2.404757704 | 8.92231E-05 | 0.000743024 | K13993 |
| *CHI4* | 63.59512975 | 20.55173401 | 0.323165219 | -1.629656161 | 9.02833E-05 | 0.000751123 | K01183 |
| *AKR1* | 161.7422592 | 75.46247246 | 0.466560025 | -1.099865394 | 9.24382E-05 | 0.000767808 | - |
| *PAP27* | 24.21021875 | 3.296148202 | 0.136146981 | -2.876763099 | 9.37679E-05 | 0.000777023 | - |
| *CBL2* | 157.9546357 | 73.08715857 | 0.462709804 | -1.111820426 | 9.40246E-05 | 0.000778468 | K06268 |
| *ABCG36* | 151.6734037 | 61.14284728 | 0.403121746 | -1.310712486 | 9.41565E-05 | 0.000779068 | - |
| *HEME1* | 217.2387518 | 107.7801021 | 0.496136629 | -1.011190621 | 9.41578E-05 | 0.000779068 | K01599 |
| *CYT1* | 268.0241683 | 102.3878649 | 0.382009822 | -1.388318361 | 9.41987E-05 | 0.000779156 | K00966 |
| *BAT1* | 59.98035951 | 18.85632855 | 0.314375051 | -1.669441368 | 9.43501E-05 | 0.000779906 | K15400 |
| *BOR2* | 50.66832521 | 6.204776612 | 0.122458688 | -3.029632967 | 9.45346E-05 | 0.00078118 | - |
| *GAPC* | 268.7800149 | 119.8332538 | 0.445841384 | -1.165397558 | 9.49781E-05 | 0.00078434 | K00134 |
| *At1g64390* | 31.08733417 | 2.358296176 | 0.075860354 | -3.720510085 | 9.52157E-05 | 0.00078605 | - |
| *SB09* | 1217.484326 | 120.302526 | 0.098812382 | -3.339164359 | 9.59075E-05 | 0.000790745 | K03671 |
| *CESA2* | 11.01737985 | 0 | 0 | -Inf | 9.69081E-05 | 0.000798738 | K10999 |
| *GGCT2;1* | 39.54571197 | 8.273035483 | 0.209201834 | -2.257032594 | 9.71112E-05 | 0.000799806 | - |
| *DOF5.7* | 70.14232412 | 24.18958794 | 0.344864363 | -1.53589904 | 9.74503E-05 | 0.000802178 | - |
| *RBOHF* | 10.95858957 | 0 | 0 | -Inf | 9.89788E-05 | 0.000812416 | K13447 |
| *ATJ8* | 29.14000124 | 5.020827606 | 0.172300185 | -2.537003844 | 0.000101144 | 0.000828337 | - |
| *At3g01520* | 15.68967704 | 0 | 0 | -Inf | 0.000101333 | 0.000829619 | - |
| *CLC-E* | 279.3872188 | 135.8989571 | 0.486417946 | -1.039731638 | 0.000102626 | 0.000839938 | - |
| *uncharacterized protein_50766* | 15.90743052 | 0 | 0 | -Inf | 0.000104067 | 0.00085092 | - |
| *DHAPS-1* | 13.58330897 | 0 | 0 | -Inf | 0.000105221 | 0.000859027 | K01626 |
| *uncharacterized protein_04845* | 59.53799028 | 18.99453114 | 0.319032118 | -1.648226423 | 0.000105226 | 0.000859027 | - |
| *DCOR* | 115.0242541 | 7.395136646 | 0.064291977 | -3.959217485 | 0.000105365 | 0.000859888 | K01581 |
| *MST3* | 696.8734406 | 294.7408099 | 0.4229474 | -1.241449841 | 0.000106129 | 0.000865573 | - |
| *DCOR* | 140.4454923 | 17.47616548 | 0.124433794 | -3.006549743 | 0.000106313 | 0.000866801 | K01581 |
| *MGD A* | 149.3737247 | 68.82117118 | 0.460731439 | -1.118002048 | 0.000106729 | 0.00086964 | K03715 |
| *TCEA1* | 1318.249978 | 403.6534257 | 0.306204007 | -1.707434932 | 0.000108369 | 0.000881886 | - |
| *PCMP-E76* | 162.0705654 | 76.12573422 | 0.46970734 | -1.090165955 | 0.000109398 | 0.000888853 | - |
| *CIPK5* | 249.1016562 | 95.17411059 | 0.382069361 | -1.388093526 | 0.000109772 | 0.000891607 | K07198 |
| *At4g33820* | 44.27165997 | 10.03524418 | 0.226674224 | -2.141307747 | 0.000112444 | 0.000909856 | - |
| *WAXY* | 16813.90718 | 6468.870826 | 0.384733349 | -1.378069204 | 0.000113755 | 0.000919613 | K13679 |
| *DREB3* | 20.14776709 | 2.059564493 | 0.102222965 | -3.29020875 | 0.000113757 | 0.000919613 | K09286 |
| *CYP75B137* | 36.49577922 | 0.88155808 | 0.024155069 | -5.371530192 | 0.000113885 | 0.000920359 | K05280 |
| *uncharacterized protein_14370* | 194.9237151 | 68.85006493 | 0.353215436 | -1.501379705 | 0.000114009 | 0.000921073 | - |
| *uncharacterized protein_14130* | 895.7007925 | 323.6332496 | 0.361318481 | -1.468657047 | 0.000115719 | 0.000933712 | - |
| *EMB2761* | 140.4713198 | 63.9133999 | 0.454992521 | -1.136085264 | 0.000116271 | 0.000937575 | K01868 |
| *SIGA* | 506.62448 | 247.3937958 | 0.488317887 | -1.034107469 | 0.000116823 | 0.000941732 | K03086 |
| *LOX1.5* | 299.7051849 | 86.02650303 | 0.287037086 | -1.800690944 | 0.000117583 | 0.000947315 | K15718 |
| *YUC9* | 19.15078916 | 1.764492052 | 0.09213678 | -3.440079007 | 0.000117589 | 0.000947315 | K11816 |
| *5NG4* | 22.03813332 | 2.658403752 | 0.120627447 | -3.05136989 | 0.000117735 | 0.000947598 | - |
| *POPTRDRAFT_746969* | 163.5532338 | 77.22686712 | 0.472181842 | -1.082585533 | 0.000118377 | 0.000952169 | K00620 |
| *MYB16* | 18.01552137 | 1.491843502 | 0.082808789 | -3.594072297 | 0.000120381 | 0.000965564 | K09422 |
| *Prcp* | 74.93399656 | 27.13483819 | 0.362116522 | -1.465474093 | 0.000121006 | 0.000970115 | K01285 |
| *uncharacterized protein_43515* | 30.09032027 | 5.616007623 | 0.186638347 | -2.421682663 | 0.000125768 | 0.00100407 | - |
| *RAN1A* | 74.18054898 | 26.73912187 | 0.360460016 | -1.472088857 | 0.000127001 | 0.001012974 | K07936 |
| *uncharacterized protein_38958* | 25.40420489 | 3.868904327 | 0.152293856 | -2.715070352 | 0.000128195 | 0.001021862 | - |
| *DTX16* | 54.798217 | 16.86494319 | 0.307764451 | -1.700101493 | 0.000128486 | 0.001023752 | K03327 |
| *RUP1* | 22.52723337 | 1.165652792 | 0.051744161 | -4.272460114 | 0.000128512 | 0.001023752 | K10143 |
| *RD21A* | 91.07799438 | 35.29715952 | 0.387548713 | -1.367550433 | 0.000131573 | 0.001045868 | - |
| *DIR* | 390.0831303 | 113.962767 | 0.29214995 | -1.775219052 | 0.000131712 | 0.001046646 | - |
| *CYP76B10* | 124.9456446 | 55.52048746 | 0.444357125 | -1.170208473 | 0.000132285 | 0.001050554 | - |
| *ag1* | 29.18490848 | 0.877898837 | 0.030080575 | -5.055024031 | 0.000133499 | 0.001059211 | K16086 |
| *At1g28650* | 10.63376494 | 0 | 0 | -Inf | 0.000133544 | 0.001059237 | - |
| *RABA5D* | 36.48895294 | 8.235974621 | 0.225711454 | -2.147448464 | 0.00013395 | 0.001062129 | K07904 |
| *At5g48740* | 10.60609258 | 0 | 0 | -Inf | 0.000134881 | 0.001069187 | - |
| *CRJ35* | 10.59399716 | 0 | 0 | -Inf | 0.000135471 | 0.001073201 | - |
| *EMB2261* | 83.6375196 | 32.30991067 | 0.38630881 | -1.372173516 | 0.000135745 | 0.001075032 | - |
| *TCX2* | 106.8161179 | 36.08223996 | 0.337797709 | -1.565768549 | 0.000135786 | 0.001075032 | - |
| *IRX14* | 60.48316985 | 18.05073652 | 0.298442303 | -1.744476047 | 0.000138418 | 0.00109385 | - |
| *UBI11* | 1845.214092 | 852.4663706 | 0.461987785 | -1.114073389 | 0.000138786 | 0.001096081 | K08770 |
| *ACT* | 1025.465681 | 428.0132528 | 0.417384278 | -1.260551836 | 0.00014163 | 0.001116486 | K10355 |
| *SKOR* | 245.2984268 | 84.38147833 | 0.343995188 | -1.539539713 | 0.000144235 | 0.001134235 | - |
| *H1* | 21.63036354 | 2.675792508 | 0.123705388 | -3.01501976 | 0.000145725 | 0.001144897 | K11275 |
| *uncharacterized protein_39534* | 28.26222584 | 5.031805335 | 0.178039952 | -2.489727077 | 0.000146294 | 0.001148974 | - |
| *XYN5* | 55.62595987 | 17.46791013 | 0.314024426 | -1.671051312 | 0.000147201 | 0.001154732 | - |
| *uncharacterized protein_23270* | 141.9989243 | 65.63623498 | 0.462230509 | -1.11331561 | 0.000149619 | 0.001172268 | - |
| *UGT73C11* | 54.04481247 | 4.464084344 | 0.082599682 | -3.597719956 | 0.00015076 | 0.001180129 | K13496 |
| *CRSP* | 137.8444891 | 15.66132208 | 0.113615874 | -3.13776368 | 0.000153961 | 0.001200828 | - |
| *RPL31* | 550.160143 | 248.2750417 | 0.451277768 | -1.147912387 | 0.000156247 | 0.001217514 | K02909 |
| *HSP21* | 13.93063762 | 0.582826396 | 0.04183774 | -4.579051265 | 0.00015645 | 0.001218729 | K13993 |
| *At1g06550* | 306.1256672 | 110.7378713 | 0.36173991 | -1.466975318 | 0.000156803 | 0.001220363 | K05605 |
| *At5g39980* | 172.0027459 | 83.76525032 | 0.486999494 | -1.03800782 | 0.000157567 | 0.001225199 | - |
| *PCMP-H40* | 85.24451579 | 33.27418746 | 0.39033816 | -1.357203585 | 0.000159043 | 0.001235551 | - |
| *Fra a 1.06* | 25.93817621 | 0.300107576 | 0.011570111 | -6.433453493 | 0.000159664 | 0.001240004 | - |
| *ISPH* | 2096.866005 | 1024.641676 | 0.488653864 | -1.033115194 | 0.000160509 | 0.001246189 | K03527 |
| *At5g15010* | 90.46642607 | 36.25649857 | 0.400772973 | -1.319142873 | 0.00016517 | 0.001278894 | - |
| *CEPR1* | 561.4575335 | 208.9179457 | 0.372099283 | -1.426240484 | 0.000170245 | 0.001313045 | - |
| *Os01g0552300* | 169.9900553 | 45.26104292 | 0.266257005 | -1.909108616 | 0.000171709 | 0.001323151 | - |
| *MES13* | 20.4692181 | 1.172971278 | 0.057304157 | -4.125216402 | 0.000173341 | 0.001334527 | - |
| *SCL3* | 130.495933 | 59.62541849 | 0.456913998 | -1.130005453 | 0.000174161 | 0.001340431 | - |
| *BTR1* | 151.1051997 | 64.89512428 | 0.429469829 | -1.219371312 | 0.000175268 | 0.001348147 | K14944 |
| *uncharacterized protein_37773* | 36.45438947 | 8.579554087 | 0.235350371 | -2.087117967 | 0.00017629 | 0.00135479 | - |
| *uncharacterized protein_34948* | 49.56083766 | 14.73216443 | 0.297254145 | -1.750231167 | 0.000176617 | 0.0013569 | - |
| *R12* | 10.31066308 | 0 | 0 | -Inf | 0.000180152 | 0.001381165 | K00430 |
| *PLP6* | 10.30025448 | 0 | 0 | -Inf | 0.000180827 | 0.001385145 | - |
| *LDOX* | 3496.652998 | 764.8349331 | 0.218733438 | -2.192754309 | 0.000180833 | 0.001385145 | K05277 |
| *CYT1* | 666.9101243 | 202.5380233 | 0.303696129 | -1.719299574 | 0.000181222 | 0.001387297 | K00966 |
| *NPF5.3* | 50.63910989 | 15.38273094 | 0.303771748 | -1.718940394 | 0.000181754 | 0.001390545 | K14638 |
| *CSE* | 10.2760996 | 0 | 0 | -Inf | 0.000182404 | 0.001393875 | - |
| *At3g02645* | 10.27606364 | 0 | 0 | -Inf | 0.000182407 | 0.001393875 | - |
| *GDI1* | 23.83518177 | 3.529891561 | 0.148095852 | -2.755396858 | 0.000182605 | 0.001394776 | K12462 |
| *FLZ15* | 10.27261808 | 0 | 0 | -Inf | 0.000182633 | 0.001394776 | - |
| *DTX35* | 90.41973121 | 36.68658279 | 0.405736473 | -1.301385099 | 0.000185235 | 0.0014127 | K03327 |
| *SCPL42* | 18.41119574 | 1.785540051 | 0.096981211 | -3.366150927 | 0.000185255 | 0.0014127 | K16297 |
| *Bp10* | 368.8841236 | 147.113106 | 0.39880574 | -1.326241919 | 0.000185327 | 0.001412829 | - |
| *PLR_Tp2* | 18.40775018 | 1.765867944 | 0.095930677 | -3.381863945 | 0.000186051 | 0.001416621 | - |
| *HCT* | 84.22669178 | 25.73544204 | 0.305549719 | -1.710520939 | 0.000186972 | 0.001422834 | K13065 |
| *At2g27500* | 18.35595891 | 1.769527187 | 0.096400694 | -3.37481265 | 0.00018772 | 0.001427687 | - |
| *mutS2* | 154.5230554 | 73.91752582 | 0.478359204 | -1.063833737 | 0.00018847 | 0.00143296 | K07456 |
| *At1g56130* | 130.220997 | 52.48873245 | 0.403074263 | -1.310882426 | 0.000189203 | 0.001437684 | - |
| *PUB13* | 73.3667251 | 27.17924695 | 0.37045741 | -1.432620404 | 0.000191908 | 0.001457811 | - |
| *NPF5.2* | 65.29907619 | 6.763803225 | 0.103581913 | -3.271155988 | 0.000192904 | 0.001464511 | - |
| *ag1* | 80.27492247 | 23.27969279 | 0.289999567 | -1.785877347 | 0.000193016 | 0.001464929 | K16086 |
| *PCMP-E76* | 139.0040916 | 50.09438026 | 0.360380617 | -1.472406678 | 0.000193185 | 0.001465772 | - |
| *uncharacterized protein_21545* | 64.16542329 | 22.35053514 | 0.348326778 | -1.52148671 | 0.000193676 | 0.0014682 | - |
| *uncharacterized protein_34482* | 17.24656877 | 1.500537881 | 0.08700501 | -3.522757719 | 0.000194318 | 0.001472632 | - |
| *ACT7* | 101.9260024 | 43.34605348 | 0.425269828 | -1.233549594 | 0.000194704 | 0.001475123 | K10355 |
| *PGLR* | 16.1424189 | 1.172971278 | 0.07266391 | -3.782617187 | 0.000195315 | 0.001478877 | - |
| *GSTU16* | 17.22065516 | 1.485432475 | 0.086258767 | -3.535185102 | 0.000195922 | 0.001482161 | K00799 |
| *POPTRDRAFT_821063* | 320.1233924 | 118.7788515 | 0.371040837 | -1.430350114 | 0.000196449 | 0.001485276 | K01872 |
| *uncharacterized protein_30266* | 108.81535 | 47.46937812 | 0.436237885 | -1.19681303 | 0.000197021 | 0.00148916 | - |
| *ATL16* | 12.09558014 | 0.291413198 | 0.024092536 | -5.375269939 | 0.00019817 | 0.00149697 | K19041 |
| *uncharacterized protein_12336* | 55.0038462 | 17.71034787 | 0.321983808 | -1.634939954 | 0.000199024 | 0.001502979 | K18885 |
| *HB1* | 69.87267147 | 11.87479483 | 0.16994906 | -2.556825713 | 0.000200753 | 0.001514253 | - |
| *UGT85A23* | 13.61622159 | 0.590144882 | 0.04334131 | -4.528113428 | 0.000201912 | 0.001521207 | - |
| *UNG* | 50.46973098 | 15.40468639 | 0.305226243 | -1.712049084 | 0.000203119 | 0.001529408 | K03648 |
| *PT30* | 83.57866449 | 4.466367694 | 0.053439089 | -4.225960775 | 0.000206351 | 0.001549396 | K12742 |
| *LECRKS4* | 13.52459062 | 0.595180017 | 0.044007248 | -4.506115026 | 0.000206634 | 0.00155087 | - |
| *AAE14* | 609.6029853 | 181.5852439 | 0.297874598 | -1.747222998 | 0.00020712 | 0.001554068 | K14760 |
| *uncharacterized protein_41190* | 76.06387314 | 2.065975521 | 0.027161061 | -5.202316337 | 0.000207876 | 0.00155786 | - |
| *WOX4* | 22.72073118 | 3.259994797 | 0.143481069 | -2.801067696 | 0.000209833 | 0.001570752 | - |
| *GSVIVT00023967001* | 22.69474564 | 3.239854256 | 0.142757901 | -2.808357498 | 0.000211151 | 0.001580154 | K00430 |
| *ZHD4* | 65.00005728 | 23.11366013 | 0.355594458 | -1.491695256 | 0.000213529 | 0.001597023 | - |
| *uncharacterized protein_41990* | 83.67914692 | 28.43275098 | 0.339782993 | -1.557314452 | 0.000224706 | 0.001674769 | - |
| *DET2* | 435.9655131 | 164.2173053 | 0.37667499 | -1.408607849 | 0.000229107 | 0.001704604 | K10258 |
| *H1* | 559.6858804 | 255.5945153 | 0.456674939 | -1.130760474 | 0.000229685 | 0.001708408 | K11275 |
| *PAP27* | 138.6433261 | 36.26801271 | 0.2615922 | -1.93460857 | 0.000231371 | 0.001719951 | - |
| *FZR2* | 18.02241249 | 1.795610321 | 0.099632073 | -3.327245945 | 0.00023313 | 0.001730522 | K03364 |
| *uncharacterized protein_13788* | 69.96601813 | 15.02954963 | 0.214812134 | -2.218852609 | 0.000235438 | 0.001745271 | - |
| *LTPG5* | 21.72027173 | 2.99100549 | 0.137705712 | -2.860339688 | 0.000235875 | 0.001747869 | - |
| *MPAO1* | 2476.474881 | 676.1489308 | 0.273028786 | -1.872875028 | 0.000243306 | 0.001800341 | K13366 |
| *At1g01500* | 54.18827067 | 15.70019786 | 0.289734248 | -1.787197866 | 0.000244817 | 0.001810473 | - |
| *MAN1* | 15.81238996 | 1.178006413 | 0.074498948 | -3.746636142 | 0.000247352 | 0.001826072 | K19355 |
| *CYP86B1* | 73.03821025 | 10.72149566 | 0.146792968 | -2.768145236 | 0.000247901 | 0.001829596 | K15402 |
| *EMB2217* | 592.4138823 | 242.3084594 | 0.409018875 | -1.289760676 | 0.000250186 | 0.001845398 | - |
| *NRT3.2* | 15.76228551 | 1.172971278 | 0.07441632 | -3.748237147 | 0.00025032 | 0.001845505 | - |
| *BGLU12* | 9.904544148 | 0 | 0 | -Inf | 0.000250505 | 0.001846167 | K01188 |
| *At5g63180* | 9.89244873 | 0 | 0 | -Inf | 0.000251601 | 0.001853178 | K01728 |
| *ABCB19* | 86.45759635 | 35.08080492 | 0.405757347 | -1.301310877 | 0.00025294 | 0.001861974 | K05658 |
| *uncharacterized protein_07435* | 91.4824265 | 38.17567451 | 0.417300633 | -1.260840985 | 0.000256146 | 0.001883412 | - |
| *CRR2* | 129.0287985 | 59.99508109 | 0.464974345 | -1.104776977 | 0.000256696 | 0.00188638 | - |
| *pgk* | 2343.256235 | 651.5227082 | 0.278041598 | -1.846627353 | 0.000259713 | 0.001905094 | K00927 |
| *uncharacterized protein_18537* | 77.07327016 | 16.18647864 | 0.210014167 | -2.251441443 | 0.000259762 | 0.001905094 | - |
| *GER8* | 21.19140391 | 0 | 0 | -Inf | 0.000260835 | 0.001911869 | - |
| *At1g80120* | 11.73619203 | 0.291413198 | 0.024830302 | -5.331754354 | 0.000263973 | 0.001932666 | - |
| *LTPG5* | 23.17684667 | 3.547748752 | 0.15307297 | -2.707708546 | 0.000265648 | 0.001942165 | - |
| *uncharacterized protein_31758* | 740.6833361 | 144.4588979 | 0.195034627 | -2.35819781 | 0.000268594 | 0.001962024 | - |
| *HCT* | 70.08705133 | 26.29859633 | 0.375227604 | -1.41416213 | 0.000268944 | 0.001964022 | K13065 |
| *CYP703A2* | 105.1366212 | 46.05753096 | 0.438073151 | -1.190756297 | 0.000269161 | 0.001965052 | - |
| *PCMP-H42* | 109.0867325 | 48.50918195 | 0.444684526 | -1.169145892 | 0.000269738 | 0.001968705 | - |
| *DTX27* | 102.4668578 | 44.69704 | 0.436209727 | -1.196906156 | 0.000270685 | 0.001974497 | K03327 |
| *CYP71AU50* | 52.21337327 | 4.405507048 | 0.08437507 | -3.567039388 | 0.000272583 | 0.001987213 | - |
| *FL3H* | 1786.077386 | 592.8339554 | 0.331919524 | -1.591094602 | 0.000274535 | 0.001999545 | K00475 |
| *PCMP-H42* | 72.53898931 | 27.77261205 | 0.382864613 | -1.385093773 | 0.00027731 | 0.002018239 | - |
| *PT30* | 55.11453563 | 14.26968343 | 0.258909619 | -1.949479528 | 0.000278507 | 0.00202637 | K12742 |
| *CRK25* | 45.30840568 | 13.29295563 | 0.293388289 | -1.769116808 | 0.000281224 | 0.002043543 | - |
| *slr0537* | 45.25826526 | 13.33961833 | 0.294744358 | -1.762463898 | 0.000281265 | 0.002043543 | - |
| *uncharacterized protein_27219* | 501.5163597 | 165.0266925 | 0.329055452 | -1.603597368 | 0.000281968 | 0.002048075 | - |
| *cspR* | 60.57645877 | 21.33087186 | 0.352131377 | -1.505814308 | 0.000287858 | 0.002087313 | K03216 |
| *DEGP5* | 33.92471064 | 7.989409205 | 0.235504122 | -2.086175781 | 0.000291146 | 0.002109371 | - |
| *SKOR* | 28.32614849 | 5.639338972 | 0.199085978 | -2.328536483 | 0.000291869 | 0.002114014 | - |
| *PCMP-H53* | 53.1428751 | 17.43679186 | 0.328111564 | -1.607741654 | 0.000292358 | 0.002116364 | - |
| *SOK5* | 117.2356469 | 53.6947343 | 0.458006892 | -1.126558786 | 0.000293092 | 0.00212108 | - |
| *DCR* | 53.02181303 | 9.178393347 | 0.173105988 | -2.530272462 | 0.000293959 | 0.002126156 | K19747 |
| *TOR1L3* | 369.5582083 | 177.2917498 | 0.479739716 | -1.059676216 | 0.00029553 | 0.002136916 | - |
| *Rfwd3* | 20.66453949 | 0 | 0 | -Inf | 0.000296062 | 0.002140162 | K15691 |
| *yuiD* | 141.5702724 | 68.29785895 | 0.482430794 | -1.051606096 | 0.000299661 | 0.002163136 | - |
| *ZOX1* | 19.42202783 | 2.373401582 | 0.122201533 | -3.032665706 | 0.00030693 | 0.002210013 | K13495 |
| *At5g18840* | 16.53640645 | 1.491843502 | 0.090215701 | -3.470477647 | 0.000310235 | 0.002232562 | K08145 |
| *CSF7* | 1178.412819 | 479.4753072 | 0.406882291 | -1.297316605 | 0.000310701 | 0.00223529 | K00660 |
| *uncharacterized protein_21648* | 15.49793796 | 1.172971278 | 0.075685635 | -3.723836682 | 0.00031273 | 0.002246739 | - |
| *KO* | 37.47035393 | 9.748866122 | 0.260175448 | -1.942443269 | 0.000319379 | 0.00229067 | K04122 |
| *ANR* | 77.58121289 | 11.55636163 | 0.148958249 | -2.747020075 | 0.000320259 | 0.002295695 | K13082 |
| *PAP16* | 228.2281569 | 110.2234977 | 0.482953108 | -1.050044977 | 0.000320588 | 0.002297337 | - |
| *REL2* | 82.62155514 | 33.73173071 | 0.408267923 | -1.292411873 | 0.000320667 | 0.002297337 | - |
| *At4g33300* | 42.93762523 | 12.39491625 | 0.288672608 | -1.792493878 | 0.000325236 | 0.002328774 | - |
| *DTX35* | 118.9030913 | 55.08043035 | 0.463238001 | -1.110174486 | 0.000328476 | 0.002350005 | K03327 |
| *uncharacterized protein_13859* | 33.64651602 | 3.840537843 | 0.1141437 | -3.131076856 | 0.000329869 | 0.002358007 | - |
| *BOR2* | 62.74150922 | 22.82818952 | 0.363845081 | -1.458603787 | 0.000334405 | 0.002388433 | - |
| *CCNB1* | 18.39048642 | 2.095717897 | 0.113956632 | -3.133443204 | 0.000336021 | 0.002399306 | K05868 |
| *MLP423* | 9.588333408 | 0 | 0 | -Inf | 0.000337396 | 0.002408457 | - |
| *WNK4* | 69.52376389 | 15.85800458 | 0.228094736 | -2.132294942 | 0.00034169 | 0.002436401 | K08867 |
| *APK1* | 119.6236982 | 55.540628 | 0.464294524 | -1.106887829 | 0.000343325 | 0.0024467 | K00860 |
| *NACK1* | 36.01894733 | 9.161004591 | 0.254338488 | -1.975178301 | 0.000344188 | 0.002452173 | K11498 |
| *NAKR2* | 45.10971065 | 13.57705034 | 0.30097844 | -1.732267948 | 0.00034674 | 0.002468302 | - |
| *CCB1* | 127.1040126 | 60.09481752 | 0.472800317 | -1.080697091 | 0.000346956 | 0.002468473 | - |
| *R72* | 9.50711104 | 0 | 0 | -Inf | 0.000347428 | 0.002471145 | K00430 |
| *ag1* | 9.505388261 | 0 | 0 | -Inf | 0.000347645 | 0.002471915 | K16086 |
| *SCRM2* | 11.40444032 | 0.300107576 | 0.026314976 | -5.247972097 | 0.000347729 | 0.002471915 | - |
| *HSFB4* | 11.39751324 | 0.300107576 | 0.02633097 | -5.247095535 | 0.000348456 | 0.0024764 | K09419 |
| *TIP1-1* | 63.84390035 | 21.94206474 | 0.343683024 | -1.540849502 | 0.000350538 | 0.002489818 | K09873 |
| *CYP716B2* | 11.38369504 | 0.291413198 | 0.025599175 | -5.28775889 | 0.000350832 | 0.002491221 | - |
| *TAR4* | 21.02044608 | 2.963546464 | 0.140983995 | -2.826396704 | 0.00035282 | 0.00250395 | - |
| *ALS3* | 26.33726017 | 5.045534848 | 0.191574022 | -2.384026157 | 0.000354112 | 0.002512037 | K02069 |
| *GMGT1* | 50.89996882 | 12.08383095 | 0.237403504 | -2.074586866 | 0.000354253 | 0.002512037 | - |
| *GAD* | 16.79554969 | 0.300107576 | 0.017868279 | -5.806455463 | 0.000355118 | 0.002516782 | K01580 |
| *RAM2* | 79.11370509 | 19.51556002 | 0.246677361 | -2.019302775 | 0.000357574 | 0.002532096 | K13508 |
| *RBOHA* | 31.2740057 | 7.111510368 | 0.227393652 | -2.136736115 | 0.000370337 | 0.002613828 | K13447 |
| *uncharacterized protein_03480* | 107.6128508 | 45.20524682 | 0.420072942 | -1.251288234 | 0.000373285 | 0.002633185 | - |
| *PNAA* | 49.54705543 | 15.98247765 | 0.322571695 | -1.632308244 | 0.000374666 | 0.002640906 | - |
| *PCMP-H41* | 70.8697502 | 27.5301449 | 0.388461153 | -1.36415776 | 0.000381343 | 0.002684868 | - |
| *FAAH* | 137.74291 | 66.741964 | 0.484540105 | -1.045312015 | 0.000382184 | 0.002689312 | - |
| *CYP701A6* | 64.95853076 | 24.27424837 | 0.373688384 | -1.420092377 | 0.000382421 | 0.002690243 | K04122 |
| *SYP124* | 27.43276018 | 5.029521984 | 0.18333999 | -2.447406593 | 0.000384079 | 0.002699695 | K08486 |
| *At2g14510* | 14.61316355 | 0.291413198 | 0.019941828 | -5.648058507 | 0.000391198 | 0.002746726 | - |
| *A6* | 16.15451431 | 1.473078854 | 0.091186824 | -3.455030812 | 0.00039474 | 0.002767061 | - |
| *neur* | 16.14586445 | 1.478113989 | 0.091547529 | -3.449335249 | 0.000395019 | 0.002768256 | - |
| *uncharacterized protein_37835* | 22.33883905 | 3.511595348 | 0.15719686 | -2.669355698 | 0.000396875 | 0.002779259 | - |
| *PIP2-3* | 28.9827608 | 6.176410128 | 0.213106342 | -2.230354569 | 0.000400496 | 0.002802051 | K09872 |
| *ZAT2* | 81.90633237 | 21.65202744 | 0.26435108 | -1.919472873 | 0.00040247 | 0.002815102 | - |
| *CAT1* | 23.16306444 | 3.832750923 | 0.165468215 | -2.595373982 | 0.000402611 | 0.002815317 | K03294 |
| *NAT12* | 244.6264969 | 110.2066159 | 0.450509725 | -1.150369844 | 0.000411689 | 0.002874885 | K14611 |
| *CRK8* | 24.67333325 | 4.451730724 | 0.180426806 | -2.470514395 | 0.00041225 | 0.002877189 | - |
| *PCMP-H41* | 24.66461146 | 4.426555047 | 0.179469888 | -2.478186293 | 0.000413734 | 0.002885247 | - |
| *OXI1* | 16.4776881 | 0.882933972 | 0.053583608 | -4.222064475 | 0.000415253 | 0.002895053 | - |
| *CCL5* | 85.62475707 | 36.09321769 | 0.421527826 | -1.246300225 | 0.000415866 | 0.002898537 | K10526 |
| *MYB5* | 100.7357784 | 21.43198418 | 0.212754441 | -2.232738851 | 0.000421612 | 0.002934608 | K09422 |
| *R72* | 101.1068592 | 3.837786058 | 0.037957722 | -4.719462776 | 0.000430098 | 0.002989625 | K00430 |
| *UGT85A8* | 77.2356141 | 31.42694728 | 0.406897099 | -1.297264102 | 0.000435274 | 0.00302233 | - |
| *uncharacterized protein_05641* | 66.04706773 | 25.16131002 | 0.380960289 | -1.392287474 | 0.000435659 | 0.00302419 | - |
| *At2g04570* | 12.50510867 | 0.590144882 | 0.047192303 | -4.405304602 | 0.000455429 | 0.003148662 | - |
| *FIM5* | 21.28827515 | 3.272348418 | 0.153715996 | -2.701660792 | 0.000457711 | 0.003161888 | K17275 |
| *LTL1* | 610.9740733 | 5.624702001 | 0.009206122 | -6.763190686 | 0.000459725 | 0.003174094 | - |
| *nep2* | 9.209958762 | 0 | 0 | -Inf | 0.000464917 | 0.003205676 | - |
| *TPX2* | 9.209922798 | 0 | 0 | -Inf | 0.000464923 | 0.003205676 | - |
| *uncharacterized protein_43345* | 11.02771652 | 0.300107576 | 0.027213936 | -5.199510537 | 0.000465455 | 0.003208482 | - |
| *ALMT12* | 9.201308902 | 0 | 0 | -Inf | 0.000466369 | 0.003213916 | - |
| *ag4* | 9.180563625 | 0 | 0 | -Inf | 0.000469873 | 0.003236328 | K04120 |
| *XTH5* | 9.170190986 | 0 | 0 | -Inf | 0.000471637 | 0.003245871 | K08235 |
| *VIT_06s0061g00120* | 9.170190986 | 0 | 0 | -Inf | 0.000471637 | 0.003245871 | - |
| *SWEET3B* | 9.158095568 | 0 | 0 | -Inf | 0.000473706 | 0.003259231 | K15382 |
| *CRK2* | 63.10455867 | 11.84227126 | 0.187661106 | -2.413798424 | 0.000474144 | 0.003261372 | - |
| *GH5FP* | 25.54077197 | 0.600215152 | 0.023500274 | -5.411178581 | 0.000490353 | 0.003362963 | - |
| *FATB* | 15.85039899 | 1.478113989 | 0.093254056 | -3.42268972 | 0.00049208 | 0.003371205 | K10781 |
| *uncharacterized protein_42636* | 68.10512606 | 22.1652694 | 0.325456697 | -1.619462492 | 0.00049772 | 0.003406213 | - |
| *LBD4* | 48.94389232 | 5.600433782 | 0.114425591 | -3.127518357 | 0.000498033 | 0.003407447 | - |
| *At2g24130* | 24.92213982 | 4.72072003 | 0.189418728 | -2.400349114 | 0.000501341 | 0.003429165 | - |
| *EXL2* | 15.71390384 | 1.481773232 | 0.094296952 | -3.406645055 | 0.000504859 | 0.003452309 | - |
| *KAN4* | 19.43936351 | 2.641014996 | 0.135859129 | -2.879816584 | 0.000506738 | 0.003464235 | - |
| *BHLH49* | 13.58682645 | 0.874239594 | 0.06434465 | -3.958035988 | 0.000509314 | 0.003479996 | - |
| *SLC44A5* | 13.58338089 | 0.874239594 | 0.064360972 | -3.957670081 | 0.000509723 | 0.003481867 | - |
| *BXL5* | 286.337742 | 0.300107576 | 0.001048089 | -9.898022389 | 0.000514722 | 0.00351508 | K15920 |
| *CYP92C6* | 14.68404925 | 1.185324899 | 0.080721937 | -3.630895396 | 0.000516444 | 0.003525899 | - |
| *abkB* | 72.68072473 | 29.31108884 | 0.403285588 | -1.310126243 | 0.000522843 | 0.003564848 | K08869 |
| *R12* | 28.49896586 | 1.17663052 | 0.04128678 | -4.5981763 | 0.000539212 | 0.003661882 | K00430 |
| *PLGG1* | 42.52120558 | 13.0207461 | 0.306217708 | -1.707370382 | 0.00055375 | 0.003751684 | - |
| *nep1* | 431.2638998 | 170.1994137 | 0.394652587 | -1.341344889 | 0.000555817 | 0.003763705 | - |
| *At4g27220* | 26.0988622 | 5.31405572 | 0.203612544 | -2.296101653 | 0.000581696 | 0.003919318 | K13459 |
| *PLT1* | 35.23273097 | 9.430462332 | 0.267661974 | -1.901515899 | 0.000583432 | 0.003928953 | K09285 |
| *RPV1* | 26.03486763 | 5.31955929 | 0.204324422 | -2.291066439 | 0.000584786 | 0.003937035 | - |
| *CESA9* | 80.34581525 | 33.97416845 | 0.422849259 | -1.241784644 | 0.000585773 | 0.003942649 | - |
| *A6* | 12.19754779 | 0.586485639 | 0.048082258 | -4.378351544 | 0.000587549 | 0.003948875 | - |
| *QKY* | 11.30764101 | 0.300107576 | 0.026540246 | -5.235674437 | 0.00058762 | 0.003948875 | - |
| *uncharacterized protein_44598* | 49.15823622 | 16.57994102 | 0.337276971 | -1.567994278 | 0.000595593 | 0.003998273 | - |
| *TJ* | 200.6139295 | 81.22709458 | 0.404892595 | -1.304388836 | 0.000596152 | 0.004000977 | K08906 |
| *CYP720B1* | 12.11456668 | 0.595180017 | 0.049129286 | -4.347272903 | 0.000599001 | 0.004019051 | - |
| *At1g02150* | 84.84543183 | 36.62662961 | 0.431686525 | -1.211944036 | 0.000603449 | 0.004044668 | - |
| *At5g48740* | 59.89238988 | 3.571080101 | 0.059624939 | -4.067940301 | 0.000608817 | 0.004077457 | - |
| *RAP2-4* | 17.16017807 | 1.491843502 | 0.086936365 | -3.523896416 | 0.000613008 | 0.004103386 | K09286 |
| *PCMP-H8* | 66.97501951 | 26.31507763 | 0.392908846 | -1.347733446 | 0.000614197 | 0.004110273 | - |
| *At5g13980* | 19.0315218 | 2.670757373 | 0.140333359 | -2.833070103 | 0.000622616 | 0.004161752 | K01191 |
| *BZIP19* | 35.61106965 | 5.612348381 | 0.15760123 | -2.6656493 | 0.000623263 | 0.004164433 | - |
| *CRK2* | 10.64410161 | 0.300107576 | 0.02819473 | -5.148430638 | 0.000624155 | 0.004169307 | - |
| *KIN7K* | 39.27784694 | 11.54034877 | 0.293813171 | -1.767029024 | 0.000625889 | 0.004177631 | K11498 |
| *At5g07050* | 8.873002744 | 0 | 0 | -Inf | 0.000631446 | 0.004212538 | - |
| *OMT1* | 89.56094235 | 39.64234234 | 0.442629804 | -1.175827499 | 0.000633126 | 0.004222649 | K13066 |
| *MYB61* | 15.36657518 | 1.485432475 | 0.096666463 | -3.370840731 | 0.000634201 | 0.004228719 | K09422 |
| *At4g01130* | 8.840162049 | 0 | 0 | -Inf | 0.000638957 | 0.004258067 | - |
| *WAV3* | 8.809044132 | 0 | 0 | -Inf | 0.000646179 | 0.004296322 | - |
| *At3g09930* | 8.809044132 | 0 | 0 | -Inf | 0.000646179 | 0.004296322 | - |
| *EXPB16* | 20.23239905 | 0 | 0 | -Inf | 0.000649529 | 0.004315711 | - |
| *PCMP-H40* | 90.04997047 | 39.90904829 | 0.4431878 | -1.174009927 | 0.00064972 | 0.004315711 | - |
| *GT11* | 8.76758954 | 0 | 0 | -Inf | 0.000655963 | 0.004353483 | - |
| *TOP2* | 28.59910993 | 6.17228245 | 0.215820788 | -2.21209426 | 0.000660103 | 0.004377572 | K03164 |
| *yhfK* | 511.9379422 | 195.1990723 | 0.381294404 | -1.391022739 | 0.000663377 | 0.004398148 | - |
| *BACOVA_02659* | 39.61308018 | 11.80567883 | 0.298024763 | -1.746495885 | 0.000678548 | 0.00449293 | K05349 |
| *CAND7* | 407.3533145 | 191.5393124 | 0.470204379 | -1.088640119 | 0.000688037 | 0.004553418 | - |
| *uncharacterized protein_14209* | 47.26273761 | 9.709960932 | 0.205446435 | -2.283165801 | 0.000709087 | 0.004680667 | - |
| *PCMP-H81* | 68.39721079 | 27.47616372 | 0.401714681 | -1.315756908 | 0.000713108 | 0.004706001 | - |
| *MES13* | 17.94639442 | 2.364707204 | 0.13176503 | -2.923960557 | 0.000715407 | 0.004717538 | - |
| *CRK8* | 60.43318039 | 4.75412165 | 0.078667408 | -3.668090143 | 0.000715622 | 0.004717745 | - |
| *GGP5* | 43.70985066 | 0 | 0 | -Inf | 0.000717276 | 0.004727434 | - |
| *PCMP-H40* | 165.2566838 | 67.97444944 | 0.411326476 | -1.281644157 | 0.000722006 | 0.004754946 | - |
| *LTPG15* | 11.81737844 | 0.291413198 | 0.024659716 | -5.341699995 | 0.00072912 | 0.004798112 | - |
| *MLP423* | 42.98754986 | 0 | 0 | -Inf | 0.000734378 | 0.00483008 | - |
| *RPS2* | 47.88309255 | 15.36349785 | 0.320854336 | -1.640009616 | 0.000735259 | 0.004832317 | K13459 |
| *SULTR3;1* | 86.56959118 | 2.641014996 | 0.030507421 | -5.034695948 | 0.000736762 | 0.004838482 | K17471 |
| *OFP8* | 14.23995725 | 1.165652792 | 0.081857886 | -3.610734786 | 0.000737195 | 0.004840093 | - |
| *At1g67720* | 15.46168767 | 0 | 0 | -Inf | 0.000737846 | 0.004843129 | - |
| *LAC17* | 84.97130969 | 10.08741045 | 0.118715487 | -3.074419935 | 0.000740252 | 0.004856441 | K05909 |
| *RPS13* | 38.58505626 | 3.842821193 | 0.09959351 | -3.327804455 | 0.000740745 | 0.004858435 | K02952 |
| *GGP3* | 40.94701413 | 9.768538228 | 0.238565337 | -2.067543656 | 0.000744107 | 0.004876748 | - |
| *sigA* | 3401.977466 | 1543.358032 | 0.453664978 | -1.140300804 | 0.000754446 | 0.004940725 | - |
| *SLC15A2* | 12.06618501 | 0.586485639 | 0.048605722 | -4.362730016 | 0.000760966 | 0.004979619 | K14638 |
| *At5g18840* | 39.4575914 | 11.84273969 | 0.300138434 | -1.736300021 | 0.000762582 | 0.004988918 | K08145 |
| *CYP76T24* | 79.84658722 | 25.02686406 | 0.313436866 | -1.673753217 | 0.000766271 | 0.005009232 | - |
| *ASPG2* | 24.74238827 | 2.938839222 | 0.118777508 | -3.073666424 | 0.000768095 | 0.005019877 | - |
| *uncharacterized protein_01927* | 59.24600634 | 22.46081027 | 0.379110959 | -1.399307934 | 0.000771272 | 0.005035517 | - |
| *pitC* | 53.38298875 | 19.19762467 | 0.359620642 | -1.475452263 | 0.000772675 | 0.005043394 | - |
| *PCMP-H32* | 53.16875275 | 19.15140099 | 0.360200306 | -1.473128689 | 0.000777197 | 0.005066392 | - |
| *PCMP-H12* | 113.9352207 | 54.95870906 | 0.48236804 | -1.051793774 | 0.0007858 | 0.00511736 | - |
| *At3g47570* | 24.81841343 | 2.0581886 | 0.082929902 | -3.591963798 | 0.000786547 | 0.005120927 | - |
| *TPX2* | 11.72406065 | 0.595180017 | 0.050765689 | -4.300002437 | 0.000788341 | 0.005130007 | - |
| *TMEM53* | 118.4504213 | 57.91765941 | 0.488961194 | -1.032208124 | 0.000788776 | 0.005131538 | - |
| *B&apos;GAMMA* | 52.30992083 | 11.59298347 | 0.221621124 | -2.173832693 | 0.000791465 | 0.005146427 | K11584 |
| *At5g24080* | 42.58860975 | 13.6154871 | 0.319697853 | -1.64521904 | 0.000809401 | 0.005251091 | - |
| *PCMP-H85* | 103.8941814 | 49.04440877 | 0.472061169 | -1.08295428 | 0.000822981 | 0.005326668 | - |
| *RAV1* | 24.63704699 | 5.052853333 | 0.205091679 | -2.285659135 | 0.000825012 | 0.005335796 | K09287 |
| *WSD5* | 10.31062712 | 0.295072441 | 0.028618283 | -5.126919091 | 0.000825498 | 0.005336658 | - |
| *OPT4* | 10.31234989 | 0.291413198 | 0.028258661 | -5.145163091 | 0.000825975 | 0.005338394 | - |
| *PCMP-H42* | 39.68389395 | 12.07010144 | 0.304156176 | -1.717115795 | 0.00082837 | 0.005351186 | - |
| *FBX6* | 71.98279459 | 8.8682155 | 0.1231991 | -3.02093638 | 0.000832281 | 0.005369697 | - |
| *ag1* | 34.90787037 | 9.747490229 | 0.279234744 | -1.840449636 | 0.000839109 | 0.005409007 | K16086 |
| *STI* | 13.92374651 | 1.178006413 | 0.084604127 | -3.563128156 | 0.000843823 | 0.005434611 | - |
| *FMO1* | 32.98466344 | 8.875533985 | 0.269080629 | -1.893889559 | 0.000844394 | 0.005436925 | K00485 |
| *CYCD3-3* | 13.92550525 | 1.169312035 | 0.083969092 | -3.573997798 | 0.000845035 | 0.005439687 | K14505 |
| *PLY* | 11.65841522 | 0 | 0 | -Inf | 0.000860334 | 0.005528476 | K01728 |
| *uncharacterized protein_43899* | 703.1161574 | 231.1828727 | 0.328797554 | -1.604728529 | 0.000860834 | 0.005530306 | - |
| *abhd17c* | 43.12767039 | 11.5302785 | 0.267352222 | -1.903186429 | 0.000864821 | 0.005553143 | - |
| *SAMC1* | 85.73878417 | 38.1903409 | 0.445426667 | -1.166740161 | 0.000867057 | 0.005565563 | K15111 |
| *At1g80120* | 17.69758785 | 2.356012826 | 0.133126212 | -2.909133439 | 0.000867216 | 0.005565563 | - |
| *ALF1* | 62.91795197 | 20.0814661 | 0.3191691 | -1.64760711 | 0.000867996 | 0.005567964 | K01623 |
| *XTH5* | 57.94494906 | 7.755197411 | 0.133837332 | -2.901447499 | 0.000869195 | 0.005572869 | K08235 |
| *At4g01130* | 8.492833392 | 0 | 0 | -Inf | 0.000871458 | 0.00558459 | - |
| *CRJ32* | 8.485906311 | 0 | 0 | -Inf | 0.000873636 | 0.005597152 | - |
| *PIP5K1* | 165.5451072 | 37.29596076 | 0.225291834 | -2.150133075 | 0.000880233 | 0.005635203 | K00889 |
| *TPSD2* | 8.456547138 | 0 | 0 | -Inf | 0.000882946 | 0.005648613 | K12742 |
| *FPP7* | 72.07069938 | 30.18948553 | 0.41888709 | -1.255366674 | 0.000885932 | 0.005661812 | - |
| *APCB1* | 122.5162466 | 60.99241787 | 0.497831264 | -1.006271261 | 0.000886239 | 0.005662363 | - |
| *ACA7* | 12.40489977 | 0.291413198 | 0.023491782 | -5.411700049 | 0.000886615 | 0.005663357 | K01674 |
| *SLC15A2* | 27.17361694 | 6.223541261 | 0.229028814 | -2.12639898 | 0.000888315 | 0.005671397 | K14638 |
| *TPS-Lon* | 17.01509789 | 0.300107576 | 0.017637723 | -5.825191901 | 0.000889056 | 0.005674718 | K18108 |
| *DOT4* | 121.0215188 | 60.05550272 | 0.496238217 | -1.010895248 | 0.000892194 | 0.005687679 | - |
| *PCMP-H85* | 110.1304053 | 53.19008933 | 0.4829737 | -1.049983465 | 0.000897777 | 0.005721856 | - |
| *PUX10* | 29.78451814 | 7.387818161 | 0.248042225 | -2.011342357 | 0.000898868 | 0.005727388 | - |
| *At5g09300* | 157.7130008 | 54.42895639 | 0.345113948 | -1.534855312 | 0.00090327 | 0.005751154 | K00166 |
| *CAO* | 897.2985133 | 423.2375836 | 0.4716798 | -1.084120276 | 0.000906431 | 0.005766995 | K12271 |
| *AAO* | 67.09073342 | 15.64896846 | 0.233250818 | -2.100045954 | 0.000910018 | 0.005788381 | K00423 |
| *CYSEP* | 15.78127204 | 1.752138431 | 0.111026439 | -3.17102483 | 0.000912099 | 0.005800184 | K16292 |
| *AAP2* | 35.25868055 | 10.0279257 | 0.284410124 | -1.813955275 | 0.000916034 | 0.005819446 | - |
| *At1g80640* | 50.01185675 | 17.78127878 | 0.355541264 | -1.491911085 | 0.0009185 | 0.005832706 | - |
| *ZFNL* | 140.1293752 | 67.84312631 | 0.484146355 | -1.046484861 | 0.000927693 | 0.005883328 | - |
| *At1g80150* | 120.5810451 | 59.66982725 | 0.494852464 | -1.014929633 | 0.000929796 | 0.005892302 | - |
| *CYP92C6* | 29.07439177 | 7.101440098 | 0.244250685 | -2.033565488 | 0.000932097 | 0.005905428 | - |
| *At3g47570* | 11.39927198 | 0.291413198 | 0.025564194 | -5.28973166 | 0.000934497 | 0.005919174 | - |
| *ARL8B* | 71.59032113 | 30.14326185 | 0.421052195 | -1.247929009 | 0.000964941 | 0.006093976 | K07955 |
| *At1g11050* | 169.1012741 | 39.70554516 | 0.234803347 | -2.090475121 | 0.000965804 | 0.006097926 | - |
| *ELI* | 1784.891991 | 664.1331825 | 0.372085922 | -1.426292288 | 0.000972341 | 0.00613317 | K00616 |
| *UGT85A1* | 572.0531455 | 270.1113391 | 0.47217875 | -1.082594979 | 0.000974492 | 0.006142214 | - |
| *WRKY28* | 44.94550008 | 15.12928606 | 0.336614033 | -1.570832772 | 0.000975835 | 0.006149171 | - |
| *TAR4* | 19.92839162 | 3.286077931 | 0.164894287 | -2.600386678 | 0.000977543 | 0.006155403 | - |
| *uncharacterized protein_31437* | 14.34878004 | 0 | 0 | -Inf | 0.000992265 | 0.006243517 | - |
| *TOR1* | 284.0456619 | 117.7413899 | 0.414515712 | -1.270501306 | 0.000997427 | 0.006274463 | - |
| *CALS2* | 102.6707642 | 49.0416864 | 0.4776597 | -1.065944933 | 0.001000576 | 0.006292726 | K11000 |
| *CYTB5-B* | 919.5080817 | 456.6731712 | 0.496649437 | -1.009700218 | 0.001001609 | 0.006297681 | - |
| *ZAT9* | 16.49836145 | 2.075577356 | 0.12580506 | -2.990738142 | 0.001005428 | 0.006318601 | - |
| *At5g48740* | 11.40960865 | 0.590144882 | 0.051723499 | -4.273036316 | 0.001018035 | 0.006389115 | - |
| *MDARS* | 103.3101416 | 49.37701051 | 0.477949306 | -1.065070488 | 0.001020418 | 0.006400393 | K08232 |
| *FRI3* | 11.38541782 | 0.591520774 | 0.051954244 | -4.266614589 | 0.00102401 | 0.006419676 | K00522 |
| *uncharacterized protein_11137* | 11.37159962 | 0.600215152 | 0.052781946 | -4.24381166 | 0.001025357 | 0.00642499 | - |
| *dnaJ* | 29.632482 | 7.345722163 | 0.247894259 | -2.012203236 | 0.001030442 | 0.0064537 | - |
| *At5g48740* | 11.3542999 | 0.595180017 | 0.052418909 | -4.253768854 | 0.001031259 | 0.006457243 | - |
| *PUB9* | 102.4341898 | 48.73882704 | 0.475806243 | -1.071553892 | 0.00104613 | 0.00653222 | - |
| *PAP4* | 26.61721353 | 2.356012826 | 0.088514631 | -3.49794025 | 0.001054161 | 0.006576601 | K14379 |
| *At1g03100* | 86.96895576 | 39.4762417 | 0.453911874 | -1.139515866 | 0.001058901 | 0.006602967 | - |
| *AMC9* | 13.61274007 | 1.185324899 | 0.087074674 | -3.521603029 | 0.001059617 | 0.006604224 | - |
| *ROPGEF14* | 38.98579107 | 12.08977354 | 0.310107175 | -1.689161188 | 0.001061205 | 0.006610917 | - |
| *WNK11* | 36.54774322 | 7.61699482 | 0.20841218 | -2.262488504 | 0.001068333 | 0.006650482 | K08867 |
[truncated: 77,977 more chars]
