# Supplementary material for: Response mechanism of carbon metabolism of Pinus massoniana to gradient high temperature and drought stress
Source: BMC Genomics. 2024 Feb 12;25:166. doi: 10.1186/s12864-024-10054-2 (PMC10860282; doi:10.1186/s12864-024-10054-2)
Supplement: Supplementary file 4 — Additional file 4. [file 12864_2024_10054_MOESM4_ESM.docx]

Table S5 A total of 6632 differentially expressed genes were identified in the T35CK vs T35Z group

| **gene names** | **baseMean_T35CK** | **baseMean_T35Z** | **foldChange(T35Z/T35CK)** | **log2FoldChange** | **pval** | **padj** | **KEGG** |
| --- | --- | --- | --- | --- | --- | --- | --- |
| *SWEET3B* | 13.38727631 | 16742.50433 | 1250.62813 | 10.28843716 | 9.2296E-207 | 2.4325E-202 | K15382 |
| *BACOVA_02659* | 121.1886297 | 12545.80503 | 103.5229548 | 6.693806891 | 3.0777E-130 | 2.7038E-126 | K05349 |
| *JA2L* | 47.09744828 | 21712.79883 | 461.0185821 | 8.848681092 | 2.4744E-124 | 1.6304E-120 | - |
| *MYB2* | 11.4523382 | 2244.887896 | 196.020049 | 7.614857411 | 4.3464E-124 | 2.2911E-120 | K09422 |
| *PCBER* | 209.1454643 | 19242.5804 | 92.00572655 | 6.523651754 | 1.0894E-120 | 4.7854E-117 | - |
| *CA2* | 8.03043655 | 1670.716148 | 208.0479856 | 7.70077251 | 1.7838E-115 | 6.7162E-112 | K15746 |
| *P5CS* | 78.60318701 | 6912.141832 | 87.93717017 | 6.458401202 | 7.131E-114 | 2.3493E-110 | K12657 |
| *At2g39510* | 0.314161193 | 922.3074386 | 2935.77774 | 11.51952703 | 1.4301E-110 | 4.1879E-107 | - |
| *CHI4* | 0.628322387 | 1102.505362 | 1754.681013 | 10.77699307 | 6.717E-110 | 1.7703E-106 | K01183 |
| *At5g53970* | 115.8322858 | 5732.123558 | 49.48640629 | 5.628960372 | 2.84422E-99 | 5.76633E-96 | K00815 |
| *NAC068* | 40.6331327 | 2075.37055 | 51.07581946 | 5.674568541 | 2.41244E-89 | 4.23881E-86 | - |
| *ATL78* | 1.977931614 | 2692.536971 | 1361.289213 | 10.41075789 | 5.12301E-84 | 6.75111E-81 | K19040 |
| *uncharacterized protein_32472* | 1.048258864 | 888.857361 | 847.9368898 | 9.727813082 | 1.27971E-74 | 9.63656E-72 | - |
| *PNC1* | 4.191515118 | 2100.703619 | 501.1800173 | 8.969185083 | 1.44859E-67 | 8.2998E-65 | K00430 |
| *PCK* | 290.1027582 | 10596.6643 | 36.52727869 | 5.190902371 | 3.18921E-67 | 1.7884E-64 | K01610 |
| *MO2* | 27.86288829 | 877.0023068 | 31.47564235 | 4.976163916 | 8.02162E-64 | 3.70909E-61 | - |
| *IRL1* | 5.566746006 | 441.2278205 | 79.26135305 | 6.30854569 | 3.00193E-63 | 1.341E-60 | - |
| *OBAP1A* | 7.320316222 | 470.6222103 | 64.28987438 | 6.006519627 | 3.25848E-62 | 1.40788E-59 | - |
| *MO2* | 95.88264433 | 2734.211125 | 28.51622569 | 4.833711139 | 8.15001E-61 | 3.30464E-58 | - |
| *MO2* | 35.50053036 | 947.8471648 | 26.69952125 | 4.738741968 | 1.68091E-59 | 6.61224E-57 | - |
| *HPPD* | 1695.516074 | 24015.17371 | 14.1639316 | 3.824149877 | 8.5239E-59 | 3.16417E-56 | K00457 |
| *PLA2-ALPHA* | 0 | 304.1109927 | Inf | Inf | 6.32995E-58 | 2.19742E-55 | K01047 |
| *MYB2* | 1.283910754 | 1402.10447 | 1092.057579 | 10.09283321 | 5.90093E-55 | 1.76733E-52 | K09422 |
| *CAT3* | 0.734097671 | 250.4164797 | 341.1214743 | 8.414141768 | 1.42797E-54 | 4.13578E-52 | K03781 |
| *iolG* | 5.36800627 | 346.3776213 | 64.52630715 | 6.011815557 | 5.72407E-54 | 1.58804E-51 | - |
| *Os01g0656200* | 26.78711549 | 642.300512 | 23.97796479 | 4.583637305 | 3.7235E-53 | 1.01172E-50 | K14497 |
| *NFD4* | 155.0446537 | 2188.382387 | 14.11452981 | 3.819109165 | 1.04874E-52 | 2.79198E-50 | - |
| *OPR2* | 247.9357555 | 9687.432476 | 39.07234943 | 5.288076103 | 7.23383E-52 | 1.90655E-49 | K05894 |
| *pgk* | 47.81392615 | 884.5837654 | 18.50054653 | 4.209495985 | 1.11258E-51 | 2.90329E-49 | K00927 |
| *Os02g0190300* | 4.310101232 | 492.080123 | 114.1690407 | 6.835027677 | 1.19924E-50 | 2.98182E-48 | K05658 |
| *mpaB&apos;* | 0 | 202.0138491 | Inf | Inf | 4.54487E-50 | 1.09894E-47 | - |
| *OAT* | 11.2924029 | 399.1210049 | 35.34420517 | 5.143401794 | 6.64248E-50 | 1.5772E-47 | K00819 |
| *WRKY71* | 28.13836903 | 599.905433 | 21.31983671 | 4.414124483 | 1.13911E-49 | 2.63354E-47 | - |
| *uncharacterized protein_45269* | 12.36476309 | 413.0807956 | 33.40790218 | 5.062117488 | 1.4165E-49 | 3.21838E-47 | - |
| *TOGT1* | 34.29664925 | 1623.527671 | 47.33779265 | 5.56492053 | 1.92609E-49 | 4.30204E-47 | - |
| *Dctpp1* | 18.76161189 | 465.0088383 | 24.78512193 | 4.631402451 | 4.11999E-48 | 8.75697E-46 | K16904 |
| *MYB2* | 2.332169618 | 372.4281738 | 159.6917183 | 7.319145686 | 2.75414E-47 | 5.4991E-45 | K09422 |
| *OPR11* | 465.9281763 | 4912.597697 | 10.54368022 | 3.398306616 | 6.05928E-47 | 1.20074E-44 | K05894 |
| *Os01g0656200* | 323.9790946 | 3494.333959 | 10.78567728 | 3.431044867 | 7.63346E-47 | 1.5014E-44 | K14497 |
| *DMR6* | 79.87757557 | 1049.279944 | 13.13610155 | 3.71546528 | 1.3535E-45 | 2.52999E-43 | - |
| *PARP1* | 233.7452422 | 2485.848625 | 10.63486299 | 3.410729541 | 3.44692E-45 | 6.30882E-43 | K10798 |
| *HIPP20* | 0.301350363 | 250.3912363 | 830.8974114 | 9.698526552 | 5.50798E-44 | 9.67788E-42 | - |
| *HEL* | 17.18460059 | 436.7920639 | 25.41764422 | 4.667758418 | 2.06309E-43 | 3.60098E-41 | - |
| *TKL-1* | 70.83895696 | 890.4171948 | 12.56959776 | 3.651866578 | 6.38397E-43 | 1.09257E-40 | K00615 |
| *GAD* | 1833.012861 | 16172.1815 | 8.822732149 | 3.141225487 | 1.0583E-42 | 1.78798E-40 | K01580 |
| *NAAT1* | 9.352779797 | 297.8741146 | 31.84872531 | 4.993163727 | 1.47234E-42 | 2.45601E-40 | K00815 |
| *TPPD* | 0.969749561 | 177.1185527 | 182.6436018 | 7.512887405 | 9.44883E-42 | 1.53724E-39 | K01087 |
| *uncharacterized protein_24004* | 0.314161193 | 161.5124348 | 514.1068922 | 9.005924543 | 2.38844E-41 | 3.81513E-39 | - |
| *BAM1* | 1688.848499 | 14025.50941 | 8.304776546 | 3.053941351 | 9.62902E-41 | 1.50167E-38 | K01177 |
| *uncharacterized protein_15056* | 53.08236247 | 680.2125171 | 12.81428492 | 3.679681069 | 2.0389E-40 | 3.16102E-38 | - |
| *LOX1.1* | 0.681210029 | 1216.610119 | 1785.954503 | 10.80247961 | 3.68213E-40 | 5.51399E-38 | K15718 |
| *HSL1* | 141.0052017 | 1392.546386 | 9.875851172 | 3.303905095 | 7.31638E-40 | 1.08332E-37 | - |
| *RFS2* | 1538.357405 | 36910.6659 | 23.99355688 | 4.584575138 | 9.78451E-40 | 1.43734E-37 | K06617 |
| *SRK2A* | 476.1075505 | 4008.942081 | 8.420244705 | 3.073862161 | 1.29756E-39 | 1.87904E-37 | K14498 |
| *AAE3* | 66.16561968 | 778.3177009 | 11.76317406 | 3.55620549 | 3.55569E-39 | 5.03838E-37 | - |
| *GEK1* | 321.0222914 | 2768.688634 | 8.624599311 | 3.108457432 | 3.80059E-39 | 5.35659E-37 | K09716 |
| *AAE1* | 316.1218044 | 2887.188216 | 9.133151134 | 3.191112707 | 6.64542E-39 | 9.31631E-37 | - |
| *DDB_G0268948* | 22.92446467 | 3976.915341 | 173.4790931 | 7.438617996 | 1.07994E-38 | 1.49805E-36 | - |
| *GALS1* | 22.75627955 | 390.7942641 | 17.17302968 | 4.102072678 | 3.23281E-38 | 4.30322E-36 | - |
| *GIS2* | 0 | 135.6572303 | Inf | Inf | 7.40675E-38 | 9.47632E-36 | - |
| *DDB_G0268948* | 0.301350363 | 248.5923764 | 824.9280807 | 9.688124537 | 1.21761E-37 | 1.53547E-35 | - |
| *CHI4* | 0 | 384.980891 | Inf | Inf | 1.34663E-37 | 1.69008E-35 | K01183 |
| *OPR11* | 341.0430929 | 3184.167212 | 9.33655388 | 3.22289015 | 1.64957E-37 | 2.02214E-35 | K05894 |
| *pgk* | 359.5558657 | 2891.056876 | 8.040633324 | 3.00730914 | 5.05911E-37 | 6.06082E-35 | K00927 |
| *GA2OX2* | 25.30939795 | 2015.013601 | 79.61523245 | 6.314972577 | 1.31916E-36 | 1.54523E-34 | K04125 |
| *NCPR* | 7006.910472 | 50358.76532 | 7.187014237 | 2.845392544 | 1.34888E-36 | 1.57306E-34 | K00327 |
| *HIPP23* | 0 | 614.3892292 | Inf | Inf | 1.94467E-36 | 2.24797E-34 | - |
| *PHM8* | 83.06282913 | 2498.109495 | 30.07493871 | 4.910489892 | 5.12117E-36 | 5.84301E-34 | K07025 |
| *OAT* | 69.03212717 | 690.8936192 | 10.00829103 | 3.323123742 | 1.22316E-35 | 1.37767E-33 | K00819 |
| *ADCS* | 1176.955039 | 8301.627187 | 7.053478603 | 2.818334935 | 2.73475E-35 | 3.06711E-33 | K13950 |
| *XRN4* | 162.1439254 | 1357.854784 | 8.374379617 | 3.065982318 | 3.61581E-35 | 4.02103E-33 | K20553 |
| *PHOS34* | 11.69776025 | 256.8361978 | 21.95601486 | 4.456544316 | 4.07453E-35 | 4.47452E-33 | - |
| *FG2* | 27.90284112 | 2374.07373 | 85.08358414 | 6.410808903 | 5.36486E-35 | 5.86707E-33 | - |
| *uncharacterized protein_18539* | 41.20171399 | 484.0639204 | 11.74863552 | 3.554421307 | 5.46372E-35 | 5.95049E-33 | - |
| *SGPP* | 282.0876446 | 2137.798856 | 7.578491637 | 2.921910734 | 8.00418E-35 | 8.68141E-33 | - |
| *XRN3* | 181.0347658 | 1447.487882 | 7.995634848 | 2.999212587 | 9.86413E-35 | 1.06114E-32 | K20553 |
| *DHNAT1* | 10.02421967 | 414.6312303 | 41.36294334 | 5.370266945 | 1.60357E-34 | 1.71108E-32 | - |
| *PM19L* | 946.3795473 | 6481.769724 | 6.849017123 | 2.775896967 | 3.09611E-34 | 3.29037E-32 | - |
| *HCT* | 8.187579137 | 219.032656 | 26.75182155 | 4.741565224 | 4.29291E-34 | 4.54393E-32 | K13065 |
| *HMT/HLT* | 2.35779128 | 428.9728911 | 181.9384501 | 7.507306658 | 4.49343E-34 | 4.73715E-32 | K19861 |
| *At5g58730* | 170.9737579 | 1339.573594 | 7.83496608 | 2.969927029 | 1.01705E-33 | 1.0512E-31 | K19517 |
| *HEL* | 5.4097274 | 219.5889165 | 40.59149385 | 5.34310553 | 1.66618E-33 | 1.71539E-31 | - |
| *PDC4* | 362.1936988 | 4755.081426 | 13.12855923 | 3.714636694 | 6.97069E-33 | 6.95907E-31 | K01568 |
| *NAC002* | 10.09004212 | 345.6108363 | 34.25266536 | 5.09814435 | 8.44596E-33 | 8.40006E-31 | - |
| *MYB306* | 8.4889295 | 212.8626992 | 25.0753289 | 4.648196719 | 1.18558E-32 | 1.17031E-30 | K09422 |
| *MYB2* | 3.851732263 | 974.2357489 | 252.9344416 | 7.982619689 | 2.16061E-32 | 2.10908E-30 | K09422 |
| *COR413PM2* | 228.1165823 | 1642.492563 | 7.200233083 | 2.84804361 | 2.28121E-32 | 2.21858E-30 | - |
| *PHOS34* | 82.18691624 | 703.2899849 | 8.557201281 | 3.097139026 | 3.14616E-32 | 3.01528E-30 | - |
| *PLIP2* | 10.48131626 | 224.5825618 | 21.42694258 | 4.421354101 | 3.28817E-32 | 3.13996E-30 | - |
| *At1g75220* | 99.78577211 | 3861.544992 | 38.69835258 | 5.274200246 | 6.22868E-32 | 5.84211E-30 | K08145 |
| *RD19D* | 4.468764158 | 513.7133536 | 114.9564702 | 6.844943858 | 1.11648E-31 | 1.02888E-29 | K01373 |
| *uncharacterized protein_47446* | 1.218212282 | 128.361544 | 105.3687817 | 6.719303683 | 1.35365E-31 | 1.23878E-29 | - |
| *STA* | 5.069820564 | 239.6051433 | 47.26106974 | 5.56258038 | 1.49766E-31 | 1.36582E-29 | - |
| *CHIT5* | 6.930810383 | 183.0647936 | 26.41318742 | 4.723186504 | 4.9078E-31 | 4.36993E-29 | K01183 |
| *LAR* | 0.615511556 | 899.3359622 | 1461.119541 | 10.5128585 | 7.30129E-31 | 6.37194E-29 | K13081 |
| *PXG* | 71.39701986 | 619.1927434 | 8.672529254 | 3.116452802 | 7.56999E-31 | 6.56298E-29 | K17991 |
| *Acx* | 60.01342081 | 546.3099627 | 9.103129856 | 3.186362661 | 8.21381E-31 | 7.09781E-29 | K00232 |
| *SUS3* | 2752.312136 | 16394.21699 | 5.956525344 | 2.574471 | 1.00108E-30 | 8.59426E-29 | K00695 |
| *NAC068* | 408.107026 | 4647.987141 | 11.38913776 | 3.509586624 | 1.66295E-30 | 1.41383E-28 | - |
| *CLT1* | 18.95718697 | 414.9509187 | 21.88884455 | 4.452123896 | 1.77815E-30 | 1.50691E-28 | - |
| *NAC018* | 49.79362606 | 478.1132188 | 9.601895999 | 3.26331931 | 2.63761E-30 | 2.22099E-28 | - |
| *Os02g0190300* | 5.711077762 | 169.2911163 | 29.64258645 | 4.88959943 | 2.81889E-30 | 2.36607E-28 | K05658 |
| *INT1* | 1807.208889 | 10755.04414 | 5.951190373 | 2.573178269 | 3.37335E-30 | 2.82248E-28 | K08150 |
| *uncharacterized protein_47185* | 163.6548381 | 1137.175507 | 6.948621381 | 2.796726773 | 4.98427E-30 | 4.15713E-28 | - |
| *MYB2* | 0 | 448.0508834 | Inf | Inf | 9.20583E-30 | 7.48854E-28 | K09422 |
| *HEL* | 12.38874043 | 222.3096972 | 17.94449552 | 4.16546946 | 1.12003E-29 | 9.05506E-28 | - |
| *NAAT1* | 18.70872425 | 725.4474178 | 38.77588916 | 5.277087958 | 1.26407E-29 | 1.00957E-27 | K00815 |
| *EMB1187* | 660.1734397 | 3933.956342 | 5.958973969 | 2.575063945 | 1.28611E-29 | 1.02407E-27 | K00894 |
| *ATHB-13* | 40.94196078 | 399.4221612 | 9.755814171 | 3.286262278 | 1.46529E-29 | 1.16323E-27 | K09338 |
| *KAB1* | 610.5240127 | 3653.702658 | 5.98453555 | 2.581239286 | 3.50674E-29 | 2.68674E-27 | - |
| *BEH3* | 181.391392 | 1215.010839 | 6.698282788 | 2.743791285 | 4.02912E-29 | 3.06027E-27 | - |
| *ERF110* | 5.842598688 | 2865.762017 | 490.4944135 | 8.938092895 | 1.29293E-28 | 9.62611E-27 | - |
| *LRL1* | 18.59026212 | 254.0748764 | 13.66709489 | 3.772634708 | 1.42203E-28 | 1.05575E-26 | - |
| *At3g01520* | 19.60300519 | 253.5592432 | 12.93471286 | 3.693176122 | 1.46419E-28 | 1.08399E-26 | - |
| *PUMP4* | 43.19599304 | 961.0900991 | 22.24951973 | 4.47570229 | 1.76937E-28 | 1.30626E-26 | K15104 |
| *TOM1* | 273.6186556 | 1687.305679 | 6.166632443 | 2.624482858 | 1.96741E-28 | 1.44437E-26 | - |
| *MSSP2* | 633.1423899 | 4773.704698 | 7.539701611 | 2.914507429 | 2.19699E-28 | 1.60399E-26 | - |
| *DTX40* | 230.8500724 | 11118.63107 | 48.16386218 | 5.589879179 | 2.50755E-28 | 1.82063E-26 | K03327 |
| *IDH1* | 288.8978483 | 1760.001752 | 6.09212482 | 2.606945501 | 3.45346E-28 | 2.47336E-26 | K00030 |
| *uncharacterized protein_51568* | 298.034797 | 1791.25361 | 6.010216348 | 2.587416924 | 4.28626E-28 | 3.0532E-26 | - |
| *Galm* | 316.5312181 | 10031.49323 | 31.69195534 | 4.986044769 | 4.30993E-28 | 3.06179E-26 | K01785 |
| *PHY* | 44.64036323 | 413.1804544 | 9.255759239 | 3.210351338 | 5.33531E-28 | 3.75983E-26 | K12121 |
| *ANR* | 0.301350363 | 138.488083 | 459.5583748 | 8.84410432 | 5.50739E-28 | 3.87074E-26 | K08695 |
| *GOLS4* | 13.5331284 | 909.277007 | 67.1889736 | 6.070152586 | 5.61956E-28 | 3.93907E-26 | K18819 |
| *uncharacterized protein_04262* | 114.4098767 | 793.3597985 | 6.934364596 | 2.793763693 | 5.71158E-28 | 3.99295E-26 | - |
| *SPAC24B11.05* | 22.8345409 | 270.8827544 | 11.86285092 | 3.568378859 | 1.05994E-27 | 7.31301E-26 | K07025 |
| *TP53I3* | 913.7035792 | 5022.687391 | 5.497064372 | 2.458661374 | 1.45411E-27 | 9.92866E-26 | - |
| *CSE* | 121.7111768 | 823.0150871 | 6.762033767 | 2.757457221 | 1.8747E-27 | 1.27017E-25 | - |
| *NFYC2* | 54.19796412 | 2645.113038 | 48.80465679 | 5.608946907 | 2.21895E-27 | 1.48811E-25 | K08066 |
| *WRKY11* | 512.5031057 | 3380.567959 | 6.596190193 | 2.721632998 | 2.70147E-27 | 1.80711E-25 | - |
| *CDT1A* | 17.37040552 | 234.0257325 | 13.47266949 | 3.751963832 | 2.90213E-27 | 1.93521E-25 | K10727 |
| *CRK2* | 16.50643124 | 227.6563973 | 13.79198168 | 3.785757858 | 2.90766E-27 | 1.93521E-25 | - |
| *At5g15710* | 35.0675351 | 403.6737417 | 11.51132352 | 3.524981813 | 3.28842E-27 | 2.18311E-25 | - |
| *GLYI4* | 11.89333532 | 203.5798038 | 17.11713311 | 4.097369184 | 4.1792E-27 | 2.73998E-25 | K14611 |
| *uncharacterized protein_00744* | 12.32772695 | 274.8323399 | 22.29383738 | 4.47857306 | 4.45058E-27 | 2.91066E-25 | - |
| *AMC4* | 394.0663341 | 2223.610012 | 5.642730218 | 2.496393375 | 4.76903E-27 | 3.10352E-25 | - |
| *MSL6* | 324.7475315 | 1869.702998 | 5.757404806 | 2.525418652 | 5.13733E-27 | 3.33496E-25 | - |
| *CFAT* | 6.353855299 | 154.550132 | 24.32383564 | 4.604298841 | 5.60172E-27 | 3.6186E-25 | - |
| *LAR* | 261.3374544 | 12654.22377 | 48.42101108 | 5.597561299 | 6.91287E-27 | 4.4438E-25 | K13081 |
| *HSR201* | 2.502123036 | 1043.033207 | 416.85928 | 8.703416642 | 9.96531E-27 | 6.3441E-25 | K19861 |
| *Pdcd2l* | 1028.306858 | 5424.10749 | 5.274794627 | 2.399114924 | 1.50091E-26 | 9.46362E-25 | K14801 |
| *NCPR* | 39.96777418 | 360.7863421 | 9.026931058 | 3.174235589 | 1.63329E-26 | 1.02493E-24 | K00327 |
| *LOG3* | 14.77531803 | 208.0883156 | 14.08350841 | 3.81593487 | 3.29668E-26 | 2.02535E-24 | K06966 |
| *AMY1.6* | 133.0953957 | 849.8155442 | 6.385010839 | 2.674689069 | 5.14088E-26 | 3.10053E-24 | K01176 |
| *SD25* | 2.030819256 | 176.6148806 | 86.96730648 | 6.442401247 | 7.01378E-26 | 4.18224E-24 | - |
| *NFD4* | 89.68403934 | 624.7912852 | 6.966582793 | 2.800451167 | 7.12266E-26 | 4.23758E-24 | - |
| *NPF6.4* | 747.5921237 | 3887.77404 | 5.200394595 | 2.378621096 | 1.02723E-25 | 6.05673E-24 | K14638 |
| *PHYPADRAFT_128349* | 38.24146995 | 407.7590425 | 10.662745 | 3.414506986 | 1.57507E-25 | 9.17593E-24 | K08967 |
| *At1g73050* | 3.090368612 | 4871.973608 | 1576.502424 | 10.62251167 | 1.72887E-25 | 9.99401E-24 | - |
| *yihQ* | 0.904051088 | 94.20086587 | 104.198609 | 6.703192208 | 3.48202E-25 | 1.97359E-23 | - |
| *EPSPS* | 2238.450962 | 11088.1969 | 4.953513429 | 2.308452163 | 3.96846E-25 | 2.24448E-23 | K00800 |
| *ERF4* | 474.0550463 | 2468.258446 | 5.206691639 | 2.380366967 | 4.52147E-25 | 2.55178E-23 | K09286 |
| *At1g73050* | 0 | 78.52035194 | Inf | Inf | 8.03674E-25 | 4.49716E-23 | K15403 |
| *uncharacterized protein_48557* | 425.7134597 | 2195.119296 | 5.1563305 | 2.366344738 | 8.74644E-25 | 4.85308E-23 | - |
| *MSL2* | 1335.235083 | 6479.721642 | 4.852869525 | 2.278838072 | 9.35223E-25 | 5.15664E-23 | - |
| *LBD11* | 3.353286482 | 118.0085497 | 35.19190809 | 5.137171833 | 1.01111E-24 | 5.55183E-23 | - |
| *ARF19* | 213.0172372 | 1178.609813 | 5.532931649 | 2.468044102 | 1.13162E-24 | 6.20062E-23 | - |
| *PUB45* | 41.44713604 | 339.6264656 | 8.194208286 | 3.034604565 | 2.08258E-24 | 1.12246E-22 | - |
| *MAMYB* | 220.2091971 | 1240.653582 | 5.633977135 | 2.494153709 | 2.83521E-24 | 1.51265E-22 | - |
| *uncharacterized protein_05393* | 4.97850043 | 1011.943995 | 203.2628116 | 7.667202478 | 3.59094E-24 | 1.90428E-22 | - |
| *PTEN1* | 207.3494291 | 1130.366379 | 5.451504659 | 2.44665448 | 5.10405E-24 | 2.67973E-22 | K01110 |
| *TPS5* | 63.33742505 | 1124.165955 | 17.74884207 | 4.149653002 | 5.18034E-24 | 2.71437E-22 | K16055 |
| *TUR2* | 13.61176169 | 503.5407191 | 36.99306017 | 5.209182744 | 6.45337E-24 | 3.335E-22 | - |
| *PR4B* | 6.092581747 | 198.6005414 | 32.59710738 | 5.026672042 | 6.54041E-24 | 3.37337E-22 | - |
| *STY13* | 411.0513228 | 2078.546131 | 5.056658418 | 2.338184326 | 7.62433E-24 | 3.90947E-22 | - |
| *ATL77* | 0.301350363 | 123.1458174 | 408.646654 | 8.674710111 | 1.27028E-23 | 6.46323E-22 | K19040 |
| *MED26B* | 140.8717885 | 797.3591959 | 5.660176566 | 2.500847058 | 1.82932E-23 | 9.20106E-22 | - |
| *PAO2* | 581.6536906 | 4194.752163 | 7.211769186 | 2.850353224 | 2.11805E-23 | 1.05927E-21 | K17839 |
| *At4g33300* | 318.4213749 | 1594.288351 | 5.006850911 | 2.323903497 | 2.13231E-23 | 1.06438E-21 | - |
| *CYP750A1* | 141.7328461 | 2400.514422 | 16.93689564 | 4.082097562 | 2.42299E-23 | 1.19813E-21 | - |
| *MYBS3* | 234.4878377 | 2164.475333 | 9.230650743 | 3.206432359 | 3.28451E-23 | 1.61551E-21 | - |
| *HXK1* | 207.3283685 | 1079.743929 | 5.207892855 | 2.380699767 | 5.4371E-23 | 2.62936E-21 | K00844 |
| *gluA* | 11.98946444 | 163.1668637 | 13.60918701 | 3.76650898 | 5.83453E-23 | 2.81124E-21 | K05349 |
| *At4g19900* | 4.755659371 | 115.8900622 | 24.36887361 | 4.606967663 | 6.7044E-23 | 3.20692E-21 | K01988 |
| *uncharacterized protein_34546* | 2.619064832 | 211.6683272 | 80.8182847 | 6.336609827 | 7.22392E-23 | 3.44916E-21 | - |
| *CYP750A1* | 24.75325555 | 395.6818027 | 15.98504091 | 3.998650531 | 9.38765E-23 | 4.46608E-21 | - |
| *MYB123* | 1.873800649 | 394.3353086 | 210.4467776 | 7.717311609 | 1.0732E-22 | 5.07816E-21 | K09422 |
| *LFS* | 564.880433 | 2648.110121 | 4.687912638 | 2.228945685 | 1.54757E-22 | 7.28353E-21 | - |
| *PXG* | 0.314161193 | 310.9325012 | 989.7228164 | 9.950880728 | 2.0638E-22 | 9.66138E-21 | K17991 |
| *RAB28* | 16.72762798 | 438.9118138 | 26.23873596 | 4.713626315 | 2.14174E-22 | 9.99075E-21 | - |
| *CYSZ* | 495.5815751 | 2326.966423 | 4.695425616 | 2.231255937 | 2.15668E-22 | 1.00426E-20 | K01647 |
| *At1g73050* | 0 | 1613.137407 | Inf | Inf | 2.23504E-22 | 1.03709E-20 | K00108 |
| *SUS4* | 1434.445298 | 6518.063263 | 4.54396084 | 2.183950402 | 2.31423E-22 | 1.07195E-20 | K00695 |
| *ELIP1* | 1.519562644 | 243.9638144 | 160.5487048 | 7.326867216 | 2.49633E-22 | 1.15224E-20 | - |
| *CIPK26* | 47.02985753 | 338.001425 | 7.186954049 | 2.845380462 | 2.54377E-22 | 1.17004E-20 | K07198 |
| *PUMP5* | 37.0874358 | 691.5909438 | 18.64758048 | 4.220916548 | 2.62003E-22 | 1.20302E-20 | K15104 |
| *HIPP20* | 0 | 200.8569715 | Inf | Inf | 2.70473E-22 | 1.2376E-20 | - |
| *At2g02240* | 16.30120999 | 212.3846878 | 13.02876829 | 3.703628796 | 2.75657E-22 | 1.25913E-20 | - |
| *HVA22* | 0.367048835 | 671.7954836 | 1830.261859 | 10.83783436 | 2.8113E-22 | 1.2797E-20 | K17279 |
| *CYP707A1* | 0 | 272.9634406 | Inf | Inf | 2.97777E-22 | 1.35081E-20 | K09843 |
| *TBL11* | 47.91793313 | 405.8691615 | 8.470089066 | 3.08237714 | 3.64386E-22 | 1.63329E-20 | - |
| *CKL12* | 2166.202092 | 9467.801418 | 4.370691661 | 2.127861604 | 4.34056E-22 | 1.92917E-20 | K02218 |
| *GT7* | 34.49411661 | 278.6714705 | 8.078811633 | 3.014143093 | 4.63157E-22 | 2.05159E-20 | K13496 |
| *GLYI4* | 68.06162937 | 454.6637511 | 6.680177295 | 2.739886393 | 7.31687E-22 | 3.21942E-20 | - |
| *Cyp1* | 4439.172453 | 19110.93564 | 4.305067182 | 2.106035752 | 7.83634E-22 | 3.44224E-20 | K01802 |
| *CHI4* | 10.46686111 | 19897.51803 | 1901.001438 | 10.89254391 | 8.25349E-22 | 3.61945E-20 | K01183 |
| *GID1C* | 367.0938139 | 1723.737797 | 4.69563292 | 2.231319631 | 9.18224E-22 | 4.01339E-20 | K14493 |
| *NCED1* | 13.52031757 | 166.2049174 | 12.29297437 | 3.619762123 | 9.39576E-22 | 4.09991E-20 | K09840 |
| *PNC1* | 91.00663054 | 1150.26006 | 12.6392995 | 3.659844603 | 1.0506E-21 | 4.56171E-20 | - |
| *Tbc1d5* | 17.38157203 | 196.2934494 | 11.29319311 | 3.497381555 | 1.25004E-21 | 5.41877E-20 | K18469 |
| *ATG13B* | 179.4564821 | 1834.175521 | 10.22072593 | 3.353425763 | 1.70419E-21 | 7.36322E-20 | K08331 |
| *At3g11320* | 421.3768646 | 1931.869868 | 4.584660504 | 2.196814906 | 2.04924E-21 | 8.79637E-20 | - |
| *MYB123* | 2.266471146 | 631.4489804 | 278.6044647 | 8.122074567 | 3.00215E-21 | 1.28034E-19 | K09422 |
| *SUD1* | 176.8597221 | 882.825079 | 4.991668361 | 2.319522087 | 3.03633E-21 | 1.29282E-19 | - |
| *GOLS4* | 2.724840115 | 261.4916076 | 95.96585359 | 6.584449255 | 3.30366E-21 | 1.39761E-19 | K18819 |
| *HMT-2* | 423.9583974 | 1911.451324 | 4.508582294 | 2.172673855 | 3.55829E-21 | 1.50292E-19 | K00547 |
| *IRL1* | 2.147885031 | 89.62596019 | 41.72754076 | 5.382927992 | 3.59181E-21 | 1.51465E-19 | - |
| *At5g63930* | 51.95215346 | 537.0665812 | 10.33771548 | 3.369845497 | 4.48542E-21 | 1.87945E-19 | - |
| *VIT_06s0061g00120* | 1.808102176 | 196.6137282 | 108.7403858 | 6.764744041 | 4.49382E-21 | 1.87999E-19 | - |
| *uncharacterized protein_50841* | 47.91932949 | 322.5057686 | 6.730181161 | 2.750645339 | 4.56195E-21 | 1.90546E-19 | - |
| *EXL2* | 357.973865 | 1634.853489 | 4.566963258 | 2.191235183 | 5.21245E-21 | 2.17029E-19 | - |
| *VIT_06s0061g00120* | 0.301350363 | 68.60180332 | 227.6479865 | 7.830660889 | 6.03337E-21 | 2.50813E-19 | - |
| *VQ22* | 42.53230705 | 301.1776423 | 7.08114991 | 2.823983659 | 6.3193E-21 | 2.62286E-19 | - |
| *TKTC* | 10.25835122 | 140.121344 | 13.65924611 | 3.771805954 | 7.28788E-21 | 3.00594E-19 | K00615 |
| *MUR4* | 105.0171441 | 573.2857679 | 5.458973131 | 2.448629596 | 7.578E-21 | 3.11585E-19 | K12448 |
| *uncharacterized protein_44618* | 151.6432886 | 1077.561462 | 7.105896159 | 2.829016606 | 1.0434E-20 | 4.25695E-19 | - |
| *IRL1* | 131.870206 | 679.9062233 | 5.155874431 | 2.366217128 | 1.07321E-20 | 4.36005E-19 | - |
| *MSL3* | 112.5301187 | 596.6686214 | 5.302301538 | 2.406618717 | 1.55562E-20 | 6.26911E-19 | - |
| *DLO1* | 437.715487 | 1911.955472 | 4.368032497 | 2.12698359 | 1.57703E-20 | 6.34567E-19 | - |
| *DMP3* | 0 | 71.77557032 | Inf | Inf | 1.65694E-20 | 6.65705E-19 | - |
| *CA2* | 13.63573903 | 158.5757417 | 11.6294204 | 3.539707291 | 1.78255E-20 | 7.12912E-19 | K15746 |
| *GIF3* | 687.1864044 | 4046.20438 | 5.88807397 | 2.557795796 | 2.03771E-20 | 8.05186E-19 | - |
| *CAF1-7* | 448.2534079 | 1951.497728 | 4.353559155 | 2.122195326 | 2.50965E-20 | 9.85758E-19 | K12581 |
| *FAH* | 909.0556156 | 3805.850754 | 4.186598365 | 2.065778522 | 2.56519E-20 | 1.00608E-18 | K01555 |
| *ZAT9* | 159.3950797 | 1168.534134 | 7.331055239 | 2.874020876 | 2.83403E-20 | 1.10657E-18 | - |
| *STA* | 1.638148759 | 82.89862509 | 50.6050654 | 5.661209896 | 3.37249E-20 | 1.30522E-18 | - |
| *uncharacterized protein_08349* | 665.1970978 | 2822.988213 | 4.243837237 | 2.085369326 | 3.85987E-20 | 1.48512E-18 | - |
| *UBP5* | 708.1982922 | 2978.829157 | 4.206207767 | 2.072520114 | 4.46571E-20 | 1.70577E-18 | K11835 |
| *DRE22* | 121.1245755 | 846.7640286 | 6.990852394 | 2.805468374 | 5.77666E-20 | 2.19697E-18 | - |
| *CHIT5B* | 45.74911143 | 297.0957535 | 6.494022379 | 2.699112356 | 7.40369E-20 | 2.80765E-18 | K01183 |
| *UGT89B1* | 183.0125734 | 1429.455835 | 7.81069742 | 2.965451373 | 9.72508E-20 | 3.64601E-18 | K13496 |
| *ABCI17* | 14.32011372 | 154.6662797 | 10.80063209 | 3.433043841 | 1.05288E-19 | 3.925E-18 | - |
| *LEA14-A* | 161.0068347 | 2702.475713 | 16.78485089 | 4.069087816 | 1.35445E-19 | 5.02786E-18 | - |
| *FUM1* | 193.5707547 | 885.6450168 | 4.575303838 | 2.193867553 | 1.6929E-19 | 6.22287E-18 | K01679 |
| *YPQ1* | 15.43407105 | 1121.521941 | 72.66533485 | 6.183195382 | 2.15536E-19 | 7.88982E-18 | - |
| *PDC4* | 1478.052895 | 5892.764223 | 3.986842583 | 1.995246643 | 2.48816E-19 | 9.07025E-18 | K01568 |
| *ANN1* | 300.1680465 | 1277.367647 | 4.255508414 | 2.089331504 | 3.04762E-19 | 1.10334E-17 | K17095 |
| *PNC1* | 0.969749561 | 110.8910675 | 114.3502117 | 6.837315226 | 3.37943E-19 | 1.22011E-17 | K00430 |
| *PNS1* | 20.51201745 | 182.8248679 | 8.913061251 | 3.15592102 | 3.40386E-19 | 1.22725E-17 | - |
| *RR23* | 0.602700725 | 403.6872665 | 669.7972134 | 9.387580564 | 4.34964E-19 | 1.56184E-17 | K14491 |
| *CRY2* | 53.29276464 | 324.9829953 | 6.098069738 | 2.608352649 | 4.59391E-19 | 1.64731E-17 | K12119 |
| *SK3* | 898.0835987 | 3605.301747 | 4.014438915 | 2.005198361 | 5.25078E-19 | 1.8752E-17 | K00891 |
| *CSA* | 66.36702815 | 4413.92843 | 66.50785116 | 6.055452754 | 7.91638E-19 | 2.79309E-17 | K00432 |
| *TH2* | 139.3328377 | 651.6167803 | 4.676692092 | 2.225488447 | 1.02282E-18 | 3.58476E-17 | - |
| *At3g15810* | 0.734097671 | 63.75464525 | 86.84763322 | 6.440414628 | 1.35968E-18 | 4.72145E-17 | - |
| *uncharacterized protein_08775* | 12.64024383 | 138.3282313 | 10.94347808 | 3.451999427 | 1.5775E-18 | 5.45625E-17 | - |
| *uncharacterized protein_11339* | 20.30844052 | 172.9206027 | 8.514715965 | 3.089958405 | 1.86659E-18 | 6.43083E-17 | - |
| *CYP707A1* | 1.205401451 | 69.42345116 | 57.59363497 | 5.847837474 | 1.88417E-18 | 6.48292E-17 | K09843 |
| *UGT72B2* | 54.9273768 | 663.6881885 | 12.08301265 | 3.5949083 | 1.92592E-18 | 6.61793E-17 | K08237 |
| *At3g15890* | 182.4595675 | 801.5651589 | 4.393111142 | 2.135242999 | 1.95979E-18 | 6.72556E-17 | - |
| *chi1* | 4.794215843 | 215.4106508 | 44.93136268 | 5.489650911 | 2.24632E-18 | 7.6689E-17 | K20547 |
| *Os12g0628600* | 12.573149 | 365.6935263 | 29.0852774 | 4.862217159 | 2.49035E-18 | 8.48005E-17 | - |
| *ncd-2* | 200.6271286 | 870.3730955 | 4.338262236 | 2.117117262 | 3.4479E-18 | 1.15841E-16 | - |
| *HGO* | 1485.582757 | 5600.674376 | 3.770018432 | 1.914571577 | 3.66559E-18 | 1.22758E-16 | K00451 |
| *Os01g0656200* | 9.525897872 | 1444.013092 | 151.5881349 | 7.244013025 | 3.92354E-18 | 1.31229E-16 | K14497 |
| *CRPK1* | 4.113005815 | 89.25690081 | 21.70113655 | 4.439698698 | 4.83024E-18 | 1.6074E-16 | - |
| *uncharacterized protein_09249* | 1947.160992 | 10927.35968 | 5.611944635 | 2.488500776 | 5.54375E-18 | 1.84019E-16 | - |
| *uncharacterized protein_27854* | 5.331218096 | 96.8168373 | 18.16035952 | 4.182720859 | 5.71938E-18 | 1.8961E-16 | - |
| *At3g20040* | 96.40329915 | 471.212153 | 4.887925591 | 2.289222323 | 6.61994E-18 | 2.18094E-16 | K00844 |
| *uncharacterized protein_03821* | 106.7659053 | 508.2764626 | 4.760662695 | 2.251162414 | 7.66879E-18 | 2.51079E-16 | - |
| *ANR* | 295.5480578 | 1186.300481 | 4.013900447 | 2.005004835 | 9.59656E-18 | 3.13028E-16 | K08695 |
| *MRF1* | 1128.916672 | 4176.796623 | 3.699827214 | 1.887457897 | 9.91385E-18 | 3.22182E-16 | - |
| *DTX54* | 86.7136532 | 426.0462326 | 4.913254336 | 2.296678922 | 1.05388E-17 | 3.42071E-16 | K03327 |
| *WRKY42* | 3.340351672 | 86.63759309 | 25.93666823 | 4.696921261 | 1.09877E-17 | 3.54891E-16 | - |
| *At1g26930* | 572.7385911 | 2205.331676 | 3.850503022 | 1.945046929 | 1.10809E-17 | 3.57028E-16 | - |
| *uncharacterized protein_07671* | 72.16942605 | 372.9963805 | 5.168343452 | 2.369701945 | 1.12621E-17 | 3.62422E-16 | - |
| *HMG1* | 48.80104755 | 297.9062682 | 6.104505602 | 2.609874457 | 1.24916E-17 | 4.015E-16 | K00021 |
| *DDB_G0289029* | 103.0858948 | 491.220862 | 4.765160773 | 2.25252489 | 1.36996E-17 | 4.39789E-16 | K19476 |
| *At4g34215* | 116.4149503 | 532.6635012 | 4.575559236 | 2.193948084 | 1.48071E-17 | 4.73036E-16 | - |
| *POT1* | 492.6862575 | 1894.073315 | 3.844380244 | 1.942751039 | 1.50057E-17 | 4.78803E-16 | K03549 |
| *WRKY6* | 0.301350363 | 55.99643528 | 185.8183769 | 7.537749377 | 1.53246E-17 | 4.88387E-16 | - |
| *MED26A* | 119.037332 | 543.587461 | 4.566529271 | 2.191098081 | 1.6314E-17 | 5.18039E-16 | - |
| *HSR201* | 4.964169261 | 856.6390609 | 172.5644344 | 7.430991345 | 1.89271E-17 | 5.99572E-16 | K19861 |
| *URGT6* | 174.0947094 | 1269.574974 | 7.29243857 | 2.866401328 | 2.09428E-17 | 6.6104E-16 | - |
| *At2g25737* | 205.0276495 | 2136.856095 | 10.42228255 | 3.381599368 | 2.28117E-17 | 7.16598E-16 | - |
| *uncharacterized protein_09037* | 569.3086963 | 3231.680536 | 5.676499511 | 2.505001547 | 2.67727E-17 | 8.40025E-16 | - |
| *HIPP23* | 67.34338321 | 1909.264904 | 28.35118779 | 4.825337274 | 3.05765E-17 | 9.55959E-16 | - |
| *AAP2* | 311.8485871 | 1337.102522 | 4.287665802 | 2.100192461 | 3.48359E-17 | 1.08398E-15 | - |
| *RLT1* | 102.4793813 | 469.9391147 | 4.585694301 | 2.197140183 | 3.53735E-17 | 1.09942E-15 | - |
| *FDH1* | 33.73566966 | 217.5718218 | 6.449310892 | 2.689145017 | 3.94131E-17 | 1.22352E-15 | K00122 |
| *ALKBH10B* | 153.8521871 | 663.1229725 | 4.310130295 | 2.107731482 | 4.12322E-17 | 1.27849E-15 | - |
| *CHI4* | 2.253660315 | 768.1795714 | 340.8586317 | 8.413029707 | 5.01797E-17 | 1.54502E-15 | K01183 |
| *TPPD* | 1.035448033 | 60.81623744 | 58.73422468 | 5.876129508 | 5.09844E-17 | 1.56796E-15 | K01087 |
| *ACOHC* | 3305.17787 | 15916.66536 | 4.815675884 | 2.267738296 | 5.12946E-17 | 1.57566E-15 | K01681 |
| *HMGR1* | 36.21052671 | 226.6928481 | 6.260412887 | 2.646257809 | 5.17628E-17 | 1.5882E-15 | K00021 |
| *MFP* | 3071.898054 | 10914.83865 | 3.553125286 | 1.82908856 | 5.34343E-17 | 1.63758E-15 | K10527 |
| *ABCG36* | 0.995371222 | 60.54407807 | 60.82562637 | 5.926607367 | 6.1947E-17 | 1.88531E-15 | - |
| *BABL* | 4.675629729 | 120.4825009 | 25.76818695 | 4.687519127 | 6.51269E-17 | 1.97524E-15 | - |
| *At5g40670* | 172.291264 | 714.3738786 | 4.146315153 | 2.051829777 | 8.02887E-17 | 2.41839E-15 | K12386 |
| *CRK6* | 22.3249286 | 675.9743891 | 30.27890486 | 4.920241121 | 8.16691E-17 | 2.45716E-15 | - |
| *SYT2* | 67.30774343 | 453.3874237 | 6.736036607 | 2.751899979 | 8.94716E-17 | 2.68578E-15 | - |
| *NPF7.3* | 2.487667886 | 72.58738891 | 29.17889052 | 4.866853123 | 9.4364E-17 | 2.82299E-15 | K14638 |
| *MUR4* | 117.2966966 | 519.8451913 | 4.431882622 | 2.147919672 | 1.11605E-16 | 3.3247E-15 | K12448 |
| *At2g34160* | 101.2165312 | 457.3587867 | 4.518617477 | 2.175881431 | 1.38028E-16 | 4.08902E-15 | - |
| *FATB1* | 1198.743522 | 4375.146247 | 3.649776758 | 1.867808223 | 1.3808E-16 | 4.08902E-15 | K10781 |
| *TOGT1* | 21.33768322 | 165.765302 | 7.768664495 | 2.957666607 | 1.55205E-16 | 4.57559E-15 | - |
| *uncharacterized protein_26866* | 57.43545721 | 295.4721163 | 5.144420027 | 2.363008439 | 1.7009E-16 | 4.9921E-15 | - |
| *ANN1* | 39.05407692 | 231.1774204 | 5.919418372 | 2.565455427 | 1.74724E-16 | 5.1167E-15 | K17095 |
| *uncharacterized protein_15372* | 6.523808717 | 96.04182534 | 14.72174147 | 3.879876436 | 2.04525E-16 | 5.97611E-15 | - |
| *ARF22* | 216.7904601 | 1064.775211 | 4.911540897 | 2.296175712 | 2.08762E-16 | 6.08641E-15 | - |
| *LTPG7* | 0.982560392 | 70.18053859 | 71.42618326 | 6.158381126 | 2.11179E-16 | 6.14329E-15 | - |
| *GOLS2* | 19.58398902 | 153.8038922 | 7.85355282 | 2.973345453 | 2.92203E-16 | 8.42592E-15 | K18819 |
| *ctdspl2b* | 492.6256159 | 1783.121905 | 3.619628878 | 1.855841785 | 3.5999E-16 | 1.03129E-14 | K17616 |
| *NHX2* | 480.0509466 | 1738.207152 | 3.620880584 | 1.856340598 | 3.70681E-16 | 1.06077E-14 | - |
| *PHOS34* | 1181.461773 | 4087.267174 | 3.459500145 | 1.790563601 | 3.83615E-16 | 1.09659E-14 | - |
| *uncharacterized protein_47260* | 185.2203235 | 741.5150924 | 4.003421861 | 2.001233648 | 4.24369E-16 | 1.20265E-14 | K19476 |
| *RBOHC* | 180.1660783 | 1050.955968 | 5.833262164 | 2.544302915 | 4.99406E-16 | 1.40623E-14 | K13447 |
| *PA1024* | 79.90028053 | 374.0358673 | 4.68128353 | 2.226904147 | 5.69899E-16 | 1.60131E-14 | - |
| *Os01g0656200* | 1036.687824 | 3548.371168 | 3.422796221 | 1.775175403 | 6.40581E-16 | 1.79227E-14 | K14497 |
| *At1g74360* | 56.51111758 | 416.2796393 | 7.366331744 | 2.880946371 | 6.44473E-16 | 1.80124E-14 | - |
| *JGB* | 0 | 161.0203175 | Inf | Inf | 7.53532E-16 | 2.09716E-14 | - |
| *TMEM205* | 192.6556611 | 749.6952723 | 3.891374218 | 1.960279725 | 9.32654E-16 | 2.58204E-14 | - |
| *BAT1* | 269.3990023 | 2081.608178 | 7.72685927 | 2.949882122 | 9.60627E-16 | 2.65669E-14 | - |
| *SDL5A* | 1461.955241 | 6813.392132 | 4.660465616 | 2.220474098 | 1.21027E-15 | 3.32964E-14 | - |
| *VIT_06s0061g00120* | 5.462615041 | 84.73186783 | 15.51122808 | 3.955241009 | 1.4136E-15 | 3.86081E-14 | - |
| *FDH1* | 34.46660267 | 214.8974716 | 6.234947891 | 2.640377503 | 1.42931E-15 | 3.89967E-14 | K00122 |
| *uncharacterized protein_07382* | 71.6826427 | 1312.84767 | 18.31472196 | 4.194931894 | 1.44247E-15 | 3.93151E-14 | - |
| *POD1* | 198.813193 | 773.495754 | 3.890565521 | 1.959979876 | 1.50534E-15 | 4.09863E-14 | - |
| *SYT5* | 179.2062511 | 770.030383 | 4.296894658 | 2.103294409 | 1.53837E-15 | 4.17994E-14 | - |
| *SUR1* | 96.94334208 | 421.5897926 | 4.348826681 | 2.120626212 | 1.54409E-15 | 4.19114E-14 | K00815 |
| *DDB_G0268948* | 3.07907812 | 69.49927641 | 22.57145603 | 4.496427581 | 1.55363E-15 | 4.2127E-14 | - |
| *NTF2* | 683.3762975 | 2366.256376 | 3.4625965 | 1.791854279 | 1.55615E-15 | 4.21519E-14 | - |
| *LCB2a* | 1227.478287 | 4155.38177 | 3.385299614 | 1.759283524 | 1.60911E-15 | 4.34971E-14 | K00654 |
| *RUS4* | 97.6151539 | 423.7645141 | 4.341175495 | 2.118085746 | 1.61961E-15 | 4.3736E-14 | - |
| *NAC025* | 143.3353833 | 579.1514032 | 4.040533398 | 2.014545758 | 1.63837E-15 | 4.41975E-14 | - |
| *B3GALT7* | 61.64688457 | 863.6507999 | 14.00964227 | 3.808348213 | 1.87635E-15 | 5.04108E-14 | - |
| *GIS2* | 0 | 44.68810585 | Inf | Inf | 1.89429E-15 | 5.07893E-14 | - |
| *LPAT4* | 114.5912446 | 480.3312206 | 4.191692152 | 2.067532766 | 1.94719E-15 | 5.21547E-14 | K13513 |
| *CSA* | 65.15666552 | 2673.796951 | 41.03643011 | 5.358833327 | 2.02965E-15 | 5.42529E-14 | K00432 |
| *MYB2* | 2.659141643 | 80.63053236 | 30.32201484 | 4.922293716 | 2.36725E-15 | 6.28312E-14 | K09422 |
| *NAAT1* | 141.0000208 | 564.3604338 | 4.002555679 | 2.000921472 | 2.42277E-15 | 6.42399E-14 | K00815 |
| *PROT1* | 11.77487319 | 115.7951881 | 9.834092158 | 3.297791875 | 2.58839E-15 | 6.84937E-14 | - |
| *pabpn1-b* | 669.0584805 | 2295.1302 | 3.430388026 | 1.778371775 | 2.64595E-15 | 6.99464E-14 | - |
| *MYC2* | 175.8773139 | 1197.343273 | 6.807832385 | 2.767195517 | 2.91521E-15 | 7.69871E-14 | K13422 |
| *BC10* | 382.5854294 | 1354.806592 | 3.541187112 | 1.824233076 | 2.93211E-15 | 7.71244E-14 | - |
| *LECRK81* | 120.5901137 | 534.6199923 | 4.433365026 | 2.148402153 | 2.95087E-15 | 7.75406E-14 | - |
| *SUR1* | 111.6856565 | 572.3704117 | 5.12483366 | 2.357505179 | 3.10679E-15 | 8.13943E-14 | K00815 |
| *At2g45590* | 343.6368798 | 1221.011923 | 3.553203964 | 1.829120506 | 3.43819E-15 | 8.92778E-14 | - |
| *At4g27520* | 173.7443799 | 676.313062 | 3.892575186 | 1.960724905 | 3.54747E-15 | 9.20246E-14 | - |
| *PILS6* | 105.2094305 | 690.4252638 | 6.562389516 | 2.714221229 | 3.58319E-15 | 9.286E-14 | - |
| *MKK9* | 34.68323839 | 205.1679447 | 5.915478318 | 2.564494828 | 3.67449E-15 | 9.4946E-14 | K20604 |
| *AFP3* | 829.1565073 | 2808.944186 | 3.387712888 | 1.76031161 | 4.19492E-15 | 1.0755E-13 | - |
| *VQ22* | 0.314161193 | 65.88731669 | 209.7245556 | 7.712351979 | 4.21408E-15 | 1.07936E-13 | - |
| *ctdspl2* | 167.9093829 | 648.4474502 | 3.861889305 | 1.949306812 | 4.97786E-15 | 1.27252E-13 | K15731 |
| *ATL60* | 45.53616451 | 694.600312 | 15.25381682 | 3.931098375 | 5.22643E-15 | 1.33347E-13 | - |
| *UGT85A8* | 24.78356221 | 206.1422357 | 8.31770001 | 3.056184653 | 5.88045E-15 | 1.48881E-13 | - |
| *OsI_12825* | 92.62374688 | 399.7829392 | 4.316203487 | 2.109762882 | 6.29255E-15 | 1.58857E-13 | K16275 |
| *XYL1* | 8.318852102 | 1507.062431 | 181.1623061 | 7.501138999 | 6.64162E-15 | 1.67189E-13 | K15925 |
| *CIPK5* | 0.628322387 | 257.6093219 | 409.9954534 | 8.679464101 | 7.0899E-15 | 1.78303E-13 | K07198 |
| *NPF7.3* | 7.07349782 | 225.6785874 | 31.90480766 | 4.995701931 | 7.36845E-15 | 1.84779E-13 | K14638 |
| *EPHX2* | 24.43261284 | 362.4763796 | 14.83575997 | 3.891006926 | 8.3895E-15 | 2.0919E-13 | - |
| *uncharacterized protein_38601* | 290.6809137 | 1032.107332 | 3.550653943 | 1.828084758 | 9.51245E-15 | 2.36355E-13 | - |
| *uncharacterized protein_32500* | 245.6567215 | 889.8890988 | 3.622490333 | 1.85698184 | 9.5148E-15 | 2.36355E-13 | - |
| *SRP* | 984.6785616 | 3223.281743 | 3.273435483 | 1.710805545 | 9.93713E-15 | 2.4649E-13 | - |
| *KPHMT1* | 166.9235339 | 634.3922817 | 3.800496352 | 1.92618785 | 1.09974E-14 | 2.70884E-13 | K00606 |
| *At5g51380* | 89.48579552 | 467.9758 | 5.229609876 | 2.386703327 | 1.18668E-14 | 2.92029E-13 | K10268 |
| *uncharacterized protein_00552* | 9.365714608 | 161.792683 | 17.27499607 | 4.110613477 | 1.2894E-14 | 3.16715E-13 | - |
| *MKK4* | 44.99283294 | 234.2121379 | 5.205543251 | 2.380048731 | 1.39731E-14 | 3.42581E-13 | K20604 |
| *At3g21360* | 5.477070191 | 78.95185275 | 14.41497918 | 3.849496848 | 1.76117E-14 | 4.27417E-13 | - |
| *At3g21360* | 231.7743163 | 970.0330385 | 4.185248194 | 2.06531318 | 1.84376E-14 | 4.47047E-13 | - |
| *FBL8* | 311.7285089 | 1083.235423 | 3.474932166 | 1.796984815 | 1.96884E-14 | 4.76498E-13 | K10268 |
| *At3g45310* | 154.2977452 | 1647.300237 | 10.67611347 | 3.41631464 | 2.04138E-14 | 4.92697E-13 | K01366 |
| *Os01g0270100* | 42.02117442 | 221.7680249 | 5.277530386 | 2.39986298 | 2.08926E-14 | 5.03333E-13 | - |
| *DODA* | 1.218212282 | 752.102494 | 617.3821306 | 9.270019916 | 2.50491E-14 | 5.99086E-13 | K15777 |
| *YPQ1* | 5.803918235 | 260.0852476 | 44.81201097 | 5.485813565 | 2.8016E-14 | 6.68225E-13 | - |
| *RING1* | 491.8547301 | 1625.943578 | 3.305739435 | 1.724973013 | 2.98688E-14 | 7.1113E-13 | K11982 |
| *uncharacterized protein_27624* | 46.77835413 | 470.1669254 | 10.05095058 | 3.329260047 | 3.1596E-14 | 7.50896E-13 | - |
| *ATL40* | 1.716658062 | 55.26835116 | 32.19531739 | 5.008778967 | 3.1971E-14 | 7.59123E-13 | K19038 |
| *MST4* | 845.9648567 | 2727.294231 | 3.223885969 | 1.688800715 | 3.31814E-14 | 7.86446E-13 | - |
| *10HGO* | 7.858962794 | 720.6511801 | 91.69800126 | 6.518818383 | 3.36077E-14 | 7.95835E-13 | K00083 |
| *AVT1C* | 0.995371222 | 50.01035629 | 50.24291959 | 5.650848396 | 3.65009E-14 | 8.63571E-13 | K15015 |
| *TSJT1* | 31.97729046 | 180.7111523 | 5.651234036 | 2.498565937 | 4.21036E-14 | 9.89903E-13 | - |
| *Rpp25l* | 992.5425138 | 3581.848385 | 3.608760668 | 1.851503467 | 4.40681E-14 | 1.03333E-12 | - |
| *RMR2* | 154.595807 | 600.4578986 | 3.884050353 | 1.957561904 | 4.83402E-14 | 1.13149E-12 | K15692 |
| *TUR2* | 49.1333527 | 1652.67472 | 33.6365143 | 5.071956304 | 4.88225E-14 | 1.14176E-12 | - |
| *Os12g0628600* | 80.47381864 | 3056.679324 | 37.98352527 | 5.247301904 | 5.06781E-14 | 1.18306E-12 | - |
| *uncharacterized protein_40296* | 683.4641768 | 2203.195144 | 3.223570773 | 1.688659658 | 5.47667E-14 | 1.27737E-12 | - |
| *SRK2A* | 4948.086781 | 15209.77639 | 3.073870177 | 1.620056235 | 5.51499E-14 | 1.28409E-12 | K14498 |
| *FGT1* | 118.6574441 | 455.6919667 | 3.840399311 | 1.941256325 | 5.79223E-14 | 1.3474E-12 | - |
| *SPAC24B11.05* | 89.35567095 | 370.4274686 | 4.145539557 | 2.051559886 | 5.81699E-14 | 1.35196E-12 | K07025 |
| *uncharacterized protein_47075* | 150.8478055 | 921.5556526 | 6.109175069 | 2.610977584 | 5.97298E-14 | 1.38577E-12 | - |
| *MSL2* | 38.30248342 | 203.1576156 | 5.304032466 | 2.407089606 | 6.23468E-14 | 1.44268E-12 | - |
| *At3g24760* | 511.488814 | 1672.049074 | 3.268984635 | 1.708842596 | 6.39709E-14 | 1.47896E-12 | - |
| *UBP25* | 55.66628344 | 257.5918432 | 4.627430237 | 2.21021124 | 6.71565E-14 | 1.54718E-12 | K11855 |
| *TIFY10B* | 0.301350363 | 43.34454371 | 143.8343837 | 7.168264784 | 6.87401E-14 | 1.5809E-12 | K13464 |
| *ZAT5* | 49.40374828 | 898.787598 | 18.19270054 | 4.185287809 | 7.36464E-14 | 1.68932E-12 | - |
| *CRPK1* | 36.8872997 | 272.8692693 | 7.397377186 | 2.887013839 | 7.45046E-14 | 1.70752E-12 | - |
| *DDI1* | 152.5775506 | 561.3040576 | 3.678811565 | 1.879239781 | 7.48202E-14 | 1.71326E-12 | K11885 |
| *ERD4* | 410.9123003 | 1346.821559 | 3.277637486 | 1.712656298 | 8.32829E-14 | 1.90208E-12 | - |
| *ALMT9* | 1.859345499 | 232.8054208 | 125.2082633 | 6.968185967 | 8.55214E-14 | 1.95152E-12 | - |
| *HAT14* | 15.91325271 | 122.9522034 | 7.726403 | 2.949796929 | 9.56736E-14 | 2.17565E-12 | K09338 |
| *CRK25* | 18.03880471 | 610.2140872 | 33.82785594 | 5.080139835 | 1.04982E-13 | 2.37912E-12 | - |
| *MSRB2* | 2505.661516 | 7596.981854 | 3.031926622 | 1.600234838 | 1.11262E-13 | 2.51279E-12 | K07305 |
| *uncharacterized protein_47608* | 92.18399391 | 382.6861881 | 4.151330094 | 2.053573653 | 1.17605E-13 | 2.65146E-12 | - |
| *GCL1* | 641.5206676 | 2043.669928 | 3.185664985 | 1.671594556 | 1.19723E-13 | 2.69233E-12 | - |
| *PCMP-E76* | 318.0851569 | 1056.688872 | 3.322031377 | 1.7320657 | 1.22829E-13 | 2.75748E-12 | - |
| *BSK2* | 443.4805007 | 1445.073001 | 3.258481486 | 1.704199798 | 1.29244E-13 | 2.89165E-12 | K14500 |
| *FDH1* | 552.1975393 | 1812.937769 | 3.283132646 | 1.715073039 | 1.31189E-13 | 2.93121E-12 | K00122 |
| *uncharacterized protein_11911* | 292.0532672 | 971.8542987 | 3.327661107 | 1.734508515 | 1.31831E-13 | 2.94204E-12 | - |
| *MPK* | 180.6363859 | 646.3966927 | 3.57844124 | 1.83933129 | 1.39943E-13 | 3.12041E-12 | K14512 |
| *MAPKKK18* | 0 | 38.19764565 | Inf | Inf | 1.65265E-13 | 3.67261E-12 | K04373 |
| *VSR3* | 409.4042153 | 1331.795708 | 3.253009272 | 1.701774934 | 1.78742E-13 | 3.95107E-12 | - |
| *rihA* | 108.75854 | 420.3761836 | 3.865224592 | 1.950552246 | 1.78844E-13 | 3.95107E-12 | - |
| *OTU5* | 1166.436567 | 3568.737993 | 3.059521703 | 1.613306133 | 1.84213E-13 | 4.06625E-12 | K18342 |
| *STY13* | 35.65767295 | 205.2568489 | 5.756316437 | 2.525145902 | 2.00161E-13 | 4.40353E-12 | - |
| *uncharacterized protein_08456* | 1.205401451 | 64.853205 | 53.80216271 | 5.749592262 | 2.00489E-13 | 4.40708E-12 | - |
| *FDM1* | 17.30622738 | 121.9404096 | 7.046042266 | 2.816813129 | 2.05045E-13 | 4.50348E-12 | - |
| *MYB5* | 194.6782305 | 706.7896434 | 3.630553049 | 1.860189334 | 2.12691E-13 | 4.66363E-12 | K09422 |
| *STK38L* | 432.8902163 | 1388.788614 | 3.208177413 | 1.681753926 | 2.2751E-13 | 4.96789E-12 | K08790 |
| *EIL1* | 3503.382951 | 10499.70814 | 2.997019819 | 1.583528624 | 2.30971E-13 | 5.03096E-12 | K14514 |
| *SDH* | 19.68988828 | 219.8800639 | 11.1671565 | 3.481189973 | 2.3386E-13 | 5.0855E-12 | K00008 |
| *At3g01520* | 6.459506602 | 102.1231892 | 15.80975072 | 3.982742715 | 2.35417E-13 | 5.11512E-12 | - |
| *ARGAH1* | 87.76672104 | 521.9863945 | 5.947429599 | 2.572266289 | 2.40473E-13 | 5.22068E-12 | K01476 |
| *VEP1* | 58.31909577 | 259.877469 | 4.456129945 | 2.155791304 | 2.50038E-13 | 5.42386E-12 | - |
| *XERICO* | 302.9805983 | 2313.895034 | 7.637106294 | 2.933026103 | 2.52897E-13 | 5.47687E-12 | K16285 |
| *pip* | 266.2374781 | 889.7838917 | 3.34206851 | 1.740741308 | 2.62335E-13 | 5.6673E-12 | - |
| *QS* | 252.5714042 | 845.2144806 | 3.346437746 | 1.742626176 | 2.77573E-13 | 5.97202E-12 | K03517 |
| *RBL1* | 147.9786618 | 557.1347345 | 3.764966702 | 1.912637106 | 3.22985E-13 | 6.92081E-12 | - |
| *uncharacterized protein_30216* | 195.0375254 | 667.0707503 | 3.420217462 | 1.774088057 | 3.26718E-13 | 6.99512E-12 | K10268 |
| *GSVIVT00026920001* | 4.492865481 | 67.67575057 | 15.0629372 | 3.912931211 | 3.70698E-13 | 7.91102E-12 | - |
| *NAC068* | 0.982560392 | 154.9766813 | 157.7273851 | 7.301289357 | 3.72116E-13 | 7.93486E-12 | - |
| *RRN3* | 553.8566573 | 1721.735667 | 3.108630445 | 1.636279119 | 4.08827E-13 | 8.67556E-12 | K15216 |
| *VPS2.2* | 843.0545315 | 2555.967267 | 3.031793521 | 1.600171503 | 4.17902E-13 | 8.85388E-12 | K12191 |
| *LOX1.1* | 0 | 86.7518337 | Inf | Inf | 4.32654E-13 | 9.13415E-12 | K15718 |
| *CRRSP55* | 0 | 36.8188055 | Inf | Inf | 4.32864E-13 | 9.13415E-12 | - |
| *MYB2* | 1.610882779 | 637.5917865 | 395.8027207 | 8.628637719 | 4.3497E-13 | 9.17126E-12 | K09422 |
| *CRL1* | 0 | 36.78180638 | Inf | Inf | 4.38147E-13 | 9.23086E-12 | - |
| *ASK5* | 1487.973308 | 4641.930912 | 3.119633186 | 1.641376403 | 4.42746E-13 | 9.3203E-12 | K03083 |
| *At5g25050* | 42.29513483 | 206.704816 | 4.887200782 | 2.289008376 | 4.53441E-13 | 9.53022E-12 | - |
| *PAP27* | 15.23861995 | 357.3989197 | 23.4534965 | 4.551731114 | 4.63174E-13 | 9.72703E-12 | - |
| *UTR2* | 37.49595781 | 206.3168008 | 5.502374466 | 2.460054326 | 4.9115E-13 | 1.02818E-11 | K15277 |
| *uncharacterized protein_42611* | 33.24839039 | 174.4268683 | 5.246174816 | 2.391265883 | 5.0908E-13 | 1.06402E-11 | - |
| *CHI4* | 1.598071948 | 1674.912007 | 1048.082979 | 10.03353723 | 5.38034E-13 | 1.12276E-11 | K01183 |
| *LBD11* | 7.950406908 | 563.615603 | 70.89141594 | 6.147539041 | 5.6646E-13 | 1.18114E-11 | - |
| *SFR2* | 147.7126751 | 520.4876273 | 3.523649051 | 1.817070242 | 5.81783E-13 | 1.21213E-11 | - |
| *PME51* | 70.7638885 | 293.1194068 | 4.142217351 | 2.050403257 | 6.86539E-13 | 1.42028E-11 | - |
| *ROQ1* | 0 | 36.15518232 | Inf | Inf | 6.96318E-13 | 1.43852E-11 | - |
| *XYL1* | 98.49956892 | 371.4608389 | 3.771192534 | 1.915020808 | 7.17709E-13 | 1.48012E-11 | K01187 |
| *TUBB1* | 76.24235505 | 308.0822691 | 4.040828342 | 2.014651066 | 7.20417E-13 | 1.48454E-11 | K07375 |
| *HXK5* | 27.47173813 | 154.1168972 | 5.610016244 | 2.488004948 | 7.31981E-13 | 1.50602E-11 | K00844 |
| *SSL3* | 681.967567 | 2073.327065 | 3.04021359 | 1.604172683 | 8.65971E-13 | 1.77615E-11 | - |
| *ALDR* | 0 | 35.7700061 | Inf | Inf | 8.99367E-13 | 1.84287E-11 | - |
| *HIPP33* | 557.1539547 | 1716.846035 | 3.081457145 | 1.623612727 | 9.98377E-13 | 2.03505E-11 | - |
| *GRXC1* | 60.57719312 | 270.8271974 | 4.470778249 | 2.16052599 | 1.07447E-12 | 2.18847E-11 | K03676 |
| *uncharacterized protein_36957* | 291.0001036 | 929.8372712 | 3.195315945 | 1.675958588 | 1.17606E-12 | 2.38433E-11 | - |
| *ACR4* | 3.353162502 | 4166.23806 | 1242.480213 | 10.27900716 | 1.21599E-12 | 2.46148E-11 | - |
| *At1g67190* | 57.88774482 | 247.6829378 | 4.278676576 | 2.09716463 | 1.26036E-12 | 2.54154E-11 | - |
| *NCED3* | 370.2724381 | 3668.855465 | 9.908529742 | 3.308671002 | 1.32178E-12 | 2.65931E-11 | K09840 |
| *AVT1B* | 121.6919126 | 438.3953334 | 3.602501792 | 1.848999148 | 1.42997E-12 | 2.87039E-11 | K15015 |
| *At1g62600* | 149.683005 | 519.7064079 | 3.472046863 | 1.79578642 | 1.43541E-12 | 2.87913E-11 | - |
| *At3g22104* | 44.59684559 | 329.6663771 | 7.392145627 | 2.885993179 | 1.4892E-12 | 2.98248E-11 | - |
| *D6PKL1* | 384.2270515 | 1731.838421 | 4.507330792 | 2.172273333 | 1.62454E-12 | 3.24061E-11 | - |
| *uncharacterized protein_25948* | 432.3237796 | 1322.895457 | 3.059964591 | 1.613514959 | 1.64034E-12 | 3.26778E-11 | K03122 |
| *OLE9* | 319.5587529 | 1003.596945 | 3.140570979 | 1.651026876 | 1.74736E-12 | 3.46528E-11 | - |
| *At1g47710* | 81.17876241 | 312.3297869 | 3.847432231 | 1.943895915 | 2.01328E-12 | 3.98064E-11 | K13963 |
| *AK1* | 280.8826716 | 888.307821 | 3.162558288 | 1.66109207 | 2.07265E-12 | 4.09189E-11 | K00928 |
| *ERF115* | 0 | 48.76522276 | Inf | Inf | 2.12644E-12 | 4.19494E-11 | - |
| *ATHB-13* | 35.81785622 | 197.7894621 | 5.522091019 | 2.465214668 | 2.22814E-12 | 4.38899E-11 | K09338 |
| *Os02g0190300* | 0 | 34.50388439 | Inf | Inf | 2.29518E-12 | 4.5143E-11 | K05658 |
| *At1g64890* | 190.5110647 | 628.7198475 | 3.300174972 | 1.722542517 | 2.29748E-12 | 4.51547E-11 | - |
| *MYB2* | 0 | 34.48300212 | Inf | Inf | 2.31115E-12 | 4.53896E-11 | K09422 |
| *GOLS4* | 14.8923838 | 175.8988421 | 11.81132883 | 3.562099379 | 2.36194E-12 | 4.63525E-11 | K18819 |
| *PGLR* | 0 | 47.45247162 | Inf | Inf | 2.37971E-12 | 4.66665E-11 | K01213 |
| *PPT1* | 811.9337376 | 2373.360111 | 2.923095815 | 1.54749712 | 2.53198E-12 | 4.93586E-11 | K01074 |
| *At4g31480* | 641.758488 | 1920.303775 | 2.992253022 | 1.581232173 | 3.09741E-12 | 6.01144E-11 | K17301 |
| *BZIP17* | 440.9820382 | 1342.444323 | 3.044215426 | 1.606070456 | 3.15172E-12 | 6.11233E-11 | - |
| *sll0005* | 1187.214039 | 3442.908043 | 2.899989329 | 1.536047592 | 3.2224E-12 | 6.24482E-11 | K08869 |
| *SPCC777.06c* | 14.91000361 | 423.4910122 | 28.40314619 | 4.827978839 | 3.2994E-12 | 6.37998E-11 | - |
| *PILS7* | 111.0775269 | 434.5452642 | 3.912089838 | 1.967939501 | 3.32036E-12 | 6.41579E-11 | K07088 |
| *BLH1* | 2182.93783 | 6219.771549 | 2.849266463 | 1.510590548 | 3.46977E-12 | 6.69958E-11 | - |
| *11S2* | 41.04013437 | 226.6336435 | 5.522244187 | 2.465254684 | 3.52366E-12 | 6.79368E-11 | - |
| *MFT1* | 6.574928059 | 275.7796749 | 41.94413573 | 5.390397214 | 3.56414E-12 | 6.86169E-11 | - |
| *MBD10* | 1195.132871 | 3920.901475 | 3.28072432 | 1.714014369 | 3.6658E-12 | 7.05226E-11 | - |
| *uncharacterized protein_19224* | 113.9529323 | 405.4692911 | 3.558217264 | 1.831154604 | 3.68695E-12 | 7.0826E-11 | - |
| *EPHX2* | 201.7041456 | 652.9797892 | 3.237314669 | 1.694797603 | 3.7267E-12 | 7.15374E-11 | - |
| *uncharacterized protein_31073* | 6.209647523 | 539.3406 | 86.85526803 | 6.44054145 | 3.96201E-12 | 7.58886E-11 | - |
| *FUM1* | 41.65716627 | 192.6415457 | 4.624451517 | 2.209282265 | 4.06211E-12 | 7.77495E-11 | K01679 |
| *SDH2-1* | 676.4085749 | 2057.950897 | 3.042467191 | 1.605241705 | 4.12946E-12 | 7.88667E-11 | K00235 |
| *UBP25* | 105.1051756 | 377.215524 | 3.588933865 | 1.843555338 | 4.21844E-12 | 8.04495E-11 | K11855 |
| *RIBA1* | 210.2262027 | 826.1651973 | 3.929886888 | 1.974487789 | 4.25115E-12 | 8.10146E-11 | K14652 |
| *CHI4* | 0.314161193 | 244.9541098 | 779.7083628 | 9.606790798 | 4.70117E-12 | 8.9268E-11 | K01183 |
| *SAT5* | 312.4509159 | 954.862787 | 3.056040928 | 1.611663865 | 4.78299E-12 | 9.07563E-11 | K00640 |
| *CIPK32* | 17.00487702 | 130.8212984 | 7.693163454 | 2.943576961 | 5.07153E-12 | 9.6024E-11 | K07198 |
| *uncharacterized protein_34118* | 80.64858539 | 304.1402558 | 3.771179052 | 1.91501565 | 5.28615E-12 | 1.00016E-10 | - |
| *uncharacterized protein_32300* | 102.723035 | 496.1634587 | 4.830109027 | 2.272055755 | 5.57083E-12 | 1.05326E-10 | - |
| *CAD* | 32.6171513 | 2497.15771 | 76.55965069 | 6.258512342 | 5.71884E-12 | 1.07892E-10 | K00083 |
| *uncharacterized protein_24093* | 76.38327419 | 291.8805415 | 3.821262503 | 1.934049368 | 5.87785E-12 | 1.10655E-10 | - |
| *MAPKKK17* | 3.236220707 | 252.724754 | 78.09255823 | 6.287113169 | 6.02711E-12 | 1.13303E-10 | - |
| *ALDH7B4* | 120.504255 | 708.964981 | 5.883319068 | 2.556630279 | 6.21373E-12 | 1.16728E-10 | K14085 |
| *APY7* | 311.3295766 | 949.6902477 | 3.05043375 | 1.609014398 | 6.38669E-12 | 1.19891E-10 | - |
| *CYP750A1* | 9.626988159 | 190.5703038 | 19.7954231 | 4.307094998 | 7.18859E-12 | 1.34657E-10 | - |
| *GPX4* | 13.58753638 | 1272.128421 | 93.62465608 | 6.548816609 | 7.63832E-12 | 1.42575E-10 | K00432 |
| *DCR* | 0.314161193 | 472.4352019 | 1503.798724 | 10.55439577 | 7.83088E-12 | 1.46066E-10 | - |
| *PLIP2* | 118.3544777 | 409.944631 | 3.463701916 | 1.792314778 | 7.89985E-12 | 1.47248E-10 | - |
| *Sb03g046810* | 606.4610497 | 7263.060016 | 11.97613601 | 3.582090606 | 7.97107E-12 | 1.4847E-10 | K00430 |
| *ATJ49* | 831.5418844 | 2377.333157 | 2.858945775 | 1.515483257 | 8.35026E-12 | 1.55314E-10 | K09518 |
| *uncharacterized protein_23707* | 0.367048835 | 36.11311937 | 98.38777813 | 6.620407208 | 8.60965E-12 | 1.60025E-10 | - |
| *SAC2* | 130.9244338 | 444.600153 | 3.395853166 | 1.763774079 | 8.95052E-12 | 1.6601E-10 | - |
| *BSK2* | 224.2968292 | 799.2260148 | 3.563251507 | 1.833194318 | 9.03699E-12 | 1.67378E-10 | K14500 |
| *SD25* | 194.4310358 | 628.1952976 | 3.230941475 | 1.691954618 | 9.13497E-12 | 1.69074E-10 | - |
| *TPR2* | 82.56134267 | 410.3269683 | 4.969964816 | 2.313235639 | 9.19712E-12 | 1.70105E-10 | - |
| *ERF113* | 36.12389157 | 167.8735495 | 4.647161261 | 2.216349709 | 9.63497E-12 | 1.77953E-10 | - |
| *LHW* | 813.1511624 | 2333.238812 | 2.86937893 | 1.520738503 | 9.64626E-12 | 1.78037E-10 | - |
| *PSI1* | 523.3655258 | 1519.910355 | 2.904108659 | 1.538095434 | 9.77855E-12 | 1.80226E-10 | - |
| *uncharacterized protein_08496* | 139.8976019 | 470.5004821 | 3.363177608 | 1.749824969 | 9.81707E-12 | 1.8081E-10 | - |
| *uncharacterized protein_51532* | 93.92983846 | 412.067149 | 4.386967505 | 2.13322402 | 1.04663E-11 | 1.92633E-10 | - |
| *OTU9* | 44.86916167 | 195.6797919 | 4.361119858 | 2.124698641 | 1.05066E-11 | 1.93239E-10 | - |
| *OTU12* | 55.5284332 | 228.5016476 | 4.115038628 | 2.040905973 | 1.07163E-11 | 1.96822E-10 | - |
| *zntB* | 83.38155134 | 486.1796813 | 5.830782391 | 2.543689481 | 1.15278E-11 | 2.10844E-10 | - |
| *RBOHC* | 148.8509817 | 1134.168741 | 7.619491175 | 2.929694659 | 1.15413E-11 | 2.10946E-10 | K13447 |
| *ACX3* | 1264.660551 | 3563.414181 | 2.817684301 | 1.494509978 | 1.19499E-11 | 2.1796E-10 | K00232 |
| *uncharacterized protein_31982* | 2785.165767 | 7857.656177 | 2.821252605 | 1.496335846 | 1.25701E-11 | 2.28638E-10 | - |
| *NAC068* | 0.301350363 | 50.34211814 | 167.0551105 | 7.384180307 | 1.28857E-11 | 2.33894E-10 | - |
| *At4g32940* | 950.9411801 | 7044.116035 | 7.407520236 | 2.888990662 | 1.30007E-11 | 2.35821E-10 | K01369 |
| *NAGS1* | 356.6295933 | 1054.311073 | 2.956319645 | 1.563802266 | 1.30515E-11 | 2.36579E-10 | K14682 |
| *WAPL2* | 614.7349529 | 1754.263982 | 2.853691617 | 1.512829439 | 1.31514E-11 | 2.38225E-10 | - |
| *UNC* | 13.64854986 | 97.73356458 | 7.160728838 | 2.840106436 | 1.33986E-11 | 2.42536E-10 | - |
| *CBSCBS2* | 27.61278125 | 143.7812499 | 5.207054249 | 2.380467437 | 1.4205E-11 | 2.56078E-10 | - |
| *CIPK26* | 27.72324153 | 141.5404265 | 5.105478967 | 2.352046313 | 1.46664E-11 | 2.63855E-10 | K07198 |
| *AOX2* | 77.57918167 | 569.8837443 | 7.345833407 | 2.876926178 | 1.72442E-11 | 3.09385E-10 | K17893 |
| *At1g60710* | 7.626475561 | 103.8229371 | 13.61348847 | 3.766964901 | 1.7659E-11 | 3.16182E-10 | - |
| *INT2* | 321.1183923 | 1078.934014 | 3.359925935 | 1.748429431 | 1.77401E-11 | 3.17419E-10 | K08150 |
| *uncharacterized protein_07478* | 1287.392982 | 3893.095104 | 3.024014547 | 1.59646508 | 1.8645E-11 | 3.32933E-10 | - |
| *RUN1* | 112.5075377 | 435.116878 | 3.867446456 | 1.951381318 | 1.87987E-11 | 3.35449E-10 | - |
| *ATL23* | 4.819837505 | 72.93851731 | 15.13298264 | 3.919624459 | 1.98047E-11 | 3.52923E-10 | - |
| *7OMT* | 0.92967275 | 40.00990666 | 43.0365488 | 5.427490484 | 2.11253E-11 | 3.74683E-10 | - |
| *uncharacterized protein_30005* | 152.3515166 | 631.3374599 | 4.143952577 | 2.051007493 | 2.35612E-11 | 4.15928E-10 | - |
| *CPSF30* | 89.31723846 | 319.601123 | 3.578269196 | 1.839261926 | 2.5322E-11 | 4.45816E-10 | K14404 |
| *Pabpc1* | 925.2533145 | 2578.024002 | 2.78628994 | 1.478345392 | 2.6119E-11 | 4.59234E-10 | K13126 |
| *INVE* | 1397.211806 | 5468.005416 | 3.913512176 | 1.968463934 | 2.61486E-11 | 4.59448E-10 | - |
| *XYL1* | 126.5134902 | 587.9636861 | 4.647438665 | 2.216435825 | 2.77494E-11 | 4.8725E-10 | K01187 |
| *At5g05130* | 311.2644305 | 1110.778366 | 3.568600384 | 1.835358356 | 2.81156E-11 | 4.93023E-10 | K15711 |
| *MLS* | 33.08808314 | 1052.792284 | 31.81786868 | 4.991765295 | 2.88655E-11 | 5.05501E-10 | K01638 |
| *At4g33300* | 0.94248358 | 553.5707918 | 587.3532476 | 9.198084624 | 3.01084E-11 | 5.2587E-10 | - |
| *XRN3* | 598.0463729 | 1696.563688 | 2.836843035 | 1.504286327 | 3.06355E-11 | 5.34721E-10 | K12619 |
| *MMS21* | 116.6920754 | 386.8385879 | 3.315037346 | 1.729025123 | 3.08941E-11 | 5.38523E-10 | - |
| *DBR* | 146.1010441 | 477.4249535 | 3.267772359 | 1.708307486 | 3.15201E-11 | 5.48708E-10 | - |
| *AGD7* | 1659.542476 | 4520.302811 | 2.723824714 | 1.445633865 | 3.43699E-11 | 5.96456E-10 | K12492 |
| *ELI3* | 1.415307699 | 106.6674892 | 75.36699563 | 6.235860978 | 3.49298E-11 | 6.05497E-10 | K00083 |
| *SRG1* | 319.2565347 | 926.6020425 | 2.902374554 | 1.537233713 | 3.54732E-11 | 6.14279E-10 | - |
| *NAC018* | 0 | 38.03029552 | Inf | Inf | 3.70772E-11 | 6.40372E-10 | - |
| *uncharacterized protein_39161* | 1128.262808 | 3080.915302 | 2.730671685 | 1.449255866 | 3.73737E-11 | 6.45069E-10 | - |
| *APM1* | 153.9928583 | 491.208075 | 3.189810752 | 1.673470833 | 3.75888E-11 | 6.47934E-10 | K08776 |
| *At5g34940* | 460.8881621 | 1317.865511 | 2.859404124 | 1.515714533 | 3.91285E-11 | 6.73153E-10 | K07964 |
| *HGO* | 73.15834397 | 270.0740667 | 3.691637236 | 1.884260792 | 4.08308E-11 | 7.01524E-10 | K00451 |
| *CYP707A1* | 0 | 30.56046767 | Inf | Inf | 4.15695E-11 | 7.13284E-10 | K09843 |
| *ABCG14* | 52.72306753 | 1081.825355 | 20.51901389 | 4.358889494 | 4.41227E-11 | 7.55619E-10 | - |
| *At2g19810* | 1426.722818 | 3854.687478 | 2.701777409 | 1.43390882 | 4.42612E-11 | 7.57499E-10 | - |
| *CSA* | 0 | 64.22497219 | Inf | Inf | 4.43716E-11 | 7.58404E-10 | K00432 |
| *PP2A* | 1212.152727 | 3301.928073 | 2.724019835 | 1.445737209 | 4.51981E-11 | 7.72029E-10 | K04382 |
| *INT1* | 64.82996968 | 252.2011665 | 3.890194115 | 1.959842145 | 4.80727E-11 | 8.19536E-10 | K08150 |
| *MYB2* | 1.952309952 | 282.148439 | 144.5203097 | 7.175128442 | 4.92798E-11 | 8.39031E-10 | K09422 |
| *GAT1* | 41.447384 | 404.9903715 | 9.771192594 | 3.288534657 | 5.45426E-11 | 9.24055E-10 | - |
| *At1g60710* | 18.19265866 | 3831.244332 | 210.5928772 | 7.718312831 | 5.48549E-11 | 9.27362E-10 | - |
| *EXPA8* | 0 | 97.35458292 | Inf | Inf | 5.81851E-11 | 9.79888E-10 | - |
| *NANMT1* | 276.3005022 | 812.2323196 | 2.939670081 | 1.55565425 | 5.98437E-11 | 1.00654E-09 | - |
| *AKR4C9* | 533.1213987 | 1479.413237 | 2.775002543 | 1.472489094 | 6.25691E-11 | 1.0514E-09 | - |
| *SWEET2A* | 69.02744217 | 251.3716159 | 3.64161858 | 1.864579823 | 6.25909E-11 | 1.0514E-09 | K15382 |
| *At1g54290* | 2808.042739 | 7398.324111 | 2.634690708 | 1.397633611 | 6.28419E-11 | 1.05494E-09 | K03113 |
| *TIFY10B* | 5.921107991 | 403.5631007 | 68.15668644 | 6.090783293 | 6.32915E-11 | 1.06114E-09 | K13464 |
| *HIP1* | 113.0201907 | 661.2734289 | 5.850931811 | 2.548666405 | 6.34905E-11 | 1.0638E-09 | K10635 |
| *uncharacterized protein_19242* | 125.0163846 | 408.816776 | 3.270105573 | 1.709337213 | 6.43345E-11 | 1.07726E-09 | - |
| *At2g42990* | 3.039125289 | 468.343471 | 154.1046934 | 7.267766991 | 6.54407E-11 | 1.09508E-09 | - |
| *PLAT1* | 22.60878314 | 182.9572384 | 8.092308078 | 3.016551245 | 6.57312E-11 | 1.09925E-09 | - |
| *uncharacterized protein_19233* | 186.3529251 | 578.413572 | 3.103860976 | 1.63406394 | 7.12351E-11 | 1.18827E-09 | - |
| *uncharacterized protein_21477* | 1.415307699 | 90.43736802 | 63.899439 | 5.99773136 | 7.17503E-11 | 1.19611E-09 | - |
| *pgk* | 62.32300944 | 347.5864971 | 5.577177678 | 2.479535233 | 7.23693E-11 | 1.20567E-09 | K00927 |
| *BETA-OHASE 1* | 7.637642073 | 68.99965419 | 9.034156554 | 3.175389915 | 7.37984E-11 | 1.2287E-09 | K15746 |
| *ABCC10* | 173.2200645 | 539.8320862 | 3.116452402 | 1.639904679 | 7.7429E-11 | 1.28671E-09 | - |
| *LTI6B* | 2.671952473 | 220.4873407 | 82.51918508 | 6.366657669 | 8.09135E-11 | 1.34292E-09 | - |
| *SCPL50* | 19.52133122 | 820.8592712 | 42.04934908 | 5.394011563 | 8.19599E-11 | 1.35943E-09 | K09645 |
| *At5g07050* | 17.82889847 | 895.9788772 | 50.25430365 | 5.651175245 | 8.23518E-11 | 1.36336E-09 | - |
| *ERF1A* | 0 | 34.33805642 | Inf | Inf | 8.27117E-11 | 1.36846E-09 | K09286 |
| *ACX2* | 881.6274865 | 6692.821139 | 7.591438835 | 2.924373351 | 8.47327E-11 | 1.40013E-09 | K00232 |
| *Os01g0939600* | 516.0292623 | 1432.004822 | 2.77504577 | 1.472511567 | 8.63201E-11 | 1.42458E-09 | K00006 |
| *NPF8.3* | 32.30767511 | 262.8668545 | 8.136359353 | 3.024383399 | 8.72985E-11 | 1.43983E-09 | K14638 |
| *NCL2* | 287.6267766 | 837.8060264 | 2.912823473 | 1.542418272 | 8.83748E-11 | 1.45575E-09 | - |
| *PDC4* | 48.24806981 | 194.1006524 | 4.022972382 | 2.008261834 | 9.04296E-11 | 1.48867E-09 | K01568 |
| *PAB1* | 144.6290359 | 456.4853461 | 3.156249664 | 1.658211329 | 9.09885E-11 | 1.49694E-09 | K13126 |
| *At1g48650* | 572.0775977 | 1596.685486 | 2.791029561 | 1.480797405 | 9.11972E-11 | 1.49943E-09 | K13026 |
| *MC410* | 246.4198817 | 942.7954416 | 3.825971489 | 1.935826123 | 9.26869E-11 | 1.52203E-09 | - |
| *SCPL50* | 50.12174648 | 198.3218025 | 3.956801517 | 1.984334698 | 1.04575E-10 | 1.71191E-09 | K09645 |
| *DIVARICATA* | 391.9476074 | 1100.495984 | 2.807762985 | 1.489421157 | 1.133E-10 | 1.84443E-09 | - |
| *BSPA* | 12.44935374 | 485.4381768 | 38.99304227 | 5.285144814 | 1.15849E-10 | 1.88476E-09 | - |
| *AIP2* | 440.6390907 | 1235.588429 | 2.804082649 | 1.487528873 | 1.17187E-10 | 1.90536E-09 | K16274 |
| *ppt-1* | 611.5361402 | 1663.094554 | 2.719536008 | 1.443360528 | 1.17306E-10 | 1.90611E-09 | K01074 |
| *ALP1* | 208.4183484 | 617.0264199 | 2.960518709 | 1.565849971 | 1.19672E-10 | 1.94217E-09 | - |
| *cys-12* | 23.30420036 | 120.9883048 | 5.191695187 | 2.376205683 | 1.2008E-10 | 1.94639E-09 | K01738 |
| *NHX3* | 112.5414092 | 476.8333047 | 4.236958717 | 2.083029072 | 1.26082E-10 | 2.04113E-09 | - |
| *TSN1* | 1011.843596 | 2724.42759 | 2.692538254 | 1.428966842 | 1.38683E-10 | 2.23692E-09 | K15979 |
| *uncharacterized protein_34114* | 7.847672302 | 69.1095337 | 8.806373538 | 3.13854804 | 1.39746E-10 | 2.25132E-09 | - |
| *CCR1* | 1758.088012 | 7821.830507 | 4.449055141 | 2.153498979 | 1.40239E-10 | 2.25787E-09 | - |
| *GSTU19* | 1292.938911 | 3436.210242 | 2.65767409 | 1.410164198 | 1.40682E-10 | 2.26363E-09 | K00799 |
| *RBL1* | 363.448671 | 1433.876304 | 3.945196168 | 1.980097038 | 1.46928E-10 | 2.36124E-09 | - |
| *grx* | 101.3433671 | 335.7926042 | 3.313414719 | 1.728318788 | 1.48268E-10 | 2.38133E-09 | - |
| *CYP710A11* | 5.789587066 | 60.90177959 | 10.51919228 | 3.394952026 | 1.54124E-10 | 2.47086E-09 | K09832 |
| *AGAL2* | 372.1184725 | 1037.951173 | 2.789303004 | 1.479904664 | 1.63355E-10 | 2.61566E-09 | K07407 |
| *MES1* | 1.205401451 | 598.0417506 | 496.1349185 | 8.954588689 | 1.71111E-10 | 2.73487E-09 | - |
| *TEB* | 207.33485 | 611.7359881 | 2.950473537 | 1.560946519 | 1.71491E-10 | 2.73928E-09 | K14574 |
| *At1g75220* | 35.14616838 | 483.9675692 | 13.77013744 | 3.783471054 | 1.74808E-10 | 2.79057E-09 | K08145 |
| *PAO2* | 368.6023384 | 1607.356955 | 4.360680298 | 2.124553224 | 1.80397E-10 | 2.87631E-09 | K17839 |
| *RBL1* | 21.51219765 | 1858.643194 | 86.39950341 | 6.432951115 | 1.8421E-10 | 2.93534E-09 | - |
| *PAB8* | 664.5842832 | 1795.284326 | 2.701364404 | 1.433688267 | 1.89247E-10 | 3.01195E-09 | K13126 |
| *uncharacterized protein_35098* | 61.06295205 | 224.2194916 | 3.671939925 | 1.876542455 | 1.93283E-10 | 3.07062E-09 | - |
| *CRK19* | 1365.100424 | 3579.409643 | 2.622085218 | 1.390714574 | 1.9766E-10 | 3.13827E-09 | - |
| *LTI6B* | 2.752106096 | 142.8546068 | 51.90737633 | 5.697867663 | 2.04481E-10 | 3.2407E-09 | - |
| *PGL3* | 65.85273086 | 241.3539509 | 3.665056069 | 1.873835269 | 2.06971E-10 | 3.27624E-09 | - |
| *SCL1* | 643.4096717 | 1730.250147 | 2.689188899 | 1.427171099 | 2.14528E-10 | 3.38975E-09 | - |
| *LOX1.5* | 2.319358788 | 50.43253807 | 21.74417271 | 4.442556916 | 2.26715E-10 | 3.57374E-09 | K15718 |
| *rbm24* | 499.9019424 | 1362.200995 | 2.72493639 | 1.446222553 | 2.30156E-10 | 3.62582E-09 | - |
| *ABCC10* | 170.0623813 | 545.0430731 | 3.204959667 | 1.680306201 | 2.30364E-10 | 3.62693E-09 | - |
| *uncharacterized protein_09494* | 284.1482027 | 808.5572049 | 2.845547489 | 1.508706257 | 2.37606E-10 | 3.73871E-09 | - |
| *CG13604* | 172.8524916 | 517.2579856 | 2.992482092 | 1.581342614 | 2.37867E-10 | 3.74058E-09 | - |
| *SCPL26* | 10.06125581 | 76.18131174 | 7.57174981 | 2.920626742 | 2.39453E-10 | 3.76104E-09 | K16297 |
| *CHX20* | 8.974564449 | 623.3359336 | 69.45584235 | 6.118024148 | 2.40723E-10 | 3.77873E-09 | - |
| *PPC* | 2776.374418 | 7615.47489 | 2.742956729 | 1.455731863 | 2.4394E-10 | 3.82467E-09 | K01595 |
| *PYL4* | 0 | 28.08295542 | Inf | Inf | 2.45952E-10 | 3.85393E-09 | K14496 |
| *RCD1* | 67.06306527 | 1311.56763 | 19.55722759 | 4.289629965 | 2.55755E-10 | 4.00041E-09 | - |
| *NCL2* | 80.45629895 | 318.8367296 | 3.962856032 | 1.986540556 | 2.57136E-10 | 4.01827E-09 | - |
| *uncharacterized protein_02495* | 1.035448033 | 346.8227629 | 334.9494631 | 8.387799629 | 2.57202E-10 | 4.01827E-09 | - |
| *HMA5* | 5.004246072 | 106.6208598 | 21.30607853 | 4.413193178 | 2.85391E-10 | 4.44811E-09 | K17686 |
| *EREBP1* | 206.1844764 | 624.0764419 | 3.026786753 | 1.597787036 | 3.05883E-10 | 4.75345E-09 | K09286 |
| *UGT89B2* | 1.218212282 | 67.93362654 | 55.76501531 | 5.801288413 | 3.10337E-10 | 4.81982E-09 | - |
| *MPK9* | 677.3356791 | 1791.162649 | 2.644423886 | 1.402953451 | 3.1792E-10 | 4.92888E-09 | K20538 |
| *B3GALT2* | 283.8212025 | 1506.251354 | 5.307043101 | 2.407908266 | 3.18373E-10 | 4.933E-09 | - |
| *CRJ34* | 126.381841 | 2571.101956 | 20.34391916 | 4.346525729 | 3.28576E-10 | 5.08213E-09 | - |
| *SB09* | 10007.72507 | 25568.28556 | 2.554854914 | 1.353241365 | 3.40832E-10 | 5.26552E-09 | K03671 |
| *SAP4* | 2287.681682 | 5830.718301 | 2.548745461 | 1.349787301 | 3.43567E-10 | 5.30466E-09 | - |
| *ERD4* | 72.18540153 | 251.7762775 | 3.487911297 | 1.802363351 | 3.44829E-10 | 5.32103E-09 | - |
| *CLC-C* | 470.28608 | 1264.741091 | 2.689301566 | 1.427231542 | 3.59646E-10 | 5.53346E-09 | K05016 |
| *tmem214-a* | 524.4429993 | 1408.740155 | 2.686164476 | 1.425547645 | 3.77814E-10 | 5.79609E-09 | - |
| *uncharacterized protein_35310* | 116.9730132 | 664.7785599 | 5.683178894 | 2.506698129 | 4.00246E-10 | 6.1295E-09 | - |
| *FKBP20-1* | 222.0400043 | 638.0405442 | 2.873538696 | 1.522828477 | 4.05934E-10 | 6.20939E-09 | K01802 |
| *uncharacterized protein_06871* | 4.284479571 | 52.3302482 | 12.21391007 | 3.610453223 | 4.06262E-10 | 6.21081E-09 | - |
| *APS1* | 191.9348984 | 561.7717317 | 2.926886858 | 1.549366978 | 4.26445E-10 | 6.50805E-09 | - |
| *RZPF34* | 143.1703628 | 1069.636467 | 7.471074644 | 2.901315776 | 4.41459E-10 | 6.7216E-09 | K10144 |
| *LBD40* | 7.153651442 | 191.9366661 | 26.83058683 | 4.745806704 | 4.41741E-10 | 6.72201E-09 | - |
| *PCK* | 365.4949981 | 995.1529261 | 2.722753885 | 1.445066581 | 4.84156E-10 | 7.33357E-09 | K01610 |
| *At4g19185* | 112.3115907 | 350.6692023 | 3.122288627 | 1.642603908 | 4.97225E-10 | 7.5272E-09 | - |
| *MST3* | 9.941149353 | 178.907992 | 17.99671101 | 4.169661366 | 5.03419E-10 | 7.61222E-09 | - |
| *CEPR1* | 156.8833105 | 811.0762413 | 5.169933236 | 2.37014565 | 5.12252E-10 | 7.73691E-09 | - |
| *ALDH7B4* | 89.91969123 | 526.0029836 | 5.849697396 | 2.548361996 | 5.33953E-10 | 8.05084E-09 | K14085 |
| *RAB2A* | 15.03659158 | 98.7419291 | 6.566776027 | 2.71518525 | 5.43244E-10 | 8.18156E-09 | K07877 |
| *APM1* | 138.8410932 | 421.2174098 | 3.033809372 | 1.601130437 | 5.64245E-10 | 8.47848E-09 | K08776 |
| *LRK10L-1.2* | 164.939149 | 737.6442105 | 4.472220301 | 2.160991257 | 5.79437E-10 | 8.69683E-09 | - |
| *DDI1* | 346.2681355 | 933.5071731 | 2.695908394 | 1.430771475 | 5.8537E-10 | 8.77588E-09 | K11885 |
| *At4g33300* | 2.186317524 | 1970.427687 | 901.2541249 | 9.815790147 | 5.96702E-10 | 8.94068E-09 | - |
| *ABR1* | 3.970318377 | 274.7373056 | 69.19780216 | 6.112654311 | 6.22752E-10 | 9.32571E-09 | - |
| *SDR1* | 236.1043015 | 668.8867197 | 2.833013695 | 1.502337576 | 6.48667E-10 | 9.70276E-09 | K00059 |
| *GDH1* | 59.2547259 | 249.8363055 | 4.216310204 | 2.075981014 | 6.66466E-10 | 9.9577E-09 | K00261 |
| *SPL16* | 293.6977974 | 1042.254749 | 3.548731923 | 1.827303595 | 6.694E-10 | 9.99587E-09 | - |
| *GAUT13* | 61.35161557 | 218.8544582 | 3.567215895 | 1.834798533 | 6.77762E-10 | 1.0115E-08 | K13648 |
| *EIF4B1* | 915.272645 | 2353.47956 | 2.571342619 | 1.362521855 | 6.989E-10 | 1.04128E-08 | - |
| *NUP50B* | 900.3876543 | 2390.054634 | 2.654472907 | 1.408425416 | 7.22196E-10 | 1.07295E-08 | K15304 |
| *CTU2* | 55.82634273 | 205.1270991 | 3.674378243 | 1.877500146 | 7.30099E-10 | 1.08347E-08 | K14169 |
| *At1g73050* | 0 | 84.95770905 | Inf | Inf | 7.71627E-10 | 1.14253E-08 | K00108 |
| *D1* | 452.4077347 | 4155.318891 | 9.184897984 | 3.199263698 | 8.06609E-10 | 1.19232E-08 | K07513 |
| *CHIT1* | 323.9326885 | 1461.473661 | 4.51165848 | 2.173657864 | 8.32857E-10 | 1.22836E-08 | - |
| *uncharacterized protein_35980* | 402.4899371 | 1460.82078 | 3.629459136 | 1.859754573 | 8.56709E-10 | 1.26212E-08 | - |
| *HSP21* | 590.6816712 | 1537.749722 | 2.603347619 | 1.380367964 | 8.62723E-10 | 1.26957E-08 | K13993 |
| *CINV2* | 748.0450638 | 1923.325613 | 2.571136027 | 1.362405939 | 8.84541E-10 | 1.30022E-08 | - |
| *NAC025* | 51.02592155 | 189.6886298 | 3.717495423 | 1.894330966 | 8.87491E-10 | 1.30383E-08 | - |
| *AGT2* | 1110.809456 | 3337.575058 | 3.00463328 | 1.587188918 | 9.40965E-10 | 1.37931E-08 | K00827 |
| *ILL6* | 452.2544288 | 2445.737285 | 5.407879127 | 2.435062906 | 9.70478E-10 | 1.42021E-08 | K14664 |
| *AMO* | 8.527361992 | 68.15094884 | 7.992031874 | 2.998562337 | 9.80552E-10 | 1.43415E-08 | K00276 |
| *CCC1* | 144.3820936 | 426.887526 | 2.956651448 | 1.563964178 | 1.0085E-09 | 1.47258E-08 | K13627 |
| *GRMZM2G124911* | 246.1952441 | 1271.716867 | 5.165481045 | 2.368902709 | 1.02763E-09 | 1.49885E-08 | K15108 |
| *NAC068* | 8.161833495 | 715.2855456 | 87.63785074 | 6.453482198 | 1.07555E-09 | 1.56442E-08 | - |
| *MFPA* | 40.97406396 | 476.9158347 | 11.63945649 | 3.540951787 | 1.1045E-09 | 1.60475E-08 | K10527 |
| *ALMT9* | 75.00485042 | 293.0654907 | 3.907287183 | 1.966167297 | 1.10668E-09 | 1.60704E-08 | - |
| *SAMDC* | 320.0331538 | 1890.01767 | 5.905693355 | 2.562106448 | 1.12064E-09 | 1.62462E-08 | K01611 |
| *GLT1* | 1107.854717 | 2834.076241 | 2.558165975 | 1.35510987 | 1.13329E-09 | 1.64115E-08 | K00264 |
| *MPK* | 166.1955176 | 478.9717535 | 2.881977567 | 1.527059106 | 1.14215E-09 | 1.65261E-08 | K14512 |
| *Def1* | 3.537571069 | 60.36030624 | 17.0626413 | 4.092769089 | 1.14279E-09 | 1.65261E-08 | - |
| *G6PGH1* | 1633.526412 | 4621.596038 | 2.82921415 | 1.500401382 | 1.14308E-09 | 1.65261E-08 | K00033 |
| *uncharacterized protein_32482* | 78.49601536 | 681.6248296 | 8.683559624 | 3.118286563 | 1.24327E-09 | 1.79059E-08 | - |
| *HSL1* | 213.597109 | 886.3367867 | 4.149572956 | 2.052962872 | 1.25834E-09 | 1.8113E-08 | K00924 |
| *uncharacterized protein_26639* | 310.9448797 | 833.0783825 | 2.679183472 | 1.421793381 | 1.27156E-09 | 1.82933E-08 | - |
| *INV1* | 9.117375867 | 105.1937686 | 11.53772424 | 3.528286782 | 1.28981E-09 | 1.85457E-08 | K01193 |
| *NET1D* | 801.2131892 | 2710.163102 | 3.382574249 | 1.758121602 | 1.30378E-09 | 1.87261E-08 | - |
| *NAC018* | 61.09682353 | 793.537745 | 12.9881997 | 3.699129567 | 1.3069E-09 | 1.87608E-08 | - |
| *MED26B* | 140.4230375 | 412.6252816 | 2.938444354 | 1.555052578 | 1.38732E-09 | 1.98073E-08 | - |
| *EDR2* | 230.7165352 | 640.8950988 | 2.777846409 | 1.473966833 | 1.4107E-09 | 2.01193E-08 | - |
| *Ifrd1* | 141.3555311 | 800.9513303 | 5.666218533 | 2.502386244 | 1.42655E-09 | 2.03343E-08 | - |
| *uncharacterized protein_34968* | 622.7465211 | 1603.290901 | 2.574548145 | 1.364319249 | 1.65935E-09 | 2.35381E-08 | - |
| *GT4* | 0.94248358 | 63.9466247 | 67.84905969 | 6.084256917 | 1.6953E-09 | 2.39968E-08 | - |
| *SDH1* | 906.4120405 | 2300.849546 | 2.538414587 | 1.343927717 | 1.69533E-09 | 2.39968E-08 | K00234 |
| *At2g33840* | 707.5618439 | 1795.581353 | 2.537702349 | 1.343522863 | 1.69695E-09 | 2.40069E-08 | K01866 |
| *GLIP4* | 19.96068403 | 103.3942688 | 5.179896071 | 2.372923152 | 1.6999E-09 | 2.40357E-08 | - |
| *At5g26710* | 430.4803138 | 1120.584655 | 2.603103136 | 1.380232473 | 1.70564E-09 | 2.41039E-08 | K01885 |
| *AHRI* | 2931.336136 | 7191.418293 | 2.453290227 | 1.294717916 | 1.80259E-09 | 2.54059E-08 | K00053 |
| *uncharacterized protein_29508* | 0 | 25.41194798 | Inf | Inf | 1.8221E-09 | 2.56671E-08 | - |
| *COR2* | 293.5820084 | 782.4972641 | 2.665344748 | 1.41432215 | 1.84173E-09 | 2.5916E-08 | - |
| *RER1B* | 387.8558144 | 1020.607415 | 2.631409346 | 1.395835694 | 1.84333E-09 | 2.59246E-08 | - |
| *SGF11* | 94.56969929 | 388.4504842 | 4.10755757 | 2.038280796 | 1.9047E-09 | 2.67734E-08 | K11363 |
| *NPC6* | 17.13639794 | 93.06195139 | 5.430660031 | 2.441127551 | 1.90855E-09 | 2.68133E-08 | K01114 |
| *ACO3* | 65.217679 | 221.5154374 | 3.396555057 | 1.76407224 | 1.93127E-09 | 2.71036E-08 | K01681 |
| *At1g32860* | 132.1669953 | 2719.038153 | 20.57274697 | 4.362662537 | 2.08075E-09 | 2.90776E-08 | - |
| *uncharacterized protein_18322* | 54.1438041 | 196.2128652 | 3.623920935 | 1.85755148 | 2.08346E-09 | 2.90999E-08 | - |
| *At1g58060* | 137.146616 | 397.4062516 | 2.897674498 | 1.534895543 | 2.10916E-09 | 2.94122E-08 | K18995 |
| *GLOX* | 0 | 129.9085493 | Inf | Inf | 2.15075E-09 | 2.99604E-08 | - |
| *uncharacterized protein_45647* | 67.28401405 | 227.8040825 | 3.38570886 | 1.75945792 | 2.22569E-09 | 3.09716E-08 | - |
| *MAO1B* | 4788.217472 | 29057.22384 | 6.06848457 | 2.60133629 | 2.30313E-09 | 3.19929E-08 | K05933 |
| *SFC1* | 218.7345203 | 885.3623404 | 4.047657129 | 2.017087087 | 2.30394E-09 | 3.19929E-08 | K15100 |
| *At3g51130* | 279.9993767 | 745.1167384 | 2.661137132 | 1.412042856 | 2.31145E-09 | 3.20804E-08 | - |
| *uncharacterized protein_42366* | 106.3079083 | 327.370386 | 3.079454683 | 1.622674898 | 2.34102E-09 | 3.24566E-08 | K06100 |
| *TUR2* | 20.98788225 | 147.4184595 | 7.02397973 | 2.812288682 | 2.47832E-09 | 3.42699E-08 | - |
| *CIP7* | 1147.265024 | 2861.982017 | 2.494612803 | 1.318815907 | 2.53934E-09 | 3.50872E-08 | - |
| *sec61a* | 1628.276107 | 4008.393685 | 2.461740775 | 1.299678852 | 2.64842E-09 | 3.65072E-08 | K10956 |
| *IDD14* | 5.685456101 | 143.1574777 | 25.17959424 | 4.65418313 | 2.71611E-09 | 3.74012E-08 | - |
| *CIPK5* | 1.820913007 | 38.14939459 | 20.95069585 | 4.388926257 | 2.77961E-09 | 3.82156E-08 | K07198 |
| *ORP1A* | 549.5657877 | 2063.291401 | 3.754402925 | 1.90858349 | 2.81433E-09 | 3.86527E-08 | K20456 |
| *CIPK2* | 63.29595188 | 285.1108471 | 4.504408871 | 2.171337789 | 2.81859E-09 | 3.8691E-08 | K07198 |
| *tfa2* | 747.8825599 | 1859.562486 | 2.486436488 | 1.31407958 | 2.82356E-09 | 3.8739E-08 | K03137 |
| *DI19-4* | 1001.943997 | 2456.701417 | 2.451934864 | 1.293920654 | 3.29465E-09 | 4.48521E-08 | - |
| *uncharacterized protein_13325* | 273.629422 | 1023.880024 | 3.741849166 | 1.903751405 | 3.32098E-09 | 4.51873E-08 | - |
| *OST3B* | 227.1676737 | 816.4914829 | 3.594223904 | 1.845680285 | 3.39373E-09 | 4.61057E-08 | K12669 |
| *uncharacterized protein_23643* | 28.80853653 | 124.7096467 | 4.328912945 | 2.114004788 | 3.45101E-09 | 4.68598E-08 | - |
| *CLF* | 446.428158 | 1127.238163 | 2.525015823 | 1.336292429 | 3.47782E-09 | 4.71995E-08 | K11430 |
| *slx8* | 255.7109999 | 679.6624318 | 2.657931932 | 1.410304158 | 3.52972E-09 | 4.78792E-08 | - |
| *At1g80440* | 25.94076102 | 201.1817623 | 7.755430236 | 2.955206818 | 3.53861E-09 | 4.79751E-08 | - |
| *PIN2* | 346.4178679 | 892.1312241 | 2.57530372 | 1.364742588 | 3.6189E-09 | 4.90022E-08 | K13947 |
| *At4g16230* | 584.6330748 | 1738.845563 | 2.974251096 | 1.572526449 | 3.66673E-09 | 4.961E-08 | - |
| *VSR4* | 214.9161636 | 578.8634254 | 2.693438296 | 1.429449015 | 3.67226E-09 | 4.96593E-08 | - |
| *P85* | 75.6782108 | 246.0929699 | 3.251833881 | 1.70125356 | 3.70858E-09 | 5.00992E-08 | K19366 |
| *LKR/SDH* | 37.25890956 | 146.8643981 | 3.941725612 | 1.978827352 | 3.71212E-09 | 5.01213E-08 | K14157 |
| *LOX1.1* | 0 | 24.51640782 | Inf | Inf | 3.74583E-09 | 5.05246E-08 | K15718 |
| *At1g64760* | 561.8730782 | 1408.609949 | 2.506989574 | 1.325955996 | 3.77406E-09 | 5.08273E-08 | - |
| *GH5FP* | 129.1453659 | 526.0848375 | 4.073586643 | 2.026299594 | 3.84754E-09 | 5.17376E-08 | - |
| *TUR2* | 676.1130297 | 3436.407065 | 5.082592575 | 2.345564588 | 3.86085E-09 | 5.18902E-08 | - |
| *GCH1* | 121.1309048 | 355.769994 | 2.937070391 | 1.554377842 | 3.90457E-09 | 5.23976E-08 | K01495 |
| *ARAC11* | 235.5676756 | 630.8156909 | 2.677853357 | 1.421076959 | 3.99018E-09 | 5.34866E-08 | K04392 |
| *FAMA* | 283.7668903 | 1507.590524 | 5.312778115 | 2.409466461 | 3.99181E-09 | 5.34866E-08 | - |
| *uncharacterized protein_37847* | 23.48544427 | 139.5779442 | 5.943168143 | 2.571232198 | 4.02254E-09 | 5.38436E-08 | - |
| *uncharacterized protein_16858* | 226.4703359 | 611.5509252 | 2.700357743 | 1.433150548 | 4.14856E-09 | 5.54178E-08 | - |
| *LPA1* | 23.88127942 | 609.234434 | 25.51096293 | 4.67304545 | 4.17246E-09 | 5.56807E-08 | - |
| *PDIA6* | 1717.937341 | 4182.380738 | 2.434536254 | 1.283646985 | 4.1777E-09 | 5.57224E-08 | K09584 |
| *sympk* | 302.9368326 | 790.616592 | 2.609839765 | 1.383961233 | 4.43817E-09 | 5.91069E-08 | K06100 |
| *FAB1B* | 496.6780084 | 1285.753469 | 2.588706259 | 1.372231271 | 4.58167E-09 | 6.09466E-08 | K00921 |
| *VAMP714* | 123.00675 | 363.0201623 | 2.951221477 | 1.561312193 | 4.60094E-09 | 6.1151E-08 | K08515 |
| *RITF1* | 53.398044 | 186.4124967 | 3.490998598 | 1.803639777 | 4.6168E-09 | 6.12999E-08 | - |
| *BIP4* | 5464.624213 | 13104.04784 | 2.397977853 | 1.261818335 | 4.67603E-09 | 6.20238E-08 | K09490 |
| *SPAC29B12.11c* | 812.6212094 | 1994.310517 | 2.454169906 | 1.295235133 | 4.7295E-09 | 6.267E-08 | - |
| *XYL1* | 660.5130855 | 6927.380639 | 10.48787797 | 3.3906509 | 4.73404E-09 | 6.26987E-08 | K15925 |
| *GSTF9* | 37.70611201 | 852.1131289 | 22.59880649 | 4.498174677 | 4.86019E-09 | 6.42725E-08 | K00799 |
| *uncharacterized protein_25844* | 0.94248358 | 879.0080572 | 932.6507915 | 9.86519319 | 4.89797E-09 | 6.47397E-08 | - |
| *ADH2* | 597.4582231 | 8268.16676 | 13.83890361 | 3.790657745 | 5.10357E-09 | 6.73559E-08 | K18857 |
| *At4g33300* | 4.428687347 | 4951.407662 | 1118.03053 | 10.12674387 | 5.1094E-09 | 6.73991E-08 | - |
| *GOLS4* | 0.314161193 | 31.92481261 | 101.619211 | 6.667029358 | 5.14115E-09 | 6.77501E-08 | K18819 |
| *BIOF* | 42.34662611 | 231.3406341 | 5.463023986 | 2.449699757 | 5.15165E-09 | 6.78546E-08 | K00652 |
| *TPPD* | 60.95450803 | 359.0756579 | 5.890879436 | 2.558483027 | 5.17297E-09 | 6.80672E-08 | K01087 |
| *uncharacterized protein_26446* | 408.9457506 | 1048.988714 | 2.565104814 | 1.359017778 | 5.28828E-09 | 6.95499E-08 | - |
| *MST3* | 1.66377042 | 58.11004502 | 34.92672084 | 5.126259294 | 5.54615E-09 | 7.28685E-08 | - |
| *GCH1* | 110.447408 | 323.1153762 | 2.925513437 | 1.548689845 | 5.56515E-09 | 7.30817E-08 | K01495 |
| *KIN13A* | 456.3295981 | 1156.693233 | 2.534775825 | 1.341858161 | 5.70587E-09 | 7.47435E-08 | K10393 |
| *PFK2* | 567.1818 | 1643.989152 | 2.8985224 | 1.535317635 | 5.77054E-09 | 7.55156E-08 | K00850 |
| *PDP5* | 271.336085 | 710.9424667 | 2.62015451 | 1.38965189 | 5.8026E-09 | 7.58974E-08 | - |
| *At5g19680* | 158.1012748 | 439.4947146 | 2.779830302 | 1.474996815 | 6.27887E-09 | 8.18832E-08 | K17550 |
| *MPT1* | 70.56170792 | 227.8239528 | 3.228719365 | 1.69096205 | 6.37764E-09 | 8.3089E-08 | K15102 |
| *UGT86A1* | 0.602700725 | 97.05727964 | 161.0372703 | 7.331250813 | 6.81752E-09 | 8.8776E-08 | - |
| *BZR2* | 977.2079606 | 3234.35584 | 3.309792767 | 1.72674089 | 7.07173E-09 | 9.19953E-08 | - |
| *TOGT1* | 0.602700725 | 28.97048689 | 48.06778168 | 5.58699832 | 7.1119E-09 | 9.24723E-08 | K13496 |
| *BCE2* | 24.86536015 | 110.0229892 | 4.424749472 | 2.145595773 | 7.17403E-09 | 9.31881E-08 | K09699 |
| *ULP2B* | 226.1704101 | 601.4446222 | 2.65925424 | 1.411021714 | 7.21413E-09 | 9.36628E-08 | - |
| *LARP6B* | 1645.119813 | 3904.542103 | 2.373408959 | 1.246960711 | 7.86821E-09 | 1.01804E-07 | K15191 |
| *POX1* | 406.8372549 | 2106.813883 | 5.178517596 | 2.372539171 | 7.87279E-09 | 1.01813E-07 | K00318 |
| *MPT3* | 13.308767 | 110.5998529 | 8.310300487 | 3.054900643 | 8.1595E-09 | 1.05366E-07 | K15102 |
| *NAC056* | 11.35481273 | 75.9248649 | 6.686580104 | 2.741268523 | 8.18933E-09 | 1.05648E-07 | - |
| *MYB305* | 0.615511556 | 28.8899091 | 46.9364203 | 5.552635911 | 8.26038E-09 | 1.06512E-07 | K09422 |
| *SB09* | 2078.825281 | 6381.879167 | 3.069944947 | 1.618212784 | 8.26464E-09 | 1.06515E-07 | K03671 |
| *At1g76660* | 184.8336104 | 498.5682352 | 2.697389474 | 1.431563846 | 8.29768E-09 | 1.06888E-07 | - |
| *FIM5* | 1337.229105 | 3195.041306 | 2.389299854 | 1.256587922 | 8.45833E-09 | 1.08798E-07 | K17275 |
| *NHO1* | 138.3119406 | 385.9674716 | 2.79055785 | 1.480553555 | 8.46534E-09 | 1.08835E-07 | - |
| *SBE1* | 424.5793942 | 1067.76987 | 2.51488858 | 1.330494484 | 8.68251E-09 | 1.1141E-07 | K00700 |
| *HMGS* | 1234.150399 | 3704.799553 | 3.001902812 | 1.58587727 | 8.73021E-09 | 1.11913E-07 | K01641 |
| *CLC-D* | 123.7079723 | 1023.247098 | 8.271472557 | 3.048144193 | 8.79646E-09 | 1.12708E-07 | - |
| *AGL19* | 123.5881421 | 356.0715589 | 2.881114264 | 1.526626878 | 8.90653E-09 | 1.13952E-07 | K09260 |
| *GAD1* | 2.881982702 | 40.56567628 | 14.07561407 | 3.815125959 | 8.98863E-09 | 1.14946E-07 | K01580 |
| *POB1* | 1117.047974 | 2681.301122 | 2.400345539 | 1.263242102 | 9.03747E-09 | 1.15515E-07 | - |
| *SNL6* | 729.7179675 | 1774.200376 | 2.431350817 | 1.281758073 | 9.07542E-09 | 1.15831E-07 | - |
| *At1g17710* | 308.0183156 | 792.7963487 | 2.573861061 | 1.363934178 | 9.10545E-09 | 1.16158E-07 | K13248 |
| *UXT3* | 387.4334571 | 1082.735331 | 2.794635597 | 1.482660177 | 9.17384E-09 | 1.16974E-07 | K15285 |
| *PSE1* | 360.9123046 | 903.6850275 | 2.503890879 | 1.32417169 | 9.37312E-09 | 1.19284E-07 | - |
| *VAC14* | 126.2857162 | 359.6761132 | 2.848113975 | 1.510006881 | 9.8167E-09 | 1.24509E-07 | K15305 |
| *R12* | 13.58614002 | 113.0949784 | 8.324290653 | 3.05732734 | 9.84571E-09 | 1.24816E-07 | K00430 |
| *At3g55350* | 74.88133135 | 234.9103191 | 3.137101262 | 1.649432099 | 9.8856E-09 | 1.25262E-07 | - |
| *PUB38* | 88.03307975 | 267.6481612 | 3.040313504 | 1.604220096 | 9.9678E-09 | 1.26182E-07 | - |
| *UGT84A23* | 121.8684433 | 1561.113675 | 12.80982699 | 3.679179086 | 1.00514E-08 | 1.27118E-07 | K13691 |
| *BCE2* | 28.36589509 | 123.9300489 | 4.368980727 | 2.127296742 | 1.04184E-08 | 1.31508E-07 | K09699 |
| *PUB45* | 197.2019101 | 527.8891305 | 2.67689664 | 1.420561435 | 1.05939E-08 | 1.33595E-07 | K08332 |
| *uncharacterized protein_04940* | 440.9403453 | 1104.091801 | 2.503948239 | 1.32420474 | 1.06472E-08 | 1.34172E-07 | - |
| *UBP13* | 1418.279598 | 3376.583022 | 2.380759778 | 1.251422058 | 1.06499E-08 | 1.34172E-07 | K11838 |
| *uncharacterized protein_44540* | 77.28425638 | 332.4169419 | 4.301224563 | 2.104747455 | 1.06794E-08 | 1.3448E-07 | - |
| *RFS6* | 20.60814656 | 1251.042519 | 60.70621222 | 5.923772254 | 1.09454E-08 | 1.37632E-07 | K06617 |
| *MPK4b* | 377.3841443 | 946.862956 | 2.509016264 | 1.327121823 | 1.16846E-08 | 1.46438E-07 | K04464 |
| *DET2* | 233.2996841 | 604.2358337 | 2.589955645 | 1.372927391 | 1.18361E-08 | 1.48266E-07 | K09591 |
| *STP7* | 57.95369126 | 460.7681463 | 7.950626377 | 2.991068525 | 1.19561E-08 | 1.49448E-07 | - |
| *EPHX2* | 27.94304191 | 378.5618342 | 13.54762432 | 3.759967981 | 1.22825E-08 | 1.53203E-07 | - |
| *GPXMC1* | 569.7746908 | 1384.3738 | 2.429686369 | 1.280770099 | 1.23909E-08 | 1.54409E-07 | K00432 |
| *At5g17165* | 267.7182363 | 685.3018064 | 2.559787543 | 1.356024075 | 1.25338E-08 | 1.56115E-07 | - |
| *Os12g0192500* | 46.90326956 | 317.2943902 | 6.764867209 | 2.758061615 | 1.26787E-08 | 1.57622E-07 | - |
| *Sec24a* | 579.0019591 | 1411.897302 | 2.438501769 | 1.285995019 | 1.27827E-08 | 1.5884E-07 | - |
| *uncharacterized protein_22138* | 356.0702862 | 889.6453939 | 2.498510626 | 1.321068354 | 1.28732E-08 | 1.5989E-07 | - |
| *ABCF4* | 446.8538278 | 1099.745667 | 2.461085927 | 1.299295029 | 1.3234E-08 | 1.64139E-07 | K06184 |
| *MAPKKK17* | 1.205401451 | 205.5815468 | 170.5502732 | 7.414053256 | 1.37243E-08 | 1.7006E-07 | - |
| *CHI4* | 1.66377042 | 33.62912059 | 20.21259675 | 4.337182774 | 1.39798E-08 | 1.72901E-07 | K01183 |
| *HRD1A* | 1023.227948 | 2430.065278 | 2.374901196 | 1.247867494 | 1.42825E-08 | 1.76479E-07 | K10601 |
| *METK1* | 81.3010091 | 248.5688541 | 3.057389531 | 1.612300373 | 1.45925E-08 | 1.80225E-07 | K00789 |
| *BAM1* | 3308.067798 | 7540.156035 | 2.279323308 | 1.188605576 | 1.56108E-08 | 1.9235E-07 | K01177 |
| *EMB2271* | 174.0808741 | 472.8715591 | 2.716390077 | 1.441690667 | 1.56686E-08 | 1.92972E-07 | K14793 |
| *NAC018* | 6.196836692 | 53.69307097 | 8.664593508 | 3.115132068 | 1.56792E-08 | 1.93013E-07 | - |
| *PDAT1* | 9.588307707 | 100.391307 | 10.47017994 | 3.388214331 | 1.56939E-08 | 1.93014E-07 | K00679 |
| *ASR3* | 791.3047108 | 1880.405978 | 2.376336135 | 1.248738921 | 1.57196E-08 | 1.9324E-07 | - |
| *clz9* | 366.271937 | 903.8880249 | 2.467805839 | 1.303228891 | 1.59991E-08 | 1.96493E-07 | - |
| *Os10g0513300* | 192.5261564 | 653.9602203 | 3.39673441 | 1.764148419 | 1.63339E-08 | 2.00417E-07 | - |
| *PANC* | 107.0398657 | 308.6689556 | 2.883682202 | 1.52791218 | 1.68321E-08 | 2.06007E-07 | K01918 |
| *EXPA1* | 0 | 143.1198753 | Inf | Inf | 1.68587E-08 | 2.06185E-07 | - |
| *At1g67340* | 19.59667587 | 92.88964333 | 4.740071426 | 2.244908798 | 1.69293E-08 | 2.06856E-07 | - |
| *At4g33300* | 19.74100763 | 294.2263187 | 14.90432121 | 3.897658767 | 1.71983E-08 | 2.09828E-07 | - |
| *CCX4* | 539.2441587 | 1307.811584 | 2.425267967 | 1.278144159 | 1.72044E-08 | 2.09828E-07 | K13754 |
| *GLOX1* | 0 | 22.46888066 | Inf | Inf | 1.7533E-08 | 2.13737E-07 | - |
| *At1g30090* | 18.76009155 | 98.09127898 | 5.228720697 | 2.386458008 | 1.82277E-08 | 2.21693E-07 | - |
| *AAP2* | 4.585829934 | 104.3817575 | 22.76180298 | 4.508542934 | 1.824E-08 | 2.21741E-07 | - |
| *NPF5.4* | 145.2964389 | 403.1964072 | 2.774991666 | 1.472483439 | 1.83451E-08 | 2.22915E-07 | K14638 |
| *endoub* | 68.38351624 | 215.6903086 | 3.154127201 | 1.657240843 | 1.87008E-08 | 2.27133E-07 | K14648 |
| *SSL3* | 104.8420097 | 298.718154 | 2.849221936 | 1.510568003 | 1.89019E-08 | 2.2947E-07 | - |
| *EXLA2* | 0 | 27.87181479 | Inf | Inf | 1.89682E-08 | 2.30169E-07 | - |
| *OPR1* | 71.39714384 | 615.4491738 | 8.62008115 | 3.107701451 | 1.96662E-08 | 2.382E-07 | K05894 |
| *SEC23* | 28.70111693 | 117.7555515 | 4.102821217 | 2.036616289 | 1.97203E-08 | 2.38745E-07 | - |
| *uncharacterized protein_03603* | 27.92985914 | 112.9728415 | 4.044876879 | 2.016095789 | 1.98167E-08 | 2.39802E-07 | K13116 |
| *APF1* | 211.903804 | 1035.067301 | 4.884609344 | 2.288243185 | 2.01143E-08 | 2.4318E-07 | - |
| *PIRL1* | 420.0182899 | 1024.856728 | 2.440028809 | 1.286898182 | 2.05726E-08 | 2.48607E-07 | - |
| *PBL27* | 958.7252592 | 2249.63113 | 2.346481548 | 1.230499116 | 2.08778E-08 | 2.51948E-07 | - |
| *JOX1* | 0.602700725 | 107.9192216 | 179.0593856 | 7.484294331 | 2.09162E-08 | 2.5227E-07 | - |
| *ZAT9* | 0.314161193 | 25.16067089 | 80.08841134 | 6.323521597 | 2.09607E-08 | 2.52602E-07 | - |
| *At1g67340* | 129.1628617 | 357.0887343 | 2.764639383 | 1.467091309 | 2.12E-08 | 2.55369E-07 | - |
| *GATL7* | 280.7007752 | 1836.04126 | 6.540919808 | 2.709493527 | 2.14471E-08 | 2.5811E-07 | - |
| *Os02g0642300* | 825.1845839 | 1955.013037 | 2.369182695 | 1.244389454 | 2.15366E-08 | 2.59068E-07 | K08360 |
| *MTH_273* | 0.301350363 | 29.90151045 | 99.22506871 | 6.632632751 | 2.16182E-08 | 2.59931E-07 | K06910 |
| *Os12g0580900* | 112.3883317 | 316.1290537 | 2.812828066 | 1.492021371 | 2.20357E-08 | 2.64709E-07 | - |
| *CRJ34* | 22.63756946 | 447.3477492 | 19.7612977 | 4.304605785 | 2.36563E-08 | 2.83367E-07 | - |
| *NAAT1* | 21.95763181 | 107.5259243 | 4.896972738 | 2.291890164 | 2.36731E-08 | 2.83367E-07 | K00815 |
| *At4g28440* | 468.7239522 | 1136.821615 | 2.42535422 | 1.278195466 | 2.37957E-08 | 2.84556E-07 | K07466 |
| *NSP1* | 217.7120353 | 561.5746969 | 2.579438 | 1.36705677 | 2.38531E-08 | 2.85112E-07 | - |
| *GLCAT14B* | 665.632914 | 2796.275133 | 4.200926778 | 2.07070764 | 2.49509E-08 | 2.97292E-07 | - |
| *HSP21.7* | 0.301350363 | 27.4450794 | 91.07365644 | 6.508961902 | 2.4951E-08 | 2.97292E-07 | K13993 |
| *ABCC14* | 375.7958099 | 1121.069694 | 2.983188381 | 1.576855083 | 2.53072E-08 | 3.00991E-07 | - |
| *MFP2* | 1007.98483 | 2364.258563 | 2.345529905 | 1.229913895 | 2.6131E-08 | 3.0981E-07 | K10527 |
| *APS2* | 985.1334787 | 5163.586471 | 5.241509483 | 2.389982348 | 2.80939E-08 | 3.32336E-07 | K13811 |
| *EMB3004* | 722.7230661 | 4067.834567 | 5.628483105 | 2.492746164 | 2.81756E-08 | 3.33003E-07 | K13832 |
| *At3g51250* | 84.77123738 | 251.5922008 | 2.967895817 | 1.569440449 | 2.88312E-08 | 3.40598E-07 | - |
| *RCS3* | 3425.552277 | 7785.510081 | 2.272775148 | 1.184454961 | 2.93019E-08 | 3.45849E-07 | K01738 |
| *EULS3* | 177.1801887 | 1479.822704 | 8.352077705 | 3.062135134 | 2.9984E-08 | 3.53426E-07 | - |
| *gtf2a1* | 293.9980081 | 719.8091502 | 2.448347031 | 1.291808062 | 3.02336E-08 | 3.56207E-07 | K03122 |
| *URED* | 147.2224791 | 520.9221681 | 3.538333081 | 1.823069863 | 3.05126E-08 | 3.58853E-07 | K03190 |
| *NIT4A* | 2332.315813 | 5328.804136 | 2.284769544 | 1.192048653 | 3.06443E-08 | 3.60241E-07 | K13035 |
| *APF1* | 20.60814656 | 99.32010316 | 4.819458308 | 2.268871001 | 3.11698E-08 | 3.65929E-07 | - |
| *GSTU19* | 373.7467553 | 911.0642836 | 2.437651353 | 1.285491798 | 3.13887E-08 | 3.68171E-07 | K00799 |
| *Os06g0717800* | 829.0326838 | 1949.457872 | 2.351484941 | 1.233572093 | 3.14555E-08 | 3.68791E-07 | K01102 |
| *NDL2* | 201.9093668 | 520.1090436 | 2.575953022 | 1.365106283 | 3.20691E-08 | 3.75483E-07 | - |
| *CSA* | 0.367048835 | 240.2703273 | 654.600435 | 9.354470751 | 3.46324E-08 | 4.0406E-07 | K00432 |
| *DSP1* | 471.6190895 | 1128.554674 | 2.392936798 | 1.258782293 | 3.68928E-08 | 4.29862E-07 | K18045 |
| *uncharacterized protein_07876* | 156.7396312 | 408.4396821 | 2.605848176 | 1.381753031 | 3.74339E-08 | 4.35588E-07 | - |
| *RFS1* | 517.555126 | 3631.611941 | 7.016860153 | 2.81082561 | 3.76138E-08 | 4.37489E-07 | K06617 |
| *AHRI* | 564.5482518 | 2142.646992 | 3.795330133 | 1.924225383 | 3.78879E-08 | 4.39965E-07 | K00053 |
| *uncharacterized protein_12040* | 177.690349 | 465.7943995 | 2.621382658 | 1.390327968 | 3.82162E-08 | 4.43126E-07 | - |
| *AAP4* | 222.3960106 | 1112.515955 | 5.002409676 | 2.322623213 | 3.86683E-08 | 4.48171E-07 | - |
| *VPS60-1* | 120.2410609 | 329.0428775 | 2.736526732 | 1.45234595 | 3.9116E-08 | 4.52962E-07 | K12198 |
| *uncharacterized protein_32904* | 19.49418922 | 89.57912537 | 4.595170609 | 2.200118427 | 3.94664E-08 | 4.56218E-07 | - |
| *ZIP4* | 154.382984 | 416.1726724 | 2.69571595 | 1.430668487 | 3.9913E-08 | 4.60975E-07 | K14709 |
| *CLINT1* | 102.1909657 | 907.489675 | 8.880331726 | 3.15061357 | 4.0548E-08 | 4.67695E-07 | K12471 |
| *Os08g0500300* | 126.8243628 | 346.6664207 | 2.733437118 | 1.450716187 | 4.26736E-08 | 4.90924E-07 | - |
| *uncharacterized protein_33679* | 4.664339237 | 115.4940512 | 24.76107447 | 4.630002014 | 4.32743E-08 | 4.97617E-07 | - |
| *SBP1* | 1655.491169 | 3744.103638 | 2.261627068 | 1.177361055 | 4.38738E-08 | 5.03851E-07 | K17285 |
| *RSH3* | 1911.906195 | 4364.712798 | 2.282911583 | 1.190874985 | 4.56109E-08 | 5.22434E-07 | - |
| *Dnajc2* | 334.0917178 | 809.3428957 | 2.422517089 | 1.276506843 | 4.6256E-08 | 5.29593E-07 | K09522 |
| *EPHX2* | 17.63484373 | 84.20194484 | 4.774748569 | 2.255424765 | 4.66585E-08 | 5.33969E-07 | - |
| *COAC2* | 173.2464344 | 454.9261027 | 2.625890132 | 1.392806555 | 4.90068E-08 | 5.59871E-07 | K15084 |
| *At3g04930* | 104.212539 | 291.4895751 | 2.797068164 | 1.483915413 | 4.97199E-08 | 5.67772E-07 | - |
| *SCPL50* | 117.6812413 | 371.3930309 | 3.155923806 | 1.658062375 | 5.05424E-08 | 5.76665E-07 | K09645 |
| *SK2* | 23.6859523 | 180.4302622 | 7.617606413 | 2.929337749 | 5.31567E-08 | 6.044E-07 | K00891 |
| *uncharacterized protein_29748* | 7.204894765 | 53.06959071 | 7.365769027 | 2.880836159 | 5.33307E-08 | 6.06116E-07 | - |
| *ERF9* | 7.33629171 | 52.90355736 | 7.211212346 | 2.850241825 | 5.35728E-08 | 6.08344E-07 | K00164 |
| *XXT2* | 2101.01471 | 5325.873537 | 2.534905401 | 1.341931909 | 5.42413E-08 | 6.15405E-07 | K08238 |
| *FKBP15-1* | 259.335454 | 640.4380498 | 2.469535267 | 1.304239572 | 5.4273E-08 | 6.15498E-07 | K09569 |
| *At5g13200* | 119.2037206 | 3527.649042 | 29.59344745 | 4.887205866 | 5.54034E-08 | 6.27508E-07 | - |
| *OTU4* | 543.559965 | 2765.532919 | 5.08781569 | 2.347046409 | 5.61378E-08 | 6.35553E-07 | K18342 |
| *At5g07050* | 76.02940812 | 227.6285054 | 2.993953406 | 1.582051769 | 5.6426E-08 | 6.38542E-07 | - |
| *KMS1* | 112.1242654 | 308.6990575 | 2.753186889 | 1.461102545 | 5.77916E-08 | 6.53434E-07 | - |
| *AVP* | 4450.606999 | 9912.944932 | 2.227324258 | 1.155311604 | 5.82196E-08 | 6.57991E-07 | K01507 |
| *uncharacterized protein_37013* | 118.0676782 | 320.5273941 | 2.714776803 | 1.440833591 | 5.82884E-08 | 6.58486E-07 | - |
| *PCO1* | 263.2700087 | 643.115027 | 2.442796391 | 1.288533619 | 5.85439E-08 | 6.61089E-07 | K10712 |
| *At1g75220* | 368.524201 | 875.2682799 | 2.375063232 | 1.247965923 | 6.01131E-08 | 6.77648E-07 | K08145 |
| *PMK* | 797.6309521 | 1836.98234 | 2.303047964 | 1.203544457 | 6.02434E-08 | 6.78826E-07 | K00938 |
| *ATG4B* | 496.3297278 | 1162.980709 | 2.343161499 | 1.228456394 | 6.09942E-08 | 6.86875E-07 | K08342 |
| *At3g53970* | 1713.870182 | 4420.751652 | 2.579397026 | 1.367033853 | 6.10098E-08 | 6.86875E-07 | K06700 |
| *UBC16* | 352.0535335 | 841.7257327 | 2.390902668 | 1.2575554 | 6.12585E-08 | 6.8938E-07 | K10688 |
| *XTH5* | 0 | 114.7999219 | Inf | Inf | 6.14896E-08 | 6.90802E-07 | K08235 |
| *CID12* | 307.7907896 | 812.375907 | 2.639376923 | 1.400197393 | 6.18708E-08 | 6.94492E-07 | - |
| *CYN* | 735.2118854 | 1688.833435 | 2.297070366 | 1.199795051 | 6.20753E-08 | 6.96491E-07 | K01725 |
| *uncharacterized protein_10660* | 2.869171871 | 37.32308723 | 13.00831351 | 3.701362028 | 6.31632E-08 | 7.08094E-07 | - |
| *HMA5* | 53.43014718 | 618.96952 | 11.58464935 | 3.534142472 | 6.35655E-08 | 7.12301E-07 | K17686 |
| *clptm1* | 1041.376189 | 2797.740395 | 2.686579956 | 1.425770775 | 6.46653E-08 | 7.24009E-07 | - |
| *At5g64460* | 61.38409068 | 194.284636 | 3.165064984 | 1.662235121 | 6.6278E-08 | 7.41751E-07 | - |
| *uncharacterized protein_10512* | 1.769545704 | 31.13711956 | 17.59610927 | 4.13718456 | 6.78767E-08 | 7.58677E-07 | - |
| *TIFY10A* | 2290.93935 | 5839.522815 | 2.548964387 | 1.349911217 | 6.81045E-08 | 7.60577E-07 | K13464 |
| *FBA3* | 5276.334701 | 11789.30683 | 2.234374332 | 1.159870906 | 6.87311E-08 | 7.66925E-07 | K01623 |
| *GALM* | 129.0567145 | 488.7034359 | 3.786733901 | 1.920954045 | 6.97391E-08 | 7.77514E-07 | K01785 |
| *uncharacterized protein_08975* | 198.522609 | 626.137005 | 3.153983358 | 1.657175048 | 6.99924E-08 | 7.80008E-07 | - |
| *TIM17-2* | 426.3999071 | 1005.537052 | 2.358201855 | 1.237687214 | 7.09473E-08 | 7.90316E-07 | K17795 |
| *VHA-a2* | 766.1040287 | 1759.407254 | 2.296564419 | 1.199477252 | 7.30569E-08 | 8.12442E-07 | K02154 |
| *DCR* | 0.602700725 | 47.10113784 | 78.15012634 | 6.2881763 | 7.39026E-08 | 8.215E-07 | K19747 |
| *UGT85A24* | 80.00493564 | 1079.500949 | 13.49292941 | 3.754131696 | 7.61829E-08 | 8.45778E-07 | - |
| *chi1* | 181.9810578 | 5884.262693 | 32.33447901 | 5.01500146 | 7.94916E-08 | 8.81768E-07 | K20547 |
| *AOX1A* | 176.1532906 | 1112.687605 | 6.316587112 | 2.659145273 | 8.01077E-08 | 8.87855E-07 | K17893 |
| *CYP94B1* | 0.995371222 | 27.49333047 | 27.62118279 | 4.787703194 | 8.04263E-08 | 8.91011E-07 | - |
| *ROQ1* | 0.92967275 | 27.53195122 | 29.61466949 | 4.88824008 | 8.10346E-08 | 8.97373E-07 | K19613 |
| *uncharacterized protein_30688* | 175.1128813 | 547.6842514 | 3.127606874 | 1.645059184 | 8.13752E-08 | 9.00388E-07 | - |
| *SPL15* | 678.0708207 | 1574.884438 | 2.322595797 | 1.215738103 | 8.2885E-08 | 9.16325E-07 | - |
| *uncharacterized protein_13083* | 258.1727981 | 627.1140483 | 2.429047726 | 1.280390837 | 8.44425E-08 | 9.3237E-07 | K19525 |
| *TIM23-2* | 152.1836077 | 597.5668358 | 3.926617622 | 1.973287114 | 8.46557E-08 | 9.34332E-07 | K17794 |
| *uncharacterized protein_25742* | 155.3666646 | 400.3676799 | 2.576921382 | 1.365648523 | 8.65282E-08 | 9.538E-07 | - |
| *uncharacterized protein_17816* | 3.995940039 | 40.34987614 | 10.09771812 | 3.335957405 | 8.71661E-08 | 9.60029E-07 | - |
| *PAO2* | 160.0222819 | 419.455015 | 2.621228806 | 1.390243291 | 9.08397E-08 | 9.98565E-07 | K17839 |
| *APM1* | 698.1163759 | 2107.971634 | 3.019513232 | 1.594315995 | 9.08544E-08 | 9.98565E-07 | K08776 |
| *ostc-b* | 327.1770068 | 779.2924892 | 2.381868141 | 1.252093549 | 9.31646E-08 | 1.02268E-06 | - |
| *PAZX* | 87.68988427 | 248.401597 | 2.832728074 | 1.502192118 | 9.46921E-08 | 1.03858E-06 | K13963 |
| *RR23* | 460.7886529 | 1068.043954 | 2.317860796 | 1.212793925 | 9.55284E-08 | 1.04732E-06 | K14491 |
| *AOX1A* | 34.29348459 | 325.0525157 | 9.478550213 | 3.244666409 | 9.66183E-08 | 1.05838E-06 | K17893 |
| *SYT1* | 51.07400022 | 351.0855459 | 6.874056162 | 2.781161639 | 1.00099E-07 | 1.09515E-06 | - |
| *ABCD1* | 18.24871096 | 87.517103 | 4.795796436 | 2.261770423 | 1.03021E-07 | 1.12572E-06 | K05677 |
| *AAP2* | 0.995371222 | 27.04378633 | 27.16954813 | 4.763918667 | 1.03685E-07 | 1.1325E-06 | - |
| *THE1* | 636.6465461 | 2561.117214 | 4.022824328 | 2.008208739 | 1.1066E-07 | 1.20669E-06 | - |
| *PR4B* | 32.22435683 | 309.0142938 | 9.589463506 | 3.261450104 | 1.12912E-07 | 1.23073E-06 | - |
| *uncharacterized protein_43301* | 321.0600758 | 758.4129732 | 2.362215144 | 1.240140367 | 1.13147E-07 | 1.23227E-06 | - |
| *maoI* | 336.5659744 | 1045.278421 | 3.105716265 | 1.634926033 | 1.14072E-07 | 1.24183E-06 | K00276 |
| *SEI1* | 129.1338274 | 338.481363 | 2.621167278 | 1.390209427 | 1.14324E-07 | 1.24304E-06 | K19365 |
| *URA6* | 67.69442833 | 202.3973973 | 2.989867885 | 1.580081737 | 1.14779E-07 | 1.24695E-06 | K13800 |
| *TOL1* | 739.099962 | 1683.123151 | 2.277260503 | 1.187299335 | 1.18498E-07 | 1.28524E-06 | - |
| *At5g03905* | 207.4875273 | 514.0479415 | 2.477488397 | 1.308878301 | 1.19318E-07 | 1.29361E-06 | - |
| *uncharacterized protein_14775* | 0 | 27.62226524 | Inf | Inf | 1.19656E-07 | 1.29673E-06 | - |
| *uncharacterized protein_24158* | 51.10443085 | 162.6657322 | 3.183006433 | 1.670390073 | 1.2278E-07 | 1.32894E-06 | - |
| *PSS1* | 481.1104002 | 1108.530974 | 2.304109356 | 1.204209191 | 1.24312E-07 | 1.34333E-06 | K08730 |
| *CYP716B2* | 14.64708574 | 72.77734241 | 4.96872509 | 2.312875723 | 1.27637E-07 | 1.37587E-06 | - |
| *exgA* | 14.86980282 | 1200.553462 | 80.73768538 | 6.335170323 | 1.29825E-07 | 1.39888E-06 | - |
| *UCC1* | 7.910206117 | 66.2387435 | 8.373832808 | 3.065888114 | 1.30851E-07 | 1.40879E-06 | - |
| *PAD4* | 52.97038185 | 484.136249 | 9.139753804 | 3.192155304 | 1.32814E-07 | 1.427E-06 | - |
| *B0103C08-B0602B01.13* | 294.5589833 | 705.5774635 | 2.395369021 | 1.260247929 | 1.34065E-07 | 1.43869E-06 | K11450 |
| *ARR1* | 492.3908883 | 1139.232666 | 2.313675361 | 1.21018645 | 1.35759E-07 | 1.45628E-06 | K14491 |
| *ARF3* | 208.225938 | 567.9015262 | 2.727333259 | 1.447490997 | 1.37791E-07 | 1.47747E-06 | K07937 |
| *OTU9* | 44.67358659 | 145.9108706 | 3.266155278 | 1.70759338 | 1.39318E-07 | 1.49142E-06 | - |
| *CBSX6* | 69.85323192 | 204.7523618 | 2.931179506 | 1.551481322 | 1.39453E-07 | 1.49226E-06 | - |
| *At4g19900* | 0 | 28.65474885 | Inf | Inf | 1.40163E-07 | 1.49924E-06 | K01988 |
| *WIN2* | 134.6460415 | 561.4372017 | 4.169726756 | 2.059952846 | 1.4154E-07 | 1.51213E-06 | K17506 |
| *ARF* | 1908.727512 | 4202.751331 | 2.2018603 | 1.138722938 | 1.42434E-07 | 1.52107E-06 | K07937 |
| *PDIL1-5* | 179.9231009 | 453.8480946 | 2.52245594 | 1.33482907 | 1.44495E-07 | 1.54183E-06 | K09580 |
| *At4g24290* | 747.3476935 | 1690.42103 | 2.261893688 | 1.177531122 | 1.4516E-07 | 1.54829E-06 | - |
| *IFRD2* | 8.54333748 | 59.17497415 | 6.926446987 | 2.792115492 | 1.47825E-07 | 1.57545E-06 | - |
| *DTC* | 713.0236869 | 1604.611993 | 2.250432942 | 1.170202576 | 1.49439E-07 | 1.59201E-06 | K15104 |
| *RK1* | 73.74023201 | 214.2708476 | 2.905752283 | 1.538911718 | 1.50827E-07 | 1.60549E-06 | - |
| *At4g33300* | 1.610882779 | 84.57840966 | 52.50438504 | 5.714366013 | 1.51903E-07 | 1.61629E-06 | - |
| *uncharacterized protein_22155* | 215.6767508 | 528.7001404 | 2.451354347 | 1.293579043 | 1.52466E-07 | 1.62163E-06 | - |
| *TOP6B* | 419.7987375 | 965.9948258 | 2.301090355 | 1.202317634 | 1.53931E-07 | 1.63654E-06 | - |
| *OFUT20* | 7.743541337 | 52.60392972 | 6.793265179 | 2.764105172 | 1.55913E-07 | 1.65695E-06 | - |
| *PBL27* | 111.5522433 | 299.0804542 | 2.681079693 | 1.422814103 | 1.59306E-07 | 1.69097E-06 | - |
| *PAP23* | 98.07051041 | 271.3200218 | 2.766581112 | 1.468104222 | 1.60973E-07 | 1.70798E-06 | - |
| *PCK2* | 10.76821148 | 69.88079861 | 6.489545526 | 2.698117447 | 1.64119E-07 | 1.73995E-06 | K01610 |
| *GN* | 676.2313635 | 1527.359748 | 2.258634885 | 1.175451074 | 1.66681E-07 | 1.76499E-06 | K18443 |
| *Fra a 1.07* | 0 | 28.84155213 | Inf | Inf | 1.67524E-07 | 1.77177E-06 | - |
| *VIT1* | 43.78411463 | 253.7799819 | 5.796165666 | 2.535098831 | 1.70867E-07 | 1.80568E-06 | - |
| *ORP1A* | 1549.257577 | 4958.171201 | 3.200353044 | 1.678231063 | 1.76757E-07 | 1.86446E-06 | K20456 |
| *SAP* | 0 | 19.36950977 | Inf | Inf | 1.85026E-07 | 1.94595E-06 | - |
| *Os07g0679700* | 447.8125348 | 1022.685639 | 2.283736071 | 1.19139593 | 1.8761E-07 | 1.97234E-06 | - |
| *At5g48480* | 1016.082503 | 2218.641972 | 2.183525418 | 1.126659325 | 1.92177E-07 | 2.01874E-06 | - |
| *uncharacterized protein_30038* | 478.0845817 | 1086.916538 | 2.27348168 | 1.184903379 | 1.94249E-07 | 2.03969E-06 | - |
| *ERF017* | 2.109452539 | 32.53694146 | 15.42435341 | 3.947138107 | 1.95994E-07 | 2.0572E-06 | - |
| *uncharacterized protein_34199* | 2.81628423 | 35.22406574 | 12.50728366 | 3.644696593 | 1.97335E-07 | 2.0688E-06 | - |
| *uncharacterized protein_34483* | 3.837277113 | 175.6302631 | 45.76950215 | 5.516314694 | 1.97909E-07 | 2.07317E-06 | - |
| *At3g20240* | 37.30698822 | 129.4620366 | 3.470181936 | 1.795011303 | 1.98831E-07 | 2.082E-06 | K14684 |
| *STK11IP* | 491.379794 | 1115.790284 | 2.27072887 | 1.183155455 | 1.99008E-07 | 2.08302E-06 | - |
| *NTF2* | 827.7406472 | 1845.917514 | 2.230067497 | 1.157087377 | 2.02716E-07 | 2.12015E-06 | K17265 |
| *RPS7* | 186.3551611 | 456.6672781 | 2.450521227 | 1.293088644 | 2.03623E-07 | 2.12879E-06 | K02993 |
| *uncharacterized protein_22493* | 226.4321796 | 550.5301267 | 2.431324592 | 1.281742512 | 2.04568E-07 | 2.13782E-06 | - |
| *MRD1* | 66.19543041 | 194.2380066 | 2.934311407 | 1.553021987 | 2.05166E-07 | 2.14322E-06 | K14787 |
| *KCS11* | 392.1246622 | 1306.473697 | 3.331781505 | 1.736293793 | 2.07625E-07 | 2.16757E-06 | K15397 |
| *CXE15* | 0 | 20.89988806 | Inf | Inf | 2.07661E-07 | 2.16757E-06 | - |
| *UBI11* | 139.2165158 | 359.4699563 | 2.582092751 | 1.368540824 | 2.08176E-07 | 2.17209E-06 | K08770 |
| *GBP1* | 103.345524 | 379.8314568 | 3.675354694 | 1.877883486 | 2.08475E-07 | 2.17434E-06 | - |
| *PUB34* | 234.8197709 | 565.8601456 | 2.409763639 | 1.268891647 | 2.14068E-07 | 2.23059E-06 | - |
| *At4g12770* | 1882.628119 | 4135.159391 | 2.196482327 | 1.135194892 | 2.14122E-07 | 2.23059E-06 | - |
| *BIOF* | 37.61454392 | 165.2920431 | 4.394365207 | 2.135654774 | 2.22265E-07 | 2.31177E-06 | K00652 |
| *GAPN* | 1284.931579 | 2829.648655 | 2.202178467 | 1.138931391 | 2.3407E-07 | 2.42975E-06 | K00131 |
| *LEA34* | 0 | 19.10049419 | Inf | Inf | 2.34424E-07 | 2.43248E-06 | - |
| *APCB1* | 7.79478466 | 51.66490392 | 6.628137424 | 2.728603515 | 2.35005E-07 | 2.43754E-06 | - |
| *SIS8* | 32.29157564 | 115.652876 | 3.581518513 | 1.840571399 | 2.35324E-07 | 2.43989E-06 | - |
| *GALT29A* | 603.6293467 | 1352.260804 | 2.240217133 | 1.163638572 | 2.36386E-07 | 2.44994E-06 | - |
| *A36* | 208.6894879 | 501.6787328 | 2.403948267 | 1.265405849 | 2.4462E-07 | 2.5313E-06 | - |
| *uncharacterized protein_51258* | 70.79583948 | 204.3223896 | 2.886079057 | 1.529110819 | 2.46816E-07 | 2.55133E-06 | - |
| *APS1* | 22.41776908 | 90.03992146 | 4.01645325 | 2.005922084 | 2.46847E-07 | 2.55133E-06 | - |
| *RSL1* | 133.7323442 | 339.4514982 | 2.538290196 | 1.343857018 | 2.48997E-07 | 2.57154E-06 | K11975 |
| *PAO* | 3087.739967 | 6626.177798 | 2.145963672 | 1.101625654 | 2.54676E-07 | 2.6271E-06 | K13071 |
| *ZHD1* | 399.6087309 | 905.3983582 | 2.265712154 | 1.179964586 | 2.58624E-07 | 2.66573E-06 | - |
| *Os12g0628600* | 1.519562644 | 61.24358252 | 40.30342727 | 5.332830621 | 2.60086E-07 | 2.67871E-06 | - |
| *RID3* | 200.6097285 | 487.2593955 | 2.428892153 | 1.280298433 | 2.68111E-07 | 2.75705E-06 | K14829 |
| *GALT1* | 44.7361204 | 143.7505384 | 3.213299167 | 1.684055306 | 2.69232E-07 | 2.7675E-06 | K14413 |
| *BZIP44* | 39.25765386 | 220.8504044 | 5.625664877 | 2.492023613 | 2.74954E-07 | 2.82301E-06 | - |
| *uncharacterized protein_36665* | 196.0842922 | 474.902857 | 2.421932179 | 1.276158466 | 2.76261E-07 | 2.83533E-06 | - |
| *UBP5* | 19.49583354 | 83.20148954 | 4.267654899 | 2.093443518 | 2.81919E-07 | 2.88666E-06 | K11835 |
| *LAC3* | 0.615511556 | 23.64650891 | 38.41765223 | 5.263697451 | 2.82836E-07 | 2.89493E-06 | K05909 |
| *uncharacterized protein_10321* | 38.84252635 | 130.670078 | 3.364098329 | 1.750219875 | 2.84413E-07 | 2.90994E-06 | - |
| *uncharacterized protein_35706* | 115.8123736 | 299.7555154 | 2.588285743 | 1.371996898 | 2.91008E-07 | 2.97049E-06 | - |
| *ABCC14* | 46.11147527 | 146.5423596 | 3.178001977 | 1.668120022 | 2.9624E-07 | 3.02038E-06 | - |
| *RH14* | 2426.038236 | 5226.92616 | 2.154511039 | 1.10736049 | 2.97784E-07 | 3.03379E-06 | K12823 |
| *uncharacterized protein_27386* | 194.0103554 | 571.5029946 | 2.945734486 | 1.558627399 | 3.01041E-07 | 3.0646E-06 | K15891 |
| *uncharacterized protein_41869* | 0 | 18.73802081 | Inf | Inf | 3.07462E-07 | 3.12513E-06 | - |
| *CRL1* | 0.301350363 | 24.62253384 | 81.707331 | 6.352393622 | 3.11767E-07 | 3.16645E-06 | - |
| *GLOX1* | 0 | 41.44834286 | Inf | Inf | 3.24272E-07 | 3.28838E-06 | - |
| *uncharacterized protein_15757* | 38.65343279 | 356.3916924 | 9.220182184 | 3.204795257 | 3.26075E-07 | 3.3054E-06 | - |
| *ABCG36* | 7.844507644 | 154.9221491 | 19.74912335 | 4.303716709 | 3.29533E-07 | 3.3366E-06 | - |
| *SUVH1* | 1134.277185 | 2461.289806 | 2.169919169 | 1.117641302 | 3.32985E-07 | 3.36767E-06 | K11420 |
| *At1g16350* | 1959.691278 | 4224.291123 | 2.155590102 | 1.108082867 | 3.33147E-07 | 3.36802E-06 | K00088 |
| *BCE2* | 14.83937218 | 70.84263317 | 4.77396431 | 2.255187781 | 3.35635E-07 | 3.39186E-06 | K09699 |
| *At1g21890* | 903.4438316 | 2279.445517 | 2.523062793 | 1.335176112 | 3.37384E-07 | 3.40693E-06 | - |
| *CIA1* | 465.3730821 | 1051.596506 | 2.259684856 | 1.176121583 | 3.40302E-07 | 3.43246E-06 | - |
| *TSJT1* | 8.895931166 | 145.0390388 | 16.30397494 | 4.027151834 | 3.46258E-07 | 3.49119E-06 | - |
| *PUB23* | 1.101146506 | 67.02795871 | 60.87106334 | 5.927684663 | 3.5097E-07 | 3.53697E-06 | - |
| *STY13* | 547.0371751 | 1217.474565 | 2.225579212 | 1.154180849 | 3.55815E-07 | 3.57934E-06 | - |
| *PTPA* | 747.1519945 | 1641.299575 | 2.196741208 | 1.13536492 | 3.57906E-07 | 3.599E-06 | K17605 |
| *SMG7* | 1941.522482 | 4138.408553 | 2.131527496 | 1.091887666 | 3.62812E-07 | 3.64555E-06 | K14409 |
| *ICI1* | 235.0056998 | 554.5930319 | 2.35991311 | 1.238733742 | 3.63966E-07 | 3.65575E-06 | - |
| *NFXL2* | 43.022627 | 137.3181521 | 3.191765861 | 1.674354823 | 3.64511E-07 | 3.65983E-06 | K15683 |
| *DMP4* | 0.628322387 | 34.70701633 | 55.23759308 | 5.787578552 | 3.66141E-07 | 3.6748E-06 | - |
| *At1g01350* | 153.1032623 | 400.9781614 | 2.619004685 | 1.389018641 | 3.70336E-07 | 3.71548E-06 | K13127 |
| *APY1* | 325.9374816 | 1838.75757 | 5.641442529 | 2.49606411 | 3.71256E-07 | 3.7233E-06 | K14641 |
| *uncharacterized protein_29023* | 0 | 18.48998698 | Inf | Inf | 3.86898E-07 | 3.87427E-06 | - |
| *TPS5* | 1248.747431 | 2681.134085 | 2.147058739 | 1.102361661 | 3.87214E-07 | 3.87596E-06 | K16055 |
| *FLS* | 50.86242143 | 154.5755679 | 3.039091799 | 1.603640254 | 3.92067E-07 | 3.92008E-06 | - |
| *TSJT1* | 9.734283782 | 326.0645737 | 33.49651407 | 5.065939059 | 3.95416E-07 | 3.95206E-06 | - |
| *ZHD1* | 472.456794 | 1052.249828 | 2.227187421 | 1.155222968 | 3.96221E-07 | 3.95788E-06 | - |
| *CREG1* | 187.114069 | 448.1552433 | 2.39509111 | 1.260080538 | 3.9632E-07 | 3.95788E-06 | - |
| *AAT* | 658.0214288 | 2233.797973 | 3.394719191 | 1.763292241 | 3.96449E-07 | 3.95788E-06 | K15849 |
| *At5g60370* | 5.842474708 | 44.31113725 | 7.584309641 | 2.923017866 | 3.97131E-07 | 3.96319E-06 | K17815 |
| *uncharacterized protein_16959* | 8.815901524 | 260.4357076 | 29.54158538 | 4.884675347 | 3.9973E-07 | 3.9846E-06 | - |
| *TIFY10A* | 0 | 18.36115168 | Inf | Inf | 4.05604E-07 | 4.04163E-06 | K13464 |
| *ERI2* | 551.7440119 | 1213.439868 | 2.19928054 | 1.137031646 | 4.10379E-07 | 4.08612E-06 | K18417 |
| *NSN1* | 451.9583877 | 1014.432972 | 2.244527372 | 1.16641169 | 4.10774E-07 | 4.08851E-06 | K14538 |
| *LHT1* | 69.35478613 | 500.0862931 | 7.210552018 | 2.850109712 | 4.19364E-07 | 4.17243E-06 | - |
| *ACT2* | 84.54166684 | 543.1281548 | 6.424384273 | 2.68355819 | 4.35439E-07 | 4.31607E-06 | K10355 |
| *GLYI4* | 174.5454485 | 625.009092 | 3.580781381 | 1.84027444 | 4.40031E-07 | 4.35667E-06 | - |
| *At1g32860* | 18.24238165 | 80.06674116 | 4.389050877 | 2.133908994 | 4.41345E-07 | 4.3664E-06 | - |
| *JOX2* | 619.8186719 | 1402.549373 | 2.262838208 | 1.178133436 | 4.45657E-07 | 4.4041E-06 | - |
| *uncharacterized protein_06380* | 47.23697106 | 148.6621574 | 3.147156858 | 1.654049086 | 4.4861E-07 | 4.43162E-06 | - |
| *XYL1* | 121.4802099 | 308.3781433 | 2.53850519 | 1.34397921 | 4.52396E-07 | 4.46568E-06 | K15925 |
| *ATG13A* | 387.8125165 | 872.5526146 | 2.249934124 | 1.169882761 | 4.55462E-07 | 4.49426E-06 | K08331 |
| *BEBT1* | 0.668399198 | 80.02013105 | 119.719071 | 6.903509179 | 4.63485E-07 | 4.56488E-06 | K19861 |
| *At5g54830* | 279.1150138 | 924.3529849 | 3.311727923 | 1.727584152 | 4.70278E-07 | 4.62832E-06 | - |
| *PLD1* | 78.06035136 | 218.0114307 | 2.79285741 | 1.481741919 | 4.77545E-07 | 4.69634E-06 | K01115 |
| *ADH1* | 21.79592821 | 87.82312412 | 4.029336273 | 2.010542212 | 4.86477E-07 | 4.77705E-06 | K18857 |
| *Os04g0338000* | 105.0172681 | 271.9301054 | 2.589384683 | 1.37260931 | 4.98796E-07 | 4.88709E-06 | - |
| *CT1* | 84.73587378 | 244.9945032 | 2.891272518 | 1.531704597 | 5.01724E-07 | 4.91395E-06 | K00967 |
| *PGLR* | 0.301350363 | 30.66365527 | 101.7541674 | 6.668944073 | 5.08375E-07 | 4.97539E-06 | K01213 |
| *AGLU* | 24.10247616 | 92.95866432 | 3.856809719 | 1.94740797 | 5.18769E-07 | 5.07524E-06 | K01187 |
| *CR4* | 108.9895069 | 284.9063855 | 2.614071699 | 1.386298712 | 5.25503E-07 | 5.13907E-06 | - |
| *uncharacterized protein_19790* | 0 | 18.02756926 | Inf | Inf | 5.26895E-07 | 5.14709E-06 | - |
| *NHX1* | 10.40103866 | 57.13544923 | 5.493244578 | 2.457658527 | 5.27269E-07 | 5.14884E-06 | - |
| *GRDP2* | 60.57275609 | 1543.430736 | 25.48060937 | 4.671327875 | 5.28782E-07 | 5.16169E-06 | - |
| *CYP98A2* | 1899.288388 | 5619.959472 | 2.958981641 | 1.565100745 | 5.308E-07 | 5.17756E-06 | K09754 |
| *CPL4* | 15.48531437 | 70.28199865 | 4.538622656 | 2.182254547 | 5.37009E-07 | 5.23232E-06 | K18999 |
| *SB09* | 67.75038487 | 192.7554815 | 2.845083195 | 1.50847084 | 5.4132E-07 | 5.27042E-06 | K03671 |
| *GRMZM2G118515* | 154.0629938 | 596.2881389 | 3.870417706 | 1.952489274 | 5.45568E-07 | 5.30982E-06 | K15108 |
| *At4g39280* | 432.0561767 | 958.0872546 | 2.217506209 | 1.148938144 | 5.55776E-07 | 5.40717E-06 | K01889 |
| *WRKY6* | 241.659757 | 3653.816755 | 15.11967404 | 3.918355132 | 5.73415E-07 | 5.5685E-06 | - |
| *MGLL* | 292.3488407 | 840.0010241 | 2.87328324 | 1.522700217 | 5.7838E-07 | 5.61126E-06 | - |
| *P4H3* | 160.4158247 | 390.2238132 | 2.432576799 | 1.282485353 | 5.78456E-07 | 5.61126E-06 | K00472 |
| *TOP3A* | 30.13087978 | 106.8705088 | 3.54687648 | 1.826549089 | 5.79821E-07 | 5.62243E-06 | K03165 |
| *MTP5* | 106.1888263 | 274.2610632 | 2.58276763 | 1.368917851 | 5.80687E-07 | 5.62875E-06 | - |
| *GATL3* | 19.33400596 | 81.78069235 | 4.229888649 | 2.080619685 | 5.93992E-07 | 5.7535E-06 | - |
| *MES1* | 140.6990706 | 3735.373795 | 26.54867428 | 4.730567916 | 5.95105E-07 | 5.76128E-06 | - |
| *YSL12* | 973.7130871 | 2084.540573 | 2.140816017 | 1.098160815 | 5.95233E-07 | 5.76128E-06 | - |
| *ZIFL2* | 15.78362406 | 72.6646304 | 4.603798857 | 2.202824802 | 5.96239E-07 | 5.7689E-06 | - |
| *ACX4* | 505.0026657 | 1100.610457 | 2.179415143 | 1.123941032 | 6.04589E-07 | 5.84325E-06 | K00232 |
| *Os02g0194200* | 903.1300185 | 1920.180481 | 2.126139583 | 1.088236314 | 6.08858E-07 | 5.8802E-06 | - |
| *At4g26910* | 305.0130336 | 695.2716697 | 2.279481836 | 1.188705913 | 6.11495E-07 | 5.89918E-06 | K00658 |
| *GPX4* | 648.4896061 | 1410.883252 | 2.175645128 | 1.121443256 | 6.20922E-07 | 5.97894E-06 | K00432 |
| *PGL3* | 29.77512143 | 113.2062935 | 3.80204305 | 1.926774867 | 6.21124E-07 | 5.97894E-06 | - |
| *LHY* | 141.5835813 | 349.0564385 | 2.465373705 | 1.301806349 | 6.29243E-07 | 6.05268E-06 | K12133 |
| *VIR* | 86.99179832 | 236.4077481 | 2.717586631 | 1.442326026 | 6.29519E-07 | 6.05312E-06 | - |
| *APSR1* | 38.25747366 | 172.09793 | 4.498413345 | 2.169416232 | 6.29934E-07 | 6.0549E-06 | - |
| *IAA11* | 65.6074328 | 1216.54609 | 18.54280891 | 4.212787899 | 6.42317E-07 | 6.16943E-06 | K14484 |
| *X13* | 1410.685826 | 2975.055229 | 2.108942454 | 1.07651973 | 6.78687E-07 | 6.5069E-06 | K13344 |
| *ELM1* | 236.7512681 | 582.2551694 | 2.459353963 | 1.29827939 | 6.82509E-07 | 6.53879E-06 | - |
| *At4g33300* | 0.668399198 | 22.35119784 | 33.43989326 | 5.063498337 | 6.96917E-07 | 6.67198E-06 | - |
| *SPBC119.09c* | 458.6375989 | 1013.008501 | 2.208734094 | 1.143219745 | 7.00148E-07 | 6.70048E-06 | - |
| *Os07g0555200* | 60.19097592 | 170.3802447 | 2.830660943 | 1.501138954 | 7.03728E-07 | 6.73229E-06 | K03260 |
| *VAMP711* | 261.6001569 | 597.3164325 | 2.283318327 | 1.191132006 | 7.06644E-07 | 6.75774E-06 | K08515 |
| *MYB2* | 0 | 17.60072796 | Inf | Inf | 7.08817E-07 | 6.77605E-06 | K09422 |
| *SLAC1* | 105.1759592 | 272.4105922 | 2.590046187 | 1.372977825 | 7.19581E-07 | 6.87646E-06 | - |
| *YSL14* | 682.9404813 | 1471.43503 | 2.154558224 | 1.107392086 | 7.36523E-07 | 7.02563E-06 | - |
| *ECA4* | 1970.894995 | 4150.115523 | 2.10570098 | 1.074300581 | 7.40874E-07 | 7.06457E-06 | K01537 |
| *eif3J* | 48.39100521 | 148.5767212 | 3.070337567 | 1.618397281 | 7.41419E-07 | 7.06721E-06 | K03245 |
| *MUB3* | 165.8474849 | 393.3180945 | 2.371565024 | 1.245839425 | 7.41873E-07 | 7.06898E-06 | - |
| *GAPCP1* | 1181.235878 | 18647.10637 | 15.78609888 | 3.980582786 | 7.44377E-07 | 7.09028E-06 | K00134 |
| *SEC* | 2387.352891 | 5014.314008 | 2.10036565 | 1.070640507 | 7.4621E-07 | 7.10517E-06 | K09667 |
| *VATL* | 301.2118965 | 678.2406291 | 2.251705981 | 1.171018458 | 7.53016E-07 | 7.16738E-06 | K02155 |
| *uncharacterized protein_50193* | 382.3575878 | 851.1027665 | 2.22593403 | 1.154410836 | 7.6354E-07 | 7.26231E-06 | - |
| *JOX2* | 175.3865938 | 1485.736788 | 8.471210688 | 3.082568171 | 7.64833E-07 | 7.27198E-06 | K05278 |
| *At1g18900* | 184.6996687 | 438.5942037 | 2.37463449 | 1.247705467 | 7.73951E-07 | 7.34807E-06 | - |
| *SSL5* | 227.1836491 | 526.5032936 | 2.31752283 | 1.212583551 | 7.97731E-07 | 7.56023E-06 | - |
| *SAT1* | 98.17892621 | 460.5041323 | 4.690458024 | 2.229728809 | 8.15714E-07 | 7.72233E-06 | K00640 |
| *uncharacterized protein_49529* | 752.7922493 | 1615.939413 | 2.146594116 | 1.102049428 | 8.17362E-07 | 7.73515E-06 | - |
| *BZIP1-A* | 84.71834974 | 224.9847737 | 2.655679371 | 1.409080976 | 8.25182E-07 | 7.80635E-06 | K09060 |
| *arl5* | 289.1199455 | 650.8408366 | 2.251110125 | 1.170636636 | 8.34664E-07 | 7.89039E-06 | K07950 |
| *ROC8* | 125.1382876 | 313.9489506 | 2.508816101 | 1.327006723 | 8.42285E-07 | 7.95388E-06 | K09338 |
| *MYB308* | 0 | 17.50575443 | Inf | Inf | 8.43939E-07 | 7.96664E-06 | K09422 |
| *TUR2* | 1726.915742 | 3857.260473 | 2.233612434 | 1.159378878 | 8.69569E-07 | 8.19684E-06 | - |
| *At1g54290* | 8471.510163 | 17284.186 | 2.04027212 | 1.028761584 | 8.70154E-07 | 8.19942E-06 | K03113 |
| *HCT* | 2.80182908 | 32.26477565 | 11.51561167 | 3.525519139 | 9.16144E-07 | 8.61431E-06 | K13065 |
| *RIC1* | 316.0322808 | 709.4825643 | 2.244968655 | 1.166695302 | 9.1869E-07 | 8.63208E-06 | K07874 |
| *uncharacterized protein_16434* | 279.4097912 | 629.5496816 | 2.25314109 | 1.171937657 | 9.19421E-07 | 8.63587E-06 | K09705 |
| *CYP750A1* | 0 | 208.5575964 | Inf | Inf | 9.22122E-07 | 8.65816E-06 | - |
| *SUD1* | 687.5848689 | 1472.546725 | 2.141621772 | 1.09870371 | 9.23285E-07 | 8.66599E-06 | K10661 |
| *ERF016* | 41.38168552 | 411.0933268 | 9.934185173 | 3.312401639 | 9.28678E-07 | 8.71351E-06 | - |
| *SPRING* | 54.79762417 | 161.0274202 | 2.938583974 | 1.555121126 | 9.59515E-07 | 8.9916E-06 | - |
| *uncharacterized protein_48119* | 587.8361047 | 1263.84995 | 2.150003954 | 1.104339313 | 9.59682E-07 | 8.9916E-06 | - |
| *BRM* | 155.2776369 | 376.4742615 | 2.424523383 | 1.277701168 | 9.85192E-07 | 9.2077E-06 | K11647 |
| *MAPKKK20* | 0.94248358 | 255.3648703 | 270.9488797 | 8.081876872 | 1.01135E-06 | 9.43207E-06 | - |
| *At1g01540* | 568.0207117 | 1231.174422 | 2.167481566 | 1.116019723 | 1.02092E-06 | 9.51462E-06 | - |
| *TUR2* | 81.66842988 | 1858.60757 | 22.75796869 | 4.508299888 | 1.0293E-06 | 9.58254E-06 | - |
| *WRKY35* | 189.6793175 | 589.3473388 | 3.107072223 | 1.635555777 | 1.04323E-06 | 9.69167E-06 | - |
| *RFS2* | 38.84265033 | 305.7422234 | 7.871301798 | 2.976602256 | 1.05082E-06 | 9.7588E-06 | K06617 |
| *SRG1* | 10.15422026 | 61.81536952 | 6.087653009 | 2.605886129 | 1.05592E-06 | 9.79923E-06 | - |
| *ERF2* | 1.835244177 | 310.4814735 | 169.1772013 | 7.402391351 | 1.06183E-06 | 9.84719E-06 | K09286 |
| *TAR2* | 254.5881685 | 576.9963144 | 2.266390924 | 1.18039673 | 1.08722E-06 | 1.00614E-05 | K16903 |
| *UGT72B1* | 150.9349648 | 363.7020213 | 2.409660491 | 1.268829892 | 1.08796E-06 | 1.00647E-05 | K08237 |
| *At3g03770* | 17.85604047 | 74.69117583 | 4.182964077 | 2.064525608 | 1.10943E-06 | 1.02561E-05 | - |
| *NHX2* | 10.32100902 | 56.2383869 | 5.448923337 | 2.445971193 | 1.11303E-06 | 1.02785E-05 | - |
| *HSP70-17* | 461.7578696 | 1011.928923 | 2.191470876 | 1.131899506 | 1.12406E-06 | 1.03768E-05 | K09486 |
| *uncharacterized protein_17958* | 53.15147356 | 155.4699966 | 2.925036433 | 1.548454594 | 1.15417E-06 | 1.06399E-05 | - |
| *DRTI* | 1.218212282 | 25.13502319 | 20.63271202 | 4.366861561 | 1.16153E-06 | 1.07039E-05 | - |
| *TSK* | 12.91457617 | 64.01565213 | 4.95685273 | 2.309424396 | 1.16616E-06 | 1.07429E-05 | - |
| *ZIP5* | 261.7157981 | 588.6223663 | 2.249089931 | 1.169341349 | 1.18275E-06 | 1.08881E-05 | K14709 |
| *DDB_G0268948* | 49.78221159 | 558.020952 | 11.20924391 | 3.486617063 | 1.18361E-06 | 1.08922E-05 | - |
| *SDH* | 38.51403399 | 161.7669359 | 4.200207538 | 2.070460615 | 1.18617E-06 | 1.09119E-05 | K00008 |
| *CYPRO4* | 12.09219904 | 140.4382192 | 11.613952 | 3.537787071 | 1.21248E-06 | 1.11268E-05 | - |
| *GSTU19* | 313.9370072 | 693.49865 | 2.20903759 | 1.143417969 | 1.22486E-06 | 1.12247E-05 | K00799 |
| *SETH3* | 6.324945 | 43.73113619 | 6.914073749 | 2.789535992 | 1.23383E-06 | 1.12951E-05 | K06041 |
| *SULTR1;3* | 148.8462967 | 356.8249815 | 2.397271477 | 1.261393295 | 1.23742E-06 | 1.13242E-05 | K17470 |
| *MYB44* | 1280.288057 | 2665.735092 | 2.082136967 | 1.058064975 | 1.2535E-06 | 1.14593E-05 | K09422 |
| *PRCP* | 200.0324015 | 459.873296 | 2.298994026 | 1.201002717 | 1.26801E-06 | 1.1588E-05 | K01285 |
| *CIPK5* | 707.6413733 | 11887.44718 | 16.79868876 | 4.070276721 | 1.27086E-06 | 1.16019E-05 | K07198 |
| *ERF017* | 0 | 20.08624891 | Inf | Inf | 1.27216E-06 | 1.16098E-05 | - |
| *TRIP4* | 301.2703696 | 658.6351054 | 2.186192775 | 1.128420621 | 1.29096E-06 | 1.17691E-05 | - |
| *uncharacterized protein_20345* | 315.5721153 | 696.9124421 | 2.208409452 | 1.143007681 | 1.30061E-06 | 1.18448E-05 | - |
| *Eef1akmt4* | 72.09432936 | 194.9159067 | 2.703623273 | 1.434894138 | 1.3174E-06 | 1.19687E-05 | - |
| *fabZ* | 853.4053076 | 1790.192644 | 2.097705074 | 1.068811857 | 1.32835E-06 | 1.206E-05 | K02372 |
| *UGT73C3* | 3.391718975 | 34.05768299 | 10.04142243 | 3.327891745 | 1.33117E-06 | 1.20814E-05 | - |
| *WRKY57* | 16.8975814 | 73.30555074 | 4.338227408 | 2.11710568 | 1.33421E-06 | 1.21042E-05 | - |
| *TUR2* | 19.20564969 | 208.7613895 | 10.86979055 | 3.442252236 | 1.3346E-06 | 1.21042E-05 | - |
| *GH3.6* | 104.1162859 | 854.4005284 | 8.206214054 | 3.036716787 | 1.33941E-06 | 1.21437E-05 | K14487 |
| *TOL9* | 1739.39005 | 3594.076568 | 2.066285575 | 1.047039658 | 1.35911E-06 | 1.2318E-05 | - |
| *ZAT3* | 3.798844621 | 35.24018258 | 9.276552767 | 3.213588789 | 1.36522E-06 | 1.23691E-05 | - |
| *PXG* | 8.333307252 | 74.65884911 | 8.959089933 | 3.16335219 | 1.37054E-06 | 1.2413E-05 | K17991 |
| *Os04g0671100* | 338.4230036 | 833.8547649 | 2.463942332 | 1.300968491 | 1.37906E-06 | 1.24817E-05 | K00939 |
| *EO* | 330.5495857 | 727.354414 | 2.200439648 | 1.137791803 | 1.38658E-06 | 1.25454E-05 | K18980 |
| *TPC1* | 256.9851405 | 571.8773147 | 2.225332226 | 1.154020736 | 1.39279E-06 | 1.25973E-05 | K16900 |
| *PAH1* | 1149.24935 | 2389.583241 | 2.079255682 | 1.056067174 | 1.43592E-06 | 1.29607E-05 | K15728 |
| *ADH2* | 1592.33412 | 3493.028366 | 2.193652904 | 1.13333527 | 1.45482E-06 | 1.31223E-05 | K18857 |
| *3MMP* | 45.60149104 | 616.6140537 | 13.52179588 | 3.757214869 | 1.46955E-06 | 1.32415E-05 | - |
| *TMN1* | 498.1056549 | 1103.982166 | 2.216361438 | 1.148193172 | 1.48459E-06 | 1.33679E-05 | K17087 |
| *UGT85A8* | 36.3629843 | 261.2465601 | 7.184409231 | 2.84486953 | 1.49901E-06 | 1.34932E-05 | - |
| *uncharacterized protein_31661* | 98.63729518 | 249.2324515 | 2.526756751 | 1.337286784 | 1.50091E-06 | 1.35056E-05 | - |
| *EXPA8* | 0 | 29.63268738 | Inf | Inf | 1.50923E-06 | 1.35759E-05 | - |
| *MYB106* | 62.74117762 | 172.4614411 | 2.748775966 | 1.458789327 | 1.544E-06 | 1.38839E-05 | K09422 |
| *uncharacterized protein_24182* | 145.7096459 | 350.3297752 | 2.404300504 | 1.265617224 | 1.54808E-06 | 1.39158E-05 | - |
| *NPC1* | 611.0100522 | 1301.631634 | 2.130294958 | 1.091053198 | 1.56567E-06 | 1.40596E-05 | K12385 |
| *uncharacterized protein_29433* | 61.42848055 | 343.3698962 | 5.589750766 | 2.482783958 | 1.58057E-06 | 1.41885E-05 | - |
| *MSL2* | 3.444606616 | 33.67594897 | 9.776428114 | 3.289307463 | 1.58453E-06 | 1.42192E-05 | - |
| *uncharacterized protein_18459* | 71.70001454 | 223.9293948 | 3.123142948 | 1.642998604 | 1.59942E-06 | 1.4343E-05 | - |
| *ERF2* | 6.286512507 | 43.37363361 | 6.899474638 | 2.786486512 | 1.60163E-06 | 1.4358E-05 | K09286 |
| *ACO* | 0 | 17.52339344 | Inf | Inf | 1.6136E-06 | 1.44555E-05 | K05933 |
| *FDH1* | 13.45613944 | 85.78391694 | 6.375076398 | 2.672442631 | 1.61811E-06 | 1.44909E-05 | K00122 |
| *DES6* | 1369.811946 | 2824.438124 | 2.061916698 | 1.043986049 | 1.62366E-06 | 1.45308E-05 | K13076 |
| *uncharacterized protein_42143* | 23.34579751 | 111.8787487 | 4.792243602 | 2.260701246 | 1.64243E-06 | 1.46838E-05 | - |
| *PAS1* | 220.720482 | 498.9078869 | 2.260360627 | 1.176552964 | 1.64888E-06 | 1.47365E-05 | - |
| *UBC16* | 83.88812296 | 218.8830379 | 2.609225599 | 1.383621688 | 1.69828E-06 | 1.51421E-05 | K10688 |
| *PDR2* | 373.5157559 | 814.5422828 | 2.180744105 | 1.12482049 | 1.72006E-06 | 1.53258E-05 | K14950 |
| *EO* | 400.5568869 | 873.4987539 | 2.180710861 | 1.124798497 | 1.72499E-06 | 1.53646E-05 | K18980 |
| *XA21* | 2.147885031 | 31.62994397 | 14.72608799 | 3.880302322 | 1.74E-06 | 1.54931E-05 | - |
| *PAB2* | 992.0625839 | 2057.52234 | 2.073984417 | 1.052405055 | 1.74885E-06 | 1.55666E-05 | K13126 |
| *CBSDUF1* | 230.8406785 | 513.2841493 | 2.223542889 | 1.152860233 | 1.76335E-06 | 1.56851E-05 | K16302 |
| *INT2* | 1.585261117 | 968.7378861 | 611.0904227 | 9.25524206 | 1.76615E-06 | 1.57047E-05 | K08150 |
| *LHY* | 148.523542 | 348.8065777 | 2.348493532 | 1.23173562 | 1.78212E-06 | 1.5836E-05 | K12133 |
| *ECT2* | 1472.452841 | 3030.225763 | 2.057944185 | 1.041203855 | 1.81916E-06 | 1.61488E-05 | K20102 |
| *LHT1* | 92.52302853 | 651.0192047 | 7.036293721 | 2.814815707 | 1.8283E-06 | 1.62244E-05 | - |
| *GAPN* | 2270.814398 | 5053.003797 | 2.225194539 | 1.15393147 | 1.83034E-06 | 1.62371E-05 | K00131 |
| *DSP1* | 17.76307601 | 73.01899563 | 4.110717962 | 2.039390391 | 1.85383E-06 | 1.64234E-05 | K18045 |
| *uncharacterized protein_04301* | 173.5126692 | 400.4103634 | 2.307672202 | 1.206438308 | 1.8745E-06 | 1.66009E-05 | - |
| *lipl-1* | 301.6129691 | 664.0436651 | 2.201641618 | 1.138579647 | 1.90646E-06 | 1.68449E-05 | - |
| *GSTU19* | 237.8998452 | 526.9185588 | 2.214875585 | 1.147225661 | 1.923E-06 | 1.69848E-05 | K00799 |
| *At2g43240* | 229.5095852 | 535.1106325 | 2.331539365 | 1.221282788 | 1.93431E-06 | 1.70675E-05 | - |
| *exgA* | 5.606822817 | 340.8911924 | 60.7993517 | 5.925984035 | 1.97286E-06 | 1.73612E-05 | - |
| *IAA16* | 41.95839265 | 127.7221458 | 3.044019032 | 1.605977379 | 1.97878E-06 | 1.74075E-05 | K14484 |
| *HSP70-14* | 3260.442402 | 6643.722886 | 2.037675281 | 1.026924165 | 1.98838E-06 | 1.74861E-05 | K09489 |
| *AAO* | 2.555010678 | 29.60187638 | 11.58581318 | 3.534287401 | 1.99842E-06 | 1.75685E-05 | K00423 |
| *CALX* | 574.7900752 | 1201.313657 | 2.090004176 | 1.063505825 | 2.003E-06 | 1.76029E-05 | K08054 |
| *At4g01130* | 197.2457039 | 455.8181877 | 2.310915668 | 1.208464613 | 2.02398E-06 | 1.77663E-05 | - |
| *TPR2* | 1343.781004 | 2771.024339 | 2.062110069 | 1.044121341 | 2.02473E-06 | 1.77663E-05 | - |
| *ORP4C* | 140.6109151 | 335.6367049 | 2.386988981 | 1.255191907 | 2.02496E-06 | 1.77663E-05 | K20464 |
| *HSL1* | 19.38537326 | 337.9416558 | 17.43281655 | 4.123733773 | 2.05656E-06 | 1.80315E-05 | - |
| *MTN1* | 378.599788 | 807.0313738 | 2.131621304 | 1.091951157 | 2.05959E-06 | 1.80521E-05 | K01244 |
| *MNAT1* | 55.67884631 | 155.0171033 | 2.784129226 | 1.477226176 | 2.07279E-06 | 1.81617E-05 | K10842 |
| *At1g06690* | 2413.122268 | 4881.5645 | 2.022924642 | 1.016442578 | 2.13689E-06 | 1.87109E-05 | - |
| *At2g34160* | 605.1026598 | 1257.275329 | 2.077788469 | 1.055048787 | 2.14376E-06 | 1.87648E-05 | - |
| *EXPA8* | 9.928338522 | 764.3059806 | 76.98226435 | 6.266454202 | 2.17192E-06 | 1.90051E-05 | - |
| *At1g20050* | 140.8913288 | 333.6182612 | 2.367911951 | 1.243615436 | 2.20772E-06 | 1.93119E-05 | K01824 |
| *NRAMP3* | 32.46128109 | 105.3046536 | 3.244007938 | 1.69777735 | 2.22523E-06 | 1.94521E-05 | - |
| *GGCT2;2* | 1164.42374 | 2369.766055 | 2.035140623 | 1.025128485 | 2.25003E-06 | 1.96559E-05 | - |
| *UGT86A1* | 2.737774926 | 30.68605326 | 11.20839152 | 3.486507351 | 2.28262E-06 | 1.99273E-05 | - |
| *ERDJ7* | 744.1943277 | 1533.338818 | 2.060401108 | 1.042925221 | 2.30355E-06 | 2.01034E-05 | K19371 |
| *BIP* | 3381.613427 | 6883.149899 | 2.035463263 | 1.025357183 | 2.3202E-06 | 2.02353E-05 | K09490 |
| *HAK5* | 105.624806 | 342.1153485 | 3.238967829 | 1.695534139 | 2.33437E-06 | 2.03455E-05 | K03549 |
| *MYB308* | 335.4770625 | 809.8824833 | 2.41412178 | 1.271498454 | 2.35194E-06 | 2.04783E-05 | K09422 |
| *CPRF2* | 448.5471002 | 948.3696803 | 2.114314595 | 1.080190055 | 2.3662E-06 | 2.05889E-05 | - |
| *TRAF1A* | 825.7447958 | 1686.541974 | 2.042449414 | 1.030300347 | 2.37908E-06 | 2.06941E-05 | - |
| *MUR3* | 461.9033216 | 1308.467564 | 2.832773662 | 1.502215335 | 2.38778E-06 | 2.07629E-05 | - |
| *HSP21.7* | 0 | 25.4572542 | Inf | Inf | 2.39748E-06 | 2.08267E-05 | K13993 |
| *At3g20650* | 80.33277988 | 260.2442971 | 3.239577885 | 1.695805843 | 2.51777E-06 | 2.18284E-05 | K00565 |
| *At2g31810* | 486.6978067 | 1019.23291 | 2.0941802 | 1.066385589 | 2.57291E-06 | 2.22918E-05 | K01653 |
| *MYB1* | 639.2757292 | 1327.683322 | 2.076855512 | 1.054400851 | 2.60641E-06 | 2.25672E-05 | K09422 |
| *RRT1* | 496.2524865 | 1049.355224 | 2.114559125 | 1.0803569 | 2.61177E-06 | 2.26062E-05 | - |
| *uncharacterized protein_24330* | 9.904113219 | 51.08175906 | 5.157630767 | 2.366708495 | 2.62984E-06 | 2.27551E-05 | - |
| *CPK17* | 229.1920396 | 1136.636046 | 4.959317295 | 2.310141531 | 2.7153E-06 | 2.34484E-05 | K13412 |
| *uncharacterized protein_15354* | 16.33495748 | 68.48594752 | 4.192600293 | 2.067845295 | 2.7177E-06 | 2.34615E-05 | - |
| *CYP19-4* | 927.8921958 | 1911.794753 | 2.060363005 | 1.042898541 | 2.73314E-06 | 2.35639E-05 | K01802 |
| *EX1* | 60.29879568 | 166.7479194 | 2.765360692 | 1.467467667 | 2.78023E-06 | 2.39307E-05 | - |
| *UPS5* | 109.4008217 | 268.5490829 | 2.45472638 | 1.295562221 | 2.78785E-06 | 2.39884E-05 | - |
| *Os01g0693900* | 72.66458319 | 212.8819534 | 2.929652165 | 1.550729385 | 2.79595E-06 | 2.40502E-05 | K01056 |
| *uncharacterized protein_23496* | 0.995371222 | 22.10965054 | 22.21246711 | 4.473297735 | 2.85558E-06 | 2.45312E-05 | - |
| *E134* | 237.2237203 | 757.609297 | 3.193648999 | 1.675205761 | 2.86951E-06 | 2.46428E-05 | - |
| *RBP1* | 1676.91393 | 3393.963778 | 2.023934393 | 1.017162525 | 2.88E-06 | 2.47249E-05 | - |
| *At2g02240* | 35.11266884 | 110.6352175 | 3.15086324 | 1.655747137 | 2.88644E-06 | 2.4772E-05 | - |
| *NCER1* | 63.97536539 | 169.9035371 | 2.655765013 | 1.4091275 | 2.90644E-06 | 2.49275E-05 | K12349 |
| *CHI4* | 54.58352885 | 445.9583613 | 8.170200256 | 3.03037144 | 2.91576E-06 | 2.49992E-05 | K01183 |
| *yihQ* | 1.048258864 | 21.9598335 | 20.94886507 | 4.388800181 | 2.93287E-06 | 2.51309E-05 | - |
| *Prcp* | 179.4848965 | 405.6731108 | 2.260207509 | 1.176455232 | 2.94277E-06 | 2.52014E-05 | K01285 |
| *GPX4* | 149.547171 | 390.4577541 | 2.610933737 | 1.384565844 | 2.96719E-06 | 2.53907E-05 | K00432 |
| *PDAT2* | 8.79332054 | 57.77495331 | 6.570322673 | 2.715964224 | 3.00323E-06 | 2.56824E-05 | K00679 |
| *DDB_G0268948* | 10.28232857 | 394.2859654 | 38.34597999 | 5.261003435 | 3.06668E-06 | 2.61809E-05 | - |
| *At1g07160* | 3.603269542 | 56.51987818 | 15.68572029 | 3.971379875 | 3.06748E-06 | 2.61809E-05 | K17506 |
| *NUDT16* | 2.947681175 | 30.10443701 | 10.21292169 | 3.352323743 | 3.10892E-06 | 2.65002E-05 | K07766 |
| *Os08g0191100* | 27.81304133 | 93.99155869 | 3.379405998 | 1.756769685 | 3.1344E-06 | 2.67088E-05 | K01681 |
| *PTI1* | 357.267714 | 758.5498043 | 2.123197184 | 1.086238362 | 3.27542E-06 | 2.78834E-05 | K13436 |
| *uncharacterized protein_02690* | 129.7796456 | 388.9659901 | 2.997126307 | 1.583579884 | 3.28296E-06 | 2.79295E-05 | - |
| *ATX5* | 296.6717245 | 641.4794739 | 2.162253497 | 1.112535671 | 3.29132E-06 | 2.79826E-05 | - |
| *uncharacterized protein_21041* | 3.563192731 | 116.0530705 | 32.56996724 | 5.025470363 | 3.3239E-06 | 2.82476E-05 | - |
| *PUB45* | 12.67715598 | 71.82991004 | 5.666090262 | 2.502353584 | 3.32464E-06 | 2.82476E-05 | - |
| *uncharacterized protein_15644* | 6.052504936 | 49.17116891 | 8.12410224 | 3.022208396 | 3.33812E-06 | 2.83439E-05 | - |
| *UBC5* | 73.66007839 | 191.2621953 | 2.596551613 | 1.376596903 | 3.34298E-06 | 2.83761E-05 | K10576 |
| *Vps13* | 24.55284328 | 86.32519134 | 3.515893877 | 1.813891525 | 3.39937E-06 | 2.88268E-05 | K19525 |
| *AM9* | 434.4986826 | 905.4519779 | 2.083900399 | 1.059286325 | 3.42292E-06 | 2.90078E-05 | - |
| *DLO2* | 0 | 20.50852371 | Inf | Inf | 3.48478E-06 | 2.94942E-05 | - |
| *RAP2-9* | 132.6217037 | 311.3664367 | 2.347778892 | 1.231296545 | 3.50383E-06 | 2.96459E-05 | K09286 |
| *CRK8* | 13.9257989 | 206.5521772 | 14.83233951 | 3.890674268 | 3.50508E-06 | 2.96469E-05 | - |
| *PARP3* | 7.729086187 | 45.01013147 | 5.823473873 | 2.54188002 | 3.52263E-06 | 2.97763E-05 | K10798 |
| *At2g30020* | 342.9713015 | 790.2456963 | 2.304116096 | 1.204213411 | 3.53477E-06 | 2.98613E-05 | - |
| *RHY1A* | 45.49912837 | 597.8799725 | 13.1404709 | 3.715945072 | 3.53495E-06 | 2.98613E-05 | - |
| *HIR4* | 162.1436492 | 473.9458266 | 2.922999629 | 1.547449646 | 3.59381E-06 | 3.03488E-05 | - |
| *VHA-a3* | 406.8996365 | 857.8846946 | 2.108344706 | 1.076110761 | 3.60664E-06 | 3.04473E-05 | K02154 |
| *DTX16* | 157.6626941 | 364.4175431 | 2.311374578 | 1.20875108 | 3.6249E-06 | 3.05623E-05 | K03327 |
| *GT-3B* | 387.2551299 | 2272.033574 | 5.867019953 | 2.552627899 | 3.63247E-06 | 3.06163E-05 | - |
| *CDKG-2* | 324.030338 | 688.4825008 | 2.124747038 | 1.087291091 | 3.63679E-06 | 3.0643E-05 | K08818 |
| *FDX3* | 188.1856245 | 924.085324 | 4.910499016 | 2.295869642 | 3.66719E-06 | 3.08893E-05 | K02639 |
| *CYP59* | 128.6212027 | 304.5429844 | 2.367751024 | 1.243517386 | 3.68463E-06 | 3.10263E-05 | K12735 |
| *Os04g0338000* | 2127.609577 | 4532.412564 | 2.130283965 | 1.091045753 | 3.70219E-06 | 3.11641E-05 | - |
| *alaXL* | 9.184346718 | 47.68763187 | 5.192272606 | 2.37636613 | 3.72861E-06 | 3.13765E-05 | - |
| *uncharacterized protein_11719* | 159.9687462 | 367.0830152 | 2.294717086 | 1.198316296 | 3.7712E-06 | 3.17146E-05 | K12501 |
| *FAHD1* | 67.83193485 | 178.174137 | 2.626699907 | 1.393251386 | 3.7898E-06 | 3.1861E-05 | K01557 |
| *At4g33300* | 0.314161193 | 35.5959705 | 113.3047978 | 6.824065142 | 3.79465E-06 | 3.18915E-05 | - |
| *At3g58180* | 29.81342994 | 97.88661844 | 3.283306168 | 1.715149288 | 3.84138E-06 | 3.22534E-05 | K06072 |
| *ESMD1* | 34.2007681 | 124.589218 | 3.642877774 | 1.865078591 | 3.86022E-06 | 3.24013E-05 | - |
| *TOL6* | 6.523684736 | 41.11192149 | 6.301947926 | 2.655797833 | 3.89686E-06 | 3.26361E-05 | - |
| *WOX8* | 20.18833406 | 76.71924341 | 3.800177031 | 1.926066628 | 3.89981E-06 | 3.26504E-05 | - |
| *AHA6* | 7739.02159 | 17155.07886 | 2.21669867 | 1.148412669 | 3.91235E-06 | 3.2745E-05 | K01535 |
| *RBM19* | 246.7394717 | 543.7318377 | 2.203667836 | 1.139906779 | 3.92605E-06 | 3.28492E-05 | K14787 |
| *UBP19* | 179.07433 | 398.9518407 | 2.227856112 | 1.155656058 | 3.9392E-06 | 3.29488E-05 | K11855 |
| *AMY1.1* | 50.21154628 | 140.9537336 | 2.807197627 | 1.489130633 | 3.97008E-06 | 3.3186E-05 | K01176 |
| *FLZ10* | 514.6246534 | 1061.023627 | 2.061742709 | 1.043864306 | 3.97956E-06 | 3.32547E-05 | - |
| *uncharacterized protein_00405* | 161.4626872 | 369.4014779 | 2.287844235 | 1.193988831 | 4.04847E-06 | 3.3777E-05 | - |
| *exgA* | 11.23939128 | 530.2005506 | 47.17342226 | 5.559902362 | 4.06958E-06 | 3.39423E-05 | - |
| *SIS8* | 251.2738104 | 551.0932216 | 2.193198013 | 1.133036072 | 4.07401E-06 | 3.39685E-05 | - |
| *SAC9* | 1232.941189 | 2472.408277 | 2.005292952 | 1.003813015 | 4.09128E-06 | 3.41017E-05 | - |
| *SULTR3;1* | 0 | 64.87874679 | Inf | Inf | 4.10363E-06 | 3.41939E-05 | K17471 |
| *CNGC5* | 265.9915601 | 568.4817326 | 2.137217182 | 1.095733521 | 4.10767E-06 | 3.42167E-05 | K05391 |
| *FACE1* | 325.460129 | 690.5786834 | 2.121853406 | 1.085324987 | 4.11725E-06 | 3.42857E-05 | K06013 |
| *NFYC4* | 0 | 16.48928819 | Inf | Inf | 4.12317E-06 | 3.43242E-05 | K08066 |
| *MYC2* | 349.553331 | 1374.595243 | 3.93243354 | 1.975422384 | 4.2001E-06 | 3.48874E-05 | K13422 |
| *uncharacterized protein_26434* | 395.985077 | 829.592432 | 2.095009333 | 1.066956671 | 4.2209E-06 | 3.50381E-05 | - |
| *WAKL20* | 0.301350363 | 33.18281972 | 110.113754 | 6.782850873 | 4.2362E-06 | 3.51541E-05 | - |
| *At4g01130* | 1672.241898 | 5631.350744 | 3.36754554 | 1.751697455 | 4.24782E-06 | 3.52283E-05 | - |
| *GMII* | 101.3227066 | 248.1791114 | 2.449392833 | 1.292424172 | 4.31591E-06 | 3.57256E-05 | K01231 |
| *PYD3* | 547.6235566 | 1126.262648 | 2.056636597 | 1.040286896 | 4.32526E-06 | 3.57917E-05 | K01431 |
| *TAF1* | 254.7259229 | 553.0418901 | 2.171125277 | 1.118442974 | 4.33439E-06 | 3.5856E-05 | K03125 |
| *Os03g0405900* | 533.6917439 | 1099.995839 | 2.061107093 | 1.043419468 | 4.3621E-06 | 3.60399E-05 | K17609 |
| *RXW8* | 17.1219428 | 90.07398216 | 5.26073374 | 2.395264033 | 4.39916E-06 | 3.63234E-05 | - |
| *DR* | 3.327664821 | 124.5933995 | 37.44169145 | 5.226573706 | 4.42716E-06 | 3.65317E-05 | - |
| *ERDJ3B* | 1136.469451 | 2296.995286 | 2.021167647 | 1.015188992 | 4.45685E-06 | 3.67537E-05 | K09517 |
| *CYP59* | 38.48664403 | 116.400532 | 3.024439645 | 1.596667871 | 4.4744E-06 | 3.68522E-05 | K12735 |
| *Tmem45b* | 23.40553861 | 147.8771173 | 6.318039493 | 2.659476955 | 4.49379E-06 | 3.70004E-05 | - |
| *PROT1* | 36.82755861 | 111.3809471 | 3.024391279 | 1.5966448 | 4.51442E-06 | 3.71355E-05 | - |
| *FPS1* | 287.0633806 | 611.8864277 | 2.131537734 | 1.091894595 | 4.65304E-06 | 3.82161E-05 | K00787 |
| *CYP750A1* | 2.018008425 | 1310.289366 | 649.2982635 | 9.342737541 | 4.74814E-06 | 3.89729E-05 | - |
| *At1g08370* | 636.533365 | 1295.466914 | 2.035190903 | 1.025164127 | 4.83901E-06 | 3.96817E-05 | K12611 |
| *uncharacterized protein_50929* | 49.87328376 | 138.1028939 | 2.769075614 | 1.469404449 | 4.99186E-06 | 4.0897E-05 | K15305 |
| *CBG06644* | 239.6918479 | 513.5952515 | 2.142731411 | 1.099451021 | 5.07243E-06 | 4.15442E-05 | - |
| *AEL1* | 490.0414426 | 1012.727842 | 2.066616727 | 1.047270853 | 5.07914E-06 | 4.15732E-05 | - |
| *MOT2* | 198.9279664 | 437.7680022 | 2.200635789 | 1.137920395 | 5.10955E-06 | 4.18092E-05 | - |
| *HIPP23* | 158.7838812 | 356.2729633 | 2.243760264 | 1.165918539 | 5.11545E-06 | 4.18445E-05 | - |
| *NHL3* | 225.5933311 | 686.3484582 | 3.042414662 | 1.605216797 | 5.16027E-06 | 4.21588E-05 | - |
| *uncharacterized protein_04110* | 80.54916764 | 201.7739041 | 2.50497814 | 1.324798013 | 5.1631E-06 | 4.21688E-05 | - |
| *CYP76T24* | 3.104823762 | 31.88456379 | 10.26936349 | 3.36027486 | 5.20689E-06 | 4.25001E-05 | - |
| *smek1* | 431.4855792 | 891.7342942 | 2.066660712 | 1.047301558 | 5.22801E-06 | 4.26461E-05 | K17491 |
| *MES1* | 82.68929878 | 1506.743649 | 18.22174902 | 4.187589539 | 5.24351E-06 | 4.27329E-05 | - |
| *MYB308* | 18.11895834 | 70.13208215 | 3.870646471 | 1.952574544 | 5.28255E-06 | 4.29845E-05 | K09422 |
| *PXN* | 83.09478011 | 208.2351878 | 2.505996015 | 1.325384121 | 5.296E-06 | 4.30807E-05 | K13354 |
| *At3g27220* | 20.9994207 | 123.1197783 | 5.86300832 | 2.551641104 | 5.29836E-06 | 4.30866E-05 | - |
| *uncharacterized protein_17210* | 10.375417 | 302.1174159 | 29.11858057 | 4.863868126 | 5.34168E-06 | 4.33853E-05 | - |
| *TYW1* | 341.1730652 | 711.7829516 | 2.086281199 | 1.060933624 | 5.40484E-06 | 4.38578E-05 | K15449 |
| *MTB1* | 446.2809052 | 924.8845037 | 2.07242679 | 1.051321138 | 5.5201E-06 | 4.47517E-05 | - |
| *At5g26710* | 353.404911 | 738.0775855 | 2.088475747 | 1.06245039 | 5.55254E-06 | 4.50009E-05 | K01885 |
| *B0616E02-H0507E05.7* | 216.5384326 | 466.5678893 | 2.154665496 | 1.107463914 | 5.64212E-06 | 4.56426E-05 | - |
| *LOH3* | 104.8744566 | 249.666004 | 2.380617855 | 1.251336053 | 5.6446E-06 | 4.56487E-05 | K04710 |
| *HSD* | 6.065315767 | 234.5497444 | 38.67065679 | 5.273167363 | 5.74929E-06 | 4.64383E-05 | - |
| *METK1* | 826.5525175 | 2274.568884 | 2.751874607 | 1.460414733 | 5.75119E-06 | 4.64394E-05 | K00789 |
| *OFUT1* | 57.02262215 | 156.4011196 | 2.742790734 | 1.455644553 | 5.7783E-06 | 4.66298E-05 | - |
| *GIP2* | 3.353286482 | 109.4722641 | 32.64626052 | 5.028845842 | 5.79196E-06 | 4.67257E-05 | - |
| *GH3.6* | 0.301350363 | 17.49602464 | 58.05874757 | 5.859441546 | 5.81189E-06 | 4.68722E-05 | K14487 |
| *uncharacterized protein_12763* | 32.83810008 | 224.116933 | 6.824905595 | 2.77080909 | 5.84336E-06 | 4.70971E-05 | - |
| *ATL60* | 0.301350363 | 17.44118758 | 57.87677645 | 5.854912665 | 5.91776E-06 | 4.76677E-05 | - |
| *DRP5A* | 161.5442371 | 362.0778067 | 2.241353904 | 1.164370465 | 6.00449E-06 | 4.83515E-05 | - |
| *LOX1.1* | 8026.085321 | 34207.1861 | 4.262001303 | 2.091531034 | 6.0724E-06 | 4.88089E-05 | K15718 |
| *HAK5* | 41.62670741 | 169.5070288 | 4.072073901 | 2.025763744 | 6.15807E-06 | 4.94371E-05 | K03549 |
| *BIO2* | 374.2000109 | 770.3095113 | 2.058550211 | 1.041628638 | 6.33266E-06 | 5.07769E-05 | K01012 |
| *ERF9* | 15.36685224 | 105.3045412 | 6.852707344 | 2.776674076 | 6.34626E-06 | 5.08705E-05 | K09286 |
| *At2g16710* | 207.7433438 | 448.2663594 | 2.157789276 | 1.109553982 | 6.46421E-06 | 5.17373E-05 | K13628 |
| *MAVI* | 13.42735312 | 349.3808163 | 26.02008104 | 4.70155355 | 6.46852E-06 | 5.1756E-05 | - |
| *Mgst3* | 83.99718688 | 226.1319152 | 2.692136767 | 1.428751704 | 6.54864E-06 | 5.23177E-05 | K00799 |
| *HSP21* | 115.4148941 | 269.6501896 | 2.336355214 | 1.224259635 | 6.59282E-06 | 5.26547E-05 | K13993 |
| *DTX41* | 332.9965243 | 700.0270911 | 2.102205399 | 1.071903637 | 6.60878E-06 | 5.27661E-05 | K03327 |
| *GINT1* | 454.457718 | 937.1857751 | 2.062206753 | 1.044188982 | 6.75011E-06 | 5.37968E-05 | - |
| *uncharacterized protein_23012* | 181.485905 | 402.124706 | 2.215735189 | 1.14778547 | 6.80832E-06 | 5.42115E-05 | - |
| *IP5P4* | 106.65076 | 360.7075463 | 3.382137606 | 1.757935359 | 6.97054E-06 | 5.54529E-05 | - |
| *DIT1* | 553.178575 | 1134.231285 | 2.050389036 | 1.035897669 | 7.03314E-06 | 5.59172E-05 | - |
| *CKL2* | 379.0030965 | 782.8235598 | 2.065480644 | 1.046477541 | 7.05818E-06 | 5.60656E-05 | K02218 |
| *ESD4* | 304.4449244 | 634.1900815 | 2.083102823 | 1.058734054 | 7.13166E-06 | 5.6598E-05 | K08592 |
| *NTF2* | 122.1871655 | 282.8861277 | 2.315186922 | 1.211128678 | 7.19753E-06 | 5.71036E-05 | - |
| *uncharacterized protein_51264* | 23.8571781 | 98.2406917 | 4.117867222 | 2.041897312 | 7.23226E-06 | 5.73619E-05 | - |
| *fabG* | 22.9130502 | 161.5688069 | 7.051387988 | 2.817907264 | 7.30542E-06 | 5.79072E-05 | - |
| *NAGS2* | 62.95945767 | 216.4798094 | 3.438400161 | 1.781737455 | 7.31728E-06 | 5.79838E-05 | K14682 |
| *PLIP2* | 35.03251522 | 116.5796443 | 3.327755475 | 1.734549427 | 7.41619E-06 | 5.86794E-05 | - |
| *Ermp1* | 58.58669864 | 154.6258384 | 2.63926526 | 1.400136357 | 7.48298E-06 | 5.91368E-05 | - |
| *XLG3* | 67.14945245 | 173.9454819 | 2.590422939 | 1.373187666 | 7.59546E-06 | 5.99539E-05 | - |
| *Cwc22* | 1033.774854 | 2582.747691 | 2.498365751 | 1.320984698 | 7.70827E-06 | 6.07897E-05 | K13100 |
| *uncharacterized protein_07223* | 455.0994472 | 923.149301 | 2.028456212 | 1.02038216 | 7.71106E-06 | 6.07935E-05 | - |
| *uncharacterized protein_27700* | 23.93556342 | 80.29875762 | 3.354788697 | 1.7462219 | 7.88E-06 | 6.20327E-05 | - |
| *FBL3* | 115.5319599 | 267.6479558 | 2.316657279 | 1.212044631 | 7.91457E-06 | 6.22862E-05 | K10268 |
| *At1g06900* | 93.5086296 | 225.6347103 | 2.412982751 | 1.270817603 | 8.00714E-06 | 6.29395E-05 | K01411 |
| *SH3P2* | 352.5643224 | 722.1851767 | 2.048378497 | 1.034482319 | 8.18208E-06 | 6.4238E-05 | - |
| *PLD1* | 1988.392732 | 4206.184204 | 2.115368929 | 1.080909297 | 8.24983E-06 | 6.47506E-05 | K01115 |
| *VPS41* | 138.2893878 | 311.025038 | 2.249088256 | 1.169340274 | 8.29439E-06 | 6.50422E-05 | K20184 |
| *uncharacterized protein_08103* | 22.57162303 | 78.14642766 | 3.462153677 | 1.791669764 | 8.30642E-06 | 6.51172E-05 | K06100 |
| *At3g20240* | 15.77220959 | 62.60772208 | 3.96949595 | 1.988955824 | 8.32459E-06 | 6.52402E-05 | K14684 |
| *At2g40090* | 84.39785923 | 207.3699484 | 2.457052232 | 1.296928527 | 8.3674E-06 | 6.55562E-05 | K08869 |
| *EAP3* | 9.931379199 | 104.4399373 | 10.51615643 | 3.394535603 | 8.53824E-06 | 6.6855E-05 | - |
| *uncharacterized protein_34596* | 105.9135935 | 251.9307605 | 2.378644253 | 1.25013952 | 8.55105E-06 | 6.69354E-05 | K15032 |
| *UAM1* | 3.223409876 | 29.27974484 | 9.08346936 | 3.183243429 | 8.5879E-06 | 6.7164E-05 | K13379 |
| *UGT89B2* | 228.9647051 | 489.3721858 | 2.137325863 | 1.095806882 | 8.64602E-06 | 6.75584E-05 | K13496 |
| *uncharacterized protein_25609* | 177.8455993 | 2198.327085 | 12.36087423 | 3.627708877 | 8.80897E-06 | 6.87501E-05 | - |
| *SAB* | 474.9615814 | 967.1416209 | 2.036252318 | 1.025916341 | 8.84274E-06 | 6.89729E-05 | - |
| *PAPS1* | 288.6582596 | 598.5325756 | 2.073498872 | 1.052067262 | 8.85884E-06 | 6.90779E-05 | K14376 |
| *HS1* | 237.8346709 | 503.7724722 | 2.118162463 | 1.082813248 | 8.96068E-06 | 6.98102E-05 | - |
| *QCR6-1* | 147.6459804 | 331.5269436 | 2.24541801 | 1.166984044 | 8.97785E-06 | 6.99232E-05 | K00416 |
| *CGR2* | 134.7410222 | 305.2857756 | 2.265722574 | 1.179971221 | 9.03539E-06 | 7.03506E-05 | - |
| *GSVIVT00026920001* | 229.4228543 | 490.4001158 | 2.137538203 | 1.095950205 | 9.11575E-06 | 7.09343E-05 | - |
| *ACS* | 378.2168833 | 934.4726037 | 2.470732125 | 1.304938603 | 9.17448E-06 | 7.13071E-05 | K01895 |
| *RH32* | 212.1889027 | 455.8854876 | 2.148488832 | 1.103322278 | 9.21557E-06 | 7.16054E-05 | K14776 |
| *TIC* | 307.3109837 | 715.5433615 | 2.32840152 | 1.219339865 | 9.25562E-06 | 7.1853E-05 | - |
| *NAC045* | 1424.903858 | 2872.089865 | 2.015637651 | 1.01123631 | 9.26654E-06 | 7.19167E-05 | - |
| *NPF7.3* | 2.82909506 | 77.68461964 | 27.45917616 | 4.779216437 | 9.31308E-06 | 7.22353E-05 | K14638 |
| *MAPKKK17* | 0 | 14.45149081 | Inf | Inf | 9.3276E-06 | 7.23266E-05 | - |
| *ncl1* | 557.1010039 | 1221.214878 | 2.192088812 | 1.13230625 | 9.43454E-06 | 7.30659E-05 | - |
| *IRE1A* | 445.3020097 | 897.6488245 | 2.015820286 | 1.011367026 | 9.43681E-06 | 7.30659E-05 | K08852 |
| *ML2* | 427.7259109 | 1502.215531 | 3.512098501 | 1.812333308 | 9.43957E-06 | 7.30659E-05 | - |
| *CNR6* | 113.4610638 | 262.8835074 | 2.316949079 | 1.212226338 | 9.49466E-06 | 7.34061E-05 | - |
| *ACD11* | 132.62372 | 301.1775299 | 2.270917525 | 1.183275311 | 9.50238E-06 | 7.34442E-05 | - |
| *INV1* | 7.28188373 | 41.84812021 | 5.746881132 | 2.522779209 | 9.68318E-06 | 7.46883E-05 | K01193 |
| *At3g16150* | 887.7151353 | 4797.016723 | 5.403779357 | 2.433968769 | 9.69806E-06 | 7.47812E-05 | K13051 |
| *CRJ33* | 62.56222615 | 721.7141419 | 11.53594087 | 3.52806377 | 9.86993E-06 | 7.60619E-05 | - |
| *DVL11* | 243.1236719 | 510.222398 | 2.098612587 | 1.069435863 | 1.00736E-05 | 7.75863E-05 | - |
| *MAP2B* | 90.36044039 | 216.5421642 | 2.396426614 | 1.260884761 | 1.00981E-05 | 7.77292E-05 | K01265 |
| *WRKY4* | 85.77650274 | 213.831338 | 2.492889442 | 1.317818902 | 1.01194E-05 | 7.78479E-05 | - |
| *uncharacterized protein_37019* | 0 | 65.41707634 | Inf | Inf | 1.01824E-05 | 7.82642E-05 | - |
| *ATL60* | 71.2622103 | 178.6605679 | 2.507087096 | 1.326012116 | 1.02708E-05 | 7.88743E-05 | - |
| *HSP12* | 10.66547687 | 75.23914214 | 7.054456454 | 2.818534927 | 1.02871E-05 | 7.89766E-05 | K13993 |
| *SEC23* | 462.271795 | 1454.537573 | 3.146498636 | 1.653747318 | 1.03625E-05 | 7.95161E-05 | - |
| *PNC1* | 0 | 19.81885496 | Inf | Inf | 1.03755E-05 | 7.95623E-05 | K00430 |
| *ACA5* | 615.186588 | 2030.936088 | 3.301333494 | 1.723048884 | 1.04775E-05 | 8.02978E-05 | K01537 |
| *DR* | 2.750585757 | 59.18602716 | 21.51760839 | 4.427445831 | 1.05843E-05 | 8.10932E-05 | - |
| *uncharacterized protein_21086* | 48.17588982 | 130.442827 | 2.707637108 | 1.437034394 | 1.0601E-05 | 8.1174E-05 | - |
| *Os07g0673200* | 7.453357486 | 85.84696162 | 11.51789134 | 3.525804711 | 1.08155E-05 | 8.2648E-05 | K16275 |
| *TH* | 3.643346353 | 145.4646499 | 39.92611072 | 5.319260638 | 1.08256E-05 | 8.27016E-05 | K00500 |
| *VCL* | 183.8056683 | 1050.934331 | 5.717638314 | 2.515419361 | 1.09508E-05 | 8.35366E-05 | - |
| *At3g58180* | 17.27744106 | 69.6233334 | 4.029724838 | 2.010681331 | 1.09924E-05 | 8.38298E-05 | K06072 |
| *uncharacterized protein_27427* | 118.4431008 | 271.5144359 | 2.292361766 | 1.196834739 | 1.10089E-05 | 8.39312E-05 | - |
| *PHOS32* | 415.3008826 | 835.2028677 | 2.011078962 | 1.007969728 | 1.1043E-05 | 8.41671E-05 | - |
| *uncharacterized protein_27363* | 52.25415195 | 6837.748991 | 130.8556112 | 7.03183198 | 1.10499E-05 | 8.41706E-05 | - |
| *prmC* | 138.6514755 | 337.6901025 | 2.435531979 | 1.284236926 | 1.11499E-05 | 8.48588E-05 | K02493 |
| *uncharacterized protein_00911* | 43.93809256 | 275.4374978 | 6.268763202 | 2.648180834 | 1.11565E-05 | 8.48846E-05 | - |
| *uncharacterized protein_06213* | 3.681778845 | 176.643592 | 47.97778448 | 5.584294633 | 1.13341E-05 | 8.61617E-05 | - |
| *uncharacterized protein_23461* | 4.675629729 | 42.00603896 | 8.984038814 | 3.167364161 | 1.13412E-05 | 8.61908E-05 | - |
| *BLOS2* | 217.6444163 | 646.7198949 | 2.971451811 | 1.571167984 | 1.145E-05 | 8.69671E-05 | K16750 |
| *uncharacterized protein_42840* | 81.51448017 | 201.0568859 | 2.466517427 | 1.302475481 | 1.14541E-05 | 8.69731E-05 | - |
| *uncharacterized protein_04156* | 146.1309788 | 420.2465675 | 2.875821205 | 1.523973984 | 1.16221E-05 | 8.8173E-05 | - |
| *TIFY6A* | 0.602700725 | 44.48660198 | 73.81209297 | 6.205785294 | 1.17584E-05 | 8.91044E-05 | K13464 |
| *BAM9* | 215.4414752 | 658.6286797 | 3.057111817 | 1.612169321 | 1.19775E-05 | 9.06599E-05 | - |
| *MHZ4* | 101.2556118 | 239.5237332 | 2.365535391 | 1.242166745 | 1.20087E-05 | 9.087E-05 | - |
| *RLK7* | 21.62901546 | 73.8341505 | 3.413662107 | 1.771320264 | 1.20752E-05 | 9.13475E-05 | - |
| *CET1* | 0 | 14.12925981 | Inf | Inf | 1.21331E-05 | 9.17587E-05 | - |
| *uncharacterized protein_15858* | 19.1672172 | 69.63287069 | 3.63291499 | 1.861127607 | 1.22143E-05 | 9.23201E-05 | - |
| *PSL4* | 101.9734578 | 238.8602288 | 2.342376477 | 1.227972971 | 1.2351E-05 | 9.32997E-05 | K08288 |
| *BC10* | 402.7592125 | 814.8867579 | 2.023260381 | 1.016681998 | 1.24497E-05 | 9.39918E-05 | - |
| *At1g34470* | 337.5722403 | 693.4427015 | 2.054205348 | 1.038580408 | 1.2472E-05 | 9.41058E-05 | - |
| *OPR11* | 0 | 14.0309371 | Inf | Inf | 1.259E-05 | 9.49688E-05 | K05894 |
| *At1g06900* | 47.20120731 | 126.9555275 | 2.689666954 | 1.427427543 | 1.287E-05 | 9.68874E-05 | K01411 |
| *RPK1* | 20.72837699 | 92.48550487 | 4.461782266 | 2.157620112 | 1.30787E-05 | 9.83462E-05 | K13420 |
| *NPF2.13* | 17.38473669 | 2445.871424 | 140.6907374 | 7.136383539 | 1.31804E-05 | 9.90261E-05 | - |
| *ZAT3* | 54.98178478 | 290.7408527 | 5.287948616 | 2.402708158 | 1.34233E-05 | 0.00010065 | - |
| *TOL6* | 119.3280118 | 273.2756197 | 2.290121285 | 1.195424006 | 1.35494E-05 | 0.000101509 | - |
| *XRN3* | 176.794924 | 375.8443748 | 2.125877634 | 1.088058557 | 1.38045E-05 | 0.000103218 | K12619 |
| *sec61a* | 465.9755067 | 934.4246353 | 2.005308481 | 1.003824186 | 1.38049E-05 | 0.000103218 | K10956 |
| *TAF8* | 99.06396113 | 229.5133484 | 2.316819818 | 1.212145848 | 1.39524E-05 | 0.000104232 | K14649 |
| *APF1* | 228.0175647 | 481.9337895 | 2.113581864 | 1.079689992 | 1.40016E-05 | 0.00010457 | - |
| *DAAT* | 38.27825812 | 110.0777268 | 2.875724555 | 1.523925497 | 1.412E-05 | 0.000105424 | K18482 |
| *HIR4* | 23.67314147 | 93.28567363 | 3.940570107 | 1.978404369 | 1.41441E-05 | 0.000105574 | K18999 |
| *HIT4* | 209.4635666 | 441.7207637 | 2.10881907 | 1.076435322 | 1.44037E-05 | 0.000107421 | - |
| *SCPL51* | 34.4587248 | 181.982312 | 5.281167922 | 2.400857015 | 1.4574E-05 | 0.000108599 | K09646 |
| *LPCAT1* | 158.900947 | 344.2674602 | 2.166553861 | 1.115402103 | 1.45787E-05 | 0.000108602 | K13519 |
| *LBD38* | 311.328613 | 629.992951 | 2.023562643 | 1.016897511 | 1.46663E-05 | 0.000109194 | - |
| *NAC047* | 0 | 184.4469156 | Inf | Inf | 1.47184E-05 | 0.000109519 | - |
| *LRK10L-2.4* | 7.086308651 | 74.6619929 | 10.53609101 | 3.397267808 | 1.5304E-05 | 0.000113492 | - |
| *uncharacterized protein_30668* | 5.961060822 | 36.23242382 | 6.078183884 | 2.603640322 | 1.5346E-05 | 0.00011374 | - |
| *uncharacterized protein_16578* | 120.2125226 | 499.0763956 | 4.15161736 | 2.053673482 | 1.53728E-05 | 0.000113883 | - |
| *MES13* | 11.97500929 | 130.1080337 | 10.86496307 | 3.441611366 | 1.5374E-05 | 0.000113883 | - |
| *SNX2A* | 219.382135 | 458.5842682 | 2.090344632 | 1.063740817 | 1.57847E-05 | 0.000116696 | - |
| *JAL3* | 15.74671191 | 61.56854656 | 3.909930335 | 1.967142903 | 1.58343E-05 | 0.00011703 | - |
| *GDPDL4* | 35.85957735 | 104.2930586 | 2.908373895 | 1.540212751 | 1.59574E-05 | 0.000117874 | - |
| *ATG2* | 203.9703688 | 427.769386 | 2.097213378 | 1.068473654 | 1.59835E-05 | 0.000118034 | K17906 |
| *BACOVA_02659* | 34.96175981 | 101.9782306 | 2.916850614 | 1.544411501 | 1.60161E-05 | 0.000118241 | K05349 |
| *PNC2* | 0.314161193 | 16.0558609 | 51.10707888 | 5.675451229 | 1.6134E-05 | 0.000119045 | - |
| *FAO4A* | 19.80987076 | 69.43805229 | 3.505224902 | 1.809507013 | 1.61667E-05 | 0.000119253 | K17756 |
| *LECRKS4* | 86.88221026 | 208.6145911 | 2.401119751 | 1.263707357 | 1.63587E-05 | 0.000120467 | - |
| *NRAMP2* | 639.4011688 | 1554.183616 | 2.43068623 | 1.281363672 | 1.64004E-05 | 0.00012074 | K12347 |
| *WNK2* | 12.57783399 | 53.53028089 | 4.255922039 | 2.089471723 | 1.68272E-05 | 0.000123537 | K08867 |
| *OXI1* | 0.995371222 | 145.9338525 | 146.6124892 | 7.195864194 | 1.70193E-05 | 0.000124912 | - |
| *MDH* | 160.2586821 | 384.5668625 | 2.399663204 | 1.262831936 | 1.71014E-05 | 0.000125445 | K00026 |
| *luxQ* | 206.5128448 | 434.5714522 | 2.104331344 | 1.073361887 | 1.74357E-05 | 0.00012772 | - |
| *SEC15A* | 303.89706 | 1118.59689 | 3.680841432 | 1.880035601 | 1.7676E-05 | 0.000129408 | K19985 |
| *DHS* | 156.0268422 | 335.4220226 | 2.14977127 | 1.104183169 | 1.77043E-05 | 0.000129579 | K00809 |
| *CYP75B137* | 367.2736614 | 741.683264 | 2.019429493 | 1.013947776 | 1.77159E-05 | 0.000129628 | K05280 |
| *UPM1* | 28.93993347 | 89.29552156 | 3.085546884 | 1.625526216 | 1.79196E-05 | 0.000131046 | - |
| *CPK11* | 211.2236184 | 442.4827032 | 2.09485429 | 1.066849899 | 1.8417E-05 | 0.000134273 | K13412 |
| *HSL1* | 2.502123036 | 25.75191747 | 10.29202685 | 3.363455221 | 1.85316E-05 | 0.000134922 | K00924 |
| *At3g01520* | 469.7101775 | 979.7681897 | 2.085899426 | 1.060669598 | 1.85378E-05 | 0.00013493 | - |
| *SWI3B* | 347.3563472 | 712.0827568 | 2.050006463 | 1.035628458 | 1.88252E-05 | 0.000136871 | K11649 |
| *uncharacterized protein_41885* | 18.06278206 | 66.03590997 | 3.655910245 | 1.870230652 | 1.88513E-05 | 0.000137023 | - |
| *C1GALT1* | 234.5827226 | 945.7191565 | 4.031495354 | 2.01131506 | 1.89423E-05 | 0.000137608 | - |
| *HSP12* | 61.84714465 | 228.7377288 | 3.698436364 | 1.886915453 | 1.90109E-05 | 0.000138069 | K13993 |
| *AKR4C9* | 127.2714413 | 281.442018 | 2.211352486 | 1.144929007 | 1.92498E-05 | 0.000139688 | - |
| *FPS* | 239.7885012 | 495.6332824 | 2.066960176 | 1.047510592 | 1.9329E-05 | 0.000140147 | K00787 |
| *GPXMC1* | 0.301350363 | 70.59745116 | 234.2703374 | 7.872030486 | 1.94824E-05 | 0.000141182 | K00432 |
| *At5g61540* | 73.01069536 | 175.2229551 | 2.39996283 | 1.263012062 | 2.00695E-05 | 0.000145077 | K01444 |
| *LWD1* | 156.1109326 | 339.9876221 | 2.177859145 | 1.12291065 | 2.01342E-05 | 0.000145465 | K11805 |
| *AS* | 7.033421009 | 38.94032446 | 5.536470006 | 2.468966423 | 2.09311E-05 | 0.000150685 | K08237 |
| *uncharacterized protein_38539* | 231.1873149 | 1356.730993 | 5.868535624 | 2.553000552 | 2.10544E-05 | 0.000151408 | K14315 |
| *PANC* | 119.0436613 | 263.8095537 | 2.216073924 | 1.148006008 | 2.10937E-05 | 0.000151649 | K01918 |
| *CBG* | 61.69279478 | 161.0624799 | 2.61071784 | 1.384446543 | 2.113E-05 | 0.000151827 | K05350 |
| *FAB1B* | 149.978522 | 480.8648491 | 3.20622475 | 1.680875559 | 2.18246E-05 | 0.000156604 | K00921 |
| *PLIP2* | 14.8681585 | 57.35784181 | 3.857763677 | 1.947764768 | 2.19227E-05 | 0.000157266 | - |
| *AMT1-1* | 271.846041 | 663.2082578 | 2.439646556 | 1.286672153 | 2.22435E-05 | 0.000159307 | K03320 |
| *Rnf4* | 50.86077711 | 131.6125396 | 2.587702097 | 1.37167154 | 2.25845E-05 | 0.000161617 | - |
| *uncharacterized protein_42751* | 44.39646153 | 239.671457 | 5.398436018 | 2.432541504 | 2.26713E-05 | 0.000162195 | - |
| *DDB_G0268948* | 9.290245982 | 223.5780352 | 24.06588972 | 4.588917856 | 2.28827E-05 | 0.000163529 | - |
| *uncharacterized protein_40470* | 68.62233278 | 167.4872619 | 2.4407107 | 1.287301301 | 2.34463E-05 | 0.000167376 | K13456 |
| *MIC60* | 245.7060725 | 501.8521973 | 2.042490005 | 1.030329018 | 2.36671E-05 | 0.000168815 | K17785 |
| *SOK1* | 34.16245959 | 210.0421059 | 6.1483309 | 2.620194812 | 2.38892E-05 | 0.000170307 | - |
| *CYP750A1* | 0 | 93.37079214 | Inf | Inf | 2.39945E-05 | 0.000171011 | - |
| *LBD1* | 1.729468893 | 110.7926583 | 64.06166581 | 6.001389408 | 2.43201E-05 | 0.000173145 | - |
| *I-2* | 131.7833511 | 286.933837 | 2.177314771 | 1.122549991 | 2.44615E-05 | 0.000174104 | K16833 |
| *WRKY46* | 130.0752866 | 1548.678398 | 11.90601565 | 3.573618791 | 2.48567E-05 | 0.000176488 | K18835 |
| *At3g02290* | 239.5189778 | 488.3281583 | 2.038786917 | 1.027711001 | 2.49146E-05 | 0.000176852 | - |
| *uncharacterized protein_14259* | 64.94855579 | 160.1624728 | 2.465989748 | 1.302166802 | 2.50746E-05 | 0.000177844 | - |
| *AMT3-1* | 84.06059291 | 283.0918131 | 3.367711353 | 1.75176849 | 2.52616E-05 | 0.000179073 | K03320 |
| *CDA1* | 246.5176551 | 538.0918297 | 2.182771978 | 1.126161428 | 2.54802E-05 | 0.000180477 | K01489 |
| *HSL1* | 29.10672223 | 741.9458031 | 25.49053092 | 4.671889517 | 2.54936E-05 | 0.000180524 | - |
| *UBP3* | 79.04607641 | 188.5818624 | 2.385720721 | 1.254425167 | 2.56007E-05 | 0.000181185 | K11842 |
| *CYCB1-5* | 11.27946809 | 49.25175958 | 4.366496646 | 2.126476232 | 2.57047E-05 | 0.000181872 | K05868 |
| *At1g66480* | 23.24002222 | 75.59979495 | 3.253000114 | 1.701770873 | 2.63157E-05 | 0.000185846 | - |
| *IRX15* | 106.3849255 | 410.8276411 | 3.861709159 | 1.949239513 | 2.63325E-05 | 0.000185914 | - |
| *SRL2* | 254.5668599 | 516.3952022 | 2.028524854 | 1.020430979 | 2.67977E-05 | 0.000188795 | - |
| *At3g20240* | 5.279850793 | 33.01546958 | 6.253106551 | 2.644573101 | 2.68895E-05 | 0.000189391 | K14684 |
| *NPF7.3* | 0 | 13.13529747 | Inf | Inf | 2.70551E-05 | 0.000190506 | K14638 |
| *XYL1* | 26.31770399 | 344.3698735 | 13.08510323 | 3.709853401 | 2.71035E-05 | 0.000190796 | K15925 |
| *uncharacterized protein_13992* | 63.8519703 | 156.8198528 | 2.455990818 | 1.296305167 | 2.71641E-05 | 0.000191171 | - |
| *CYP71AU50* | 41.18282181 | 113.5684492 | 2.757665555 | 1.4634475 | 2.74658E-05 | 0.000193192 | - |
| *uncharacterized protein_11987* | 18.95706299 | 65.68792536 | 3.465089787 | 1.792892736 | 2.75915E-05 | 0.000194024 | - |
| *CKB1* | 115.2115934 | 256.0287339 | 2.222248009 | 1.152019834 | 2.77883E-05 | 0.000195303 | K03115 |
| *CHS* | 0 | 13.05633487 | Inf | Inf | 2.7874E-05 | 0.000195854 | K00660 |
| *MYB78* | 1.820913007 | 70.9363028 | 38.95644796 | 5.283790234 | 2.79456E-05 | 0.000196264 | K09422 |
| *TIFY10B* | 0 | 13.04346129 | Inf | Inf | 2.80103E-05 | 0.000196654 | K13464 |
| *yihQ* | 0 | 13.0403175 | Inf | Inf | 2.80437E-05 | 0.000196784 | - |
| *CAT7* | 1.271099924 | 20.08310511 | 15.79978469 | 3.981832993 | 2.84792E-05 | 0.00019968 | - |
| *AMPD* | 337.0254679 | 733.8363939 | 2.177391514 | 1.12260084 | 2.87631E-05 | 0.000201479 | K01490 |
| *yuiD* | 92.31628694 | 212.3765539 | 2.300531801 | 1.201967399 | 2.91195E-05 | 0.00020379 | K09775 |
| *NPF5.2* | 376.2051084 | 1388.674277 | 3.691269061 | 1.884116901 | 2.91742E-05 | 0.000204118 | K14638 |
| *PGDH1* | 964.9127565 | 2413.859015 | 2.501634473 | 1.322871005 | 2.93083E-05 | 0.000205002 | K00058 |
| *MYB1* | 139.6185845 | 507.42697 | 3.634379848 | 1.861709211 | 2.9408E-05 | 0.000205591 | K09422 |
| *At2g46620* | 234.5629344 | 853.5982612 | 3.639101223 | 1.863582181 | 2.96961E-05 | 0.00020733 | K08900 |
| *PFDN6* | 51.70052608 | 130.454265 | 2.523267651 | 1.335293245 | 2.97322E-05 | 0.000207515 | K04798 |
| *TIM23-2* | 547.8018274 | 1811.060269 | 3.306050068 | 1.725108573 | 3.07606E-05 | 0.000214194 | K17794 |
| *SRPK3* | 165.5817743 | 347.6133492 | 2.099345478 | 1.069939603 | 3.10072E-05 | 0.000215741 | K08832 |
| *PPX2* | 120.7220109 | 262.6318066 | 2.17550888 | 1.121352905 | 3.12175E-05 | 0.000217032 | K15423 |
| *NCER2* | 134.0400239 | 285.0106717 | 2.126310212 | 1.08835209 | 3.14773E-05 | 0.00021855 | K12349 |
| *At5g64030* | 94.18642701 | 214.3985521 | 2.27632111 | 1.186704087 | 3.16219E-05 | 0.000219438 | - |
| *At3g01520* | 0 | 17.5458909 | Inf | Inf | 3.18689E-05 | 0.000220978 | - |
| *At1g60710* | 3.707400507 | 27.8476957 | 7.511380453 | 2.909078073 | 3.33609E-05 | 0.000230716 | - |
| *FDH1* | 1.048258864 | 18.34007047 | 17.49574566 | 4.128932248 | 3.34011E-05 | 0.000230873 | K00122 |
| *KING1* | 9.315867643 | 42.77112864 | 4.591212572 | 2.19887523 | 3.40155E-05 | 0.000234628 | - |
| *LPPD* | 484.3240073 | 1117.701347 | 2.307755409 | 1.206490326 | 3.41686E-05 | 0.00023556 | - |
| *BHLH121* | 196.5219724 | 403.4073682 | 2.052734171 | 1.037546811 | 3.59841E-05 | 0.000246785 | - |
| *CYP21-4* | 75.64257102 | 175.876749 | 2.325102738 | 1.217294465 | 3.60414E-05 | 0.000247114 | K12733 |
| *TD1* | 28.29259492 | 249.3667006 | 8.813850453 | 3.139772418 | 3.65285E-05 | 0.000250194 | K01754 |
| *LOX1.1* | 0 | 78.29980569 | Inf | Inf | 3.714E-05 | 0.00025425 | K15718 |
| *NTAQ1* | 61.52156898 | 150.0587425 | 2.439124115 | 1.286363172 | 3.77904E-05 | 0.000258433 | - |
| *PYL8* | 235.3883522 | 472.2314324 | 2.006180119 | 1.00445114 | 3.78925E-05 | 0.000259065 | K14496 |
| *RABA1F* | 22.80727492 | 77.98719213 | 3.419399837 | 1.77374313 | 3.79298E-05 | 0.000259253 | K07904 |
| *NUDT15* | 222.6586805 | 450.6971299 | 2.024161505 | 1.017324406 | 3.84427E-05 | 0.000262486 | - |
| *CBSX1* | 1426.998738 | 4529.37421 | 3.174056213 | 1.666327679 | 3.96161E-05 | 0.000270148 | - |
| *EOBII* | 2.474857056 | 24.35047394 | 9.839143591 | 3.298532747 | 3.96491E-05 | 0.000270233 | K09422 |
| *OFUT20* | 4.45278867 | 39.32044329 | 8.830520873 | 3.142498539 | 3.98631E-05 | 0.000271551 | - |
| *ERV1* | 97.73513637 | 216.4007602 | 2.214155198 | 1.146756349 | 4.05081E-05 | 0.000275589 | K17783 |
| *At5g27430* | 183.6104652 | 376.6378538 | 2.051287509 | 1.036529715 | 4.05942E-05 | 0.000276104 | K12948 |
| *At4g33300* | 6.470921075 | 101.7445059 | 15.72334212 | 3.974836001 | 4.10056E-05 | 0.000278542 | - |
| *uncharacterized protein_45656* | 486.1528026 | 1203.012121 | 2.474555562 | 1.307169436 | 4.12115E-05 | 0.000279869 | - |
| *AERO2* | 174.9394871 | 360.4252785 | 2.060285442 | 1.04284423 | 4.18731E-05 | 0.000283996 | K10950 |
| *ZIP5* | 2.81628423 | 25.25250063 | 8.966602292 | 3.164561409 | 4.19303E-05 | 0.000284311 | K14709 |
| *CIPK26* | 786.8750273 | 2073.668888 | 2.635321768 | 1.397979123 | 4.19892E-05 | 0.000284637 | K07198 |
| *UGT84A23* | 7.244971576 | 103.5716664 | 14.29566222 | 3.837505546 | 4.22143E-05 | 0.000286016 | K13691 |
| *GPA3* | 103.4718358 | 232.0495442 | 2.242634843 | 1.165194733 | 4.29195E-05 | 0.000290345 | - |
| *GCH1* | 188.2305385 | 550.2279085 | 2.923159615 | 1.547528608 | 4.31179E-05 | 0.000291538 | K01495 |
| *SRG1* | 1.218212282 | 19.39049151 | 15.91716961 | 3.992511913 | 4.43969E-05 | 0.000299725 | - |
| *At1g75220* | 31.54618748 | 90.87120606 | 2.880576492 | 1.526357568 | 4.44943E-05 | 0.000300228 | K08145 |
| *MAVI* | 0.314161193 | 14.62370584 | 46.54841573 | 5.540660162 | 4.49761E-05 | 0.000303324 | - |
| *uncharacterized protein_21942* | 90.69690639 | 202.6935763 | 2.234845535 | 1.16017512 | 4.50354E-05 | 0.000303568 | - |
| *CYCU1-1* | 110.1266695 | 262.1794107 | 2.380707705 | 1.251390503 | 4.53352E-05 | 0.000305385 | - |
| *PDC4* | 66.32403464 | 193.1063981 | 2.911559876 | 1.541792288 | 4.54223E-05 | 0.000305765 | K01568 |
| *VAC14* | 232.5450499 | 587.5443732 | 2.526583014 | 1.337187582 | 4.83069E-05 | 0.000323565 | K15305 |
| *ASNSD1* | 95.65487031 | 257.9032374 | 2.696185114 | 1.430919552 | 4.8317E-05 | 0.000323565 | - |
| *uncharacterized protein_22957* | 0.367048835 | 37.12026014 | 101.1316658 | 6.660090986 | 5.03358E-05 | 0.000336371 | - |
| *GAPC* | 142.9207517 | 808.3229844 | 5.655742605 | 2.499716464 | 5.06101E-05 | 0.000338033 | K00134 |
| *HSP12* | 8.226011629 | 61.88095475 | 7.522595097 | 2.911230439 | 5.06263E-05 | 0.000338056 | K13993 |
| *brcc3* | 166.6859897 | 341.9802865 | 2.051643855 | 1.036780315 | 5.1362E-05 | 0.000342621 | K11864 |
| *dph4* | 68.39316241 | 162.1841597 | 2.371350498 | 1.245708916 | 5.22275E-05 | 0.000347691 | - |
| *At5g01750* | 181.9902799 | 368.6101244 | 2.025438526 | 1.018234299 | 5.26855E-05 | 0.000350563 | - |
| *DGK1* | 23.66008268 | 149.5467763 | 6.320636249 | 2.660069791 | 5.27077E-05 | 0.000350622 | K00901 |
| *MAPKKK17* | 0 | 15.08602409 | Inf | Inf | 5.28107E-05 | 0.000351219 | - |
| *Dctpp1* | 25.76764294 | 77.38124496 | 3.003039321 | 1.586423365 | 5.30667E-05 | 0.000352654 | K16904 |
| *TSK* | 11.97500929 | 47.70354334 | 3.983591344 | 1.994069656 | 5.31395E-05 | 0.000353049 | - |
| *STOP1* | 36.69780598 | 109.8104195 | 2.992288408 | 1.581249234 | 5.34854E-05 | 0.000355202 | - |
| *uncharacterized protein_04865* | 212.4132923 | 542.0264183 | 2.551753765 | 1.351489121 | 5.44032E-05 | 0.000360899 | - |
| *WIP5* | 123.2229855 | 263.6544545 | 2.139653194 | 1.097376976 | 5.47286E-05 | 0.000362875 | - |
| *DNAJB6* | 0 | 17.20926415 | Inf | Inf | 5.48183E-05 | 0.000363378 | - |
| *uncharacterized protein_42580* | 37.2043776 | 101.4645239 | 2.727220033 | 1.447431102 | 5.49194E-05 | 0.000363957 | - |
| *CRJ34* | 3.168877915 | 52.45592682 | 16.55347041 | 4.049061803 | 5.55296E-05 | 0.000367815 | - |
| *COR2* | 172.212411 | 350.6659848 | 2.036241075 | 1.025908375 | 5.59281E-05 | 0.000370176 | - |
| *MPT3* | 13.51563258 | 575.4933863 | 42.57983362 | 5.412098407 | 5.62526E-05 | 0.00037195 | K15102 |
| *SEC* | 3.183333065 | 25.88561766 | 8.131608326 | 3.023540727 | 5.72509E-05 | 0.000378077 | K09667 |
| *AGD5* | 171.4535595 | 352.9312257 | 2.058465434 | 1.041569223 | 5.7463E-05 | 0.000379205 | K12486 |
| *At1g06550* | 255.2839858 | 581.292217 | 2.277041449 | 1.187160553 | 5.75388E-05 | 0.000379503 | K05605 |
| *UDP-GALT1* | 227.6553531 | 635.0508926 | 2.789527608 | 1.48002083 | 5.79778E-05 | 0.000382111 | - |
| *uncharacterized protein_04846* | 32.82705755 | 167.1655411 | 5.092309624 | 2.348320141 | 5.84449E-05 | 0.000384901 | - |
| *PMLN* | 98.61877494 | 398.525665 | 4.041072962 | 2.0147384 | 5.88896E-05 | 0.000387636 | - |
| *uncharacterized protein_31414* | 31.58474395 | 90.24276143 | 2.857162989 | 1.514583338 | 5.99481E-05 | 0.000394289 | - |
| *MRS2-B* | 198.8970116 | 543.3790204 | 2.731961712 | 1.449937265 | 6.08074E-05 | 0.000399761 | K16075 |
| *At5g42350* | 133.1256066 | 289.9164396 | 2.177766149 | 1.122849044 | 6.12185E-05 | 0.000402262 | - |
| *uncharacterized protein_34470* | 0.668399198 | 99.349007 | 148.6372325 | 7.215651735 | 6.17306E-05 | 0.000405325 | - |
| *uncharacterized protein_29465* | 64.79305752 | 153.1293155 | 2.363359923 | 1.240839359 | 6.3278E-05 | 0.000415174 | - |
| *PAP2* | 108.5690745 | 543.9441047 | 5.010120119 | 2.324845193 | 6.35056E-05 | 0.00041646 | - |
| *ABCI20* | 191.1769473 | 462.9972053 | 2.421825496 | 1.276094916 | 6.39526E-05 | 0.000419079 | K12608 |
| *sec61a* | 50.06898282 | 133.7356995 | 2.671028888 | 1.417395579 | 6.41183E-05 | 0.00042006 | K10956 |
| *Fra a 1.06* | 0 | 253.4678335 | Inf | Inf | 6.44025E-05 | 0.000421817 | - |
| *At4g33300* | 11.37078822 | 46.30848687 | 4.072583709 | 2.025944353 | 6.68847E-05 | 0.000437314 | - |
| *SWEET16* | 5.279850793 | 31.10488587 | 5.89124335 | 2.558572148 | 6.69185E-05 | 0.000437427 | K15382 |
| *uncharacterized protein_48480* | 230.8046907 | 593.9456501 | 2.573369061 | 1.363658377 | 6.88154E-05 | 0.000449035 | K19525 |
| *Phrf1* | 107.6043819 | 233.4779715 | 2.169781262 | 1.11754961 | 6.92737E-05 | 0.000451702 | - |
| *DIR21* | 10.79535348 | 482.6622513 | 44.71018502 | 5.482531611 | 6.93598E-05 | 0.000452039 | - |
| *UCC1* | 0 | 12.81955299 | Inf | Inf | 6.99748E-05 | 0.000455822 | - |
| *ABCC4* | 341.6564316 | 767.7675817 | 2.247191947 | 1.168123359 | 7.00369E-05 | 0.000456114 | - |
| *uncharacterized protein_36804* | 66.74890407 | 222.6539605 | 3.335694624 | 1.737987219 | 7.03544E-05 | 0.000458069 | - |
| *DELTA-ADR* | 209.2619102 | 499.8419934 | 2.388595196 | 1.256162376 | 7.12191E-05 | 0.000463126 | K12396 |
| *GPX4* | 1258.985361 | 3026.017383 | 2.403536591 | 1.265158767 | 7.19715E-05 | 0.000467673 | K00432 |
| *UGE1* | 595.0452518 | 1687.956302 | 2.836685609 | 1.504206265 | 7.413E-05 | 0.00048073 | K01784 |
| *APD2* | 8.674734425 | 39.35277645 | 4.536481985 | 2.18157393 | 7.41451E-05 | 0.00048073 | - |
| *WRKY42* | 0.602700725 | 15.73687316 | 26.11059269 | 4.706563301 | 7.42722E-05 | 0.000481144 | - |
| *RH26* | 47.76711986 | 121.4780849 | 2.543131871 | 1.346606273 | 7.4281E-05 | 0.000481144 | K17679 |
| *AL1* | 603.5470203 | 1586.702701 | 2.62896286 | 1.394493761 | 7.46155E-05 | 0.000483067 | - |
| *ALDH10A9* | 430.6933847 | 889.6264595 | 2.065567968 | 1.046538533 | 7.51869E-05 | 0.000486647 | K00130 |
| *PHR2* | 43.12384127 | 112.0672796 | 2.598731381 | 1.377807516 | 7.52147E-05 | 0.000486707 | - |
| *ATL5* | 59.49227007 | 252.3949122 | 4.242482458 | 2.084908695 | 7.90178E-05 | 0.000509565 | - |
| *MTM1* | 128.7957171 | 270.065059 | 2.096848133 | 1.068222376 | 7.94216E-05 | 0.000511918 | K15119 |
| *AMO* | 8.907345638 | 41.56937485 | 4.666864466 | 2.22245357 | 8.04891E-05 | 0.000518419 | K00276 |
| *uncharacterized protein_06115* | 1.545184306 | 19.66102924 | 12.72406739 | 3.669488013 | 8.05665E-05 | 0.000518791 | - |
| *RFS6* | 66.81956372 | 158.309671 | 2.369211383 | 1.244406923 | 8.1263E-05 | 0.00052302 | K06617 |
| *SIS8* | 132.0991566 | 325.8225483 | 2.466499837 | 1.302465192 | 8.16465E-05 | 0.000525282 | - |
| *PAT10* | 40.48055113 | 104.7587067 | 2.587877482 | 1.371769318 | 8.16543E-05 | 0.000525282 | K18932 |
| *alaS* | 95.93084697 | 370.2090413 | 3.859124077 | 1.94827343 | 8.21318E-05 | 0.000527967 | - |
| *JOX4* | 18.58697348 | 62.07769319 | 3.339849452 | 1.739783073 | 8.30477E-05 | 0.000533725 | - |
| *DEGP7* | 136.2495705 | 284.7982271 | 2.090268806 | 1.063688483 | 8.35786E-05 | 0.000536875 | - |
| *PAP10* | 92.06261508 | 200.4289386 | 2.177093692 | 1.122403496 | 8.36569E-05 | 0.000537116 | - |
| *GSTU17* | 298.7978006 | 1623.489687 | 5.433405745 | 2.441856786 | 8.5701E-05 | 0.000549437 | K00799 |
| *AHP1* | 0.668399198 | 16.52953057 | 24.73002753 | 4.62819194 | 8.6059E-05 | 0.000551464 | K14490 |
| *TIFY10A* | 3.563192731 | 51.96909804 | 14.58498093 | 3.866411595 | 8.64714E-05 | 0.000553837 | K13464 |
| *At3g02910* | 24.14394933 | 165.6228775 | 6.859808859 | 2.778168378 | 8.81651E-05 | 0.000563863 | K19761 |
| *ROQ1* | 39.77207512 | 103.2021899 | 2.594840464 | 1.375645841 | 8.84131E-05 | 0.000565175 | - |
| *CMLN* | 2.240849484 | 21.763294 | 9.7120731 | 3.27977928 | 9.27197E-05 | 0.000590984 | - |
| *CSI1* | 1305.569804 | 2694.701858 | 2.064004429 | 1.045446067 | 9.34588E-05 | 0.000595407 | - |
| *DCR* | 1.66377042 | 839.140789 | 504.3609254 | 8.978312699 | 9.37736E-05 | 0.000597124 | - |
| *SWEET16* | 0 | 44.94069329 | Inf | Inf | 9.49311E-05 | 0.0006031 | K15382 |
| *At1g18000* | 63.6678097 | 168.7724517 | 2.650828614 | 1.406443398 | 9.55241E-05 | 0.000606074 | - |
| *PME1* | 86.83884481 | 189.9175025 | 2.187010927 | 1.128960428 | 9.5681E-05 | 0.000606924 | K01051 |
| *IBR5* | 741.3779195 | 1512.979835 | 2.040767326 | 1.029111706 | 9.61902E-05 | 0.000609567 | K04459 |
| *PME1* | 165.0305931 | 483.9658574 | 2.93258267 | 1.552171779 | 9.68542E-05 | 0.000613479 | K01051 |
| *RSH1* | 47.13259214 | 175.6014651 | 3.725690805 | 1.89750795 | 9.99841E-05 | 0.000632849 | - |
| *E134* | 382.0844996 | 1135.630497 | 2.972197244 | 1.571529861 | 0.000100009 | 0.000632853 | - |
| *uncharacterized protein_24981* | 840.1545809 | 2007.607523 | 2.389569215 | 1.256750557 | 0.000100397 | 0.000635004 | - |
| *uncharacterized protein_09565* | 97.65613115 | 205.665634 | 2.106018655 | 1.074518216 | 0.000100695 | 0.000636733 | - |
| *Ccdc22* | 22.96466546 | 71.48232975 | 3.112709387 | 1.638170888 | 0.000101102 | 0.000639159 | - |
| *CPIJ013394* | 22.98699849 | 71.44341059 | 3.107992139 | 1.635982855 | 0.000101168 | 0.000639424 | - |
| *RAMDAZC7* | 0 | 18.93943165 | Inf | Inf | 0.00010126 | 0.00063985 | - |
| *RDN1* | 133.5226859 | 274.6190824 | 2.056722275 | 1.040346996 | 0.000102672 | 0.000648149 | - |
| *RAMDAZC7* | 0 | 76.33286276 | Inf | Inf | 0.000102918 | 0.000648997 | - |
| *FH6* | 78.57908568 | 173.7619219 | 2.21129987 | 1.144894679 | 0.000103205 | 0.000650484 | - |
| *At3g10130* | 828.6069902 | 1746.610612 | 2.107887856 | 1.075798114 | 0.000103215 | 0.000650484 | - |
| *CML11* | 117.6286016 | 430.9623464 | 3.663754738 | 1.873322929 | 0.000103358 | 0.000651234 | K13448 |
| *B3GALT2* | 72.33658675 | 165.9213007 | 2.293739699 | 1.197701679 | 0.000104637 | 0.000658975 | - |
| *At3g05230* | 119.8196041 | 247.9761655 | 2.069579242 | 1.049337489 | 0.000105844 | 0.000665942 | K12948 |
| *CXE15* | 0 | 13.89885854 | Inf | Inf | 0.000106132 | 0.000667432 | - |
| *CHI4* | 10.38835181 | 79.852675 | 7.686751129 | 2.94237396 | 0.000107114 | 0.000672966 | K01183 |
| *UGT86A1* | 0.301350363 | 27.38700552 | 90.88094426 | 6.505905919 | 0.00010726 | 0.000673582 | - |
| *SEC22* | 144.2635074 | 379.0412876 | 2.627423209 | 1.393648599 | 0.000107862 | 0.000676699 | K08517 |
| *DALL2* | 0 | 11.37300219 | Inf | Inf | 0.000108239 | 0.000678578 | - |
| *ADH1* | 34.29689721 | 92.68173953 | 2.702335986 | 1.434207058 | 0.000109351 | 0.000684898 | K18857 |
| *CYC2* | 129.2820959 | 269.350748 | 2.083434261 | 1.058963579 | 0.000111833 | 0.00069978 | - |
| *CYP75B137* | 35.54237547 | 115.6254077 | 3.253170509 | 1.70184644 | 0.000112193 | 0.000701868 | K05280 |
| *GLOX1* | 0 | 29.10581515 | Inf | Inf | 0.00011364 | 0.000710246 | - |
| *uncharacterized protein_26439* | 83.91852537 | 223.4291778 | 2.662453574 | 1.412756369 | 0.00011563 | 0.000721655 | - |
| *PIP5K9* | 27.82281148 | 80.05559512 | 2.877336648 | 1.524734026 | 0.000117446 | 0.000731777 | K00889 |
| *GFAT1* | 157.3968878 | 903.6983432 | 5.741526125 | 2.521434263 | 0.000119998 | 0.000746265 | K00820 |
| *At4g04980* | 162.7606811 | 446.3800265 | 2.742554426 | 1.455520251 | 0.000122169 | 0.000758335 | K02184 |
| *GPX4* | 24.64416341 | 72.1458599 | 2.927502902 | 1.549670601 | 0.000122319 | 0.000758786 | K00432 |
| *At1g56220* | 0.615511556 | 271.9052686 | 441.7549367 | 8.787102447 | 0.000123101 | 0.0007634 | - |
| *uncharacterized protein_36805* | 471.7549234 | 2085.825094 | 4.421416694 | 2.144508707 | 0.000123759 | 0.000767303 | - |
| *MAPKKK17* | 6.983698025 | 40.37420705 | 5.781207451 | 2.531370842 | 0.00012483 | 0.000773484 | - |
| *ACA5* | 2.894793533 | 26.94222037 | 9.307130219 | 3.218336393 | 0.000125176 | 0.000774807 | K01537 |
| *At4g26910* | 103.1515932 | 219.3988828 | 2.126955832 | 1.088790075 | 0.000125386 | 0.000775745 | K00658 |
| *PTI5* | 1.349609227 | 17.64097034 | 13.07116904 | 3.708316271 | 0.00012621 | 0.000780658 | - |
| *Rnf14* | 552.3994481 | 1343.227973 | 2.431624394 | 1.281920397 | 0.000129867 | 0.000802151 | K11971 |
| *BETV1D* | 0.628322387 | 347.0595891 | 552.3591015 | 9.109462691 | 0.000130154 | 0.000803735 | - |
| *DRTI* | 0.916861919 | 19.05386476 | 20.78160775 | 4.377235366 | 0.000132253 | 0.000815741 | - |
| *Ctps1* | 87.9016828 | 349.2969288 | 3.973722888 | 1.990491268 | 0.000132629 | 0.000817867 | K01937 |
| *TMEM135* | 120.8105383 | 250.1156348 | 2.070313056 | 1.049848937 | 0.00013277 | 0.000818548 | - |
| *MYOB5* | 149.6348024 | 1026.296121 | 6.858672612 | 2.777929392 | 0.000133018 | 0.000819882 | K11844 |
| *OFUT19* | 88.36206803 | 247.6268963 | 2.802411734 | 1.486668934 | 0.000134602 | 0.000828483 | - |
| *At4g08455* | 1.937854803 | 28.93197205 | 14.92989671 | 3.90013228 | 0.000136198 | 0.000836751 | K10523 |
| *MIMI_L728* | 5.933918822 | 32.24866525 | 5.434632022 | 2.442182354 | 0.000137181 | 0.000842086 | - |
| *At1g18250* | 7.818885983 | 42.62628241 | 5.451707891 | 2.446708263 | 0.000138389 | 0.000848818 | - |
| *NLRP5* | 127.7760924 | 261.7094208 | 2.0481877 | 1.034347933 | 0.000139449 | 0.000855123 | - |
| *SUR1* | 6.041214444 | 31.21608216 | 5.167186573 | 2.369378976 | 0.00014099 | 0.00086417 | K00815 |
| *CPK3* | 37.19826802 | 168.2414109 | 4.522829149 | 2.177225499 | 0.000142555 | 0.000873356 | K13412 |
| *XLG1* | 28.53432817 | 79.28554752 | 2.778602218 | 1.474359314 | 0.000143479 | 0.00087861 | - |
| *uncharacterized protein_39668* | 0.367048835 | 13.09667672 | 35.68101969 | 5.15708494 | 0.000146337 | 0.000895275 | - |
| *CAD* | 118.7282278 | 705.9828216 | 5.946208706 | 2.571970101 | 0.000147367 | 0.000900952 | K00083 |
| *At5g04720* | 28.37578922 | 79.25634528 | 2.793097477 | 1.481865923 | 0.000147714 | 0.000902865 | - |
| *uncharacterized protein_37327* | 62.43349794 | 142.48126 | 2.28212842 | 1.190379977 | 0.000147772 | 0.000903007 | - |
| *ILL6* | 25.83194506 | 82.65250485 | 3.199623748 | 1.677902265 | 0.00014889 | 0.000909209 | K14664 |
| *POX1* | 3.983129208 | 25.83736659 | 6.486700591 | 2.697484849 | 0.000150333 | 0.000917169 | K00318 |
| *DDB_G0268948* | 9.442579592 | 218.06552 | 23.09385035 | 4.529436823 | 0.000154052 | 0.000938123 | - |
| *ABCG34* | 18.14597636 | 83.87827827 | 4.622417478 | 2.208647564 | 0.000154582 | 0.000940915 | - |
| *VAR3* | 83.4372317 | 177.711408 | 2.129881401 | 1.090773099 | 0.000155202 | 0.000944033 | - |
| *MYB106* | 66.03270239 | 171.788393 | 2.601565388 | 1.379379968 | 0.000155874 | 0.000947685 | K09422 |
| *PHOS34* | 100.2567997 | 211.3391188 | 2.107977906 | 1.075859746 | 0.000157088 | 0.000954405 | - |
| *NHL6* | 0 | 49.12932422 | Inf | Inf | 0.000157645 | 0.000957346 | - |
| *ATG2* | 22.83302056 | 68.41023463 | 2.996109711 | 1.583090453 | 0.000163983 | 0.000993779 | K17906 |
| *ORP1A* | 257.2723119 | 600.7008449 | 2.334883379 | 1.223350493 | 0.000164486 | 0.000996138 | K20456 |
| *DBR* | 131.7272706 | 264.0863726 | 2.004796511 | 1.003455809 | 0.000167594 | 0.001012866 | K07119 |
| *uncharacterized protein_35463* | 136.4541437 | 462.5698924 | 3.389929247 | 1.761255162 | 0.000168489 | 0.001017111 | - |
| *ISY1* | 48.1277154 | 114.8217037 | 2.385770917 | 1.254455522 | 0.0001696 | 0.001023222 | - |
| *ADH2* | 17.24204925 | 55.48435024 | 3.217967275 | 1.686149655 | 0.000169618 | 0.001023222 | K18857 |
| *Abhd11* | 67.68620674 | 151.5022425 | 2.238303043 | 1.162405376 | 0.000170783 | 0.001029544 | - |
| *pcp* | 69.12077856 | 153.3904283 | 2.219165227 | 1.150017087 | 0.00017167 | 0.001034052 | K01304 |
| *MIMI_L728* | 42.86573237 | 200.6493246 | 4.680879421 | 2.226779602 | 0.000172627 | 0.001038757 | - |
| *At2g21160* | 1055.652561 | 2239.893912 | 2.121809765 | 1.085295314 | 0.00017485 | 0.001050935 | K13249 |
| *RUS6* | 30.14369061 | 81.75808253 | 2.712278453 | 1.439505299 | 0.000175093 | 0.001052155 | - |
| *CYP707A1* | 316.6455913 | 1025.173781 | 3.237606362 | 1.694927589 | 0.000175309 | 0.001052976 | K09843 |
| *GOLS4* | 15.41645124 | 256.0414601 | 16.60832678 | 4.05383483 | 0.000175656 | 0.001054574 | K18819 |
| *uncharacterized protein_31766* | 57.14893394 | 149.8786311 | 2.622597148 | 1.390996215 | 0.000179415 | 0.001074694 | - |
| *LBD25* | 0 | 10.80435899 | Inf | Inf | 0.000179542 | 0.001075213 | - |
| *SDL5A* | 6.038049787 | 31.50445784 | 5.217654533 | 2.383401425 | 0.000182099 | 0.001088739 | - |
| *CNGC8* | 73.00309366 | 161.7023819 | 2.215007252 | 1.147311422 | 0.000183166 | 0.001093686 | K05391 |
| *At5g03795* | 0 | 10.68829781 | Inf | Inf | 0.000187693 | 0.001119699 | - |
| *POB1* | 12.06632942 | 43.90831558 | 3.638912386 | 1.863507316 | 0.000188417 | 0.001123764 | - |
| *CIPK5* | 0 | 10.66903717 | Inf | Inf | 0.000189088 | 0.001127514 | K07198 |
| *ACS* | 688.613655 | 1609.4752 | 2.337268784 | 1.224823653 | 0.000193451 | 0.001150246 | K01895 |
| *PILS2* | 40.78050514 | 102.2118687 | 2.506390451 | 1.325611178 | 0.000193512 | 0.001150246 | - |
| *GDPDL1* | 276.681978 | 1415.431644 | 5.115734876 | 2.354941498 | 0.000194247 | 0.001154095 | - |
| *ERF114* | 0 | 13.63136513 | Inf | Inf | 0.000195199 | 0.001159491 | - |
| *DAO* | 0.628322387 | 43.22990522 | 68.80210879 | 6.104380879 | 0.000195536 | 0.00116097 | - |
| *EDS1B* | 87.23772064 | 182.7309929 | 2.094632821 | 1.066697369 | 0.000196007 | 0.001162953 | K18875 |
| *PNC1* | 2.764916926 | 139.4269356 | 50.4271699 | 5.656129355 | 0.000196027 | 0.001162953 | K00430 |
| *At2g40430* | 33.2099579 | 86.75354192 | 2.612274975 | 1.385306767 | 0.000196992 | 0.001167246 | K14840 |
| *At2g29640* | 27.47946381 | 113.3899659 | 4.126352925 | 2.04486722 | 0.000198704 | 0.001176866 | K15235 |
| *RTNLB22* | 46.56171841 | 110.5547392 | 2.374369825 | 1.247544663 | 0.000201732 | 0.001192117 | - |
| *CSA* | 3.07907812 | 28.63538875 | 9.299987734 | 3.217228813 | 0.000201902 | 0.001192807 | K00432 |
| *UGT89B2* | 0.668399198 | 46.00278347 | 68.82531218 | 6.104867344 | 0.000202601 | 0.001196447 | - |
| *NIT4A* | 2.907604364 | 22.26746982 | 7.658356171 | 2.937034758 | 0.000204593 | 0.001206861 | K13035 |
| *CYP750A1* | 0 | 105.0734372 | Inf | Inf | 0.000207425 | 0.001223291 | - |
| *HAC12* | 76.99562109 | 167.0197739 | 2.169211333 | 1.117170613 | 0.000208439 | 0.001228723 | K04498 |
| *SDHAF4* | 128.8602672 | 260.9844676 | 2.025329244 | 1.018156456 | 0.000209961 | 0.001237141 | - |
| *RGLG2* | 726.5824652 | 1501.754211 | 2.066873732 | 1.047450255 | 0.000210021 | 0.001237216 | K16280 |
| *LECRKS2* | 473.8480568 | 1648.3284 | 3.478601159 | 1.798507276 | 0.000211916 | 0.001247825 | - |
| *SWEET3B* | 51.92516366 | 122.0033548 | 2.349599813 | 1.232415056 | 0.00021301 | 0.001253423 | K15382 |
| *ANR* | 0.314161193 | 2324.692915 | 7399.681958 | 12.85324755 | 0.000214158 | 0.001259054 | K08695 |
| *MFSD14A* | 15.36672826 | 60.46196524 | 3.934602358 | 1.97621784 | 0.000214421 | 0.00126004 | - |
| *LTI6A* | 3.367617652 | 69.94872751 | 20.77098256 | 4.376497559 | 0.00021483 | 0.00126188 | - |
| *ALA1* | 369.3710752 | 1430.351592 | 3.872397401 | 1.953227016 | 0.000215874 | 0.001267729 | K14802 |
| *PCS1* | 24.72431703 | 69.83275936 | 2.824456557 | 1.497973311 | 0.000216859 | 0.001273233 | K05941 |
| *TIFY10B* | 1.598071948 | 93.09258921 | 58.2530651 | 5.864262057 | 0.000218385 | 0.001281903 | K13464 |
| *FRI3* | 2078.353544 | 10005.76802 | 4.814276207 | 2.267318915 | 0.000222032 | 0.001301573 | K00522 |
| *Fra a 1.06* | 0 | 23.78496809 | Inf | Inf | 0.000223597 | 0.001309585 | - |
| *ERF1-3* | 1891.327587 | 4401.130716 | 2.327006039 | 1.218474954 | 0.000223776 | 0.001310341 | K03265 |
| *uncharacterized protein_27374* | 121.1294127 | 245.9961694 | 2.030854141 | 1.022086627 | 0.000225902 | 0.001321615 | - |
| *uncharacterized protein_25505* | 8.333307252 | 52.57352304 | 6.308842511 | 2.657375337 | 0.000226559 | 0.001325165 | - |
| *BSPA* | 88.68866812 | 411.8796966 | 4.644107363 | 2.215401325 | 0.000227848 | 0.001331816 | - |
| *TOP3A* | 37.52958132 | 93.92860703 | 2.502788566 | 1.323536418 | 0.000235301 | 0.001372642 | K03165 |
| *SAMDC* | 73.45321282 | 386.0920038 | 5.256298383 | 2.394047175 | 0.000236062 | 0.001376499 | K01611 |
| *LOX1.5* | 0 | 27.7768477 | Inf | Inf | 0.000236067 | 0.001376499 | K15718 |
| *ASNSD1* | 88.18234446 | 250.0065683 | 2.835109111 | 1.50340426 | 0.000239887 | 0.001397848 | - |
| *SEC14L2* | 426.1165485 | 1147.405817 | 2.692704193 | 1.429055751 | 0.000242732 | 0.001413176 | - |
| *GOLS4* | 0 | 10.38704854 | Inf | Inf | 0.000243468 | 0.001416833 | K18819 |
| *HSPRO2* | 0 | 10.34680616 | Inf | Inf | 0.000247265 | 0.001437344 | - |
| *ERD2* | 229.8347889 | 870.9463781 | 3.789445376 | 1.921986711 | 0.000247533 | 0.001438585 | K10949 |
| *LYK3* | 46.62297984 | 197.8800939 | 4.244260975 | 2.085513369 | 0.000250513 | 0.00145462 | - |
| *JA2L* | 1.610882779 | 17.85363313 | 11.08313613 | 3.470294265 | 0.000255526 | 0.001482424 | - |
| *At5g40670* | 24.09599465 | 67.71599939 | 2.810259563 | 1.490703388 | 0.000260913 | 0.001511347 | K12386 |
| *GOLS4* | 6.090937428 | 78.96808839 | 12.96484972 | 3.696533578 | 0.000264763 | 0.001532637 | K18819 |
| *DDB_G0290631* | 9.615573687 | 37.92547985 | 3.944172348 | 1.979722594 | 0.000268004 | 0.001550375 | K13348 |
| *AMY1.3* | 78.93347589 | 296.2215909 | 3.752800539 | 1.907967613 | 0.000271223 | 0.001567623 | K01176 |
| *At2g16250* | 94.55992914 | 195.191733 | 2.064211921 | 1.045591092 | 0.000273088 | 0.001578056 | - |
| *exgA* | 2.527744698 | 164.9956909 | 65.27387481 | 6.028433778 | 0.000273171 | 0.001578186 | - |
| *uncharacterized protein_41936* | 14.475488 | 48.50420944 | 3.350782331 | 1.744497971 | 0.000277806 | 0.001603559 | - |
| *RNR1* | 74.85747799 | 159.4888149 | 2.130566234 | 1.091236902 | 0.000282947 | 0.00163002 | - |
| *MNS2* | 73.84588332 | 162.9026328 | 2.205981235 | 1.141420519 | 0.000285414 | 0.001643156 | K01230 |
| *RHM1* | 336.9173958 | 1734.193843 | 5.147237467 | 2.363798343 | 0.000289478 | 0.001664009 | K12450 |
| *MIS12* | 23.56382959 | 67.25358167 | 2.854102361 | 1.513037077 | 0.000290181 | 0.001666957 | - |
| *uncharacterized protein_02415* | 22.99307984 | 64.76644553 | 2.816779917 | 1.494046846 | 0.000291243 | 0.001672695 | - |
| *VTI13* | 20.17375493 | 60.04800397 | 2.976540766 | 1.573636653 | 0.000293187 | 0.001681294 | K08493 |
| *uncharacterized protein_24130* | 0.681210029 | 13.737796 | 20.16675536 | 4.333907081 | 0.000293346 | 0.001681602 | - |
| *At1g19450* | 26.01951828 | 94.22348212 | 3.621261589 | 1.856492397 | 0.000297467 | 0.001702507 | K08145 |
| *TIFY10B* | 0.602700725 | 13.74103926 | 22.79910854 | 4.51090551 | 0.00029977 | 0.001714572 | K13464 |
| *CT1* | 133.8162824 | 380.6326911 | 2.844442278 | 1.508145805 | 0.000311028 | 0.001772556 | K00967 |
| *TBR* | 271.9305315 | 780.3371844 | 2.869619605 | 1.520859507 | 0.000313103 | 0.001783478 | - |
| *ICL 8* | 3.431795786 | 169.4636906 | 49.38047051 | 5.625868678 | 0.000313982 | 0.001787711 | K01637 |
| *URIC1* | 55.84892371 | 125.1075842 | 2.240107344 | 1.163567867 | 0.000314217 | 0.001788278 | K00365 |
| *At4g26910* | 77.91397512 | 163.7646161 | 2.101864471 | 1.071669647 | 0.000315156 | 0.00179323 | K00658 |
| *CRK2* | 0.314161193 | 12.05598548 | 38.37515813 | 5.26210079 | 0.000318273 | 0.001809403 | - |
| *exgA* | 0 | 10.06491701 | Inf | Inf | 0.000318512 | 0.001810375 | - |
| *Galm* | 0 | 10.05366506 | Inf | Inf | 0.000319893 | 0.001817438 | K01785 |
| *MTV1* | 382.6200729 | 828.8800017 | 2.166326495 | 1.115250693 | 0.000326202 | 0.001850886 | - |
| *RPL8* | 86.55092518 | 181.9785843 | 2.102560821 | 1.072147534 | 0.000331883 | 0.001879479 | K02938 |
| *CYTC* | 359.4388 | 1741.395726 | 4.844762797 | 2.276426032 | 0.000334777 | 0.001894651 | K08738 |
| *LPL1* | 17.05624432 | 53.8427821 | 3.156778308 | 1.658452948 | 0.000340634 | 0.001926141 | - |
| *UBI11* | 6.735235304 | 37.54516852 | 5.57444051 | 2.478827013 | 0.000341036 | 0.001927586 | K08770 |
| *BBE1* | 0.314161193 | 17.49916843 | 55.70124126 | 5.799637572 | 0.000341861 | 0.001931835 | - |
| *At4g38730* | 47.24153208 | 110.0135643 | 2.328746749 | 1.219553755 | 0.000342828 | 0.00193647 | - |
| *MO2* | 147.4054913 | 335.7527468 | 2.277749246 | 1.187608932 | 0.000348419 | 0.001964879 | - |
| *At4g33300* | 3.091888951 | 30.81803235 | 9.967380086 | 3.317214344 | 0.000349874 | 0.001972469 | - |
| *SUS2* | 3.707400507 | 23.206701 | 6.259561372 | 2.646061566 | 0.000355104 | 0.001996827 | K00695 |
| *NFP* | 37.62595839 | 92.62072723 | 2.461617755 | 1.299606755 | 0.000364288 | 0.002043672 | - |
| *Elac2* | 92.76476178 | 191.0212642 | 2.059200719 | 1.042084462 | 0.000364783 | 0.002045278 | K00784 |
| *uncharacterized protein_10571* | 5.041034245 | 44.13710165 | 8.755564732 | 3.130200235 | 0.000364807 | 0.002045278 | K13464 |
| *uncharacterized protein_42826* | 28.18986032 | 164.9322834 | 5.850766254 | 2.548625582 | 0.000366143 | 0.00205146 | - |
| *LAC3* | 0 | 16.56328642 | Inf | Inf | 0.000372966 | 0.002086215 | K05909 |
| *APD4* | 12.28929446 | 43.70377383 | 3.556247592 | 1.830355771 | 0.000374379 | 0.002093157 | K01931 |
| *FHA2* | 134.3416222 | 306.6966633 | 2.282960845 | 1.190906116 | 0.000376797 | 0.002104445 | - |
| *PAP2* | 40.8828678 | 531.810562 | 13.00815208 | 3.701344125 | 0.0003778 | 0.002109596 | - |
| *NPF5.10* | 69.44598229 | 309.4785973 | 4.456393114 | 2.155876504 | 0.000378104 | 0.002110848 | K14638 |
| *ACR4* | 4.938423619 | 26.52349367 | 5.370842138 | 2.425148318 | 0.000379204 | 0.002115642 | - |
| *EXPA1* | 0.668399198 | 42.6649096 | 63.83147933 | 5.996196178 | 0.000381452 | 0.002126384 | - |
| *At5g54860* | 99.46488145 | 202.4134275 | 2.035024067 | 1.025045856 | 0.000382293 | 0.002130173 | - |
| *HMGB15* | 197.1293864 | 413.0823564 | 2.095488471 | 1.067286584 | 0.000386455 | 0.002151542 | - |
| *GGCT2;1* | 12.56502316 | 43.95818828 | 3.498456606 | 1.806718597 | 0.000387364 | 0.002155691 | - |
| *ADC2* | 14.17413764 | 54.97511059 | 3.878550638 | 1.955517637 | 0.000391746 | 0.002177319 | K01583 |
| *BTS* | 96.2335937 | 272.678568 | 2.833507068 | 1.502588801 | 0.000392194 | 0.002178895 | K16276 |
| *SOP2* | 1.506751814 | 17.14490268 | 11.37871713 | 3.508266008 | 0.000394004 | 0.002186642 | - |
| *ACO3* | 3.170522234 | 22.04356152 | 6.952659497 | 2.797564936 | 0.000394476 | 0.002188803 | K01681 |
| *SGR* | 17.57066559 | 53.40479476 | 3.03942924 | 1.603800432 | 0.000397392 | 0.002203588 | - |
| *RFS2* | 72.75754764 | 236.6295245 | 3.252302094 | 1.70146127 | 0.00039754 | 0.002203947 | - |
| *At4g35930* | 16.41359077 | 114.5641584 | 6.979835186 | 2.803192971 | 0.000400908 | 0.002221218 | - |
| *WAKL20* | 0.367048835 | 11.72260199 | 31.93744501 | 4.997176997 | 0.000401226 | 0.00222251 | - |
| *F3H-2* | 0 | 10.92031426 | Inf | Inf | 0.000401508 | 0.002223609 | - |
| *FOLT1* | 79.78309078 | 165.267003 | 2.071454006 | 1.050643788 | 0.000401906 | 0.002225346 | K15115 |
| *APCB1* | 8.108945853 | 71.19721021 | 8.780082084 | 3.134234427 | 0.00040403 | 0.002235223 | - |
| *PSY* | 333.1002551 | 745.5914249 | 2.2383394 | 1.162428809 | 0.000408443 | 0.002258216 | K02291 |
| *PAP3* | 6.289677165 | 29.58434328 | 4.703634623 | 2.233775996 | 0.000411101 | 0.002270533 | K14379 |
| *uncharacterized protein_46529* | 54.04094552 | 120.645198 | 2.232477557 | 1.158645672 | 0.000414474 | 0.002288205 | - |
| *IPCS2* | 49.00626881 | 112.4877338 | 2.295374378 | 1.198729478 | 0.00041557 | 0.00229329 | - |
| *RSL1* | 10.17832158 | 38.90646914 | 3.822483778 | 1.934510379 | 0.000417206 | 0.002300392 | K11975 |
| *NPC2* | 0 | 9.726668628 | Inf | Inf | 0.000419481 | 0.002311969 | K01114 |
| *TRP4* | 265.1137506 | 738.5484677 | 2.785779561 | 1.478081102 | 0.000431 | 0.002368522 | - |
| *CXE15* | 0 | 9.634832449 | Inf | Inf | 0.000434637 | 0.002387513 | - |
| *uncharacterized protein_40918* | 138.9647645 | 304.5533239 | 2.191586659 | 1.131975727 | 0.000436248 | 0.002395864 | - |
| *PI4KG6* | 7.714631038 | 33.11531445 | 4.292533796 | 2.101829493 | 0.000447056 | 0.002450628 | - |
| *At3g21360* | 33.41365881 | 713.4472798 | 21.35196519 | 4.416296954 | 0.000449678 | 0.002462951 | - |
| *SPS3* | 117.8128862 | 789.8406861 | 6.704196049 | 2.745064338 | 0.000451586 | 0.002472373 | K00696 |
| *OsI_03083* | 7.430776502 | 47.38171665 | 6.376415256 | 2.672745586 | 0.000455417 | 0.002490465 | - |
| *GLIP* | 110.1267935 | 329.6021215 | 2.992933064 | 1.581560014 | 0.000455541 | 0.002490465 | - |
| *UVR3* | 43.82102678 | 166.8294032 | 3.807062851 | 1.928678388 | 0.000457885 | 0.002502181 | K02295 |
| *HMGS* | 23.76269331 | 66.22749155 | 2.787036414 | 1.478731852 | 0.00046487 | 0.002537721 | K01641 |
| *uncharacterized protein_04724* | 57.2223863 | 134.135464 | 2.344108182 | 1.229039153 | 0.000465385 | 0.002539864 | - |
| *CPRF2* | 62.73165543 | 133.9769355 | 2.135714969 | 1.094719119 | 0.000470004 | 0.0025615 | - |
| *YY1* | 69.29693732 | 144.8392753 | 2.090125206 | 1.063589367 | 0.000470295 | 0.002562556 | K09201 |
| *VIT_06s0061g00120* | 3.090368612 | 21.74565499 | 7.036589392 | 2.814876329 | 0.00047088 | 0.002565213 | - |
| *At5g15710* | 63.74935968 | 135.3494816 | 2.123150449 | 1.086206606 | 0.000473133 | 0.002575893 | - |
| *WRKY71* | 39.29137313 | 137.1115909 | 3.489610567 | 1.803066044 | 0.000474509 | 0.002582317 | - |
| *LOC_Os07g01090* | 2.555010678 | 31.27396355 | 12.24024769 | 3.613560847 | 0.000480971 | 0.002614782 | - |
| *RUS6* | 21.04241421 | 58.6754514 | 2.78843724 | 1.4794568 | 0.000482656 | 0.002622859 | - |
| *LOX1.1* | 0 | 15.9109152 | Inf | Inf | 0.000484368 | 0.002630537 | K15718 |
| *uncharacterized protein_09829* | 30.19797461 | 90.0110305 | 2.980697602 | 1.575650018 | 0.000484601 | 0.002631263 | - |
| *CYP71AU50* | 39.50319988 | 493.932252 | 12.50360106 | 3.644271749 | 0.000485235 | 0.002634163 | - |
| *KAPP* | 90.52214399 | 195.0415918 | 2.154628505 | 1.107439145 | 0.00048638 | 0.002639831 | K01090 |
| *RABB1C* | 12.77492941 | 71.2726247 | 5.579101255 | 2.480032735 | 0.000487523 | 0.002645492 | K07877 |
| *LOG2* | 215.5787294 | 446.7608222 | 2.072378957 | 1.051287839 | 0.00048876 | 0.002651661 | K10604 |
| *IRL7* | 62.47509509 | 133.6615889 | 2.139437942 | 1.097231832 | 0.000494083 | 0.002677784 | - |
| *EXPA1* | 0 | 23.04239519 | Inf | Inf | 0.00050054 | 0.002710545 | - |
| *CCL7* | 87.03643615 | 178.135205 | 2.046673932 | 1.033281276 | 0.000501606 | 0.002715762 | K01904 |
| *ACO1* | 21.85998236 | 62.05377303 | 2.838692731 | 1.505226696 | 0.000504075 | 0.002726888 | K14677 |
| *uncharacterized protein_27586* | 0.94248358 | 124.1693863 | 131.7470022 | 7.041626324 | 0.000506357 | 0.002738669 | - |
| *PYD2* | 65.70356191 | 139.6748378 | 2.125833573 | 1.088028656 | 0.000509114 | 0.002753017 | K01464 |
| *IMA* | 0.314161193 | 18.59956163 | 59.20388011 | 5.887619825 | 0.000511807 | 0.002765877 | - |
| *LTA3* | 183.9956297 | 458.9828475 | 2.494531246 | 1.31876874 | 0.000525612 | 0.002835832 | K00627 |
| *At1g06690* | 64.75906207 | 135.6844867 | 2.095220073 | 1.067101786 | 0.000526824 | 0.00284179 | - |
| *uncharacterized protein_25685* | 261.9123976 | 1338.424028 | 5.110197302 | 2.353378994 | 0.000527987 | 0.002846896 | - |
| *FMO1* | 0 | 12.51515994 | Inf | Inf | 0.000532803 | 0.002871686 | K00485 |
| *At5g26710* | 89.16518103 | 183.1742623 | 2.05432502 | 1.038664452 | 0.000539123 | 0.002903553 | K01885 |
| *PGL3* | 25.67669475 | 82.36403614 | 3.207735145 | 1.681555027 | 0.000542939 | 0.002922135 | - |
| *ORP3A* | 2415.934987 | 5752.941333 | 2.381248404 | 1.251718126 | 0.000545597 | 0.002935241 | - |
| *HSP70* | 9.315867643 | 35.22882472 | 3.781593521 | 1.918994298 | 0.000547661 | 0.002942741 | K03283 |
| *uncharacterized protein_38961* | 51.75862286 | 116.3749902 | 2.248417438 | 1.168909909 | 0.000552869 | 0.002967701 | - |
| *45572* | 25.74226924 | 85.17176877 | 3.308634836 | 1.726236075 | 0.000555761 | 0.002982617 | - |
| *ARP1* | 184.033066 | 377.0721613 | 2.048937017 | 1.034875637 | 0.000556452 | 0.002985108 | - |
| *SDR1* | 6.076482279 | 28.85939651 | 4.749359117 | 2.247732848 | 0.000560337 | 0.00300473 | K00059 |
| *WNK2* | 4.702771729 | 24.65324536 | 5.242279827 | 2.390194365 | 0.000565782 | 0.003030846 | K08867 |
| *PAP27* | 8.437438216 | 33.19103379 | 3.933780957 | 1.975916627 | 0.00056794 | 0.003039316 | - |
| *ORC3* | 13.61315804 | 44.25153476 | 3.250644312 | 1.700725704 | 0.00056952 | 0.003046535 | K02605 |
| *PHT1-4* | 120.4735482 | 490.9647651 | 4.075290988 | 2.026903076 | 0.00057735 | 0.003083411 | K08176 |
| *ASR1* | 0.916861919 | 51.13609234 | 55.77294823 | 5.80149363 | 0.000578167 | 0.003087148 | - |
| *MST3* | 2.542199847 | 24.95450107 | 9.816105172 | 3.295150706 | 0.000580141 | 0.00309581 | - |
| *OXR1* | 8.411816555 | 32.96387578 | 3.918758281 | 1.970396587 | 0.000580586 | 0.003097554 | - |
| *At2g29640* | 4.441622158 | 26.28032473 | 5.916830337 | 2.564824527 | 0.000585021 | 0.003119321 | K15235 |
| *COMT1* | 3.143256254 | 196.2989497 | 62.45082612 | 5.964648751 | 0.000591404 | 0.003148897 | K13066 |
| *OMT2* | 0.681210029 | 48.44958419 | 71.12282871 | 6.152240799 | 0.000599424 | 0.003189023 | - |
| *MANA* | 35.33197331 | 110.0149676 | 3.113751012 | 1.638653585 | 0.000599748 | 0.003190101 | - |
| *CYCB2-1* | 34.80675746 | 86.48453279 | 2.484705244 | 1.313074718 | 0.000602558 | 0.003203109 | K05868 |
| *Os08g0536000* | 7.244971576 | 33.68406356 | 4.649302376 | 2.217014257 | 0.000609498 | 0.003235436 | K00162 |
| *uncharacterized protein_00680* | 37.51689447 | 89.57264529 | 2.387528247 | 1.255517802 | 0.000613073 | 0.00325179 | - |
| *GLR3.3* | 349.4098671 | 729.8609489 | 2.088838976 | 1.062701282 | 0.000622059 | 0.003292166 | K05387 |
| *DDB_G0268948* | 9.064240265 | 34.98069142 | 3.859197285 | 1.948300797 | 0.000626944 | 0.003314693 | - |
| *IQD6* | 72.69590991 | 230.0705729 | 3.164835177 | 1.662130367 | 0.000635645 | 0.003355311 | - |
| *uncharacterized protein_11466* | 6.156635901 | 28.5178054 | 4.632043515 | 2.211648807 | 0.000639361 | 0.003372687 | - |
| *PAP15* | 34.42841814 | 154.8094915 | 4.496561267 | 2.168822126 | 0.000639449 | 0.003372687 | - |
| *ABCG39* | 8.019146058 | 32.11982995 | 4.005392808 | 2.001943735 | 0.00064044 | 0.003376563 | - |
| *RUN1* | 15.48519039 | 48.07747405 | 3.104738968 | 1.634471979 | 0.000646772 | 0.003405856 | - |
| *Os12g0628600* | 3.090368612 | 21.14964299 | 6.843728256 | 2.774782476 | 0.000653165 | 0.00343335 | - |
| *GCH1* | 9.785527105 | 39.37690198 | 4.023993962 | 2.00862814 | 0.000654863 | 0.003440902 | K01495 |
| *uncharacterized protein_23691* | 190.7869173 | 651.1092203 | 3.412756123 | 1.770937322 | 0.000659625 | 0.003463163 | - |
| *SWEET16* | 193.3573794 | 472.1734194 | 2.441972584 | 1.288047003 | 0.000668217 | 0.00350269 | K15382 |
| *pgk* | 32.11833358 | 137.0849312 | 4.268120912 | 2.093601047 | 0.00067212 | 0.003522446 | K00927 |
| *HAK5* | 410.3006015 | 5509.435329 | 13.42780222 | 3.747151288 | 0.000673905 | 0.003529698 | K03549 |
| *EDR2L* | 6.483607925 | 29.01559417 | 4.47522344 | 2.161959716 | 0.000675066 | 0.003533672 | - |
| *ERD3* | 777.1344055 | 3766.12847 | 4.846173897 | 2.276846174 | 0.000682745 | 0.003568201 | - |
| *BAM4* | 1291.791963 | 3061.973033 | 2.370329837 | 1.245087828 | 0.000684925 | 0.003578164 | K01177 |
| *At3g21360* | 21.2237821 | 80.93532329 | 3.813426039 | 1.931087719 | 0.00068814 | 0.003592123 | - |
| *LOGL1* | 66.13950209 | 252.5593239 | 3.818585201 | 1.933038214 | 0.000689562 | 0.003598829 | K06966 |
| *luxQ* | 9.801502593 | 35.35603839 | 3.607205941 | 1.850881791 | 0.000690701 | 0.003602636 | - |
| *SPS3* | 121.5719019 | 735.9103294 | 6.053292888 | 2.597720157 | 0.000696805 | 0.003631597 | K00696 |
| *MYB4* | 0.301350363 | 31.95359766 | 106.0347078 | 6.728392762 | 0.000703433 | 0.003663968 | K09422 |
| *CCDC22* | 20.13684278 | 56.18517147 | 2.790167857 | 1.480351918 | 0.000703673 | 0.003664494 | - |
| *BSP* | 4.034496511 | 3975.661681 | 985.4170575 | 9.944590635 | 0.000713616 | 0.003711139 | - |
| *WRKY42* | 0 | 9.100044567 | Inf | Inf | 0.000714796 | 0.003716543 | - |
| *NIT4A* | 3.470228278 | 22.61697015 | 6.517430077 | 2.7043032 | 0.000718557 | 0.003733887 | K13035 |
| *MIZ1* | 188.5670045 | 408.1183049 | 2.164314515 | 1.113910164 | 0.000720384 | 0.003741909 | - |
| *DDB_G0268948* | 75.27096117 | 394.6412331 | 5.242941328 | 2.390376401 | 0.000726292 | 0.003767674 | - |
| *Os04g0452000* | 58.55130682 | 123.8107574 | 2.114568642 | 1.080363393 | 0.000727507 | 0.003772218 | K17506 |
| *uncharacterized protein_00087* | 310.2707079 | 699.0657562 | 2.253083318 | 1.171900665 | 0.000730541 | 0.003787201 | - |
| *At5g47540* | 134.8275616 | 392.6413944 | 2.912174557 | 1.542096834 | 0.000733784 | 0.003800773 | K08272 |
| *At3g26720* | 50.65747636 | 112.7425526 | 2.225585652 | 1.154185024 | 0.000755864 | 0.003909251 | K01191 |
| *hX* | 72.59404752 | 160.9168207 | 2.216666878 | 1.148391978 | 0.00076113 | 0.00393417 | - |
| *uncharacterized protein_03044* | 13.8986569 | 125.9913622 | 9.065002688 | 3.180307448 | 0.000790644 | 0.004073144 | - |
| *ACA5* | 7.07349782 | 30.27209199 | 4.279649582 | 2.097492674 | 0.000803172 | 0.004131227 | K01537 |
| *uncharacterized protein_34176* | 10.25683088 | 37.2972406 | 3.636331828 | 1.862483856 | 0.000807388 | 0.004149674 | - |
| *LAC3* | 0.916861919 | 651.3766091 | 710.4413386 | 9.47257172 | 0.000813043 | 0.004173853 | K05909 |
| *HCF136* | 2785.778051 | 6394.957978 | 2.295573395 | 1.198854559 | 0.000819858 | 0.00420556 | - |
| *Os04g0656100* | 13949.3354 | 28705.09515 | 2.057810952 | 1.04111045 | 0.000840798 | 0.004304599 | K01535 |
| *At4g17830* | 7.79478466 | 31.4367472 | 4.033048835 | 2.011870875 | 0.000845453 | 0.00432591 | K01438 |
| *CCL5* | 326.0961727 | 2425.015587 | 7.436504287 | 2.894624606 | 0.000849731 | 0.004346098 | K10526 |
| *RPV1* | 0.314161193 | 10.75276519 | 34.22690457 | 5.097058918 | 0.00085232 | 0.004356818 | K03283 |
| *uncharacterized protein_25754* | 1.545184306 | 32.79967588 | 21.22703147 | 4.407830724 | 0.000855221 | 0.004369104 | - |
| *NDT1* | 171.2120504 | 358.2960751 | 2.092703605 | 1.065367994 | 0.000870254 | 0.004439881 | K15115 |
| *PAD4* | 12.1721047 | 39.55894627 | 3.24996763 | 1.700425349 | 0.000874061 | 0.004456714 | - |
| *At3g04930* | 16.82211277 | 49.13093297 | 2.920616075 | 1.546272723 | 0.000878213 | 0.004476151 | - |
| *RAD50* | 33.0658741 | 80.24574757 | 2.426844889 | 1.279081902 | 0.000881289 | 0.004490962 | K10866 |
| *APM1* | 168.0418325 | 377.0356245 | 2.243700981 | 1.16588042 | 0.000881831 | 0.004492854 | K08776 |
| *45510* | 71.41603603 | 144.5331612 | 2.023819428 | 1.017080574 | 0.00088267 | 0.004496263 | - |
| *ANR* | 0.301350363 | 10.63031695 | 35.27560695 | 5.140599001 | 0.000890496 | 0.004530871 | K13082 |
| *ANT1* | 28.41738637 | 264.8246452 | 9.319106328 | 3.220191612 | 0.000890923 | 0.004532168 | K14209 |
| *CRK41* | 160.5488941 | 334.593008 | 2.084056759 | 1.05939457 | 0.000891243 | 0.004532415 | - |
| *RSH1* | 11.32106524 | 38.08006231 | 3.363646575 | 1.750026127 | 0.000895345 | 0.004551149 | - |
| *SPA3* | 3.196143896 | 20.34573363 | 6.36571265 | 2.670322035 | 0.000918687 | 0.004659954 | - |
| *NAAT1* | 0.969749561 | 13.3608274 | 13.77760603 | 3.784253325 | 0.000920137 | 0.004664576 | K00815 |
| *AAE13* | 51.79060206 | 113.3720156 | 2.189046102 | 1.130302339 | 0.000922073 | 0.004673078 | K18660 |
| *GULLO5* | 14.89542448 | 48.79917755 | 3.276118624 | 1.711987596 | 0.000932538 | 0.004720181 | - |
| *POB1* | 24.49831132 | 63.60158495 | 2.596162002 | 1.376380411 | 0.000934767 | 0.004728411 | - |
| *CDCA7L* | 101.2490063 | 205.3654031 | 2.028320185 | 1.02028541 | 0.000939218 | 0.004749428 | - |
| *DLO1* | 0 | 8.76331835 | Inf | Inf | 0.000942204 | 0.004759962 | - |
| *PNC1* | 0 | 8.76331835 | Inf | Inf | 0.000942204 | 0.004759962 | K00430 |
| *IF4G* | 16.99054585 | 50.35844036 | 2.963909506 | 1.5675014 | 0.000951456 | 0.004799346 | K03260 |
| *At1g32860* | 5.698266932 | 26.67816916 | 4.681804043 | 2.227064552 | 0.000967434 | 0.004873414 | - |
| *VCL* | 0 | 8.645735005 | Inf | Inf | 0.000986109 | 0.0049599 | - |
| *AATP1* | 1193.123232 | 5134.714092 | 4.303590739 | 2.105540888 | 0.000993298 | 0.004988446 | K03301 |
| *ELI3* | 0 | 8.79393041 | Inf | Inf | 0.00099398 | 0.004989969 | K00083 |
| *CAX1a* | 2.724840115 | 18.28056745 | 6.708858752 | 2.746067369 | 0.001007959 | 0.005054368 | K07300 |
| *ERD7* | 252.4794642 | 942.2336405 | 3.731921895 | 1.899918793 | 0.001019013 | 0.005104943 | K19366 |
| *ABCC8* | 20.20266523 | 87.93281113 | 4.352535179 | 2.121855958 | 0.001019855 | 0.005108192 | - |
| *DTX16* | 32.15689005 | 96.46278336 | 2.99975474 | 1.584844551 | 0.001024839 | 0.00513023 | K03327 |
| *At1g33420* | 48.20811698 | 318.1526929 | 6.599566895 | 2.722371349 | 0.001034426 | 0.005170365 | - |
| *LAC3* | 1.205401451 | 14.34992484 | 11.90468523 | 3.573457569 | 0.001042006 | 0.005206277 | K05909 |
| *At3g56230* | 10.15269992 | 36.32901898 | 3.578261868 | 1.839258972 | 0.001044217 | 0.005215348 | K10523 |
| *RGLG2* | 8.961629639 | 51.23816853 | 5.717505699 | 2.515385899 | 0.001046437 | 0.005224455 | K16280 |
| *RXW8* | 5.002601753 | 67.56477254 | 13.50592669 | 3.755520727 | 0.001048136 | 0.005231943 | - |
| *UTR1* | 25.13956851 | 64.66162986 | 2.572105795 | 1.362949984 | 0.001054279 | 0.005256673 | K15275 |
| *MANA* | 72.89933464 | 156.3480037 | 2.144710983 | 1.100783246 | 0.001054287 | 0.005256673 | K01191 |
| *GLCAT14B* | 182.7199688 | 382.9831822 | 2.096011644 | 1.067646732 | 0.001060482 | 0.005283564 | - |
| *uncharacterized protein_32756* | 0 | 118.3973992 | Inf | Inf | 0.001061717 | 0.005288719 | - |
| *OFUT23* | 47.92274211 | 104.4253361 | 2.179035079 | 1.123689422 | 0.001063584 | 0.005294015 | - |
| *LYK5* | 13.99149737 | 166.6130276 | 11.90816274 | 3.573878937 | 0.001068557 | 0.005314754 | - |
| *PI4KG7* | 368.3376478 | 849.2969627 | 2.3057566 | 1.205240228 | 0.001069051 | 0.005316209 | - |
| *NPK1* | 47.99960709 | 103.8564811 | 2.163694401 | 1.113496748 | 0.001070954 | 0.005323661 | - |
| *IDM1* | 1.532373475 | 15.4082551 | 10.05515649 | 3.32986363 | 0.001074165 | 0.005338619 | - |
| *ALDH10A8* | 378.5350813 | 771.7903431 | 2.038887229 | 1.027781982 | 0.001076823 | 0.005348803 | K00130 |
| *uncharacterized protein_38058* | 34.23755627 | 128.0843272 | 3.741047585 | 1.903442316 | 0.001079519 | 0.005361186 | - |
| *PAP15* | 1.833723838 | 16.44083818 | 8.965820175 | 3.164435564 | 0.001080034 | 0.005362732 | - |
| *HIR4* | 6.090937428 | 36.08109106 | 5.923733659 | 2.566506776 | 0.001080712 | 0.005365087 | - |
| *GT5* | 9.535544045 | 139.1746123 | 14.595351 | 3.867437001 | 0.001082472 | 0.005372813 | K13691 |
| *At1g56140* | 22.87322135 | 90.00758186 | 3.935063649 | 1.976386971 | 0.0010856 | 0.005387323 | K04733 |
| *uncharacterized protein_08416* | 0.367048835 | 10.36616627 | 28.24192662 | 4.819766605 | 0.001092872 | 0.00541933 | K19037 |
| *GGCT2;2* | 1159.422534 | 2334.890972 | 2.013839564 | 1.009948753 | 0.001095647 | 0.005432066 | - |
| *SD25* | 3.354806821 | 255.1930724 | 76.06788887 | 6.249215661 | 0.001099817 | 0.005448642 | - |
| *GGT2* | 0.301350363 | 10.40164323 | 34.51677685 | 5.109225848 | 0.001103547 | 0.005464041 | K18592 |
| *At5g01750* | 31.66489757 | 76.13285529 | 2.404329751 | 1.265634774 | 0.001105709 | 0.005473716 | - |
| *D1* | 43.29239833 | 98.62120197 | 2.278025838 | 1.18778411 | 0.001108406 | 0.005485008 | K07513 |
| *At4g33300* | 0.301350363 | 10.35805811 | 34.37214417 | 5.103167946 | 0.001119171 | 0.005531011 | - |
| *OsI_12825* | 28.20279513 | 89.08287165 | 3.158654001 | 1.659309913 | 0.001148375 | 0.005666835 | K16275 |
| *uncharacterized protein_09083* | 71.97220665 | 171.8110028 | 2.387185426 | 1.255310633 | 0.00116506 | 0.00573521 | - |
| *GATA26* | 47.19981095 | 102.2260655 | 2.165815146 | 1.114910113 | 0.00117544 | 0.005778755 | - |
| *Afg1l* | 19.81799659 | 55.01088595 | 2.775804592 | 1.47290601 | 0.001187336 | 0.005832884 | - |
| *NTR1* | 0 | 13.98735199 | Inf | Inf | 0.00119346 | 0.005861876 | K00384 |
| *LBD12* | 37.48175062 | 682.9032425 | 18.21961972 | 4.187420942 | 0.001196802 | 0.005875007 | - |
| *At3g56230* | 18.03868073 | 50.61143856 | 2.805717298 | 1.488369651 | 0.001199789 | 0.005885286 | K10523 |
| *ATL78* | 36.79712797 | 93.20051003 | 2.532820227 | 1.340744682 | 0.001205533 | 0.00591016 | K19040 |
| *uncharacterized protein_14994* | 69.58398473 | 184.9639393 | 2.658139513 | 1.410416827 | 0.001207984 | 0.005918876 | - |
| *SUN3* | 9.287081324 | 117.9584974 | 12.70135291 | 3.666910271 | 0.001208337 | 0.005919503 | - |
| *CDC48B* | 37.91272962 | 86.07370887 | 2.270311574 | 1.182890304 | 0.00120872 | 0.005920279 | - |
| *EPHX4* | 1.415307699 | 14.05516211 | 9.930817243 | 3.311912447 | 0.001210195 | 0.005925299 | - |
| *COMT1* | 0.301350363 | 90.766721 | 301.1999726 | 8.234577828 | 0.00121618 | 0.005952392 | K13066 |
| *uncharacterized protein_38041* | 42.27444612 | 104.4476411 | 2.470703952 | 1.304922153 | 0.001217414 | 0.005957325 | K14009 |
| *WRKY41* | 1.296721585 | 14.09053959 | 10.86627982 | 3.441786199 | 0.001233132 | 0.006020826 | - |
| *MBD9* | 30.74651531 | 74.57997954 | 2.42564007 | 1.278365491 | 0.001235957 | 0.006032385 | - |
| *PR4B* | 5.135643017 | 49.61119507 | 9.660172038 | 3.272048882 | 0.001238302 | 0.006042713 | - |
| *NFYC4* | 0 | 8.42506997 | Inf | Inf | 0.001243303 | 0.006060383 | K08066 |
| *ELIP1* | 0 | 8.407331493 | Inf | Inf | 0.001251847 | 0.006096391 | - |
| *UGT92A1* | 0 | 8.40408823 | Inf | Inf | 0.001253417 | 0.00610291 | - |
| *NHL13* | 72.79166708 | 642.0569444 | 8.820473142 | 3.140856046 | 0.001256098 | 0.006113705 | - |
| *HAK5* | 51.21529129 | 2487.49133 | 48.56930942 | 5.601973068 | 0.001256763 | 0.006115813 | K03549 |
| *CRSP* | 0.367048835 | 171.0244426 | 465.9446539 | 8.864014788 | 0.00126731 | 0.006161485 | - |
| *GOLS4* | 1.65095959 | 15.10862746 | 9.15142173 | 3.193995892 | 0.001267317 | 0.006161485 | K18819 |
| *AVT3C* | 249.091558 | 854.2222984 | 3.429350659 | 1.777935431 | 0.001274546 | 0.006193203 | K14209 |
| *SCRM2* | 2.030819256 | 16.02048341 | 7.888680081 | 2.979783931 | 0.0012778 | 0.006207869 | - |
| *chi1* | 7.714755018 | 1019.638573 | 132.1673301 | 7.046221797 | 0.001282415 | 0.006227994 | K20547 |
| *XTH26* | 0 | 18.30632106 | Inf | Inf | 0.001334771 | 0.006460832 | K08235 |
| *ULT1* | 22.33773943 | 58.52574027 | 2.620038632 | 1.389588084 | 0.001337142 | 0.006471121 | - |
| *slx1* | 53.24725895 | 111.2086262 | 2.088532412 | 1.062489533 | 0.001346757 | 0.006512869 | K15078 |
| *ADT2* | 88.71112512 | 434.644875 | 4.899553179 | 2.292650187 | 0.001357358 | 0.006556917 | K05359 |
| *ROQ1* | 56.38428165 | 116.2522307 | 2.061784372 | 1.043893459 | 0.001374214 | 0.006628621 | - |
| *CRPK1* | 14.72559504 | 79.76184432 | 5.416544737 | 2.437372837 | 0.001376592 | 0.006637662 | - |
| *TCHQD* | 16.24667803 | 132.3696463 | 8.147489974 | 3.026355672 | 0.001377167 | 0.006638005 | - |
| *TIFY10A* | 0.904051088 | 12.82603952 | 14.18729504 | 3.826527645 | 0.00139663 | 0.006721982 | K13464 |
| *AMY3* | 504.1764039 | 1025.22966 | 2.033474102 | 1.023946617 | 0.001405158 | 0.006756096 | - |
| *IQD17* | 14.54599545 | 42.35381819 | 2.911716722 | 1.541870004 | 0.001411375 | 0.006781806 | - |
| *ZUP1* | 43.56114959 | 93.97361484 | 2.157280414 | 1.109213718 | 0.001412836 | 0.006786348 | - |
| *MTPC4* | 49.40235192 | 114.5290728 | 2.318291911 | 1.213062237 | 0.001423073 | 0.006833034 | K14696 |
| *uncharacterized protein_37984* | 0 | 27.67851211 | Inf | Inf | 0.001426566 | 0.006848557 | - |
| *NAC100* | 37.5025633 | 162.7788743 | 4.340473289 | 2.117852364 | 0.001436288 | 0.00689021 | - |
| *TFIIB* | 63.2396234 | 145.8964942 | 2.307042426 | 1.206044535 | 0.001439774 | 0.006905677 | K03124 |
| *TSJT1* | 900.4910457 | 2465.621791 | 2.73808585 | 1.453167682 | 0.001441944 | 0.006914822 | - |
| *CHIT1* | 2.686407623 | 37.83203492 | 14.08276041 | 3.815858244 | 0.001447706 | 0.006938668 | - |
| *uncharacterized protein_35207* | 24.62006209 | 74.48337149 | 3.02531209 | 1.597083978 | 0.001456819 | 0.006974735 | - |
| *ROQ1* | 2.240849484 | 21.60709635 | 9.642368439 | 3.269387557 | 0.001472403 | 0.00704295 | - |
| *LAR* | 351.9854055 | 20332.00696 | 57.76377837 | 5.852093208 | 0.001479273 | 0.007073245 | K13081 |
| *NADP-ME4* | 1.048258864 | 27.41761758 | 26.15538825 | 4.70903628 | 0.001481617 | 0.007083168 | K00029 |
| *NTR2* | 2.62070915 | 195.0800373 | 74.43788153 | 6.217965092 | 0.0014963 | 0.007141701 | K00384 |
| *RAP2-12* | 9.77740127 | 58.61586179 | 5.995034895 | 2.583768151 | 0.001496728 | 0.007142451 | K09286 |
| *MYB20* | 0.615511556 | 11.4148662 | 18.54533207 | 4.212984196 | 0.001503459 | 0.007170679 | K09422 |
| *Os07g0103200* | 4.898346808 | 23.28555769 | 4.753758483 | 2.249068608 | 0.001508334 | 0.007190047 | - |
| *DGK1* | 117.6755319 | 928.7682478 | 7.892619929 | 2.980504278 | 0.001510658 | 0.007198501 | K00901 |
| *CRK8* | 61.71435134 | 127.3130365 | 2.062940528 | 1.04470223 | 0.001524696 | 0.007250972 | - |
| *uncharacterized protein_43009* | 24.2994476 | 60.72946509 | 2.499211755 | 1.321473145 | 0.00152777 | 0.007260349 | K07441 |
| *SIS8* | 58.71809558 | 119.6158582 | 2.037120874 | 1.026531586 | 0.001532156 | 0.007279879 | - |
| *At3g61590* | 31.97729046 | 74.65894214 | 2.33474885 | 1.223267367 | 0.001534429 | 0.007286743 | - |
| *UGT84A23* | 1.703847232 | 14.73003724 | 8.645163113 | 3.111893184 | 0.001540274 | 0.007310544 | K13691 |
| *At2g30020* | 0 | 9.81039665 | Inf | Inf | 0.001551784 | 0.007358548 | K17506 |
| *PMGI* | 5.398560888 | 25.59247655 | 4.740610893 | 2.245072982 | 0.001571552 | 0.007448269 | K15633 |
| *BHLH148* | 1.035448033 | 70.59157433 | 68.17490792 | 6.091168941 | 0.001578883 | 0.007477637 | - |
| *OFUT23* | 46.1851756 | 113.2803912 | 2.452743542 | 1.294396394 | 0.001588422 | 0.00751741 | - |
| *CCL9* | 496.0291495 | 3887.090968 | 7.836416412 | 2.970194062 | 0.001604975 | 0.007584854 | - |
| *R12* | 5.619757628 | 140.2279001 | 24.95265977 | 4.641121699 | 0.001609123 | 0.007601729 | K00430 |
| *Ccdc90b* | 25.34795442 | 67.24739355 | 2.652971219 | 1.407609024 | 0.001610368 | 0.007604885 | - |
| *PBL3* | 520.2342689 | 1104.80894 | 2.123675824 | 1.086563558 | 0.001635971 | 0.007707089 | K04733 |
| *QRT3* | 1.231023112 | 19.09552983 | 15.51191821 | 3.955305196 | 0.001644538 | 0.00774405 | - |
| *VIT_06s0061g00120* | 2.81628423 | 18.32881852 | 6.508156503 | 2.702248944 | 0.001650615 | 0.007768123 | - |
| *EIF4G* | 56.69667454 | 115.656331 | 2.039913839 | 1.028508218 | 0.001650829 | 0.007768123 | K03260 |
| *TIC32B* | 85.34071475 | 274.6386608 | 3.218143433 | 1.686228629 | 0.001652495 | 0.007774573 | - |
| *ROQ1* | 0 | 8.052866798 | Inf | Inf | 0.001663146 | 0.007820497 | - |
| *HPCA1* | 4.127336984 | 20.75645808 | 5.029019476 | 2.330277141 | 0.001674045 | 0.007866133 | - |
| *UGT85A1* | 3.07907812 | 55.54393985 | 18.03914603 | 4.173059138 | 0.001675539 | 0.007871746 | - |
| *uncharacterized protein_09498* | 43.57091975 | 243.9248473 | 5.598340561 | 2.484999252 | 0.001676812 | 0.007876325 | - |
| *TIFY10B* | 0 | 8.025498001 | Inf | Inf | 0.001680913 | 0.007891371 | K13464 |
| *MYB2* | 0 | 8.02387637 | Inf | Inf | 0.001681974 | 0.007894942 | K09422 |
| *GDP1* | 30.44668529 | 71.84956856 | 2.35984863 | 1.238694322 | 0.001684139 | 0.007900963 | K11135 |
| *uncharacterized protein_02783* | 0 | 8.017489313 | Inf | Inf | 0.001686158 | 0.007908623 | - |
| *CRK8* | 2.462046225 | 22.38819696 | 9.093329253 | 3.184808591 | 0.001721565 | 0.008054956 | - |
| *CHI4* | 0.916861919 | 19.60132728 | 21.37871241 | 4.41810306 | 0.001734648 | 0.00811041 | K01183 |
| *RAMDAZC7* | 0 | 29.89198604 | Inf | Inf | 0.001740463 | 0.008133272 | - |
| *Vps13* | 70.13504197 | 172.726301 | 2.462767485 | 1.300280427 | 0.001747073 | 0.008159729 | K19525 |
| *XRN3* | 20.94944976 | 53.71546897 | 2.564051543 | 1.358425263 | 0.001747363 | 0.008159729 | K12619 |
| *dnaJ* | 6.156635901 | 34.73600676 | 5.642043369 | 2.496217756 | 0.001748325 | 0.008162775 | K03686 |
| *PLC2* | 903.7641023 | 2232.478537 | 2.4702005 | 1.304628147 | 0.001753964 | 0.0081862 | K05857 |
| *BUBR1* | 11.13513633 | 36.07621973 | 3.239854336 | 1.695928951 | 0.001762098 | 0.008219797 | K02178 |
| *SRD2* | 28.0133014 | 66.10017842 | 2.35959973 | 1.238542149 | 0.001764908 | 0.008229991 | K15210 |
| *LECRK44* | 9.787171423 | 33.58564139 | 3.431598358 | 1.778880707 | 0.001789014 | 0.008330608 | - |
| *R35* | 0 | 23.72374397 | Inf | Inf | 0.001802108 | 0.008381753 | K00430 |
| *CKB4* | 27.24889707 | 66.00834224 | 2.422422532 | 1.276450529 | 0.001804476 | 0.00838779 | K03115 |
| *ACA5* | 5.659710459 | 24.4825525 | 4.325760598 | 2.112953824 | 0.001808234 | 0.008402293 | K01537 |
| *UVR8* | 38.64543094 | 85.83043402 | 2.220972362 | 1.15119144 | 0.001841019 | 0.008530632 | - |
| *GSTU19* | 23.49481426 | 62.46754181 | 2.658779981 | 1.410764396 | 0.00187081 | 0.008656739 | K00799 |
| *AZG1* | 62.11462354 | 189.5691586 | 3.051924778 | 1.609719404 | 0.001872546 | 0.008662951 | K06901 |
| *CAD* | 38.29106895 | 186.7499622 | 4.877115404 | 2.28602811 | 0.001904526 | 0.008795393 | K00083 |
| *DTX41* | 199.0557941 | 3604.678764 | 18.1088864 | 4.178625926 | 0.001915009 | 0.008836129 | K03327 |
| *At2g42960* | 0.301350363 | 85.02228878 | 282.1376686 | 8.140255485 | 0.001925494 | 0.008876737 | - |
| *ETR2* | 56.82006963 | 113.7186705 | 2.001382104 | 1.000996633 | 0.001942256 | 0.008946189 | K14509 |
| *XTH32* | 38.18085663 | 249.0248332 | 6.522243218 | 2.705368241 | 0.001981412 | 0.009110624 | K08235 |
| *CYP750A1* | 0 | 47.77885836 | Inf | Inf | 0.001983014 | 0.0091164 | - |
| *ALMT9* | 45.10202084 | 153.0233819 | 3.392827618 | 1.762488132 | 0.001993401 | 0.009160955 | - |
| *CHI4* | 7.97602857 | 233.1851739 | 29.23574958 | 4.869661676 | 0.002005653 | 0.009210964 | K01183 |
| *KIC* | 2.87069221 | 127.0711235 | 44.26497661 | 5.468093754 | 0.002011179 | 0.00922937 | - |
| *ATJ11* | 2.173506693 | 28.39384144 | 13.06360893 | 3.707481603 | 0.002011192 | 0.00922937 | - |
| *uncharacterized protein_15771* | 127.465372 | 375.7269582 | 2.94767867 | 1.559579263 | 0.00201144 | 0.00922937 | - |
| *HXK1* | 6.640750512 | 27.15183883 | 4.088670216 | 2.031631703 | 0.002018566 | 0.009260455 | K00844 |
| *CIPK5* | 28.6304855 | 68.07350197 | 2.377657968 | 1.249541195 | 0.002028419 | 0.00930404 | K07198 |
| *CAX1a* | 8.660279276 | 98.37244609 | 11.35903854 | 3.505768822 | 0.002032569 | 0.009318636 | K07300 |
| *MTP5* | 300.6168257 | 773.2437835 | 2.572190634 | 1.36299757 | 0.002032954 | 0.009318636 | - |
| *CYP76T24* | 3.276297518 | 23.80767737 | 7.266640847 | 2.861288603 | 0.002033016 | 0.009318636 | - |
| *MYB36* | 9.089861926 | 31.82983264 | 3.501684943 | 1.808049286 | 0.002041818 | 0.009349229 | K09422 |
| *OFUT20* | 9.391212289 | 32.4515918 | 3.455527445 | 1.788905938 | 0.002044948 | 0.009358684 | - |
| *uncharacterized protein_23688* | 2.62070915 | 18.73792134 | 7.149943113 | 2.837931763 | 0.00207664 | 0.00948396 | - |
| *ELI* | 0.367048835 | 20.22014159 | 55.08842325 | 5.783677265 | 0.002087105 | 0.009523499 | - |
| *GT5* | 5.175719828 | 23.32093518 | 4.505834155 | 2.171794214 | 0.002093302 | 0.009550123 | K13691 |
| *RT* | 10.36096185 | 67.57083543 | 6.521675922 | 2.705242752 | 0.002095 | 0.009554561 | - |
| *BGLU24* | 80.41749452 | 280.4935157 | 3.487966361 | 1.802386126 | 0.002096692 | 0.009560624 | K01188 |
| *SWEET3B* | 0 | 278.6605899 | Inf | Inf | 0.002098047 | 0.009565149 | K15382 |
| *ELM1* | 199.7861116 | 444.0187743 | 2.222470675 | 1.152164383 | 0.002117855 | 0.00964378 | - |
| *BARD1* | 17.61722392 | 91.90085073 | 5.21653418 | 2.383091611 | 0.00212502 | 0.009671392 | K10683 |
| *UGD3* | 139.7808123 | 1532.893725 | 10.9664102 | 3.455019439 | 0.002137191 | 0.009723424 | K00012 |
| *VHA-a1* | 4.125816645 | 40.88163258 | 9.908737128 | 3.308701197 | 0.002157191 | 0.009807644 | K02154 |
| *LOB* | 0 | 7.732356895 | Inf | Inf | 0.002181781 | 0.009909186 | - |
| *LYK3* | 0 | 7.727492001 | Inf | Inf | 0.002185899 | 0.009924471 | - |
| *CYP76T24* | 13.41289797 | 125.7225391 | 9.373256948 | 3.228550432 | 0.002196927 | 0.009969388 | - |
| *TBL14* | 115.432266 | 266.5278067 | 2.308954125 | 1.20723951 | 0.002197496 | 0.009970252 | - |
| *CBSX5* | 516.3429188 | 2060.681633 | 3.990916808 | 1.996720206 | 0.002201255 | 0.009982154 | - |
| *GSTU20* | 3.131965762 | 17.65870882 | 5.638218985 | 2.495239512 | 0.002208704 | 0.010010767 | K00799 |
| *KU80* | 10.99092856 | 35.32218306 | 3.213757863 | 1.684261235 | 0.002223713 | 0.010070133 | K10885 |
| *GFAT1* | 10.83226563 | 35.46561304 | 3.27407158 | 1.711085863 | 0.002225797 | 0.010074376 | K00820 |
| *HCF136* | 95.45877109 | 209.9209487 | 2.199074494 | 1.136896476 | 0.002238792 | 0.010124504 | - |
| *MTV1* | 226.4962338 | 496.1477176 | 2.190534073 | 1.131282656 | 0.002242505 | 0.010134337 | - |
| *NPC6* | 2.070896067 | 14.98627869 | 7.236615557 | 2.855315131 | 0.002244814 | 0.010143034 | K01114 |
| *DLO1* | 0 | 7.650151035 | Inf | Inf | 0.002252736 | 0.010174848 | - |
| *MED15A* | 43.56635873 | 94.09119818 | 2.159721421 | 1.110845233 | 0.002287282 | 0.010313707 | K14972 |
| *CIPK32* | 1.309532416 | 13.05633487 | 9.970226561 | 3.317626288 | 0.002291741 | 0.010330276 | K07198 |
| *SrpRalpha* | 29.7109433 | 69.54276205 | 2.340644703 | 1.226905958 | 0.002303027 | 0.010379374 | K13431 |
| *Os08g0191100* | 1.256644774 | 13.05481271 | 10.38862611 | 3.376932966 | 0.002319423 | 0.010446124 | K01681 |
| *CRRSP55* | 1.833723838 | 14.98455759 | 8.171654468 | 3.030628202 | 0.002347311 | 0.01055369 | - |
| *CRJ34* | 1.205401451 | 13.02095739 | 10.80217497 | 3.433249917 | 0.002363562 | 0.010617699 | - |
| *ACA5* | 15.62812579 | 44.52531576 | 2.849050254 | 1.510481069 | 0.002365521 | 0.010624687 | K01537 |
| *PGD1* | 26.50186459 | 68.57474581 | 2.587544192 | 1.371583502 | 0.002403414 | 0.01077652 | K00033 |
| *uncharacterized protein_10096* | 25.44395955 | 60.58127613 | 2.38096889 | 1.25154877 | 0.002421908 | 0.010852059 | - |
| *RAMDAZC7* | 276.0289626 | 2573.208312 | 9.322240275 | 3.220676698 | 0.002427784 | 0.010876539 | - |
| *TIR* | 77.65434589 | 216.7634647 | 2.791388714 | 1.48098304 | 0.002449924 | 0.010960823 | - |
| *HMGB7* | 51.50038999 | 104.6992102 | 2.032978977 | 1.023595297 | 0.002462333 | 0.011003262 | K11296 |
| *CEPR1* | 13.19486589 | 38.80510854 | 2.940924817 | 1.556269903 | 0.002470989 | 0.01103633 | - |
| *ywbO* | 54.73028138 | 110.5367889 | 2.019664181 | 1.014115429 | 0.002501612 | 0.011153844 | - |
| *L6* | 23.50141976 | 58.28895839 | 2.480231365 | 1.310474707 | 0.002504242 | 0.01116215 | - |
| *SGS3* | 5.868096369 | 24.3246273 | 4.1452331 | 2.051453232 | 0.002512604 | 0.011193886 | - |
| *GSTU19* | 897.3740513 | 2268.488619 | 2.527918671 | 1.33795005 | 0.002515653 | 0.011204597 | K00799 |
| *GSTU16* | 93.29391438 | 471.3555788 | 5.052372194 | 2.336960922 | 0.002539657 | 0.011300894 | K00799 |
| *OFUT19* | 189.5465242 | 434.1695644 | 2.290569907 | 1.195706594 | 0.002540889 | 0.011304468 | - |
| *ureH* | 228.0892206 | 482.6922323 | 2.11624307 | 1.081505344 | 0.002545352 | 0.011320502 | - |
| *LAX4* | 206.086827 | 542.5665971 | 2.632708772 | 1.39654794 | 0.002546852 | 0.011325263 | K13946 |
| *DDB_G0268948* | 0 | 10.82534073 | Inf | Inf | 0.002576376 | 0.011450752 | - |
| *BAG6* | 10760.43917 | 32003.5356 | 2.974184891 | 1.572494336 | 0.002628908 | 0.011658674 | - |
| *MST3* | 20.9191431 | 109.2917742 | 5.224486188 | 2.38528916 | 0.002660319 | 0.011784097 | - |
| *Acy1* | 48.02687307 | 97.99770882 | 2.040476561 | 1.028906139 | 0.002664642 | 0.01179928 | K14677 |
| *PAS1* | 43.89776779 | 91.00611713 | 2.073137695 | 1.051815941 | 0.00269236 | 0.011912009 | - |
| *EXL2* | 2.854716722 | 28.72408113 | 10.06197249 | 3.330841246 | 0.002701009 | 0.011940253 | - |
| *YSL12* | 63.32675446 | 170.3912176 | 2.690667144 | 1.42796393 | 0.002701683 | 0.01194123 | - |
| *ppt-1* | 2.894793533 | 17.32522586 | 5.984960816 | 2.581341802 | 0.002710229 | 0.011972981 | K01074 |
| *CLPX3* | 31.23038196 | 70.90872862 | 2.270504687 | 1.183013015 | 0.002736409 | 0.012076491 | K03544 |
| *At1g17710* | 38.2483234 | 344.0239277 | 8.994483865 | 3.169040497 | 0.002752835 | 0.012146946 | K13248 |
| *At1g78280* | 340.3083709 | 1526.960012 | 4.48698928 | 2.165747737 | 0.002762497 | 0.012183463 | - |
| *DDB_G0270580* | 256.1398343 | 669.0934034 | 2.61221924 | 1.385275986 | 0.002799442 | 0.012340207 | - |
| *GC2* | 54.31692218 | 109.6478412 | 2.018668157 | 1.01340377 | 0.002800788 | 0.012344077 | - |
| *At3g55350* | 93.83535366 | 316.3721554 | 3.371566718 | 1.753419147 | 0.002820944 | 0.012424598 | - |
| *XTH2* | 0.734097671 | 18.89260327 | 25.73581694 | 4.685705674 | 0.002825499 | 0.012440504 | K08235 |
| *ANT* | 28.58265479 | 888.4462786 | 31.08340653 | 4.958072717 | 0.002836191 | 0.01247924 | K05863 |
| *GPXMC1* | 34.92357528 | 209.561541 | 6.000575237 | 2.585100809 | 0.002853213 | 0.012539484 | K00432 |
| *ENO1* | 171.1087197 | 1978.0439 | 11.56015838 | 3.531089259 | 0.002888869 | 0.012672943 | K01689 |
| *ACO* | 0 | 25.80828313 | Inf | Inf | 0.002891676 | 0.012683143 | K05933 |
| *RWDD2B* | 43.27285802 | 90.62965876 | 2.094376542 | 1.066520844 | 0.00289668 | 0.01269875 | - |
| *N* | 16.00938182 | 55.42302021 | 3.461908826 | 1.79156773 | 0.002897589 | 0.012700624 | - |
| *YSL12* | 3.208954726 | 18.05493806 | 5.626423429 | 2.49221813 | 0.002900582 | 0.012709516 | - |
| *AAP2* | 1.243833943 | 12.63588063 | 10.15881638 | 3.344660416 | 0.002904919 | 0.012726404 | - |
| *GSO1* | 0.314161193 | 9.111296519 | 29.00197958 | 4.858079472 | 0.002923507 | 0.012797202 | - |
| *GLOX1* | 0 | 7.353766666 | Inf | Inf | 0.002928894 | 0.012816523 | - |
| *UFO* | 0 | 8.45568203 | Inf | Inf | 0.002929753 | 0.012816755 | - |
| *ENPL* | 96.67573922 | 214.1321508 | 2.214952299 | 1.147275629 | 0.002930441 | 0.012816911 | K09487 |
| *LECRKS5* | 0 | 7.348901772 | Inf | Inf | 0.002934456 | 0.012825958 | - |
| *PNC1* | 0 | 11.51795433 | Inf | Inf | 0.002943134 | 0.012861755 | K00430 |
| *CYP734A1* | 0 | 7.339271452 | Inf | Inf | 0.002945508 | 0.012865731 | K15639 |
| *CYP76T24* | 17.7276842 | 57.93317047 | 3.267949148 | 1.708385534 | 0.002955176 | 0.012903682 | - |
| *AFP3* | 0 | 7.321532975 | Inf | Inf | 0.002966007 | 0.01294883 | - |
| *LYK3* | 8.26596446 | 28.57253655 | 3.456648851 | 1.789374053 | 0.002969527 | 0.012959626 | - |
| *DDB_G0271664* | 16.44073277 | 44.81531307 | 2.725870781 | 1.446717174 | 0.0029738 | 0.012974254 | - |
| *uncharacterized protein_36659* | 35.78590524 | 80.27931092 | 2.243322067 | 1.165636759 | 0.002990276 | 0.013041818 | K19525 |
| *HSP12* | 11.73783706 | 128.0610082 | 10.91010274 | 3.447592782 | 0.002996546 | 0.013064841 | K13993 |
| *LECRK44* | 209.2011729 | 462.9887186 | 2.213126782 | 1.1460861 | 0.003012587 | 0.013130437 | - |
| *XTH2* | 3.668968015 | 18.74765113 | 5.109788653 | 2.353263621 | 0.003020605 | 0.013158854 | K08235 |
| *uncharacterized protein_22126* | 2.279281977 | 15.50171291 | 6.801138721 | 2.765776318 | 0.00304628 | 0.013261937 | - |
| *SD25* | 181.4331413 | 1669.669703 | 9.202672075 | 3.202052821 | 0.00306716 | 0.013346221 | - |
| *uncharacterized protein_27370* | 1.218212282 | 26.23206722 | 21.53324803 | 4.428494044 | 0.003070477 | 0.013356245 | - |
| *At1g56220* | 0.367048835 | 74.89005049 | 204.0329332 | 7.672658228 | 0.00307604 | 0.01337603 | - |
| *GIL1* | 2.266471146 | 15.43410173 | 6.809749931 | 2.76760182 | 0.003087995 | 0.013419157 | - |
| *DALL4* | 1.729468893 | 53.81856998 | 31.11855333 | 4.959703087 | 0.003105668 | 0.013489285 | - |
| *SETH3* | 38.17729181 | 280.4555884 | 7.346136278 | 2.87698566 | 0.003120825 | 0.013546191 | K06041 |
| *DCTPP1* | 25.56927514 | 65.7859561 | 2.57285182 | 1.36336837 | 0.003128154 | 0.013575767 | K16904 |
| *ADF* | 934.4607425 | 1948.419671 | 2.085073864 | 1.060098492 | 0.003144273 | 0.013638983 | K05765 |
| *VPS41* | 25.50497303 | 60.5747896 | 2.375018767 | 1.247938913 | 0.003158222 | 0.013685976 | K20184 |
| *PDL2* | 62.42208347 | 152.511098 | 2.443223447 | 1.288785813 | 0.003160866 | 0.013692928 | K01724 |
| *GOLS4* | 2.202293012 | 14.34019506 | 6.511483702 | 2.702986312 | 0.003170686 | 0.013726788 | K18819 |
| *CCL12* | 310.3251116 | 932.9198094 | 3.006265928 | 1.587972633 | 0.003194563 | 0.013813329 | - |
| *LOX1* | 0 | 7.443981214 | Inf | Inf | 0.003196364 | 0.013817184 | K15718 |
| *EDS1B* | 14.39710268 | 94.89359826 | 6.591159372 | 2.720532255 | 0.003222039 | 0.013914478 | - |
| *GIP2* | 5.948249991 | 38.18172131 | 6.418983964 | 2.682344957 | 0.003237784 | 0.013977893 | - |
| *GGCT2;2* | 195.6748741 | 403.6830478 | 2.063029552 | 1.044764487 | 0.003260192 | 0.014067719 | - |
| *MTP11* | 89.9238803 | 199.2425732 | 2.215680334 | 1.147749753 | 0.003269608 | 0.014099115 | - |
| *Ncoa7* | 17.69725356 | 45.97834665 | 2.598049832 | 1.377429103 | 0.003307624 | 0.014242074 | - |
| *E134* | 93.40449864 | 252.1991555 | 2.700075042 | 1.432999504 | 0.003341903 | 0.014375582 | - |
| *uncharacterized protein_20942* | 142.9753119 | 288.8611666 | 2.020356961 | 1.014610214 | 0.003382134 | 0.014539149 | - |
| *CRJ37* | 4.637197237 | 78.55098332 | 16.93932333 | 4.082304339 | 0.003396217 | 0.014597308 | - |
| *FRI3* | 5.972351314 | 24.33760036 | 4.075044999 | 2.026815991 | 0.003400013 | 0.01461124 | K00522 |
| *uncharacterized protein_24635* | 0 | 10.48050635 | Inf | Inf | 0.00342775 | 0.014718441 | - |
| *CYP74A* | 214.8094878 | 865.9565594 | 4.031277055 | 2.011236938 | 0.003435307 | 0.014743682 | K01723 |
| *NTR1* | 22.38885878 | 636.0961549 | 28.41128086 | 4.828391969 | 0.003464754 | 0.014857964 | K00384 |
| *PCR2* | 0 | 15.61615246 | Inf | Inf | 0.003471804 | 0.014885775 | - |
| *uncharacterized protein_24931* | 29.35974597 | 65.34136989 | 2.225542753 | 1.154157216 | 0.003483244 | 0.01492754 | - |
| *GFAT1* | 2.567821509 | 24.99960834 | 9.735726669 | 3.283288665 | 0.00348617 | 0.014937651 | K00820 |
| *HSP17.7* | 14.11008349 | 39.47826257 | 2.797875903 | 1.484331975 | 0.003487224 | 0.014939739 | K13993 |
| *uncharacterized protein_04768* | 12.24934163 | 37.18928757 | 3.03602338 | 1.602182901 | 0.003510165 | 0.01502581 | K15340 |
| *VIT_06s0061g00120* | 13.22709304 | 48.94230269 | 3.700155622 | 1.887585949 | 0.003514773 | 0.015043093 | - |
| *LOX3.1* | 336.1180718 | 2480.155848 | 7.378823265 | 2.883390761 | 0.00355433 | 0.015182807 | K00454 |
| *GULLO5* | 0.314161193 | 34.30713308 | 109.202326 | 6.770859776 | 0.00357093 | 0.015248775 | - |
| *BAM3* | 5.133998698 | 59.1652508 | 11.52420448 | 3.526595259 | 0.003587251 | 0.01530607 | K01177 |
| *uncharacterized protein_14832* | 3.484683428 | 18.49160861 | 5.306539028 | 2.407771229 | 0.003588847 | 0.0153104 | - |
| *AAO* | 0 | 51.7681044 | Inf | Inf | 0.003604418 | 0.015366878 | K00423 |
| *AERO1* | 313.1392273 | 2744.089923 | 8.763162463 | 3.131451606 | 0.003622902 | 0.01542572 | K10950 |
| *45506* | 49.61073783 | 100.0402781 | 2.01650454 | 1.011856654 | 0.003653611 | 0.015541409 | K16585 |
| *DHNAT1* | 32.82997425 | 71.54994737 | 2.179409183 | 1.123937087 | 0.003663636 | 0.015581538 | - |
| *PAL* | 31.48849086 | 469.7973491 | 14.91965275 | 3.899142052 | 0.003698024 | 0.015704981 | K10775 |
| *uncharacterized protein_02876* | 30.92267406 | 68.13645363 | 2.203446361 | 1.139761777 | 0.003734049 | 0.015835011 | - |
| *LOX1.5* | 0 | 7.083129463 | Inf | Inf | 0.003772083 | 0.015970866 | K15718 |
| *uncharacterized protein_29764* | 0.301350363 | 8.74233661 | 29.01053953 | 4.858505221 | 0.003801525 | 0.016082341 | - |
| *LARP6B* | 29.80378377 | 66.09845087 | 2.217787224 | 1.149120959 | 0.003806739 | 0.016099232 | K15191 |
| *MT2089* | 0 | 7.036400557 | Inf | Inf | 0.003841176 | 0.016213652 | - |
| *Os12g0628600* | 0.301350363 | 8.69246391 | 28.84504213 | 4.850251466 | 0.003861845 | 0.01629046 | - |
| *ACBP1* | 285.4006302 | 611.0652099 | 2.141078699 | 1.098337825 | 0.003864864 | 0.016297978 | - |
| *AAP3* | 0.314161193 | 8.623131634 | 27.44811203 | 4.778635014 | 0.003931704 | 0.016548068 | - |
| *PCO2* | 16.1393824 | 43.61851721 | 2.702613775 | 1.434355354 | 0.003949524 | 0.016612457 | K10712 |
| *LAT2* | 27.39146053 | 62.29349977 | 2.274194167 | 1.185355435 | 0.003958886 | 0.016641214 | K13510 |
| *uncharacterized protein_22925* | 0.314161193 | 8.594141205 | 27.35583319 | 4.773776592 | 0.003968167 | 0.016674906 | - |
| *CNR9* | 40.63921406 | 83.52706328 | 2.05533166 | 1.039371214 | 0.003999443 | 0.016782251 | - |
| *AAE6* | 37.78945851 | 289.3696692 | 7.657417719 | 2.93685796 | 0.004009448 | 0.016818878 | - |
| *ALA9* | 373.7180648 | 1106.844839 | 2.961710827 | 1.566430787 | 0.004011435 | 0.016824536 | K01530 |
| *BAC2* | 119.723351 | 327.9429366 | 2.739172716 | 1.453740237 | 0.004056411 | 0.016991538 | K15109 |
| *UGT86A1* | 0 | 13.83601279 | Inf | Inf | 0.004068272 | 0.017035807 | - |
| *TTM3* | 44.33215942 | 88.94753695 | 2.006388548 | 1.004601019 | 0.004080918 | 0.017080621 | - |
| *PGD3* | 5.67252129 | 22.47698881 | 3.962433575 | 1.986386751 | 0.004164487 | 0.017402761 | K00033 |
| *CPIJ013394* | 1.035448033 | 10.94930469 | 10.57446085 | 3.402512203 | 0.004167363 | 0.01741202 | - |
| *R40C1* | 0 | 13.83763442 | Inf | Inf | 0.004197127 | 0.017522491 | - |
| *GLR2.7* | 6.380997299 | 23.70114704 | 3.714332717 | 1.893103053 | 0.004256962 | 0.017752608 | K05387 |
| *DCTPP1* | 47.23684708 | 95.11252288 | 2.013523949 | 1.009722632 | 0.004291392 | 0.017879219 | K16904 |
| *CYP750A1* | 0 | 56.00880508 | Inf | Inf | 0.004408726 | 0.018313063 | - |
| *UGT85A8* | 3.143256254 | 78.53472836 | 24.9851498 | 4.642998963 | 0.00443553 | 0.018406896 | - |
| *At4g33300* | 44.96023384 | 112.7181093 | 2.507062345 | 1.325997873 | 0.004467764 | 0.018505797 | - |
| *Fra a 1.06* | 0 | 6.934934058 | Inf | Inf | 0.004502667 | 0.018644507 | - |
| *ACOT13* | 47.16302278 | 99.44132764 | 2.10845959 | 1.076189371 | 0.004504399 | 0.018648751 | K17362 |
| *Os12g0628600* | 0.314161193 | 17.03837235 | 54.23449076 | 5.761138729 | 0.004507069 | 0.018656875 | - |
| *MYB4* | 2.226394335 | 14.65269627 | 6.581357148 | 2.718385114 | 0.00452526 | 0.018726293 | K09422 |
| *XTH2* | 2.450755733 | 32.72700087 | 13.35384038 | 3.739182796 | 0.004558673 | 0.01883794 | K08235 |
| *APS1* | 11.44777719 | 34.34443704 | 3.000096567 | 1.585008939 | 0.004569736 | 0.018880697 | - |
| *BAG4* | 243.0500956 | 577.4416871 | 2.375813454 | 1.248421562 | 0.004577055 | 0.018907971 | - |
| *HSL1* | 1699.291159 | 3928.00921 | 2.311557492 | 1.208865245 | 0.004602487 | 0.018992193 | - |
| *XTH2* | 5.305596435 | 134.8562916 | 25.41774393 | 4.667764078 | 0.004614788 | 0.019034012 | K08235 |
| *At1g78830* | 1.415307699 | 18.28867561 | 12.92204912 | 3.691762959 | 0.004619039 | 0.019048568 | - |
| *crcB* | 36.74436431 | 154.1139781 | 4.194220828 | 2.068402822 | 0.004627348 | 0.019079845 | - |
| *ChiC* | 48.64492503 | 139.2755771 | 2.863106007 | 1.517581088 | 0.004635071 | 0.019102728 | K01183 |
| *GMPM1* | 0 | 14.16949575 | Inf | Inf | 0.004645372 | 0.019139194 | - |
| *FDH1* | 5.842474708 | 27.32415977 | 4.676812675 | 2.225525644 | 0.004646298 | 0.019140017 | K00122 |
| *uncharacterized protein_43773* | 8.03043655 | 52.9329521 | 6.591541041 | 2.720615793 | 0.004671792 | 0.019224004 | - |
| *ATX4* | 8.475994689 | 28.21493451 | 3.328805119 | 1.735004412 | 0.004685646 | 0.019271986 | - |
| *D6PKL1* | 4.545753122 | 19.95254871 | 4.389272399 | 2.133981807 | 0.004718713 | 0.019398907 | - |
| *uncharacterized protein_19196* | 45.06473675 | 127.28079 | 2.824398835 | 1.497943827 | 0.004742735 | 0.019491585 | - |
| *PGM1* | 556.5233963 | 1172.905339 | 2.107558006 | 1.075572338 | 0.00478657 | 0.019647227 | K15633 |
| *yoxD* | 241.9663209 | 831.7511928 | 3.437466792 | 1.781345776 | 0.004823283 | 0.019779437 | - |
| *BANGLUC* | 10.34979534 | 271.8576893 | 26.26696282 | 4.71517749 | 0.004829779 | 0.019796834 | - |
| *GSO1* | 1.506751814 | 18.90405416 | 12.54622957 | 3.649181961 | 0.004840472 | 0.019834495 | - |
| *uncharacterized protein_23181* | 12.1562532 | 35.2465632 | 2.899459449 | 1.535783962 | 0.004860605 | 0.019904614 | - |
| *uncharacterized protein_50920* | 0.301350363 | 15.24881418 | 50.60161216 | 5.661111445 | 0.004930868 | 0.020145398 | - |
| *SWEET3B* | 0 | 6.77224988 | Inf | Inf | 0.004937102 | 0.020167742 | K15382 |
| *Os04g0671100* | 16.75489396 | 43.60078517 | 2.602271627 | 1.37977156 | 0.004940359 | 0.020177916 | K00939 |
| *Os12g0628600* | 0 | 6.759376297 | Inf | Inf | 0.004961744 | 0.020258982 | - |
| *GT5* | 18.86447048 | 216.7405351 | 11.48935165 | 3.522225484 | 0.004966947 | 0.020277084 | K13691 |
| *uncharacterized protein_44683* | 0 | 6.754511402 | Inf | Inf | 0.004971099 | 0.02029089 | - |
| *GOLS2* | 0 | 6.704539234 | Inf | Inf | 0.005068555 | 0.020647115 | K18819 |
| *uncharacterized protein_17627* | 0 | 7.300551235 | Inf | Inf | 0.005077001 | 0.020675474 | - |
| *TMEM184A* | 31.56064262 | 68.05901319 | 2.156452072 | 1.108659652 | 0.005167703 | 0.020992598 | - |
| *MSSP2* | 0.916861919 | 10.7173877 | 11.68920585 | 3.547105013 | 0.005175338 | 0.021013897 | - |
| *At1g73050* | 0 | 6.64980164 | Inf | Inf | 0.00517823 | 0.021022403 | K08248 |
| *ACO* | 0 | 30.6200766 | Inf | Inf | 0.005185692 | 0.021046211 | K05933 |
| *ASNS* | 0 | 9.261007646 | Inf | Inf | 0.005244413 | 0.021251808 | K01953 |
| *UGT86A2* | 0.904051088 | 10.65616358 | 11.78712544 | 3.559140022 | 0.005266852 | 0.021329618 | - |
| *RMA3* | 18.06442638 | 45.52089973 | 2.51991947 | 1.333377629 | 0.005295143 | 0.021434311 | K10666 |
| *ERF017* | 0 | 7.0798862 | Inf | Inf | 0.005310488 | 0.021493124 | - |
| *DGAT2D* | 50.44580181 | 106.2167213 | 2.105561167 | 1.074204787 | 0.005317627 | 0.021512109 | K14457 |
| *LYK3* | 14.71430455 | 38.61505557 | 2.624320805 | 1.39194409 | 0.005402804 | 0.02181316 | - |
| *uncharacterized protein_47964* | 2.633519981 | 80.07728395 | 30.40693996 | 4.926328731 | 0.005403996 | 0.021814629 | - |
| *LIP1* | 2.487667886 | 16.56490161 | 6.658807513 | 2.735263837 | 0.005426264 | 0.021894462 | K01052 |
| *At1g56220* | 1.676581251 | 199.920497 | 119.2429516 | 6.897760182 | 0.005470392 | 0.022052255 | - |
| *At2g30000* | 38.04577089 | 77.49071371 | 2.036776017 | 1.026287337 | 0.005472502 | 0.022054017 | K12834 |
| *ANNAT8* | 3.813175791 | 17.47027747 | 4.581555751 | 2.195837575 | 0.005526118 | 0.02224288 | K17095 |
| *OBL1* | 0 | 9.489886747 | Inf | Inf | 0.005542277 | 0.022304511 | - |
| *ERG3* | 49.21617505 | 158.5674153 | 3.221855723 | 1.687891891 | 0.005544888 | 0.022311612 | - |
| *CHI4* | 9.824207557 | 31.43350393 | 3.199596889 | 1.677890154 | 0.005577633 | 0.022425783 | K01183 |
| *BACOVA_02659* | 95.59270844 | 558.151142 | 5.838846405 | 2.54568336 | 0.005603342 | 0.022505745 | K05349 |
| *DHNAT1* | 19.06600293 | 44.98772704 | 2.359578313 | 1.238529055 | 0.00560697 | 0.022509869 | - |
| *OsI_009784* | 1.205401451 | 24.05085275 | 19.95256661 | 4.318502435 | 0.005645742 | 0.022655172 | - |
| *YLS7* | 214.781602 | 485.1741943 | 2.25891878 | 1.175632399 | 0.005650686 | 0.022666647 | - |
| *At2g30650* | 27.23444192 | 60.36040571 | 2.216326146 | 1.148170199 | 0.005670617 | 0.022726939 | K05605 |
| *GSO1* | 5.316762947 | 21.75032095 | 4.090895375 | 2.032416641 | 0.005692997 | 0.02280499 | - |
| *MKK9* | 34.66546639 | 73.83556675 | 2.129945864 | 1.090816763 | 0.005749256 | 0.022986557 | K20604 |
| *VCR* | 32.90060568 | 92.26464734 | 2.804344949 | 1.487663819 | 0.005784717 | 0.023096955 | K12616 |
| *At1g07160* | 5.424058569 | 30.83090594 | 5.684102696 | 2.50693262 | 0.00578739 | 0.023103976 | K17506 |
| *CYP750A1* | 0 | 10.17773493 | Inf | Inf | 0.005811771 | 0.02319077 | - |
| *LETM1* | 123.5592318 | 265.7300919 | 2.150629201 | 1.104758805 | 0.005846402 | 0.023318369 | K17800 |
| *uncharacterized protein_16138* | 0 | 10.51598331 | Inf | Inf | 0.005848575 | 0.023321762 | - |
| *AMY2* | 76.88348827 | 164.8948929 | 2.144737402 | 1.100801017 | 0.005892491 | 0.02346668 | K01176 |
| *PAD4* | 90.40392982 | 224.3334338 | 2.48145666 | 1.311187258 | 0.005915164 | 0.023539193 | - |
| *IP5P8* | 36.43513607 | 75.93480007 | 2.084109139 | 1.05943083 | 0.005929666 | 0.023593337 | - |
| *EXLA1* | 13.29583219 | 36.60452752 | 2.753082845 | 1.461048024 | 0.005940005 | 0.023630907 | - |
| *DDB_G0268948* | 5.581325136 | 26.8714654 | 4.814531449 | 2.267395402 | 0.005954207 | 0.02368026 | - |
| *HSP18.1* | 0.681210029 | 9.310979814 | 13.66829527 | 3.772761414 | 0.005974194 | 0.023745417 | K13993 |
| *uncharacterized protein_14183* | 0 | 13.51225963 | Inf | Inf | 0.005987952 | 0.023778584 | - |
| *uncharacterized protein_24378* | 10.40103866 | 31.13073251 | 2.993040745 | 1.581611919 | 0.005994187 | 0.023789007 | - |
| *ANNAT8* | 0.367048835 | 8.122199075 | 22.12838809 | 4.467826459 | 0.006014571 | 0.023862718 | K17095 |
| *ADH2* | 14.47396766 | 58.35181058 | 4.031500687 | 2.011316968 | 0.006085347 | 0.024132622 | K18857 |
| *CESA7* | 11.60948079 | 50.59978229 | 4.358487964 | 2.123827726 | 0.00613859 | 0.024329125 | - |
| *Os01g0875500* | 29.32779499 | 63.74024306 | 2.17337318 | 1.119935914 | 0.006148358 | 0.024356849 | - |
| *At2g46260* | 7.58639875 | 25.35558876 | 3.342243085 | 1.740816666 | 0.006194903 | 0.024515444 | - |
| *At3g21360* | 1.101146506 | 10.32906769 | 9.380284667 | 3.229631705 | 0.006206122 | 0.024548785 | - |
| *AGD9* | 1059.127093 | 3559.603578 | 3.360884261 | 1.748840861 | 0.006239213 | 0.024659454 | K12493 |
| *ENO1* | 534.5287566 | 2226.468862 | 4.165292952 | 2.058417966 | 0.006254502 | 0.024703081 | K01689 |
| *EO* | 27.58867993 | 68.5377467 | 2.484270609 | 1.312822334 | 0.006263648 | 0.024724385 | K18980 |
| *ENO1* | 2185.296048 | 4472.632546 | 2.046694109 | 1.033295499 | 0.006311756 | 0.024895636 | K01689 |
| *HMG1* | 25.42001043 | 68.22312007 | 2.683835251 | 1.424296113 | 0.006321667 | 0.024927267 | K00021 |
| *R12* | 0.314161193 | 13.01437139 | 41.42577652 | 5.372456836 | 0.006395002 | 0.025175006 | K00430 |
| *UGT86A1* | 0.314161193 | 7.961030619 | 25.34059198 | 4.663378322 | 0.006428596 | 0.025288371 | - |
| *REV1* | 40.22259444 | 89.95093711 | 2.23632857 | 1.16113217 | 0.006457509 | 0.025394527 | K03515 |
| *ogdh* | 33.31256852 | 70.00659602 | 2.101507003 | 1.071424264 | 0.006461793 | 0.025400001 | K00164 |
| *CYP78A4* | 7.360517013 | 49.96698299 | 6.788515386 | 2.763096099 | 0.006471837 | 0.025431896 | - |
| *CHS* | 42.07685478 | 195.0665646 | 4.635958785 | 2.21286774 | 0.006484344 | 0.025469653 | K00660 |
| *OMT2* | 0.367048835 | 555.0670112 | 1512.242944 | 10.56247421 | 0.006491152 | 0.025492253 | - |
| *JMJ706* | 164.0274962 | 594.4947793 | 3.624360507 | 1.857726464 | 0.006537117 | 0.025638729 | - |
| *At3g03770* | 8.9601093 | 28.43722117 | 3.17375829 | 1.666192258 | 0.006544027 | 0.025658194 | - |
| *KIC* | 0 | 6.424271711 | Inf | Inf | 0.006558475 | 0.025711018 | - |
| *Tango2* | 76.5100819 | 164.3216159 | 2.147711933 | 1.102800502 | 0.006572158 | 0.025753166 | - |
| *XA21* | 1.362420058 | 53.17956968 | 39.03316703 | 5.286628618 | 0.006600215 | 0.025851579 | K04730 |
| *AAE18* | 84.24909044 | 292.3011166 | 3.469486912 | 1.794722324 | 0.00666466 | 0.026072995 | - |
| *ERF114* | 0 | 6.366290854 | Inf | Inf | 0.006707963 | 0.02622294 | - |
| *P85* | 3.681778845 | 16.94835674 | 4.603306567 | 2.202670525 | 0.006716063 | 0.026246186 | K19366 |
| *FAB1A* | 3.563192731 | 17.06594008 | 4.789507997 | 2.259877463 | 0.006716897 | 0.026246186 | K00921 |
| *ACO3* | 2.030819256 | 13.07893825 | 6.440227612 | 2.687111677 | 0.006741164 | 0.026331128 | K01681 |
| *P4H7* | 188.1694011 | 615.7678889 | 3.27241244 | 1.710354591 | 0.006817068 | 0.026586364 | K00472 |
| *BRG3* | 180.9308828 | 709.5895698 | 3.921881986 | 1.971546124 | 0.006844952 | 0.026687214 | K19042 |
| *ADH2* | 28.96847183 | 60.64898677 | 2.09362051 | 1.065999963 | 0.006853603 | 0.026712845 | K18857 |
| *Os01g0706700* | 0 | 6.306688366 | Inf | Inf | 0.006866432 | 0.026755126 | K19327 |
| *EO* | 2.59344317 | 14.58832836 | 5.625081176 | 2.491873916 | 0.006962326 | 0.027084732 | K18980 |
| *ALP1* | 17.78705336 | 43.92129507 | 2.469284496 | 1.304093064 | 0.006990334 | 0.027181652 | - |
| *CIPK8* | 31.46122488 | 66.70429213 | 2.120206457 | 1.084204755 | 0.007024988 | 0.027281876 | - |
| *Dctpp1* | 7.336415691 | 25.19939111 | 3.43483687 | 1.780241583 | 0.007031175 | 0.027300185 | K16904 |
| *DES5* | 16.79497077 | 46.81611777 | 2.787508142 | 1.478976019 | 0.007062729 | 0.027410584 | - |
| *45569* | 95.09907163 | 219.8352163 | 2.311644189 | 1.208919354 | 0.007070325 | 0.027427948 | K08202 |
| *GSO1* | 1.243833943 | 16.36826264 | 13.15952401 | 3.718035402 | 0.007102614 | 0.027528896 | - |
| *TIFY10B* | 1.205401451 | 51.81804436 | 42.98820474 | 5.425868957 | 0.007129795 | 0.027606537 | K13464 |
| *PGD1* | 39.50127938 | 95.23680457 | 2.410980253 | 1.269619835 | 0.007129978 | 0.027606537 | K00033 |
| *SUC2* | 0.904051088 | 11.3182646 | 12.51949668 | 3.646104658 | 0.007174433 | 0.027754198 | K15378 |
| *NPF5.3* | 0.681210029 | 9.111296519 | 13.37516498 | 3.741484781 | 0.007229997 | 0.027947919 | K14638 |
| *HVA22A* | 1.769545704 | 11.96252767 | 6.760225317 | 2.757071332 | 0.007279658 | 0.028107629 | K17279 |
| *LYSMD1* | 308.90906 | 650.5192604 | 2.105860088 | 1.074409588 | 0.007299957 | 0.028179671 | - |
| *TAF1* | 22.25606547 | 52.05788989 | 2.339042809 | 1.225918266 | 0.007322075 | 0.028250712 | K03125 |
| *ERD4* | 37.7269247 | 225.0369164 | 5.964888954 | 2.57649528 | 0.007330203 | 0.028277931 | - |
| *PAP4* | 0.628322387 | 9.109674887 | 14.4984089 | 3.857822678 | 0.007333759 | 0.02828337 | K14379 |
| *COMT1* | 0 | 187.8984354 | Inf | Inf | 0.007357276 | 0.028358013 | - |
| *DHNAT1* | 20.07126829 | 47.19135239 | 2.351189357 | 1.233390734 | 0.007375773 | 0.028420448 | - |
| *TIFY10B* | 0.301350363 | 17.30110677 | 57.41193278 | 5.84327872 | 0.007392011 | 0.028478853 | K13464 |
| *TH2* | 5.711077762 | 21.63770197 | 3.788724802 | 1.921712352 | 0.007469745 | 0.028757318 | - |
| *PLP6* | 0.602700725 | 9.064567613 | 15.03991489 | 3.910724497 | 0.007471327 | 0.028759209 | - |
| *CYP709B2* | 0 | 7.64366451 | Inf | Inf | 0.007520971 | 0.028920735 | - |
| *RCOM_1506700* | 25.99060798 | 91.3982901 | 3.516589153 | 1.814176793 | 0.007522994 | 0.02892138 | K01267 |
| *At1g71810* | 17.73745435 | 43.74573731 | 2.466291749 | 1.302343473 | 0.007577322 | 0.029069565 | - |
| *hxnY* | 187.2187917 | 708.9014076 | 3.786486395 | 1.920859745 | 0.007606792 | 0.029169882 | - |
| *SWEET16* | 0.301350363 | 20.46635486 | 67.91548107 | 6.085668564 | 0.007633578 | 0.029262521 | K15382 |
| *ABCG34* | 3.825986621 | 16.68572822 | 4.36115697 | 2.124710918 | 0.007634281 | 0.029262521 | - |
| *copb2* | 2.266471146 | 14.24683671 | 6.285911356 | 2.652121927 | 0.007663119 | 0.029360252 | K17302 |
| *Os09g0505700* | 256.1829236 | 704.731426 | 2.750891497 | 1.459899237 | 0.007777372 | 0.029728848 | K01783 |
| *ZIP5* | 7.451713167 | 24.7563335 | 3.322233819 | 1.732153614 | 0.007928898 | 0.030246638 | K14709 |
| *ROQ1* | 0.314161193 | 7.741987215 | 24.64335945 | 4.623127036 | 0.007933992 | 0.030259724 | - |
| *SYP81* | 15.60402447 | 69.44110305 | 4.450204701 | 2.153871699 | 0.007984003 | 0.030429396 | K08492 |
| *RAV2* | 46.25428669 | 111.2203754 | 2.404542009 | 1.265762131 | 0.008053877 | 0.030670132 | K09287 |
| *cfxQ* | 128.6596352 | 474.5756331 | 3.688613234 | 1.883078525 | 0.008143388 | 0.030957326 | - |
| *URIC1* | 13.19157725 | 34.719784 | 2.631966091 | 1.396140902 | 0.0081503 | 0.030979133 | K00365 |
| *ATJ20* | 22.86028654 | 52.14496065 | 2.281028305 | 1.189684349 | 0.008174404 | 0.031061791 | - |
| *HSP18.1* | 2.070896067 | 16.33927221 | 7.889952797 | 2.980016669 | 0.008196759 | 0.031133274 | K13993 |
| *OMT2* | 0 | 241.1663125 | Inf | Inf | 0.008199238 | 0.0311382 | - |
| *EPHX2* | 4.874369466 | 23.27593381 | 4.775168148 | 2.255551536 | 0.008228992 | 0.031220535 | - |
| *TCEA1* | 10.50845826 | 30.0642941 | 2.86096146 | 1.516500063 | 0.008232997 | 0.031225913 | - |
| *WRKY57* | 20.48791613 | 47.69401893 | 2.327909712 | 1.219035104 | 0.008240888 | 0.031249328 | - |
| *uncharacterized protein_35255* | 2.018008425 | 12.64551095 | 6.266332091 | 2.647621229 | 0.008241542 | 0.031249328 | - |
| *SBT6.1* | 18.36754504 | 46.88523823 | 2.552613217 | 1.351974951 | 0.008256083 | 0.031295457 | K08653 |
| *NRPB3* | 0.314161193 | 7.608287024 | 24.21778113 | 4.597994784 | 0.008270472 | 0.031345494 | K03011 |
| *CYP750A1* | 144.4046745 | 440.2414933 | 3.048665112 | 1.608177683 | 0.008387261 | 0.031726391 | - |
| *uncharacterized protein_00637* | 21.51207367 | 48.32215871 | 2.246280831 | 1.167538305 | 0.008387824 | 0.031726391 | K06100 |
| *At3g55350* | 243.012283 | 599.9764642 | 2.468914151 | 1.303876672 | 0.008458108 | 0.031964711 | - |
| *TOM1* | 69.81163477 | 154.0816385 | 2.207105435 | 1.14215555 | 0.008506424 | 0.032115072 | - |
| *BANGLUC* | 615.3460143 | 10479.34004 | 17.02999581 | 4.090006175 | 0.008602296 | 0.032439852 | - |
| *VIT_06s0061g00120* | 17.95889905 | 54.61263076 | 3.040978771 | 1.604535745 | 0.008622436 | 0.032506499 | - |
| *uncharacterized protein_42229* | 43.71512752 | 104.3657401 | 2.387405596 | 1.255443687 | 0.008628552 | 0.032524902 | - |
| *EXPA2* | 0 | 16.15417716 | Inf | Inf | 0.008672893 | 0.03265468 | - |
| *SRC2* | 35.80327709 | 388.1581275 | 10.84141339 | 3.438480947 | 0.008683228 | 0.032688924 | - |
| *zgc:73324* | 87.2463424 | 704.2439223 | 8.071901962 | 3.012908652 | 0.008686055 | 0.032693637 | K18588 |
| *EOBII* | 0 | 6.081158436 | Inf | Inf | 0.008700088 | 0.032733692 | K09422 |
| *HAT14* | 22.82008575 | 66.51708455 | 2.914848142 | 1.543420724 | 0.008728371 | 0.032816683 | K09338 |
| *uncharacterized protein_02165* | 38.25567714 | 82.34151936 | 2.152399997 | 1.10594621 | 0.008730168 | 0.032818756 | - |
| *PAO1* | 0 | 6.069906485 | Inf | Inf | 0.008738154 | 0.032834729 | K13366 |
| *At2g30020* | 5.64537929 | 35.98581268 | 6.374383515 | 2.672285822 | 0.008748618 | 0.032869362 | K17506 |
| *RCOM_0530710* | 103.9673649 | 539.608374 | 5.190170729 | 2.375781996 | 0.008796245 | 0.033015357 | - |
| *FRI3* | 10.92523008 | 31.05349101 | 2.842364946 | 1.507091801 | 0.008803601 | 0.033030003 | K00522 |
| *uncharacterized protein_13876* | 0 | 6.048924745 | Inf | Inf | 0.008809744 | 0.033042496 | - |
| *NAC056* | 0 | 6.045681482 | Inf | Inf | 0.008820882 | 0.033074855 | - |
| *ROQ1* | 6.339400149 | 22.92481185 | 3.616243069 | 1.854491653 | 0.00882582 | 0.033088666 | - |
| *VHA-a2* | 13.21896721 | 34.92108893 | 2.641741097 | 1.401489082 | 0.008887737 | 0.033287649 | K02154 |
| *RR22* | 0 | 6.021555948 | Inf | Inf | 0.008904331 | 0.033330853 | K14491 |
| *CYCT1-3* | 18.69439308 | 44.42070547 | 2.376151249 | 1.248626671 | 0.008972767 | 0.033548906 | K15188 |
| *LOX1.1* | 0 | 15.64027799 | Inf | Inf | 0.009030424 | 0.03373577 | K15718 |
| *MUCI70* | 27.66045976 | 56.43797073 | 2.040384405 | 1.028840979 | 0.009069203 | 0.033856646 | - |
| *GLR3.4* | 0 | 5.958710197 | Inf | Inf | 0.009126808 | 0.034057219 | K05387 |
| *SFC1* | 6.784958288 | 23.19859285 | 3.419120923 | 1.773625447 | 0.009145585 | 0.034103148 | K15100 |
| *MARCHF1* | 46.36487095 | 94.0898814 | 2.029335561 | 1.021007442 | 0.009179615 | 0.034211723 | - |
| *BGAL8* | 4.350178043 | 21.18977946 | 4.871014301 | 2.284222219 | 0.009204432 | 0.03429347 | - |
| *UGT75C1* | 0 | 7.977246933 | Inf | Inf | 0.009234168 | 0.034394537 | K13691 |
| *APS1* | 6.746525796 | 22.99231712 | 3.408023302 | 1.7689352 | 0.009317875 | 0.034691611 | - |
| *ABCG34* | 0.681210029 | 8.655365325 | 12.70586891 | 3.667423134 | 0.009350471 | 0.034788399 | - |
| *Rnf14* | 13.41606263 | 38.56326283 | 2.87440987 | 1.523265794 | 0.009382229 | 0.034877013 | K11971 |
| *AGL80* | 13.49305159 | 35.51396358 | 2.632018661 | 1.396169718 | 0.009402394 | 0.034936885 | K04454 |
| *FLS2* | 38.96123645 | 259.5840574 | 6.662623701 | 2.736090414 | 0.00949975 | 0.035229411 | K13420 |
| *GTE9* | 8.227407988 | 24.84492641 | 3.019775663 | 1.594441377 | 0.009532109 | 0.035339467 | - |
| *LRK10L-1.2* | 26.50502925 | 55.60182768 | 2.097784053 | 1.068866174 | 0.009546631 | 0.035388326 | - |
| *SUS3* | 0.995371222 | 9.684705148 | 9.729741961 | 3.282401544 | 0.009638809 | 0.035684851 | K00695 |
| *SCPL27* | 2.555010678 | 13.97772167 | 5.470709689 | 2.451727999 | 0.009714937 | 0.035931361 | K16297 |
| *uncharacterized protein_34862* | 19.37560311 | 45.15690418 | 2.330606378 | 1.220705365 | 0.009875889 | 0.036429522 | - |
| *At4g26790* | 1.977931614 | 12.29763225 | 6.217420343 | 2.636316119 | 0.009962808 | 0.036724443 | - |
| *SKIP2* | 7.507889447 | 24.11845748 | 3.212415107 | 1.68365833 | 0.010011991 | 0.03687995 | - |
| *CRK8* | 21.78628203 | 48.62837234 | 2.23206384 | 1.158378291 | 0.010028104 | 0.036928981 | - |
| *ATL11* | 14.46280115 | 79.34982241 | 5.48647676 | 2.455879994 | 0.010035044 | 0.036941685 | K10664 |
| *SEC23* | 12.95756968 | 33.44393251 | 2.581034356 | 1.367949345 | 0.01010436 | 0.037163062 | K14006 |
| *CRK8* | 2.803473399 | 14.75264061 | 5.26227237 | 2.395685923 | 0.010133669 | 0.037250067 | - |
| *Os12g0628600* | 0.314161193 | 7.402117203 | 23.56152624 | 4.55836109 | 0.010162962 | 0.037342119 | - |
| *HAL3B* | 38.01686059 | 84.40192011 | 2.220118095 | 1.15063642 | 0.010183322 | 0.037411606 | K01598 |
| *RAB7* | 28.95603294 | 60.17693873 | 2.07821765 | 1.055346754 | 0.010242921 | 0.037599224 | K07897 |
| *rcc2* | 30.58583613 | 62.11631393 | 2.030884939 | 1.022108505 | 0.010296627 | 0.037764808 | - |
| *At1g73050* | 0 | 12.55225852 | Inf | Inf | 0.010304249 | 0.037782245 | - |
| *TPRL3* | 10.13824477 | 29.49565089 | 2.909344917 | 1.540694345 | 0.010310524 | 0.037799996 | K11147 |
| *ERF1* | 0.314161193 | 7.350523403 | 23.39729908 | 4.548270094 | 0.010325313 | 0.037843687 | K09286 |
| *ALDH1* | 0.314161193 | 7.350523403 | 23.39729908 | 4.548270094 | 0.010325313 | 0.037843687 | K12355 |
| *PUB44* | 350.9559518 | 851.8604157 | 2.427257356 | 1.279327082 | 0.010413681 | 0.038093403 | - |
| *At4g33300* | 125.6992672 | 406.6301212 | 3.234944247 | 1.693740848 | 0.010477505 | 0.038273752 | - |
| *UGT86A1* | 0.301350363 | 7.311902655 | 24.26379245 | 4.600733157 | 0.010490637 | 0.03831641 | - |
| *EXPA1* | 0.301350363 | 7.310281024 | 24.25841123 | 4.600413161 | 0.010495907 | 0.03832061 | - |
| *NRT3.1* | 27.72184517 | 69.97112551 | 2.524042865 | 1.335736411 | 0.010497833 | 0.03832061 | - |
| *CCN2* | 2.253660315 | 13.15141432 | 5.835579669 | 2.544875971 | 0.010528208 | 0.038405739 | K05868 |
| *YUC8* | 6.536495567 | 21.97595035 | 3.362038591 | 1.749336285 | 0.01061492 | 0.038631158 | K11816 |
| *PHT1-5* | 351.9770969 | 2343.797081 | 6.658947704 | 2.73529421 | 0.010643844 | 0.038725726 | K08176 |
| *NPF5.7* | 12.36628342 | 134.8523283 | 10.90483888 | 3.446896549 | 0.010678367 | 0.038819179 | K14638 |
| *LBD40* | 1.638148759 | 11.37138056 | 6.941604356 | 2.79526914 | 0.010727484 | 0.038986978 | - |
| *dnajb6-b* | 3.970318377 | 16.73083549 | 4.213978302 | 2.075182886 | 0.010731416 | 0.03899589 | - |
| *OBAP2C* | 0.734097671 | 14.53024803 | 19.79334442 | 4.306943495 | 0.010815644 | 0.03928571 | - |
| *EMH5* | 0 | 7.576053333 | Inf | Inf | 0.010896318 | 0.039540597 | - |
| *EDA2* | 355.7681876 | 997.6448769 | 2.804199227 | 1.487588851 | 0.010924638 | 0.039632453 | - |
| *UGT85A2* | 29.8183629 | 96.09950135 | 3.222829559 | 1.688327893 | 0.010966148 | 0.039758428 | - |
| *At4g34480* | 5.592491648 | 20.66300027 | 3.694775347 | 1.885486647 | 0.011127245 | 0.040273232 | - |
| *At5g63930* | 26.17057951 | 330.2814408 | 12.62033348 | 3.657678127 | 0.011130353 | 0.04027895 | - |
| *PPX2* | 1.415307699 | 10.40965191 | 7.355045068 | 2.878734182 | 0.011132552 | 0.040281374 | K15423 |
| *HAT14* | 73.52067959 | 179.5097098 | 2.441622014 | 1.287839875 | 0.011160115 | 0.040364484 | K09338 |
| *KIN13A* | 9.182950359 | 27.46748384 | 2.991139314 | 1.580695106 | 0.011164439 | 0.040374581 | K10393 |
| *ANR* | 0 | 34.30276553 | Inf | Inf | 0.011202315 | 0.040485398 | K08695 |
| *ADC* | 1956.991397 | 6176.265036 | 3.156000097 | 1.658097249 | 0.011204014 | 0.040485398 | K01583 |
| *UGT91C1* | 28.32442192 | 79.89716614 | 2.820787177 | 1.496097821 | 0.011204298 | 0.040485398 | - |
| *uncharacterized protein_03622* | 5.736699424 | 65.74278174 | 11.46003597 | 3.518539667 | 0.011502357 | 0.041431749 | - |
| *ROQ1* | 0 | 5.739666793 | Inf | Inf | 0.011539143 | 0.041539039 | - |
| *RAMDAZC7* | 0 | 5.738045162 | Inf | Inf | 0.011546408 | 0.041550672 | - |
| *ERF113* | 0.615511556 | 8.428213764 | 13.69302278 | 3.775369057 | 0.01162374 | 0.041787222 | - |
| *LOX1.1* | 0 | 5.720306685 | Inf | Inf | 0.011626284 | 0.041787222 | K15718 |
| *EXLA1* | 0 | 5.720306685 | Inf | Inf | 0.011626284 | 0.041787222 | - |
| *GDPDL7* | 1.939499122 | 12.15430818 | 6.266725284 | 2.647711751 | 0.011626411 | 0.041787222 | - |
| *PAP1* | 27.05167768 | 58.42123589 | 2.159615998 | 1.110774809 | 0.011666435 | 0.041913926 | - |
| *DLO1* | 0 | 15.31328156 | Inf | Inf | 0.011670507 | 0.041922841 | - |
| *POLX* | 29.70129713 | 60.45406246 | 2.035401424 | 1.025313352 | 0.011701545 | 0.042011432 | K05658 |
| *LECRKS5* | 0 | 5.688072993 | Inf | Inf | 0.011773395 | 0.042212316 | - |
| *rplX* | 6.850656761 | 22.35119784 | 3.262635777 | 1.706037942 | 0.011793276 | 0.04226905 | K02895 |
| *At1g73050* | 0 | 5.683307568 | Inf | Inf | 0.011795362 | 0.04226905 | - |
| *TOPP3* | 0.982560392 | 9.393185674 | 9.559906703 | 3.256996539 | 0.011802083 | 0.042285986 | K06269 |
| *uncharacterized protein_13314* | 0 | 5.680064305 | Inf | Inf | 0.011810344 | 0.04230408 | - |
| *AZF2* | 6.733714965 | 50.41500497 | 7.48695263 | 2.904378627 | 0.011816418 | 0.042320087 | - |
| *PR4B* | 9.339968966 | 52.36907432 | 5.606985903 | 2.487225442 | 0.011898522 | 0.042573641 | - |
| *ERH1* | 218.0340743 | 504.0441012 | 2.311767565 | 1.208996351 | 0.011901104 | 0.0425771 | - |
| *rsmF* | 16.91507722 | 39.37537338 | 2.327826995 | 1.21898384 | 0.011980277 | 0.042796448 | - |
| *HYP1* | 1.808102176 | 12.06085037 | 6.670447351 | 2.737783518 | 0.012064764 | 0.043063235 | - |
| *BIP131* | 5.161264679 | 29.0221866 | 5.623076592 | 2.491359698 | 0.012095867 | 0.043150875 | - |
| *TIP1-1* | 9.485945041 | 27.82347069 | 2.933125859 | 1.552438978 | 0.012101033 | 0.043157621 | K09873 |
| *SAMDC* | 17.80467316 | 44.77679823 | 2.51489021 | 1.330495419 | 0.012130997 | 0.043241083 | K01611 |
| *GPXMC1* | 0 | 5.625227242 | Inf | Inf | 0.012229258 | 0.04353246 | K00432 |
| *uncharacterized protein_49086* | 41.88292402 | 122.8537076 | 2.933264819 | 1.552507326 | 0.012302847 | 0.043723548 | - |
| *OBL1* | 0 | 57.7560104 | Inf | Inf | 0.012315264 | 0.043761777 | - |
| *GTE9* | 49.03542707 | 135.4529934 | 2.762349622 | 1.465895928 | 0.012369895 | 0.043904351 | - |
| *At1g67480* | 0 | 8.048001904 | Inf | Inf | 0.012370379 | 0.043904351 | - |
| *PILS2* | 7.857318475 | 24.76444165 | 3.151767582 | 1.656161151 | 0.012381924 | 0.043927579 | - |
| *uncharacterized protein_45487* | 18.17336632 | 41.44530498 | 2.280551894 | 1.189382999 | 0.01242015 | 0.044045408 | - |
| *LOX1.4* | 0 | 17.01921762 | Inf | Inf | 0.012428999 | 0.044070859 | K15718 |
| *At2g24130* | 11.2681776 | 233.6618944 | 20.73644051 | 4.374096366 | 0.012440255 | 0.044104837 | - |
| *LOX1.1* | 0 | 9.515633913 | Inf | Inf | 0.01248895 | 0.04426557 | K15718 |
| *GOLS4* | 4.324556382 | 17.05296703 | 3.943287015 | 1.979398722 | 0.012511356 | 0.04433902 | K18819 |
| *ALKBH8* | 25.68125576 | 54.5595148 | 2.124487809 | 1.087115065 | 0.012548545 | 0.0444557 | K10770 |
| *AAO* | 0.602700725 | 65.05960596 | 107.9467855 | 6.754176473 | 0.012579981 | 0.044534314 | K00423 |
| *JMJD7* | 5.292661624 | 18.942569 | 3.579025138 | 1.839566677 | 0.012593611 | 0.044576579 | K19219 |
| *ADC2* | 1.782356535 | 11.02978945 | 6.188318237 | 2.62954739 | 0.012625309 | 0.044668671 | K01583 |
| *At4g27270* | 110.0422028 | 220.4820264 | 2.003613348 | 1.002604128 | 0.012744292 | 0.045025276 | K03809 |
| *NFD4* | 2.292092807 | 12.63273684 | 5.511442119 | 2.462429863 | 0.012787483 | 0.045135516 | - |
| *At2g23790* | 1.256644774 | 10.79625083 | 8.591330705 | 3.102881607 | 0.012794627 | 0.045154686 | - |
| *GT4* | 0.367048835 | 7.055760666 | 19.22294797 | 4.264757695 | 0.012828268 | 0.045249174 | - |
| *CESA7* | 7.387659013 | 23.38053767 | 3.164810074 | 1.662118923 | 0.012852182 | 0.045315334 | - |
| *SAP8* | 25.52094852 | 190.4844825 | 7.463848077 | 2.899919621 | 0.012907676 | 0.045474497 | - |
| *uncharacterized protein_35377* | 8.764534221 | 25.79692527 | 2.943331 | 1.557449793 | 0.012945588 | 0.04558978 | - |
| *R12* | 12.46724973 | 102.5472974 | 8.225334346 | 3.040074323 | 0.012974956 | 0.045680997 | K00430 |
| *COMT1* | 0.314161193 | 228.031108 | 725.8411056 | 9.503509951 | 0.013029179 | 0.045836853 | - |
| *SCRM2* | 9.429644781 | 28.96410628 | 3.071600993 | 1.618990819 | 0.013093122 | 0.046041669 | - |
| *GBF* | 364.7368948 | 1033.364195 | 2.833177036 | 1.502420754 | 0.013242064 | 0.046484796 | K06630 |
| *GDPD6* | 166.1063377 | 370.0641951 | 2.227875229 | 1.155668437 | 0.013275081 | 0.046575883 | K01126 |
| *JOX4* | 37.83409634 | 253.8099513 | 6.70849778 | 2.745989743 | 0.013288049 | 0.046608971 | - |
| *FEY* | 3.837277113 | 23.40304157 | 6.09886669 | 2.608541182 | 0.013353743 | 0.046820708 | K11153 |
| *uncharacterized protein_20061* | 11.59198496 | 35.03229166 | 3.022113277 | 1.595557738 | 0.013384291 | 0.046909093 | - |
| *AMY1.3* | 18.59813999 | 42.77102917 | 2.299747673 | 1.201475578 | 0.013443231 | 0.047071847 | K01176 |
| *ME1* | 341.9736053 | 4212.429669 | 12.31799649 | 3.622695717 | 0.013565656 | 0.047456388 | K00029 |
| *NADP-ME4* | 3.667447676 | 36.22117831 | 9.876399477 | 3.303985191 | 0.013700054 | 0.047884783 | K00029 |
| *uncharacterized protein_14427* | 3.013379647 | 14.02282895 | 4.653522154 | 2.218323075 | 0.013702391 | 0.047884783 | - |
| *Cht1* | 2.827450741 | 14.14699829 | 5.003446419 | 2.322922179 | 0.01381187 | 0.048189785 | K20547 |
| *WRKY57* | 10.50681394 | 28.3921139 | 2.702257226 | 1.43416501 | 0.01388392 | 0.048396323 | - |
| *UGT86A2* | 30.27825221 | 76.664294 | 2.531992054 | 1.340272877 | 0.013981982 | 0.048673771 | - |
| *CBK1* | 11.54226198 | 29.99830455 | 2.598997026 | 1.377954983 | 0.014012186 | 0.048759597 | - |
| *GOLS4* | 12.0889104 | 43.65208056 | 3.610919355 | 1.8523662 | 0.014050408 | 0.048848187 | K18819 |
| *GOX* | 5.804042215 | 20.01549393 | 3.448543824 | 1.785987301 | 0.014111524 | 0.049008214 | K11517 |
| *SDR1* | 1.899422311 | 11.764466 | 6.193707391 | 2.630803228 | 0.014118684 | 0.049025688 | K00059 |
| *PATL4* | 5.345549266 | 18.75890308 | 3.50925642 | 1.811165369 | 0.01413775 | 0.049072911 | K19996 |
| *NPR4* | 3.249031537 | 14.69141649 | 4.521783281 | 2.176891849 | 0.014208293 | 0.049285834 | - |
| *CSN2* | 7.546321939 | 23.05201907 | 3.054735706 | 1.611047564 | 0.014292816 | 0.049539774 | K12176 |
| *PUB24* | 8.804611032 | 25.55537796 | 2.902499369 | 1.537295753 | 0.014344945 | 0.049687919 | - |
| *uncharacterized protein_28331* | 20.86789977 | 46.17489259 | 2.212723517 | 1.145823196 | 0.014470536 | 0.050071156 | - |
| *At1g77840* | 4.664339237 | 17.33009076 | 3.715443898 | 1.893534585 | 0.014482197 | 0.050097493 | K03262 |
| *NUP43* | 12.85027405 | 32.91400952 | 2.561346893 | 1.356902656 | 0.014512083 | 0.050181114 | K14305 |
| *MST3* | 0.602700725 | 8.089965384 | 13.42285655 | 3.746619823 | 0.014541446 | 0.050262866 | - |
| *CHS* | 107.0367011 | 1450.967564 | 13.55579488 | 3.760837806 | 0.01455758 | 0.050306235 | K00660 |
| *UGT85A19* | 107.772815 | 414.993799 | 3.850635237 | 1.945096466 | 0.014772969 | 0.050942871 | - |
| *vps13A* | 27.62748436 | 74.37057289 | 2.691905348 | 1.428627683 | 0.014856703 | 0.051189648 | K19525 |
| *Os02g0190300* | 137.5797352 | 738.9498381 | 5.371066 | 2.42520845 | 0.015160706 | 0.052068664 | K05658 |
| *HSL1* | 0 | 5.411048733 | Inf | Inf | 0.015235311 | 0.052270485 | K00924 |
| *SCPL49* | 87.56835324 | 268.8375001 | 3.070030326 | 1.618252907 | 0.015289742 | 0.05242311 | K16298 |
| *At5g47540* | 43.72502165 | 95.03720786 | 2.173519972 | 1.120033353 | 0.015322328 | 0.052514339 | K08272 |
| *LYM1* | 13.36153067 | 33.17167368 | 2.48262527 | 1.311866516 | 0.015338649 | 0.052550478 | - |
| *uncharacterized protein_15834* | 8.056058212 | 24.07811563 | 2.98882096 | 1.579576477 | 0.015360174 | 0.052609843 | - |
| *ogdh* | 15.34923243 | 48.81012466 | 3.179971694 | 1.669013924 | 0.015366104 | 0.052623314 | K00164 |
| *PCO1* | 52.07405643 | 133.4375618 | 2.562457603 | 1.357528135 | 0.015393281 | 0.052709537 | K10712 |
| *mgl* | 473.6154173 | 1737.316031 | 3.668199909 | 1.875072265 | 0.015477654 | 0.052964041 | K01739 |
| *LAC3* | 0 | 5.34658135 | Inf | Inf | 0.015622701 | 0.053397992 | K05909 |
| *ABCG39* | 0 | 5.344959719 | Inf | Inf | 0.015632623 | 0.053424976 | - |
| *GIP2* | 0 | 7.250678536 | Inf | Inf | 0.015640989 | 0.053446636 | - |
| *GLCAT14A* | 108.7500423 | 293.2399499 | 2.696458262 | 1.431065703 | 0.015659055 | 0.053501434 | K00771 |
| *uncharacterized protein_05081* | 0 | 5.332086136 | Inf | Inf | 0.015711702 | 0.053618753 | - |
| *LOC_Os07g01090* | 30.86345711 | 154.8019322 | 5.015702928 | 2.326451902 | 0.015738712 | 0.05369007 | - |
| *ANR* | 4.231591929 | 221.0865179 | 52.24665364 | 5.707266732 | 0.015817116 | 0.053929612 | K13082 |
| *ALDH2C4* | 57.02857952 | 119.6558887 | 2.098174104 | 1.069134396 | 0.015839946 | 0.053986502 | K12355 |
| *CRK7* | 24.52393298 | 50.87386814 | 2.074457967 | 1.052734425 | 0.015848253 | 0.054007831 | - |
| *ZIFL1* | 5.68697644 | 19.06988213 | 3.353254992 | 1.745562194 | 0.015942993 | 0.054295585 | - |
| *GSTU20* | 0 | 5.288600494 | Inf | Inf | 0.015983007 | 0.054417792 | K00799 |
| *XTH* | 17.89472092 | 61.37838768 | 3.429971775 | 1.778196705 | 0.015990817 | 0.05443735 | K08235 |
| *CARA* | 2.960492005 | 13.77803838 | 4.653969121 | 2.218461639 | 0.015993898 | 0.054440809 | K01956 |
| *uncharacterized protein_02349* | 7.610376093 | 22.67028505 | 2.978865272 | 1.574762875 | 0.016044134 | 0.054562476 | - |
| *PLT4* | 28.77962623 | 58.14249053 | 2.020265658 | 1.014545015 | 0.016048109 | 0.054568955 | - |
| *At4g33300* | 67.74390336 | 181.8611326 | 2.684538735 | 1.424674222 | 0.016219088 | 0.055114785 | - |
| *CYP76C1* | 16.05922878 | 37.75316535 | 2.350870385 | 1.233194998 | 0.016226255 | 0.055132034 | - |
| *CRPK1* | 39.95800403 | 136.4640523 | 3.41518691 | 1.771964538 | 0.01642908 | 0.055759161 | - |
| *CHI4* | 5.515626663 | 76.59569021 | 13.88703313 | 3.795666505 | 0.016714467 | 0.056575922 | K01183 |
| *uncharacterized protein_37760* | 12.23159784 | 31.8604447 | 2.604765552 | 1.381153525 | 0.016715613 | 0.056575922 | - |
| *DDB_G0268948* | 15.10229005 | 62.98275776 | 4.17041108 | 2.060189598 | 0.01682653 | 0.056913369 | - |
| *At3g17430* | 138.0768128 | 522.1042348 | 3.781259315 | 1.918866791 | 0.016852096 | 0.056972018 | - |
| *GOLS2* | 1.296721585 | 18.61233575 | 14.35337852 | 3.843318456 | 0.016916637 | 0.057175543 | K18819 |
| *YML018C* | 266.6436075 | 554.87518 | 2.080961869 | 1.05725053 | 0.016920915 | 0.057180147 | K15289 |
| *NPC1* | 32.89415238 | 494.7104657 | 15.03946537 | 3.910681377 | 0.016941427 | 0.057229974 | K01114 |
| *SUC3* | 211.8536808 | 554.8230087 | 2.618897187 | 1.388959423 | 0.016953112 | 0.05725477 | K15378 |
| *uncharacterized protein_04087* | 13.15162442 | 32.69810347 | 2.486240668 | 1.313965956 | 0.017049773 | 0.057559089 | K13348 |
| *NRP* | 6.642270851 | 20.79183556 | 3.130230012 | 1.646268671 | 0.017066759 | 0.057603523 | - |
| *ENO1* | 0.301350363 | 6.648180009 | 22.06129752 | 4.463445739 | 0.017086867 | 0.057649701 | K01689 |
| *uncharacterized protein_05241* | 1.846534669 | 11.38911904 | 6.167833852 | 2.624763903 | 0.017112476 | 0.057704248 | - |
| *CAMRLK* | 2.922059513 | 19.20672612 | 6.573009903 | 2.716554159 | 0.017139545 | 0.057765964 | - |
| *uncharacterized protein_49676* | 5.973871653 | 44.70270697 | 7.48303773 | 2.903624049 | 0.017160879 | 0.057824065 | - |
| *PROT2* | 1.101146506 | 8.795552041 | 7.987631067 | 2.997767699 | 0.01716428 | 0.057824065 | - |
| *JOX1* | 55.39551592 | 146.3516905 | 2.641941104 | 1.401598305 | 0.017165559 | 0.057824065 | - |
| *At5g27430* | 13.313452 | 32.12459538 | 2.412942592 | 1.270793592 | 0.017249264 | 0.058061507 | K12948 |
| *SRG1* | 17.79350665 | 45.47244328 | 2.555563902 | 1.353641666 | 0.017334295 | 0.058295607 | - |
| *uncharacterized protein_25301* | 7.376368521 | 147.8539385 | 20.0442722 | 4.32511813 | 0.017369984 | 0.058393278 | - |
| *Os12g0628600* | 0.681210029 | 8.682833591 | 12.74619166 | 3.671994355 | 0.017607151 | 0.059100111 | - |
| *uncharacterized protein_41526* | 36.69616166 | 78.77975007 | 2.146811724 | 1.102195672 | 0.017758178 | 0.059531231 | - |
| *GATA5* | 82.89628833 | 290.9908259 | 3.510299819 | 1.811594258 | 0.017762864 | 0.059539368 | - |
| *uncharacterized protein_35705* | 0 | 7.129858368 | Inf | Inf | 0.017816807 | 0.059704994 | - |
| *At1g73050* | 0 | 12.24938763 | Inf | Inf | 0.017855386 | 0.059803857 | - |
| *JOX2* | 4.03601685 | 24.49704127 | 6.069608275 | 2.60160341 | 0.017955877 | 0.060071731 | K05278 |
| *uncharacterized protein_39199* | 159.7330943 | 377.6622679 | 2.364333262 | 1.241433403 | 0.018019888 | 0.060240001 | - |
| *ABCC14* | 0.982560392 | 8.677869227 | 8.831894 | 3.142722857 | 0.01805846 | 0.060315395 | - |
| *OBL1* | 0 | 25.94208279 | Inf | Inf | 0.018089736 | 0.060404545 | - |
| *RLK7* | 8.645948106 | 24.73697339 | 2.861105929 | 1.516572913 | 0.018167967 | 0.060619692 | - |
| *CHI4* | 1.035448033 | 8.594141205 | 8.29992518 | 3.053098331 | 0.018179855 | 0.060646022 | K01183 |
| *uncharacterized protein_12319* | 16.70352666 | 37.97393629 | 2.273408309 | 1.184856819 | 0.018208427 | 0.060723941 | - |
| *uncharacterized protein_26302* | 6.536619547 | 47.97743669 | 7.33979335 | 2.875739445 | 0.018362483 | 0.061160319 | - |
| *CSF7* | 207.8734922 | 4497.594272 | 21.63620876 | 4.435375818 | 0.018397906 | 0.061240459 | K00660 |
| *uncharacterized protein_42383* | 0 | 6.089266593 | Inf | Inf | 0.018453826 | 0.061371509 | - |
| *At3g21360* | 0 | 21.48960603 | Inf | Inf | 0.018453832 | 0.061371509 | - |
| *RUN1* | 11.69928058 | 29.24113054 | 2.49939561 | 1.321579273 | 0.018498719 | 0.06148975 | - |
| *Os02g0512300* | 19.42684643 | 42.35858362 | 2.180414807 | 1.124602623 | 0.01850187 | 0.061492471 | - |
| *RZPF34* | 2.894793533 | 14.22585497 | 4.914290022 | 2.296983002 | 0.018626332 | 0.061842138 | K10144 |
| *NAC018* | 0 | 7.476214906 | Inf | Inf | 0.018640379 | 0.061874791 | - |
| *uncharacterized protein_26643* | 0 | 5.610732028 | Inf | Inf | 0.01864636 | 0.061886848 | - |
| *PAP4* | 24.76619036 | 122.2536329 | 4.936311603 | 2.303433467 | 0.018678006 | 0.061980507 | K14379 |
| *uncharacterized protein_42058* | 18.67816963 | 40.95248058 | 2.192531784 | 1.132597756 | 0.018776061 | 0.062246775 | - |
| *RHM1* | 451.0983501 | 1726.201962 | 3.826664322 | 1.936087352 | 0.019017614 | 0.062928844 | K12450 |
| *Mfsd5* | 19.18167235 | 41.85765106 | 2.182169015 | 1.125762847 | 0.019040191 | 0.062975367 | - |
| *GDPD2* | 5.988326802 | 21.92760625 | 3.661725048 | 1.872523466 | 0.019044865 | 0.062975367 | - |
| *PDC4* | 2.881982702 | 13.37856588 | 4.642139548 | 2.214789892 | 0.019072511 | 0.063054813 | K01568 |
| *RAD50* | 6.917999552 | 20.97073606 | 3.03132949 | 1.599950674 | 0.019074823 | 0.063054813 | K10866 |
| *ATXR5* | 19.06308624 | 41.80301294 | 2.192877503 | 1.132825223 | 0.019127336 | 0.063189218 | - |
| *PAE8* | 401.1151064 | 803.8425011 | 2.004019515 | 1.002896558 | 0.019127469 | 0.063189218 | K19882 |
| *At1g75140* | 10.20394324 | 27.3934856 | 2.684597998 | 1.42470607 | 0.019137493 | 0.063206486 | - |
| *TOC34* | 17.76155567 | 62.20054574 | 3.501976228 | 1.808169291 | 0.01925622 | 0.063558791 | - |
| *ROQ1* | 22.49311372 | 46.98032411 | 2.088653652 | 1.062573279 | 0.019391579 | 0.063965513 | - |
| *At1g73050* | 0 | 9.520498807 | Inf | Inf | 0.019408742 | 0.064014116 | - |
| *EFR* | 0.615511556 | 36.40829287 | 59.15127425 | 5.886337343 | 0.019444966 | 0.064125567 | K13420 |
| *IPT2* | 23.51891558 | 47.57146478 | 2.02268955 | 1.016274907 | 0.019449207 | 0.064131528 | K00791 |
| *At2g21480* | 49.00372405 | 103.1975303 | 2.105911996 | 1.074445149 | 0.019511904 | 0.064308363 | - |
| *PME51* | 60.9203886 | 171.3234327 | 2.812251146 | 1.491725439 | 0.01987526 | 0.065372814 | - |
| *WRKY35* | 50.39303815 | 145.3431334 | 2.884190729 | 1.528166572 | 0.019935662 | 0.065546943 | - |
| *HAK5* | 0 | 5.07918741 | Inf | Inf | 0.020149633 | 0.066143196 | K03549 |
| *MYB1* | 78.57793729 | 180.8788201 | 2.301903389 | 1.202827285 | 0.020193307 | 0.066259627 | K09422 |
| *CDKB2-2* | 0 | 5.064692195 | Inf | Inf | 0.020263122 | 0.0664155 | K07760 |
| *EXO84B* | 18.93472997 | 40.44323448 | 2.13592877 | 1.094863536 | 0.020265346 | 0.0664155 | K19986 |
| *ALA7* | 22.41308408 | 47.35891434 | 2.11300302 | 1.079294829 | 0.02027349 | 0.066417414 | K01530 |
| *NFD4* | 19.03417594 | 51.25265086 | 2.692664554 | 1.429034514 | 0.020401633 | 0.066787409 | - |
| *ISY1* | 0 | 5.045332087 | Inf | Inf | 0.020416129 | 0.06681119 | - |
| *MKK9* | 1.362420058 | 9.352943294 | 6.864948326 | 2.779248861 | 0.02043357 | 0.066848337 | K20604 |
| *CAD* | 0 | 5.035701767 | Inf | Inf | 0.020492855 | 0.06702776 | K00083 |
| *GLR3.4* | 0.301350363 | 7.258687224 | 24.08720254 | 4.590194945 | 0.020501996 | 0.067049336 | K05387 |
| *GGPS1* | 0 | 5.034080136 | Inf | Inf | 0.020505815 | 0.067053508 | K13789 |
| *LOC_Os07g01090* | 45.4865655 | 94.07162626 | 2.06811891 | 1.048319138 | 0.02053692 | 0.067138558 | - |
| *EOBII* | 0 | 5.02759361 | Inf | Inf | 0.020557773 | 0.067190064 | K09422 |
| *CSLD4* | 98.02584436 | 247.2510649 | 2.522304873 | 1.334742666 | 0.020561714 | 0.06719461 | - |
| *PAD4* | 76.65428967 | 775.4856817 | 10.11666385 | 3.33866171 | 0.020585625 | 0.067264411 | - |
| *GLR2.7* | 4.138627476 | 23.30168098 | 5.630291954 | 2.493209734 | 0.020635233 | 0.067384826 | K05387 |
| *ASR1* | 0 | 5.01796329 | Inf | Inf | 0.020635261 | 0.067384826 | - |
| *At1g78530* | 1.218212282 | 9.422176102 | 7.734428755 | 2.951294742 | 0.020725604 | 0.067654695 | - |
| *UCC1* | 0 | 4.99698155 | Inf | Inf | 0.020805534 | 0.067856781 | - |
| *UCC1* | 0 | 4.99698155 | Inf | Inf | 0.020805534 | 0.067856781 | - |
| *KPYC* | 2024.828092 | 5640.851302 | 2.785842079 | 1.478113478 | 0.020808668 | 0.067858607 | K00873 |
| *MO1* | 59.16048906 | 128.5775237 | 2.173368168 | 1.119932587 | 0.020823827 | 0.06789124 | - |
| *BIP* | 0.367048835 | 6.393659651 | 17.41909805 | 4.122598019 | 0.020872037 | 0.068014763 | K09490 |
| *TPS-mISO1* | 0 | 5.699324945 | Inf | Inf | 0.020891028 | 0.06805982 | K04120 |
| *At2g23790* | 0 | 4.985729599 | Inf | Inf | 0.020897674 | 0.068073055 | - |
| *CYP750A1* | 1.205401451 | 27.51918355 | 22.82989084 | 4.512852056 | 0.020908817 | 0.068100938 | - |
| *ECA3* | 24.73408719 | 50.66607669 | 2.048431232 | 1.034519461 | 0.020997188 | 0.068354976 | K05853 |
| *NPF5.10* | 46.94195001 | 125.8737853 | 2.681477553 | 1.423028176 | 0.021045088 | 0.068482025 | K14638 |
| *CYP76B6* | 0 | 5.715541259 | Inf | Inf | 0.021053748 | 0.0684968 | - |
| *EIX1* | 0.367048835 | 6.35179564 | 17.30504235 | 4.113120567 | 0.021132843 | 0.068737159 | - |
| *DHNAT1* | 7.033421009 | 21.2897238 | 3.026937214 | 1.597858751 | 0.021198475 | 0.068923071 | - |
| *MBD9* | 18.57580697 | 40.95400274 | 2.204695754 | 1.140579579 | 0.021418814 | 0.069487231 | - |
| *GH3.6* | 0.367048835 | 6.274454675 | 17.09433206 | 4.095446147 | 0.021626817 | 0.070067167 | K14487 |
| *Os03g0120900* | 0.301350363 | 6.337300426 | 21.02967578 | 4.394354703 | 0.021645346 | 0.070109959 | - |
| *RPS7* | 21.47212084 | 44.72347689 | 2.082862575 | 1.058567655 | 0.021679715 | 0.070212654 | K02993 |
| *PLT4* | 0 | 7.123471311 | Inf | Inf | 0.021816405 | 0.070577288 | - |
| *GOLS4* | 2.527744698 | 12.38622517 | 4.900109249 | 2.292813915 | 0.021946055 | 0.070909677 | K18819 |
| *At3g27390* | 2.567821509 | 12.34273953 | 4.806696839 | 2.265045815 | 0.021955863 | 0.070932671 | - |
| *AVT6E* | 2.567821509 | 12.3411179 | 4.806065318 | 2.264856256 | 0.021960531 | 0.070939056 | - |
| *MYB2* | 0 | 6.045780951 | Inf | Inf | 0.02206954 | 0.071222479 | K09422 |
| *PLP6* | 2.881982702 | 13.51064444 | 4.687968608 | 2.228962909 | 0.022253277 | 0.071700167 | - |
| *uncharacterized protein_09482* | 4.27166874 | 15.6515235 | 3.664030255 | 1.873431416 | 0.022292516 | 0.071817817 | - |
| *hsp70* | 3.970318377 | 15.01526912 | 3.781880367 | 1.919103727 | 0.022337276 | 0.071944426 | K03283 |
| *CRK8* | 0.92967275 | 8.357359324 | 8.989571144 | 3.168252292 | 0.022366888 | 0.0720222 | - |
| *CYP76T24* | 1.545184306 | 12.98395827 | 8.402854094 | 3.070879434 | 0.022407127 | 0.072134146 | - |
| *ZAT9* | 1.048258864 | 8.790687147 | 8.38598885 | 3.067980912 | 0.022459875 | 0.072268646 | - |
| *uncharacterized protein_46330* | 234.5217374 | 824.6476231 | 3.516295045 | 1.814056129 | 0.023042509 | 0.073845863 | - |
| *OTU7* | 11.38359905 | 28.27453055 | 2.483795364 | 1.312546317 | 0.023067667 | 0.073908512 | K13717 |
| *XPR1* | 24.18415012 | 48.63810857 | 2.011156411 | 1.008025287 | 0.023102154 | 0.073992026 | - |
| *SDR2a* | 16.96175953 | 38.53923677 | 2.272124935 | 1.184042165 | 0.023157499 | 0.074142256 | - |
| *HHP4* | 280.991331 | 1278.209285 | 4.548927828 | 2.185526545 | 0.023249612 | 0.074368664 | K07297 |
| *DHS* | 40.11514662 | 82.68108452 | 2.061093913 | 1.043410242 | 0.023470549 | 0.074926089 | K00809 |
| *RR41* | 0 | 9.16279085 | Inf | Inf | 0.0235279 | 0.075090983 | - |
| *PDC4* | 4.638717575 | 35.11956135 | 7.570963479 | 2.920476909 | 0.023730903 | 0.075619839 | K01568 |
| *MSH7* | 0 | 8.344386273 | Inf | Inf | 0.024004275 | 0.076310211 | K08737 |
| *CCR1* | 0 | 42.42823362 | Inf | Inf | 0.024005462 | 0.076310211 | K09753 |
| *TTL* | 15.91806168 | 35.02103327 | 2.200081515 | 1.137556978 | 0.024056603 | 0.07644512 | - |
| *GAUT1* | 11.64651692 | 28.84804509 | 2.476967602 | 1.308574999 | 0.02428849 | 0.07709833 | K13648 |
| *ROQ1* | 3.930241566 | 28.79817883 | 7.327330483 | 2.873287687 | 0.024310832 | 0.077150668 | - |
| *CSF7* | 56.04474675 | 719.0627664 | 12.83015462 | 3.681466652 | 0.024360397 | 0.077298655 | K00660 |
| *GOLS4* | 8.136211834 | 23.5029859 | 2.888689034 | 1.530414907 | 0.024593901 | 0.077962864 | K18819 |
| *ROQ1* | 4.717226879 | 16.10096817 | 3.413227429 | 1.771136547 | 0.024723034 | 0.078307929 | - |
| *GLOX* | 22.08155104 | 130.1899476 | 5.895869694 | 2.559704639 | 0.024726654 | 0.078309983 | - |
| *R53* | 2.35779128 | 11.33103871 | 4.805785317 | 2.264772202 | 0.024807632 | 0.078503574 | K00430 |
| *LAC3* | 0 | 8.165684717 | Inf | Inf | 0.024869245 | 0.078648167 | K05909 |
| *ACO* | 0 | 6.459748665 | Inf | Inf | 0.024955559 | 0.078873812 | K05933 |
| *VIT_16s0100g00290* | 8.686024917 | 23.76723605 | 2.736261555 | 1.452206142 | 0.02498253 | 0.078940123 | K00008 |
| *ACO* | 0 | 6.456505402 | Inf | Inf | 0.025027223 | 0.079052912 | K05933 |
| *SEC* | 8.73726824 | 23.65928303 | 2.707858152 | 1.437152167 | 0.025057387 | 0.079119743 | K09667 |
| *PMGI* | 3.119154931 | 12.77768898 | 4.096522701 | 2.034399809 | 0.0251439 | 0.079316893 | K15633 |
| *HIPP39* | 21.83752535 | 56.61678463 | 2.592637385 | 1.37442044 | 0.025254275 | 0.07962695 | - |
| *uncharacterized protein_25343* | 0 | 5.093682624 | Inf | Inf | 0.025325025 | 0.079811831 | K13464 |
| *At2g16250* | 3.588814392 | 22.68478671 | 6.320969609 | 2.660145878 | 0.025664061 | 0.080677719 | - |
| *NPC1* | 1.912233141 | 34.66678039 | 18.12895072 | 4.180223521 | 0.025780197 | 0.081023476 | K01114 |
| *Os12g0628600* | 9.41872623 | 28.54679583 | 3.030855249 | 1.599724952 | 0.025817003 | 0.081110137 | - |
| *CYP71AU50* | 1.101146506 | 8.120577443 | 7.374656687 | 2.882575889 | 0.025943599 | 0.081458229 | - |
| *ALA10* | 17.7229992 | 42.30090761 | 2.386780427 | 1.255065851 | 0.025946344 | 0.081458229 | K14802 |
| *ACO3* | 2.474857056 | 12.03185994 | 4.861638339 | 2.281442574 | 0.026023117 | 0.081679799 | K01681 |
| *arl5* | 3.524760239 | 14.07442275 | 3.993015636 | 1.997478721 | 0.02608881 | 0.081847004 | K10532 |
| *At5g57200* | 12.7348526 | 30.09166289 | 2.362937668 | 1.240581573 | 0.026129581 | 0.081955401 | K20043 |
| *CXXS1* | 0.681210029 | 15.54529158 | 22.82011556 | 4.512234192 | 0.026181689 | 0.082079766 | K03671 |
| *COMT1* | 0 | 5.025971978 | Inf | Inf | 0.02635637 | 0.082558652 | K13066 |
| *HSP17.8* | 392.54029 | 1143.518338 | 2.913123486 | 1.542566858 | 0.026430511 | 0.08275155 | K13993 |
| *UBC16* | 3.170522234 | 13.32697208 | 4.203399659 | 2.071556634 | 0.026447808 | 0.082786036 | K10688 |
| *COMT1* | 0 | 53.78512541 | Inf | Inf | 0.026586122 | 0.083189341 | - |
| *GLR3.4* | 0.301350363 | 9.62682376 | 31.94561862 | 4.997546172 | 0.026744826 | 0.083646212 | K05387 |
| *MYB2* | 0 | 4.736074135 | Inf | Inf | 0.026776713 | 0.083716139 | K09422 |
| *GOLS2* | 0 | 4.736074135 | Inf | Inf | 0.026776713 | 0.083716139 | K18819 |
| *CYP94B1* | 0 | 4.734452504 | Inf | Inf | 0.026793527 | 0.083748836 | - |
| *uncharacterized protein_31393* | 0 | 4.732830872 | Inf | Inf | 0.026810355 | 0.083781566 | - |
| *ABCC10* | 1.703847232 | 22.0756893 | 12.9563783 | 3.695590593 | 0.026822013 | 0.083798125 | - |
| *MYB2* | 0 | 7.912680082 | Inf | Inf | 0.026895502 | 0.08398518 | K09422 |
| *AVT6E* | 0.367048835 | 6.021555948 | 16.4053264 | 4.036092393 | 0.026913501 | 0.084021474 | - |
| *CRK15* | 2.567821509 | 14.21298139 | 5.535034792 | 2.468592386 | 0.026927127 | 0.084038192 | - |
| *At1g73050* | 0 | 4.721578921 | Inf | Inf | 0.026927551 | 0.084038192 | - |
| *R12* | 0 | 4.71995729 | Inf | Inf | 0.026944503 | 0.08408114 | K00430 |
| *LOX1.6* | 0 | 13.97782758 | Inf | Inf | 0.026997732 | 0.084197399 | K15718 |
| *PR4B* | 0.301350363 | 6.073149748 | 20.15311909 | 4.332931236 | 0.027025294 | 0.084253449 | - |
| *At3g47110* | 15.88459037 | 88.29204769 | 5.558345897 | 2.474655616 | 0.027080701 | 0.084356334 | - |
| *XLT2* | 314.8132638 | 892.136836 | 2.833860382 | 1.502768682 | 0.027088385 | 0.084370299 | - |
| *At4g33300* | 18.02599388 | 38.58099487 | 2.14029779 | 1.09781154 | 0.027095476 | 0.084372445 | - |
| *PHYPADRAFT_128349* | 1.598071948 | 9.721803734 | 6.083458099 | 2.604891647 | 0.027107475 | 0.08438362 | K08967 |
| *chl1* | 0 | 4.700597181 | Inf | Inf | 0.027148091 | 0.084466426 | K11273 |
| *NMT3* | 544.1554404 | 1124.725337 | 2.066919217 | 1.047482004 | 0.027246294 | 0.084731957 | K05929 |
| *uncharacterized protein_24415* | 0.301350363 | 11.87728392 | 39.41353783 | 5.300619349 | 0.027255373 | 0.08475019 | - |
| *At1g73050* | 0 | 4.687723598 | Inf | Inf | 0.027284712 | 0.08483141 | - |
| *PGLR* | 0 | 4.674850016 | Inf | Inf | 0.027422336 | 0.085190696 | K01213 |
| *MCA0413* | 97.51713251 | 196.9537907 | 2.019683984 | 1.014129575 | 0.027550361 | 0.085516112 | K10808 |
| *At5g41620* | 17.89611728 | 42.77590051 | 2.390233582 | 1.25715161 | 0.027631855 | 0.085738776 | - |
| *GLCAT14A* | 49.65996489 | 120.3071679 | 2.422618867 | 1.276567454 | 0.027743358 | 0.085983532 | - |
| *CRK8* | 0 | 5.354590039 | Inf | Inf | 0.027908545 | 0.086464984 | - |
| *RR23* | 0 | 4.623256216 | Inf | Inf | 0.027984168 | 0.086668712 | K14491 |
| *DOF3.2* | 19.15276205 | 40.04214035 | 2.090671844 | 1.063966631 | 0.028001892 | 0.086693043 | - |
| *DHAPS-1* | 718.5689916 | 2854.565891 | 3.972570378 | 1.990072779 | 0.028033162 | 0.086769468 | K01626 |
| *CYP750A1* | 0 | 4.610382633 | Inf | Inf | 0.028126967 | 0.087018939 | - |
| *DMP3* | 0 | 5.370806353 | Inf | Inf | 0.028164158 | 0.087103328 | - |
| *uncharacterized protein_17289* | 0 | 4.729587609 | Inf | Inf | 0.028353633 | 0.087576276 | - |
| *BAM3* | 0 | 4.729587609 | Inf | Inf | 0.028353633 | 0.087576276 | K01177 |
| *RMA3* | 7.285048387 | 33.04628058 | 4.536178598 | 2.181477443 | 0.028377461 | 0.087639602 | K10666 |
| *STLP5* | 6.235269185 | 19.37265356 | 3.106947429 | 1.63549783 | 0.028549602 | 0.088099964 | - |
| *LECRKS5* | 85.8197442 | 247.943058 | 2.889114391 | 1.530627327 | 0.028646927 | 0.088337242 | - |
| *At4g33300* | 8.644303787 | 26.97455997 | 3.120501157 | 1.641777746 | 0.028688603 | 0.088455408 | - |
| *BAM3* | 4.337367213 | 15.15859963 | 3.494885005 | 1.805244986 | 0.028764207 | 0.088647034 | K01177 |
| *UGT75L6* | 0.668399198 | 6.933312427 | 10.37301129 | 3.374762865 | 0.028855982 | 0.088888297 | K13691 |
| *LAX4* | 0.628322387 | 6.946186009 | 11.05513054 | 3.466644156 | 0.029048592 | 0.089439799 | K13946 |
| *uncharacterized protein_49594* | 110.6115562 | 447.3080142 | 4.043953719 | 2.015766487 | 0.029091049 | 0.089549602 | - |
| *YUC9* | 4.24440276 | 15.09899714 | 3.557390285 | 1.830819263 | 0.029162625 | 0.089738488 | K11816 |
| *YPTC1* | 122.2636586 | 248.1647414 | 2.029750657 | 1.021302512 | 0.029174988 | 0.089763548 | K07874 |
| *GPXMC1* | 668.5143725 | 2370.421654 | 3.545805073 | 1.826113228 | 0.029343742 | 0.090212721 | K00432 |
| *TAD1* | 35.05649256 | 70.16583156 | 2.001507465 | 1.001086996 | 0.029395737 | 0.090353791 | K15440 |
| *RING1B* | 20.8564853 | 41.8786328 | 2.007942958 | 1.005718286 | 0.029712814 | 0.091195645 | K10695 |
| *ABCA2* | 8.972920131 | 25.24297622 | 2.813239821 | 1.492232543 | 0.029750338 | 0.091280269 | - |
| *AO1* | 0.615511556 | 7.315046449 | 11.88449896 | 3.571009177 | 0.029767564 | 0.091280269 | K09842 |
| *AATP1* | 5.921107991 | 27.04389224 | 4.567370209 | 2.191363732 | 0.02982033 | 0.091420799 | K08900 |
| *PTM* | 2.633519981 | 11.71611547 | 4.448842443 | 2.153430006 | 0.029855743 | 0.09151872 | K11971 |
| *PAP4* | 107.2412698 | 663.6603651 | 6.188479175 | 2.629584909 | 0.029939072 | 0.091720815 | K14379 |
| *OFUT19* | 38.85204855 | 87.91020776 | 2.2626917 | 1.178040025 | 0.030179006 | 0.092380706 | - |
| *PAD4* | 82.90720688 | 240.3420491 | 2.898928309 | 1.535519656 | 0.030285269 | 0.092630678 | - |
| *uncharacterized protein_38987* | 31.23215026 | 229.3665169 | 7.343923329 | 2.876550997 | 0.030320412 | 0.092716648 | - |
| *RIBA1* | 44.30653775 | 177.5395364 | 4.007073119 | 2.002548836 | 0.030431008 | 0.093000887 | K14652 |
| *XYL1* | 7.783618148 | 21.50076495 | 2.762309834 | 1.465875148 | 0.030673831 | 0.093623564 | K15925 |
| *ADH2* | 1.243833943 | 8.655365325 | 6.958618047 | 2.798800821 | 0.030826346 | 0.094001988 | K18857 |
| *EDA2* | 7.111930312 | 22.891056 | 3.218683957 | 1.686470925 | 0.030918437 | 0.094206512 | - |
| *At3g25290* | 133.6828692 | 324.0399519 | 2.423945221 | 1.277357095 | 0.030972271 | 0.094348727 | - |
| *ZIP1* | 0.916861919 | 10.90734121 | 11.89638372 | 3.572451183 | 0.031125986 | 0.094737534 | K14709 |
| *NSF* | 3.013503627 | 12.99673239 | 4.312831173 | 2.108635243 | 0.03122156 | 0.094965429 | K06027 |
| *CYP76T24* | 6.903668383 | 21.03043801 | 3.046270019 | 1.607043827 | 0.031241273 | 0.095014424 | - |
| *LDOX* | 4.48005465 | 105.4327946 | 23.53381887 | 4.556663543 | 0.031341269 | 0.095241611 | K05277 |
| *ADH* | 0.602700725 | 16.49871313 | 27.37463625 | 4.77476789 | 0.031588584 | 0.095904703 | K18857 |
| *CYP78A4* | 88.31172515 | 356.5290904 | 4.037165957 | 2.013342893 | 0.031602743 | 0.095925589 | - |
| *APF1* | 11.01655022 | 54.74968657 | 4.969766895 | 2.313178184 | 0.031873466 | 0.096625078 | - |
| *HIPP28* | 1.101146506 | 7.775842538 | 7.061587623 | 2.819992574 | 0.031877184 | 0.096625078 | - |
| *QRT3* | 1.101146506 | 7.754860798 | 7.042533174 | 2.816094454 | 0.032011912 | 0.096964305 | - |
| *DPAGT1* | 13.88572209 | 31.32088495 | 2.255618019 | 1.173522773 | 0.03217584 | 0.097395939 | K01001 |
| *ARID2* | 6.445175433 | 24.32787701 | 3.774587249 | 1.916318894 | 0.032283079 | 0.097686893 | - |
| *NUDT1* | 16.59775137 | 35.38665045 | 2.132014732 | 1.092217407 | 0.032522584 | 0.098279169 | K03574 |
| *BZIP53* | 13.7285795 | 46.53868276 | 3.389912463 | 1.76124802 | 0.032523559 | 0.098279169 | - |
| *SAG20* | 1.048258864 | 7.724248738 | 7.36864624 | 2.881399593 | 0.032556263 | 0.09834417 | - |
| *PILS6* | 5.43699338 | 17.25751522 | 3.174091637 | 1.66634378 | 0.03272198 | 0.098799462 | - |
| *CYP704C1* | 4.651528406 | 15.3244276 | 3.294492964 | 1.720056446 | 0.032831241 | 0.099072612 | - |
| *uncharacterized protein_06768* | 2.043630086 | 12.24127303 | 5.989965165 | 2.582547613 | 0.032847289 | 0.09910969 | - |
| *nep2* | 2033.291405 | 4.99698155 | 0.002457583 | -8.668544386 | 7.2581E-131 | 9.5648E-127 | - |
| *UGT74AC1* | 870.3892197 | 1.973329993 | 0.002267181 | -8.784884645 | 9.0379E-101 | 2.16549E-97 | K13691 |
| *At4g32940* | 3227.857993 | 56.88731593 | 0.01762386 | -5.826326287 | 2.38246E-99 | 5.23267E-96 | K01369 |
| *DHAPS-1* | 12656.08279 | 305.7294429 | 0.02415672 | -5.371431637 | 1.02277E-97 | 1.92543E-94 | K01626 |
| *nep2* | 787.038895 | 5.957088566 | 0.007568989 | -7.045683716 | 1.96415E-86 | 3.23544E-83 | - |
| *XTH8* | 1546.451822 | 8.68111249 | 0.005613568 | -7.476866236 | 4.40746E-85 | 6.83312E-82 | K08235 |
| *SPAC24B11.05* | 521.0155166 | 0.330239692 | 0.000633838 | -10.62359709 | 1.33196E-84 | 1.95029E-81 | K07025 |
| *CHI4* | 818.5633694 | 3.00591308 | 0.003672181 | -8.089147005 | 2.70101E-84 | 3.74673E-81 | K01183 |
| *GAT1* | 618.3760982 | 2.648304591 | 0.004282676 | -7.867271685 | 1.15059E-83 | 1.44404E-80 | - |
| *BAM3* | 7272.184502 | 245.2589523 | 0.033725623 | -4.8900111 | 1.69632E-83 | 2.03219E-80 | K01177 |
| *GAPB* | 25187.14952 | 908.6150529 | 0.036074549 | -4.792874842 | 1.89188E-83 | 2.16792E-80 | K05298 |
| *RBCS* | 5289.611191 | 171.836934 | 0.03248574 | -4.94404962 | 8.28428E-83 | 9.09752E-80 | K01602 |
| *SBT5.6* | 734.939193 | 6.364669223 | 0.00866013 | -6.851395542 | 1.42948E-82 | 1.50701E-79 | - |
| *LAC6* | 551.9914785 | 1.659207147 | 0.003005856 | -8.378008171 | 6.56414E-82 | 6.65402E-79 | K05909 |
| *CRK8* | 667.9648118 | 5.657460934 | 0.008469699 | -6.883473576 | 1.14799E-79 | 1.12061E-76 | - |
| *ag4* | 471.7063727 | 0.657236121 | 0.001393316 | -9.487261606 | 3.7045E-79 | 3.48699E-76 | K18108 |
| *Os07g0190000* | 714.2785069 | 7.667889512 | 0.010735154 | -6.541513351 | 1.26002E-78 | 1.14514E-75 | K01662 |
| *ag4* | 453.525533 | 0.689469812 | 0.001520245 | -9.361480675 | 2.44782E-77 | 2.15049E-74 | K18108 |
| *NRT3.2* | 702.888635 | 8.769804876 | 0.012476806 | -6.324607574 | 2.48943E-76 | 2.1165E-73 | - |
| *ag4* | 455.6431114 | 1.018087872 | 0.002234398 | -8.805898359 | 2.99744E-76 | 2.46877E-73 | K18108 |
| *A6* | 887.1126586 | 16.30065147 | 0.018374951 | -5.766115802 | 1.48085E-75 | 1.1827E-72 | - |
| *At5g48740* | 867.422518 | 16.37150591 | 0.018873739 | -5.727475959 | 9.10322E-75 | 7.0566E-72 | - |
| *TIP4-1* | 472.8246431 | 1.987825207 | 0.004204149 | -7.893970515 | 2.01564E-74 | 1.47567E-71 | K09873 |
| *AMO* | 494.5441784 | 3.307162343 | 0.006687294 | -7.224361728 | 2.43428E-72 | 1.734E-69 | K00276 |
| *NPF6.3* | 1316.811516 | 41.57575547 | 0.03157305 | -4.985162569 | 1.42486E-70 | 9.88253E-68 | K14638 |
| *JAL3* | 1403.687293 | 47.48136258 | 0.033826168 | -4.885716427 | 2.75769E-70 | 1.86363E-67 | - |
| *PT1* | 465.3443916 | 3.357134511 | 0.007214301 | -7.114924666 | 3.80422E-70 | 2.5066E-67 | K12742 |
| *XTH32* | 414.9880176 | 1.678567256 | 0.004044857 | -7.949695527 | 4.371E-70 | 2.80981E-67 | K08235 |
| *UFO* | 426.5030136 | 2.384153913 | 0.005590005 | -7.482934751 | 3.53447E-69 | 2.21797E-66 | - |
| *CSLE1* | 652.3328645 | 11.62093656 | 0.017814428 | -5.810810065 | 5.55933E-69 | 3.40748E-66 | - |
| *CYP704C1* | 466.9957231 | 4.33011511 | 0.00927228 | -6.75286015 | 5.48051E-68 | 3.28283E-65 | - |
| *AATL1* | 1181.401279 | 39.97605778 | 0.033837832 | -4.88521906 | 1.36237E-67 | 7.97923E-65 | - |
| *RCA1* | 185379.6631 | 10583.2355 | 0.057089517 | -4.130630323 | 4.59369E-67 | 2.52232E-64 | - |
| *CRK2* | 975.7535525 | 29.54856148 | 0.030282812 | -5.045357008 | 5.36668E-67 | 2.88662E-64 | - |
| *FLZ5* | 714.9455618 | 16.53601709 | 0.023129058 | -5.434149705 | 7.71323E-67 | 4.0658E-64 | - |
| *GGPS1* | 612.1055894 | 11.40037099 | 0.018624844 | -5.746627864 | 1.27834E-66 | 6.60625E-64 | K13789 |
| *CAHC* | 10736.75725 | 622.76514 | 0.058003094 | -4.107726331 | 1.02892E-64 | 5.21502E-62 | K01673 |
| *MLP43* | 721.8677548 | 19.00379312 | 0.026325865 | -5.24737525 | 4.38317E-64 | 2.17007E-61 | - |
| *uncharacterized protein_18413* | 6845.486039 | 398.7018912 | 0.058243036 | -4.101770625 | 4.44619E-64 | 2.17007E-61 | - |
| *TAT* | 313.141176 | 0.330239692 | 0.001054603 | -9.889083981 | 5.18091E-64 | 2.48269E-61 | K15400 |
| *NPF5.7* | 300.3578446 | 0 | 0 | -Inf | 5.65247E-64 | 2.6603E-61 | K14638 |
| *PLD1* | 797.5731597 | 24.07811563 | 0.030189225 | -5.049822465 | 1.1972E-63 | 5.44023E-61 | K01115 |
| *Tmem45b* | 300.9567282 | 0 | 0 | -Inf | 2.39959E-62 | 1.05406E-59 | - |
| *At4g02290* | 422.0374423 | 5.277249073 | 0.01250422 | -6.321441111 | 8.88799E-62 | 3.77826E-59 | - |
| *BAM1* | 519.5692497 | 10.41603897 | 0.020047451 | -5.640437392 | 1.2772E-61 | 5.34317E-59 | - |
| *PM19L* | 310.9451559 | 0.989097444 | 0.003180939 | -8.296331768 | 7.25804E-61 | 2.98895E-58 | - |
| *TIP2-1* | 2610.604111 | 153.898699 | 0.058951374 | -4.084330754 | 1.01004E-59 | 4.03342E-57 | K09873 |
| *Os07g0190000* | 548.4232006 | 13.63308624 | 0.024858697 | -5.330105505 | 3.10291E-59 | 1.20265E-56 | K01662 |
| *uncharacterized protein_25495* | 3454.652119 | 213.2877006 | 0.06173927 | -4.01766776 | 3.36313E-59 | 1.28462E-56 | - |
| *ag4* | 261.0561728 | 0 | 0 | -Inf | 6.55215E-59 | 2.46698E-56 | - |
| *LDOX* | 7855.554343 | 544.0233162 | 0.069253332 | -3.851972696 | 9.07612E-59 | 3.32236E-56 | K05277 |
| *LPS* | 258.8422174 | 0 | 0 | -Inf | 1.6348E-58 | 5.90231E-56 | K04120 |
| *DNAJB4* | 555.6621865 | 15.05226824 | 0.027088884 | -5.206155257 | 3.17022E-58 | 1.12911E-55 | - |
| *LFNR1* | 29391.04452 | 2124.66056 | 0.072289386 | -3.790072355 | 6.33645E-58 | 2.19742E-55 | K02641 |
| *HSL1* | 1916.86566 | 113.2155131 | 0.059062831 | -4.081605673 | 2.12209E-57 | 7.26359E-55 | K00924 |
| *UGT73B4* | 572.0116318 | 16.9498789 | 0.029632053 | -5.076697613 | 5.52029E-57 | 1.86529E-54 | - |
| *EO* | 790.1740059 | 32.83849557 | 0.041558562 | -4.588710448 | 1.47057E-56 | 4.90612E-54 | K18980 |
| *PTAC16* | 3888.881783 | 161.9641889 | 0.04164801 | -4.585608613 | 1.55886E-56 | 5.13567E-54 | - |
| *DRP4C* | 1090.92508 | 55.20560488 | 0.050604396 | -4.30459347 | 2.73508E-56 | 8.89949E-54 | K14754 |
| *RBCS* | 3096.292622 | 135.5810873 | 0.043788202 | -4.513313972 | 6.8606E-56 | 2.2051E-53 | K01602 |
| *UGT91C1* | 332.6426626 | 3.616420295 | 0.010871787 | -6.523267046 | 7.16971E-56 | 2.27669E-53 | - |
| *LHCB5* | 32588.34755 | 2529.974227 | 0.077634321 | -3.687161606 | 1.9752E-55 | 6.19743E-53 | K08916 |
| *TDR* | 1174.590334 | 63.54979221 | 0.054103793 | -4.208126452 | 2.12796E-55 | 6.59817E-53 | - |
| *DEGP7* | 736.3261428 | 30.68757543 | 0.041676607 | -4.584618354 | 2.18533E-55 | 6.69728E-53 | - |
| *R53* | 424.5040214 | 9.314223077 | 0.021941425 | -5.510198975 | 3.38583E-55 | 1.02571E-52 | K00430 |
| *SRG1* | 527.5401932 | 16.15732096 | 0.030627659 | -5.029021107 | 7.01312E-55 | 2.07683E-52 | - |
| *uncharacterized protein_23686* | 527.1332197 | 16.56966704 | 0.031433547 | -4.991551095 | 1.41071E-54 | 4.13119E-52 | - |
| *AGPS1* | 4139.991462 | 307.8288473 | 0.074354947 | -3.749427451 | 2.37303E-54 | 6.79822E-52 | K00975 |
| *WAKL20* | 561.1642021 | 18.89574063 | 0.033672391 | -4.892290007 | 2.74369E-54 | 7.77556E-52 | - |
| *LRK10* | 228.8394177 | 0 | 0 | -Inf | 5.21395E-54 | 1.4619E-51 | - |
| *TAT* | 328.5888976 | 1.330589087 | 0.004049404 | -7.948074819 | 3.28518E-53 | 9.01918E-51 | K15400 |
| *RL6* | 233.9030047 | 0.344734906 | 0.001473837 | -9.406207294 | 4.28297E-53 | 1.15186E-50 | - |
| *PT1* | 295.0351918 | 3.748598323 | 0.012705597 | -6.298391996 | 1.32002E-51 | 3.41083E-49 | K12742 |
| *AAP3* | 2795.793856 | 214.8248896 | 0.076838601 | -3.702024931 | 1.36153E-51 | 3.48393E-49 | - |
| *RACD* | 824.0562759 | 20.13469891 | 0.024433645 | -5.35498706 | 2.91179E-51 | 7.37915E-49 | - |
| *At1g17710* | 589.8171446 | 25.03497939 | 0.042445323 | -4.558250586 | 5.09607E-51 | 1.27916E-48 | K13248 |
| *PT5* | 238.4923995 | 1.332210718 | 0.005585967 | -7.483977185 | 3.11507E-50 | 7.67296E-48 | K12742 |
| *AGT1* | 20458.70032 | 1846.348371 | 0.090247589 | -3.469967805 | 4.20028E-50 | 1.02502E-47 | K00830 |
| *uncharacterized protein_14130* | 817.0251037 | 45.84130372 | 0.056107583 | -4.155660427 | 5.00895E-50 | 1.20014E-47 | - |
| *GSVIVT00037159001* | 212.3300698 | 0.344734906 | 0.00162358 | -9.266605597 | 7.19292E-50 | 1.69265E-47 | K00430 |
| *CYP75B137* | 596.4674411 | 27.77998505 | 0.046574185 | -4.424325661 | 1.00917E-49 | 2.35377E-47 | K05280 |
| *LDOX* | 11502.27652 | 1061.394958 | 0.092276947 | -3.437885921 | 1.18251E-49 | 2.71012E-47 | K05277 |
| *algC* | 863.3313211 | 51.23471345 | 0.059345366 | -4.074720803 | 1.90346E-49 | 4.28783E-47 | - |
| *PM19L* | 462.3952902 | 16.87396063 | 0.036492501 | -4.776256158 | 3.79743E-49 | 8.41051E-47 | - |
| *PMIR1* | 3757.734719 | 326.7037121 | 0.086941665 | -3.52380847 | 9.99644E-49 | 2.19555E-46 | - |
| *RFS2* | 420.7141704 | 14.64783138 | 0.034816587 | -4.844081418 | 1.80895E-48 | 3.94023E-46 | K06617 |
| *CBP1* | 1324.368752 | 97.24205696 | 0.073425212 | -3.767580655 | 2.3847E-48 | 5.15173E-46 | K16296 |
| *FLZ10* | 565.3416948 | 15.46633542 | 0.0273575 | -5.19191979 | 2.86325E-48 | 6.13527E-46 | - |
| *RBCS* | 518.0288506 | 22.56051146 | 0.043550685 | -4.521160768 | 4.31888E-48 | 9.10627E-46 | K01602 |
| *MAPKKK17* | 248.7653298 | 2.662799805 | 0.010704063 | -6.545697654 | 4.80628E-48 | 1.00535E-45 | - |
| *H1* | 699.2466568 | 39.50583031 | 0.056497704 | -4.145663963 | 8.45527E-48 | 1.7547E-45 | K11275 |
| *CYP71AU50* | 350.6424387 | 10.11012375 | 0.028833143 | -5.116128057 | 1.81271E-47 | 3.73248E-45 | - |
| *uncharacterized protein_19765* | 220.6995453 | 1.332210718 | 0.006036309 | -7.372117553 | 2.21264E-47 | 4.52064E-45 | - |
| *AED1* | 246.5838539 | 2.661178174 | 0.010792183 | -6.53386942 | 2.39057E-47 | 4.84661E-45 | - |
| *HMA2* | 254.9297196 | 3.353891248 | 0.013156141 | -6.248119852 | 2.56001E-47 | 5.15051E-45 | K01534 |
| *LHCB4.1* | 31130.25682 | 1683.461438 | 0.054077981 | -4.208814899 | 1.40001E-46 | 2.73323E-44 | K08915 |
| *CYP76T24* | 750.5738329 | 47.4245926 | 0.063184447 | -3.984286709 | 4.83462E-46 | 9.36921E-44 | - |
| *TUBB8* | 180.6566744 | 0 | 0 | -Inf | 4.98833E-46 | 9.56997E-44 | K07375 |
| *CSLE1* | 4497.987913 | 436.1753794 | 0.096971221 | -3.366299542 | 5.01084E-46 | 9.56997E-44 | - |
| *uncharacterized protein_33452* | 197.3215206 | 0.674974598 | 0.003420684 | -8.191499387 | 7.39905E-46 | 1.40295E-43 | - |
| *VAB* | 613.9617985 | 35.50909868 | 0.057836007 | -4.111888245 | 1.15441E-45 | 2.17326E-43 | - |
| *GAPA* | 30791.82527 | 3195.809003 | 0.103787579 | -3.268294296 | 1.51779E-45 | 2.8171E-43 | K05298 |
| *BRL2* | 212.7467458 | 1.675323993 | 0.007874734 | -6.988553127 | 1.97158E-45 | 3.63378E-43 | - |
| *SBT5.6* | 205.278905 | 1.330589087 | 0.00648186 | -7.269376463 | 3.77963E-45 | 6.87007E-43 | - |
| *uncharacterized protein_47823* | 621.6957937 | 36.37888524 | 0.058515572 | -4.095035578 | 4.67063E-45 | 8.43145E-43 | - |
| *FBP* | 1037.260395 | 80.25324603 | 0.077370395 | -3.692074558 | 1.42989E-44 | 2.56369E-42 | K03841 |
| *PPD* | 2715.823081 | 262.0361038 | 0.096484968 | -3.373551992 | 1.94671E-44 | 3.46671E-42 | K01006 |
| *PCR6* | 295.5854659 | 7.961030619 | 0.026933092 | -5.214476312 | 5.35001E-44 | 9.46341E-42 | - |
| *EO* | 173.9484571 | 0.32861806 | 0.001889169 | -9.048032397 | 3.39414E-43 | 5.88527E-41 | K18980 |
| *AAE14* | 636.4227851 | 41.47895492 | 0.065175157 | -3.939534037 | 3.91269E-43 | 6.74005E-41 | K14760 |
| *AIR3* | 715.7778049 | 51.97892729 | 0.072618803 | -3.783513043 | 8.01268E-43 | 1.36247E-40 | - |
| *CHLM* | 3099.003838 | 320.935948 | 0.103561004 | -3.271447242 | 1.29041E-42 | 2.16624E-40 | K03428 |
| *S17P* | 11110.04461 | 1260.84357 | 0.113486814 | -3.13940341 | 2.93468E-42 | 4.86456E-40 | K01100 |
| *uncharacterized protein_02058* | 432.6146115 | 22.71539233 | 0.052507224 | -4.251340265 | 4.32245E-42 | 7.08557E-40 | - |
| *CYP75A5* | 1229.747405 | 112.5801668 | 0.091547391 | -3.449337415 | 4.32834E-42 | 7.08557E-40 | K13083 |
| *At2g18940* | 507.1889157 | 31.10002098 | 0.061318416 | -4.027535758 | 1.31758E-41 | 2.13044E-39 | - |
| *A6* | 281.9820215 | 8.605393157 | 0.030517524 | -5.034218266 | 2.02756E-41 | 3.25844E-39 | - |
| *BIP* | 258.5204545 | 4.657111538 | 0.01801448 | -5.794699188 | 3.43755E-41 | 5.45783E-39 | K09490 |
| *PT1* | 151.7254867 | 0 | 0 | -Inf | 6.08284E-41 | 9.59996E-39 | K12742 |
| *DGAT3* | 1871.126231 | 193.9979814 | 0.103679794 | -3.269793344 | 7.39962E-41 | 1.16086E-38 | - |
| *uncharacterized protein_04027* | 1626.436839 | 166.1704524 | 0.102168402 | -3.29097902 | 2.16072E-40 | 3.32022E-38 | - |
| *AGD11* | 198.3605053 | 2.635431008 | 0.013286067 | -6.23394207 | 2.16678E-40 | 3.32022E-38 | K12486 |
| *IQD30* | 1444.656452 | 145.9749337 | 0.101044739 | -3.306933891 | 2.30406E-40 | 3.51016E-38 | - |
| *BCP* | 157.6312717 | 0.32861806 | 0.002084726 | -8.905926286 | 2.75599E-40 | 4.17454E-38 | - |
| *At5g18840* | 250.3792815 | 7.06062556 | 0.02819972 | -5.148175367 | 3.12627E-40 | 4.70834E-38 | K08145 |
| *uncharacterized protein_03647* | 332.9530674 | 14.63820106 | 0.043964758 | -4.50750866 | 4.38692E-40 | 6.53229E-38 | - |
| *UGT85A8* | 337.8570911 | 15.65304567 | 0.046330375 | -4.431897836 | 9.81641E-40 | 1.43734E-37 | - |
| *PATL6* | 749.9771766 | 63.92371649 | 0.085234216 | -3.552423499 | 1.11946E-39 | 1.63008E-37 | - |
| *ag4* | 144.673826 | 0 | 0 | -Inf | 2.04966E-39 | 2.95196E-37 | - |
| *MYB1* | 191.0436299 | 2.64668296 | 0.013853814 | -6.173572955 | 2.4514E-39 | 3.51137E-37 | K09422 |
| *NPF4.3* | 217.5964616 | 4.666741858 | 0.021446773 | -5.54309562 | 3.17316E-39 | 4.52064E-37 | - |
| *At5g63180* | 477.3864913 | 0.657236121 | 0.001376738 | -9.504530257 | 6.84586E-39 | 9.54653E-37 | K01728 |
| *ABCB1* | 816.0519764 | 74.86683306 | 0.091742726 | -3.446262411 | 1.18401E-38 | 1.63381E-36 | K05658 |
| *PORA* | 746.736495 | 65.47761062 | 0.087685028 | -3.51152566 | 1.1982E-38 | 1.64478E-36 | K00218 |
| *EFR* | 154.3266838 | 0.689469812 | 0.004467599 | -7.806284431 | 1.42356E-38 | 1.94401E-36 | K13420 |
| *PSAF* | 19542.38881 | 2392.621279 | 0.122432385 | -3.029942869 | 1.5261E-38 | 2.0733E-36 | K02694 |
| *RBCS* | 557.1940641 | 42.03138181 | 0.075434009 | -3.728641097 | 1.58444E-38 | 2.14151E-36 | K01602 |
| *PILS7* | 406.7522076 | 24.89651377 | 0.061208061 | -4.030134533 | 2.63894E-38 | 3.54856E-36 | K07088 |
| *XTH8* | 394.5006256 | 24.07315127 | 0.061021833 | -4.034530674 | 2.91623E-38 | 3.90153E-36 | K08235 |
| *CYP71AU50* | 782.0720136 | 24.59830883 | 0.031452741 | -4.990670421 | 3.63382E-38 | 4.81271E-36 | - |
| *IP5P8* | 332.0889648 | 17.19811811 | 0.051787683 | -4.271247171 | 3.65218E-38 | 4.81284E-36 | - |
| *PT5* | 168.7942939 | 1.659207147 | 0.009829759 | -6.66862831 | 3.89226E-38 | 5.1037E-36 | K12742 |
| *At3g02645* | 227.9018671 | 6.434001499 | 0.028231456 | -5.146552661 | 4.22195E-38 | 5.5086E-36 | - |
| *TUBB2* | 2526.968118 | 305.4484469 | 0.120875465 | -3.048406652 | 5.80312E-38 | 7.53434E-36 | K07375 |
| *H1* | 8210.985161 | 1062.823285 | 0.129439192 | -2.949653588 | 6.47755E-38 | 8.33647E-36 | K11275 |
| *Prcp* | 554.1129654 | 43.65866012 | 0.07879018 | -3.665840364 | 6.4842E-38 | 8.33647E-36 | K01285 |
| *GGPS1* | 163.5169596 | 1.316093873 | 0.008048669 | -6.957034071 | 7.92876E-38 | 1.00952E-35 | K13789 |
| *BAM2* | 717.8062598 | 58.83003386 | 0.081958095 | -3.608969733 | 9.05007E-38 | 1.14675E-35 | - |
| *SB09* | 668.1169606 | 58.98815799 | 0.088290167 | -3.501603419 | 1.42075E-37 | 1.77466E-35 | K03671 |
| *TPS14* | 171.3362175 | 2.005563685 | 0.011705428 | -6.416678556 | 1.45999E-37 | 1.8125E-35 | K04120 |
| *CURT1B* | 10282.92341 | 1343.423999 | 0.130646115 | -2.936263866 | 1.4648E-37 | 1.8125E-35 | - |
| *Os07g0682400* | 857.4781126 | 83.7105367 | 0.097624109 | -3.356618714 | 1.60287E-37 | 1.97407E-35 | - |
| *MWL2* | 438.6322206 | 30.27685098 | 0.069025597 | -3.856724725 | 2.13247E-37 | 2.60201E-35 | - |
| *CCOAOMT* | 291.0583244 | 13.44293379 | 0.046186392 | -4.436388348 | 2.6133E-37 | 3.17402E-35 | K00588 |
| *uncharacterized protein_43899* | 721.9783434 | 14.9073161 | 0.020647872 | -5.597863118 | 3.7167E-37 | 4.49346E-35 | - |
| *PSBP* | 21333.87092 | 2236.018292 | 0.104810716 | -3.254141863 | 3.79641E-37 | 4.56887E-35 | K02717 |
| *TOGT1* | 3200.954873 | 412.7085079 | 0.128932935 | -2.955307253 | 7.52547E-37 | 8.97472E-35 | - |
| *At3g47110* | 670.6061814 | 61.10805532 | 0.091123609 | -3.456031306 | 1.16675E-36 | 1.38518E-34 | - |
| *ag4* | 144.3855344 | 0.689469812 | 0.0047752 | -7.710223109 | 1.25124E-36 | 1.47882E-34 | K04120 |
| *CYP71AU50* | 227.6997147 | 7.337649821 | 0.032225116 | -4.955670618 | 1.29902E-36 | 1.52844E-34 | - |
| *OPR2* | 443.0565035 | 31.97133614 | 0.072160855 | -3.792639756 | 1.42779E-36 | 1.65774E-34 | K05894 |
| *PSBW* | 13872.5509 | 1508.900686 | 0.108768798 | -3.20066334 | 2.03134E-36 | 2.3379E-34 | K02721 |
| *CB21* | 917.5271211 | 94.86378632 | 0.103390716 | -3.273821446 | 2.23691E-36 | 2.5633E-34 | K08913 |
| *chi1* | 248.7166595 | 10.13577145 | 0.040752282 | -4.616975341 | 5.21303E-36 | 5.92218E-34 | K20547 |
| *At5g10770* | 127.2045944 | 0 | 0 | -Inf | 5.83279E-36 | 6.59781E-34 | - |
| *MMAR_1059* | 1097.545788 | 125.1620169 | 0.114038082 | -3.132412411 | 2.86256E-35 | 3.19685E-33 | - |
| *CYCU4-1* | 443.830678 | 34.61659641 | 0.077995051 | -3.680473604 | 3.81447E-35 | 4.22413E-33 | - |
| *MAP65-6* | 852.5668028 | 91.75843913 | 0.107626099 | -3.215900125 | 4.04337E-35 | 4.45887E-33 | K16732 |
| *CCL7* | 964.400669 | 109.1391053 | 0.113167803 | -3.14346453 | 9.4644E-35 | 1.02231E-32 | K01904 |
| *uncharacterized protein_31758* | 776.0876236 | 81.55019161 | 0.105078588 | -3.250459376 | 1.05014E-34 | 1.1251E-32 | - |
| *DIVARICATA* | 1138.268035 | 139.4168752 | 0.122481587 | -3.029363216 | 5.33274E-34 | 5.59959E-32 | - |
| *At2g19130* | 188.2997454 | 5.299852445 | 0.028145829 | -5.150935045 | 6.1032E-34 | 6.38317E-32 | - |
| *UGT86A1* | 176.9591681 | 4.313998264 | 0.024378495 | -5.358247102 | 8.39608E-34 | 8.74652E-32 | - |
| *R53* | 339.9010367 | 23.58357013 | 0.069383637 | -3.849260724 | 9.54495E-34 | 9.9042E-32 | K00430 |
| *uncharacterized protein_34009* | 487.9799448 | 44.66863983 | 0.09153786 | -3.449487626 | 1.76734E-33 | 1.81245E-31 | - |
| *AGPS1* | 3806.555566 | 554.6038944 | 0.145697044 | -2.778956486 | 1.90876E-33 | 1.9499E-31 | K00975 |
| *CB21* | 92195.52752 | 3441.600663 | 0.037329367 | -4.743545153 | 2.49028E-33 | 2.53412E-31 | K08913 |
| *At5g10770* | 127.7525152 | 0.673352966 | 0.005270761 | -7.567773028 | 2.77503E-33 | 2.81302E-31 | - |
| *Prcp* | 183.6670134 | 5.351446245 | 0.029136676 | -5.101019894 | 3.54469E-33 | 3.57945E-31 | K01285 |
| *MYB61* | 164.5400252 | 3.627771715 | 0.022047959 | -5.503211085 | 5.14674E-33 | 5.17738E-31 | K09422 |
| *APF2* | 645.4663888 | 69.43298845 | 0.107570262 | -3.2166488 | 5.61937E-33 | 5.63134E-31 | - |
| *CB2A* | 42262.81283 | 1649.106732 | 0.039020279 | -4.67963211 | 1.10495E-32 | 1.09481E-30 | K08912 |
| *FEI1* | 191.6095424 | 6.644936746 | 0.034679571 | -4.849770135 | 1.77034E-32 | 1.74101E-30 | - |
| *IAA9* | 4344.237314 | 657.7007499 | 0.151396138 | -2.723599695 | 1.9602E-32 | 1.92056E-30 | K14484 |
| *uncharacterized protein_03475* | 1018.143005 | 17.6265746 | 0.017312474 | -5.852044261 | 2.34282E-32 | 2.27013E-30 | - |
| *CYP720B2* | 885.0613029 | 109.5658536 | 0.123794649 | -3.013979136 | 2.44376E-32 | 2.35926E-30 | - |
| *TIP1-1* | 1061.218354 | 135.5169312 | 0.127699385 | -2.969176517 | 2.47867E-32 | 2.38422E-30 | K09873 |
| *ag1* | 153.3581783 | 3.057506879 | 0.019937032 | -5.64840555 | 3.55655E-32 | 3.38398E-30 | K16086 |
| *PSBQ2* | 26851.15936 | 2172.779572 | 0.080919395 | -3.627370656 | 3.74285E-32 | 3.54844E-30 | K08901 |
| *ROPGAP1* | 1887.028405 | 271.1811518 | 0.143708039 | -2.798787325 | 5.44075E-32 | 5.13965E-30 | - |
| *At3g47110* | 361.4940925 | 30.12228139 | 0.083327175 | -3.585069125 | 6.06289E-32 | 5.70691E-30 | - |
| *ATHB-5* | 596.9297794 | 65.35282722 | 0.1094816 | -3.191239674 | 6.81731E-32 | 6.37153E-30 | K09338 |
| *CAS* | 8019.531321 | 1261.621507 | 0.157318608 | -2.668238766 | 7.06204E-32 | 6.57693E-30 | - |
| *KPPR* | 22184.16813 | 3547.256976 | 0.159900383 | -2.644754702 | 8.25216E-32 | 7.65824E-30 | K00855 |
| *PT30* | 214.605167 | 9.639597874 | 0.044917828 | -4.476568038 | 9.09112E-32 | 8.40721E-30 | K12742 |
| *NAC035* | 138.6871153 | 1.987825207 | 0.014333164 | -6.124499046 | 1.25818E-31 | 1.15542E-29 | - |
| *CAB6A* | 24548.56498 | 1484.059008 | 0.060454002 | -4.04801833 | 1.59593E-31 | 1.45043E-29 | K08907 |
| *PIP2-8* | 5657.429964 | 900.244139 | 0.159125989 | -2.651758613 | 1.74052E-31 | 1.5764E-29 | K09872 |
| *TUBA* | 4485.789133 | 276.4849675 | 0.061635748 | -4.020088856 | 1.91032E-31 | 1.72426E-29 | K07374 |
| *CYP735A2* | 293.0704474 | 4.95673917 | 0.016913132 | -5.885712342 | 2.17687E-31 | 1.95815E-29 | - |
| *PIP2-8* | 3366.412874 | 215.0957 | 0.063894629 | -3.968161538 | 2.25994E-31 | 2.02596E-29 | K09872 |
| *CRK8* | 639.5145457 | 73.64094729 | 0.115151325 | -3.118397083 | 3.46806E-31 | 3.09845E-29 | - |
| *PSAH* | 538.5067768 | 57.626652 | 0.107011935 | -3.224156391 | 5.0315E-31 | 4.46499E-29 | K02695 |
| *45537* | 1109.853918 | 150.6275846 | 0.135718388 | -2.881311895 | 5.10928E-31 | 4.5188E-29 | - |
| *TAT* | 125.6417903 | 1.314472241 | 0.010462062 | -6.578688919 | 5.94963E-31 | 5.24443E-29 | K15400 |
| *ACA7* | 138.2082098 | 2.351920222 | 0.017017225 | -5.876860382 | 5.98977E-31 | 5.26221E-29 | K01674 |
| *PGK1* | 10606.10531 | 1736.709036 | 0.163746162 | -2.610467004 | 7.26567E-31 | 6.36193E-29 | K00927 |
| *IQD2* | 294.1862251 | 20.66796463 | 0.070254699 | -3.831261471 | 7.40129E-31 | 6.4379E-29 | - |
| *PATL3* | 1281.012985 | 180.7001923 | 0.141060391 | -2.825615153 | 8.60869E-31 | 7.41472E-29 | - |
| *CYP75A5* | 338.3057856 | 28.26500614 | 0.083548693 | -3.581238934 | 1.17292E-30 | 1.00368E-28 | K13083 |
| *PT30* | 103.1372621 | 0 | 0 | -Inf | 1.20446E-30 | 1.02734E-28 | K12742 |
| *NIP6-1* | 255.3954705 | 16.6986018 | 0.065383312 | -3.934933725 | 2.48543E-30 | 2.09955E-28 | K09874 |
| *PHT2-1* | 844.5474088 | 112.2424266 | 0.132902458 | -2.911560307 | 5.56706E-30 | 4.62856E-28 | K14640 |
| *RIC10* | 257.1182056 | 4.028865846 | 0.015669314 | -5.995914188 | 5.74831E-30 | 4.76423E-28 | - |
| *RNR1* | 264.8662797 | 18.59631193 | 0.07021019 | -3.832175749 | 5.80682E-30 | 4.79763E-28 | K10807 |
| *IRK* | 517.7073595 | 58.79121418 | 0.113560708 | -3.138464349 | 6.46708E-30 | 5.32645E-28 | - |
| *PSAL* | 2834.972078 | 225.5543165 | 0.079561389 | -3.651787725 | 7.47227E-30 | 6.13518E-28 | K02699 |
| *ALDH3F1* | 308.534105 | 25.0768434 | 0.081277379 | -3.62100231 | 7.6946E-30 | 6.2981E-28 | K00128 |
| *CYP750A1* | 2008.730061 | 7.402117203 | 0.003684974 | -8.084130009 | 7.92677E-30 | 6.46804E-28 | - |
| *RBCS* | 817.5430138 | 34.21874554 | 0.04185559 | -4.578435861 | 1.10424E-29 | 8.95485E-28 | K01602 |
| *FTIP3* | 446.1572296 | 17.09483104 | 0.03831571 | -4.705920149 | 1.18786E-29 | 9.5741E-28 | - |
| *NFD4* | 99.09476371 | 0 | 0 | -Inf | 1.20134E-29 | 9.65322E-28 | - |
| *GAT1* | 783.3658706 | 103.8110754 | 0.132519273 | -2.915725904 | 1.24658E-29 | 9.98632E-28 | - |
| *PCR6* | 119.9314607 | 0 | 0 | -Inf | 1.4698E-29 | 1.16331E-27 | - |
| *UGT86A1* | 318.4391143 | 27.21610083 | 0.085467204 | -3.548485259 | 1.50271E-29 | 1.18579E-27 | - |
| *ag1* | 204.7134927 | 5.361076565 | 0.026188194 | -5.254939644 | 1.51612E-29 | 1.1928E-27 | K18108 |
| *guaA* | 817.887897 | 111.8237063 | 0.136722535 | -2.870677047 | 1.58366E-29 | 1.24223E-27 | K01246 |
| *At1g01500* | 137.7014184 | 3.054263617 | 0.022180335 | -5.494575022 | 1.75627E-29 | 1.37354E-27 | - |
| *CYP720B2* | 378.9561944 | 36.73325691 | 0.096932726 | -3.366872368 | 1.89691E-29 | 1.47914E-27 | - |
| *R53* | 247.883392 | 17.22052254 | 0.069470255 | -3.847460789 | 2.16233E-29 | 1.68113E-27 | K00430 |
| *CSLE6* | 2049.85282 | 329.2174341 | 0.160605401 | -2.638407685 | 2.28222E-29 | 1.76912E-27 | - |
| *uncharacterized protein_42501* | 276.8976501 | 20.91904279 | 0.075547925 | -3.726464067 | 2.56115E-29 | 1.97952E-27 | - |
| *LSI2* | 130.4929589 | 2.384153913 | 0.018270364 | -5.774350779 | 2.74564E-29 | 2.11591E-27 | - |
| *uncharacterized protein_29718* | 626.2614591 | 78.58796311 | 0.125487465 | -2.994384832 | 3.36457E-29 | 2.58532E-27 | - |
| *PT1* | 324.6949483 | 28.95751384 | 0.089183752 | -3.487075289 | 3.71359E-29 | 2.83697E-27 | K08176 |
| *GDCSH* | 8267.616429 | 1329.116267 | 0.160761724 | -2.637004145 | 3.99351E-29 | 3.04199E-27 | K02437 |
| *ag1* | 142.8125882 | 3.685752572 | 0.025808317 | -5.276020116 | 4.62581E-29 | 3.50339E-27 | K16086 |
| *HHT1* | 371.9069545 | 10.01494484 | 0.02692863 | -5.214715346 | 4.97232E-29 | 3.75502E-27 | K15400 |
| *PLGG1* | 2445.302722 | 399.6180889 | 0.163422747 | -2.613319284 | 5.21879E-29 | 3.9299E-27 | - |
| *comta* | 251.0078562 | 17.54274711 | 0.069889235 | -3.83878593 | 7.08711E-29 | 5.32159E-27 | K00545 |
| *LTPG5* | 165.8391393 | 5.035701767 | 0.030364978 | -5.04144788 | 1.08336E-28 | 8.11168E-27 | - |
| *GSTU6* | 425.785172 | 45.89279806 | 0.107783927 | -3.213786034 | 1.15968E-28 | 8.65851E-27 | K00799 |
| *LRR1* | 94.15292748 | 0 | 0 | -Inf | 1.89238E-28 | 1.39317E-26 | - |
| *KRP7* | 597.7038256 | 78.22275025 | 0.130872092 | -2.933770611 | 2.14437E-28 | 1.56991E-26 | - |
| *BGAL3* | 2621.857168 | 234.5101568 | 0.089444291 | -3.48286678 | 2.24587E-28 | 1.63514E-26 | - |
| *TRXM* | 3587.449525 | 617.7935206 | 0.172209676 | -2.537761888 | 2.61558E-28 | 1.89385E-26 | K03671 |
| *CYP75A3* | 1091.964025 | 169.4679128 | 0.155195509 | -2.687841285 | 2.70394E-28 | 1.95247E-26 | K13083 |
| *LAC1* | 158.106484 | 5.952223671 | 0.03764693 | -4.731323983 | 2.71279E-28 | 1.9535E-26 | K05909 |
| *uncharacterized protein_50037* | 3102.017757 | 530.1065076 | 0.170890868 | -2.548852788 | 2.85161E-28 | 2.04787E-26 | - |
| *uncharacterized protein_15657* | 103.7332615 | 0.657236121 | 0.006335828 | -7.302251075 | 3.85247E-28 | 2.75164E-26 | - |
| *RANBP1C* | 386.3969608 | 41.15530767 | 0.106510433 | -3.230933339 | 4.86353E-28 | 3.44578E-26 | K15306 |
| *At4g27220* | 107.6605582 | 1.005214289 | 0.009336885 | -6.742842915 | 5.30129E-28 | 3.74587E-26 | K13459 |
| *CEPR1* | 834.3001283 | 120.7433024 | 0.144724061 | -2.788623301 | 6.83104E-28 | 4.76293E-26 | - |
| *LPA3* | 645.3538602 | 81.84150571 | 0.126816481 | -2.979185842 | 6.88282E-28 | 4.78637E-26 | - |
| *CHLH* | 14171.86751 | 2603.995412 | 0.183743985 | -2.444231072 | 8.56257E-28 | 5.93882E-26 | K03403 |
| *UCNL* | 240.5122088 | 17.61674534 | 0.073246782 | -3.771090811 | 1.03248E-27 | 7.14225E-26 | - |
| *WAXY* | 15406.09907 | 2877.04008 | 0.186746825 | -2.420844383 | 1.10087E-27 | 7.57557E-26 | K13679 |
| *nep2* | 96.93065523 | 0.344734906 | 0.003556511 | -8.135321808 | 1.16693E-27 | 8.0093E-26 | - |
| *CSE* | 180.7835104 | 9.385077516 | 0.051913349 | -4.267750621 | 1.18631E-27 | 8.12113E-26 | - |
| *PXC3* | 249.3640262 | 19.68839804 | 0.078954444 | -3.662835719 | 1.63602E-27 | 1.11418E-25 | - |
| *SBT5.1* | 681.423248 | 95.31920292 | 0.139882523 | -2.837712376 | 1.73927E-27 | 1.18145E-25 | - |
| *ag1* | 154.0341792 | 3.286180603 | 0.0213341 | -5.550694931 | 1.91041E-27 | 1.29105E-25 | K16086 |
| *uncharacterized protein_04893* | 1129.627801 | 178.50386 | 0.158020066 | -2.661820323 | 1.95889E-27 | 1.32042E-25 | - |
| *MGP* | 1611.641948 | 268.8524426 | 0.166818966 | -2.583644777 | 2.02756E-27 | 1.36322E-25 | K12309 |
| *CYP720B2* | 88.59266298 | 0 | 0 | -Inf | 3.35457E-27 | 2.22143E-25 | - |
| *EO* | 7731.017161 | 1434.725056 | 0.185580374 | -2.429883948 | 3.36953E-27 | 2.22575E-25 | K18980 |
| *GLYM1* | 12609.80074 | 2369.488137 | 0.187908452 | -2.411898135 | 3.92668E-27 | 2.58729E-25 | K00600 |
| *GLOX* | 610.293446 | 56.35414324 | 0.092339421 | -3.436909509 | 4.12742E-27 | 2.71277E-25 | - |
| *H1* | 1744.540892 | 301.6883868 | 0.172932826 | -2.531716347 | 4.50368E-27 | 2.93809E-25 | K11275 |
| *H1* | 3293.572937 | 596.5887571 | 0.181137254 | -2.464844804 | 5.27587E-27 | 3.41648E-25 | K11275 |
| *At4g16563* | 121.675537 | 2.662799805 | 0.02188443 | -5.513951364 | 6.87935E-27 | 4.43306E-25 | - |
| *uncharacterized protein_48451* | 113.9163921 | 2.053914221 | 0.018030015 | -5.793455619 | 7.51889E-27 | 4.82161E-25 | - |
| *BASS3* | 3553.763903 | 400.1365437 | 0.11259514 | -3.150783539 | 8.62216E-27 | 5.51567E-25 | K03453 |
| *WAXY* | 8300.820129 | 1583.151906 | 0.190722348 | -2.390454193 | 9.30662E-27 | 5.93911E-25 | K13679 |
| *KCS11* | 268.9672512 | 24.04598141 | 0.089401149 | -3.48356281 | 1.08444E-26 | 6.88712E-25 | K15397 |
| *PT30* | 129.8023506 | 3.682509309 | 0.028370128 | -5.139483527 | 1.11352E-26 | 7.0548E-25 | K12742 |
| *SRG1* | 444.2792529 | 2.397027496 | 0.005395317 | -7.534076503 | 1.27631E-26 | 8.06676E-25 | - |
| *SRG1* | 108.8224702 | 1.694684101 | 0.015572924 | -6.0048163 | 1.59416E-26 | 1.00276E-24 | - |
| *LOX1.1* | 453.789595 | 54.45957046 | 0.12001062 | -3.058766021 | 2.00951E-26 | 1.25802E-24 | K15718 |
| *OPT3* | 5483.656514 | 1045.264418 | 0.190614495 | -2.391270262 | 2.17218E-26 | 1.35663E-24 | - |
| *CYP75A1* | 181.6714338 | 10.80273736 | 0.059463049 | -4.071862751 | 2.40645E-26 | 1.4994E-24 | K13083 |
| *tC1* | 12872.94741 | 2479.698144 | 0.192628624 | -2.376105997 | 2.48522E-26 | 1.54482E-24 | K02636 |
| *GASA2* | 91.75937244 | 0.32861806 | 0.003581302 | -8.125299944 | 2.84863E-26 | 1.76656E-24 | - |
| *DJC76* | 1032.071988 | 168.3647458 | 0.163132754 | -2.615881616 | 3.00475E-26 | 1.85899E-24 | - |
| *PRXQ* | 5760.075011 | 1095.793807 | 0.190239503 | -2.394111244 | 3.0619E-26 | 1.88992E-24 | K03564 |
| *BRG3* | 314.3029076 | 32.74645401 | 0.104187563 | -3.262745022 | 3.12764E-26 | 1.92598E-24 | K19042 |
| *CSLE1* | 2910.872295 | 544.0233487 | 0.186893582 | -2.419711071 | 3.70353E-26 | 2.27001E-24 | - |
| *RBCS* | 1326.189906 | 226.0887996 | 0.170479958 | -2.552325952 | 3.85875E-26 | 2.35966E-24 | K01602 |
| *At3g02645* | 206.9996281 | 14.59299431 | 0.070497684 | -3.826280333 | 4.02429E-26 | 2.45519E-24 | - |
| *MYB20* | 335.043319 | 37.45993122 | 0.111806232 | -3.160927484 | 4.52451E-26 | 2.75399E-24 | K09422 |
| *EXPB16* | 84.14303897 | 0 | 0 | -Inf | 4.71383E-26 | 2.86005E-24 | - |
| *CYCD2-2* | 301.6557146 | 30.70379174 | 0.10178422 | -3.296414177 | 4.72045E-26 | 2.86005E-24 | K18810 |
| *RPI3* | 1402.323765 | 241.9975721 | 0.172568973 | -2.534754996 | 5.12114E-26 | 3.09571E-24 | K01807 |
| *CRK2* | 174.1611561 | 3.966020095 | 0.022772128 | -5.456587092 | 5.28673E-26 | 3.18121E-24 | - |
| *TCEA1* | 2151.828857 | 396.794728 | 0.184398832 | -2.439098577 | 5.3204E-26 | 3.19418E-24 | - |
| *CYP75A5* | 527.724382 | 73.716872 | 0.139688206 | -2.839717881 | 6.14688E-26 | 3.68198E-24 | K13083 |
| *UNI* | 128.2301483 | 4.015992263 | 0.031318628 | -4.996835195 | 6.58412E-26 | 3.93494E-24 | K13459 |
| *Os08g0500300* | 337.2909306 | 37.79827262 | 0.112064302 | -3.157601316 | 8.92262E-26 | 5.2965E-24 | - |
| *WAV3* | 145.7450095 | 6.355038903 | 0.043603818 | -4.519401718 | 9.26805E-26 | 5.48918E-24 | - |
| *slr0575* | 3029.645608 | 568.7800414 | 0.187738143 | -2.413206298 | 9.43678E-26 | 5.57659E-24 | - |
| *At2g23540* | 112.77148 | 2.321308162 | 0.020584178 | -5.602320386 | 1.06445E-25 | 6.26222E-24 | - |
| *TPS5* | 2958.152968 | 563.6396749 | 0.190537704 | -2.391851585 | 1.09354E-25 | 6.41898E-24 | K16055 |
| *CYP735A2* | 123.9823329 | 3.664770832 | 0.029558815 | -5.08026776 | 1.28676E-25 | 7.53641E-24 | K10717 |
| *H2A* | 3063.507024 | 582.931433 | 0.190282388 | -2.393786057 | 1.39544E-25 | 8.15484E-24 | K11251 |
| *UBC23* | 151.0143463 | 7.018761549 | 0.046477449 | -4.42732531 | 1.57713E-25 | 9.17593E-24 | K10581 |
| *COL16* | 211.2068426 | 7.265074281 | 0.034397912 | -4.861535216 | 1.66561E-25 | 9.66934E-24 | - |
| *CB21* | 113133.1279 | 4037.339351 | 0.035686624 | -4.80847278 | 1.72912E-25 | 9.99401E-24 | K08913 |
| *SRG1* | 131.261052 | 4.610382633 | 0.035123767 | -4.8314086 | 1.83864E-25 | 1.06038E-23 | - |
| *lhcA-P4* | 32631.72039 | 1442.414697 | 0.044202839 | -4.499717143 | 1.88095E-25 | 1.08241E-23 | K08910 |
| *H1* | 805.1234903 | 130.030176 | 0.161503394 | -2.630363614 | 1.92459E-25 | 1.10474E-23 | K11275 |
| *At5g07050* | 105.666003 | 0.32861806 | 0.00310997 | -8.328883792 | 1.92814E-25 | 1.10474E-23 | - |
| *REC2* | 18007.7382 | 3639.564406 | 0.202111135 | -2.306779288 | 2.05194E-25 | 1.17312E-23 | K03255 |
| *SLC1* | 87.36784521 | 0.330239692 | 0.003779877 | -8.04744507 | 2.26691E-25 | 1.29322E-23 | - |
| *RK8* | 210.9870378 | 16.99671371 | 0.080558094 | -3.633826633 | 2.61922E-25 | 1.49098E-23 | - |
| *CYP716B1* | 1912.269551 | 359.6551315 | 0.188077633 | -2.410599804 | 2.91661E-25 | 1.65668E-23 | - |
| *At5g48900* | 85.45257139 | 0.344734906 | 0.004034225 | -7.95349271 | 5.44722E-25 | 3.06767E-23 | K01728 |
| *PT30* | 85.39168189 | 0.32861806 | 0.003848361 | -8.021539961 | 6.13068E-25 | 3.44521E-23 | K12742 |
| *DIR* | 433.1630283 | 58.46116698 | 0.134963428 | -2.889359577 | 7.18805E-25 | 4.03082E-23 | - |
| *SIGA* | 1221.79864 | 217.8349456 | 0.178290381 | -2.487699222 | 8.09662E-25 | 4.52107E-23 | K03086 |
| *LAC17* | 142.585806 | 4.120702026 | 0.028899805 | -5.112796419 | 8.37116E-25 | 4.66449E-23 | K05909 |
| *At5g10770* | 147.9419977 | 7.374748406 | 0.049848917 | -4.32629402 | 8.69113E-25 | 4.83256E-23 | - |
| *CSLE1* | 590.4785989 | 16.05596037 | 0.027191435 | -5.200703906 | 8.85624E-25 | 4.90368E-23 | - |
| *mcfF* | 1327.25995 | 239.0055639 | 0.180074419 | -2.473334849 | 8.94339E-25 | 4.94155E-23 | K15113 |
| *At1g80170* | 463.9692326 | 64.69377053 | 0.139435475 | -2.842330434 | 9.38974E-25 | 5.16651E-23 | - |
| *SPAC24B11.05* | 130.153176 | 4.961504596 | 0.038120503 | -4.713289017 | 1.17204E-24 | 6.40878E-23 | K07025 |
| *PSB28* | 1160.29175 | 164.1373145 | 0.141462106 | -2.821512451 | 1.18351E-24 | 6.45809E-23 | K08903 |
| *HHT1* | 110.5337952 | 2.662799805 | 0.024090368 | -5.375399756 | 1.4955E-24 | 8.14369E-23 | K15400 |
| *At4g39970* | 1819.277556 | 344.8405318 | 0.18954806 | -2.399364402 | 1.51675E-24 | 8.24237E-23 | - |
| *A6* | 281.8761223 | 30.00621377 | 0.106451776 | -3.231728078 | 1.63473E-24 | 8.86522E-23 | - |
| *MOT2* | 1053.011143 | 186.2006469 | 0.176826853 | -2.499590713 | 1.81115E-24 | 9.80176E-23 | - |
| *CIPK18* | 871.6114971 | 148.8880852 | 0.170819322 | -2.54945692 | 1.9656E-24 | 1.06159E-22 | K07198 |
| *At4g01130* | 197.2086396 | 15.84969108 | 0.080370166 | -3.63719613 | 2.17457E-24 | 1.16965E-22 | - |
| *HPCA1* | 201.0280489 | 16.20404986 | 0.080605915 | -3.632970476 | 2.43058E-24 | 1.30469E-22 | - |
| *LAC11* | 91.99350399 | 1.018087872 | 0.011066954 | -6.497597998 | 2.6619E-24 | 1.42595E-22 | K05909 |
| *mhpC* | 192.6818069 | 15.02327781 | 0.077969363 | -3.680948835 | 2.77836E-24 | 1.48533E-22 | - |
| *mhpC* | 116.1989627 | 3.616420295 | 0.031122656 | -5.005891025 | 3.40967E-24 | 1.81546E-22 | - |
| *TIC55* | 529.2282171 | 80.40772902 | 0.151933942 | -2.718483895 | 3.45279E-24 | 1.83471E-22 | - |
| *CYP720B1* | 1975.535122 | 20.18132835 | 0.010215626 | -6.613078549 | 3.97589E-24 | 2.10419E-22 | - |
| *ag1* | 152.6828017 | 3.291045497 | 0.021554788 | -5.535847784 | 4.00352E-24 | 2.11457E-22 | K16086 |
| *WNK4* | 121.3361261 | 4.334980004 | 0.035727035 | -4.806840007 | 4.06939E-24 | 2.14506E-22 | K08867 |
| *TRXM2* | 1635.608257 | 312.5695745 | 0.191102957 | -2.387577993 | 4.52376E-24 | 2.3798E-22 | K03671 |
| *CAT1* | 331.3448208 | 40.96657148 | 0.123637277 | -3.015814306 | 5.20274E-24 | 2.7207E-22 | K03294 |
| *At4g33300* | 1261.964069 | 236.1161292 | 0.187102101 | -2.418102333 | 5.30565E-24 | 2.76902E-22 | - |
| *Prcp* | 183.4826048 | 13.69096762 | 0.074617251 | -3.744346975 | 5.42843E-24 | 2.82751E-22 | K01285 |
| *At1g32780* | 76.35281533 | 0 | 0 | -Inf | 5.88133E-24 | 3.05736E-22 | K00001 |
| *TPS-mISO1* | 75.84968455 | 0 | 0 | -Inf | 6.09429E-24 | 3.16183E-22 | K04120 |
| *ANR* | 3863.791411 | 802.1143273 | 0.20759773 | -2.268137424 | 6.44672E-24 | 3.335E-22 | K08695 |
| *HPCA1* | 128.0670483 | 4.661976433 | 0.036402623 | -4.779813796 | 6.813E-24 | 3.5071E-22 | - |
| *BGLU1* | 1319.412209 | 250.7258436 | 0.19002844 | -2.395712745 | 7.0981E-24 | 3.64673E-22 | K05350 |
| *uncharacterized protein_28594* | 333.7977211 | 41.34536064 | 0.12386352 | -3.013176746 | 7.95544E-24 | 4.07133E-22 | - |
| *XTH9* | 1452.688196 | 281.2089703 | 0.193578341 | -2.369010556 | 8.08341E-24 | 4.12881E-22 | K08235 |
| *NAKR2* | 102.5216265 | 2.321308162 | 0.022642132 | -5.464846404 | 8.71524E-24 | 4.44292E-22 | - |
| *UCC1* | 110.7906317 | 3.416737 | 0.030839584 | -5.019072882 | 1.34317E-23 | 6.82091E-22 | - |
| *IRL1* | 470.33015 | 70.14504232 | 0.149140008 | -2.745260771 | 1.39759E-23 | 7.08362E-22 | - |
| *PLP7* | 222.8004957 | 12.75194182 | 0.0572348 | -4.126963586 | 1.49284E-23 | 7.55188E-22 | - |
| *UGT73C13* | 153.2460737 | 9.346357299 | 0.060989212 | -4.035302111 | 1.51113E-23 | 7.62975E-22 | K13496 |
| *TPS-LAS2* | 74.37171906 | 0 | 0 | -Inf | 1.69909E-23 | 8.56239E-22 | K16086 |
| *OASB* | 4133.160953 | 868.9921214 | 0.210248798 | -2.249830544 | 2.00595E-23 | 1.00702E-21 | K01738 |
| *ROQ1* | 106.917991 | 3.054263617 | 0.028566414 | -5.129536236 | 2.03329E-23 | 1.01881E-21 | - |
| *ASPG1* | 94.49435465 | 1.647955196 | 0.017439721 | -5.841479217 | 2.23227E-23 | 1.11217E-21 | - |
| *IRK* | 224.1865212 | 21.85502427 | 0.097485898 | -3.358662656 | 2.24038E-23 | 1.1141E-21 | - |
| *AATL1* | 360.27548 | 48.50744626 | 0.134639877 | -2.892822333 | 2.35474E-23 | 1.16686E-21 | - |
| *slr0537* | 1624.55528 | 321.2711843 | 0.197759466 | -2.338181344 | 2.35532E-23 | 1.16686E-21 | - |
| *DJC76* | 539.4721175 | 85.8674267 | 0.159169351 | -2.651365534 | 3.15508E-23 | 1.55722E-21 | - |
| *uncharacterized protein_03554* | 687.2409689 | 117.9044604 | 0.171562037 | -2.543197746 | 3.28544E-23 | 1.61551E-21 | - |
| *LHCA3* | 33857.34462 | 3747.47957 | 0.110684391 | -3.175476311 | 3.32599E-23 | 1.6324E-21 | K08909 |
| *PIP1-5* | 291.5822396 | 34.96305241 | 0.119908032 | -3.059999799 | 3.4104E-23 | 1.67071E-21 | K09872 |
| *FANCM* | 240.0696913 | 24.68375796 | 0.102819135 | -3.281819317 | 3.48169E-23 | 1.70248E-21 | K10896 |
| *At1g67720* | 72.76576924 | 0 | 0 | -Inf | 4.45845E-23 | 2.17605E-21 | - |
| *ag1* | 96.93028329 | 2.003942053 | 0.020674055 | -5.596034771 | 4.60717E-23 | 2.24448E-21 | K16086 |
| *CYP716B2* | 654.1291846 | 111.4551314 | 0.170387034 | -2.553112543 | 4.76297E-23 | 2.3161E-21 | - |
| *PTAC5* | 1275.119363 | 246.3932853 | 0.193231546 | -2.371597457 | 5.20731E-23 | 2.52751E-21 | - |
| *EXPA2* | 222.4448613 | 3.995010523 | 0.017959554 | -5.799104658 | 5.30204E-23 | 2.56876E-21 | - |
| *At4g34480* | 505.4680598 | 2.036175744 | 0.004028298 | -7.955614035 | 5.51508E-23 | 2.66219E-21 | - |
| *ABCA7* | 460.2850938 | 70.41914104 | 0.152990271 | -2.708488187 | 6.09557E-23 | 2.93166E-21 | - |
| *DCR* | 1122.908243 | 19.67390282 | 0.01752049 | -5.834813053 | 6.38464E-23 | 3.06509E-21 | K19747 |
| *CRK3* | 119.8101101 | 5.029215241 | 0.041976551 | -4.574272545 | 6.44601E-23 | 3.08893E-21 | - |
| *IRKI* | 112.2033946 | 1.676945624 | 0.014945587 | -6.064136604 | 9.23099E-23 | 4.39949E-21 | - |
| *DAD2* | 71.57776785 | 0 | 0 | -Inf | 9.43291E-23 | 4.47953E-21 | - |
| *EPHX4* | 91.122052 | 1.66245041 | 0.018244216 | -5.776417024 | 1.00624E-22 | 4.76989E-21 | - |
| *SULTR3;1* | 91.24696743 | 1.66245041 | 0.01821924 | -5.7783934 | 1.18229E-22 | 5.5843E-21 | K17471 |
| *uncharacterized protein_50712* | 423.6226514 | 63.411625 | 0.149688938 | -2.73996048 | 1.2812E-22 | 6.04064E-21 | - |
| *ISPG* | 9365.205686 | 2093.456272 | 0.223535536 | -2.161423897 | 1.72251E-22 | 8.09244E-21 | K03526 |
| *BAM1* | 753.0681695 | 138.036407 | 0.183298687 | -2.44773164 | 1.99719E-22 | 9.3662E-21 | - |
| *CYP75A3* | 223.5532094 | 23.52903148 | 0.105250251 | -3.248104416 | 2.10765E-22 | 9.84914E-21 | K13083 |
| *HSP22* | 351.4535015 | 48.90185493 | 0.139141749 | -2.845372733 | 2.20682E-22 | 1.0258E-20 | K13993 |
| *XTH9* | 990.4082356 | 193.6545633 | 0.195530041 | -2.354537813 | 2.43903E-22 | 1.12777E-20 | K08235 |
| *NPF3.1* | 2062.431315 | 434.8816033 | 0.210858708 | -2.245651489 | 2.50722E-22 | 1.15525E-20 | K14638 |
| *uncharacterized protein_13035* | 399.0633787 | 60.53921318 | 0.151703254 | -2.720676062 | 2.66246E-22 | 1.22038E-20 | - |
| *WAXY* | 1977.26883 | 421.7479252 | 0.213298222 | -2.229056157 | 2.78489E-22 | 1.26987E-20 | K13679 |
| *PAD4* | 223.4263059 | 23.78325343 | 0.106447866 | -3.23178107 | 2.9088E-22 | 1.3218E-20 | - |
| *TCP14* | 386.5100179 | 57.94259541 | 0.149912273 | -2.737809594 | 3.0234E-22 | 1.36915E-20 | - |
| *PATL4* | 913.3883935 | 175.9311882 | 0.192613777 | -2.376217198 | 3.27435E-22 | 1.47972E-20 | K19996 |
| *CYP75A5* | 1891.231858 | 404.206925 | 0.213726796 | -2.2261603 | 3.27878E-22 | 1.47972E-20 | K13083 |
| *SMC1* | 994.2088008 | 192.0092288 | 0.19312767 | -2.372373219 | 3.39549E-22 | 1.52977E-20 | K06636 |
| *At3g18200* | 98.66152048 | 2.693411865 | 0.027299517 | -5.194980758 | 3.44846E-22 | 1.55098E-20 | - |
| *PORA* | 7446.180305 | 1132.936016 | 0.152149957 | -2.716434165 | 3.48485E-22 | 1.56468E-20 | K00218 |
| *OPT4* | 296.9156262 | 38.92440655 | 0.131095851 | -2.931306073 | 3.76677E-22 | 1.68552E-20 | - |
| *N* | 1037.907637 | 203.6996164 | 0.196259869 | -2.349162896 | 3.7953E-22 | 1.6954E-20 | - |
| *UNI* | 112.7720041 | 4.652246644 | 0.04125356 | -4.599337565 | 4.10693E-22 | 1.83151E-20 | K13459 |
| *RGI1* | 152.0869827 | 10.57720099 | 0.06954705 | -3.845866869 | 4.16349E-22 | 1.8536E-20 | - |
| *PGLP1A* | 1601.874477 | 318.7909132 | 0.19901117 | -2.32907869 | 4.58409E-22 | 2.03398E-20 | K19269 |
| *uncharacterized protein_33491* | 1287.773476 | 156.442175 | 0.121482681 | -3.041177437 | 5.18014E-22 | 2.29073E-20 | - |
| *At4g11680* | 456.1358521 | 74.10002872 | 0.162451665 | -2.621917563 | 6.35474E-22 | 2.80545E-20 | - |
| *MYR2* | 288.5349885 | 37.22465862 | 0.129012633 | -2.954415751 | 6.66198E-22 | 2.93617E-20 | - |
| *PCMP-H42* | 763.9920597 | 145.4451102 | 0.190375159 | -2.39308285 | 8.61438E-22 | 3.77144E-20 | - |
| *DIT1* | 5442.391875 | 1232.826276 | 0.226522879 | -2.142271325 | 9.85561E-22 | 4.29346E-20 | - |
| *SWEET3B* | 80.68701788 | 0.990719075 | 0.012278544 | -6.34771673 | 1.02566E-21 | 4.46078E-20 | K15382 |
| *At2g18940* | 205.1761704 | 21.37659561 | 0.104186542 | -3.262759167 | 1.53851E-21 | 6.65829E-20 | - |
| *DOF5.3* | 134.2278733 | 8.375097801 | 0.062394625 | -4.00243444 | 1.74975E-21 | 7.54768E-20 | - |
| *SIGA* | 507.34501 | 68.41154497 | 0.134842255 | -2.890655441 | 1.86382E-21 | 8.02661E-20 | K03086 |
| *At4g34480* | 103.4324071 | 0.673352966 | 0.006510077 | -7.263109609 | 1.94127E-21 | 8.3465E-20 | - |
| *uncharacterized protein_32593* | 689.0396164 | 128.9763064 | 0.187182715 | -2.417480874 | 2.3949E-21 | 1.02634E-19 | - |
| *AIR9* | 659.0879838 | 125.3090079 | 0.190124856 | -2.394980941 | 2.48889E-21 | 1.06489E-19 | - |
| *LAC17* | 84.2726394 | 0.32861806 | 0.003899463 | -8.00250873 | 2.67902E-21 | 1.14438E-19 | K05909 |
| *ATS3A* | 185.5689805 | 17.66023098 | 0.095168012 | -3.393379454 | 3.11375E-21 | 1.32364E-19 | - |
| *VTC2* | 2919.89842 | 658.3330024 | 0.225464351 | -2.149028753 | 3.23366E-21 | 1.3724E-19 | K14190 |
| *SBT5.6* | 70.96542095 | 0.330239692 | 0.00465353 | -7.74745888 | 3.24318E-21 | 1.37423E-19 | - |
| *HIPP22* | 80.52376572 | 0.689469812 | 0.008562315 | -6.867783446 | 3.79361E-21 | 1.59719E-19 | - |
| *Hgsnat* | 623.778748 | 116.5875342 | 0.186905268 | -2.41962086 | 3.8878E-21 | 1.63424E-19 | K10532 |
| *THI1-2* | 24671.64355 | 5370.896824 | 0.217695137 | -2.199618914 | 4.02893E-21 | 1.69087E-19 | K03146 |
| *At4g33300* | 2571.695534 | 583.487637 | 0.226888304 | -2.139945857 | 4.78222E-21 | 1.99431E-19 | - |
| *At2g27500* | 101.851707 | 4.011127369 | 0.039382034 | -4.666318571 | 6.49321E-21 | 2.6908E-19 | - |
| *PSAN* | 7032.935855 | 322.1574899 | 0.045806971 | -4.448289017 | 6.82151E-21 | 2.82241E-19 | K02701 |
| *FDH* | 76.95083106 | 1.001971027 | 0.013020925 | -6.263024211 | 7.03263E-21 | 2.9052E-19 | K15397 |
| *ACT* | 1202.78658 | 257.4767138 | 0.214066833 | -2.22386681 | 7.42042E-21 | 3.05582E-19 | K10355 |
| *ISPH* | 11456.46378 | 2725.343451 | 0.23788697 | -2.071651845 | 7.73754E-21 | 3.17649E-19 | K03527 |
| *CHER1* | 524.4192699 | 95.33299542 | 0.181787743 | -2.459673166 | 9.12928E-21 | 3.74201E-19 | K15377 |
| *CAMT* | 2795.063521 | 361.5159206 | 0.12934086 | -2.950749983 | 9.39324E-21 | 3.84423E-19 | K00588 |
| *uncharacterized protein_28512* | 623.420669 | 118.7428219 | 0.190469819 | -2.392365683 | 9.77167E-21 | 3.9929E-19 | - |
| *MYB123* | 408.2416202 | 67.71285559 | 0.165864655 | -2.591921605 | 1.07297E-20 | 4.36005E-19 | K09422 |
| *UGT92A1* | 252.2066804 | 32.26791945 | 0.127942366 | -2.966434025 | 1.07364E-20 | 4.36005E-19 | - |
| *GDCST* | 11789.27891 | 2825.200616 | 0.239641511 | -2.061050257 | 1.07654E-20 | 4.36511E-19 | K00605 |
| *ZIF1* | 174.2638907 | 16.28615625 | 0.093456861 | -3.419555605 | 1.14722E-20 | 4.64456E-19 | - |
| *At3g47570* | 190.2087181 | 20.04448436 | 0.105381523 | -3.246306162 | 1.16211E-20 | 4.69762E-19 | - |
| *DAD2* | 63.56671941 | 0 | 0 | -Inf | 1.32734E-20 | 5.35733E-19 | - |
| *uncharacterized protein_40390* | 494.5856798 | 88.55577473 | 0.179050422 | -2.481562175 | 1.70406E-20 | 6.83066E-19 | - |
| *AMT3-1* | 2495.671139 | 580.568771 | 0.232630318 | -2.103888963 | 1.70533E-20 | 6.83066E-19 | K03320 |
| *R40* | 695.1808292 | 137.208478 | 0.197370917 | -2.341018671 | 1.80007E-20 | 7.18826E-19 | K00430 |
| *GLCAT14A* | 224.8953409 | 27.97815263 | 0.124405212 | -3.006881163 | 1.81243E-20 | 7.22667E-19 | - |
| *CYP720B2* | 269.144182 | 37.64693987 | 0.139876477 | -2.837774734 | 1.84016E-20 | 7.32619E-19 | - |
| *BMY1* | 691.8672476 | 135.8254929 | 0.196317275 | -2.348740964 | 1.84587E-20 | 7.33597E-19 | - |
| *ag1* | 75.49684291 | 1.018087872 | 0.013485171 | -6.212482323 | 1.84819E-20 | 7.33597E-19 | K16086 |
| *HHT1* | 252.724734 | 33.11865718 | 0.131046363 | -2.931850783 | 1.99387E-20 | 7.90231E-19 | K15400 |
| *At2g39510* | 160.8274156 | 14.27258388 | 0.088744719 | -3.494194911 | 2.03179E-20 | 8.04052E-19 | - |
| *HCT* | 1008.719881 | 218.3939798 | 0.216506073 | -2.207520603 | 2.2666E-20 | 8.94289E-19 | K13065 |
| *SHM1* | 807.3332892 | 164.8606461 | 0.204203949 | -2.291917326 | 2.42809E-20 | 9.56573E-19 | K00600 |
| *At2g05160* | 557.2780305 | 104.9852615 | 0.188389378 | -2.408210472 | 2.49853E-20 | 9.82856E-19 | - |
| *DF1* | 238.2653737 | 30.25586924 | 0.126983912 | -2.977282366 | 2.72086E-20 | 1.06554E-18 | - |
| *ELI* | 1133.476049 | 245.7432256 | 0.216804957 | -2.205530355 | 2.7707E-20 | 1.08345E-18 | K00616 |
| *CURT1A* | 2750.103298 | 321.483032 | 0.11689853 | -3.096671311 | 2.90245E-20 | 1.13161E-18 | - |
| *BGLU4* | 846.6351482 | 176.3567062 | 0.208303077 | -2.263243946 | 2.9233E-20 | 1.13806E-18 | K01188 |
| *ABA2* | 5210.16396 | 1252.731846 | 0.240440005 | -2.056251142 | 2.99844E-20 | 1.16559E-18 | K09838 |
| *rmpB* | 695.8526454 | 137.7174192 | 0.197911756 | -2.337070781 | 3.02607E-20 | 1.17459E-18 | - |
| *APS1* | 91.71979155 | 3.00591308 | 0.032772786 | -4.931357879 | 3.05536E-20 | 1.18422E-18 | - |
| *At3g61590* | 333.2921739 | 14.25150267 | 0.042759788 | -4.547601492 | 3.68378E-20 | 1.4236E-18 | - |
| *KIN5A* | 459.6862015 | 83.72503835 | 0.1821352 | -2.456918324 | 3.7718E-20 | 1.45548E-18 | K10398 |
| *At1g60630* | 105.0738445 | 0.658857752 | 0.006270426 | -7.317220856 | 3.8495E-20 | 1.4833E-18 | - |
| *CHIT5* | 61.36138572 | 0 | 0 | -Inf | 3.91485E-20 | 1.50408E-18 | K01183 |
| *PHT2-1* | 323.4956564 | 50.8012926 | 0.157038562 | -2.67080923 | 4.0589E-20 | 1.55715E-18 | K14640 |
| *uncharacterized protein_08602* | 682.1962502 | 37.12806345 | 0.054424315 | -4.199604852 | 4.20313E-20 | 1.61014E-18 | - |
| *At1g56130* | 123.8764336 | 8.064218218 | 0.065098889 | -3.941223265 | 4.30032E-20 | 1.64498E-18 | - |
| *uncharacterized protein_10569* | 1013.162971 | 220.118355 | 0.217258587 | -2.202514898 | 5.08436E-20 | 1.93927E-18 | - |
| *CRK2* | 126.276442 | 8.619888371 | 0.068262047 | -3.87277252 | 5.73589E-20 | 2.18461E-18 | - |
| *TPS14* | 93.39600086 | 3.357134511 | 0.035945163 | -4.798058528 | 6.08428E-20 | 2.31063E-18 | K04120 |
| *CP12-1* | 15243.52668 | 3733.216715 | 0.244905053 | -2.029705552 | 7.9832E-20 | 3.02306E-18 | - |
| *PMEU1* | 117.2682822 | 7.368261881 | 0.062832522 | -3.992344709 | 8.04258E-20 | 3.04118E-18 | K01051 |
| *XA21* | 241.2871271 | 32.31292725 | 0.133918985 | -2.900567592 | 8.39455E-20 | 3.16972E-18 | - |
| *uncharacterized protein_38924* | 937.5702838 | 201.0191712 | 0.214404375 | -2.221593747 | 8.75903E-20 | 3.30262E-18 | - |
| *EO* | 251.3844272 | 34.26070902 | 0.136288112 | -2.875268367 | 8.97241E-20 | 3.37824E-18 | K18980 |
| *APX2* | 379.4177563 | 64.87915754 | 0.17099663 | -2.547960205 | 9.01212E-20 | 3.38835E-18 | K00434 |
| *At3g47110* | 148.3624301 | 13.05471324 | 0.087992042 | -3.50648313 | 9.38861E-20 | 3.52488E-18 | - |
| *At5g18840* | 225.0004963 | 28.95761331 | 0.12870022 | -2.957913578 | 1.00567E-19 | 3.76498E-18 | K08145 |
| *FBX6* | 117.7962626 | 0.689469812 | 0.00585307 | -7.416590665 | 1.02655E-19 | 3.83768E-18 | - |
| *CCB1* | 492.4999284 | 91.42798762 | 0.185640611 | -2.429415744 | 1.03993E-19 | 3.88221E-18 | - |
| *ag4* | 68.17360999 | 0.674974598 | 0.009900819 | -6.658236362 | 1.25584E-19 | 4.67497E-18 | K18108 |
| *ADG2* | 452.147205 | 83.13702216 | 0.183871583 | -2.443229567 | 1.29614E-19 | 4.81819E-18 | K00975 |
| *At1g17220* | 1977.530989 | 463.8376623 | 0.234553929 | -2.09200843 | 1.36597E-19 | 5.0635E-18 | K02519 |
| *MIOX1* | 80.22079926 | 2.005563685 | 0.025000545 | -5.321896657 | 1.49408E-19 | 5.5306E-18 | K00469 |
| *TMK1* | 688.2744834 | 44.08053061 | 0.064044987 | -3.96477053 | 1.60585E-19 | 5.936E-18 | - |
| *NPF3.1* | 659.3146181 | 134.045429 | 0.203310264 | -2.298245045 | 1.63753E-19 | 6.04463E-18 | K14638 |
| *uncharacterized protein_13679* | 726.8269629 | 150.2581081 | 0.20673161 | -2.274169098 | 1.65835E-19 | 6.11295E-18 | - |
| *HIPP23* | 416.2573536 | 74.12414781 | 0.178072885 | -2.489460241 | 1.66353E-19 | 6.12346E-18 | - |
| *UGT83A1* | 60.66913316 | 0 | 0 | -Inf | 1.93201E-19 | 7.09193E-18 | - |
| *At3g01520* | 164.1946331 | 13.88750712 | 0.084579544 | -3.563547414 | 2.07274E-19 | 7.59794E-18 | - |
| *TAB2* | 484.9223711 | 91.89374807 | 0.189501977 | -2.399715195 | 2.39404E-19 | 8.75136E-18 | - |
| *RGI1* | 82.10893108 | 2.322929794 | 0.02829083 | -5.143521697 | 2.47518E-19 | 9.03545E-18 | - |
| *SWEET3B* | 410.0050237 | 74.39286498 | 0.181443789 | -2.462405423 | 2.63273E-19 | 9.58402E-18 | K15382 |
| *HIPP37* | 66.73268063 | 0.657236121 | 0.009848789 | -6.665837877 | 2.82151E-19 | 1.0257E-17 | - |
| *AGAL2* | 390.3160923 | 71.02966758 | 0.181979859 | -2.458149306 | 2.97205E-19 | 1.07894E-17 | K07407 |
| *PAM68* | 844.712773 | 183.4876696 | 0.217219007 | -2.202777749 | 3.04052E-19 | 1.10228E-17 | - |
| *CRD1* | 6785.325786 | 1112.595662 | 0.163970854 | -2.608488701 | 3.22329E-19 | 1.16534E-17 | K04035 |
| *IP5P2* | 128.4205142 | 9.567121803 | 0.074498392 | -3.746646904 | 3.50854E-19 | 1.26327E-17 | - |
| *uncharacterized protein_03770* | 360.8400288 | 62.22750378 | 0.172451776 | -2.535735109 | 4.17565E-19 | 1.50141E-17 | - |
| *At3g02645* | 111.3838384 | 6.942942746 | 0.062333484 | -4.003848831 | 4.67094E-19 | 1.67265E-17 | - |
| *SOK5* | 180.7996098 | 20.73223307 | 0.114669678 | -3.124444141 | 4.68923E-19 | 1.67692E-17 | - |
| *At1g74320* | 1056.434045 | 82.02366235 | 0.07764201 | -3.687018729 | 5.58935E-19 | 1.99341E-17 | K14156 |
| *RD22* | 372.4280727 | 66.46760972 | 0.178470998 | -2.486238445 | 5.86367E-19 | 2.08842E-17 | - |
| *CHS* | 1643.414707 | 2.321308162 | 0.001412491 | -9.467542809 | 5.88025E-19 | 2.0915E-17 | K00660 |
| *CAS1* | 2159.634621 | 532.818103 | 0.246716781 | -2.019072245 | 5.96139E-19 | 2.1175E-17 | K13034 |
| *ALF1* | 1133.154706 | 261.1067104 | 0.230424592 | -2.1176334 | 6.04197E-19 | 2.14323E-17 | K01623 |
| *Tmem45b* | 87.94532443 | 0 | 0 | -Inf | 6.2608E-19 | 2.21787E-17 | - |
| *UGT86A2* | 140.3079204 | 12.72609518 | 0.090701189 | -3.462734729 | 6.84785E-19 | 2.42258E-17 | - |
| *SAUR32* | 290.9940223 | 46.74353579 | 0.160634007 | -2.638150745 | 7.12059E-19 | 2.51569E-17 | K14488 |
| *At3g47110* | 82.62335235 | 2.638674271 | 0.03193618 | -4.968664423 | 8.27211E-19 | 2.9147E-17 | - |
| *GA20ox1B* | 389.8309533 | 71.53381764 | 0.183499584 | -2.446151305 | 8.32166E-19 | 2.92825E-17 | K05282 |
| *tmem45b* | 68.12249064 | 1.034204718 | 0.015181546 | -6.041537486 | 8.4663E-19 | 2.97517E-17 | - |
| *OMT1* | 298.9437767 | 31.48652043 | 0.105325894 | -3.247067939 | 1.00251E-18 | 3.51827E-17 | K13066 |
| *agaA* | 61.32258129 | 0.32861806 | 0.005358843 | -7.543862846 | 1.03441E-18 | 3.62059E-17 | - |
| *uncharacterized protein_04764* | 492.1148878 | 96.59434525 | 0.196284135 | -2.348984523 | 1.03779E-18 | 3.62757E-17 | - |
| *PSBR* | 71011.06443 | 9695.342078 | 0.136532837 | -2.872680126 | 1.12338E-18 | 3.92157E-17 | K03541 |
| *uncharacterized protein_08788* | 152.7093477 | 15.00868313 | 0.098282675 | -3.346919071 | 1.23866E-18 | 4.31826E-17 | K00574 |
| *ATS1* | 1862.730634 | 457.8158401 | 0.245776728 | -2.024579779 | 1.27894E-18 | 4.45281E-17 | K00630 |
| *P67* | 476.4761197 | 93.53379405 | 0.196303215 | -2.348844296 | 1.33237E-18 | 4.63271E-17 | - |
| *B2* | 3267.74974 | 834.4556075 | 0.255360928 | -1.969390293 | 1.52665E-18 | 5.29427E-17 | - |
| *CXXS1* | 570.2489113 | 117.6311639 | 0.206280383 | -2.277321465 | 1.56931E-18 | 5.43505E-17 | K03671 |
| *OMT1* | 242.2901283 | 35.78784405 | 0.147706571 | -2.759194086 | 1.62032E-18 | 5.59702E-17 | K13066 |
| *HPCA1* | 156.3813281 | 16.29264278 | 0.104185346 | -3.262775723 | 1.68078E-18 | 5.79826E-17 | - |
| *LTPG5* | 64.03625489 | 0.689469812 | 0.010766867 | -6.53725774 | 2.0534E-18 | 7.03765E-17 | - |
| *IF2CP* | 4303.120214 | 1116.181995 | 0.259388987 | -1.946810865 | 2.20421E-18 | 7.54471E-17 | K02519 |
| *TPS4* | 378.6725879 | 69.85180818 | 0.184464919 | -2.438581621 | 2.21266E-18 | 7.56381E-17 | K04120 |
| *GLR3.3* | 429.4209798 | 84.11801144 | 0.195887056 | -2.351906027 | 2.35892E-18 | 8.04292E-17 | K05387 |
| *PHL2* | 419.243687 | 79.84252802 | 0.190444199 | -2.392559754 | 2.5512E-18 | 8.67604E-17 | - |
| *XA21* | 135.0450413 | 12.00601331 | 0.08890377 | -3.491611589 | 2.55935E-18 | 8.69257E-17 | - |
| *CYP26-2* | 1296.454057 | 311.9426391 | 0.240612182 | -2.05521841 | 2.57452E-18 | 8.73281E-17 | K03768 |
| *TKTC* | 20996.86666 | 5578.221139 | 0.265669218 | -1.912297018 | 2.90116E-18 | 9.82815E-17 | K00615 |
| *At3g47110* | 122.5698179 | 9.66706614 | 0.078869874 | -3.664381845 | 3.04907E-18 | 1.0316E-16 | - |
| *yugF* | 614.3479353 | 113.1221483 | 0.184133683 | -2.441174533 | 3.16332E-18 | 1.06888E-16 | - |
| *NRT3.1* | 62.73661661 | 0.689469812 | 0.010989911 | -6.507676531 | 3.25764E-18 | 1.09934E-16 | - |
| *GGPS* | 69.67034369 | 1.320958767 | 0.01896013 | -5.720887341 | 3.36078E-18 | 1.1327E-16 | K13789 |
| *CYT1* | 354.7002645 | 64.96764455 | 0.183162098 | -2.448807101 | 3.43915E-18 | 1.15763E-16 | K00966 |
| *CSE* | 79.99464134 | 2.669286331 | 0.033368314 | -4.905377385 | 3.45027E-18 | 1.15841E-16 | - |
| *PHOS34* | 467.7083882 | 94.60661952 | 0.202276936 | -2.305596267 | 3.54597E-18 | 1.18903E-16 | - |
| *CYP750A1* | 73.87987877 | 1.994311733 | 0.026993977 | -5.211218657 | 4.19251E-18 | 1.40048E-16 | - |
| *HSP21* | 628.5433097 | 137.3670172 | 0.218548213 | -2.193976512 | 4.63435E-18 | 1.54611E-16 | K13993 |
| *RRF* | 1439.026572 | 353.2154371 | 0.245454423 | -2.02647293 | 4.81875E-18 | 1.6056E-16 | K02838 |
| *nep1* | 740.9448154 | 75.84600177 | 0.102363901 | -3.288221061 | 4.91097E-18 | 1.6322E-16 | - |
| *YAB5* | 226.9363066 | 33.24272061 | 0.146484805 | -2.771177075 | 6.00538E-18 | 1.98841E-16 | - |
| *UNI* | 78.32630991 | 2.648304591 | 0.033811175 | -4.886356026 | 6.04022E-18 | 1.99744E-16 | K13459 |
| *At1g32780* | 1019.561969 | 242.3200342 | 0.237670727 | -2.072963872 | 6.27104E-18 | 2.07117E-16 | K00001 |
| *At2g18940* | 463.5625353 | 94.39244101 | 0.203623964 | -2.296020736 | 6.30636E-18 | 2.08023E-16 | K17710 |
| *CA2* | 724.4620702 | 126.3953081 | 0.174467806 | -2.518967248 | 6.92839E-18 | 2.27971E-16 | K15746 |
| *Os04g0533500* | 578.5993098 | 124.5704655 | 0.215296602 | -2.215602543 | 6.97465E-18 | 2.29207E-16 | K08360 |
| *uncharacterized protein_33341* | 155.1333051 | 12.88391447 | 0.083050603 | -3.589865555 | 7.48721E-18 | 2.45745E-16 | - |
| *APSR1* | 245.0630188 | 38.07195415 | 0.155355771 | -2.686352264 | 7.58106E-18 | 2.48515E-16 | - |
| *SCPL45* | 301.4427677 | 52.6426435 | 0.174635616 | -2.517580274 | 8.28102E-18 | 2.70787E-16 | K16297 |
| *CPN20* | 3325.097015 | 873.097284 | 0.262577988 | -1.929182115 | 9.24188E-18 | 3.01833E-16 | K04078 |
| *UGT84A13* | 57.66183487 | 0.344734906 | 0.005978563 | -7.38598555 | 9.75004E-18 | 3.17642E-16 | K13691 |
| *SDR2a* | 1292.1055 | 320.0118604 | 0.247666975 | -2.013526589 | 9.79053E-18 | 3.18567E-16 | - |
| *PME34* | 518.9954593 | 110.949135 | 0.213776697 | -2.225823497 | 1.06891E-17 | 3.46522E-16 | - |
| *ROC1* | 12251.78841 | 3319.441967 | 0.2709353 | -1.883979722 | 1.08655E-17 | 3.51807E-16 | K11294 |
| *CYP92C6* | 90.66824405 | 4.692588493 | 0.05175559 | -4.272141488 | 1.09488E-17 | 3.5407E-16 | - |
| *At5g64970* | 343.8344711 | 63.78838822 | 0.185520632 | -2.430348459 | 1.10715E-17 | 3.57028E-16 | - |
| *PGLP1A* | 1465.734175 | 368.2882828 | 0.251265399 | -1.992716077 | 1.39217E-17 | 4.46376E-16 | K19269 |
| *BGLU13* | 52.0678511 | 0 | 0 | -Inf | 1.43184E-17 | 4.58537E-16 | K01188 |
| *RPS20* | 1667.877832 | 424.6342055 | 0.254595509 | -1.973721125 | 1.44863E-17 | 4.6335E-16 | K02968 |
| *PAT22* | 345.6986494 | 17.36871151 | 0.050242347 | -4.31495233 | 1.58173E-17 | 5.03479E-16 | K20027 |
| *At5g48740* | 256.8332352 | 42.42943806 | 0.165202288 | -2.597694428 | 1.61067E-17 | 5.12072E-16 | - |
| *FZL* | 443.8167187 | 91.2860798 | 0.205684184 | -2.28149723 | 1.88588E-17 | 5.98126E-16 | - |
| *FGM3* | 1420.242087 | 360.1098368 | 0.25355525 | -1.979627951 | 1.92831E-17 | 6.10113E-16 | K11717 |
| *F16P2* | 2315.259676 | 390.3957213 | 0.168618547 | -2.568164867 | 2.0561E-17 | 6.49767E-16 | K03841 |
| *BPA1* | 285.4371704 | 50.89505526 | 0.178305633 | -2.487575817 | 2.09773E-17 | 6.61338E-16 | - |
| *MST3* | 271.6097931 | 34.34271594 | 0.126441376 | -2.983459456 | 2.21585E-17 | 6.9774E-16 | - |
| *RPS1* | 2828.564546 | 749.1052696 | 0.264835841 | -1.916829717 | 2.25305E-17 | 7.0861E-16 | K02945 |
| *XTH8* | 1669.784214 | 12.06885906 | 0.007227796 | -7.112228569 | 2.91515E-17 | 9.13067E-16 | K08235 |
| *RPL11* | 1770.084963 | 450.136275 | 0.254302073 | -1.975384874 | 2.91699E-17 | 9.13067E-16 | K02867 |
| *rumi* | 85.86605457 | 4.391339229 | 0.051141738 | -4.289355012 | 3.36469E-17 | 1.05071E-15 | - |
| *PPD2* | 309.2436337 | 56.69563488 | 0.183336466 | -2.447434327 | 3.41098E-17 | 1.0639E-15 | - |
| *SPL12* | 102.0820258 | 7.005788498 | 0.068629011 | -3.86503762 | 3.42975E-17 | 1.06849E-15 | - |
| *PMEI10* | 292.7245156 | 52.54584296 | 0.179506123 | -2.477895038 | 4.13179E-17 | 1.27964E-15 | - |
| *WRKY42* | 221.3113681 | 33.84826347 | 0.152944079 | -2.708923834 | 4.29582E-17 | 1.32888E-15 | - |
| *EIX2* | 364.3087042 | 72.71419825 | 0.199595007 | -2.324852466 | 4.37683E-17 | 1.35235E-15 | - |
| *uncharacterized protein_15377* | 248.3291981 | 42.40856223 | 0.170775578 | -2.549826424 | 4.48364E-17 | 1.38373E-15 | - |
| *ALDH3H1* | 1049.665082 | 263.4752513 | 0.251008875 | -1.994189721 | 4.84082E-17 | 1.49222E-15 | K00128 |
| *CP33* | 4751.738607 | 1303.675798 | 0.274357642 | -1.865870336 | 5.5137E-17 | 1.6878E-15 | K11294 |
| *CRK8* | 111.8103522 | 9.439914579 | 0.084427912 | -3.566136154 | 5.67552E-17 | 1.73531E-15 | - |
| *tyrP-A* | 293.9528418 | 53.81703494 | 0.183080506 | -2.449449912 | 5.70349E-17 | 1.74185E-15 | K03834 |
| *SLAC1* | 138.958035 | 15.06514182 | 0.108415046 | -3.205363099 | 5.89777E-17 | 1.79909E-15 | - |
| *DMP4* | 61.5805662 | 0.674974598 | 0.010960838 | -6.511498114 | 6.09431E-17 | 1.8569E-15 | - |
| *OsI_15603* | 50.53203679 | 0 | 0 | -Inf | 6.3619E-17 | 1.93396E-15 | K03921 |
| *FKBP16-4* | 129.7393209 | 10.2919691 | 0.079328064 | -3.656024856 | 6.42013E-17 | 1.94941E-15 | - |
| *menG* | 745.9243208 | 177.837906 | 0.2384128 | -2.0684664 | 6.5254E-17 | 1.97682E-15 | - |
| *LRK10L-2.4* | 74.23855381 | 2.725645556 | 0.036714691 | -4.767498736 | 6.9412E-17 | 2.10037E-15 | - |
| *FBP* | 85.2758885 | 4.297881418 | 0.050399726 | -4.310440298 | 7.34342E-17 | 2.21953E-15 | K03841 |
| *YSL12* | 55.68216317 | 0 | 0 | -Inf | 7.78263E-17 | 2.34959E-15 | - |
| *ZOX1* | 1027.815494 | 258.5930249 | 0.251594791 | -1.990826039 | 7.82664E-17 | 2.36017E-15 | K13496 |
| *Os05g0428700* | 363.2153116 | 73.59067672 | 0.202608961 | -2.303230115 | 8.60336E-17 | 2.58552E-15 | - |
| *uncharacterized protein_21809* | 107.3174867 | 8.65050043 | 0.080606625 | -3.632957768 | 9.18349E-17 | 2.75359E-15 | - |
| *CAB6A* | 271.7364855 | 0.32861806 | 0.001209326 | -9.6915808 | 9.39441E-17 | 2.81363E-15 | K08907 |
| *RPS31* | 1364.942896 | 354.3078216 | 0.259577029 | -1.945765376 | 9.69154E-17 | 2.89603E-15 | K19033 |
| *CSP41A* | 7206.253503 | 2023.952728 | 0.280860606 | -1.83207381 | 1.02634E-16 | 3.06345E-15 | - |
| *At3g47570* | 77.27957138 | 3.329765715 | 0.043087269 | -4.536594516 | 1.11639E-16 | 3.3247E-15 | - |
| *LBD12* | 66.29307986 | 1.643090302 | 0.024785246 | -5.334374604 | 1.15445E-16 | 3.43416E-15 | - |
| *uncharacterized protein_08237* | 193.9979165 | 29.31046281 | 0.151086482 | -2.726553505 | 1.16326E-16 | 3.45646E-15 | - |
| *T5AT* | 56.76262097 | 0.657236121 | 0.011578678 | -6.432385626 | 1.326E-16 | 3.9356E-15 | K19861 |
| *UTR4* | 102.6130707 | 8.040092684 | 0.078353495 | -3.673858568 | 1.4943E-16 | 4.42018E-15 | K15277 |
| *GLR3.1* | 75.22099022 | 2.991417865 | 0.039768393 | -4.652233941 | 1.50673E-16 | 4.45195E-15 | K05387 |
| *Prcp* | 68.97998341 | 0.673352966 | 0.00976157 | -6.678671019 | 1.52245E-16 | 4.49337E-15 | K01285 |
| *FTSZ2-1* | 2246.898064 | 616.6631336 | 0.274450872 | -1.86538017 | 1.58799E-16 | 4.67631E-15 | K03531 |
| *PPH* | 381.0212615 | 77.50672464 | 0.203418372 | -2.297478112 | 1.6486E-16 | 4.84937E-15 | - |
| *slr1780* | 1632.688083 | 435.4833798 | 0.266727849 | -1.906559633 | 1.67288E-16 | 4.91533E-15 | - |
| *At1g56130* | 212.8196458 | 33.82090111 | 0.158918135 | -2.653644331 | 1.72584E-16 | 5.05964E-15 | - |
| *NRT3.1* | 62.44947343 | 1.317715504 | 0.021100506 | -5.566578577 | 1.85327E-16 | 5.42118E-15 | - |
| *uncharacterized protein_04754* | 112.0539777 | 10.07779059 | 0.089936929 | -3.474942574 | 2.06306E-16 | 6.02148E-15 | - |
| *CYP76T24* | 131.7446425 | 13.89237202 | 0.105449237 | -3.245379439 | 2.09354E-16 | 6.09694E-15 | - |
| *UGT85A1* | 720.1319328 | 90.14544422 | 0.125179068 | -2.997934754 | 2.15413E-16 | 6.25956E-15 | - |
| *At5g56590* | 485.6609297 | 110.4643193 | 0.227451525 | -2.136368985 | 2.25613E-16 | 6.54873E-15 | - |
| *O10* | 312.3953595 | 61.37241138 | 0.1964575 | -2.347710849 | 2.35938E-16 | 6.84089E-15 | - |
| *UNC* | 1917.12373 | 249.7934818 | 0.130295963 | -2.940135714 | 2.51154E-16 | 7.2741E-15 | - |
| *CYP750A1* | 54.39974454 | 0 | 0 | -Inf | 2.65701E-16 | 7.68695E-15 | - |
| *PAP15* | 1313.60553 | 349.5348651 | 0.26608815 | -1.910023833 | 2.83538E-16 | 8.19401E-15 | - |
| *CAO* | 714.1653147 | 175.0149582 | 0.245062249 | -2.028779835 | 2.90281E-16 | 8.37967E-15 | K12271 |
| *ACP1* | 572.5020757 | 133.5551452 | 0.233283251 | -2.099845368 | 2.92987E-16 | 8.43931E-15 | - |
| *At1g32780* | 634.609626 | 152.1449899 | 0.239745796 | -2.060422578 | 3.05908E-16 | 8.80188E-15 | K00001 |
| *HSP22* | 131.7766216 | 14.33533016 | 0.108785079 | -3.200447409 | 3.12819E-16 | 8.99091E-15 | K13993 |
| *uncharacterized protein_24642* | 79.21578187 | 4.075594752 | 0.051449278 | -4.280705369 | 3.2553E-16 | 9.34604E-15 | - |
| *HPR-A* | 10455.98862 | 2096.742235 | 0.200530271 | -2.318108058 | 3.34921E-16 | 9.6052E-15 | K15893 |
| *At5g09300* | 371.0337735 | 43.37667149 | 0.116907609 | -3.096559258 | 3.86028E-16 | 1.10229E-14 | K00166 |
| *RPL27* | 3890.488788 | 1100.659251 | 0.282910275 | -1.821583522 | 3.87002E-16 | 1.10388E-14 | K02899 |
| *A6* | 166.0000947 | 23.03265897 | 0.138750878 | -2.849431189 | 3.91228E-16 | 1.11378E-14 | - |
| *FUC1* | 228.9640852 | 39.11588866 | 0.170838534 | -2.549294671 | 3.91319E-16 | 1.11378E-14 | K01206 |
| *CYP76C4* | 58.3637336 | 1.018087872 | 0.017443844 | -5.841138185 | 4.02333E-16 | 1.14389E-14 | K05280 |
| *TOGT1* | 55.12763687 | 0.660479384 | 0.011980912 | -6.38311842 | 4.03741E-16 | 1.14666E-14 | - |
| *ag4* | 47.08922669 | 0 | 0 | -Inf | 4.20113E-16 | 1.19187E-14 | - |
| *SOQ1* | 987.8872291 | 257.2365763 | 0.260390628 | -1.941250569 | 4.24902E-16 | 1.20287E-14 | - |
| *ZAR1* | 138.386413 | 12.93388663 | 0.093462113 | -3.419474529 | 4.25525E-16 | 1.20334E-14 | - |
| *At1g68400* | 241.8040888 | 43.41042735 | 0.179527268 | -2.47772511 | 4.42345E-16 | 1.24956E-14 | - |
| *PSAK* | 13048.98402 | 1763.169414 | 0.135119287 | -2.887694477 | 4.44345E-16 | 1.25387E-14 | K02698 |
| *ag4* | 46.77683379 | 0 | 0 | -Inf | 4.64949E-16 | 1.31061E-14 | K18108 |
| *HOMT1* | 63.41897503 | 1.018087872 | 0.016053364 | -5.960980569 | 5.51269E-16 | 1.55061E-14 | K13066 |
| *uncharacterized protein_39336* | 3315.557698 | 942.2398516 | 0.284187439 | -1.815085305 | 5.91985E-16 | 1.66159E-14 | - |
| *At4g17486* | 77.65310173 | 4.014370632 | 0.051696205 | -4.273797825 | 5.94857E-16 | 1.66788E-14 | - |
| *At3g15890* | 253.213962 | 46.41005284 | 0.183283941 | -2.447847711 | 6.0753E-16 | 1.7016E-14 | - |
| *PM19L* | 141.7852378 | 17.26228064 | 0.121749492 | -3.038012345 | 6.67143E-16 | 1.86263E-14 | - |
| *CSLH1* | 914.7087205 | 238.0143474 | 0.260207804 | -1.942263864 | 6.97699E-16 | 1.94588E-14 | - |
| *CPK17* | 452.6346126 | 102.0441142 | 0.225444788 | -2.149153939 | 7.09093E-16 | 1.97557E-14 | K13412 |
| *At4g25835* | 132.5033374 | 15.26158185 | 0.115178849 | -3.118052288 | 7.76427E-16 | 2.1586E-14 | K08900 |
| *RAM2* | 50.3448355 | 0.330239692 | 0.006559554 | -7.252186451 | 7.78662E-16 | 2.16253E-14 | K13508 |
| *FEY* | 56.87956277 | 0.985854181 | 0.017332309 | -5.850392294 | 8.36625E-16 | 2.32106E-14 | K11153 |
| *MTERF8* | 256.1016259 | 42.60347366 | 0.16635378 | -2.587673442 | 8.53979E-16 | 2.36672E-14 | - |
| *PHO1-3* | 262.90572 | 49.85233162 | 0.189620567 | -2.398812644 | 9.62146E-16 | 2.65811E-14 | - |
| *chi1* | 101.1319405 | 8.782678458 | 0.086843765 | -3.525433919 | 1.03996E-15 | 2.87006E-14 | K20547 |
| *CYP75A5* | 147.4928747 | 19.14570094 | 0.129807633 | -2.945552876 | 1.19393E-15 | 3.29154E-14 | K13083 |
| *Os04g0590900* | 911.0490943 | 241.3296264 | 0.264892011 | -1.916523763 | 1.20884E-15 | 3.32917E-14 | - |
| *BOR2* | 81.72931937 | 5.04857535 | 0.0617719 | -4.016905482 | 1.22638E-15 | 3.37042E-14 | - |
| *GLO1* | 21945.54684 | 6523.953221 | 0.297279137 | -1.750109877 | 1.23032E-15 | 3.37774E-14 | K11517 |
| *CYCD2-2* | 55.92274802 | 1.001971027 | 0.017917056 | -5.802522559 | 1.34036E-15 | 3.67269E-14 | K18810 |
| *LYM1* | 224.1914541 | 39.92617864 | 0.178089655 | -2.489324383 | 1.34054E-15 | 3.67269E-14 | - |
| *POPTR_0001s40980g* | 1248.299852 | 342.3432873 | 0.274247639 | -1.866448894 | 1.35862E-15 | 3.71835E-14 | K16054 |
| *LTPG5* | 61.65448627 | 1.651198459 | 0.026781481 | -5.222620441 | 1.39788E-15 | 3.82185E-14 | - |
| *LAC11* | 169.8270732 | 9.869999139 | 0.058117937 | -4.104872697 | 1.52653E-15 | 4.15204E-14 | K05909 |
| *MDHP* | 7014.622991 | 1553.8698 | 0.221518648 | -2.174499942 | 1.58894E-15 | 4.29961E-14 | K00051 |
| *At5g10770* | 52.78430074 | 0.689469812 | 0.013062024 | -6.258477707 | 1.68116E-15 | 4.53054E-14 | - |
| *PBL8* | 144.2915455 | 7.03964382 | 0.048787639 | -4.357340525 | 1.76876E-15 | 4.76174E-14 | - |
| *GLCAT14B* | 163.7565483 | 24.12322291 | 0.147311501 | -2.763058026 | 1.80158E-15 | 4.84515E-14 | - |
| *SBE1* | 804.9535806 | 160.6433425 | 0.199568455 | -2.325044397 | 1.88673E-15 | 5.06382E-14 | K00700 |
| *BXL4* | 255.7256986 | 12.72933845 | 0.049777314 | -4.3283678 | 1.97948E-15 | 5.29656E-14 | K15920 |
| *uncharacterized protein_48389* | 2373.517149 | 686.5550653 | 0.289256417 | -1.789579132 | 2.0422E-15 | 5.45332E-14 | - |
| *SMXL3* | 193.8231259 | 32.20680124 | 0.166165936 | -2.58930343 | 2.08013E-15 | 5.54899E-14 | - |
| *NEK5* | 104.440189 | 9.681461885 | 0.092698625 | -3.431308255 | 2.18186E-15 | 5.81209E-14 | K08857 |
| *uncharacterized protein_47349* | 177.8185813 | 28.35187796 | 0.159442718 | -2.648889884 | 2.18317E-15 | 5.81209E-14 | - |
| *NUP133* | 834.429596 | 223.0616084 | 0.267322264 | -1.903348099 | 2.20952E-15 | 5.8763E-14 | - |
| *uncharacterized protein_14385* | 306.0219639 | 63.20373408 | 0.206533326 | -2.275553502 | 2.26874E-15 | 6.02772E-14 | - |
| *uncharacterized protein_35297* | 254.046453 | 49.04720495 | 0.193063923 | -2.372849492 | 2.54559E-15 | 6.74287E-14 | K15032 |
| *uncharacterized protein_00298* | 1051.978207 | 288.4755732 | 0.274222005 | -1.86658375 | 2.91981E-15 | 7.70315E-14 | - |
| *TAT* | 51.65436792 | 0.658857752 | 0.012755122 | -6.292779519 | 2.92551E-15 | 7.71048E-14 | K15400 |
| *uncharacterized protein_00813* | 5156.757016 | 1532.182178 | 0.297121267 | -1.750876222 | 2.92977E-15 | 7.71244E-14 | K06995 |
| *TET8* | 54.47229647 | 0.989097444 | 0.018157807 | -5.783266221 | 3.07994E-15 | 8.08516E-14 | - |
| *Os05g0361200* | 728.2292118 | 189.4081698 | 0.260094166 | -1.942894053 | 3.09423E-15 | 8.11458E-14 | K01772 |
| *ROC1* | 3694.651088 | 1040.559902 | 0.281639559 | -1.828078108 | 3.11502E-15 | 8.15289E-14 | K11294 |
| *At2g34460* | 1347.285928 | 376.5814667 | 0.27951117 | -1.839022155 | 3.18543E-15 | 8.3289E-14 | - |
| *SLC1* | 43.98452691 | 0 | 0 | -Inf | 3.21934E-15 | 8.40921E-14 | - |
| *SPAC24B11.05* | 334.2534214 | 72.97724397 | 0.218329086 | -2.195423755 | 3.30605E-15 | 8.62715E-14 | K07025 |
| *H1* | 642.9003356 | 165.879841 | 0.258017972 | -1.954456534 | 3.34527E-15 | 8.72087E-14 | K11275 |
| *At4g31140* | 48.41954357 | 0.344734906 | 0.007119747 | -7.133958284 | 3.40716E-15 | 8.87342E-14 | - |
| *PCBER* | 214.7964334 | 1.001971027 | 0.004664747 | -7.743985437 | 3.41411E-15 | 8.88275E-14 | - |
| *TBL36* | 43.79072013 | 0 | 0 | -Inf | 3.4327E-15 | 8.92231E-14 | - |
| *ROQ1* | 409.6014955 | 95.29600274 | 0.232655407 | -2.103733377 | 3.6348E-15 | 9.41048E-14 | - |
| *CBL2* | 174.2263261 | 27.48045689 | 0.157728499 | -2.66448474 | 3.66832E-15 | 9.48794E-14 | K06268 |
| *SDR2a* | 531.0329786 | 130.1862678 | 0.245156653 | -2.02822418 | 3.70012E-15 | 9.55144E-14 | - |
| *At1g61300* | 110.3034764 | 11.32941708 | 0.102711333 | -3.283332722 | 3.74448E-15 | 9.6565E-14 | K13459 |
| *chi1* | 76.09814727 | 4.43320324 | 0.058256389 | -4.10143992 | 3.90748E-15 | 1.0067E-13 | K20547 |
| *Os06g0254200* | 295.1142296 | 60.69074487 | 0.205651706 | -2.281725053 | 3.92706E-15 | 1.01076E-13 | - |
| *ABCB19* | 133.5890325 | 9.660579614 | 0.072315664 | -3.789548009 | 4.01424E-15 | 1.03219E-13 | K05658 |
| *At1g74460* | 44.33203544 | 0 | 0 | -Inf | 4.06233E-15 | 1.04354E-13 | - |
| *HCT* | 78.11767604 | 4.66836349 | 0.059760655 | -4.064660228 | 4.10963E-15 | 1.05466E-13 | K13065 |
| *At3g51990* | 454.9517029 | 110.0890911 | 0.241979732 | -2.047041881 | 4.94507E-15 | 1.26536E-13 | K04733 |
| *SYNC1* | 2451.11934 | 724.7891004 | 0.29569719 | -1.757807563 | 5.10609E-15 | 1.30403E-13 | K01893 |
| *NAKR2* | 321.4642848 | 69.10597915 | 0.214972494 | -2.217776015 | 5.28038E-15 | 1.34593E-13 | - |
| *MTERF9* | 269.5437386 | 54.40656041 | 0.201846872 | -2.308666867 | 5.41687E-15 | 1.37939E-13 | K15032 |
| *ASOL* | 302.5924323 | 63.8044056 | 0.210859224 | -2.245647958 | 5.43903E-15 | 1.3837E-13 | - |
| *OMT1* | 131.9502356 | 16.74046581 | 0.126869541 | -2.97858235 | 5.44589E-15 | 1.38411E-13 | K13066 |
| *CAR3* | 93.56722666 | 1.646333564 | 0.017595195 | -5.828674717 | 5.54632E-15 | 1.40827E-13 | K12486 |
| *At2g05160* | 67.45067883 | 3.054263617 | 0.045281436 | -4.46493647 | 5.68861E-15 | 1.44301E-13 | - |
| *Os01g0323300* | 1398.768642 | 397.4496248 | 0.284142504 | -1.815313438 | 5.71716E-15 | 1.44886E-13 | - |
| *PTC52* | 738.2883556 | 195.5843082 | 0.264915878 | -1.916393777 | 5.97662E-15 | 1.51171E-13 | - |
| *At2g24130* | 43.47947563 | 0 | 0 | -Inf | 6.01914E-15 | 1.521E-13 | - |
| *IQD18* | 465.8650138 | 48.35591456 | 0.103798124 | -3.268147723 | 6.3777E-15 | 1.60852E-13 | - |
| *At3g02645* | 1075.770144 | 301.6025506 | 0.280359659 | -1.83464932 | 6.47745E-15 | 1.63212E-13 | - |
| *CYP704B1* | 239.0480742 | 46.42769185 | 0.194219058 | -2.364243319 | 7.11946E-15 | 1.78876E-13 | - |
| *uncharacterized protein_24444* | 222.5513566 | 41.33430763 | 0.1857293 | -2.428726669 | 7.29416E-15 | 1.8309E-13 | - |
| *uncharacterized protein_20118* | 919.6195738 | 252.3344624 | 0.274390052 | -1.865699919 | 7.4363E-15 | 1.86303E-13 | - |
| *DCOR* | 215.907598 | 9.328718291 | 0.043206994 | -4.532591327 | 7.4601E-15 | 1.86722E-13 | K01581 |
| *PAP27* | 145.1078174 | 20.14118544 | 0.138801519 | -2.848904741 | 7.54555E-15 | 1.88682E-13 | - |
| *uncharacterized protein_13825* | 68.06808266 | 1.678567256 | 0.024660122 | -5.341676224 | 7.90377E-15 | 1.97452E-13 | - |
| *HCT* | 205.5155813 | 36.59641292 | 0.178071233 | -2.489473625 | 7.9872E-15 | 1.99347E-13 | K19747 |
| *COL16* | 454.5505107 | 18.64932842 | 0.041028066 | -4.607245035 | 8.99338E-15 | 2.24036E-13 | - |
| *At1g74460* | 60.26504819 | 2.003942053 | 0.033252144 | -4.910408831 | 9.29604E-15 | 2.31356E-13 | - |
| *FBP* | 682.1215211 | 179.8821964 | 0.263709897 | -1.922976376 | 9.94154E-15 | 2.4649E-13 | K03841 |
| *At2g26730* | 728.08918 | 172.4006278 | 0.236785043 | -2.078350142 | 9.99692E-15 | 2.47631E-13 | - |
| *UGT85K4* | 615.8911034 | 101.6022868 | 0.16496794 | -2.599742415 | 1.0065E-14 | 2.49082E-13 | - |
| *uncharacterized protein_30656* | 72.46748777 | 4.044982692 | 0.055817896 | -4.163128447 | 1.01262E-14 | 2.50362E-13 | - |
| *At1g06840* | 116.4247205 | 13.42205152 | 0.115285237 | -3.116720313 | 1.01769E-14 | 2.5138E-13 | - |
| *MYB4* | 72.30259129 | 4.046604323 | 0.055967625 | -4.159263659 | 1.0457E-14 | 2.58023E-13 | K09422 |
| *ISE2* | 619.6526834 | 163.1617998 | 0.263311697 | -1.925156482 | 1.04654E-14 | 2.58023E-13 | - |
| *WAK3* | 193.233236 | 33.94152234 | 0.17565054 | -2.50922008 | 1.26309E-14 | 3.10542E-13 | - |
| *SYM1* | 4615.664876 | 1417.861374 | 0.307184644 | -1.702821995 | 1.33272E-14 | 3.2705E-13 | K13348 |
| *At5g46580* | 793.8784572 | 217.6438678 | 0.274152631 | -1.866948776 | 1.42864E-14 | 3.49937E-13 | - |
| *CKX3* | 84.09942556 | 6.375921174 | 0.075814087 | -3.721390248 | 1.43796E-14 | 3.51893E-13 | K00279 |
| *PT5* | 52.37869543 | 0.985854181 | 0.018821664 | -5.731462046 | 1.45851E-14 | 3.56592E-13 | K12742 |
| *UGT86A1* | 477.2860297 | 118.7021688 | 0.248702374 | -2.007507814 | 1.47723E-14 | 3.60501E-13 | - |
| *uncharacterized protein_22018* | 1251.133637 | 358.9082691 | 0.286866453 | -1.801548828 | 1.47724E-14 | 3.60501E-13 | - |
| *At1g06840* | 182.3110466 | 31.23057737 | 0.171303813 | -2.545370834 | 1.49574E-14 | 3.64679E-13 | - |
| *NDL2* | 307.331368 | 68.42442499 | 0.222640551 | -2.167211713 | 1.54057E-14 | 3.75261E-13 | K18266 |
| *CEP1* | 190.6275105 | 30.55550331 | 0.160289054 | -2.641252187 | 1.56457E-14 | 3.80754E-13 | K16292 |
| *PSRP2* | 1089.812428 | 311.077432 | 0.285441259 | -1.808734209 | 1.71803E-14 | 4.17715E-13 | K13126 |
| *PRS1* | 333.8121763 | 75.11638905 | 0.225025911 | -2.151836963 | 1.73452E-14 | 4.21336E-13 | K00948 |
| *PCAP1* | 111.9488505 | 0 | 0 | -Inf | 1.84947E-14 | 4.48019E-13 | - |
| *uncharacterized protein_27219* | 413.0901157 | 101.0259269 | 0.244561472 | -2.031730952 | 2.03447E-14 | 4.9193E-13 | - |
| *R* | 8806.681094 | 2734.568636 | 0.310510691 | -1.687285151 | 2.03782E-14 | 4.92291E-13 | K01783 |
| *At3g21360* | 524.9356399 | 136.528539 | 0.260086244 | -1.942937997 | 2.06102E-14 | 4.96983E-13 | - |
| *CRK2* | 51.4059052 | 0.985854181 | 0.019177839 | -5.704416015 | 2.12932E-14 | 5.12516E-13 | - |
| *uncharacterized protein_07790* | 3893.568831 | 1222.099653 | 0.313876473 | -1.6717312 | 2.24856E-14 | 5.40722E-13 | K20283 |
| *PHOS34* | 133.92159 | 18.35466516 | 0.137055311 | -2.867169861 | 2.2814E-14 | 5.48118E-13 | - |
| *At1g32780* | 73.73272608 | 0 | 0 | -Inf | 2.29411E-14 | 5.5067E-13 | K00001 |
| *uncharacterized protein_09975* | 2607.546455 | 578.8580116 | 0.221993365 | -2.171411538 | 2.30705E-14 | 5.53273E-13 | - |
| *NPF5.1* | 44.99764192 | 0.344734906 | 0.007661177 | -7.028218204 | 2.4137E-14 | 5.78322E-13 | - |
| *MDAR5* | 369.7342155 | 89.17956629 | 0.241199117 | -2.05170347 | 2.4672E-14 | 5.90605E-13 | K08232 |
| *BHLH25* | 55.91170549 | 1.678567256 | 0.03002175 | -5.057848104 | 2.56558E-14 | 6.13041E-13 | - |
| *GLNA1* | 276.3771192 | 59.0183593 | 0.213542856 | -2.227402463 | 2.77005E-14 | 6.61298E-13 | K01915 |
| *GSTU19* | 1739.859731 | 8.629518691 | 0.004959893 | -7.655475189 | 2.93109E-14 | 6.9848E-13 | K00799 |
| *PMIR2* | 238.9048626 | 47.93555979 | 0.200647066 | -2.317268033 | 3.05054E-14 | 7.25633E-13 | - |
| *ROQ1* | 64.95299283 | 3.088118939 | 0.047543905 | -4.394595778 | 3.24133E-14 | 7.68933E-13 | - |
| *uncharacterized protein_42412* | 14761.99541 | 4747.906456 | 0.321630398 | -1.63652433 | 3.71836E-14 | 8.78933E-13 | - |
| *uncharacterized protein_36021* | 94.97213995 | 9.302971126 | 0.097954738 | -3.351740907 | 3.76304E-14 | 8.88698E-13 | - |
| *SBT1.7* | 1370.98219 | 373.2040962 | 0.27221659 | -1.877173104 | 3.77641E-14 | 8.91057E-13 | - |
| *At1g73050* | 214.2822557 | 41.5565943 | 0.193933903 | -2.366363058 | 3.84556E-14 | 9.0656E-13 | - |
| *APS1* | 392.5341129 | 81.25280819 | 0.206995534 | -2.272328456 | 3.91724E-14 | 9.22633E-13 | - |
| *ATPD* | 6718.661653 | 1577.664611 | 0.234818286 | -2.090383338 | 3.96506E-14 | 9.33064E-13 | K02113 |
| *RSH3* | 331.7518225 | 77.26355571 | 0.232895648 | -2.102244413 | 4.26918E-14 | 1.00284E-12 | - |
| *HOX32* | 100.5788388 | 10.76401714 | 0.107020694 | -3.224038304 | 4.33489E-14 | 1.01737E-12 | K09338 |
| *ufaA1* | 224.9316331 | 44.95033005 | 0.199839967 | -2.323082954 | 4.49106E-14 | 1.05215E-12 | K00574 |
| *NIFU1* | 3228.951908 | 1012.389897 | 0.313535143 | -1.673300938 | 4.9958E-14 | 1.16728E-12 | - |
| *ag1* | 67.6763126 | 3.680887677 | 0.054389602 | -4.200525333 | 5.51521E-14 | 1.28409E-12 | K15086 |
| *PCMP-H42* | 421.9964412 | 106.3888304 | 0.252108359 | -1.98788414 | 5.87397E-14 | 1.364E-12 | - |
| *uncharacterized protein_41388* | 1227.290753 | 364.7679173 | 0.297213938 | -1.750426323 | 6.05534E-14 | 1.40365E-12 | - |
| *PLR_Tp2* | 43.5021806 | 0.330239692 | 0.007591337 | -7.04143038 | 6.09536E-14 | 1.41168E-12 | - |
| *TBL26* | 235.8169104 | 49.19540035 | 0.208616932 | -2.261071839 | 6.52889E-14 | 1.50811E-12 | - |
| *DXR* | 1793.635845 | 551.5320699 | 0.307493894 | -1.701370333 | 6.56789E-14 | 1.51579E-12 | K00099 |
| *CYP71AU50* | 66.29676866 | 3.647032355 | 0.055010711 | -4.184143651 | 6.66842E-14 | 1.53765E-12 | - |
| *LSMT-L* | 2161.100196 | 666.8574199 | 0.308573115 | -1.696315724 | 6.77654E-14 | 1.55985E-12 | K00592 |
| *UGT73C10* | 39.98033705 | 0 | 0 | -Inf | 6.90648E-14 | 1.58699E-12 | K13496 |
| *At5g48740* | 39.81544057 | 0 | 0 | -Inf | 7.29707E-14 | 1.67528E-12 | - |
| *SCPL31* | 54.07633733 | 1.665693673 | 0.030802635 | -5.020802426 | 7.76327E-14 | 1.77612E-12 | K16297 |
| *FRO6* | 4187.782657 | 1341.675057 | 0.320378388 | -1.642151265 | 8.02994E-14 | 1.83553E-12 | - |
| *TBL6* | 179.2167651 | 13.12394605 | 0.073229455 | -3.771432129 | 8.61121E-14 | 1.9633E-12 | - |
| *SUS3* | 231.0948463 | 48.35936321 | 0.209261972 | -2.256617934 | 9.04151E-14 | 2.05962E-12 | K00695 |
| *TBR* | 40.38466998 | 0 | 0 | -Inf | 9.29626E-14 | 2.11582E-12 | - |
| *BAM3* | 65.20526833 | 0.673352966 | 0.010326665 | -6.597481771 | 9.7313E-14 | 2.21102E-12 | K01177 |
| *SK1* | 409.4172109 | 103.6387545 | 0.253137269 | -1.982008168 | 9.95296E-14 | 2.25943E-12 | K00891 |
| *IN37* | 5439.664707 | 1757.297908 | 0.323052615 | -1.630158942 | 1.00818E-13 | 2.2867E-12 | K12502 |
| *At1g56130* | 188.2089537 | 34.61020935 | 0.18389247 | -2.44306569 | 1.05137E-13 | 2.38058E-12 | - |
| *SULTR3;1* | 1245.667801 | 270.6089456 | 0.217240058 | -2.20263794 | 1.06667E-13 | 2.41315E-12 | K17471 |
| *ROQ1* | 784.3197687 | 226.6506792 | 0.288977389 | -1.790971479 | 1.08988E-13 | 2.46353E-12 | - |
| *CYCU2-1* | 225.2615219 | 46.53888813 | 0.206599368 | -2.275092251 | 1.12607E-13 | 2.54097E-12 | - |
| *LRK10L-1.1* | 262.8166966 | 58.00210487 | 0.22069414 | -2.179879771 | 1.17704E-13 | 2.65146E-12 | - |
| *At5g48740* | 210.1763557 | 36.17728191 | 0.17212822 | -2.538444455 | 1.18351E-13 | 2.66375E-12 | - |
| *uncharacterized protein_21490* | 303.5038654 | 70.23536277 | 0.231415052 | -2.11144539 | 1.22138E-13 | 2.7443E-12 | - |
| *A6* | 56.01382019 | 2.024923793 | 0.036150432 | -4.789843306 | 1.25241E-13 | 2.80924E-12 | - |
| *uncharacterized protein_05993* | 98.87840417 | 10.88960273 | 0.110131255 | -3.182704136 | 1.28211E-13 | 2.87342E-12 | - |
| *ag4* | 95.33041483 | 0.658857752 | 0.006911307 | -7.176825744 | 1.28978E-13 | 2.88815E-12 | K16086 |
| *EO* | 1455.030697 | 449.756768 | 0.309104659 | -1.693832693 | 1.31235E-13 | 2.93121E-12 | K18980 |
| *uncharacterized protein_33326* | 1739.212727 | 538.3129256 | 0.309515287 | -1.691917431 | 1.52724E-13 | 3.40252E-12 | - |
| *CIPK26* | 64.93410064 | 3.700247786 | 0.056984662 | -4.133282531 | 1.57698E-13 | 3.51037E-12 | K07198 |
| *CYP76B10* | 84.28572641 | 7.688871252 | 0.091223883 | -3.454444608 | 1.58863E-13 | 3.53334E-12 | - |
| *ABCB14* | 526.8099365 | 145.487279 | 0.276166543 | -1.856389544 | 1.6622E-13 | 3.69074E-12 | K05658 |
| *At5g04720* | 227.3426884 | 29.77105352 | 0.130952325 | -2.932886425 | 1.68439E-13 | 3.73685E-12 | - |
| *GRXS12* | 249.8156375 | 54.12314265 | 0.216652341 | -2.20654627 | 1.75301E-13 | 3.88581E-12 | K03676 |
| *CBP1* | 62.78469527 | 3.3152705 | 0.0528038 | -4.24321443 | 1.76497E-13 | 3.90903E-12 | K16296 |
| *CSLA9* | 157.3298887 | 0.32861806 | 0.00208872 | -8.903165287 | 1.77066E-13 | 3.91836E-12 | K13680 |
| *MP3* | 690.9093845 | 196.4580407 | 0.284347043 | -1.814275292 | 1.85422E-13 | 4.08953E-12 | K17278 |
| *GSTL3* | 964.468075 | 288.0598715 | 0.298672272 | -1.743364786 | 1.86839E-13 | 4.11734E-12 | K00799 |
| *LTPG5* | 107.7766039 | 0.657236121 | 0.006098134 | -7.357416547 | 1.99856E-13 | 4.40051E-12 | - |
| *SCPL45* | 804.8220116 | 238.4969468 | 0.29633502 | -1.754698966 | 2.12561E-13 | 4.66363E-12 | K16297 |
| *CYP720B2* | 140.4587337 | 11.37128109 | 0.080958163 | -3.62667963 | 2.1342E-13 | 4.67572E-12 | - |
| *NIP3-1* | 73.53324246 | 5.375571779 | 0.073103968 | -3.77390648 | 2.16143E-13 | 4.73144E-12 | K09874 |
| *PARC* | 41.62837995 | 0.344734906 | 0.008281247 | -6.915936219 | 2.17192E-13 | 4.75047E-12 | K00799 |
| *ATJ8* | 118.028902 | 15.96726798 | 0.135282695 | -2.885950787 | 2.21615E-13 | 4.84318E-12 | - |
| *ATI1* | 3348.124397 | 1085.755537 | 0.324287693 | -1.624653822 | 2.28855E-13 | 4.99312E-12 | - |
| *At3g03770* | 375.6609765 | 95.60383156 | 0.254494977 | -1.974290913 | 2.29297E-13 | 4.99864E-12 | - |
| *ROC2* | 252.0311415 | 56.1899369 | 0.222948389 | -2.165218322 | 2.32773E-13 | 5.06603E-12 | K09338 |
| *SecA* | 2312.718944 | 740.5549833 | 0.320209676 | -1.642911192 | 2.52724E-13 | 5.47687E-12 | K03070 |
| *GLR2.7* | 111.4058953 | 14.23872856 | 0.127809471 | -2.967933346 | 2.5585E-13 | 5.53627E-12 | K05387 |
| *guaD* | 812.4137522 | 239.0365931 | 0.294230117 | -1.76498317 | 2.5655E-13 | 5.54686E-12 | K01487 |
| *GSTU20* | 257.3936668 | 0 | 0 | -Inf | 2.63469E-13 | 5.68713E-12 | K00799 |
| *NPF5.6* | 41.33031822 | 0.344734906 | 0.008340969 | -6.905569276 | 2.67675E-13 | 5.77319E-12 | K14638 |
| *CSPL5* | 94.22926832 | 1.335453981 | 0.01417239 | -6.140773074 | 2.70378E-13 | 5.82673E-12 | - |
| *BPA1* | 835.0898499 | 233.7608811 | 0.27992303 | -1.836897909 | 2.77222E-13 | 5.96933E-12 | - |
| *TPS1* | 333.5587806 | 81.1038843 | 0.243147202 | -2.040098103 | 2.78359E-13 | 5.98403E-12 | K16055 |
| *B&apos;GAMMA* | 50.04323718 | 1.332210718 | 0.026621194 | -5.231280918 | 2.79375E-13 | 6.00099E-12 | K11584 |
| *GSTU20* | 681.1721971 | 1.348327564 | 0.001979422 | -8.980704712 | 3.14411E-13 | 6.74807E-12 | K00799 |
| *IAN9* | 237.8183234 | 52.04500987 | 0.218843566 | -2.192028128 | 3.19634E-13 | 6.85458E-12 | - |
| *At3g21360* | 535.0451941 | 150.8029563 | 0.281850875 | -1.826996046 | 3.3063E-13 | 7.07312E-12 | - |
| *BOR2* | 58.03536522 | 2.635431008 | 0.045410777 | -4.460821478 | 3.37153E-13 | 7.20681E-12 | - |
| *HCF173* | 770.2800969 | 226.8155798 | 0.294458575 | -1.763863407 | 3.55241E-13 | 7.58731E-12 | - |
| *ACR3* | 644.917076 | 184.7283361 | 0.286437347 | -1.803708486 | 3.77162E-13 | 8.03596E-12 | - |
| *UTR4* | 168.8836935 | 30.90509667 | 0.182996333 | -2.450113353 | 3.79017E-13 | 8.06897E-12 | K15277 |
| *mutS2* | 186.838808 | 36.39348637 | 0.194785477 | -2.360041978 | 3.82312E-13 | 8.13254E-12 | K07456 |
| *ABCB11* | 1124.331594 | 350.7201191 | 0.311936551 | -1.680675487 | 3.91647E-13 | 8.3244E-12 | K05658 |
| *R42* | 287.3656509 | 1.659207147 | 0.005773853 | -7.436249801 | 3.96597E-13 | 8.42282E-12 | K00430 |
| *VIT_19s0014g02480* | 627.9557841 | 179.5572195 | 0.285939272 | -1.806219318 | 4.12774E-13 | 8.75227E-12 | K16054 |
| *uncharacterized protein_29881* | 748.4110644 | 219.0385199 | 0.292671408 | -1.772646284 | 4.18278E-13 | 8.85473E-12 | - |
| *VTE4* | 603.5781992 | 172.6241189 | 0.286001249 | -1.805906647 | 4.20616E-13 | 8.89708E-12 | K05928 |
| *UGT86A1* | 276.2840308 | 64.53736105 | 0.233590631 | -2.097945685 | 4.27737E-13 | 9.04044E-12 | - |
| *HOX32* | 253.4390673 | 58.28399403 | 0.229972414 | -2.120467281 | 4.47163E-13 | 9.40578E-12 | K09338 |
| *DEGP1* | 412.0133228 | 108.4989915 | 0.263338551 | -1.925009356 | 4.65396E-13 | 9.76032E-12 | - |
| *RNP1* | 165.2563509 | 30.21704955 | 0.182849551 | -2.451271011 | 4.655E-13 | 9.76032E-12 | K14411 |
| *PCR3* | 53.18345275 | 2.005563685 | 0.037710295 | -4.728897759 | 4.75122E-13 | 9.95414E-12 | - |
| *IAN9* | 238.4464936 | 52.96001605 | 0.222104403 | -2.170690103 | 4.94568E-13 | 1.03451E-11 | - |
| *At5g37690* | 167.6714386 | 30.76815965 | 0.183502688 | -2.4461269 | 5.25004E-13 | 1.09643E-11 | - |
| *At5g09300* | 142.0433467 | 23.80109137 | 0.16756217 | -2.577231624 | 5.84515E-13 | 1.21686E-11 | K00166 |
| *GAT1* | 36.42868278 | 0 | 0 | -Inf | 5.90018E-13 | 1.22735E-11 | - |
| *NRT3.2* | 36.73307382 | 0 | 0 | -Inf | 5.97795E-13 | 1.24255E-11 | - |
| *LTPG15* | 36.68018617 | 0 | 0 | -Inf | 6.08691E-13 | 1.2642E-11 | - |
| *At2g20020* | 591.203718 | 171.2080678 | 0.289592339 | -1.787904656 | 6.20598E-13 | 1.28791E-11 | - |
| *uncharacterized protein_41274* | 1244.890037 | 397.0977285 | 0.318982172 | -1.648452299 | 6.27828E-13 | 1.30189E-11 | K09955 |
| *AAP2* | 104.243994 | 8.358980956 | 0.080186691 | -3.640493382 | 6.60982E-13 | 1.36956E-11 | - |
| *rlmL* | 237.7587063 | 52.81333637 | 0.222129979 | -2.170523983 | 6.77201E-13 | 1.40207E-11 | - |
| *IMPL1* | 613.4132688 | 177.9274199 | 0.290061251 | -1.785570516 | 6.96447E-13 | 1.43852E-11 | K01092 |
| *PCS1* | 203.1514325 | 42.1442992 | 0.207452631 | -2.269146139 | 7.1121E-13 | 1.46787E-11 | - |
| *VIPP1* | 2357.674032 | 773.7060667 | 0.328164986 | -1.607506776 | 7.26462E-13 | 1.49583E-11 | K03969 |
| *GGAT1* | 5571.4212 | 1870.03978 | 0.33564861 | -1.574976428 | 7.7148E-13 | 1.58605E-11 | K14272 |
| *UGT85A8* | 113.1145515 | 15.72552174 | 0.139022977 | -2.846604747 | 8.36429E-13 | 1.71823E-11 | - |
| *BGLU13* | 39.91311824 | 0.32861806 | 0.008233335 | -6.924307414 | 8.42181E-13 | 1.7287E-11 | K01188 |
| *Fra a 1.06* | 72.98963471 | 0.660479384 | 0.009048948 | -6.788034261 | 8.999E-13 | 1.84287E-11 | - |
| *uncharacterized protein_06770* | 163.0890495 | 30.39929921 | 0.186396937 | -2.423549945 | 9.47501E-13 | 1.93885E-11 | - |
| *EO* | 539.0614946 | 123.9912602 | 0.230013201 | -2.120211429 | 9.6786E-13 | 1.97897E-11 | K18980 |
| *GSTU20* | 345.9950799 | 1.33383235 | 0.003855062 | -8.019030368 | 9.72348E-13 | 1.98661E-11 | K00799 |
| *uncharacterized protein_49231* | 116.844657 | 17.23988265 | 0.147545323 | -2.760769905 | 9.82753E-13 | 2.00631E-11 | - |
| *GRF3* | 209.6318757 | 44.92650292 | 0.21431141 | -2.222219432 | 9.89214E-13 | 2.01793E-11 | - |
| *At2g42960* | 133.6894747 | 22.07093032 | 0.165091009 | -2.59866654 | 1.0786E-12 | 2.19518E-11 | - |
| *PAE8* | 329.8165406 | 84.0113752 | 0.254721534 | -1.973007165 | 1.10009E-12 | 2.23719E-11 | K19882 |
| *EMB2761* | 557.3617251 | 160.3150293 | 0.287631931 | -1.797704252 | 1.12567E-12 | 2.28744E-11 | K01868 |
| *At1g31830* | 99.07776816 | 0 | 0 | -Inf | 1.13346E-12 | 2.30149E-11 | - |
| *At3g47110* | 58.05121672 | 3.012399605 | 0.051892101 | -4.268341239 | 1.14E-12 | 2.31299E-11 | - |
| *DCOR* | 165.6019628 | 8.424970501 | 0.050874823 | -4.296904327 | 1.20792E-12 | 2.44704E-11 | K01581 |
| *MLO6* | 47.03022947 | 1.314472241 | 0.027949518 | -5.161032791 | 1.21769E-12 | 2.46305E-11 | K08472 |
| *PCBER* | 721.6042933 | 17.25913685 | 0.02391773 | -5.385775702 | 1.22665E-12 | 2.47926E-11 | - |
| *CA1P* | 552.1319736 | 109.9151357 | 0.199074027 | -2.328623085 | 1.24302E-12 | 2.51043E-11 | K15634 |
| *uncharacterized protein_47676* | 866.1145822 | 244.494146 | 0.282288454 | -1.824757975 | 1.25857E-12 | 2.53989E-11 | - |
| *CRSP* | 47.70991916 | 0 | 0 | -Inf | 1.31109E-12 | 2.64183E-11 | - |
| *BIC1* | 230.6150448 | 51.75024069 | 0.224400974 | -2.155849156 | 1.31951E-12 | 2.65677E-11 | - |
| *pds* | 560.0737544 | 161.4409578 | 0.288249461 | -1.794610185 | 1.35252E-12 | 2.71906E-11 | - |
| *PCMP-H40* | 253.9514723 | 59.77716139 | 0.235388127 | -2.086886543 | 1.40138E-12 | 2.81515E-11 | - |
| *PCMP-H40* | 159.5564396 | 30.01756519 | 0.18813133 | -2.41018797 | 1.44037E-12 | 2.88687E-11 | - |
| *RPL5* | 4257.342782 | 1426.231259 | 0.335005033 | -1.577745324 | 1.52989E-12 | 3.06163E-11 | K02931 |
| *UGT85K4* | 163.6964352 | 30.91634862 | 0.188863909 | -2.40458106 | 1.55997E-12 | 3.11947E-11 | - |
| *uncharacterized protein_15144* | 746.6863087 | 227.4096738 | 0.304558516 | -1.715208651 | 1.56145E-12 | 3.12007E-11 | - |
| *GGP3* | 35.28097794 | 0 | 0 | -Inf | 1.582E-12 | 3.15872E-11 | - |
| *UGT85K4* | 614.8224559 | 183.9700635 | 0.299224698 | -1.740698836 | 1.62547E-12 | 3.24061E-11 | - |
| *PPC4* | 3453.657411 | 1173.219361 | 0.339703457 | -1.557652196 | 1.67104E-12 | 3.32451E-11 | K01595 |
| *YLMG2* | 2155.204214 | 713.7658884 | 0.331182486 | -1.594301716 | 1.67134E-12 | 3.32451E-11 | K02221 |
| *At1g07700* | 439.1239412 | 121.1205759 | 0.275823212 | -1.858184223 | 1.71537E-12 | 3.40953E-11 | - |
| *uncharacterized protein_04845* | 55.40159727 | 2.630566114 | 0.047481774 | -4.396482356 | 1.73509E-12 | 3.44612E-11 | - |
| *ycf23* | 1685.320203 | 551.2600773 | 0.327095158 | -1.612217693 | 1.74402E-12 | 3.46125E-11 | - |
| *PME34* | 34.81615568 | 0 | 0 | -Inf | 1.85933E-12 | 3.68456E-11 | - |
| *Sb10g008780* | 992.7976734 | 317.1300107 | 0.319430655 | -1.646425325 | 1.96801E-12 | 3.89699E-11 | K01872 |
| *At2g42990* | 128.5001719 | 7.075120774 | 0.055059232 | -4.182871704 | 1.99342E-12 | 3.94435E-11 | - |
| *VQ4* | 126.1222161 | 20.28450951 | 0.160832168 | -2.636372102 | 2.07092E-12 | 4.09155E-11 | - |
| *At5g48740* | 38.34408057 | 0.32861806 | 0.008570242 | -6.866448296 | 2.21244E-12 | 4.36134E-11 | - |
| *PLP7* | 237.8114374 | 56.59751755 | 0.237993253 | -2.071007421 | 2.24947E-12 | 4.42772E-11 | - |
| *CYP90A3* | 441.4762035 | 125.0142323 | 0.283173207 | -1.820243328 | 2.38327E-12 | 4.67014E-11 | K09588 |
| *ag1* | 48.69691659 | 1.659207147 | 0.034072119 | -4.875264507 | 2.38809E-12 | 4.67611E-11 | K16086 |
| *At1g54780* | 1824.231384 | 385.5407213 | 0.211344199 | -2.242333578 | 2.39166E-12 | 4.67963E-11 | - |
| *TOP2* | 58.08660854 | 3.326522452 | 0.057268319 | -4.126118923 | 2.40273E-12 | 4.69781E-11 | K03164 |
| *CHUP1* | 349.0287676 | 93.379125 | 0.26753991 | -1.902173976 | 2.45236E-12 | 4.79128E-11 | - |
| *MCM7* | 38.30223546 | 0.344734906 | 0.009000386 | -6.7957974 | 2.50567E-12 | 4.8918E-11 | K02210 |
| *ag1* | 34.96656879 | 0 | 0 | -Inf | 2.52463E-12 | 4.92518E-11 | K16086 |
| *LTPG16* | 43.92338945 | 1.001971027 | 0.022811788 | -5.454076691 | 2.77015E-12 | 5.39616E-11 | - |
| *NRT2.1* | 98.35902172 | 13.14340563 | 0.133626844 | -2.903718241 | 2.8817E-12 | 5.6093E-11 | K02575 |
| *At1g64390* | 68.82299302 | 2.957562543 | 0.042973466 | -4.540410052 | 2.97921E-12 | 5.79483E-11 | - |
| *CRK2* | 45.70447361 | 1.332210718 | 0.029148366 | -5.100441185 | 3.00506E-12 | 5.8408E-11 | - |
| *RUN1* | 43.14629827 | 1.003592658 | 0.023260226 | -5.425991062 | 3.08955E-12 | 6.0006E-11 | - |
| *TUBB5* | 1041.321028 | 340.7980116 | 0.327274685 | -1.611426081 | 3.25498E-12 | 6.30333E-11 | K07375 |
| *sigA* | 2881.964787 | 981.5706065 | 0.34059077 | -1.553888755 | 3.28729E-12 | 6.36122E-11 | - |
| *KAS2* | 1755.561544 | 589.3882324 | 0.335726329 | -1.574642412 | 3.51209E-12 | 6.77632E-11 | K09458 |
| *IRL1* | 618.1571263 | 189.4646285 | 0.306499141 | -1.706045062 | 3.55846E-12 | 6.85577E-11 | - |
| *ROPGEF8* | 126.2566819 | 21.07696798 | 0.166937446 | -2.582620493 | 3.67198E-12 | 7.05898E-11 | - |
| *HSL1* | 583.7447795 | 175.9280186 | 0.301378316 | -1.730352475 | 3.81121E-12 | 7.31064E-11 | - |
| *MIK1* | 299.1918393 | 78.06269964 | 0.260911861 | -1.938365564 | 3.8895E-12 | 7.4554E-11 | - |
| *CA2* | 158.9388836 | 30.54901034 | 0.192206021 | -2.379274567 | 4.09549E-12 | 7.83314E-11 | K15746 |
| *uncharacterized protein_37773* | 34.25644846 | 0 | 0 | -Inf | 4.09958E-12 | 7.83528E-11 | - |
| *YSL12* | 73.54174024 | 7.076742406 | 0.096227563 | -3.377406005 | 4.13896E-12 | 7.89908E-11 | - |
| *WRI1* | 37.72832106 | 0.32861806 | 0.008710116 | -6.84309232 | 4.30066E-12 | 8.18989E-11 | K09285 |
| *CRK2* | 79.45459841 | 8.310630419 | 0.104595965 | -3.257100893 | 4.35828E-12 | 8.29363E-11 | - |
| *At3g47110* | 63.82634864 | 4.666841327 | 0.073117786 | -3.773633795 | 4.47017E-12 | 8.50042E-11 | - |
| *OPS* | 238.9704089 | 56.9099193 | 0.238146303 | -2.070079945 | 4.63302E-12 | 8.80375E-11 | - |
| *XTH33* | 79.86792939 | 8.366989644 | 0.104760318 | -3.254835757 | 4.87179E-12 | 9.23747E-11 | K08235 |
| *RER6* | 428.3417988 | 122.3202236 | 0.285566863 | -1.808099517 | 5.03904E-12 | 9.54774E-11 | - |
| *At1g02270* | 510.7556732 | 152.0867972 | 0.2977682 | -1.747738407 | 5.59939E-12 | 1.0579E-10 | - |
| *At4g26790* | 37.23316391 | 0.32861806 | 0.00882595 | -6.824032635 | 5.66785E-12 | 1.07007E-10 | - |
| *ANR* | 317.1571002 | 84.10808272 | 0.265193756 | -1.914881286 | 5.72513E-12 | 1.07934E-10 | K08695 |
| *CAT1* | 52.35651461 | 2.6676647 | 0.050951915 | -4.29471981 | 5.81574E-12 | 1.09564E-10 | K03294 |
| *uncharacterized protein_06624* | 301.0501929 | 79.03253645 | 0.26252279 | -1.929485427 | 6.00514E-12 | 1.1297E-10 | - |
| *AMY1.3* | 187.3947307 | 40.61717061 | 0.216746599 | -2.205918736 | 6.53178E-12 | 1.22528E-10 | K01176 |
| *PAD4* | 118.6338669 | 19.87531366 | 0.167534905 | -2.577466388 | 7.14837E-12 | 1.33999E-10 | - |
| *CRYD* | 171.0582246 | 35.44138804 | 0.207189032 | -2.270980459 | 7.43746E-12 | 1.3922E-10 | - |
| *IP5P4* | 52.61105869 | 2.653169485 | 0.050429882 | -4.309577338 | 7.53268E-12 | 1.40902E-10 | K20279 |
| *CPK17* | 287.0173746 | 75.53998709 | 0.263189597 | -1.925825631 | 7.56937E-12 | 1.41488E-10 | K13412 |
| *LSF2* | 1371.811945 | 460.3316382 | 0.335564681 | -1.575337217 | 7.57872E-12 | 1.41563E-10 | - |
| *uncharacterized protein_42544* | 72.27799405 | 4.044982692 | 0.055964236 | -4.159351033 | 8.18203E-12 | 1.52292E-10 | - |
| *MYB1* | 124.164945 | 20.96415006 | 0.168841134 | -2.566261674 | 8.69109E-12 | 1.61425E-10 | K09422 |
| *IMPL1* | 810.189746 | 261.3159181 | 0.322536689 | -1.632464817 | 8.78635E-12 | 1.6308E-10 | K01092 |
| *CIPK5* | 268.7083984 | 68.8083839 | 0.256070835 | -1.965385148 | 8.98191E-12 | 1.66475E-10 | K07198 |
| *SPT* | 200.0842691 | 22.46888066 | 0.112297087 | -3.154607585 | 9.5746E-12 | 1.76962E-10 | - |
| *NEAP3* | 78.85189769 | 8.734327922 | 0.110768773 | -3.174376874 | 9.69015E-12 | 1.78722E-10 | - |
| *PHL3* | 697.1038287 | 221.4542004 | 0.317677498 | -1.654365189 | 1.06887E-11 | 1.96452E-10 | - |
| *ISE2* | 432.5144217 | 126.5265608 | 0.292537207 | -1.773307967 | 1.09E-11 | 2.00056E-10 | - |
| *At1g56140* | 88.4066101 | 11.25369775 | 0.127294755 | -2.973755122 | 1.10544E-11 | 2.02748E-10 | - |
| *HIPP37* | 599.3450869 | 187.7480502 | 0.313255342 | -1.674588983 | 1.1267E-11 | 2.06423E-10 | - |
| *TIC32* | 215.8869375 | 29.79345796 | 0.138004913 | -2.85720847 | 1.12704E-11 | 2.06423E-10 | - |
| *uncharacterized protein_17326* | 36.51327343 | 0.32861806 | 0.008999962 | -6.795865432 | 1.14599E-11 | 2.09748E-10 | - |
| *uncharacterized protein_39029* | 78.87320629 | 1.989446839 | 0.025223354 | -5.309096038 | 1.17104E-11 | 2.13887E-10 | - |
| *uncharacterized protein_09062* | 1276.275813 | 430.2557873 | 0.337118186 | -1.568673638 | 1.17286E-11 | 2.14071E-10 | - |
| *ZHD7* | 38.0633907 | 0 | 0 | -Inf | 1.21277E-11 | 2.2105E-10 | - |
| *ANR* | 107.963057 | 16.65825995 | 0.154295927 | -2.696228116 | 1.23429E-11 | 2.24817E-10 | K13082 |
| *PDR17* | 41.04013437 | 0.985854181 | 0.024021709 | -5.379517373 | 1.24691E-11 | 2.26957E-10 | - |
| *UGT85A23* | 94.9173318 | 13.11755899 | 0.138199829 | -2.855172268 | 1.28541E-11 | 2.33643E-10 | - |
| *EXL2* | 70.9817966 | 7.055760666 | 0.099402396 | -3.33057556 | 1.28643E-11 | 2.33668E-10 | - |
| *ag4* | 32.45011458 | 0 | 0 | -Inf | 1.40276E-11 | 2.53748E-10 | - |
| *CHUP1* | 112.9103785 | 2.364793805 | 0.02094399 | -5.577319897 | 1.40905E-11 | 2.54711E-10 | - |
| *Kdsr* | 121.8428217 | 21.37497398 | 0.17543072 | -2.511026689 | 1.41319E-11 | 2.55274E-10 | K04708 |
| *PAP12* | 876.0680028 | 287.6516747 | 0.328344003 | -1.606719991 | 1.41417E-11 | 2.55274E-10 | - |
| *RAM1* | 382.7593195 | 111.7227436 | 0.291887716 | -1.776514598 | 1.41507E-11 | 2.55274E-10 | - |
| *At5g46170* | 219.305394 | 53.21787914 | 0.242665619 | -2.042958375 | 1.4334E-11 | 2.58227E-10 | - |
| *PRR37* | 7085.992132 | 2561.639133 | 0.361507476 | -1.467902611 | 1.45369E-11 | 2.61704E-10 | K12129 |
| *DLO1* | 49.36050681 | 2.306812948 | 0.04673398 | -4.419384277 | 1.48957E-11 | 2.67797E-10 | - |
| *CRN* | 121.653204 | 20.96911442 | 0.172367959 | -2.536436477 | 1.59503E-11 | 2.86561E-10 | - |
| *FLA17* | 1363.334417 | 56.46048751 | 0.041413528 | -4.593754093 | 1.72201E-11 | 3.09164E-10 | - |
| *Prcp* | 104.4640424 | 15.81897311 | 0.151429839 | -2.723278582 | 1.75398E-11 | 3.14476E-10 | K01285 |
| *GGP3* | 108.9094773 | 12.29601062 | 0.112901199 | -3.146867281 | 1.7564E-11 | 3.14696E-10 | - |
| *DIM* | 644.4315042 | 206.24026 | 0.320034416 | -1.643701037 | 1.82575E-11 | 3.26455E-10 | K09828 |
| *comta* | 93.01942983 | 12.69233933 | 0.13644826 | -2.8735741 | 1.83099E-11 | 3.27169E-10 | K00545 |
| *CYP92C6* | 69.34970098 | 2.983409177 | 0.043019784 | -4.538855899 | 1.90934E-11 | 3.40478E-10 | - |
| *MOT2* | 105.1307972 | 16.50530556 | 0.156997816 | -2.671183605 | 1.98666E-11 | 3.53786E-10 | - |
| *At5g39980* | 192.3751234 | 44.52531576 | 0.231450486 | -2.111224504 | 1.9927E-11 | 3.54623E-10 | - |
| *FLA17* | 1069.950517 | 368.9711944 | 0.34484884 | -1.535963982 | 2.01532E-11 | 3.58407E-10 | - |
| *At2g02240* | 253.6991968 | 64.8805738 | 0.255738192 | -1.967260468 | 2.05979E-11 | 3.66067E-10 | - |
| *NAT12* | 227.0081103 | 48.47998444 | 0.213560583 | -2.227282701 | 2.07554E-11 | 3.68617E-10 | K14611 |
| *FAR1* | 40.79294403 | 0.989097444 | 0.024246778 | -5.366063161 | 2.10454E-11 | 3.73518E-10 | K13356 |
| *At5g42610* | 68.7362339 | 6.598207841 | 0.095993153 | -3.38092468 | 2.12601E-11 | 3.76819E-10 | - |
| *GSTU17* | 124.3178027 | 22.84899305 | 0.18379502 | -2.443830417 | 2.14192E-11 | 3.79384E-10 | K00799 |
| *uncharacterized protein_07225* | 257.5888656 | 57.4093297 | 0.222871938 | -2.165713118 | 2.14949E-11 | 3.80469E-10 | - |
| *PCMP-H40* | 478.7333448 | 146.192256 | 0.305373038 | -1.711355405 | 2.17464E-11 | 3.84662E-10 | - |
| *RBCS* | 52.20849406 | 1.316093873 | 0.025208424 | -5.309950244 | 2.17804E-11 | 3.85007E-10 | K01602 |
| *GT7* | 327.8617817 | 92.88792223 | 0.283314273 | -1.819524812 | 2.19662E-11 | 3.88029E-10 | - |
| *RPL6* | 1592.922833 | 553.4377758 | 0.347435396 | -1.525183353 | 2.39322E-11 | 4.22193E-10 | K02933 |
| *HCF173* | 381.9151661 | 110.976292 | 0.290578384 | -1.783000707 | 2.40388E-11 | 4.23791E-10 | - |
| *At1g80870* | 42.39126394 | 1.330589087 | 0.031388285 | -4.993629971 | 2.50825E-11 | 4.41895E-10 | - |
| *FPP4* | 291.3563861 | 80.57265097 | 0.276543281 | -1.854422805 | 2.60472E-11 | 4.58278E-10 | - |
| *MFSD14A* | 212.6977711 | 51.55532282 | 0.242387697 | -2.044611624 | 2.80019E-11 | 4.91357E-10 | - |
| *ISE2* | 460.8582599 | 141.143886 | 0.306263114 | -1.707156472 | 2.88396E-11 | 5.05383E-10 | - |
| *CYP86B1* | 67.49354836 | 6.366290854 | 0.094324436 | -3.406224628 | 2.90127E-11 | 5.07742E-10 | K15402 |
| *NORK* | 34.67827722 | 0.330239692 | 0.009522956 | -6.714374889 | 2.95282E-11 | 5.16421E-10 | - |
| *ARR4* | 224.1652126 | 56.46838385 | 0.251905205 | -1.989047162 | 2.99114E-11 | 5.22775E-10 | K14492 |
| *uncharacterized protein_23244* | 227.3990168 | 56.61028523 | 0.248946922 | -2.006089919 | 3.07367E-11 | 5.36133E-10 | - |
| *MDHG* | 8603.958316 | 3155.902929 | 0.366796632 | -1.446947704 | 3.11232E-11 | 5.42157E-10 | K00026 |
| *CCR1* | 31.49469619 | 0 | 0 | -Inf | 3.18812E-11 | 5.54627E-10 | K09753 |
| *uncharacterized protein_46533* | 31.09898502 | 0 | 0 | -Inf | 3.24125E-11 | 5.63499E-10 | - |
| *nep1* | 34.38328439 | 0.330239692 | 0.009604658 | -6.702050022 | 3.25234E-11 | 5.65053E-10 | - |
| *dri1* | 606.3345551 | 162.5241356 | 0.268043664 | -1.899460063 | 3.43761E-11 | 5.96456E-10 | - |
| *DRT100* | 40.24046221 | 0 | 0 | -Inf | 3.49431E-11 | 6.05497E-10 | - |
| *RAN1A* | 88.66431883 | 11.98513104 | 0.13517423 | -2.887107955 | 3.6338E-11 | 6.28841E-10 | K07936 |
| *At3g21360* | 178.3908514 | 41.53866332 | 0.232851982 | -2.102514933 | 3.68562E-11 | 6.3739E-10 | - |
| *uncharacterized protein_02297* | 231.2474279 | 59.84335631 | 0.25878496 | -1.950174323 | 3.69681E-11 | 6.38906E-10 | - |
| *HSP90-1* | 1068.815776 | 374.1563025 | 0.350066224 | -1.514300225 | 3.75108E-11 | 6.47013E-10 | K04079 |
| *SCL6* | 1237.423169 | 430.3407455 | 0.347771689 | -1.523787602 | 3.76537E-11 | 6.48628E-10 | - |
| *uncharacterized protein_10454* | 76.48067568 | 3.998253786 | 0.052277961 | -4.257653315 | 3.87167E-11 | 6.66504E-10 | K10258 |
| *slr0537* | 123.4624545 | 22.05319184 | 0.178622658 | -2.485012996 | 4.00651E-11 | 6.88816E-10 | - |
| *At1g67720* | 174.2865675 | 38.72766167 | 0.222206807 | -2.170025082 | 4.14189E-11 | 7.11164E-10 | - |
| *MAN1* | 67.77650245 | 0.344734906 | 0.005086348 | -7.619153994 | 4.31499E-11 | 7.39921E-10 | K19355 |
| *ag1* | 42.0271318 | 1.348327564 | 0.032082312 | -4.962078067 | 4.32209E-11 | 7.40658E-10 | K16086 |
| *PSPPH_1014* | 495.6743918 | 146.3906225 | 0.295336263 | -1.759569586 | 4.43416E-11 | 7.58383E-10 | - |
| *XA21* | 85.39307825 | 11.42936142 | 0.13384412 | -2.901374334 | 4.55435E-11 | 7.77425E-10 | - |
| *AAF* | 1602.190803 | 566.8546641 | 0.353799724 | -1.498995172 | 4.6855E-11 | 7.99294E-10 | - |
| *FBN5* | 558.4638002 | 176.4416387 | 0.315941049 | -1.662272703 | 4.9268E-11 | 8.39031E-10 | - |
| *ABCG3* | 134.8546753 | 26.70381685 | 0.198019214 | -2.33628767 | 4.95027E-11 | 8.42281E-10 | - |
| *CHX19* | 244.1170225 | 48.39006829 | 0.198224883 | -2.334790019 | 5.0677E-11 | 8.61706E-10 | - |
| *At1g48100* | 38.6728209 | 0.689469812 | 0.017828278 | -5.809688807 | 5.16879E-11 | 8.78327E-10 | - |
| *uncharacterized protein_07435* | 122.7355823 | 23.08901819 | 0.188120004 | -2.410274829 | 5.19433E-11 | 8.82099E-10 | - |
| *SLC4A1* | 44.99764192 | 2.023302162 | 0.044964626 | -4.475065705 | 5.26555E-11 | 8.93618E-10 | - |
| *At1g34300* | 193.8505159 | 46.79664531 | 0.241405833 | -2.050467558 | 5.42025E-11 | 9.19279E-10 | - |
| *CYP90D2* | 36.45582478 | 0.658857752 | 0.01807277 | -5.790038511 | 5.45542E-11 | 9.24055E-10 | K12638 |
| *At5g07830* | 167.310495 | 24.47272324 | 0.146271298 | -2.773281391 | 5.46293E-11 | 9.24734E-10 | K07964 |
| *FLZ5* | 928.0745643 | 317.9294402 | 0.342568854 | -1.545534105 | 5.47357E-11 | 9.2594E-10 | - |
| *EO* | 57.47236936 | 4.365592064 | 0.075959841 | -3.718619296 | 5.52063E-11 | 9.32704E-10 | K18980 |
| *At5g15710* | 152.6693666 | 33.00573979 | 0.216190979 | -2.209621772 | 5.61118E-11 | 9.47147E-10 | - |
| *GSVIVT00026920001* | 33.44092479 | 0.330239692 | 0.009875316 | -6.661957405 | 5.61331E-11 | 9.47147E-10 | - |
| *HPCA1* | 135.372009 | 0.657236121 | 0.004855037 | -7.686301976 | 5.7741E-11 | 9.73654E-10 | - |
| *At5g01610* | 142.3398881 | 29.4682821 | 0.207027577 | -2.272105143 | 5.78554E-11 | 9.74959E-10 | - |
| *ISPH* | 2617.240318 | 636.3210456 | 0.243126717 | -2.040219657 | 5.95441E-11 | 1.00213E-09 | K03527 |
| *GATC* | 216.703577 | 54.75453858 | 0.252670211 | -1.984672508 | 6.31347E-11 | 1.05918E-09 | K02435 |
| *HIPP30* | 96.88257657 | 14.96529695 | 0.154468404 | -2.694616327 | 6.75733E-11 | 1.12934E-09 | - |
| *BANGLUC* | 79.30443326 | 1.316093873 | 0.016595464 | -5.913067217 | 6.99053E-11 | 1.16757E-09 | - |
| *21KD* | 488.6955027 | 145.0007422 | 0.296709795 | -1.752875543 | 7.00555E-11 | 1.16934E-09 | - |
| *Os07g0190000* | 66.6825857 | 6.678792069 | 0.100157965 | -3.319650943 | 7.39041E-11 | 1.22968E-09 | K01662 |
| *LRK10L-2.6* | 43.00169034 | 0.330239692 | 0.007679691 | -7.024736032 | 7.50527E-11 | 1.24801E-09 | - |
| *CBP1* | 402.1784446 | 122.7439082 | 0.30519763 | -1.712184335 | 8.06176E-11 | 1.33885E-09 | K16296 |
| *TUBA* | 16648.64525 | 6391.69632 | 0.383916903 | -1.381134016 | 8.22088E-11 | 1.36258E-09 | K07374 |
| *At5g10770* | 29.84234024 | 0 | 0 | -Inf | 8.22534E-11 | 1.36258E-09 | - |
| *GYRBM* | 723.6718877 | 244.6751977 | 0.338102394 | -1.564467863 | 8.34309E-11 | 1.37949E-09 | K02470 |
| *PUX10* | 287.6247603 | 81.72402827 | 0.28413419 | -1.815355653 | 8.50817E-11 | 1.40502E-09 | K18726 |
| *ARF4* | 308.1746861 | 88.80106909 | 0.28815173 | -1.795099411 | 8.75478E-11 | 1.44303E-09 | K14486 |
| *RUN1* | 157.4335238 | 20.44060769 | 0.129836436 | -2.945232787 | 9.25661E-11 | 1.52099E-09 | - |
| *CLPF* | 856.3257947 | 292.9565974 | 0.342108809 | -1.547472844 | 9.38603E-11 | 1.54034E-09 | - |
| *XTH8* | 570.4478751 | 84.53147537 | 0.148184399 | -2.75453453 | 9.63605E-11 | 1.58038E-09 | K08235 |
| *UGT92A1* | 397.3668852 | 122.5013361 | 0.3082827 | -1.697674165 | 1.00644E-10 | 1.64961E-09 | - |
| *DIR1* | 44.57097596 | 2.024923793 | 0.045431444 | -4.460165033 | 1.01091E-10 | 1.65591E-09 | - |
| *CSP41B* | 1850.701064 | 674.166105 | 0.364276067 | -1.456895882 | 1.0497E-10 | 1.71731E-09 | - |
| *At2g01630* | 257.904819 | 72.20850027 | 0.279981198 | -1.836598146 | 1.05466E-10 | 1.72436E-09 | K19891 |
| *uncharacterized protein_08399* | 29.45131407 | 0 | 0 | -Inf | 1.06804E-10 | 1.74515E-09 | - |
| *PME68* | 101.3489482 | 1.691440838 | 0.016689279 | -5.904934589 | 1.0693E-10 | 1.74612E-09 | - |
| *TPS-mISO1* | 46.2748232 | 2.398649127 | 0.051834863 | -4.269933436 | 1.10308E-10 | 1.80018E-09 | K04120 |
| *BGLU42* | 142.5358069 | 30.56533257 | 0.214439678 | -2.221356219 | 1.11184E-10 | 1.81335E-09 | K01188 |
| *UNC* | 120.6147153 | 22.98755169 | 0.190586627 | -2.391481206 | 1.11553E-10 | 1.81824E-09 | - |
| *uncharacterized protein_30070* | 50.65570806 | 3.307162343 | 0.065287062 | -3.937059074 | 1.12288E-10 | 1.82909E-09 | - |
| *GDPDL7* | 171.1773067 | 40.06140099 | 0.234034533 | -2.095206673 | 1.18912E-10 | 1.93103E-09 | - |
| *At1g01540* | 258.807974 | 71.81713593 | 0.277491975 | -1.849482044 | 1.20014E-10 | 1.94639E-09 | - |
| *LECRK41* | 117.8664219 | 10.31943737 | 0.087551969 | -3.513716559 | 1.22345E-10 | 1.98188E-09 | - |
| *PUB9* | 58.08825286 | 5.077565778 | 0.087411233 | -3.516037509 | 1.26157E-10 | 2.04113E-09 | - |
| *uncharacterized protein_39935* | 372.0313414 | 113.6685989 | 0.305535008 | -1.710590402 | 1.28974E-10 | 2.08543E-09 | - |
| *uncharacterized protein_28484* | 165.8128652 | 38.57308565 | 0.232630234 | -2.103889484 | 1.29591E-10 | 2.09411E-09 | - |
| *uncharacterized protein_12990* | 305.3595503 | 88.14038432 | 0.288644597 | -1.792633875 | 1.30646E-10 | 2.10988E-09 | - |
| *uncharacterized protein_34627* | 660.4577619 | 222.5844901 | 0.337015481 | -1.569113232 | 1.31353E-10 | 2.11998E-09 | - |
| *TLP40* | 499.9374344 | 159.4722873 | 0.31898449 | -1.648441819 | 1.388E-10 | 2.23743E-09 | - |
| *BRH1* | 79.2762712 | 10.40316539 | 0.131226724 | -2.92986654 | 1.4442E-10 | 2.32236E-09 | K16281 |
| *At3g23880* | 79.90319723 | 10.65930738 | 0.133402764 | -2.906139535 | 1.51189E-10 | 2.42675E-09 | - |
| *relA* | 276.6703438 | 78.82778932 | 0.284915934 | -1.811391789 | 1.5156E-10 | 2.43124E-09 | - |
| *R63* | 72.42790688 | 6.258337829 | 0.086407824 | -3.53269424 | 1.6021E-10 | 2.56687E-09 | K00430 |
| *At1g62810* | 317.3562401 | 94.82243254 | 0.298788618 | -1.742802901 | 1.66771E-10 | 2.66874E-09 | K00276 |
| *PUB45* | 129.5924726 | 26.61532341 | 0.205377078 | -2.283652919 | 1.6877E-10 | 2.6991E-09 | - |
| *CBG* | 1517.68353 | 563.2548293 | 0.371127984 | -1.430011305 | 1.78286E-10 | 2.84438E-09 | K01188 |
| *MSRA3* | 7947.209103 | 2929.989639 | 0.368681584 | -1.439552742 | 1.84579E-10 | 2.93943E-09 | K07304 |
| *RPT3* | 37.12245567 | 0.985854181 | 0.026556815 | -5.234774067 | 1.89915E-10 | 3.02076E-09 | - |
| *DAD2* | 84.36510793 | 0.660479384 | 0.007828822 | -6.996989107 | 1.90399E-10 | 3.02662E-09 | - |
| *CBP1* | 107.1746753 | 19.23743121 | 0.179496053 | -2.477975974 | 2.0007E-10 | 3.17462E-09 | K16296 |
| *CCR1* | 164.3459422 | 38.68093921 | 0.23536291 | -2.087041106 | 2.02082E-10 | 3.20461E-09 | - |
| *CESA5* | 1658.061992 | 102.1986967 | 0.06163744 | -4.020049245 | 2.04833E-10 | 3.24434E-09 | K10999 |
| *CYP75B137* | 318.3025126 | 72.15031404 | 0.226672147 | -2.141320966 | 2.0962E-10 | 3.31618E-09 | K05280 |
| *IGPS* | 1989.334324 | 742.2606081 | 0.373120093 | -1.422288041 | 2.10687E-10 | 3.33105E-09 | K01609 |
| *uncharacterized protein_32466* | 95.54413385 | 15.66439709 | 0.163949334 | -2.608678056 | 2.16611E-10 | 3.42061E-09 | - |
| *uncharacterized protein_04541* | 1408.920254 | 501.6114981 | 0.356025472 | -1.489947633 | 2.20039E-10 | 3.47266E-09 | - |
| *NOL* | 226.447507 | 60.94993119 | 0.269156998 | -1.893480159 | 2.22977E-10 | 3.51692E-09 | K13606 |
| *PIP2-8* | 42.6666207 | 0.657236121 | 0.015403988 | -6.020552269 | 2.38849E-10 | 3.75379E-09 | K09872 |
| *HI_0077* | 246.4816152 | 68.08941344 | 0.276245404 | -1.855977631 | 2.42588E-10 | 3.80574E-09 | - |
| *uncharacterized protein_25584* | 335.5353397 | 104.2930586 | 0.310825854 | -1.685821583 | 2.46648E-10 | 3.86254E-09 | - |
| *EXPA2* | 31.42608102 | 0.32861806 | 0.010456858 | -6.579406792 | 2.50354E-10 | 3.91825E-09 | - |
| *ag1* | 28.29551162 | 0 | 0 | -Inf | 2.63414E-10 | 4.11287E-09 | K16086 |
| *BHLH80* | 848.4578295 | 301.6525164 | 0.355530359 | -1.491955339 | 2.69275E-10 | 4.2019E-09 | - |
| *45572* | 533.7080043 | 85.37936772 | 0.159973932 | -2.644091264 | 2.73121E-10 | 4.2594E-09 | - |
| *LRK10L-1.2* | 56.29144117 | 5.045332087 | 0.08962876 | -3.479894448 | 2.87081E-10 | 4.47182E-09 | - |
| *CSLA9* | 186.9256867 | 2.007185316 | 0.010737879 | -6.541147202 | 2.87324E-10 | 4.47295E-09 | K13680 |
| *ZOX1* | 54.56374058 | 4.669985121 | 0.085587701 | -3.546452692 | 2.96879E-10 | 4.61897E-09 | K13495 |
| *ag1* | 28.25859947 | 0 | 0 | -Inf | 3.01948E-10 | 4.69507E-09 | K16086 |
| *SAMDC* | 378.3767033 | 18.33682721 | 0.048461829 | -4.367007326 | 3.12224E-10 | 4.84628E-09 | K01611 |
| *MCM3* | 27.78273467 | 0 | 0 | -Inf | 3.17072E-10 | 4.91863E-09 | K02541 |
| *uncharacterized protein_42574* | 185.7826713 | 47.01092973 | 0.253042598 | -1.982547824 | 3.22996E-10 | 5.0017E-09 | - |
| *uncharacterized protein_44598* | 75.310914 | 9.994062569 | 0.13270404 | -2.913715799 | 3.26718E-10 | 5.05636E-09 | - |
| *ARC5* | 2292.76124 | 867.4264457 | 0.378332654 | -1.402272792 | 3.3361E-10 | 5.15696E-09 | - |
| *PSBY* | 3387.101003 | 618.5794604 | 0.182627994 | -2.453020173 | 3.50784E-10 | 5.40976E-09 | K02723 |
| *NPF5.2* | 33.7614153 | 0.657236121 | 0.019467078 | -5.682819803 | 3.56943E-10 | 5.50151E-09 | - |
| *uncharacterized protein_19787* | 473.3940966 | 156.653906 | 0.330916476 | -1.595460973 | 3.58969E-10 | 5.52676E-09 | - |
| *uncharacterized protein_22835* | 603.0262979 | 209.4167793 | 0.347276363 | -1.525843878 | 3.59E-10 | 5.52676E-09 | - |
| *NIC1* | 211.0277671 | 56.47973527 | 0.26764125 | -1.90162761 | 3.60567E-10 | 5.5444E-09 | - |
| *ROC1* | 398.7393952 | 73.34700399 | 0.183947222 | -2.442636207 | 3.6134E-10 | 5.55304E-09 | K11294 |
| *PXC1* | 397.2421851 | 100.2738231 | 0.252424911 | -1.986073804 | 3.77077E-10 | 5.79152E-09 | - |
| *5NG4* | 446.600928 | 147.3597695 | 0.329958494 | -1.599643539 | 3.77679E-10 | 5.79609E-09 | - |
| *XTH5* | 27.56153793 | 0 | 0 | -Inf | 3.88059E-10 | 5.94978E-09 | K08235 |
| *WNK4* | 59.2452037 | 5.678442674 | 0.095846454 | -3.383131136 | 3.901E-10 | 5.9776E-09 | K08867 |
| *AHP1* | 396.238216 | 19.07961192 | 0.048151872 | -4.376264298 | 4.03603E-10 | 6.17732E-09 | K14490 |
| *PIP1.4* | 7626.639966 | 2965.127901 | 0.388785614 | -1.362953258 | 4.10755E-10 | 6.27586E-09 | K09872 |
| *HCF101* | 327.9314213 | 100.8729725 | 0.307603864 | -1.700854467 | 4.1318E-10 | 6.30925E-09 | - |
| *XA21* | 38.164357 | 1.348327564 | 0.035329498 | -4.822982951 | 4.29778E-10 | 6.55511E-09 | - |
| *SMXL3* | 63.09439121 | 1.643090302 | 0.026041781 | -5.263028086 | 4.36813E-10 | 6.65855E-09 | - |
| *CRSP* | 51.22478527 | 4.028865846 | 0.078650712 | -3.668396359 | 4.40817E-10 | 6.7157E-09 | - |
| *MCM4* | 30.78646814 | 0.330239692 | 0.010726781 | -6.54263903 | 4.42506E-10 | 6.72977E-09 | K02212 |
| *OPT4* | 121.2515354 | 25.52314427 | 0.210497493 | -2.248125041 | 4.4324E-10 | 6.73704E-09 | - |
| *SCP26* | 27.44447215 | 0 | 0 | -Inf | 4.57493E-10 | 6.94967E-09 | K16297 |
| *Ta1476* | 272.6877387 | 81.07023435 | 0.297300622 | -1.750005613 | 4.58355E-10 | 6.95876E-09 | - |
| *HCT* | 214.0454598 | 52.45441109 | 0.245062012 | -2.02878123 | 4.61612E-10 | 7.00417E-09 | K13065 |
| *CYP724B1* | 398.914586 | 128.1278866 | 0.32119128 | -1.638495368 | 4.71989E-10 | 7.15751E-09 | K12639 |
| *At3g10130* | 818.8005221 | 291.6193035 | 0.356154272 | -1.489425801 | 4.76306E-10 | 7.21881E-09 | - |
| *rsmH* | 220.4030039 | 60.69875356 | 0.27539894 | -1.86040509 | 4.99205E-10 | 7.55284E-09 | - |
| *uncharacterized protein_14429* | 260.9099206 | 77.51991595 | 0.297113716 | -1.750912889 | 5.11258E-10 | 7.72633E-09 | - |
| *RCCR* | 1690.818153 | 637.2319475 | 0.376877872 | -1.407831004 | 5.21469E-10 | 7.87005E-09 | K13545 |
| *MAN1* | 283.5366041 | 84.95263878 | 0.299617889 | -1.738804331 | 5.21664E-10 | 7.87005E-09 | K19355 |
| *BXL4* | 626.3480093 | 8.325125633 | 0.013291534 | -6.233348611 | 5.39666E-10 | 8.13233E-09 | K15920 |
| *PXN* | 398.0205813 | 129.0041661 | 0.32411431 | -1.625425376 | 5.45784E-10 | 8.21513E-09 | K13354 |
| *uncharacterized protein_34540* | 97.06980607 | 17.04982323 | 0.175644971 | -2.509265828 | 5.47187E-10 | 8.23155E-09 | - |
| *OBL1* | 39.55087838 | 1.664072042 | 0.042074212 | -4.570919938 | 5.62146E-10 | 8.45175E-09 | - |
| *FDC1* | 470.6591426 | 157.2319742 | 0.334067608 | -1.581787991 | 5.78253E-10 | 8.684E-09 | K02639 |
| *AGD12* | 66.0442452 | 0 | 0 | -Inf | 5.82083E-10 | 8.73158E-09 | K12486 |
| *At3g18200* | 58.84669981 | 4.748947718 | 0.080700324 | -3.631281731 | 6.33289E-10 | 9.47812E-09 | - |
[truncated: 275,145 more chars]
