# Supplementary material for: Response mechanism of carbon metabolism of Pinus massoniana to gradient high temperature and drought stress
Source: BMC Genomics. 2024 Feb 12;25:166. doi: 10.1186/s12864-024-10054-2 (PMC10860282; doi:10.1186/s12864-024-10054-2)
Supplement: Supplementary file 5 — Additional file 5. [file 12864_2024_10054_MOESM5_ESM.docx]

Table S6 The KEGG enrichment analysis results comparing T25CK and T25Z treatments are summarized.

| **#Kegg_pathway** | **ko_id** | **Cluter_frequency** | **Genome_frequency** | **P-value** |
| --- | --- | --- | --- | --- |
| Carbon metabolism | ko01200 | 33 out of 640 5.15625% | 33 out of 683 4.83162518301611% | 0.110848713 |
| Flavonoid biosynthesis | ko00941 | 30 out of 640 4.6875% | 30 out of 683 4.39238653001464% | 0.136025933 |
| Glycolysis / Gluconeogenesis | ko00010 | 28 out of 640 4.375% | 28 out of 683 4.099560761347% | 0.155828944 |
| Biosynthesis of amino acids | ko01230 | 28 out of 640 4.375% | 28 out of 683 4.099560761347% | 0.155828944 |
| Biosynthesis of cofactors | ko01240 | 27 out of 640 4.21875% | 27 out of 683 3.95314787701318% | 0.166759849 |
| Terpenoid backbone biosynthesis | ko00900 | 26 out of 640 4.0625% | 26 out of 683 3.80673499267936% | 0.178438471 |
| Galactose metabolism | ko00052 | 20 out of 640 3.125% | 20 out of 683 2.92825768667643% | 0.26724212 |
| Cysteine and methionine metabolism | ko00270 | 18 out of 640 2.8125% | 18 out of 683 2.63543191800878% | 0.305501008 |
| Starch and sucrose metabolism | ko00500 | 53 out of 640 8.28125% | 55 out of 683 8.05270863836018% | 0.307545422 |
| Motor proteins | ko04814 | 17 out of 640 2.65625% | 17 out of 683 2.48901903367496% | 0.326586952 |
| Tyrosine metabolism | ko00350 | 16 out of 640 2.5% | 16 out of 683 2.34260614934114% | 0.349092143 |
| Pyruvate metabolism | ko00620 | 15 out of 640 2.34375% | 15 out of 683 2.19619326500732% | 0.373109682 |
| Nitrogen metabolism | ko00910 | 15 out of 640 2.34375% | 15 out of 683 2.19619326500732% | 0.373109682 |
| Carbon fixation in photosynthetic organisms | ko00710 | 14 out of 640 2.1875% | 14 out of 683 2.0497803806735% | 0.398738622 |
| Biosynthesis of nucleotide sugars | ko01250 | 14 out of 640 2.1875% | 14 out of 683 2.0497803806735% | 0.398738622 |
| Zeatin biosynthesis | ko00908 | 13 out of 640 2.03125% | 13 out of 683 1.90336749633968% | 0.426084333 |
| Glutathione metabolism | ko00480 | 28 out of 640 4.375% | 29 out of 683 4.24597364568082% | 0.442268714 |
| Ubiquitin mediated proteolysis | ko04120 | 12 out of 640 1.875% | 12 out of 683 1.75695461200586% | 0.455258897 |
| Inositol phosphate metabolism | ko00562 | 11 out of 640 1.71875% | 11 out of 683 1.61054172767204% | 0.486381525 |
| Fatty acid metabolism | ko01212 | 11 out of 640 1.71875% | 11 out of 683 1.61054172767204% | 0.486381525 |
| Fatty acid degradation | ko00071 | 11 out of 640 1.71875% | 11 out of 683 1.61054172767204% | 0.486381525 |
| Fructose and mannose metabolism | ko00051 | 10 out of 640 1.5625% | 10 out of 683 1.46412884333821% | 0.519578994 |
| Peroxisome | ko04146 | 10 out of 640 1.5625% | 10 out of 683 1.46412884333821% | 0.519578994 |
| Purine metabolism | ko00230 | 10 out of 640 1.5625% | 10 out of 683 1.46412884333821% | 0.519578994 |
| Ascorbate and aldarate metabolism | ko00053 | 9 out of 640 1.40625% | 9 out of 683 1.31771595900439% | 0.55498612 |
| Phosphatidylinositol signaling system | ko04070 | 9 out of 640 1.40625% | 9 out of 683 1.31771595900439% | 0.55498612 |
| Flavone and flavonol biosynthesis | ko00944 | 9 out of 640 1.40625% | 9 out of 683 1.31771595900439% | 0.55498612 |
| Cyanoamino acid metabolism | ko00460 | 21 out of 640 3.28125% | 22 out of 683 3.22108345534407% | 0.59079855 |
| Pyrimidine metabolism | ko00240 | 8 out of 640 1.25% | 8 out of 683 1.17130307467057% | 0.592746252 |
| Biosynthesis of unsaturated fatty acids | ko01040 | 8 out of 640 1.25% | 8 out of 683 1.17130307467057% | 0.592746252 |
| Stilbenoid, diarylheptanoid and gingerol biosynthesis | ko00945 | 8 out of 640 1.25% | 8 out of 683 1.17130307467057% | 0.592746252 |
| Glyoxylate and dicarboxylate metabolism | ko00630 | 7 out of 640 1.09375% | 7 out of 683 1.02489019033675% | 0.633011795 |
| Pentose phosphate pathway | ko00030 | 7 out of 640 1.09375% | 7 out of 683 1.02489019033675% | 0.633011795 |
| Ubiquinone and other terpenoid-quinone biosynthesis | ko00130 | 7 out of 640 1.09375% | 7 out of 683 1.02489019033675% | 0.633011795 |
| Fatty acid biosynthesis | ko00061 | 7 out of 640 1.09375% | 7 out of 683 1.02489019033675% | 0.633011795 |
| Pantothenate and CoA biosynthesis | ko00770 | 7 out of 640 1.09375% | 7 out of 683 1.02489019033675% | 0.633011795 |
| Glycine, serine and threonine metabolism | ko00260 | 7 out of 640 1.09375% | 7 out of 683 1.02489019033675% | 0.633011795 |
| Phenylalanine metabolism | ko00360 | 7 out of 640 1.09375% | 7 out of 683 1.02489019033675% | 0.633011795 |
| Nucleotide metabolism | ko01232 | 7 out of 640 1.09375% | 7 out of 683 1.02489019033675% | 0.633011795 |
| Diterpenoid biosynthesis | ko00904 | 19 out of 640 2.96875% | 20 out of 683 2.92825768667643% | 0.637335876 |
| Metabolic pathways | ko01100 | 404 out of 640 63.125% | 432 out of 683 63.2503660322108% | 0.660703613 |
| Folate biosynthesis | ko00790 | 6 out of 640 0.9375% | 6 out of 683 0.878477306002928% | 0.675944771 |
| Fatty acid elongation | ko00062 | 6 out of 640 0.9375% | 6 out of 683 0.878477306002928% | 0.675944771 |
| Tropane, piperidine and pyridine alkaloid biosynthesis | ko00960 | 6 out of 640 0.9375% | 6 out of 683 0.878477306002928% | 0.675944771 |
| DNA replication | ko03030 | 6 out of 640 0.9375% | 6 out of 683 0.878477306002928% | 0.675944771 |
| Phagosome | ko04145 | 6 out of 640 0.9375% | 6 out of 683 0.878477306002928% | 0.675944771 |
| Isoquinoline alkaloid biosynthesis | ko00950 | 6 out of 640 0.9375% | 6 out of 683 0.878477306002928% | 0.675944771 |
| Glycerolipid metabolism | ko00561 | 6 out of 640 0.9375% | 6 out of 683 0.878477306002928% | 0.675944771 |
| 2-Oxocarboxylic acid metabolism | ko01210 | 6 out of 640 0.9375% | 6 out of 683 0.878477306002928% | 0.675944771 |
| Nucleotide excision repair | ko03420 | 6 out of 640 0.9375% | 6 out of 683 0.878477306002928% | 0.675944771 |
| Phenylalanine, tyrosine and tryptophan biosynthesis | ko00400 | 6 out of 640 0.9375% | 6 out of 683 0.878477306002928% | 0.675944771 |
| Sulfur metabolism | ko00920 | 6 out of 640 0.9375% | 6 out of 683 0.878477306002928% | 0.675944771 |
| Biosynthesis of various plant secondary metabolites | ko00999 | 16 out of 640 2.5% | 17 out of 683 2.48901903367496% | 0.709175193 |
| Selenocompound metabolism | ko00450 | 5 out of 640 0.78125% | 5 out of 683 0.732064421669107% | 0.721717409 |
| ATP-dependent chromatin remodeling | ko03082 | 5 out of 640 0.78125% | 5 out of 683 0.732064421669107% | 0.721717409 |
| Arginine biosynthesis | ko00220 | 5 out of 640 0.78125% | 5 out of 683 0.732064421669107% | 0.721717409 |
| Ribosome biogenesis in eukaryotes | ko03008 | 4 out of 640 0.625% | 4 out of 683 0.585651537335286% | 0.770512769 |
| mRNA surveillance pathway | ko03015 | 4 out of 640 0.625% | 4 out of 683 0.585651537335286% | 0.770512769 |
| ABC transporters | ko02010 | 4 out of 640 0.625% | 4 out of 683 0.585651537335286% | 0.770512769 |
| Base excision repair | ko03410 | 4 out of 640 0.625% | 4 out of 683 0.585651537335286% | 0.770512769 |
| Riboflavin metabolism | ko00740 | 4 out of 640 0.625% | 4 out of 683 0.585651537335286% | 0.770512769 |
| Vitamin B6 metabolism | ko00750 | 4 out of 640 0.625% | 4 out of 683 0.585651537335286% | 0.770512769 |
| Thiamine metabolism | ko00730 | 4 out of 640 0.625% | 4 out of 683 0.585651537335286% | 0.770512769 |
| Circadian rhythm - plant | ko04712 | 4 out of 640 0.625% | 4 out of 683 0.585651537335286% | 0.770512769 |
| beta-Alanine metabolism | ko00410 | 13 out of 640 2.03125% | 14 out of 683 2.0497803806735% | 0.781578575 |
| Arginine and proline metabolism | ko00330 | 13 out of 640 2.03125% | 14 out of 683 2.0497803806735% | 0.781578575 |
| Protein processing in endoplasmic reticulum | ko04141 | 12 out of 640 1.875% | 13 out of 683 1.90336749633968% | 0.805353668 |
| Propanoate metabolism | ko00640 | 3 out of 640 0.46875% | 3 out of 683 0.439238653001464% | 0.822525405 |
| Citrate cycle (TCA cycle) | ko00020 | 3 out of 640 0.46875% | 3 out of 683 0.439238653001464% | 0.822525405 |
| Homologous recombination | ko03440 | 3 out of 640 0.46875% | 3 out of 683 0.439238653001464% | 0.822525405 |
| Glycosphingolipid biosynthesis - globo and isoglobo series | ko00603 | 3 out of 640 0.46875% | 3 out of 683 0.439238653001464% | 0.822525405 |
| Brassinosteroid biosynthesis | ko00905 | 3 out of 640 0.46875% | 3 out of 683 0.439238653001464% | 0.822525405 |
| Sphingolipid metabolism | ko00600 | 3 out of 640 0.46875% | 3 out of 683 0.439238653001464% | 0.822525405 |
| Lysine degradation | ko00310 | 3 out of 640 0.46875% | 3 out of 683 0.439238653001464% | 0.822525405 |
| Mismatch repair | ko03430 | 3 out of 640 0.46875% | 3 out of 683 0.439238653001464% | 0.822525405 |
| Plant-pathogen interaction | ko04626 | 43 out of 640 6.71875% | 47 out of 683 6.8814055636896% | 0.834590727 |
| Linoleic acid metabolism | ko00591 | 10 out of 640 1.5625% | 11 out of 683 1.61054172767204% | 0.851553685 |
| Glycerophospholipid metabolism | ko00564 | 9 out of 640 1.40625% | 10 out of 683 1.46412884333821% | 0.873650257 |
| Monoterpenoid biosynthesis | ko00902 | 9 out of 640 1.40625% | 10 out of 683 1.46412884333821% | 0.873650257 |
| RNA polymerase | ko03020 | 2 out of 640 0.3125% | 2 out of 683 0.292825768667643% | 0.87796207 |
| Ribosome | ko03010 | 2 out of 640 0.3125% | 2 out of 683 0.292825768667643% | 0.87796207 |
| Viral life cycle - HIV-1 | ko03250 | 2 out of 640 0.3125% | 2 out of 683 0.292825768667643% | 0.87796207 |
| Basal transcription factors | ko03022 | 2 out of 640 0.3125% | 2 out of 683 0.292825768667643% | 0.87796207 |
| Glycosphingolipid biosynthesis - lacto and neolacto series | ko00601 | 2 out of 640 0.3125% | 2 out of 683 0.292825768667643% | 0.87796207 |
| Valine, leucine and isoleucine biosynthesis | ko00290 | 2 out of 640 0.3125% | 2 out of 683 0.292825768667643% | 0.87796207 |
| Valine, leucine and isoleucine degradation | ko00280 | 2 out of 640 0.3125% | 2 out of 683 0.292825768667643% | 0.87796207 |
| Exopolysaccharide biosynthesis | ko00543 | 2 out of 640 0.3125% | 2 out of 683 0.292825768667643% | 0.87796207 |
| Other glycan degradation | ko00511 | 2 out of 640 0.3125% | 2 out of 683 0.292825768667643% | 0.87796207 |
| Photosynthesis | ko00195 | 2 out of 640 0.3125% | 2 out of 683 0.292825768667643% | 0.87796207 |
| Polycomb repressive complex | ko03083 | 2 out of 640 0.3125% | 2 out of 683 0.292825768667643% | 0.87796207 |
| Nucleocytoplasmic transport | ko03013 | 2 out of 640 0.3125% | 2 out of 683 0.292825768667643% | 0.87796207 |
| Histidine metabolism | ko00340 | 2 out of 640 0.3125% | 2 out of 683 0.292825768667643% | 0.87796207 |
| Aminoacyl-tRNA biosynthesis | ko00970 | 2 out of 640 0.3125% | 2 out of 683 0.292825768667643% | 0.87796207 |
| Arachidonic acid metabolism | ko00590 | 2 out of 640 0.3125% | 2 out of 683 0.292825768667643% | 0.87796207 |
| Oxidative phosphorylation | ko00190 | 2 out of 640 0.3125% | 2 out of 683 0.292825768667643% | 0.87796207 |
| RNA degradation | ko03018 | 2 out of 640 0.3125% | 2 out of 683 0.292825768667643% | 0.87796207 |
| Biotin metabolism | ko00780 | 2 out of 640 0.3125% | 2 out of 683 0.292825768667643% | 0.87796207 |
| Spliceosome | ko03040 | 2 out of 640 0.3125% | 2 out of 683 0.292825768667643% | 0.87796207 |
| Photosynthesis - antenna proteins | ko00196 | 2 out of 640 0.3125% | 2 out of 683 0.292825768667643% | 0.87796207 |
| MAPK signaling pathway - plant | ko04016 | 26 out of 640 4.0625% | 29 out of 683 4.24597364568082% | 0.897859865 |
| Biosynthesis of secondary metabolites | ko01110 | 299 out of 640 46.71875% | 323 out of 683 47.2913616398243% | 0.905559722 |
| Cutin, suberine and wax biosynthesis | ko00073 | 15 out of 640 2.34375% | 17 out of 683 2.48901903367496% | 0.914854631 |
| Alanine, aspartate and glutamate metabolism | ko00250 | 6 out of 640 0.9375% | 7 out of 683 1.02489019033675% | 0.933542631 |
| Amino sugar and nucleotide sugar metabolism | ko00520 | 22 out of 640 3.4375% | 25 out of 683 3.66032210834553% | 0.934663011 |
| Pentose and glucuronate interconversions | ko00040 | 22 out of 640 3.4375% | 25 out of 683 3.66032210834553% | 0.934663011 |
| Neomycin, kanamycin and gentamicin biosynthesis | ko00524 | 1 out of 640 0.15625% | 1 out of 683 0.146412884333821% | 0.93704246 |
| N-Glycan biosynthesis | ko00510 | 1 out of 640 0.15625% | 1 out of 683 0.146412884333821% | 0.93704246 |
| Phosphonate and phosphinate metabolism | ko00440 | 1 out of 640 0.15625% | 1 out of 683 0.146412884333821% | 0.93704246 |
| Monobactam biosynthesis | ko00261 | 1 out of 640 0.15625% | 1 out of 683 0.146412884333821% | 0.93704246 |
| Steroid biosynthesis | ko00100 | 1 out of 640 0.15625% | 1 out of 683 0.146412884333821% | 0.93704246 |
| Autophagy - other | ko04136 | 1 out of 640 0.15625% | 1 out of 683 0.146412884333821% | 0.93704246 |
| Glucosinolate biosynthesis | ko00966 | 1 out of 640 0.15625% | 1 out of 683 0.146412884333821% | 0.93704246 |
| Glycosaminoglycan degradation | ko00531 | 1 out of 640 0.15625% | 1 out of 683 0.146412884333821% | 0.93704246 |
| Other types of O-glycan biosynthesis | ko00514 | 1 out of 640 0.15625% | 1 out of 683 0.146412884333821% | 0.93704246 |
| Nicotinate and nicotinamide metabolism | ko00760 | 1 out of 640 0.15625% | 1 out of 683 0.146412884333821% | 0.93704246 |
| Sulfur relay system | ko04122 | 1 out of 640 0.15625% | 1 out of 683 0.146412884333821% | 0.93704246 |
| Ether lipid metabolism | ko00565 | 5 out of 640 0.78125% | 6 out of 683 0.878477306002928% | 0.9505806 |
| Carotenoid biosynthesis | ko00906 | 11 out of 640 1.71875% | 13 out of 683 1.90336749633968% | 0.95730259 |
| Endocytosis | ko04144 | 10 out of 640 1.5625% | 12 out of 683 1.75695461200586% | 0.965669985 |
| Tryptophan metabolism | ko00380 | 8 out of 640 1.25% | 10 out of 683 1.46412884333821% | 0.979535493 |
| alpha-Linolenic acid metabolism | ko00592 | 14 out of 640 2.1875% | 17 out of 683 2.48901903367496% | 0.982209718 |
| Betalain biosynthesis | ko00965 | 2 out of 640 0.3125% | 3 out of 683 0.439238653001464% | 0.9888354 |
| Plant hormone signal transduction | ko04075 | 16 out of 640 2.5% | 20 out of 683 2.92825768667643% | 0.994025926 |
| Butanoate metabolism | ko00650 | 1 out of 640 0.15625% | 2 out of 683 0.292825768667643% | 0.996122849 |
| Porphyrin metabolism | ko00860 | 1 out of 640 0.15625% | 2 out of 683 0.292825768667643% | 0.996122849 |
| Taurine and hypotaurine metabolism | ko00430 | 1 out of 640 0.15625% | 2 out of 683 0.292825768667643% | 0.996122849 |
| Phenylpropanoid biosynthesis | ko00940 | 45 out of 640 7.03125% | 56 out of 683 8.199121522694% | 0.999938481 |

Table S7 The KEGG enrichment analysis results comparing T30CK and T30Z treatments are summarized.

| **#Kegg_pathway** | **ko_id** | **Cluter_frequency** | **Genome_frequency** | **P-value** |
| --- | --- | --- | --- | --- |
| Carbon metabolism | ko01200 | 91 out of 1171 7.77113578138343% | 91 out of 1275 7.13725490196078% | 0.000321281 |
| Glycolysis / Gluconeogenesis | ko00010 | 58 out of 1171 4.9530315969257% | 58 out of 1275 4.54901960784314% | 0.006383739 |
| Biosynthesis of cofactors | ko01240 | 49 out of 1171 4.18445772843723% | 49 out of 1275 3.84313725490196% | 0.014214824 |
| Carbon fixation in photosynthetic organisms | ko00710 | 43 out of 1171 3.67207514944492% | 43 out of 1275 3.37254901960784% | 0.024156842 |
| Pyruvate metabolism | ko00620 | 38 out of 1171 3.24508966695132% | 38 out of 1275 2.98039215686275% | 0.037502671 |
| Flavonoid biosynthesis | ko00941 | 74 out of 1171 6.31938514090521% | 76 out of 1275 5.96078431372549% | 0.04246117 |
| Glyoxylate and dicarboxylate metabolism | ko00630 | 28 out of 1171 2.39111870196413% | 28 out of 1275 2.19607843137255% | 0.089887239 |
| alpha-Linolenic acid metabolism | ko00592 | 28 out of 1171 2.39111870196413% | 28 out of 1275 2.19607843137255% | 0.089887239 |
| Fatty acid degradation | ko00071 | 27 out of 1171 2.30572160546541% | 27 out of 1275 2.11764705882353% | 0.098058806 |
| Motor proteins | ko04814 | 25 out of 1171 2.13492741246798% | 25 out of 1275 1.96078431372549% | 0.11667262 |
| Ubiquitin mediated proteolysis | ko04120 | 21 out of 1171 1.7933390264731% | 21 out of 1275 1.64705882352941% | 0.165027386 |
| Plant-pathogen interaction | ko04626 | 52 out of 1171 4.44064901793339% | 54 out of 1275 4.23529411764706% | 0.16665129 |
| Protein processing in endoplasmic reticulum | ko04141 | 36 out of 1171 3.07429547395389% | 37 out of 1275 2.90196078431373% | 0.17974831 |
| Biosynthesis of nucleotide sugars | ko01250 | 20 out of 1171 1.70794192997438% | 20 out of 1275 1.56862745098039% | 0.179938635 |
| Tropane, piperidine and pyridine alkaloid biosynthesis | ko00960 | 20 out of 1171 1.70794192997438% | 20 out of 1275 1.56862745098039% | 0.179938635 |
| Ubiquinone and other terpenoid-quinone biosynthesis | ko00130 | 19 out of 1171 1.62254483347566% | 19 out of 1275 1.49019607843137% | 0.196183095 |
| Stilbenoid, diarylheptanoid and gingerol biosynthesis | ko00945 | 19 out of 1171 1.62254483347566% | 19 out of 1275 1.49019607843137% | 0.196183095 |
| Glycine, serine and threonine metabolism | ko00260 | 19 out of 1171 1.62254483347566% | 19 out of 1275 1.49019607843137% | 0.196183095 |
| Nucleotide metabolism | ko01232 | 18 out of 1171 1.53714773697694% | 18 out of 1275 1.41176470588235% | 0.213878708 |
| mRNA surveillance pathway | ko03015 | 18 out of 1171 1.53714773697694% | 18 out of 1275 1.41176470588235% | 0.213878708 |
| Circadian rhythm - plant | ko04712 | 18 out of 1171 1.53714773697694% | 18 out of 1275 1.41176470588235% | 0.213878708 |
| Pentose phosphate pathway | ko00030 | 17 out of 1171 1.45175064047822% | 17 out of 1275 1.33333333333333% | 0.233153739 |
| Fatty acid metabolism | ko01212 | 17 out of 1171 1.45175064047822% | 17 out of 1275 1.33333333333333% | 0.233153739 |
| Nitrogen metabolism | ko00910 | 17 out of 1171 1.45175064047822% | 17 out of 1275 1.33333333333333% | 0.233153739 |
| Tyrosine metabolism | ko00350 | 31 out of 1171 2.64730999146029% | 32 out of 1275 2.50980392156863% | 0.248562127 |
| Pyrimidine metabolism | ko00240 | 16 out of 1171 1.3663535439795% | 16 out of 1275 1.25490196078431% | 0.254147669 |
| Citrate cycle (TCA cycle) | ko00020 | 15 out of 1171 1.28095644748079% | 15 out of 1275 1.17647058823529% | 0.277012165 |
| Photosynthesis | ko00195 | 15 out of 1171 1.28095644748079% | 15 out of 1275 1.17647058823529% | 0.277012165 |
| Valine, leucine and isoleucine degradation | ko00280 | 15 out of 1171 1.28095644748079% | 15 out of 1275 1.17647058823529% | 0.277012165 |
| Phagosome | ko04145 | 14 out of 1171 1.19555935098207% | 14 out of 1275 1.09803921568627% | 0.301912135 |
| Porphyrin metabolism | ko00860 | 14 out of 1171 1.19555935098207% | 14 out of 1275 1.09803921568627% | 0.301912135 |
| RNA degradation | ko03018 | 14 out of 1171 1.19555935098207% | 14 out of 1275 1.09803921568627% | 0.301912135 |
| Flavone and flavonol biosynthesis | ko00944 | 14 out of 1171 1.19555935098207% | 14 out of 1275 1.09803921568627% | 0.301912135 |
| Purine metabolism | ko00230 | 14 out of 1171 1.19555935098207% | 14 out of 1275 1.09803921568627% | 0.301912135 |
| 2-Oxocarboxylic acid metabolism | ko01210 | 13 out of 1171 1.11016225448335% | 13 out of 1275 1.01960784313725% | 0.329026869 |
| Spliceosome | ko03040 | 12 out of 1171 1.02476515798463% | 12 out of 1275 0.941176470588235% | 0.358551282 |
| ABC transporters | ko02010 | 12 out of 1171 1.02476515798463% | 12 out of 1275 0.941176470588235% | 0.358551282 |
| Lysine degradation | ko00310 | 11 out of 1171 0.939368061485909% | 11 out of 1275 0.862745098039216% | 0.390697259 |
| Phosphatidylinositol signaling system | ko04070 | 11 out of 1171 0.939368061485909% | 11 out of 1275 0.862745098039216% | 0.390697259 |
| Sulfur metabolism | ko00920 | 11 out of 1171 0.939368061485909% | 11 out of 1275 0.862745098039216% | 0.390697259 |
| Aminoacyl-tRNA biosynthesis | ko00970 | 11 out of 1171 0.939368061485909% | 11 out of 1275 0.862745098039216% | 0.390697259 |
| Endocytosis | ko04144 | 23 out of 1171 1.96413321947054% | 24 out of 1275 1.88235294117647% | 0.403923686 |
| Ascorbate and aldarate metabolism | ko00053 | 10 out of 1171 0.85397096498719% | 10 out of 1275 0.784313725490196% | 0.425695119 |
| Photosynthesis - antenna proteins | ko00196 | 10 out of 1171 0.85397096498719% | 10 out of 1275 0.784313725490196% | 0.425695119 |
| Arginine biosynthesis | ko00220 | 10 out of 1171 0.85397096498719% | 10 out of 1275 0.784313725490196% | 0.425695119 |
| Isoquinoline alkaloid biosynthesis | ko00950 | 10 out of 1171 0.85397096498719% | 10 out of 1275 0.784313725490196% | 0.425695119 |
| Biosynthesis of amino acids | ko01230 | 68 out of 1171 5.80700256191289% | 73 out of 1275 5.72549019607843% | 0.442048859 |
| Lipoic acid metabolism | ko00785 | 9 out of 1171 0.768573868488471% | 9 out of 1275 0.705882352941177% | 0.463795198 |
| ATP-dependent chromatin remodeling | ko03082 | 9 out of 1171 0.768573868488471% | 9 out of 1275 0.705882352941177% | 0.463795198 |
| Propanoate metabolism | ko00640 | 8 out of 1171 0.683176771989752% | 8 out of 1275 0.627450980392157% | 0.505269575 |
| Thiamine metabolism | ko00730 | 8 out of 1171 0.683176771989752% | 8 out of 1275 0.627450980392157% | 0.505269575 |
| Glycerolipid metabolism | ko00561 | 18 out of 1171 1.53714773697694% | 19 out of 1275 1.49019607843137% | 0.532399744 |
| Vitamin B6 metabolism | ko00750 | 7 out of 1171 0.597779675491033% | 7 out of 1275 0.549019607843137% | 0.550413936 |
| Base excision repair | ko03410 | 7 out of 1171 0.597779675491033% | 7 out of 1275 0.549019607843137% | 0.550413936 |
| Biosynthesis of unsaturated fatty acids | ko01040 | 7 out of 1171 0.597779675491033% | 7 out of 1275 0.549019607843137% | 0.550413936 |
| Ribosome biogenesis in eukaryotes | ko03008 | 7 out of 1171 0.597779675491033% | 7 out of 1275 0.549019607843137% | 0.550413936 |
| Fructose and mannose metabolism | ko00051 | 26 out of 1171 2.2203245089667% | 28 out of 1275 2.19607843137255% | 0.596552951 |
| Basal transcription factors | ko03022 | 6 out of 1171 0.512382578992314% | 6 out of 1275 0.470588235294118% | 0.599549601 |
| Nucleocytoplasmic transport | ko03013 | 6 out of 1171 0.512382578992314% | 6 out of 1275 0.470588235294118% | 0.599549601 |
| Fatty acid biosynthesis | ko00061 | 6 out of 1171 0.512382578992314% | 6 out of 1275 0.470588235294118% | 0.599549601 |
| SNARE interactions in vesicular transport | ko04130 | 6 out of 1171 0.512382578992314% | 6 out of 1275 0.470588235294118% | 0.599549601 |
| Riboflavin metabolism | ko00740 | 6 out of 1171 0.512382578992314% | 6 out of 1275 0.470588235294118% | 0.599549601 |
| Starch and sucrose metabolism | ko00500 | 68 out of 1171 5.80700256191289% | 74 out of 1275 5.80392156862745% | 0.60062528 |
| Oxidative phosphorylation | ko00190 | 5 out of 1171 0.426985482493595% | 5 out of 1275 0.392156862745098% | 0.653025723 |
| Histidine metabolism | ko00340 | 5 out of 1171 0.426985482493595% | 5 out of 1275 0.392156862745098% | 0.653025723 |
| Autophagy - other | ko04136 | 5 out of 1171 0.426985482493595% | 5 out of 1275 0.392156862745098% | 0.653025723 |
| Tryptophan metabolism | ko00380 | 13 out of 1171 1.11016225448335% | 14 out of 1275 1.09803921568627% | 0.681518412 |
| Glycosaminoglycan degradation | ko00531 | 4 out of 1171 0.341588385994876% | 4 out of 1275 0.313725490196078% | 0.711221674 |
| Other glycan degradation | ko00511 | 4 out of 1171 0.341588385994876% | 4 out of 1275 0.313725490196078% | 0.711221674 |
| Pantothenate and CoA biosynthesis | ko00770 | 4 out of 1171 0.341588385994876% | 4 out of 1275 0.313725490196078% | 0.711221674 |
| Polycomb repressive complex | ko03083 | 4 out of 1171 0.341588385994876% | 4 out of 1275 0.313725490196078% | 0.711221674 |
| Arachidonic acid metabolism | ko00590 | 4 out of 1171 0.341588385994876% | 4 out of 1275 0.313725490196078% | 0.711221674 |
| Biotin metabolism | ko00780 | 4 out of 1171 0.341588385994876% | 4 out of 1275 0.313725490196078% | 0.711221674 |
| Arginine and proline metabolism | ko00330 | 20 out of 1171 1.70794192997438% | 22 out of 1275 1.72549019607843% | 0.735364841 |
| Peroxisome | ko04146 | 19 out of 1171 1.62254483347566% | 21 out of 1275 1.64705882352941% | 0.758137865 |
| Sulfur relay system | ko04122 | 3 out of 1171 0.256191289496157% | 3 out of 1275 0.235294117647059% | 0.774549631 |
| Neomycin, kanamycin and gentamicin biosynthesis | ko00524 | 3 out of 1171 0.256191289496157% | 3 out of 1275 0.235294117647059% | 0.774549631 |
| Exopolysaccharide biosynthesis | ko00543 | 3 out of 1171 0.256191289496157% | 3 out of 1275 0.235294117647059% | 0.774549631 |
| Steroid biosynthesis | ko00100 | 3 out of 1171 0.256191289496157% | 3 out of 1275 0.235294117647059% | 0.774549631 |
| Protein export | ko03060 | 3 out of 1171 0.256191289496157% | 3 out of 1275 0.235294117647059% | 0.774549631 |
| One carbon pool by folate | ko00670 | 3 out of 1171 0.256191289496157% | 3 out of 1275 0.235294117647059% | 0.774549631 |
| Nicotinate and nicotinamide metabolism | ko00760 | 3 out of 1171 0.256191289496157% | 3 out of 1275 0.235294117647059% | 0.774549631 |
| Phenylalanine metabolism | ko00360 | 10 out of 1171 0.85397096498719% | 11 out of 1275 0.862745098039216% | 0.775673714 |
| Fatty acid elongation | ko00062 | 10 out of 1171 0.85397096498719% | 11 out of 1275 0.862745098039216% | 0.775673714 |
| Glycerophospholipid metabolism | ko00564 | 18 out of 1171 1.53714773697694% | 20 out of 1275 1.56862745098039% | 0.780546905 |
| Zeatin biosynthesis | ko00908 | 17 out of 1171 1.45175064047822% | 19 out of 1275 1.49019607843137% | 0.802480189 |
| Carotenoid biosynthesis | ko00906 | 9 out of 1171 0.768573868488471% | 10 out of 1275 0.784313725490196% | 0.806695913 |
| Ribosome | ko03010 | 9 out of 1171 0.768573868488471% | 10 out of 1275 0.784313725490196% | 0.806695913 |
| Glutathione metabolism | ko00480 | 34 out of 1171 2.90350128095645% | 38 out of 1275 2.98039215686275% | 0.807800344 |
| MAPK signaling pathway - plant | ko04016 | 43 out of 1171 3.67207514944492% | 48 out of 1275 3.76470588235294% | 0.80903576 |
| Plant hormone signal transduction | ko04075 | 42 out of 1171 3.5866780529462% | 47 out of 1275 3.68627450980392% | 0.821772477 |
| Viral life cycle - HIV-1 | ko03250 | 2 out of 1171 0.170794192997438% | 2 out of 1275 0.156862745098039% | 0.843457383 |
| N-Glycan biosynthesis | ko00510 | 2 out of 1171 0.170794192997438% | 2 out of 1275 0.156862745098039% | 0.843457383 |
| Phosphonate and phosphinate metabolism | ko00440 | 2 out of 1171 0.170794192997438% | 2 out of 1275 0.156862745098039% | 0.843457383 |
| Non-homologous end-joining | ko03450 | 2 out of 1171 0.170794192997438% | 2 out of 1275 0.156862745098039% | 0.843457383 |
| Galactose metabolism | ko00052 | 31 out of 1171 2.64730999146029% | 35 out of 1275 2.74509803921569% | 0.849785127 |
| Cysteine and methionine metabolism | ko00270 | 31 out of 1171 2.64730999146029% | 35 out of 1275 2.74509803921569% | 0.849785127 |
| Phenylpropanoid biosynthesis | ko00940 | 75 out of 1171 6.40478223740393% | 84 out of 1275 6.58823529411765% | 0.861655053 |
| Inositol phosphate metabolism | ko00562 | 13 out of 1171 1.11016225448335% | 15 out of 1275 1.17647058823529% | 0.883061951 |
| Metabolic pathways | ko01100 | 737 out of 1171 62.9376601195559% | 808 out of 1275 63.3725490196078% | 0.883272646 |
| Phenylalanine, tyrosine and tryptophan biosynthesis | ko00400 | 20 out of 1171 1.70794192997438% | 23 out of 1275 1.80392156862745% | 0.888905551 |
| Brassinosteroid biosynthesis | ko00905 | 6 out of 1171 0.512382578992314% | 7 out of 1275 0.549019607843137% | 0.894363589 |
| Amino sugar and nucleotide sugar metabolism | ko00520 | 43 out of 1171 3.67207514944492% | 49 out of 1275 3.84313725490196% | 0.902791283 |
| Other types of O-glycan biosynthesis | ko00514 | 1 out of 1171 0.085397096498719% | 1 out of 1275 0.0784313725490196% | 0.918431373 |
| Valine, leucine and isoleucine biosynthesis | ko00290 | 1 out of 1171 0.085397096498719% | 1 out of 1275 0.0784313725490196% | 0.918431373 |
| Glycosphingolipid biosynthesis - lacto and neolacto series | ko00601 | 1 out of 1171 0.085397096498719% | 1 out of 1275 0.0784313725490196% | 0.918431373 |
| Glycosphingolipid biosynthesis - ganglio series | ko00604 | 1 out of 1171 0.085397096498719% | 1 out of 1275 0.0784313725490196% | 0.918431373 |
| Various types of N-glycan biosynthesis | ko00513 | 1 out of 1171 0.085397096498719% | 1 out of 1275 0.0784313725490196% | 0.918431373 |
| Sesquiterpenoid and triterpenoid biosynthesis | ko00909 | 1 out of 1171 0.085397096498719% | 1 out of 1275 0.0784313725490196% | 0.918431373 |
| Proteasome | ko03050 | 1 out of 1171 0.085397096498719% | 1 out of 1275 0.0784313725490196% | 0.918431373 |
| RNA polymerase | ko03020 | 1 out of 1171 0.085397096498719% | 1 out of 1275 0.0784313725490196% | 0.918431373 |
| Cyanoamino acid metabolism | ko00460 | 25 out of 1171 2.13492741246798% | 29 out of 1275 2.27450980392157% | 0.919390087 |
| Betalain biosynthesis | ko00965 | 5 out of 1171 0.426985482493595% | 6 out of 1275 0.470588235294118% | 0.920406334 |
| DNA replication | ko03030 | 5 out of 1171 0.426985482493595% | 6 out of 1275 0.470588235294118% | 0.920406334 |
| Nucleotide excision repair | ko03420 | 5 out of 1171 0.426985482493595% | 6 out of 1275 0.470588235294118% | 0.920406334 |
| Glycosphingolipid biosynthesis - globo and isoglobo series | ko00603 | 5 out of 1171 0.426985482493595% | 6 out of 1275 0.470588235294118% | 0.920406334 |
| Folate biosynthesis | ko00790 | 5 out of 1171 0.426985482493595% | 6 out of 1275 0.470588235294118% | 0.920406334 |
| Biosynthesis of secondary metabolites | ko01110 | 523 out of 1171 44.6626814688301% | 577 out of 1275 45.2549019607843% | 0.936512088 |
| Homologous recombination | ko03440 | 4 out of 1171 0.341588385994876% | 5 out of 1275 0.392156862745098% | 0.944005479 |
| Selenocompound metabolism | ko00450 | 4 out of 1171 0.341588385994876% | 5 out of 1275 0.392156862745098% | 0.944005479 |
| beta-Alanine metabolism | ko00410 | 15 out of 1171 1.28095644748079% | 18 out of 1275 1.41176470588235% | 0.947581509 |
| Linoleic acid metabolism | ko00591 | 15 out of 1171 1.28095644748079% | 18 out of 1275 1.41176470588235% | 0.947581509 |
| Monoterpenoid biosynthesis | ko00902 | 8 out of 1171 0.683176771989752% | 10 out of 1275 0.784313725490196% | 0.958539308 |
| Biosynthesis of various plant secondary metabolites | ko00999 | 19 out of 1171 1.62254483347566% | 23 out of 1275 1.80392156862745% | 0.966308995 |
| Mismatch repair | ko03430 | 2 out of 1171 0.170794192997438% | 3 out of 1275 0.235294117647059% | 0.981272886 |
| Taurine and hypotaurine metabolism | ko00430 | 10 out of 1171 0.85397096498719% | 13 out of 1275 1.01960784313725% | 0.983197281 |
| Sphingolipid metabolism | ko00600 | 15 out of 1171 1.28095644748079% | 19 out of 1275 1.49019607843137% | 0.984872178 |
| Alanine, aspartate and glutamate metabolism | ko00250 | 15 out of 1171 1.28095644748079% | 19 out of 1275 1.49019607843137% | 0.984872178 |
| Cutin, suberine and wax biosynthesis | ko00073 | 19 out of 1171 1.62254483347566% | 24 out of 1275 1.88235294117647% | 0.989802053 |
| Butanoate metabolism | ko00650 | 7 out of 1171 0.597779675491033% | 10 out of 1275 0.784313725490196% | 0.994021614 |
| Ether lipid metabolism | ko00565 | 3 out of 1171 0.256191289496157% | 5 out of 1275 0.392156862745098% | 0.995325539 |
| Terpenoid backbone biosynthesis | ko00900 | 28 out of 1171 2.39111870196413% | 37 out of 1275 2.90196078431373% | 0.999545003 |
| Diterpenoid biosynthesis | ko00904 | 25 out of 1171 2.13492741246798% | 35 out of 1275 2.74509803921569% | 0.999950022 |
| Pentose and glucuronate interconversions | ko00040 | 17 out of 1171 1.45175064047822% | 26 out of 1275 2.03921568627451% | 0.999985017 |

Table S8 The KEGG enrichment analysis results comparing T35CK and T35Z treatments are summarized.

| **#Kegg_pathway** | **ko_id** | **Cluter_frequency** | **Genome_frequency** | **P-value** |
| --- | --- | --- | --- | --- |
| Carbon metabolism | ko01200 | 139 out of 1535 9.05537459283388% | 141 out of 1713 8.23117338003503% | 1.68E-05 |
| Biosynthesis of cofactors | ko01240 | 76 out of 1535 4.95114006514658% | 78 out of 1713 4.55341506129597% | 0.008561117 |
| Glyoxylate and dicarboxylate metabolism | ko00630 | 41 out of 1535 2.67100977198697% | 41 out of 1713 2.39346176298891% | 0.010516578 |
| Biosynthesis of amino acids | ko01230 | 90 out of 1535 5.86319218241042% | 94 out of 1713 5.48744892002335% | 0.024584417 |
| Starch and sucrose metabolism | ko00500 | 84 out of 1535 5.47231270358306% | 88 out of 1713 5.13718622300058% | 0.038307042 |
| Protein processing in endoplasmic reticulum | ko04141 | 57 out of 1535 3.71335504885993% | 59 out of 1713 3.44424985405721% | 0.044987447 |
| Ribosome | ko03010 | 28 out of 1535 1.82410423452769% | 28 out of 1713 1.63455925277291% | 0.045142753 |
| Citrate cycle (TCA cycle) | ko00020 | 27 out of 1535 1.75895765472313% | 27 out of 1713 1.57618213660245% | 0.050471274 |
| Pentose phosphate pathway | ko00030 | 27 out of 1535 1.75895765472313% | 27 out of 1713 1.57618213660245% | 0.050471274 |
| Biosynthesis of nucleotide sugars | ko01250 | 27 out of 1535 1.75895765472313% | 27 out of 1713 1.57618213660245% | 0.050471274 |
| Purine metabolism | ko00230 | 26 out of 1535 1.69381107491857% | 26 out of 1713 1.51780502043199% | 0.05642481 |
| Porphyrin metabolism | ko00860 | 26 out of 1535 1.69381107491857% | 26 out of 1713 1.51780502043199% | 0.05642481 |
| Aminoacyl-tRNA biosynthesis | ko00970 | 25 out of 1535 1.62866449511401% | 25 out of 1713 1.45942790426153% | 0.063076212 |
| Carbon fixation in photosynthetic organisms | ko00710 | 50 out of 1535 3.25732899022801% | 52 out of 1713 3.03561004086398% | 0.079548573 |
| Glycolysis / Gluconeogenesis | ko00010 | 73 out of 1535 4.7557003257329% | 77 out of 1713 4.49503794512551% | 0.082549226 |
| 2-Oxocarboxylic acid metabolism | ko01210 | 22 out of 1535 1.43322475570033% | 22 out of 1713 1.28429655575015% | 0.088078596 |
| RNA degradation | ko03018 | 19 out of 1535 1.23778501628665% | 19 out of 1713 1.10916520723876% | 0.122914578 |
| Metabolic pathways | ko01100 | 961 out of 1535 62.6058631921824% | 1064 out of 1713 62.1132516053707% | 0.124844084 |
| Phenylalanine metabolism | ko00360 | 18 out of 1535 1.17263843648208% | 18 out of 1713 1.0507880910683% | 0.137336988 |
| mRNA surveillance pathway | ko03015 | 18 out of 1535 1.17263843648208% | 18 out of 1713 1.0507880910683% | 0.137336988 |
| Flavone and flavonol biosynthesis | ko00944 | 18 out of 1535 1.17263843648208% | 18 out of 1713 1.0507880910683% | 0.137336988 |
| Photosynthesis | ko00195 | 18 out of 1535 1.17263843648208% | 18 out of 1713 1.0507880910683% | 0.137336988 |
| Flavonoid biosynthesis | ko00941 | 74 out of 1535 4.82084690553746% | 79 out of 1713 4.61179217746643% | 0.151977222 |
| Tyrosine metabolism | ko00350 | 41 out of 1535 2.67100977198697% | 43 out of 1713 2.51021599532983% | 0.158527304 |
| Oxidative phosphorylation | ko00190 | 16 out of 1535 1.04234527687296% | 16 out of 1713 0.934033858727379% | 0.171421647 |
| Isoquinoline alkaloid biosynthesis | ko00950 | 16 out of 1535 1.04234527687296% | 16 out of 1713 0.934033858727379% | 0.171421647 |
| Lipoic acid metabolism | ko00785 | 16 out of 1535 1.04234527687296% | 16 out of 1713 0.934033858727379% | 0.171421647 |
| Fructose and mannose metabolism | ko00051 | 28 out of 1535 1.82410423452769% | 29 out of 1713 1.69293636894337% | 0.178668853 |
| MAPK signaling pathway - plant | ko04016 | 50 out of 1535 3.25732899022801% | 53 out of 1713 3.09398715703444% | 0.181218049 |
| Phagosome | ko04145 | 26 out of 1535 1.69381107491857% | 27 out of 1713 1.57618213660245% | 0.211216762 |
| Propanoate metabolism | ko00640 | 14 out of 1535 0.912052117263844% | 14 out of 1713 0.817279626386456% | 0.213906472 |
| Polycomb repressive complex | ko03083 | 14 out of 1535 0.912052117263844% | 14 out of 1713 0.817279626386456% | 0.213906472 |
| Arginine biosynthesis | ko00220 | 14 out of 1535 0.912052117263844% | 14 out of 1713 0.817279626386456% | 0.213906472 |
| Protein export | ko03060 | 14 out of 1535 0.912052117263844% | 14 out of 1713 0.817279626386456% | 0.213906472 |
| Phosphatidylinositol signaling system | ko04070 | 14 out of 1535 0.912052117263844% | 14 out of 1713 0.817279626386456% | 0.213906472 |
| Pyruvate metabolism | ko00620 | 47 out of 1535 3.06188925081433% | 50 out of 1713 2.91885580852306% | 0.219042176 |
| Endocytosis | ko04144 | 36 out of 1535 2.34527687296417% | 38 out of 1713 2.21833041447752% | 0.226606666 |
| Nucleotide metabolism | ko01232 | 25 out of 1535 1.62866449511401% | 26 out of 1713 1.51780502043199% | 0.229361249 |
| Spliceosome | ko03040 | 13 out of 1535 0.846905537459283% | 13 out of 1713 0.758902510215995% | 0.238923129 |
| Sulfur metabolism | ko00920 | 13 out of 1535 0.846905537459283% | 13 out of 1713 0.758902510215995% | 0.238923129 |
| Ubiquinone and other terpenoid-quinone biosynthesis | ko00130 | 23 out of 1535 1.49837133550489% | 24 out of 1713 1.40105078809107% | 0.269716355 |
| Tropane, piperidine and pyridine alkaloid biosynthesis | ko00960 | 22 out of 1535 1.43322475570033% | 23 out of 1713 1.34267367192061% | 0.292050081 |
| Glycerolipid metabolism | ko00561 | 22 out of 1535 1.43322475570033% | 23 out of 1713 1.34267367192061% | 0.292050081 |
| Thiamine metabolism | ko00730 | 11 out of 1535 0.716612377850163% | 11 out of 1713 0.642148277875073% | 0.298014361 |
| Nitrogen metabolism | ko00910 | 11 out of 1535 0.716612377850163% | 11 out of 1713 0.642148277875073% | 0.298014361 |
| Ribosome biogenesis in eukaryotes | ko03008 | 11 out of 1535 0.716612377850163% | 11 out of 1713 0.642148277875073% | 0.298014361 |
| ATP-dependent chromatin remodeling | ko03082 | 11 out of 1535 0.716612377850163% | 11 out of 1713 0.642148277875073% | 0.298014361 |
| Pyrimidine metabolism | ko00240 | 21 out of 1535 1.36807817589577% | 22 out of 1713 1.28429655575015% | 0.315896153 |
| Alanine, aspartate and glutamate metabolism | ko00250 | 21 out of 1535 1.36807817589577% | 22 out of 1713 1.28429655575015% | 0.315896153 |
| beta-Alanine metabolism | ko00410 | 31 out of 1535 2.01954397394137% | 33 out of 1713 1.92644483362522% | 0.316661726 |
| Folate biosynthesis | ko00790 | 10 out of 1535 0.651465798045603% | 10 out of 1713 0.583771161704612% | 0.332798988 |
| Fatty acid elongation | ko00062 | 10 out of 1535 0.651465798045603% | 10 out of 1713 0.583771161704612% | 0.332798988 |
| Photosynthesis - antenna proteins | ko00196 | 10 out of 1535 0.651465798045603% | 10 out of 1713 0.583771161704612% | 0.332798988 |
| Peroxisome | ko04146 | 30 out of 1535 1.95439739413681% | 32 out of 1713 1.86806771745476% | 0.337530362 |
| Stilbenoid, diarylheptanoid and gingerol biosynthesis | ko00945 | 20 out of 1535 1.30293159609121% | 21 out of 1713 1.22591943957968% | 0.341302629 |
| Valine, leucine and isoleucine degradation | ko00280 | 20 out of 1535 1.30293159609121% | 21 out of 1713 1.22591943957968% | 0.341302629 |
| Glycine, serine and threonine metabolism | ko00260 | 29 out of 1535 1.88925081433225% | 31 out of 1713 1.8096906012843% | 0.359368121 |
| Inositol phosphate metabolism | ko00562 | 19 out of 1535 1.23778501628665% | 20 out of 1713 1.16754232340922% | 0.368308382 |
| Plant-pathogen interaction | ko04626 | 74 out of 1535 4.82084690553746% | 81 out of 1713 4.72854640980736% | 0.381735777 |
| Motor proteins | ko04814 | 28 out of 1535 1.82410423452769% | 30 out of 1713 1.75131348511384% | 0.382169291 |
| Circadian rhythm - plant | ko04712 | 18 out of 1535 1.17263843648208% | 19 out of 1713 1.10916520723876% | 0.396940356 |
| Tryptophan metabolism | ko00380 | 27 out of 1535 1.75895765472313% | 29 out of 1713 1.69293636894337% | 0.405920005 |
| Butanoate metabolism | ko00650 | 8 out of 1535 0.521172638436482% | 8 out of 1713 0.467016929363689% | 0.414937227 |
| Glycosaminoglycan degradation | ko00531 | 8 out of 1535 0.521172638436482% | 8 out of 1713 0.467016929363689% | 0.414937227 |
| Biotin metabolism | ko00780 | 8 out of 1535 0.521172638436482% | 8 out of 1713 0.467016929363689% | 0.414937227 |
| Basal transcription factors | ko03022 | 8 out of 1535 0.521172638436482% | 8 out of 1713 0.467016929363689% | 0.414937227 |
| Other glycan degradation | ko00511 | 8 out of 1535 0.521172638436482% | 8 out of 1713 0.467016929363689% | 0.414937227 |
| Riboflavin metabolism | ko00740 | 8 out of 1535 0.521172638436482% | 8 out of 1713 0.467016929363689% | 0.414937227 |
| Ubiquitin mediated proteolysis | ko04120 | 17 out of 1535 1.10749185667752% | 18 out of 1713 1.0507880910683% | 0.427210314 |
| Various types of N-glycan biosynthesis | ko00513 | 7 out of 1535 0.456026058631922% | 7 out of 1713 0.408639813193228% | 0.463274156 |
| Base excision repair | ko03410 | 7 out of 1535 0.456026058631922% | 7 out of 1713 0.408639813193228% | 0.463274156 |
| Fatty acid degradation | ko00071 | 33 out of 1535 2.14983713355049% | 36 out of 1713 2.1015761821366% | 0.47575075 |
| Glycerophospholipid metabolism | ko00564 | 33 out of 1535 2.14983713355049% | 36 out of 1713 2.1015761821366% | 0.47575075 |
| Pantothenate and CoA biosynthesis | ko00770 | 15 out of 1535 0.977198697068404% | 16 out of 1713 0.934033858727379% | 0.492611679 |
| Galactose metabolism | ko00052 | 40 out of 1535 2.60586319218241% | 44 out of 1713 2.56859311150029% | 0.511374339 |
| SNARE interactions in vesicular transport | ko04130 | 6 out of 1535 0.390879478827362% | 6 out of 1713 0.350262697022767% | 0.51720666 |
| Autophagy - other | ko04136 | 6 out of 1535 0.390879478827362% | 6 out of 1713 0.350262697022767% | 0.51720666 |
| Steroid biosynthesis | ko00100 | 6 out of 1535 0.390879478827362% | 6 out of 1713 0.350262697022767% | 0.51720666 |
| Sphingolipid metabolism | ko00600 | 14 out of 1535 0.912052117263844% | 15 out of 1713 0.875656742556918% | 0.527652751 |
| Lysine degradation | ko00310 | 14 out of 1535 0.912052117263844% | 15 out of 1713 0.875656742556918% | 0.527652751 |
| Arginine and proline metabolism | ko00330 | 30 out of 1535 1.95439739413681% | 33 out of 1713 1.92644483362522% | 0.54621672 |
| N-Glycan biosynthesis | ko00510 | 5 out of 1535 0.325732899022801% | 5 out of 1713 0.291885580852306% | 0.577378415 |
| Nicotinate and nicotinamide metabolism | ko00760 | 4 out of 1535 0.260586319218241% | 4 out of 1713 0.233508464681845% | 0.64450667 |
| One carbon pool by folate | ko00670 | 4 out of 1535 0.260586319218241% | 4 out of 1713 0.233508464681845% | 0.64450667 |
| Valine, leucine and isoleucine biosynthesis | ko00290 | 4 out of 1535 0.260586319218241% | 4 out of 1713 0.233508464681845% | 0.64450667 |
| Viral life cycle - HIV-1 | ko03250 | 4 out of 1535 0.260586319218241% | 4 out of 1713 0.233508464681845% | 0.64450667 |
| Neomycin, kanamycin and gentamicin biosynthesis | ko00524 | 4 out of 1535 0.260586319218241% | 4 out of 1713 0.233508464681845% | 0.64450667 |
| Betalain biosynthesis | ko00965 | 4 out of 1535 0.260586319218241% | 4 out of 1713 0.233508464681845% | 0.64450667 |
| Plant hormone signal transduction | ko04075 | 57 out of 1535 3.71335504885993% | 64 out of 1713 3.73613543490952% | 0.655099033 |
| Biosynthesis of various plant secondary metabolites | ko00999 | 25 out of 1535 1.62866449511401% | 28 out of 1713 1.63455925277291% | 0.669252062 |
| Glutathione metabolism | ko00480 | 48 out of 1535 3.12703583061889% | 54 out of 1713 3.1523642732049% | 0.673463243 |
| Amino sugar and nucleotide sugar metabolism | ko00520 | 63 out of 1535 4.1042345276873% | 71 out of 1713 4.14477524810274% | 0.685839554 |
| Ascorbate and aldarate metabolism | ko00053 | 16 out of 1535 1.04234527687296% | 18 out of 1713 1.0507880910683% | 0.71431658 |
| Phenylalanine, tyrosine and tryptophan biosynthesis | ko00400 | 23 out of 1535 1.49837133550489% | 26 out of 1713 1.51780502043199% | 0.718392246 |
| Nucleocytoplasmic transport | ko03013 | 3 out of 1535 0.195439739413681% | 3 out of 1713 0.175131348511384% | 0.719390604 |
| Non-homologous end-joining | ko03450 | 3 out of 1535 0.195439739413681% | 3 out of 1713 0.175131348511384% | 0.719390604 |
| Vitamin B6 metabolism | ko00750 | 3 out of 1535 0.195439739413681% | 3 out of 1713 0.175131348511384% | 0.719390604 |
| Homologous recombination | ko03440 | 9 out of 1535 0.586319218241042% | 10 out of 1713 0.583771161704612% | 0.720991779 |
| Selenocompound metabolism | ko00450 | 9 out of 1535 0.586319218241042% | 10 out of 1713 0.583771161704612% | 0.720991779 |
| Taurine and hypotaurine metabolism | ko00430 | 9 out of 1535 0.586319218241042% | 10 out of 1713 0.583771161704612% | 0.720991779 |
| Glycosphingolipid biosynthesis - globo and isoglobo series | ko00603 | 8 out of 1535 0.521172638436482% | 9 out of 1713 0.525394045534151% | 0.761488906 |
| ABC transporters | ko02010 | 13 out of 1535 0.846905537459283% | 15 out of 1713 0.875656742556918% | 0.801304648 |
| Glycosphingolipid biosynthesis - ganglio series | ko00604 | 2 out of 1535 0.130293159609121% | 2 out of 1713 0.116754232340922% | 0.802920629 |
| Caffeine metabolism | ko00232 | 2 out of 1535 0.130293159609121% | 2 out of 1713 0.116754232340922% | 0.802920629 |
| Exopolysaccharide biosynthesis | ko00543 | 2 out of 1535 0.130293159609121% | 2 out of 1713 0.116754232340922% | 0.802920629 |
| Proteasome | ko03050 | 2 out of 1535 0.130293159609121% | 2 out of 1713 0.116754232340922% | 0.802920629 |
| Monobactam biosynthesis | ko00261 | 2 out of 1535 0.130293159609121% | 2 out of 1713 0.116754232340922% | 0.802920629 |
| Sulfur relay system | ko04122 | 2 out of 1535 0.130293159609121% | 2 out of 1713 0.116754232340922% | 0.802920629 |
| Phosphonate and phosphinate metabolism | ko00440 | 2 out of 1535 0.130293159609121% | 2 out of 1713 0.116754232340922% | 0.802920629 |
| Sesquiterpenoid and triterpenoid biosynthesis | ko00909 | 2 out of 1535 0.130293159609121% | 2 out of 1713 0.116754232340922% | 0.802920629 |
| RNA polymerase | ko03020 | 2 out of 1535 0.130293159609121% | 2 out of 1713 0.116754232340922% | 0.802920629 |
| Cyanoamino acid metabolism | ko00460 | 32 out of 1535 2.08469055374593% | 37 out of 1713 2.15995329830706% | 0.82089411 |
| Fatty acid metabolism | ko01212 | 25 out of 1535 1.62866449511401% | 29 out of 1713 1.69293636894337% | 0.824165288 |
| Arachidonic acid metabolism | ko00590 | 6 out of 1535 0.390879478827362% | 7 out of 1713 0.408639813193228% | 0.840801688 |
| Brassinosteroid biosynthesis | ko00905 | 6 out of 1535 0.390879478827362% | 7 out of 1713 0.408639813193228% | 0.840801688 |
| Nucleotide excision repair | ko03420 | 6 out of 1535 0.390879478827362% | 7 out of 1713 0.408639813193228% | 0.840801688 |
| alpha-Linolenic acid metabolism | ko00592 | 30 out of 1535 1.95439739413681% | 35 out of 1713 2.04319906596614% | 0.851938274 |
| Histidine metabolism | ko00340 | 5 out of 1535 0.325732899022801% | 6 out of 1713 0.350262697022767% | 0.878237192 |
| Carotenoid biosynthesis | ko00906 | 15 out of 1535 0.977198697068404% | 18 out of 1713 1.0507880910683% | 0.891617292 |
| C5-Branched dibasic acid metabolism | ko00660 | 1 out of 1535 0.0651465798045603% | 1 out of 1713 0.0583771161704612% | 0.896088733 |
| Lysine biosynthesis | ko00300 | 1 out of 1535 0.0651465798045603% | 1 out of 1713 0.0583771161704612% | 0.896088733 |
| Cysteine and methionine metabolism | ko00270 | 39 out of 1535 2.54071661237785% | 46 out of 1713 2.68534734384121% | 0.9032193 |
| Biosynthesis of secondary metabolites | ko01110 | 641 out of 1535 41.7589576547231% | 724 out of 1713 42.2650321074139% | 0.907124155 |
| Terpenoid backbone biosynthesis | ko00900 | 32 out of 1535 2.08469055374593% | 38 out of 1713 2.21833041447752% | 0.908564658 |
| Ether lipid metabolism | ko00565 | 13 out of 1535 0.846905537459283% | 16 out of 1713 0.934033858727379% | 0.924217003 |
| Zeatin biosynthesis | ko00908 | 17 out of 1535 1.10749185667752% | 21 out of 1713 1.22591943957968% | 0.941308145 |
| DNA replication | ko03030 | 7 out of 1535 0.456026058631922% | 9 out of 1713 0.525394045534151% | 0.942135772 |
| Other types of O-glycan biosynthesis | ko00514 | 3 out of 1535 0.195439739413681% | 4 out of 1713 0.233508464681845% | 0.944042407 |
| Biosynthesis of unsaturated fatty acids | ko01040 | 11 out of 1535 0.716612377850163% | 14 out of 1713 0.817279626386456% | 0.950928791 |
| Mismatch repair | ko03430 | 6 out of 1535 0.390879478827362% | 8 out of 1713 0.467016929363689% | 0.958308793 |
| Glycosphingolipid biosynthesis - lacto and neolacto series | ko00601 | 2 out of 1535 0.130293159609121% | 3 out of 1713 0.175131348511384% | 0.969980678 |
| Cutin, suberine and wax biosynthesis | ko00073 | 24 out of 1535 1.56351791530945% | 30 out of 1713 1.75131348511384% | 0.970149411 |
| Fatty acid biosynthesis | ko00061 | 9 out of 1535 0.586319218241042% | 13 out of 1713 0.758902510215995% | 0.992638507 |
| Pentose and glucuronate interconversions | ko00040 | 19 out of 1535 1.23778501628665% | 26 out of 1713 1.51780502043199% | 0.996495092 |
| Phenylpropanoid biosynthesis | ko00940 | 75 out of 1535 4.88599348534202% | 94 out of 1713 5.48744892002335% | 0.998982036 |
| Monoterpenoid biosynthesis | ko00902 | 6 out of 1535 0.390879478827362% | 11 out of 1713 0.642148277875073% | 0.999656505 |
| Diterpenoid biosynthesis | ko00904 | 24 out of 1535 1.56351791530945% | 37 out of 1713 2.15995329830706% | 0.999992576 |
| Linoleic acid metabolism | ko00591 | 11 out of 1535 0.716612377850163% | 32 out of 1713 1.86806771745476% | 1 |
