# Supplementary material for: Response mechanism of carbon metabolism of Pinus massoniana to gradient high temperature and drought stress
Source: BMC Genomics. 2024 Feb 12;25:166. doi: 10.1186/s12864-024-10054-2 (PMC10860282; doi:10.1186/s12864-024-10054-2)
Supplement: Supplementary file 6 — Additional file 6. [file 12864_2024_10054_MOESM6_ESM.docx]

Table S9 Carbon metabolism enrichment pathway under T25CK and T25Z treatment

| **#Kegg_pathway** | **ko_id** | **Cluster_frequency** | **Genome_frequency** | **P-value** |
| --- | --- | --- | --- | --- |
| Glycolysis / Gluconeogenesis | ko00010 | 28 out of 640 4.375% | 28 out of 683 4.099560761347% | 0.155828944 |
| Galactose metabolism | ko00052 | 20 out of 640 3.125% | 20 out of 683 2.92825768667643% | 0.26724212 |
| Starch and sucrose metabolism | ko00500 | 53 out of 640 8.28125% | 55 out of 683 8.05270863836018% | 0.307545422 |
| Pyruvate metabolism | ko00620 | 15 out of 640 2.34375% | 15 out of 683 2.19619326500732% | 0.373109682 |
| Inositol phosphate metabolism | ko00562 | 11 out of 640 1.71875% | 11 out of 683 1.61054172767204% | 0.486381525 |
| Fructose and mannose metabolism | ko00051 | 10 out of 640 1.5625% | 10 out of 683 1.46412884333821% | 0.519578994 |
| Ascorbate and aldarate metabolism | ko00053 | 9 out of 640 1.40625% | 9 out of 683 1.31771595900439% | 0.55498612 |
| Glyoxylate and dicarboxylate metabolism | ko00630 | 7 out of 640 1.09375% | 7 out of 683 1.02489019033675% | 0.633011795 |
| Pentose phosphate pathway | ko00030 | 7 out of 640 1.09375% | 7 out of 683 1.02489019033675% | 0.633011795 |
| Propanoate metabolism | ko00640 | 3 out of 640 0.46875% | 3 out of 683 0.439238653001464% | 0.822525405 |
| Citrate cycle (TCA cycle) | ko00020 | 3 out of 640 0.46875% | 3 out of 683 0.439238653001464% | 0.822525405 |
| Amino sugar and nucleotide sugar metabolism | ko00520 | 22 out of 640 3.4375% | 25 out of 683 3.66032210834553% | 0.934663011 |
| Butanoate metabolism | ko00650 | 1 out of 640 0.15625% | 2 out of 683 0.292825768667643% | 0.996122849 |
