# Supplementary material for: Response mechanism of carbon metabolism of Pinus massoniana to gradient high temperature and drought stress
Source: BMC Genomics. 2024 Feb 12;25:166. doi: 10.1186/s12864-024-10054-2 (PMC10860282; doi:10.1186/s12864-024-10054-2)
Supplement: Supplementary file 7 — Additional file 7. [file 12864_2024_10054_MOESM7_ESM.docx]

Table S10 Carbon metabolism enrichment pathway under T30CK and T30Z treatment

| **#Kegg_pathway** | **ko_id** | **Cluster_frequency** | **Genome_frequency** | **P-value** |
| --- | --- | --- | --- | --- |
| Glycolysis / Gluconeogenesis | ko00010 | 58 out of 1171 4.9530315969257% | 58 out of 1275 4.54901960784314% | 0.006383739 |
| Pyruvate metabolism | ko00620 | 38 out of 1171 3.24508966695132% | 38 out of 1275 2.98039215686275% | 0.037502671 |
| Glyoxylate and dicarboxylate metabolism | ko00630 | 28 out of 1171 2.39111870196413% | 28 out of 1275 2.19607843137255% | 0.089887239 |
| Pentose phosphate pathway | ko00030 | 17 out of 1171 1.45175064047822% | 17 out of 1275 1.33333333333333% | 0.233153739 |
| Citrate cycle (TCA cycle) | ko00020 | 15 out of 1171 1.28095644748079% | 15 out of 1275 1.17647058823529% | 0.277012165 |
| Ascorbate and aldarate metabolism | ko00053 | 10 out of 1171 0.85397096498719% | 10 out of 1275 0.784313725490196% | 0.425695119 |
| Propanoate metabolism | ko00640 | 8 out of 1171 0.683176771989752% | 8 out of 1275 0.627450980392157% | 0.505269575 |
| Fructose and mannose metabolism | ko00051 | 26 out of 1171 2.2203245089667% | 28 out of 1275 2.19607843137255% | 0.596552951 |
| Starch and sucrose metabolism | ko00500 | 68 out of 1171 5.80700256191289% | 74 out of 1275 5.80392156862745% | 0.60062528 |
| Galactose metabolism | ko00052 | 31 out of 1171 2.64730999146029% | 35 out of 1275 2.74509803921569% | 0.849785127 |
| Inositol phosphate metabolism | ko00562 | 13 out of 1171 1.11016225448335% | 15 out of 1275 1.17647058823529% | 0.883061951 |
| Amino sugar and nucleotide sugar metabolism | ko00520 | 43 out of 1171 3.67207514944492% | 49 out of 1275 3.84313725490196% | 0.902791283 |
| Butanoate metabolism | ko00650 | 7 out of 1171 0.597779675491033% | 10 out of 1275 0.784313725490196% | 0.994021614 |
