# Supplementary material for: Response mechanism of carbon metabolism of Pinus massoniana to gradient high temperature and drought stress
Source: BMC Genomics. 2024 Feb 12;25:166. doi: 10.1186/s12864-024-10054-2 (PMC10860282; doi:10.1186/s12864-024-10054-2)
Supplement: Supplementary file 8 — Additional file 8. [file 12864_2024_10054_MOESM8_ESM.docx]

Table S11 Carbon metabolism enrichment pathway under T35CK and T35Z treatment

| #Kegg_pathway | ko_id | Cluster_frequency | Genome_frequency | P-value |
| --- | --- | --- | --- | --- |
| Glyoxylate and dicarboxylate metabolism | ko00630 | 41 out of 1535 2.67100977198697% | 41 out of 1713 2.39346176298891% | 0.010516578 |
| Starch and sucrose metabolism | ko00500 | 84 out of 1535 5.47231270358306% | 88 out of 1713 5.13718622300058% | 0.038307042 |
| Citrate cycle (TCA cycle) | ko00020 | 27 out of 1535 1.75895765472313% | 27 out of 1713 1.57618213660245% | 0.050471274 |
| Pentose phosphate pathway | ko00030 | 27 out of 1535 1.75895765472313% | 27 out of 1713 1.57618213660245% | 0.050471274 |
| Glycolysis / Gluconeogenesis | ko00010 | 73 out of 1535 4.7557003257329% | 77 out of 1713 4.49503794512551% | 0.082549226 |
| Fructose and mannose metabolism | ko00051 | 28 out of 1535 1.82410423452769% | 29 out of 1713 1.69293636894337% | 0.178668853 |
| Propanoate metabolism | ko00640 | 14 out of 1535 0.912052117263844% | 14 out of 1713 0.817279626386456% | 0.213906472 |
| Pyruvate metabolism | ko00620 | 47 out of 1535 3.06188925081433% | 50 out of 1713 2.91885580852306% | 0.219042176 |
| Inositol phosphate metabolism | ko00562 | 19 out of 1535 1.23778501628665% | 20 out of 1713 1.16754232340922% | 0.368308382 |
| Butanoate metabolism | ko00650 | 8 out of 1535 0.521172638436482% | 8 out of 1713 0.467016929363689% | 0.414937227 |
| Galactose metabolism | ko00052 | 40 out of 1535 2.60586319218241% | 44 out of 1713 2.56859311150029% | 0.511374339 |
| Amino sugar and nucleotide sugar metabolism | ko00520 | 63 out of 1535 4.1042345276873% | 71 out of 1713 4.14477524810274% | 0.685839554 |
| Ascorbate and aldarate metabolism | ko00053 | 16 out of 1535 1.04234527687296% | 18 out of 1713 1.0507880910683% | 0.71431658 |
