# Supplementary material for: Response mechanism of carbon metabolism of Pinus massoniana to gradient high temperature and drought stress
Source: BMC Genomics. 2024 Feb 12;25:166. doi: 10.1186/s12864-024-10054-2 (PMC10860282; doi:10.1186/s12864-024-10054-2)
Supplement: Supplementary file 9 — Additional file 9. [file 12864_2024_10054_MOESM9_ESM.docx]

Table S12 Co-enrichment of genes in starch and sucrose metabolic pathways under three treatments

| **id** | **KEGG** | **T25CK vs T25Z** | **T30CK vs T30Z** | **T35CK vs T35Z** |
| --- | --- | --- | --- | --- |
| PITA_27138 | K05349 | down | up | up |
| PITA_28625 | K05350 | up | down | down |
| PITA_48713 | K01193 | down | up | up |
| PITA_10586 | K00695 | up | up | up |
| PITA_10650 | K05349 | up | up | up |
| PITA_11281 | K01177 | up | up | up |
| PITA_27768 | K01177 | up | up | up |
| PITA_41038 | K00700 | up | up | up |
| PITA_41069 | K05349 | up | up | up |
| PITA_41530 | K01187 | up | up | up |
| PITA_41655 | K01177 | up | up | up |
| PITA_47736 | K01177 | up | up | up |
| PITA_04249 | K00975 | down | down | down |
| PITA_05060 | K16055 | down | down | down |
| PITA_05742 | K00695 | down | down | down |
| PITA_10269 | K01188 | down | down | down |
| PITA_15310 | K01177 | down | down | down |
| PITA_20321 | K00705 | down | down | down |
| PITA_22168 | K00695 | down | down | down |
| PITA_23271 | K05349 | down | down | down |
| PITA_24200 | K00695 | down | down | down |
| PITA_33988 | K19891 | down | down | down |
| PITA_34393 | K16055 | down | down | down |
| PITA_36401 | K01179 | down | down | down |
| PITA_37093 | K01188 | down | down | down |
| PITA_38689 | K00847 | down | down | down |
| PITA_48635 | K00695 | down | down | down |
