# Supplementary material for: Response mechanism of carbon metabolism of Pinus massoniana to gradient high temperature and drought stress
Source: BMC Genomics. 2024 Feb 12;25:166. doi: 10.1186/s12864-024-10054-2 (PMC10860282; doi:10.1186/s12864-024-10054-2)
Supplement: Supplementary file 10 — Additional file 10. [file 12864_2024_10054_MOESM10_ESM.docx]

Table S13 The metabolome summary table of 652 metabolites.

| **name** | **formula** | **KEGG** | **FC** | **log2FC** | **P.value** | **-log10(P.value)** |
| --- | --- | --- | --- | --- | --- | --- |
| Triethylamine | C6H15N | C14691 | 0.73 | -0.45 | 0.002164502 | 2.66 |
| Isovaleric acid | C5H10O2 | C08262 | 0.39 | -1.37 | 0.002164502 | 2.66 |
| 3-Methylthiopropanamine | C4H11NS | C03354 | 0.64 | -0.63 | 0.004329004 | 2.36 |
| 2-Phenylethanol | C8H10O | C05853 | 0.51 | -0.97 | 0.002164502 | 2.66 |
| Benzaldehyde | C7H6O | C00261 | 0.16 | -2.62 | 0.002164502 | 2.66 |
| m-Cresol | C7H8O | C01467 | 2.14 | 1.1 | 0.002164502 | 2.66 |
| Cytosine | C4H5N3O | C00380 | 1.18 | 0.24 | 0.002164502 | 2.66 |
| 5-Methyl-2-furancarboxaldehyde | C6H6O2 | C11115 | 0.49 | -1.04 | 0.002164502 | 2.66 |
| Histamine | C5H9N3 | C00388 | 1.29 | 0.37 | 0.025974026 | 1.59 |
| Imidazole-4-acetaldehyde | C5H6N2O | C05130 | 1.91 | 0.94 | 0.008658009 | 2.06 |
| Uracil | C4H4N2O2 | C00106 | 0.56 | -0.83 | 0.002164502 | 2.66 |
| Creatinine | C4H7N3O | C00791 | 0.53 | -0.92 | 0.002164502 | 2.66 |
| Deoxyribose | C5H10O4 | C01801 | 1.32 | 0.4 | 0.002164502 | 2.66 |
| 5-Hydroxypentanoic acid | C5H10O3 | C02804 | 0.06 | -4.17 | 0.002164502 | 2.66 |
| L-Allothreonine | C4H9NO3 | C05519 | 2.07 | 1.05 | 0.002164502 | 2.66 |
| Tyrosol | C8H10O2 | C06044 | 2.39 | 1.26 | 0.002164502 | 2.66 |
| N,N-Dimethylaniline | C8H11N | C02846 | 1.73 | 0.79 | 0.008658009 | 2.06 |
| Phenylethylamine | C8H11N | C05332 | 0.06 | -3.99 | 0.002164502 | 2.66 |
| Niacinamide | C6H6N2O | C00153 | 0.06 | -4.04 | 0.002164502 | 2.66 |
| 4-Hydroxybenzaldehyde | C7H6O2 | C00633 | 0.23 | -2.09 | 0.002164502 | 2.66 |
| Erythritol | C4H10O4 | C00503 | 2.59 | 1.38 | 0.002164502 | 2.66 |
| Taurine | C2H7NO3S | C00245 | 0.35 | -1.52 | 0.025974026 | 1.59 |
| Triacetate lactone | C6H6O3 | C02752 | 2.61 | 1.39 | 0.004329004 | 2.36 |
| Phloroglucinol | C6H6O3 | C02183 | 1.49 | 0.57 | 0.002164502 | 2.66 |
| Dihydrothymine | C5H8N2O2 | C00906 | 1.36 | 0.44 | 0.002164502 | 2.66 |
| Quinoline | C9H7N | C06413 | 3.97 | 1.99 | 0.002164502 | 2.66 |
| 1,1-Dimethylbiguanide | C4H11N5 | C07151 | 0.35 | -1.52 | 0.002164502 | 2.66 |
| Pipecolic acid | C6H11NO2 | C00408 | 1.87 | 0.9 | 0.002164502 | 2.66 |
| L-Isoleucine | C6H13NO2 | C00407 | 32.14 | 5.01 | 0.002164502 | 2.66 |
| 3-Methylindole | C9H9N | C08313 | 0.14 | -2.79 | 0.002164502 | 2.66 |
| L-Ribulose | C5H10O5 | C00310 | 1.32 | 0.4 | 0.002164502 | 2.66 |
| 1,3-Dihydro-(2H)-indol-2-one | C8H7NO | C12312 | 2.34 | 1.23 | 0.002164502 | 2.66 |
| L-Asparagine | C4H8N2O3 | C00152 | 1.22 | 0.29 | 0.015151515 | 1.82 |
| Isochavicol | C9H10O | C20464 | 0.64 | -0.63 | 0.002164502 | 2.66 |
| D-Xylitol | C5H12O5 | C00379 | 1.37 | 0.45 | 0.002164502 | 2.66 |
| 2-Phenylacetamide | C8H9NO | C02505 | 0.82 | -0.28 | 0.002164502 | 2.66 |
| Perillyl alcohol | C10H16O | C02452 | 1.69 | 0.76 | 0.002164502 | 2.66 |
| Chavicol | C9H10O | C16930 | 0.38 | -1.38 | 0.002164502 | 2.66 |
| (-)-3-Isothujone | C10H16O | C09906 | 1.49 | 0.57 | 0.002164502 | 2.66 |
| Phenelzine | C8H12N2 | C07430 | 0.39 | -1.34 | 0.002164502 | 2.66 |
| Gamma-terpinene | C10H16 | C09900 | 1.41 | 0.5 | 0.002164502 | 2.66 |
| Eucalyptol | C10H18O | C09844 | 0.37 | -1.43 | 0.002164502 | 2.66 |
| 4-Methoxybenzaldehyde | C8H8O2 | C10761 | 0.8 | -0.33 | 0.002164502 | 2.66 |
| p-Aminobenzoic acid | C7H7NO2 | C00568 | 0.14 | -2.88 | 0.002164502 | 2.66 |
| 3,4-Dihydroxybenzaldehyde | C7H6O3 | C16700 | 1.3 | 0.38 | 0.002164502 | 2.66 |
| Phosphonoacetate | C2H5O5P | C05682 | 1.62 | 0.69 | 0.002164502 | 2.66 |
| N-methyl-L-glutamic Acid | C6H11NO4 | C01046 | 17.91 | 4.16 | 0.002164502 | 2.66 |
| Tryptophanol | C10H11NO | C00955 | 8.22 | 3.04 | 0.002164502 | 2.66 |
| Acetylcholine | C7H16NO2 | C01996 | 0.37 | -1.45 | 0.002164502 | 2.66 |
| 2-Keto-6-aminocaproate | C6H11NO3 | C03239 | 0.47 | -1.07 | 0.002164502 | 2.66 |
| (2R,5S)-2,5-Diaminohexanoate | C6H14N2O2 | C05161 | 0.12 | -3.11 | 0.002164502 | 2.66 |
| Coumarin | C9H6O2 | C05851 | 1.24 | 0.31 | 0.002164502 | 2.66 |
| L-Glutamine | C5H10N2O3 | C00064 | 1.85 | 0.88 | 0.002164502 | 2.66 |
| 3,4-Dihydro-2H-1-benzopyran-2-one | C9H8O2 | C02274 | 1.83 | 0.87 | 0.002164502 | 2.66 |
| Estragole | C10H12O | C10452 | 1.17 | 0.23 | 0.002164502 | 2.66 |
| Phthalic acid | C8H6O4 | C01606 | 2.37 | 1.24 | 0.002164502 | 2.66 |
| L-2-Hydroxyglutaric acid | C5H8O5 | C03196 | 1.25 | 0.33 | 0.002164502 | 2.66 |
| D-Lyxose | C5H10O5 | C00476 | 1.18 | 0.24 | 0.002164502 | 2.66 |
| Homogentisic acid | C8H8O4 | C00544 | 0.35 | -1.52 | 0.002164502 | 2.66 |
| D-Ribose | C5H10O5 | C00121 | 0.65 | -0.62 | 0.002164502 | 2.66 |
| Guanine | C5H5N5O | C00242 | 2.7 | 1.43 | 0.002164502 | 2.66 |
| N-Methyltyramine | C9H13NO | C02442 | 1.8 | 0.85 | 0.002164502 | 2.66 |
| p-Hydroxyphenylacetic acid | C8H8O3 | C00642 | 0.05 | -4.44 | 0.002164502 | 2.66 |
| Ortho-Hydroxyphenylacetic acid | C8H8O3 | C05852 | 0.38 | -1.41 | 0.002164502 | 2.66 |
| (-)-cis-Carveol | C10H16O | C11395 | 1.53 | 0.62 | 0.002164502 | 2.66 |
| 4,5-Dihydroorotic acid | C5H6N2O4 | C00337 | 0.88 | -0.19 | 0.002164502 | 2.66 |
| L-Carnitine | C7H15NO3 | C00318 | 0.29 | -1.8 | 0.002164502 | 2.66 |
| cis-1,2-Dihydronaphthalene-1,2-diol | C10H10O2 | C04314 | 2.98 | 1.57 | 0.004329004 | 2.36 |
| 2-Deoxystreptamine | C6H14N2O3 | C02627 | 1.99 | 1 | 0.002164502 | 2.66 |
| Isoeugenol | C10H12O2 | C10469 | 0.26 | -1.95 | 0.002164502 | 2.66 |
| Eugenol | C10H12O2 | C10453 | 0.71 | -0.5 | 0.002164502 | 2.66 |
| 2-Phenylethyl acetate | C10H12O2 | C12303 | 1.6 | 0.68 | 0.002164502 | 2.66 |
| 7-Methylxanthine | C6H6N4O2 | C16353 | 1.13 | 0.17 | 0.002164502 | 2.66 |
| 3-(2-Hydroxyphenyl)propanoic acid | C9H10O3 | C01198 | 1.07 | 0.1 | 0.015151515 | 1.82 |
| (S)-4-Hydroxymandelate | C8H8O4 | C03198 | 2.05 | 1.04 | 0.002164502 | 2.66 |
| Pyridoxine | C8H11NO3 | C00314 | 0.63 | -0.67 | 0.002164502 | 2.66 |
| 8-Amino-7-oxononanoate | C9H17NO3 | C01092 | 32.94 | 5.04 | 0.002164502 | 2.66 |
| 2-Biphenylol | C12H10O | C02499 | 3.85 | 1.95 | 0.002164502 | 2.66 |
| Levetiracetam | C8H14N2O2 | C07841 | 0.69 | -0.53 | 0.002164502 | 2.66 |
| Dihydroxyacetone phosphate | C3H7O6P | C00111 | 1.3 | 0.38 | 0.002164502 | 2.66 |
| (2S,5S)-trans-Carboxymethylproline | C7H11NO4 | C17366 | 2 | 1 | 0.002164502 | 2.66 |
| N-Acetylleucine | C8H15NO3 | C02710 | 0.74 | -0.44 | 0.008658009 | 2.06 |
| N-Acetyl-L-glutamate 5-semialdehyde | C7H11NO4 | C01250 | 0.82 | -0.29 | 0.002164502 | 2.66 |
| N-Acetyl-L-aspartic acid | C6H9NO5 | C01042 | 0.92 | -0.13 | 0.015151515 | 1.82 |
| Ascorbate | C6H8O6 | C00072 | 5.33 | 2.41 | 0.002164502 | 2.66 |
| D-Galacturonolactone | C6H8O6 | C06430 | 1.11 | 0.16 | 0.002164502 | 2.66 |
| L-Bornesitol | C7H14O6 | C03660 | 4.49 | 2.17 | 0.002164502 | 2.66 |
| Coniferyl aldehyde | C10H10O3 | C02666 | 0.44 | -1.19 | 0.002164502 | 2.66 |
| Methylisoeugenol | C11H14O2 | C10478 | 1.36 | 0.45 | 0.002164502 | 2.66 |
| 5-Deoxy-D-glucuronate | C6H10O6 | C16737 | 1.68 | 0.75 | 0.002164502 | 2.66 |
| 1-Amino-1-deoxy-scyllo-inositol | C6H13NO5 | C01214 | 0.69 | -0.54 | 0.002164502 | 2.66 |
| D-Psicose | C6H12O6 | C06468 | 0.56 | -0.83 | 0.002164502 | 2.66 |
| 1D-chiro-Inositol | C6H12O6 | C19891 | 0.54 | -0.89 | 0.002164502 | 2.66 |
| (S)-beta-Tyrosine | C9H11NO3 | C21308 | 0.02 | -5.91 | 0.002164502 | 2.66 |
| Hydroxyphenyllactic acid | C9H10O4 | C03672 | 0.61 | -0.7 | 0.002164502 | 2.66 |
| 5-Oxo-1,2-campholide | C10H14O3 | C02952 | 2 | 1 | 0.002164502 | 2.66 |
| Choline sulfate | C5H13NO4S | C00919 | 1.71 | 0.77 | 0.002164502 | 2.66 |
| 3-O-Methylgallate | C8H8O5 | C05616 | 0.26 | -1.93 | 0.002164502 | 2.66 |
| Sebacic acid | C10H18O4 | C08277 | 2.5 | 1.32 | 0.002164502 | 2.66 |
| Phosphoserine | C3H8NO6P | C01005 | 1.41 | 0.5 | 0.025974026 | 1.59 |
| 6-Acetamido-3-oxohexanoate | C8H13NO4 | C03682 | 0.77 | -0.38 | 0.004329004 | 2.36 |
| N-Alpha-acetyllysine | C8H16N2O3 | C12989 | 2.18 | 1.13 | 0.002164502 | 2.66 |
| Homocitrulline | C7H15N3O3 | C02427 | 0.32 | -1.64 | 0.002164502 | 2.66 |
| Homo-L-arginine | C7H16N4O2 | C01924 | 1.6 | 0.68 | 0.002164502 | 2.66 |
| Glycylleucine | C8H16N2O3 | C02155 | 1.16 | 0.22 | 0.002164502 | 2.66 |
| N-Acetylglutamic acid | C7H11NO5 | C00624 | 2.26 | 1.18 | 0.002164502 | 2.66 |
| Diaminopimelic acid | C7H14N2O4 | C00666 | 1.95 | 0.96 | 0.002164502 | 2.66 |
| 4-Methoxy-2,2'-bipyrrole-5-carbaldehyde | C10H10N2O2 | C21570 | 0.15 | -2.75 | 0.002164502 | 2.66 |
| 6-Methoxymellein | C11H12O4 | C02381 | 0.75 | -0.42 | 0.002164502 | 2.66 |
| 5-Hydroxyindoleacetic acid | C10H9NO3 | C05635 | 0.14 | -2.87 | 0.002164502 | 2.66 |
| Myristicin | C11H12O3 | C10480 | 1.33 | 0.41 | 0.002164502 | 2.66 |
| Methoxamine | C11H17NO3 | C07513 | 1.16 | 0.21 | 0.002164502 | 2.66 |
| Leucodopachrome | C9H9NO4 | C05604 | 0.12 | -3.04 | 0.002164502 | 2.66 |
| (-)-Bornesitol | C7H14O6 | C03659 | 0.32 | -1.66 | 0.002164502 | 2.66 |
| Metanephrine | C10H15NO3 | C05588 | 0.45 | -1.14 | 0.002164502 | 2.66 |
| Syringic acid | C9H10O5 | C10833 | 0.48 | -1.06 | 0.002164502 | 2.66 |
| Ecgonine methyl ester | C10H17NO3 | C12448 | 0.56 | -0.83 | 0.002164502 | 2.66 |
| Dodecanoic acid | C12H24O2 | C02679 | 0.31 | -1.7 | 0.002164502 | 2.66 |
| Zerumbone | C15H22O | C20262 | 0.44 | -1.2 | 0.008658009 | 2.06 |
| Thiabendazole | C10H7N3S | C07131 | 1.5 | 0.59 | 0.015151515 | 1.82 |
| Caryophyllene alpha-oxide | C15H24O | C16908 | 3.06 | 1.61 | 0.004329004 | 2.36 |
| Apiole | C12H14O4 | C10429 | 1.11 | 0.16 | 0.015151515 | 1.82 |
| Pantothenol | C9H19NO4 | C05944 | 1.48 | 0.57 | 0.002164502 | 2.66 |
| Ibuprofen | C13H18O2 | C01588 | 0.4 | -1.32 | 0.002164502 | 2.66 |
| 3-[(1-Carboxyvinyl)oxy]benzoate | C10H8O5 | C20772 | 1.3 | 0.38 | 0.004329004 | 2.36 |
| L-Kynurenine | C10H12N2O3 | C00328 | 1.33 | 0.41 | 0.025974026 | 1.59 |
| Sinapoyl aldehyde | C11H12O4 | C05610 | 0.54 | -0.89 | 0.002164502 | 2.66 |
| trans-Isoasarone | C12H16O3 | C17846 | 0.35 | -1.51 | 0.002164502 | 2.66 |
| N-Acetyldemethylphosphinothricin | C6H12NO5P | C17949 | 0.06 | -4.15 | 0.002164502 | 2.66 |
| (+)-7-Isojasmonic acid | C12H18O3 | C16317 | 1.13 | 0.17 | 0.004329004 | 2.36 |
| Dethiobiotin | C10H18N2O3 | C01909 | 1.6 | 0.67 | 0.002164502 | 2.66 |
| Cis-zeatin | C10H13N5O | C00371 | 1.13 | 0.18 | 0.002164502 | 2.66 |
| 2-trans,6-trans-Farnesal | C15H24O | C03461 | 0.58 | -0.78 | 0.002164502 | 2.66 |
| Diethyl phthalate | C12H14O4 | C14175 | 1.24 | 0.31 | 0.002164502 | 2.66 |
| Cerulenin | C12H17NO3 | C12058 | 0.61 | -0.71 | 0.002164502 | 2.66 |
| Prephenate | C10H10O6 | C00254 | 0.12 | -3.06 | 0.002164502 | 2.66 |
| Genipin | C11H14O5 | C09780 | 1.18 | 0.23 | 0.015151515 | 1.82 |
| Myristoleic acid | C14H26O2 | C08322 | 0.18 | -2.46 | 0.002164502 | 2.66 |
| Myristic acid | C14H28O2 | C06424 | 6.08 | 2.61 | 0.002164502 | 2.66 |
| 6-Hydroxymelatonin | C13H16N2O3 | C05643 | 1.39 | 0.48 | 0.002164502 | 2.66 |
| Alantolactone | C15H20O2 | C09289 | 0.04 | -4.66 | 0.002164502 | 2.66 |
| N1-Acetylspermine | C12H28N4O | C02567 | 0.86 | -0.22 | 0.004329004 | 2.36 |
| Uridine | C9H12N2O6 | C00299 | 0.75 | -0.42 | 0.015151515 | 1.82 |
| Pindolol | C14H20N2O2 | C07445 | 1.44 | 0.53 | 0.008658009 | 2.06 |
| gamma-L-Glutamyl-L-cysteine | C8H14N2O5S | C00669 | 0.36 | -1.48 | 0.002164502 | 2.66 |
| 5'-Deoxyadenosine | C10H13N5O3 | C05198 | 0.72 | -0.47 | 0.015151515 | 1.82 |
| Benzo[k]fluoranthene | C20H12 | C14321 | 1.57 | 0.65 | 0.002164502 | 2.66 |
| 16-Oxopalmitate | C16H30O3 | C19614 | 0.44 | -1.19 | 0.015151515 | 1.82 |
| Nicotinamide riboside | C11H15N2O5 | C03150 | 0.37 | -1.43 | 0.002164502 | 2.66 |
| Nandrolone | C18H26O2 | C07254 | 11.23 | 3.49 | 0.002164502 | 2.66 |
| Glycerophosphocholine | C8H21NO6P | C00670 | 0.64 | -0.64 | 0.002164502 | 2.66 |
| Parthenin | C15H18O4 | C09523 | 1.51 | 0.59 | 0.002164502 | 2.66 |
| Qing Hau Sau | C15H22O5 | C09538 | 4.26 | 2.09 | 0.002164502 | 2.66 |
| Mirtazapine | C17H19N3 | C07570 | 1.39 | 0.48 | 0.002164502 | 2.66 |
| S-Ribosyl-L-homocysteine | C9H17NO6S | C03539 | 0.88 | -0.19 | 0.004329004 | 2.36 |
| Xanthoxic acid | C15H22O4 | C13454 | 0.26 | -1.92 | 0.002164502 | 2.66 |
| Adenosine | C10H13N5O4 | C00212 | 0.09 | -3.53 | 0.002164502 | 2.66 |
| (S)-Coclaurine | C17H19NO3 | C06161 | 0.88 | -0.19 | 0.002164502 | 2.66 |
| (R)-Coclaurine | C17H19NO3 | C06349 | 24.71 | 4.63 | 0.002164502 | 2.66 |
| Androstenedione | C19H26O2 | C00280 | 0.4 | -1.32 | 0.008658009 | 2.06 |
| Luteolinidin | C15H11O5 | C08652 | 0.1 | -3.31 | 0.002164502 | 2.66 |
| Norizalpinin | C15H10O5 | C10044 | 0.55 | -0.86 | 0.002164502 | 2.66 |
| Genistein | C15H10O5 | C06563 | 0.68 | -0.55 | 0.002164502 | 2.66 |
| All-trans-13,14-dihydroretinol | C20H32O | C15492 | 0.68 | -0.56 | 0.002164502 | 2.66 |
| Etiocholanedione | C19H28O2 | C03772 | 0.62 | -0.69 | 0.002164502 | 2.66 |
| Thienamycin | C11H16N2O4S | C06664 | 1.47 | 0.56 | 0.002164502 | 2.66 |
| Etiocholanolone | C19H30O2 | C04373 | 0.44 | -1.17 | 0.008658009 | 2.06 |
| Naringenin | C15H12O5 | C00509 | 1.69 | 0.76 | 0.002164502 | 2.66 |
| Androsterone | C19H30O2 | C00523 | 0.37 | -1.42 | 0.002164502 | 2.66 |
| Apiforol | C15H14O5 | C12124 | 0.2 | -2.3 | 0.002164502 | 2.66 |
| Afzelechin | C15H14O5 | C09320 | 4.07 | 2.03 | 0.002164502 | 2.66 |
| 5a-Androstane-3b,17b-diol | C19H32O2 | C12525 | 1.79 | 0.84 | 0.002164502 | 2.66 |
| N6-(L-1,3-Dicarboxypropyl)-L-lysine | C11H20N2O6 | C00449 | 1.3 | 0.37 | 0.002164502 | 2.66 |
| Cyclopeptine | C17H16N2O2 | C20579 | 1.41 | 0.49 | 0.002164502 | 2.66 |
| 9-Riburonosyladenine | C10H11N5O5 | C11501 | 0.48 | -1.05 | 0.002164502 | 2.66 |
| 1-Methyladenosine | C11H15N5O4 | C02494 | 0.2 | -2.36 | 0.002164502 | 2.66 |
| Guanosine | C10H13N5O5 | C00387 | 0.56 | -0.85 | 0.002164502 | 2.66 |
| (R,S)-Coclaurine | C17H19NO3 | C06348 | 1.32 | 0.4 | 0.002164502 | 2.66 |
| N1,N12-Diacetylspermine | C14H30N4O2 | C03413 | 0.06 | -3.95 | 0.002164502 | 2.66 |
| Fisetin | C15H10O6 | C10041 | 0.48 | -1.07 | 0.002164502 | 2.66 |
| Eriodictyol | C15H12O6 | C05631 | 50.45 | 5.66 | 0.002164502 | 2.66 |
| Dyclonine | C18H27NO2 | C07881 | 2.9 | 1.54 | 0.002164502 | 2.66 |
| Aurin | C19H14O3 | C14213 | 3.47 | 1.79 | 0.002164502 | 2.66 |
| (+_-)-5-[(tert-Butylamino)-2'-hydroxypropoxy]-3,4-dihydro-1(2H)-naphthalenone | C17H25NO3 | C04883 | 2.7 | 1.43 | 0.002164502 | 2.66 |
| Sclareol | C20H36O2 | C09183 | 0.53 | -0.92 | 0.002164502 | 2.66 |
| 9(S)-HPOT | C18H30O4 | C16321 | 1.91 | 0.94 | 0.002164502 | 2.66 |
| Prunasin | C14H17NO6 | C00844 | 2.77 | 1.47 | 0.002164502 | 2.66 |
| 5'-Methylthioadenosine | C11H15N5O3S | C00170 | 0.13 | -2.94 | 0.002164502 | 2.66 |
| Norethindrone | C20H26O2 | C05028 | 2.63 | 1.39 | 0.002164502 | 2.66 |
| Cafestol | C20H28O3 | C09066 | 0.09 | -3.45 | 0.002164502 | 2.66 |
| all-trans-5,6-Epoxyretinoic acid | C20H28O3 | C16680 | 1.57 | 0.65 | 0.002164502 | 2.66 |
| Metoclopramide | C14H22ClN3O2 | C07868 | 0.34 | -1.54 | 0.041125541 | 1.39 |
| 5-Nitro-2-(3-phenylpropylamino)benzoic acid | C16H16N2O4 | C13705 | 0.58 | -0.79 | 0.002164502 | 2.66 |
| Isotretinoin | C20H28O2 | D00348 | 0.69 | -0.54 | 0.015151515 | 1.82 |
| Questinol | C16H12O6 | C17811 | 1.76 | 0.82 | 0.025974026 | 1.59 |
| Kaempferide | C16H12O6 | C10098 | 0.37 | -1.45 | 0.002164502 | 2.66 |
| 2-Methoxy-17beta-estradiol | C19H26O3 | C05302 | 0.33 | -1.6 | 0.002164502 | 2.66 |
| Sphinganine | C18H39NO2 | C00836 | 17.76 | 4.15 | 0.002164502 | 2.66 |
| Norethandrolone | C20H30O2 | D07127 | 2.01 | 1.01 | 0.002164502 | 2.66 |
| Tricetin | C15H10O7 | C10192 | 2.84 | 1.51 | 0.008658009 | 2.06 |
| Ferreirin | C16H14O6 | C10419 | 1.34 | 0.42 | 0.002164502 | 2.66 |
| Abietate | C20H30O2 | C06087 | 4.22 | 2.08 | 0.002164502 | 2.66 |
| cis-Dihydroquercetin | C15H12O7 | C12316 | 5.28 | 2.4 | 0.002164502 | 2.66 |
| Leucodelphinidin | C15H14O8 | C05909 | 0.59 | -0.76 | 0.002164502 | 2.66 |
| 11alpha,17beta-Dihydroxyandrost-4-en-3-one | C19H28O3 | C15306 | 10.41 | 3.38 | 0.002164502 | 2.66 |
| Glutathione | C10H17N3O6S | C00051 | 2.27 | 1.18 | 0.004329004 | 2.36 |
| Bisdemethoxycurcumin | C19H16O4 | C17743 | 2.84 | 1.51 | 0.002164502 | 2.66 |
| Alprazolam | C17H13ClN4 | C06817 | 0.04 | -4.5 | 0.002164502 | 2.66 |
| beta-D-Galactosyl-(1->4)-L-rhamnose | C12H22O10 | C19758 | 1.55 | 0.63 | 0.002164502 | 2.66 |
| Phenylbutazone | C19H20N2O2 | C07440 | 1.59 | 0.67 | 0.004329004 | 2.36 |
| 2,3-Dinor-8-iso prostaglandin F2alpha | C18H30O5 | C14794 | 1.27 | 0.34 | 0.002164502 | 2.66 |
| N-Acetyl-a-neuraminic acid | C11H19NO9 | C19909 | 0.06 | -4.06 | 0.002164502 | 2.66 |
| Methoprene | C19H34O3 | C14308 | 1.27 | 0.34 | 0.002164502 | 2.66 |
| Aflatoxin B1 | C17H12O6 | C06800 | 0.64 | -0.64 | 0.002164502 | 2.66 |
| Progesterone | C21H30O2 | C00410 | 0.71 | -0.5 | 0.008658009 | 2.06 |
| 5a-Pregnane-3,20-dione | C21H32O2 | C03681 | 0.75 | -0.42 | 0.004329004 | 2.36 |
| N(beta)-Epoxysuccinamoyl-DAP-Val | C12H20N4O6 | C20965 | 10.93 | 3.45 | 0.002164502 | 2.66 |
| Penicillin G | C16H18N2O4S | C05551 | 0.88 | -0.19 | 0.004329004 | 2.36 |
| Chlorpromazine | C17H19ClN2S | C06906 | 1.28 | 0.35 | 0.002164502 | 2.66 |
| Myricetin | C15H10O8 | C10107 | 1.61 | 0.69 | 0.002164502 | 2.66 |
| 11alpha,17beta-Dihydroxy-17-methylandrost-4-en-3-one | C20H30O3 | C14555 | 0.65 | -0.61 | 0.004329004 | 2.36 |
| Deoxy-5-methylcytidylate | C10H16N3O7P | C03495 | 1.56 | 0.64 | 0.002164502 | 2.66 |
| gamma-L-Glutamyl-L-cysteinyl-beta-alanine | C11H19N3O6S | C04544 | 0.41 | -1.29 | 0.002164502 | 2.66 |
| dTMP | C10H15N2O8P | C00364 | 12.08 | 3.59 | 0.002164502 | 2.66 |
| Stylopine | C19H17NO4 | C05175 | 0.62 | -0.68 | 0.008658009 | 2.06 |
| Galactinol | C12H22O11 | C01235 | 0.71 | -0.5 | 0.004329004 | 2.36 |
| Coniferin | C16H22O8 | C00761 | 1.46 | 0.55 | 0.002164502 | 2.66 |
| Citalopram | C20H21FN2O | C07572 | 1.43 | 0.51 | 0.002164502 | 2.66 |
| Cellobiose | C12H22O11 | C00185 | 0.05 | -4.37 | 0.002164502 | 2.66 |
| 2,4-Dioxotetrahydropyrimidine D-ribonucleotide | C9H15N2O9P | C04639 | 1.66 | 0.73 | 0.002164502 | 2.66 |
| Corticosterone | C21H30O4 | C02140 | 0.44 | -1.2 | 0.002164502 | 2.66 |
| Carnosol | C20H26O4 | C09069 | 1.25 | 0.32 | 0.002164502 | 2.66 |
| Cannabielsoin | C21H30O3 | C20218 | 1.49 | 0.58 | 0.002164502 | 2.66 |
| 5'-O-beta-D-Glucosylpyridoxine | C14H21NO8 | C03996 | 0.32 | -1.64 | 0.002164502 | 2.66 |
| Adrenic acid | C22H36O2 | C16527 | 5.06 | 2.34 | 0.002164502 | 2.66 |
| Norsanguinarine | C20H15NO4 | C05191 | 0.13 | -3 | 0.002164502 | 2.66 |
| Gibberellin A4 | C19H24O5 | C11864 | 1.58 | 0.66 | 0.002164502 | 2.66 |
| Prostaglandin D2 | C20H32O5 | C00696 | 2.02 | 1.01 | 0.002164502 | 2.66 |
| Isopentenyl adenosine | C15H21N5O4 | C16427 | 0.25 | -1.99 | 0.002164502 | 2.66 |
| p-Coumaroyl quinic acid | C16H18O8 | C12208 | 10.86 | 3.44 | 0.002164502 | 2.66 |
| (S)-cis-N-Methylstylopine | C20H20NO4 | C06163 | 1.62 | 0.7 | 0.002164502 | 2.66 |
| Isocorypalmine | C20H23NO4 | C04118 | 0.9 | -0.16 | 0.004329004 | 2.36 |
| Turanose | C12H22O11 | C19636 | 0.12 | -3.02 | 0.002164502 | 2.66 |
| Clotrimazole | C22H17ClN2 | C06922 | 0.23 | -2.15 | 0.002164502 | 2.66 |
| Cyclic GMP | C10H12N5O7P | C00942 | 0.86 | -0.22 | 0.008658009 | 2.06 |
| N-Acetylmuramoyl-Ala | C14H24N2O9 | C02999 | 3.17 | 1.66 | 0.002164502 | 2.66 |
| 21-Deoxycortisol | C21H30O4 | C05497 | 0.14 | -2.81 | 0.002164502 | 2.66 |
| 2-Hydroxy-6-pentadecylbenzoic acid | C22H36O3 | C10759 | 0.28 | -1.86 | 0.004329004 | 2.36 |
| 6-Keto-prostaglandin F1a | C20H34O6 | C05961 | 1.19 | 0.26 | 0.002164502 | 2.66 |
| 17-O-Acetylnorajmaline | C21H26N2O3 | C11809 | 3.68 | 1.88 | 0.002164502 | 2.66 |
| Chlorogenic acid | C16H18O9 | C00852 | 15.78 | 3.98 | 0.002164502 | 2.66 |
| Laudanosine | C21H27NO4 | C09558 | 3.89 | 1.96 | 0.002164502 | 2.66 |
| Niaprazine | C20H25FN4O | D07333 | 0.29 | -1.78 | 0.002164502 | 2.66 |
| Aldosterone | C21H28O5 | C01780 | 1.17 | 0.22 | 0.008658009 | 2.06 |
| Lariciresinol | C20H24O6 | C10646 | 0.74 | -0.43 | 0.002164502 | 2.66 |
| Cortisol | C21H30O5 | C00735 | 0.19 | -2.38 | 0.002164502 | 2.66 |
| 11b,21-Dihydroxy-3,20-oxo-5b-pregnan-18-al | C21H30O5 | C05473 | 2.86 | 1.52 | 0.002164502 | 2.66 |
| N-Acetylbialaphos | C13H24N3O7P | C17951 | 0.19 | -2.4 | 0.002164502 | 2.66 |
| Tamoxifen | C26H29NO | C07108 | 1.47 | 0.56 | 0.002164502 | 2.66 |
| Riboflavin | C17H20N4O6 | C00255 | 0.43 | -1.22 | 0.002164502 | 2.66 |
| Celecoxib | C17H14F3N3O2S | C07589 | 2.45 | 1.29 | 0.002164502 | 2.66 |
| Mesoridazine | C21H26N2OS2 | C07143 | 0.28 | -1.84 | 0.002164502 | 2.66 |
| Sufentanil | C22H30N2O2S | C08022 | 1.73 | 0.79 | 0.002164502 | 2.66 |
| Ursodeoxycholic acid | C24H40O4 | C07880 | 0.57 | -0.81 | 0.002164502 | 2.66 |
| Dihydromacarpine | C22H19NO6 | C05316 | 0.6 | -0.73 | 0.015151515 | 1.82 |
| Aloesin | C19H22O9 | C08994 | 0.04 | -4.75 | 0.002164502 | 2.66 |
| Ergocalciferol | C28H44O | C05441 | 3.92 | 1.97 | 0.002164502 | 2.66 |
| beta-Sitosterol | C29H50O | C01753 | 1.6 | 0.68 | 0.002164502 | 2.66 |
| S-Adenosylmethionine | C15H22N6O5S | C00019 | 2.51 | 1.33 | 0.002164502 | 2.66 |
| Colchicine | C22H25NO6 | C07592 | 2.35 | 1.24 | 0.004329004 | 2.36 |
| Besonprodil | C21H23FN2O3S | D03100 | 0.76 | -0.4 | 0.004329004 | 2.36 |
| (-)-alpha-Narcotine | C22H23NO7 | C09592 | 0.78 | -0.35 | 0.008658009 | 2.06 |
| Paspalicine | C27H31NO3 | C20553 | 0.47 | -1.07 | 0.002164502 | 2.66 |
| Kaempferol 3-O-beta-D-xyloside | C20H18O10 | C20727 | 0.29 | -1.76 | 0.002164502 | 2.66 |
| Blasticidin S | C17H26N8O5 | C02010 | 3.45 | 1.79 | 0.008658009 | 2.06 |
| Ginkgolide B | C20H24O10 | C07602 | 0.46 | -1.13 | 0.002164502 | 2.66 |
| Afzelin | C21H20O10 | C16911 | 0.4 | -1.32 | 0.002164502 | 2.66 |
| 8-C-Glucosylnaringenin | C21H22O10 | C16492 | 0.69 | -0.54 | 0.025974026 | 1.59 |
| Naringenin 7-O-beta-D-glucoside | C21H22O10 | C09099 | 0.12 | -3.12 | 0.002164502 | 2.66 |
| 3-Dehydroecdysone | C27H42O6 | C02513 | 0.46 | -1.11 | 0.002164502 | 2.66 |
| Estrone glucuronide | C24H30O8 | C11133 | 1.51 | 0.59 | 0.025974026 | 1.59 |
| Etiocholanolone glucuronide | C25H38O8 | C11136 | 0.54 | -0.9 | 0.002164502 | 2.66 |
| Isoquercitrin | C21H20O12 | C05623 | 1.59 | 0.67 | 0.002164502 | 2.66 |
| Withaferin A | C28H38O6 | C08841 | 2.83 | 1.5 | 0.002164502 | 2.66 |
| Retinoyl b-glucuronide | C26H36O8 | C11061 | 0.38 | -1.38 | 0.002164502 | 2.66 |
| Loperamide | C29H33ClN2O2 | C07080 | 1.85 | 0.89 | 0.002164502 | 2.66 |
| Asiatic acid | C30H48O5 | C08617 | 0.05 | -4.23 | 0.002164502 | 2.66 |
| Antibiotic JI-20B | C20H41N5O9 | C17705 | 0.13 | -2.98 | 0.002164502 | 2.66 |
| Mupirocin | C26H44O9 | C11758 | 1.75 | 0.81 | 0.002164502 | 2.66 |
| 3-alpha(S)-Strictosidine | C27H34N2O9 | C03470 | 0.41 | -1.3 | 0.002164502 | 2.66 |
| Flavonol 3-O-beta-D-glucosyl-(1->2)-beta-D-glucoside | C27H30O13 | C15581 | 0.43 | -1.23 | 0.002164502 | 2.66 |
| N-Acetyl-O-demethylpuromycin-5'-phosphate | C23H30N7O9P | C07030 | 0.35 | -1.52 | 0.002164502 | 2.66 |
| Naringin | C27H32O14 | C09789 | 2.42 | 1.27 | 0.002164502 | 2.66 |
| Astaxanthin | C40H52O4 | C08580 | 0.63 | -0.66 | 0.002164502 | 2.66 |
| Candletoxin A | C35H44O9 | C09068 | 0.59 | -0.75 | 0.002164502 | 2.66 |
| Glutathione amide disulfide | C20H34N8O10S2 | C19690 | 3.21 | 1.68 | 0.008658009 | 2.06 |
| Cyanin | C27H31O16 | C08639 | 1.94 | 0.95 | 0.002164502 | 2.66 |
| Stachyose | C24H42O21 | C01613 | 0.46 | -1.14 | 0.002164502 | 2.66 |
| Cyanidin 3-O-(6-O-p-coumaroyl)glucoside-5-O-glucoside | C36H37O18 | C12096 | 2.06 | 1.05 | 0.002164502 | 2.66 |
| R-Methylmalonyl-CoA | C25H40N7O19P3S | C01213 | 3.44 | 1.78 | 0.002164502 | 2.66 |
| (R)-Malyl-CoA | C25H40N7O20P3S | C20747 | 2.17 | 1.12 | 0.002164502 | 2.66 |
| Glyceric acid | C3H6O4 | C00258 | 0.26 | -1.92 | 0.002164502 | 2.66 |
| Phenylacetaldehyde | C8H8O | C00601 | 1.77 | 0.83 | 0.002164502 | 2.66 |
| 3-Methylthiopropionic acid | C4H8O2S | C08276 | 1.56 | 0.64 | 0.002164502 | 2.66 |
| Adipic acid | C6H10O4 | C06104 | 0.82 | -0.29 | 0.002164502 | 2.66 |
| Pyrrolidonecarboxylic acid | C5H7NO3 | C02237 | 0.6 | -0.73 | 0.002164502 | 2.66 |
| Glutaric acid | C5H8O4 | C00489 | 3.39 | 1.76 | 0.002164502 | 2.66 |
| L-Aspartic acid | C4H7NO4 | C00049 | 0.26 | -1.97 | 0.002164502 | 2.66 |
| Mandelonitrile | C8H7NO | C00561 | 1.67 | 0.74 | 0.002164502 | 2.66 |
| L-Malic acid | C4H6O5 | C00149 | 2.11 | 1.08 | 0.002164502 | 2.66 |
| Adenine | C5H5N5 | C00147 | 1.68 | 0.75 | 0.002164502 | 2.66 |
| Salicylic acid | C7H6O3 | C00805 | 0.72 | -0.48 | 0.015151515 | 1.82 |
| Citramalic acid | C5H8O5 | C00815 | 1.87 | 0.9 | 0.002164502 | 2.66 |
| D-Xylose | C5H10O5 | C00181 | 0.58 | -0.78 | 0.002164502 | 2.66 |
| 2',4'-Dihydroxyacetophenone | C8H8O3 | C03663 | 0.47 | -1.08 | 0.002164502 | 2.66 |
| 2-Isopropylmalic acid | C7H12O5 | C02504 | 0.24 | -2.05 | 0.004329004 | 2.36 |
| Oxoadipic acid | C6H8O5 | C00322 | 2.49 | 1.32 | 0.008658009 | 2.06 |
| (Z)-4-Hydroxy-6-dodecenoic acid lactone | C6H10O6 | C03107 | 9.31 | 3.22 | 0.002164502 | 2.66 |
| myo-Inositol | C6H12O6 | C00137 | 2.6 | 1.38 | 0.025974026 | 1.59 |
| Fructose-1P | C6H12O6 | C10906 | 1.46 | 0.54 | 0.002164502 | 2.66 |
| trans-2-Hydroxycinnamate | C9H8O3 | C01772 | 1.29 | 0.36 | 0.002164502 | 2.66 |
| Vanylglycol | C9H12O4 | C05594 | 1.34 | 0.42 | 0.015151515 | 1.82 |
| Phenyllactate | C9H10O3 | C05607 | 3.71 | 1.89 | 0.002164502 | 2.66 |
| Tropate | C9H10O3 | C01456 | 3.67 | 1.87 | 0.002164502 | 2.66 |
| Norepinephrine | C8H11NO3 | C00547 | 1.94 | 0.95 | 0.002164502 | 2.66 |
| Gallic acid | C7H6O5 | C01424 | 0.33 | -1.61 | 0.015151515 | 1.82 |
| Beta-Glycerophosphoric acid | C3H9O6P | C02979 | 2.85 | 1.51 | 0.002164502 | 2.66 |
| Dehydroascorbate | C6H6O6 | C05422 | 4.85 | 2.28 | 0.002164502 | 2.66 |
| Isocitric acid | C6H8O7 | C00311 | 0.52 | -0.93 | 0.008658009 | 2.06 |
| Guanidinosuccinic acid | C5H9N3O4 | C03139 | 0.54 | -0.89 | 0.008658009 | 2.06 |
| Gluconolactone | C6H10O6 | C00198 | 2.35 | 1.23 | 0.002164502 | 2.66 |
| Aesculetin | C9H6O4 | C09263 | 0.57 | -0.82 | 0.025974026 | 1.59 |
| Gluconic acid | C6H12O7 | C00257 | 0.57 | -0.8 | 0.002164502 | 2.66 |
| D-(+)-Glucose | C6H12O6 | C00293 | 1.5 | 0.58 | 0.025974026 | 1.59 |
| Alpha-D-Glucose | C6H12O6 | C00267 | 1.3 | 0.38 | 0.002164502 | 2.66 |
| D-Fructose | C6H12O6 | C00095 | 0.7 | -0.51 | 0.002164502 | 2.66 |
| Azelaic acid | C9H16O4 | C08261 | 1.13 | 0.17 | 0.008658009 | 2.06 |
| Xanthoxylin | C10H12O4 | C10726 | 0.49 | -1.03 | 0.002164502 | 2.66 |
| Vanillylmandelic acid | C9H10O5 | C05584 | 1.77 | 0.83 | 0.002164502 | 2.66 |
| L-Tryptophan | C11H12N2O2 | C00078 | 33.6 | 5.07 | 0.002164502 | 2.66 |
| N-Acetyl-L-phenylalanine | C11H13NO3 | C03519 | 0.07 | -3.93 | 0.002164502 | 2.66 |
| (-)-Jasmonic acid | C12H18O3 | C08491 | 0.55 | -0.87 | 0.002164502 | 2.66 |
| Galactaric acid | C6H10O8 | C00879 | 1.59 | 0.67 | 0.002164502 | 2.66 |
| N-Acetyl-D-glucosamine | C8H15NO6 | C00140 | 0.19 | -2.43 | 0.002164502 | 2.66 |
| 6-Acetyl-D-glucose | C8H14O7 | C02655 | 1.79 | 0.84 | 0.002164502 | 2.66 |
| Methyl jasmonate | C13H20O3 | C11512 | 1.11 | 0.15 | 0.002164502 | 2.66 |
| Thymidine | C10H14N2O5 | C00214 | 3.08 | 1.62 | 0.008658009 | 2.06 |
| Citrinin | C13H14O5 | C16765 | 0.04 | -4.77 | 0.002164502 | 2.66 |
| Galactosylglycerol | C9H18O8 | C05401 | 0.54 | -0.88 | 0.002164502 | 2.66 |
| Shikimate 3-phosphate | C7H11O8P | C03175 | 2.66 | 1.41 | 0.002164502 | 2.66 |
| Galactose 1-phosphate | C6H13O9P | C00103 | 1.86 | 0.9 | 0.002164502 | 2.66 |
| (S)-Abscisic acid | C15H20O4 | C06082 | 3.86 | 1.95 | 0.002164502 | 2.66 |
| Apigenin | C15H10O5 | C01477 | 1.57 | 0.65 | 0.002164502 | 2.66 |
| Phloretin | C15H14O5 | C00774 | 13.27 | 3.73 | 0.002164502 | 2.66 |
| Pantetheine | C11H22N2O4S | C00831 | 0.93 | -0.1 | 0.008658009 | 2.06 |
| Hexadecanedioate | C16H30O4 | C19615 | 1.89 | 0.92 | 0.002164502 | 2.66 |
| Kaempferol | C15H10O6 | C05903 | 2.76 | 1.46 | 0.002164502 | 2.66 |
| 13(S)-HPOT | C18H30O4 | C04785 | 0.12 | -3.07 | 0.002164502 | 2.66 |
| 13S-hydroxyoctadecadienoic acid | C18H32O3 | C14762 | 1.16 | 0.21 | 0.015151515 | 1.82 |
| 12,13-DHOME | C18H34O4 | C14829 | 1.78 | 0.83 | 0.002164502 | 2.66 |
| 4-(beta-D-Glucosyloxy)benzoate | C13H16O8 | C03993 | 2 | 1 | 0.002164502 | 2.66 |
| Diosmetin | C16H12O6 | C10038 | 1.41 | 0.49 | 0.002164502 | 2.66 |
| 2-Methoxyestrone | C19H24O3 | C05299 | 9.8 | 3.29 | 0.002164502 | 2.66 |
| 4-Coumaroylshikimate | C16H16O7 | C02947 | 1.85 | 0.88 | 0.002164502 | 2.66 |
| EPA (d5) | C20H30O2 | C06428 | 3.32 | 1.73 | 0.002164502 | 2.66 |
| Pentahydroxyflavanone | C15H12O7 | C05911 | 1.3 | 0.38 | 0.002164502 | 2.66 |
| (-)-Epigallocatechin | C15H14O7 | C12136 | 1.84 | 0.88 | 0.002164502 | 2.66 |
| 9(S)-HPODE | C18H32O4 | C14827 | 1.15 | 0.2 | 0.002164502 | 2.66 |
| Isorhamnetin | C16H12O7 | C10084 | 4.63 | 2.21 | 0.041125541 | 1.39 |
| 15-Deoxy-d-12,14-PGJ2 | C20H28O3 | C14717 | 0.41 | -1.29 | 0.002164502 | 2.66 |
| 12-KETE | C20H30O3 | C14807 | 1.37 | 0.46 | 0.002164502 | 2.66 |
| Melibiitol | C12H24O11 | C05399 | 2.09 | 1.06 | 0.002164502 | 2.66 |
| 1-O-Vanilloyl-beta-D-glucose | C14H18O9 | C20470 | 0.4 | -1.32 | 0.002164502 | 2.66 |
| Prostaglandin A2 | C20H30O4 | C05953 | 2.84 | 1.51 | 0.002164502 | 2.66 |
| Delta-12-Prostaglandin J2 | C20H30O4 | C05958 | 2.97 | 1.57 | 0.002164502 | 2.66 |
| Dicumarol | C19H12O6 | C00796 | 2.54 | 1.35 | 0.002164502 | 2.66 |
| (5Z,9E,14Z)-(8xi,11R,12S)-11,12-epoxy-8-hydroxyicosa-5,9,14-trienoic Acid | C20H32O4 | C04849 | 2.62 | 1.39 | 0.002164502 | 2.66 |
| Dattelic acid | C16H16O8 | C10434 | 0.41 | -1.28 | 0.002164502 | 2.66 |
| 11,12-DiHETrE | C20H34O4 | C14774 | 1.34 | 0.42 | 0.002164502 | 2.66 |
| Erucic acid | C22H42O2 | C08316 | 0.13 | -2.91 | 0.002164502 | 2.66 |
| 3-Ketosucrose | C12H20O11 | C05731 | 3.12 | 1.64 | 0.002164502 | 2.66 |
| 3'-Ketolactose | C12H20O11 | C05403 | 1.8 | 0.85 | 0.025974026 | 1.59 |
| Fructose 1,6-bisphosphate | C6H14O12P2 | C00354 | 0.27 | -1.9 | 0.002164502 | 2.66 |
| Trehalose | C12H22O11 | C01083 | 2.47 | 1.3 | 0.002164502 | 2.66 |
| Melibiose | C12H22O11 | C05400 | 1.92 | 0.94 | 0.002164502 | 2.66 |
| 11-Dehydro-thromboxane B2 | C20H32O6 | C05964 | 3.4 | 1.76 | 0.002164502 | 2.66 |
| 19-Hydroxytabersonine | C21H24N2O3 | C11642 | 0.2 | -2.3 | 0.002164502 | 2.66 |
| (13E)-11a-Hydroxy-9,15-dioxoprost-13-enoic acid | C20H32O5 | C04654 | 0.42 | -1.26 | 0.002164502 | 2.66 |
| 13,14-Dihydro-15-keto-PGE2 | C20H32O5 | C04671 | 1.23 | 0.3 | 0.002164502 | 2.66 |
| Neochlorogenic acid | C16H18O9 | C17147 | 0.39 | -1.37 | 0.002164502 | 2.66 |
| Pioglitazone | C19H20N2O3S | C07675 | 0.11 | -3.22 | 0.002164502 | 2.66 |
| Rosmarinic acid | C18H16O8 | C01850 | 0.11 | -3.21 | 0.002164502 | 2.66 |
| Nervonic acid | C24H46O2 | C08323 | 1.6 | 0.68 | 0.025974026 | 1.59 |
| Dehydroepiandrosterone sulfate | C19H28O5S | C04555 | 1.41 | 0.5 | 0.002164502 | 2.66 |
| Curcumin | C21H20O6 | C10443 | 1.26 | 0.34 | 0.002164502 | 2.66 |
| (-)-Wikstromol | C20H22O7 | C10725 | 1.49 | 0.58 | 0.002164502 | 2.66 |
| Gardenoside | C17H24O11 | C09779 | 1.92 | 0.94 | 0.002164502 | 2.66 |
| Carvedilol | C24H26N2O4 | C06875 | 1.55 | 0.64 | 0.002164502 | 2.66 |
| Podofilox | C22H22O8 | C10874 | 1.77 | 0.83 | 0.002164502 | 2.66 |
| Quinacrine | C23H30ClN3O | C07339 | 0.64 | -0.64 | 0.002164502 | 2.66 |
| Neolinustatin | C17H29NO11 | C08336 | 0.09 | -3.41 | 0.004329004 | 2.36 |
| Linustatin | C16H27NO11 | C08333 | 0.28 | -1.86 | 0.002164502 | 2.66 |
| Puerarin | C21H20O9 | C10524 | 0.46 | -1.13 | 0.015151515 | 1.82 |
| Lamioside | C18H28O11 | C11645 | 1.67 | 0.74 | 0.002164502 | 2.66 |
| Cosmosiin | C21H20O10 | C04608 | 0.09 | -3.55 | 0.002164502 | 2.66 |
| 2',4,4',6'-Tetrahydroxychalcone 4'-O-glucoside | C21H22O10 | C16407 | 2.94 | 1.55 | 0.004329004 | 2.36 |
| Epigallocatechin gallate | C22H18O11 | C09731 | 2.91 | 1.54 | 0.025974026 | 1.59 |
| Folic acid | C19H19N7O6 | C00504 | 3.57 | 1.84 | 0.002164502 | 2.66 |
| Cyanidin 3-glucoside | C21H21O11 | C08604 | 3.87 | 1.95 | 0.008658009 | 2.06 |
| 6-Methoxyluteolin 7-rhamnoside | C22H22O11 | C10104 | 1.52 | 0.6 | 0.041125541 | 1.39 |
| Myricitrin | C21H20O12 | C10108 | 0.05 | -4.23 | 0.002164502 | 2.66 |
| Iridodial glucoside tetraacetate | C24H34O11 | C11657 | 5.1 | 2.35 | 0.002164502 | 2.66 |
| Isochlorogenic acid b | C25H24O12 | C10468 | 1.83 | 0.87 | 0.002164502 | 2.66 |
| 10-Deoxygeniposide tetraacetate | C25H32O13 | C11664 | 0.17 | -2.52 | 0.002164502 | 2.66 |
| 7-Dehydrologanin tetraacetate | C25H32O14 | C11668 | 0.37 | -1.45 | 0.004329004 | 2.36 |
| Asperuloside tetraacetate | C26H30O15 | C11655 | 4.57 | 2.19 | 0.002164502 | 2.66 |
| Delphinidin 3-(6-p-coumaroyl)glucoside | C30H27O14 | C16370 | 1.72 | 0.78 | 0.002164502 | 2.66 |
| Kaempferol 3-O-rhamnoside-7-O-glucoside | C27H30O15 | C21854 | 13.29 | 3.73 | 0.002164502 | 2.66 |
| Neomycin | C23H46N6O13 | C01737 | 0.1 | -3.37 | 0.002164502 | 2.66 |
| Kaempferol 3-O-beta-D-glucosylgalactoside | C27H30O16 | C16490 | 1.67 | 0.74 | 0.015151515 | 1.82 |
| Quercetin 3-O-beta-D-glucosyl-(1->2)-beta-D-glucoside | C27H30O17 | C12667 | 3.59 | 1.84 | 0.004329004 | 2.36 |
| Delphin | C27H31O17 | C16312 | 1.54 | 0.63 | 0.002164502 | 2.66 |
| Lacto-N-tetraose | C26H45NO21 | C06371 | 1.9 | 0.93 | 0.002164502 | 2.66 |
| Geranyl acetate | C12H20O2 | C09861 | 9 | 3.17 | 0.002164502 | 2.66 |
| (-)-alpha-Curcumene | C15H22 | C09649 | 6.72 | 2.75 | 0.002164502 | 2.66 |
| Copal-8-ol diphosphate | C20H38O8P2 | C20270 | 5.89 | 2.56 | 0.002164502 | 2.66 |
| Trehalose 6-phosphate | C12H23O14P | C00689 | 4.88 | 2.29 | 0.002164502 | 2.66 |
| PC(18_3(6Z,9Z,12Z)_18_3(6Z,9Z,12Z)) | C44H76NO8P | C00157 | 4.29 | 2.1 | 0.002164502 | 2.66 |
| Propazine | C9H16ClN5 | C14312 | 3.76 | 1.91 | 0.004329004 | 2.36 |
| Rhodoxanthin | C40H50O2 | C08610 | 3.64 | 1.87 | 0.008658009 | 2.06 |
| Raffinose | C18H32O16 | C00492 | 3.48 | 1.8 | 0.002164502 | 2.66 |
| L-Arginine | C6H14N4O2 | C00062 | 3.36 | 1.75 | 0.002164502 | 2.66 |
| Lamiide | C17H26O12 | C11644 | 3.22 | 1.69 | 0.002164502 | 2.66 |
| Menadione | C11H8O2 | C05377 | 3.19 | 1.68 | 0.002164502 | 2.66 |
| Estradiol-17beta 3-sulfate | C18H24O5S | C08357 | 3.2 | 1.68 | 0.002164502 | 2.66 |
| Delphinidin 3-glucoside | C21H21O12 | C12138 | 3.13 | 1.65 | 0.002164502 | 2.66 |
| Dihydrotestosterone | C19H30O2 | C03917 | 3.1 | 1.63 | 0.002164502 | 2.66 |
| alpha-Mangostin | C24H26O6 | C10080 | 3.1 | 1.63 | 0.008658009 | 2.06 |
| Eriocitrin | C27H32O15 | C09732 | 3.09 | 1.63 | 0.002164502 | 2.66 |
| 2-Heptanone | C7H14O | C08380 | 3.02 | 1.59 | 0.002164502 | 2.66 |
| Mitomycin | C15H18N4O5 | C06681 | 3 | 1.58 | 0.002164502 | 2.66 |
| Xanthurenic acid | C10H7NO4 | C02470 | 3 | 1.58 | 0.002164502 | 2.66 |
| Neocnidilide | C12H18O2 | C17002 | 2.97 | 1.57 | 0.002164502 | 2.66 |
| Stearidonic acid | C18H28O2 | C16300 | 2.84 | 1.51 | 0.002164502 | 2.66 |
| N,N-Diethyl-m-toluamide | C12H17NO | C10935 | 2.73 | 1.45 | 0.002164502 | 2.66 |
| Creatine | C4H9N3O2 | C00300 | 2.62 | 1.39 | 0.002164502 | 2.66 |
| 1-O-Galloyl-beta-D-glucose | C13H16O10 | C01158 | 2.61 | 1.39 | 0.002164502 | 2.66 |
| 7a-Hydroxyandrost-4-ene-3,17-dione | C19H26O3 | C05296 | 2.46 | 1.3 | 0.041125541 | 1.39 |
| Irisxanthone | C20H20O11 | C10067 | 2.44 | 1.29 | 0.002164502 | 2.66 |
| trans-Piceid | C20H22O8 | C10275 | 2.37 | 1.24 | 0.002164502 | 2.66 |
| Norfloxacin | C16H18FN3O3 | C06687 | 2.32 | 1.22 | 0.002164502 | 2.66 |
| Pentamidine | C19H24N4O2 | C07420 | 2.32 | 1.21 | 0.002164502 | 2.66 |
| 2'-Dehydrokanamycin A | C18H34N4O11 | C20509 | 2.31 | 1.21 | 0.002164502 | 2.66 |
| Ribitol | C5H12O5 | C00474 | 2.27 | 1.19 | 0.002164502 | 2.66 |
| Alprenolol | C15H23NO2 | D07156 | 2.23 | 1.15 | 0.002164502 | 2.66 |
| trans-Ferulic acid | C10H10O4 | C01494 | 2.22 | 1.15 | 0.002164502 | 2.66 |
| 2'-Deamino-2'-hydroxy-6'-dehydroparomamine | C12H22N2O8 | C20351 | 2.18 | 1.12 | 0.025974026 | 1.59 |
| Methaqualone | C16H14N2O | C07560 | 2.12 | 1.09 | 0.002164502 | 2.66 |
| Pyridaben | C19H25ClN2OS | C18614 | 2.12 | 1.09 | 0.008658009 | 2.06 |
| 8-Epiiridodial glucoside tetraacetate | C24H34O11 | C11658 | 2.13 | 1.09 | 0.002164502 | 2.66 |
| Coniferyl alcohol | C10H12O3 | C00590 | 2.1 | 1.07 | 0.002164502 | 2.66 |
| Quinate | C7H12O6 | C00296 | 2.1 | 1.07 | 0.015151515 | 1.82 |
| Thymine | C5H6N2O2 | C00178 | 2.09 | 1.06 | 0.002164502 | 2.66 |
| (+)-(S)-Carvone | C10H14O | C11383 | 2.09 | 1.06 | 0.015151515 | 1.82 |
| Desaminotyrosine | C9H10O3 | C01744 | 2.05 | 1.04 | 0.008658009 | 2.06 |
| Myclobutanil | C15H17ClN4 | C18477 | 2.05 | 1.04 | 0.002164502 | 2.66 |
| Cyanidin 3-O-(6-O-p-coumaroyl)glucoside | C30H27O13 | C12095 | 2.01 | 1.01 | 0.004329004 | 2.36 |
| Eriodictyol chalcone | C15H12O6 | C15525 | 2 | 1 | 0.002164502 | 2.66 |
| Delphinidin 3-rutinoside | C27H31O16 | C16315 | 1.98 | 0.99 | 0.002164502 | 2.66 |
| 2,3-Butanediol | C4H10O2S2 | C00265 | 1.98 | 0.98 | 0.002164502 | 2.66 |
| 5-Aminopentanoic acid | C5H11NO2 | C00431 | 1.97 | 0.98 | 0.002164502 | 2.66 |
| m-Coumaric acid | C9H8O3 | C12621 | 1.98 | 0.98 | 0.002164502 | 2.66 |
| D-Glucuronic Acid | C6H10O7 | C00191 | 1.98 | 0.98 | 0.002164502 | 2.66 |
| 4-Quinolinecarboxylic acid | C10H7NO2 | C06414 | 1.96 | 0.97 | 0.002164502 | 2.66 |
| Sinapate | C11H12O5 | C00482 | 1.95 | 0.97 | 0.025974026 | 1.59 |
| U50488 | C19H26Cl2N2O | C11796 | 1.96 | 0.97 | 0.002164502 | 2.66 |
| Tyramine | C8H11NO | C00483 | 1.9 | 0.93 | 0.002164502 | 2.66 |
| Malvidin | C17H15O7 | C08716 | 1.88 | 0.91 | 0.002164502 | 2.66 |
| Vitexin | C21H20O10 | C01460 | 1.88 | 0.91 | 0.002164502 | 2.66 |
| 8,11,14-Eicosatrienoic acid | C20H34O2 | C03242 | 1.85 | 0.89 | 0.002164502 | 2.66 |
| Resveratrol | C14H12O3 | C03582 | 1.77 | 0.82 | 0.002164502 | 2.66 |
| (10S)-Juvenile hormone III diol phosphate | C16H29O7P | C16507 | 1.72 | 0.78 | 0.002164502 | 2.66 |
| Aspartame | C14H18N2O5 | C11045 | 1.69 | 0.76 | 0.041125541 | 1.39 |
| 17alpha,21-Dihydroxypregnenolone | C21H32O4 | C05487 | 1.66 | 0.74 | 0.002164502 | 2.66 |
| Biocytin | C16H28N4O4S | C05552 | 1.58 | 0.66 | 0.025974026 | 1.59 |
| 4-Guanidinobutanoic acid | C5H11N3O2 | C01035 | 1.57 | 0.65 | 0.025974026 | 1.59 |
| Hydroxyzine | C21H27ClN2O2 | C07045 | 1.55 | 0.63 | 0.002164502 | 2.66 |
| Prostaglandin B2 | C20H30O4 | C05954 | 1.55 | 0.63 | 0.025974026 | 1.59 |
| Aflatoxin G2 | C17H14O7 | C16754 | 1.52 | 0.6 | 0.002164502 | 2.66 |
| 1,5-Naphthalenediamine | C10H10N2 | C19463 | 1.5 | 0.59 | 0.004329004 | 2.36 |
| (1S,4R)-1-Hydroxy-2-oxolimonene | C10H16O2 | C11937 | 1.5 | 0.58 | 0.002164502 | 2.66 |
| (+)-6a-Hydroxymaackiain | C16H12O6 | C16230 | 1.5 | 0.58 | 0.002164502 | 2.66 |
| Nobiletin | C21H22O8 | C10112 | 1.5 | 0.58 | 0.004329004 | 2.36 |
| Pergolide | C19H26N2S | C07425 | 1.47 | 0.55 | 0.002164502 | 2.66 |
| Prostaglandin E1 | C20H34O5 | C04741 | 1.43 | 0.51 | 0.008658009 | 2.06 |
| Psoralidin | C20H16O5 | C10523 | 1.36 | 0.44 | 0.015151515 | 1.82 |
| Bovinic acid | C18H32O2 | C04056 | 1.36 | 0.44 | 0.008658009 | 2.06 |
| Phlorizin | C21H24O10 | C01604 | 1.36 | 0.44 | 0.015151515 | 1.82 |
| L-Glutamic acid | C5H9NO4 | C00025 | 1.33 | 0.41 | 0.002164502 | 2.66 |
| Pelargonic acid | C9H18O2 | C01601 | 1.32 | 0.4 | 0.002164502 | 2.66 |
| 9S-hydroxy-11,15-dioxo-5Z,13E-prostadienoic acid | C20H30O5 | C04758 | 1.32 | 0.4 | 0.002164502 | 2.66 |
| all-trans-Retinoic acid | C20H28O2 | C00777 | 1.31 | 0.39 | 0.002164502 | 2.66 |
| 3-Methylindolepyruvate | C12H11NO3 | C05644 | 1.3 | 0.38 | 0.002164502 | 2.66 |
| 2-Methylserine | C4H9NO3 | C02115 | 1.28 | 0.36 | 0.002164502 | 2.66 |
| Glutethimide | C13H15NO2 | C07489 | 1.28 | 0.36 | 0.008658009 | 2.06 |
| Warfarin | C19H16O4 | C01541 | 1.28 | 0.36 | 0.002164502 | 2.66 |
| Sinapyl alcohol | C11H14O4 | C02325 | 1.27 | 0.34 | 0.008658009 | 2.06 |
| trans-trans-Muconic acid | C6H6O4 | C02480 | 1.25 | 0.32 | 0.002164502 | 2.66 |
| 3-Dehydrosphinganine | C18H37NO2 | C02934 | 1.23 | 0.3 | 0.002164502 | 2.66 |
| gamma-Aminobutyric acid | C4H9NO2 | C00334 | 1.21 | 0.27 | 0.002164502 | 2.66 |
| Tetrahydrocortisol | C21H34O5 | C05472 | 1.2 | 0.27 | 0.002164502 | 2.66 |
| p-Octopamine | C8H11NO2 | C04227 | 1.18 | 0.24 | 0.025974026 | 1.59 |
| trans-beta-D-Glucosyl-2-hydroxycinnamate | C15H18O8 | C05158 | 1.18 | 0.24 | 0.025974026 | 1.59 |
| N-Demethylindolmycin | C13H13N3O2 | C21443 | 1.17 | 0.22 | 0.002164502 | 2.66 |
| Epicatechin | C15H14O6 | C09727 | 1.13 | 0.18 | 0.008658009 | 2.06 |
| Imidazol-5-yl-pyruvate | C6H6N2O3 | C03277 | 0.94 | -0.09 | 0.015151515 | 1.82 |
| L-Fucose | C6H12O5 | C00507 | 0.93 | -0.11 | 0.002164502 | 2.66 |
| 5-Hydroxymethyluracil | C5H6N2O3 | C03088 | 0.86 | -0.22 | 0.015151515 | 1.82 |
| Methyl 2-hydroxybenzoate | C8H8O3 | C12305 | 0.82 | -0.29 | 0.002164502 | 2.66 |
| Aflatoxin M1 | C17H12O7 | C16756 | 0.8 | -0.33 | 0.002164502 | 2.66 |
| Limonoate | C26H34O10 | C01593 | 0.76 | -0.39 | 0.004329004 | 2.36 |
| Linoleic acid | C18H32O2 | C01595 | 0.76 | -0.4 | 0.015151515 | 1.82 |
| Undecanoic acid | C11H22O2 | C17715 | 0.75 | -0.42 | 0.002164502 | 2.66 |
| 11,12,15-THETA | C20H34O5 | C14782 | 0.75 | -0.42 | 0.002164502 | 2.66 |
| Quinolinic acid | C7H5NO4 | C03722 | 0.74 | -0.44 | 0.004329004 | 2.36 |
| Nicotine | C10H14N2 | C00745 | 0.73 | -0.45 | 0.002164502 | 2.66 |
| Lusitanicoside | C21H30O10 | C10474 | 0.72 | -0.48 | 0.002164502 | 2.66 |
| Prostaglandin E2 | C20H32O5 | C00584 | 0.71 | -0.5 | 0.002164502 | 2.66 |
| 2-Hydroxyisophthalic acid | C8H6O5 | C14097 | 0.7 | -0.51 | 0.002164502 | 2.66 |
| 4-Hydroxy-3-(3-methyl-2-butenyl)acetophenone | C13H16O2 | C10702 | 0.69 | -0.53 | 0.002164502 | 2.66 |
| Methotrimeprazine | C19H24N2OS | C07192 | 0.65 | -0.62 | 0.002164502 | 2.66 |
| Cortisone | C21H28O5 | C00762 | 0.64 | -0.64 | 0.002164502 | 2.66 |
| (S)-Tetrahydropapaverine | C20H25NO4 | C21631 | 0.62 | -0.7 | 0.002164502 | 2.66 |
| dGMP | C10H14N5O7P | C00362 | 0.61 | -0.72 | 0.002164502 | 2.66 |
| 5(S)-HpETE | C20H32O4 | C05356 | 0.6 | -0.74 | 0.002164502 | 2.66 |
| Nivalenol | C15H20O7 | C06080 | 0.59 | -0.75 | 0.025974026 | 1.59 |
| Pyrophosphate | H4P2O7 | C00013 | 0.59 | -0.76 | 0.002164502 | 2.66 |
| Acetyl-maltose | C14H24O12 | C02130 | 0.59 | -0.77 | 0.002164502 | 2.66 |
| trans-Cinnamate | C9H8O2 | C00423 | 0.58 | -0.79 | 0.015151515 | 1.82 |
| (+)-Pinoresinol | C20H22O6 | C05366 | 0.57 | -0.82 | 0.002164502 | 2.66 |
| Secologanin | C17H24O10 | C01852 | 0.57 | -0.82 | 0.002164502 | 2.66 |
| D-Galactose | C6H12O6 | C00124 | 0.55 | -0.87 | 0.025974026 | 1.59 |
| Confertifolin | C15H22O2 | C09376 | 0.54 | -0.88 | 0.002164502 | 2.66 |
| Melezitose | C18H32O16 | C08243 | 0.52 | -0.94 | 0.002164502 | 2.66 |
| (S)-Pinocembrin | C15H12O4 | C09827 | 0.51 | -0.97 | 0.002164502 | 2.66 |
| N-Acetylserotonin | C12H14N2O2 | C00978 | 0.49 | -1.04 | 0.002164502 | 2.66 |
| Arachidic acid | C20H40O2 | C06425 | 0.45 | -1.14 | 0.002164502 | 2.66 |
| 20-HETE | C20H32O3 | C14748 | 0.45 | -1.15 | 0.002164502 | 2.66 |
| Eupatilin | C18H16O7 | C10040 | 0.44 | -1.17 | 0.002164502 | 2.66 |
| Homocitric acid | C7H10O7 | C01251 | 0.42 | -1.26 | 0.002164502 | 2.66 |
| 6-Ketoprostaglandin E1 | C20H32O6 | C05962 | 0.41 | -1.27 | 0.002164502 | 2.66 |
| D-Xylonate | C5H10O6 | C00502 | 0.41 | -1.3 | 0.002164502 | 2.66 |
| Estrone | C18H22O2 | C00468 | 0.38 | -1.39 | 0.002164502 | 2.66 |
| Glutarate semialdehyde | C5H8O3 | C03273 | 0.31 | -1.7 | 0.002164502 | 2.66 |
| Benz[a]anthracene | C18H12 | C14317 | 0.29 | -1.79 | 0.015151515 | 1.82 |
| Testosterone glucuronide | C25H36O8 | C11134 | 0.28 | -1.83 | 0.002164502 | 2.66 |
| Bursehernin | C21H22O6 | C21183 | 0.27 | -1.88 | 0.002164502 | 2.66 |
| Kynurenic acid | C10H7NO3 | C01717 | 0.25 | -2.03 | 0.002164502 | 2.66 |
| beta-Alanyl-L-lysine | C9H19N3O3 | C05341 | 0.22 | -2.16 | 0.002164502 | 2.66 |
| 12-Keto-tetrahydro-leukotriene B4 | C20H32O4 | C02165 | 0.16 | -2.61 | 0.002164502 | 2.66 |
| Spermine | C10H26N4 | C00750 | 0.11 | -3.24 | 0.002164502 | 2.66 |
| S-Adenosylmethioninamine | C14H23N6O3S | C01137 | 0.1 | -3.26 | 0.002164502 | 2.66 |
| Dihydromyricetin | C15H12O8 | C02906 | 0.08 | -3.59 | 0.002164502 | 2.66 |
| Carbenicillin | C17H18N2O6S | C06869 | 0.08 | -3.65 | 0.002164502 | 2.66 |
| 5-Valerolactone | C5H8O2 | C02240 | 2.91 | 1.54 | 0.008658009 | 2.06 |
| Succinic acid semialdehyde | C4H6O3 | C00232 | 1.46 | 0.55 | 0.002164502 | 2.66 |
| Benzylamine | C7H9N | C15562 | 0.77 | -0.37 | 0.002164502 | 2.66 |
| p-Cresol | C7H8O | C01468 | 2.61 | 1.38 | 0.004329004 | 2.36 |
| 3-Methyl-2-oxovaleric acid | C6H10O3 | C03465 | 1.62 | 0.7 | 0.008658009 | 2.06 |
| 1,2,3-Trihydroxybenzene | C6H6O3 | C01108 | 2.87 | 1.52 | 0.002164502 | 2.66 |
| L-Glutamic gamma-semialdehyde | C5H9NO3 | C01165 | 2.88 | 1.52 | 0.002164502 | 2.66 |
| Anabasine | C10H14N2 | C06180 | 1.41 | 0.49 | 0.004329004 | 2.36 |
| Methyloxaloacetate | C5H6O5 | C06030 | 2.51 | 1.33 | 0.002164502 | 2.66 |
| L-Lysine | C6H14N2O2 | C00047 | 0.44 | -1.18 | 0.002164502 | 2.66 |
| 1,2-Epoxy-p-menth-8-ene | C10H16O | C07271 | 2.07 | 1.05 | 0.002164502 | 2.66 |
| Indoleacetaldehyde | C10H9NO | C00637 | 1.73 | 0.79 | 0.002164502 | 2.66 |
| 3D-3,5_4-Trihydroxycyclohexane-1,2-dione | C6H8O5 | C04287 | 0.56 | -0.83 | 0.002164502 | 2.66 |
| D-synephrine | C9H13NO2 | C01869 | 0.89 | -0.16 | 0.002164502 | 2.66 |
| (R)-4-Hydroxymandelate | C8H8O4 | C05343 | 1.89 | 0.92 | 0.002164502 | 2.66 |
| Nalpha-Methylhistidine | C7H11N3O2 | C03298 | 1.51 | 0.6 | 0.002164502 | 2.66 |
| Citrulline | C6H13N3O3 | C00327 | 2.05 | 1.03 | 0.002164502 | 2.66 |
| Herniarin | C10H8O3 | C09268 | 10.17 | 3.35 | 0.002164502 | 2.66 |
| Sorbitol | C6H14O6 | C00794 | 0.74 | -0.44 | 0.002164502 | 2.66 |
| Phosphorylcholine | C5H15NO4P | C00588 | 0.3 | -1.72 | 0.002164502 | 2.66 |
| Homoisocitrate | C7H10O7 | C05662 | 0.73 | -0.45 | 0.008658009 | 2.06 |
| N6-Acetyl-L-lysine | C8H16N2O3 | C02727 | 2.39 | 1.25 | 0.002164502 | 2.66 |
| Carbendazim | C9H9N3O2 | C10897 | 0.57 | -0.82 | 0.002164502 | 2.66 |
| 2-Amino-2-deoxy-D-gluconate | C6H13NO6 | C03752 | 4.1 | 2.04 | 0.002164502 | 2.66 |
| Deoxycytidine | C9H13N3O4 | C00881 | 0.25 | -2.02 | 0.008658009 | 2.06 |
| Deoxyuridine | C9H12N2O5 | C00526 | 0.7 | -0.52 | 0.002164502 | 2.66 |
| Ubiquinone-1 | C14H18O4 | C00399 | 0.53 | -0.92 | 0.002164502 | 2.66 |
| Equol | C15H14O3 | C14131 | 1.26 | 0.33 | 0.002164502 | 2.66 |
| Abscisic alcohol | C15H22O3 | C13456 | 1.24 | 0.31 | 0.002164502 | 2.66 |
| (9Z,11E,13E)-Octadecatrienoic acid | C18H30O2 | C08315 | 5.3 | 2.41 | 0.002164502 | 2.66 |
| N2-gamma-Glutamylglutamine | C10H17N3O6 | C05283 | 0.69 | -0.54 | 0.002164502 | 2.66 |
| Glutathione amide | C10H18N4O5S | C19689 | 0.65 | -0.62 | 0.002164502 | 2.66 |
| Dehydroepiandrosterone | C19H28O2 | C01227 | 0.17 | -2.55 | 0.002164502 | 2.66 |
| Eicosadienoic acid | C20H36O2 | C16525 | 0.59 | -0.76 | 0.002164502 | 2.66 |
| Palmitoylethanolamide | C18H37NO2 | C16512 | 0.46 | -1.12 | 0.002164502 | 2.66 |
| 11b-Hydroxyandrost-4-ene-3,17-dione | C19H26O3 | C05284 | 1.53 | 0.62 | 0.025974026 | 1.59 |
| Quercetin | C15H10O7 | C00389 | 2.95 | 1.56 | 0.002164502 | 2.66 |
| Arachidonic acid | C20H32O2 | C00219 | 1.85 | 0.89 | 0.002164502 | 2.66 |
| 7-Methylcapillarisin | C17H14O6 | C17785 | 1.24 | 0.31 | 0.002164502 | 2.66 |
| Phenolphthalein | C20H14O4 | C14286 | 4.01 | 2 | 0.002164502 | 2.66 |
| Brompheniramine | C16H19BrN2 | C06857 | 1.3 | 0.38 | 0.002164502 | 2.66 |
| 8,9-EET | C20H32O3 | C14769 | 1.21 | 0.27 | 0.002164502 | 2.66 |
| 8,9-DiHETrE | C20H34O4 | C14773 | 0.43 | -1.21 | 0.002164502 | 2.66 |
| Cyclic AMP | C10H12N5O6P | C00575 | 2.87 | 1.52 | 0.002164502 | 2.66 |
| Docosapentaenoic acid (22n-3) | C22H34O2 | C16513 | 0.05 | -4.29 | 0.002164502 | 2.66 |
| Andrographolide | C20H30O5 | C20214 | 0.44 | -1.18 | 0.002164502 | 2.66 |
| Prostaglandin H2 | C20H32O5 | C00427 | 1.47 | 0.55 | 0.002164502 | 2.66 |
| Griseofulvin | C17H17ClO6 | C06686 | 2.4 | 1.27 | 0.002164502 | 2.66 |
| Dihydrocortisol | C21H32O5 | C05471 | 2.34 | 1.23 | 0.002164502 | 2.66 |
| Strictosidine aglycone | C21H24N2O4 | C03309 | 0.54 | -0.89 | 0.002164502 | 2.66 |
| Lithocholic acid | C24H40O3 | C03990 | 0.62 | -0.69 | 0.002164502 | 2.66 |
| Cholesterol | C27H46O | C00187 | 5.88 | 2.55 | 0.002164502 | 2.66 |
| S-Adenosyl-4-methylthio-2-oxobutanoate | C15H20N5O6S | C04425 | 0.62 | -0.69 | 0.002164502 | 2.66 |
| GF 109203X | C25H24N4O2 | C11238 | 2.58 | 1.37 | 0.002164502 | 2.66 |
| alpha-Tocopherol | C29H50O2 | C02477 | 0.12 | -3.06 | 0.002164502 | 2.66 |
| Quinapril | C25H30N2O5 | C07398 | 0.44 | -1.18 | 0.002164502 | 2.66 |
| Cyanidin 3-galactoside | C21H21O11 | C08647 | 2.25 | 1.17 | 0.002164502 | 2.66 |
| 20-Hydroxyecdysone | C27H44O7 | C02633 | 0.35 | -1.52 | 0.002164502 | 2.66 |
| Leukotriene D4 | C25H40N2O6S | C05951 | 0.67 | -0.57 | 0.002164502 | 2.66 |
| Taurocholic acid | C26H45NO7S | C05122 | 0.49 | -1.02 | 0.025974026 | 1.59 |
| Lutein | C40H56O2 | C08601 | 0.51 | -0.96 | 0.008658009 | 2.06 |
| Cyanidin 3-O-sophoroside | C27H31O16 | C16306 | 0.44 | -1.18 | 0.008658009 | 2.06 |
| Reduced coenzyme F420 | C29H38N5O18P | C01080 | 0.43 | -1.2 | 0.004329004 | 2.36 |
| Ansamitocinoside P-3 | C37H51ClN2O14 | C20139 | 0.27 | -1.89 | 0.002164502 | 2.66 |
| Itaconic acid | C5H6O4 | C00490 | 0.78 | -0.37 | 0.004329004 | 2.36 |
| Leucine | C6H13NO2 | C16439 | 3.03 | 1.6 | 0.025974026 | 1.59 |
| L-Phenylalanine | C9H11NO2 | C00079 | 1.52 | 0.6 | 0.002164502 | 2.66 |
| Terephthalate | C8H6O4 | C06337 | 3.98 | 1.99 | 0.008658009 | 2.06 |
| 3,4-Dihydroxybenzeneacetic acid | C8H8O4 | C01161 | 0.59 | -0.77 | 0.002164502 | 2.66 |
| D-Mannose | C6H12O6 | C00159 | 2.77 | 1.47 | 0.015151515 | 1.82 |
| 3,4-Dihydroxymandelic acid | C8H8O5 | C05580 | 3.5 | 1.81 | 0.002164502 | 2.66 |
| Pantothenic acid | C9H17NO5 | C00864 | 1.23 | 0.3 | 0.002164502 | 2.66 |
| Catechin | C15H14O6 | C06562 | 0.35 | -1.51 | 0.002164502 | 2.66 |
| Gingerol | C17H26O4 | C10462 | 1.97 | 0.97 | 0.002164502 | 2.66 |
| 9-cis-Retinoic acid | C20H28O2 | C15493 | 0.48 | -1.05 | 0.002164502 | 2.66 |
| Dhurrin | C14H17NO7 | C05143 | 0.67 | -0.59 | 0.004329004 | 2.36 |
| Indole-3-acetyl-beta-1-D-glucoside | C16H19NO7 | C04197 | 2.08 | 1.06 | 0.002164502 | 2.66 |
| Prostaglandin F2a | C20H34O5 | C00639 | 1.98 | 0.99 | 0.002164502 | 2.66 |
| 20-Carboxy-leukotriene B4 | C20H30O6 | C05950 | 2.08 | 1.06 | 0.002164502 | 2.66 |
| Geniposidic acid | C16H22O10 | C11673 | 2.47 | 1.31 | 0.015151515 | 1.82 |
| Mitragynine | C23H30N2O4 | C09226 | 0.06 | -4.01 | 0.002164502 | 2.66 |
| Losartan | C22H23ClN6O | C07072 | 1.37 | 0.45 | 0.041125541 | 1.39 |
| Abscisic acid glucose ester | C21H30O9 | C15970 | 2.3 | 1.2 | 0.002164502 | 2.66 |
| Petunidin 3-glucoside | C22H23O12 | C12139 | 1.76 | 0.81 | 0.002164502 | 2.66 |
| 1-Kestose | C18H32O16 | C03661 | 3.62 | 1.86 | 0.002164502 | 2.66 |
| 11-Hydroxyiridodial glucoside pentaacetate | C26H36O13 | C11666 | 0.31 | -1.68 | 0.002164502 | 2.66 |
| Loganin pentaacetate | C27H36O15 | C11663 | 0.55 | -0.87 | 0.002164502 | 2.66 |
| Delphinidin 3-O-(6-caffeoyl-beta-D-glucoside) | C30H27O15 | C16367 | 0.04 | -4.83 | 0.002164502 | 2.66 |
