# Supplementary material for: Response mechanism of carbon metabolism of Pinus massoniana to gradient high temperature and drought stress
Source: BMC Genomics. 2024 Feb 12;25:166. doi: 10.1186/s12864-024-10054-2 (PMC10860282; doi:10.1186/s12864-024-10054-2)
Supplement: Supplementary file 11 — Additional file 11. [file 12864_2024_10054_MOESM11_ESM.docx]

Table S14 summary table of 192 common differential metabolites under three treatments.

| **name** | **KEGG** |
| --- | --- |
| Isovaleric acid | C08262 |
| 3-Methylthiopropanamine | C03354 |
| 2-Phenylethanol | C05853 |
| Benzaldehyde | C00261 |
| m-Cresol | C01467 |
| 5-Methyl-2-furancarboxaldehyde | C11115 |
| Imidazole-4-acetaldehyde | C05130 |
| Uracil | C00106 |
| Creatinine | C00791 |
| Deoxyribose | C01801 |
| 5-Hydroxypentanoic acid | C02804 |
| L-Allothreonine | C05519 |
| Tyrosol | C06044 |
| N,N-Dimethylaniline | C02846 |
| Phenylethylamine | C05332 |
| Niacinamide | C00153 |
| Phloroglucinol | C02183 |
| Quinoline | C06413 |
| 1,1-Dimethylbiguanide | C07151 |
| Pipecolic acid | C00408 |
| L-Isoleucine | C00407 |
| L-Ribulose | C00310 |
| Phenelzine | C07430 |
| Gamma-terpinene | C09900 |
| p-Aminobenzoic acid | C00568 |
| 3,4-Dihydroxybenzaldehyde | C16700 |
| N-methyl-L-glutamic Acid | C01046 |
| (2R,5S)-2,5-Diaminohexanoate | C05161 |
| L-Glutamine | C00064 |
| 3,4-Dihydro-2H-1-benzopyran-2-one | C02274 |
| Phthalic acid | C01606 |
| L-2-Hydroxyglutaric acid | C03196 |
| D-Lyxose | C00476 |
| D-Ribose | C00121 |
| 2-Phenylethyl acetate | C12303 |
| 7-Methylxanthine | C16353 |
| (S)-4-Hydroxymandelate | C03198 |
| 8-Amino-7-oxononanoate | C01092 |
| 2-Biphenylol | C02499 |
| Levetiracetam | C07841 |
| (2S,5S)-trans-Carboxymethylproline | C17366 |
| N-Acetylleucine | C02710 |
| D-Galacturonolactone | C06430 |
| L-Bornesitol | C03660 |
| 5-Deoxy-D-glucuronate | C16737 |
| D-Psicose | C06468 |
| (S)-beta-Tyrosine | C21308 |
| Hydroxyphenyllactic acid | C03672 |
| 5-Oxo-1,2-campholide | C02952 |
| Choline sulfate | C00919 |
| Sebacic acid | C08277 |
| Homocitrulline | C02427 |
| Homo-L-arginine | C01924 |
| N-Acetylglutamic acid | C00624 |
| Diaminopimelic acid | C00666 |
| 6-Methoxymellein | C02381 |
| Myristicin | C10480 |
| Methoxamine | C07513 |
| (-)-Bornesitol | C03659 |
| Dodecanoic acid | C02679 |
| Apiole | C10429 |
| Ibuprofen | C01588 |
| 3-[(1-Carboxyvinyl)oxy]benzoate | C20772 |
| L-Kynurenine | C00328 |
| N-Acetyldemethylphosphinothricin | C17949 |
| (+)-7-Isojasmonic acid | C16317 |
| Cis-zeatin | C00371 |
| 2-trans,6-trans-Farnesal | C03461 |
| Cerulenin | C12058 |
| Prephenate | C00254 |
| Myristoleic acid | C08322 |
| 6-Hydroxymelatonin | C05643 |
| Alantolactone | C09289 |
| gamma-L-Glutamyl-L-cysteine | C00669 |
| Benzo[k]fluoranthene | C14321 |
| Glycerophosphocholine | C00670 |
| Parthenin | C09523 |
| Qing Hau Sau | C09538 |
| Adenosine | C00212 |
| (S)-Coclaurine | C06161 |
| (R)-Coclaurine | C06349 |
| Norizalpinin | C10044 |
| Genistein | C06563 |
| All-trans-13,14-dihydroretinol | C15492 |
| Thienamycin | C06664 |
| 5a-Androstane-3b,17b-diol | C12525 |
| Cyclopeptine | C20579 |
| 9-Riburonosyladenine | C11501 |
| 1-Methyladenosine | C02494 |
| (R,S)-Coclaurine | C06348 |
| N1,N12-Diacetylspermine | C03413 |
| Eriodictyol | C05631 |
| Aurin | C14213 |
| 9(S)-HPOT | C16321 |
| Prunasin | C00844 |
| 5-Nitro-2-(3-phenylpropylamino)benzoic acid | C13705 |
| Kaempferide | C10098 |
| Sphinganine | C00836 |
| cis-Dihydroquercetin | C12316 |
| 11alpha,17beta-Dihydroxyandrost-4-en-3-one | C15306 |
| Bisdemethoxycurcumin | C17743 |
| Alprazolam | C06817 |
| beta-D-Galactosyl-(1->4)-L-rhamnose | C19758 |
| Aflatoxin B1 | C06800 |
| N(beta)-Epoxysuccinamoyl-DAP-Val | C20965 |
| Myricetin | C10107 |
| Deoxy-5-methylcytidylate | C03495 |
| gamma-L-Glutamyl-L-cysteinyl-beta-alanine | C04544 |
| dTMP | C00364 |
| Citalopram | C07572 |
| 2,4-Dioxotetrahydropyrimidine D-ribonucleotide | C04639 |
| Carnosol | C09069 |
| Cannabielsoin | C20218 |
| Norsanguinarine | C05191 |
| Isopentenyl adenosine | C16427 |
| p-Coumaroyl quinic acid | C12208 |
| 21-Deoxycortisol | C05497 |
| 2-Hydroxy-6-pentadecylbenzoic acid | C10759 |
| 6-Keto-prostaglandin F1a | C05961 |
| 17-O-Acetylnorajmaline | C11809 |
| Laudanosine | C09558 |
| Niaprazine | D07333 |
| Cortisol | C00735 |
| 11b,21-Dihydroxy-3,20-oxo-5b-pregnan-18-al | C05473 |
| Tamoxifen | C07108 |
| Mesoridazine | C07143 |
| Sufentanil | C08022 |
| Ursodeoxycholic acid | C07880 |
| Aloesin | C08994 |
| Ergocalciferol | C05441 |
| Paspalicine | C20553 |
| Kaempferol 3-O-beta-D-xyloside | C20727 |
| Etiocholanolone glucuronide | C11136 |
| Isoquercitrin | C05623 |
| Withaferin A | C08841 |
| N-Acetyl-O-demethylpuromycin-5'-phosphate | C07030 |
| Cyanidin 3-O-(6-O-p-coumaroyl)glucoside-5-O-glucoside | C12096 |
| Phenylacetaldehyde | C00601 |
| 3-Methylthiopropionic acid | C08276 |
| Glutaric acid | C00489 |
| L-Aspartic acid | C00049 |
| Mandelonitrile | C00561 |
| Adenine | C00147 |
| Citramalic acid | C00815 |
| D-Xylose | C00181 |
| (Z)-4-Hydroxy-6-dodecenoic acid lactone | C03107 |
| Fructose-1P | C10906 |
| trans-2-Hydroxycinnamate | C01772 |
| Vanylglycol | C05594 |
| Phenyllactate | C05607 |
| Tropate | C01456 |
| Norepinephrine | C00547 |
| Beta-Glycerophosphoric acid | C02979 |
| Dehydroascorbate | C05422 |
| Guanidinosuccinic acid | C03139 |
| Gluconic acid | C00257 |
| D-(+)-Glucose | C00293 |
| Azelaic acid | C08261 |
| Xanthoxylin | C10726 |
| Vanillylmandelic acid | C05584 |
| L-Tryptophan | C00078 |
| (-)-Jasmonic acid | C08491 |
| N-Acetyl-D-glucosamine | C00140 |
| 6-Acetyl-D-glucose | C02655 |
| Methyl jasmonate | C11512 |
| Galactosylglycerol | C05401 |
| Shikimate 3-phosphate | C03175 |
| Galactose 1-phosphate | C00103 |
| (S)-Abscisic acid | C06082 |
| Phloretin | C00774 |
| Hexadecanedioate | C19615 |
| 4-(beta-D-Glucosyloxy)benzoate | C03993 |
| Diosmetin | C10038 |
| EPA (d5) | C06428 |
| (-)-Epigallocatechin | C12136 |
| 12-KETE | C14807 |
| 1-O-Vanilloyl-beta-D-glucose | C20470 |
| Prostaglandin A2 | C05953 |
| Delta-12-Prostaglandin J2 | C05958 |
| (5Z,9E,14Z)-(8xi,11R,12S)-11,12-epoxy-8-hydroxyicosa-5,9,14-trienoic Acid | C04849 |
| Dattelic acid | C10434 |
| Erucic acid | C08316 |
| Fructose 1,6-bisphosphate | C00354 |
| Trehalose | C01083 |
| (-)-Wikstromol | C10725 |
| Epigallocatechin gallate | C09731 |
| 6-Methoxyluteolin 7-rhamnoside | C10104 |
| Myricitrin | C10108 |
| Delphinidin 3-(6-p-coumaroyl)glucoside | C16370 |
| Neomycin | C01737 |
| Quercetin 3-O-beta-D-glucosyl-(1->2)-beta-D-glucoside | C12667 |
| Delphin | C16312 |
