# Supplementary material for: Response mechanism of carbon metabolism of Pinus massoniana to gradient high temperature and drought stress
Source: BMC Genomics. 2024 Feb 12;25:166. doi: 10.1186/s12864-024-10054-2 (PMC10860282; doi:10.1186/s12864-024-10054-2)
Supplement: Supplementary file 12 — Additional file 12. [file 12864_2024_10054_MOESM12_ESM.docx]

Table S15 KEGG enrichment analysis of T25CK vs T25Z differential metabolites.

| **name** | **KEGG** | **T25Z_Mean** | **T25Z_Median** | **T25Z_RSD** | **T25CK_Mean** | **T25CK_Median** | **T25CK_RSD** | **FC** | **log2FC** |
| --- | --- | --- | --- | --- | --- | --- | --- | --- | --- |
| Isovaleric acid | C08262 | 9240650.28 | 8927375.49 | 11.59 | 17039572.37 | 16376799.36 | 11.21 | 0.54 | -0.88 |
| gamma-Aminobutyric acid | C00334 | 2367476899 | 2377372291 | 9.41 | 1958675446 | 1965710389 | 4.12 | 1.21 | 0.27 |
| 3-Methylthiopropanamine | C03354 | 9826628.17 | 9903798.43 | 4.38 | 12265576.19 | 12264954.78 | 3.72 | 0.8 | -0.32 |
| 2-Phenylethanol | C05853 | 150397450.5 | 150086496.5 | 2 | 111202904.3 | 111487931.1 | 2.06 | 1.35 | 0.44 |
| Benzaldehyde | C00261 | 5471534.74 | 5323374.37 | 9.11 | 6789002.54 | 6799643.42 | 5.81 | 0.81 | -0.31 |
| m-Cresol | C01467 | 16131751.54 | 16197803.6 | 31.78 | 7999955.43 | 7858063.49 | 24.55 | 2.02 | 1.01 |
| 5-Methyl-2-furancarboxaldehyde | C11115 | 117765979.4 | 116894357.9 | 7.31 | 174441688 | 176812152.1 | 9.9 | 0.68 | -0.57 |
| Imidazole-4-acetaldehyde | C05130 | 6718941 | 6228848.94 | 31.47 | 2715438.38 | 2647403.26 | 8.82 | 2.47 | 1.31 |
| Uracil | C00106 | 131038629.3 | 131080774.5 | 2.41 | 90712788.84 | 90529927.54 | 3.05 | 1.44 | 0.53 |
| Creatinine | C00791 | 191117959.5 | 191362836.7 | 1.23 | 70025168.98 | 69774682.06 | 2.85 | 2.73 | 1.45 |
| 2-Heptanone | C08380 | 87669629.5 | 87708922.98 | 5.98 | 29060531.33 | 17048977.93 | 72.12 | 3.02 | 1.59 |
| Deoxyribose | C01801 | 14354466.37 | 14246371.9 | 3.51 | 16625530.48 | 16477108.01 | 5.25 | 0.86 | -0.21 |
| Glutarate semialdehyde | C03273 | 37065675.15 | 37030998.58 | 1.73 | 120588221.3 | 135752002 | 32.09 | 0.31 | -1.7 |
| 5-Hydroxypentanoic acid | C02804 | 1706952.15 | 1838505.35 | 24.57 | 5510211.88 | 5640315.44 | 6.48 | 0.31 | -1.69 |
| 2-Methylserine | C02115 | 9632226.08 | 9564768.58 | 3.31 | 7511688.19 | 7469263.07 | 2.98 | 1.28 | 0.36 |
| L-Allothreonine | C05519 | 6261408.06 | 6237899.99 | 2.14 | 2626702.91 | 2651673.81 | 3.47 | 2.38 | 1.25 |
| Tyrosol | C06044 | 27826227.7 | 28584351.93 | 16.27 | 16377847.75 | 16522578.76 | 4.87 | 1.7 | 0.76 |
| N,N-Dimethylaniline | C02846 | 5983377.65 | 4238054.57 | 75.92 | 2547440.97 | 2601077.64 | 6.49 | 2.35 | 1.23 |
| Phenylethylamine | C05332 | 59400335.54 | 59192096.31 | 1.8 | 100654096.1 | 100280192.4 | 1.31 | 0.59 | -0.76 |
| Niacinamide | C00153 | 9681225 | 9570068.89 | 3.29 | 16522293.45 | 16515669.17 | 2.13 | 0.59 | -0.77 |
| Thymine | C00178 | 7525106.74 | 7329258.37 | 9.29 | 3601767.3 | 3287511.85 | 22.08 | 2.09 | 1.06 |
| Phloroglucinol | C02183 | 33954787.71 | 33913337.4 | 3.27 | 52787261.99 | 52875062.04 | 1.8 | 0.64 | -0.64 |
| Dihydrothymine | C00906 | 10190263.46 | 10135082.43 | 5.22 | 15636970.41 | 15677960.3 | 3.28 | 0.65 | -0.62 |
| Quinoline | C06413 | 379365845.3 | 391570037.1 | 10.63 | 307905891 | 305986466.2 | 8.03 | 1.23 | 0.3 |
| 1,1-Dimethylbiguanide | C07151 | 80291789.39 | 78619710.33 | 5.96 | 142478083.2 | 141085096.7 | 3.14 | 0.56 | -0.83 |
| Pipecolic acid | C00408 | 830373409.4 | 871018050.5 | 15.71 | 483241765.1 | 480589294.8 | 21.2 | 1.72 | 0.78 |
| L-Isoleucine | C00407 | 307330105.5 | 306424428.6 | 5.85 | 66237188.58 | 66229297.49 | 5.42 | 4.64 | 2.21 |
| Creatine | C00300 | 7968602.33 | 7920858.5 | 3.42 | 3038353.58 | 2996630.03 | 7.75 | 2.62 | 1.39 |
| L-Ribulose | C00310 | 15764300.84 | 15884683.77 | 2.43 | 18256894.47 | 18300792.5 | 3.8 | 0.86 | -0.21 |
| 1,3-Dihydro-(2H)-indol-2-one | C12312 | 1272222.25 | 1276751.16 | 8.41 | 54889453.33 | 55301576.9 | 2.95 | 0.02 | -5.43 |
| L-Asparagine | C00152 | 7716057.05 | 7560167.74 | 6.45 | 11440968.7 | 11749125.14 | 6.54 | 0.67 | -0.57 |
| Chavicol | C16930 | 2081488.75 | 2149012.25 | 10.86 | 2732110.12 | 2709266.48 | 10.15 | 0.76 | -0.39 |
| Ribitol | C00474 | 56796373.59 | 61230640.25 | 23.55 | 24972822.4 | 24999534.87 | 11.95 | 2.27 | 1.19 |
| p-Octopamine | C04227 | 132753713.6 | 132496278.4 | 9.38 | 112147592.6 | 115175728.7 | 9.12 | 1.18 | 0.24 |
| Phenelzine | C07430 | 4313994.96 | 4306348.7 | 1.28 | 5353426.96 | 5321215.38 | 1.56 | 0.81 | -0.31 |
| Gamma-terpinene | C09900 | 23822198.76 | 23910086.37 | 2.19 | 20973568.65 | 20882328.61 | 1.72 | 1.14 | 0.18 |
| Eucalyptol | C09844 | 15639886 | 15592490.78 | 2.11 | 21677407.92 | 21512577.89 | 5.74 | 0.72 | -0.47 |
| Tyramine | C00483 | 44055832.29 | 46807824.32 | 19.29 | 23161515.4 | 22256211.29 | 9.61 | 1.9 | 0.93 |
| p-Aminobenzoic acid | C00568 | 2322688.47 | 2309745.6 | 7.21 | 4436983.75 | 4451023.36 | 6.78 | 0.52 | -0.93 |
| 3,4-Dihydroxybenzaldehyde | C16700 | 46548953.25 | 46967660.8 | 6.13 | 19461516.86 | 19071619.95 | 16.81 | 2.39 | 1.26 |
| 5-Hydroxymethyluracil | C03088 | 77671596.39 | 76014795.49 | 8.69 | 90236701.26 | 90083457.24 | 3.66 | 0.86 | -0.22 |
| trans-trans-Muconic acid | C02480 | 26167705.69 | 26202889.3 | 3.46 | 21012302.29 | 20889162.94 | 2.91 | 1.25 | 0.32 |
| N-methyl-L-glutamic Acid | C01046 | 9096770.58 | 9194027.77 | 2.09 | 2612639.55 | 2760965.2 | 14.06 | 3.48 | 1.8 |
| 4-Guanidinobutanoic acid | C01035 | 24950161.88 | 25199354.33 | 8.77 | 15868056.04 | 13798990.79 | 32.37 | 1.57 | 0.65 |
| (2R,5S)-2,5-Diaminohexanoate | C05161 | 3797782.68 | 3789915.27 | 5.57 | 8582397.5 | 8326048.51 | 5.72 | 0.44 | -1.18 |
| L-Glutamine | C00064 | 485262945.4 | 485929789.1 | 4.01 | 242079089.9 | 250579618.4 | 8.6 | 2 | 1 |
| L-Glutamic acid | C00025 | 2400987719 | 2397682435 | 1.69 | 1801965251 | 1816522800 | 4.82 | 1.33 | 0.41 |
| 3,4-Dihydro-2H-1-benzopyran-2-one | C02274 | 10428339.95 | 10397249.73 | 16.8 | 6342234.25 | 5870427.51 | 18.47 | 1.64 | 0.72 |
| Phthalic acid | C01606 | 20805971.56 | 21450153.42 | 21.35 | 34618951.41 | 34691237.9 | 9.84 | 0.6 | -0.73 |
| L-2-Hydroxyglutaric acid | C03196 | 13888095.57 | 13830225.49 | 1.94 | 13314224.7 | 13242136.68 | 1.93 | 1.04 | 0.06 |
| D-Lyxose | C00476 | 42651074.12 | 43292840.76 | 4.13 | 48087466.33 | 48446571.63 | 2.76 | 0.89 | -0.17 |
| D-Ribose | C00121 | 3171639.24 | 3099528.72 | 12.59 | 4478144.5 | 4302723.42 | 25.86 | 0.71 | -0.5 |
| (+)-(S)-Carvone | C11383 | 60025876.64 | 63041174.28 | 38.79 | 28734892.88 | 26036688.86 | 28.41 | 2.09 | 1.06 |
| N-Methyltyramine | C02442 | 24538581.56 | 24686671.3 | 6.25 | 3235301.52 | 3104279.58 | 9.86 | 7.58 | 2.92 |
| Methyl 2-hydroxybenzoate | C12305 | 10539113.5 | 10425878.9 | 3.25 | 12863041.2 | 12540735.54 | 5.23 | 0.82 | -0.29 |
| Imidazol-5-yl-pyruvate | C03277 | 8086028.04 | 8039991.04 | 3.45 | 8635162.74 | 8716515.87 | 3.45 | 0.94 | -0.09 |
| 2,3-Butanediol | C00265 | 165607942 | 168011458.4 | 6.64 | 83708331.05 | 83029898.07 | 25.01 | 1.98 | 0.98 |
| Pelargonic acid | C01601 | 5280937.82 | 5111693.27 | 13.87 | 3996059.91 | 3966784.91 | 7.54 | 1.32 | 0.4 |
| 1,5-Naphthalenediamine | C19463 | 107465409.4 | 112855207.6 | 12.68 | 71540075.62 | 74741048.86 | 16.39 | 1.5 | 0.59 |
| Nicotine | C00745 | 16435788.2 | 16638645.9 | 4.13 | 22506213.58 | 22631059.89 | 4.71 | 0.73 | -0.45 |
| 2-Phenylethyl acetate | C12303 | 26296784.07 | 26789796.41 | 6.02 | 14394010.68 | 14623532.54 | 8.14 | 1.83 | 0.87 |
| 7-Methylxanthine | C16353 | 11294501.45 | 11287924.4 | 1.94 | 10722036.41 | 10747878.94 | 2.31 | 1.05 | 0.08 |
| Desaminotyrosine | C01744 | 11398518.9 | 12281087.83 | 33.59 | 5553874.99 | 4691197.3 | 37 | 2.05 | 1.04 |
| Quinolinic acid | C03722 | 86632951.72 | 81872207.05 | 11.55 | 117202368 | 118125130.5 | 7.3 | 0.74 | -0.44 |
| 3-(2-Hydroxyphenyl)propanoic acid | C01198 | 171234039 | 171551156 | 2.07 | 80428150.03 | 80303918.37 | 2.4 | 2.13 | 1.09 |
| (1S,4R)-1-Hydroxy-2-oxolimonene | C11937 | 13671088.71 | 13546803.2 | 8.16 | 9142941.85 | 9239386.26 | 9.16 | 1.5 | 0.58 |
| (S)-4-Hydroxymandelate | C03198 | 221787795.2 | 213552707.8 | 13.87 | 113663210.2 | 110516210.9 | 17.77 | 1.95 | 0.96 |
| 8-Amino-7-oxononanoate | C01092 | 9715564.66 | 9679017.97 | 1.36 | 2162445.16 | 2167107.02 | 3.63 | 4.49 | 2.17 |
| 2-Biphenylol | C02499 | 14603583.41 | 15091843.25 | 12.11 | 5974272.84 | 5867396.86 | 19.63 | 2.44 | 1.29 |
| Levetiracetam | C07841 | 78868524.27 | 79795034.11 | 4.16 | 28666155.36 | 29095281.92 | 9.12 | 2.75 | 1.46 |
| Dihydroxyacetone phosphate | C00111 | 21619338.37 | 21709325.45 | 3.27 | 20025292.79 | 19956219.67 | 2.5 | 1.08 | 0.11 |
| Menadione | C05377 | 31010854.62 | 30895154.21 | 2.66 | 9706334.48 | 9841729.24 | 4.53 | 3.19 | 1.68 |
| 4-Quinolinecarboxylic acid | C06414 | 32567055.98 | 37223541.28 | 25.52 | 16626381.06 | 18014994.04 | 29.74 | 1.96 | 0.97 |
| (2S,5S)-trans-Carboxymethylproline | C17366 | 60107246.71 | 57186017.71 | 12.79 | 23364898.56 | 20958291.45 | 25.22 | 2.57 | 1.36 |
| N-Acetylleucine | C02710 | 9703453.58 | 9491625.23 | 12.44 | 6108552.02 | 6110442.87 | 12.58 | 1.59 | 0.67 |
| N-Acetyl-L-glutamate 5-semialdehyde | C01250 | 30435247.99 | 30879136.08 | 16.29 | 22567801 | 22627568.3 | 9.95 | 1.35 | 0.43 |
| L-Arginine | C00062 | 30296582.39 | 30584742.33 | 47.13 | 9013640.71 | 8545382.93 | 15.67 | 3.36 | 1.75 |
| N-Acetyl-L-aspartic acid | C01042 | 14213146.08 | 14352739.44 | 2.73 | 15441980.31 | 15287302.99 | 4.51 | 0.92 | -0.12 |
| Ascorbate | C00072 | 109060881.1 | 79746520.73 | 49.58 | 916064489.4 | 919786967.4 | 4.33 | 0.12 | -3.07 |
| D-Galacturonolactone | C06430 | 49469281.19 | 49563915.33 | 7.17 | 33025393.26 | 32719917.36 | 3.75 | 1.5 | 0.58 |
| L-Bornesitol | C03660 | 25456098 | 22522980.21 | 65.95 | 6054575.53 | 5003893.37 | 44.83 | 4.2 | 2.07 |
| Coniferyl aldehyde | C02666 | 279268424.8 | 275036459.2 | 5.16 | 123302947.6 | 96212794.48 | 52.87 | 2.26 | 1.18 |
| 5-Deoxy-D-glucuronate | C16737 | 23372731.81 | 23302958.05 | 5.13 | 17608865.7 | 17783465.17 | 1.81 | 1.33 | 0.41 |
| Geranyl acetate | C09861 | 27930212.9 | 27712419.5 | 8.07 | 3102081.66 | 2863298.26 | 65.54 | 9 | 3.17 |
| D-Psicose | C06468 | 112068534.5 | 114686622.4 | 4.29 | 146217095.4 | 146369435.9 | 9.6 | 0.77 | -0.38 |
| Coniferyl alcohol | C00590 | 64136795.53 | 63481013.6 | 2.8 | 30571074.58 | 31349137.23 | 9.96 | 2.1 | 1.07 |
| (S)-beta-Tyrosine | C21308 | 6192961.63 | 6066560.7 | 5.38 | 6962509.79 | 6968117.18 | 4.41 | 0.89 | -0.17 |
| Hydroxyphenyllactic acid | C03672 | 43626944.1 | 42942905.45 | 4.15 | 38430910.1 | 38393923.28 | 2.54 | 1.14 | 0.18 |
| 5-Oxo-1,2-campholide | C02952 | 19748590.3 | 20897273.55 | 21.03 | 8947471.92 | 8980700.44 | 34.38 | 2.21 | 1.14 |
| Choline sulfate | C00919 | 385390335.7 | 387509622.6 | 3.45 | 69339493.44 | 69032284.66 | 3.38 | 5.56 | 2.47 |
| Sebacic acid | C08277 | 17909807.62 | 17232200.34 | 9.63 | 3500657 | 3596705.78 | 6.18 | 5.12 | 2.36 |
| Phosphoserine | C01005 | 4583551.05 | 4692341.09 | 7 | 3020584.14 | 2945982.49 | 8.16 | 1.52 | 0.6 |
| Undecanoic acid | C17715 | 7542522.46 | 7592666.86 | 8.84 | 10094983.35 | 10107859.97 | 4.41 | 0.75 | -0.42 |
| 6-Acetamido-3-oxohexanoate | C03682 | 44627932.99 | 42897021.13 | 17.35 | 32046776.92 | 29955136.37 | 22.84 | 1.39 | 0.48 |
| Homocitrulline | C02427 | 19110294.58 | 18750459.96 | 15.61 | 33288979.19 | 32817511.92 | 8.07 | 0.57 | -0.8 |
| Homo-L-arginine | C01924 | 66240475.71 | 66134988.23 | 1.84 | 40642223.02 | 38111858.02 | 17.74 | 1.63 | 0.7 |
| N-Acetylglutamic acid | C00624 | 3212724.04 | 3238535.37 | 4.7 | 22919647.02 | 22910082.91 | 2.58 | 0.14 | -2.83 |
| Diaminopimelic acid | C00666 | 29561109.36 | 29252170.37 | 3.16 | 13285592.23 | 13376702.18 | 3.24 | 2.23 | 1.15 |
| Kynurenic acid | C01717 | 8464955.85 | 4851192.04 | 70.58 | 34462972.51 | 34253931.62 | 22.81 | 0.25 | -2.03 |
| 6-Methoxymellein | C02381 | 36274859.36 | 36175322.21 | 4.2 | 44275958.1 | 43592377.18 | 7.7 | 0.82 | -0.29 |
| N,N-Diethyl-m-toluamide | C10935 | 9500225.15 | 9484727.21 | 3.13 | 3482878.61 | 3508524.2 | 13.29 | 2.73 | 1.45 |
| Sinapyl alcohol | C02325 | 110679389.7 | 108960509.6 | 6.56 | 87346981.49 | 87172754.32 | 12.72 | 1.27 | 0.34 |
| Myristicin | C10480 | 12680094.13 | 12544141.55 | 7.03 | 7969458.81 | 7697632.13 | 11.51 | 1.59 | 0.67 |
| Methoxamine | C07513 | 1662931.61 | 1743123.71 | 10.68 | 1020954.4 | 1030544.86 | 9.3 | 1.63 | 0.7 |
| Neocnidilide | C17002 | 44833257.75 | 36843625.11 | 29.1 | 15118686.82 | 14198635.41 | 15.58 | 2.97 | 1.57 |
| (-)-Bornesitol | C03659 | 516593972.4 | 518951518.6 | 4.75 | 820711905.3 | 817648315.6 | 2.84 | 0.63 | -0.67 |
| Metanephrine | C05588 | 14355260.82 | 14244138.09 | 4.13 | 11970581.5 | 12502723.7 | 7.44 | 1.2 | 0.26 |
| Syringic acid | C10833 | 109900841.2 | 110193979.7 | 2.06 | 65522896.33 | 64844054.27 | 5.5 | 1.68 | 0.75 |
| Dodecanoic acid | C02679 | 76259637.76 | 70751920.87 | 17.07 | 18026470.71 | 16433520.43 | 29.5 | 4.23 | 2.08 |
| Spermine | C00750 | 3521007 | 3495216.76 | 4.31 | 33322023.09 | 28376863.6 | 26.25 | 0.11 | -3.24 |
| (-)-alpha-Curcumene | C09649 | 39954637.51 | 39972837.2 | 1.07 | 5943609.4 | 4657586.18 | 52.44 | 6.72 | 2.75 |
| 4-Hydroxy-3-(3-methyl-2-butenyl)acetophenone | C10702 | 30212669.67 | 30121248.69 | 5.34 | 43599306.57 | 44105636.11 | 5.25 | 0.69 | -0.53 |
| Apiole | C10429 | 7954779.3 | 8023895.79 | 4.13 | 6884827.1 | 6836083.06 | 3.35 | 1.16 | 0.21 |
| Homocitric acid | C01251 | 19354639.26 | 19662961.27 | 23.02 | 46453564.11 | 46390731.47 | 3.28 | 0.42 | -1.26 |
| Sinapate | C00482 | 7373070.28 | 7450582 | 3.73 | 3773764.77 | 1965866.96 | 74.38 | 1.95 | 0.97 |
| Ibuprofen | C01588 | 14172351.66 | 16886129.27 | 42.44 | 35971831.89 | 34673384.06 | 9.86 | 0.39 | -1.34 |
| 3-[(1-Carboxyvinyl)oxy]benzoate | C20772 | 28984022.83 | 28728872.01 | 16.42 | 5962511.08 | 6037973.4 | 8.55 | 4.86 | 2.28 |
| L-Kynurenine | C00328 | 7208435.21 | 7964655.12 | 25.28 | 2820830.83 | 2621653.1 | 17.63 | 2.56 | 1.35 |
| N-Acetyldemethylphosphinothricin | C17949 | 26504434.93 | 26663411.57 | 47.49 | 98683766.88 | 99347450.96 | 2.36 | 0.27 | -1.9 |
| (+)-7-Isojasmonic acid | C16317 | 6266519.88 | 6255939.95 | 2.67 | 22872258.18 | 23304876.44 | 4.65 | 0.27 | -1.87 |
| 3-Methylindolepyruvate | C05644 | 22737815.38 | 22704363.25 | 2.5 | 17471407.61 | 17657316.1 | 2.8 | 1.3 | 0.38 |
| beta-Alanyl-L-lysine | C05341 | 25084433.81 | 20340593.71 | 51.47 | 112053742.8 | 93799153.18 | 40.71 | 0.22 | -2.16 |
| Glutethimide | C07489 | 13769770.53 | 13611983.3 | 8.62 | 10750076.35 | 10477200.94 | 12.8 | 1.28 | 0.36 |
| N-Acetylserotonin | C00978 | 57596175.7 | 65606320.84 | 38.18 | 118422785 | 113801735.2 | 10.78 | 0.49 | -1.04 |
| Cis-zeatin | C00371 | 7883828.93 | 7855975.32 | 2.63 | 7056817.32 | 7105164.32 | 1.65 | 1.12 | 0.16 |
| 2-trans,6-trans-Farnesal | C03461 | 33021557.86 | 32919268.66 | 4.98 | 17983149.43 | 18141643.76 | 7.09 | 1.84 | 0.88 |
| Diethyl phthalate | C14175 | 5206546.51 | 5325904.22 | 5.39 | 18766774.74 | 18504552.93 | 5.8 | 0.28 | -1.85 |
| Cerulenin | C12058 | 13716527.3 | 13466910.5 | 6.62 | 11418649.8 | 11132773.88 | 11.67 | 1.2 | 0.26 |
| Prephenate | C00254 | 26133937.91 | 26327296.11 | 3.7 | 12034222.61 | 12505222.5 | 9.21 | 2.17 | 1.12 |
| Myristoleic acid | C08322 | 13249392.13 | 12938022.48 | 6.74 | 16921197.84 | 16945369.98 | 3.96 | 0.78 | -0.35 |
| Benz[a]anthracene | C14317 | 3609854.04 | 2438006.22 | 88.01 | 12518055.65 | 10838233.9 | 71.74 | 0.29 | -1.79 |
| Resveratrol | C03582 | 58574723.03 | 58058089.84 | 17.17 | 33168589 | 34823168.09 | 14.08 | 1.77 | 0.82 |
| Propazine | C14312 | 4311202.2 | 2218978.19 | 84.99 | 1145190.92 | 1100870.8 | 28.09 | 3.76 | 1.91 |
| 6-Hydroxymelatonin | C05643 | 18737512.39 | 18142564.85 | 10.58 | 47687059.32 | 45050264.07 | 12.83 | 0.39 | -1.35 |
| Alantolactone | C09289 | 2669216.08 | 2659290.25 | 5.52 | 4110924.62 | 4136557.97 | 5.84 | 0.65 | -0.62 |
| Confertifolin | C09376 | 6190194.47 | 6326592.34 | 4.3 | 11385893.57 | 11661788.17 | 38.62 | 0.54 | -0.88 |
| N-Demethylindolmycin | C21443 | 5521628.56 | 5453912.06 | 2.97 | 4730013.55 | 4737707.68 | 1.73 | 1.17 | 0.22 |
| Alprenolol | D07156 | 93808532.99 | 93637876.32 | 10.51 | 42126940.53 | 44149595.63 | 36.89 | 2.23 | 1.15 |
| gamma-L-Glutamyl-L-cysteine | C00669 | 18706098.88 | 18728627.5 | 11.45 | 30662684.42 | 24924455.69 | 39.59 | 0.61 | -0.71 |
| Methaqualone | C07560 | 14865570.88 | 14310898.38 | 8.12 | 7002175.84 | 5808734 | 42.4 | 2.12 | 1.09 |
| Benzo[k]fluoranthene | C14321 | 11518142.88 | 11050347.03 | 8.68 | 1660823.89 | 1636082.03 | 6.99 | 6.94 | 2.79 |
| Nicotinamide riboside | C03150 | 79476584.29 | 82329131.5 | 14.83 | 37306067.7 | 41944371.57 | 37.28 | 2.13 | 1.09 |
| (S)-Pinocembrin | C09827 | 4739832.67 | 4891018.12 | 25.38 | 9267820.08 | 9288649.15 | 15.83 | 0.51 | -0.97 |
| Glycerophosphocholine | C00670 | 104636288 | 116440368.1 | 29.45 | 156791852.5 | 164797347.4 | 15.1 | 0.67 | -0.58 |
| Parthenin | C09523 | 24782468.36 | 25104167.29 | 5.79 | 13093114.67 | 13167489.21 | 5.52 | 1.89 | 0.92 |
| Linoleic acid | C01595 | 24830331 | 23878369.59 | 10.68 | 32775609.89 | 30121262.72 | 22.29 | 0.76 | -0.4 |
| Qing Hau Sau | C09538 | 4433627.49 | 3835994.92 | 52.99 | 1361522.94 | 1357813.86 | 12.65 | 3.26 | 1.7 |
| Mirtazapine | C07570 | 23822905.6 | 24061760.31 | 7.53 | 37294926.74 | 37886731.49 | 5.22 | 0.64 | -0.65 |
| Adenosine | C00212 | 3283724248 | 2809451065 | 32.09 | 8968354845 | 8664404846 | 14.97 | 0.37 | -1.45 |
| (S)-Coclaurine | C06161 | 237025443.1 | 239187789 | 5.45 | 135434046.6 | 137925722.9 | 6.71 | 1.75 | 0.81 |
| (R)-Coclaurine | C06349 | 5152785.61 | 5267502.02 | 5.92 | 146812.54 | 147975.15 | 17.69 | 35.1 | 5.13 |
| Estrone | C00468 | 16941146.31 | 17015159.46 | 7.97 | 44540807.29 | 42682358.53 | 14.72 | 0.38 | -1.39 |
| Norizalpinin | C10044 | 145803624.8 | 146014637.4 | 1.86 | 151542583.8 | 151875897.5 | 2.13 | 0.96 | -0.06 |
| Genistein | C06563 | 22404112.09 | 21893725.96 | 15.38 | 15627078.61 | 15766251.51 | 7.88 | 1.43 | 0.52 |
| All-trans-13,14-dihydroretinol | C15492 | 142004648.3 | 142146254.9 | 5.17 | 113795776.8 | 111165392.3 | 7.98 | 1.25 | 0.32 |
| Thienamycin | C06664 | 9057850.28 | 8965830.51 | 4.8 | 11655751.58 | 11503763.36 | 6.22 | 0.78 | -0.36 |
| Androsterone | C00523 | 213299705.6 | 243139730.2 | 33.59 | 89833697.35 | 59230928.07 | 57.27 | 2.37 | 1.25 |
| 5a-Androstane-3b,17b-diol | C12525 | 8627077.28 | 8594373.55 | 0.87 | 3737788.14 | 3719859.32 | 1.39 | 2.31 | 1.21 |
| Stearidonic acid | C16300 | 108065989.1 | 105879723.7 | 11.02 | 38016514.85 | 36358535.84 | 8.28 | 2.84 | 1.51 |
| Cyclopeptine | C20579 | 4372547.85 | 4248970.29 | 9.27 | 5993746.48 | 5771387.44 | 10.91 | 0.73 | -0.45 |
| 9-Riburonosyladenine | C11501 | 5411914.15 | 5421005.99 | 9.42 | 10397563.08 | 9072242.54 | 33.13 | 0.52 | -0.94 |
| 1-Methyladenosine | C02494 | 669191.54 | 590451.09 | 37.12 | 1611617.45 | 1692303.12 | 11.65 | 0.42 | -1.27 |
| (R,S)-Coclaurine | C06348 | 101823883.9 | 104354202.3 | 14.36 | 60789794.63 | 62878017.96 | 12.33 | 1.68 | 0.74 |
| 7a-Hydroxyandrost-4-ene-3,17-dione | C05296 | 35584184.53 | 34269030.12 | 42.54 | 14446883.59 | 9780919.81 | 100.53 | 2.46 | 1.3 |
| N1,N12-Diacetylspermine | C03413 | 7565913.84 | 7824489.33 | 10.55 | 27993833.8 | 27797674.15 | 11.41 | 0.27 | -1.89 |
| Myclobutanil | C18477 | 55512951.69 | 59303834.19 | 23.93 | 27059203.31 | 27262912.45 | 17.34 | 2.05 | 1.04 |
| Eriodictyol chalcone | C15525 | 52898934.26 | 53719285.53 | 4.79 | 26440969.53 | 26536920.99 | 6.84 | 2 | 1 |
| Eriodictyol | C05631 | 22101122.38 | 21884118.51 | 7.15 | 1031266.58 | 996321.4 | 16.18 | 21.43 | 4.42 |
| 8,11,14-Eicosatrienoic acid | C03242 | 32522021.09 | 33689199.09 | 8.01 | 17575705.49 | 19190141.86 | 22.49 | 1.85 | 0.89 |
| Aurin | C14213 | 50680617.87 | 50678167.47 | 4.7 | 41092083.34 | 41033421.45 | 4.17 | 1.23 | 0.3 |
| Epicatechin | C09727 | 1369103216 | 1372741582 | 7.15 | 1208280993 | 1213184168 | 4.21 | 1.13 | 0.18 |
| (+_-)-5-[(tert-Butylamino)-2'-hydroxypropoxy]-3,4-dihydro-1(2H)-naphthalenone | C04883 | 12840893.2 | 12073909.38 | 13.82 | 8808082.25 | 8655571.48 | 9.98 | 1.46 | 0.54 |
| Dihydrotestosterone | C03917 | 21295357.45 | 21548311.73 | 6.78 | 6879329.41 | 7004885.41 | 65.96 | 3.1 | 1.63 |
| 9(S)-HPOT | C16321 | 20003964.4 | 20973796.6 | 10.34 | 8947921.01 | 8902537.79 | 2.55 | 2.24 | 1.16 |
| Aspartame | C11045 | 1762990.85 | 1790594.58 | 26.02 | 1040901.05 | 1013261.35 | 29.24 | 1.69 | 0.76 |
| Nivalenol | C06080 | 114559099.4 | 115678319 | 40.14 | 193221599.7 | 179175220.8 | 17.07 | 0.59 | -0.75 |
| Prunasin | C00844 | 108485209.3 | 110252627.3 | 9.63 | 25189207.35 | 25242226.78 | 4.66 | 4.31 | 2.11 |
| Norethindrone | C05028 | 29732294.9 | 26788896.05 | 19.92 | 20015704.42 | 18489681.57 | 19.33 | 1.49 | 0.57 |
| Metoclopramide | C07868 | 14736002.94 | 14831685.13 | 5.55 | 11693080.75 | 11807285.46 | 7.66 | 1.26 | 0.33 |
| 3-Dehydrosphinganine | C02934 | 78207078.47 | 80662099.79 | 7 | 63597335.67 | 63949116.04 | 1.32 | 1.23 | 0.3 |
| (+)-6a-Hydroxymaackiain | C16230 | 2315260.08 | 2191101.71 | 15.38 | 1545574.87 | 1536919.33 | 7.54 | 1.5 | 0.58 |
| 5-Nitro-2-(3-phenylpropylamino)benzoic acid | C13705 | 11825812.62 | 11996870.76 | 5.83 | 8388538.2 | 7891587.04 | 19.3 | 1.41 | 0.5 |
| Kaempferide | C10098 | 1731979.52 | 1722726.1 | 11.01 | 2602865.58 | 2534187.66 | 5.62 | 0.67 | -0.59 |
| all-trans-Retinoic acid | C00777 | 10605562.34 | 10695323.63 | 2.43 | 8092902.86 | 8042995.19 | 4.15 | 1.31 | 0.39 |
| 2-Methoxy-17beta-estradiol | C05302 | 8374594.22 | 9050193.62 | 23.88 | 13181671.19 | 13480279.47 | 22.7 | 0.64 | -0.65 |
| Sphinganine | C00836 | 470845916.2 | 459748771.8 | 9.15 | 84532621.35 | 88724331.14 | 34.8 | 5.57 | 2.48 |
| Norethandrolone | D07127 | 58948498.08 | 58342396.5 | 5.95 | 51143169.24 | 51973174.8 | 6.91 | 1.15 | 0.2 |
| cis-Dihydroquercetin | C12316 | 204548921.4 | 200154365.7 | 8.67 | 60326870.22 | 57017480.75 | 14.02 | 3.39 | 1.76 |
| 11alpha,17beta-Dihydroxyandrost-4-en-3-one | C15306 | 24964055.53 | 24371139.62 | 11.8 | 516630.27 | 367113.69 | 59.13 | 48.32 | 5.59 |
| Glutathione | C00051 | 1849824561 | 2095026830 | 26.16 | 553749979.2 | 550814031.2 | 22.87 | 3.34 | 1.74 |
| Bisdemethoxycurcumin | C17743 | 79627064.15 | 69258306.11 | 33.92 | 50519130.71 | 51493143.12 | 20.25 | 1.58 | 0.66 |
| Alprazolam | C06817 | 12960964.98 | 10925428.89 | 30.28 | 20937890.82 | 20753056.3 | 12.41 | 0.62 | -0.69 |
| Warfarin | C01541 | 18779725.52 | 18628361.09 | 6.93 | 14630230.25 | 15319025.78 | 13.89 | 1.28 | 0.36 |
| beta-D-Galactosyl-(1->4)-L-rhamnose | C19758 | 6474451.97 | 6279623.89 | 7.66 | 4521686.76 | 4514981.66 | 1.6 | 1.43 | 0.52 |
| N-Acetyl-a-neuraminic acid | C19909 | 57138599.37 | 58554153.4 | 12.45 | 42769480.65 | 42867422.08 | 12.55 | 1.34 | 0.42 |
| Aflatoxin B1 | C06800 | 24230693.22 | 24711109.72 | 8.57 | 28193956.76 | 27934345.36 | 6.13 | 0.86 | -0.22 |
| Pergolide | C07425 | 7360691.93 | 7354615.7 | 4.7 | 5021129.77 | 5012211.95 | 4.43 | 1.47 | 0.55 |
| N(beta)-Epoxysuccinamoyl-DAP-Val | C20965 | 21117794.73 | 21419023.08 | 3.68 | 827394.61 | 817667.73 | 18.09 | 25.52 | 4.67 |
| Myricetin | C10107 | 24664656.33 | 24750636.61 | 19.49 | 12475546.22 | 11566903.59 | 17.24 | 1.98 | 0.98 |
| 5(S)-HpETE | C05356 | 51630723.98 | 57267452.29 | 25.49 | 86456639.18 | 77209874.68 | 26.24 | 0.6 | -0.74 |
| Norfloxacin | C06687 | 20188426.03 | 19523962.4 | 24.41 | 8696494.78 | 9025083.48 | 14.34 | 2.32 | 1.22 |
| Deoxy-5-methylcytidylate | C03495 | 35712727.11 | 36397856.2 | 6.77 | 14093972.33 | 13826464.7 | 8.98 | 2.53 | 1.34 |
| gamma-L-Glutamyl-L-cysteinyl-beta-alanine | C04544 | 322373585.6 | 330112248.4 | 10.74 | 223128876.1 | 227046904.9 | 11.83 | 1.44 | 0.53 |
| dTMP | C00364 | 6146433.74 | 5450581.65 | 22.82 | 682350.32 | 685405.79 | 9.6 | 9.01 | 3.17 |
| 2'-Deamino-2'-hydroxy-6'-dehydroparomamine | C20351 | 4483110.06 | 4872809.87 | 31.92 | 2057664.01 | 2062663.51 | 30.52 | 2.18 | 1.12 |
| Coniferin | C00761 | 81451662.35 | 81827678.96 | 20.25 | 63174939.98 | 62929070.06 | 4.33 | 1.29 | 0.37 |
| Citalopram | C07572 | 19350213.6 | 19221869.13 | 8.13 | 2087668.85 | 2073942.22 | 11.9 | 9.27 | 3.21 |
| Cellobiose | C00185 | 48951513.82 | 43874616.12 | 36.17 | 164443366.9 | 167547222.3 | 15.27 | 0.3 | -1.75 |
| (S)-Tetrahydropapaverine | C21631 | 3679129.19 | 3761504.94 | 7.24 | 5963831 | 6161539.95 | 9.62 | 0.62 | -0.7 |
| 2,4-Dioxotetrahydropyrimidine D-ribonucleotide | C04639 | 13103054.83 | 12849948.76 | 11.89 | 5042881.37 | 4993365.52 | 13.43 | 2.6 | 1.38 |
| Methotrimeprazine | C07192 | 23547919.01 | 25329883.55 | 36.87 | 36127825.56 | 36501595.75 | 5.09 | 0.65 | -0.62 |
| Aflatoxin M1 | C16756 | 3030638.57 | 3008585.37 | 5.77 | 3796546.19 | 3658616.51 | 14.11 | 0.8 | -0.33 |
| Corticosterone | C02140 | 22101585.96 | 22302369.66 | 6.83 | 25753767.09 | 25739457.12 | 3.07 | 0.86 | -0.22 |
| Aflatoxin G2 | C16754 | 26287881.43 | 27423203.73 | 12.59 | 17295515.49 | 17646872.86 | 10.48 | 1.52 | 0.6 |
| Malvidin | C08716 | 94527622.33 | 95367690.69 | 3.15 | 50381759.94 | 50400312.3 | 5.06 | 1.88 | 0.91 |
| Carnosol | C09069 | 5605619.85 | 5592364.2 | 2.87 | 6954066.21 | 6860053.49 | 6.49 | 0.81 | -0.31 |
| Cannabielsoin | C20218 | 6309855.87 | 6198168.77 | 12.29 | 8246938.56 | 8396876.43 | 8.01 | 0.77 | -0.39 |
| Norsanguinarine | C05191 | 192096843.8 | 182683977.6 | 21.56 | 20470617.12 | 17625293.52 | 27.9 | 9.38 | 3.23 |
| Gibberellin A4 | C11864 | 42106029.93 | 41931960.4 | 4.03 | 56260939.83 | 56501465.2 | 5.31 | 0.75 | -0.42 |
| 9S-hydroxy-11,15-dioxo-5Z,13E-prostadienoic acid | C04758 | 7714437.96 | 7807550.39 | 5.4 | 5851297.2 | 5970253.77 | 6.89 | 1.32 | 0.4 |
| Mitomycin | C06681 | 123412002.8 | 118341065.2 | 21.48 | 41199296.77 | 35015906.81 | 35.65 | 3 | 1.58 |
| Prostaglandin E2 | C00584 | 4505009.13 | 4648663.06 | 9.91 | 6356696.53 | 6383347.67 | 2.27 | 0.71 | -0.5 |
| Isopentenyl adenosine | C16427 | 5494041.12 | 5010341.84 | 22.25 | 3941805.25 | 4014049.41 | 6.82 | 1.39 | 0.48 |
| Prostaglandin E1 | C04741 | 866983781.9 | 853791739.5 | 19.08 | 607983343.4 | 637261748.1 | 13.94 | 1.43 | 0.51 |
| 11,12,15-THETA | C14782 | 46312550.25 | 47816305.76 | 13.32 | 62077988.28 | 62148989.43 | 4.83 | 0.75 | -0.42 |
| Psoralidin | C10523 | 97943167.31 | 103492541.2 | 13.42 | 71976121.47 | 73868698.58 | 13.83 | 1.36 | 0.44 |
| p-Coumaroyl quinic acid | C12208 | 2246308.96 | 2206733.15 | 7.87 | 427824.42 | 430279.62 | 67.73 | 5.25 | 2.39 |
| Pentamidine | C07420 | 33711972.19 | 33618886.96 | 8.57 | 14551628.35 | 14551260.2 | 19.43 | 2.32 | 1.21 |
| Cortisone | C00762 | 5366068.81 | 5463524.34 | 6 | 8333836.96 | 8179231.45 | 7.6 | 0.64 | -0.64 |
| Cyclic GMP | C00942 | 10930058.15 | 10936573.38 | 8.07 | 12167264.64 | 12174930.2 | 4.32 | 0.9 | -0.15 |
| dGMP | C00362 | 13263071.28 | 12977674.93 | 5.62 | 21824572.63 | 21625571.17 | 9.42 | 0.61 | -0.72 |
| (10S)-Juvenile hormone III diol phosphate | C16507 | 112034495.4 | 104471552.4 | 26.19 | 65188722.98 | 66000056.92 | 9.81 | 1.72 | 0.78 |
| 21-Deoxycortisol | C05497 | 8196288.89 | 8114956.72 | 10.71 | 19201707.81 | 19349332.49 | 4.72 | 0.43 | -1.23 |
| Tetrahydrocortisol | C05472 | 4403019.44 | 4450013.27 | 4.08 | 3655059.48 | 3738743.65 | 6.47 | 1.2 | 0.27 |
| 17alpha,21-Dihydroxypregnenolone | C05487 | 13470129.91 | 13567760.74 | 12.97 | 8091760.95 | 8174253.28 | 11.26 | 1.66 | 0.74 |
| 2-Hydroxy-6-pentadecylbenzoic acid | C10759 | 2535067.85 | 2583774.6 | 9.83 | 14214496.59 | 13528055.47 | 17.16 | 0.18 | -2.49 |
| 6-Keto-prostaglandin F1a | C05961 | 23987228.39 | 24192293.91 | 2.9 | 20277843.42 | 20247777.7 | 2.08 | 1.18 | 0.24 |
| 17-O-Acetylnorajmaline | C11809 | 2888371.77 | 2197411.58 | 59.35 | 1255031.05 | 1252824.14 | 7.59 | 2.3 | 1.2 |
| Chlorogenic acid | C00852 | 5739004.73 | 5759772.94 | 7 | 518290.49 | 524036.16 | 12.27 | 11.07 | 3.47 |
| S-Adenosylmethioninamine | C01137 | 173433.62 | 200893.51 | 40.72 | 1664795.57 | 1559729.3 | 16.81 | 0.1 | -3.26 |
| Laudanosine | C09558 | 45248391.92 | 46489478.47 | 7.69 | 13604757.05 | 11849332.4 | 28.32 | 3.33 | 1.73 |
| Niaprazine | D07333 | 133356532.3 | 132961214 | 6.97 | 266088699.4 | 272815461.4 | 8.44 | 0.5 | -1 |
| (+)-Pinoresinol | C05366 | 35575547.1 | 38133062.71 | 18.16 | 62694570.7 | 60761461.63 | 20.17 | 0.57 | -0.82 |
| Lariciresinol | C10646 | 9621895.72 | 9703155.27 | 2.01 | 6112283.65 | 6254685.67 | 12.03 | 1.57 | 0.65 |
| Cortisol | C00735 | 5973918.16 | 6368262.94 | 14.88 | 12193444.44 | 13496540.96 | 33.68 | 0.49 | -1.03 |
| 11b,21-Dihydroxy-3,20-oxo-5b-pregnan-18-al | C05473 | 10354941.54 | 9076556.62 | 30.07 | 34361838.49 | 33922282.28 | 56.9 | 0.3 | -1.73 |
| Pyridaben | C18614 | 17585101.23 | 14193073.18 | 44.75 | 8285410.76 | 8231434.74 | 30.05 | 2.12 | 1.09 |
| U50488 | C11796 | 7442836.12 | 7505643.29 | 5 | 3803542.1 | 3804834.27 | 6.13 | 1.96 | 0.97 |
| Bursehernin | C21183 | 37888848.36 | 21985865.84 | 80.93 | 139773861.1 | 137240965.3 | 8.93 | 0.27 | -1.88 |
| Tamoxifen | C07108 | 5586142.24 | 5533109.48 | 22.86 | 3089907.51 | 2659077.53 | 36.27 | 1.81 | 0.85 |
| Biocytin | C05552 | 15034046.36 | 16271932.4 | 22.94 | 9489159.91 | 9738717.94 | 30.09 | 1.58 | 0.66 |
| Hydroxyzine | C07045 | 12359558.94 | 12546189.93 | 12.57 | 7995761 | 7846277.68 | 4.84 | 1.55 | 0.63 |
| Carbenicillin | C06869 | 1029095.13 | 971293 | 25.35 | 12915980.77 | 12362892.75 | 38.88 | 0.08 | -3.65 |
| Mesoridazine | C07143 | 18677997.17 | 20649155.47 | 45.27 | 36018861.82 | 36076826.04 | 6.45 | 0.52 | -0.95 |
| Sufentanil | C08022 | 13129576.44 | 13026602.79 | 10.2 | 5512694.68 | 5692452.62 | 9.11 | 2.38 | 1.25 |
| Secologanin | C01852 | 5667088.6 | 5396165.63 | 54.15 | 9999436.76 | 9789456.57 | 9.21 | 0.57 | -0.82 |
| Ursodeoxycholic acid | C07880 | 8868522.88 | 9102617.57 | 7.19 | 2480838.58 | 1939208.4 | 40.23 | 3.57 | 1.84 |
| Aloesin | C08994 | 1438507.82 | 1075712.29 | 67.48 | 18448205.98 | 20169763.08 | 33.39 | 0.08 | -3.68 |
| Ergocalciferol | C05441 | 2234864.98 | 2226818.49 | 4.29 | 4725174.02 | 4496428.39 | 12.61 | 0.47 | -1.08 |
| Besonprodil | D03100 | 14579597.42 | 14102098.83 | 5.77 | 8211734.34 | 8196120.18 | 9.66 | 1.78 | 0.83 |
| Nobiletin | C10112 | 13708248.3 | 14507850.37 | 16.48 | 9145390.17 | 9123170.41 | 14.76 | 1.5 | 0.58 |
| alpha-Mangostin | C10080 | 17188790.98 | 22174672.14 | 48.53 | 5537887.2 | 4163181.82 | 62.24 | 3.1 | 1.63 |
| Paspalicine | C20553 | 8542230.62 | 8262873.01 | 15.5 | 12453669.6 | 12394070 | 11.08 | 0.69 | -0.54 |
| Kaempferol 3-O-beta-D-xyloside | C20727 | 53918527.78 | 54165314.59 | 4.54 | 19030550.85 | 18676089.21 | 15.88 | 2.83 | 1.5 |
| Trehalose 6-phosphate | C00689 | 252080531.6 | 249423946.5 | 6.22 | 51678048.04 | 54229957.31 | 32.81 | 4.88 | 2.29 |
| Vitexin | C01460 | 299808656.9 | 299263146.2 | 14.62 | 159662133.2 | 159908971.3 | 14.39 | 1.88 | 0.91 |
| 8-C-Glucosylnaringenin | C16492 | 437390604.8 | 412589387.7 | 14.7 | 216916967.9 | 225170498.9 | 23.92 | 2.02 | 1.01 |
| 3-Dehydroecdysone | C02513 | 21838483.15 | 21559399.45 | 3.93 | 12645553.05 | 12656097.31 | 10.26 | 1.73 | 0.79 |
| Testosterone glucuronide | C11134 | 21435768.53 | 27445304.5 | 51.03 | 76399994.32 | 70923334.33 | 11.74 | 0.28 | -1.83 |
| Estrone glucuronide | C11133 | 20863831.23 | 20183005.88 | 12.71 | 10677666.72 | 10165793.35 | 17.74 | 1.95 | 0.97 |
| Etiocholanolone glucuronide | C11136 | 6624571.11 | 6298151.19 | 9.39 | 5685714.17 | 5598456.49 | 3.32 | 1.17 | 0.22 |
| Isoquercitrin | C05623 | 4073936.2 | 4983148.7 | 48.65 | 1050535.97 | 674317.39 | 96.17 | 3.88 | 1.96 |
| Copal-8-ol diphosphate | C20270 | 23019014.95 | 24004786.38 | 16.57 | 3911051.95 | 2444257.52 | 88.13 | 5.89 | 2.56 |
| Withaferin A | C08841 | 3575334.81 | 3620111.18 | 7.56 | 2425378.35 | 2438502.2 | 21.38 | 1.47 | 0.56 |
| 2'-Dehydrokanamycin A | C20509 | 14763648.94 | 12696396.03 | 26.28 | 6399503.51 | 6135699.32 | 16.43 | 2.31 | 1.21 |
| Raffinose | C00492 | 11607209.38 | 12341787.25 | 19.47 | 3339110.73 | 3146027.91 | 39.61 | 3.48 | 1.8 |
| Limonoate | C01593 | 598419098.5 | 615420488.3 | 13.23 | 783163107.7 | 790971411.6 | 8.61 | 0.76 | -0.39 |
| Rhodoxanthin | C08610 | 118282110.5 | 96304056.33 | 64.02 | 32450762.02 | 23641254.56 | 69.47 | 3.64 | 1.87 |
| N-Acetyl-O-demethylpuromycin-5'-phosphate | C07030 | 2921907.86 | 2468100.94 | 37.48 | 5212174.97 | 4971644.31 | 23.01 | 0.56 | -0.83 |
| Cyanidin 3-O-(6-O-p-coumaroyl)glucoside | C12095 | 39791675.79 | 37331581.1 | 21.69 | 19772457.54 | 18152722.76 | 37.81 | 2.01 | 1.01 |
| Astaxanthin | C08580 | 4464563.08 | 4478976.75 | 8.34 | 5398479.88 | 5588497.88 | 12.45 | 0.83 | -0.27 |
| Glutathione amide disulfide | C19690 | 16296352.58 | 14821190.14 | 39.17 | 7130394.69 | 5799219.92 | 68.94 | 2.29 | 1.19 |
| Delphinidin 3-rutinoside | C16315 | 99630889.96 | 91763475.4 | 14.27 | 50255397.62 | 54620297.53 | 42.73 | 1.98 | 0.99 |
| Cyanidin 3-O-(6-O-p-coumaroyl)glucoside-5-O-glucoside | C12096 | 27148160.88 | 26547513.45 | 19.42 | 36120321.01 | 36847726.74 | 15.72 | 0.75 | -0.41 |
| PC(18_3(6Z,9Z,12Z)_18_3(6Z,9Z,12Z)) | C00157 | 19316799.46 | 16951312.97 | 25.47 | 4499732.63 | 5152332.95 | 37.38 | 4.29 | 2.1 |
| R-Methylmalonyl-CoA | C01213 | 11096703.07 | 10666883.95 | 36.19 | 20818800.16 | 22357441.13 | 19.66 | 0.53 | -0.91 |
| 5-Aminopentanoic acid | C00431 | 6062945.81 | 6198609.25 | 6.39 | 3081040.36 | 2917808.22 | 16.68 | 1.97 | 0.98 |
| Phenylacetaldehyde | C00601 | 1291875.75 | 1299225.66 | 3.2 | 1065862 | 1059554.26 | 3.1 | 1.21 | 0.28 |
| 3-Methylthiopropionic acid | C08276 | 6988903.69 | 6938441.08 | 3.1 | 6395767.41 | 6459081.11 | 3.79 | 1.09 | 0.13 |
| Pyrrolidonecarboxylic acid | C02237 | 7581355.86 | 7275255.05 | 10.49 | 6213472.48 | 6101926.43 | 8.27 | 1.22 | 0.29 |
| Glutaric acid | C00489 | 31771363.19 | 31715992.52 | 1.8 | 27661467.63 | 27724238.64 | 2.51 | 1.15 | 0.2 |
| L-Aspartic acid | C00049 | 33183927.38 | 32724586.03 | 3.98 | 18345058.32 | 18596360.7 | 5.73 | 1.81 | 0.86 |
| Mandelonitrile | C00561 | 10578786.78 | 10585078.34 | 1.27 | 13003597.94 | 13026831.35 | 1.71 | 0.81 | -0.3 |
| Adenine | C00147 | 99607759.91 | 99698226.04 | 1.24 | 123499650.7 | 123692138.6 | 1.45 | 0.81 | -0.31 |
| D-Xylonate | C00502 | 40907982.49 | 31754668.2 | 41.77 | 100939685.7 | 101507344.2 | 3.93 | 0.41 | -1.3 |
| trans-Cinnamate | C00423 | 20506419.58 | 17968575.62 | 26.74 | 35448675.25 | 30187683.18 | 49.78 | 0.58 | -0.79 |
| Citramalic acid | C00815 | 51750437.13 | 51182211.44 | 3.09 | 56111431.46 | 55697595.08 | 4.3 | 0.92 | -0.12 |
| D-Xylose | C00181 | 319033802.6 | 313697634.1 | 5.82 | 428173383.6 | 428171479.3 | 2.53 | 0.75 | -0.42 |
| (Z)-4-Hydroxy-6-dodecenoic acid lactone | C03107 | 2851193.38 | 2812702.22 | 8.83 | 782025.54 | 774269.91 | 7.57 | 3.65 | 1.87 |
| Pyrophosphate | C00013 | 4418321.82 | 4826554.07 | 27.78 | 7502507.72 | 7558496.46 | 2.32 | 0.59 | -0.76 |
| Fructose-1P | C10906 | 61980517.75 | 61692690.79 | 1.9 | 24105722.4 | 23977020.49 | 4.04 | 2.57 | 1.36 |
| m-Coumaric acid | C12621 | 141573903.1 | 139067910.4 | 8.09 | 71536887.11 | 70625868.48 | 3.18 | 1.98 | 0.98 |
| L-Fucose | C00507 | 13578693.95 | 13718475.72 | 3.61 | 14653885.66 | 14653683.38 | 2.57 | 0.93 | -0.11 |
| trans-2-Hydroxycinnamate | C01772 | 21376649.63 | 21544571.97 | 1.97 | 7819831.34 | 7738870.87 | 2.8 | 2.73 | 1.45 |
| Vanylglycol | C05594 | 21918493.11 | 21023400.34 | 9.84 | 7323947.77 | 7232552.23 | 13.38 | 2.99 | 1.58 |
| Phenyllactate | C05607 | 4796665.25 | 4953690.16 | 12.96 | 3518412.44 | 3505262.96 | 6.42 | 1.36 | 0.45 |
| Tropate | C01456 | 13497109.93 | 13680381.05 | 25.61 | 8335778.01 | 7413318.62 | 35.14 | 1.62 | 0.7 |
| Norepinephrine | C00547 | 11086155.93 | 11234170.52 | 6.57 | 8634379.35 | 8644425.48 | 11.21 | 1.28 | 0.36 |
| Beta-Glycerophosphoric acid | C02979 | 1765819.33 | 1840860.67 | 19.25 | 4637510.71 | 4567725.12 | 5.79 | 0.38 | -1.39 |
| Dehydroascorbate | C05422 | 804224425 | 803180691.2 | 1.3 | 240862104.3 | 241019206.5 | 4.78 | 3.34 | 1.74 |
| Guanidinosuccinic acid | C03139 | 1235689924 | 1013046221 | 30.19 | 676802145.6 | 645668061.1 | 13.68 | 1.83 | 0.87 |
| Gluconolactone | C00198 | 123527456.4 | 155498520.7 | 49.63 | 35634174.89 | 17081237.91 | 85.55 | 3.47 | 1.79 |
| Gluconic acid | C00257 | 6270896.68 | 5702257.48 | 26.5 | 3678047.66 | 3474811.77 | 12.39 | 1.7 | 0.77 |
| D-(+)-Glucose | C00293 | 23723445.57 | 25327878.25 | 39.64 | 37906176.79 | 37312768.89 | 14.4 | 0.63 | -0.68 |
| D-Galactose | C00124 | 11352899.87 | 9448691.49 | 44.91 | 20807923.11 | 17142853.8 | 31.62 | 0.55 | -0.87 |
| 2-Hydroxyisophthalic acid | C14097 | 4069440.5 | 3983793.88 | 9.51 | 5783348.25 | 5595436.74 | 11.98 | 0.7 | -0.51 |
| Azelaic acid | C08261 | 15961279.22 | 16293491.86 | 4.77 | 10690437.06 | 10519674.88 | 5.05 | 1.49 | 0.58 |
| Quinate | C00296 | 72573521717 | 80601059163 | 28.93 | 34554399589 | 32645102997 | 43.15 | 2.1 | 1.07 |
| D-Glucuronic Acid | C00191 | 5975777.11 | 6047900.62 | 5.97 | 3024772.86 | 3018339.82 | 54.93 | 1.98 | 0.98 |
| trans-Ferulic acid | C01494 | 6532580.34 | 6421443.01 | 9.04 | 2945387.72 | 3279107.57 | 43.91 | 2.22 | 1.15 |
| Xanthoxylin | C10726 | 8835417.11 | 8841397.79 | 6.96 | 11120781.94 | 11293598.63 | 8.18 | 0.79 | -0.33 |
| Vanillylmandelic acid | C05584 | 25250026.75 | 25298332.56 | 5.06 | 31633466.2 | 31598900.97 | 6.8 | 0.8 | -0.33 |
| L-Tryptophan | C00078 | 325474697.2 | 323833688.9 | 1.56 | 78125041.65 | 78038927.52 | 0.89 | 4.17 | 2.06 |
| Xanthurenic acid | C02470 | 1549228.11 | 1555465.54 | 20.09 | 516541.07 | 273068.11 | 80.16 | 3 | 1.58 |
| N-Acetyl-L-phenylalanine | C03519 | 110123234.4 | 111146865.9 | 71.25 | 237905730.8 | 234048184.2 | 12.27 | 0.46 | -1.11 |
| (-)-Jasmonic acid | C08491 | 4504958.59 | 4539502.52 | 4.72 | 2994622.7 | 2991653.65 | 5.82 | 1.5 | 0.59 |
| Galactaric acid | C00879 | 12731500.19 | 12890629.34 | 10.9 | 7015645.52 | 6770364.37 | 56.16 | 1.81 | 0.86 |
| N-Acetyl-D-glucosamine | C00140 | 6682557.56 | 6998027.6 | 13.52 | 12846157.1 | 12777378.5 | 7.25 | 0.52 | -0.94 |
| 6-Acetyl-D-glucose | C02655 | 8591541.27 | 8771065.68 | 4.88 | 2911800.69 | 2875099.42 | 4.12 | 2.95 | 1.56 |
| Methyl jasmonate | C11512 | 102376014.1 | 104145238.6 | 5.84 | 57329941.67 | 57539296.38 | 3.15 | 1.79 | 0.84 |
| Citrinin | C16765 | 15693573.28 | 16079489.21 | 7.94 | 12150672.72 | 12017326.43 | 7.82 | 1.29 | 0.37 |
| Galactosylglycerol | C05401 | 156104669.8 | 156065674.2 | 3.67 | 94426530.78 | 93493337.86 | 3.68 | 1.65 | 0.73 |
| Shikimate 3-phosphate | C03175 | 3382098.72 | 3462521.36 | 12.41 | 6273947.37 | 5954624.55 | 14.67 | 0.54 | -0.89 |
| Galactose 1-phosphate | C00103 | 75691346.54 | 78286322.25 | 19.89 | 46956351.3 | 50585416.87 | 13.91 | 1.61 | 0.69 |
| (S)-Abscisic acid | C06082 | 58260433.9 | 66199627.5 | 38.54 | 101617657.4 | 104818330.4 | 10.71 | 0.57 | -0.8 |
| Phloretin | C00774 | 4684384.16 | 4672813.08 | 1.66 | 3816720.58 | 3909830.17 | 6.96 | 1.23 | 0.3 |
| Bovinic acid | C04056 | 268682075.5 | 278765806.4 | 13.94 | 197601799.6 | 189664482.1 | 14.43 | 1.36 | 0.44 |
| Hexadecanedioate | C19615 | 820384184.5 | 819032876.5 | 1.96 | 652125018.5 | 658295032.2 | 3.91 | 1.26 | 0.33 |
| 13S-hydroxyoctadecadienoic acid | C14762 | 6339397.7 | 6427642.8 | 4.17 | 8054487.62 | 8204618.98 | 5.51 | 0.79 | -0.35 |
| 12,13-DHOME | C14829 | 12109563.77 | 12209459.05 | 21.27 | 6767614.01 | 6424541.88 | 18.66 | 1.79 | 0.84 |
| 4-(beta-D-Glucosyloxy)benzoate | C03993 | 32653942.3 | 34464960.51 | 9.66 | 20843612.48 | 21036420.75 | 5.08 | 1.57 | 0.65 |
| Diosmetin | C10038 | 6617340.91 | 6543326.21 | 5.77 | 4082371.93 | 4064891.7 | 6.19 | 1.62 | 0.7 |
| 2-Methoxyestrone | C05299 | 462305.48 | 454821.66 | 6.59 | 1026035.2 | 1012432.95 | 10.09 | 0.45 | -1.15 |
| EPA (d5) | C06428 | 37036301.06 | 36211349.87 | 6.67 | 46410175.28 | 44873205.12 | 7.66 | 0.8 | -0.33 |
| Pentahydroxyflavanone | C05911 | 32359108.37 | 32270046.36 | 4.24 | 28873353.36 | 28838502.01 | 5.32 | 1.12 | 0.16 |
| (-)-Epigallocatechin | C12136 | 401576634 | 396302663.8 | 14.1 | 64135028.38 | 55726471.16 | 35.03 | 6.26 | 2.65 |
| Arachidic acid | C06425 | 13594256.43 | 13875418.28 | 15.57 | 29985640.49 | 29969138.64 | 4.49 | 0.45 | -1.14 |
| 9(S)-HPODE | C14827 | 13727863.01 | 13435649.3 | 4.59 | 16431692.61 | 16562387.22 | 2.19 | 0.84 | -0.26 |
| Isorhamnetin | C10084 | 8332217.68 | 7928840.21 | 61.83 | 65016913.44 | 84404154.93 | 51.87 | 0.13 | -2.96 |
| 15-Deoxy-d-12,14-PGJ2 | C14717 | 18735171.13 | 18053437.68 | 17.37 | 142036545.7 | 160086922.8 | 34.51 | 0.13 | -2.92 |
| 12-KETE | C14807 | 505575613.3 | 499923210.9 | 2.76 | 407467027.7 | 421279979.3 | 8.55 | 1.24 | 0.31 |
| Dihydromyricetin | C02906 | 33484394.22 | 20575511.23 | 117.32 | 404103382.8 | 364031404.1 | 23.87 | 0.08 | -3.59 |
| 20-HETE | C14748 | 43814197.96 | 39070955.32 | 27.32 | 97308635.17 | 97825507.26 | 32.24 | 0.45 | -1.15 |
| Melibiitol | C05399 | 40976640.43 | 39882655.72 | 7.03 | 30588506.87 | 30303224.23 | 9.68 | 1.34 | 0.42 |
| trans-beta-D-Glucosyl-2-hydroxycinnamate | C05158 | 17132381.75 | 17522250.6 | 10.78 | 14520505.89 | 14220424.91 | 8.36 | 1.18 | 0.24 |
| 1-O-Vanilloyl-beta-D-glucose | C20470 | 57803410.83 | 56505875.75 | 13.03 | 347551863 | 338607009.5 | 6.84 | 0.17 | -2.59 |
| 1-O-Galloyl-beta-D-glucose | C01158 | 14757455.86 | 16011201.69 | 21.76 | 5648094.67 | 5861764.05 | 14.35 | 2.61 | 1.39 |
| Prostaglandin A2 | C05953 | 64630125.94 | 62633680.07 | 10.2 | 78895732.01 | 79552930.72 | 2.24 | 0.82 | -0.29 |
| Delta-12-Prostaglandin J2 | C05958 | 2334239359 | 2395526035 | 7.03 | 2844511877 | 2776426506 | 7.99 | 0.82 | -0.29 |
| Prostaglandin B2 | C05954 | 51917313.45 | 53509922.13 | 8.45 | 33593096.49 | 32879000 | 45.79 | 1.55 | 0.63 |
| 12-Keto-tetrahydro-leukotriene B4 | C02165 | 20305609.92 | 13966536.58 | 83.32 | 123703961.9 | 105900702.7 | 33.98 | 0.16 | -2.61 |
| (5Z,9E,14Z)-(8xi,11R,12S)-11,12-epoxy-8-hydroxyicosa-5,9,14-trienoic Acid | C04849 | 12918153.26 | 13485658.47 | 18.97 | 129651762.2 | 129284425.5 | 2.64 | 0.1 | -3.33 |
| Dattelic acid | C10434 | 26875553.76 | 27749609.53 | 10.38 | 20043429.05 | 20614263.13 | 13.77 | 1.34 | 0.42 |
| Erucic acid | C08316 | 20561397.6 | 20821585.63 | 17.44 | 26999050.96 | 26803379.19 | 8 | 0.76 | -0.39 |
| 3'-Ketolactose | C05403 | 15593659.3 | 15447400.06 | 7.63 | 21209363.6 | 21246480.49 | 3.03 | 0.74 | -0.44 |
| Fructose 1,6-bisphosphate | C00354 | 90107273.52 | 90756385.71 | 5.18 | 265077203.5 | 263448351.3 | 6.07 | 0.34 | -1.56 |
| Trehalose | C01083 | 9577983.74 | 9499472.63 | 8.69 | 3843261.2 | 3850254.31 | 44.38 | 2.49 | 1.32 |
| Melibiose | C05400 | 30731147.37 | 29094548.81 | 17.89 | 21695997.56 | 21713921.97 | 21.22 | 1.42 | 0.5 |
| Eupatilin | C10040 | 25212031.74 | 27139944.1 | 50.77 | 56906590.54 | 55684974.29 | 26.35 | 0.44 | -1.17 |
| 6-Ketoprostaglandin E1 | C05962 | 8795586.47 | 8485239.05 | 20.73 | 21226653.51 | 20801494.88 | 15.5 | 0.41 | -1.27 |
| Estradiol-17beta 3-sulfate | C08357 | 1550251.07 | 1508593.88 | 44.71 | 484100.48 | 486032.23 | 2.74 | 3.2 | 1.68 |
| (13E)-11a-Hydroxy-9,15-dioxoprost-13-enoic acid | C04654 | 1380628.11 | 1405263.64 | 11.87 | 10768139.48 | 10691536.03 | 5.39 | 0.13 | -2.96 |
| Neochlorogenic acid | C17147 | 51290610.31 | 53046156.53 | 6.12 | 29029568.93 | 31374433.97 | 15.02 | 1.77 | 0.82 |
| Nervonic acid | C08323 | 7156984.79 | 5394672.3 | 46.01 | 38804657.52 | 41918922.51 | 31.53 | 0.18 | -2.44 |
| Curcumin | C10443 | 20622163.07 | 20460745.22 | 5.56 | 27976406.16 | 28293535.68 | 3.34 | 0.74 | -0.44 |
| (-)-Wikstromol | C10725 | 135644216.2 | 153150275.3 | 39.64 | 8277189.96 | 7646026.87 | 27.85 | 16.39 | 4.03 |
| Acetyl-maltose | C02130 | 1055226.17 | 1093948.9 | 13.3 | 1795074.63 | 1790401.62 | 12.26 | 0.59 | -0.77 |
| trans-Piceid | C10275 | 22570546.85 | 23751335.07 | 27.23 | 9528003.59 | 9634839.17 | 7.83 | 2.37 | 1.24 |
| Linustatin | C08333 | 26864605.45 | 27210133.41 | 7.42 | 11753718.59 | 11763482.15 | 6.21 | 2.29 | 1.19 |
| Lamiide | C11644 | 66842711.36 | 72267962.97 | 23.38 | 20728873.4 | 21117848.13 | 10.33 | 3.22 | 1.69 |
| Cosmosiin | C04608 | 45681025.13 | 44905577.12 | 12.66 | 9774397.82 | 9559069.36 | 65.46 | 4.67 | 2.22 |
| Phlorizin | C01604 | 466771672.7 | 443087860.4 | 16.62 | 344130128.2 | 342311916.9 | 22.49 | 1.36 | 0.44 |
| Irisxanthone | C10067 | 19135846.25 | 19205530.37 | 36.67 | 7844651.96 | 8328186.94 | 32.61 | 2.44 | 1.29 |
| Epigallocatechin gallate | C09731 | 3507940.26 | 3450432.42 | 8.13 | 2226789.24 | 2190964.12 | 5.99 | 1.58 | 0.66 |
| Lusitanicoside | C10474 | 70395812.88 | 69031628.4 | 6.18 | 98249490.79 | 100368524 | 8.5 | 0.72 | -0.48 |
| 6-Methoxyluteolin 7-rhamnoside | C10104 | 34029065.28 | 35686944.56 | 17.94 | 20863960.93 | 18863456.64 | 20.12 | 1.63 | 0.71 |
| Myricitrin | C10108 | 8404582.49 | 6446125.29 | 71.14 | 28970585.58 | 26847197.96 | 61.96 | 0.29 | -1.79 |
| Delphinidin 3-glucoside | C12138 | 236591213.6 | 161447214 | 51.27 | 75539657.39 | 74682128.71 | 6.02 | 3.13 | 1.65 |
| 8-Epiiridodial glucoside tetraacetate | C11658 | 29079323.09 | 27601496.97 | 18.83 | 13627049.61 | 14206527.09 | 22.65 | 2.13 | 1.09 |
| Iridodial glucoside tetraacetate | C11657 | 3576105 | 3422274.71 | 41.6 | 1527651.65 | 1589113.04 | 9.84 | 2.34 | 1.23 |
| Melezitose | C08243 | 6053946.31 | 5913793.74 | 28.7 | 11603855.13 | 11692452.42 | 14.07 | 0.52 | -0.94 |
| Isochlorogenic acid b | C10468 | 6884613.06 | 7268064.95 | 30.26 | 16232695.63 | 17771120.01 | 21.82 | 0.42 | -1.24 |
| 10-Deoxygeniposide tetraacetate | C11664 | 507547546.7 | 522963751.1 | 12.19 | 363010931 | 364522614.9 | 30.9 | 1.4 | 0.48 |
| Delphinidin 3-(6-p-coumaroyl)glucoside | C16370 | 3987417.16 | 3504716.61 | 26.3 | 7888995.21 | 7775016.39 | 24.78 | 0.51 | -0.98 |
| Kaempferol 3-O-rhamnoside-7-O-glucoside | C21854 | 3910178.76 | 3770401.79 | 16.15 | 1224214.91 | 979998.67 | 58.73 | 3.19 | 1.68 |
| Neomycin | C01737 | 1720501.75 | 2149265.52 | 55.08 | 4693937.49 | 4623566.01 | 24.23 | 0.37 | -1.45 |
| Eriocitrin | C09732 | 26549273.11 | 27016665.64 | 21.88 | 8594449.13 | 8990724.51 | 9.98 | 3.09 | 1.63 |
| Quercetin 3-O-beta-D-glucosyl-(1->2)-beta-D-glucoside | C12667 | 3438619.45 | 3365422.97 | 10.78 | 1015363.19 | 787511.16 | 60.94 | 3.39 | 1.76 |
| Delphin | C16312 | 4131834.38 | 4107111.25 | 12.27 | 1241823.83 | 1378899.62 | 37.94 | 3.33 | 1.73 |
| Lacto-N-tetraose | C06371 | 2952384.08 | 2985189.42 | 20.86 | 13789781.94 | 13827578.21 | 12.38 | 0.21 | -2.22 |
