# Supplementary material for: Response mechanism of carbon metabolism of Pinus massoniana to gradient high temperature and drought stress
Source: BMC Genomics. 2024 Feb 12;25:166. doi: 10.1186/s12864-024-10054-2 (PMC10860282; doi:10.1186/s12864-024-10054-2)
Supplement: Supplementary file 13 — Additional file 13. [file 12864_2024_10054_MOESM13_ESM.docx]

Table S16 KEGG enrichment analysis of T30CK vs T30Z differential metabolites.

| **name** | **KEGG** | **T30Z_Mean** | **T30Z_Median** | **T30Z_RSD** | **T30CK_Mean** | **T30CK_Median** | **T30CK_RSD** | **FC** | **log2FC** |
| --- | --- | --- | --- | --- | --- | --- | --- | --- | --- |
| Triethylamine | C14691 | 118280363.4 | 120444479.8 | 8.8 | 161959207.1 | 161065233.6 | 11.69 | 0.73 | -0.45 |
| Isovaleric acid | C08262 | 7232474.8 | 7097889.59 | 10.79 | 18756401.32 | 18862636.62 | 9.6 | 0.39 | -1.37 |
| 3-Methylthiopropanamine | C03354 | 5697399.03 | 5785548.28 | 6.68 | 8834729.47 | 9739277.95 | 18.21 | 0.64 | -0.63 |
| 2-Phenylethanol | C05853 | 197895371.3 | 200094414.3 | 5.82 | 388438259.3 | 386947817.2 | 1.89 | 0.51 | -0.97 |
| Benzaldehyde | C00261 | 9950987.53 | 10283230.69 | 8.93 | 61026732.57 | 61781393.2 | 4.02 | 0.16 | -2.62 |
| m-Cresol | C01467 | 58671362.21 | 55040249.11 | 18.61 | 27411271.04 | 27027537.38 | 7.02 | 2.14 | 1.1 |
| Cytosine | C00380 | 482289703.1 | 469927459.3 | 7.94 | 408541717.8 | 404406379.8 | 3.84 | 1.18 | 0.24 |
| 5-Methyl-2-furancarboxaldehyde | C11115 | 107072284.2 | 104853964.9 | 9.7 | 220088647.6 | 219803267.1 | 2.35 | 0.49 | -1.04 |
| Histamine | C00388 | 6870917.96 | 6967811.18 | 16.62 | 5313652.32 | 5328151.99 | 10.19 | 1.29 | 0.37 |
| Imidazole-4-acetaldehyde | C05130 | 7708235.68 | 7755222.66 | 32.99 | 4030803.11 | 3967448.4 | 23.74 | 1.91 | 0.94 |
| Uracil | C00106 | 135824613.5 | 135109643.3 | 5.24 | 240909169.5 | 243142227.4 | 3.92 | 0.56 | -0.83 |
| Creatinine | C00791 | 109314409.7 | 107839373.4 | 4.01 | 206699905.7 | 206055343.3 | 0.98 | 0.53 | -0.92 |
| Deoxyribose | C01801 | 21038202.36 | 20885151.3 | 7.08 | 15996894.06 | 16134234.26 | 5.03 | 1.32 | 0.4 |
| 5-Hydroxypentanoic acid | C02804 | 1348296.6 | 1425104.36 | 31.97 | 24341893.85 | 24097559.89 | 4.36 | 0.06 | -4.17 |
| L-Allothreonine | C05519 | 7313551.01 | 7404756.98 | 3.93 | 3532779.38 | 3531251.82 | 0.76 | 2.07 | 1.05 |
| Tyrosol | C06044 | 81650411.98 | 79728159.03 | 7.15 | 34128706.12 | 33625565.04 | 6.05 | 2.39 | 1.26 |
| N,N-Dimethylaniline | C02846 | 48103614.93 | 48434987.04 | 10.84 | 27733711.39 | 24206820.57 | 31.76 | 1.73 | 0.79 |
| Phenylethylamine | C05332 | 24148010.41 | 24140718.48 | 4.64 | 382397539 | 382798983.2 | 2.63 | 0.06 | -3.99 |
| Niacinamide | C00153 | 3817315.62 | 3795120.15 | 7.29 | 62963971.22 | 62627954.92 | 3.11 | 0.06 | -4.04 |
| 4-Hydroxybenzaldehyde | C00633 | 8006986.67 | 8014678.07 | 3.1 | 34129531.42 | 29893485.66 | 30.23 | 0.23 | -2.09 |
| Erythritol | C00503 | 35890208.79 | 35838031.78 | 0.56 | 13832945.43 | 13824895.11 | 1.89 | 2.59 | 1.38 |
| Taurine | C00245 | 39952292.89 | 48492929.67 | 47.63 | 114961183.9 | 127974942.8 | 37.76 | 0.35 | -1.52 |
| Triacetate lactone | C02752 | 408230787.5 | 493178653.5 | 37.37 | 156124124.1 | 148102718.6 | 18.29 | 2.61 | 1.39 |
| Phloroglucinol | C02183 | 55144994.08 | 54441313.9 | 4.43 | 37129177.15 | 36872645.26 | 2.74 | 1.49 | 0.57 |
| Dihydrothymine | C00906 | 16333799.99 | 16440650.14 | 3.9 | 12002089.29 | 11822195.91 | 4.97 | 1.36 | 0.44 |
| Quinoline | C06413 | 1123520376 | 1123724982 | 10.07 | 283271159.5 | 284761764.5 | 5.06 | 3.97 | 1.99 |
| 1,1-Dimethylbiguanide | C07151 | 59428882.99 | 58077654.47 | 8.61 | 169937567.9 | 164417983.5 | 8.87 | 0.35 | -1.52 |
| Pipecolic acid | C00408 | 1086021466 | 1100475090 | 3.52 | 580686536.5 | 650030621.8 | 25.74 | 1.87 | 0.9 |
| L-Isoleucine | C00407 | 778208391 | 787922664.3 | 4.05 | 24215299.91 | 24790779.07 | 15.94 | 32.14 | 5.01 |
| 3-Methylindole | C08313 | 3522148.87 | 2524594.95 | 69.66 | 24336736.31 | 11213329.42 | 87.62 | 0.14 | -2.79 |
| L-Ribulose | C00310 | 31164289.01 | 30968667.81 | 4.12 | 23603016.07 | 23578055.38 | 2.64 | 1.32 | 0.4 |
| 1,3-Dihydro-(2H)-indol-2-one | C12312 | 6844840.8 | 6762173.48 | 9.75 | 2924963.61 | 3133614.1 | 31.07 | 2.34 | 1.23 |
| L-Asparagine | C00152 | 9622144.1 | 9350808.57 | 10.22 | 7894960.65 | 7846221.22 | 10.82 | 1.22 | 0.29 |
| Isochavicol | C20464 | 83482354.98 | 82467994.8 | 14.36 | 129548527.7 | 128443359.4 | 4.5 | 0.64 | -0.63 |
| D-Xylitol | C00379 | 14582990.99 | 13199773.11 | 25.18 | 10680397.62 | 10686785.5 | 9.5 | 1.37 | 0.45 |
| 2-Phenylacetamide | C02505 | 4626644.99 | 4568649.56 | 7.34 | 5635959.8 | 5567782.43 | 4.12 | 0.82 | -0.28 |
| Perillyl alcohol | C02452 | 198544239.2 | 194585152.5 | 6.5 | 117611067.2 | 117194488.1 | 2.14 | 1.69 | 0.76 |
| Chavicol | C16930 | 3421052.07 | 3379933.47 | 7.11 | 8906337.09 | 9157098.06 | 7.59 | 0.38 | -1.38 |
| (-)-3-Isothujone | C09906 | 12129331.23 | 12066575.42 | 2.91 | 8163666.7 | 8960740.2 | 17.55 | 1.49 | 0.57 |
| Phenelzine | C07430 | 6946603.71 | 6959453.67 | 3.97 | 17620190.97 | 17844853.71 | 2.86 | 0.39 | -1.34 |
| Gamma-terpinene | C09900 | 31113312.35 | 31288109.74 | 3.73 | 22036175.96 | 22153812.39 | 2 | 1.41 | 0.5 |
| Eucalyptol | C09844 | 11198772.81 | 14816484.52 | 53.3 | 30180136.46 | 26774294.23 | 28.52 | 0.37 | -1.43 |
| 4-Methoxybenzaldehyde | C10761 | 6935768.73 | 6794316.42 | 8.4 | 8720222.95 | 8786012.3 | 4.1 | 0.8 | -0.33 |
| p-Aminobenzoic acid | C00568 | 1000746.43 | 1018452.26 | 12.3 | 7358678.93 | 7436726.16 | 8.24 | 0.14 | -2.88 |
| 3,4-Dihydroxybenzaldehyde | C16700 | 45263177.16 | 45643644.47 | 6.22 | 34686926.71 | 34608336.48 | 3.22 | 1.3 | 0.38 |
| Phosphonoacetate | C05682 | 19990325.75 | 20567436.62 | 8.63 | 12357830.75 | 12335237.79 | 8.78 | 1.62 | 0.69 |
| N-methyl-L-glutamic Acid | C01046 | 22473364.6 | 22521754.07 | 2.85 | 1254877.44 | 1027356.47 | 35.36 | 17.91 | 4.16 |
| Tryptophanol | C00955 | 12721955.08 | 12626332.54 | 8.12 | 1548085.97 | 990114.16 | 70.82 | 8.22 | 3.04 |
| Acetylcholine | C01996 | 4027048.82 | 3979102.9 | 7.88 | 11012403.84 | 11006441.82 | 9.69 | 0.37 | -1.45 |
| 2-Keto-6-aminocaproate | C03239 | 17179545.84 | 18395552.89 | 25.52 | 36174134.27 | 35936338.43 | 3.09 | 0.47 | -1.07 |
| (2R,5S)-2,5-Diaminohexanoate | C05161 | 978755.56 | 516764.56 | 82.59 | 8431302.26 | 8616941.25 | 6.14 | 0.12 | -3.11 |
| Coumarin | C05851 | 34894619.37 | 34745691.88 | 5.83 | 28140014.45 | 27934054.52 | 3.77 | 1.24 | 0.31 |
| L-Glutamine | C00064 | 471884916.6 | 476453326.8 | 5.38 | 255707181.9 | 263489767.1 | 9.24 | 1.85 | 0.88 |
| 3,4-Dihydro-2H-1-benzopyran-2-one | C02274 | 14102762.24 | 14081379.42 | 9.78 | 7701311.1 | 7519302.89 | 20.16 | 1.83 | 0.87 |
| Estragole | C10452 | 279032805 | 272248833.9 | 7.87 | 238103603.4 | 236357256.8 | 2.78 | 1.17 | 0.23 |
| Phthalic acid | C01606 | 87550807.94 | 77367927.77 | 24.9 | 36977361.46 | 34781748.13 | 14.59 | 2.37 | 1.24 |
| L-2-Hydroxyglutaric acid | C03196 | 16451322.97 | 16449173.01 | 1.83 | 13130607.73 | 13080366.57 | 3.09 | 1.25 | 0.33 |
| D-Lyxose | C00476 | 35870011.49 | 35839297.22 | 3.73 | 30286962.51 | 30724242.65 | 3.92 | 1.18 | 0.24 |
| Homogentisic acid | C00544 | 10704981.49 | 10707298.6 | 5.99 | 30774401.37 | 30840552.96 | 4.5 | 0.35 | -1.52 |
| D-Ribose | C00121 | 5167044.44 | 5113816.61 | 10.42 | 7920686.53 | 7701457.55 | 9.02 | 0.65 | -0.62 |
| Guanine | C00242 | 29179078.48 | 31715152.61 | 23.06 | 10820675.35 | 11647200.18 | 32 | 2.7 | 1.43 |
| N-Methyltyramine | C02442 | 10133066.88 | 9990829.23 | 10.02 | 5637016.15 | 5305392.09 | 30.22 | 1.8 | 0.85 |
| p-Hydroxyphenylacetic acid | C00642 | 2080399.49 | 2386868.67 | 34.82 | 45224155.63 | 45599308.18 | 5.94 | 0.05 | -4.44 |
| Ortho-Hydroxyphenylacetic acid | C05852 | 1755913.38 | 1770514.87 | 3.43 | 4653595.31 | 4667724.65 | 9.77 | 0.38 | -1.41 |
| (-)-cis-Carveol | C11395 | 208241114.9 | 199839033.6 | 9.45 | 135805215.5 | 131547341.8 | 7.22 | 1.53 | 0.62 |
| 4,5-Dihydroorotic acid | C00337 | 39517392.42 | 39385306.7 | 2.92 | 45095460.48 | 45607107.66 | 2.54 | 0.88 | -0.19 |
| L-Carnitine | C00318 | 9766288.25 | 12153427.09 | 49.61 | 34096450.49 | 33754903.93 | 5.54 | 0.29 | -1.8 |
| cis-1,2-Dihydronaphthalene-1,2-diol | C04314 | 7294879.48 | 7801732.45 | 24.26 | 2451946.45 | 2143773.28 | 34.27 | 2.98 | 1.57 |
| 2-Deoxystreptamine | C02627 | 49343536.36 | 49948021.98 | 27.72 | 24741223.93 | 24644868.99 | 16.81 | 1.99 | 1 |
| Isoeugenol | C10469 | 30829838.48 | 31827429.26 | 39.68 | 118991240.4 | 119964254.8 | 10.91 | 0.26 | -1.95 |
| Eugenol | C10453 | 103536832.6 | 102909221.7 | 2.64 | 146151797.9 | 145898435.9 | 3.01 | 0.71 | -0.5 |
| 2-Phenylethyl acetate | C12303 | 38456927.23 | 38379352.2 | 9.23 | 24044968.79 | 23783076.24 | 6.04 | 1.6 | 0.68 |
| 7-Methylxanthine | C16353 | 11727095.68 | 11715531.46 | 2.97 | 10395793.35 | 10294619.61 | 2.64 | 1.13 | 0.17 |
| 3-(2-Hydroxyphenyl)propanoic acid | C01198 | 125707501.9 | 126323320.4 | 2.06 | 117233009.6 | 116348215.9 | 4.75 | 1.07 | 0.1 |
| (S)-4-Hydroxymandelate | C03198 | 336570260.6 | 342732759.5 | 11.98 | 163797405.4 | 160108413.3 | 7.52 | 2.05 | 1.04 |
| Pyridoxine | C00314 | 147493145.8 | 146555911.3 | 6.36 | 234870071.6 | 237104014.1 | 3.41 | 0.63 | -0.67 |
| 8-Amino-7-oxononanoate | C01092 | 25043282.39 | 25187614.96 | 2.13 | 760339.14 | 765814.42 | 7.34 | 32.94 | 5.04 |
| 2-Biphenylol | C02499 | 33151109.74 | 33379416.13 | 7.39 | 8608366.34 | 8441088.7 | 4.9 | 3.85 | 1.95 |
| Levetiracetam | C07841 | 26223751.82 | 26274581.87 | 9 | 37955044.53 | 38948009.64 | 11.08 | 0.69 | -0.53 |
| Dihydroxyacetone phosphate | C00111 | 26219833.61 | 26131137.29 | 3.67 | 20101542.69 | 20243357.22 | 6.16 | 1.3 | 0.38 |
| (2S,5S)-trans-Carboxymethylproline | C17366 | 64875913.35 | 64484352.33 | 3.44 | 32438952.21 | 29041161.29 | 19.2 | 2 | 1 |
| N-Acetylleucine | C02710 | 5443344.54 | 5203210.22 | 8.53 | 7382467.54 | 7384369.77 | 19.13 | 0.74 | -0.44 |
| N-Acetyl-L-glutamate 5-semialdehyde | C01250 | 24607465.76 | 24389423.64 | 2.62 | 30072552.44 | 29984520.24 | 12.72 | 0.82 | -0.29 |
| N-Acetyl-L-aspartic acid | C01042 | 17233125.96 | 17362525.89 | 4.77 | 18824430.39 | 18743567.97 | 3.56 | 0.92 | -0.13 |
| Ascorbate | C00072 | 872561817.8 | 860244215.1 | 4.67 | 163634752.1 | 159708612.2 | 16.77 | 5.33 | 2.41 |
| D-Galacturonolactone | C06430 | 36847115.52 | 36495805.95 | 5.46 | 33070526.15 | 33342862.78 | 3.2 | 1.11 | 0.16 |
| L-Bornesitol | C03660 | 249392894.1 | 243377353.4 | 30.8 | 55554199.24 | 62732134.56 | 35.7 | 4.49 | 2.17 |
| Coniferyl aldehyde | C02666 | 193831570.8 | 140804104.8 | 50.66 | 441058853.7 | 438201562.8 | 3.37 | 0.44 | -1.19 |
| Methylisoeugenol | C10478 | 19214612.8 | 19066558.02 | 2.63 | 14096170.83 | 13988200.87 | 4.69 | 1.36 | 0.45 |
| 5-Deoxy-D-glucuronate | C16737 | 34264290.85 | 33437247.17 | 6.37 | 20414760.19 | 20443898.17 | 2.01 | 1.68 | 0.75 |
| 1-Amino-1-deoxy-scyllo-inositol | C01214 | 18910118.14 | 18669390.54 | 7.24 | 27553299.23 | 26199165.65 | 18.12 | 0.69 | -0.54 |
| D-Psicose | C06468 | 69519202.7 | 68701076.38 | 3.81 | 123755847.1 | 123931729.5 | 3.33 | 0.56 | -0.83 |
| 1D-chiro-Inositol | C19891 | 10468786.63 | 10613599.53 | 5.22 | 19404573.14 | 19411575.43 | 1.94 | 0.54 | -0.89 |
| (S)-beta-Tyrosine | C21308 | 243876.78 | 147847.34 | 81.93 | 14656726.68 | 14715811.06 | 3.57 | 0.02 | -5.91 |
| Hydroxyphenyllactic acid | C03672 | 60149542.25 | 60020111.32 | 2.99 | 97925457.07 | 98147585.08 | 2.04 | 0.61 | -0.7 |
| 5-Oxo-1,2-campholide | C02952 | 31188234.97 | 32426979.85 | 14.69 | 15598677.77 | 16512784.11 | 19.37 | 2 | 1 |
| Choline sulfate | C00919 | 112197038.1 | 112065331.1 | 1.78 | 65719741.37 | 65756528.93 | 5.65 | 1.71 | 0.77 |
| 3-O-Methylgallate | C05616 | 3711348.45 | 4338075.11 | 60.59 | 14143139.67 | 14152198.48 | 13.03 | 0.26 | -1.93 |
| Sebacic acid | C08277 | 20421156.98 | 20882799.33 | 5.12 | 8177782.96 | 7752050.45 | 15.93 | 2.5 | 1.32 |
| Phosphoserine | C01005 | 3496783.65 | 3813701.68 | 19.32 | 2471393.87 | 2538722.06 | 12.32 | 1.41 | 0.5 |
| 6-Acetamido-3-oxohexanoate | C03682 | 33592798.65 | 32357802.11 | 12.24 | 43696607.1 | 44946811.94 | 5.3 | 0.77 | -0.38 |
| N-Alpha-acetyllysine | C12989 | 2707553804 | 2479765952 | 33.84 | 1241045666 | 1196411396 | 30.12 | 2.18 | 1.13 |
| Homocitrulline | C02427 | 15184424.4 | 15269575.53 | 11.23 | 47476717.12 | 47966827.96 | 6.32 | 0.32 | -1.64 |
| Homo-L-arginine | C01924 | 112240074.1 | 108079828.3 | 8.81 | 70109671.49 | 70078607.26 | 1.34 | 1.6 | 0.68 |
| Glycylleucine | C02155 | 2587884.83 | 2553911.3 | 2.61 | 2223275.79 | 2237200.12 | 1.31 | 1.16 | 0.22 |
| N-Acetylglutamic acid | C00624 | 11798002.57 | 11782916.72 | 3.44 | 5224512.21 | 5263295.42 | 3.01 | 2.26 | 1.18 |
| Diaminopimelic acid | C00666 | 36748974.96 | 36690446.85 | 2.71 | 18839782.97 | 21321652.64 | 34.46 | 1.95 | 0.96 |
| 4-Methoxy-2,2'-bipyrrole-5-carbaldehyde | C21570 | 8016383.86 | 7999954.48 | 26.02 | 53791848 | 54913020.2 | 9.51 | 0.15 | -2.75 |
| 6-Methoxymellein | C02381 | 35725777.73 | 36348016.59 | 4.17 | 47724950 | 47481792.89 | 4.6 | 0.75 | -0.42 |
| 5-Hydroxyindoleacetic acid | C05635 | 704819.33 | 384578.41 | 116.21 | 5135229.51 | 5124506.6 | 10.02 | 0.14 | -2.87 |
| Myristicin | C10480 | 9992253.94 | 9912577.24 | 8.26 | 7535104.27 | 7408562.69 | 10.64 | 1.33 | 0.41 |
| Methoxamine | C07513 | 3250460.84 | 3266143.38 | 2.01 | 2812028.56 | 2812892.26 | 5.9 | 1.16 | 0.21 |
| Leucodopachrome | C05604 | 1533621.61 | 1007857.56 | 70.99 | 12582713.56 | 11717297.11 | 18.93 | 0.12 | -3.04 |
| (-)-Bornesitol | C03659 | 257370526.4 | 256192643.8 | 11.06 | 812105275.4 | 811653105.8 | 2.06 | 0.32 | -1.66 |
| Metanephrine | C05588 | 5046587.82 | 5755373.42 | 36 | 11126857.58 | 11393164 | 8.16 | 0.45 | -1.14 |
| Syringic acid | C10833 | 37394591.13 | 37262534.41 | 2.94 | 78083570.57 | 78419898.89 | 2.67 | 0.48 | -1.06 |
| Ecgonine methyl ester | C12448 | 11488797.78 | 11875092.97 | 10.67 | 20398455.24 | 20617735.15 | 6.23 | 0.56 | -0.83 |
| Dodecanoic acid | C02679 | 10932062.03 | 8187178.68 | 63.07 | 35492422.89 | 35881618.57 | 4.11 | 0.31 | -1.7 |
| Zerumbone | C20262 | 2389646.11 | 2310688.26 | 62.29 | 5482519.71 | 6025029.2 | 23.53 | 0.44 | -1.2 |
| Thiabendazole | C07131 | 779150409.4 | 836573798.7 | 18.51 | 518677020.4 | 516372514.7 | 17.59 | 1.5 | 0.59 |
| Caryophyllene alpha-oxide | C16908 | 24652806.55 | 27961052.4 | 33.98 | 8066794.91 | 5346960.73 | 87.22 | 3.06 | 1.61 |
| Apiole | C10429 | 7419058.41 | 7571255.17 | 5.56 | 6654059.45 | 6625569.57 | 4.85 | 1.11 | 0.16 |
| Pantothenol | C05944 | 5979587.8 | 6059606 | 6.34 | 4032428.21 | 3994209.68 | 4.32 | 1.48 | 0.57 |
| Ibuprofen | C01588 | 16109590.22 | 15930289.04 | 7.76 | 40352447.65 | 39411280.88 | 7.94 | 0.4 | -1.32 |
| 3-[(1-Carboxyvinyl)oxy]benzoate | C20772 | 14938026.26 | 14454775.93 | 17.01 | 11485394.12 | 11316853.64 | 7.44 | 1.3 | 0.38 |
| L-Kynurenine | C00328 | 11324492.79 | 11980419.58 | 19.92 | 8522294.66 | 8507975.32 | 2.72 | 1.33 | 0.41 |
| Sinapoyl aldehyde | C05610 | 3155669.47 | 3251652.32 | 8.13 | 5861123.62 | 5449054.79 | 19.05 | 0.54 | -0.89 |
| trans-Isoasarone | C17846 | 7875386.39 | 7924808.99 | 4.08 | 22494723.78 | 22474784.45 | 14.73 | 0.35 | -1.51 |
| N-Acetyldemethylphosphinothricin | C17949 | 5365162.66 | 5412687.01 | 12.69 | 95434626.4 | 93312304.18 | 6.32 | 0.06 | -4.15 |
| (+)-7-Isojasmonic acid | C16317 | 59382636.36 | 59291393.3 | 6.13 | 52662282.53 | 53257165.42 | 6.49 | 1.13 | 0.17 |
| Dethiobiotin | C01909 | 18267653.92 | 18413366.67 | 4.81 | 11444158.06 | 11788198.94 | 7.28 | 1.6 | 0.67 |
| Cis-zeatin | C00371 | 10419073.8 | 10478662.32 | 2.35 | 9210056.56 | 9242179.05 | 1.37 | 1.13 | 0.18 |
| 2-trans,6-trans-Farnesal | C03461 | 41755826.59 | 41560798.88 | 5.99 | 71644878.27 | 71430674.73 | 10.74 | 0.58 | -0.78 |
| Diethyl phthalate | C14175 | 9348068.55 | 9300707.63 | 4.23 | 7550112.64 | 7544262.09 | 2.82 | 1.24 | 0.31 |
| Cerulenin | C12058 | 9097168.86 | 9667026.8 | 19.64 | 14836441.7 | 14953831.06 | 4.73 | 0.61 | -0.71 |
| Prephenate | C00254 | 3164136.04 | 1773001.81 | 78.31 | 26350470.82 | 26363239.19 | 3.44 | 0.12 | -3.06 |
| Genipin | C09780 | 16203721.87 | 15996570.01 | 8.33 | 13783388.93 | 13575674.8 | 9.42 | 1.18 | 0.23 |
| Myristoleic acid | C08322 | 6096653.09 | 4152634.46 | 52.75 | 33473268.29 | 34342676.95 | 11.56 | 0.18 | -2.46 |
| Myristic acid | C06424 | 11059531.01 | 11626263.4 | 51.44 | 1817741.55 | 1764479.62 | 9.79 | 6.08 | 2.61 |
| 6-Hydroxymelatonin | C05643 | 42524347.22 | 41590452.12 | 9.98 | 30484280.76 | 30602377.15 | 4.29 | 1.39 | 0.48 |
| Alantolactone | C09289 | 498515.27 | 545355.23 | 31.56 | 12639403.08 | 12713302.83 | 3.52 | 0.04 | -4.66 |
| N1-Acetylspermine | C02567 | 3547410.91 | 3546670.88 | 3.42 | 4129962.91 | 4202905.95 | 7.05 | 0.86 | -0.22 |
| Uridine | C00299 | 36407982.62 | 35712849.89 | 9.13 | 48818089.42 | 46304243.06 | 18.88 | 0.75 | -0.42 |
| Pindolol | C07445 | 480012.32 | 493179.82 | 15.45 | 332481.48 | 328098.42 | 3.34 | 1.44 | 0.53 |
| gamma-L-Glutamyl-L-cysteine | C00669 | 11793237.35 | 11760409.08 | 13.38 | 32935580.31 | 33850138.51 | 11.19 | 0.36 | -1.48 |
| 5'-Deoxyadenosine | C05198 | 6402139.18 | 6292752.57 | 18.78 | 8853241.37 | 9436845.7 | 13.88 | 0.72 | -0.47 |
| Benzo[k]fluoranthene | C14321 | 13331502.71 | 13658503.67 | 8.24 | 8479143.61 | 8682647.07 | 6.79 | 1.57 | 0.65 |
| 16-Oxopalmitate | C19614 | 6921989.72 | 6560616.74 | 69.75 | 15804695.48 | 14467400.53 | 29.34 | 0.44 | -1.19 |
| Nicotinamide riboside | C03150 | 22371724.65 | 15475998.65 | 63.54 | 60114973.08 | 58927085.51 | 11.9 | 0.37 | -1.43 |
| Nandrolone | C07254 | 133433552.3 | 132906031.8 | 3.78 | 11883412.4 | 11800264.51 | 2.62 | 11.23 | 3.49 |
| Glycerophosphocholine | C00670 | 106977919.9 | 115438617 | 20.34 | 166188006.6 | 165151864.1 | 6.23 | 0.64 | -0.64 |
| Parthenin | C09523 | 18687592.87 | 18684913.55 | 4.59 | 12397467.23 | 12813281.94 | 8.14 | 1.51 | 0.59 |
| Qing Hau Sau | C09538 | 12739470.7 | 12442686.86 | 12.15 | 2988989.94 | 2795938.31 | 20.72 | 4.26 | 2.09 |
| Mirtazapine | C07570 | 62789382.37 | 61131786.14 | 7.26 | 45070393.59 | 45054343.93 | 4.23 | 1.39 | 0.48 |
| S-Ribosyl-L-homocysteine | C03539 | 19913497.66 | 19896305.68 | 4.56 | 22720361.99 | 23093082.79 | 5.17 | 0.88 | -0.19 |
| Xanthoxic acid | C13454 | 17927699.76 | 17602980.88 | 4.97 | 67854556.87 | 68043078.36 | 16.12 | 0.26 | -1.92 |
| Adenosine | C00212 | 1175129135 | 1203000903 | 33.57 | 13533269456 | 13495789520 | 3.99 | 0.09 | -3.53 |
| (S)-Coclaurine | C06161 | 176913807.4 | 175302378.8 | 5.07 | 201820821.9 | 200357864.3 | 3.49 | 0.88 | -0.19 |
| (R)-Coclaurine | C06349 | 11029917.38 | 11216898.02 | 5.19 | 446341.82 | 238032.06 | 80.32 | 24.71 | 4.63 |
| Androstenedione | C00280 | 2112688.13 | 1245357.21 | 67.2 | 5256647.98 | 5857379.48 | 33.02 | 0.4 | -1.32 |
| Luteolinidin | C08652 | 288643.52 | 288856.73 | 51.36 | 2867281.57 | 3454509.6 | 54.66 | 0.1 | -3.31 |
| Norizalpinin | C10044 | 92358077.08 | 92432668.8 | 3.38 | 167669702.8 | 167241077.1 | 2.53 | 0.55 | -0.86 |
| Genistein | C06563 | 20797278.87 | 19499890.67 | 23.58 | 30543892.37 | 30216683.91 | 5.35 | 0.68 | -0.55 |
| All-trans-13,14-dihydroretinol | C15492 | 124774917.6 | 125166886.9 | 8.31 | 183485989 | 185202262.2 | 6.34 | 0.68 | -0.56 |
| Etiocholanedione | C03772 | 35717214.07 | 34799794.15 | 9.13 | 57528214.33 | 59832112.97 | 12.57 | 0.62 | -0.69 |
| Thienamycin | C06664 | 11240351.93 | 10808626.78 | 9.11 | 7641505.36 | 7723074.82 | 5.49 | 1.47 | 0.56 |
| Etiocholanolone | C04373 | 4805396.94 | 4990630.89 | 54.01 | 10810778.3 | 11294110.62 | 33.89 | 0.44 | -1.17 |
| Naringenin | C00509 | 33305009.71 | 33673509.01 | 5.18 | 19680565.42 | 23505378.21 | 39.04 | 1.69 | 0.76 |
| Androsterone | C00523 | 54318181.45 | 53481793.82 | 6.52 | 145186140.7 | 175094701.9 | 42.83 | 0.37 | -1.42 |
| Apiforol | C12124 | 10800928.13 | 5583461.16 | 83.13 | 53200186.43 | 46813173.01 | 26.3 | 0.2 | -2.3 |
| Afzelechin | C09320 | 111033171 | 143866434.1 | 54.79 | 27266584.43 | 27152697.9 | 10.66 | 4.07 | 2.03 |
| 5a-Androstane-3b,17b-diol | C12525 | 9124007.68 | 9060674.39 | 5.01 | 5091149.92 | 5148267.36 | 4.72 | 1.79 | 0.84 |
| N6-(L-1,3-Dicarboxypropyl)-L-lysine | C00449 | 6595130.17 | 6465436.33 | 14.16 | 5088008.67 | 5151397.8 | 4.5 | 1.3 | 0.37 |
| Cyclopeptine | C20579 | 7236365.72 | 7272757.93 | 6.45 | 5148843.08 | 5005915.46 | 8.6 | 1.41 | 0.49 |
| 9-Riburonosyladenine | C11501 | 4072187.72 | 4058653.27 | 9.9 | 8444525.06 | 7203582.23 | 25.47 | 0.48 | -1.05 |
| 1-Methyladenosine | C02494 | 1103756.16 | 1407759.14 | 48.5 | 5652062.79 | 5666137.86 | 8 | 0.2 | -2.36 |
| Guanosine | C00387 | 251372424.9 | 251529949.5 | 5.64 | 452314118.5 | 457442756.5 | 9.14 | 0.56 | -0.85 |
| (R,S)-Coclaurine | C06348 | 86882123.07 | 86395204.68 | 9.82 | 65681805.17 | 66847844.05 | 10.26 | 1.32 | 0.4 |
| N1,N12-Diacetylspermine | C03413 | 1665574.16 | 1653070.5 | 29.66 | 25740371.19 | 24992236.01 | 10.98 | 0.06 | -3.95 |
| Fisetin | C10041 | 49891446.81 | 49286197.18 | 11.06 | 104460370.2 | 99659000.96 | 10.29 | 0.48 | -1.07 |
| Eriodictyol | C05631 | 11184296.32 | 5844652.81 | 76.73 | 221702.5 | 143143.32 | 67.31 | 50.45 | 5.66 |
| Dyclonine | C07881 | 4619107.89 | 3563588.08 | 54.8 | 1592869.51 | 1523534.77 | 10.75 | 2.9 | 1.54 |
| Aurin | C14213 | 113054538.4 | 112592913 | 2.36 | 32599069.73 | 32486886.67 | 1.17 | 3.47 | 1.79 |
| (+_-)-5-[(tert-Butylamino)-2'-hydroxypropoxy]-3,4-dihydro-1(2H)-naphthalenone | C04883 | 20783214.15 | 14389754.15 | 59.32 | 7698839.33 | 7411567.9 | 9.5 | 2.7 | 1.43 |
| Sclareol | C09183 | 14277997.88 | 14335608.8 | 3.18 | 26959946.06 | 26496607.21 | 6.31 | 0.53 | -0.92 |
| 9(S)-HPOT | C16321 | 16251456.54 | 16866707.91 | 12.2 | 8495788.16 | 8545290.05 | 3.04 | 1.91 | 0.94 |
| Prunasin | C00844 | 77615025.59 | 83222543.06 | 23.84 | 27975642.98 | 28476196.36 | 14.87 | 2.77 | 1.47 |
| 5'-Methylthioadenosine | C00170 | 7494528.61 | 7449844.1 | 19.83 | 57629715.86 | 60831835.41 | 56.81 | 0.13 | -2.94 |
| Norethindrone | C05028 | 35955814.19 | 33694495.11 | 23.78 | 13694097.69 | 13489108.84 | 10.32 | 2.63 | 1.39 |
| Cafestol | C09066 | 15319723.86 | 9242580.28 | 75.49 | 167243842.6 | 166264034.6 | 16.44 | 0.09 | -3.45 |
| all-trans-5,6-Epoxyretinoic acid | C16680 | 58754031.41 | 57660993.59 | 15.02 | 37439803.13 | 41303139.53 | 25.03 | 1.57 | 0.65 |
| Metoclopramide | C07868 | 4922258.16 | 4885499.33 | 6.83 | 14293761.34 | 15978454.27 | 33.24 | 0.34 | -1.54 |
| 5-Nitro-2-(3-phenylpropylamino)benzoic acid | C13705 | 6980976.68 | 7136871.57 | 9.01 | 12045251.91 | 10174253.68 | 31.35 | 0.58 | -0.79 |
| Isotretinoin | D00348 | 6160471.95 | 6118190.93 | 21.3 | 8986584.03 | 8387220.78 | 20.27 | 0.69 | -0.54 |
| Questinol | C17811 | 4405394.87 | 4003502.66 | 36.62 | 2497131.48 | 2531804.08 | 27.3 | 1.76 | 0.82 |
| Kaempferide | C10098 | 4089066.14 | 4198570.55 | 7.97 | 11172549.29 | 10745054.94 | 8.03 | 0.37 | -1.45 |
| 2-Methoxy-17beta-estradiol | C05302 | 4858737.2 | 3658736.92 | 58.04 | 14686454.39 | 14890768.02 | 11.23 | 0.33 | -1.6 |
| Sphinganine | C00836 | 1910007349 | 1894123061 | 12.57 | 107517846.4 | 95740630.53 | 29.36 | 17.76 | 4.15 |
| Norethandrolone | D07127 | 131541651.1 | 130969765.3 | 6.06 | 65449992.81 | 63769836.39 | 11.07 | 2.01 | 1.01 |
| Tricetin | C10192 | 27662303.06 | 29198907.57 | 17.79 | 9743373.25 | 5194298.7 | 73.35 | 2.84 | 1.51 |
| Ferreirin | C10419 | 14429382.56 | 14453841.83 | 4.38 | 10797774.28 | 11035764.81 | 7.44 | 1.34 | 0.42 |
| Abietate | C06087 | 11360489.47 | 11097114.77 | 8.3 | 2689636.61 | 1629564.65 | 64 | 4.22 | 2.08 |
| cis-Dihydroquercetin | C12316 | 633393497.7 | 645952725.7 | 6.7 | 120028000.7 | 121014101.4 | 12.8 | 5.28 | 2.4 |
| Leucodelphinidin | C05909 | 16053405.47 | 16331361.14 | 8.81 | 27167596.23 | 28003401.24 | 11.66 | 0.59 | -0.76 |
| 11alpha,17beta-Dihydroxyandrost-4-en-3-one | C15306 | 24360571.28 | 26783018.83 | 38.01 | 2339993.2 | 2380788.68 | 18.4 | 10.41 | 3.38 |
| Glutathione | C00051 | 2909356318 | 2225452229 | 47.37 | 1284358870 | 1207360884 | 23.96 | 2.27 | 1.18 |
| Bisdemethoxycurcumin | C17743 | 207495895 | 209415814.5 | 16.5 | 73084305.84 | 68473768.88 | 13.87 | 2.84 | 1.51 |
| Alprazolam | C06817 | 1066812.21 | 1051458.3 | 32.14 | 24201386.43 | 23425901.55 | 13.93 | 0.04 | -4.5 |
| beta-D-Galactosyl-(1->4)-L-rhamnose | C19758 | 13339356.92 | 11662494 | 34.05 | 8608844.47 | 8546766.01 | 4.26 | 1.55 | 0.63 |
| Phenylbutazone | C07440 | 59935415.37 | 56356080.2 | 21.04 | 37642040.09 | 34889311.01 | 18.82 | 1.59 | 0.67 |
| 2,3-Dinor-8-iso prostaglandin F2alpha | C14794 | 43736548.28 | 43461938.74 | 6.26 | 34470558.82 | 34386423.81 | 2.18 | 1.27 | 0.34 |
| N-Acetyl-a-neuraminic acid | C19909 | 5515451.28 | 3388592.46 | 117.61 | 91901465.69 | 96597506.66 | 9.39 | 0.06 | -4.06 |
| Methoprene | C14308 | 12891650.62 | 13076838.24 | 11.54 | 10173538.19 | 10234337.63 | 4.5 | 1.27 | 0.34 |
| Aflatoxin B1 | C06800 | 25289864.19 | 24825840.18 | 9.19 | 39425835.62 | 39729776.27 | 6.85 | 0.64 | -0.64 |
| Progesterone | C00410 | 133690792.2 | 128149980.9 | 13.28 | 188583634.5 | 194055356.2 | 11.25 | 0.71 | -0.5 |
| 5a-Pregnane-3,20-dione | C03681 | 31925317.29 | 31821179.3 | 9.25 | 42645624.18 | 42690215.65 | 11.87 | 0.75 | -0.42 |
| N(beta)-Epoxysuccinamoyl-DAP-Val | C20965 | 20397270.99 | 20385727.71 | 5.16 | 1865818.59 | 1914952.12 | 11.85 | 10.93 | 3.45 |
| Penicillin G | C05551 | 3693116.4 | 3657785.6 | 3.22 | 4210399.41 | 4220819.37 | 4.38 | 0.88 | -0.19 |
| Chlorpromazine | C06906 | 50334555.1 | 50630615.82 | 4.76 | 39381681.44 | 42929257.6 | 18.6 | 1.28 | 0.35 |
| Myricetin | C10107 | 47264255.33 | 54407114.88 | 27.32 | 29295093.81 | 29177292.57 | 2.89 | 1.61 | 0.69 |
| 11alpha,17beta-Dihydroxy-17-methylandrost-4-en-3-one | C14555 | 69300481 | 67172350.83 | 16.71 | 106019900.8 | 107062352.5 | 14.19 | 0.65 | -0.61 |
| Deoxy-5-methylcytidylate | C03495 | 34091597.78 | 33730931.44 | 5.99 | 21815750.58 | 21159705.5 | 8.32 | 1.56 | 0.64 |
| gamma-L-Glutamyl-L-cysteinyl-beta-alanine | C04544 | 192757865.3 | 179793396.5 | 20.83 | 470963999 | 462330256.4 | 14.37 | 0.41 | -1.29 |
| dTMP | C00364 | 7769768.36 | 7851203.97 | 3.4 | 643079.03 | 639797.22 | 15.23 | 12.08 | 3.59 |
| Stylopine | C05175 | 39744998.37 | 35409465.13 | 25.21 | 63854213.93 | 66308063.3 | 12.12 | 0.62 | -0.68 |
| Galactinol | C01235 | 10555811.38 | 11265487.97 | 18.06 | 14951387.5 | 14907492.92 | 15.33 | 0.71 | -0.5 |
| Coniferin | C00761 | 150188755.9 | 156244572.2 | 13.34 | 102933332.9 | 104973043 | 12.13 | 1.46 | 0.55 |
| Citalopram | C07572 | 18596083.2 | 18508938.29 | 9.83 | 13027550.98 | 12993486.96 | 15.28 | 1.43 | 0.51 |
| Cellobiose | C00185 | 118288009.9 | 112651401.1 | 28.75 | 2438144959 | 2256240248 | 17.43 | 0.05 | -4.37 |
| 2,4-Dioxotetrahydropyrimidine D-ribonucleotide | C04639 | 20441665.62 | 20469704.93 | 5.13 | 12345455.19 | 11736644.21 | 11.61 | 1.66 | 0.73 |
| Corticosterone | C02140 | 18294273.74 | 18873288.63 | 8.42 | 42016990.62 | 42189403.38 | 3.61 | 0.44 | -1.2 |
| Carnosol | C09069 | 8139316.01 | 8146746.46 | 3.78 | 6501118.44 | 6462805.57 | 4.14 | 1.25 | 0.32 |
| Cannabielsoin | C20218 | 10090210.52 | 10198947.05 | 7.28 | 6749919.02 | 6724119.34 | 10.82 | 1.49 | 0.58 |
| 5'-O-beta-D-Glucosylpyridoxine | C03996 | 269384990.3 | 260781082.9 | 52.94 | 837641997.2 | 812709587.9 | 7.13 | 0.32 | -1.64 |
| Adrenic acid | C16527 | 41647939.88 | 40175852.03 | 16.23 | 8225581.64 | 6033940.85 | 76.78 | 5.06 | 2.34 |
| Norsanguinarine | C05191 | 17457424.96 | 17612485.36 | 4.57 | 139295884.7 | 136484709.4 | 10.1 | 0.13 | -3 |
| Gibberellin A4 | C11864 | 40336365.41 | 36488319.33 | 17.35 | 25487878.07 | 24173481.17 | 9.77 | 1.58 | 0.66 |
| Prostaglandin D2 | C00696 | 23696291.29 | 19670745.12 | 31.86 | 11755621.57 | 11944208.78 | 7.51 | 2.02 | 1.01 |
| Isopentenyl adenosine | C16427 | 2146862.1 | 1430750.49 | 58.58 | 8555913.67 | 8700258.09 | 10.72 | 0.25 | -1.99 |
| p-Coumaroyl quinic acid | C12208 | 15893094.1 | 15873059.88 | 6.49 | 1463255.76 | 1621265.26 | 34.49 | 10.86 | 3.44 |
| (S)-cis-N-Methylstylopine | C06163 | 17214865.07 | 17629563.21 | 9.19 | 10622437.98 | 10949750.9 | 9.14 | 1.62 | 0.7 |
| Isocorypalmine | C04118 | 16010302.51 | 15746730.68 | 3.98 | 17829183.76 | 17657652.96 | 3.57 | 0.9 | -0.16 |
| Turanose | C19636 | 34323361.78 | 36312905.75 | 24.08 | 279075369.8 | 259033525.8 | 13.67 | 0.12 | -3.02 |
| Clotrimazole | C06922 | 33530603.24 | 27856943.52 | 30.57 | 148470550.3 | 155392924.8 | 14.95 | 0.23 | -2.15 |
| Cyclic GMP | C00942 | 9788855.88 | 9636994.27 | 6.18 | 11435829.47 | 11663013.72 | 6.46 | 0.86 | -0.22 |
| N-Acetylmuramoyl-Ala | C02999 | 6696395.62 | 6638612.82 | 3.83 | 2112073.49 | 2000850.68 | 49.2 | 3.17 | 1.66 |
| 21-Deoxycortisol | C05497 | 1864611.17 | 963395.37 | 80.3 | 13061472.79 | 13774730.41 | 10.6 | 0.14 | -2.81 |
| 2-Hydroxy-6-pentadecylbenzoic acid | C10759 | 3981452.22 | 2672552.16 | 91.67 | 14408027.56 | 14190709.07 | 20.21 | 0.28 | -1.86 |
| 6-Keto-prostaglandin F1a | C05961 | 28367132.51 | 28887698.01 | 7.87 | 23738680.97 | 23794559.11 | 2.24 | 1.19 | 0.26 |
| 17-O-Acetylnorajmaline | C11809 | 17748087.26 | 18577795.83 | 16 | 4825708.26 | 5428449.68 | 33.81 | 3.68 | 1.88 |
| Chlorogenic acid | C00852 | 16595034.88 | 17393383.9 | 10.03 | 1051681.99 | 1023335.55 | 5.98 | 15.78 | 3.98 |
| Laudanosine | C09558 | 61806850.16 | 63078880.97 | 4.7 | 15891359.54 | 15686262.85 | 9.92 | 3.89 | 1.96 |
| Niaprazine | D07333 | 87719889.04 | 86967247.05 | 14.63 | 301505403.9 | 295898969.8 | 6.1 | 0.29 | -1.78 |
| Aldosterone | C01780 | 41442569.22 | 41085921.84 | 7.42 | 35515198.67 | 36613584.34 | 8.37 | 1.17 | 0.22 |
| Lariciresinol | C10646 | 16289964.98 | 16636127.3 | 8.3 | 21899043.46 | 22208627.4 | 4.59 | 0.74 | -0.43 |
| Cortisol | C00735 | 2128080.35 | 2214028.5 | 15.23 | 11067705.43 | 10991188.84 | 11.94 | 0.19 | -2.38 |
| 11b,21-Dihydroxy-3,20-oxo-5b-pregnan-18-al | C05473 | 23837796.15 | 26090443.45 | 29.46 | 8335805.38 | 8290851.37 | 4.96 | 2.86 | 1.52 |
| N-Acetylbialaphos | C17951 | 2923368.13 | 2604751.97 | 52.65 | 15433664.71 | 15649873.57 | 6.57 | 0.19 | -2.4 |
| Tamoxifen | C07108 | 7902905.17 | 7912223.95 | 11.94 | 5359180.56 | 5082889.06 | 24.21 | 1.47 | 0.56 |
| Riboflavin | C00255 | 34535817.16 | 35112668.06 | 6.4 | 80335232.68 | 76795369.8 | 12.56 | 0.43 | -1.22 |
| Celecoxib | C07589 | 420587914.2 | 409022308.5 | 16.88 | 171829897.7 | 189450078.3 | 32.06 | 2.45 | 1.29 |
| Mesoridazine | C07143 | 8085392.61 | 6824316.31 | 49.05 | 29045064.99 | 28548992.26 | 7.96 | 0.28 | -1.84 |
| Sufentanil | C08022 | 24150847.27 | 24214314.17 | 6.91 | 13981933.99 | 14157906.47 | 3.72 | 1.73 | 0.79 |
| Ursodeoxycholic acid | C07880 | 4689703.46 | 5393322.22 | 28.89 | 8245416.65 | 7581519.82 | 28.26 | 0.57 | -0.81 |
| Dihydromacarpine | C05316 | 4083341.19 | 4142641.14 | 21.13 | 6760625.7 | 6980902.27 | 16.59 | 0.6 | -0.73 |
| Aloesin | C08994 | 837459.7 | 762182.74 | 26.17 | 22544625.44 | 20659561.15 | 23.41 | 0.04 | -4.75 |
| Ergocalciferol | C05441 | 6938463.25 | 6737572.36 | 18.47 | 1771510.13 | 1766725.77 | 6.09 | 3.92 | 1.97 |
| beta-Sitosterol | C01753 | 32278173.5 | 31050556.45 | 12.36 | 20202763.76 | 19955037.29 | 14.5 | 1.6 | 0.68 |
| S-Adenosylmethionine | C00019 | 77720804.48 | 78061319.49 | 8.12 | 30968092.02 | 30957547.45 | 2.4 | 2.51 | 1.33 |
| Colchicine | C07592 | 16041706.87 | 17582893.14 | 25.38 | 6813644.07 | 6474209.29 | 25.2 | 2.35 | 1.24 |
| Besonprodil | D03100 | 12328672 | 11994726.98 | 12.39 | 16225501.88 | 16276354.54 | 6.37 | 0.76 | -0.4 |
| (-)-alpha-Narcotine | C09592 | 3562143.43 | 3573134.99 | 9.65 | 4546143.81 | 4577279.87 | 11.43 | 0.78 | -0.35 |
| Paspalicine | C20553 | 4841527.19 | 5058541.51 | 14.6 | 10196096.24 | 9800646.2 | 12.53 | 0.47 | -1.07 |
| Kaempferol 3-O-beta-D-xyloside | C20727 | 30045824.73 | 28477413.32 | 16.87 | 102078458.1 | 111021523.6 | 21.46 | 0.29 | -1.76 |
| Blasticidin S | C02010 | 2011117.22 | 2311201.98 | 52.66 | 582506.14 | 376300.92 | 94.05 | 3.45 | 1.79 |
| Ginkgolide B | C07602 | 9475406.18 | 10239679.18 | 34.49 | 20673922.18 | 20938022.6 | 4.65 | 0.46 | -1.13 |
| Afzelin | C16911 | 34410671.14 | 34356278.53 | 10.46 | 85647680.01 | 88797514 | 11.25 | 0.4 | -1.32 |
| 8-C-Glucosylnaringenin | C16492 | 198833510.5 | 214091981.2 | 16.34 | 289802201.9 | 286555184.2 | 21.37 | 0.69 | -0.54 |
| Naringenin 7-O-beta-D-glucoside | C09099 | 3397164.92 | 3665074.44 | 27.07 | 29519279.29 | 30061247.69 | 16.36 | 0.12 | -3.12 |
| 3-Dehydroecdysone | C02513 | 9220715.29 | 9123050.94 | 11.19 | 19921682.82 | 18257311.02 | 21.24 | 0.46 | -1.11 |
| Estrone glucuronide | C11133 | 17294549.99 | 17879218.39 | 19.01 | 11485658.7 | 11272452.26 | 8.84 | 1.51 | 0.59 |
| Etiocholanolone glucuronide | C11136 | 8053630.27 | 8009534.49 | 10.49 | 15023339.91 | 14936317.27 | 4.2 | 0.54 | -0.9 |
| Isoquercitrin | C05623 | 15543679.15 | 15815630.37 | 13.14 | 9757566.49 | 10160813.66 | 16.17 | 1.59 | 0.67 |
| Withaferin A | C08841 | 15720681.51 | 16583484.53 | 18.54 | 5555793.82 | 6395705.58 | 38.25 | 2.83 | 1.5 |
| Retinoyl b-glucuronide | C11061 | 18769893.46 | 19003832.4 | 8.03 | 49018709.35 | 49425427.43 | 20.67 | 0.38 | -1.38 |
| Loperamide | C07080 | 16009018.63 | 12637732.21 | 36.06 | 8657182.63 | 8596364.92 | 3.77 | 1.85 | 0.89 |
| Asiatic acid | C08617 | 593026.73 | 609042.05 | 51.55 | 11137299.94 | 11218436.63 | 12.19 | 0.05 | -4.23 |
| Antibiotic JI-20B | C17705 | 3054424.42 | 2937399.19 | 18 | 24182046.44 | 24548577.58 | 9.97 | 0.13 | -2.98 |
| Mupirocin | C11758 | 28486471.8 | 27700260.14 | 23.19 | 16237987.17 | 16356607.72 | 23.53 | 1.75 | 0.81 |
| 3-alpha(S)-Strictosidine | C03470 | 2340990.23 | 2347860.88 | 2.64 | 5778170.07 | 6033725.1 | 13.74 | 0.41 | -1.3 |
| Flavonol 3-O-beta-D-glucosyl-(1->2)-beta-D-glucoside | C15581 | 3861217.68 | 4229739.08 | 33.09 | 9070997.81 | 9395414.97 | 26.06 | 0.43 | -1.23 |
| N-Acetyl-O-demethylpuromycin-5'-phosphate | C07030 | 3714277.88 | 3902072.71 | 31.71 | 10624958.5 | 10843337.12 | 20.14 | 0.35 | -1.52 |
| Naringin | C09789 | 95377211.67 | 100924927.4 | 31.42 | 39458099 | 40908945.35 | 12.2 | 2.42 | 1.27 |
| Astaxanthin | C08580 | 3475352.15 | 3646663.66 | 11.67 | 5495311.83 | 5620642.85 | 8.78 | 0.63 | -0.66 |
| Candletoxin A | C09068 | 9597701.52 | 9984293.27 | 23.05 | 16176878.47 | 16506818.2 | 18.85 | 0.59 | -0.75 |
| Glutathione amide disulfide | C19690 | 26397684.37 | 29662634.42 | 34.04 | 8231448.83 | 6134043.46 | 68.76 | 3.21 | 1.68 |
| Cyanin | C08639 | 3995129.15 | 4134785.49 | 12.19 | 2062920.13 | 2153480.42 | 24.01 | 1.94 | 0.95 |
| Stachyose | C01613 | 7738603.96 | 7945327.71 | 9.13 | 17004305.29 | 13732568.46 | 55.45 | 0.46 | -1.14 |
| Cyanidin 3-O-(6-O-p-coumaroyl)glucoside-5-O-glucoside | C12096 | 46812051.4 | 47059003.94 | 4.77 | 22682278.63 | 22763453.46 | 30.78 | 2.06 | 1.05 |
| R-Methylmalonyl-CoA | C01213 | 37335683.23 | 39324200.06 | 11.8 | 10843558.3 | 9000860.41 | 44.65 | 3.44 | 1.78 |
| (R)-Malyl-CoA | C20747 | 8956923.86 | 8443278.69 | 16.93 | 4118146.68 | 4462034.35 | 22.57 | 2.17 | 1.12 |
| Glyceric acid | C00258 | 11963534.28 | 11884458.75 | 18.88 | 45270971.48 | 42460443.75 | 17.23 | 0.26 | -1.92 |
| Phenylacetaldehyde | C00601 | 1948274.26 | 1965067.61 | 3.72 | 1098552.58 | 1092597.31 | 2.72 | 1.77 | 0.83 |
| 3-Methylthiopropionic acid | C08276 | 8315736.4 | 8263290.71 | 4.84 | 5342691.55 | 5321276.08 | 5.92 | 1.56 | 0.64 |
| Adipic acid | C06104 | 31443152.52 | 31454455.26 | 6.06 | 38403144.75 | 38857955.9 | 6.11 | 0.82 | -0.29 |
| Pyrrolidonecarboxylic acid | C02237 | 5499821.68 | 5282287.79 | 10.93 | 9120288.85 | 9257805.21 | 7.65 | 0.6 | -0.73 |
| Glutaric acid | C00489 | 110280920.8 | 121339695.6 | 20.98 | 32528207.44 | 29761839.93 | 14.05 | 3.39 | 1.76 |
| L-Aspartic acid | C00049 | 12827647.22 | 12797764.7 | 6.62 | 50268231.52 | 50281150.11 | 4.33 | 0.26 | -1.97 |
| Mandelonitrile | C00561 | 16908892.03 | 16911707.27 | 0.68 | 10132141.75 | 10132396.95 | 2.66 | 1.67 | 0.74 |
| L-Malic acid | C00149 | 59618692.22 | 61466093.83 | 9.58 | 28237821.33 | 34828305.46 | 55.64 | 2.11 | 1.08 |
| Adenine | C00147 | 158890873.7 | 158485569.2 | 1.49 | 94613351.36 | 94072580.38 | 1.51 | 1.68 | 0.75 |
| Salicylic acid | C00805 | 311763855.6 | 309450056.3 | 14.98 | 434728160.8 | 426531179 | 20.56 | 0.72 | -0.48 |
| Citramalic acid | C00815 | 90250389.06 | 90303078.25 | 2.06 | 48262829.85 | 48616634.13 | 3.11 | 1.87 | 0.9 |
| D-Xylose | C00181 | 220556309.5 | 227199038.2 | 8.29 | 379539720.1 | 376425089.6 | 4.57 | 0.58 | -0.78 |
| 2',4'-Dihydroxyacetophenone | C03663 | 5676614.59 | 5838443.46 | 10.07 | 12024225.08 | 12066763.49 | 4.62 | 0.47 | -1.08 |
| 2-Isopropylmalic acid | C02504 | 7016621.22 | 5110425.13 | 69.11 | 29089254.49 | 33855159.71 | 46.51 | 0.24 | -2.05 |
| Oxoadipic acid | C00322 | 1471981890 | 1578709234 | 31.13 | 590379764.7 | 700336992.3 | 57.05 | 2.49 | 1.32 |
| (Z)-4-Hydroxy-6-dodecenoic acid lactone | C03107 | 8814080.39 | 8679104.02 | 6.01 | 946284.59 | 956634.39 | 7.96 | 9.31 | 3.22 |
| myo-Inositol | C00137 | 253654895.1 | 295770742.1 | 33.21 | 97405399.43 | 93814324.6 | 63.98 | 2.6 | 1.38 |
| Fructose-1P | C10906 | 55166358.97 | 55274933.28 | 1.58 | 37844344.26 | 37478062.16 | 3.74 | 1.46 | 0.54 |
| trans-2-Hydroxycinnamate | C01772 | 5909436.67 | 5890051.17 | 3.6 | 4588530.14 | 4567087.28 | 2.73 | 1.29 | 0.36 |
| Vanylglycol | C05594 | 5484572.11 | 5259863.33 | 20.5 | 4099877.25 | 4116515.38 | 13.66 | 1.34 | 0.42 |
| Phenyllactate | C05607 | 10457907.63 | 10909633.72 | 16.1 | 2820304.52 | 3362813.3 | 38.9 | 3.71 | 1.89 |
| Tropate | C01456 | 15828911.68 | 15234561.61 | 11.12 | 4315985.6 | 5248299.78 | 51.53 | 3.67 | 1.87 |
| Norepinephrine | C00547 | 17978059.66 | 17975092.85 | 4 | 9278513.64 | 9132960.76 | 9.02 | 1.94 | 0.95 |
| Gallic acid | C01424 | 2718958.85 | 2690075.36 | 9.2 | 8294458.1 | 10772174.68 | 50.5 | 0.33 | -1.61 |
| Beta-Glycerophosphoric acid | C02979 | 9779876.56 | 9900712.01 | 3.47 | 3433173.21 | 3456656.74 | 10.18 | 2.85 | 1.51 |
| Dehydroascorbate | C05422 | 1147211805 | 1151175737 | 4.02 | 236402496.5 | 238732575.1 | 4.52 | 4.85 | 2.28 |
| Isocitric acid | C00311 | 5485160.35 | 5359303.27 | 40.03 | 10476833.12 | 10927781.81 | 26.62 | 0.52 | -0.93 |
| Guanidinosuccinic acid | C03139 | 464078374.3 | 394851462.9 | 38.23 | 861348780.2 | 877479564.5 | 5.27 | 0.54 | -0.89 |
| Gluconolactone | C00198 | 204777916.1 | 206256806.6 | 5.42 | 87254425.33 | 93487002.13 | 36.83 | 2.35 | 1.23 |
| Aesculetin | C09263 | 5688430.09 | 5917462.95 | 22.32 | 10020794 | 10098513.91 | 32.07 | 0.57 | -0.82 |
| Gluconic acid | C00257 | 3034270.04 | 2875239.9 | 19.9 | 5289890.56 | 4979689.49 | 16.08 | 0.57 | -0.8 |
| D-(+)-Glucose | C00293 | 61209648.32 | 59880442.14 | 22.5 | 40896300.52 | 42436709.56 | 7.09 | 1.5 | 0.58 |
| Alpha-D-Glucose | C00267 | 64343464.24 | 63755322.06 | 4.4 | 49476804.63 | 49794869.7 | 4.99 | 1.3 | 0.38 |
| D-Fructose | C00095 | 4452403138 | 4419008387 | 4.21 | 6326190944 | 6297661130 | 1.71 | 0.7 | -0.51 |
| Azelaic acid | C08261 | 17623746.09 | 17534817.08 | 4.31 | 15639189.3 | 15294572.07 | 6.44 | 1.13 | 0.17 |
| Xanthoxylin | C10726 | 5349890.39 | 5218881.82 | 23.59 | 10889760.38 | 10393259.3 | 11.72 | 0.49 | -1.03 |
| Vanillylmandelic acid | C05584 | 69203035.85 | 68155443.42 | 3.47 | 39009041.96 | 39208986.59 | 1.74 | 1.77 | 0.83 |
| L-Tryptophan | C00078 | 940598456.7 | 950111873.2 | 3.32 | 27993661.48 | 27973386.64 | 2.13 | 33.6 | 5.07 |
| N-Acetyl-L-phenylalanine | C03519 | 6226001.8 | 5611008.95 | 27.25 | 94826303.29 | 95711827.55 | 73.69 | 0.07 | -3.93 |
| (-)-Jasmonic acid | C08491 | 3089838.42 | 3080578.76 | 6.79 | 5646838.04 | 5570563.71 | 3.53 | 0.55 | -0.87 |
| Galactaric acid | C00879 | 20315739.81 | 20222962.71 | 8.19 | 12771944.06 | 12243093.16 | 19.19 | 1.59 | 0.67 |
| N-Acetyl-D-glucosamine | C00140 | 974768.27 | 1012631.52 | 8.66 | 5263133.75 | 5270362.96 | 5.56 | 0.19 | -2.43 |
| 6-Acetyl-D-glucose | C02655 | 11133446.91 | 11223146.3 | 2.88 | 6219094.8 | 6226386.16 | 2.47 | 1.79 | 0.84 |
| Methyl jasmonate | C11512 | 75194804.33 | 74561274.42 | 2.46 | 67993171.8 | 67908651.69 | 0.61 | 1.11 | 0.15 |
| Thymidine | C00214 | 67667243.63 | 82249179.59 | 46.47 | 21947286.49 | 15647474.56 | 73.62 | 3.08 | 1.62 |
| Citrinin | C16765 | 338099.48 | 333667.34 | 22.05 | 9231237.87 | 9090131.8 | 4.57 | 0.04 | -4.77 |
| Galactosylglycerol | C05401 | 63006827.85 | 62254133.07 | 4.11 | 116197339.4 | 113872023.8 | 6.3 | 0.54 | -0.88 |
| Shikimate 3-phosphate | C03175 | 30324407.48 | 30592999.26 | 8.31 | 11415882.66 | 11088353.39 | 13.33 | 2.66 | 1.41 |
| Galactose 1-phosphate | C00103 | 96101183.97 | 92754620.25 | 9.18 | 51602190.04 | 51308593.61 | 23.35 | 1.86 | 0.9 |
| (S)-Abscisic acid | C06082 | 84921874.25 | 87645055.77 | 11.11 | 21998664.78 | 13742552.54 | 66.61 | 3.86 | 1.95 |
| Apigenin | C01477 | 10669914.4 | 10497901.47 | 23.8 | 6792318.46 | 6940613.22 | 6.07 | 1.57 | 0.65 |
| Phloretin | C00774 | 28646307.85 | 29045905.7 | 3.61 | 2158248.41 | 2195644.61 | 5.74 | 13.27 | 3.73 |
| Pantetheine | C00831 | 10055936.51 | 10113108.28 | 2.9 | 10771574.07 | 10762180.87 | 3.75 | 0.93 | -0.1 |
| Hexadecanedioate | C19615 | 1254784737 | 1281684967 | 5.24 | 663503511.2 | 647670661.3 | 5.12 | 1.89 | 0.92 |
| Kaempferol | C05903 | 26235239.41 | 27042428.62 | 10.34 | 9507492.24 | 9053508.32 | 65.01 | 2.76 | 1.46 |
| 13(S)-HPOT | C04785 | 2815604.82 | 2811274.66 | 3.54 | 23682830.22 | 24346219.2 | 11.67 | 0.12 | -3.07 |
| 13S-hydroxyoctadecadienoic acid | C14762 | 7004518.46 | 6977646.56 | 5 | 6057976.39 | 5904293.58 | 9.28 | 1.16 | 0.21 |
| 12,13-DHOME | C14829 | 15218784.75 | 15411062.91 | 10.1 | 8569742.1 | 8829402.32 | 24.79 | 1.78 | 0.83 |
| 4-(beta-D-Glucosyloxy)benzoate | C03993 | 42168170.47 | 43008020.12 | 6.13 | 21110758.92 | 21143810.65 | 5.16 | 2 | 1 |
| Diosmetin | C10038 | 6233321.92 | 6275433.94 | 4.14 | 4423454.39 | 4398884.18 | 3.37 | 1.41 | 0.49 |
| 2-Methoxyestrone | C05299 | 8760038.36 | 8388790.05 | 16.63 | 893721.79 | 786277.65 | 27.74 | 9.8 | 3.29 |
| 4-Coumaroylshikimate | C02947 | 12318869.19 | 12456213.78 | 5.49 | 6673766.72 | 8526963.2 | 47.98 | 1.85 | 0.88 |
| EPA (d5) | C06428 | 72792799.18 | 72157533.14 | 8.13 | 21922694.66 | 22593128.3 | 10.24 | 3.32 | 1.73 |
| Pentahydroxyflavanone | C05911 | 22955756.27 | 23317981.12 | 8.71 | 17660976.98 | 17748221.79 | 6.58 | 1.3 | 0.38 |
| (-)-Epigallocatechin | C12136 | 367345500.3 | 378898742.9 | 9.11 | 200178372.2 | 203671478.1 | 5.91 | 1.84 | 0.88 |
| 9(S)-HPODE | C14827 | 17864639.57 | 17849927.73 | 1.24 | 15575557.92 | 16000473.59 | 8.99 | 1.15 | 0.2 |
| Isorhamnetin | C10084 | 51401222.61 | 51222620.94 | 72.31 | 11109438.17 | 7935828.54 | 89.51 | 4.63 | 2.21 |
| 15-Deoxy-d-12,14-PGJ2 | C14717 | 59414059.92 | 58278643.58 | 56.92 | 145102340.9 | 144286150.6 | 7.03 | 0.41 | -1.29 |
| 12-KETE | C14807 | 570969324.4 | 576929396.4 | 4.16 | 415729502.1 | 422308108.7 | 7 | 1.37 | 0.46 |
| Melibiitol | C05399 | 56089976.8 | 54068762.47 | 8.62 | 26817060.12 | 26584524.43 | 7.02 | 2.09 | 1.06 |
| 1-O-Vanilloyl-beta-D-glucose | C20470 | 78445734.17 | 80688016.53 | 9.59 | 196522867.2 | 192940584.6 | 11.61 | 0.4 | -1.32 |
| Prostaglandin A2 | C05953 | 91412294.01 | 89009626.35 | 11.06 | 32133206.56 | 31540438.43 | 28.57 | 2.84 | 1.51 |
| Delta-12-Prostaglandin J2 | C05958 | 6704870613 | 6857585921 | 9.17 | 2260190495 | 2239764256 | 5.77 | 2.97 | 1.57 |
| Dicumarol | C00796 | 12213435.45 | 12004258.34 | 9.06 | 4802630.05 | 5566974.78 | 31.06 | 2.54 | 1.35 |
| (5Z,9E,14Z)-(8xi,11R,12S)-11,12-epoxy-8-hydroxyicosa-5,9,14-trienoic Acid | C04849 | 105947563.4 | 103640080.5 | 5.9 | 40466468.01 | 41514787.48 | 11.04 | 2.62 | 1.39 |
| Dattelic acid | C10434 | 8977070.9 | 8911462.03 | 5.7 | 21853583.97 | 21786099.96 | 11.13 | 0.41 | -1.28 |
| 11,12-DiHETrE | C14774 | 32669481.72 | 32577946.83 | 10.47 | 24450562.78 | 25113676.97 | 7.35 | 1.34 | 0.42 |
| Erucic acid | C08316 | 2725985.8 | 2611337.39 | 23.48 | 20521876.33 | 21835065.42 | 18.06 | 0.13 | -2.91 |
| 3-Ketosucrose | C05731 | 105438304.8 | 99704257.75 | 13.45 | 33821382.76 | 33411813.03 | 20.43 | 3.12 | 1.64 |
| 3'-Ketolactose | C05403 | 38210877.96 | 34356706.09 | 42.21 | 21254985.56 | 22094291.5 | 9.58 | 1.8 | 0.85 |
| Fructose 1,6-bisphosphate | C00354 | 73983378.57 | 76078144.17 | 9.42 | 275992712.1 | 278830126.6 | 5.65 | 0.27 | -1.9 |
| Trehalose | C01083 | 13472196.22 | 13431426.96 | 1.85 | 5452700.02 | 5475211.09 | 10.57 | 2.47 | 1.3 |
| Melibiose | C05400 | 49354653.73 | 50142681.84 | 5.62 | 25728930.75 | 25765948.45 | 6.42 | 1.92 | 0.94 |
| 11-Dehydro-thromboxane B2 | C05964 | 13237465.2 | 11768469.6 | 22.88 | 3896695.34 | 3700752.08 | 17.89 | 3.4 | 1.76 |
| 19-Hydroxytabersonine | C11642 | 610993.03 | 587254.82 | 10.66 | 3007035.21 | 3207710.94 | 31.37 | 0.2 | -2.3 |
| (13E)-11a-Hydroxy-9,15-dioxoprost-13-enoic acid | C04654 | 3043425.88 | 2939978.62 | 13.98 | 7295995.52 | 7465068.4 | 4.89 | 0.42 | -1.26 |
| 13,14-Dihydro-15-keto-PGE2 | C04671 | 2567065.41 | 2582498.04 | 2.19 | 2079855.01 | 2068321.92 | 8.9 | 1.23 | 0.3 |
| Neochlorogenic acid | C17147 | 37636822.39 | 38381288.27 | 12.79 | 97098635.53 | 97396746.08 | 5.46 | 0.39 | -1.37 |
| Pioglitazone | C07675 | 8641500.44 | 2478823.25 | 125.08 | 80643228.89 | 79733627.42 | 7.42 | 0.11 | -3.22 |
| Rosmarinic acid | C01850 | 14549690.8 | 13072942.72 | 62.46 | 134596366.6 | 79735414.5 | 76.43 | 0.11 | -3.21 |
| Nervonic acid | C08323 | 29837864.6 | 31277035.36 | 18.11 | 18630284.38 | 18055223.68 | 32.11 | 1.6 | 0.68 |
| Dehydroepiandrosterone sulfate | C04555 | 25738977.12 | 24779747.74 | 13.27 | 18249602.01 | 18099625.45 | 15.42 | 1.41 | 0.5 |
| Curcumin | C10443 | 36862908.88 | 37146238.72 | 4.32 | 29163457.76 | 29178977.8 | 3.49 | 1.26 | 0.34 |
| (-)-Wikstromol | C10725 | 22219804.96 | 20875642.56 | 17.55 | 14903203.59 | 15260541.44 | 17.77 | 1.49 | 0.58 |
| Gardenoside | C09779 | 26118819.24 | 27213355.71 | 19.72 | 13637471.43 | 14147801.08 | 15.42 | 1.92 | 0.94 |
| Carvedilol | C06875 | 210487670.8 | 212846055 | 7.78 | 135454847.2 | 132136097 | 14.23 | 1.55 | 0.64 |
| Podofilox | C10874 | 14583808.5 | 14603390.17 | 9.67 | 8225477.35 | 8283953.34 | 4.84 | 1.77 | 0.83 |
| Quinacrine | C07339 | 18104142.68 | 17786018.06 | 9.05 | 28240129.54 | 28231807.15 | 6.85 | 0.64 | -0.64 |
| Neolinustatin | C08336 | 10236893.66 | 8271295.49 | 46.83 | 109145207 | 129400756.3 | 67.06 | 0.09 | -3.41 |
| Linustatin | C08333 | 4262681.77 | 4243905.21 | 10.25 | 15496346.66 | 15730584.03 | 19.54 | 0.28 | -1.86 |
| Puerarin | C10524 | 37378735.03 | 36896668.49 | 23.33 | 81605178.06 | 83618322.52 | 32.29 | 0.46 | -1.13 |
| Lamioside | C11645 | 2169046.17 | 2193551.63 | 6.01 | 1301927.71 | 1335340.36 | 14.28 | 1.67 | 0.74 |
| Cosmosiin | C04608 | 3223307.77 | 2812617.09 | 40.94 | 37791034.87 | 36661692.97 | 10.25 | 0.09 | -3.55 |
| 2',4,4',6'-Tetrahydroxychalcone 4'-O-glucoside | C16407 | 366798299.4 | 383852296.5 | 32 | 124900597 | 93096869.4 | 65.14 | 2.94 | 1.55 |
| Epigallocatechin gallate | C09731 | 7278142.91 | 7966183.93 | 34.01 | 2497021.98 | 2818808.17 | 34.26 | 2.91 | 1.54 |
| Folic acid | C00504 | 17581803.89 | 17572019.47 | 15.5 | 4926830.73 | 4871543.89 | 13.72 | 3.57 | 1.84 |
| Cyanidin 3-glucoside | C08604 | 244815016.7 | 259445696.5 | 33.7 | 63291921.56 | 60815149.36 | 58.54 | 3.87 | 1.95 |
| 6-Methoxyluteolin 7-rhamnoside | C10104 | 48215279.72 | 48131214.73 | 2.48 | 31718561.2 | 30258287.2 | 32.77 | 1.52 | 0.6 |
| Myricitrin | C10108 | 1475856.09 | 570552.21 | 104.11 | 27788347.18 | 27556327.07 | 40.68 | 0.05 | -4.23 |
| Iridodial glucoside tetraacetate | C11657 | 14956690.33 | 17542059.22 | 31.65 | 2930685.89 | 3380536.18 | 27.75 | 5.1 | 2.35 |
| Isochlorogenic acid b | C10468 | 21932161.43 | 21472291.08 | 7.87 | 11965882.76 | 11942429.29 | 9.73 | 1.83 | 0.87 |
| 10-Deoxygeniposide tetraacetate | C11664 | 49249005.76 | 45180921.28 | 99.7 | 282060041.5 | 253337995.3 | 34.49 | 0.17 | -2.52 |
| 7-Dehydrologanin tetraacetate | C11668 | 5658286.36 | 6302085.06 | 25.42 | 15472797.24 | 15733950.51 | 33.83 | 0.37 | -1.45 |
| Asperuloside tetraacetate | C11655 | 6696622.67 | 6661469.81 | 32.09 | 1464029.83 | 1327938.26 | 57.55 | 4.57 | 2.19 |
| Delphinidin 3-(6-p-coumaroyl)glucoside | C16370 | 9577049.7 | 9547045.21 | 6.08 | 5558622.17 | 5563956.76 | 11.59 | 1.72 | 0.78 |
| Kaempferol 3-O-rhamnoside-7-O-glucoside | C21854 | 8346780.51 | 9184688.45 | 27 | 628076.52 | 606703.34 | 14.41 | 13.29 | 3.73 |
| Neomycin | C01737 | 297585.24 | 207146.65 | 104.64 | 3082303.38 | 3166785.95 | 11.79 | 0.1 | -3.37 |
| Kaempferol 3-O-beta-D-glucosylgalactoside | C16490 | 5109834.34 | 4664714.62 | 40.99 | 3064989.17 | 3186076.57 | 13.74 | 1.67 | 0.74 |
| Quercetin 3-O-beta-D-glucosyl-(1->2)-beta-D-glucoside | C12667 | 5790994.01 | 5716148.35 | 38.91 | 1614425.57 | 1166390.1 | 57.16 | 3.59 | 1.84 |
| Delphin | C16312 | 6201420.73 | 6174960.78 | 7.28 | 4018071.23 | 3922382.22 | 6.73 | 1.54 | 0.63 |
| Lacto-N-tetraose | C06371 | 24977024.99 | 23507501.76 | 23.37 | 13119574.77 | 13266361.96 | 37.21 | 1.9 | 0.93 |
