# Supplementary material for: Response mechanism of carbon metabolism of Pinus massoniana to gradient high temperature and drought stress
Source: BMC Genomics. 2024 Feb 12;25:166. doi: 10.1186/s12864-024-10054-2 (PMC10860282; doi:10.1186/s12864-024-10054-2)
Supplement: Supplementary file 14 — Additional file 14. [file 12864_2024_10054_MOESM14_ESM.docx]

Table S17 KEGG enrichment analysis of T35CK vs T35Z differential metabolites.

| **name** | **KEGG** | **T35Z_Mean** | **T35Z_Median** | **T35Z_RSD** | **T35CK_Mean** | **T35CK_Median** | **T35CK_RSD** | **FC** | **log2FC** |
| --- | --- | --- | --- | --- | --- | --- | --- | --- | --- |
| 5-Valerolactone | C02240 | 77948046.66 | 78539622.95 | 35.22 | 26750341.12 | 22653542.92 | 79.61 | 2.91 | 1.54 |
| Succinic acid semialdehyde | C00232 | 2969895.85 | 2912809.43 | 4.41 | 2030606.14 | 1866308.63 | 20.83 | 1.46 | 0.55 |
| Isovaleric acid | C08262 | 6597882.26 | 6457006.38 | 11.35 | 10205611.18 | 10496363 | 9.9 | 0.65 | -0.63 |
| gamma-Aminobutyric acid | C00334 | 2823323503 | 2510919625 | 19.83 | 1518215893 | 1420286250 | 17.67 | 1.86 | 0.9 |
| 3-Methylthiopropanamine | C03354 | 1168107.51 | 824967.21 | 75.34 | 6592587.11 | 6717017.44 | 9.03 | 0.18 | -2.5 |
| 2-Phenylethanol | C05853 | 147370087.8 | 144265281.3 | 5.67 | 438414531.6 | 444441687.6 | 4.57 | 0.34 | -1.57 |
| Benzaldehyde | C00261 | 27169729.22 | 13851087.47 | 78.18 | 85160951.54 | 75907208.19 | 24.8 | 0.32 | -1.65 |
| Benzylamine | C15562 | 13897067.99 | 13865041.32 | 10.54 | 18020000.91 | 17571922.89 | 8.28 | 0.77 | -0.37 |
| p-Cresol | C01468 | 10691301.19 | 11551490.44 | 22.73 | 4102519.14 | 3401256.58 | 28.34 | 2.61 | 1.38 |
| m-Cresol | C01467 | 50041311.54 | 43543879.2 | 31.2 | 16841734.76 | 16508300.99 | 10.43 | 2.97 | 1.57 |
| 5-Methyl-2-furancarboxaldehyde | C11115 | 129727574.5 | 127498699.3 | 9.59 | 178236646.8 | 171278004 | 9.67 | 0.73 | -0.46 |
| Imidazole-4-acetaldehyde | C05130 | 7300538.41 | 7404435.38 | 4.9 | 3462015.31 | 3027924.77 | 30.45 | 2.11 | 1.08 |
| Uracil | C00106 | 200648122.1 | 201453965.3 | 1.53 | 248426173.7 | 248685856.8 | 3.84 | 0.81 | -0.31 |
| 3-Methyl-2-oxovaleric acid | C03465 | 29542589.3 | 30630598.49 | 20.95 | 18201414.42 | 17963954.42 | 21.48 | 1.62 | 0.7 |
| Creatinine | C00791 | 87243902.71 | 87454723.35 | 1.87 | 100741301 | 99887422.9 | 4.9 | 0.87 | -0.21 |
| Deoxyribose | C01801 | 19284403.63 | 19265896.86 | 6.57 | 12967630.42 | 13301266.84 | 5.77 | 1.49 | 0.57 |
| 5-Hydroxypentanoic acid | C02804 | 2589390.69 | 2593016.85 | 8.01 | 19195729.74 | 19071110.56 | 4.45 | 0.13 | -2.89 |
| L-Allothreonine | C05519 | 23395958.5 | 23798634.48 | 3.89 | 2075422.26 | 2064624.3 | 1.41 | 11.27 | 3.49 |
| Tyrosol | C06044 | 82568373.43 | 83860118.88 | 4.38 | 22879254.46 | 24147681.32 | 13.05 | 3.61 | 1.85 |
| N,N-Dimethylaniline | C02846 | 75893917.61 | 66666238.94 | 22.63 | 25796613.2 | 26151690.19 | 10.08 | 2.94 | 1.56 |
| Phenylethylamine | C05332 | 25108576.87 | 24927151.13 | 4.68 | 42643098.9 | 42548226.07 | 3.68 | 0.59 | -0.76 |
| Niacinamide | C00153 | 3906253.56 | 3899887.31 | 5.62 | 6772123.33 | 6709121.45 | 4.56 | 0.58 | -0.79 |
| 4-Hydroxybenzaldehyde | C00633 | 6431336.19 | 6414145.24 | 4.44 | 29604829.73 | 28261813.34 | 23.26 | 0.22 | -2.2 |
| Erythritol | C00503 | 33767038.77 | 34142879.6 | 2.73 | 22262906.85 | 22303697.18 | 2.65 | 1.52 | 0.6 |
| Taurine | C00245 | 26752967.7 | 14824022.08 | 72.21 | 94062541.87 | 104954263.6 | 35.02 | 0.28 | -1.81 |
| 1,2,3-Trihydroxybenzene | C01108 | 1197764575 | 1114635784 | 42.06 | 416981960.5 | 536738495 | 49.63 | 2.87 | 1.52 |
| Thymine | C00178 | 5623298.91 | 5632821.19 | 2.2 | 3798858.94 | 3771297.37 | 2.93 | 1.48 | 0.57 |
| Phloroglucinol | C02183 | 31729069.64 | 31668278.8 | 2.1 | 45167925.4 | 45416270.08 | 2.11 | 0.7 | -0.51 |
| Quinoline | C06413 | 813792335.9 | 782740035.3 | 11.21 | 323668348 | 328957409.8 | 7.81 | 2.51 | 1.33 |
| 1,1-Dimethylbiguanide | C07151 | 79684405.85 | 77421576.6 | 7.41 | 126601477.9 | 125353771.8 | 4.66 | 0.63 | -0.67 |
| Pipecolic acid | C00408 | 1249984853 | 1335318234 | 16.06 | 439401773.7 | 445325276.1 | 16.04 | 2.84 | 1.51 |
| L-Glutamic gamma-semialdehyde | C01165 | 155821820.6 | 155655739.7 | 2.98 | 54178507.78 | 47342685.7 | 55.07 | 2.88 | 1.52 |
| L-Isoleucine | C00407 | 1316979046 | 1333542971 | 3.01 | 299177704.6 | 295693587.4 | 3.25 | 4.4 | 2.14 |
| Creatine | C00300 | 8190800.55 | 8270430 | 2.94 | 5226851.24 | 5307289.74 | 5.41 | 1.57 | 0.65 |
| L-Ribulose | C00310 | 29268268.7 | 29599551.78 | 3.17 | 26317547.26 | 26132537.34 | 2.5 | 1.11 | 0.15 |
| D-Xylitol | C00379 | 15768085.79 | 15892619.05 | 29.65 | 8521080.98 | 7709974.88 | 23.72 | 1.85 | 0.89 |
| Perillyl alcohol | C02452 | 190134309.9 | 188969697.9 | 3.66 | 89254208.36 | 88452659.87 | 5.08 | 2.13 | 1.09 |
| (-)-3-Isothujone | C09906 | 4872916.51 | 5098816.07 | 9.41 | 4256274.66 | 4256368.2 | 1.97 | 1.14 | 0.2 |
| Phenelzine | C07430 | 14290379.6 | 14593747.95 | 3.98 | 23671196.27 | 23781456.92 | 2.38 | 0.6 | -0.73 |
| Gamma-terpinene | C09900 | 24952438.6 | 24989119.9 | 1.79 | 27956681.02 | 28063827.74 | 2.7 | 0.89 | -0.16 |
| p-Aminobenzoic acid | C00568 | 1073418.22 | 1059742.1 | 11.78 | 4628989.89 | 4553060.86 | 9.78 | 0.23 | -2.11 |
| 3,4-Dihydroxybenzaldehyde | C16700 | 43434672.18 | 42185488.34 | 6.99 | 26233097.34 | 25163863.27 | 17.35 | 1.66 | 0.73 |
| Phosphonoacetate | C05682 | 14281481.42 | 14835235.78 | 10.52 | 20987350.64 | 21081906.35 | 3.2 | 0.68 | -0.56 |
| 5-Hydroxymethyluracil | C03088 | 88540508.66 | 85870533.39 | 9.57 | 111949784.4 | 108693310.1 | 12.16 | 0.79 | -0.34 |
| trans-trans-Muconic acid | C02480 | 38270406.87 | 38306527.13 | 4.64 | 29642636.15 | 29377488.63 | 3.85 | 1.29 | 0.37 |
| N-methyl-L-glutamic Acid | C01046 | 40545711.19 | 40258329.69 | 3.84 | 9538472.49 | 9352243.2 | 6.56 | 4.25 | 2.09 |
| Tryptophanol | C00955 | 13808269.33 | 13867657.41 | 5.74 | 6230651.55 | 6181850.75 | 9.46 | 2.22 | 1.15 |
| Anabasine | C06180 | 22653851.86 | 22277481.65 | 12.01 | 16105928.76 | 15461041.47 | 11.48 | 1.41 | 0.49 |
| Acetylcholine | C01996 | 5571666.06 | 5455286.53 | 8.73 | 4737065.08 | 4659580.01 | 7.68 | 1.18 | 0.23 |
| (2R,5S)-2,5-Diaminohexanoate | C05161 | 99065.9 | 104248.2 | 41.46 | 6123249.88 | 5950816.47 | 10.96 | 0.02 | -5.95 |
| Methyloxaloacetate | C06030 | 83374635.86 | 84262020.73 | 7.22 | 33170846.58 | 32649359.31 | 66.85 | 2.51 | 1.33 |
| Coumarin | C05851 | 38154285.56 | 37638307.18 | 4.37 | 66958831.83 | 67003894.58 | 9.53 | 0.57 | -0.81 |
| L-Lysine | C00047 | 7666442.43 | 7375566.57 | 9.67 | 17337244.73 | 16056851.04 | 21.6 | 0.44 | -1.18 |
| L-Glutamine | C00064 | 717292112 | 708072409.8 | 33.42 | 290573558 | 217750483.8 | 56.72 | 2.47 | 1.3 |
| L-Glutamic acid | C00025 | 2510050919 | 2549767686 | 4.7 | 2205657558 | 2202480857 | 2.57 | 1.14 | 0.19 |
| 3,4-Dihydro-2H-1-benzopyran-2-one | C02274 | 27730681.49 | 27602670.2 | 11.02 | 14844607.27 | 13736156.97 | 14.85 | 1.87 | 0.9 |
| Estragole | C10452 | 329348009.2 | 312901024.7 | 14.95 | 243429840.1 | 242312425.8 | 1.66 | 1.35 | 0.44 |
| Phthalic acid | C01606 | 81629212.85 | 80406611.65 | 5.61 | 46481835.61 | 45479344.91 | 9.37 | 1.76 | 0.81 |
| L-2-Hydroxyglutaric acid | C03196 | 16109394.69 | 16156047.89 | 1.82 | 13753719.63 | 13747249.85 | 2.26 | 1.17 | 0.23 |
| D-Lyxose | C00476 | 35212356.54 | 35015912.91 | 7.22 | 26998654.36 | 27111593.73 | 5 | 1.3 | 0.38 |
| Homogentisic acid | C00544 | 10254316.31 | 10309136.38 | 4.6 | 17267305.06 | 17178748.44 | 2.36 | 0.59 | -0.75 |
| D-Ribose | C00121 | 9616008.97 | 9749264.98 | 3.07 | 12701304.47 | 12625455.38 | 4.98 | 0.76 | -0.4 |
| Guanine | C00242 | 36994537.58 | 38695018.62 | 20.61 | 19500841.35 | 20500834.77 | 16.85 | 1.9 | 0.92 |
| p-Hydroxyphenylacetic acid | C00642 | 4749387.5 | 1871281.02 | 100.99 | 20908525.52 | 23265193.26 | 41.46 | 0.23 | -2.14 |
| 1,2-Epoxy-p-menth-8-ene | C07271 | 29388830.08 | 28650127.26 | 21.43 | 14189326.16 | 12634058.64 | 34.22 | 2.07 | 1.05 |
| (-)-cis-Carveol | C11395 | 61467663.48 | 60959077.59 | 2.15 | 42703165.14 | 42858582.01 | 4.53 | 1.44 | 0.53 |
| Imidazol-5-yl-pyruvate | C03277 | 7053610.28 | 7057760.08 | 3.23 | 8361255.08 | 8327023.47 | 1.26 | 0.84 | -0.25 |
| 2,3-Butanediol | C00265 | 190862211 | 191016198.6 | 2.28 | 139750678.6 | 117579402.7 | 25.37 | 1.37 | 0.45 |
| Pelargonic acid | C01601 | 5243407.68 | 5185309.85 | 12.11 | 4164355.08 | 4100740.64 | 7.05 | 1.26 | 0.33 |
| 4,5-Dihydroorotic acid | C00337 | 40427227.53 | 40536302.57 | 1.98 | 36820195.15 | 36784428.52 | 1.09 | 1.1 | 0.13 |
| Indoleacetaldehyde | C00637 | 6104755.24 | 6141990.57 | 6.27 | 3525318.49 | 3539212.05 | 18.33 | 1.73 | 0.79 |
| 3D-3,5_4-Trihydroxycyclohexane-1,2-dione | C04287 | 3170923.37 | 3135417.26 | 4.23 | 5624215.06 | 5512443.23 | 19.56 | 0.56 | -0.83 |
| L-Carnitine | C00318 | 5313136.85 | 4888464.63 | 53.95 | 23966221.31 | 28884082.17 | 34.7 | 0.22 | -2.17 |
| Nicotine | C00745 | 7341749.71 | 7395801.7 | 2.38 | 21799872.37 | 21782156.26 | 2.57 | 0.34 | -1.57 |
| cis-1,2-Dihydronaphthalene-1,2-diol | C04314 | 9103603.85 | 9184449.25 | 3.37 | 3966998.07 | 3150170.39 | 43.37 | 2.29 | 1.2 |
| 2-Deoxystreptamine | C02627 | 52328935.38 | 51254479.03 | 17.94 | 26854786.99 | 24564691.58 | 30.99 | 1.95 | 0.96 |
| 2-Phenylethyl acetate | C12303 | 44725852.89 | 44663223.8 | 2.62 | 25292839.99 | 25611125.62 | 4.83 | 1.77 | 0.82 |
| 7-Methylxanthine | C16353 | 13746115.38 | 13839093.39 | 1.85 | 15133730.77 | 15155455.88 | 1.77 | 0.91 | -0.14 |
| Desaminotyrosine | C01744 | 10798285.22 | 11558488.35 | 18.6 | 6618122.61 | 6751178.9 | 7.58 | 1.63 | 0.71 |
| D-synephrine | C01869 | 5831709.58 | 5776226.6 | 2.91 | 6533448.15 | 6504212.9 | 2.67 | 0.89 | -0.16 |
| (R)-4-Hydroxymandelate | C05343 | 5767848.96 | 5900738.12 | 6.77 | 3054037.19 | 2835113.03 | 19.83 | 1.89 | 0.92 |
| (1S,4R)-1-Hydroxy-2-oxolimonene | C11937 | 16802967.43 | 16843262.38 | 7.93 | 60613578.96 | 61432149.69 | 4.04 | 0.28 | -1.85 |
| Nalpha-Methylhistidine | C03298 | 27990668.19 | 27957056.88 | 2.76 | 18488344.17 | 18591357.35 | 4.92 | 1.51 | 0.6 |
| (S)-4-Hydroxymandelate | C03198 | 210102738.2 | 214730293.2 | 9.62 | 124028771.3 | 127076849.1 | 12.25 | 1.69 | 0.76 |
| 8-Amino-7-oxononanoate | C01092 | 44064314.61 | 44316845.55 | 3.19 | 9832719.37 | 9835328.5 | 2.35 | 4.48 | 2.16 |
| 2-Biphenylol | C02499 | 62814292.12 | 56850351.81 | 29.71 | 16170159.93 | 16059971.28 | 7.5 | 3.88 | 1.96 |
| Levetiracetam | C07841 | 40116019.75 | 40396309.75 | 8.88 | 18866327.9 | 18575931.21 | 5.96 | 2.13 | 1.09 |
| Menadione | C05377 | 26061318.88 | 26432486.5 | 7.52 | 13599177.12 | 13038719.4 | 15.72 | 1.92 | 0.94 |
| (2S,5S)-trans-Carboxymethylproline | C17366 | 72191541.36 | 66104694.58 | 21.19 | 37288131.52 | 35387329.45 | 13.38 | 1.94 | 0.95 |
| N-Acetylleucine | C02710 | 8732042.9 | 8729284.63 | 4.21 | 6790250.35 | 6816109.41 | 7.86 | 1.29 | 0.36 |
| Citrulline | C00327 | 43408713.36 | 43286268.36 | 3.41 | 21217095.15 | 22149364.76 | 16.19 | 2.05 | 1.03 |
| D-Galacturonolactone | C06430 | 26217226.79 | 25728895.93 | 6.38 | 52863507.95 | 53195906.82 | 3.63 | 0.5 | -1.01 |
| Herniarin | C09268 | 35781361.05 | 39381823.17 | 32.51 | 3520013.45 | 3530152.61 | 4.37 | 10.17 | 3.35 |
| L-Bornesitol | C03660 | 105942510.7 | 105050490 | 7.29 | 21163554.68 | 16391408.21 | 43.13 | 5.01 | 2.32 |
| Methylisoeugenol | C10478 | 11459525.61 | 11410514.93 | 1.38 | 10828434.81 | 10873373.21 | 4.03 | 1.06 | 0.08 |
| 5-Deoxy-D-glucuronate | C16737 | 58727748.21 | 58488027.56 | 2.32 | 40440299.02 | 40416150.68 | 2.22 | 1.45 | 0.54 |
| Geranyl acetate | C09861 | 932789.59 | 969322.87 | 22.88 | 2873771.83 | 1677247.35 | 67.75 | 0.32 | -1.62 |
| D-Psicose | C06468 | 41064692.8 | 40720661.32 | 8.55 | 105719068.2 | 106753556.1 | 5.9 | 0.39 | -1.36 |
| 1D-chiro-Inositol | C19891 | 6267538.29 | 6262202.51 | 3.46 | 13872794.78 | 13847569.4 | 1.54 | 0.45 | -1.15 |
| Coniferyl alcohol | C00590 | 23702935.63 | 23653366.49 | 3.22 | 33515157.77 | 30960192.91 | 13.99 | 0.71 | -0.5 |
| (S)-beta-Tyrosine | C21308 | 36297.48 | 37619.05 | 62.7 | 2899906.02 | 2943233.52 | 8.57 | 0.01 | -6.32 |
| Hydroxyphenyllactic acid | C03672 | 126267949 | 126746592 | 1.67 | 157005018.1 | 156561355.3 | 2.01 | 0.8 | -0.31 |
| 5-Oxo-1,2-campholide | C02952 | 29147688.27 | 33217581.17 | 28.65 | 14546912.51 | 14525663.18 | 27.68 | 2 | 1 |
| Sorbitol | C00794 | 22419884.46 | 22948877.03 | 4.9 | 30492374.5 | 31313770.25 | 9.64 | 0.74 | -0.44 |
| Choline sulfate | C00919 | 64099813.42 | 64177586.93 | 3.88 | 76177604.93 | 76187855.5 | 2.08 | 0.84 | -0.25 |
| Phosphorylcholine | C00588 | 20324535.73 | 20657912.98 | 5.45 | 66935430.58 | 70323300.16 | 29.09 | 0.3 | -1.72 |
| Sebacic acid | C08277 | 4189680.48 | 4448313.94 | 41.05 | 12736567.68 | 12721521.55 | 3.17 | 0.33 | -1.6 |
| Homocitrulline | C02427 | 40969491.83 | 39902606.57 | 6.52 | 147336229.4 | 148502716 | 2.47 | 0.28 | -1.85 |
| Homoisocitrate | C05662 | 89371305.55 | 85541343.15 | 14.81 | 121816099.4 | 126799402.4 | 11.04 | 0.73 | -0.45 |
| N6-Acetyl-L-lysine | C02727 | 58435194.16 | 52959342.76 | 27.99 | 24494091.55 | 24667524.78 | 12.67 | 2.39 | 1.25 |
| Homo-L-arginine | C01924 | 85369090.3 | 80417121.4 | 11.63 | 56349888.8 | 52343437.49 | 25.25 | 1.51 | 0.6 |
| Glycylleucine | C02155 | 9036300.6 | 9113769.15 | 2.72 | 3946489.08 | 3942311.09 | 34.94 | 2.29 | 1.2 |
| N-Acetylglutamic acid | C00624 | 580943.3 | 583643.68 | 8.09 | 4312974.01 | 4263003.93 | 3.22 | 0.13 | -2.89 |
| Diaminopimelic acid | C00666 | 8801511.5 | 8795197.37 | 3.06 | 20678619.45 | 20516673.22 | 2.41 | 0.43 | -1.23 |
| 4-Methoxy-2,2'-bipyrrole-5-carbaldehyde | C21570 | 80116940.72 | 82060421.05 | 17.26 | 5570817.24 | 5683246.91 | 16.81 | 14.38 | 3.85 |
| Kynurenic acid | C01717 | 6069180.11 | 5676640.27 | 55.5 | 18669191.9 | 18682420.85 | 29.02 | 0.33 | -1.62 |
| 6-Methoxymellein | C02381 | 32578568.12 | 32614923.29 | 1.58 | 38480804.35 | 38593214.52 | 2.88 | 0.85 | -0.24 |
| Carbendazim | C10897 | 1153926.06 | 1255780.19 | 26.89 | 2041553.46 | 2039895.46 | 5.59 | 0.57 | -0.82 |
| Sinapyl alcohol | C02325 | 15547960.71 | 15813239.35 | 13.91 | 53917275.53 | 67315736.2 | 49.61 | 0.29 | -1.79 |
| Myristicin | C10480 | 9004919.44 | 9206706.92 | 11.21 | 6367512.34 | 6443185.44 | 10.02 | 1.41 | 0.5 |
| Methoxamine | C07513 | 4334364.94 | 4355600.07 | 1.63 | 2643781.24 | 2613661.41 | 3.38 | 1.64 | 0.71 |
| Leucodopachrome | C05604 | 480221.56 | 442492.37 | 42.76 | 3568556.65 | 3669092.11 | 45.83 | 0.13 | -2.89 |
| 2-Amino-2-deoxy-D-gluconate | C03752 | 19706265.15 | 16036027.03 | 30.09 | 4802343.65 | 4624105.66 | 26.97 | 4.1 | 2.04 |
| (-)-Bornesitol | C03659 | 300128918.5 | 299449843.3 | 2.13 | 607266779.7 | 596508974.8 | 6.67 | 0.49 | -1.02 |
| Dodecanoic acid | C02679 | 38453265.43 | 37134710.05 | 13.45 | 7491722.24 | 7806414.12 | 16.18 | 5.13 | 2.36 |
| Thiabendazole | C07131 | 1335618857 | 1368286455 | 8.36 | 974879011.7 | 966834598.7 | 11.15 | 1.37 | 0.45 |
| Caryophyllene alpha-oxide | C16908 | 3843231.3 | 1177113.47 | 177.76 | 55616920.5 | 51716745.17 | 18.25 | 0.07 | -3.86 |
| 4-Hydroxy-3-(3-methyl-2-butenyl)acetophenone | C10702 | 20340963.73 | 20277088.85 | 6.56 | 28438162.5 | 28398728.82 | 4.38 | 0.72 | -0.48 |
| Apiole | C10429 | 7120315.75 | 7172620.07 | 4.94 | 7870899.93 | 7863625.58 | 5.38 | 0.9 | -0.14 |
| Pantothenol | C05944 | 2346455.79 | 2365757.34 | 8.02 | 3801470.84 | 3843333.57 | 6.79 | 0.62 | -0.7 |
| Sinapate | C00482 | 3132100.92 | 3003643.08 | 62.21 | 7249829.77 | 7319501.57 | 19.14 | 0.43 | -1.21 |
| Ibuprofen | C01588 | 18516643.28 | 18282405.95 | 17.43 | 25114581.51 | 23530218.72 | 15.83 | 0.74 | -0.44 |
| 3-[(1-Carboxyvinyl)oxy]benzoate | C20772 | 13346111.11 | 12044792.23 | 33.85 | 3076641.36 | 3861488.81 | 52.43 | 4.34 | 2.12 |
| L-Kynurenine | C00328 | 12678346.48 | 13226788.35 | 13.03 | 4001549.98 | 3985689.23 | 2.39 | 3.17 | 1.66 |
| Sinapoyl aldehyde | C05610 | 2797687.8 | 2699357.76 | 21.7 | 5464965.3 | 5420967.93 | 11.47 | 0.51 | -0.97 |
| trans-Isoasarone | C17846 | 10506371.05 | 9024951.7 | 35.21 | 17689741.66 | 18519496.08 | 24.62 | 0.59 | -0.75 |
| N-Acetyldemethylphosphinothricin | C17949 | 5926353.84 | 5695292.61 | 20.09 | 39311873.29 | 43806063.93 | 32.56 | 0.15 | -2.73 |
| (+)-7-Isojasmonic acid | C16317 | 16682458.69 | 16388975.79 | 8.19 | 6340544.35 | 4327001.94 | 78.11 | 2.63 | 1.4 |
| Dethiobiotin | C01909 | 3223776.83 | 3017526.85 | 55.33 | 9867372.72 | 9166504.26 | 16.72 | 0.33 | -1.61 |
| Glutethimide | C07489 | 46910533.64 | 49564020.3 | 62.29 | 9089198.45 | 7289774.23 | 61.87 | 5.16 | 2.37 |
| Cis-zeatin | C00371 | 16981094.06 | 17020179.59 | 2.01 | 7471616.72 | 7432743.28 | 2.86 | 2.27 | 1.18 |
| 2-trans,6-trans-Farnesal | C03461 | 35194137.29 | 35777846.24 | 3.49 | 255506888.4 | 247197447.7 | 7.14 | 0.14 | -2.86 |
| Cerulenin | C12058 | 4353513.03 | 4058761.18 | 19.01 | 12754164.8 | 12792370.95 | 6.33 | 0.34 | -1.55 |
| Prephenate | C00254 | 1181865.77 | 1185591.92 | 15.09 | 4697239.08 | 5752628.86 | 51.88 | 0.25 | -1.99 |
| Genipin | C09780 | 25800853.23 | 25456918.15 | 5.44 | 19044840.43 | 18272587.77 | 11.33 | 1.35 | 0.44 |
| Deoxycytidine | C00881 | 15436572.97 | 8573660.55 | 141.95 | 62611793.07 | 69180422.41 | 39.01 | 0.25 | -2.02 |
| Myristoleic acid | C08322 | 3186064.49 | 3205105.78 | 3.76 | 18280696.17 | 17870886.92 | 6.83 | 0.17 | -2.52 |
| Benz[a]anthracene | C14317 | 10805886.77 | 9629043.28 | 59.18 | 22989283.68 | 22707962.6 | 12.9 | 0.47 | -1.09 |
| Deoxyuridine | C00526 | 30708799.68 | 31063238.88 | 3.53 | 44106222.7 | 46821079.51 | 15.32 | 0.7 | -0.52 |
| Myristic acid | C06424 | 5122071.09 | 3571636.46 | 76.81 | 19549412.2 | 20256250.91 | 17.83 | 0.26 | -1.93 |
| 6-Hydroxymelatonin | C05643 | 19729728.42 | 17992332.83 | 24.88 | 11902068.45 | 11962860.57 | 5.71 | 1.66 | 0.73 |
| Ubiquinone-1 | C00399 | 21716377.42 | 20458466.39 | 23.92 | 41116785.19 | 41325334.03 | 6.2 | 0.53 | -0.92 |
| Alantolactone | C09289 | 1608512.04 | 1596145.36 | 13.42 | 43202541.91 | 43253094.81 | 4.14 | 0.04 | -4.75 |
| Confertifolin | C09376 | 11709113.05 | 11466289.66 | 5.2 | 5505188.4 | 5492643.45 | 3.59 | 2.13 | 1.09 |
| Equol | C14131 | 72517942.46 | 66291505.33 | 16.6 | 57640252.9 | 58095224.44 | 2.69 | 1.26 | 0.33 |
| N-Demethylindolmycin | C21443 | 7514493.99 | 7594641.87 | 4.6 | 3019433.44 | 2987657.32 | 3.22 | 2.49 | 1.32 |
| N1-Acetylspermine | C02567 | 135111.69 | 53039.96 | 99.58 | 3672368.57 | 3638576.08 | 7.5 | 0.04 | -4.76 |
| gamma-L-Glutamyl-L-cysteine | C00669 | 9137035.29 | 9283465.53 | 13.65 | 16013044.71 | 16161202 | 9.44 | 0.57 | -0.81 |
| Abscisic alcohol | C13456 | 2196919.47 | 2188781.84 | 3.46 | 1768213.68 | 1759387.36 | 2.75 | 1.24 | 0.31 |
| Methaqualone | C07560 | 7085429.5 | 8051774.3 | 23.41 | 3856277.2 | 3636795.37 | 21.32 | 1.84 | 0.88 |
| 5'-Deoxyadenosine | C05198 | 534335.46 | 547856.65 | 8.12 | 7301569.45 | 7614837.84 | 8.64 | 0.07 | -3.77 |
| Benzo[k]fluoranthene | C14321 | 47393809.31 | 47694406.38 | 3.57 | 12538052.39 | 12193616.76 | 13.17 | 3.78 | 1.92 |
| Nandrolone | C07254 | 1683387.31 | 1640475.63 | 6.74 | 5718456.31 | 5704342.1 | 2.59 | 0.29 | -1.76 |
| Glycerophosphocholine | C00670 | 79678914.99 | 86562297.66 | 28.01 | 163307037.4 | 163068270.2 | 5.95 | 0.49 | -1.04 |
| (9Z,11E,13E)-Octadecatrienoic acid | C08315 | 80545712.58 | 77307629.72 | 13.14 | 15192361.9 | 15260035.64 | 7.74 | 5.3 | 2.41 |
| Parthenin | C09523 | 43400118.74 | 44094814 | 9.16 | 18895606.57 | 18802376.02 | 4.95 | 2.3 | 1.2 |
| Qing Hau Sau | C09538 | 27253893.3 | 27173458.44 | 5.47 | 13893577.18 | 15657204.49 | 33.2 | 1.96 | 0.97 |
| Xanthoxic acid | C13454 | 15650777.65 | 15359004.85 | 12.58 | 84234456.25 | 85499799.74 | 10.92 | 0.19 | -2.43 |
| Adenosine | C00212 | 1804836651 | 1761304260 | 8.87 | 4644736764 | 5303903275 | 27.98 | 0.39 | -1.36 |
| (S)-Coclaurine | C06161 | 46065633.73 | 47334065.06 | 10.69 | 73245294.93 | 73291489.94 | 5.56 | 0.63 | -0.67 |
| (R)-Coclaurine | C06349 | 13628718.59 | 13947870.05 | 5.23 | 1043746.68 | 1114512.39 | 39.96 | 13.06 | 3.71 |
| Androstenedione | C00280 | 1142334.46 | 791525.86 | 76.98 | 5894677.25 | 5881562.75 | 2.33 | 0.19 | -2.37 |
| Norizalpinin | C10044 | 36652936.65 | 36710971.59 | 4.55 | 71813968.13 | 71680569.11 | 2.72 | 0.51 | -0.97 |
| Genistein | C06563 | 17633033.14 | 16776436.24 | 12.03 | 22170327.84 | 21993263.74 | 8.7 | 0.8 | -0.33 |
| All-trans-13,14-dihydroretinol | C15492 | 601178252.8 | 608022425.5 | 5.17 | 85786490.34 | 86378874.43 | 5.99 | 7.01 | 2.81 |
| Etiocholanedione | C03772 | 16181395.77 | 15717629.97 | 13.11 | 31092891.57 | 27597809.99 | 44.47 | 0.52 | -0.94 |
| Thienamycin | C06664 | 14017883.44 | 14124939.52 | 5.31 | 11734171.42 | 11617494.77 | 6.26 | 1.19 | 0.26 |
| Apiforol | C12124 | 4878372.95 | 4681565.24 | 22.34 | 27011708.43 | 27047261.48 | 15.14 | 0.18 | -2.47 |
| 5a-Androstane-3b,17b-diol | C12525 | 8802200.12 | 8677220.61 | 3.25 | 3509481.4 | 3529259.04 | 2.34 | 2.51 | 1.33 |
| N2-gamma-Glutamylglutamine | C05283 | 8492694.01 | 8297097.27 | 14.79 | 12309633.94 | 12478638.17 | 5.51 | 0.69 | -0.54 |
| Stearidonic acid | C16300 | 404724644.5 | 315100263.5 | 81.08 | 53850411.18 | 36063842.67 | 60.23 | 7.52 | 2.91 |
| N6-(L-1,3-Dicarboxypropyl)-L-lysine | C00449 | 6308687.79 | 6364993.66 | 8.02 | 3496167.19 | 3037550.93 | 34.58 | 1.8 | 0.85 |
| Cyclopeptine | C20579 | 5323501.02 | 5333034.72 | 6.88 | 6330714.17 | 6305551.1 | 5.92 | 0.84 | -0.25 |
| 9-Riburonosyladenine | C11501 | 2930622.34 | 3155630.18 | 32.84 | 8521186.51 | 8084217.2 | 13.14 | 0.34 | -1.54 |
| 1-Methyladenosine | C02494 | 3495799 | 4003645.35 | 50.92 | 10640141.41 | 10599449.19 | 2.52 | 0.33 | -1.61 |
| Guanosine | C00387 | 402836224.5 | 404597150.5 | 5.34 | 539884517.4 | 533619126.8 | 4.33 | 0.75 | -0.42 |
| (R,S)-Coclaurine | C06348 | 101261331.8 | 99707027.07 | 7.21 | 159677404.8 | 160198368.6 | 3.31 | 0.63 | -0.66 |
| N1,N12-Diacetylspermine | C03413 | 1131649.85 | 1073088.14 | 33.72 | 18582696.72 | 18331831.17 | 7.31 | 0.06 | -4.04 |
| Fisetin | C10041 | 161006225 | 166312133 | 11.12 | 54757675.83 | 54827163.19 | 11.43 | 2.94 | 1.56 |
| Glutathione amide | C19689 | 75195845.95 | 76713301.4 | 9.36 | 115901161.4 | 114071016.8 | 4.49 | 0.65 | -0.62 |
| Eriodictyol | C05631 | 68641530.96 | 66502628.16 | 12.17 | 21145730.33 | 21573863.7 | 9.16 | 3.25 | 1.7 |
| Dehydroepiandrosterone | C01227 | 3693541.4 | 3959095.43 | 20.22 | 21672846.1 | 23877139.85 | 54.93 | 0.17 | -2.55 |
| Aurin | C14213 | 67301266.6 | 66935749.81 | 2.47 | 60204863.28 | 59935710.71 | 1.05 | 1.12 | 0.16 |
| Epicatechin | C09727 | 4108056949 | 4046914244 | 4.63 | 2764006846 | 2745374143 | 7.71 | 1.49 | 0.57 |
| Dihydrotestosterone | C03917 | 20537360.42 | 20783722 | 5.72 | 8907137.97 | 10132713.16 | 34.22 | 2.31 | 1.21 |
| Eicosadienoic acid | C16525 | 18341041.25 | 19831428.67 | 26.46 | 31139603.57 | 29923977.51 | 18.87 | 0.59 | -0.76 |
| 9(S)-HPOT | C16321 | 2026575.48 | 2069138.55 | 5.1 | 7270695.8 | 7334818.88 | 6.32 | 0.28 | -1.84 |
| Nivalenol | C06080 | 95277509.18 | 93543692.57 | 10.49 | 154005415.7 | 160302971.6 | 20.59 | 0.62 | -0.69 |
| Prunasin | C00844 | 702646880.2 | 717952502.2 | 9.36 | 22577312.37 | 23815900.95 | 20.79 | 31.12 | 4.96 |
| 5'-Methylthioadenosine | C00170 | 2062394.03 | 1935200.95 | 30.37 | 31263853.33 | 31188445.36 | 60.61 | 0.07 | -3.92 |
| Cafestol | C09066 | 7880334.52 | 8039485.14 | 5.33 | 37445499.96 | 39549243.85 | 41.54 | 0.21 | -2.25 |
| all-trans-5,6-Epoxyretinoic acid | C16680 | 21488101.6 | 26460097.79 | 37.3 | 88102745.11 | 89149658.75 | 7.42 | 0.24 | -2.04 |
| Palmitoylethanolamide | C16512 | 11932163.64 | 11286111.27 | 21.85 | 26019615.8 | 25247702.33 | 9.47 | 0.46 | -1.12 |
| 5-Nitro-2-(3-phenylpropylamino)benzoic acid | C13705 | 8269701.97 | 8306370.9 | 5.34 | 6226932.43 | 6118185 | 10.53 | 1.33 | 0.41 |
| Isotretinoin | D00348 | 2274434.75 | 2517551.21 | 37.77 | 7001493.04 | 6829530.53 | 18.53 | 0.32 | -1.62 |
| Kaempferide | C10098 | 6225117.35 | 6490723.44 | 11.11 | 10711191.95 | 10619793.2 | 7.33 | 0.58 | -0.78 |
| all-trans-Retinoic acid | C00777 | 12777580 | 12343648.31 | 11.08 | 8875569.53 | 9153875.1 | 10.2 | 1.44 | 0.53 |
| Sphinganine | C00836 | 1541620837 | 1349503008 | 27.52 | 228664466.6 | 284007978 | 50.03 | 6.74 | 2.75 |
| Tricetin | C10192 | 21097683.43 | 22299907.44 | 15.18 | 10374350.73 | 9529326.65 | 24.15 | 2.03 | 1.02 |
| 11b-Hydroxyandrost-4-ene-3,17-dione | C05284 | 13400430.71 | 13934401.74 | 16.35 | 8732566.69 | 7542809.7 | 27.25 | 1.53 | 0.62 |
| Quercetin | C00389 | 21753929.36 | 22317183.19 | 6.68 | 7367319.45 | 7111518.12 | 64.14 | 2.95 | 1.56 |
| cis-Dihydroquercetin | C12316 | 527832954.5 | 521097442.3 | 7.01 | 449842467.4 | 455072499.7 | 6.63 | 1.17 | 0.23 |
| Arachidonic acid | C00219 | 20116965.82 | 21313318.18 | 14.18 | 10851729.04 | 11194166.29 | 8.48 | 1.85 | 0.89 |
| 11alpha,17beta-Dihydroxyandrost-4-en-3-one | C15306 | 95457105.21 | 95033581.09 | 2.75 | 12902156.86 | 15079145 | 38.64 | 7.4 | 2.89 |
| Bisdemethoxycurcumin | C17743 | 230372487.1 | 263555227.4 | 34.78 | 78617482.96 | 77669479.81 | 7.96 | 2.93 | 1.55 |
| Alprazolam | C06817 | 449917.37 | 460478.22 | 42.77 | 9281433.73 | 8303443.69 | 27.88 | 0.05 | -4.37 |
| beta-D-Galactosyl-(1->4)-L-rhamnose | C19758 | 23413928.57 | 20751940.92 | 31.2 | 4839109.59 | 5382171.05 | 31.17 | 4.84 | 2.27 |
| Phenylbutazone | C07440 | 52550740.77 | 53500974.22 | 11.25 | 2210547.09 | 2230287.31 | 19.97 | 23.77 | 4.57 |
| 2,3-Dinor-8-iso prostaglandin F2alpha | C14794 | 28634797 | 28848663.75 | 3.48 | 12522454.87 | 11951099.47 | 8.81 | 2.29 | 1.19 |
| Aflatoxin B1 | C06800 | 14299866.04 | 14199984.25 | 6.84 | 33916699.93 | 34036415.27 | 10.55 | 0.42 | -1.25 |
| Pergolide | C07425 | 8765096.97 | 8744209.9 | 5.33 | 6985951.29 | 6958955.97 | 9.66 | 1.25 | 0.33 |
| 7-Methylcapillarisin | C17785 | 17228713.13 | 17587340.01 | 7.45 | 13912567.13 | 13880450.73 | 3.44 | 1.24 | 0.31 |
| Progesterone | C00410 | 16201813.8 | 16156472.2 | 17.81 | 141519852.7 | 138414013 | 12.9 | 0.11 | -3.13 |
| 5a-Pregnane-3,20-dione | C03681 | 11229814.73 | 11150683.53 | 5.88 | 25103979.88 | 24551745.27 | 13.47 | 0.45 | -1.16 |
| N(beta)-Epoxysuccinamoyl-DAP-Val | C20965 | 15762940.56 | 15515005.66 | 7.2 | 130919942.4 | 131152098.8 | 2.07 | 0.12 | -3.05 |
| Penicillin G | C05551 | 3306472.43 | 3223677.16 | 11.49 | 2527064.62 | 2511066.99 | 5.03 | 1.31 | 0.39 |
| Phenolphthalein | C14286 | 240390690 | 236758456.3 | 6.59 | 60008596.22 | 49614510.58 | 51.54 | 4.01 | 2 |
| Brompheniramine | C06857 | 69915395.03 | 70269870.11 | 2.76 | 53893604.99 | 54558204.29 | 5.17 | 1.3 | 0.38 |
| Chlorpromazine | C06906 | 79403626.88 | 76573324.11 | 13.54 | 32945085.56 | 31696298.06 | 17.37 | 2.41 | 1.27 |
| Myricetin | C10107 | 38086760.85 | 37411529.93 | 32.01 | 13587708.98 | 10947598.97 | 38.82 | 2.8 | 1.49 |
| 5(S)-HpETE | C05356 | 15167790.34 | 14250018.5 | 29.19 | 85012042.53 | 78983469.25 | 21.72 | 0.18 | -2.49 |
| 11alpha,17beta-Dihydroxy-17-methylandrost-4-en-3-one | C14555 | 131319431.9 | 130854303 | 11.9 | 504180819.8 | 554192985.5 | 29.2 | 0.26 | -1.94 |
| Deoxy-5-methylcytidylate | C03495 | 24165461.57 | 24554276.68 | 6.38 | 13415880.38 | 13710945.8 | 7.4 | 1.8 | 0.85 |
| 8,9-EET | C14769 | 5168371.82 | 5188651.67 | 4.63 | 4279129.34 | 4343869.32 | 8.36 | 1.21 | 0.27 |
| 8,9-DiHETrE | C14773 | 2112499.83 | 2125672.87 | 3.71 | 4885699.59 | 4798448.33 | 12.73 | 0.43 | -1.21 |
| gamma-L-Glutamyl-L-cysteinyl-beta-alanine | C04544 | 60535267.63 | 69257485.27 | 43.36 | 178803174.5 | 180012476.8 | 12.32 | 0.34 | -1.56 |
| dTMP | C00364 | 11509503.79 | 11384147.19 | 5.18 | 375769 | 356565.03 | 20.31 | 30.63 | 4.94 |
| 2'-Deamino-2'-hydroxy-6'-dehydroparomamine | C20351 | 1389830.93 | 1395313.11 | 6.99 | 4973975.08 | 4984203.47 | 6.4 | 0.28 | -1.84 |
| Citalopram | C07572 | 3730613.69 | 3790342.11 | 8.65 | 7728379.77 | 7422565.31 | 16.69 | 0.48 | -1.05 |
| (S)-Tetrahydropapaverine | C21631 | 4341996.91 | 4402273.06 | 3.84 | 7405667.23 | 7471656.1 | 4.07 | 0.59 | -0.77 |
| 2,4-Dioxotetrahydropyrimidine D-ribonucleotide | C04639 | 16055633.89 | 15542200.88 | 11.45 | 12058239.63 | 12140646.43 | 9.36 | 1.33 | 0.41 |
| Aflatoxin M1 | C16756 | 449443.67 | 461585.43 | 15.99 | 2815126.93 | 3105828.66 | 42.57 | 0.16 | -2.65 |
| Cyclic AMP | C00575 | 11378225.25 | 12276649.15 | 20.94 | 3961228.1 | 4003049.82 | 10.81 | 2.87 | 1.52 |
| Docosapentaenoic acid (22n-3) | C16513 | 308105.8 | 314603.82 | 12.96 | 6025093.73 | 6064084.75 | 4.35 | 0.05 | -4.29 |
| Aflatoxin G2 | C16754 | 42620812.35 | 41966306.38 | 11.03 | 26433793.28 | 27498946.02 | 11.02 | 1.61 | 0.69 |
| Malvidin | C08716 | 133391892.5 | 133061774.1 | 1.67 | 82956616.7 | 82739100.95 | 4.63 | 1.61 | 0.69 |
| Carnosol | C09069 | 9057637.25 | 9109962.4 | 2.52 | 7271378.27 | 7253030.27 | 2.74 | 1.25 | 0.32 |
| Cannabielsoin | C20218 | 5761459.28 | 5436453.33 | 15.21 | 11355268.46 | 11185683.25 | 7.87 | 0.51 | -0.98 |
| Adrenic acid | C16527 | 19626847.9 | 19414354.56 | 5.7 | 47440966.7 | 46794745.27 | 8.65 | 0.41 | -1.27 |
| Norsanguinarine | C05191 | 46131294.62 | 40045186.94 | 33.22 | 83252795.37 | 84578147.11 | 9.91 | 0.55 | -0.85 |
| Andrographolide | C20214 | 13026817.03 | 12748712.58 | 7.73 | 29415443.89 | 31517391.15 | 30.8 | 0.44 | -1.18 |
| 9S-hydroxy-11,15-dioxo-5Z,13E-prostadienoic acid | C04758 | 2014944.48 | 2373998.83 | 48.2 | 8595134.54 | 8978903.01 | 9.04 | 0.23 | -2.09 |
| Prostaglandin E2 | C00584 | 753862.78 | 748634.84 | 3.83 | 3579937.49 | 3602329.59 | 5.26 | 0.21 | -2.25 |
| Prostaglandin H2 | C00427 | 1975466708 | 1988546021 | 5.28 | 1348085680 | 1386478264 | 25.27 | 1.47 | 0.55 |
| Isopentenyl adenosine | C16427 | 1161668.47 | 1175211.13 | 5.28 | 6680660.16 | 6696415.68 | 8.91 | 0.17 | -2.52 |
| 11,12,15-THETA | C14782 | 20553880.29 | 20727483.12 | 25.49 | 59754475.14 | 60042864.99 | 25.46 | 0.34 | -1.54 |
| Psoralidin | C10523 | 34704945.36 | 34399566.62 | 27.01 | 124667115.3 | 125383985.7 | 13.2 | 0.28 | -1.84 |
| p-Coumaroyl quinic acid | C12208 | 9971678.31 | 9830894.32 | 7.15 | 16126530 | 14882624.07 | 19.76 | 0.62 | -0.69 |
| (S)-cis-N-Methylstylopine | C06163 | 12356093.73 | 12099411.41 | 8.61 | 9683209.21 | 9359557.95 | 11.14 | 1.28 | 0.35 |
| Isocorypalmine | C04118 | 12592225.19 | 12654180.12 | 3.94 | 15861052.7 | 15824924.64 | 2.23 | 0.79 | -0.33 |
| Turanose | C19636 | 60494868.6 | 40344586.56 | 59.38 | 475751120.8 | 475562911.3 | 3.96 | 0.13 | -2.98 |
| Cortisone | C00762 | 6775545.28 | 6947065.38 | 11.1 | 3445265.31 | 3504193.74 | 11.86 | 1.97 | 0.98 |
| dGMP | C00362 | 15219729.18 | 14904298.74 | 5.93 | 23416645.98 | 23663418.86 | 7.31 | 0.65 | -0.62 |
| N-Acetylmuramoyl-Ala | C02999 | 6763347.24 | 6827891.23 | 6.51 | 4372030.87 | 4333616.5 | 14.38 | 1.55 | 0.63 |
| 21-Deoxycortisol | C05497 | 281442.47 | 297956.6 | 38.12 | 2679712.4 | 2437502.76 | 66.44 | 0.11 | -3.25 |
| 17alpha,21-Dihydroxypregnenolone | C05487 | 15826754.14 | 15943275.5 | 5.82 | 12625835.74 | 12509667.03 | 11.28 | 1.25 | 0.33 |
| 2-Hydroxy-6-pentadecylbenzoic acid | C10759 | 659989.59 | 572802.05 | 68.88 | 4428493.08 | 3917999.88 | 55.63 | 0.15 | -2.75 |
| Griseofulvin | C06686 | 31876917.71 | 31762394.06 | 3.63 | 13261844.95 | 6992015.49 | 92.45 | 2.4 | 1.27 |
| 6-Keto-prostaglandin F1a | C05961 | 27449550.72 | 27242125.02 | 8.72 | 23723811.67 | 23863284.75 | 4.96 | 1.16 | 0.21 |
| 17-O-Acetylnorajmaline | C11809 | 25001881.97 | 24928215.48 | 1.89 | 20490171.11 | 20243397.2 | 6.72 | 1.22 | 0.29 |
| S-Adenosylmethioninamine | C01137 | 5124392.88 | 5143091.19 | 3.93 | 10945971.81 | 10955648.48 | 6.85 | 0.47 | -1.09 |
| Laudanosine | C09558 | 50856795.13 | 50810349.38 | 12.02 | 7177075.08 | 7009061.43 | 16.32 | 7.09 | 2.82 |
| Niaprazine | D07333 | 8340497.58 | 8726244.65 | 19.86 | 220813457.7 | 218646133 | 8.76 | 0.04 | -4.73 |
| (+)-Pinoresinol | C05366 | 25839613.06 | 27108836.09 | 12.79 | 42732526.13 | 42705035.2 | 3.72 | 0.6 | -0.73 |
| Cortisol | C00735 | 1040785.57 | 516934.07 | 81.26 | 4551938.87 | 4421673.82 | 7.57 | 0.23 | -2.13 |
| 11b,21-Dihydroxy-3,20-oxo-5b-pregnan-18-al | C05473 | 6657370.91 | 6708291.3 | 4.25 | 55838447.03 | 62330828.89 | 30.92 | 0.12 | -3.07 |
| Pyridaben | C18614 | 24768658.06 | 29385217.87 | 49.12 | 5820796.68 | 4062177.64 | 52.79 | 4.26 | 2.09 |
| Dihydrocortisol | C05471 | 10432314.87 | 10536306.81 | 4.15 | 4459855.86 | 4568356.31 | 11.07 | 2.34 | 1.23 |
| U50488 | C11796 | 17148087.89 | 17366187.18 | 7.12 | 6316151.82 | 6223925.15 | 13.58 | 2.71 | 1.44 |
| Strictosidine aglycone | C03309 | 8423777.51 | 8456767.55 | 10.28 | 15593837.93 | 14694806.12 | 23.93 | 0.54 | -0.89 |
| Tamoxifen | C07108 | 8394248.51 | 8410091.16 | 4.07 | 6465618.4 | 5755262.32 | 21.97 | 1.3 | 0.38 |
| Biocytin | C05552 | 28303238.81 | 26294222.6 | 21.86 | 17831378.4 | 16770054.39 | 12.25 | 1.59 | 0.67 |
| Lithocholic acid | C03990 | 12418671.95 | 11775518.34 | 12.12 | 20076464.5 | 20122772 | 3.06 | 0.62 | -0.69 |
| Riboflavin | C00255 | 11881554.53 | 13066477 | 63.09 | 38503199.61 | 37940049.08 | 10.88 | 0.31 | -1.7 |
| Mesoridazine | C07143 | 6969114.19 | 5425358.03 | 59.32 | 28568847.75 | 29931849.47 | 17.37 | 0.24 | -2.04 |
| Sufentanil | C08022 | 10019528.31 | 9878183.88 | 12.75 | 6895678.12 | 6853861.42 | 7.08 | 1.45 | 0.54 |
| Cholesterol | C00187 | 34800483.98 | 39229327.52 | 35.9 | 5922017.37 | 6746359.72 | 27.64 | 5.88 | 2.55 |
| Ursodeoxycholic acid | C07880 | 2832046.78 | 2849954.45 | 2.14 | 7780621.4 | 8164899.78 | 14.43 | 0.36 | -1.46 |
| Aloesin | C08994 | 930716.41 | 941704.41 | 23.33 | 20390793.06 | 17586460.41 | 43.05 | 0.05 | -4.45 |
| Ergocalciferol | C05441 | 11202226.12 | 11345566.39 | 14.75 | 5758734.48 | 6007463.45 | 11.95 | 1.95 | 0.96 |
| S-Adenosylmethionine | C00019 | 268224233.9 | 272093555.4 | 6.37 | 13595635.02 | 13746611.23 | 6.25 | 19.73 | 4.3 |
| S-Adenosyl-4-methylthio-2-oxobutanoate | C04425 | 179032390 | 186604785.8 | 9.22 | 289635111.1 | 285028605 | 8.39 | 0.62 | -0.69 |
| Colchicine | C07592 | 36806234.47 | 36813915.77 | 3.23 | 23013117.17 | 20490608.4 | 25.83 | 1.6 | 0.68 |
| (-)-alpha-Narcotine | C09592 | 4006291.96 | 3758618.95 | 16.63 | 5725858 | 5537500.89 | 8.77 | 0.7 | -0.52 |
| GF 109203X | C11238 | 27699432.94 | 28127854.92 | 8.95 | 10716997.57 | 11101288.54 | 24.26 | 2.58 | 1.37 |
| Paspalicine | C20553 | 824924.73 | 844970.53 | 9.31 | 8549609.56 | 8862080.19 | 9.81 | 0.1 | -3.37 |
| Kaempferol 3-O-beta-D-xyloside | C20727 | 29067631.52 | 27589599.39 | 11.4 | 14544016.42 | 14923048.43 | 7.5 | 2 | 1 |
| Trehalose 6-phosphate | C00689 | 145559324.4 | 152248328.5 | 26.62 | 45088954.68 | 43656687.16 | 28.71 | 3.23 | 1.69 |
| Ginkgolide B | C07602 | 12050848.68 | 13125558.71 | 25.2 | 19854279.55 | 19928195.02 | 8.38 | 0.61 | -0.72 |
| alpha-Tocopherol | C02477 | 1529781.2 | 1504865.04 | 30.5 | 12766765.96 | 13067253.54 | 23.5 | 0.12 | -3.06 |
| Vitexin | C01460 | 414760207.9 | 413306978.9 | 7.91 | 560836130.4 | 561065956.8 | 16.57 | 0.74 | -0.44 |
| Afzelin | C16911 | 24648556.29 | 24979125.9 | 8.02 | 40626288.49 | 42139893.53 | 8.71 | 0.61 | -0.72 |
| Quinapril | C07398 | 2938899.4 | 2977089.66 | 4.88 | 6646464.78 | 6251100.57 | 23.13 | 0.44 | -1.18 |
| Cyanidin 3-galactoside | C08647 | 42973939.91 | 43130908.94 | 17.38 | 19093220.94 | 18498538.67 | 10.89 | 2.25 | 1.17 |
| Etiocholanolone glucuronide | C11136 | 9748727.48 | 10031255.21 | 7.97 | 17539507.41 | 17297034.69 | 10.04 | 0.56 | -0.85 |
| 20-Hydroxyecdysone | C02633 | 7375023.68 | 7166003.07 | 13.01 | 21115130.02 | 19870245.72 | 40.3 | 0.35 | -1.52 |
| Isoquercitrin | C05623 | 15348689.42 | 14871056.92 | 14.35 | 10677514.81 | 10589619.69 | 19.75 | 1.44 | 0.52 |
| Copal-8-ol diphosphate | C20270 | 55251964.71 | 54879245.26 | 8.51 | 30316101.37 | 29766978.13 | 13.67 | 1.82 | 0.87 |
| Withaferin A | C08841 | 18893509.83 | 21094046.31 | 46.65 | 5109716.39 | 4655981.5 | 42.4 | 3.7 | 1.89 |
| Retinoyl b-glucuronide | C11061 | 18579694.3 | 18893847.6 | 6.97 | 32753187.58 | 32801467.89 | 29.32 | 0.57 | -0.82 |
| Loperamide | C07080 | 20784237.05 | 23673499.51 | 26.89 | 9817540.32 | 7938647 | 34.1 | 2.12 | 1.08 |
| Leukotriene D4 | C05951 | 14168583.98 | 13494108.34 | 13.59 | 21079330.26 | 20766035.18 | 5.29 | 0.67 | -0.57 |
| Antibiotic JI-20B | C17705 | 2253953.87 | 2380072.17 | 26.33 | 33229236.15 | 32459015.97 | 11.92 | 0.07 | -3.88 |
| Taurocholic acid | C05122 | 43406494.79 | 34194658.33 | 75.21 | 87790298.99 | 75675054.44 | 29.31 | 0.49 | -1.02 |
| Limonoate | C01593 | 569369860.8 | 563597028.9 | 6.17 | 260216344.7 | 264930805.6 | 23.2 | 2.19 | 1.13 |
| 3-alpha(S)-Strictosidine | C03470 | 2100783.77 | 2085056.32 | 1.98 | 3948156.25 | 3965575.83 | 3.11 | 0.53 | -0.91 |
| Flavonol 3-O-beta-D-glucosyl-(1->2)-beta-D-glucoside | C15581 | 1355021.58 | 1207141.22 | 36.9 | 454307.53 | 385559.71 | 46.94 | 2.98 | 1.58 |
| Lutein | C08601 | 320307565.3 | 290429208.3 | 47.8 | 624881439.9 | 624690707.9 | 23.73 | 0.51 | -0.96 |
| N-Acetyl-O-demethylpuromycin-5'-phosphate | C07030 | 1004795.95 | 241085.45 | 125.68 | 5275727.57 | 4732583.93 | 30.55 | 0.19 | -2.39 |
| Cyanidin 3-O-sophoroside | C16306 | 86859917.85 | 68653464.36 | 57.13 | 196574186.8 | 192268952.6 | 36.42 | 0.44 | -1.18 |
| Delphinidin 3-rutinoside | C16315 | 32395027.42 | 22372174.18 | 55.2 | 63803805.7 | 64561876.01 | 23.03 | 0.51 | -0.98 |
| Stachyose | C01613 | 1764186.73 | 1613746.62 | 35.21 | 27058488.84 | 20949252.16 | 69.1 | 0.07 | -3.94 |
| Cyanidin 3-O-(6-O-p-coumaroyl)glucoside-5-O-glucoside | C12096 | 29182454.06 | 28805018.44 | 4.9 | 35321968.3 | 35210550.98 | 5.94 | 0.83 | -0.28 |
| Reduced coenzyme F420 | C01080 | 6660680.13 | 6381402.38 | 52.87 | 15320442.54 | 15151288.54 | 24.06 | 0.43 | -1.2 |
| PC(18_3(6Z,9Z,12Z)_18_3(6Z,9Z,12Z)) | C00157 | 11400956.81 | 10716147.94 | 14.99 | 5217537.86 | 5548346.52 | 15.21 | 2.19 | 1.13 |
| Ansamitocinoside P-3 | C20139 | 8012341.17 | 7949503.63 | 6.04 | 29793794.63 | 31540152.93 | 35.49 | 0.27 | -1.89 |
| Glyceric acid | C00258 | 22578543.48 | 22384161.6 | 11.85 | 41162020.65 | 39739691.38 | 7.16 | 0.55 | -0.87 |
| 5-Aminopentanoic acid | C00431 | 13930912.02 | 13782336.5 | 5.36 | 3059832.44 | 3027071.09 | 7.79 | 4.55 | 2.19 |
| Phenylacetaldehyde | C00601 | 1518117.92 | 1511332.5 | 3.53 | 1070671.68 | 1056284.87 | 4.1 | 1.42 | 0.5 |
| 3-Methylthiopropionic acid | C08276 | 8316342.14 | 8298532.22 | 3.75 | 6232962.6 | 6280560.99 | 4.83 | 1.33 | 0.42 |
| Itaconic acid | C00490 | 33749527.66 | 30648191.37 | 18.07 | 43468507.01 | 42995613.38 | 3.84 | 0.78 | -0.37 |
| Leucine | C16439 | 24376933.51 | 27516507.05 | 32.36 | 8057252.9 | 9880371.12 | 44.86 | 3.03 | 1.6 |
| Glutaric acid | C00489 | 70011045.68 | 69558318.4 | 26.13 | 24587415.71 | 24409361.8 | 6.93 | 2.85 | 1.51 |
| L-Aspartic acid | C00049 | 13409810.89 | 13329535.45 | 3.99 | 22022042.24 | 22231319.82 | 6.64 | 0.61 | -0.72 |
| Mandelonitrile | C00561 | 15831120.21 | 15799735.96 | 1.56 | 11387141.6 | 11429189.58 | 1.45 | 1.39 | 0.48 |
| Adenine | C00147 | 149828562.9 | 148554216.7 | 2.41 | 107861375.6 | 108055690.3 | 1.57 | 1.39 | 0.47 |
| D-Xylonate | C00502 | 49160543.31 | 48670347.04 | 14.28 | 97915402.27 | 97752537.49 | 12.19 | 0.5 | -0.99 |
| Citramalic acid | C00815 | 90001314.88 | 89814108.8 | 3.11 | 79237234.41 | 79080604.11 | 3.01 | 1.14 | 0.18 |
| D-Xylose | C00181 | 369486667 | 369916074 | 4.89 | 579131440.6 | 581112565.8 | 4.28 | 0.64 | -0.65 |
| 2',4'-Dihydroxyacetophenone | C03663 | 10199415.14 | 10164394.84 | 8.95 | 17592822.07 | 17529519.14 | 2.3 | 0.58 | -0.79 |
| (Z)-4-Hydroxy-6-dodecenoic acid lactone | C03107 | 18950325.59 | 18966583.08 | 1.98 | 3132379.03 | 3057191.37 | 6.73 | 6.05 | 2.6 |
| Pyrophosphate | C00013 | 4493382.4 | 4437128.08 | 5.71 | 8691902.75 | 8710341.08 | 1.14 | 0.52 | -0.95 |
| Fructose-1P | C10906 | 47115716.02 | 46720853.61 | 5.04 | 22273625.23 | 22201374.66 | 3.91 | 2.12 | 1.08 |
| m-Coumaric acid | C12621 | 34543215.6 | 34650782.75 | 1.91 | 33482701.24 | 33494899.07 | 0.98 | 1.03 | 0.04 |
| L-Fucose | C00507 | 17653214.16 | 17635733.32 | 4.44 | 11891318.87 | 11943546.64 | 1.3 | 1.48 | 0.57 |
| trans-2-Hydroxycinnamate | C01772 | 13188634.53 | 13073578.3 | 2.5 | 7393218.66 | 7421738.87 | 3.43 | 1.78 | 0.84 |
| L-Phenylalanine | C00079 | 251621785.8 | 247991008.8 | 6.05 | 165927741.8 | 168661694.1 | 7.89 | 1.52 | 0.6 |
| Terephthalate | C06337 | 138664131.3 | 138303508 | 52.5 | 34871947.56 | 24551264.24 | 79.12 | 3.98 | 1.99 |
| Vanylglycol | C05594 | 12560898.72 | 12592574.48 | 3.92 | 6344905.28 | 6241266.77 | 7.9 | 1.98 | 0.99 |
| Phenyllactate | C05607 | 11600339.72 | 11744499.3 | 9.45 | 4785816.2 | 4762338.32 | 5.54 | 2.42 | 1.28 |
| Tropate | C01456 | 20177259.66 | 20035877.46 | 2.52 | 2218234.39 | 1622844.94 | 68.42 | 9.1 | 3.19 |
| 3,4-Dihydroxybenzeneacetic acid | C01161 | 22793042.47 | 22864341.43 | 7.57 | 38807720.63 | 32937238.57 | 34.46 | 0.59 | -0.77 |
| Norepinephrine | C00547 | 16049598.78 | 16128698.81 | 5 | 9439642.17 | 9392157.28 | 4.76 | 1.7 | 0.77 |
| Beta-Glycerophosphoric acid | C02979 | 8117896.9 | 7890289.7 | 6.04 | 9988338.13 | 10252156.24 | 6.09 | 0.81 | -0.3 |
| Dehydroascorbate | C05422 | 3714985972 | 3641410173 | 4.92 | 316963461 | 319493648.1 | 3.98 | 11.72 | 3.55 |
| Isocitric acid | C00311 | 6295289.92 | 6255360.56 | 5.08 | 1339287.64 | 1280563.04 | 47.72 | 4.7 | 2.23 |
| Guanidinosuccinic acid | C03139 | 331099079.9 | 429729715 | 49.71 | 99378118.89 | 76747160.35 | 38.02 | 3.33 | 1.74 |
| Aesculetin | C09263 | 7381330.06 | 7702642.59 | 14.31 | 1359028.69 | 1237372.96 | 57.04 | 5.43 | 2.44 |
| Gluconic acid | C00257 | 3392719.84 | 3319376.01 | 19.07 | 888027.65 | 887214.13 | 4.36 | 3.82 | 1.93 |
| D-Mannose | C00159 | 555889415.4 | 486620079.6 | 52.14 | 200857688.6 | 179245091.7 | 43.07 | 2.77 | 1.47 |
| D-(+)-Glucose | C00293 | 99211645.76 | 97336686.06 | 5.85 | 57979550.25 | 57889264.85 | 2.96 | 1.71 | 0.77 |
| D-Fructose | C00095 | 7163458726 | 7240449905 | 2.87 | 5763829086 | 5746821687 | 2.63 | 1.24 | 0.31 |
| 2-Hydroxyisophthalic acid | C14097 | 6787951.05 | 6966433.67 | 9.26 | 5792567.37 | 5831071.72 | 7.14 | 1.17 | 0.23 |
| 3,4-Dihydroxymandelic acid | C05580 | 6403659.94 | 3862698.91 | 70.08 | 1829213.71 | 1781082.57 | 10.98 | 3.5 | 1.81 |
| Azelaic acid | C08261 | 10820275.32 | 10597545.44 | 10.62 | 5588506.74 | 5482865.64 | 7.87 | 1.94 | 0.95 |
| D-Glucuronic Acid | C00191 | 6152171.9 | 5627971.64 | 21.51 | 3799878.81 | 3715645.62 | 10.45 | 1.62 | 0.7 |
| trans-Ferulic acid | C01494 | 17954123.22 | 17467771.36 | 5.42 | 7486008.8 | 7310428.9 | 6.25 | 2.4 | 1.26 |
| Xanthoxylin | C10726 | 1867426.72 | 1092909.99 | 75.34 | 10036199.66 | 10255305.77 | 7.94 | 0.19 | -2.43 |
| Vanillylmandelic acid | C05584 | 93746899.99 | 94936730.49 | 3.1 | 64239799.42 | 64639687.2 | 1.93 | 1.46 | 0.55 |
| L-Tryptophan | C00078 | 1920856934 | 1925552553 | 1.41 | 363058430.9 | 362342568 | 1.62 | 5.29 | 2.4 |
| Xanthurenic acid | C02470 | 3527023.44 | 2998335.89 | 39.36 | 719335.72 | 816892.15 | 54.28 | 4.9 | 2.29 |
| (-)-Jasmonic acid | C08491 | 3211274.81 | 3306122.5 | 9.3 | 2170590.22 | 2195600.6 | 8.93 | 1.48 | 0.57 |
| Pantothenic acid | C00864 | 130039242.5 | 132593198.3 | 7.64 | 105690503.4 | 105964270.1 | 5.64 | 1.23 | 0.3 |
| N-Acetyl-D-glucosamine | C00140 | 625842.82 | 642636.08 | 13.69 | 16479671.3 | 16454167.81 | 7.7 | 0.04 | -4.72 |
| 6-Acetyl-D-glucose | C02655 | 20429143.12 | 20678514.77 | 3.95 | 4641437.69 | 4654761.67 | 2.33 | 4.4 | 2.14 |
| Methyl jasmonate | C11512 | 95317679 | 95993927.66 | 3.07 | 106122899.5 | 107265275 | 6.15 | 0.9 | -0.15 |
| Thymidine | C00214 | 71372087.39 | 62045517.43 | 58.7 | 14320773.65 | 14251033.69 | 8.19 | 4.98 | 2.32 |
| Galactosylglycerol | C05401 | 51804132.41 | 52728500.88 | 15.06 | 40312504.3 | 40269581.31 | 4.68 | 1.29 | 0.36 |
| Shikimate 3-phosphate | C03175 | 4479220.72 | 4482062.56 | 3.52 | 968969.85 | 991484.82 | 7.67 | 4.62 | 2.21 |
| Galactose 1-phosphate | C00103 | 107051795.4 | 106422093.6 | 9.41 | 159841981.7 | 162706288.1 | 15.46 | 0.67 | -0.58 |
| (S)-Abscisic acid | C06082 | 105452631.3 | 105184461.1 | 22.05 | 45357798.71 | 49374479.47 | 39.08 | 2.32 | 1.22 |
| Phloretin | C00774 | 11422033.2 | 11258340.42 | 2.8 | 3868714.79 | 3900277 | 3.26 | 2.95 | 1.56 |
| Pantetheine | C00831 | 257030.07 | 248998.37 | 24.27 | 18438639.12 | 18632788.14 | 7.18 | 0.01 | -6.16 |
| Hexadecanedioate | C19615 | 1346271268 | 1325289429 | 5.31 | 797409752.6 | 805400445.7 | 2.4 | 1.69 | 0.76 |
| Catechin | C06562 | 507197111.4 | 507261917.6 | 7.32 | 1440595208 | 1419393554 | 5.67 | 0.35 | -1.51 |
| 13(S)-HPOT | C04785 | 15451613.18 | 19732403.1 | 52.28 | 3389961.98 | 3340422.98 | 4.87 | 4.56 | 2.19 |
| Gingerol | C10462 | 23885800.04 | 24242717.14 | 4.78 | 12153362.08 | 12187517.46 | 8.28 | 1.97 | 0.97 |
| 4-(beta-D-Glucosyloxy)benzoate | C03993 | 43603691.92 | 43856420.36 | 2.05 | 19403443.65 | 19636460.29 | 7.51 | 2.25 | 1.17 |
| Diosmetin | C10038 | 7914435.11 | 7890371.04 | 3.09 | 4665445.15 | 4629470.51 | 2.64 | 1.7 | 0.76 |
| 9-cis-Retinoic acid | C15493 | 47151069.03 | 47444090.15 | 8.23 | 97786824.97 | 97609268.4 | 16.01 | 0.48 | -1.05 |
| EPA (d5) | C06428 | 194354338.2 | 193001889.3 | 2.71 | 107094691.4 | 101762482.4 | 13.13 | 1.81 | 0.86 |
| (-)-Epigallocatechin | C12136 | 306367392.1 | 309929991.3 | 6.99 | 230212344.7 | 231044920.9 | 9.37 | 1.33 | 0.41 |
| Dhurrin | C05143 | 3982677.98 | 3897601.86 | 20.86 | 5985682.17 | 6120215.72 | 15.82 | 0.67 | -0.59 |
| 12-KETE | C14807 | 366031106.7 | 364787405.1 | 4.39 | 585031366.3 | 595071798.9 | 5.27 | 0.63 | -0.68 |
| Dihydromyricetin | C02906 | 69103062.91 | 31860631.08 | 105.67 | 246469779.5 | 241292103.9 | 32.08 | 0.28 | -1.83 |
| trans-beta-D-Glucosyl-2-hydroxycinnamate | C05158 | 19004328.16 | 16087058.39 | 38.24 | 11454354.15 | 11504773.89 | 3.29 | 1.66 | 0.73 |
| 1-O-Vanilloyl-beta-D-glucose | C20470 | 131399935.9 | 133848080.5 | 5.98 | 243441085.5 | 251433726.5 | 7.89 | 0.54 | -0.89 |
| Prostaglandin A2 | C05953 | 221821497.4 | 215825735 | 7.19 | 139020171.9 | 147979003.1 | 13.21 | 1.6 | 0.67 |
| Delta-12-Prostaglandin J2 | C05958 | 5531947558 | 5792577283 | 12.96 | 2400645244 | 2509487730 | 15.24 | 2.3 | 1.2 |
| Dicumarol | C00796 | 13263898.55 | 13334948.66 | 11.25 | 6921506.67 | 6766348.3 | 6.29 | 1.92 | 0.94 |
| (5Z,9E,14Z)-(8xi,11R,12S)-11,12-epoxy-8-hydroxyicosa-5,9,14-trienoic Acid | C04849 | 121645938.4 | 126703739.3 | 10.88 | 180968661.2 | 180260509.9 | 4.78 | 0.67 | -0.57 |
| Dattelic acid | C10434 | 6531884.89 | 6582986.17 | 9.09 | 12871995.24 | 12797569.71 | 9.78 | 0.51 | -0.98 |
| Indole-3-acetyl-beta-1-D-glucoside | C04197 | 10645011.38 | 10746009.57 | 7.44 | 5113244.58 | 5007324.12 | 7.29 | 2.08 | 1.06 |
| 11,12-DiHETrE | C14774 | 41145473.5 | 40398224.33 | 11.75 | 19584313.97 | 19902346.58 | 8.66 | 2.1 | 1.07 |
| Erucic acid | C08316 | 13903457.02 | 11863220.43 | 76.83 | 33065106.19 | 32145360.97 | 11.85 | 0.42 | -1.25 |
| Fructose 1,6-bisphosphate | C00354 | 345363.36 | 194185.78 | 83.49 | 238070106.3 | 236731180.9 | 11.14 | 0 | -9.43 |
| Trehalose | C01083 | 15559741 | 15578343.64 | 2.15 | 5418541.67 | 5256348.47 | 8.45 | 2.87 | 1.52 |
| 11-Dehydro-thromboxane B2 | C05964 | 11470546.09 | 11877282.19 | 11.12 | 14451707.84 | 14141276.23 | 8.12 | 0.79 | -0.33 |
| 19-Hydroxytabersonine | C11642 | 905932.36 | 899327.64 | 6.04 | 2878395.67 | 2796796.47 | 43.2 | 0.31 | -1.67 |
| 13,14-Dihydro-15-keto-PGE2 | C04671 | 1420046.19 | 1336900.59 | 13.95 | 3310902.67 | 3347759.52 | 6.49 | 0.43 | -1.22 |
| Prostaglandin F2a | C00639 | 3209322.28 | 3210883.52 | 5.77 | 1618465.43 | 1640084.08 | 28.84 | 1.98 | 0.99 |
| Pioglitazone | C07675 | 5543347.89 | 5339939.29 | 32.42 | 134955582.8 | 132165201.9 | 6.99 | 0.04 | -4.61 |
| 20-Carboxy-leukotriene B4 | C05950 | 31343180.07 | 31173473.74 | 4.47 | 15039817.85 | 11039041.62 | 64.32 | 2.08 | 1.06 |
| Dehydroepiandrosterone sulfate | C04555 | 26162588 | 29142061.04 | 28.56 | 12532220.88 | 13466525.05 | 34.69 | 2.09 | 1.06 |
| Geniposidic acid | C11673 | 13960587.8 | 16374076.03 | 43.29 | 5645423.62 | 5089780.76 | 44.56 | 2.47 | 1.31 |
| (-)-Wikstromol | C10725 | 24627666.74 | 18466181.29 | 64.25 | 6366944.4 | 6382221.72 | 9.65 | 3.87 | 1.95 |
| Acetyl-maltose | C02130 | 3778899.75 | 3424757.9 | 26.16 | 1598599.67 | 1599301.9 | 5.94 | 2.36 | 1.24 |
| Gardenoside | C09779 | 13559979.98 | 14410632.26 | 30.25 | 3375652.6 | 3241883.17 | 44.9 | 4.02 | 2.01 |
| Carvedilol | C06875 | 34632830.98 | 33374367.79 | 7.01 | 168492014.6 | 163143501.6 | 12.64 | 0.21 | -2.28 |
| trans-Piceid | C10275 | 42052330.38 | 42519456.55 | 42.96 | 15247456.78 | 14495750.94 | 20.99 | 2.76 | 1.46 |
| Podofilox | C10874 | 16041284.87 | 16095530.45 | 2.72 | 6877200.27 | 6929599.16 | 9.97 | 2.33 | 1.22 |
| Mitragynine | C09226 | 118814.85 | 118286.12 | 15.37 | 1911201.9 | 1732144.06 | 56.24 | 0.06 | -4.01 |
| Quinacrine | C07339 | 13749429.76 | 13310447.47 | 7.94 | 27276830.2 | 27862813.23 | 7.07 | 0.5 | -0.99 |
| Puerarin | C10524 | 116213071 | 124846073.6 | 28.77 | 30038234.85 | 25917076.93 | 40.1 | 3.87 | 1.95 |
| Lamioside | C11645 | 4355789.98 | 4286934.54 | 5.3 | 1697606.27 | 1613848.51 | 16.49 | 2.57 | 1.36 |
| Lamiide | C11644 | 89751633.55 | 90074789.42 | 8.15 | 49944536.81 | 55180548.32 | 27.67 | 1.8 | 0.85 |
| Losartan | C07072 | 18866499.94 | 18307654.97 | 11.89 | 13764661.75 | 12886845.78 | 23.72 | 1.37 | 0.45 |
| Abscisic acid glucose ester | C15970 | 84016520.65 | 82476603.09 | 20.42 | 36552309.84 | 37262695.55 | 20.04 | 2.3 | 1.2 |
| Epigallocatechin gallate | C09731 | 7772233.4 | 7627613.36 | 12.1 | 4526734.86 | 4396266.56 | 30.11 | 1.72 | 0.78 |
| Folic acid | C00504 | 18821744.04 | 19103673.47 | 13.1 | 13545189.34 | 13740794.56 | 15.83 | 1.39 | 0.47 |
| 6-Methoxyluteolin 7-rhamnoside | C10104 | 141581700.3 | 160714717.2 | 33.39 | 49735181.98 | 45388487.09 | 24.12 | 2.85 | 1.51 |
| Myricitrin | C10108 | 3456553.9 | 3947346.83 | 58.64 | 31456438.49 | 35105695.07 | 35.29 | 0.11 | -3.19 |
| Delphinidin 3-glucoside | C12138 | 1597750945 | 1923224656 | 39.17 | 199272572.8 | 131134329 | 85 | 8.02 | 3 |
| Petunidin 3-glucoside | C12139 | 13489260.02 | 13072697.23 | 25.38 | 7679481.9 | 7807755.76 | 10.56 | 1.76 | 0.81 |
| 8-Epiiridodial glucoside tetraacetate | C11658 | 7568018.7 | 7325158.19 | 13.71 | 3852916.91 | 3880932.64 | 20.43 | 1.96 | 0.97 |
| 1-Kestose | C03661 | 50602025.75 | 43394831.11 | 47.82 | 13969770.16 | 13674805.71 | 17.51 | 3.62 | 1.86 |
| 11-Hydroxyiridodial glucoside pentaacetate | C11666 | 212822955.6 | 225572670.1 | 18.37 | 679706511.7 | 577688849.5 | 37.31 | 0.31 | -1.68 |
| Loganin pentaacetate | C11663 | 12917605.34 | 10697089 | 53.63 | 23546585.15 | 22801354.62 | 9.5 | 0.55 | -0.87 |
| Asperuloside tetraacetate | C11655 | 5901713.26 | 5899766.99 | 1.71 | 3633163.95 | 3394825.16 | 33.86 | 1.62 | 0.7 |
| Delphinidin 3-(6-p-coumaroyl)glucoside | C16370 | 4511077.2 | 4554200.82 | 11.54 | 7647295.82 | 7472237.36 | 25.21 | 0.59 | -0.76 |
| Neomycin | C01737 | 207951.41 | 147416.08 | 95.8 | 2083568.31 | 2442768.04 | 51.32 | 0.1 | -3.32 |
| Eriocitrin | C09732 | 64523421.6 | 70824346.21 | 18.12 | 14015861.02 | 15828785.66 | 40.27 | 4.6 | 2.2 |
| Quercetin 3-O-beta-D-glucosyl-(1->2)-beta-D-glucoside | C12667 | 10249173.31 | 8314125.07 | 32.2 | 4939624.9 | 3591603.88 | 53.13 | 2.07 | 1.05 |
| Delphinidin 3-O-(6-caffeoyl-beta-D-glucoside) | C16367 | 1710498.8 | 1258932.43 | 56.85 | 48729967.16 | 51349314.33 | 17.44 | 0.04 | -4.83 |
| Delphin | C16312 | 4908994.58 | 4727855.51 | 12.21 | 1887778.12 | 2033967.25 | 30.49 | 2.6 | 1.38 |
