# Supplementary material for: Response mechanism of carbon metabolism of Pinus massoniana to gradient high temperature and drought stress
Source: BMC Genomics. 2024 Feb 12;25:166. doi: 10.1186/s12864-024-10054-2 (PMC10860282; doi:10.1186/s12864-024-10054-2)
Supplement: Supplementary file 15 — Additional file 15. [file 12864_2024_10054_MOESM15_ESM.docx]

Table S18 Summary table of 68 metabolic pathways.

| **pathway_id** | **pathway_name** | **Total** | **Hits** | **Pvalue** | **-Log10(Pvalue)** | **FDR** | **Impact** |
| --- | --- | --- | --- | --- | --- | --- | --- |
| ath04075 | Plant hormone signal transduction | 12 | 3 | 0.020646889 | 1.68514537159941 | 1 | 0.25 |
| ath00941 | Flavonoid biosynthesis | 74 | 7 | 0.082467876 | 1.08371518890134 | 1 | 0.0909 |
| ath00400 | Phenylalanine, tyrosine and tryptophan biosynthesis | 34 | 4 | 0.093367373 | 1.02980486250767 | 1 | 0.1774 |
| ath00220 | Arginine biosynthesis | 23 | 3 | 0.110448713 | 0.956839341951163 | 1 | 0.1433 |
| ath00945 | Stilbenoid, diarylheptanoid and gingerol biosynthesis | 25 | 3 | 0.133359128 | 0.874977251943299 | 1 | 0.1429 |
| ath02010 | ABC transporters | 138 | 10 | 0.167202262 | 0.776757852210586 | 1 | 0.0725 |
| ath00592 | alpha-Linolenic acid metabolism | 44 | 4 | 0.186251772 | 0.729899586823777 | 1 | 0.1279 |
| ath00360 | Phenylalanine metabolism | 60 | 5 | 0.191449676 | 0.717945363497616 | 1 | 0.093 |
| ath00460 | Cyanoamino acid metabolism | 45 | 4 | 0.196830975 | 0.705906555172596 | 1 | 0.094 |
| ath00310 | Lysine degradation | 50 | 4 | 0.252151696 | 0.598338106802114 | 1 | 0.051 |
| ath00944 | Flavone and flavonol biosynthesis | 51 | 4 | 0.263603214 | 0.579049298984915 | 1 | 0.0704 |
| ath00030 | Pentose phosphate pathway | 35 | 3 | 0.265482668 | 0.575963826724292 | 1 | 0.0405 |
| ath00970 | Aminoacyl-tRNA biosynthesis | 52 | 4 | 0.27515189 | 0.560427499048518 | 1 | 0.0727 |
| ath00040 | Pentose and glucuronate interconversions | 56 | 4 | 0.3220555 | 0.49206927968386 | 1 | 0.1667 |
| ath00908 | Zeatin biosynthesis | 39 | 3 | 0.322439203 | 0.491552161444812 | 1 | 0.065 |
| ath00710 | Carbon fixation in photosynthetic organisms | 23 | 2 | 0.331167184 | 0.479952704336401 | 1 | 0.0429 |
| ath00250 | Alanine, aspartate and glutamate metabolism | 28 | 2 | 0.424230946 | 0.372397654188178 | 1 | 0.2327 |
| ath00562 | Inositol phosphate metabolism | 47 | 3 | 0.435506617 | 0.361005241713236 | 1 | 0.0095 |
| ath00770 | Pantothenate and CoA biosynthesis | 30 | 2 | 0.459604668 | 0.337615568625123 | 1 | 0.0294 |
| ath00960 | Tropane, piperidine and pyridine alkaloid biosynthesis | 68 | 4 | 0.46320281 | 0.33422881511071 | 1 | 0.0526 |
| ath00260 | Glycine, serine and threonine metabolism | 50 | 3 | 0.476274266 | 0.322142883735746 | 1 | 0.0288 |
| ath00410 | beta-Alanine metabolism | 32 | 2 | 0.493692538 | 0.30654343707338 | 1 | 0.0667 |
| ath00590 | Arachidonic acid metabolism | 75 | 4 | 0.541026649 | 0.266781342984655 | 1 | 0.043 |
| ath00300 | Lysine biosynthesis | 35 | 2 | 0.542211086 | 0.265831607081629 | 1 | 0.12 |
| ath00350 | Tyrosine metabolism | 78 | 4 | 0.572607104 | 0.242143268061873 | 1 | 0.029 |
| ath00500 | Starch and sucrose metabolism | 37 | 2 | 0.572730214 | 0.242049905667734 | 1 | 0.2429 |
| ath00561 | Glycerolipid metabolism | 38 | 2 | 0.587429681 | 0.231044113755688 | 1 | 0.0079 |
| ath00480 | Glutathione metabolism | 38 | 2 | 0.587429681 | 0.231044113755688 | 1 | 0.0567 |
| ath00910 | Nitrogen metabolism | 19 | 1 | 0.632610122 | 0.19886386335043 | 1 | 0.0404 |
| ath00240 | Pyrimidine metabolism | 65 | 3 | 0.655550031 | 0.183394157730596 | 1 | 0.1429 |
| ath00942 | Anthocyanin biosynthesis | 66 | 3 | 0.665808832 | 0.176650448328574 | 1 | 0.0595 |
| ath00261 | Monobactam biosynthesis | 45 | 2 | 0.67985756 | 0.167582068838812 | 1 | 0.0526 |
| ath00940 | Phenylpropanoid biosynthesis | 68 | 3 | 0.685659589 | 0.163891446113711 | 1 | 0.0165 |
| ath00232 | Caffeine metabolism | 22 | 1 | 0.686487246 | 0.16336752669475 | 1 | 0.0625 |
| ath00052 | Galactose metabolism | 46 | 2 | 0.691593986 | 0.160148791699789 | 1 | 0.0769 |
| ath00290 | Valine, leucine and isoleucine biosynthesis | 23 | 1 | 0.702638158 | 0.153268268581169 | 1 | 0.0374 |
| ath00340 | Histidine metabolism | 47 | 2 | 0.702975647 | 0.153059719723649 | 1 | 0.013 |
| ath00565 | Ether lipid metabolism | 25 | 1 | 0.732498456 | 0.135193286305554 | 1 | 0.0222 |
| ath00600 | Sphingolipid metabolism | 25 | 1 | 0.732498456 | 0.135193286305554 | 1 | 0.0755 |
| ath00073 | Cutin, suberine and wax biosynthesis | 27 | 1 | 0.759374307 | 0.119544100782706 | 1 | 0.0175 |
| ath00780 | Biotin metabolism | 28 | 1 | 0.771787025 | 0.112502527152782 | 1 | 0.0317 |
| ath00760 | Nicotinate and nicotinamide metabolism | 55 | 2 | 0.781967078 | 0.106811531091973 | 1 | 0.0609 |
| ath00750 | Vitamin B6 metabolism | 29 | 1 | 0.783562593 | 0.10592630508253 | 1 | 0.0411 |
| ath00998 | Biosynthesis of various secondary metabolites - part 2 | 81 | 3 | 0.793596183 | 0.10040042958376 | 1 | 0.0323 |
| ath00010 | Glycolysis / Gluconeogenesis | 31 | 1 | 0.805330802 | 0.09402569 | 1 | 0.0157 |
| ath00380 | Tryptophan metabolism | 83 | 3 | 0.807138534 | 0.0930519185313053 | 1 | 0.0336 |
| ath00920 | Sulfur metabolism | 33 | 1 | 0.824919926 | 0.0835882060317913 | 1 | 0.0052 |
| ath00270 | Cysteine and methionine metabolism | 63 | 2 | 0.841990099 | 0.0746930152806164 | 1 | 0.0062 |
| ath00230 | Purine metabolism | 95 | 3 | 0.873581395 | 0.0586966238281131 | 1 | 0.0266 |
| ath00901 | Indole alkaloid biosynthesis | 70 | 2 | 0.881825563 | 0.0546173157241421 | 1 | 0.0581 |
| ath00280 | Valine, leucine and isoleucine degradation | 42 | 1 | 0.891445238 | 0.0499053306604998 | 1 | 0.0242 |
| ath01040 | Biosynthesis of unsaturated fatty acids | 74 | 2 | 0.900220169 | 0.0456512613609045 | 1 | 0.0312 |
| ath00900 | Terpenoid backbone biosynthesis | 45 | 1 | 0.907458208 | 0.0421733665573075 | 1 | 0.022 |
| ath00966 | Glucosinolate biosynthesis | 77 | 2 | 0.912233561 | 0.0398939539473609 | 1 | 0.0182 |
| ath00520 | Amino sugar and nucleotide sugar metabolism | 108 | 3 | 0.922031775 | 0.0352541121848783 | 1 | 0.0636 |
| ath00071 | Fatty acid degradation | 50 | 1 | 0.929092627 | 0.0319409862931522 | 1 | 0.0031 |
| ath00053 | Ascorbate and aldarate metabolism | 50 | 1 | 0.929092627 | 0.0319409862931522 | 1 | 0.0169 |
| ath00564 | Glycerophospholipid metabolism | 52 | 1 | 0.936263555 | 0.0286018815200884 | 1 | 0.0137 |
| ath00440 | Phosphonate and phosphinate metabolism | 56 | 1 | 0.948512301 | 0.0229570326569089 | 1 | 0.0152 |
| ath00061 | Fatty acid biosynthesis | 58 | 1 | 0.953727534 | 0.0205756789945893 | 1 | 0.006 |
| ath00100 | Steroid biosynthesis | 58 | 1 | 0.953727534 | 0.0205756789945893 | 1 | 0.0068 |
| ath00790 | Folate biosynthesis | 58 | 1 | 0.953727534 | 0.0205756789945893 | 1 | 0.0279 |
| ath00130 | Ubiquinone and other terpenoid-quinone biosynthesis | 92 | 2 | 0.954506923 | 0.0202209173312613 | 1 | 0.0061 |
| ath00950 | Isoquinoline alkaloid biosynthesis | 122 | 3 | 0.954813273 | 0.0200815525061259 | 1 | 0.0214 |
| ath00630 | Glyoxylate and dicarboxylate metabolism | 62 | 1 | 0.96263341 | 0.0165390693840203 | 1 | 0.0114 |
| ath00330 | Arginine and proline metabolism | 78 | 1 | 0.984147805 | 0.00693967162313011 | 1 | 0.0039 |
| ath00909 | Sesquiterpenoid and triterpenoid biosynthesis | 88 | 1 | 0.990742462 | 0.0040392233391983 | 1 | 0.0152 |
| ath00906 | Carotenoid biosynthesis | 115 | 1 | 0.997849681 | 0.000934877129058485 | 1 | 0.0028 |
