# Supplementary material for: Response mechanism of carbon metabolism of Pinus massoniana to gradient high temperature and drought stress
Source: BMC Genomics. 2024 Feb 12;25:166. doi: 10.1186/s12864-024-10054-2 (PMC10860282; doi:10.1186/s12864-024-10054-2)
Supplement: Supplementary file 16 — Additional file 16. [file 12864_2024_10054_MOESM16_ESM.docx]

Table S19 There are 6 metabolic pathways of carbon metabolism

| **pathway_id** | **pathway_name** | **Total** | **Hits** | **Pvalue** | **-Log10(Pvalue)** | **Impact** | **compounds** | **Compound_name** |
| --- | --- | --- | --- | --- | --- | --- | --- | --- |
| ath00030 | Pentose phosphate pathway | 35 | 3 | 0.265482668 | 0.575963826724292 | 0.0405 | C00121; C00257; C01801 | D-Ribose; Gluconic acid; Deoxyribose |
| ath00040 | Pentose and glucuronate interconversions | 56 | 4 | 0.3220555 | 0.49206927968386 | 0.1667 | C00103; C00181; C00310; C00476 | Galactose 1-phosphate; D-Xylose; L-Ribulose; D-Lyxose |
| ath00710 | Carbon fixation in photosynthetic organisms | 23 | 2 | 0.331167184 | 0.479952704336401 | 0.0429 | C00049; C00354 | L-Aspartic acid; Fructose 1,6-bisphosphate |
| ath00500 | Starch and sucrose metabolism | 37 | 2 | 0.572730214 | 0.242049905667734 | 0.2429 | C00103; C01083 | Galactose 1-phosphate; Trehalose |
| ath00052 | Galactose metabolism | 46 | 2 | 0.691593986 | 0.160148791699789 | 0.0769 | C00103; C05401 | Galactose 1-phosphate; Galactosylglycerol |
| ath00010 | Glycolysis / Gluconeogenesis | 31 | 1 | 0.805330802 | 0.09402569 | 0.0157 | C00103 | Galactose 1-phosphate |
