# Supplementary material for: Response mechanism of carbon metabolism of Pinus massoniana to gradient high temperature and drought stress
Source: BMC Genomics. 2024 Feb 12;25:166. doi: 10.1186/s12864-024-10054-2 (PMC10860282; doi:10.1186/s12864-024-10054-2)
Supplement: Supplementary file 17 — Additional file 17. [file 12864_2024_10054_MOESM17_ESM.docx]

Table S20 The metabolic pathways of starch and sucrose were analyzed jointly by metabolome and transcriptome

| **Treatment** | **Description** | **counts** | **Compounds** | **Genes_count** | **Genes** |
| --- | --- | --- | --- | --- | --- |
| 25℃ | Starch and sucrose metabolism | 4 | Galactose 1-phosphate;Cellobiose;Trehalose 6-phosphate;Trehalose; | 59 | PITA_26569;PITA_13650;PITA_10269;PITA_30053;PITA_34725;PITA_37093;PITA_07189;PITA_08511;PITA_19633;PITA_35427;PITA_48713;PITA_33157;PITA_07384;PITA_28625;PITA_21358;PITA_05742;PITA_22168;PITA_48635;PITA_24200;PITA_10586;PITA_17694;PITA_20321;PITA_41530;PITA_11514;PITA_07159;PITA_15370;PITA_41038;PITA_27138;PITA_23271;PITA_43916;PITA_41069;PITA_10650;PITA_04249;PITA_07279;PITA_39609;PITA_04722;PITA_14282;PITA_45107;PITA_47529;PITA_12422;PITA_36401;PITA_31267;PITA_28584;PITA_13827;PITA_28004;PITA_38689;PITA_34393;PITA_05060;PITA_44888;PITA_01070;PITA_38156;PITA_15310;PITA_41655;PITA_27768;PITA_47736;PITA_11281;PITA_02822;PITA_33988;PITA_41191 |
| 30℃ | Starch and sucrose metabolism | 5 | D-Fructose;Galactose 1-phosphate;Cellobiose;Trehalose;3-Ketosucrose; | 78 | PITA_11463;PITA_28625;PITA_07932;PITA_49446;PITA_16672;PITA_49979;PITA_19548;PITA_10099;PITA_41038;PITA_21542;PITA_20586;PITA_23271;PITA_41069;PITA_10650;PITA_12029;PITA_37252;PITA_27138;PITA_24090;PITA_50298;PITA_51701;PITA_07244;PITA_11651;PITA_04249;PITA_07279;PITA_38204;PITA_04250;PITA_38689;PITA_18428;PITA_34393;PITA_05060;PITA_44888;PITA_13650;PITA_23307;PITA_37093;PITA_30570;PITA_10269;PITA_08511;PITA_07189;PITA_39026;PITA_34725;PITA_23479;PITA_35427;PITA_31902;PITA_11562;PITA_34113;PITA_18845;PITA_47810;PITA_20157;PITA_12108;PITA_30901;PITA_01907;PITA_48713;PITA_22238;PITA_05742;PITA_22168;PITA_48635;PITA_24200;PITA_10586;PITA_18789;PITA_20321;PITA_41530;PITA_49282;PITA_19103;PITA_15565;PITA_45107;PITA_36401;PITA_28584;PITA_13827;PITA_28323;PITA_15310;PITA_20247;PITA_01070;PITA_27768;PITA_47736;PITA_41655;PITA_11281;PITA_38156;PITA_33988 |
| 35℃ | Starch and sucrose metabolism | 4 | D-Fructose;Galactose 1-phosphate;Trehalose 6-phosphate;Trehalose; | 93 | PITA_07384;PITA_28625;PITA_38197;PITA_10099;PITA_08359;PITA_41038;PITA_20586;PITA_21542;PITA_23271;PITA_41069;PITA_37252;PITA_27138;PITA_10650;PITA_38689;PITA_23307;PITA_13650;PITA_10269;PITA_30570;PITA_26569;PITA_39026;PITA_37093;PITA_28120;PITA_26713;PITA_23479;PITA_10721;PITA_29884;PITA_36623;PITA_35427;PITA_27278;PITA_23192;PITA_07189;PITA_34725;PITA_34113;PITA_48713;PITA_33157;PITA_30901;PITA_20321;PITA_14469;PITA_44315;PITA_00474;PITA_07932;PITA_49979;PITA_43062;PITA_43807;PITA_31153;PITA_07159;PITA_24090;PITA_50298;PITA_51701;PITA_43102;PITA_04249;PITA_07244;PITA_11651;PITA_01499;PITA_38204;PITA_37767;PITA_04250;PITA_16854;PITA_18428;PITA_34393;PITA_05060;PITA_44888;PITA_13221;PITA_48635;PITA_05742;PITA_22168;PITA_33668;PITA_24200;PITA_10586;PITA_18789;PITA_25292;PITA_17694;PITA_24667;PITA_36871;PITA_41530;PITA_15565;PITA_19103;PITA_49282;PITA_36401;PITA_31267;PITA_28584;PITA_28323;PITA_33829;PITA_13827;PITA_15310;PITA_20247;PITA_27768;PITA_41655;PITA_47736;PITA_11281;PITA_22009;PITA_40534;PITA_33988 |
