# Supplementary material for: Response mechanism of carbon metabolism of Pinus massoniana to gradient high temperature and drought stress
Source: BMC Genomics. 2024 Feb 12;25:166. doi: 10.1186/s12864-024-10054-2 (PMC10860282; doi:10.1186/s12864-024-10054-2)
Supplement: Supplementary file 18 — Additional file 18. [file 12864_2024_10054_MOESM18_ESM.docx]

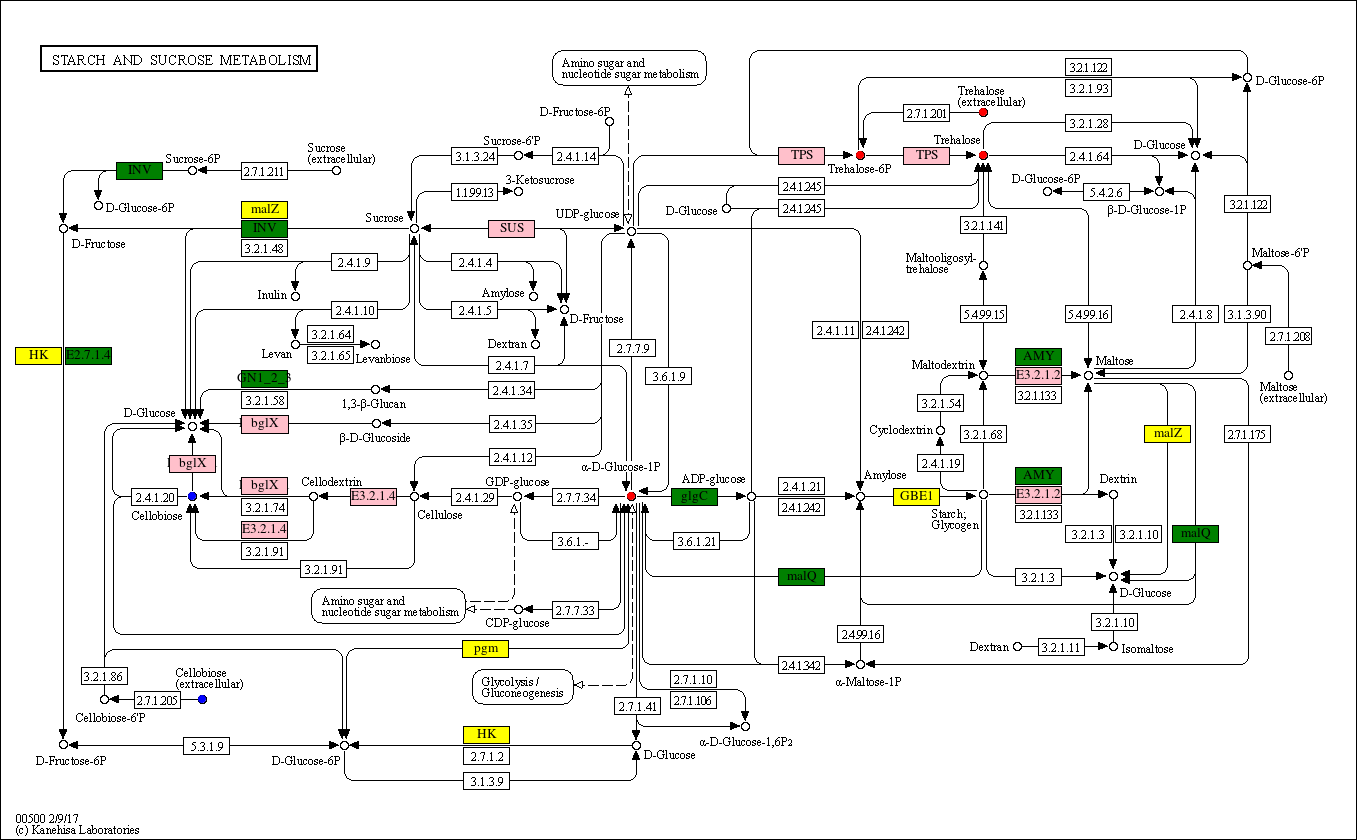


Fig. S3 Combined transcriptome and metabolome analysis of starch and sucrose metabolic pathways in T25CK vs T25Z


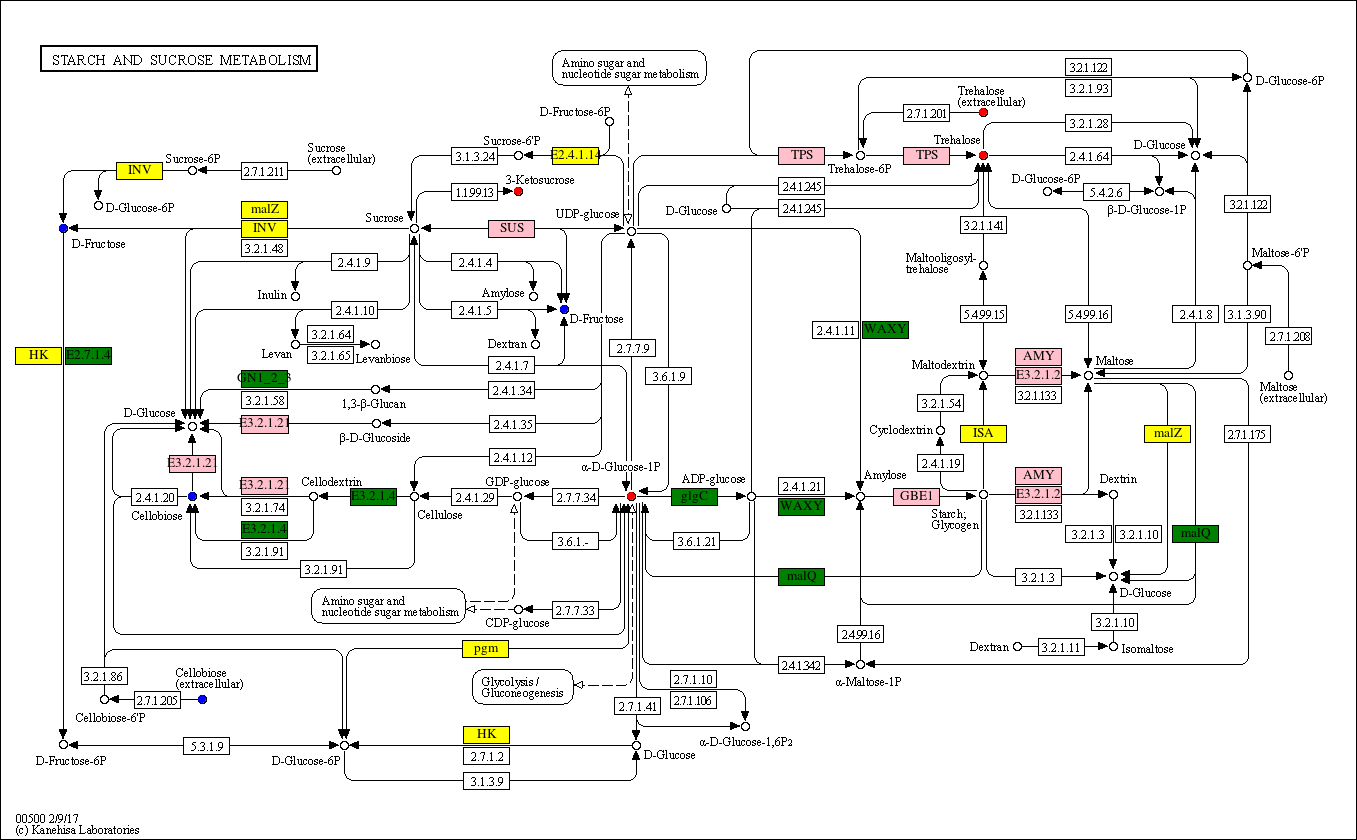


Fig. S4 Combined transcriptome and metabolome analysis of starch and sucrose metabolic pathways in T30CK vs T30Z


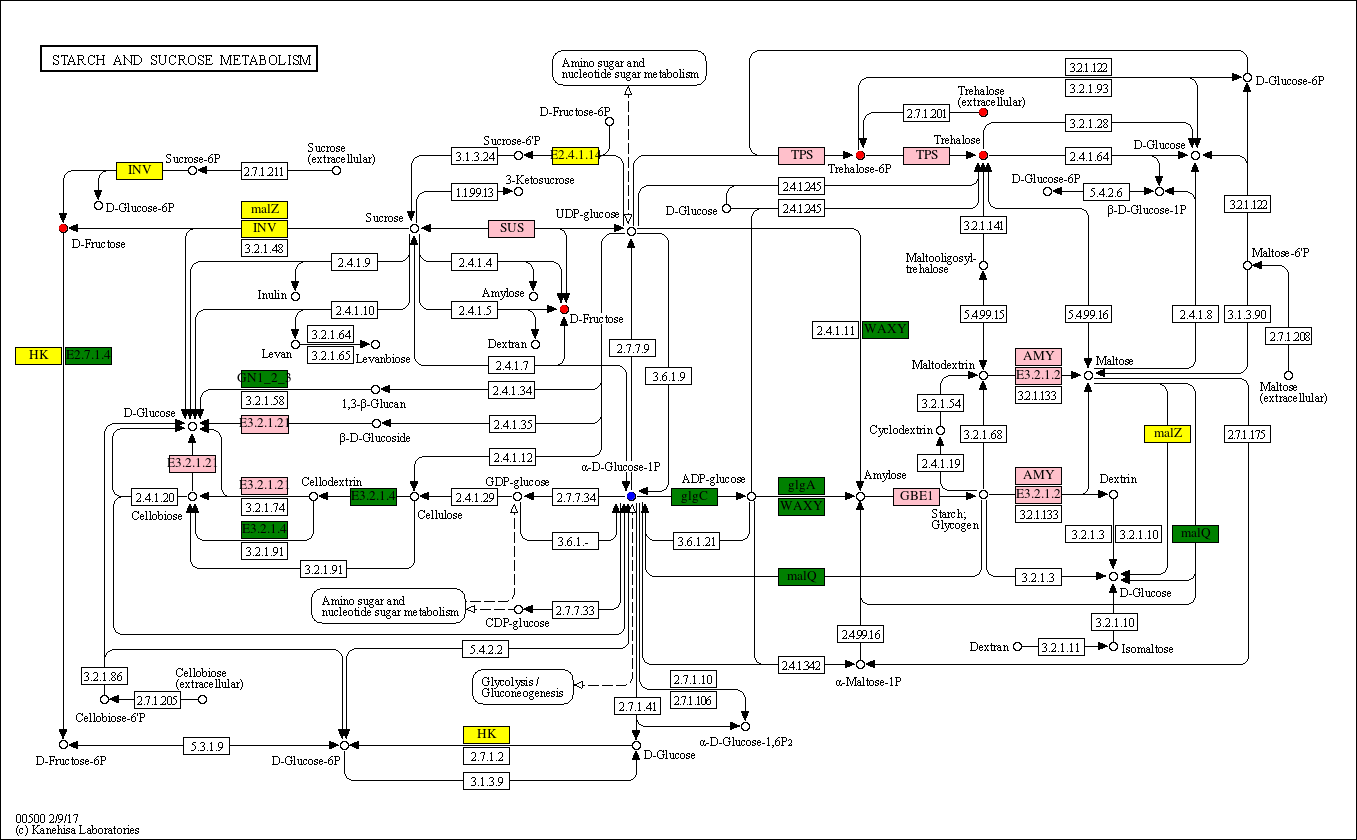


Fig. S5 Combined transcriptome and metabolome analysis of starch and sucrose metabolic pathways in T35CK vs T35Z
