# Supplementary material for: Response mechanism of carbon metabolism of Pinus massoniana to gradient high temperature and drought stress
Source: BMC Genomics. 2024 Feb 12;25:166. doi: 10.1186/s12864-024-10054-2 (PMC10860282; doi:10.1186/s12864-024-10054-2)
Supplement: Supplementary file 19 — Additional file1 9. [file 12864_2024_10054_MOESM19_ESM.docx]

***>PmTPS1***

ATGCATGAGAAGAGGAATTTGACAATTGTTCTTCTGCAGGGTTTTAATGCAACAATGACTGAACAAGTTGATGTGCCTGGAAGAAGAGGGAATGGCCAAATTAAAGAAATGAAGCTTGTTTTGCATCCTGAACTGAGAGAACCCCTTTCAATTCTCTGCAATGATCCAAAGACAACCATAGTTATTCTTAGTGGAAGTGAGAGGAGTATCTTAGATGAGAATTTCAAAGATTTCAACGTGTGGTTGGCAGCAGAGAATGGCATGTTTCTTCGCCTCACATCAGGAGAGTGGATGAGTACGATGCCTGAGCATCTTAATATGGACTGGGTTGAAAGTGTTCAGCTTGTTTTGGACTATTTCCGAGAAAGGACACCACGGTCATATGTCGAGGTCCGAGAAACATCACTAGTATGGAACTACAAATATGCAGATGTTGAATTTGGAAGAGTTCAGGCACGGGATATGTTACAGCACTTGTGGACTGGTCCTATTTCAAATGCTGCTGTTGACGTTGTCCAGGGCAGCAGGTCAGTGGAGGTCCGTTCTGTTGGTGTTTCAAAGGGTGCAGCAATTGACCGGATATTGGGAGAAATAGTTCATAGTAAATGCATGACAACACCGATAGATTATGTTCTCTGTATTGGCCACTTTTTAGGCAAGGATGAAGACATCTACACCTTCTTTGAGCCAGAACTTCCCTTTGATAAAGAAGTAACTGGGATTGGAAAGTTGTTGGAGAATAAAGCTTGTCTGGAAAAAAGAACAGGACCAAAGCCTAACGCTGGGAAAAGTGGCAGTAAAGGGGTACGCAACAAAGTTTTTCCGTTGTCACCGGAAAAGGGACCACAGTCAAGAAGTAACAACAGTAAAGATTGGGGGATGCAAGATGAGAGTGTTGGCTGGCATGAGGGGTCTTCTGTGCTGGACTTGAAAGGGGAGAACTATTTTTCTTGTGCGGTAGGTCGAAAACGTTCGAATGCACGCTATTCTCTAGCATCCTCTGAGGAAGTTGTCACTTTTATTAGAGCTATGGCAAAAAGTTATGCAGAAGCTGGTCAAGATATAGATGTTGATAATCATGCAGATCCTGATATCAAAGAATCAAACAATGATGATAGTGAAGAAGTTGAAGAAATGAGTGAAATTGAATTTCAAAATGAAGTTGGTAGAGTTATACAGGAACTCAAGAGTGAAAAGGAAAGGGATAAATTTTTGGAAGAAGAATTGAAGATAGCCAAAGGACATGTCGAAAGACTTTATAAGAAGAATGAAGAATCTAGGGAAATTATCGCCAGTCTTAGAATCTAG

***>PmTPS5***

TCAATGCAGGCTCTCGAAAGCAACTTGAGGAGATAGACTTTTGGCTGCTACATCAGAAGCTGTAGCCAGACCCTGAAGCATTCTCATGACCTCCACAGTATCATCTAAATAAAACTTTGCTTTACTTGGTTTCCGGTAAACGGTACAAGCAAACACTTCGACCATAGAGGCCATAGATGGTTGTGCCATTGCAATGCTCTCAAACATATCTTCATCAGATCTGTCATCTCCTATGCACAAAACAAAATCTGGTGGCTTTCCTCTTTCAACCATTGTTGACAGAAGTTGTTGTGCTACCAAACCCTTACTAACACCCTGGGGTTTAACTTCAACAATATGCTGACCACTTTTGACCTCCACAGGCTCATTTGCAAGCACACTCTCCAGATGATCTAAAAGCTCCTTTGATTGGCATGATCCAAAGTCTGGATCAGCATCCTGATGATGCCAAACCAAAGCACTTTCTTTAGCTTCTATGGCAGAACCATCAGTAGTTTCAGTATATAATTTCATAACAGGCTCTGCTGTTTTCTTCCAGTCAAAGTCTGCAGTGGGTAGCGTTGTTTCCCATTCGGAATCTTGGTTCCACCTAATGAAATAGCCATGCTCTGCTGCAATCCCTAGCTTATGGCAAGGTGAAAACCATTCACTGAGAGATTTCCGACCTCTTCCACTCACAATAAAGACAAGGTTTTTAGGGTCATTGCATAAGCTGTTTACAACAGATATTACTTGTGGACTTGGTGTTTTGTTGATAGAAGTCTGAGGCATCATAGTACCATCATAATCCAACAAAATTGCCCTGCTGTAGGTTCTCCTGTATGCAGAGACAATATGTTCTGGAGAAAGCTTCCTAAAGCTTGGACTAAGTGCGACAACCCTGAAACTCAATCCAAATCCAATACCCCAGCACCTTCTCTTAAAGTGATCCCGGCATACTCTCTCCAAATCCTGCTCAAAGCTGTGAGCCCAATAATTTACACCATGTGTGCTCACATACTTGTAATGTTTTTCATGCCGTAGTTGCTGCTCAGCCTCAGGAATTGTCAGGGATACATTCATGGCATCAGCAACAGCTTCTATGTTCCAGGGATTGACCCTGATGGCTCCACTGAGAGAAGGAGAGCACCCTATGAACTCAGACACAACTAACATACTCTTCTTTGAAGTTCCTGAACTTAATCCTAAGGCTTCATCTACTTTGGAGCTACCCTGTCTGCAACCAACGTATTCATAAGGAGTGAGATTCATTCCATCCCTCACAGCGTTAACCACACAACAATCCGCAATTGTGTAGAAAGCATATCTCTCAAACAATGGAACTGCTCTGTCTACTAACACGACAGGCTCATAGCCAGGGCGCCCAAATGTCTCATTGATTCTCTTGGTAATTGAATATGTCTCATTCTGTATTTCCTCGACATCCTTTCCCTTGCCCCTTGCAGGATTTGCAATCTGCACTAATACCACTTTGCCCCTCCACTCAGGATGTTGCTTAAGCAACTGTTCCAATGCTAGTAACTTCAAACTGATACCCTTGAATATATCCATGTCATCAACACCCAACAGCATTTTCTTCCCCTTGAATTGTTCCTTCAGCTCCCCAATTCTCAATGCAGTGTCCGGAAGATTTAACACTGATTCAAGCTGGCCCATGTGAATGCCCACAGGTAGAATCTTTATACCAACAGAACGACCATAGTATTCAAGTCCAATGTAGCCTCTCTTCGATTCATAATCAAGACCAAGCATCCTACTGCAACAGGAGAGAAAATGTCGAGCATAATCAAATGTATGGAAACCAATCAAGTCCGAGTTAAGTAGAGCCCTGAGGATTTCTTCCCTCACAGGAAGGGTCCTAAAAATCTCCGATGAAGGGAATGGGCTATGCAGAAAGAATCCAAGCTTCACCCTGTTAAATCGCTTCCTTAGAAATGTGGCAAGCACCATGAGATGGTAGTCATGTACCCAAACATAATCATCATCAGGGCTGATCACTTCCATGACCTTGTCTGCAAAGATCTTATTAGCAGACACATAAGCCTGCCAAAGGGATCGATCAAAGCGGCCTCCATGATCTGGAGAAAGGGGCAACATGTAGTGAAACAGTGGCCACAGCTGCTGCTTACAAAATCCATGGTAAAATTTACTTCGAAGCTCAGGTGACAGAAAAGCCGGTACACACTTGAAATTCTCTAGCAAAATCTGAGCAACATCATCCTGTTCACTTGGATCTATGTCTGCCTTTAAACAACCAACATAAACCACCTCCAAATCCTCTGAAAGCCCATCCTTAAGCTGCAATACCAAAGAATCCTCATCCCAACTAAAAGACCAACCTTTACCTTCCAGCCTTTTGTGAGCTCGCAAAGGAAGCTGGTTAGCTACAATGATAATGCGCTGTGAGCTGACAGATGAGGGTACATCTGAGGCCACGCTATTACAGTTATCATCATCAAACTCAGATAATATCCCTGGCACAGT

***>PmTPPD***

ATGGCATACGGATTAGAGCAAAATGCTGTTCTTACTGATGCTCCCGCAGTAGTCCCAAATTTACCCTTGCCTACAAATTCATCATCATCCCTGTATTCTCCAAGTTCAACTTCTGGTAGCTCAGTACCTACAGCTCAACGAAAGATGCTTTCAAGATCTATTAGTGCTGTTCTTACTGATTCTTCTACAAGAGTTCCAAATTCATCCGTGTTTTCATATTCATCAATAACACCATATTCTCCAAGTTCAACATCTGGTAGCTCAGCATCGACAGCTCAAAAAAAGGTGCTTTCAAGATCAATCAGTGCTGTTCTTACTGATTCTTCCACGGTTGTTCTTACTGATCCTCCCATGCCAGTTCCAGTTCCACCCTTGCTTTCATTTTCATTATCATCAAGGTATTCTCCAAGGTCAGCATCTGGTAGCCCAGTATCTAGAGCTCAAAGAATGGTTCTTCCAAACAGGATCGATGTTGAGAATCGTGTTAGCCTTATCAATGCAATGAGAGCGTCATCTCCTACTCATGCCCGTCAGGAAGCAGCCTATCGTGCCTGGACTGGTAAGCATCCTTCAGCATTGACCATGTTTGAGATGATGATGGATGCCTCAAAAGGCAAGCAAATTGTAGTGTTCTTGGATTATGATGGCACTCTTTCACCTATTGTAAATGATCCTGATCGTGCTTACATGTCGAAACAGATGCGTGCAACGGTTAGAAAAGTAGCAAGATATTTTCCTACTGCTATCATTACTGGAAGGTGCACAGCTAAGGTATATGAGTTTGTAAAGTTAGCCGAACTGTATTATGCTGGTAGCCATGGCATGGACATTATGGGACCAGCCAAAAGTTCAAAGGGCTCTAAGGCGAACTGTTCCAGGACAACAGACAAAAAAGGTGGTGTTGTTCTCTACCAGCCTGCTAGTGAGTTCTTACCAATGATAGACAAGGTTTTTGCATTGCTGGTTGAAAAGACAAAGAATATCAGTGGAGTCATAGTTGAAAATCACAAATTTTGTGCTTCTGTGCATTATCGACGTGTTGAAGAGAAGAGTTGGGCTGCACTTGCAGAGCAAGTAGCAAGTGTAGTTAAAGAGTATCCTCAATTGCGACTGACACAAGGAAGGAAGGTCTTAGAGATTCGTCCTATAATTAAATGGGACAAAGGAAAAGCCCTTGAATATTTATTAGTGTCACTTGGATTGGCAAATCGGAATGATGTACTACCATTGTATATAGGAGATGATCGTTCAGATGAAGATGCATTTAAGGTACTGCGGGACAGAGGTCAAGGTTATGGGATTCTTGTGTCCACTGCCCCCCAAGAAACAAATGCAGCATATTCACTTAAAGAACCGGCTGAGGTTATGAATTTTTTGAATCGCCTCGTGAATTGGAAGCGGGATTCCAAAAAGCGTCCATTGTCTAAAGTGTAA
